# MicroRNA 3' end nucleotide modification patterns and arm selection preference in liver tissues

**DOI:** 10.1186/1752-0509-6-S2-S14

**Published:** 2012-12-12

**Authors:** Sung-Chou Li, Kuo-Wang Tsai, Hung-Wei Pan, Yung-Ming Jeng, Meng-Ru Ho, Wen-Hsiung Li

**Affiliations:** 1 Genomics Research Center, Academia Sinica, Taipei, Taiwan; 2 Department of Medical Education and Research, Kaohsiung Veterans General Hospital, Kaohsiung, Taiwan; 3 Graduate Institute of Pathology, National Taiwan University, Taipei, Taiwan; 4 Biodiversity Research Center, Academia Sinica, Taipei, Taiwan; 5 Department of Ecology and Evolution, University of Chicago, Chicago, IL 60637, USA

## Abstract

**Background:**

The expression of microRNA (miRNA) genes undergoes several maturation steps. Recent studies brought new insights into the maturation process, but also raised debates on the maturation mechanism. To understand the mechanism better, we downloaded small RNA sequence reads from NCBI SRA and quantified the expression profiles of miRNAs in normal and tumor liver tissues.

**Results:**

From these miRNA expression profiles, we studied several issues related to miRNA biogenesis. First of all, the 3' ends of mature miRNAs usually carried modified nucleotides, generated from nucleotide addition or RNA editing. We found that adenine accounted for more than 50% of all miRNA 3' end modification events in all libraries. However, uracil dominated over adenine in several miRNA types. Moreover, the miRNA reads in the HBV-associated libraries have much lower rates of nucleotide modification. These results indicate that miRNA 3' end modifications are miRNA specific and may differ between normal and tumor tissues. Secondly, according to the hydrogen-bonding theory, the expression ratio of 5p arm to 3p arm miRNAs, derived from the same pre-miRNA, should be constant over tissues. However, a comparison of the expression profiles of the 5p arm and 3p arm miRNAs showed that one arm is preferred in the normal liver tissue whereas the other is preferred in the tumor liver tissue. In other words, different liver tissues have their own preferences on selecting either arm to be mature miRNAs.

**Conclusions:**

The results suggest that besides the traditional miRNA biogenesis theory, another mechanism may also participate in the miRNA biogenesis pathways.

## Background

MicroRNA (miRNA) genes are small non-protein-coding genes. Their final functional products are single-strand RNAs of ~22 nucleotides. MiRNAs repress the expression of their target genes. It has been commonly observed that in the tissues of genetic disorders or tumors, the expression levels of many miRNA genes are significantly different from those in the normal tissues [1-7]. In our previous study, we observed miRNA 3' end modification and arm selection preference in one pair of normal and tumor gastric tissues [8]. To better understand these phenomena, we analyzed NGS data of 15 liver tissues in this study. We first characterized the expression profiles of mature miRNAs in liver tissues by analyzing small RNA sequence reads downloaded from NCBI SRA (SRP002272) [9]. Then, we used the expression profiles to address the issues to be described below.

The messenger RNAs (mRNAs) of protein-coding genes are usually subject to modifications, including poly-adenylation, which prolongs the lives of mRNAs. Recently, similar sequence modifications at the 3' end of miRNAs were detected by small RNA cloning and sequencing [10]. Further studies confirmed that such modifications were prevalent among animal and plant miRNAs and were caused by either RNA editing or nucleotide addition rather than by sequencing errors [11,12]. In this study, using the miRNA expression profiles in liver tissues, we showed that different tissues and different miRNAs have their own preferred nucleotide modifications although adenine accounted for the majority of the modification events. Moreover, HBV (hepatitis B virus)-associated tissues exhibited lower adenine modification rates than normal liver tissues.

Although mature miRNAs are derived from pre-miRNAs, the expression of pre-miRNAs does not guarantee the expression of mature miRNA. In other words, not all pre-miRNAs are processed into mature miRNAs [13-15]. Moreover, the 5p arm and 3p arm of the same pre-miRNA usually have unequal likelihoods to be processed into mature miRNAs [16]. Such arm selection preference is commonly thought to result from the fact that the RISC unwinds the miRNA/miRNA* duplex at the end with weaker hydrogen binding. Therefore, the arm with the freer 5' end is preferentially incorporated into RISC to serve as the mature miRNA [17,18]. This hydrogen-bonding-based selection rule is currently the majority view.

However, recent studies showed that the orthologous pre-miRNAs, although highly similar to each other and thought to have the same miRNA/miRNA* duplex, showed preference of the 5p arm in one species but the 3p arm in another species. Therefore, the major and minor forms of mature miRNA in different species may be switched [19,20]. In this study, we are curious whether the same pre-miRNAs have different arm selection preferences between normal and tumor tissues. Using miRNA expression profiles, we showed that the major and minor mature miRNAs of the same pre-miRNA are not always consistent with the miRBase annotation. Moreover, we found that the 5p arm and 3p arm miRNA derived from the same pre-miRNA have different tissue expression preferences, one preferring the normal tissue while the other preferring the tumor tissue. The result points to the existence of another selection mechanism in addition to the hydrogen-bonding theory.

## Results and discussion

### Analysis of sequence reads

**Figure 1:**
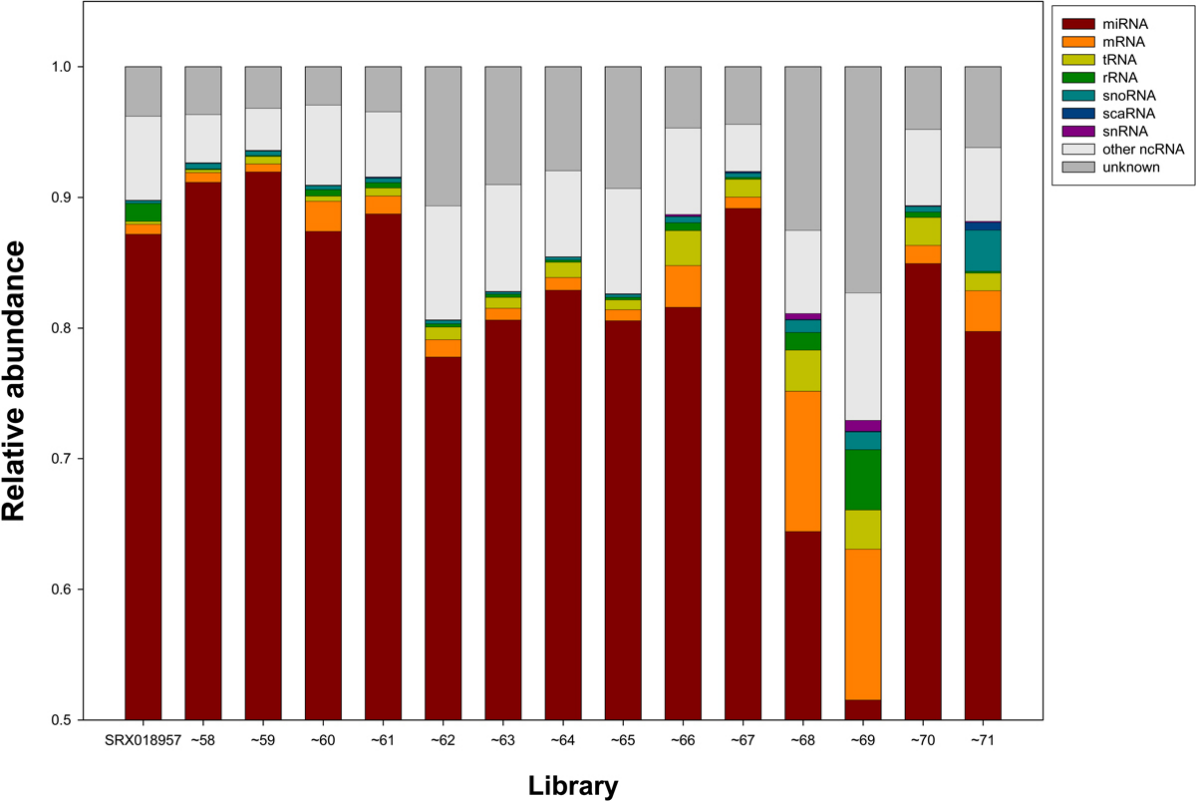
**Classification of analyzed sequence reads in libraries**. We classified the analyzed sequence reads into 10 classes, including miRNA, mRNA, etc. As shown in Additional File 1, repeat elements accounted for less than 0.001% in all libraries and therefore were not included in the figure. The X-axis denotes the analyzed libraries. To simplify the figure, the same prefix of a library name is denoted by "~".

We downloaded small RNA sequence reads of 15 liver tissues for analysis. The accession number and description of each library are given in Table 1. Under our criteria, there were about 8 to 11 million sequence reads available for analysis in each library except for SRX018968, which had only about 5.7 million reads (Table 1). Under our mapping criteria, about 80% to 90% of analyzed sequence reads were mapped to human miRNAs in most libraries. However, in SRX018968 and SRX018969, only about 64% and 52% sequence reads, respectively, belonged to miRNAs. We were curious about the remaining reads and analyzed the non-miRNA reads in the libraries to see what kinds of molecules they were. We divided the reads into 10 categories (Additional File 1) and their frequencies are shown in Figure 1. The non-miRNA categories, especially mRNA (p < 0.01) and tRNA (p < 0.001), showed significantly higher percentages in SRX018968 and SRX018969 than in other libraries.

**Table 1:** Summary of miRNA reads and library information.

Library	# of reads analyzed	# of miRNA reads	% miRNA reads	# of detected pre-miRNAs	# of detected miRNAs	# of detected miRNAs at opposite arm	Description
SRX018957	8,057,617	7,025,801	87.19%	381	475	42	Normal
SRX018958	11,234,315	10,241,350	91.16%	439	555	62	Normal
SRX018959	10,035,888	9,229,254	91.96%	424	535	65	Normal
SRX018960	8,493,004	7,424,150	87.41%	400	486	39	HBV(+)
SRX018961	8,214,755	7,290,360	88.75%	507	609	82	Severe HBV(+)
SRX018962	7,413,793	5,767,890	77.80%	417	519	48	HBV(+) distal
SRX018963	8,214,714	6,623,536	80.63%	418	529	51	HBV(+) adjacent
SRX018964	8,042,722	6,668,203	82.91%	426	540	44	HBV(+) side
SRX018965	7,859,049	6,332,172	80.57%	472	598	64	HBV(+) HCC
SRX018966	10,640,841	8,683,683	81.61%	467	596	60	HBV(+) adjacent
SRX018967	10,979,156	9,790,644	89.17%	465	584	64	HBV(+) HCC
SRX018968	5,686,232	3,663,927	64.44%	443	556	58	HCV(+) adjacent
SRX018969	9,224,742	4,754,658	51.54%	430	521	68	HCV(+) HCC
SRX018970	8,967,260	7,618,011	84.95%	407	519	56	HBV(-) HCV(-) adjacent
SRX018971	9,102,563	7,259,865	79.76%	546	682	112	HBV(-) HCV(-) HCC

We mapped the sequence reads back to human pre-miRNAs, quantifying the expression levels of pre-miRNAs, mature miRNAs and isomiRs. According to miRBase 16, 730 out of the 1,048 human pre-miRNAs encode only one mature miRNA at one arm, either 5p or 3p arm. With the improvement on NGS's sequencing depth, we also detected the opposite-arm mature miRNAs at the pre-miRNAs originally annotated to encode only one miRNA. HBV, HCV and HCC are abbreviations of hepatitis B virus, hepatitis C virus and hepatocellular carcinoma, respectively. Distal, adjacent and side mean that the sites at which the tissues were sampled were >10cm, 2-5cm and 0-2cm far from the HCC sites.

Vaz and colleagues observed a much lower miRNA percentage in small RNA collection from the K562 cell line, due to the reduced expression of the Dicer gene [21]. Among all of the 15 libraries we studied, only SRX018968 and SRX018969 were hepatitis C virus (HCV) associated libraries: SRX018969 was sampled from HCV-positive HCC tissue and SRX018968 was sampled from the adjacent region of HCV-positive HCC tissue. Examining the miRNAs in these two libraries, we found that the first several abundant miRNAs are highly similar to the ones in the HCV-negative libraries. We wonder whether the infection of HCV represses the function of *Drosha *or *Dicer *so that the expressions of all miRNAs are evenly reduced, without altering the abundance ranking of individual miRNAs. Therefore, we examined the expression levels of *Drosha *and *Dicer *in HCV-positive and HCC-negative samples. Additional File 2 shows that the expression levels of *Drosha *and *Dicer *are not significantly different among samples. Thus, the lower numbers of miRNA reads in the two HCV-positive libraries could be simply due to sample preparation or the intrinsic characteristic of disease tissue.

In Morin's study, repeat elements accounted for about 16% of the total sequence reads from the human embryo stem cell [11]. In this study, however, repeat elements accounted for a very low percentage in all libraries. The difference might have occurred because we analyzed only the reads with 3' adapter ligated and excluded the one-copy clean reads.

### Analysis of detected miRNAs

**Figure 2:**
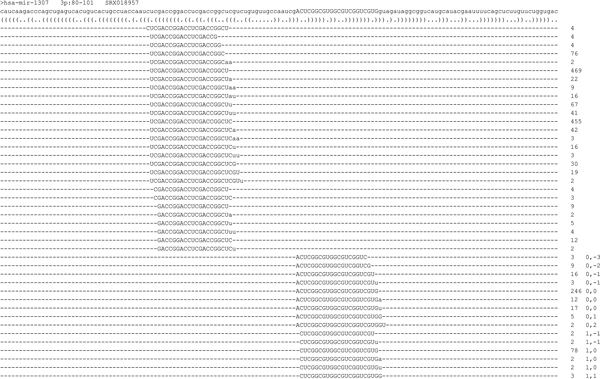
**Mapping result of hsa-mir-1307 reads in the SRX018957 library**. Hsa-mir-1307 encodes mature miRNA hsa-miR-1307 at its 3p arm, ranging from nt 80 to 101 of pre-miRNA, as presented in uppercase. The sequence reads mapped to hsa-mir-1307 were arranged according to their mapped loci within the pre-miRNA. The expression level of a mature miRNA is determined by summing the read counts (initial read counts, not normalized as TPM, transcript per million) of all its iso-reads, isomiRs. The mismatches at the 3' ends, shown in lowercase, denoted the modified fragments. In the figure, only the modified fragments, including A, U, AA, AU and UU, with frequency $\underset{-}{\geq }$ 1% were recovered.

By mapping the sequence reads back to pre-miRNAs, one can quantify the expression levels of mature miRNAs. In this study, we detected about 500 to 600 mature miRNAs in most libraries. For each pre-miRNA, we arranged the miRNA reads according to the mapped locations within the pre-miRNA. As shown in Figure 2, hsa-mir-1307 encodes mature miRNA at only the 3p arm according to miRBase 16. The integers denote the read counts. The numbers with comma in the right column denote the location offset relative to the reference miRNA annotated by miRBase. For example, the reads with "0,0" are exactly the same with the reference miRNAs. As reported previously [11,22,23], we also observed that the reference miRNAs from miRBase are not necessary the most abundant ones. The mapping results of all pre-miRNAs in 15 libraries are shown in Additional File 3 (the read counts are not normalized as TPM, transcript per million).

Table 1 shows that about 500 to 600 mature miRNAs were detected in most libraries; however, most of the sequence reads belonged to a few miRNAs. For example, hsa-miR-122 accounted for $\underset{-}{\geq }$ 50% of all reads in a library in most cases, except for SRX018961 (37.10%) and SRX018971 (2.57%). SRX018971 is the only HBV-negative and HCV-negative HCC sample. Hsa-miR-122 was reported to activate HCV translation [24] and to be highly expressed in HCV infected patients [25]. However, comparing with other HCV-positive (SRX018968 and SRX018969) and HCV-negative samples, the expression level of hsa-miR-122 in SRX018971 dropped dramatically. This is an interesting observation for further study.

### Detection of additional miRNAs at the opposite arms

Among the 1,048 human pre-miRNAs, 730 were annotated to encode mature miRNAs at only one arm in miRBase 16. With the increased sequencing depth of NGS platforms, more mature miRNA can be detected at the opposite arm. As shown in Figure 2, we detected also the mature miRNA at the opposite arm, i.e., the 5p arm of hsa-mir-1307 from SRX018957. Furthermore, like common mature miRNAs, the opposite-arm miRNA of hsa-mir-1307 also has many isomiR types, highly overlapping with each other. Actually, in all of the 15 libraries, the opposite-arm miRNA of hsa-mir-1307 was detected.

As described in a previous study [22], the opposite-arm miRNAs are not necessarily kept at lower expression levels than the annotated ones. In this study, the opposite-arm miRNA of hsa-mir-1307 has a higher expression level than the annotated one in most libraries, except for SRX018968 and SRX018971. In other pre-miRNAs, this phenomenon can also be observed (Additional File 3), which challenges the nomenclature of miRBase. The finding of opposite-arm miRNAs was made because we mapped the sequence reads back to pre-miRNAs rather than to mature miRNAs. In this study, we detected many opposite-arm miRNAs in all 15 libraries (Table 1).

### Analysis of modification fragments generated by 3' end modification

Previous studies reported nucleotide variations preferentially locating at the 3' end of miRNAs [10-12,26,27]. Such variations can arise from either nucleotide additions, elongating the miRNAs, or RNA editing that does not alter the miRNA length. Regardless of how the modified nucleotides occur, they can cause mismatches in the mapping procedure, making the originally perfect match reads fail to be mapped back to miRNAs. Therefore, we trimmed the terminal 3' end mismatch one nucleotide at one time until mapping succeeded, and then, analyzed the trimmed nucleotides. These trimmed nucleotides are thought to have occurred by modifications so that we call them modified fragments, which were recovered and presented in lower case (Figure 2 and Additional File 3).

Table 2 shows that in most libraries the most abundant modified fragments are A, U, AA, AU and UU in the descending order of relative abundance, where A and U denoted adenine and uracile, respectively. These modified fragments are AU rich and A accounts for at least 50% of all modification events in all libraries. In addition, one-nucleotide modifications (A and U) are much more frequent than two-nucleotide modifications (AA, AU, UU, etc). In summary, these five modification events account for almost 90% of all modification events in most libraries except for SRX018965 and ~71 (ie., SRX018971).

**Table 2:** Frequencies of the 3' end modified fragments in libraries.

Library	A	U	AA	AU	UU	SUM	**miRNA reads with 3' end modi**.
**SRX018957**	64.74%	11.86%	6.46%	4.61%	4.58%	92.25%	29.78%
**SRX018958**	67.17%	11.95%	4.98%	2.77%	3.90%	90.77%	30.68%
**SRX018959**	73.16%	9.40%	4.90%	2.72%	2.47%	92.65%	28.55%
**SRX018960**	72.54%	11.39%	5.06%	2.29%	2.56%	93.84%	30.11%
**SRX018961**	60.76%	19.78%	5.57%	2.83%	2.04%	90.98%	15.02%
**SRX018962**	66.21%	13.10%	5.31%	3.89%	2.72%	91.23%	19.90%
**SRX018963**	64.39%	14.56%	5.52%	3.82%	3.34%	91.63%	20.31%
**SRX018964**	60.02%	16.08%	5.96%	4.81%	4.37%	91.24%	20.55%
**SRX018965**	62.39%	11.89%	4.22%	3.66%	1.61%	83.77%	12.91%
**SRX018966**	68.71%	10.60%	5.92%	1.17%	2.94%	89.34%	27.19%
**SRX018967**	73.14%	8.56%	6.30%	2.70%	1.77%	92.47%	31.05%
**SRX018968**	69.95%	8.60%	7.66%	3.17%	1.66%	91.04%	21.93%
**SRX018969**	63.61%	14.26%	5.01%	2.59%	4.18%	89.65%	18.28%
**SRX018970**	67.59%	9.56%	4.80%	3.99%	4.82%	90.76%	35.91%
**SRX018971**	50.00%	14.03%	11.32%	4.48%	2.42%	82.25%	16.81%

Using an alternative mapping criterion, we collected the sequences of the 3' end modified fragments by analyzing their relative abundances in all modification events. A and U account for almost 80% of all modification events. Only the modified fragments accounting for $\underset{-}{\geq }$ 1% of all modification events are listed here.

**Figure 3:**
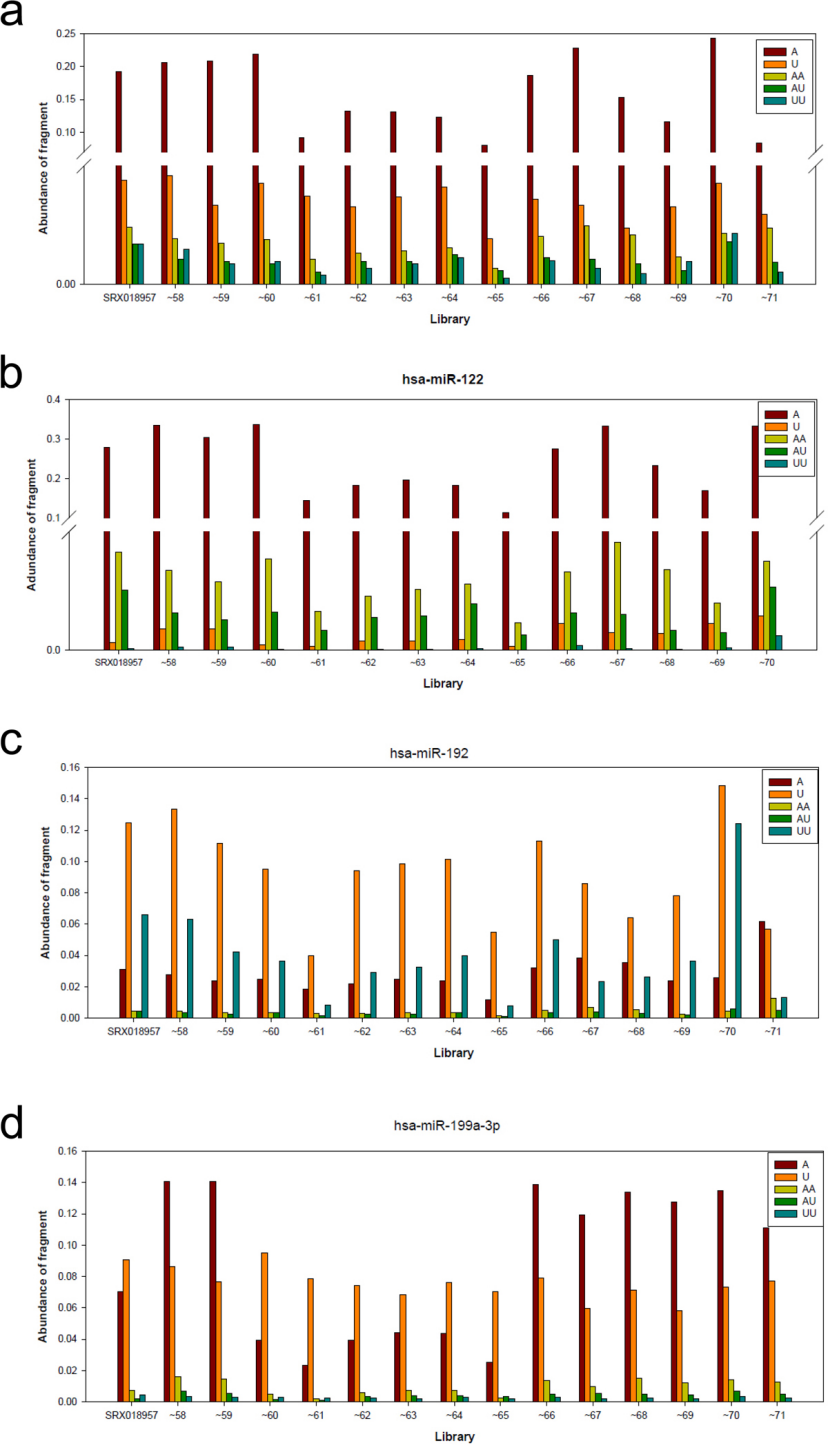
**The frequencies of abundant modified fragments in all libraries**. Only the modification events with $\underset{-}{\geq }$ 1% frequency in all libraries were plotted. (a) The global patterns of modification events. (b,c) Several miRNAs have their specific patterns of modification events different from the global one. The expression level of hsa-miR-122 is much lower in SRX018971 than in other libraries, so that SRX018971 was not included in Figure 2a. (d) The patterns of modification events of some miRNAs are library specific.

Moreover, almost half of all libraries, including SRX018957, ~58, ~59, ~60, ~66, ~67 and ~70, had about 30% of miRNAs reads undergone modification at their 3' ends. The modification rate ranged from 13% to 20% in the remaining libraries. Upon further examination, most of the low-modification-rate libraries belong to hepatitis B virus (HBV) associated samples (including SRX018961, ~62, ~63, ~64 and ~65). Furthermore, we found that the reduced modification rates were mainly owing to the decrease of A modification (Figure 3a), implying that HBV infection can repress the mechanisms responsible for the A modification without affecting the other modifications. In summary, with our mapping method, we can use these modified sequence reads to quantify miRNAs.

Figure 3a shows the global distribution patterns of modification events in libraries. However, we are more interested in whether individual miRNAs have preferred modifications different from the global pattern. Therefore, we examined 16 mature miRNAs with a high expression level in most libraries. We found that four mature miRNAs, miR-30d, miR-101, miR-140-3p and miR-378, have similar patterns to the global patterns (Figure 3a). However, in let-7a, let-7b, let-7c, miR-122 and miR148a, the patterns are somewhat different from the global pattern. For example, in miR-122 (Figure 3b), the frequencies of U and UU events decreased so dramatically in all libraries that U was even much rarer than AA, which was originally dominated by U in the global pattern.

Another interesting pattern can be illustrated by miR-192 and miR-29a. Contrary to the pattern of miR-122, the frequencies of A and AA events dramatically decreased in miR-192 (Figure 3c); in other words, U and UU dominated over other modification events except in the SRX018971 library. The A and U decreases in Figure 3c and 3b were consistently found in almost all libraries, demonstrating that different individual miRNAs may have their own preferred modifications.

Although the above modification patterns were consistent in almost all libraries, we observed inconsistent patterns in miR-99a, miR-100, miR-103, miR-191 and miR-199a-3p. For example, in miR-199a-3p (Figure 3d), the U modification has a significantly higher frequency than the A modification in SRX018960, ~61, ~62, ~63, ~64 and ~65, most of which are HBV associated libraries. However, the A modification obviously dominated over the U modification in the remaining libraries, most of which were not HBV associated libraries. The global pattern of modifications (Figure 3a) implied that HBV infection can repress the A modification, which seems to be consistent with the result for miR-199a-3p (Figure 3d).

Previous studies reported several types of RNA editing, such as A to G transition catalyzed by adenine deaminase and C to U transition catalyzed by cytidine deaminase [11,28], responsible for generating 3' end variations without altering the miRNA length. However, nucleotide addition can also cause the same variations by elongating miRNA length. Owing to the existence of isomiR, the lengths of miRNAs can be dynamic [29]. Therefore, it is difficult to determine whether nucleotide addition or RNA editing contributes to such 3' end variations. Hence, instead of RNA editing or nucleotide addition, we used the term "modifications" to avoid the debate. In summary, 3' end modifications can be miRNA dependent and can depend on the tissue condition (normal or abnormal), making the modification patterns more complicated.

In this study, we showed that the 3' end modification event of miRNA cab be library dependent and that HBV-positive samples tend to have a lower chance of A modification. MiRNAs recognize their target site mainly by the complementary pairing between their seed region, nucleotides 2~7, and the 3' UTR. The modification event occurring in the 3' end can hardly alter their selection of target gene. Therefore, 3' end modification may have little impact on miRNAs' target gene selection and function.

### Inconsistent expression ratios of 5p arm to 3p arm

According to the hydrogen-bonding theory, the selection preference between pre-miRNA's 5p arm and 3p arm is an intrinsic characteristic of pre-miRNA [17]. If this selection mechanism is the only one in deciding arm selection preference, the expression ratio of 5p arm to 3p arm should be largely constant in all tissues. To examine this theory, we investigated whether for a pre-miRNA the expression ratio of 5p arm to 3p arm differs between normal and tumor tissues. As shown in Figure 2, the 5p and 3p sequence reads of hsa-mir-1307 in SRX018957 were, respectively, 1325 and 402, resulting in a 3.30 expression ratio of 5p arm to 3p arm.

**Figure 4:**
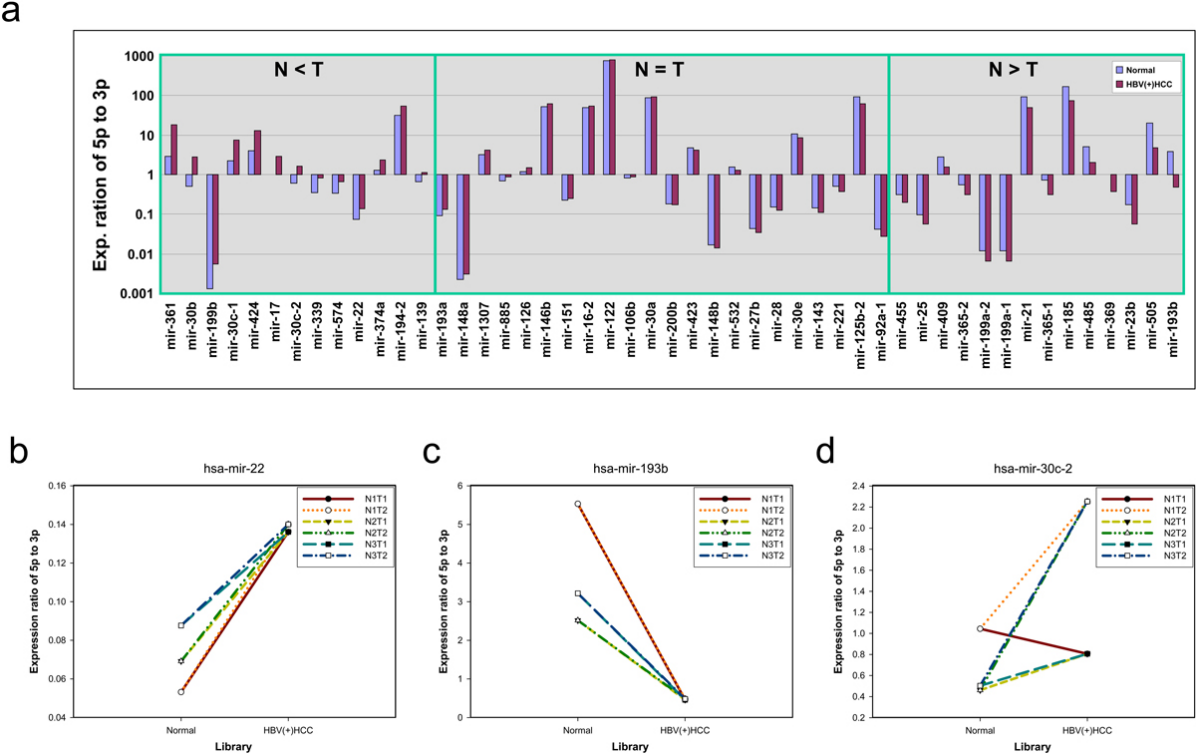
**The expression ratios of 5p arm miRNA to 3p arm miRNA**. In order to avoid extreme values caused by low expression levels, we examined only 49 pre-miRNAs whose read counts of 5p arm and 3p arm were $\underset{-}{\geq }$ 5 in TPM (transcript per million) unit. The cutoff of significant difference of expression ratios between libraries is 1.5 fold change. (a) The expression ratios of 5p arm to 3p arm were averaged from three normal and two HBV(+)HCC libraries. (b,c,d) The N1, N2, N3, T1 and T2 denoted SRX018957, SRX018958, SRX018959, SRX018965 and SRX018967 libraries, respectively. So, NIT1 denoted the comparison in the NT pair composed of SRX018957 and SRX018965.

Among the 15 libraries, SRX018957, ~58 and ~59 were three replicates of normal liver tissue, while SRX018965 and ~67 were two replicates of HBV-positive HCC (HBV(+)HCC) tissue. We compared the expression ratios averaged from the three normal and the two HBV(+)HCC liver tissues. In Figure 4a, an upward bar means that the 5p arms had a larger read count than the 3p arm, while a downward bar means that the 3p arm was the major arms in terms of abundance. Among the 49 examined pre-miRNAs, 27 have unequal averaged expression ratios between normal and HBV(+)HCC tissues under the cutoff of 1.5 fold change. Moreover, 13 and 14 out of the 27 pre-miRNAs had higher expression ratios in tumor and normal tissues, respectively. Figure 4a shows that more than half of the examined pre-miRNAs had unequal expression ratios and several of them had a fold change up to 10, demonstrating that in many pre-miRNAs the arm selection preference is different between the normal liver and HBV(+)HCC tissues.

Although Figure 4a shows a significant difference, an analysis based on average values may neglect the diversities among tissues. Hence, we also examined pre-miRNAs' expression ratios of 5p arm to 3p arm among six normal HBV(+)HCC (NT) pairs. Several pre-miRNAs showed consistent patterns between NT pairs. Figure 4b shows that hsa-mir-22's expression ratios of 5p arm to 3p arm had the same tendency between NT pairs and that they were all higher in HBV(+)HCC tissues than in normal tissues. This result reflected the fact that the 5p arm of hsa-mir-22 was more preferred in HBV(+)HCC tissues than in normal tissues. A similar phenomenon was also observed in other pre-miRNAs such as hsa-mir-17 and hsa-mir-30b.

Although hsa-mir-193b's expression ratios of 5p arm to 3p arm also had the same tendency between NT pairs, the values were all higher in normal tissues rather than in HBV(+)HCC tissues (Figure 4c). Furthermore, the 5p arm of hsa-mir-193b was more preferred in normal tissues than in HBV(+)HCC tissues. Other pre-miRNAs such as hsa-mir-23b showed a similar phenomenon. The above cases showed consistent patterns of expression ratios between all NT pairs, but diversities among NT pairs were observed in several pre-miRNAs, such as has-mir30c-2 (Figure 4d).

In summary, the 5p and 3p arm selection preferences are not always consistent between normal and tumor tissues, implying there is another selection mechanism, in addition to the hydrogen-bonding-based selection rule. If so, this selection mechanism may play a regulatory role in the oncogenesis pathway of HCC.

### Arm selection preference between normal and tumor tissues

In our previous study, we showed that the arm selection preference of the same orthologous pre-miRNAs can vary between species, so that some species preferred the 5p arm and the other preferred the 3p arm [19]. In Figure 4a, the expression ratio bars of the same pre-miRNAs showed different directions (upward or downward) between libraries. An upward bar means that the 5p arm was the major arm, while a downward bar means that the 3p arm was the major arm. Therefore, we were curious about whether different arm selection preferences can be found between tissues, especially between the normal and HBV(+)HCC libraries.

We first examined whether the arm selection preference of pre-miRNA annotated by miRBase is consistent with the NGS expression data from the three normal and two HBV(+)HCC libraries. Table 3 shows that the arm selection preferences of 13 pre-miRNAs were opposite to the miRBase annotation in more than half of the libraries studied. According to the miRBase annotation, hsa-mir-511-1, hsa-mir-382, hsa-mir-548h-3 encode mature miRNA at only their 5p arm. In more than half of the analyzed libraries, however, we not only detected mature miRNAs at their 3p arms but also observed that the newly detected 3p arms had a higher expression level than the originally annotated 5p arms. In addition, the newly detected 5p arm of hsa-mir-1307 also had a higher expression level than the originally annotated 3p arm.

**Table 3:** The pre-miRNAs whose arm selection preferences are not consistent with the miRBase annotation.

pre-miRNA	Location	N1	N2	N3	T1	T2
hsa-mir-30c-2	MA:7-29;mi:47-68	-	Y	Y	Y	-
hsa-mir-30b	MA:17-38;mi:55-76	Y	Y	Y	-	-
hsa-mir-126	mi:15-35;MA:52-73	Y	-	Y	Y	-
hsa-mir-106b	MA:12-32;mi:52-73	Y	-	Y	Y	-
hsa-mir-377	mi:7-28;MA:45-66	Y	Y	Y	Y	-
hsa-mir-382	5p:11-32	Y	Y	Y	Y	-
hsa-mir-511-1	5p:16-36	Y	Y	Y	-	-
hsa-mir-193b	mi:14-35;MA:51-72	Y	Y	Y	-	-
hsa-mir-500a	MA:13-35;mi:52-73	Y	Y	Y	Y	Y
hsa-mir-505	mi:15-36;MA:51-72	Y	Y	Y	-	Y
hsa-mir-548h-3	5p:29-50	Y	Y	Y	Y	Y
hsa-mir-664	mi:11-34;MA:49-71	Y	Y	Y	Y	Y
hsa-mir-1307	3p:80-101	Y	Y	Y	Y	Y

**'**Y' indicates that the major-to-minor arm switch event was observed in the corresponding library; while '-' means "not observed". N1, N2, N3, T1 and T2 denote the SRX018957, SRX018958, SRX018959, SRX018965 and SRX018967 libraries, respectively.

For the pre-miRNAs originally annotated to encode miRNAs at both arms, the major arms of hsa-mir-30c-2, hsa-mir-30b, hsa-mir-106b and hsa-mir-500a were their 5p arms, while the major arms of hsa-mir-126, hsa-mir-377, hsa-mir-193b, hsa-mir-505 and hsa-mir-664 were their 3p arms. From the NGS expression data, however, we found that their major arms and minor arms were switched, contrary to the miRBase annotation. Among them, hsa-mir-500a, hsa-mir-548h-3, hsa-mir-664 and hsa-mir-1307 were the extreme cases, at which the major-minor switch was observed in all of the analyzed libraries, including three normal and two HBV(+)HCC libraries. In conclusion, the major and minor arms of pre-miRNA can vary among tissues.

**Figure 5:**
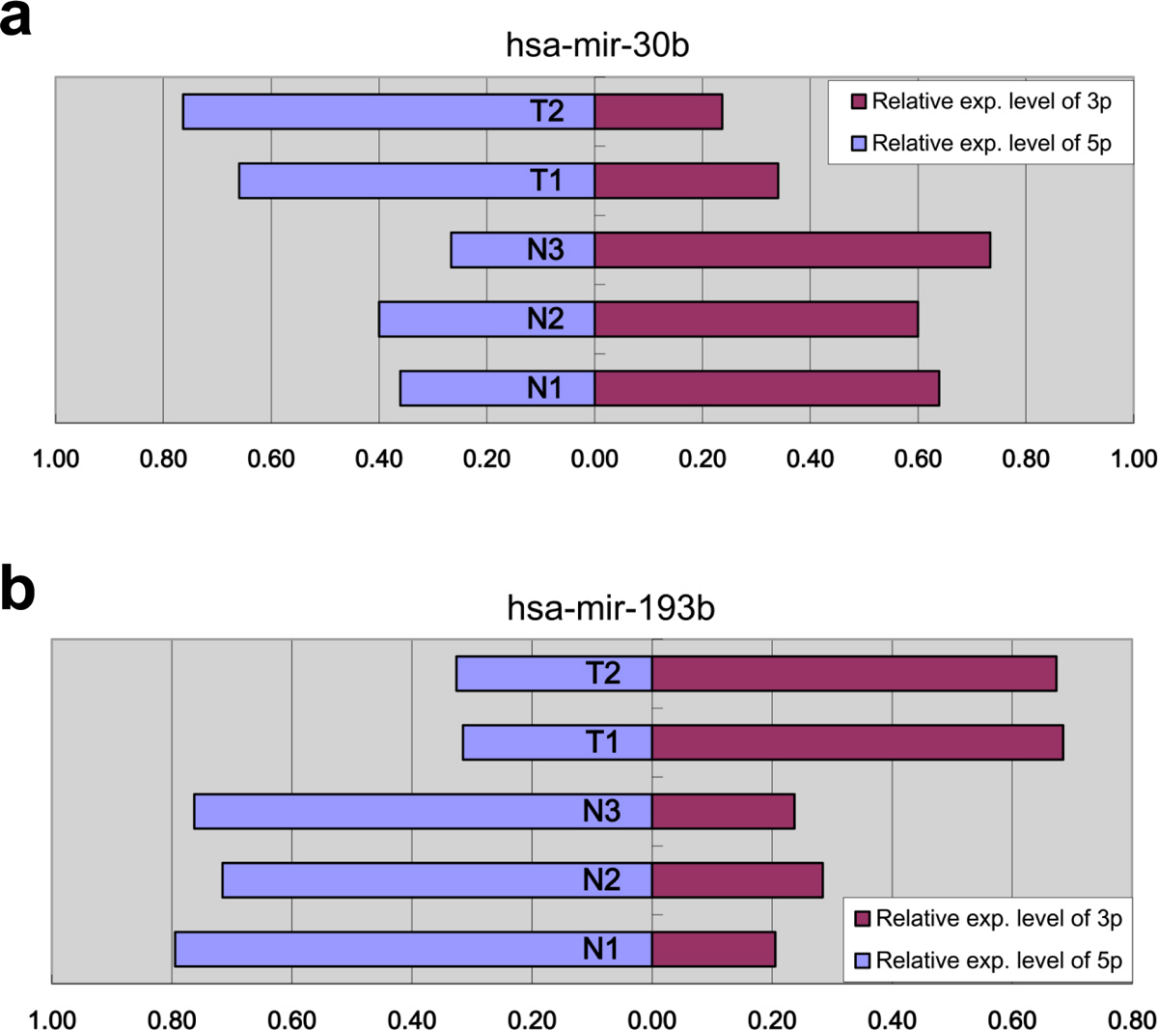
**Arm selection preferences of 5p arm and 3p arm differ between normal liver and HBV(+)HCC tissues**. In hsa-mir-30b, the 5p arm miRNA is preferred in tumor tissue, whereas the 3p arm miRNA is preferred in normal tissue (p value = 0.013). In hsa-mir-193b, the 5p arm miRNA is preferred in normal tissue, whereas the 3p arm miRNA is preferred in tumor tissue (p value = 0.007).

Since the arm selection preference annotated by miRBase was inconsistent with our NGS expression data, we investigated whether arm selection preference could vary between tissues. In other words, we investigated whether the 5p arm and 3p arm miRNA derived from the same pre-miRNA have reversed tissue expression preferences without considering the miRBase annotation. For each pre-miRNA, we first tabulated the expression ratios of 5p arm to 3p arm miRNAs in three normal and two HBV(+)HCC libraries (Additional File 3), excluding the 743 pre-miRNAs rarely expressed throughout all examined samples (the sum of total reads count in all samples < 100). As a consequence, each pre-miRNA had five values. Then, we conducted the generalized linear model test to detect the differential arm ratios between normal and tumor with log2 transform as the link function. We indeed found several statistically significant pre-miRNAs, among which hsa-mir-30b and hsa-mir-193b are the perfect cases (Figure 5). In hsa-mir-30b, the arm rations are significantly tissue dependent. Their 3p arm miRNAs had higher expression levels in the normal tissues, while their 5p arm miRNAs were predominated in the HBV(+)HCC libraries (p value = 0.013). In contrast, the 5p arm of hsa-mir-193b was the major miRNA in normal libraries and the 3p arm miRNA was the major one in HBV(+)HCC libraries (p value = 0.007).

In this study, we show that although the 5p arm and 3p arm miRNAs are derived from the same gene locus and transcribed by the same transcription factors, they have significant reversal expression preference between two tissues. We therefore conclude that there was another selection mechanism, in addition to the hydrogen-bonding-based selection rule. However, any factors affecting the precise measurement of miRNA's expression levels, such as PCR artifacts and unequal degradation rates, could also be possible explanations of the reversal tissue preference. During the sample preparation procedure of NGS, RNA molecules were amplified with PCR after adapter ligation. If the 5p arm and 3p arm miRNAs have different affinities with adapters, they may have unequal percentages of amplification, causing biased estimates of expression levels and false reversal tissue preference.

After miRNA/miRNA* (in terms of abundance) duplex is unwound, the miRNA is incorporated into RISC and usually has longer life, while the miRNA* is degraded soon. In addition to the protection of RISC, the nucleotide composition of the RNA molecule may also have unequal resistances to RNase. Therefore, the detected read counts by NGS may not really reflect the true miRNA's expression levels, causing biased expression preferences.

Although we can not completely rule out other factors causing biased measurement of miRNA's expression level, we presented a statistically significant result of miRNA's reversal preference in normal and tumor tissues. This new observation deserves further investigation.

## Materials and methods

### Collecting and processing of sequence reads

We first downloaded sequence reads with the accession number SRP002272 from NCBI SRA. This accession contains 15 libraries from different liver tissues. All library information is given in Table 1. All of the 15 libraries were sequenced using the Illumina (Solexa) small RNA sequencing technology. After downloading, all sequence reads were first processed to remove the 3' end adapter and the ones with adapter detected and trimmed were called "clean reads". In view of the length distribution of mature miRNAs, only 18~25 nt clean reads were collected for analysis. Moreover, for higher confidence, only the unique clean reads with read count $\underset{-}{\geq }$ 2 were mapped back to human pre-miRNAs (miRBase 16).

### The criteria of mapping sequence reads back to pre-miRNAs

In previous studies using NGS reads to quantify miRNA expression levels, nucleotide variations were usually allowed when mapping sequence reads back to the genomes or the reference miRNAs [28], resulting in ambiguously mapped loci caused by the high similarity between human mature miRNAs, such as hsa-miR-548a, hsa-miR-548b and hsa-miR-548z. In order to eliminate this type of ambiguity, we mapped the sequence reads back to pre-miRNAs with bowtie [30] by allowing no mismatch, as suggested previously [11,19].

In this study, we map the sequence reads to pre-miRNAs rather than the mature miRNAs. We then have additional constraint to avoid random match. Mature miRNAs have many variants with different length, named isomiR. The isomiRs shift from their corresponding miRBase reference miRNAs in terms of location. When sequence reads were mapped back to mature miRNAs, the alignment shift may result in mismatches. Therefore, in addition to the perfect match constraint, we adopted an alternative procedure. In order to exclude random match, the difference in start position between mature miRNA and mapped reads must be equal to or less than two nucleotides and the difference in the end position between mature miRNA and mapped reads must be equal to or less than five nucleotides.

Previous studies reported nucleotide additions at the 3' end of miRNAs [10-12,26,27], which may cause mismatches. Therefore, following Fernandez-Valverde's strategy [31], we trimmed the last 3' end mismatch one by one until the perfect-match reads are at least 18 nucleotides in length.

### Analyzing the classes of sequence reads

The non-miRNA sequence reads were further classified into nine classes by mapping to different data sets with bowtie [30] by allowing one-nucleotide variation. The sequences of mRNAs came from the records with NM or XM accession prefix in RefSeq 47 [32]. The sequences of tRNAs were downloaded from the Genomic tRNA database [33]. The sequences of rRNAs were provided by the silva database [34]. The sequences of snoRNAs, scaRNAs and snRNAs were downloaded from NONCODE [35]. The sequences of other ncRNAs came from the records with NR or XR accession prefix in RefSeq 47 [32]. The sequence reads not belonging to any RNA classes were uploaded to RepaetMasker for identifying repeat elements [36]. The sequence reads not belonging to any of the previous classes were classified into the unknown class.

## Examining *Drosha *and *Dicer *expression levels in liver tissues

Total RNA was isolated from the frozen tissues using a guanidium isothiocyanate/CsCl method. RNA was quantified by spectrophotometry at 260 nm. Complementary DNA (cDNA) was prepared from the 2 microgram total RNA of paired HCCs and nontumorous liver samples. One microliter reverse transcription product, 1.25 units Pro Taq polymerase (Protech Technology Enterprise, Taipei, Taiwan), Pro Taq buffer, and 200 μ M dATP, dCTP, dGTP, and dTTP (each) were mixed with primer pairs for *Dicer, Drosha, PBGD *and *S26 *in a total volume of 30 μ l. PCR was performed in an automated DNA thermal cycler, 30 cycles for *Dicer *and *Drosha*, 28 cycles for *PBDG *and *S26*, with initial heating at 94 °C for 2 minutes, followed by the Touchdown PCR: 30 or 28 cycles of 94 °C for 30 seconds, annealing for 1 minute (the annealing temperature is reduced by 1 °C per 2 cycles from 65 °C to 55 °C for 20 cycles, than constant the annealing temperature at 55°C for final 10 or 8 cycles), 72 °C for 1 minute, and final 72 °C for 10 minutes. Primers for amplified genes were as follows: Dicer-F (5'- GTACGACTACCACAAGTACTTC -3'), Dicer-R (5'- ATAGTACACCTGCCAGACTGT -3'), Drosha-F (5'-GTGCTGTCCATGCACCAGATT -3'), Drosha-R(5'-TGCATAACTCAACTGTGCAG G -3'), S26-F (5'-CCGTGCCTCCAAGATGACAAAG-3') and S26-R (5'-TGTCTGGTAACGGCAATGCGGCT-3').

## Authors' contributions

SCL conducted this analysis and wrote the draft of the manuscript. KWT, HWP and YMJ were responsible for tissues collection and performed the PCR experiments. MRH dealt with the conducted the generalized linear model test. WHL supervised this project and edited the manuscript.

## Competing interests

The authors declare that they have no competing interests.

## Funding

This work was supported by grants from National Science Council of Taiwan (NSC99-2628-B-001-009-MY3).

## Supplementary Material

Additional File 1 **RNA classes of the sequence reads in libraries**. We first mapped the sequence reads back to pre-miRNAs, followed by mapping the non-miRNA reads back to different datasets for identifying their RNA categories. Click here for file

Additional File 2 **Expression of Dorsha and Dicer in HCV-positive and HCV-negative samples**. Reverse transcription-polymerase chain reaction (RT-PCR) was used to determine the mRNA levels of *Dicer *and *Drosha *in HCCs with hepatitis C virus (HCV) infection or not and their non-tumor liver samples. *ribosomal protein S26 *was used as internal control. Click here for file

Additional File 3 **The mapping result of all miRNA reads in all libraries**. Click here for file
