# Supplementary material for: MicroRNA 3' end nucleotide modification patterns and arm selection preference in liver tissues
Source: BMC Syst Biol. 2012 Dec 12;6(Suppl 2):S14. doi: 10.1186/1752-0509-6-S2-S14 (PMC3521178; doi:10.1186/1752-0509-6-S2-S14)
Supplement: Additional File 1 — RNA classes of the sequence reads in libraries. We first mapped the sequence reads back to pre-miRNAs, followed by mapping the non-miRNA reads back to different datasets for identifying their RNA categories. [file 1752-0509-6-S2-S14-S1.pdf]

Additional file 1. RNA classes of the sequence reads in libraries. We first mapped the sequence reads back to pre-miRNAs, followed by mapping the non-miRNA reads back to different datasets for identifying their RNA categories.

Library	ALL	miRNA	mRNA	tRNA	rRNA	snoRNA	scaRNA	snRNA	other ncRNA	repeat	unknown
SRX018957	8057617	87.19%	0.76%	0.23%	1.38%	0.21%	0.01%	0.02%	6.42%	0.0002%	3.78%
SRX018958	11234315	91.16%	0.75%	0.22%	0.07%	0.41%	0.04%	0.02%	3.69%	0.0003%	3.65%
SRX018959	10035888	91.96%	0.62%	0.57%	0.08%	0.35%	0.03%	0.01%	3.23%	0.0000%	3.16%
SRX018960	8493004	87.41%	2.30%	0.40%	0.50%	0.31%	0.03%	0.01%	6.12%	0.0002%	2.92%
SRX018961	8214755	88.75%	1.38%	0.61%	0.41%	0.33%	0.07%	0.03%	4.99%	0.0003%	3.43%
SRX018962	7413793	77.80%	1.33%	0.97%	0.26%	0.26%	0.02%	0.03%	8.72%	0.0001%	10.63%
SRX018963	8214714	80.63%	0.90%	0.83%	0.25%	0.16%	0.02%	0.03%	8.17%	0.0001%	9.00%
SRX018964	8042722	82.91%	0.97%	1.17%	0.17%	0.22%	0.02%	0.02%	6.58%	0.0000%	7.94%
SRX018965	7859049	80.57%	0.85%	0.75%	0.20%	0.23%	0.04%	0.02%	8.05%	0.0001%	9.30%
SRX018966	10640841	81.61%	3.20%	2.67%	0.60%	0.47%	0.03%	0.13%	6.61%	0.0003%	4.67%
SRX018967	10979156	89.17%	0.86%	1.36%	0.14%	0.33%	0.10%	0.04%	3.61%	0.0003%	4.39%
SRX018968	5686232	64.44%	10.74%	3.16%	1.35%	0.95%	0.05%	0.45%	6.36%	0.0007%	12.51%
SRX018969	9224742	51.54%	11.54%	3.00%	4.62%	1.36%	0.06%	0.84%	9.75%	0.0005%	17.30%
SRX018970	8967260	84.95%	1.39%	2.13%	0.42%	0.43%	0.02%	0.04%	5.83%	0.0002%	4.78%
SRX018971	9102563	79.76%	3.12%	1.35%	0.13%	3.16%	0.59%	0.09%	5.64%	0.0002%	6.17%
