# Supplementary figures and images for: MicroRNA 3' end nucleotide modification patterns and arm selection preference in liver tissues
Source: BMC Syst Biol. 2012 Dec 12;6(Suppl 2):S14. doi: 10.1186/1752-0509-6-S2-S14 (PMC3521178; doi:10.1186/1752-0509-6-S2-S14)

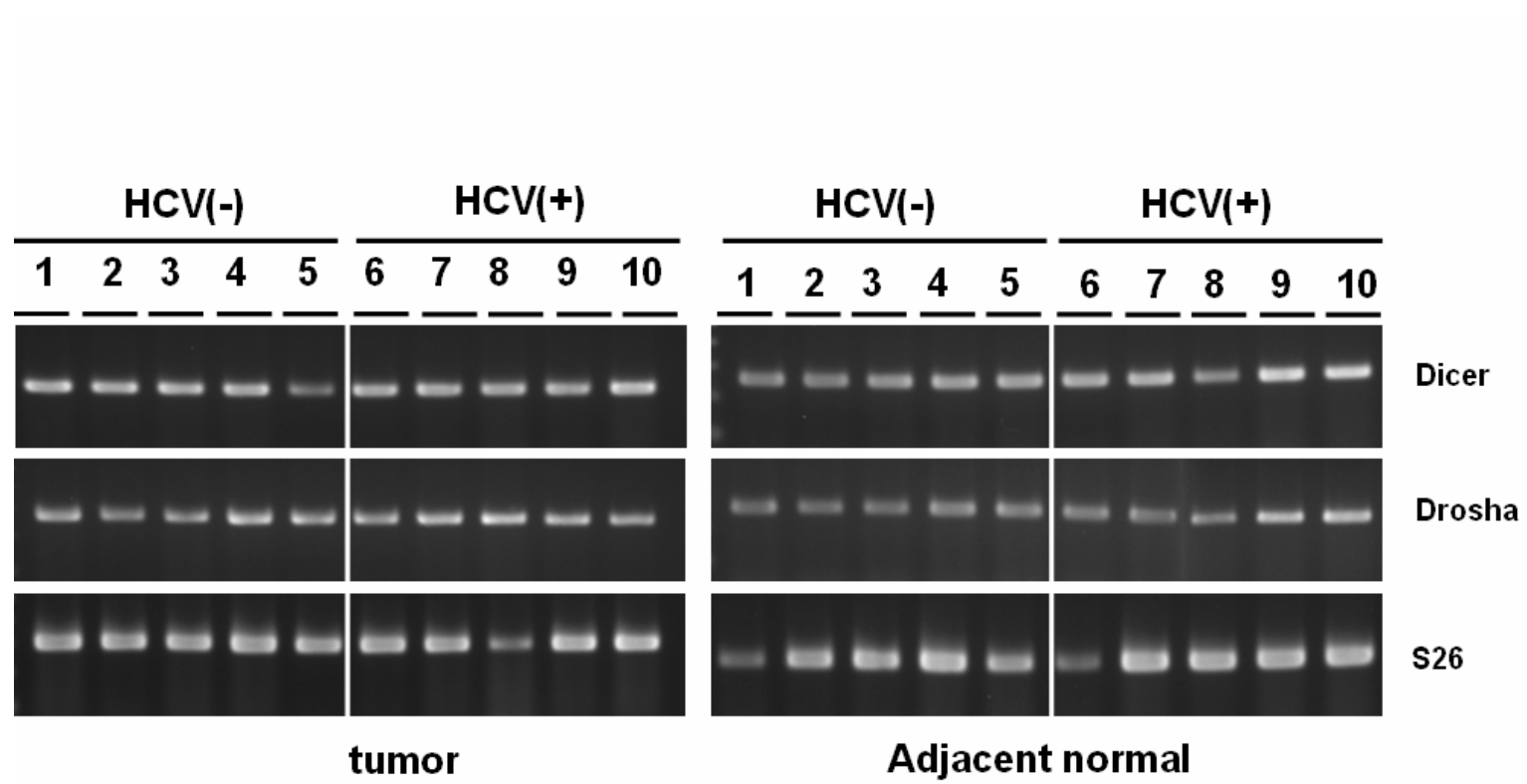

Supplement: Additional File 2 — Expression of Dorsha and Dicer in HCV-positive and HCV-negative samples. Reverse transcription-polymerase chain reaction (RT-PCR) was used to determine the mRNA levels of Dicer and Drosha in HCCs with hepatitis C virus (HCV) infection or not and their non-tumor liver samples. ribosomal protein S26 was used as internal control. [file 1752-0509-6-S2-S14-S2.pdf]
