# Supplementary material for: MicroRNA 3' end nucleotide modification patterns and arm selection preference in liver tissues
Source: BMC Syst Biol. 2012 Dec 12;6(Suppl 2):S14. doi: 10.1186/1752-0509-6-S2-S14 (PMC3521178; doi:10.1186/1752-0509-6-S2-S14)
Supplement: Additional File 3 — The mapping result of all miRNA reads in all libraries. [file 1752-0509-6-S2-S14-S3.pdf]

>hsa-let-7a-1 MA:6-27;mi:57-77 SRX018957

ugggaUGAGGUAGUAGGUUGUAUAGUUuuagggucacacccaccacugggagauaaCUAUACAAUCUACUGUCUUUCcua

(((((.(((..((((((((((((((((...........................))))))))))))))))..))))))))

----AUGAGGUAGUAGGUUGUAU---

3 -1,-4

----AUGAGGUAGUAGGUUGUAUAGUU---

3 -1,0

-----UGAGGUAGUAGGUUGUAU---

1073 0,-4

-----UGAGGUAGUAGGUUGUAUu--

77 0,-4

-----UGAGGUAGUAGGUUGUAUuu---

34 0,-4

-----UGAGGUAGUAGGUUGUAUA--

1552 0,-3

-----UGAGGUAGUAGGUUGUAUAa---

342 0,-3

-----UGAGGUAGUAGGUUGUAUAaa--

82 0,-3

-----UGAGGUAGUAGGUUGUAUAau--

5 0,-3

-----UGAGGUAGUAGGUUGUAUAu---

118 0,-3

-----UGAGGUAGUAGGUUGUAUAuu--

17 0,-3

-----UGAGGUAGUAGGUUGUAUAG---

6252 0,-2

-----UGAGGUAGUAGGUUGUAUAGa--

526 0,-2

-----UGAGGUAGUAGGUUGUAUAGaa---

107 0,-2

-----UGAGGUAGUAGGUUGUAUAGau---

21 0,-2

-----UGAGGUAGUAGGUUGUAUAGU--

42284 0,-1

-----UGAGGUAGUAGGUUGUAUAGUa---

1970 0,-1

-----UGAGGUAGUAGGUUGUAUAGUaa--

2371 0,-1

-----UGAGGUAGUAGGUUGUAUAGUau--

541 0,-1

-----UGAGGUAGUAGGUUGUAUAGUU---

93028 0,0

-----UGAGGUAGUAGGUUGUAUAGUUa--

19250 0,0

-----UGAGGUAGUAGGUUGUAUAGUUaa---

2679 0,0

-----UGAGGUAGUAGGUUGUAUAGUUau---

881 0,0

-----UGAGGUAGUAGGUUGUAUAGUUU--

9042 0,1

-----UGAGGUAGUAGGUUGUAUAGUUUU---

145 0,2

-----UGAGGUAGUAGGUUGUAUAGUUUUu--

2 0,2

------GAGGUAGUAGGUUGUAUAG---

11 1,-2

------GAGGUAGUAGGUUGUAUAGa--

2 1,-2

------GAGGUAGUAGGUUGUAUAGU--

61 1,-1

------GAGGUAGUAGGUUGUAUAGUa---

4 1,-1

------GAGGUAGUAGGUUGUAUAGUaa--

4 1,-1

------GAGGUAGUAGGUUGUAUAGUau--

2 1,-1

------GAGGUAGUAGGUUGUAUAGUU---

98 1,0

------GAGGUAGUAGGUUGUAUAGUUa--

23 1,0

------GAGGUAGUAGGUUGUAUAGUUaa---

7 1,0

------GAGGUAGUAGGUUGUAUAGUUU--

16 1,1

-------AGGUAGUAGGUUGUAUAGU--

7   2,-1

-------AGGUAGUAGGUUGUAUAGUU---

27 2,0

-------AGGUAGUAGGUUGUAUAGUUa--

3   2,0

-------AGGUAGUAGGUUGUAUAGUUU--

10 2,1

--CUAUACAAUCUACUGUCUUUC---

2   0,0

--CUAUACAAUCUACUGUCUUUCu--

2   0,0

>hsa-let-7a-2   MA:5-26;mi:50-71   SRX018957

agguUGAGGUAGUAGGUUGUAUAGUUuagaauuacaucaagggagauaaCUGUACAGCCUCCUAGCUUUCCu

(((..(((.(((.(((((((((((((.....(..(.....)..)...))))))))))))).))).))).)))

---UUGAGGUAGUAGGUUGUA---   2

-1,-5

---UUGAGGUAGUAGGUUGUAUAG---   3

-1,-2

---UUGAGGUAGUAGGUUGUAUAGU--   6

-1,-1

----UGAGGUAGUAGGUUGUAU--   1073

0,-4

----UGAGGUAGUAGGUUGUAUu---   77

0,-4

----UGAGGUAGUAGGUUGUAUuu--   34

0,-4

----UGAGGUAGUAGGUUGUAUA---   1552

0,-3

----UGAGGUAGUAGGUUGUAUAa--   342

0,-3

----UGAGGUAGUAGGUUGUAUAaa---   82

0,-3

----UGAGGUAGUAGGUUGUAUAau---   5

0,-3

----UGAGGUAGUAGGUUGUAUAu--   118

0,-3

----UGAGGUAGUAGGUUGUAUAuu---   17

0,-3
----UGAGGUAGUAGGUUGUAUAG-- 6252
0,-2
----UGAGGUAGUAGGUUGUAUAGa--- 526
0,-2
----UGAGGUAGUAGGUUGUAUAGaa-- 107
0,-2
----UGAGGUAGUAGGUUGUAUAGau-- 21
0,-2
----UGAGGUAGUAGGUUGUAUAGU---
42284 0,-1
----UGAGGUAGUAGGUUGUAUAGUa-- 1970
0,-1
----UGAGGUAGUAGGUUGUAUAGUaa--- 2371
0,-1
----UGAGGUAGUAGGUUGUAUAGUau--- 541
0,-1
----UGAGGUAGUAGGUUGUAUAGUU--
93028 0,0
----UGAGGUAGUAGGUUGUAUAGUUa---
19250 0,0
----UGAGGUAGUAGGUUGUAUAGUUaa-- 2679
0,0
----UGAGGUAGUAGGUUGUAUAGUUau-- 881
0,0
----UGAGGUAGUAGGUUGUAUAGUUU--- 9042
0,1
----UGAGGUAGUAGGUUGUAUAGUUUA-- 210
0,2
----UGAGGUAGUAGGUUGUAUAGUUUAa--- 19
0,2
----UGAGGUAGUAGGUUGUAUAGUUUAu--- 5
0,2
----UGAGGUAGUAGGUUGUAUAGUUUAG--- 3
0,3
-----GAGGUAGUAGGUUGUAUAG-- 11
1,-2
-----GAGGUAGUAGGUUGUAUAGa--- 2

1,-2

-----GAGGUAGUAGGUUGUAUAGU--- 61

1,-1

-----GAGGUAGUAGGUUGUAUAGUa-- 4

1,-1

-----GAGGUAGUAGGUUGUAUAGUaa--- 4

1,-1

-----GAGGUAGUAGGUUGUAUAGUau--- 2

1,-1

-----GAGGUAGUAGGUUGUAUAGUU-- 98

1,0

-----GAGGUAGUAGGUUGUAUAGUUa--- 23

1,0

-----GAGGUAGUAGGUUGUAUAGUUaa-- 7

1,0

-----GAGGUAGUAGGUUGUAUAGUUU--- 16

1,1

------AGGUAGUAGGUUGUAUAGU--- 7

2,-1

------AGGUAGUAGGUUGUAUAGUU-- 27

2,0

------AGGUAGUAGGUUGUAUAGUUa--- 3

2,0

------AGGUAGUAGGUUGUAUAGUUU--- 10

2,1

>hsa-let-7a-3 MA:4-25;mi:52-72 SRX018957

gggUGAGGUAGUAGGUUGUAUAGUUuggggcucugcccugcuaugggauaaCUAUACAAUCUACUGUCUUUCcu

(((.(((..((((((((((((((((((((((...))))))).........))))))))))))))))..))).)))

--GUGAGGUAGUAGGUUGUAUAGU-- 29

-1,-1

--GUGAGGUAGUAGGUUGUAUAGUU--- 7

-1,0

--GUGAGGUAGUAGGUUGUAUAGUUUu--- 2

-1,1

---UGAGGUAGUAGGUUGUAU--- 1073

0,-4

---UGAGGUAGUAGGUUGUAUu-- 77

0,-4

---UGAGGUAGUAGGUUGUAUuu--- 34

0,-4

---UGAGGUAGUAGGUUGUAUA--- 1552

0,-3

---UGAGGUAGUAGGUUGUAUAa-- 342

0,-3

---UGAGGUAGUAGGUUGUAUAaa--- 82

0,-3

---UGAGGUAGUAGGUUGUAUAau--- 5

0,-3

---UGAGGUAGUAGGUUGUAUAu-- 118

0,-3

---UGAGGUAGUAGGUUGUAUAuu--- 17

0,-3

---UGAGGUAGUAGGUUGUAUAG-- 6252

0,-2

---UGAGGUAGUAGGUUGUAUAGa--- 526

0,-2

---UGAGGUAGUAGGUUGUAUAGaa-- 107

0,-2

---UGAGGUAGUAGGUUGUAUAGau-- 21

0,-2

---UGAGGUAGUAGGUUGUAUAGU---

42284 0,-1

---UGAGGUAGUAGGUUGUAUAGUa-- 1970

0,-1

---UGAGGUAGUAGGUUGUAUAGUaa--- 2371

0,-1

---UGAGGUAGUAGGUUGUAUAGUau--- 541

0,-1

---UGAGGUAGUAGGUUGUAUAGUU--

93028 0,0

---UGAGGUAGUAGGUUGUAUAGUUa---

19250 0,0

---UGAGGUAGUAGGUUGUAUAGUUaa-- 2679

0,0

---UGAGGUAGUAGGUUGUAUAGUUau-- 881

0,0

---UGAGGUAGUAGGUUGUAUAGUUU-- 9042

0,1

---UGAGGUAGUAGGUUGUAUAGUUUG--- 16

0,2

----GAGGUAGUAGGUUGUAUAG--- 11

1,-2

----GAGGUAGUAGGUUGUAUAGa-- 2

1,-2

----GAGGUAGUAGGUUGUAUAGU-- 61

1,-1

----GAGGUAGUAGGUUGUAUAGUa--- 4

1,-1

----GAGGUAGUAGGUUGUAUAGUaa-- 4

1,-1

----GAGGUAGUAGGUUGUAUAGUau-- 2

1,-1

----GAGGUAGUAGGUUGUAUAGUU--- 98

1,0

----GAGGUAGUAGGUUGUAUAGUUa-- 23

1,0

----GAGGUAGUAGGUUGUAUAGUUaa--- 7

1,0

----GAGGUAGUAGGUUGUAUAGUUU-- 16

1,1

-----AGGUAGUAGGUUGUAUAGU-- 7

2,-1

-----AGGUAGUAGGUUGUAUAGUU--- 27

2,0

-----AGGUAGUAGGUUGUAUAGUUa-- 3

2,0

-----AGGUAGUAGGUUGUAUAGUUU-- 10

2,1

---CUAUACAAUCUACUGUCUUUC-- 2

0,0

---CUAUACAAUCUACUGUCUUUCu- 2

0,0

>hsa-let-7b MA:6-27;mi:60-81 SRX018957
cggggUGAGGUAGUAGGUUGUGUGGUUucagggcagugauguugccccucggaagauaaCUAUACAACCUACUGCCUUCC
Cug
(((((.((((((((((((((((((((...........................)))))))))))))))))))))))
)))
-----UGAGGUAGUAGGUUGUGU---
--- 6021 0,-4
-----UGAGGUAGUAGGUUGUGUa--
--- 102 0,-4
-----UGAGGUAGUAGGUUGUGUaa---
--- 330 0,-4
-----UGAGGUAGUAGGUUGUGUau---
--- 51 0,-4
-----UGAGGUAGUAGGUUGUGUu--
--- 26 0,-4
-----UGAGGUAGUAGGUUGUGUuu---
--- 11 0,-4
-----UGAGGUAGUAGGUUGUGUG--
--- 17429 0,-3
-----UGAGGUAGUAGGUUGUGUGa---
--- 316 0,-3
-----UGAGGUAGUAGGUUGUGUGaa--
--- 38 0,-3
-----UGAGGUAGUAGGUUGUGUGau--
--- 6 0,-3
-----UGAGGUAGUAGGUUGUGUGu---
--- 345 0,-3
-----UGAGGUAGUAGGUUGUGUGuu--
--- 55 0,-3
-----UGAGGUAGUAGGUUGUGUGG---
--- 11765 0,-2
-----UGAGGUAGUAGGUUGUGUGGa--
--- 428 0,-2
-----UGAGGUAGUAGGUUGUGUGGaa---
--- 126 0,-2
-----UGAGGUAGUAGGUUGUGUGGau---
--- 63 0,-2
-----UGAGGUAGUAGGUUGUGUGGU--

--- 19264 0,-1
-----UGAGGUAGUAGGUUGUGUGGUa---
--- 3627 0,-1
-----UGAGGUAGUAGGUUGUGUGGUaa--
--- 7695 0,-1
-----UGAGGUAGUAGGUUGUGUGGUau--
--- 11041 0,-1
-----UGAGGUAGUAGGUUGUGUGGUU---
--- 51825 0,0
-----UGAGGUAGUAGGUUGUGUGGUUa--
--- 25419 0,0
-----UGAGGUAGUAGGUUGUGUGGUUaa---
--- 5837 0,0
-----UGAGGUAGUAGGUUGUGUGGUUau---
--- 9693 0,0
-----UGAGGUAGUAGGUUGUGUGGUUU--
--- 16818 0,1
-----UGAGGUAGUAGGUUGUGUGGUUUa---
--- 1809 0,1
-----UGAGGUAGUAGGUUGUGUGGUUUaa--
--- 357 0,1
-----UGAGGUAGUAGGUUGUGUGGUUUau--
--- 119 0,1
-----UGAGGUAGUAGGUUGUGUGGUUUu---
--- 856 0,1
-----UGAGGUAGUAGGUUGUGUGGUUUuu--
--- 20 0,1
-----UGAGGUAGUAGGUUGUGUGGUUUC---
--- 77 0,2
-----UGAGGUAGUAGGUUGUGUGGUUUCu--
--- 2 0,2
------GAGGUAGUAGGUUGUGUG--
--- 38 1,-3
------GAGGUAGUAGGUUGUGUGG---
--- 19 1,-2
------GAGGUAGUAGGUUGUGUGGU--
--- 24 1,-1
------GAGGUAGUAGGUUGUGUGGUa---

--- 8 1,-1
------GAGGUAGUAGGUUGUGUGGUaa--
--- 24 1,-1
------GAGGUAGUAGGUUGUGUGGUau--
--- 42 1,-1
------GAGGUAGUAGGUUGUGUGGUU---
--- 89 1,0
------GAGGUAGUAGGUUGUGUGGUUa--
--- 80 1,0
------GAGGUAGUAGGUUGUGUGGUUaa---
--- 16 1,0
------GAGGUAGUAGGUUGUGUGGUUau---
--- 33 1,0
------GAGGUAGUAGGUUGUGUGGUUU--
--- 94 1,1
------GAGGUAGUAGGUUGUGUGGUUUa---
--- 8 1,1
------GAGGUAGUAGGUUGUGUGGUUUaa--
--- 2 1,1
------GAGGUAGUAGGUUGUGUGGUUUu---
--- 2 1,1
-------AGGUAGUAGGUUGUGUGG---
--- 2 2,-2
-------AGGUAGUAGGUUGUGUGGU--
--- 4 2,-1
-------AGGUAGUAGGUUGUGUGGUaa--
--- 2 2,-1
-------AGGUAGUAGGUUGUGUGGUau--
--- 3 2,-1
-------AGGUAGUAGGUUGUGUGGUU---
--- 9 2,0
-------AGGUAGUAGGUUGUGUGGUUa--
--- 9 2,0
-------AGGUAGUAGGUUGUGUGGUUau---
--- 5 2,0
-------AGGUAGUAGGUUGUGUGGUUU--
--- 7 2,1
---CUAUACAACCUACUGCCUUC-

--- 3   0,-2
---CUAUACAACCUACUGCCUUCC
--- 7   0,-1
---CUAUACAACCUACUGCCUUCC
a-- 5   0,-1
---CUAUACAACCUACUGCCUUCC
u-- 4   0,-1

>hsa-let-7c MA:11-32;mi:33-54   SRX018957
gcauccggguUGAGGUAGUAGGUUGUAUGGUUUAGAGUUACACCCUGGGAGUUAacuguacaaccuucuagcuuuccuug
gagc
((.((((((..(((.(((.(((((((((((((..((.(..(.....)..).))))))))))))))).))).)))..))))
))))
--------GUUGAGGUAGUAGGUUGUAUGGU---
----   7   -2,-1
--------GUUGAGGUAGUAGGUUGUAUGGUU--
----   2   -2,0
---------UUGAGGUAGUAGGUUGUA---
----   2   -1,-5
---------UUGAGGUAGUAGGUUGUAUG---
----   4   -1,-3
---------UUGAGGUAGUAGGUUGUAUGG--
----   32 -1,-2
---------UUGAGGUAGUAGGUUGUAUGGaa--
----   2   -1,-2
---------UUGAGGUAGUAGGUUGUAUGGU---
----   223 -1,-1
---------UUGAGGUAGUAGGUUGUAUGGUa--
----   93 -1,-1
---------UUGAGGUAGUAGGUUGUAUGGUaa---
----   7   -1,-1
---------UUGAGGUAGUAGGUUGUAUGGUau---
----   33 -1,-1
---------UUGAGGUAGUAGGUUGUAUGGUU--
----   364 -1,0
---------UUGAGGUAGUAGGUUGUAUGGUUa---
----   8   -1,0
---------UUGAGGUAGUAGGUUGUAUGGUUau--

---- 4 -1,0
---------UUGAGGUAGUAGGUUGUAUGGUUU---
---- 19 -1,1
---------UUGAGGUAGUAGGUUGUAUGGUUUA--
---- 2 -1,2
----------UGAGGUAGUAGGUUGUAU--
---- 1073 0,-4
----------UGAGGUAGUAGGUUGUAUu---
---- 77 0,-4
----------UGAGGUAGUAGGUUGUAUuu--
---- 34 0,-4
----------UGAGGUAGUAGGUUGUAUG---
---- 2023 0,-3
----------UGAGGUAGUAGGUUGUAUGa--
---- 25 0,-3
----------UGAGGUAGUAGGUUGUAUGaa---
---- 6 0,-3
----------UGAGGUAGUAGGUUGUAUGau---
---- 3 0,-3
----------UGAGGUAGUAGGUUGUAUGu--
---- 49 0,-3
----------UGAGGUAGUAGGUUGUAUGuu---
---- 29 0,-3
----------UGAGGUAGUAGGUUGUAUGG--
---- 5482 0,-2
----------UGAGGUAGUAGGUUGUAUGGa---
---- 223 0,-2
----------UGAGGUAGUAGGUUGUAUGGaa--
---- 240 0,-2
----------UGAGGUAGUAGGUUGUAUGGau--
---- 38 0,-2
----------UGAGGUAGUAGGUUGUAUGGU---
---- 22597 0,-1
----------UGAGGUAGUAGGUUGUAUGGUa--
---- 6048 0,-1
----------UGAGGUAGUAGGUUGUAUGGUaa---
---- 4148 0,-1
----------UGAGGUAGUAGGUUGUAUGGUau---

----	2882	0,-1
----------UGAGGUAGUAGGUUGUAUGGUU--
----	104725	0,0
----------UGAGGUAGUAGGUUGUAUGGUUa---
----	17291	0,0
----------UGAGGUAGUAGGUUGUAUGGUUaa--
----	2308	0,0
----------UGAGGUAGUAGGUUGUAUGGUUau--
----	2257	0,0
----------UGAGGUAGUAGGUUGUAUGGUUU---
----	10940	0,1
----------UGAGGUAGUAGGUUGUAUGGUUUu--
----	216	0,1
----------UGAGGUAGUAGGUUGUAUGGUUUuu---
----	3	0,1
----------UGAGGUAGUAGGUUGUAUGGUUUA--
----	453	0,2
----------UGAGGUAGUAGGUUGUAUGGUUUAa---
----	81	0,2
----------UGAGGUAGUAGGUUGUAUGGUUUAu---
----	13	0,2
----------UGAGGUAGUAGGUUGUAUGGUUUAG---
----	6	0,3
-----------GAGGUAGUAGGUUGUAUG---
----	16	1,-3
-----------GAGGUAGUAGGUUGUAUGG--
----	15	1,-2
-----------GAGGUAGUAGGUUGUAUGGU---
----	68	1,-1
-----------GAGGUAGUAGGUUGUAUGGUa--
----	28	1,-1
-----------GAGGUAGUAGGUUGUAUGGUaa---
----	20	1,-1
-----------GAGGUAGUAGGUUGUAUGGUau---
----	11	1,-1
-----------GAGGUAGUAGGUUGUAUGGUU--
----	411	1,0
-----------GAGGUAGUAGGUUGUAUGGUUa---

-----------GAGGUAGUAGGUUGUAUGGUUaa--
----   82 1,0
-----------GAGGUAGUAGGUUGUAUGGUUau--
----   8   1,0
-----------GAGGUAGUAGGUUGUAUGGUUU---
----   9   1,0
-----------GAGGUAGUAGGUUGUAUGGUUUA--
----   71 1,1
------------AGGUAGUAGGUUGUAUGG--
----   2   1,2
------------AGGUAGUAGGUUGUAUGGU---
----   2   2,-2
------------AGGUAGUAGGUUGUAUGGUU--
----   3   2,-1
------------AGGUAGUAGGUUGUAUGGUUa---
----   27 2,0
------------AGGUAGUAGGUUGUAUGGUUU---
----   6   2,0
----   7   2,1

>hsa-let-7d MA:8-29;mi:62-83   SRX018957
ccuaggaAGAGGUAGUAGGUUGCAUAGUUuuagggcagggauuuugcccacaaggagguaaCUAUACGACCUGCUGCCUU
UCUuagg
(((((((.((((((((((((((.((((((..............................)))))).))))))))))))))
)))))))
------AAGAGGUAGUAGGUUGCAUAGU--
------- 3   -1,-1
------AAGAGGUAGUAGGUUGCAUAGUU---
------- 5   -1,0
-------AGAGGUAGUAGGUUGCAU---
------- 463 0,-4
-------AGAGGUAGUAGGUUGCAUu--
------- 36 0,-4
-------AGAGGUAGUAGGUUGCAUuu---
------- 35 0,-4
-------AGAGGUAGUAGGUUGCAUA--
------- 375 0,-3
-------AGAGGUAGUAGGUUGCAUAa---

------- 57 0,-3
-------AGAGGUAGUAGGUUGCAUAaa--
------- 6 0,-3
-------AGAGGUAGUAGGUUGCAUAau--
------- 2 0,-3
-------AGAGGUAGUAGGUUGCAUAu---
------- 11 0,-3
-------AGAGGUAGUAGGUUGCAUAG---
------- 1123 0,-2
-------AGAGGUAGUAGGUUGCAUAGa--
------- 44 0,-2
-------AGAGGUAGUAGGUUGCAUAGaa---
------- 26 0,-2
-------AGAGGUAGUAGGUUGCAUAGau---
------- 15 0,-2
-------AGAGGUAGUAGGUUGCAUAGU--
------- 2463 0,-1
-------AGAGGUAGUAGGUUGCAUAGUa---
------- 747 0,-1
-------AGAGGUAGUAGGUUGCAUAGUaa--
------- 158 0,-1
-------AGAGGUAGUAGGUUGCAUAGUau--
------- 86 0,-1
-------AGAGGUAGUAGGUUGCAUAGUU---
------- 33544 0,0
-------AGAGGUAGUAGGUUGCAUAGUUa--
------- 4451 0,0
-------AGAGGUAGUAGGUUGCAUAGUUaa---
------- 170 0,0
-------AGAGGUAGUAGGUUGCAUAGUUau---
------- 146 0,0
-------AGAGGUAGUAGGUUGCAUAGUUU--
------- 6160 0,1
-------AGAGGUAGUAGGUUGCAUAGUUUa---
------- 90 0,1
-------AGAGGUAGUAGGUUGCAUAGUUUau--
------- 4 0,1
-------AGAGGUAGUAGGUUGCAUAGUUUU---

------- 309 0,2
-------AGAGGUAGUAGGUUGCAUAGUUUUu--
------- 17 0,2
--------GAGGUAGUAGGUUGCAUA--
------- 2   1,-3
--------GAGGUAGUAGGUUGCAUAG---
------- 2   1,-2
--------GAGGUAGUAGGUUGCAUAGU--
------- 12 1,-1
--------GAGGUAGUAGGUUGCAUAGUU---
------- 69 1,0
--------GAGGUAGUAGGUUGCAUAGUUa--
------- 26 1,0
--------GAGGUAGUAGGUUGCAUAGUUU--
------- 22 1,1
---------AGGUAGUAGGUUGCAUAGUU---
------- 6   2,0
---------AGGUAGUAGGUUGCAUAGUUU--
------- 2   2,1
---CUAUACGACCUGCUGCCUU
UC----- 2   0,-1
---CUAUACGACCUGCUGCCUU
UCa---- 4   0,-1
---CUAUACGACCUGCUGCCUU
UCU---- 8   0,0
--UAUACGACCUGCUGCCUU
UCa---- 2   1,-1

>hsa-let-7e MA:8-29;mi:53-74   SRX018957
cccgggcUGAGGUAGGAGGUUGUAUAGUUgaggaggacacccaaggagaucaCUAUACGGCCUCCUAGCUUUCCccagg
((.(((..(((.((((((((((((((((.((...((....)).......)))))))))))))))))).)))..))).))
------CUGAGGUAGGAGGUUGUAU--
     2   -1,-4
------CUGAGGUAGGAGGUUGUAUAGU---
     3   -1,-1
------CUGAGGUAGGAGGUUGUAUAGUU--
     9   -1,0
------CUGAGGUAGGAGGUUGUAUAGUUa---

4 -1,0

-------UGAGGUAGGAGGUUGUAU---

1887 0,-4

-------UGAGGUAGGAGGUUGUAUu--

6 0,-4

-------UGAGGUAGGAGGUUGUAUuu---

4 0,-4

-------UGAGGUAGGAGGUUGUAUA--

331 0,-3

-------UGAGGUAGGAGGUUGUAUAa---

60 0,-3

-------UGAGGUAGGAGGUUGUAUAaa--

31 0,-3

-------UGAGGUAGGAGGUUGUAUAu---

14 0,-3

-------UGAGGUAGGAGGUUGUAUAG---

922 0,-2

-------UGAGGUAGGAGGUUGUAUAGa--

112 0,-2

-------UGAGGUAGGAGGUUGUAUAGaa---

45 0,-2

-------UGAGGUAGGAGGUUGUAUAGau---

6 0,-2

-------UGAGGUAGGAGGUUGUAUAGU--

3127 0,-1

-------UGAGGUAGGAGGUUGUAUAGUa---

321 0,-1

-------UGAGGUAGGAGGUUGUAUAGUaa--

632 0,-1

-------UGAGGUAGGAGGUUGUAUAGUau--

198 0,-1

-------UGAGGUAGGAGGUUGUAUAGUU---

5768 0,0

-------UGAGGUAGGAGGUUGUAUAGUUa--

3173 0,0

-------UGAGGUAGGAGGUUGUAUAGUUaa---

515 0,0

-------UGAGGUAGGAGGUUGUAUAGUUau---

157 0,0

-------UGAGGUAGGAGGUUGUAUAGUUu--

1180 0,0

-------UGAGGUAGGAGGUUGUAUAGUUuu---

11 0,0

-------UGAGGUAGGAGGUUGUAUAGUUG--

764 0,1

-------UGAGGUAGGAGGUUGUAUAGUUGu---

92 0,1

-------UGAGGUAGGAGGUUGUAUAGUUGA---

61 0,2

--------GAGGUAGGAGGUUGUAUAGU--

3 1,-1

--------GAGGUAGGAGGUUGUAUAGUaa--

2 1,-1

--------GAGGUAGGAGGUUGUAUAGUU---

3 1,0

--------GAGGUAGGAGGUUGUAUAGUUa--

3 1,0

--------GAGGUAGGAGGUUGUAUAGUUG--

2 1,1

---CUAUACGGCCUCCUAGCUUUC------

2 0,-1

---CUAUACGGCCUCCUAGCUUUCCa----

2 0,0

>hsa-let-7f-1 MA:7-28;mi:63-84 SRX018957

ucagagUGAGGUAGUAGAUUGUAUAGUUgugggguagugauuuuacccuguucaggagauaaCUAUACAAUCUAUUGCCU
UCCCuga

((((.(..(((((((((((((((((((((((((((((.....))))))).........))))))))))))))))))))))
..)))))

------UGAGGUAGUAGAUUGUAU--
------- 466 0,-4

------UGAGGUAGUAGAUUGUAUu---
------- 11 0,-4

------UGAGGUAGUAGAUUGUAUuu--
------- 14 0,-4

------UGAGGUAGUAGAUUGUAUA---

------- 1270	0,-3
------UGAGGUAGUAGAUUGUAUAa--
------- 71	0,-3
------UGAGGUAGUAGAUUGUAUAaa---
------- 31	0,-3
------UGAGGUAGUAGAUUGUAUAau---
------- 2	0,-3
------UGAGGUAGUAGAUUGUAUAu--
------- 3	0,-3
------UGAGGUAGUAGAUUGUAUAuu---
------- 4	0,-3
------UGAGGUAGUAGAUUGUAUAG--
------- 1800	0,-2
------UGAGGUAGUAGAUUGUAUAGa---
------- 183	0,-2
------UGAGGUAGUAGAUUGUAUAGaa--
------- 28	0,-2
------UGAGGUAGUAGAUUGUAUAGau--
------- 7	0,-2
------UGAGGUAGUAGAUUGUAUAGU---
------- 13328	0,-1
------UGAGGUAGUAGAUUGUAUAGUa--
------- 706	0,-1
------UGAGGUAGUAGAUUGUAUAGUaa---
------- 2057	0,-1
------UGAGGUAGUAGAUUGUAUAGUau---
------- 295	0,-1
------UGAGGUAGUAGAUUGUAUAGUU--
------- 23633	0,0
------UGAGGUAGUAGAUUGUAUAGUUa---
------- 20008	0,0
------UGAGGUAGUAGAUUGUAUAGUUaa--
------- 1837	0,0
------UGAGGUAGUAGAUUGUAUAGUUau--
------- 371	0,0
------UGAGGUAGUAGAUUGUAUAGUUG---
------- 1119	0,1
------UGAGGUAGUAGAUUGUAUAGUUGa--

------- 58   0,1
------UGAGGUAGUAGAUUGUAUAGUUGaa---
------- 3   0,1
------UGAGGUAGUAGAUUGUAUAGUUGU--
------- 54   0,2
-------GAGGUAGUAGAUUGUAUA---
------- 5   1,-3
-------GAGGUAGUAGAUUGUAUAGU---
------- 27   1,-1
-------GAGGUAGUAGAUUGUAUAGUa--
------- 2   1,-1
-------GAGGUAGUAGAUUGUAUAGUaa---
------- 4   1,-1
-------GAGGUAGUAGAUUGUAUAGUau---
------- 2   1,-1
-------GAGGUAGUAGAUUGUAUAGUU--
------- 39   1,0
-------GAGGUAGUAGAUUGUAUAGUUa---
------- 45   1,0
-------GAGGUAGUAGAUUGUAUAGUUaa--
------- 6   1,0
--------AGGUAGUAGAUUGUAUAGUU--
------- 5   2,0
--------AGGUAGUAGAUUGUAUAGUUa---
------- 4   2,0
--------AGGUAGUAGAUUGUAUAGUUG---
------- 5   2,1
--------AGGUAGUAGAUUGUAUAGUUGU--
------- 8   2,2

>hsa-let-7f-2   MA:8-29;mi:58-79   SRX018957
ugugggaUGAGGUAGUAGAUUGUAUAGUUuuagggucauaccccaucuuggagauaaCUAUACAGUCUACUGUCUUUCCc
acg
.((((((.(((..((((((((((((((((..........................))))))))))))))))..)))))))
)).
------AUGAGGUAGUAGAUUGUAU---
--- 3   -1,-4
------AUGAGGUAGUAGAUUGUAUAGU--

--- 16 -1,-1
------AUGAGGUAGUAGAUUGUAUAGUaa--
--- 2 -1,-1
------AUGAGGUAGUAGAUUGUAUAGUU---
--- 7 -1,0
-------UGAGGUAGUAGAUUGUAU---
--- 466 0,-4
-------UGAGGUAGUAGAUUGUAUu--
--- 11 0,-4
-------UGAGGUAGUAGAUUGUAUuu---
--- 14 0,-4
-------UGAGGUAGUAGAUUGUAUA--
--- 1270 0,-3
-------UGAGGUAGUAGAUUGUAUAa---
--- 71 0,-3
-------UGAGGUAGUAGAUUGUAUAaa--
--- 31 0,-3
-------UGAGGUAGUAGAUUGUAUAau--
--- 2 0,-3
-------UGAGGUAGUAGAUUGUAUAu---
--- 3 0,-3
-------UGAGGUAGUAGAUUGUAUAuu--
--- 4 0,-3
-------UGAGGUAGUAGAUUGUAUAG---
--- 1800 0,-2
-------UGAGGUAGUAGAUUGUAUAGa--
--- 183 0,-2
-------UGAGGUAGUAGAUUGUAUAGaa---
--- 28 0,-2
-------UGAGGUAGUAGAUUGUAUAGau---
--- 7 0,-2
-------UGAGGUAGUAGAUUGUAUAGU--
--- 13328 0,-1
-------UGAGGUAGUAGAUUGUAUAGUa---
--- 706 0,-1
-------UGAGGUAGUAGAUUGUAUAGUaa--
--- 2057 0,-1
-------UGAGGUAGUAGAUUGUAUAGUau--

--- 295 0,-1

-------UGAGGUAGUAGAUUGUAUAGUU---

--- 23633 0,0

-------UGAGGUAGUAGAUUGUAUAGUUa--

--- 20008 0,0

-------UGAGGUAGUAGAUUGUAUAGUUaa---

--- 1837 0,0

-------UGAGGUAGUAGAUUGUAUAGUUau---

--- 371 0,0

-------UGAGGUAGUAGAUUGUAUAGUUU--

--- 4565 0,1

-------UGAGGUAGUAGAUUGUAUAGUUUa---

--- 42 0,1

-------UGAGGUAGUAGAUUGUAUAGUUUaa--

--- 4 0,1

-------UGAGGUAGUAGAUUGUAUAGUUUau--

--- 2 0,1

-------UGAGGUAGUAGAUUGUAUAGUUUU---

--- 29 0,2

--------GAGGUAGUAGAUUGUAUA--

--- 5 1,-3

--------GAGGUAGUAGAUUGUAUAGU--

--- 27 1,-1

--------GAGGUAGUAGAUUGUAUAGUa---

--- 2 1,-1

--------GAGGUAGUAGAUUGUAUAGUaa--

--- 4 1,-1

--------GAGGUAGUAGAUUGUAUAGUau--

--- 2 1,-1

--------GAGGUAGUAGAUUGUAUAGUU---

--- 39 1,0

--------GAGGUAGUAGAUUGUAUAGUUa--

--- 45 1,0

--------GAGGUAGUAGAUUGUAUAGUUaa---

--- 6 1,0

--------GAGGUAGUAGAUUGUAUAGUUU--

--- 7 1,1

---------AGGUAGUAGAUUGUAUAGUU---

--- 5	2,0
---------AGGUAGUAGAUUGUAUAGUUa--
--- 4	2,0
---------AGGUAGUAGAUUGUAUAGUUU--
--- 2	2,1

>hsa-let-7g MA:5-26;mi:62-82	SRX018957
aggcUGAGGUAGUAGUUUGUACAGUUugagggucuaugauaccacccgguacaggagauaaCUGUACAGGCCACUGCCUU
GCca
.(((.((((((((.(((((((((((.....((((.((.((((....))))))..)))))))))))))))).))))))))
))).
---CUGAGGUAGUAGUUUGUACAG--
----	3	-1,-2
---CUGAGGUAGUAGUUUGUACAGU---
----	13	-1,-1
---CUGAGGUAGUAGUUUGUACAGUa--
----	4	-1,-1
---CUGAGGUAGUAGUUUGUACAGUU--
----	27	-1,0
---CUGAGGUAGUAGUUUGUACAGUUa---
----	5	-1,0
---CUGAGGUAGUAGUUUGUACAGUUU---
----	3	-1,1
----UGAGGUAGUAGUUUGUAC--
----	46	0,-4
----UGAGGUAGUAGUUUGUACu---
----	2	0,-4
----UGAGGUAGUAGUUUGUACA---
----	60	0,-3
----UGAGGUAGUAGUUUGUACAa--
----	27	0,-3
----UGAGGUAGUAGUUUGUACAaa---
----	7	0,-3
----UGAGGUAGUAGUUUGUACAG--
----	580	0,-2
----UGAGGUAGUAGUUUGUACAGa---
----	40	0,-2
----UGAGGUAGUAGUUUGUACAGaa--

---- 8 0,-2
----UGAGGUAGUAGUUUGUACAGU--
---- 3349 0,-1
----UGAGGUAGUAGUUUGUACAGUa---
---- 72 0,-1
----UGAGGUAGUAGUUUGUACAGUaa--
---- 43 0,-1
----UGAGGUAGUAGUUUGUACAGUau--
---- 24 0,-1
----UGAGGUAGUAGUUUGUACAGUU---
---- 6213 0,0
----UGAGGUAGUAGUUUGUACAGUUa--
---- 1339 0,0
----UGAGGUAGUAGUUUGUACAGUUaa---
---- 95 0,0
----UGAGGUAGUAGUUUGUACAGUUau---
---- 98 0,0
----UGAGGUAGUAGUUUGUACAGUUU--
---- 1267 0,1
----UGAGGUAGUAGUUUGUACAGUUUa---
---- 31 0,1
----UGAGGUAGUAGUUUGUACAGUUUu---
---- 36 0,1
----UGAGGUAGUAGUUUGUACAGUUUG---
---- 4 0,2
-----GAGGUAGUAGUUUGUACAG---
---- 5 1,-2
-----GAGGUAGUAGUUUGUACAGU--
---- 31 1,-1
-----GAGGUAGUAGUUUGUACAGUa---
---- 2 1,-1
-----GAGGUAGUAGUUUGUACAGUU---
---- 46 1,0
-----GAGGUAGUAGUUUGUACAGUUa--
---- 3 1,0
-----GAGGUAGUAGUUUGUACAGUUU--
---- 5 1,1
------AGGUAGUAGUUUGUACAGUU---

----   3   2,0

>hsa-let-7i MA:6-27;mi:62-83   SRX018957
cuggcUGAGGUAGUAGUUUGUGCUGUUggucggguugugacauugcccgcuguggagauaaCUGCGCAAGCUACUGCCUU
GCUa
.((((.(((((((((((((((((.(((((.(((((.........))))))).......))).)))))))))))))))))
))))
----CUGAGGUAGUAGUUUGUGCU--
----   2   -1,-3
-----UGAGGUAGUAGUUUGUGC---
----   244 0,-4
-----UGAGGUAGUAGUUUGUGCa--
----   3   0,-4
-----UGAGGUAGUAGUUUGUGCaa---
----   12 0,-4
-----UGAGGUAGUAGUUUGUGCau---
----   3   0,-4
-----UGAGGUAGUAGUUUGUGCU--
----   796 0,-3
-----UGAGGUAGUAGUUUGUGCUa---
----   3   0,-3
-----UGAGGUAGUAGUUUGUGCUu---
----   2   0,-3
-----UGAGGUAGUAGUUUGUGCUuu--
----   2   0,-3
-----UGAGGUAGUAGUUUGUGCUG---
----   604 0,-2
-----UGAGGUAGUAGUUUGUGCUGa--
----   10 0,-2
-----UGAGGUAGUAGUUUGUGCUGaa---
----   13 0,-2
-----UGAGGUAGUAGUUUGUGCUGU--
----   338 0,-1
-----UGAGGUAGUAGUUUGUGCUGUa---
----   17 0,-1
-----UGAGGUAGUAGUUUGUGCUGUaa--
----   17 0,-1
-----UGAGGUAGUAGUUUGUGCUGUau--

---- 3 0,-1
-----UGAGGUAGUAGUUUGUGCUGUU---
---- 452 0,0
-----UGAGGUAGUAGUUUGUGCUGUUa--
---- 180 0,0
-----UGAGGUAGUAGUUUGUGCUGUUaa---
---- 44 0,0
-----UGAGGUAGUAGUUUGUGCUGUUau---
---- 8 0,0
-----UGAGGUAGUAGUUUGUGCUGUUu--
---- 202 0,0
-----UGAGGUAGUAGUUUGUGCUGUUuu---
---- 24 0,0
-----UGAGGUAGUAGUUUGUGCUGUUG--
---- 9 0,1
-----UGAGGUAGUAGUUUGUGCUGUUGa---
---- 2 0,1
-----UGAGGUAGUAGUUUGUGCUGUUGu---
---- 2 0,1
------GAGGUAGUAGUUUGUGCUGUU---
---- 2 1,0
---CUGCGCAAGCUACUGCCUU
---- 2 0,-3
---CUGCGCAAGCUACUGCCUU
G--- 2 0,-2

>hsa-mir-1-1 3p:46-67 SRX018957
ugggaaacauacuucuuuauaugcccauauggaccugcuaagcuaUGGAAUGUAAAGAAGUAUGUAUcuca
.((((.(((((((((((((((((..(((((((.....)))...))))).)))))))))))))))).)))).
---UGGAAUGUAAAGAAGUAU-------- 4 0,-4
---UGGAAUGUAAAGAAGUAUG------- 14 0,-3
---UGGAAUGUAAAGAAGUAUGU------ 35 0,-2
---UGGAAUGUAAAGAAGUAUGUA----- 92 0,-1
---UGGAAUGUAAAGAAGUAUGUAa---- 38 0,-1
---UGGAAUGUAAAGAAGUAUGUAaa--- 2 0,-1
---UGGAAUGUAAAGAAGUAUGUAU---- 1147
0,0
---UGGAAUGUAAAGAAGUAUGUAUa--- 23 0,0

--GGAAUGUAAAGAAGUAUGUAU---- 3	1,0
---GAAUGUAAAGAAGUAUGUAU---- 3	2,0

>hsa-mir-1-2	3p:53-74	SRX018957
accuacucagaguacauacuucuuuauguacccauaugaacauacaaugcuaUGGAAUGUAAAGAAGUAUGUAUuuuugg
uaggc
.((((((.(((((((((((((((((((((..(((((..............))))).))))))))))))))))))))).))
)))).
--UGGAAUGUAAAGAAGUAU----------
-----	4	0,-4
--UGGAAUGUAAAGAAGUAUG---------
-----	14	0,-3
--UGGAAUGUAAAGAAGUAUGU--------
-----	35	0,-2
--UGGAAUGUAAAGAAGUAUGUA-------
-----	92	0,-1
--UGGAAUGUAAAGAAGUAUGUAa------
-----	38	0,-1
--UGGAAUGUAAAGAAGUAUGUAaa-----
-----	2	0,-1
--UGGAAUGUAAAGAAGUAUGUAU------
-----	1147	0,0
--UGGAAUGUAAAGAAGUAUGUAUa-----
-----	23	0,0
--UGGAAUGUAAAGAAGUAUGUAUU-----
-----	13	0,1
---GGAAUGUAAAGAAGUAUGUAU------
-----	3	1,0
--GAAUGUAAAGAAGUAUGUAU------
-----	3	2,0

>hsa-mir-100	MA:13-34;mi:48-69	SRX018957
ccuguugccacaAACCCGUAGAUCCGAACUUGUGguauuaguccgcaCAAGCUUGUAUCUAUAGGUAUGgugucuguuagg
((((..(.((((.(((..(((((.(((.(((((((........).)))))).))).)))))..))).)))).)...))))
------------AACCCGUAGAUCCGAACU--
	261 0,-4
------------AACCCGUAGAUCCGAACUa---
	7	0,-4

------------AACCCGUAGAUCCGAACUU---
157 0,-3
------------AACCCGUAGAUCCGAACUUa--
2 0,-3
------------AACCCGUAGAUCCGAACUUu--
3 0,-3
------------AACCCGUAGAUCCGAACUUuu---
4 0,-3
------------AACCCGUAGAUCCGAACUUG--
1049 0,-2
------------AACCCGUAGAUCCGAACUUGa---
21 0,-2
------------AACCCGUAGAUCCGAACUUGaa--
2 0,-2
------------AACCCGUAGAUCCGAACUUGU---
8973 0,-1
------------AACCCGUAGAUCCGAACUUGUa--
40 0,-1
------------AACCCGUAGAUCCGAACUUGUaa---
45 0,-1
------------AACCCGUAGAUCCGAACUUGUau---
10 0,-1
------------AACCCGUAGAUCCGAACUUGUu--
32 0,-1
------------AACCCGUAGAUCCGAACUUGUuu---
6 0,-1
------------AACCCGUAGAUCCGAACUUGUG--
7832 0,0
------------AACCCGUAGAUCCGAACUUGUGa---
6589 0,0
------------AACCCGUAGAUCCGAACUUGUGaa--
576 0,0
------------AACCCGUAGAUCCGAACUUGUGau--
34 0,0
------------AACCCGUAGAUCCGAACUUGUGu---
8632 0,0
------------AACCCGUAGAUCCGAACUUGUGuu--
61 0,0

------------AACCCGUAGAUCCGAACUUGUGG---
49 0,1
------------AACCCGUAGAUCCGAACUUGUGGU--
3 0,2
-------------ACCCGUAGAUCCGAACUUGU---
5 1,-1
-------------ACCCGUAGAUCCGAACUUGUG--
6 1,0
-------------ACCCGUAGAUCCGAACUUGUGa---
8 1,0
-------------ACCCGUAGAUCCGAACUUGUGaa--
4 1,0
-------------ACCCGUAGAUCCGAACUUGUGu---
5 1,0

>hsa-mir-101-1 mi:11-32;MA:47-67 SRX018957
ugcccuggcuCAGUUAUCACAGUGCUGAUGCUgucuauucuaaaggUACAGUACUGUGAUAACUGAAggauggca
.(((.....((((((((((((((((((.((((...........)))))))))))))))))))))).....))).
---------UCAGUUAUCACAGUGCUGAUGCU--- 2
-1,0
---GUACAGUACUGUGAUAAC------------ 161
-1,-4
---GUACAGUACUGUGAUAACU----------- 4257
-1,-3
---GUACAGUACUGUGAUAACUa---------- 12
-1,-3
---GUACAGUACUGUGAUAACUaa--------- 8
-1,-3
---GUACAGUACUGUGAUAACUau--------- 3
-1,-3
---GUACAGUACUGUGAUAACUu---------- 2
-1,-3
---GUACAGUACUGUGAUAACUG---------- 2593
-1,-2
---GUACAGUACUGUGAUAACUGu--------- 23
-1,-2
---GUACAGUACUGUGAUAACUGA---------
17005 -1,-1

--GUACAGUACUGUGAUAACUGAu-------- 70

-1,-1

--GUACAGUACUGUGAUAACUGAA--------

26914 -1,0

--GUACAGUACUGUGAUAACUGAAa------- 6060

-1,0

--GUACAGUACUGUGAUAACUGAAaa------ 256

-1,0

--GUACAGUACUGUGAUAACUGAAau------ 5

-1,0

--GUACAGUACUGUGAUAACUGAAu------- 31

-1,0

--GUACAGUACUGUGAUAACUGAAuu------ 2

-1,0

--GUACAGUACUGUGAUAACUGAAG------- 26

-1,1

--GUACAGUACUGUGAUAACUGAAGu------ 2

-1,1

---UACAGUACUGUGAUAACU----------- 5567

0,-3

---UACAGUACUGUGAUAACUa---------- 23

0,-3

---UACAGUACUGUGAUAACUaa--------- 17

0,-3

---UACAGUACUGUGAUAACUu---------- 2

0,-3

---UACAGUACUGUGAUAACUG---------- 3186

0,-2

---UACAGUACUGUGAUAACUGu--------- 28

0,-2

---UACAGUACUGUGAUAACUGA---------

31682 0,-1

---UACAGUACUGUGAUAACUGAu-------- 70

0,-1

---UACAGUACUGUGAUAACUGAuu------- 37

0,-1

---UACAGUACUGUGAUAACUGAA--------

69934 0,0

--UACAGUACUGUGAUAACUGAAa-------
17465 0,0
--UACAGUACUGUGAUAACUGAAaa------ 640
0,0
--UACAGUACUGUGAUAACUGAAau------ 40
0,0
--UACAGUACUGUGAUAACUGAAu------- 877
0,0
--UACAGUACUGUGAUAACUGAAuu------ 37
0,0
--UACAGUACUGUGAUAACUGAAG------- 3692
0,1
--UACAGUACUGUGAUAACUGAAGu------ 490
0,1
--UACAGUACUGUGAUAACUGAAGG------ 7
0,2
--UACAGUACUGUGAUAACUGAAGGA----- 6
0,3
---ACAGUACUGUGAUAACUG---------- 19
1,-2
---ACAGUACUGUGAUAACUGA--------- 199
1,-1
---ACAGUACUGUGAUAACUGAA-------- 500
1,0
---ACAGUACUGUGAUAACUGAAa------- 131
1,0
---ACAGUACUGUGAUAACUGAAaa------ 9
1,0
---ACAGUACUGUGAUAACUGAAu------- 9
1,0
---ACAGUACUGUGAUAACUGAAG------- 36
1,1
---ACAGUACUGUGAUAACUGAAGu------ 11
1,1
--CAGUACUGUGAUAACUGA--------- 36
2,-1
--CAGUACUGUGAUAACUGAA-------- 92
2,0

--CAGUACUGUGAUAACUGAAa------- 24
2,0
--CAGUACUGUGAUAACUGAAaa------ 2
2,0
--CAGUACUGUGAUAACUGAAau------ 2
2,0
--CAGUACUGUGAUAACUGAAu------- 3
2,0
--CAGUACUGUGAUAACUGAAuu------ 2
2,0
--CAGUACUGUGAUAACUGAAG------- 2
2,1
--CAGUACUGUGAUAACUGAAGu------ 2
2,1
--CAGUACUGUGAUAACUGAAGuu----- 2
2,1

>hsa-mir-101-2 3p:49-69 SRX018957
acuguccuuuuucgguuaucaugguaccgaugcuguauaucugaaaggUACAGUACUGUGAUAACUGAAgaaugguggu
((..((.((((((((((((((((((((.(.((((............))))).)))))))))))))))))))))).))..))
---GUACAGUACUGUGAUAAC--------------
161 -1,-4
---GUACAGUACUGUGAUAACU-------------
4257 -1,-3
---GUACAGUACUGUGAUAACUa------------
12 -1,-3
---GUACAGUACUGUGAUAACUaa-----------
8 -1,-3
---GUACAGUACUGUGAUAACUau-----------
3 -1,-3
---GUACAGUACUGUGAUAACUu------------
2 -1,-3
---GUACAGUACUGUGAUAACUG------------
2593 -1,-2
---GUACAGUACUGUGAUAACUGu-----------
23 -1,-2
---GUACAGUACUGUGAUAACUGA-----------
17005 -1,-1

---GUACAGUACUGUGAUAACUGAu----------

70 -1,-1

---GUACAGUACUGUGAUAACUGAA----------

26914 -1,0

---GUACAGUACUGUGAUAACUGAAa---------

6060 -1,0

---GUACAGUACUGUGAUAACUGAAaa--------

256 -1,0

---GUACAGUACUGUGAUAACUGAAau--------

5 -1,0

---GUACAGUACUGUGAUAACUGAAu---------

31 -1,0

---GUACAGUACUGUGAUAACUGAAuu--------

2 -1,0

---GUACAGUACUGUGAUAACUGAAG---------

26 -1,1

---GUACAGUACUGUGAUAACUGAAGu--------

2 -1,1

---GUACAGUACUGUGAUAACUGAAGA--------

4 -1,2

--UACAGUACUGUGAUAACU-------------

5567 0,-3

--UACAGUACUGUGAUAACUa------------

23 0,-3

--UACAGUACUGUGAUAACUaa-----------

17 0,-3

--UACAGUACUGUGAUAACUu------------

2 0,-3

--UACAGUACUGUGAUAACUG------------

3186 0,-2

--UACAGUACUGUGAUAACUGu-----------

28 0,-2

--UACAGUACUGUGAUAACUGA-----------

31682 0,-1

--UACAGUACUGUGAUAACUGAu----------

70 0,-1

--UACAGUACUGUGAUAACUGAuu---------

37 0,-1

--UACAGUACUGUGAUAACUGAA----------

69934 0,0

--UACAGUACUGUGAUAACUGAAa---------

17465 0,0

--UACAGUACUGUGAUAACUGAAaa--------

640 0,0

--UACAGUACUGUGAUAACUGAAau--------

40 0,0

--UACAGUACUGUGAUAACUGAAu---------

877 0,0

--UACAGUACUGUGAUAACUGAAuu--------

37 0,0

--UACAGUACUGUGAUAACUGAAG---------

3692 0,1

--UACAGUACUGUGAUAACUGAAGu--------

490 0,1

--UACAGUACUGUGAUAACUGAAGA--------

1240 0,2

--UACAGUACUGUGAUAACUGAAGAu-------

2 0,2

--UACAGUACUGUGAUAACUGAAGAA-------

78 0,3

--UACAGUACUGUGAUAACUGAAGAAa------

13 0,3

---ACAGUACUGUGAUAACUG------------

19 1,-2

---ACAGUACUGUGAUAACUGA-----------

199 1,-1

---ACAGUACUGUGAUAACUGAA----------

500 1,0

---ACAGUACUGUGAUAACUGAAa---------

131 1,0

---ACAGUACUGUGAUAACUGAAaa--------

9 1,0

---ACAGUACUGUGAUAACUGAAu---------

9 1,0

---ACAGUACUGUGAUAACUGAAG---------

36 1,1

---ACAGUACUGUGAUAACUGAAGu--------

11 1,1

---ACAGUACUGUGAUAACUGAAGA--------

20 1,2

---ACAGUACUGUGAUAACUGAAGAA-------

6 1,3

--CAGUACUGUGAUAACUGA-----------

36 2,-1

--CAGUACUGUGAUAACUGAA----------

92 2,0

--CAGUACUGUGAUAACUGAAa---------

24 2,0

--CAGUACUGUGAUAACUGAAaa--------

2 2,0

--CAGUACUGUGAUAACUGAAau--------

2 2,0

--CAGUACUGUGAUAACUGAAu---------

3 2,0

--CAGUACUGUGAUAACUGAAuu--------

2 2,0

--CAGUACUGUGAUAACUGAAG---------

2 2,1

--CAGUACUGUGAUAACUGAAGu--------

2 2,1

--CAGUACUGUGAUAACUGAAGuu-------

2 2,1

--CAGUACUGUGAUAACUGAAGA--------

10 2,2

--CAGUACUGUGAUAACUGAAGAA-------

17 2,3

--CAGUACUGUGAUAACUGAAGAAa------

15 2,3

>hsa-mir-103-1 3p:48-70 SRX018957

uacugcccucggcuucuuuacagugcugccuuguugcauauggaucaAGCAGCAUUGUACAGGGCUAUGAaggcauug

...((((.((((((.((.(((((((((((.(((.(.(....).).)))))))))))))).)))))))..)).))))...

--AAGCAGCAUUGUACAGGGCUAU----------

5 -1,-2

--AAGCAGCAUUGUACAGGGCUAUG---------

2 -1,-1

---AGCAGCAUUGUACAGGGC-------------

747 0,-5

---AGCAGCAUUGUACAGGGCa------------

3 0,-5

---AGCAGCAUUGUACAGGGCaa-----------

5 0,-5

---AGCAGCAUUGUACAGGGCau-----------

3 0,-5

---AGCAGCAUUGUACAGGGCU------------

441 0,-4

---AGCAGCAUUGUACAGGGCUu-----------

39 0,-4

---AGCAGCAUUGUACAGGGCUuu----------

14 0,-4

---AGCAGCAUUGUACAGGGCUA-----------

2206 0,-3

---AGCAGCAUUGUACAGGGCUAa----------

109 0,-3

---AGCAGCAUUGUACAGGGCUAaa---------

29 0,-3

---AGCAGCAUUGUACAGGGCUAau---------

19 0,-3

---AGCAGCAUUGUACAGGGCUAU----------

9095 0,-2

---AGCAGCAUUGUACAGGGCUAUa---------

303 0,-2

---AGCAGCAUUGUACAGGGCUAUaa--------

240 0,-2

---AGCAGCAUUGUACAGGGCUAUau--------

236 0,-2

---AGCAGCAUUGUACAGGGCUAUu---------

645 0,-2

---AGCAGCAUUGUACAGGGCUAUuu--------

86 0,-2

---AGCAGCAUUGUACAGGGCUAUG---------

7632 0,-1

---AGCAGCAUUGUACAGGGCUAUGu--------

2614 0,-1

---AGCAGCAUUGUACAGGGCUAUGuu-------

212 0,-1

---AGCAGCAUUGUACAGGGCUAUGA--------

16442 0,0

---AGCAGCAUUGUACAGGGCUAUGAu-------

1631 0,0

---AGCAGCAUUGUACAGGGCUAUGAuu------

40 0,0

---AGCAGCAUUGUACAGGGCUAUGAA-------

494 0,1

---AGCAGCAUUGUACAGGGCUAUGAAu------

14 0,1

---AGCAGCAUUGUACAGGGCUAUGAAG------

4 0,2

--GCAGCAUUGUACAGGGCU------------

3 1,-4

--GCAGCAUUGUACAGGGCUA-----------

3 1,-3

--GCAGCAUUGUACAGGGCUAU----------

33 1,-2

--GCAGCAUUGUACAGGGCUAUaa--------

3 1,-2

--GCAGCAUUGUACAGGGCUAUu---------

2 1,-2

--GCAGCAUUGUACAGGGCUAUuu--------

3 1,-2

--GCAGCAUUGUACAGGGCUAUG---------

34 1,-1

--GCAGCAUUGUACAGGGCUAUGu--------

11 1,-1

--GCAGCAUUGUACAGGGCUAUGA--------

89 1,0

--GCAGCAUUGUACAGGGCUAUGAu-------

9 1,0

--GCAGCAUUGUACAGGGCUAUGAA-------

3 1,1

---CAGCAUUGUACAGGGCUAU----------
5 2,-2
---CAGCAUUGUACAGGGCUAUG---------
10 2,-1
---CAGCAUUGUACAGGGCUAUGA--------
33 2,0

>hsa-mir-103-2 mi:11-33;MA:48-70 SRX018957
uugugcuuucAGCUUCUUUACAGUGCUGCCUUGuagcauucaggucaAGCAGCAUUGUACAGGGCUAUGAaagaacca
..((.(((((((((.((.(((((((((((((((.......))))....))))))))))).)))))..)))))).))..
--AAGCAGCAUUGUACAGGGCUAU----------
5 -1,-2
--AAGCAGCAUUGUACAGGGCUAUG---------
2 -1,-1
---AGCAGCAUUGUACAGGGC-------------
747 0,-5
---AGCAGCAUUGUACAGGGCa------------
3 0,-5
---AGCAGCAUUGUACAGGGCaa-----------
5 0,-5
---AGCAGCAUUGUACAGGGCau-----------
3 0,-5
---AGCAGCAUUGUACAGGGCU------------
441 0,-4
---AGCAGCAUUGUACAGGGCUu-----------
39 0,-4
---AGCAGCAUUGUACAGGGCUuu----------
14 0,-4
---AGCAGCAUUGUACAGGGCUA-----------
2206 0,-3
---AGCAGCAUUGUACAGGGCUAa----------
109 0,-3
---AGCAGCAUUGUACAGGGCUAaa---------
29 0,-3
---AGCAGCAUUGUACAGGGCUAau---------
19 0,-3
---AGCAGCAUUGUACAGGGCUAU----------
9095 0,-2

---AGCAGCAUUGUACAGGGCUAUa---------

303 0,-2

---AGCAGCAUUGUACAGGGCUAUaa--------

240 0,-2

---AGCAGCAUUGUACAGGGCUAUau--------

236 0,-2

---AGCAGCAUUGUACAGGGCUAUu---------

645 0,-2

---AGCAGCAUUGUACAGGGCUAUuu--------

86 0,-2

---AGCAGCAUUGUACAGGGCUAUG---------

7632 0,-1

---AGCAGCAUUGUACAGGGCUAUGu--------

2614 0,-1

---AGCAGCAUUGUACAGGGCUAUGuu-------

212 0,-1

---AGCAGCAUUGUACAGGGCUAUGA--------

16442 0,0

---AGCAGCAUUGUACAGGGCUAUGAu-------

1631 0,0

---AGCAGCAUUGUACAGGGCUAUGAuu------

40 0,0

---AGCAGCAUUGUACAGGGCUAUGAA-------

494 0,1

---AGCAGCAUUGUACAGGGCUAUGAAu------

14 0,1

---AGCAGCAUUGUACAGGGCUAUGAAA------

91 0,2

--GCAGCAUUGUACAGGGCU------------

3 1,-4

--GCAGCAUUGUACAGGGCUA-----------

3 1,-3

--GCAGCAUUGUACAGGGCUAU----------

33 1,-2

--GCAGCAUUGUACAGGGCUAUaa--------

3 1,-2

--GCAGCAUUGUACAGGGCUAUu---------

2 1,-2

```
------------------------------------------------GCAGCAUUGUACAGGGCUAUuu--------
    3   1,-2
------------------------------------------------GCAGCAUUGUACAGGGCUAUG---------
    34  1,-1
------------------------------------------------GCAGCAUUGUACAGGGCUAUGu--------
    11  1,-1
------------------------------------------------GCAGCAUUGUACAGGGCUAUGA--------
    89  1,0
------------------------------------------------GCAGCAUUGUACAGGGCUAUGAu-------
    9   1,0
------------------------------------------------GCAGCAUUGUACAGGGCUAUGAA-------
    3   1,1
-------------------------------------------------CAGCAUUGUACAGGGCUAU----------
    5   2,-2
-------------------------------------------------CAGCAUUGUACAGGGCUAUG---------
    10  2,-1
-------------------------------------------------CAGCAUUGUACAGGGCUAUGA--------
    33  2,0

>hsa-mir-106a   MA:13-35;mi:50-71   SRX018957
ccuuggccauguAAAAGUGCUUACAGUGCAGGUAGcuuuuugagaucuaCUGCAAUGUAAGCACUUCUUACauuaccaug
g
((.(((..(((((((((((((((((.(((((.(((.((.....)).)))))))).))))))))))).))))))..))).)
)
-------------AAAGUGCUUACAGUGCAGGU-----------------------------------------------
-   4   1,-2
-------------AAAGUGCUUACAGUGCAGGUAu---------------------------------------------
-   2   1,-1
-------------AAAGUGCUUACAGUGCAGGUAG---------------------------------------------
-   3   1,0

>hsa-mir-106b   MA:12-32;mi:52-73   SRX018957
ccugccggggcUAAAGUGCUGACAGUGCAGAUagugguccucuccgugcuaCCGCACUGUGGGUACUUGCUGCuccagca
gg
(((((.(((((..(((((((.(((((((.(.((((((......))..))))).))))))).)))))))...))))).)))
))
-----------UAAAGUGCUGACAGUGCAG--------------------------------------------------
--  8   0,-2
```

-----------UAAAGUGCUGACAGUGCAGA---
-- 5 0,-1
-----------UAAAGUGCUGACAGUGCAGAU--
-- 12 0,0
-----------UAAAGUGCUGACAGUGCAGAUA---
-- 23 0,1
-----------UAAAGUGCUGACAGUGCAGAUAa--
-- 21 0,1
-----------UAAAGUGCUGACAGUGCAGAUAu--
-- 12 0,1
--ACCGCACUGUGGGUACUUGCUG--------
-- 6 -1,-1
--ACCGCACUGUGGGUACUUGCUGa-------
-- 2 -1,-1
---CCGCACUGUGGGUACUUGC----------
-- 2 0,-3
---CCGCACUGUGGGUACUUGCU---------
-- 4 0,-2
---CCGCACUGUGGGUACUUGCUG--------
-- 57 0,-1
---CCGCACUGUGGGUACUUGCUGa-------
-- 30 0,-1
---CCGCACUGUGGGUACUUGCUGaa------
-- 7 0,-1
---CCGCACUGUGGGUACUUGCUGu-------
-- 8 0,-1
---CCGCACUGUGGGUACUUGCUGC-------
-- 97 0,0
---CCGCACUGUGGGUACUUGCUGCau-----
-- 2 0,0
---CCGCACUGUGGGUACUUGCUGCU------
-- 2 0,1
--CGCACUGUGGGUACUUGCUGC-------
-- 2 1,0

>hsa-mir-107 3p:50-72 SRX018957
cucucugcuuucagcuucuuuacaguguugccuuguggcauggaguucaAGCAGCAUUGUACAGGGCUAUCAaagcacag
a

.(((.((((((.((((.((.(((((((((((.(((.(((.....))))))))))))))))).)))))).)))...)))))).))
)
--AAGCAGCAUUGUACAGGGCUAU----------
-	5	-1,-2
---AGCAGCAUUGUACAGGGC-------------
-	747	0,-5
---AGCAGCAUUGUACAGGGCa------------
-	3	0,-5
---AGCAGCAUUGUACAGGGCaa-----------
-	5	0,-5
---AGCAGCAUUGUACAGGGCau-----------
-	3	0,-5
---AGCAGCAUUGUACAGGGCU------------
-	441	0,-4
---AGCAGCAUUGUACAGGGCUu-----------
-	39	0,-4
---AGCAGCAUUGUACAGGGCUuu----------
-	14	0,-4
---AGCAGCAUUGUACAGGGCUA-----------
-	2206	0,-3
---AGCAGCAUUGUACAGGGCUAa----------
-	109	0,-3
---AGCAGCAUUGUACAGGGCUAaa---------
-	29	0,-3
---AGCAGCAUUGUACAGGGCUAau---------
-	19	0,-3
---AGCAGCAUUGUACAGGGCUAU----------
-	9095	0,-2
---AGCAGCAUUGUACAGGGCUAUa---------
-	303	0,-2
---AGCAGCAUUGUACAGGGCUAUaa--------
-	240	0,-2
---AGCAGCAUUGUACAGGGCUAUau--------
-	236	0,-2
---AGCAGCAUUGUACAGGGCUAUu---------
-	645	0,-2
---AGCAGCAUUGUACAGGGCUAUuu--------
-	86	0,-2

---AGCAGCAUUGUACAGGGCUAUC---------
- 2014 0,-1
---AGCAGCAUUGUACAGGGCUAUCu--------
- 1074 0,-1
---AGCAGCAUUGUACAGGGCUAUCuu-------
- 39 0,-1
---AGCAGCAUUGUACAGGGCUAUCA--------
- 3490 0,0
---AGCAGCAUUGUACAGGGCUAUCAu-------
- 227 0,0
---AGCAGCAUUGUACAGGGCUAUCAuu------
- 4 0,0
---AGCAGCAUUGUACAGGGCUAUCAA-------
- 38 0,1
---AGCAGCAUUGUACAGGGCUAUCAAu------
- 2 0,1
--GCAGCAUUGUACAGGGCU------------
- 3 1,-4
--GCAGCAUUGUACAGGGCUA-----------
- 3 1,-3
--GCAGCAUUGUACAGGGCUAU----------
- 33 1,-2
--GCAGCAUUGUACAGGGCUAUaa--------
- 3 1,-2
--GCAGCAUUGUACAGGGCUAUu---------
- 2 1,-2
--GCAGCAUUGUACAGGGCUAUuu--------
- 3 1,-2
--GCAGCAUUGUACAGGGCUAUCA--------
- 11 1,0
---CAGCAUUGUACAGGGCUAU----------
- 5 2,-2
---CAGCAUUGUACAGGGCUAUCA--------
- 2 2,0

>hsa-mir-10a MA:22-44;mi:63-84 SRX018957
gaucugucugucuucuguauaUACCCUGUAGAUCCGAAUUUGUGuaaggaauuuuguggucaCAAAUUCGUAUCUAGGGG
AAUAuguaguugacauaaacacuccgcucu

....(((.((((..((((((((.((((.(((((.((((((((((................)))))))))).)))))))))
.))))))))..))))...))).........
--------------------AUACCCUGUAGAUCCGAAUUU---------------------------------------
------------------------------ 2 -1,-3
--------------------AUACCCUGUAGAUCCGAAUUUG--------------------------------------
------------------------------ 2 -1,-2
---------------------UACCCUGUAGAUCCGAAU---
------------------------------ 15 0,-5
---------------------UACCCUGUAGAUCCGAAUaa---------------------------------------
------------------------------ 3 0,-5
---------------------UACCCUGUAGAUCCGAAUU--
------------------------------ 23 0,-4
---------------------UACCCUGUAGAUCCGAAUUa---------------------------------------
------------------------------ 2 0,-4
---------------------UACCCUGUAGAUCCGAAUUU---------------------------------------
------------------------------ 18 0,-3
---------------------UACCCUGUAGAUCCGAAUUUG--------------------------------------
------------------------------ 126 0,-2
---------------------UACCCUGUAGAUCCGAAUUUGa-------------------------------------
------------------------------ 7 0,-2
---------------------UACCCUGUAGAUCCGAAUUUGU-------------------------------------
------------------------------ 481 0,-1
---------------------UACCCUGUAGAUCCGAAUUUGUa------------------------------------
------------------------------ 5 0,-1
---------------------UACCCUGUAGAUCCGAAUUUGUu------------------------------------
------------------------------ 2 0,-1
---------------------UACCCUGUAGAUCCGAAUUUGUG------------------------------------
------------------------------ 191 0,0
---------------------UACCCUGUAGAUCCGAAUUUGUGa-----------------------------------
------------------------------ 52 0,0
---------------------UACCCUGUAGAUCCGAAUUUGUGaa----------------------------------
------------------------------ 5 0,0
---------------------UACCCUGUAGAUCCGAAUUUGUGU-----------------------------------
------------------------------ 32 0,1
----------------------ACCCUGUAGAUCCGAAUUUa--------------------------------------
------------------------------ 2 1,-3
----------------------ACCCUGUAGAUCCGAAUUUG--------------------------------------
------------------------------ 12 1,-2

----------------------ACCCUGUAGAUCCGAAUUUGU-------------------------------------
------------------------------ 71 1,-1
----------------------ACCCUGUAGAUCCGAAUUUGUG------------------------------------
------------------------------ 78 1,0
----------------------ACCCUGUAGAUCCGAAUUUGUGa-----------------------------------
------------------------------ 69 1,0
----------------------ACCCUGUAGAUCCGAAUUUGUGaa----------------------------------
------------------------------ 16 1,0
----------------------ACCCUGUAGAUCCGAAUUUGUGU-----------------------------------
------------------------------ 24 1,1
--CAAAUUCGUAUCUAGGGG
------------------------------ 4 0,-4
---AAAUUCGUAUCUAGGGG
AAUAa------------------------- 2 1,0

>hsa-mir-10b MA:27-49;mi:66-87 SRX018957
ccagagguuguaacguugucuauauaUACCCUGUAGAACCGAAUUUGUGugguauccguauagucACAGAUUCGAUUCUA
GGGGAAUauauggucgaugcaaaaacuuca
...((((((....(((((.((((((((.((((.(((((.((((((((((..(((....)))...)))))))))).)))))
)))).)))))))).)))))....)))))).
--------------------------UACCCUGUAGAACCGAAUUU----------------------------------
------------------------------ 2 0,-3
--------------------------UACCCUGUAGAACCGAAUUUG---------------------------------
------------------------------ 6 0,-2
--------------------------UACCCUGUAGAACCGAAUUUGU--------------------------------
------------------------------ 76 0,-1
--------------------------UACCCUGUAGAACCGAAUUUGUa-------------------------------
------------------------------ 2 0,-1
--------------------------UACCCUGUAGAACCGAAUUUGUaa------------------------------
------------------------------ 2 0,-1
--------------------------UACCCUGUAGAACCGAAUUUGUG-------------------------------
------------------------------ 11 0,0
--------------------------UACCCUGUAGAACCGAAUUUGUGa------------------------------
------------------------------ 4 0,0
--------------------------UACCCUGUAGAACCGAAUUUGUGaa-----------------------------
------------------------------ 4 0,0
--------------------------UACCCUGUAGAACCGAAUUUGUGU------------------------------
------------------------------ 3 0,1

---------------------------ACCCUGUAGAACCGAAUUUGU--------------------------------
------------------------------ 2   1,-1
---------------------------ACCCUGUAGAACCGAAUUUGUG-------------------------------
------------------------------ 7   1,0
---------------------------ACCCUGUAGAACCGAAUUUGUGa------------------------------
------------------------------ 3   1,0
---------------------------ACCCUGUAGAACCGAAUUUGUGU------------------------------
------------------------------ 5   1,1
----------------------------CCCUGUAGAACCGAAUUUGUGU------------------------------
------------------------------ 2   2,1

>hsa-mir-1185-1 5p:15-35   SRX018957
uuugguacuugaagAGAGGAUACCCUUUGUAUGUUcacuugauuaauggcgaauauacagggggagacucuuauuugcgu
aucaaa
.(((((((.((((((((..(.((((((((((((((.((........)).)))))))))))))))..))))).)))).))
))))).
--------------AGAGGAUACCCUUUGUAUGUU---
------ 17 0,0
--------------AGAGGAUACCCUUUGUAUGUUa--
------ 3   0,0
--------------AGAGGAUACCCUUUGUAUGUUC--
------ 14 0,1
--------------AGAGGAUACCCUUUGUAUGUUCA---
------ 2   0,2
--AUAUACAGGGGGAGACUCUUA-------
------ 2
--AUAUACAGGGGGAGACUCUUAU------
------ 2

>hsa-mir-1185-2 5p:15-35   SRX018957
uuugguacuuaaagAGAGGAUACCCUUUGUAUGUUcacuugauuaauggcgaauauacagggggagacucucauuugcgu
aucaaa
.(((((((.((((((((..(.((((((((((((((.((........)).)))))))))))))))..))))).)))).))
))))).
--------------AGAGGAUACCCUUUGUAUGUU---
------ 17 0,0
--------------AGAGGAUACCCUUUGUAUGUUa--
------ 3   0,0

--------------AGAGGAUACCCUUUGUAUGUUC--
------ 14 0,1
--------------AGAGGAUACCCUUUGUAUGUUCA---
------ 2 0,2
--AUAUACAGGGGGAGACUCUCA-------
------ 3

>hsa-mir-122 MA:15-36;mi:51-72 SRX018957
ccuuagcagagcugUGGAGUGUGACAAUGGUGUUUGugucuaaacuaucaAACGCCAUUAUCACACUAAAUAgcuacugc
uaggc
.((((((((((((((..(((((((.(((((((((((.((....))...))))))))))).)))))))..)))))).))))
)))).
------------UGUGGAGUGUGACAAUGGUGU---
----- 10 -2,-3
------------UGUGGAGUGUGACAAUGGUGUU--
----- 254 -2,-2
------------UGUGGAGUGUGACAAUGGUGUUa---
----- 10 -2,-2
------------UGUGGAGUGUGACAAUGGUGUUU---
----- 6 -2,-1
-------------GUGGAGUGUGACAAUGGUG--
----- 3 -1,-4
-------------GUGGAGUGUGACAAUGGUGU---
----- 8 -1,-3
-------------GUGGAGUGUGACAAUGGUGUU--
----- 8 -1,-2
-------------GUGGAGUGUGACAAUGGUGUUU---
----- 69 -1,-1
-------------GUGGAGUGUGACAAUGGUGUUUG--
----- 13 -1,0
-------------GUGGAGUGUGACAAUGGUGUUUGa---
----- 6 -1,0
--------------UGGAGUGUGACAAUGGUG--
----- 12191 0,-4
--------------UGGAGUGUGACAAUGGUGa---
----- 1418 0,-4
--------------UGGAGUGUGACAAUGGUGaa--
----- 530 0,-4

--------------UGGAGUGUGACAAUGGUGau---
-----	44	0,-4
--------------UGGAGUGUGACAAUGGUGU--
-----	38116	0,-3
--------------UGGAGUGUGACAAUGGUGUa---
-----	9199	0,-3
--------------UGGAGUGUGACAAUGGUGUaa--
-----	4229	0,-3
--------------UGGAGUGUGACAAUGGUGUau--
-----	622	0,-3
--------------UGGAGUGUGACAAUGGUGUU---
-----	110927	0,-2
--------------UGGAGUGUGACAAUGGUGUUa--
-----	10559	0,-2
--------------UGGAGUGUGACAAUGGUGUUaa---
-----	2535	0,-2
--------------UGGAGUGUGACAAUGGUGUUau---
-----	993	0,-2
--------------UGGAGUGUGACAAUGGUGUUU--
-----	261158	0,-1
--------------UGGAGUGUGACAAUGGUGUUUa---
-----	42238	0,-1
--------------UGGAGUGUGACAAUGGUGUUUaa--
-----	21399	0,-1
--------------UGGAGUGUGACAAUGGUGUUUau--
-----	13928	0,-1
--------------UGGAGUGUGACAAUGGUGUUUu---
-----	4258	0,-1
--------------UGGAGUGUGACAAUGGUGUUUuu--
-----	413	0,-1
--------------UGGAGUGUGACAAUGGUGUUUG---
-----	1858034	0,0
--------------UGGAGUGUGACAAUGGUGUUUGa--
-----	1006546	0,0
--------------UGGAGUGUGACAAUGGUGUUUGaa---
-----	50836	0,0
--------------UGGAGUGUGACAAUGGUGUUUGau---
-----	31964	0,0

--------------UGGAGUGUGACAAUGGUGUUUGU---

-----	361843	0,1

--------------UGGAGUGUGACAAUGGUGUUUGUa--

-----	5309	0,1

--------------UGGAGUGUGACAAUGGUGUUUGUaa---

-----	3420	0,1

--------------UGGAGUGUGACAAUGGUGUUUGUau---

-----	3151	0,1

--------------UGGAGUGUGACAAUGGUGUUUGUu--

-----	2218	0,1

--------------UGGAGUGUGACAAUGGUGUUUGUuu---

-----	665	0,1

--------------UGGAGUGUGACAAUGGUGUUUGUG--

-----	390	0,2

--------------UGGAGUGUGACAAUGGUGUUUGUGa---

-----	95	0,2

--------------UGGAGUGUGACAAUGGUGUUUGUGU---

-----	363	0,3

---------------GGAGUGUGACAAUGGUGU---

-----	183	1,-3

---------------GGAGUGUGACAAUGGUGUa--

-----	64	1,-3

---------------GGAGUGUGACAAUGGUGUaa---

-----	26	1,-3

---------------GGAGUGUGACAAUGGUGUau---

-----	4	1,-3

---------------GGAGUGUGACAAUGGUGUU--

-----	509	1,-2

---------------GGAGUGUGACAAUGGUGUUa---

-----	55	1,-2

---------------GGAGUGUGACAAUGGUGUUaa--

-----	21	1,-2

---------------GGAGUGUGACAAUGGUGUUau--

-----	6	1,-2

---------------GGAGUGUGACAAUGGUGUUU---

-----	694	1,-1

---------------GGAGUGUGACAAUGGUGUUUa--

-----	93	1,-1

---------------GGAGUGUGACAAUGGUGUUUaa--
-----	175	1,-1
---------------GGAGUGUGACAAUGGUGUUUau--
-----	39	1,-1
---------------GGAGUGUGACAAUGGUGUUUu---
-----	11	1,-1
---------------GGAGUGUGACAAUGGUGUUUG---
-----	9884	1,0
---------------GGAGUGUGACAAUGGUGUUUGa--
-----	6590	1,0
---------------GGAGUGUGACAAUGGUGUUUGaa---
-----	380	1,0
---------------GGAGUGUGACAAUGGUGUUUGau---
-----	180	1,0
---------------GGAGUGUGACAAUGGUGUUUGU--
-----	835	1,1
---------------GGAGUGUGACAAUGGUGUUUGUa---
-----	22	1,1
---------------GGAGUGUGACAAUGGUGUUUGUaa--
-----	32	1,1
---------------GGAGUGUGACAAUGGUGUUUGUau--
-----	13	1,1
---------------GGAGUGUGACAAUGGUGUUUGUu---
-----	10	1,1
---------------GGAGUGUGACAAUGGUGUUUGUuu--
-----	9	1,1
---------------GGAGUGUGACAAUGGUGUUUGUG---
-----	2	1,2
----------------GAGUGUGACAAUGGUGUU---
-----	112	2,-2
----------------GAGUGUGACAAUGGUGUUa--
-----	7	2,-2
----------------GAGUGUGACAAUGGUGUUau---
-----	2	2,-2
----------------GAGUGUGACAAUGGUGUUU--
-----	347	2,-1
----------------GAGUGUGACAAUGGUGUUUa---
-----	30	2,-1

----------------GAGUGUGACAAUGGUGUUUaa---
-----	27	2,-1
----------------GAGUGUGACAAUGGUGUUUau---
-----	12	2,-1
----------------GAGUGUGACAAUGGUGUUUu--
-----	17	2,-1
----------------GAGUGUGACAAUGGUGUUUuu---
-----	2	2,-1
----------------GAGUGUGACAAUGGUGUUUG--
-----	1664	2,0
----------------GAGUGUGACAAUGGUGUUUGa---
-----	984	2,0
----------------GAGUGUGACAAUGGUGUUUGaa--
-----	54	2,0
----------------GAGUGUGACAAUGGUGUUUGau--
-----	38	2,0
----------------GAGUGUGACAAUGGUGUUUGU---
-----	442	2,1
----------------GAGUGUGACAAUGGUGUUUGUa--
-----	4	2,1
----------------GAGUGUGACAAUGGUGUUUGUaa---
-----	4	2,1
----------------GAGUGUGACAAUGGUGUUUGUau---
-----	3	2,1
---AAACGCCAUUAUCACACUAA-----------
-----	2	-1,-3
---AAACGCCAUUAUCACACUAAAU---------
-----	9	-1,-1
--AACGCCAUUAUCACACUA------------
-----	3	0,-4
--AACGCCAUUAUCACACUAA-----------
-----	9	0,-3
--AACGCCAUUAUCACACUAAA----------
-----	9	0,-2
--AACGCCAUUAUCACACUAAAa---------
-----	3	0,-2
--AACGCCAUUAUCACACUAAAU---------
-----	72	0,-1

--AACGCCAUUAUCACACUAAAUu--------
-----	9	0,-1
--AACGCCAUUAUCACACUAAAUA--------
-----	31	0,0
--AACGCCAUUAUCACACUAAAUAa-------
-----	6	0,0
--AACGCCAUUAUCACACUAAAUAu-------
-----	13	0,0

>hsa-mir-1228	mi:1-21;MA:52-71	SRX018957
GUGGGCGGGGGCAGGUGUGUGgugggugguggccugcggugagcagggcccUCACACCUGCCUCGCCCCCCag
..(((((((.((((((((....((((.....(((((.....))))))))).))))))))))))))).....
GUGGGCGGGGGCAGGUGUGU---	2
0,-1
GUGGGCGGGGGCAGGUGUGUGG---	5
0,1

>hsa-mir-124-1	mi:14-35;MA:53-72	SRX018957
aggccucucucucCGUGUUCACAGCGGACCUUGAUuuaaauguccauacaauUAAGGCACGCGGUGAAUGCCaagaaugg
ggcug
.((((((..(((..((((((((.(((..((((((((.............))))))))..))).))))))))..)))..))
)))).
--UAAGGCACGCGGUGAAUG----------
-----	5	0,-2
--UAAGGCACGCGGUGAAUGCCA-------
-----	2	0,1
--UAAGGCACGCGGUGAAUGCCAA------
-----	2	0,2

>hsa-mir-124-2	mi:25-46;MA:62-81	SRX018957
aucaagauuagaggcucugcucucCGUGUUCACAGCGGACCUUGAUuuaaugucauacaauUAAGGCACGCGGUGAAUGC
Caagagcggagccuacggcugcacuugaa
.(((((..((((((((((((((..((((((((.(((..((((((((............))))))))..))).))))))))
..))))))))))))....)))..))))).
---UAAGGCACGCGGUGAAUG-
-----------------------------	5	0,-2
---UAAGGCACGCGGUGAAUGC
CA---------------------------	2	0,1

---UAAGGCACGCGGUGAAUGC
CAA-------------------------   2   0,2

>hsa-mir-124-3 mi:15-36;MA:53-72   SRX018957
ugagggccccucugCGUGUUCACAGCGGACCUUGAUuuaaugucuauacaauUAAGGCACGCGGUGAAUGCCaagagagg
cgccucc
.(((((((.(((((.((((((((.(((..((((((((............))))))))..))).))))))))).)))).))
).)))).
--UAAGGCACGCGGUGAAUG----------
------- 5   0,-2
--UAAGGCACGCGGUGAAUGCCA-------
------- 2   0,1
--UAAGGCACGCGGUGAAUGCCAA------
------- 2   0,2

>hsa-mir-1244-1 3p:55-80   SRX018957
aucuuauuccgagcauuccaguaacuuuuuuguguauguacuuagcuguacuauAAGUAGUUGGUUUGUAUGAGAUGGUU
aaaaa
(((((((.....(((..((((..(((......((((.((((......)))))))))))..))))..))))))))))....
.....
--AAGUAGUUGGUUUGUAUGAGAUGGU-
-----   2   0,-1

>hsa-mir-1244-2 3p:55-80   SRX018957
aucuuauuccgagcauuccaguaacuuuuuuguguauguacuuagcuguacuauAAGUAGUUGGUUUGUAUGAGAUGGUU
aaaaa
(((((((.....(((..((((..(((......((((.((((......)))))))))))..))))..))))))))))....
.....
--AAGUAGUUGGUUUGUAUGAGAUGGU-
-----   2   0,-1

>hsa-mir-1244-3 3p:55-80   SRX018957
aucuuauuccgagcauuccaguaacuuuuuuguguauguacuuagcuguacuauAAGUAGUUGGUUUGUAUGAGAUGGUU
aaaaa
(((((((.....(((..((((..(((......((((.((((......)))))))))))..))))..))))))))))....
.....
--AAGUAGUUGGUUUGUAUGAGAUGGU-
-----   2   0,-1

>hsa-mir-1246 5p:11-29 SRX018957
uguauccuugAAUGGAUUUUUGGAGCAGGaguggacaccugacccaaaggaaaucaauccauaggcuagcaau
(((..(((...(((((((((((..((((.(....).))))..))))).......))))))))))...)))..
----------AAUGGAUUUUUGGAGCAGG--- 24
0,0
-----------AUGGAUUUUUGGAGCAGG--- 6
1,0

>hsa-mir-1254 5p:18-41 SRX018957
ggugggaggauugcuugAGCCUGGAAGCUGGAGCCUGCAGUgaacuaucauugugccacuguacuccagccuaggcaaca
aaaugaaauccugucua
...((.((((((..(((.((((((..(((((((..(((((((.((.......))..)))))))))))))))))))..))
).....)))))).))..
---------------UGAGCCUGGAAGCUGGAGCCU--
---------------- 2 -2,-5
-----------------AGCCUGGAAGCUGGAGCCUGCAGU--
---------------- 2 0,0

>hsa-mir-1255a 5p:28-50 SRX018957
auuggaaauccuuugaguugcuucucaAGGAUGAGCAAAGAAAGUAGAUUuuuuagauucuaaagaaacuaucuucuuug
cucauccuugagaagcaacuccuuauccauuaa
..((((........((((((((((((((((((((((((((..(((.((((((((...)))))))).)))..)))))))
)))))))))))))))))))).....)))).....
---------------------------AGGAUGAGCAAAGAAAGU-----------------------------------
---------------------------------- 3 0,-5
---------------------------AGGAUGAGCAAAGAAAGUAG---------------------------------
---------------------------------- 5 0,-3
---------------------------AGGAUGAGCAAAGAAAGUAGA--------------------------------
---------------------------------- 2 0,-2
---------------------------AGGAUGAGCAAAGAAAGUAGAU-------------------------------
---------------------------------- 2 0,-1
---------------------------AGGAUGAGCAAAGAAAGUAGAUU------------------------------
---------------------------------- 6 0,0

>hsa-mir-1255b-1 5p:3-24 SRX018957
uaCGGAUGAGCAAAGAAAGUGGUUucuuaaaauggaaucuacucuuugugaagaugcugugaa
(((((.(..(((((((..((((.((((......)))).)))))))))))..)....)))))..

--CGGAUGAGCAAAGAAAGUGGUU--------------------------------------- 2   0,0
--CGGAUGAGCAAAGAAAGUGGUUa-------------------------------------- 2   0,0
--CGGAUGAGCAAAGAAAGUGGUUU-------------------------------------- 6   0,1

>hsa-mir-1255b-2   5p:6-27 SRX018957
ucuuaCGGAUGAGCAAAGAAAGUGGUUugcgccucaagaaaccacuuucuuugcucauccauaagga
(((((.((((((((((((((((((((((.(.......))))))))))))))))))))))).))))).
-----CGGAUGAGCAAAGAAAGUGGUU-- 2   0,0
-----CGGAUGAGCAAAGAAAGUGGUUa--------------------------------------- 2   0,0
-----CGGAUGAGCAAAGAAAGUGGUUU--------------------------------------- 6   0,1

>hsa-mir-1258   3p:44-64   SRX018957
cuguggcuuccacgaccuaauccuaacuccugcgagucccuggAGUUAGGAUUAGGUCGUGGAAgccacagga
((((((((((((((((((((((((((((((...........))))))))))))))))))))))))))))))..
---AGUUAGGAUUAGGUCGUG------------   4
    0,-3
---AGUUAGGAUUAGGUCGUGGAAa--------   2
    0,0

>hsa-mir-125a   5p:15-38;3p:53-74   SRX018957
ugccagucucuaggUCCCUGAGACCCUUUAACCUGUGAggacauccagggucACAGGUGAGGUUCUUGGGAGCCuggcgu
cuggcc
.(((((.(.(((((..((..(((.((((..((((((((((....))....)))))))))))).)))..))..))))).).
))))).
--------------UCCCUGAGACCCUUUAACC---
------ 117 0,-5
--------------UCCCUGAGACCCUUUAACCa--
------ 2   0,-5
--------------UCCCUGAGACCCUUUAACCaa---
------ 2   0,-5
--------------UCCCUGAGACCCUUUAACCU--
------ 1337   0,-4
--------------UCCCUGAGACCCUUUAACCUG---
------ 624 0,-3
--------------UCCCUGAGACCCUUUAACCUGa--
------ 15 0,-3
--------------UCCCUGAGACCCUUUAACCUGaa---
------ 2   0,-3

--------------UCCCUGAGACCCUUUAACCUGU--
------ 424 0,-2
--------------UCCCUGAGACCCUUUAACCUGUG---
------ 138 0,-1
--------------UCCCUGAGACCCUUUAACCUGUGu--
------ 2 0,-1
--------------UCCCUGAGACCCUUUAACCUGUGA--
------ 41 0,0
---------------CCCUGAGACCCUUUAACCU--
------ 2 1,-4
---------------CCCUGAGACCCUUUAACCUGUG---
------ 2 1,-1
----------------CCUGAGACCCUUUAACCU--
------ 8 2,-4
----------------CCUGAGACCCUUUAACCUG---
------ 7 2,-3
----------------CCUGAGACCCUUUAACCUGU--
------ 22 2,-2
----------------CCUGAGACCCUUUAACCUGUG---
------ 4 2,-1
--ACAGGUGAGGUUCUUGGGAG--------
------ 3 0,-2

>hsa-mir-125b-1 MA:15-36;mi:55-76 SRX018957
ugcgcuccucucagUCCCUGAGACCCUAACUUGUGAuguuuaccguuuaaauccACGGGUUAGGCUCUUGGGAGCUgcga
gucgugcu
.((((..(((.(((..((..(((.(((((((((((..(((((.....))))).))))))))))).)))..))..))).))
)..)))).
--------------UCCCUGAGACCCUAACUU--
-------- 20 0,-4
--------------UCCCUGAGACCCUAACUUa---
-------- 9 0,-4
--------------UCCCUGAGACCCUAACUUaa--
-------- 2 0,-4
--------------UCCCUGAGACCCUAACUUu---
-------- 2 0,-4
--------------UCCCUGAGACCCUAACUUG---
-------- 155 0,-3

--------------UCCCUGAGACCCUAACUUGa--
--------	11	0,-3

--------------UCCCUGAGACCCUAACUUGaa---
--------	2	0,-3

--------------UCCCUGAGACCCUAACUUGU--
--------	1645	0,-2

--------------UCCCUGAGACCCUAACUUGUa---
--------	6	0,-2

--------------UCCCUGAGACCCUAACUUGUaa--
--------	7	0,-2

--------------UCCCUGAGACCCUAACUUGUu---
--------	2	0,-2

--------------UCCCUGAGACCCUAACUUGUG---
--------	1566	0,-1

--------------UCCCUGAGACCCUAACUUGUGu--
--------	171	0,-1

--------------UCCCUGAGACCCUAACUUGUGuu---
--------	3	0,-1

--------------UCCCUGAGACCCUAACUUGUGA--
--------	5083	0,0

--------------UCCCUGAGACCCUAACUUGUGAa---
--------	2048	0,0

--------------UCCCUGAGACCCUAACUUGUGAaa--
--------	248	0,0

--------------UCCCUGAGACCCUAACUUGUGAau--
--------	14	0,0

--------------UCCCUGAGACCCUAACUUGUGAU---
--------	139	0,1

--------------UCCCUGAGACCCUAACUUGUGAUa--
--------	8	0,1

--------------UCCCUGAGACCCUAACUUGUGAUu--
--------	4	0,1

---------------CCCUGAGACCCUAACUUGUGAau--
--------	2	1,0

---------------CCCUGAGACCCUAACUUGUGAU---
--------	14	1,1

----------------CCUGAGACCCUAACUUGU--
--------	13	2,-2

----------------CCUGAGACCCUAACUUGUG---
--------	21	2,-1
----------------CCUGAGACCCUAACUUGUGu--
--------	2	2,-1
----------------CCUGAGACCCUAACUUGUGA--
--------	58	2,0
----------------CCUGAGACCCUAACUUGUGAa---
--------	21	2,0
----------------CCUGAGACCCUAACUUGUGAaa--
--------	2	2,0
--ACGGGUUAGGCUCUUGGGAG------
--------	9	0,-2
--ACGGGUUAGGCUCUUGGGAGa-----
--------	3	0,-2
--ACGGGUUAGGCUCUUGGGAGu-----
--------	2	0,-2
--ACGGGUUAGGCUCUUGGGAGC-----
--------	3	0,-1
--ACGGGUUAGGCUCUUGGGAGCUa---
--------	2	0,0

>hsa-mir-125b-2 MA:17-38;mi:54-75	SRX018957
accagacuuuuccuagUCCCUGAGACCCUAACUUGUGAgguauuuuaguaacaUCACAAGUCAGGCUCUUGGGACcuagg
cggagggga
.((...(((..(((((..((..(((.(((.((((((((..(((....)))...)))))))).))).)))..))..)))))
..))).)).
----------------UCCCUGAGACCCUAACUU--
---------	20	0,-4
----------------UCCCUGAGACCCUAACUUa---
---------	9	0,-4
----------------UCCCUGAGACCCUAACUUaa--
---------	2	0,-4
----------------UCCCUGAGACCCUAACUUu---
---------	2	0,-4
----------------UCCCUGAGACCCUAACUUG---
---------	155	0,-3
----------------UCCCUGAGACCCUAACUUGa--
---------	11	0,-3

----------------UCCCUGAGACCCUAACUUGaa---

---------   2   0,-3

----------------UCCCUGAGACCCUAACUUGU--

---------   1645   0,-2

----------------UCCCUGAGACCCUAACUUGUa---

---------   6   0,-2

----------------UCCCUGAGACCCUAACUUGUaa--

---------   7   0,-2

----------------UCCCUGAGACCCUAACUUGUu---

---------   2   0,-2

----------------UCCCUGAGACCCUAACUUGUG---

---------   1566   0,-1

----------------UCCCUGAGACCCUAACUUGUGu--

---------   171 0,-1

----------------UCCCUGAGACCCUAACUUGUGuu---

---------   3   0,-1

----------------UCCCUGAGACCCUAACUUGUGA--

---------   5083   0,0

----------------UCCCUGAGACCCUAACUUGUGAa---

---------   2048   0,0

----------------UCCCUGAGACCCUAACUUGUGAaa--

---------   248 0,0

----------------UCCCUGAGACCCUAACUUGUGAau--

---------   14   0,0

----------------UCCCUGAGACCCUAACUUGUGAG---

---------   11   0,1

----------------UCCCUGAGACCCUAACUUGUGAGa--

---------   9   0,1

-----------------CCCUGAGACCCUAACUUGUGAau--

---------   2   1,0

------------------CCUGAGACCCUAACUUGU--

---------   13   2,-2

------------------CCUGAGACCCUAACUUGUG---

---------   21   2,-1

------------------CCUGAGACCCUAACUUGUGu--

---------   2   2,-1

------------------CCUGAGACCCUAACUUGUGA--

---------   58   2,0

------------------CCUGAGACCCUAACUUGUGAa---
---------   21   2,0
------------------CCUGAGACCCUAACUUGUGAaa--
---------   2   2,0
--AUCACAAGUCAGGCUCUUGGG-------
---------   9   -1,-2
--AUCACAAGUCAGGCUCUUGGGA------
---------   5   -1,-1
---UCACAAGUCAGGCUCUUGG--------
---------   4   0,-3
---UCACAAGUCAGGCUCUUGGGA------
---------   4   0,-1
---UCACAAGUCAGGCUCUUGGGAC-----
---------   3   0,0
---ACAAGUCAGGCUCUUGGG-------
---------   80   2,-2
---ACAAGUCAGGCUCUUGGGA------
---------   32   2,-1
---ACAAGUCAGGCUCUUGGGAa-----
---------   2   2,-1
---ACAAGUCAGGCUCUUGGGAC-----
---------   32   2,0
---ACAAGUCAGGCUCUUGGGACa----
---------   2   2,0
---ACAAGUCAGGCUCUUGGGACC----
---------   5   2,1
---ACAAGUCAGGCUCUUGGGACCU---
---------   8   2,2

>hsa-mir-126   mi:15-35;MA:52-73   SRX018957
cgcuggcgacgggaCAUUAUUACUUUUGGUACGCGcugugacacuucaaacUCGUACCGUGAGUAAUAAUGCGccgucca
cggca
.((((..((((((.(((((((((((.(((((((.(...(((....)))..).))))))).))))))))))).))))))..
)))).
--------------CAUUAUUACUUUUGGUAC--
-----   10   0,-3
--------------CAUUAUUACUUUUGGUACG---
-----   19   0,-2

--------------CAUUAUUACUUUUGGUACGC---
-----   39   0,-1
--------------CAUUAUUACUUUUGGUACGCa--
-----   3   0,-1
--------------CAUUAUUACUUUUGGUACGCaa---
-----   2   0,-1
--------------CAUUAUUACUUUUGGUACGCG--
-----   366 0,0
--------------CAUUAUUACUUUUGGUACGCGa---
-----   129 0,0
--------------CAUUAUUACUUUUGGUACGCGaa--
-----   3   0,0
--------------CAUUAUUACUUUUGGUACGCGu---
-----   54   0,0
--------------CAUUAUUACUUUUGGUACGCGC---
-----   7   0,1
--------------CAUUAUUACUUUUGGUACGCGCU--
-----   5   0,2
---------------AUUAUUACUUUUGGUACGC---
-----   4   1,-1
---------------AUUAUUACUUUUGGUACGCG--
-----   39   1,0
---------------AUUAUUACUUUUGGUACGCGa---
-----   3   1,0
---------------AUUAUUACUUUUGGUACGCGCU--
-----   8   1,2
---CUCGUACCGUGAGUAAUAAUaa--------
-----   2   -1,-3
---CUCGUACCGUGAGUAAUAAUG---------
-----   5   -1,-2
---CUCGUACCGUGAGUAAUAAUGa--------
-----   5   -1,-2
---CUCGUACCGUGAGUAAUAAUGC--------
-----   12   -1,-1
---CUCGUACCGUGAGUAAUAAUGCG-------
-----   25   -1,0
--UCGUACCGUGAGUAAUAAU----------
-----   4   0,-3

---UCGUACCGUGAGUAAUAAUG---------
-----   2   0,-2
---UCGUACCGUGAGUAAUAAUGa--------
-----   9   0,-2
---UCGUACCGUGAGUAAUAAUGaa-------
-----   5   0,-2
---UCGUACCGUGAGUAAUAAUGu--------
-----   2   0,-2
---UCGUACCGUGAGUAAUAAUGC--------
-----   28   0,-1
---UCGUACCGUGAGUAAUAAUGCa-------
-----   3   0,-1
---UCGUACCGUGAGUAAUAAUGCG-------
-----   51   0,0
---UCGUACCGUGAGUAAUAAUGCGa------
-----   41   0,0
--CGUACCGUGAGUAAUAAUG---------
-----   2   1,-2
--CGUACCGUGAGUAAUAAUGC--------
-----   8   1,-1
--CGUACCGUGAGUAAUAAUGCG-------
-----   38   1,0
--CGUACCGUGAGUAAUAAUGCGa------
-----   13   1,0
--CGUACCGUGAGUAAUAAUGCGu------
-----   8   1,0
---GUACCGUGAGUAAUAAUGC--------
-----   7   2,-1
---GUACCGUGAGUAAUAAUGCG-------
-----   9   2,0
---GUACCGUGAGUAAUAAUGCGa------
-----   5   2,0

>hsa-mir-1268   5p:5-22 SRX018957
uagcCGGGCGUGGUGGUGGGGGccuguggucccagcuacuuuggaggcugag
((((...((.((((((.((((((...)))))).)))))).))..)))))..
----CGGGCGUGGUGGUGGGGG------------------------------   14   0,0
----CGGGCGUGGUGGUGGGGGu-----------------------------   2   0,0

>hsa-mir-127   5p:23-44;3p:57-78   SRX018957
ugugaucacugucuccagccugCUGAAGCUCAGAGGGCUCUGAUucagaaagaucaUCGGAUCCGUCUGAGCUUGGCUgg
ucggaagucucaucauc
((.(((..(((...((((((.....(((((((((.((.((((((((.....))..)))))).)).)))))))))))))))
.)))..))).)).....
--------------------UGCUGAAGCUCAGAGGGCUCU---------------------------------------
-----------------   3   -2,-3
----------------------CUGAAGCUCAGAGGGCUC--
-----------------   8   0,-4
----------------------CUGAAGCUCAGAGGGCUCU---------------------------------------
-----------------   15   0,-3
----------------------CUGAAGCUCAGAGGGCUCUG--------------------------------------
-----------------   2   0,-2
----------------------CUGAAGCUCAGAGGGCUCUGA-------------------------------------
-----------------   10   0,-1
----------------------CUGAAGCUCAGAGGGCUCUGAU------------------------------------
-----------------   3   0,0
----------------------CUGAAGCUCAGAGGGCUCUGAUU-----------------------------------
-----------------   2   0,1
--UCGGAUCCGUCUGAGCUUGG----
-----------------   9   0,-2
--UCGGAUCCGUCUGAGCUUGGa---
-----------------   4   0,-2
--UCGGAUCCGUCUGAGCUUGGaa--
-----------------   7   0,-2
--UCGGAUCCGUCUGAGCUUGGC---
-----------------   59   0,-1
--UCGGAUCCGUCUGAGCUUGGCa--
-----------------   41   0,-1
--UCGGAUCCGUCUGAGCUUGGCaa-
-----------------   39   0,-1
--UCGGAUCCGUCUGAGCUUGGCau-
-----------------   97   0,-1
--UCGGAUCCGUCUGAGCUUGGCU--
-----------------   263 0,0
--UCGGAUCCGUCUGAGCUUGGCUa-
-----------------   114 0,0

--UCGGAUCCGUCUGAGCUUGGCUaa
----------------- 16 0,0
--UCGGAUCCGUCUGAGCUUGGCUau
----------------- 153 0,0
--UCGGAUCCGUCUGAGCUUGGCUu-
----------------- 99 0,0
--UCGGAUCCGUCUGAGCUUGGCUuu
----------------- 25 0,0
--UCGGAUCCGUCUGAGCUUGGCUG-
----------------- 6 0,1
--UCGGAUCCGUCUGAGCUUGGCUGu
----------------- 3 0,1
---CGGAUCCGUCUGAGCUUGGCUa-
----------------- 2 1,0
--GGAUCCGUCUGAGCUUGGCa--
----------------- 2 2,-1
--GGAUCCGUCUGAGCUUGGCU--
----------------- 5 2,0
--GGAUCCGUCUGAGCUUGGCUa-
----------------- 2 2,0
--GGAUCCGUCUGAGCUUGGCUau
----------------- 2 2,0
--GGAUCCGUCUGAGCUUGGCUu-
----------------- 3 2,0

>hsa-mir-1270-1 5p:13-35 SRX018957
cacagaguuauaCUGGAGAUAUGGAAGAGCUGUGUuggguauaaguaacaggcuuuucuuuaucuucuauguggcucuuu
gca
((.(((((((((..(((((((.(((((((((.(((((........)))))))))))))).)))))))..))))))))).)
)..
------------CUGGAGAUAUGGAAGAGCUGUGU---
--- 2 0,0

>hsa-mir-1270-2 5p:13-35 SRX018957
cacagaguuauaCUGGAGAUAUGGAAGAGCUGUGUuggguauaaguaacaggcuuuucuuuaucuucuauguggcucuuu
gca
((.(((((((((..(((((((.(((((((((.(((((........)))))))))))))).)))))))..))))))))).)
)..

------------CUGGAGAUAUGGAAGAGCUGUGU---
--- 2 0,0

>hsa-mir-1271 5p:15-36 SRX018957
cacccagaucagugCUUGGCACCUAGCAAGCACUCAguaaauauuuguugagugccugcuaugugccaggcauugugcug
agggcu
..(((((..(((((((((((((.(((((.(((((((((((....))))))))))).))))).)))))))))))))..)))
.))...
--------------CUUGGCACCUAGCAAGCA--
------ 4 0,-4
--------------CUUGGCACCUAGCAAGCAC---
------ 22 0,-3
--------------CUUGGCACCUAGCAAGCACU--
------ 40 0,-2
--------------CUUGGCACCUAGCAAGCACUCA--
------ 3 0,0
--------------CUUGGCACCUAGCAAGCACUCAa---
------ 3 0,0
--AGUGCCUGCUAUGUGCCA------------
------ 3

>hsa-mir-1273 3p:76-100 SRX018957
ugaggcaggagaauugcuugaacccgggugguggagguugcagugagccaagauugcgccacugcacuccagccuGGGCG
ACAAAGCAAGACUCUUUCUUgga
.((((.(((((..((((((...(((((((..(((((..(((((((.((((....)).)))))))))))))))))))))..
...))))))..)))))))))...
--------GAGAAUUGCUUGAACCCGG---
----------------------- 2

>hsa-mir-1273c 5p:10-31 SRX018957
ugcagccugGGCGACAAAACGAGACCCUGUCuuuuuuuuuuucugagacagagucucguucuguugcccaagcugga
..((((.(((((((((.((((((((.(((((((...........))))))).)))))))).))))))))).))))..
---------GGCGACAAAACGAGACCCU---
5 0,-3
---------GGCGACAAAACGAGACCCUGU---
3 0,-1
---------GGCGACAAAACGAGACCCUGUCa---
4 0,0

---------GGCGACAAAACGAGACCCUGUCau--
2 0,0

>hsa-mir-1274b 5p:15-31 SRX018957
cuucuucacucacgUCCCUGUUCGGGCGCCAcuuguggcugucgguucgggacugaaugaagaagga
(((((((.(((.(((((.(..(.(((((((....)))).))).)..))))))))).))))))))..
-------------GUCCCUGUUCGGGCGCCA------------------------------------ 4 -1,0

>hsa-mir-1277 3p:47-68 SRX018957
accucccaaauauauauauauauguacguauguguauauaaauguaUACGUAGAUAUAUAUGUAUUUUuggugggguuu
(((..(((((.((((((((((((.((((((((..(......)..)))))))).))))))))))))).))))).)))..
--UACGUAGAUAUAUAUGUAUUUU----------
7 0,0

>hsa-mir-1278 3p:50-71 SRX018957
auuugcucauagaugauaugcauaguacucccagaacucauuaaguuggUAGUACUGUGCAUAUCAUCUAUgagcgaaua
g
(((((((((((((((((((((((((((((.(((..(((.....)))))).))))))))))))))))))))))))))))).
.
---UAGUACUGUGCAUAUCAUCU-----------
- 6 0,-2
---UAGUACUGUGCAUAUCAUCUA----------
- 6 0,-1
---UAGUACUGUGCAUAUCAUCUAU---------
- 28 0,0
---UAGUACUGUGCAUAUCAUCUAUa--------
- 5 0,0

>hsa-mir-128-1 3p:50-70 SRX018957
ugagcuguuggauucggggccguagcacugucugagagguuuacauuucUCACAGUGAACCGGUCUCUUUuucagcugcu
uc
.((((.((((((...(((((((...((((((..(((((((....))))))))))))...)))))))...)))))).))))
).
--------------CGGGGCCGUAGCACUGUCUGA---
-- 3
---UCACAGUGAACCGGUCUCU------------
-- 5 0,-2
---UCACAGUGAACCGGUCUCUU-----------

-- 15 0,-1
---UCACAGUGAACCGGUCUCUUa----------
-- 7 0,-1
---UCACAGUGAACCGGUCUCUUaa---------
-- 4 0,-1
---UCACAGUGAACCGGUCUCUUau---------
-- 2 0,-1
---UCACAGUGAACCGGUCUCUUU----------
-- 72 0,0
---UCACAGUGAACCGGUCUCUUUa---------
-- 47 0,0
---UCACAGUGAACCGGUCUCUUUaa--------
-- 11 0,0
---UCACAGUGAACCGGUCUCUUUau--------
-- 9 0,0
---UCACAGUGAACCGGUCUCUUUU---------
-- 22 0,1
---UCACAGUGAACCGGUCUCUUUUa--------
-- 3 0,1
---UCACAGUGAACCGGUCUCUUUUU--------
-- 2 0,2
--CACAGUGAACCGGUCUCUUUa---------
-- 3 1,0

>hsa-mir-128-2 3p:52-72 SRX018957
ugugcagugggaaggggggccgauacacuguacgagagugaguagcaggucUCACAGUGAACCGGUCUCUUUcccuacug
uguc
.(..(((((((.((((((((((...((((((..((((.(........).))))))))))...)))))))))).)))))))
..).
--------------GGGGGCCGAUACACUGUACGA---
---- 2
--------------GGGGGCCGAUACACUGUACGAGA---
---- 2
---UCACAGUGAACCGGUCUCU----------
---- 5 0,-2
---UCACAGUGAACCGGUCUCUU---------
---- 15 0,-1
---UCACAGUGAACCGGUCUCUUa--------

----   7   0,-1
---UCACAGUGAACCGGUCUCUUaa-------
----   4   0,-1
---UCACAGUGAACCGGUCUCUUau-------
----   2   0,-1
---UCACAGUGAACCGGUCUCUUU--------
----   72   0,0
---UCACAGUGAACCGGUCUCUUUa-------
----   47   0,0
---UCACAGUGAACCGGUCUCUUUaa------
----   11   0,0
---UCACAGUGAACCGGUCUCUUUau------
----   9   0,0
---UCACAGUGAACCGGUCUCUUUC-------
----   3   0,1
---UCACAGUGAACCGGUCUCUUUCa------
----   2   0,1
--CACAGUGAACCGGUCUCUUUa-------
----   3   1,0

>hsa-mir-1285-1 3p:51-72   SRX018957
uguagagauaggaucucacuuuguugcccaggcuggucucaaacuccuggUCUGGGCAACAAAGUGAGACCUuaucucua
caag
((((((((((((.((((((((((((((((((((..(..........)..)))))))))))))))))))).))))))))))
))..
--UCUGGGCAACAAAGUGAG------------
----   2   0,-4
--UCUGGGCAACAAAGUGAGA-----------
----   3   0,-3
--UCUGGGCAACAAAGUGAGAC----------
----   9   0,-2
--UCUGGGCAACAAAGUGAGACa---------
----   12   0,-2
--UCUGGGCAACAAAGUGAGACaa--------
----   4   0,-2
--UCUGGGCAACAAAGUGAGACC---------
----   5   0,-1
--UCUGGGCAACAAAGUGAGACCa--------

----   3   0,-1
--UCUGGGCAACAAAGUGAGACCaa-------
----   2   0,-1
--UCUGGGCAACAAAGUGAGACCU--------
----   22   0,0
--UCUGGGCAACAAAGUGAGACCUa-------
----   36   0,0
--UCUGGGCAACAAAGUGAGACCUaa------
----   8   0,0
--UCUGGGCAACAAAGUGAGACCUau------
----   24   0,0
--UCUGGGCAACAAAGUGAGACCUU-------
----   22   0,1
--UCUGGGCAACAAAGUGAGACCUUu------
----   15   0,1
--UCUGGGCAACAAAGUGAGACCUUuu-----
----   2   0,1
--UCUGGGCAACAAAGUGAGACCUUA------
----   4   0,2

>hsa-mir-1285-2 3p:52-73   SRX018957
uuugggaggccgaggcuggugcaucacuugagcccagcaauuugagaccaaUCUGGGCAACAAAGUGAGACCUccgucuc
uacaaaga
((((((((((.((((........((((((..((((((....(((....))).))))))....))))))..)))).)))))
).))))..
---UCUGGGCAACAAAGUGAG-----------
--------   2   0,-4
---UCUGGGCAACAAAGUGAGA----------
--------   3   0,-3
---UCUGGGCAACAAAGUGAGAC---------
--------   9   0,-2
---UCUGGGCAACAAAGUGAGACa--------
--------   12   0,-2
---UCUGGGCAACAAAGUGAGACaa-------
--------   4   0,-2
---UCUGGGCAACAAAGUGAGACC--------
--------   5   0,-1
---UCUGGGCAACAAAGUGAGACCa-------

-------- 3 0,-1
---UCUGGGCAACAAAGUGAGACCaa------
-------- 2 0,-1
---UCUGGGCAACAAAGUGAGACCU-------
-------- 22 0,0
---UCUGGGCAACAAAGUGAGACCUa------
-------- 36 0,0
---UCUGGGCAACAAAGUGAGACCUaa-----
-------- 8 0,0
---UCUGGGCAACAAAGUGAGACCUau-----
-------- 24 0,0

>hsa-mir-1286 3p:47-67 SRX018957
uguccucuggggacucagcuugcucuggcugcuggauugaauuagcUGCAGGACCAAGAUGAGCCCUugguggagaca
..(((.(..(((.((((.((((.(((.((.(((((......))))).)).))).)))).)))).)))..).)))....
--UGCAGGACCAAGAUGAGCCCUau---------
2 0,0

>hsa-mir-1287 5p:16-37 SRX018957
guugugcuguccaggUGCUGGAUCAGUGGUUCGAGUCugagccuuuaaaagccacucuagccacagaugcagugauugga
gccaugacaa
((..((...(((((..((((.(((.((((((.((((..(.((........))))))).)))))).))).))))..)))))
..))..))..
---------------UGCUGGAUCAGUGGUUCGAGU--
---------- 4 0,-1
---------------UGCUGGAUCAGUGGUUCGAGUa---
---------- 3 0,-1
---------------UGCUGGAUCAGUGGUUCGAGUC---
---------- 9 0,0
---------------UGCUGGAUCAGUGGUUCGAGUCa--
---------- 6 0,0
---------------UGCUGGAUCAGUGGUUCGAGUCU--
---------- 4 0,1

>hsa-mir-1291 5p:14-37 SRX018957
gguagaauuccagUGGCCCUGACUGAAGACCAGCAGUuguacuguggcuguugguuucaagcagaggccuaaaggacugu
cuuccug
((.(((..(((...(((((((.((..(((((((((((..(...)..)))))))))))..))))).))))....)))...)

)).))..
------------GUGGCCCUGACUGAAGACCAG---
------- 4   -1,-4
------------GUGGCCCUGACUGAAGACCAGC--
------- 3   -1,-3
------------GUGGCCCUGACUGAAGACCAGCA---
------- 85 -1,-2
-------------UGGCCCUGACUGAAGACCAGCA---
------- 15 0,-2

>hsa-mir-1299   3p:62-83   SRX018957
ccucauggcaguguucuggaauccuacgugagggacaaucauucagacccacguagcagugUUCUGGAAUUCUGUGUGAG
GGA
(((((((.(((.((((..((((.(((((((.((..............)))))))))....))))..)))).)))))))))
)..
---UUCUGGAAUUCUGUGUGAG
GGA 2   0,0

>hsa-mir-1301   3p:48-71   SRX018957
ggauuguggggggucgcucuaggcaccgcagcacugugcuggggaugUUGCAGCUGCCUGGGAGUGACUUCacacagucc
uc
(((((((.((((((((((((((((((.((((((((.......)).))))))).).))))).))))))))))).))))))))
..
---UUGCAGCUGCCUGGGAGUGAC------------
-- 5   0,-3
---UUGCAGCUGCCUGGGAGUGACUU----------
-- 4   0,-1
--UGCAGCUGCCUGGGAGUGACU-----------
-- 2   1,-2
--UGCAGCUGCCUGGGAGUGACUU----------
-- 2   1,-1
--UGCAGCUGCCUGGGAGUGACUUu---------
-- 2   1,-1
--UGCAGCUGCCUGGGAGUGACUUC---------
-- 2   1,0
--UGCAGCUGCCUGGGAGUGACUUCA--------
-- 5   1,1

>hsa-mir-1303   3p:52-73   SRX018957
ggcugggcaacauagcgagaccucaacucuacaauuuuuuuuuuuuuaaauUUUAGAGACGGGGUCUUGCUCUguugcca
ggcuuu
((((.(((((((.(((((((((((..(((((.(((((..........))))).)))))..))))))))))).))))))).
))))..
---UGGGCAACAUAGCGAGACC--
------ 12
--UUUUAGAGACGGGGUCUUGC----------
------ 2 -1,-3
--UUUUAGAGACGGGGUCUUGCU---------
------ 5 -1,-2
---UUUAGAGACGGGGUCUUGCU---------
------ 14 0,-2
--UUAGAGACGGGGUCUUGCU---------
------ 3 1,-2

>hsa-mir-1306   3p:55-72   SRX018957
gugagcagucuccaccaccucccccugcaaacguccaguggugcagagguaauggACGUUGGCUCUGGUGGUGauggacag
uccga
.((..(.((((.(((((((......((.((((((((....(((....))).)))))))).))...)))))))..)))).)
..)).
--ACGUUGGCUCUGGUGGUGAUGu----
----- 2 0,3

>hsa-mir-1307   3p:80-101   SRX018957
caucaagacccagcugagucacugucacugccuaccaaucucgaccggaccucgaccggcucgucuguguugccaaucgA
CUCGGCGUGGCGUCGGUCGUGguagauaggcggucaugcauacgaauuuucagcucuuguucuggugac
(((((..((..(((((((((..(((.((((((((.(.(((.(((((((.((.((.(((..(((..((......))..)))
..))))).))..))))))).))).).))))))))...)))...))...)))))))...))..)))))..
---------------------------------------CUCGACCGGACCUCGACCGGCU-------------------
--- 4
--UCGACCGGACCUCGACCG----------------------
--- 4
--UCGACCGGACCUCGACCGG---------------------
--- 4
--UCGACCGGACCUCGACCGGC--------------------
--- 76
--UCGACCGGACCUCGACCGGCaa------------------

-- 2
------------------------------------UCGACCGGACCUCGACCGGCU------------------
-- 469
------------------------------------UCGACCGGACCUCGACCGGCUa-----------------
-- 22
------------------------------------UCGACCGGACCUCGACCGGCUaa----------------
-- 9
------------------------------------UCGACCGGACCUCGACCGGCUau----------------
-- 16
------------------------------------UCGACCGGACCUCGACCGGCUu-----------------
-- 67
------------------------------------UCGACCGGACCUCGACCGGCUuu----------------
-- 41
------------------------------------UCGACCGGACCUCGACCGGCUC-----------------
-- 455
------------------------------------UCGACCGGACCUCGACCGGCUCa----------------
-- 42
------------------------------------UCGACCGGACCUCGACCGGCUCaa---------------
-- 3
------------------------------------UCGACCGGACCUCGACCGGCUCu----------------
-- 16
------------------------------------UCGACCGGACCUCGACCGGCUCuu---------------
-- 3
------------------------------------UCGACCGGACCUCGACCGGCUCG----------------
-- 30
------------------------------------UCGACCGGACCUCGACCGGCUCGU---------------
-- 19
------------------------------------UCGACCGGACCUCGACCGGCUCGUu--------------
-- 2
-------------------------------------CGACCGGACCUCGACCGGCU------------------
-- 4
-------------------------------------CGACCGGACCUCGACCGGCUC-----------------
-- 3
--------------------------------------GACCGGACCUCGACCGGCU------------------
-- 9
--------------------------------------GACCGGACCUCGACCGGCUa-----------------
-- 2
--------------------------------------GACCGGACCUCGACCGGCUu-----------------

-- 5
---GACCGGACCUCGACCGGCUuu-----------------
-- 4
---GACCGGACCUCGACCGGCUC------------------
-- 12
---GACCGGACCUCGACCGGCUCu-----------------
-- 2
--A
CUCGGCGUGGCGUCGGUC-- 3 0,-3
--A
CUCGGCGUGGCGUCGGUCG--- 9 0,-2
--A
CUCGGCGUGGCGUCGGUCGU-- 16 0,-1
--A
CUCGGCGUGGCGUCGGUCGUu--- 3 0,-1
--A
CUCGGCGUGGCGUCGGUCGUG--- 246 0,0
--A
CUCGGCGUGGCGUCGGUCGUGa-- 12 0,0
--A
CUCGGCGUGGCGUCGGUCGUGu-- 17 0,0
--A
CUCGGCGUGGCGUCGGUCGUGG-- 5 0,1
--A
CUCGGCGUGGCGUCGGUCGUGGU--- 2 0,2

CUCGGCGUGGCGUCGGUCGU-- 2 1,-1

CUCGGCGUGGCGUCGGUCGUu--- 2 1,-1

CUCGGCGUGGCGUCGGUCGUG--- 78 1,0

CUCGGCGUGGCGUCGGUCGUGa-- 2 1,0

CUCGGCGUGGCGUCGGUCGUGu-- 2 1,0

CUCGGCGUGGCGUCGGUCGUGG-- 3 1,1

>hsa-mir-130a	mi:21-42;MA:55-76	SRX018957
ugcugcuggccagagcucuuUUCACAUUGUGCUACUGUCUGCaccugucacuagCAGUGCAAUGUUAAAAGGGCAUuggc
cguguagug
.((((((((((((.((((((((.((((((..((.(((...((....))...))).))..)))))).)))))))).)))))
)).))))).
--CAGUGCAAUGUUAAAAGG--------
---------	311	0,-4
--CAGUGCAAUGUUAAAAGGG-------
---------	172	0,-3
--CAGUGCAAUGUUAAAAGGGC------
---------	381	0,-2
--CAGUGCAAUGUUAAAAGGGCA-----
---------	68	0,-1
--CAGUGCAAUGUUAAAAGGGCAa----
---------	14	0,-1
--CAGUGCAAUGUUAAAAGGGCAaa---
---------	6	0,-1
--CAGUGCAAUGUUAAAAGGGCAau---
---------	3	0,-1
--CAGUGCAAUGUUAAAAGGGCAU----
---------	192	0,0
--CAGUGCAAUGUUAAAAGGGCAUa---
---------	83	0,0
--CAGUGCAAUGUUAAAAGGGCAUaa--
---------	8	0,0
--CAGUGCAAUGUUAAAAGGGCAUau--
---------	7	0,0
--CAGUGCAAUGUUAAAAGGGCAUU---
---------	126	0,1
--CAGUGCAAUGUUAAAAGGGCAUUa--
---------	11	0,1
--CAGUGCAAUGUUAAAAGGGCAUUu--
---------	9	0,1
---AGUGCAAUGUUAAAAGGG-------
---------	5	1,-3
---AGUGCAAUGUUAAAAGGGC------
---------	5	1,-2
---AGUGCAAUGUUAAAAGGGCAU----

---------   2   1,0
---AGUGCAAUGUUAAAAGGGCAUaa--
---------   2   1,0
---AGUGCAAUGUUAAAAGGGCAUU---
---------   2   1,1

>hsa-mir-130b   mi:13-33;MA:51-72   SRX018957
ggccugcccgacACUCUUUCCCUGUUGCACUACuauaggccgcugggaagCAGUGCAAUGAUGAAAGGGCAUcggucagg
uc
((((((.((((..(((((((..(((((((((.((.(((....)))...)).)))))))))..)))))))..)))).))))
))
------------ACUCUUUCCCUGUUGCACUACU--
--   3   0,1
--CAGUGCAAUGAUGAAAGG------------
--   14   0,-4
--CAGUGCAAUGAUGAAAGGG-----------
--   8   0,-3
--CAGUGCAAUGAUGAAAGGGC----------
--   22   0,-2
--CAGUGCAAUGAUGAAAGGGCA---------
--   8   0,-1
--CAGUGCAAUGAUGAAAGGGCAa--------
--   17   0,-1
--CAGUGCAAUGAUGAAAGGGCAU--------
--   95   0,0
--CAGUGCAAUGAUGAAAGGGCAUa-------
--   13   0,0
--CAGUGCAAUGAUGAAAGGGCAUau------
--   4   0,0
--CAGUGCAAUGAUGAAAGGGCAUu-------
--   17   0,0
--CAGUGCAAUGAUGAAAGGGCAUuu------
--   13   0,0

>hsa-mir-132   mi:23-44;MA:59-80   SRX018957
ccgcccccgcgucuccagggcaACCGUGGCUUUCGAUUGUUACUgugggaacuggaggUAACAGUCUACAGCCAUGGUCG
ccccgcagcacgcccacgcgc
.(((....((((((.(.((((.(((((((((...((((((((((..(....)....))))))))))...))))))))).)

))).).)).))))....))).
--------------------AACCGUGGCUUUCGAUUGUUA--
--------------------   3   -1,-2
---UAACAGUCUACAGCCAUGG---
--------------------   5   0,-3
---UAACAGUCUACAGCCAUGGU--
--------------------   6   0,-2
---UAACAGUCUACAGCCAUGGUCa
--------------------   2   0,-1
---UAACAGUCUACAGCCAUGGUCG
--------------------   14   0,0
---UAACAGUCUACAGCCAUGGUCG
a-------------------   2   0,0
---UAACAGUCUACAGCCAUGGUCG
u-------------------   13   0,0

>hsa-mir-133a-1 3p:53-74   SRX018957
acaaugcuuugcuagagcugguaaaauggaaccaaaucgccucuucaauggaUUUGGUCCCCUUCAACCAGCUGuagcua
ugcauuga
.(((((...((((.(((((((..((.((.((((((((...........)))))))).)).))..))))))).))))..
.)))))).
---------------AGCUGGUAAAAUGGAACCAA---
--------   2

>hsa-mir-133a-2 3p:59-80   SRX018957
gggagccaaaugcuuugcuagagcugguaaaauggaaccaaaucgacuguccaauggaUUUGGUCCCCUUCAACCAGCUG
uagcugugcauugauggcgccg
.((.(((((((((...((((.(((((((..((.((.((((((((.(........).)))))))).)).))..))))))).
))))...))))).)))).)).
--------------------AGCUGGUAAAAUGGAACCAA--
-------------------- 2

>hsa-mir-134   5p:8-29 SRX018957
cagggugUGUGACUGGUUGACCAGAGGGGcaugcacuguguucacccugugggccaccuagucaccaacccuc
.(((((..((((((((.((.(((.((((((((.....))))...)))).)))..)).))))))))..))))).
-------UGUGACUGGUUGACCAGA--   2
    0,-4
-------UGUGACUGGUUGACCAGAG---   12

0,-3

-------UGUGACUGGUUGACCAGAGa-- 6

0,-3

-------UGUGACUGGUUGACCAGAGG-- 6

0,-2

-------UGUGACUGGUUGACCAGAGGa--- 3

0,-2

-------UGUGACUGGUUGACCAGAGGaa-- 3

0,-2

-------UGUGACUGGUUGACCAGAGGG--- 17

0,-1

-------UGUGACUGGUUGACCAGAGGGa-- 13

0,-1

-------UGUGACUGGUUGACCAGAGGGaa--- 3

0,-1

-------UGUGACUGGUUGACCAGAGGGau--- 2

0,-1

-------UGUGACUGGUUGACCAGAGGGG-- 24

0,0

-------UGUGACUGGUUGACCAGAGGGGa--- 2

0,0

-------UGUGACUGGUUGACCAGAGGGGau-- 6

0,0

-------UGUGACUGGUUGACCAGAGGGGu--- 3

0,0

-------UGUGACUGGUUGACCAGAGGGGuu-- 2

0,0

>hsa-mir-136 MA:15-37;mi:49-70 SRX018957

ugagcccucggaggACUCCAUUUGUUUUGAUGAUGGAuucuuaugcucCAUCAUCGUCUCAAAUGAGUCUucagaggguu
cu

.(((((((.(((((((((.(((((...((((((((((.........))))))))))...))))))))))))).)))))))
).

--------------ACUCCAUUUGUUUUGAUGAU--
-- 6 0,-3

--CAUCAUCGUCUCAAAUGA--------------
-- 4 0,-4

--CAUCAUCGUCUCAAAUGAG-------------

-- 17 0,-3
--CAUCAUCGUCUCAAAUGAGUC-----------
-- 13 0,-1
--CAUCAUCGUCUCAAAUGAGUCU----------
-- 2 0,0

>hsa-mir-137 3p:59-81 SRX018957
gguccucugacucucuucggugacggguauucuuggguggauaaauacggauuacguugUUAUUGCUUAAGAAUACGCGUA
Gucgaggagaguaccagcggca
.(.((.((((((((((((((..(((.(((((((((((..(.(((((((.....)).))))))..))))))))))).))).
.)))))))))))..))).))).
--AUUGCUUAAGAAUACGCGUA
---------------------- 2 2,-1

>hsa-mir-138-1 MA:23-45;mi:63-84 SRX018957
cccuggcauggugugguggggcAGCUGGUGUUGUGAAUCAGGCCGuugccaaucagagaacgGCUACUUCACAACACCAG
GGCCacaccacacuacagg
.((((...((((((((((.(((..(((((((((((((...(((((((..(.....)..)))))))..)))))))))))))
.))).))))))))))))))
----------------------AGCUGGUGUUGUGAAUCA--
------------------- 2 0,-5

>hsa-mir-138-2 MA:10-32;mi:57-78 SRX018957
cguugcugcAGCUGGUGUUGUGAAUCAGGCCGacgagcagcgcauccucuuacccgGCUAUUUCACGACACCAGGGUUgc
auca
.(.(((.((..(((((((((((((...(((((..(((.((......)))))...)))))..))))))))))))).)).))
).).
---------AGCUGGUGUUGUGAAUCA---
---- 2 0,-5
---------AGCUGGUGUUGUGAAUCAGGCCGA---
---- 2 0,1

>hsa-mir-139 5p:7-28;3p:44-65 SRX018957
guguauUCUACAGUGCACGUGUCUCCAGuguggcucggaggcuGGAGACGCGGCCCUGUUGGAGUaac
...(((((((((.((.(((((((((((((.(.......).)))))))))))))).))).)))))))..
------UCUACAGUGCACGUGUCU-- 5 0,-4
------UCUACAGUGCACGUGUCUC--- 16 0,-3
------UCUACAGUGCACGUGUCUCC-- 19 0,-2

```
------UCUACAGUGCACGUGUCUCCA-----------------------------------------   107 0,-1
------UCUACAGUGCACGUGUCUCCAa----------------------------------------   5   0,-1
------UCUACAGUGCACGUGUCUCCAu----------------------------------------   14  0,-1
------UCUACAGUGCACGUGUCUCCAG----------------------------------------   90  0,0
------UCUACAGUGCACGUGUCUCCAGa---------------------------------------   19  0,0
------UCUACAGUGCACGUGUCUCCAGU---------------------------------------   187 0,1
------UCUACAGUGCACGUGUCUCCAGUa--------------------------------------   6   0,1
------UCUACAGUGCACGUGUCUCCAGUaa-------------------------------------   2   0,1
------UCUACAGUGCACGUGUCUCCAGUu--------------------------------------   16  0,1
-------CUACAGUGCACGUGUCUCCA-----------------------------------------   2   1,-1
-------CUACAGUGCACGUGUCUCCAaa---------------------------------------   2   1,-1
--------UACAGUGCACGUGUCUCCAGU---------------------------------------   2   2,1
------------------------------------------UGGAGACGCGGCCCUGUU--------   24
    -1,-5
------------------------------------------UGGAGACGCGGCCCUGUUa-------   2
    -1,-5
------------------------------------------UGGAGACGCGGCCCUGUUG-------   75
    -1,-4
------------------------------------------UGGAGACGCGGCCCUGUUGa------   6
    -1,-4
------------------------------------------UGGAGACGCGGCCCUGUUGu------   3
    -1,-4
------------------------------------------UGGAGACGCGGCCCUGUUGG------   126
    -1,-3
------------------------------------------UGGAGACGCGGCCCUGUUGGu-----   3
    -1,-3
------------------------------------------UGGAGACGCGGCCCUGUUGGA-----   219
    -1,-2
------------------------------------------UGGAGACGCGGCCCUGUUGGAa----   64
    -1,-2
------------------------------------------UGGAGACGCGGCCCUGUUGGAaa---   67
    -1,-2
------------------------------------------UGGAGACGCGGCCCUGUUGGAau---   5
    -1,-2
------------------------------------------UGGAGACGCGGCCCUGUUGGAu----   14
    -1,-2
------------------------------------------UGGAGACGCGGCCCUGUUGGAG----   476
    -1,-1
```

--UGGAGACGCGGCCCUGUUGGAGa--- 229 -1,-1
--UGGAGACGCGGCCCUGUUGGAGaa-- 15 -1,-1
--UGGAGACGCGGCCCUGUUGGAGau-- 2 -1,-1
--UGGAGACGCGGCCCUGUUGGAGU--- 832 -1,0
--UGGAGACGCGGCCCUGUUGGAGUu-- 22 -1,0
--UGGAGACGCGGCCCUGUUGGAGUuu- 7 -1,0
--UGGAGACGCGGCCCUGUUGGAGUA-- 14 -1,1
--UGGAGACGCGGCCCUGUUGGAGUAu- 4 -1,1
--UGGAGACGCGGCCCUGUUGGAGUAA- 3 -1,2
---GGAGACGCGGCCCUGUUGG------ 4 0,-3
---GGAGACGCGGCCCUGUUGGA----- 3 0,-2
---GGAGACGCGGCCCUGUUGGAa---- 2 0,-2
---GGAGACGCGGCCCUGUUGGAG---- 7 0,-1
---GGAGACGCGGCCCUGUUGGAGU--- 33 0,0
---GGAGACGCGGCCCUGUUGGAGUA-- 3 0,1
---AGACGCGGCCCUGUUGGAGUu-- 3 2,0
---AGACGCGGCCCUGUUGGAGUuu- 8 2,0
---AGACGCGGCCCUGUUGGAGUAu- 3 2,1

>hsa-mir-140 5p:23-44;3p:62-82 SRX018957
ugugucucucucuguguccugcCAGUGGUUUUACCCUAUGGUAGguuacgucaugcuguucUACCACAGGGUAGAACCACGGacaggauaccggggcacc
.(((((((.....((((((((((.(((((((((((((.(((((((..(((......)))))))))).))))))))))))))).)))))))).))))))).
--CUACCACAGGGUAGAACCACGG------------------ 5 -1,0
--CUACCACAGGGUAGAACCACGGA----------------- 8 -1,1
--CUACCACAGGGUAGAACCACGGAaa--------------- 2 -1,1
--CUACCACAGGGUAGAACCACGGAC---------------- 3 -1,2
--CUACCACAGGGUAGAACCACGGACA--------------- 3 -1,3
---UACCACAGGGUAGAACCA-

--------------------	37	0,-3
--UACCACAGGGUAGAACCAC
--------------------	39	0,-2
--UACCACAGGGUAGAACCAC
G-------------------	514	0,-1
--UACCACAGGGUAGAACCAC
Ga------------------	7	0,-1
--UACCACAGGGUAGAACCAC
Gaa-----------------	5	0,-1
--UACCACAGGGUAGAACCAC
Gu------------------	5	0,-1
--UACCACAGGGUAGAACCAC
GG------------------	7015	0,0
--UACCACAGGGUAGAACCAC
GGu-----------------	85	0,0
--UACCACAGGGUAGAACCAC
GGuu----------------	24	0,0
--UACCACAGGGUAGAACCAC
GGA-----------------	7956	0,1
--UACCACAGGGUAGAACCAC
GGAa----------------	2196	0,1
--UACCACAGGGUAGAACCAC
GGAaa---------------	917	0,1
--UACCACAGGGUAGAACCAC
GGAau---------------	425	0,1
--UACCACAGGGUAGAACCAC
GGAu----------------	229	0,1
--UACCACAGGGUAGAACCAC
GGAuu---------------	9	0,1
--UACCACAGGGUAGAACCAC
GGAC----------------	1787	0,2
--UACCACAGGGUAGAACCAC
GGACu---------------	52	0,2
--UACCACAGGGUAGAACCAC
GGACuu--------------	3	0,2
--UACCACAGGGUAGAACCAC
GGACA---------------	1083	0,3
--UACCACAGGGUAGAACCAC

GGACAa--------------   42   0,3

--UACCACAGGGUAGAACCAC

GGACAu--------------   30   0,3

---ACCACAGGGUAGAACCAC

--------------------   13   1,-2

---ACCACAGGGUAGAACCAC

G-------------------   35   1,-1

---ACCACAGGGUAGAACCAC

Ga------------------   3   1,-1

---ACCACAGGGUAGAACCAC

GG------------------   895 1,0

---ACCACAGGGUAGAACCAC

GGu-----------------   58   1,0

---ACCACAGGGUAGAACCAC

GGuu----------------   36   1,0

---ACCACAGGGUAGAACCAC

GGA-----------------   9106   1,1

---ACCACAGGGUAGAACCAC

GGAa----------------   2834   1,1

---ACCACAGGGUAGAACCAC

GGAaa---------------   1054   1,1

---ACCACAGGGUAGAACCAC

GGAau---------------   189 1,1

---ACCACAGGGUAGAACCAC

GGAu----------------   1096   1,1

---ACCACAGGGUAGAACCAC

GGAuu---------------   71   1,1

---ACCACAGGGUAGAACCAC

GGAC----------------   2751   1,2

---ACCACAGGGUAGAACCAC

GGACu---------------   44   1,2

---ACCACAGGGUAGAACCAC

GGACA---------------   254 1,3

---ACCACAGGGUAGAACCAC

GGACAa--------------   145 1,3

---ACCACAGGGUAGAACCAC

GGACAaa-------------   8   1,3

---ACCACAGGGUAGAACCAC

GGACAau-------------   4   1,3
--ACCACAGGGUAGAACCAC
GGACAu--------------   25 1,3
--ACCACAGGGUAGAACCAC
GGACAG--------------   12 1,4
--ACCACAGGGUAGAACCAC
GGACAGa-------------   4   1,4
---CCACAGGGUAGAACCAC
GG------------------   31 2,0
---CCACAGGGUAGAACCAC
GGuu----------------   2   2,0
---CCACAGGGUAGAACCAC
GGA-----------------   264 2,1
---CCACAGGGUAGAACCAC
GGAa----------------   56 2,1
---CCACAGGGUAGAACCAC
GGAaa---------------   34 2,1
---CCACAGGGUAGAACCAC
GGAau---------------   5   2,1
---CCACAGGGUAGAACCAC
GGAu----------------   42 2,1
---CCACAGGGUAGAACCAC
GGAuu---------------   3   2,1
---CCACAGGGUAGAACCAC
GGAC----------------   78 2,2
---CCACAGGGUAGAACCAC
GGACA---------------   5   2,3
---CCACAGGGUAGAACCAC
GGACAa--------------   12 2,3

>hsa-mir-142   5p:16-36;3p:52-74   SRX018957
gacagugcagucaccCAUAAAGUAGAAAGCACUACUaacagcacuggagggUGUAGUGUUUCCUACUUUAUGGAugagug
uacugug
.((((((((.(((.(((((((((((.(((((((((...(..(....)..)..))))))))).))))))))))).))).))
)))))).
-------------CCCAUAAAGUAGAAAGCA---
------- 3   -2,-5
-------------CCCAUAAAGUAGAAAGCAa--

------- 4 -2,-5
-------------CCCAUAAAGUAGAAAGCAC---
------- 11 -2,-4
-------------CCCAUAAAGUAGAAAGCACU--
------- 60 -2,-3
-------------CCCAUAAAGUAGAAAGCACUA---
------- 32 -2,-2
-------------CCCAUAAAGUAGAAAGCACUAa--
------- 4 -2,-2
-------------CCCAUAAAGUAGAAAGCACUAu--
------- 3 -2,-2
-------------CCCAUAAAGUAGAAAGCACUAC--
------- 15 -2,-1
-------------CCCAUAAAGUAGAAAGCACUACa---
------- 2 -2,-1
--------------CCAUAAAGUAGAAAGCACU--
------- 4 -1,-3
--------------CCAUAAAGUAGAAAGCACUA---
------- 2 -1,-2
--------------CCAUAAAGUAGAAAGCACUAa--
------- 2 -1,-2
---------------CAUAAAGUAGAAAGCACU--
------- 8 0,-3
---------------CAUAAAGUAGAAAGCACUA---
------- 23 0,-2
---------------CAUAAAGUAGAAAGCACUAa--
------- 6 0,-2
---------------CAUAAAGUAGAAAGCACUAu--
------- 2 0,-2
---------------CAUAAAGUAGAAAGCACUAuu---
------- 2 0,-2
---------------CAUAAAGUAGAAAGCACUAC--
------- 8 0,-1
---------------CAUAAAGUAGAAAGCACUACa---
------- 2 0,-1
---------------CAUAAAGUAGAAAGCACUACU---
------- 28 0,0
---------------CAUAAAGUAGAAAGCACUACUA--

------- 19 0,1
---------------CAUAAAGUAGAAAGCACUACUAu--
------- 3 0,1
---------------CAUAAAGUAGAAAGCACUACUAA--
------- 2 0,2
----------------AUAAAGUAGAAAGCACUA--
------- 3 1,-2
----------------AUAAAGUAGAAAGCACUAa---
------- 3 1,-2
----------------AUAAAGUAGAAAGCACUAC---
------- 2 1,-1
----------------AUAAAGUAGAAAGCACUACUA---
------- 12 1,1
----------------AUAAAGUAGAAAGCACUACUAu--
------- 3 1,1
----------------AUAAAGUAGAAAGCACUACUAA--
------- 48 1,2
--GUAGUGUUUCCUACUUUAUGG-------
------- 4 1,-1
--GUAGUGUUUCCUACUUUAUGGA------
------- 3 1,0

>hsa-mir-143 mi:27-48;MA:61-81 SRX018957
gcgcagcgcccugucucccagccugaGGUGCAGUGCUGCAUCUCUGGUcaguugggagucUGAGAUGAAGCACUGUAGCU
Caggaagagagaaguuguucugcagc
..((((.((....((((.(..((((((.((((((((.((((((.((((......)).)).))))))).))))))))).))
))))..).))))....)).))))...
-------------------------AGGUGCAGUGCUGCAUCUCU-----------------------------------
-------------------------- 12 -1,-3
--------------------------GGUGCAGUGCUGCAUCUC------------------------------------
-------------------------- 223 0,-4
--------------------------GGUGCAGUGCUGCAUCUCU-----------------------------------
-------------------------- 1124 0,-3
--------------------------GGUGCAGUGCUGCAUCUCUG----------------------------------
-------------------------- 30 0,-2
--------------------------GGUGCAGUGCUGCAUCUCUGa---------------------------------
-------------------------- 2 0,-2
--------------------------GGUGCAGUGCUGCAUCUCUGaa--------------------------------

------------------------- 5 0,-2
-------------------------GGUGCAGUGCUGCAUCUCUGG--------------------------------
------------------------- 24 0,-1
-------------------------GGUGCAGUGCUGCAUCUCUGGa-------------------------------
------------------------- 10 0,-1
-------------------------GGUGCAGUGCUGCAUCUCUGGaa------------------------------
------------------------- 15 0,-1
-------------------------GGUGCAGUGCUGCAUCUCUGGU-------------------------------
------------------------- 6 0,0
-------------------------GGUGCAGUGCUGCAUCUCUGGUa------------------------------
------------------------- 8 0,0
-------------------------GGUGCAGUGCUGCAUCUCUGGUaa-----------------------------
------------------------- 5 0,0
-------------------------GGUGCAGUGCUGCAUCUCUGGUau-----------------------------
------------------------- 11 0,0
-------------------------GGUGCAGUGCUGCAUCUCUGGUu------------------------------
------------------------- 2 0,0
-------------------------GGUGCAGUGCUGCAUCUCUGGUC------------------------------
------------------------- 2 0,1
-------------------------GGUGCAGUGCUGCAUCUCUGGUCA-----------------------------
------------------------- 2 0,2
-------------------------GGUGCAGUGCUGCAUCUCUGGUCAu----------------------------
------------------------- 2 0,2
--UCUGAGAUGAAGCACUGUAGC-
------------------------- 3 -2,-2
--UCUGAGAUGAAGCACUGUAGCU
------------------------- 4 -2,-1
--UCUGAGAUGAAGCACUGUAGCU
C------------------------ 2 -2,0
---CUGAGAUGAAGCACUGUA---
------------------------- 2 -1,-4
---CUGAGAUGAAGCACUGUAa--
------------------------- 2 -1,-4
---CUGAGAUGAAGCACUGUAGCU
------------------------- 25 -1,-1
---CUGAGAUGAAGCACUGUAGCU
C------------------------ 23 -1,0
---CUGAGAUGAAGCACUGUAGCU

Cu----------------------- 8   -1,0
---CUGAGAUGAAGCACUGUAGCU
CA----------------------- 2   -1,1
--UGAGAUGAAGCACUGUAG--
------------------------- 145 0,-3
--UGAGAUGAAGCACUGUAGa-
------------------------- 3   0,-3
--UGAGAUGAAGCACUGUAGaa
------------------------- 9   0,-3
--UGAGAUGAAGCACUGUAGuu
------------------------- 3   0,-3
--UGAGAUGAAGCACUGUAGC-
------------------------- 230 0,-2
--UGAGAUGAAGCACUGUAGCa
------------------------- 91   0,-2
--UGAGAUGAAGCACUGUAGCa
a------------------------ 116 0,-2
--UGAGAUGAAGCACUGUAGCa
u------------------------ 4   0,-2
--UGAGAUGAAGCACUGUAGCU
------------------------- 3272   0,-1
--UGAGAUGAAGCACUGUAGCU
a------------------------ 378 0,-1
--UGAGAUGAAGCACUGUAGCU
aa----------------------- 64   0,-1
--UGAGAUGAAGCACUGUAGCU
au----------------------- 37   0,-1
--UGAGAUGAAGCACUGUAGCU
u------------------------ 72   0,-1
--UGAGAUGAAGCACUGUAGCU
uu----------------------- 13   0,-1
--UGAGAUGAAGCACUGUAGCU
C------------------------ 6179   0,0
--UGAGAUGAAGCACUGUAGCU
Cu----------------------- 1445   0,0
--UGAGAUGAAGCACUGUAGCU
Cuu---------------------- 158 0,0
--UGAGAUGAAGCACUGUAGCU

CA------------------------ 436 0,1
--UGAGAUGAAGCACUGUAGCU
CAa----------------------- 43 0,1
--UGAGAUGAAGCACUGUAGCU
CAaa---------------------- 8   0,1
--UGAGAUGAAGCACUGUAGCU
CAau---------------------- 9   0,1
--UGAGAUGAAGCACUGUAGCU
CAu----------------------- 15 0,1
--UGAGAUGAAGCACUGUAGCU
CAG----------------------- 3   0,2
---GAGAUGAAGCACUGUAGCU
-------------------------- 2   1,-1
---GAGAUGAAGCACUGUAGCU
a------------------------- 2   1,-1
---GAGAUGAAGCACUGUAGCU
C------------------------- 19 1,0
---GAGAUGAAGCACUGUAGCU
Cu------------------------ 10 1,0
---GAGAUGAAGCACUGUAGCU
Cuu----------------------- 2   1,0
--AGAUGAAGCACUGUAGCU
C------------------------- 3   2,0
--AGAUGAAGCACUGUAGCU
Cu------------------------ 3   2,0

>hsa-mir-144   mi:15-36;MA:52-71   SRX018957
uggggcccuggcugGGAUAUCAUCAUAUACUGUAAGuuugcgaugagacacUACAGUAUAGAUGAUGUACUaguccgggc
accccc
.(((((((.((((((.((((((((.(((((((((.((((......))))..))))))))))))))))).)))))).))))
.)))..
------------UGGGAUAUCAUCAUAUACUG--
------ 3   -2,-4
--------------GGAUAUCAUCAUAUACUG--
------ 4   0,-4
--------------GGAUAUCAUCAUAUACUGUAAG--
------ 3   0,0
--------------GGAUAUCAUCAUAUACUGUAAGU---

------ 5 0,1
---UACAGUAUAGAUGAUGUA-----------
------ 4 0,-2
---UACAGUAUAGAUGAUGUAC----------
------ 2 0,-1
---UACAGUAUAGAUGAUGUACUA--------
------ 2 0,1
---UACAGUAUAGAUGAUGUACUAG-------
------ 3 0,2

>hsa-mir-145 MA:16-38;mi:54-75 SRX018957
caccuuguccucacgGUCCAGUUUUCCCAGGAAUCCCUuagaugcuaagauggGGAUUCCUGGAAAUACUGUUCUugagg
ucaugguu
.(((.((.(((((.((..((((.((.((((((((((((.............)))))))))))).)).)))).)))))))
.)).))).
---------------GUCCAGUUUUCCCAGGAA---
-------- 7 0,-5
---------------GUCCAGUUUUCCCAGGAAa--
-------- 12 0,-5
---------------GUCCAGUUUUCCCAGGAAaa---
-------- 2 0,-5
---------------GUCCAGUUUUCCCAGGAAU--
-------- 60 0,-4
---------------GUCCAGUUUUCCCAGGAAUa---
-------- 4 0,-4
---------------GUCCAGUUUUCCCAGGAAUaa--
-------- 6 0,-4
---------------GUCCAGUUUUCCCAGGAAUC---
-------- 20 0,-3
---------------GUCCAGUUUUCCCAGGAAUCa--
-------- 24 0,-3
---------------GUCCAGUUUUCCCAGGAAUCC--
-------- 78 0,-2
---------------GUCCAGUUUUCCCAGGAAUCCa---
-------- 25 0,-2
---------------GUCCAGUUUUCCCAGGAAUCCau--
-------- 3 0,-2
---------------GUCCAGUUUUCCCAGGAAUCCuu--

--------   4   0,-2
---------------GUCCAGUUUCCCAGGAAUCCC---
--------   134 0,-1
---------------GUCCAGUUUCCCAGGAAUCCCa--
--------   15   0,-1
---------------GUCCAGUUUCCCAGGAAUCCCau---
--------   2   0,-1
---------------GUCCAGUUUCCCAGGAAUCCCU--
--------   168 0,0
---------------GUCCAGUUUCCCAGGAAUCCCUa---
--------   3   0,0
---------------GUCCAGUUUCCCAGGAAUCCCUU---
--------   2   0,1
----------------UCCAGUUUCCCAGGAAUCCCU--
--------   3   1,0
---AUUCCUGGAAAUACUGUUCU-----
--------   15   2,0
---AUUCCUGGAAAUACUGUUCUa----
--------   4   2,0
---AUUCCUGGAAAUACUGUUCUU----
--------   5   2,1

>hsa-mir-1468   5p:13-33   SRX018957
ggugggugguuuCUCCGUUUGCCUGUUUCGCUGaugugcauucaacucauucucagcaaaauaagcaaauggaaaauucg
uccauc
((((..(..((.(((((((((.(((((.(((((.(((.........)))..))))).))))).))))))))).))..).
.)))))
------------CUCCGUUUGCCUGUUUCGC---
------ 2   0,-2
------------CUCCGUUUGCCUGUUUCGCU--
------ 13   0,-1
------------CUCCGUUUGCCUGUUUCGCUGA--
------ 4   0,1

>hsa-mir-146a   MA:21-42;mi:57-78   SRX018957
ccgauguguaucccucagcuuUGAGAACUGAAUUCCAUGGGUUgugucagugucagaCCUCUGAAAUUCAGUUCUUCAGcu
gggauaucucugucaucgu
.((((.(((((.(((((..(((((((((((.((.(((((.((.......))))))).)).))))))))))).))))

)))))))......))))).
-------------------UUGAGAACUGAAUUCCAUGGGUUG---
------------------- 2   -1,1
--------------------UGAGAACUGAAUUCCAUG--
------------------- 3   0,-4
--------------------UGAGAACUGAAUUCCAUGG---
------------------- 5   0,-3
--------------------UGAGAACUGAAUUCCAUGGG--
------------------- 20   0,-2
--------------------UGAGAACUGAAUUCCAUGGGU---
------------------- 25   0,-1
--------------------UGAGAACUGAAUUCCAUGGGUa--
------------------- 10   0,-1
--------------------UGAGAACUGAAUUCCAUGGGUaa---
------------------- 19   0,-1
--------------------UGAGAACUGAAUUCCAUGGGUau---
------------------- 36   0,-1
--------------------UGAGAACUGAAUUCCAUGGGUU--
------------------- 116 0,0
--------------------UGAGAACUGAAUUCCAUGGGUUa---
------------------- 175 0,0
--------------------UGAGAACUGAAUUCCAUGGGUUaa--
------------------- 29   0,0
--------------------UGAGAACUGAAUUCCAUGGGUUau--
------------------- 60   0,0
--------------------UGAGAACUGAAUUCCAUGGGUUu---
------------------- 63   0,0
--------------------UGAGAACUGAAUUCCAUGGGUUuu--
------------------- 8   0,0
--------------------UGAGAACUGAAUUCCAUGGGUUG---
------------------- 60   0,1
--------------------UGAGAACUGAAUUCCAUGGGUUGa--
------------------- 14   0,1
--------------------UGAGAACUGAAUUCCAUGGGUUGU--
------------------- 20   0,2
--------------------UGAGAACUGAAUUCCAUGGGUUGUa---------------------------------------
------------------- 2   0,2
---------------------GAGAACUGAAUUCCAUGGGUUa---

------------------ 2 1,0

>hsa-mir-146b 5p:9-30;3p:45-66 SRX018957
ccuggcacUGAGAACUGAAUUCCAUAGGCUgugagcucuagcaaUGCCCUGUGGACUCAGUUCUGGugcccgg
((.((((((.((((((((..((((((((..((..((....)).))..)))))))).)))))))))))))).))
-------CUGAGAACUGAAUUCCAU-- 2 -1,-5
-------CUGAGAACUGAAUUCCAUAGGC-- 4 -1,-1
-------CUGAGAACUGAAUUCCAUAGGCU--- 30 -1,0
-------CUGAGAACUGAAUUCCAUAGGCUG-- 2 -1,1
-------CUGAGAACUGAAUUCCAUAGGCUGU--- 12 -1,2
--------UGAGAACUGAAUUCCAUA--- 4 0,-4
--------UGAGAACUGAAUUCCAUAG-- 19 0,-3
--------UGAGAACUGAAUUCCAUAGG--- 84 0,-2
--------UGAGAACUGAAUUCCAUAGGa-- 2 0,-2
--------UGAGAACUGAAUUCCAUAGGau--- 2 0,-2
--------UGAGAACUGAAUUCCAUAGGC-- 228 0,-1
--------UGAGAACUGAAUUCCAUAGGCa--- 88 0,-1
--------UGAGAACUGAAUUCCAUAGGCaa-- 40 0,-1
--------UGAGAACUGAAUUCCAUAGGCau-- 24 0,-1
--------UGAGAACUGAAUUCCAUAGGCU--- 1325 0,0
--------UGAGAACUGAAUUCCAUAGGCUa-- 140 0,0
--------UGAGAACUGAAUUCCAUAGGCUaa--- 22

0,0

--------UGAGAACUGAAUUCCAUAGGCUau-- 164

0,0

--------UGAGAACUGAAUUCCAUAGGCUu--- 219

0,0

--------UGAGAACUGAAUUCCAUAGGCUuu-- 95

0,0

--------UGAGAACUGAAUUCCAUAGGCUG--- 305

0,1

--------UGAGAACUGAAUUCCAUAGGCUGa-- 486

0,1

--------UGAGAACUGAAUUCCAUAGGCUGaa--- 20

0,1

--------UGAGAACUGAAUUCCAUAGGCUGau--- 23

0,1

--------UGAGAACUGAAUUCCAUAGGCUGU-- 5726

0,2

--------UGAGAACUGAAUUCCAUAGGCUGUa--- 266

0,2

--------UGAGAACUGAAUUCCAUAGGCUGUu--- 61

0,2

--------UGAGAACUGAAUUCCAUAGGCUGUG--- 8

0,3

---------GAGAACUGAAUUCCAUAGGCa-- 4

1,-1

---------GAGAACUGAAUUCCAUAGGCU-- 18

1,0

---------GAGAACUGAAUUCCAUAGGCUa--- 3

1,0

---------GAGAACUGAAUUCCAUAGGCUau-- 3

1,0

---------GAGAACUGAAUUCCAUAGGCUuu-- 2

1,0

---------GAGAACUGAAUUCCAUAGGCUGa-- 3

1,1

---------GAGAACUGAAUUCCAUAGGCUGU-- 24

1,2

---------GAGAACUGAAUUCCAUAGGCUGUa--- 3

1,2

----------AGAACUGAAUUCCAUAGGCU---   2

2,0

--UGCCCUGUGGACUCAGUUCUGG-------   4

0,0

--UGCCCUGUGGACUCAGUUCUGGU------   3

0,1

---GCCCUGUGGACUCAGUUCUGG-------   19

1,0

---GCCCUGUGGACUCAGUUCUGGaa-----   2

1,0

---GCCCUGUGGACUCAGUUCUGGU------   9

1,1

---GCCCUGUGGACUCAGUUCUGGUa-----   5

1,1

---GCCCUGUGGACUCAGUUCUGGUau----   21

1,1

---GCCCUGUGGACUCAGUUCUGGUu-----   4

1,1

---GCCCUGUGGACUCAGUUCUGGUGu----   2

1,2

--CCCUGUGGACUCAGUUCUGGUau----   3

2,1

>hsa-mir-148a   mi:6-27;MA:44-65   SRX018957

gaggcAAAGUUCUGAGACACUCCGACUcugaguaugauagaagUCAGUGCACUACAGAACUUUGUcuc

((((((((((((((((.((((..(((((((.......))).))))))))..)).))))))))))))))

----CAAAGUUCUGAGACACUCCGA---   6

-1,-2

-----AAAGUUCUGAGACACUCCGA---   84   0,-2

-----AAAGUUCUGAGACACUCCGAC--   3   0,-1

-----AAAGUUCUGAGACACUCCGACU---   13   0,0

---UCAGUGCACUACAGAACU-------   4560

0,-4

---UCAGUGCACUACAGAACUa------   24   0,-4

---UCAGUGCACUACAGAACUaa-----   7   0,-4

---UCAGUGCACUACAGAACUU------   249   0,-3

---UCAGUGCACUACAGAACUUa-----   6   0,-3

---UCAGUGCACUACAGAACUUaa----   2   0,-3
---UCAGUGCACUACAGAACUUU-----   1160
0,-2
---UCAGUGCACUACAGAACUUUa----   23   0,-2
---UCAGUGCACUACAGAACUUUaa---   6   0,-2
---UCAGUGCACUACAGAACUUUau---   4   0,-2
---UCAGUGCACUACAGAACUUUu----   7   0,-2
---UCAGUGCACUACAGAACUUUuu---   7   0,-2
---UCAGUGCACUACAGAACUUUG----   2464
0,-1
---UCAGUGCACUACAGAACUUUGa---   3150
0,-1
---UCAGUGCACUACAGAACUUUGaa--   360 0,-1
---UCAGUGCACUACAGAACUUUGau--   2   0,-1
---UCAGUGCACUACAGAACUUUGU---   25920
0,0
---UCAGUGCACUACAGAACUUUGUa--   129 0,0
---UCAGUGCACUACAGAACUUUGUaa-   48   0,0
---UCAGUGCACUACAGAACUUUGUau-   2   0,0
---UCAGUGCACUACAGAACUUUGUu--   125 0,0
---UCAGUGCACUACAGAACUUUGUuu-   6   0,0
---UCAGUGCACUACAGAACUUUGUC--   396 0,1
---UCAGUGCACUACAGAACUUUGUCa-   22   0,1
---UCAGUGCACUACAGAACUUUGUCU-   63   0,2
---UCAGUGCACUACAGAACUUUGUCUa   2   0,2
--CAGUGCACUACAGAACUUUGU---   33   1,0
---AGUGCACUACAGAACUUU-----   33   2,-2
---AGUGCACUACAGAACUUUG----   35   2,-1
---AGUGCACUACAGAACUUUGa---   31   2,-1
---AGUGCACUACAGAACUUUGaa--   4   2,-1
---AGUGCACUACAGAACUUUGU---   361 2,0
---AGUGCACUACAGAACUUUGUa--   2   2,0
---AGUGCACUACAGAACUUUGUaa-   2   2,0
---AGUGCACUACAGAACUUUGUC--   8   2,1

>hsa-mir-148b   mi:25-46;MA:63-84   SRX018957
caagcacgauuagcauuugaggugAAGUUCUGUUAUACACUCAGGCuguggcucucugaaagUCAGUGCAUCACAGAACU
UUGUcucgaaagcuuucua

.......((..(((.(((((((..((((((((.((.((((..((((....(.....)..)))))))).)).))))))))
)..))))))).))).))..
-----------------------GAAGUUCUGUUAUACACUC--------------------------------------
-------------------- 5 -1,-4
-----------------------GAAGUUCUGUUAUACACUCAG------------------------------------
-------------------- 2 -1,-2
-----------------------GAAGUUCUGUUAUACACUCAGG-----------------------------------
-------------------- 7 -1,-1
-----------------------GAAGUUCUGUUAUACACUCAGGCa---------------------------------
-------------------- 2 -1,0
-----------------------GAAGUUCUGUUAUACACUCAGGCau--------------------------------
-------------------- 6 -1,0
-----------------------GAAGUUCUGUUAUACACUCAGGCU---------------------------------
-------------------- 12 -1,1
-----------------------GAAGUUCUGUUAUACACUCAGGCUa--------------------------------
-------------------- 3 -1,1
---UCAGUGCAUCACAGAACU
-------------------- 1373 0,-4
---UCAGUGCAUCACAGAACU
U------------------- 105 0,-3
---UCAGUGCAUCACAGAACU
Ua------------------ 3 0,-3
---UCAGUGCAUCACAGAACU
UU------------------ 369 0,-2
---UCAGUGCAUCACAGAACU
UUa----------------- 3 0,-2
---UCAGUGCAUCACAGAACU
UUG----------------- 42 0,-1
---UCAGUGCAUCACAGAACU
UUGa---------------- 63 0,-1
---UCAGUGCAUCACAGAACU
UUGaa--------------- 7 0,-1
---UCAGUGCAUCACAGAACU
UUGU---------------- 645 0,0
---UCAGUGCAUCACAGAACU
UUGUa--------------- 9 0,0
---UCAGUGCAUCACAGAACU
UUGUaa-------------- 4 0,0

--UCAGUGCAUCACAGAACU
UUGUau------------- 3   0,0
--UCAGUGCAUCACAGAACU
UUGUu-------------- 124 0,0
--UCAGUGCAUCACAGAACU
UUGUuu------------- 13 0,0
---CAGUGCAUCACAGAACU
UUGU--------------- 7   1,0
--AGUGCAUCACAGAACU
UU----------------- 8   2,-2
--AGUGCAUCACAGAACU
UUGU--------------- 8   2,0
--AGUGCAUCACAGAACU
UUGUu-------------- 2   2,0

>hsa-mir-149   MA:15-37;mi:56-76   SRX018957
gccggcgcccgagcUCUGGCUCCGUGUCUUCACUCCCgugcuuguccgaggagggAGGGAGGGACGGGGGCUGUGCuggg
gcagcugga
.(((((((((.(((.(.(.((((((.(((((.(((((.(.(((....))).))))))))))).)))))).).).))).))
)).))))).
--GAGGGAGGGACGGGGGCUGUGC----
---------   2   -1,0

>hsa-mir-150   MA:16-37;mi:51-72   SRX018957
cuccccauggcccugUCUCCCAACCCUUGUACCAGUGcugggcucagaccCUGGUACAGGCCUGGGGGACAGggaccugg
ggac
.((((((.((((((((((((((..(((.(((((((.((((....))).).))))))))))..))))))))))))).)))))
))).
---------------UCUCCCAACCCUUGUACCA--
----   4   0,-3
---------------UCUCCCAACCCUUGUACCAGU--
----   11 0,-1
---------------UCUCCCAACCCUUGUACCAGUa---
----   4   0,-1
---------------UCUCCCAACCCUUGUACCAGUG---
----   56 0,0
---------------UCUCCCAACCCUUGUACCAGUGa--
----   17 0,0

---------------UCUCCCAACCCUUGUACCAGUGu--
----   6   0,0
--CUGGUACAGGCCUGGGGGACAu--------
----   2   0,-1

>hsa-mir-151   5p:11-31;3p:47-67   SRX018957
uuuccugcccUCGAGGAGCUCACAGUCUAGUaugucucaucccccuaCUAGACUGAAGCUCCUUGAGGacagggaugguca
uacucaccuc
.((((((.((((((((((((..((((((((((.(........).))))))))))).))))))))))))).))))))......
....
---------CUCGAGGAGCUCACAGUCUAGU---
----------   18   -1,0
---------CUCGAGGAGCUCACAGUCUAGUA--
----------   9   -1,1
----------UCGAGGAGCUCACAGUCU--
----------   5   0,-3
----------UCGAGGAGCUCACAGUCUu---
----------   2   0,-3
----------UCGAGGAGCUCACAGUCUA---
----------   3   0,-2
----------UCGAGGAGCUCACAGUCUAu--
----------   2   0,-2
----------UCGAGGAGCUCACAGUCUAG--
----------   48   0,-1
----------UCGAGGAGCUCACAGUCUAGa---
----------   6   0,-1
----------UCGAGGAGCUCACAGUCUAGaa--
----------   3   0,-1
----------UCGAGGAGCUCACAGUCUAGU---
----------   408   0,0
----------UCGAGGAGCUCACAGUCUAGUu--
----------   3   0,0
----------UCGAGGAGCUCACAGUCUAGUA--
----------   265   0,1
----------UCGAGGAGCUCACAGUCUAGUAa---
----------   55   0,1
----------UCGAGGAGCUCACAGUCUAGUAU---
----------   43   0,2

----------UCGAGGAGCUCACAGUCUAGUAUG--
---------- 3 0,3
-----------CGAGGAGCUCACAGUCUAGU---
---------- 2 1,0
------------GAGGAGCUCACAGUCUAG--
---------- 2 2,-1
------------GAGGAGCUCACAGUCUAGU---
---------- 15 2,0
------------GAGGAGCUCACAGUCUAGUA--
---------- 9 2,1
------------GAGGAGCUCACAGUCUAGUAU---
---------- 2 2,2
--UACUAGACUGAAGCUCCUUGA---------------
---------- 18 -2,-2
--UACUAGACUGAAGCUCCUUGAa--------------
---------- 9 -2,-2
--UACUAGACUGAAGCUCCUUGAaa-------------
---------- 2 -2,-2
--UACUAGACUGAAGCUCCUUGAG--------------
---------- 31 -2,-1
--UACUAGACUGAAGCUCCUUGAGa-------------
---------- 19 -2,-1
--UACUAGACUGAAGCUCCUUGAGu-------------
---------- 3 -2,-1
--UACUAGACUGAAGCUCCUUGAGG-------------
---------- 23 -2,0
--UACUAGACUGAAGCUCCUUGAGGu------------
---------- 4 -2,0
--UACUAGACUGAAGCUCCUUGAGGA------------
---------- 14 -2,1
---ACUAGACUGAAGCUCCUUGAG--------------
---------- 6 -1,-1
---ACUAGACUGAAGCUCCUUGAGa-------------
---------- 4 -1,-1
---ACUAGACUGAAGCUCCUUGAGG-------------
---------- 10 -1,0
---ACUAGACUGAAGCUCCUUGAGGA------------
---------- 12 -1,1

--CUAGACUGAAGCUCCUUGA---------------
---------- 24 0,-2
--CUAGACUGAAGCUCCUUGAa--------------
---------- 7 0,-2
--CUAGACUGAAGCUCCUUGAaa-------------
---------- 2 0,-2
--CUAGACUGAAGCUCCUUGAG--------------
---------- 103 0,-1
--CUAGACUGAAGCUCCUUGAGa-------------
---------- 35 0,-1
--CUAGACUGAAGCUCCUUGAGaa------------
---------- 7 0,-1
--CUAGACUGAAGCUCCUUGAGu-------------
---------- 6 0,-1
--CUAGACUGAAGCUCCUUGAGG-------------
---------- 442 0,0
--CUAGACUGAAGCUCCUUGAGGu------------
---------- 37 0,0
--CUAGACUGAAGCUCCUUGAGGA------------
---------- 925 0,1
--CUAGACUGAAGCUCCUUGAGGAa-----------
---------- 715 0,1
--CUAGACUGAAGCUCCUUGAGGAaa----------
---------- 425 0,1
--CUAGACUGAAGCUCCUUGAGGAau----------
---------- 97 0,1
--CUAGACUGAAGCUCCUUGAGGAu-----------
---------- 98 0,1
--CUAGACUGAAGCUCCUUGAGGAuu----------
---------- 13 0,1
--CUAGACUGAAGCUCCUUGAGGAC-----------
---------- 24 0,2
--CUAGACUGAAGCUCCUUGAGGACu----------
---------- 7 0,2
---UAGACUGAAGCUCCUUGAGG-------------
---------- 13 1,0
---UAGACUGAAGCUCCUUGAGGA------------
---------- 23 1,1

---UAGACUGAAGCUCCUUGAGGAa-----------
---------- 33 1,1
---UAGACUGAAGCUCCUUGAGGAaa----------
---------- 10 1,1
---UAGACUGAAGCUCCUUGAGGAau----------
---------- 6 1,1
---UAGACUGAAGCUCCUUGAGGAu-----------
---------- 2 1,1
---UAGACUGAAGCUCCUUGAGGACA----------
---------- 2 1,3
--AGACUGAAGCUCCUUGAGGAC-----------
---------- 2 2,2
--AGACUGAAGCUCCUUGAGGACA----------
---------- 2 2,3

>hsa-mir-152 3p:54-74 SRX018957
ugucccccccggcccagguucugugauacacuccgacucgggcucuggagcagUCAGUGCAUGACAGAACUUGGgcccgg
aaggacc
.((((..((.((((((((((((((.((.((((..((((...(((....))))))))))).)).)))))))))))))).))
..)))).
---UCAGUGCAUGACAGAACU---------
------- 635 0,-3
---UCAGUGCAUGACAGAACUU--------
------- 31 0,-2
---UCAGUGCAUGACAGAACUUu-------
------- 3 0,-2
---UCAGUGCAUGACAGAACUUG-------
------- 178 0,-1
---UCAGUGCAUGACAGAACUUGa------
------- 4 0,-1
---UCAGUGCAUGACAGAACUUGu------
------- 6 0,-1
---UCAGUGCAUGACAGAACUUGuu-----
------- 3 0,-1
---UCAGUGCAUGACAGAACUUGG------
------- 1233 0,0
---UCAGUGCAUGACAGAACUUGGa-----
------- 254 0,0

--UCAGUGCAUGACAGAACUUGGaa----
------- 60 0,0
--UCAGUGCAUGACAGAACUUGGau----
------- 2 0,0
--UCAGUGCAUGACAGAACUUGGu-----
------- 859 0,0
--UCAGUGCAUGACAGAACUUGGuu----
------- 101 0,0
--UCAGUGCAUGACAGAACUUGGG-----
------- 947 0,1
--UCAGUGCAUGACAGAACUUGGGa----
------- 378 0,1
--UCAGUGCAUGACAGAACUUGGGaa---
------- 58 0,1
--UCAGUGCAUGACAGAACUUGGGau---
------- 6 0,1
--UCAGUGCAUGACAGAACUUGGGu----
------- 711 0,1
--UCAGUGCAUGACAGAACUUGGGuu---
------- 54 0,1
--UCAGUGCAUGACAGAACUUGGGC----
------- 2 0,2
--AGUGCAUGACAGAACUUG-------
------- 7 2,-1
--AGUGCAUGACAGAACUUGG------
------- 16 2,0
--AGUGCAUGACAGAACUUGGa-----
------- 3 2,0
--AGUGCAUGACAGAACUUGGu-----
------- 14 2,0
--AGUGCAUGACAGAACUUGGuu----
------- 3 2,0
--AGUGCAUGACAGAACUUGGG-----
------- 17 2,1
--AGUGCAUGACAGAACUUGGGa----
------- 6 2,1
--AGUGCAUGACAGAACUUGGGu----
------- 6 2,1

>hsa-mir-154 MA:15-36;mi:51-72 SRX018957
gugguacuugaagaUAGGUUAUCCGUGUUGCCUUCGcuuuauuugugacgAAUCAUACACGGUUGACCUAUUuuucagua
ccaa
.(((((((.((((((((((((.((((((....(((((.........).))))....)))))).)))))))))))).))))
))).
--------------UAGGUUAUCCGUGUUGCCUUCG--
---- 8 0,0
--AAUCAUACACGGUUGACCUAUU--------
---- 15 0,0

>hsa-mir-155 MA:4-26;mi:43-64 SRX018957
cugUUAAUGCUAAUCGUGAUAGGGGUuuuugccuccaacugaCUCCUACAUAUUAGCAUUAACAg
((((((((((((((.(((.((((((((.(((....)))..)))))))))))))))))))))))))
---UUAAUGCUAAUCGUGAUA-- 5 0,-5
---UUAAUGCUAAUCGUGAUAGG-- 4 0,-3
---UUAAUGCUAAUCGUGAUAGGG--- 7 0,-2
---UUAAUGCUAAUCGUGAUAGGGG-- 7 0,-1
---UUAAUGCUAAUCGUGAUAGGGGU--------------------------------------- 6 0,0

>hsa-mir-15a MA:14-35;mi:51-72 SRX018957
ccuuggaguaaagUAGCAGCACAUAAUGGUUUGUGgauuuugaaaaggugCAGGCCAUAUUGUGCUGCCUCAaaaauaca
agg
(((((.......(..((((((((..((((((((((..((.....))..))))))))))..))))))))..).......))
)))
-------------UAGCAGCACAUAAUGGUU---
--- 11 0,-4
-------------UAGCAGCACAUAAUGGUUa--
--- 2 0,-4
-------------UAGCAGCACAUAAUGGUUU--
--- 135 0,-3
-------------UAGCAGCACAUAAUGGUUUa---
--- 2 0,-3
-------------UAGCAGCACAUAAUGGUUUG---
--- 190 0,-2
-------------UAGCAGCACAUAAUGGUUUGU--
--- 129 0,-1
-------------UAGCAGCACAUAAUGGUUUGUa---

--- 6   0,-1
-------------UAGCAGCACAUAAUGGUUUGUaa---
--- 2   0,-1
-------------UAGCAGCACAUAAUGGUUUGUu--
--- 2   0,-1
-------------UAGCAGCACAUAAUGGUUUGUG--
--- 18 0,0
-------------UAGCAGCACAUAAUGGUUUGUGa---
--- 4   0,0
-------------UAGCAGCACAUAAUGGUUUGUGu---
--- 2   0,0

>hsa-mir-15b   MA:20-41;mi:58-79   SRX018957
uugaggccuuaaaguacugUAGCAGCACAUCAUGGUUUACAugcuacagucaagaugCGAAUCAUUAUUUGCUGCUCUAg
aaauuuaaggaaauucau
.((((.(((((((...(((.(((((((.((.((((((..(((.((.......)))))..)))))).)).))))))).)))
...)))))))...)))).
-------------------UAGCAGCACAUCAUGGUU--
----------------- 4   0,-4
-------------------UAGCAGCACAUCAUGGUUU---
----------------- 37 0,-3
-------------------UAGCAGCACAUCAUGGUUUu--
----------------- 3   0,-3
-------------------UAGCAGCACAUCAUGGUUUA--
----------------- 108 0,-2
-------------------UAGCAGCACAUCAUGGUUUAa---------------------------------------
----------------- 3   0,-2
-------------------UAGCAGCACAUCAUGGUUUAu---------------------------------------
----------------- 6   0,-2
-------------------UAGCAGCACAUCAUGGUUUAC---------------------------------------
----------------- 42 0,-1
-------------------UAGCAGCACAUCAUGGUUUACA--------------------------------------
----------------- 154 0,0
-------------------UAGCAGCACAUCAUGGUUUACAa-------------------------------------
----------------- 5   0,0
--------------------AGCAGCACAUCAUGGUUUACA--------------------------------------
----------------- 2   1,0

>hsa-mir-16-1 MA:14-35;mi:56-77 SRX018957
gucagcagugccuUAGCAGCACGUAAAUAUUGGCGuuaagauucuaaaauuaucuCCAGUAUUAACUGUGCUGCUGAagu
aagguugac
((((((..(((.((((((((((((.((((((((.(.(((.........))).).)))))))).)).)))))))))).))
)..))))))
-------------UAGCAGCACGUAAAUAUU---
--------- 30 0,-4
-------------UAGCAGCACGUAAAUAUUG--
--------- 123 0,-3
-------------UAGCAGCACGUAAAUAUUGa---
--------- 2 0,-3
-------------UAGCAGCACGUAAAUAUUGG---
--------- 252 0,-2
-------------UAGCAGCACGUAAAUAUUGGa--
--------- 8 0,-2
-------------UAGCAGCACGUAAAUAUUGGaa---
--------- 2 0,-2
-------------UAGCAGCACGUAAAUAUUGGu--
--------- 2 0,-2
-------------UAGCAGCACGUAAAUAUUGGC--
--------- 630 0,-1
-------------UAGCAGCACGUAAAUAUUGGCa---
--------- 81 0,-1
-------------UAGCAGCACGUAAAUAUUGGCaa--
--------- 86 0,-1
-------------UAGCAGCACGUAAAUAUUGGCau--
--------- 41 0,-1
-------------UAGCAGCACGUAAAUAUUGGCu---
--------- 5 0,-1
-------------UAGCAGCACGUAAAUAUUGGCG---
--------- 1349 0,0
-------------UAGCAGCACGUAAAUAUUGGCGa--
--------- 283 0,0
-------------UAGCAGCACGUAAAUAUUGGCGaa---
--------- 7 0,0
-------------UAGCAGCACGUAAAUAUUGGCGau---
--------- 14 0,0
-------------UAGCAGCACGUAAAUAUUGGCGU--

---------   244 0,1
-------------UAGCAGCACGUAAAUAUUGGCGUU---
---------   17 0,2
--------------AGCAGCACGUAAAUAUUG--
---------   2   1,-3
--------------AGCAGCACGUAAAUAUUGG---
---------   2   1,-2
--------------AGCAGCACGUAAAUAUUGGC--
---------   6   1,-1
--------------AGCAGCACGUAAAUAUUGGCG---
---------   7   1,0
--------------AGCAGCACGUAAAUAUUGGCGa--
---------   3   1,0
---------------GCAGCACGUAAAUAUUGGCG---
---------   4   2,0

>hsa-mir-16-2   MA:10-31;mi:53-74   SRX018957
guuccacucUAGCAGCACGUAAAUAUUGGCGuagugaaauauauauuaaacaCCAAUAUUACUGUGCUGCUUUAguguga
c
((..((((..(((((((((..((((((((.((..(((........))).)).))))))))..)))))))))..))))..)
)
---------UAGCAGCACGUAAAUAUU---
-   30 0,-4
---------UAGCAGCACGUAAAUAUUG--
-   123 0,-3
---------UAGCAGCACGUAAAUAUUGa---
-   2   0,-3
---------UAGCAGCACGUAAAUAUUGG---
-   252 0,-2
---------UAGCAGCACGUAAAUAUUGGa--
-   8   0,-2
---------UAGCAGCACGUAAAUAUUGGaa---
-   2   0,-2
---------UAGCAGCACGUAAAUAUUGGu--
-   2   0,-2
---------UAGCAGCACGUAAAUAUUGGC--
-   630 0,-1
---------UAGCAGCACGUAAAUAUUGGCa---

- 81 0,-1

---------UAGCAGCACGUAAAUAUUGGCaa--

- 86 0,-1

---------UAGCAGCACGUAAAUAUUGGCau--

- 41 0,-1

---------UAGCAGCACGUAAAUAUUGGCu---

- 5 0,-1

---------UAGCAGCACGUAAAUAUUGGCG---

- 1349 0,0

---------UAGCAGCACGUAAAUAUUGGCGa--

- 283 0,0

---------UAGCAGCACGUAAAUAUUGGCGaa---

- 7 0,0

---------UAGCAGCACGUAAAUAUUGGCGau---

- 14 0,0

---------UAGCAGCACGUAAAUAUUGGCGU--

- 244 0,1

---------UAGCAGCACGUAAAUAUUGGCGUA---

- 5 0,2

---------UAGCAGCACGUAAAUAUUGGCGUAa--

- 2 0,2

----------AGCAGCACGUAAAUAUUG---

- 2 1,-3

----------AGCAGCACGUAAAUAUUGG--

- 2 1,-2

----------AGCAGCACGUAAAUAUUGGC---

- 6 1,-1

----------AGCAGCACGUAAAUAUUGGCG--

- 7 1,0

----------AGCAGCACGUAAAUAUUGGCGa---

- 3 1,0

-----------GCAGCACGUAAAUAUUGGCG--

- 4 2,0

---ACCAAUAUUACUGUGCUG-----------

- 2 -1,-5

---ACCAAUAUUACUGUGCUGCU---------

- 2 -1,-3

---ACCAAUAUUACUGUGCUGCUUU-------

- 8 -1,-1
---ACCAAUAUUACUGUGCUGCUUUA------
- 4 -1,0
--CCAAUAUUACUGUGCUGCUUUA------
- 3 0,0
--CCAAUAUUACUGUGCUGCUUUAa-----
- 2 0,0

>hsa-mir-17 MA:14-36;mi:51-72 SRX018957
gucagaauaauguCAAAGUGCUUACAGUGCAGGUAGugauaugugcaucuACUGCAGUGAAGGCACUUGUAGcauuaugg
ugac
((((..(((((((..((((((((.((.(((((.(((((.......)).)))))))).)).))))))))...)))))))..
))))
-------------CAAAGUGCUUACAGUGCA---
---- 4 0,-5
-------------CAAAGUGCUUACAGUGCAG--
---- 23 0,-4
-------------CAAAGUGCUUACAGUGCAGG---
---- 35 0,-3
-------------CAAAGUGCUUACAGUGCAGGa--
---- 2 0,-3
-------------CAAAGUGCUUACAGUGCAGGU--
---- 45 0,-2
-------------CAAAGUGCUUACAGUGCAGGUA---
---- 68 0,-1
-------------CAAAGUGCUUACAGUGCAGGUAa--
---- 22 0,-1
-------------CAAAGUGCUUACAGUGCAGGUAaa---
---- 2 0,-1
-------------CAAAGUGCUUACAGUGCAGGUAu--
---- 33 0,-1
-------------CAAAGUGCUUACAGUGCAGGUAG--
---- 136 0,0
-------------CAAAGUGCUUACAGUGCAGGUAGU---
---- 13 0,1
--------------AAAGUGCUUACAGUGCAGGU--
---- 4 1,-2
--------------AAAGUGCUUACAGUGCAGGUAu--

---- 2 1,-1

--------------AAAGUGCUUACAGUGCAGGUAG--
---- 3 1,0

--ACUGCAGUGAAGGCACUU------------
---- 13 0,-4

--ACUGCAGUGAAGGCACUUG-----------
---- 75 0,-3

--ACUGCAGUGAAGGCACUUGU----------
---- 109 0,-2

--ACUGCAGUGAAGGCACUUGUA---------
---- 63 0,-1

--ACUGCAGUGAAGGCACUUGUAa--------
---- 4 0,-1

--ACUGCAGUGAAGGCACUUGUAG--------
---- 10 0,0

--ACUGCAGUGAAGGCACUUGUAGC-------
---- 4 0,1

--ACUGCAGUGAAGGCACUUGUAGCu------
---- 12 0,1

--ACUGCAGUGAAGGCACUUGUAGCA------
---- 8 0,2

---CUGCAGUGAAGGCACUUGUA---------
---- 6 1,-1

---CUGCAGUGAAGGCACUUGUAGCu------
---- 2 1,1

---CUGCAGUGAAGGCACUUGUAGCA------
---- 2 1,2

--UGCAGUGAAGGCACUUGU----------
---- 3 2,-2

--UGCAGUGAAGGCACUUGUAG--------
---- 2 2,0

>hsa-mir-181a-1 MA:24-46;mi:64-85 SRX018957

ugaguuuugagguugcuucagugAACAUUCAACGCUGUCGGUGAGUuuggaauuaaaaucaaaACCAUCGACCGUUGAUU
GUACCcuauggcuaaccaucaucuacucca

.((((..((((((((((..((.(.(((.((((((..(((((((.((((.((.......)).))))))))))))))))).)
)).).))..))).)))).)))...))))..

-----------------------AACAUUCAACGCUGUCGG---------------------------------------

----------------------------- 9   0,-5
-----------------------AACAUUCAACGCUGUCGGU------------------------------------
----------------------------- 3   0,-4
-----------------------AACAUUCAACGCUGUCGGUaa----------------------------------
----------------------------- 5   0,-4
-----------------------AACAUUCAACGCUGUCGGUau----------------------------------
----------------------------- 3   0,-4
-----------------------AACAUUCAACGCUGUCGGUG-----------------------------------
----------------------------- 21   0,-3
-----------------------AACAUUCAACGCUGUCGGUGu----------------------------------
----------------------------- 6   0,-3
-----------------------AACAUUCAACGCUGUCGGUGA----------------------------------
----------------------------- 143 0,-2
-----------------------AACAUUCAACGCUGUCGGUGAa---------------------------------
----------------------------- 2   0,-2
-----------------------AACAUUCAACGCUGUCGGUGAaa--------------------------------
----------------------------- 2   0,-2
-----------------------AACAUUCAACGCUGUCGGUGAG---------------------------------
----------------------------- 547 0,-1
-----------------------AACAUUCAACGCUGUCGGUGAGa--------------------------------
----------------------------- 18   0,-1
-----------------------AACAUUCAACGCUGUCGGUGAGU--------------------------------
----------------------------- 118 0,0
-----------------------AACAUUCAACGCUGUCGGUGAGUa-------------------------------
----------------------------- 10   0,0
-----------------------AACAUUCAACGCUGUCGGUGAGUU-------------------------------
----------------------------- 145 0,1
-----------------------AACAUUCAACGCUGUCGGUGAGUUa------------------------------
----------------------------- 6   0,1
-----------------------AACAUUCAACGCUGUCGGUGAGUUU------------------------------
----------------------------- 30   0,2
------------------------ACAUUCAACGCUGUCGGUGAGUU-------------------------------
----------------------------- 3   1,1
---ACCAUCGACCGUUGAUU
G---------------------------- 8   0,-4
---ACCAUCGACCGUUGAUU
GU--------------------------- 4   0,-3
---ACCAUCGACCGUUGAUU

GUAC-------------------------- 4   0,-1
---ACCAUCGACCGUUGAUU
GUACC------------------------- 4   0,0

>hsa-mir-181a-2 MA:39-61;mi:77-98   SRX018957
agaagggcuaucaggccagccuucagaggacuccaaggAACAUUCAACGCUGUCGGUGAGUuugggauuugaaaaaACCA
CUGACCGUUGACUGUACCuuggggucccuua
.........................(((((((((((((.(((.((((((..(((((((.((((...........))))))
))))))))))).))).))))))))))))).
--------------------------------------AACAUUCAACGCUGUCGG------------------------
------------------------------ 9   0,-5
--------------------------------------AACAUUCAACGCUGUCGGU-----------------------
------------------------------ 3   0,-4
--------------------------------------AACAUUCAACGCUGUCGGUaa---------------------
------------------------------ 5   0,-4
--------------------------------------AACAUUCAACGCUGUCGGUau---------------------
------------------------------ 3   0,-4
--------------------------------------AACAUUCAACGCUGUCGGUG----------------------
------------------------------ 21 0,-3
--------------------------------------AACAUUCAACGCUGUCGGUGu---------------------
------------------------------ 6   0,-3
--------------------------------------AACAUUCAACGCUGUCGGUGA---------------------
------------------------------ 143 0,-2
--------------------------------------AACAUUCAACGCUGUCGGUGAa--------------------
------------------------------ 2   0,-2
--------------------------------------AACAUUCAACGCUGUCGGUGAaa-------------------
------------------------------ 2   0,-2
--------------------------------------AACAUUCAACGCUGUCGGUGAG--------------------
------------------------------ 547 0,-1
--------------------------------------AACAUUCAACGCUGUCGGUGAGa-------------------
------------------------------ 18 0,-1
--------------------------------------AACAUUCAACGCUGUCGGUGAGU-------------------
------------------------------ 118 0,0
--------------------------------------AACAUUCAACGCUGUCGGUGAGUa------------------
------------------------------ 10 0,0
--------------------------------------AACAUUCAACGCUGUCGGUGAGUU------------------
------------------------------ 145 0,1
--------------------------------------AACAUUCAACGCUGUCGGUGAGUUa-----------------

------------------------------ 6   0,1
--------------------------------------AACAUUCAACGCUGUCGGUGAGUUU-----------------
------------------------------ 30 0,2
---------------------------------------ACAUUCAACGCUGUCGGUGAGUU------------------
------------------------------ 3   1,1
--ACCA
CUGACCGUUGACUG---------------- 14 0,-4
--ACCA
CUGACCGUUGACUGU--------------- 3   0,-3
--ACCA
CUGACCGUUGACUGUAC------------- 2   0,-1
--ACCA
CUGACCGUUGACUGUACC------------ 9   0,0

>hsa-mir-181b-1 5p:36-58   SRX018957
ccugugcagagauuauuuuuuaaaaggucacaaucAACAUUCAUUGCUGUCGGUGGGUugaacuguguggacaagcucac
ugaacaaugaaugcaacuguggccccgcuu
........................(((((((......(((((((((...((((((((((...((....))...)))))))
))).)))))))))....)))))))......
-----------------------------------AACAUUCAUUGCUGUCGGU--------------------------
------------------------------ 5   0,-4
-----------------------------------AACAUUCAUUGCUGUCGGUG-------------------------
------------------------------ 42 0,-3
-----------------------------------AACAUUCAUUGCUGUCGGUGu------------------------
------------------------------ 3   0,-3
-----------------------------------AACAUUCAUUGCUGUCGGUGG------------------------
------------------------------ 27 0,-2
-----------------------------------AACAUUCAUUGCUGUCGGUGGa-----------------------
------------------------------ 2   0,-2
-----------------------------------AACAUUCAUUGCUGUCGGUGGu-----------------------
------------------------------ 4   0,-2
-----------------------------------AACAUUCAUUGCUGUCGGUGGG-----------------------
------------------------------ 94 0,-1
-----------------------------------AACAUUCAUUGCUGUCGGUGGGa----------------------
------------------------------ 6   0,-1
-----------------------------------AACAUUCAUUGCUGUCGGUGGGU----------------------
------------------------------ 20 0,0
-----------------------------------AACAUUCAUUGCUGUCGGUGGGUa---------------------

------------------------------ 17   0,0
-----------------------------------AACAUUCAUUGCUGUCGGUGGGUaa--------------------
------------------------------ 10   0,0
-----------------------------------AACAUUCAUUGCUGUCGGUGGGUau--------------------
------------------------------ 52   0,0
-----------------------------------AACAUUCAUUGCUGUCGGUGGGUU---------------------
------------------------------ 61   0,1
-----------------------------------AACAUUCAUUGCUGUCGGUGGGUUa--------------------
------------------------------ 6   0,1
-----------------------------------AACAUUCAUUGCUGUCGGUGGGUUG--------------------
------------------------------ 2   0,2
---CUCAC
UGAACAAUGAAUGCAA-------------- 2

>hsa-mir-181b-2 5p:16-38   SRX018957
cugauggcugcacucAACAUUCAUUGCUGUCGGUGGGUuugagucugaaucaacucacugaucaaugaaugcaaacugcg
gaccaaaca
....(((((((.....((((((((..((((((((((.((.......))))))))))))))))))))))))).....))))
).)))....
---------------AACAUUCAUUGCUGUCGGU--
---------   5   0,-4
---------------AACAUUCAUUGCUGUCGGUG---
---------   42   0,-3
---------------AACAUUCAUUGCUGUCGGUGu--
---------   3   0,-3
---------------AACAUUCAUUGCUGUCGGUGG--
---------   27   0,-2
---------------AACAUUCAUUGCUGUCGGUGGa---
---------   2   0,-2
---------------AACAUUCAUUGCUGUCGGUGGu---
---------   4   0,-2
---------------AACAUUCAUUGCUGUCGGUGGG---
---------   94   0,-1
---------------AACAUUCAUUGCUGUCGGUGGGa--
---------   6   0,-1
---------------AACAUUCAUUGCUGUCGGUGGGU--
---------   20   0,0
---------------AACAUUCAUUGCUGUCGGUGGGUa---

---------   17   0,0
---------------AACAUUCAUUGCUGUCGGUGGGUaa--
---------   10   0,0
---------------AACAUUCAUUGCUGUCGGUGGGUau--
---------   52   0,0
---------------AACAUUCAUUGCUGUCGGUGGGUU---
---------   61   0,1
---------------AACAUUCAUUGCUGUCGGUGGGUUa--
---------   6   0,1
---------------AACAUUCAUUGCUGUCGGUGGGUUU--
---------   9   0,2
--ACUCACUGAUCAAUGAAUGCAA------
---------   2

>hsa-mir-181c   MA:27-48;mi:65-86   SRX018957
cggaaaauuugccaaggguuugggggAACAUUCAACCUGUCGGUGAGUuugggcagcucaggcaAACCAUCGACCGUUGA
GUGGACccugaggccuggaauugccauccu
.(((.......(((.((.((..(((...((((((((..(((((((.(((((...........))))))))))))).)))))
)))..)))..)).)))))........))).
--------------------------AACAUUCAACCUGUCGGUGAG---------------------------------
------------------------------   4   0,-1
--------------------------AACAUUCAACCUGUCGGUGAGU--------------------------------
------------------------------   2   0,0
--------------------------AACAUUCAACCUGUCGGUGAGUU-------------------------------
------------------------------   5   0,1
---ACCAUCGACCGUUGA
GUG---------------------------   17   1,-3
---ACCAUCGACCGUUGA
GUGG--------------------------   5   1,-2

>hsa-mir-181d   5p:36-58   SRX018957
gucccucccccuaggccacagccgaggucacaaucAACAUUCAUUGUUGUCGGUGGGUugugaggacugaggccagaccc
accgggggaugaaugucacuguggcugggccagacacggcuuaaggggaaugggac
(((((((((((((((((...(((..(((((((....(((((((((.(..((((((((((....((.(....))).)))))
)))))).)))))))))...))))))).)))......)))).))))))..))))))
-----------------------------------AACAUUCAUUGUUGUCGGUG-------------------------
--   8   0,-3
-----------------------------------AACAUUCAUUGUUGUCGGUGG------------------------

--- 12 0,-2
-----------------------------------AACAUUCAUUGUUGUCGGUGGG-----------------------
--- 16 0,-1
-----------------------------------AACAUUCAUUGUUGUCGGUGGGa----------------------
--- 5 0,-1
-----------------------------------AACAUUCAUUGUUGUCGGUGGGU----------------------
--- 6 0,0
-----------------------------------AACAUUCAUUGUUGUCGGUGGGUa---------------------
--- 8 0,0
-----------------------------------AACAUUCAUUGUUGUCGGUGGGUU---------------------
--- 8 0,1
-----------------------------------AACAUUCAUUGUUGUCGGUGGGUUa--------------------
--- 3 0,1
-----------------------------------AACAUUCAUUGUUGUCGGUGGGUUu--------------------
--- 3 0,1

>hsa-mir-182 MA:23-46;mi:67-87 SRX018957
gagcugcuugccuccccccguuUUUGGCAAUGGUAGAACUCACACUggugagguaacaggauccggUGGUUCUAGACUUG
CCAACUAuggggcgaggacucagccggcac
..((((((..((((.((((((..(((((((.(.((((((...((((((.............)))))))))))))..))))
))))..)))))).))))....)).))))..
----------------------UUUGGCAAUGGUAGAACUCACACU----------------------------------
------------------------------ 2 0,0

>hsa-mir-185 MA:15-36;mi:50-71 SRX018957
agggggcgagggauUGGAGAGAAAGGCAGUUCCUGAugguccccuccccAGGGGCUGGCUUUCCUCUGGUCcuucccucc
ca
.((((.(((((((..(((.(((((.(((((((((..((......)).)))))))))).))))))))..))))))).))))
).
-------------UUGGAGAGAAAGGCAGUUCCUG---
-- 3 -1,-1
--------------UGGAGAGAAAGGCAGUUC--
-- 101 0,-4
--------------UGGAGAGAAAGGCAGUUCa---
-- 2 0,-4
--------------UGGAGAGAAAGGCAGUUCC---
-- 98 0,-3
--------------UGGAGAGAAAGGCAGUUCCU--

-- 1220 0,-2

--------------UGGAGAGAAAGGCAGUUCCUaa--

-- 3 0,-2

--------------UGGAGAGAAAGGCAGUUCCUG---

-- 1246 0,-1

--------------UGGAGAGAAAGGCAGUUCCUGu--

-- 92 0,-1

--------------UGGAGAGAAAGGCAGUUCCUGuu---

-- 33 0,-1

--------------UGGAGAGAAAGGCAGUUCCUGA--

-- 6509 0,0

--------------UGGAGAGAAAGGCAGUUCCUGAa---

-- 1651 0,0

--------------UGGAGAGAAAGGCAGUUCCUGAaa--

-- 67 0,0

--------------UGGAGAGAAAGGCAGUUCCUGAau--

-- 110 0,0

--------------UGGAGAGAAAGGCAGUUCCUGAU---

-- 2039 0,1

--------------UGGAGAGAAAGGCAGUUCCUGAUa--

-- 154 0,1

--------------UGGAGAGAAAGGCAGUUCCUGAUaa---

-- 4 0,1

--------------UGGAGAGAAAGGCAGUUCCUGAUau---

-- 11 0,1

--------------UGGAGAGAAAGGCAGUUCCUGAUu--

-- 217 0,1

--------------UGGAGAGAAAGGCAGUUCCUGAUuu---

-- 11 0,1

--------------UGGAGAGAAAGGCAGUUCCUGAUG--

-- 13 0,2

---------------GGAGAGAAAGGCAGUUCCU--

-- 3 1,-2

---------------GGAGAGAAAGGCAGUUCCUG---

-- 9 1,-1

---------------GGAGAGAAAGGCAGUUCCUGA--

-- 29 1,0

---------------GGAGAGAAAGGCAGUUCCUGAa---

-- 3 1,0
---------------GGAGAGAAAGGCAGUUCCUGAaa--
-- 2 1,0
---------------GGAGAGAAAGGCAGUUCCUGAau--
-- 2 1,0
---------------GGAGAGAAAGGCAGUUCCUGAU---
-- 10 1,1
--CAGGGGCUGGCUUUCCUC--------------
-- 25 -1,-5
--CAGGGGCUGGCUUUCCUCU-------------
-- 9 -1,-4
--CAGGGGCUGGCUUUCCUCUGGU----------
-- 2 -1,-1
---AGGGGCUGGCUUUCCUCU-------------
-- 90 0,-4
---AGGGGCUGGCUUUCCUCUGG-----------
-- 3 0,-2
---AGGGGCUGGCUUUCCUCUGGU----------
-- 3 0,-1
---AGGGGCUGGCUUUCCUCUGGUa---------
-- 4 0,-1
---AGGGGCUGGCUUUCCUCUGGUaa--------
-- 6 0,-1
---AGGGGCUGGCUUUCCUCUGGUau--------
-- 3 0,-1
---AGGGGCUGGCUUUCCUCUGGUu---------
-- 2 0,-1

>hsa-mir-186 MA:15-36;mi:54-75 SRX018957
ugcuuguaacuuucCAAAGAAUUCUCCUUUUGGGCUuucugguuuuauuuuaaGCCCAAAGGUGAAUUUUUUGGGaaguu
ugagcu
.((((.((((((((((((((((.(((((.((((((.............))))))))))).))))))))))))))))
))))).
-------------CCAAAGAAUUCUCCUUUUGGGa---
------ 2 -1,-2
-------------CCAAAGAAUUCUCCUUUUGGGCa--
------ 3 -1,-1
-------------CCAAAGAAUUCUCCUUUUGGGCU--

------ 3 -1,0

-------------CCAAAGAAUUCUCCUUUUGGGCUU--

------ 3 -1,1

--------------CAAAGAAUUCUCCUUUUG--

------ 6 0,-4

--------------CAAAGAAUUCUCCUUUUGG---

------ 2 0,-3

--------------CAAAGAAUUCUCCUUUUGGG--

------ 15 0,-2

--------------CAAAGAAUUCUCCUUUUGGGC---

------ 17 0,-1

--------------CAAAGAAUUCUCCUUUUGGGCa--

------ 76 0,-1

--------------CAAAGAAUUCUCCUUUUGGGCaa---

------ 26 0,-1

--------------CAAAGAAUUCUCCUUUUGGGCau---

------ 102 0,-1

--------------CAAAGAAUUCUCCUUUUGGGCU--

------ 143 0,0

--------------CAAAGAAUUCUCCUUUUGGGCUa---

------ 48 0,0

--------------CAAAGAAUUCUCCUUUUGGGCUaa--

------ 5 0,0

--------------CAAAGAAUUCUCCUUUUGGGCUau--

------ 11 0,0

--------------CAAAGAAUUCUCCUUUUGGGCUU---

------ 338 0,1

--------------CAAAGAAUUCUCCUUUUGGGCUUa--

------ 18 0,1

--------------CAAAGAAUUCUCCUUUUGGGCUUaa---

------ 2 0,1

--------------CAAAGAAUUCUCCUUUUGGGCUUU--

------ 16 0,2

---------------AAAGAAUUCUCCUUUUGGGCa--

------ 3 1,-1

---------------AAAGAAUUCUCCUUUUGGGCau---

------ 2 1,-1

---------------AAAGAAUUCUCCUUUUGGGCU--

------ 17 1,0
---------------AAAGAAUUCUCCUUUUGGGCUa--
------ 4 1,0
---------------AAAGAAUUCUCCUUUUGGGCUau---
------ 2 1,0
---------------AAAGAAUUCUCCUUUUGGGCUU--
------ 35 1,1

>hsa-mir-187 mi:35-56;MA:71-92 SRX018957
ggucgggcucaccaugacacagugugagaccucgGGCUACAACACAGGACCCGGGCgcugcucugaccccUCGUGUCUUG
UGUUGCAGCCGGagggacgcagguccgca
.(.((((((............((((...((((.((((.((((((((((.((((.((......).).)).)).))))))
))))).)))).))))..))))))))))).
--UCGUGUCUUG
UGUUGCAGCCGG----------------- 12 0,0
--UCGUGUCUUG
UGUUGCAGCCGGu---------------- 11 0,0
--UCGUGUCUUG
UGUUGCAGCCGGuu--------------- 9 0,0

>hsa-mir-18a MA:6-28;mi:47-69 SRX018957
uguucUAAGGUGCAUCUAGUGCAGAUAGugaaguagauuagcaucuACUGCCCUAAGUGCUCCUUCUGGca
.(((..((((.(((..(((.((((.(((((..(.....)..)).))))))).)))..))).))))..))).
-----UAAGGUGCAUCUAGUGCAGA--- 3 0,-3
-----UAAGGUGCAUCUAGUGCAGAU-- 12 0,-2

>hsa-mir-1908 5p:12-32 SRX018957
cgggaaugccgCGGCGGGGACGGCGAUUGGUCcguaugugguggugccaccggccgccggcuccgccccggcccccgcccc
((((...((((.(((((((.(((((..((((..((((.....)))).))))..))))).))))))).)))).))))....
-----------CGGCGGGGACGGCGAUUGGU---
2 0,-1

>hsa-mir-190b 5p:11-31 SRX018957
ugcuucugugUGAUAUGUUUGAUAUUGGGUUguuuaauuaggaaccaacuaaaugucaaacauauucuuacagcagcag
((((.(((((.((((((((((((((((.((((((((......))).))))).))))))))))))))))..))))).)))).
----------UGAUAUGUUUGAUAUUGGGUUGa---
2 0,1
----------UGAUAUGUUUGAUAUUGGGUUGU---

3	0,2

>hsa-mir-191	MA:16-38;mi:58-79	SRX018957
cggcuggacagcgggCAACGGAAUCCCAAAAGCAGCUGuugucuccagagcauuccaGCUGCGCUUGGAUUUCGUCCCCugcucuccugccu
.(((.(((.((((((..((((((((.((..(((((((..((((....)).))..)))))))..))))))))))..))))))).))).))).
---------------CAACGGAAUCCCAAAAGC---	54	0,-5
---------------CAACGGAAUCCCAAAAGCA--	461	0,-4
---------------CAACGGAAUCCCAAAAGCAa---	9	0,-4
---------------CAACGGAAUCCCAAAAGCAG---	1568	0,-3
---------------CAACGGAAUCCCAAAAGCAGa--	13	0,-3
---------------CAACGGAAUCCCAAAAGCAGau---	21	0,-3
---------------CAACGGAAUCCCAAAAGCAGu--	10	0,-3
---------------CAACGGAAUCCCAAAAGCAGuu---	10	0,-3
---------------CAACGGAAUCCCAAAAGCAGC--	5052	0,-2
---------------CAACGGAAUCCCAAAAGCAGCa---	126	0,-2
---------------CAACGGAAUCCCAAAAGCAGCaa--	13	0,-2
---------------CAACGGAAUCCCAAAAGCAGCau--	8	0,-2
---------------CAACGGAAUCCCAAAAGCAGCU---	13182	0,-1
---------------CAACGGAAUCCCAAAAGCAGCUa--	263	0,-1
---------------CAACGGAAUCCCAAAAGCAGCUaa---	26	0,-1
---------------CAACGGAAUCCCAAAAGCAGCUau---

----------- 90 0,-1
---------------CAACGGAAUCCCAAAAGCAGCUu--
----------- 301 0,-1
---------------CAACGGAAUCCCAAAAGCAGCUuu---
----------- 142 0,-1
---------------CAACGGAAUCCCAAAAGCAGCUG--
----------- 5564 0,0
---------------CAACGGAAUCCCAAAAGCAGCUGa---
----------- 199 0,0
---------------CAACGGAAUCCCAAAAGCAGCUGaa--
----------- 23 0,0
---------------CAACGGAAUCCCAAAAGCAGCUGau--
----------- 6 0,0
---------------CAACGGAAUCCCAAAAGCAGCUGU---
----------- 2012 0,1
---------------CAACGGAAUCCCAAAAGCAGCUGUa--
----------- 28 0,1
---------------CAACGGAAUCCCAAAAGCAGCUGUU--
----------- 141 0,2
----------------AACGGAAUCCCAAAAGCA--
----------- 31 1,-4
----------------AACGGAAUCCCAAAAGCAG---
----------- 118 1,-3
----------------AACGGAAUCCCAAAAGCAGa--
----------- 2 1,-3
----------------AACGGAAUCCCAAAAGCAGu--
----------- 2 1,-3
----------------AACGGAAUCCCAAAAGCAGC--
----------- 537 1,-2
----------------AACGGAAUCCCAAAAGCAGCU---
----------- 1682 1,-1
----------------AACGGAAUCCCAAAAGCAGCUa--
----------- 13 1,-1
----------------AACGGAAUCCCAAAAGCAGCUaa---
----------- 2 1,-1
----------------AACGGAAUCCCAAAAGCAGCUau---
----------- 13 1,-1
----------------AACGGAAUCCCAAAAGCAGCUu--

-----------   12   1,-1
---------------AACGGAAUCCCAAAAGCAGCUuu--------------------------------------
-----------   12   1,-1
---------------AACGGAAUCCCAAAAGCAGCUG---------------------------------------
-----------   694 1,0
---------------AACGGAAUCCCAAAAGCAGCUGa--------------------------------------
-----------   47   1,0
---------------AACGGAAUCCCAAAAGCAGCUGaa-------------------------------------
-----------   7   1,0
---------------AACGGAAUCCCAAAAGCAGCUGau-------------------------------------
-----------   2   1,0
---------------AACGGAAUCCCAAAAGCAGCUGU--------------------------------------
-----------   452 1,1
---------------AACGGAAUCCCAAAAGCAGCUGUa-------------------------------------
-----------   4   1,1
---------------AACGGAAUCCCAAAAGCAGCUGUaa------------------------------------
-----------   4   1,1
---------------AACGGAAUCCCAAAAGCAGCUGUau------------------------------------
-----------   5   1,1
---------------AACGGAAUCCCAAAAGCAGCUGUU-------------------------------------
-----------   19   1,2
---------------AACGGAAUCCCAAAAGCAGCUGUUu------------------------------------
-----------   7   1,2
----------------ACGGAAUCCCAAAAGCAGC--
-----------   6   2,-2
----------------ACGGAAUCCCAAAAGCAGCU---------------------------------------
-----------   12   2,-1
----------------ACGGAAUCCCAAAAGCAGCUG--------------------------------------
-----------   7   2,0
---GCUGCGCUUGGAUUUCGUCCCC-
-----------   2   0,0

>hsa-mir-192   MA:24-44;mi:67-88   SRX018957
gccgagaccgagugcacagggcuCUGACCUAUGAAUUGACAGCCagugcucucgucuccccucuggCUGCCAAUUCCAUA
GGUCACAGguauguucgccucaaugccagc
.........(((.(((((.(.((.((((((((((((((.(((((((................))))))).))))).))))
))))).)).).)))..))))).........
--------------------CUCUGACCUAUGAAUUGACAGC----------------------------------

------------------------------ 2 -2,-1
---------------------CUCUGACCUAUGAAUUGACAGCC---------------------------------------
------------------------------ 4 -2,0
---------------------CUCUGACCUAUGAAUUGACAGCCu--------------------------------------
------------------------------ 3 -2,0
----------------------UCUGACCUAUGAAUUGAC---
------------------------------ 2 -1,-4
----------------------UCUGACCUAUGAAUUGACA--
------------------------------ 2 -1,-3
----------------------UCUGACCUAUGAAUUGACAG---
------------------------------ 26 -1,-2
----------------------UCUGACCUAUGAAUUGACAGa--
------------------------------ 2 -1,-2
----------------------UCUGACCUAUGAAUUGACAGu--
------------------------------ 2 -1,-2
----------------------UCUGACCUAUGAAUUGACAGC--
------------------------------ 255 -1,-1
----------------------UCUGACCUAUGAAUUGACAGCa---------------------------------------
------------------------------ 49 -1,-1
----------------------UCUGACCUAUGAAUUGACAGCaa--------------------------------------
------------------------------ 9 -1,-1
----------------------UCUGACCUAUGAAUUGACAGCau--------------------------------------
------------------------------ 2 -1,-1
----------------------UCUGACCUAUGAAUUGACAGCu---------------------------------------
------------------------------ 3 -1,-1
----------------------UCUGACCUAUGAAUUGACAGCC---------------------------------------
------------------------------ 1403 -1,0
----------------------UCUGACCUAUGAAUUGACAGCCu--------------------------------------
------------------------------ 128 -1,0
----------------------UCUGACCUAUGAAUUGACAGCCuu-------------------------------------
------------------------------ 37 -1,0
----------------------UCUGACCUAUGAAUUGACAGCCA--------------------------------------
------------------------------ 295 -1,1
----------------------UCUGACCUAUGAAUUGACAGCCAa-------------------------------------
------------------------------ 3 -1,1
----------------------UCUGACCUAUGAAUUGACAGCCAau------------------------------------
------------------------------ 3 -1,1
----------------------UCUGACCUAUGAAUUGACAGCCAu-------------------------------------

------------------------------ 83 -1,1

----------------------UCUGACCUAUGAAUUGACAGCCAuu---------------------------------

------------------------------ 30 -1,1

----------------------UCUGACCUAUGAAUUGACAGCCAG----------------------------------

------------------------------ 6 -1,2

----------------------UCUGACCUAUGAAUUGACAGCCAGU---------------------------------

------------------------------ 9 -1,3

-----------------------CUGACCUAUGAAUUGACA---------------------------------------

------------------------------ 985 0,-3

-----------------------CUGACCUAUGAAUUGACAa--------------------------------------

------------------------------ 22 0,-3

-----------------------CUGACCUAUGAAUUGACAaa-------------------------------------

------------------------------ 12 0,-3

-----------------------CUGACCUAUGAAUUGACAau-------------------------------------

------------------------------ 3 0,-3

-----------------------CUGACCUAUGAAUUGACAu--------------------------------------

------------------------------ 4 0,-3

-----------------------CUGACCUAUGAAUUGACAG--------------------------------------

------------------------------ 5445 0,-2

-----------------------CUGACCUAUGAAUUGACAGa-------------------------------------

------------------------------ 262 0,-2

-----------------------CUGACCUAUGAAUUGACAGaa------------------------------------

------------------------------ 53 0,-2

-----------------------CUGACCUAUGAAUUGACAGau------------------------------------

------------------------------ 5 0,-2

-----------------------CUGACCUAUGAAUUGACAGu-------------------------------------

------------------------------ 233 0,-2

-----------------------CUGACCUAUGAAUUGACAGuu------------------------------------

------------------------------ 12 0,-2

-----------------------CUGACCUAUGAAUUGACAGC-------------------------------------

------------------------------ 73381 0,-1

-----------------------CUGACCUAUGAAUUGACAGCa------------------------------------

------------------------------ 16691 0,-1

-----------------------CUGACCUAUGAAUUGACAGCaa-----------------------------------

------------------------------ 2507 0,-1

-----------------------CUGACCUAUGAAUUGACAGCau-----------------------------------

------------------------------ 697 0,-1

-----------------------CUGACCUAUGAAUUGACAGCu------------------------------------

------------------------------ 2508 0,-1

-----------------------CUGACCUAUGAAUUGACAGCuu----------------------------------

------------------------------ 233 0,-1

-----------------------CUGACCUAUGAAUUGACAGCC-----------------------------------

------------------------------ 405899 0,0

-----------------------CUGACCUAUGAAUUGACAGCCu----------------------------------

------------------------------ 27944 0,0

-----------------------CUGACCUAUGAAUUGACAGCCuu---------------------------------

------------------------------ 6884 0,0

-----------------------CUGACCUAUGAAUUGACAGCCA----------------------------------

------------------------------ 228890 0,1

-----------------------CUGACCUAUGAAUUGACAGCCAa---------------------------------

------------------------------ 5284 0,1

-----------------------CUGACCUAUGAAUUGACAGCCAaa--------------------------------

------------------------------ 600 0,1

-----------------------CUGACCUAUGAAUUGACAGCCAau--------------------------------

------------------------------ 2081 0,1

-----------------------CUGACCUAUGAAUUGACAGCCAu---------------------------------

------------------------------ 96343 0,1

-----------------------CUGACCUAUGAAUUGACAGCCAuu--------------------------------

------------------------------ 59485 0,1

-----------------------CUGACCUAUGAAUUGACAGCCAG---------------------------------

------------------------------ 4296 0,2

-----------------------CUGACCUAUGAAUUGACAGCCAGa--------------------------------

------------------------------ 445 0,2

-----------------------CUGACCUAUGAAUUGACAGCCAGaa-------------------------------

------------------------------ 10 0,2

-----------------------CUGACCUAUGAAUUGACAGCCAGau-------------------------------

------------------------------ 7 0,2

-----------------------CUGACCUAUGAAUUGACAGCCAGU--------------------------------

------------------------------ 3748 0,3

-----------------------CUGACCUAUGAAUUGACAGCCAGUa-------------------------------

------------------------------ 168 0,3

-----------------------CUGACCUAUGAAUUGACAGCCAGUu-------------------------------

------------------------------ 683 0,3

-----------------------CUGACCUAUGAAUUGACAGCCAGUG-------------------------------

------------------------------ 12 0,4

------------------------UGACCUAUGAAUUGACAG-------------------------------------

------------------------------ 2000 1,-2
------------------------UGACCUAUGAAUUGACAGu--
------------------------------ 25 1,-2
------------------------UGACCUAUGAAUUGACAGC--
------------------------------ 36682 1,-1
------------------------UGACCUAUGAAUUGACAGCa---------------------------------------
------------------------------ 1069 1,-1
------------------------UGACCUAUGAAUUGACAGCaa--------------------------------------
------------------------------ 274 1,-1
------------------------UGACCUAUGAAUUGACAGCau--------------------------------------
------------------------------ 35 1,-1
------------------------UGACCUAUGAAUUGACAGCu---------------------------------------
------------------------------ 103 1,-1
------------------------UGACCUAUGAAUUGACAGCuu--------------------------------------
------------------------------ 5 1,-1
------------------------UGACCUAUGAAUUGACAGCC---------------------------------------
------------------------------ 25985 1,0
------------------------UGACCUAUGAAUUGACAGCCu--------------------------------------
------------------------------ 799 1,0
------------------------UGACCUAUGAAUUGACAGCCuu-------------------------------------
------------------------------ 183 1,0
------------------------UGACCUAUGAAUUGACAGCCA--------------------------------------
------------------------------ 58022 1,1
------------------------UGACCUAUGAAUUGACAGCCAa-------------------------------------
------------------------------ 1984 1,1
------------------------UGACCUAUGAAUUGACAGCCAaa------------------------------------
------------------------------ 1481 1,1
------------------------UGACCUAUGAAUUGACAGCCAau------------------------------------
------------------------------ 1976 1,1
------------------------UGACCUAUGAAUUGACAGCCAu-------------------------------------
------------------------------ 15108 1,1
------------------------UGACCUAUGAAUUGACAGCCAuu------------------------------------
------------------------------ 12091 1,1
------------------------UGACCUAUGAAUUGACAGCCAG-------------------------------------
------------------------------ 33307 1,2
------------------------UGACCUAUGAAUUGACAGCCAGa------------------------------------
------------------------------ 8063 1,2
------------------------UGACCUAUGAAUUGACAGCCAGaa-----------------------------------

------------------------------ 230 1,2
------------------------UGACCUAUGAAUUGACAGCCAGau--------------------------------
------------------------------ 175 1,2
------------------------UGACCUAUGAAUUGACAGCCAGU---------------------------------
------------------------------ 45239 1,3
------------------------UGACCUAUGAAUUGACAGCCAGUa--------------------------------
------------------------------ 3263 1,3
------------------------UGACCUAUGAAUUGACAGCCAGUaa-------------------------------
------------------------------ 252 1,3
------------------------UGACCUAUGAAUUGACAGCCAGUau-------------------------------
------------------------------ 315 1,3
------------------------UGACCUAUGAAUUGACAGCCAGUu--------------------------------
------------------------------ 6360 1,3
------------------------UGACCUAUGAAUUGACAGCCAGUuu-------------------------------
------------------------------ 764 1,3
------------------------UGACCUAUGAAUUGACAGCCAGUG--------------------------------
------------------------------ 153 1,4
------------------------UGACCUAUGAAUUGACAGCCAGUGa-------------------------------
------------------------------ 20 1,4
------------------------UGACCUAUGAAUUGACAGCCAGUGu-------------------------------
------------------------------ 25 1,4
-------------------------GACCUAUGAAUUGACAGC-------------------------------------
------------------------------ 302 2,-1
-------------------------GACCUAUGAAUUGACAGCa------------------------------------
------------------------------ 26 2,-1
-------------------------GACCUAUGAAUUGACAGCaa-----------------------------------
------------------------------ 6 2,-1
-------------------------GACCUAUGAAUUGACAGCu------------------------------------
------------------------------ 2 2,-1
-------------------------GACCUAUGAAUUGACAGCC------------------------------------
------------------------------ 557 2,0
-------------------------GACCUAUGAAUUGACAGCCu-----------------------------------
------------------------------ 36 2,0
-------------------------GACCUAUGAAUUGACAGCCuu----------------------------------
------------------------------ 10 2,0
-------------------------GACCUAUGAAUUGACAGCCA-----------------------------------
------------------------------ 460 2,1
-------------------------GACCUAUGAAUUGACAGCCAa----------------------------------

----------------------------- 15 2,1
-------------------------GACCUAUGAAUUGACAGCCAaa---------------------------------
----------------------------- 4 2,1
-------------------------GACCUAUGAAUUGACAGCCAau---------------------------------
----------------------------- 11 2,1
-------------------------GACCUAUGAAUUGACAGCCAu----------------------------------
----------------------------- 157 2,1
-------------------------GACCUAUGAAUUGACAGCCAuu---------------------------------
----------------------------- 110 2,1
-------------------------GACCUAUGAAUUGACAGCCAG----------------------------------
----------------------------- 361 2,2
-------------------------GACCUAUGAAUUGACAGCCAGa---------------------------------
----------------------------- 85 2,2
-------------------------GACCUAUGAAUUGACAGCCAGaa--------------------------------
----------------------------- 3 2,2
-------------------------GACCUAUGAAUUGACAGCCAGau--------------------------------
----------------------------- 4 2,2
-------------------------GACCUAUGAAUUGACAGCCAGU---------------------------------
----------------------------- 471 2,3
-------------------------GACCUAUGAAUUGACAGCCAGUa--------------------------------
----------------------------- 30 2,3
-------------------------GACCUAUGAAUUGACAGCCAGUaa-------------------------------
----------------------------- 3 2,3
-------------------------GACCUAUGAAUUGACAGCCAGUu--------------------------------
----------------------------- 70 2,3
-------------------------GACCUAUGAAUUGACAGCCAGUuu-------------------------------
----------------------------- 19 2,3
--CUGCCAAUUCCAUA
GGUCACAG--------------------- 3 0,0
--CUGCCAAUUCCAUA
GGUCACAGu-------------------- 2 0,0

>hsa-mir-193a 5p:21-42;3p:55-76 SRX018957
cgaggaugggagcugagggcUGGGUCUUUGCGGGCGAGAUGAgggugucggaucAACUGGCCUACAAAGUCCCAGUucuc
ggcccccg
.......(((.(((((((((((((.(((((..(((.((.(((..........))).)).)))..))))).))))))))))
))).))).
--------------------UGGGUCUUUGCGGGCGAGAUG---------------------------------------

--------	18	0,-1
--------------------UGGGUCUUUGCGGGCGAGAUGu--------------------------------------
--------	10	0,-1
--------------------UGGGUCUUUGCGGGCGAGAUGA--------------------------------------
--------	138	0,0
--------------------UGGGUCUUUGCGGGCGAGAUGAa-------------------------------------
--------	2	0,0
--------------------UGGGUCUUUGCGGGCGAGAUGAu-------------------------------------
--------	12	0,0
--AACUGGCCUACAAAGUCC--------
--------	7	0,-4
--AACUGGCCUACAAAGUCCC-------
--------	13	0,-3
--AACUGGCCUACAAAGUCCCA------
--------	473	0,-2
--AACUGGCCUACAAAGUCCCAu-----
--------	8	0,-2
--AACUGGCCUACAAAGUCCCAuu----
--------	5	0,-2
--AACUGGCCUACAAAGUCCCAG-----
--------	32	0,-1
--AACUGGCCUACAAAGUCCCAGa----
--------	4	0,-1
--AACUGGCCUACAAAGUCCCAGaa---
--------	3	0,-1
--AACUGGCCUACAAAGUCCCAGU----
--------	14	0,0
--AACUGGCCUACAAAGUCCCAGUa---
--------	3	0,0
---ACUGGCCUACAAAGUCCCA------
--------	3	1,-2

>hsa-mir-193b	mi:14-35;MA:51-72	SRX018957
guggucucagaauCGGGGUUUUGAGGGCGAGAUGAguuuauguuuuauccAACUGGCCCUCAAAGUCCCGCUuuuggggu
cau
(((..(((((((.((((..(((((((((.((.((..(.........)..)).)).)))))))))..)))).)))))))..
)))
------------UCGGGGUUUUGAGGGCGAG---

--- 2 -1,-4

------------UCGGGGUUUUGAGGGCGAGA---

--- 2 -1,-3

------------UCGGGGUUUUGAGGGCGAGAU--

--- 6 -1,-2

------------UCGGGGUUUUGAGGGCGAGAUa---

--- 3 -1,-2

------------UCGGGGUUUUGAGGGCGAGAUG---

--- 8 -1,-1

------------UCGGGGUUUUGAGGGCGAGAUGu--

--- 10 -1,-1

------------UCGGGGUUUUGAGGGCGAGAUGA--

--- 3 -1,0

-------------CGGGGUUUUGAGGGCGAG--

--- 38 0,-4

-------------CGGGGUUUUGAGGGCGAGA---

--- 16 0,-3

-------------CGGGGUUUUGAGGGCGAGAaa---

--- 2 0,-3

-------------CGGGGUUUUGAGGGCGAGAU--

--- 31 0,-2

-------------CGGGGUUUUGAGGGCGAGAUa---

--- 9 0,-2

-------------CGGGGUUUUGAGGGCGAGAUaa--

--- 5 0,-2

-------------CGGGGUUUUGAGGGCGAGAUu---

--- 11 0,-2

-------------CGGGGUUUUGAGGGCGAGAUG---

--- 179 0,-1

-------------CGGGGUUUUGAGGGCGAGAUGu--

--- 168 0,-1

-------------CGGGGUUUUGAGGGCGAGAUGuu---

--- 10 0,-1

-------------CGGGGUUUUGAGGGCGAGAUGA--

--- 722 0,0

-------------CGGGGUUUUGAGGGCGAGAUGAa---

--- 37 0,0

-------------CGGGGUUUUGAGGGCGAGAUGAaa--

--- 4 0,0

-------------CGGGGUUUUGAGGGCGAGAUGAu--

--- 47 0,0

-------------CGGGGUUUUGAGGGCGAGAUGAG--

--- 2 0,1

--------------GGGGUUUUGAGGGCGAGAUG--

--- 3 1,-1

--------------GGGGUUUUGAGGGCGAGAUGu---

--- 6 1,-1

--------------GGGGUUUUGAGGGCGAGAUGA---

--- 21 1,0

--AACUGGCCCUCAAAGUCC------------

--- 14 0,-4

--AACUGGCCCUCAAAGUCCC-----------

--- 25 0,-3

--AACUGGCCCUCAAAGUCCCG----------

--- 15 0,-2

--AACUGGCCCUCAAAGUCCCGC---------

--- 33 0,-1

--AACUGGCCCUCAAAGUCCCGCa--------

--- 2 0,-1

--AACUGGCCCUCAAAGUCCCGCaa-------

--- 2 0,-1

--AACUGGCCCUCAAAGUCCCGCau-------

--- 10 0,-1

--AACUGGCCCUCAAAGUCCCGCU--------

--- 55 0,0

--AACUGGCCCUCAAAGUCCCGCUa-------

--- 13 0,0

--AACUGGCCCUCAAAGUCCCGCUaa------

--- 3 0,0

--AACUGGCCCUCAAAGUCCCGCUau------

--- 9 0,0

--AACUGGCCCUCAAAGUCCCGCUU-------

--- 31 0,1

--AACUGGCCCUCAAAGUCCCGCUUau-----

--- 2 0,1

--AACUGGCCCUCAAAGUCCCGCUUU------

--- 15 0,2
--AACUGGCCCUCAAAGUCCCGCUUUa-----
--- 2   0,2
--AACUGGCCCUCAAAGUCCCGCUUUU-----
--- 2   0,3
---ACUGGCCCUCAAAGUCCCGCau-------
--- 2   1,-1
---ACUGGCCCUCAAAGUCCCGCUa-------
--- 2   1,0
---ACUGGCCCUCAAAGUCCCGCUau------
--- 6   1,0

>hsa-mir-194-1 5p:15-36   SRX018957
augguguuaucaagUGUAACAGCAACUCCAUGUGGAcuguguaccaauuuccaguggagaugcuguuacuuuugaugguu
accaa
.(((((..((((((.(((((((((.((((((.((((..((......)).)))))))))).)))))))))).))))))..).
)))).
--------------UGUAACAGCAACUCCAUG--
-----   23 0,-4
--------------UGUAACAGCAACUCCAUGU---
-----   20 0,-3
--------------UGUAACAGCAACUCCAUGUa--
-----   2   0,-3
--------------UGUAACAGCAACUCCAUGUaa---
-----   2   0,-3
--------------UGUAACAGCAACUCCAUGUG--
-----   21 0,-2
--------------UGUAACAGCAACUCCAUGUGa---
-----   5   0,-2
--------------UGUAACAGCAACUCCAUGUGaa--
-----   2   0,-2
--------------UGUAACAGCAACUCCAUGUGG---
-----   770 0,-1
--------------UGUAACAGCAACUCCAUGUGGu--
-----   14 0,-1
--------------UGUAACAGCAACUCCAUGUGGA--
-----   1404   0,0
--------------UGUAACAGCAACUCCAUGUGGAu---

-----   181 0,0
--------------UGUAACAGCAACUCCAUGUGGAuu--
-----   11 0,0
--------------UGUAACAGCAACUCCAUGUGGAC---
-----   19 0,1
--------------UGUAACAGCAACUCCAUGUGGACa--
-----   10 0,1
---------------GUAACAGCAACUCCAUGUGGA--
-----   3   1,0

>hsa-mir-194-2 MA:15-36;mi:51-72   SRX018957
ugguucccgcccccUGUAACAGCAACUCCAUGUGGAagugcccacugguuCCAGUGGGGCUGCUGUUAUCUGgggcgagg
gccag
((((((.((((((..(((((((((.((((((.((((...(((....))))))))))))).)))))))))..)))))).))
)))).
------------CCUGUAACAGCAACUCCAUGUGG---
-----   8   -2,-1
-------------CUGUAACAGCAACUCCAUGUGG---
-----   21 -1,-1
-------------CUGUAACAGCAACUCCAUGUGGA--
-----   29 -1,0
-------------CUGUAACAGCAACUCCAUGUGGAA---
-----   3   -1,1
--------------UGUAACAGCAACUCCAUG--
-----   23 0,-4
--------------UGUAACAGCAACUCCAUGU---
-----   20 0,-3
--------------UGUAACAGCAACUCCAUGUa--
-----   2   0,-3
--------------UGUAACAGCAACUCCAUGUaa---
-----   2   0,-3
--------------UGUAACAGCAACUCCAUGUG--
-----   21 0,-2
--------------UGUAACAGCAACUCCAUGUGa---
-----   5   0,-2
--------------UGUAACAGCAACUCCAUGUGaa--
-----   2   0,-2
--------------UGUAACAGCAACUCCAUGUGG---

-----   770 0,-1
--------------UGUAACAGCAACUCCAUGUGGu--
-----   14 0,-1
--------------UGUAACAGCAACUCCAUGUGGA--
-----   1404   0,0
--------------UGUAACAGCAACUCCAUGUGGAu---
-----   181 0,0
--------------UGUAACAGCAACUCCAUGUGGAuu--
-----   11 0,0
--------------UGUAACAGCAACUCCAUGUGGAA---
-----   2705   0,1
--------------UGUAACAGCAACUCCAUGUGGAAa--
-----   364 0,1
--------------UGUAACAGCAACUCCAUGUGGAAaa---
-----   16 0,1
--------------UGUAACAGCAACUCCAUGUGGAAau---
-----   10 0,1
--------------UGUAACAGCAACUCCAUGUGGAAu--
-----   117 0,1
--------------UGUAACAGCAACUCCAUGUGGAAuu---
-----   38 0,1
--------------UGUAACAGCAACUCCAUGUGGAAG--
-----   8   0,2
---------------GUAACAGCAACUCCAUGUGGA--
-----   3   1,0
---------------GUAACAGCAACUCCAUGUGGAA---
-----   4   1,1
---------------GUAACAGCAACUCCAUGUGGAAa--
-----   3   1,1
---------------GUAACAGCAACUCCAUGUGGAAau---
-----   2   1,1
--CCAGUGGGGCUGCUGUUAU-----------
-----   4   0,-3
--CCAGUGGGGCUGCUGUUAUC----------
-----   14 0,-2
--CCAGUGGGGCUGCUGUUAUCU---------
-----   104 0,-1
--CCAGUGGGGCUGCUGUUAUCUu--------

-----   4   0,-1
--CCAGUGGGGCUGCUGUUAUCUG--------
-----   11   0,0
--CCAGUGGGGCUGCUGUUAUCUGa-------
-----   5   0,0
--CCAGUGGGGCUGCUGUUAUCUGu-------
-----   3   0,0
--CCAGUGGGGCUGCUGUUAUCUGuu------
-----   3   0,0
---CAGUGGGGCUGCUGUUAUCUGG-------
-----   3   1,1
--AGUGGGGCUGCUGUUAUCU---------
-----   2   2,-1

>hsa-mir-195   MA:15-35;mi:53-74   SRX018957
agcuucccuggcucUAGCAGCACAGAAAUAUUGGCacagggaagcgagucugCCAAUAUUGGCUGUGCUGCUCCaggcag
gguggug
.(((.(((((.((..((((((((((.((((((((((..............))))))))))..))))))))))..)).)))
)).))).
-------------CUAGCAGCACAGAAAUAUUGGCA--
------- 2   -1,1
--------------UAGCAGCACAGAAAUAUU--
------- 4   0,-3
--------------UAGCAGCACAGAAAUAUUa---
------- 2   0,-3
--------------UAGCAGCACAGAAAUAUUG---
------- 14   0,-2
--------------UAGCAGCACAGAAAUAUUGu--
------- 2   0,-2
--------------UAGCAGCACAGAAAUAUUGG--
------- 86   0,-1
--------------UAGCAGCACAGAAAUAUUGGa---
------- 9   0,-1
--------------UAGCAGCACAGAAAUAUUGGaa--
------- 3   0,-1
--------------UAGCAGCACAGAAAUAUUGGC---
------- 140 0,0
--------------UAGCAGCACAGAAAUAUUGGCA--

------- 594 0,1
--------------UAGCAGCACAGAAAUAUUGGCAa---
------- 570 0,1
--------------UAGCAGCACAGAAAUAUUGGCAaa--
------- 6 0,1
--------------UAGCAGCACAGAAAUAUUGGCAau--
------- 6 0,1
--------------UAGCAGCACAGAAAUAUUGGCAu---
------- 217 0,1
--------------UAGCAGCACAGAAAUAUUGGCAuu--
------- 29 0,1
---------------AGCAGCACAGAAAUAUUGGCA--
------- 5 1,1
---------------AGCAGCACAGAAAUAUUGGCAa---
------- 10 1,1
---------------AGCAGCACAGAAAUAUUGGCAu---
------- 4 1,1
----------------GCAGCACAGAAAUAUUGGCAu---
------- 2 2,1

>hsa-mir-197 3p:48-69 SRX018957
ggcugugccggguagagagggcagugggagguaagagcucuucacccUUCACCACCUUCUCCACCCAGCauggcc
((((((((.((((.(((((((..((((..((((((....))).)))..))))..))))))).)))).))))))))
--------CGGGUAGAGAGGGCAGUGG-- 2
---UUCACCACCUUCUCCACCCAG------- 2
0,-1
---UUCACCACCUUCUCCACCCAGC------ 16
0,0

>hsa-mir-199a-1 5p:6-28;3p:47-68 SRX018957
gccaaCCCAGUGUUCAGACUACCUGUUCaggaggcucucaauguguACAGUAGUCUGCACAUUGGUUAggc
(((...(((((((.((((((((.(((.((.(((...)))....)).))))))))))).)))))))...)))
-----CCCAGUGUUCAGACUACC-- 21 0,-5
-----CCCAGUGUUCAGACUACCU--- 375 0,-4
-----CCCAGUGUUCAGACUACCUG-- 12 0,-3
-----CCCAGUGUUCAGACUACCUGU--- 7 0,-2
-----CCCAGUGUUCAGACUACCUGUU-- 140 0,-1
-----CCCAGUGUUCAGACUACCUGUUa--- 24 0,-1

-----CCCAGUGUUCAGACUACCUGUUu-- 9 0,-1
-----CCCAGUGUUCAGACUACCUGUUC-- 92 0,0
-----CCCAGUGUUCAGACUACCUGUUCA--- 4 0,1
------CCAGUGUUCAGACUACCU-- 6 1,-4
------CCAGUGUUCAGACUACCUGUU--- 3 1,-1
------CCAGUGUUCAGACUACCUGUUC-- 4 1,0
---UACAGUAGUCUGCACAUU-------- 15
-1,-5
---UACAGUAGUCUGCACAUUG------- 63
-1,-4
---UACAGUAGUCUGCACAUUGuu----- 4
-1,-4
---UACAGUAGUCUGCACAUUGG------ 106
-1,-3
---UACAGUAGUCUGCACAUUGGa----- 7
-1,-3
---UACAGUAGUCUGCACAUUGGaa---- 6
-1,-3
---UACAGUAGUCUGCACAUUGGau---- 3
-1,-3
---UACAGUAGUCUGCACAUUGGU----- 868
-1,-2
---UACAGUAGUCUGCACAUUGGUa---- 202
-1,-2
---UACAGUAGUCUGCACAUUGGUaa--- 138
-1,-2
---UACAGUAGUCUGCACAUUGGUau--- 79
-1,-2
---UACAGUAGUCUGCACAUUGGUU---- 7241
-1,-1
---UACAGUAGUCUGCACAUUGGUUu--- 1195
-1,-1
---UACAGUAGUCUGCACAUUGGUUuu-- 46
-1,-1
---UACAGUAGUCUGCACAUUGGUUA--- 1497
-1,0
---UACAGUAGUCUGCACAUUGGUUAa-- 79 -1,0
---UACAGUAGUCUGCACAUUGGUUAaa- 2 -1,0

---UACAGUAGUCUGCACAUUGGUUAu-- 93 -1,0
---UACAGUAGUCUGCACAUUGGUUAuu- 2 -1,0
--ACAGUAGUCUGCACAUUG------- 290 0,-4
--ACAGUAGUCUGCACAUUGa------ 11 0,-4
--ACAGUAGUCUGCACAUUGaa----- 3 0,-4
--ACAGUAGUCUGCACAUUGu------ 30 0,-4
--ACAGUAGUCUGCACAUUGuu----- 22 0,-4
--ACAGUAGUCUGCACAUUGG------ 887 0,-3
--ACAGUAGUCUGCACAUUGGa----- 21 0,-3
--ACAGUAGUCUGCACAUUGGaa---- 20 0,-3
--ACAGUAGUCUGCACAUUGGau---- 14 0,-3
--ACAGUAGUCUGCACAUUGGU----- 3320
0,-2
--ACAGUAGUCUGCACAUUGGUa---- 671 0,-2
--ACAGUAGUCUGCACAUUGGUaa--- 539 0,-2
--ACAGUAGUCUGCACAUUGGUau--- 112 0,-2
--ACAGUAGUCUGCACAUUGGUU---- 50760
0,-1
--ACAGUAGUCUGCACAUUGGUUu--- 7307
0,-1
--ACAGUAGUCUGCACAUUGGUUuu-- 377 0,-1
--ACAGUAGUCUGCACAUUGGUUA--- 25123
0,0
--ACAGUAGUCUGCACAUUGGUUAa-- 6228
0,0
--ACAGUAGUCUGCACAUUGGUUAaa- 70 0,0
--ACAGUAGUCUGCACAUUGGUUAau- 48 0,0
--ACAGUAGUCUGCACAUUGGUUAu-- 1387
0,0
--ACAGUAGUCUGCACAUUGGUUAuu- 43 0,0
--ACAGUAGUCUGCACAUUGGUUAG-- 39 0,1
--ACAGUAGUCUGCACAUUGGUUAGu- 3 0,1
---CAGUAGUCUGCACAUUGG------ 273 1,-3
---CAGUAGUCUGCACAUUGGa----- 5 1,-3
---CAGUAGUCUGCACAUUGGaa---- 7 1,-3
---CAGUAGUCUGCACAUUGGau---- 3 1,-3
---CAGUAGUCUGCACAUUGGU----- 673 1,-2
---CAGUAGUCUGCACAUUGGUa---- 182 1,-2

---CAGUAGUCUGCACAUUGGUaa--- 153 1,-2
---CAGUAGUCUGCACAUUGGUau--- 14 1,-2
---CAGUAGUCUGCACAUUGGUU---- 10322
1,-1
---CAGUAGUCUGCACAUUGGUUu--- 1696
1,-1
---CAGUAGUCUGCACAUUGGUUuu-- 82 1,-1
---CAGUAGUCUGCACAUUGGUUA--- 11768
1,0
---CAGUAGUCUGCACAUUGGUUAa-- 2237
1,0
---CAGUAGUCUGCACAUUGGUUAaa- 80 1,0
---CAGUAGUCUGCACAUUGGUUAau- 18 1,0
---CAGUAGUCUGCACAUUGGUUAu-- 769 1,0
---CAGUAGUCUGCACAUUGGUUAuu- 12 1,0
---CAGUAGUCUGCACAUUGGUUAG-- 27 1,1
--AGUAGUCUGCACAUUGGU----- 13 2,-2
--AGUAGUCUGCACAUUGGUa---- 3 2,-2
--AGUAGUCUGCACAUUGGUU---- 149 2,-1
--AGUAGUCUGCACAUUGGUUu--- 20 2,-1
--AGUAGUCUGCACAUUGGUUuu-- 4 2,-1
--AGUAGUCUGCACAUUGGUUA--- 177 2,0
--AGUAGUCUGCACAUUGGUUAa-- 47 2,0
--AGUAGUCUGCACAUUGGUUAaa- 4 2,0
--AGUAGUCUGCACAUUGGUUAu-- 15 2,0

>hsa-mir-199a-2 5p:31-53;3p:70-91 SRX018957
aggaagcuucuggagauccugcuccgucgcCCCAGUGUUCAGACUACCUGUUCaggacaaugccguuguACAGUAGUCUG
CACAUUGGUUAgacugggcaagggagagca
.....(((.((.....(((((((.(((...((((((.((((((((.(((...((......)).....))))))))))
.)))))))...))).)))).))))))))).
------------------------------CCCAGUGUUCAGACUACC--------------------------------
------------------------------ 21 0,-5
------------------------------CCCAGUGUUCAGACUACCU-------------------------------
------------------------------ 375 0,-4
------------------------------CCCAGUGUUCAGACUACCUG------------------------------
------------------------------ 12 0,-3
------------------------------CCCAGUGUUCAGACUACCUGU-----------------------------

------------------------------ 7 0,-2
------------------------------CCCAGUGUUCAGACUACCUGUU----------------------------
------------------------------ 140 0,-1
------------------------------CCCAGUGUUCAGACUACCUGUUa---------------------------
------------------------------ 24 0,-1
------------------------------CCCAGUGUUCAGACUACCUGUUu---------------------------
------------------------------ 9 0,-1
------------------------------CCCAGUGUUCAGACUACCUGUUC---------------------------
------------------------------ 92 0,0
------------------------------CCCAGUGUUCAGACUACCUGUUCA--------------------------
------------------------------ 4 0,1
-------------------------------CCAGUGUUCAGACUACCU-------------------------------
------------------------------ 6 1,-4
-------------------------------CCAGUGUUCAGACUACCUGUU----------------------------
------------------------------ 3 1,-1
-------------------------------CCAGUGUUCAGACUACCUGUUC---------------------------
------------------------------ 4 1,0
--UACAGUAGUCUG
CACAUU------------------------ 15 -1,-5
--UACAGUAGUCUG
CACAUUG----------------------- 63 -1,-4
--UACAGUAGUCUG
CACAUUGuu--------------------- 4 -1,-4
--UACAGUAGUCUG
CACAUUGG---------------------- 106 -1,-3
--UACAGUAGUCUG
CACAUUGGa--------------------- 7 -1,-3
--UACAGUAGUCUG
CACAUUGGaa-------------------- 6 -1,-3
--UACAGUAGUCUG
CACAUUGGau-------------------- 3 -1,-3
--UACAGUAGUCUG
CACAUUGGU--------------------- 868 -1,-2
--UACAGUAGUCUG
CACAUUGGUa-------------------- 202 -1,-2
--UACAGUAGUCUG
CACAUUGGUaa------------------- 138 -1,-2
--UACAGUAGUCUG

CACAUUGGUau------------------- 79 -1,-2
---UACAGUAGUCUG
CACAUUGGUU-------------------- 7241 -1,-1
---UACAGUAGUCUG
CACAUUGGUUu------------------- 1195 -1,-1
---UACAGUAGUCUG
CACAUUGGUUuu------------------ 46 -1,-1
---UACAGUAGUCUG
CACAUUGGUUA------------------- 1497 -1,0
---UACAGUAGUCUG
CACAUUGGUUAa------------------ 79 -1,0
---UACAGUAGUCUG
CACAUUGGUUAaa----------------- 2 -1,0
---UACAGUAGUCUG
CACAUUGGUUAu------------------ 93 -1,0
---UACAGUAGUCUG
CACAUUGGUUAuu----------------- 2 -1,0
--ACAGUAGUCUG
CACAUUG----------------------- 290 0,-4
--ACAGUAGUCUG
CACAUUGa---------------------- 11 0,-4
--ACAGUAGUCUG
CACAUUGaa--------------------- 3 0,-4
--ACAGUAGUCUG
CACAUUGu---------------------- 30 0,-4
--ACAGUAGUCUG
CACAUUGuu--------------------- 22 0,-4
--ACAGUAGUCUG
CACAUUGG---------------------- 887 0,-3
--ACAGUAGUCUG
CACAUUGGa--------------------- 21 0,-3
--ACAGUAGUCUG
CACAUUGGaa-------------------- 20 0,-3
--ACAGUAGUCUG
CACAUUGGau-------------------- 14 0,-3
--ACAGUAGUCUG
CACAUUGGU--------------------- 3320 0,-2
--ACAGUAGUCUG

CACAUUGGUa-------------------- 671 0,-2

--ACAGUAGUCUG

CACAUUGGUaa------------------- 539 0,-2

--ACAGUAGUCUG

CACAUUGGUau------------------- 112 0,-2

--ACAGUAGUCUG

CACAUUGGUU-------------------- 50760 0,-1

--ACAGUAGUCUG

CACAUUGGUUu------------------- 7307 0,-1

--ACAGUAGUCUG

CACAUUGGUUuu------------------ 377 0,-1

--ACAGUAGUCUG

CACAUUGGUUA------------------- 25123 0,0

--ACAGUAGUCUG

CACAUUGGUUAa------------------ 6228 0,0

--ACAGUAGUCUG

CACAUUGGUUAaa----------------- 70 0,0

--ACAGUAGUCUG

CACAUUGGUUAau----------------- 48 0,0

--ACAGUAGUCUG

CACAUUGGUUAu------------------ 1387 0,0

--ACAGUAGUCUG

CACAUUGGUUAuu----------------- 43 0,0

--ACAGUAGUCUG

CACAUUGGUUAG------------------ 39 0,1

--ACAGUAGUCUG

CACAUUGGUUAGu----------------- 3 0,1

--ACAGUAGUCUG

CACAUUGGUUAGA----------------- 2 0,2

---CAGUAGUCUG

CACAUUGG---------------------- 273 1,-3

---CAGUAGUCUG

CACAUUGGa--------------------- 5 1,-3

---CAGUAGUCUG

CACAUUGGaa-------------------- 7 1,-3

---CAGUAGUCUG

CACAUUGGau-------------------- 3 1,-3

---CAGUAGUCUG

CACAUUGGU--------------------- 673 1,-2
--CAGUAGUCUG
CACAUUGGUa-------------------- 182 1,-2
--CAGUAGUCUG
CACAUUGGUaa------------------- 153 1,-2
--CAGUAGUCUG
CACAUUGGUau------------------- 14 1,-2
--CAGUAGUCUG
CACAUUGGUU-------------------- 10322 1,-1
--CAGUAGUCUG
CACAUUGGUUu------------------- 1696 1,-1
--CAGUAGUCUG
CACAUUGGUUuu------------------ 82 1,-1
--CAGUAGUCUG
CACAUUGGUUA------------------- 11768 1,0
--CAGUAGUCUG
CACAUUGGUUAa------------------ 2237 1,0
--CAGUAGUCUG
CACAUUGGUUAaa----------------- 80 1,0
--CAGUAGUCUG
CACAUUGGUUAau----------------- 18 1,0
--CAGUAGUCUG
CACAUUGGUUAu------------------ 769 1,0
--CAGUAGUCUG
CACAUUGGUUAuu----------------- 12 1,0
--CAGUAGUCUG
CACAUUGGUUAG------------------ 27 1,1
---AGUAGUCUG
CACAUUGGU--------------------- 13 2,-2
---AGUAGUCUG
CACAUUGGUa-------------------- 3 2,-2
---AGUAGUCUG
CACAUUGGUU-------------------- 149 2,-1
---AGUAGUCUG
CACAUUGGUUu------------------- 20 2,-1
---AGUAGUCUG
CACAUUGGUUuu------------------ 4 2,-1
---AGUAGUCUG

CACAUUGGUUA------------------- 177 2,0
---AGUAGUCUG
CACAUUGGUUAa------------------ 47 2,0
---AGUAGUCUG
CACAUUGGUUAaa----------------- 4 2,0
---AGUAGUCUG
CACAUUGGUUAu------------------ 15 2,0

>hsa-mir-199b 5p:26-48;3p:65-86 SRX018957
ccagaggacaccuccacuccgucuaCCCAGUGUUUAGACUAUCUGUUCaggacucccaaauuguACAGUAGUCUGCACAU
UGGUUAggcugggcuggguuagacccucgg
...((((.....((((..(((((((.(((((((.(((((((.((((.((((....))....)).))))))))))).))))
))).))))).))..))))......))))..
-------------------------CCCAGUGUUUAGACUAUC-------------------------------------
------------------------------ 5 0,-5
-------------------------CCCAGUGUUUAGACUAUCU------------------------------------
------------------------------ 55 0,-4
-------------------------CCCAGUGUUUAGACUAUCUGUUa--------------------------------
------------------------------ 3 0,-1
-------------------------CCCAGUGUUUAGACUAUCUGUUC--------------------------------
------------------------------ 6 0,0
--------------------------CCAGUGUUUAGACUAUCU------------------------------------
------------------------------ 2 1,-4
---UACAGUAGUCUGCACAU
U----------------------------- 15 -1,-5
---UACAGUAGUCUGCACAU
UG---------------------------- 63 -1,-4
---UACAGUAGUCUGCACAU
UGuu-------------------------- 4 -1,-4
---UACAGUAGUCUGCACAU
UGG--------------------------- 106 -1,-3
---UACAGUAGUCUGCACAU
UGGa-------------------------- 7 -1,-3
---UACAGUAGUCUGCACAU
UGGaa------------------------- 6 -1,-3
---UACAGUAGUCUGCACAU
UGGau------------------------- 3 -1,-3
---UACAGUAGUCUGCACAU

UGGU-------------------------- 868 -1,-2

---UACAGUAGUCUGCACAU

UGGUa------------------------- 202 -1,-2

---UACAGUAGUCUGCACAU

UGGUaa------------------------ 138 -1,-2

---UACAGUAGUCUGCACAU

UGGUau------------------------ 79 -1,-2

---UACAGUAGUCUGCACAU

UGGUU------------------------- 7241 -1,-1

---UACAGUAGUCUGCACAU

UGGUUu------------------------ 1195 -1,-1

---UACAGUAGUCUGCACAU

UGGUUuu----------------------- 46 -1,-1

---UACAGUAGUCUGCACAU

UGGUUA------------------------ 1497 -1,0

---UACAGUAGUCUGCACAU

UGGUUAa----------------------- 79 -1,0

---UACAGUAGUCUGCACAU

UGGUUAaa---------------------- 2 -1,0

---UACAGUAGUCUGCACAU

UGGUUAu----------------------- 93 -1,0

---UACAGUAGUCUGCACAU

UGGUUAuu---------------------- 2 -1,0

--ACAGUAGUCUGCACAU

UG---------------------------- 290 0,-4

--ACAGUAGUCUGCACAU

UGa--------------------------- 11 0,-4

--ACAGUAGUCUGCACAU

UGaa-------------------------- 3 0,-4

--ACAGUAGUCUGCACAU

UGu--------------------------- 30 0,-4

--ACAGUAGUCUGCACAU

UGuu-------------------------- 22 0,-4

--ACAGUAGUCUGCACAU

UGG--------------------------- 887 0,-3

--ACAGUAGUCUGCACAU

UGGa-------------------------- 21 0,-3

--ACAGUAGUCUGCACAU

UGGaa------------------------ 20 0,-3
---ACAGUAGUCUGCACAU
UGGau------------------------ 14 0,-3
---ACAGUAGUCUGCACAU
UGGU------------------------- 3320 0,-2
---ACAGUAGUCUGCACAU
UGGUa------------------------ 671 0,-2
---ACAGUAGUCUGCACAU
UGGUaa----------------------- 539 0,-2
---ACAGUAGUCUGCACAU
UGGUau----------------------- 112 0,-2
---ACAGUAGUCUGCACAU
UGGUU------------------------ 50760 0,-1
---ACAGUAGUCUGCACAU
UGGUUu----------------------- 7307 0,-1
---ACAGUAGUCUGCACAU
UGGUUuu---------------------- 377 0,-1
---ACAGUAGUCUGCACAU
UGGUUA----------------------- 25123 0,0
---ACAGUAGUCUGCACAU
UGGUUAa---------------------- 6228 0,0
---ACAGUAGUCUGCACAU
UGGUUAaa--------------------- 70 0,0
---ACAGUAGUCUGCACAU
UGGUUAau--------------------- 48 0,0
---ACAGUAGUCUGCACAU
UGGUUAu---------------------- 1387 0,0
---ACAGUAGUCUGCACAU
UGGUUAuu--------------------- 43 0,0
---ACAGUAGUCUGCACAU
UGGUUAG---------------------- 39 0,1
---ACAGUAGUCUGCACAU
UGGUUAGu--------------------- 3 0,1
--CAGUAGUCUGCACAU
UGG-------------------------- 273 1,-3
--CAGUAGUCUGCACAU
UGGa------------------------- 5 1,-3
--CAGUAGUCUGCACAU

UGGaa------------------------- 7   1,-3

--CAGUAGUCUGCACAU

UGGau------------------------- 3   1,-3

--CAGUAGUCUGCACAU

UGGU-------------------------- 673 1,-2

--CAGUAGUCUGCACAU

UGGUa------------------------- 182 1,-2

--CAGUAGUCUGCACAU

UGGUaa------------------------ 153 1,-2

--CAGUAGUCUGCACAU

UGGUau------------------------ 14   1,-2

--CAGUAGUCUGCACAU

UGGUU------------------------- 10322   1,-1

--CAGUAGUCUGCACAU

UGGUUu------------------------ 1696   1,-1

--CAGUAGUCUGCACAU

UGGUUuu----------------------- 82   1,-1

--CAGUAGUCUGCACAU

UGGUUA------------------------ 11768   1,0

--CAGUAGUCUGCACAU

UGGUUAa----------------------- 2237   1,0

--CAGUAGUCUGCACAU

UGGUUAaa---------------------- 80   1,0

--CAGUAGUCUGCACAU

UGGUUAau---------------------- 18   1,0

--CAGUAGUCUGCACAU

UGGUUAu----------------------- 769 1,0

--CAGUAGUCUGCACAU

UGGUUAuu---------------------- 12   1,0

--CAGUAGUCUGCACAU

UGGUUAG----------------------- 27   1,1

---AGUAGUCUGCACAU

UGGU-------------------------- 13   2,-2

---AGUAGUCUGCACAU

UGGUa------------------------- 3   2,-2

---AGUAGUCUGCACAU

UGGUU------------------------- 149 2,-1

---AGUAGUCUGCACAU

UGGUUu------------------------ 20 2,-1
--AGUAGUCUGCACAU
UGGUUuu----------------------- 4   2,-1
--AGUAGUCUGCACAU
UGGUUA------------------------ 177 2,0
--AGUAGUCUGCACAU
UGGUUAa----------------------- 47 2,0
--AGUAGUCUGCACAU
UGGUUAaa---------------------- 4   2,0
--AGUAGUCUGCACAU
UGGUUAu----------------------- 15 2,0

>hsa-mir-19b-1 mi:16-38;MA:54-76   SRX018957
cacuguucuaugguuAGUUUUGCAGGUUUGCAUCCAGCugugugauauucugcUGUGCAAAUCCAUGCAAAACUGAcugu
gguagug
(((((..(((((((((((((((((((((((((..((((.............))))))))))).)).)))))))))))))))
)))))))
---UGUGCAAAUCCAUGCAAAAC-------
------- 3   0,-3
---UGUGCAAAUCCAUGCAAAACU------
------- 28 0,-2
---UGUGCAAAUCCAUGCAAAACUG-----
------- 29 0,-1
---UGUGCAAAUCCAUGCAAAACUGu----
------- 3   0,-1
---UGUGCAAAUCCAUGCAAAACUGA----
------- 11 0,0

>hsa-mir-19b-2 mi:19-40;MA:62-84   SRX018957
acauugcuacuuacaauuAGUUUUGCAGGUUUGCAUUUCAgcguauauauguauauguggcUGUGCAAAUCCAUGCAAAA
CUGAuugugauaaugu
((((((....((((((((((((((((((((((((((....(((((((....)))))))....))))))).)).)))))))
))))))))))))))))
---UGUGCAAAUCCAUGCAAAA
C---------------   3   0,-3
---UGUGCAAAUCCAUGCAAAA
CU--------------   28 0,-2
---UGUGCAAAUCCAUGCAAAA

CUG------------ 29 0,-1
---UGUGCAAAUCCAUGCAAAA
CUGu----------- 3 0,-1
---UGUGCAAAUCCAUGCAAAA
CUGA----------- 11 0,0

>hsa-mir-200a mi:16-37;MA:54-75 SRX018957
ccgggccccugugagCAUCUUACCGGACAGUGCUGGAuuucccagcuugacucUAACACUGUCUGGUAACGAUGUucaaa
ggugacccgc
.(((((.(((.(((((((((((((((((((((((((.....)))))...........)))))))))))).)))))))).)
)).).)))).
--CUAACACUGUCUGGUAACGAUG------
---------- 2 -1,-1
---UAACACUGUCUGGUAACGAU-------
---------- 32 0,-2
---UAACACUGUCUGGUAACGAUG------
---------- 21 0,-1
---UAACACUGUCUGGUAACGAUGa-----
---------- 4 0,-1
---UAACACUGUCUGGUAACGAUGaa----
---------- 3 0,-1
---UAACACUGUCUGGUAACGAUGU-----
---------- 14 0,0
---UAACACUGUCUGGUAACGAUGUa----
---------- 4 0,0
---UAACACUGUCUGGUAACGAUGUaa---
---------- 3 0,0
---UAACACUGUCUGGUAACGAUGUU----
---------- 36 0,1
---UAACACUGUCUGGUAACGAUGUUa---
---------- 7 0,1
---UAACACUGUCUGGUAACGAUGUUu---
---------- 14 0,1
--AACACUGUCUGGUAACGAUGUa----
---------- 2 1,0
--AACACUGUCUGGUAACGAUGUU----
---------- 2 1,1
--AACACUGUCUGGUAACGAUGUUa---

---------- 5 1,1
--AACACUGUCUGGUAACGAUGUUu---
---------- 2 1,1

>hsa-mir-200b mi:21-42;MA:57-78 SRX018957
ccagcucgggcagccguggcCAUCUUACUGGGCAGCAUUGGAuggagucaggucucUAAUACUGCCUGGUAAUGAUGAcg
gcggagcccugcacg
...((..((((..((((.(.((((((((..(((((.((((((.((.......)))))))).)))))..)))).)))).).
)))).)))))).))...
--------------------CAUCUUACUGGGCAGCAUU---
--------------- 13 0,-3
--------------------CAUCUUACUGGGCAGCAUUG--
--------------- 4 0,-2
--------------------CAUCUUACUGGGCAGCAUUGG---------------------------------------
--------------- 18 0,-1
--------------------CAUCUUACUGGGCAGCAUUGGA--------------------------------------
--------------- 20 0,0
--------------------CAUCUUACUGGGCAGCAUUGGAa-------------------------------------
--------------- 7 0,0
--------------------CAUCUUACUGGGCAGCAUUGGAU-------------------------------------
--------------- 2 0,1
---------------------AUCUUACUGGGCAGCAUU---
--------------- 4 1,-3
---------------------AUCUUACUGGGCAGCAUUGG---------------------------------------
--------------- 2 1,-1
--UAAUACUGCCUGGUAAUG------
--------------- 2 0,-4
--UAAUACUGCCUGGUAAUGAU----
--------------- 4 0,-2
--UAAUACUGCCUGGUAAUGAUa---
--------------- 3 0,-2
--UAAUACUGCCUGGUAAUGAUG---
--------------- 21 0,-1
--UAAUACUGCCUGGUAAUGAUGu--
--------------- 5 0,-1
--UAAUACUGCCUGGUAAUGAUGuu-
--------------- 2 0,-1
--UAAUACUGCCUGGUAAUGAUGA--

--------------- 111 0,0

--UAAUACUGCCUGGUAAUGAUGAa-

--------------- 14 0,0

--UAAUACUGCCUGGUAAUGAUGAaa

--------------- 4 0,0

--UAAUACUGCCUGGUAAUGAUGAu-

--------------- 5 0,0

--UAAUACUGCCUGGUAAUGAUGAC-

--------------- 50 0,1

--UAAUACUGCCUGGUAAUGAUGACa

--------------- 44 0,1

--UAAUACUGCCUGGUAAUGAUGACa

a-------------- 2 0,1

--UAAUACUGCCUGGUAAUGAUGACa

u-------------- 3 0,1

--UAAUACUGCCUGGUAAUGAUGACu

--------------- 46 0,1

---AAUACUGCCUGGUAAUGAUGAC-

--------------- 6 1,1

---AAUACUGCCUGGUAAUGAUGACa

--------------- 9 1,1

---AAUACUGCCUGGUAAUGAUGACu

--------------- 22 1,1

--AUACUGCCUGGUAAUGAUGA--

--------------- 2 2,0

--AUACUGCCUGGUAAUGAUGAC-

--------------- 13 2,1

--AUACUGCCUGGUAAUGAUGACa

--------------- 4 2,1

--AUACUGCCUGGUAAUGAUGACu

--------------- 24 2,1

--AUACUGCCUGGUAAUGAUGACu

u-------------- 5 2,1

--AUACUGCCUGGUAAUGAUGACG

--------------- 2 2,2

>hsa-mir-200c mi:5-26;MA:44-66 SRX018957

cccuCGUCUUACCCAGCAGUGUUUGGgugcgguugggagucucUAAUACUGCCGGGUAAUGAUGGAgg

((..((((((((((.((((((((.(((.((........))))).)))))))).)))))).))))..))
---UAAUACUGCCGGGUAAUGAUGG---   12   0,-1
---UAAUACUGCCGGGUAAUGAUGGA--   4   0,0

>hsa-mir-203   3p:65-86   SRX018957
guguuggggacucgcgcgcuggguccagugguucuuaacaguucaacaguucuguagcgcaauuGUGAAAUGUUUAGGAC
CACUAGacccggcgggcgcggcgacagcga
.(((((..(.(.(((.(((((((((.(((((((((.((((.((((.((((((......).))))))))).)))).)))))
)))).))))))))).))).).)..))))).
---UGUGAAAUGUUUAGGAC
CACU--------------------------   2   -1,-2
---UGUGAAAUGUUUAGGAC
CACUAG------------------------   2   -1,0
--GUGAAAUGUUUAGGAC
CACUA-------------------------   8   0,-1
--GUGAAAUGUUUAGGAC
CACUAu------------------------   2   0,-1
--GUGAAAUGUUUAGGAC
CACUAG------------------------   49   0,0
--GUGAAAUGUUUAGGAC
CACUAGu-----------------------   11   0,0
--GUGAAAUGUUUAGGAC
CACUAGA-----------------------   10   0,1
--GUGAAAUGUUUAGGAC
CACUAGAa----------------------   2   0,1
---UGAAAUGUUUAGGAC
CACU--------------------------   2   1,-2
---UGAAAUGUUUAGGAC
CACUA-------------------------   2   1,-1
---UGAAAUGUUUAGGAC
CACUAG------------------------   22   1,0
---UGAAAUGUUUAGGAC
CACUAGu-----------------------   8   1,0
---UGAAAUGUUUAGGAC
CACUAGA-----------------------   5   1,1

>hsa-mir-204   5p:33-54   SRX018957
ggcuacagucuuucuucaugugacucguggacUUCCCUUUGUCAUCCUAUGCCUgagaauauaugaaggaggcugggaag

gcaaagggacguucaauugucaucacuggc
...............(((((((((...(((((.((((((((((.(((((.((((...............))))))))).)
))))))))).)))))...)))).)).))).
--------------------------------UUCCCUUUGUCAUCCUAUGCC---------------------------
------------------------------ 2 0,-1
--------------------------------UUCCCUUUGUCAUCCUAUGCCU--------------------------
------------------------------ 2 0,0
---GCUGGGAAG
GCAAAGGGACGU------------------ 9
---GCUGGGAAG
GCAAAGGGACGUaa---------------- 3
---GCUGGGAAG
GCAAAGGGACGUU----------------- 4

>hsa-mir-206 3p:53-74 SRX018957
ugcuucccgaggccacaugcuucuuuauauccccauauggauuacuuugcuaUGGAAUGUAAGGAAGUGUGUGGuuucgg
caagug
.((((.((((((((((((((((((((((((..(((((.(.(......).)))))).))))))))))))))))))))))))
.)))).
--UGGAAUGUAAGGAAGUGUGUG-------
------ 5 0,-1
--UGGAAUGUAAGGAAGUGUGUGG------
------ 20 0,0
--UGGAAUGUAAGGAAGUGUGUGGU-----
------ 2 0,1

>hsa-mir-20a MA:8-30;mi:44-65 SRX018957
guagcacUAAAGUGCUUAUAGUGCAGGUAGuguuuaguuaucuACUGCAUUAUGAGCACUUAAAGuacugc
((((.(((.(((((((((((((((((.(((((......)).))))))))))))))))))))..))).))))
-------UAAAGUGCUUAUAGUGCA-- 3 0,-5
-------UAAAGUGCUUAUAGUGCAG--- 18 0,-4
-------UAAAGUGCUUAUAGUGCAGG-- 10 0,-3
-------UAAAGUGCUUAUAGUGCAGGU--- 16 0,-2
-------UAAAGUGCUUAUAGUGCAGGUA-- 62 0,-1
-------UAAAGUGCUUAUAGUGCAGGUAa--- 22 0,-1
-------UAAAGUGCUUAUAGUGCAGGUAu--- 41 0,-1
-------UAAAGUGCUUAUAGUGCAGGUAG--- 46 0,0
-------UAAAGUGCUUAUAGUGCAGGUAGa-- 3 0,0

-------UAAAGUGCUUAUAGUGCAGGUAGU-------------------------------------- 4 0,1
--------AAAGUGCUUAUAGUGCAGGUA-- 3 1,-1
--------AAAGUGCUUAUAGUGCAGGUAG--------------------------------------- 2 1,0

>hsa-mir-21 MA:8-29;mi:46-66 SRX018957
ugucgggUAGCUUAUCAGACUGAUGUUGAcuguugaaucucauggCAACACCAGUCGAUGGGCUGUcugaca
.(((((..((((((((.(((((.(((((.((((.(.....))))))))).))))))))))))..))))).
------GUAGCUUAUCAGACUGAUGUU-- 7
-1,-2
------GUAGCUUAUCAGACUGAUGUUG--- 15
-1,-1
------GUAGCUUAUCAGACUGAUGUUGA-- 14
-1,0
------GUAGCUUAUCAGACUGAUGUUGAC--- 3
-1,1
-------UAGCUUAUCAGACUGAUG-- 191
0,-4
-------UAGCUUAUCAGACUGAUGU--- 736
0,-3
-------UAGCUUAUCAGACUGAUGUa-- 20
0,-3
-------UAGCUUAUCAGACUGAUGUaa--- 13
0,-3
-------UAGCUUAUCAGACUGAUGUU-- 5413
0,-2
-------UAGCUUAUCAGACUGAUGUUa--- 36
0,-2
-------UAGCUUAUCAGACUGAUGUUaa-- 8
0,-2
-------UAGCUUAUCAGACUGAUGUUu--- 6
0,-2
-------UAGCUUAUCAGACUGAUGUUG---
11762 0,-1
-------UAGCUUAUCAGACUGAUGUUGu-- 23
0,-1
-------UAGCUUAUCAGACUGAUGUUGA-- 4949
0,0
-------UAGCUUAUCAGACUGAUGUUGAa--- 442

0,0

-------UAGCUUAUCAGACUGAUGUUGAaa-- 34

0,0

-------UAGCUUAUCAGACUGAUGUUGAau-- 15

0,0

-------UAGCUUAUCAGACUGAUGUUGAu--- 13

0,0

-------UAGCUUAUCAGACUGAUGUUGAC--- 2501

0,1

-------UAGCUUAUCAGACUGAUGUUGACa-- 2411

0,1

-------UAGCUUAUCAGACUGAUGUUGACaa--- 11

0,1

-------UAGCUUAUCAGACUGAUGUUGACau--- 4

0,1

-------UAGCUUAUCAGACUGAUGUUGACU-- 1107

0,2

-------UAGCUUAUCAGACUGAUGUUGACUa--- 2

0,2

--------AGCUUAUCAGACUGAUGU--- 4

1,-3

--------AGCUUAUCAGACUGAUGUU-- 21

1,-2

--------AGCUUAUCAGACUGAUGUUG--- 46

1,-1

--------AGCUUAUCAGACUGAUGUUGA-- 59

1,0

--------AGCUUAUCAGACUGAUGUUGAa--- 3

1,0

--------AGCUUAUCAGACUGAUGUUGAaa-- 2

1,0

--------AGCUUAUCAGACUGAUGUUGAC--- 31

1,1

--------AGCUUAUCAGACUGAUGUUGACa-- 44

1,1

--------AGCUUAUCAGACUGAUGUUGACau--- 3

1,1

--------AGCUUAUCAGACUGAUGUUGACU-- 11

1,2

---------GCUUAUCAGACUGAUGUU--- 7

2,-2

---------GCUUAUCAGACUGAUGUUG-- 26

2,-1

---------GCUUAUCAGACUGAUGUUGA--- 9

2,0

---------GCUUAUCAGACUGAUGUUGAC-- 12

2,1

---------GCUUAUCAGACUGAUGUUGACa--- 3

2,1

---CAACACCAGUCGAUGGGC--------- 17

0,-3

---CAACACCAGUCGAUGGGCU-------- 107

0,-2

---CAACACCAGUCGAUGGGCUG------- 53

0,-1

---CAACACCAGUCGAUGGGCUGU------ 112

0,0

---CAACACCAGUCGAUGGGCUGUa----- 10

0,0

---CAACACCAGUCGAUGGGCUGUaa---- 167

0,0

---CAACACCAGUCGAUGGGCUGUau---- 72

0,0

---CAACACCAGUCGAUGGGCUGUuu---- 22

0,0

---CAACACCAGUCGAUGGGCUGUC----- 170

0,1

---CAACACCAGUCGAUGGGCUGUCa---- 236

0,1

---CAACACCAGUCGAUGGGCUGUCaa--- 6

0,1

---CAACACCAGUCGAUGGGCUGUCau--- 31

0,1

---CAACACCAGUCGAUGGGCUGUCU---- 192

0,2

---CAACACCAGUCGAUGGGCUGUCUa--- 7

0,2
---CAACACCAGUCGAUGGGCUGUCUau--	2
0,2
---CAACACCAGUCGAUGGGCUGUCUu---	31
0,2
---CAACACCAGUCGAUGGGCUGUCUuu--	23
0,2
--AACACCAGUCGAUGGGCUGUC-----	3
1,1
--AACACCAGUCGAUGGGCUGUCa----	5
1,1
--AACACCAGUCGAUGGGCUGUCU----	26
1,2
--AACACCAGUCGAUGGGCUGUCUau--	2
1,2
--AACACCAGUCGAUGGGCUGUCUu---	3
1,2
--AACACCAGUCGAUGGGCUGUCUuu--	6
1,2

>hsa-mir-210	3p:66-87	SRX018957
acccggcagugccuccaggcgcagggcagccccugcccaccgcacacugcgcugccccagacccaCUGUGCGUGUGACAG
CGGCUGAucugugccugggcagcgcgaccc
....((..((((.((((((((((((..(((((.(((..((((((((.((.((((...))).).)).)))))).))).)))
.)))))..)))))))))))..))))..)).
--ACUGUGCGUGUGACAG
CGG---------------------------	4	-1,-4
--ACUGUGCGUGUGACAG
CGGC--------------------------	4	-1,-3
--ACUGUGCGUGUGACAG
CGGCU-------------------------	8	-1,-2
--ACUGUGCGUGUGACAG
CGGCUGA-----------------------	2	-1,0
---CUGUGCGUGUGACAG
CGG---------------------------	9	0,-4
---CUGUGCGUGUGACAG
CGGC--------------------------	18	0,-3
---CUGUGCGUGUGACAG

CGGCU------------------------ 162 0,-2
--CUGUGCGUGUGACAG
CGGCUGA---------------------- 55 0,0
--CUGUGCGUGUGACAG
CGGCUGAa--------------------- 6 0,0
--CUGUGCGUGUGACAG
CGGCUGAU--------------------- 5 0,1
--CUGUGCGUGUGACAG
CGGCUGAUa-------------------- 7 0,1
--CUGUGCGUGUGACAG
CGGCUGAUu-------------------- 8 0,1

>hsa-mir-2110 5p:8-29 SRX018957
cagggguUUGGGGAAACGGCCGCUGAGUGaggcgucggcuguguuucucaccgcggucuuuuccucccacucuug
(((((((..(((((((.((((((((((.((.(((.....))).)).))))..)))))).)))))))..)))))))
-------UUGGGGAAACGGCCGCUG-- 2
0,-4
-------UUGGGGAAACGGCCGCUGA--- 5
0,-3
-------UUGGGGAAACGGCCGCUGAG-- 8
0,-2
-------UUGGGGAAACGGCCGCUGAGU--- 8
0,-1
-------UUGGGGAAACGGCCGCUGAGUa-- 2
0,-1
-------UUGGGGAAACGGCCGCUGAGUu-- 4
0,-1
-------UUGGGGAAACGGCCGCUGAGUG-- 50
0,0
-------UUGGGGAAACGGCCGCUGAGUGu--- 63
0,0
-------UUGGGGAAACGGCCGCUGAGUGuu-- 7
0,0
-------UUGGGGAAACGGCCGCUGAGUGA--- 140
0,1
-------UUGGGGAAACGGCCGCUGAGUGAa-- 3
0,1
-------UUGGGGAAACGGCCGCUGAGUGAu-- 3

0,1
-------UUGGGGAAACGGCCGCUGAGUGAuu--- 2
0,1
-------UUGGGGAAACGGCCGCUGAGUGAG-- 14
0,2
-------UUGGGGAAACGGCCGCUGAGUGAGu--- 12
0,2
--------UGGGGAAACGGCCGCUGAGUGA--- 3
1,1
--------UGGGGAAACGGCCGCUGAGUGAa-- 2
1,1

>hsa-mir-212 3p:71-91 SRX018957
cggggcaccccgcccggacagcgcgccggcaccuuggcucuagacugcuuacugcccgggccgcccucagUAACAGUCUC
CAGUCACGGCCaccgacgccuggccccgcc
..................(((.(((.(((..((.(((((..((((((.((((((...(((...))).)))))))))))).
.))))).))...))).))))))........
------------------------------ACCUUGGCUCUAGACUGCUUAC----------------------------
------------------------------ 2

>hsa-mir-214 mi:30-51;MA:71-92 SRX018957
ggccuggcuggacagaguugucaugugucUGCCUGUCUACACUUGCUGUGCagaacauccgcucaccuguACAGCAGGCA
CAGACAGGCAGUcacaugacaacccagccu
.....(((((.....(((((((((((.(((((((((((...((((((((((((.............))))))))))))..
.))))))))))).))))))))))))))))).
-----------------------------UGCCUGUCUACACUUGCUG--------------------------------
------------------------------ 2 0,-3
-----------------------------UGCCUGUCUACACUUGCUGUG------------------------------
------------------------------ 3 0,-1
--GUACAGCAGGCA
CAGACAGa---------------------- 2 -2,-5
--GUACAGCAGGCA
CAGACAGG---------------------- 2 -2,-4
---UACAGCAGGCA
CAGACAG----------------------- 12 -1,-5
---UACAGCAGGCA
CAGACAGa---------------------- 2 -1,-5
---UACAGCAGGCA

CAGACAGaa--------------------- 3 -1,-5

---UACAGCAGGCA

CAGACAGu---------------------- 3 -1,-5

---UACAGCAGGCA

CAGACAGG---------------------- 73 -1,-4

---UACAGCAGGCA

CAGACAGGa--------------------- 15 -1,-4

---UACAGCAGGCA

CAGACAGGaa-------------------- 2 -1,-4

---UACAGCAGGCA

CAGACAGGC--------------------- 6 -1,-3

---UACAGCAGGCA

CAGACAGGCA-------------------- 55 -1,-2

---UACAGCAGGCA

CAGACAGGCAa------------------- 93 -1,-2

---UACAGCAGGCA

CAGACAGGCAaa------------------ 10 -1,-2

---UACAGCAGGCA

CAGACAGGCAau------------------ 25 -1,-2

---UACAGCAGGCA

CAGACAGGCAu------------------- 50 -1,-2

---UACAGCAGGCA

CAGACAGGCAuu------------------ 12 -1,-2

---UACAGCAGGCA

CAGACAGGCAG------------------- 33 -1,-1

---UACAGCAGGCA

CAGACAGGCAGa------------------ 35 -1,-1

---UACAGCAGGCA

CAGACAGGCAGU------------------ 118 -1,0

---UACAGCAGGCA

CAGACAGGCAGUa----------------- 22 -1,0

---UACAGCAGGCA

CAGACAGGCAGUau---------------- 5 -1,0

---UACAGCAGGCA

CAGACAGGCAGUu----------------- 22 -1,0

---UACAGCAGGCA

CAGACAGGCAGUuu---------------- 2 -1,0

--ACAGCAGGCA

```
CAGACAGG----------------------  64  0,-4
----------------------------------------------------------------------ACAGCAGGCA
CAGACAGGa---------------------  7   0,-4
----------------------------------------------------------------------ACAGCAGGCA
CAGACAGGaa--------------------  5   0,-4
----------------------------------------------------------------------ACAGCAGGCA
CAGACAGGC---------------------  10  0,-3
----------------------------------------------------------------------ACAGCAGGCA
CAGACAGGCA--------------------  48  0,-2
----------------------------------------------------------------------ACAGCAGGCA
CAGACAGGCAa-------------------  65  0,-2
----------------------------------------------------------------------ACAGCAGGCA
CAGACAGGCAaa------------------  16  0,-2
----------------------------------------------------------------------ACAGCAGGCA
CAGACAGGCAau------------------  8   0,-2
----------------------------------------------------------------------ACAGCAGGCA
CAGACAGGCAu-------------------  22  0,-2
----------------------------------------------------------------------ACAGCAGGCA
CAGACAGGCAuu------------------  2   0,-2
----------------------------------------------------------------------ACAGCAGGCA
CAGACAGGCAG-------------------  65  0,-1
----------------------------------------------------------------------ACAGCAGGCA
CAGACAGGCAGa------------------  52  0,-1
----------------------------------------------------------------------ACAGCAGGCA
CAGACAGGCAGaa-----------------  18  0,-1
----------------------------------------------------------------------ACAGCAGGCA
CAGACAGGCAGau-----------------  2   0,-1
----------------------------------------------------------------------ACAGCAGGCA
CAGACAGGCAGU------------------  181 0,0
----------------------------------------------------------------------ACAGCAGGCA
CAGACAGGCAGUa-----------------  233 0,0
----------------------------------------------------------------------ACAGCAGGCA
CAGACAGGCAGUaa----------------  16  0,0
----------------------------------------------------------------------ACAGCAGGCA
CAGACAGGCAGUau----------------  43  0,0
----------------------------------------------------------------------ACAGCAGGCA
CAGACAGGCAGUu-----------------  43  0,0
----------------------------------------------------------------------ACAGCAGGCA
```

CAGACAGGCAGUuu---------------- 9 0,0
---CAGCAGGCA
CAGACAGGCAGUu----------------- 2 1,0

>hsa-mir-215 5p:27-47 SRX018957
aucauucagaaaugguauacaggaaaAUGACCUAUGAAUUGACAGACaauauagcugaguuugucugucauuucuuuagg
ccaauauucuguaugacugugcuacuucaa
........(((.((((((((((((.(.((.((((.(((.((((((((((...........)))))))))).)))..))))
.)).).))))))......)))))).)))..
--------------------------AUGACCUAUGAAUUGACAG-----------------------------------
------------------------------ 806 0,-2
--------------------------AUGACCUAUGAAUUGACAGA----------------------------------
------------------------------ 2 0,-1
--------------------------AUGACCUAUGAAUUGACAGAC---------------------------------
------------------------------ 6 0,0
--------------------------AUGACCUAUGAAUUGACAGACA--------------------------------
------------------------------ 3 0,1
---------------------------UGACCUAUGAAUUGACAG-----------------------------------
------------------------------ 2000 1,-2
---------------------------UGACCUAUGAAUUGACAGu----------------------------------
------------------------------ 25 1,-2
---------------------------UGACCUAUGAAUUGACAGA----------------------------------
------------------------------ 50 1,-1
---------------------------UGACCUAUGAAUUGACAGAa---------------------------------
------------------------------ 4 1,-1
---------------------------UGACCUAUGAAUUGACAGAaa--------------------------------
------------------------------ 3 1,-1
---------------------------UGACCUAUGAAUUGACAGAC---------------------------------
------------------------------ 17 1,0
---------------------------UGACCUAUGAAUUGACAGACA--------------------------------
------------------------------ 137 1,1
---------------------------UGACCUAUGAAUUGACAGACAu-------------------------------
------------------------------ 14 1,1
---------------------------UGACCUAUGAAUUGACAGACAuu------------------------------
------------------------------ 9 1,1
---------------------------UGACCUAUGAAUUGACAGACAAa------------------------------
------------------------------ 3 1,2
----------------------------GACCUAUGAAUUGACAGA----------------------------------

------------------------------ 3   2,-1

>hsa-mir-217   5p:35-57   SRX018957
aguauaauuauuacauaguuuuugaugucgcagaUACUGCAUCAGGAACUGAUUGGAuaagaaucagucaccaucaguuc
cuaaugcauugccuucagcaucuaaacaag
.................((((..(((((.(.((.((.(((((.((((((((((.((((........))).).))))))))
)).))))).)).)).).))))).))))...
---------------------------------AUACUGCAUCAGGAACUGAUU--------------------------
------------------------------ 3   -1,-3
---------------------------------AUACUGCAUCAGGAACUGAUUG-------------------------
------------------------------ 14 -1,-2
----------------------------------UACUGCAUCAGGAACUGAUUGGA-----------------------
------------------------------ 5   0,0

>hsa-mir-218-1 MA:25-45;mi:68-89   SRX018957
gugauaauguagcgagauuuucugUUGUGCUUGAUCUAACCAUGUgguugcgagguaugaguaaaacAUGGUUCCGUCAA
GCACCAUGGaacgucacgcagcuuucuaca
(((..((.((.(((.(((.((((((.(((((((((..((((((((..((((.........)))).))))))))..)))))
)))).)))))).))).))).))))..))).
------------------------UUGUGCUUGAUCUAACCAUGUGa----------------------------------
------------------------------ 2   0,1

>hsa-mir-218-2 MA:25-45;mi:67-88   SRX018957
gaccagucgcugcggggcuuuccuUUGUGCUUGAUCUAACCAUGUgguggaacgauggaaacggaaCAUGGUUCUGUCAA
GCACCGCGgaaagcaccgugcucuccugca
...(((..((.((((.(((((((...((((((((..(((((((((..........((....))..))))))))..)))))
))))...))))))).))))))....)))..
------------------------UUGUGCUUGAUCUAACCAUGUGa----------------------------------
------------------------------ 2   0,1

>hsa-mir-219-1 5p:21-41;3p:62-83   SRX018957
ccgccccgggccgcggcuccUGAUUGUCCAAACGCAAUUCUcgagucuauggcuccggccgAGAGUUGAGUCUGGACGUC
CCGagccgccgcccccaaaccucgagcggg
((((.(.((((.(((((((..(((.(((((.((.(((((((((.(((.........)))))))))))).)).))))))))
..))))))).)))).........).)))).
--------------------UGAUUGUCCAAACGCAAUUC--
------------------------------ 2   0,-1
--------------------UGAUUGUCCAAACGCAAUUCUCG-------------------------------------

----------------------------- 3 0,2

>hsa-mir-219-2 5p:19-39;3p:62-83 SRX018957
acucaggggcuucgccacUGAUUGUCCAAACGCAAUUCUuguacgagucugcggccaaccgAGAAUUGUGGCUGGACAUC
UGUggcugagcuccggg
.(((.(((((((.(((((.(((.(((((..(((((((((((...(.((.....)))...)))))))))))..))))))))
.))))).))))))))))
------------------UGAUUGUCCAAACGCAAUUC--
----------------- 2 0,-1
---AGAAUUGUGGCUGGACAUC
U---------------- 2 0,-2

>hsa-mir-22 mi:15-36;MA:53-74 SRX018957
ggcugagccgcaguAGUUCUUCAGUGGCAAGCUUUAuguccugacccagcuaAAGCUGCCAGUUGAAGAACUGUugcccu
cugcc
(((.(((..((((((((((((((((((((.((((((.((.........)))))))))))))).)))))))))))))).))
).)))
--------------AGUUCUUCAGUGGCAAGCU---
----- 8 0,-3
--------------AGUUCUUCAGUGGCAAGCUU--
----- 9 0,-2
--------------AGUUCUUCAGUGGCAAGCUUU---
----- 98 0,-1
--------------AGUUCUUCAGUGGCAAGCUUUu--
----- 4 0,-1
--------------AGUUCUUCAGUGGCAAGCUUUA--
----- 79 0,0
--------------AGUUCUUCAGUGGCAAGCUUUAa---
----- 5 0,0
--------------AGUUCUUCAGUGGCAAGCUUUAU---
----- 6 0,1
--AAGCUGCCAGUUGAAGAA----------
----- 441 0,-4
--AAGCUGCCAGUUGAAGAAa---------
----- 3 0,-4
--AAGCUGCCAGUUGAAGAAau--------
----- 3 0,-4
--AAGCUGCCAGUUGAAGAAu---------

----- 2 0,-4

---AAGCUGCCAGUUGAAGAAuu--------

----- 21 0,-4

---AAGCUGCCAGUUGAAGAAC---------

----- 249 0,-3

---AAGCUGCCAGUUGAAGAACa--------

----- 183 0,-3

---AAGCUGCCAGUUGAAGAACaa-------

----- 29 0,-3

---AAGCUGCCAGUUGAAGAACau-------

----- 3 0,-3

---AAGCUGCCAGUUGAAGAACU--------

----- 1285 0,-2

---AAGCUGCCAGUUGAAGAACUa-------

----- 30 0,-2

---AAGCUGCCAGUUGAAGAACUaa------

----- 11 0,-2

---AAGCUGCCAGUUGAAGAACUau------

----- 16 0,-2

---AAGCUGCCAGUUGAAGAACUu-------

----- 19 0,-2

---AAGCUGCCAGUUGAAGAACUuu------

----- 23 0,-2

---AAGCUGCCAGUUGAAGAACUG-------

----- 125 0,-1

---AAGCUGCCAGUUGAAGAACUGa------

----- 18 0,-1

---AAGCUGCCAGUUGAAGAACUGU------

----- 1114 0,0

---AAGCUGCCAGUUGAAGAACUGUa-----

----- 24 0,0

---AAGCUGCCAGUUGAAGAACUGUaa----

----- 18 0,0

---AAGCUGCCAGUUGAAGAACUGUau----

----- 27 0,0

---AAGCUGCCAGUUGAAGAACUGUU-----

----- 44 0,1

---AAGCUGCCAGUUGAAGAACUGUUa----

```
-----  5  0,1
----------------------------------------------------AAGCUGCCAGUUGAAGAACUGUUu----
-----  73  0,1
----------------------------------------------------AAGCUGCCAGUUGAAGAACUGUUuu---
-----  3  0,1
-----------------------------------------------------AGCUGCCAGUUGAAGAACa--------
-----  2  1,-3
-----------------------------------------------------AGCUGCCAGUUGAAGAACU--------
-----  14  1,-2
-----------------------------------------------------AGCUGCCAGUUGAAGAACUGU------
-----  8  1,0

>hsa-mir-221   mi:25-46;MA:65-87   SRX018957
ugaacauccaggucuggggcaugaACCUGGCAUACAAUGUAGAUUUcuguguucguuaggcaacAGCUACAUUGUCUGCU
GGGUUUCaggcuaccuggaaacauguucuc
.((((((((((((....(((.((((((..(((.(((((((((....((((((((.....))).))))))))))))).))))..
.)).)))).))))))))))....))))).. 
-----------------------AACCUGGCAUACAAUGUAGAUUU----------------------------------
------------------------------  2  -1,0
------------------------ACCUGGCAUACAAUGUAG--------------------------------------
------------------------------  8  0,-4
------------------------ACCUGGCAUACAAUGUAGA-------------------------------------
------------------------------  2  0,-3
------------------------ACCUGGCAUACAAUGUAGAUU-----------------------------------
------------------------------  24  0,-1
------------------------ACCUGGCAUACAAUGUAGAUUU----------------------------------
------------------------------  124 0,0
------------------------ACCUGGCAUACAAUGUAGAUUUa---------------------------------
------------------------------  5  0,0
------------------------ACCUGGCAUACAAUGUAGAUUUu---------------------------------
------------------------------  2  0,0
------------------------ACCUGGCAUACAAUGUAGAUUUC---------------------------------
------------------------------  38  0,1
------------------------ACCUGGCAUACAAUGUAGAUUUCU--------------------------------
------------------------------  5  0,2
-------------------------CCUGGCAUACAAUGUAGAUUU----------------------------------
------------------------------  2  1,0
---------------------------------------------------------------CAGCUACAUUGUCUGCU
```

```
GGGUUUC----------------------  3    -1,0
---------------------------------------------------------------AGCUACAUUGUCUGCU
GGG--------------------------  2    0,-4
---------------------------------------------------------------AGCUACAUUGUCUGCU
GGGU-------------------------  2    0,-3
---------------------------------------------------------------AGCUACAUUGUCUGCU
GGGUaa-----------------------  4    0,-3
---------------------------------------------------------------AGCUACAUUGUCUGCU
GGGUau-----------------------  3    0,-3
---------------------------------------------------------------AGCUACAUUGUCUGCU
GGGUU------------------------  12   0,-2
---------------------------------------------------------------AGCUACAUUGUCUGCU
GGGUUa-----------------------  15   0,-2
---------------------------------------------------------------AGCUACAUUGUCUGCU
GGGUUaa----------------------  7    0,-2
---------------------------------------------------------------AGCUACAUUGUCUGCU
GGGUUau----------------------  13   0,-2
---------------------------------------------------------------AGCUACAUUGUCUGCU
GGGUUU-----------------------  213  0,-1
---------------------------------------------------------------AGCUACAUUGUCUGCU
GGGUUUa----------------------  32   0,-1
---------------------------------------------------------------AGCUACAUUGUCUGCU
GGGUUUaa---------------------  6    0,-1
---------------------------------------------------------------AGCUACAUUGUCUGCU
GGGUUUu----------------------  20   0,-1
---------------------------------------------------------------AGCUACAUUGUCUGCU
GGGUUUC----------------------  216  0,0
---------------------------------------------------------------AGCUACAUUGUCUGCU
GGGUUUCu---------------------  13   0,0
---------------------------------------------------------------AGCUACAUUGUCUGCU
GGGUUUCA---------------------  13   0,1
----------------------------------------------------------------GCUACAUUGUCUGCU
GGGUUau----------------------  2    1,-2
----------------------------------------------------------------GCUACAUUGUCUGCU
GGGUUU-----------------------  17   1,-1
----------------------------------------------------------------GCUACAUUGUCUGCU
GGGUUUC----------------------  10   1,0
```

>hsa-mir-222   mi:31-52;MA:69-89   SRX018957
gcugcuggaagguguagguacccucaauggCUCAGUAGCCAGUGUAGAUCCUgucuuucguaaucagcAGCUACAUCUGG
CUACUGGGUcucugauggcaucuucuagcu
...((((((((((......((.(((..((((((((((((((((((((...(((...........)))...))))).))))
))))))))))..))).))))))))))))).
--AGCUACAUCUGG
CUACUG------------------------ 54 0,-3
--AGCUACAUCUGG
CUACUGG----------------------- 79 0,-2
--AGCUACAUCUGG
CUACUGGG---------------------- 106 0,-1
--AGCUACAUCUGG
CUACUGGGa--------------------- 3   0,-1
--AGCUACAUCUGG
CUACUGGGU--------------------- 141 0,0
--AGCUACAUCUGG
CUACUGGGUa-------------------- 10 0,0
--AGCUACAUCUGG
CUACUGGGUaa------------------- 20 0,0
--AGCUACAUCUGG
CUACUGGGUau------------------- 21 0,0
--AGCUACAUCUGG
CUACUGGGUu-------------------- 6   0,0
--AGCUACAUCUGG
CUACUGGGUuu------------------- 3   0,0
--AGCUACAUCUGG
CUACUGGGUC-------------------- 631 0,1
--AGCUACAUCUGG
CUACUGGGUCa------------------- 8   0,1
--AGCUACAUCUGG
CUACUGGGUCaa------------------ 2   0,1
--AGCUACAUCUGG
CUACUGGGUCU------------------- 753 0,2
--AGCUACAUCUGG
CUACUGGGUCUa------------------ 9   0,2
--AGCUACAUCUGG
CUACUGGGUCUaa----------------- 3   0,2
--AGCUACAUCUGG

CUACUGGGUCUau----------------- 3   0,2
--AGCUACAUCUGG
CUACUGGGUCUu------------------ 31 0,2
--AGCUACAUCUGG
CUACUGGGUCUuu----------------- 19 0,2
--AGCUACAUCUGG
CUACUGGGUCUC------------------ 257 0,3
--AGCUACAUCUGG
CUACUGGGUCUCa----------------- 41 0,3
--AGCUACAUCUGG
CUACUGGGUCUCU----------------- 56 0,4
---GCUACAUCUGG
CUACUGGGUC-------------------- 4   1,1
---GCUACAUCUGG
CUACUGGGUCU------------------- 3   1,2
---GCUACAUCUGG
CUACUGGGUCUC------------------ 3   1,3

>hsa-mir-223   mi:26-47;MA:68-89   SRX018957
ccuggccuccugcagugccacgcucCGUGUAUUUGACAAGCUGAGUUggacacuccaugugguagagUGUCAGUUUGUCA
AAUACCCCAagugcggcacaugcuuaccag
.((((......(((((((.((((..(.((((((((((((((......((((((.........))))))))))))))))))
))))).)..)))).))))).)))...))))
-------------------------CGUGUAUUUGACAAGCUGAGUU---------------------------------
------------------------------ 2   0,0
-------------------------CGUGUAUUUGACAAGCUGAGUUa--------------------------------
------------------------------ 2   0,0
-------------------------CGUGUAUUUGACAAGCUGAGUUG--------------------------------
------------------------------ 2   0,1
---UGUCAGUUUGUCA
AAUACCCC---------------------- 3   0,-1
---UGUCAGUUUGUCA
AAUACCCCA--------------------- 5   0,0
---UGUCAGUUUGUCA
AAUACCCCAu-------------------- 4   0,0
---UGUCAGUUUGUCA
AAUACCCCAA-------------------- 6   0,1
---UGUCAGUUUGUCA

AAUACCCCAAu------------------ 2 0,1
--GUCAGUUUGUCA
AAUACCC---------------------- 2 1,-2
--GUCAGUUUGUCA
AAUACCCCA-------------------- 2 1,0
--GUCAGUUUGUCA
AAUACCCCAu------------------- 2 1,0
--GUCAGUUUGUCA
AAUACCCCAA------------------- 4 1,1
--GUCAGUUUGUCA
AAUACCCCAAa------------------ 3 1,1

>hsa-mir-224 MA:8-28;mi:53-75 SRX018957
gggcuuuCAAGUCACUAGUGGUUCCGUUuaguagaugauugugcauuguuucAAAAUGGUGCCCUAGUGACUACAaagcc
c
((((((..((((((((((.(((.((((((.(.(((..((.....))..)))).)))))).)))))))))))..)))))))
)
-------CAAGUCACUAGUGGUUCCGUUU---
- 5 0,1
-------CAAGUCACUAGUGGUUCCGUUUA--
- 3 0,2
-------CAAGUCACUAGUGGUUCCGUUUAG---
- 15 0,3
--AAAUGGUGCCCUAGUGAC----------
- 2 1,-4

>hsa-mir-2355 5p:11-31;3p:54-75 SRX018957
cagacgugucAUCCCCAGAUACAAUGGACAAuaugcuauuauaaucguauggcAUUGUCCUUGCUGUUUGGAGAUaauac
ugcugac
(((..((((.(((.((((((((((.(((((..(((((((.........))))))))))))))).))))))).))).))))
..)))..
----------AUCCCCAGAUACAAUGGACA--
------- 2 0,-1
----------AUCCCCAGAUACAAUGGACAA---
------- 2 0,0
----------AUCCCCAGAUACAAUGGACAAU--
------- 6 0,1

```
>hsa-mir-23a     mi:9-30;MA:45-65     SRX018957
ggccggcuGGGGUUCCUGGGGAUGGGAUUUgcuuccugucacaaAUCACAUUGCCAGGGAUUUCCaaccgacc
((.(((.((((((((((((.((((.((((((..........)))))).)))).))))))).))))).))).))
-------------------------------------------AAUCACAUUGCCAGGGAUUUC---------     2
    -1,-1
-------------------------------------------AAUCACAUUGCCAGGGAUUUCa--------     3
    -1,-1
-------------------------------------------AAUCACAUUGCCAGGGAUUUCC--------     3
    -1,0
--------------------------------------------AUCACAUUGCCAGGGAUU-----------     54
    0,-3
--------------------------------------------AUCACAUUGCCAGGGAUUU----------     319
    0,-2
--------------------------------------------AUCACAUUGCCAGGGAUUUa---------     96
    0,-2
--------------------------------------------AUCACAUUGCCAGGGAUUUaa--------     18
    0,-2
--------------------------------------------AUCACAUUGCCAGGGAUUUu---------     17
    0,-2
--------------------------------------------AUCACAUUGCCAGGGAUUUC---------     387
    0,-1
--------------------------------------------AUCACAUUGCCAGGGAUUUCa--------     133
    0,-1
--------------------------------------------AUCACAUUGCCAGGGAUUUCaa-------     2
    0,-1
--------------------------------------------AUCACAUUGCCAGGGAUUUCu--------     3
    0,-1
--------------------------------------------AUCACAUUGCCAGGGAUUUCC--------     256
    0,0
--------------------------------------------AUCACAUUGCCAGGGAUUUCCu-------     2
    0,0
--------------------------------------------AUCACAUUGCCAGGGAUUUCCA-------     919
    0,1
--------------------------------------------AUCACAUUGCCAGGGAUUUCCAu------     92
    0,1
--------------------------------------------AUCACAUUGCCAGGGAUUUCCAA------     105
    0,2
--------------------------------------------AUCACAUUGCCAGGGAUUUCCAAu-----     9
```

```
    0,2
---------------------------------------------UCACAUUGCCAGGGAUUUa---------    2
    1,-2
---------------------------------------------UCACAUUGCCAGGGAUUUC---------    5
    1,-1
---------------------------------------------UCACAUUGCCAGGGAUUUCa--------    5
    1,-1
---------------------------------------------UCACAUUGCCAGGGAUUUCC--------    2
    1,0
---------------------------------------------UCACAUUGCCAGGGAUUUCCA-------    3
    1,1
---------------------------------------------UCACAUUGCCAGGGAUUUCCAACu----    2
    1,3

>hsa-mir-23b    mi:20-41;MA:58-78   SRX018957
cucaggugcucuggcugcuUGGGUUCCUGGCAUGCUGAUUUgugacuuaagauuaaaAUCACAUUGCCAGGGAUUACCac
gcaaccacgaccuuggc
.((((.(.(((((.(((.(((((((((((((((.(((((((..............)))))))).))))))))))..))).
))).))).)))))))).
------------------UUGGGUUCCUGGCAUGCUGAU-----------------------------------------
-----------------    8   -1,-2
------------------UUGGGUUCCUGGCAUGCUGAUU----------------------------------------
-----------------    19  -1,-1
------------------UUGGGUUCCUGGCAUGCUGAUUa---------------------------------------
-----------------    7   -1,-1
------------------UUGGGUUCCUGGCAUGCUGAUUU---------------------------------------
-----------------    20  -1,0
------------------UUGGGUUCCUGGCAUGCUGAUUUa--------------------------------------
-----------------    3   -1,0
------------------UUGGGUUCCUGGCAUGCUGAUUUu--------------------------------------
-----------------    4   -1,0
-------------------UGGGUUCCUGGCAUGCUG-------------------------------------------
-----------------    2   0,-4
-------------------UGGGUUCCUGGCAUGCUGAU-----------------------------------------
-----------------    32  0,-2
-------------------UGGGUUCCUGGCAUGCUGAUa----------------------------------------
-----------------    7   0,-2
-------------------UGGGUUCCUGGCAUGCUGAUU----------------------------------------
```

-----------------   121 0,-1
-------------------UGGGUUCCUGGCAUGCUGAUUa---------------------------------------
-----------------   10   0,-1
-------------------UGGGUUCCUGGCAUGCUGAUUaa--------------------------------------
-----------------   4   0,-1
-------------------UGGGUUCCUGGCAUGCUGAUUau--------------------------------------
-----------------   2   0,-1
-------------------UGGGUUCCUGGCAUGCUGAUUU---------------------------------------
-----------------   503 0,0
-------------------UGGGUUCCUGGCAUGCUGAUUUa--------------------------------------
-----------------   55   0,0
-------------------UGGGUUCCUGGCAUGCUGAUUUaa-------------------------------------
-----------------   5   0,0
-------------------UGGGUUCCUGGCAUGCUGAUUUau-------------------------------------
-----------------   2   0,0
-------------------UGGGUUCCUGGCAUGCUGAUUUu--------------------------------------
-----------------   90   0,0
-------------------UGGGUUCCUGGCAUGCUGAUUUG--------------------------------------
-----------------   7   0,1
--------------------GGGUUCCUGGCAUGCUGA--
-----------------   4   1,-3
--------------------GGGUUCCUGGCAUGCUGAU---
-----------------   46   1,-2
--------------------GGGUUCCUGGCAUGCUGAUU--
-----------------   142 1,-1
--------------------GGGUUCCUGGCAUGCUGAUUa---------------------------------------
-----------------   12   1,-1
--------------------GGGUUCCUGGCAUGCUGAUUau--------------------------------------
-----------------   4   1,-1
--------------------GGGUUCCUGGCAUGCUGAUUU---------------------------------------
-----------------   787 1,0
--------------------GGGUUCCUGGCAUGCUGAUUUa--------------------------------------
-----------------   59   1,0
--------------------GGGUUCCUGGCAUGCUGAUUUaa-------------------------------------
-----------------   7   1,0
--------------------GGGUUCCUGGCAUGCUGAUUUau-------------------------------------
-----------------   7   1,0
--------------------GGGUUCCUGGCAUGCUGAUUUu--------------------------------------

----------------- 132 1,0
--------------------GGGUUCCUGGCAUGCUGAUUUuu---------------------------------------
----------------- 2 1,0
--------------------GGGUUCCUGGCAUGCUGAUUUG--
----------------- 4 1,1
---AAUCACAUUGCCAGGGAUUAC---
----------------- 2 -1,-1
---AAUCACAUUGCCAGGGAUUACa--
----------------- 2 -1,-1
---AAUCACAUUGCCAGGGAUUACC--
----------------- 5 -1,0
---AAUCACAUUGCCAGGGAUUACCA-
----------------- 5 -1,1
--AUCACAUUGCCAGGGAUU-----
----------------- 54 0,-3
--AUCACAUUGCCAGGGAUUA----
----------------- 253 0,-2
--AUCACAUUGCCAGGGAUUAa---
----------------- 73 0,-2
--AUCACAUUGCCAGGGAUUAaa--
----------------- 5 0,-2
--AUCACAUUGCCAGGGAUUAu---
----------------- 34 0,-2
--AUCACAUUGCCAGGGAUUAC---
----------------- 345 0,-1
--AUCACAUUGCCAGGGAUUACa--
----------------- 135 0,-1
--AUCACAUUGCCAGGGAUUACaa-
----------------- 13 0,-1
--AUCACAUUGCCAGGGAUUACau-
----------------- 2 0,-1
--AUCACAUUGCCAGGGAUUACu--
----------------- 20 0,-1
--AUCACAUUGCCAGGGAUUACC--
----------------- 405 0,0
--AUCACAUUGCCAGGGAUUACCu-
----------------- 13 0,0
--AUCACAUUGCCAGGGAUUACCA-

---------------- 1642 0,1
---AUCACAUUGCCAGGGAUUACCAa
---------------- 47 0,1
---AUCACAUUGCCAGGGAUUACCAa
u--------------- 10 0,1
---AUCACAUUGCCAGGGAUUACCAu
---------------- 272 0,1
---AUCACAUUGCCAGGGAUUACCAu
u--------------- 41 0,1
---AUCACAUUGCCAGGGAUUACCAC
---------------- 332 0,2
---AUCACAUUGCCAGGGAUUACCAC
a--------------- 74 0,2
---AUCACAUUGCCAGGGAUUACCAC
au-------------- 5 0,2
---AUCACAUUGCCAGGGAUUACCAC
u--------------- 866 0,2
---AUCACAUUGCCAGGGAUUACCAC
uu-------------- 5 0,2
---AUCACAUUGCCAGGGAUUACCAC
G--------------- 10 0,3
---AUCACAUUGCCAGGGAUUACCAC
Ga-------------- 3 0,3
--UCACAUUGCCAGGGAUUA----
---------------- 7 1,-2
--UCACAUUGCCAGGGAUUAaa--
---------------- 2 1,-2
--UCACAUUGCCAGGGAUUAC---
---------------- 6 1,-1
--UCACAUUGCCAGGGAUUACa--
---------------- 2 1,-1
--UCACAUUGCCAGGGAUUACC--
---------------- 7 1,0
--UCACAUUGCCAGGGAUUACCA-
---------------- 20 1,1
--UCACAUUGCCAGGGAUUACCAa
---------------- 2 1,1
--UCACAUUGCCAGGGAUUACCAu

----------------   7   1,1
---UCACAUUGCCAGGGAUUACCAu
u---------------   8   1,1
---UCACAUUGCCAGGGAUUACCAC
----------------   6   1,2
---UCACAUUGCCAGGGAUUACCAC
a---------------   16   1,2
---UCACAUUGCCAGGGAUUACCAC
au--------------   2   1,2
---UCACAUUGCCAGGGAUUACCAC
u---------------   64   1,2
---UCACAUUGCCAGGGAUUACCAC
uu--------------   16   1,2
--CACAUUGCCAGGGAUUACCAC
u---------------   2   2,2

>hsa-mir-24-1   mi:7-28;MA:44-65   SRX018957
cuccggUGCCUACUGAGCUGAUAUCAGUucucauuuuacacacUGGCUCAGUUCAGCAGGAACAGgag
((((.((.(((.(((((((((..(((((.............))))).))))))))).))).)).))))
----GGUGCCUACUGAGCUGAUAUCA--   2
   -2,-2
-----GUGCCUACUGAGCUGAUAUC---   3
   -1,-3
-----GUGCCUACUGAGCUGAUAUCA--   10
   -1,-2
------UGCCUACUGAGCUGAUAUCA--   5   0,-2
---UGGCUCAGUUCAGCAGGA-------   30   0,-4
---UGGCUCAGUUCAGCAGGAA------   46   0,-3
---UGGCUCAGUUCAGCAGGAAa-----   2   0,-3
---UGGCUCAGUUCAGCAGGAAC-----   32   0,-2
---UGGCUCAGUUCAGCAGGAACu----   2   0,-2
---UGGCUCAGUUCAGCAGGAACA----   162 0,-1
---UGGCUCAGUUCAGCAGGAACAa---   48   0,-1
---UGGCUCAGUUCAGCAGGAACAaa--   8   0,-1
---UGGCUCAGUUCAGCAGGAACAu---   18   0,-1
---UGGCUCAGUUCAGCAGGAACAuu--   13   0,-1
---UGGCUCAGUUCAGCAGGAACAG---   1343
   0,0

---UGGCUCAGUUCAGCAGGAACAGa--	45	0,0
---UGGCUCAGUUCAGCAGGAACAGaa-	3	0,0
---UGGCUCAGUUCAGCAGGAACAGau-	6	0,0
---UGGCUCAGUUCAGCAGGAACAGu--	2351
0,0
---UGGCUCAGUUCAGCAGGAACAGuu-	747	0,0
---UGGCUCAGUUCAGCAGGAACAGG--	2	0,1
---UGGCUCAGUUCAGCAGGAACAGGu-	4	0,1
--GGCUCAGUUCAGCAGGAA------	3	1,-3
--GGCUCAGUUCAGCAGGAACA----	6	1,-1
--GGCUCAGUUCAGCAGGAACAG---	35	1,0
--GGCUCAGUUCAGCAGGAACAGau-	2	1,0
--GGCUCAGUUCAGCAGGAACAGu--	59	1,0
--GGCUCAGUUCAGCAGGAACAGuu-	17	1,0
---GCUCAGUUCAGCAGGAACAG---	8	2,0
---GCUCAGUUCAGCAGGAACAGa--	8	2,0
---GCUCAGUUCAGCAGGAACAGu--	13	2,0
---GCUCAGUUCAGCAGGAACAGuu-	19	2,0
---GCUCAGUUCAGCAGGAACAGG--	14	2,1
---GCUCAGUUCAGCAGGAACAGGu-	3	2,1
---GCUCAGUUCAGCAGGAACAGGA-	3	2,2

>hsa-mir-24-2	mi:13-34;MA:50-71	SRX018957
cucugccucccgUGCCUACUGAGCUGAAACACAGuugguuuguguacacUGGCUCAGUUCAGCAGGAACAGgg
(((((..(((..(((..(((((((((..((((((.....))))))....)))))))))...)))))).)))))
-----------GUGCCUACUGAGCUGAAACAC---	7
-1,-2
-----------GUGCCUACUGAGCUGAAACACA--	12
-1,-1
-----------GUGCCUACUGAGCUGAAACACAG---------------------------------------	8
-1,0
---UGGCUCAGUUCAGCAGGA------	30
0,-4
---UGGCUCAGUUCAGCAGGAA-----	46
0,-3
---UGGCUCAGUUCAGCAGGAAa----	2
0,-3
---UGGCUCAGUUCAGCAGGAAC----	32

0,-2

---UGGCUCAGUUCAGCAGGAACu---   2

0,-2

---UGGCUCAGUUCAGCAGGAACA---   162

0,-1

---UGGCUCAGUUCAGCAGGAACAa--   48

0,-1

---UGGCUCAGUUCAGCAGGAACAaa-   8

0,-1

---UGGCUCAGUUCAGCAGGAACAu--   18

0,-1

---UGGCUCAGUUCAGCAGGAACAuu-   13

0,-1

---UGGCUCAGUUCAGCAGGAACAG--   1343

0,0

---UGGCUCAGUUCAGCAGGAACAGa-   45

0,0

---UGGCUCAGUUCAGCAGGAACAGaa   3

0,0

---UGGCUCAGUUCAGCAGGAACAGau   6

0,0

---UGGCUCAGUUCAGCAGGAACAGu-   2351

0,0

---UGGCUCAGUUCAGCAGGAACAGuu   747

0,0

---UGGCUCAGUUCAGCAGGAACAGG-   2

0,1

---UGGCUCAGUUCAGCAGGAACAGGu   4

0,1

--GGCUCAGUUCAGCAGGAA-----   3

1,-3

--GGCUCAGUUCAGCAGGAACA---   6

1,-1

--GGCUCAGUUCAGCAGGAACAG--   35

1,0

--GGCUCAGUUCAGCAGGAACAGau   2

1,0

--GGCUCAGUUCAGCAGGAACAGu-   59

1,0
--GGCUCAGUUCAGCAGGAACAGuu   17
1,0
---GCUCAGUUCAGCAGGAACAG--   8
2,0
---GCUCAGUUCAGCAGGAACAGa-   8
2,0
---GCUCAGUUCAGCAGGAACAGu-   13
2,0
---GCUCAGUUCAGCAGGAACAGuu   19
2,0
---GCUCAGUUCAGCAGGAACAGG-   14
2,1
---GCUCAGUUCAGCAGGAACAGGu   3
2,1

>hsa-mir-25 mi:14-34;MA:52-73   SRX018957
ggccaguguugagAGGCGGAGACUUGGGCAAUUGcuggacgcugcccugggCAUUGCACUUGUCUCGGUCUGAcagugcc
ggcc
((((.(..(((..((((.(((((..(.(((((.(((((.......))..)))))))).)..))))).))))..)))..).
))))
-------------AGGCGGAGACUUGGGCAAUUG--
----   11 0,0
-------------AGGCGGAGACUUGGGCAAUUGu---
----   2   0,0
-------------AGGCGGAGACUUGGGCAAUUGC---
----   38 0,1
-------------AGGCGGAGACUUGGGCAAUUGCU--
----   118 0,2
-------------AGGCGGAGACUUGGGCAAUUGCUa---
----   4   0,2
-------------AGGCGGAGACUUGGGCAAUUGCUu---
----   5   0,2
---CAUUGCACUUGUCUCGGU-----------
----   225 0,-4
---CAUUGCACUUGUCUCGGUC----------
----   450 0,-3
---CAUUGCACUUGUCUCGGUCU---------

----   486 0,-2
--CAUUGCACUUGUCUCGGUCUa--------
----   3   0,-2
--CAUUGCACUUGUCUCGGUCUuu-------
----   2   0,-2
--CAUUGCACUUGUCUCGGUCUG--------
----   464 0,-1
--CAUUGCACUUGUCUCGGUCUGu-------
----   156 0,-1
--CAUUGCACUUGUCUCGGUCUGuu------
----   4   0,-1
--CAUUGCACUUGUCUCGGUCUGA-------
----   1206   0,0
--CAUUGCACUUGUCUCGGUCUGAa------
----   21 0,0
--CAUUGCACUUGUCUCGGUCUGAaa-----
----   4   0,0
--CAUUGCACUUGUCUCGGUCUGAu------
----   12 0,0
---AUUGCACUUGUCUCGGUC----------
----   6   1,-3
---AUUGCACUUGUCUCGGUCU---------
----   19 1,-2
---AUUGCACUUGUCUCGGUCUG--------
----   19 1,-1
---AUUGCACUUGUCUCGGUCUGu-------
----   2   1,-1
---AUUGCACUUGUCUCGGUCUGA-------
----   42 1,0

>hsa-mir-26a-1 MA:10-31;mi:49-70   SRX018957
guggccucgUUCAAGUAAUCCAGGAUAGGCUgugcaggucccaaugggCCUAUUCUUGGUUACUUGCACGggacgc
(((.((((((.(((((((((.((((((((((.((.......))...)))))))))).))))))))).)))))).)))
---------UUCAAGUAAUCCAGGAUA---
    104 0,-4
---------UUCAAGUAAUCCAGGAUAa--
    27 0,-4
---------UUCAAGUAAUCCAGGAUAu--

5 0,-4

---------UUCAAGUAAUCCAGGAUAG--

591 0,-3

---------UUCAAGUAAUCCAGGAUAGa---

6 0,-3

---------UUCAAGUAAUCCAGGAUAGu---

7 0,-3

---------UUCAAGUAAUCCAGGAUAGG---

1783 0,-2

---------UUCAAGUAAUCCAGGAUAGGa--

6 0,-2

---------UUCAAGUAAUCCAGGAUAGGaa---

3 0,-2

---------UUCAAGUAAUCCAGGAUAGGau---

17 0,-2

---------UUCAAGUAAUCCAGGAUAGGu--

15 0,-2

---------UUCAAGUAAUCCAGGAUAGGuu---

28 0,-2

---------UUCAAGUAAUCCAGGAUAGGC--

2799 0,-1

---------UUCAAGUAAUCCAGGAUAGGCa---

786 0,-1

---------UUCAAGUAAUCCAGGAUAGGCaa--

526 0,-1

---------UUCAAGUAAUCCAGGAUAGGCau--

201 0,-1

---------UUCAAGUAAUCCAGGAUAGGCU---

10010 0,0

---------UUCAAGUAAUCCAGGAUAGGCUa--

1170 0,0

---------UUCAAGUAAUCCAGGAUAGGCUaa---

329 0,0

---------UUCAAGUAAUCCAGGAUAGGCUau---

1248 0,0

---------UUCAAGUAAUCCAGGAUAGGCUu--

400 0,0

---------UUCAAGUAAUCCAGGAUAGGCUuu---

5351 0,0

---------UUCAAGUAAUCCAGGAUAGGCUG--

4 0,1

---------UUCAAGUAAUCCAGGAUAGGCUGa---

2 0,1

---------UUCAAGUAAUCCAGGAUAGGCUGU---

9 0,2

----------UCAAGUAAUCCAGGAUAG--

14 1,-3

----------UCAAGUAAUCCAGGAUAGG---

22 1,-2

----------UCAAGUAAUCCAGGAUAGGC--

41 1,-1

----------UCAAGUAAUCCAGGAUAGGCa---

18 1,-1

----------UCAAGUAAUCCAGGAUAGGCaa--

20 1,-1

----------UCAAGUAAUCCAGGAUAGGCau--

3 1,-1

----------UCAAGUAAUCCAGGAUAGGCU---

185 1,0

----------UCAAGUAAUCCAGGAUAGGCUa--

13 1,0

----------UCAAGUAAUCCAGGAUAGGCUaa---

7 1,0

----------UCAAGUAAUCCAGGAUAGGCUau---

29 1,0

----------UCAAGUAAUCCAGGAUAGGCUu--

7 1,0

----------UCAAGUAAUCCAGGAUAGGCUuu---

31 1,0

-----------CAAGUAAUCCAGGAUAGG---

3 2,-2

-----------CAAGUAAUCCAGGAUAGGC--

4 2,-1

-----------CAAGUAAUCCAGGAUAGGCaa--

3 2,-1

-----------CAAGUAAUCCAGGAUAGGCU---

    7   2,0
-----------CAAGUAAUCCAGGAUAGGCUuu--
    4   2,0

>hsa-mir-26a-2 MA:14-35;mi:52-73   SRX018957
ggcuguggcuggaUUCAAGUAAUCCAGGAUAGGCUguuuccaucugugaggCCUAUUCUUGAUUACUUGUUUCuggaggc
agcu
((((((..(..((..(((((((((.((((((((((...............)))))))))).)))))))))..))..)..))
))))
-------------UUCAAGUAAUCCAGGAUA--
----   104 0,-4
-------------UUCAAGUAAUCCAGGAUAa---
----   27   0,-4
-------------UUCAAGUAAUCCAGGAUAu---
----   5   0,-4
-------------UUCAAGUAAUCCAGGAUAG---
----   591 0,-3
-------------UUCAAGUAAUCCAGGAUAGa--
----   6   0,-3
-------------UUCAAGUAAUCCAGGAUAGu--
----   7   0,-3
-------------UUCAAGUAAUCCAGGAUAGG--
----   1783   0,-2
-------------UUCAAGUAAUCCAGGAUAGGa---
----   6   0,-2
-------------UUCAAGUAAUCCAGGAUAGGaa--
----   3   0,-2
-------------UUCAAGUAAUCCAGGAUAGGau--
----   17   0,-2
-------------UUCAAGUAAUCCAGGAUAGGu---
----   15   0,-2
-------------UUCAAGUAAUCCAGGAUAGGuu--
----   28   0,-2
-------------UUCAAGUAAUCCAGGAUAGGC---
----   2799   0,-1
-------------UUCAAGUAAUCCAGGAUAGGCa--
----   786 0,-1
-------------UUCAAGUAAUCCAGGAUAGGCaa---

---- 526 0,-1

-------------UUCAAGUAAUCCAGGAUAGGCau--

---- 201 0,-1

-------------UUCAAGUAAUCCAGGAUAGGCU---

---- 10010 0,0

-------------UUCAAGUAAUCCAGGAUAGGCUa--

---- 1170 0,0

-------------UUCAAGUAAUCCAGGAUAGGCUaa---

---- 329 0,0

-------------UUCAAGUAAUCCAGGAUAGGCUau---

---- 1248 0,0

-------------UUCAAGUAAUCCAGGAUAGGCUu--

---- 400 0,0

-------------UUCAAGUAAUCCAGGAUAGGCUuu---

---- 5351 0,0

-------------UUCAAGUAAUCCAGGAUAGGCUG--

---- 4 0,1

-------------UUCAAGUAAUCCAGGAUAGGCUGa---

---- 2 0,1

-------------UUCAAGUAAUCCAGGAUAGGCUGU---

---- 9 0,2

--------------UCAAGUAAUCCAGGAUAG--

---- 14 1,-3

--------------UCAAGUAAUCCAGGAUAGG---

---- 22 1,-2

--------------UCAAGUAAUCCAGGAUAGGC--

---- 41 1,-1

--------------UCAAGUAAUCCAGGAUAGGCa---

---- 18 1,-1

--------------UCAAGUAAUCCAGGAUAGGCaa--

---- 20 1,-1

--------------UCAAGUAAUCCAGGAUAGGCau--

---- 3 1,-1

--------------UCAAGUAAUCCAGGAUAGGCU---

---- 185 1,0

--------------UCAAGUAAUCCAGGAUAGGCUa--

---- 13 1,0

--------------UCAAGUAAUCCAGGAUAGGCUaa---

---- 7 1,0
--------------UCAAGUAAUCCAGGAUAGGCUau--
---- 29 1,0
--------------UCAAGUAAUCCAGGAUAGGCUu---
---- 7 1,0
--------------UCAAGUAAUCCAGGAUAGGCUuu--
---- 31 1,0
---------------CAAGUAAUCCAGGAUAGG--
---- 3 2,-2
---------------CAAGUAAUCCAGGAUAGGC---
---- 4 2,-1
---------------CAAGUAAUCCAGGAUAGGCaa---
---- 3 2,-1
---------------CAAGUAAUCCAGGAUAGGCU--
---- 7 2,0
---------------CAAGUAAUCCAGGAUAGGCUuu--
---- 4 2,0

>hsa-mir-26b MA:12-32;mi:47-68 SRX018957
ccgggacccagUUCAAGUAAUUCAGGAUAGGUugugugcuguccagCCUGUUCUCCAUUACUUGGCUCggggaccgg
((((..((((((.((((((((..(((((((((((.(......))))))))))))..))))))))))).))).))))
-----------UUCAAGUAAUUCAGGAUA---
15 0,-3
-----------UUCAAGUAAUUCAGGAUAa--
8 0,-3
-----------UUCAAGUAAUUCAGGAUAaa---
2 0,-3
-----------UUCAAGUAAUUCAGGAUAG--
155 0,-2
-----------UUCAAGUAAUUCAGGAUAGG---
761 0,-1
-----------UUCAAGUAAUUCAGGAUAGGa--
4 0,-1
-----------UUCAAGUAAUUCAGGAUAGGau---
6 0,-1
-----------UUCAAGUAAUUCAGGAUAGGU--
1278 0,0
-----------UUCAAGUAAUUCAGGAUAGGUa---

672 0,0

-----------UUCAAGUAAUUCAGGAUAGGUaa--

593 0,0

-----------UUCAAGUAAUUCAGGAUAGGUau--

264 0,0

-----------UUCAAGUAAUUCAGGAUAGGUU---

6449 0,1

-----------UUCAAGUAAUUCAGGAUAGGUUa--

687 0,1

-----------UUCAAGUAAUUCAGGAUAGGUUaa---

18 0,1

-----------UUCAAGUAAUUCAGGAUAGGUUau---

18 0,1

-----------UUCAAGUAAUUCAGGAUAGGUUu--

1008 0,1

-----------UUCAAGUAAUUCAGGAUAGGUUuu---

12 0,1

-----------UUCAAGUAAUUCAGGAUAGGUUG--

35 0,2

------------UCAAGUAAUUCAGGAUAGG---

5 1,-1

------------UCAAGUAAUUCAGGAUAGGU--

10 1,0

------------UCAAGUAAUUCAGGAUAGGUa---

2 1,0

------------UCAAGUAAUUCAGGAUAGGUau--

2 1,0

------------UCAAGUAAUUCAGGAUAGGUU---

52 1,1

------------UCAAGUAAUUCAGGAUAGGUUa--

8 1,1

------------UCAAGUAAUUCAGGAUAGGUUu--

21 1,1

-------------CAAGUAAUUCAGGAUAGGU--

2 2,0

-------------CAAGUAAUUCAGGAUAGGUaa--

4 2,0

-------------CAAGUAAUUCAGGAUAGGUau--

2   2,0

-------------CAAGUAAUUCAGGAUAGGUU--

14   2,1

-------------CAAGUAAUUCAGGAUAGGUUa---

3   2,1

-------------CAAGUAAUUCAGGAUAGGUUu---

5   2,1

>hsa-mir-27a   mi:10-31;MA:51-71   SRX018957

cugaggagcAGGGCUUAGCUGCUUGUGAGCAggguccacaccaagucgugUUCACAGUGGCUAAGUUCCGCcccccag

(((.((.((.(((((((((..((.(((((((.((.(........))).)))))))))..))))))))).)).)).)))

---------AGGGCUUAGCUGCUUGUGAGCA---

4   0,0

---GUUCACAGUGGCUAAGUU-----------

8   -1,-4

---GUUCACAGUGGCUAAGUUC----------

36   -1,-3

--UUCACAGUGGCUAAGUUC----------

13598   0,-3

--UUCACAGUGGCUAAGUUCa---------

37   0,-3

--UUCACAGUGGCUAAGUUCaa--------

10   0,-3

--UUCACAGUGGCUAAGUUCC---------

139 0,-2

--UUCACAGUGGCUAAGUUCCa--------

4   0,-2

--UUCACAGUGGCUAAGUUCCuu-------

5   0,-2

--UUCACAGUGGCUAAGUUCCG--------

1130   0,-1

--UUCACAGUGGCUAAGUUCCGa-------

63   0,-1

--UUCACAGUGGCUAAGUUCCGu-------

62   0,-1

--UUCACAGUGGCUAAGUUCCGuu------

3   0,-1

--UUCACAGUGGCUAAGUUCCGC-------

35 0,0
---UUCACAGUGGCUAAGUUCCGCa------
5 0,0
---UUCACAGUGGCUAAGUUCCGCaa-----
19 0,0
---UUCACAGUGGCUAAGUUCCGCau-----
5 0,0
---UUCACAGUGGCUAAGUUCCGCC------
2 0,1
---UUCACAGUGGCUAAGUUCCGCCa-----
4 0,1
--UCACAGUGGCUAAGUUCCG--------
5 1,-1

>hsa-mir-27b mi:19-40;MA:61-81 SRX018957
accucucuaacaaggugcAGAGCUUAGCUGAUUGGUGAACagugauugguuuccgcuuugUUCACAGUGGCUAAGUUCUG
Caccugaagagaaggug
((((((((....((((((((((((((((((....((((((((....(((...)))..))))))))..))))))))))))
))))).)))).)))).
-----------------CAGAGCUUAGCUGAUUGGUGA--
----------------- 3 -1,-2
-----------------CAGAGCUUAGCUGAUUGGUGAA---
----------------- 4 -1,-1
-----------------CAGAGCUUAGCUGAUUGGUGAAC--
----------------- 2 -1,0
-----------------CAGAGCUUAGCUGAUUGGUGAACA---------------------------------------
----------------- 6 -1,1
------------------AGAGCUUAGCUGAUUGGU--
----------------- 4 0,-4
------------------AGAGCUUAGCUGAUUGGUG---
----------------- 13 0,-3
------------------AGAGCUUAGCUGAUUGGUGA--
----------------- 190 0,-2
------------------AGAGCUUAGCUGAUUGGUGAA---
----------------- 412 0,-1
------------------AGAGCUUAGCUGAUUGGUGAAa--
----------------- 2 0,-1
------------------AGAGCUUAGCUGAUUGGUGAAC--

----------------   16   0,0
------------------AGAGCUUAGCUGAUUGGUGAACA--------------------------------------
----------------   20   0,1
------------------AGAGCUUAGCUGAUUGGUGAACAG-------------------------------------
----------------   6   0,2
------------------AGAGCUUAGCUGAUUGGUGAACAGU------------------------------------
----------------   8   0,3
-------------------GAGCUUAGCUGAUUGGUGAAC---------------------------------------
----------------   2   1,0
-------------------GAGCUUAGCUGAUUGGUGAACA--------------------------------------
----------------   2   1,1
-------------------GAGCUUAGCUGAUUGGUGAACAu-------------------------------------
----------------   2   1,1
--GUUCACAGUGGCUAAGUU---
----------------   8   -1,-4
--GUUCACAGUGGCUAAGUUC--
----------------   36   -1,-3
--GUUCACAGUGGCUAAGUUCU-
----------------   4   -1,-2
---UUCACAGUGGCUAAGUUC--
----------------   13598   0,-3
---UUCACAGUGGCUAAGUUCa-
----------------   37   0,-3
---UUCACAGUGGCUAAGUUCaa
----------------   10   0,-3
---UUCACAGUGGCUAAGUUCU-
----------------   3044   0,-2
---UUCACAGUGGCUAAGUUCUa
----------------   34   0,-2
---UUCACAGUGGCUAAGUUCUa
a---------------   9   0,-2
---UUCACAGUGGCUAAGUUCUa
u---------------   2   0,-2
---UUCACAGUGGCUAAGUUCUu
----------------   16   0,-2
---UUCACAGUGGCUAAGUUCUG
----------------   143   0,-1
---UUCACAGUGGCUAAGUUCUG

a---------------   26 0,-1
---UUCACAGUGGCUAAGUUCUG
aa--------------   49 0,-1
---UUCACAGUGGCUAAGUUCUG
au--------------   2   0,-1
---UUCACAGUGGCUAAGUUCUG
u---------------   8   0,-1
---UUCACAGUGGCUAAGUUCUG
uu--------------   9   0,-1
---UUCACAGUGGCUAAGUUCUG
C---------------   205 0,0
---UUCACAGUGGCUAAGUUCUG
Cu--------------   11 0,0
---UUCACAGUGGCUAAGUUCUG
Cuu-------------   3   0,0
---UUCACAGUGGCUAAGUUCUG
CA--------------   243 0,1
---UUCACAGUGGCUAAGUUCUG
CAa-------------   132 0,1
---UUCACAGUGGCUAAGUUCUG
CAaa------------   3   0,1
---UUCACAGUGGCUAAGUUCUG
CAau------------   9   0,1
---UUCACAGUGGCUAAGUUCUG
CAu-------------   149 0,1
---UUCACAGUGGCUAAGUUCUG
CAuu------------   25 0,1
--UCACAGUGGCUAAGUUCU-
----------------   22 1,-2
--UCACAGUGGCUAAGUUCUG
CAu-------------   6   1,1
--UCACAGUGGCUAAGUUCUG
CAuu------------   2   1,1

>hsa-mir-28 5p:14-35;3p:54-75   SRX018957
gguccuugcccucAAGGAGCUCACAGUCUAUUGAGuuaccuuucugacuuuccCACUAGAUUGUGAGCUCCUGGAgggca
ggcacu
(((.(((((((((.((((((((((((((((.(((((((......)))))....)).)))))))))))))))).)))))))

)).)))
------------CAAGGAGCUCACAGUCUAUUG--
------ 2 -1,-2
-------------AAGGAGCUCACAGUCUAU--
------ 2 0,-4
-------------AAGGAGCUCACAGUCUAUU---
------ 10 0,-3
-------------AAGGAGCUCACAGUCUAUUa--
------ 7 0,-3
-------------AAGGAGCUCACAGUCUAUUG--
------ 70 0,-2
-------------AAGGAGCUCACAGUCUAUUGu---
------ 2 0,-2
-------------AAGGAGCUCACAGUCUAUUGA---
------ 121 0,-1
-------------AAGGAGCUCACAGUCUAUUGAa--
------ 5 0,-1
-------------AAGGAGCUCACAGUCUAUUGAaa---
------ 2 0,-1
-------------AAGGAGCUCACAGUCUAUUGAG--
------ 62 0,0
-------------AAGGAGCUCACAGUCUAUUGAGa---
------ 16 0,0
-------------AAGGAGCUCACAGUCUAUUGAGU---
------ 4 0,1
--------------AGGAGCUCACAGUCUAUUG--
------ 2 1,-2
--CACUAGAUUGUGAGCUCC---------
------ 3 0,-4
--CACUAGAUUGUGAGCUCCU--------
------ 4 0,-3
--CACUAGAUUGUGAGCUCCUG-------
------ 5 0,-2
--CACUAGAUUGUGAGCUCCUGa------
------ 7 0,-2
--CACUAGAUUGUGAGCUCCUGG------
------ 154 0,-1
--CACUAGAUUGUGAGCUCCUGGu-----

------ 5 0,-1

--CACUAGAUUGUGAGCUCCUGGA-----

------ 1821 0,0

--CACUAGAUUGUGAGCUCCUGGAa----

------ 675 0,0

--CACUAGAUUGUGAGCUCCUGGAaa---

------ 100 0,0

--CACUAGAUUGUGAGCUCCUGGAau---

------ 21 0,0

--CACUAGAUUGUGAGCUCCUGGAu----

------ 34 0,0

--CACUAGAUUGUGAGCUCCUGGAuu---

------ 4 0,0

--CACUAGAUUGUGAGCUCCUGGAG----

------ 5 0,1

--CACUAGAUUGUGAGCUCCUGGAGa---

------ 5 0,1

--CACUAGAUUGUGAGCUCCUGGAGu---

------ 3 0,1

---ACUAGAUUGUGAGCUCCUGG------

------ 5 1,-1

---ACUAGAUUGUGAGCUCCUGGA-----

------ 65 1,0

---ACUAGAUUGUGAGCUCCUGGAa----

------ 23 1,0

---ACUAGAUUGUGAGCUCCUGGAaa---

------ 4 1,0

---ACUAGAUUGUGAGCUCCUGGAau---

------ 2 1,0

---ACUAGAUUGUGAGCUCCUGGAG----

------ 14 1,1

---ACUAGAUUGUGAGCUCCUGGAGa---

------ 18 1,1

---ACUAGAUUGUGAGCUCCUGGAGaa--

------ 2 1,1

---ACUAGAUUGUGAGCUCCUGGAGu---

------ 2 1,1

--CUAGAUUGUGAGCUCCUGGA-----

------ 6 2,0
---CUAGAUUGUGAGCUCCUGGAa----
------ 3 2,0
---CUAGAUUGUGAGCUCCUGGAaa---
------ 2 2,0
---CUAGAUUGUGAGCUCCUGGAG----
------ 3 2,1
---CUAGAUUGUGAGCUCCUGGAGa---
------ 6 2,1
---CUAGAUUGUGAGCUCCUGGAGaa--
------ 6 2,1
---CUAGAUUGUGAGCUCCUGGAGu---
------ 2 2,1

>hsa-mir-299 5p:7-28;3p:39-60 SRX018957
aagaaaUGGUUUACCGUCCCACAUACAUuuugaauaugUAUGUGGGAUGGUAAACCGCUUcuu
(((((.((((((((((((((((((((((.......)))))))))))))))))))))).)))))
------UGGUUUACCGUCCCACAUACAU----------------------------------- 3 0,0
--------------------------------------UAUGUGGGAUGGUAAACCGCU---- 2 0,-1

>hsa-mir-29a mi:4-25;MA:42-63 SRX018957
augACUGAUUUCUUUUGGUGUUCAGagucaauauaauuuucUAGCACCAUCUGAAAUCGGUUAu
((((((((((((...(((((((.((((...........)))))))))))...))))))))))))
---ACUGAUUUCUUUUGGUGUUC--- 3 0,-2
---ACUGAUUUCUUUUGGUGUUCA-- 5 0,-1
---ACUGAUUUCUUUUGGUGUUCAGA-------------------------------------- 2 0,1
--CUAGCACCAUCUGAAAUC------ 4 -1,-5
--CUAGCACCAUCUGAAAUCG----- 4 -1,-4
--CUAGCACCAUCUGAAAUCGG---- 21 -1,-3
--CUAGCACCAUCUGAAAUCGGU--- 43 -1,-2
--CUAGCACCAUCUGAAAUCGGUa-- 10 -1,-2
--CUAGCACCAUCUGAAAUCGGUaa- 8 -1,-2
--CUAGCACCAUCUGAAAUCGGUU-- 402 -1,-1
--CUAGCACCAUCUGAAAUCGGUUu- 52 -1,-1
--CUAGCACCAUCUGAAAUCGGUUA- 269 -1,0
--CUAGCACCAUCUGAAAUCGGUUAa 5 -1,0
--CUAGCACCAUCUGAAAUCGGUUAU 10 -1,1
---UAGCACCAUCUGAAAUCG----- 49 0,-4

---UAGCACCAUCUGAAAUCGuu--- 5 0,-4
---UAGCACCAUCUGAAAUCGG---- 410 0,-3
---UAGCACCAUCUGAAAUCGGa--- 2 0,-3
---UAGCACCAUCUGAAAUCGGaa-- 2 0,-3
---UAGCACCAUCUGAAAUCGGU--- 370 0,-2
---UAGCACCAUCUGAAAUCGGUa-- 69 0,-2
---UAGCACCAUCUGAAAUCGGUaa- 211 0,-2
---UAGCACCAUCUGAAAUCGGUau- 19 0,-2
---UAGCACCAUCUGAAAUCGGUU-- 7199 0,-1
---UAGCACCAUCUGAAAUCGGUUu- 2053 0,-1
---UAGCACCAUCUGAAAUCGGUUuu 40 0,-1
---UAGCACCAUCUGAAAUCGGUUA- 29478 0,0
---UAGCACCAUCUGAAAUCGGUUAa 812 0,0
---UAGCACCAUCUGAAAUCGGUUAaa 3 0,0
---UAGCACCAUCUGAAAUCGGUUAU 574 0,1
---UAGCACCAUCUGAAAUCGGUUAUa 3 0,1
---UAGCACCAUCUGAAAUCGGUUAUu 4 0,1
--AGCACCAUCUGAAAUCGG---- 6 1,-3
--AGCACCAUCUGAAAUCGGUU-- 37 1,-1
--AGCACCAUCUGAAAUCGGUUu- 12 1,-1
--AGCACCAUCUGAAAUCGGUUA- 192 1,0
--AGCACCAUCUGAAAUCGGUUAa 4 1,0
--AGCACCAUCUGAAAUCGGUUAU 6 1,1
---GCACCAUCUGAAAUCGGUU-- 5 2,-1
---GCACCAUCUGAAAUCGGUUu- 3 2,-1
---GCACCAUCUGAAAUCGGUUA- 30 2,0

>hsa-mir-29b-1 mi:10-33;MA:51-73 SRX018957
cuucaggaaGCUGGUUUCAUAUGGUGGUUUAGAuuuaaauagugauugucUAGCACCAUUUGAAAUCAGUGUUcuugggg
g
(((((((((((((((((((.((((((..((((((.............)))))))))))).)))))))))).)))))))))
.
---------GCUGGUUUCAUAUGGUGGUUUA---
- 2 0,-2
---CUAGCACCAUUUGAAAUCAGU----------
- 8 -1,-3
---CUAGCACCAUUUGAAAUCAGUG---------
- 3 -1,-2

--UAGCACCAUUUGAAAUCA------------
-   10 0,-5
--UAGCACCAUUUGAAAUCAG-----------
-   42 0,-4
--UAGCACCAUUUGAAAUCAGU----------
-   826 0,-3
--UAGCACCAUUUGAAAUCAGUG---------
-   156 0,-2
--UAGCACCAUUUGAAAUCAGUGa--------
-   4   0,-2
--UAGCACCAUUUGAAAUCAGUGU--------
-   30 0,-1
--UAGCACCAUUUGAAAUCAGUGUa-------
-   14 0,-1
--UAGCACCAUUUGAAAUCAGUGUaa------
-   3   0,-1
--UAGCACCAUUUGAAAUCAGUGUU-------
-   33 0,0
---AGCACCAUUUGAAAUCAGU----------
-   11 1,-3
---AGCACCAUUUGAAAUCAGUG---------
-   2   1,-2
--GCACCAUUUGAAAUCAGU----------
-   2   2,-3

>hsa-mir-29b-2 mi:11-32;MA:52-74   SRX018957
cuucuggaagCUGGUUUCACAUGGUGGCUUAGauuuuuccaucuuuguaucUAGCACCAUUUGAAAUCAGUGUUuuagga
g
(((((((((((((((((.((((((.((.((((...............))))))))))))).)))))))))).)))))))))
)
---------GCUGGUUUCACAUGGUGGCUUAG--
-   2   -1,0
--CUAGCACCAUUUGAAAUCAGU---------
-   8   -1,-3
--CUAGCACCAUUUGAAAUCAGUG--------
-   3   -1,-2
---UAGCACCAUUUGAAAUCA-----------
-   10 0,-5

---UAGCACCAUUUGAAAUCAG----------
-	42	0,-4
---UAGCACCAUUUGAAAUCAGU---------
-	826	0,-3
---UAGCACCAUUUGAAAUCAGUG--------
-	156	0,-2
---UAGCACCAUUUGAAAUCAGUGa-------
-	4	0,-2
---UAGCACCAUUUGAAAUCAGUGU-------
-	30	0,-1
---UAGCACCAUUUGAAAUCAGUGUa------
-	14	0,-1
---UAGCACCAUUUGAAAUCAGUGUaa-----
-	3	0,-1
---UAGCACCAUUUGAAAUCAGUGUU------
-	33	0,0
---UAGCACCAUUUGAAAUCAGUGUUU-----
-	2	0,1
--AGCACCAUUUGAAAUCAGU---------
-	11	1,-3
--AGCACCAUUUGAAAUCAGUG--------
-	2	1,-2
---GCACCAUUUGAAAUCAGU---------
-	2	2,-3

>hsa-mir-29c	mi:16-37;MA:54-75	SRX018957
aucucuuacacaggcUGACCGAUUUCUCCUGGUGUUCagagucuguuuuugucUAGCACCAUUUGAAAUCGGUUAugaug
uaggggga
.(((((((((((...(((((((((((...((((((((((((.....)))))...)))))))...))))))))))))).))
))))))).
---------------UGACCGAUUUCUCCUGGUGU---
--------	3	0,-2
---------------UGACCGAUUUCUCCUGGUGUU--
--------	12	0,-1
---------------UGACCGAUUUCUCCUGGUGUUC---
--------	2	0,0
----------------GACCGAUUUCUCCUGGUGUU--
--------	5	1,-1

----------------GACCGAUUUCUCCUGGUGUUCA--
--------   7   1,1

-----------------ACCGAUUUCUCCUGGUGUUCA--
--------   5   2,1

-----------------ACCGAUUUCUCCUGGUGUUCAG---
--------   3   2,2

-----------------ACCGAUUUCUCCUGGUGUUCAGA--
--------   3   2,3

--CUAGCACCAUUUGAAAUCGGUUu-----
--------   3   -1,-1

--CUAGCACCAUUUGAAAUCGGUUA-----
--------   16   -1,0

---UAGCACCAUUUGAAAUCG---------
--------   36   0,-4

---UAGCACCAUUUGAAAUCGa--------
--------   2   0,-4

---UAGCACCAUUUGAAAUCGuu-------
--------   2   0,-4

---UAGCACCAUUUGAAAUCGG--------
--------   190   0,-3

---UAGCACCAUUUGAAAUCGGU-------
--------   107   0,-2

---UAGCACCAUUUGAAAUCGGUa------
--------   5   0,-2

---UAGCACCAUUUGAAAUCGGUaa-----
--------   41   0,-2

---UAGCACCAUUUGAAAUCGGUU------
--------   622   0,-1

---UAGCACCAUUUGAAAUCGGUUu-----
--------   410   0,-1

---UAGCACCAUUUGAAAUCGGUUuu----
--------   8   0,-1

---UAGCACCAUUUGAAAUCGGUUA-----
--------   8734   0,0

---UAGCACCAUUUGAAAUCGGUUAa----
--------   197   0,0

---UAGCACCAUUUGAAAUCGGUUAU----
--------   120   0,1

--AGCACCAUUUGAAAUCGGUU------
--------   3   1,-1
--AGCACCAUUUGAAAUCGGUUA-----
--------   39 1,0
---GCACCAUUUGAAAUCGGUUA-----
--------   9   2,0
---GCACCAUUUGAAAUCGGUUAUu---
--------   4   2,1

>hsa-mir-301a   3p:51-73   SRX018957
acugcuaacgaaugcucugacuuuauugcacuacuguacuuuacagcuagCAGUGCAAUAGUAUUGUCAAAGCaucugaa
agcagg
.(((((..((.(((((.((((.((((((((((.(((((...))))).....))))))))))....)))).))))).))..
))))).
-------------GCUCUGACUUUAUUGCACUACU---
------ 3
--CAGUGCAAUAGUAUUGUCAAAGCA------
------ 2   0,1

>hsa-mir-3065   5p:10-32;3p:50-72   SRX018957
cugcccucuUCAACAAAAUCACUGAUGCUGGAgucgccugagucaucacUCAGCACCAGGAUAUUGUUGGAGaggacag
(((.((((((((((((.(((.(((.(((((((.((....)).))......))))).)))))).)))))))))))).)))
---------UCAACAAAAUCACUGAUGCU--
    2   0,-3
---------UCAACAAAAUCACUGAUGCUG---
    4   0,-2

>hsa-mir-30a   MA:6-27;mi:47-68   SRX018957
gcgacUGUAAACAUCCUCGACUGGAAGcugugaagccacagaugggCUUUCAGUCGGAUGUUUGCAGCugc
(((.((((((((((((..((((((((((((((....))).....))))))))))))))))))))))).)))
----CUGUAAACAUCCUCGACUG-- 4
    -1,-4
----CUGUAAACAUCCUCGACUGGA-- 6
    -1,-2
----CUGUAAACAUCCUCGACUGGAA--- 6
    -1,-1
----CUGUAAACAUCCUCGACUGGAAG-- 8   -1,0
----CUGUAAACAUCCUCGACUGGAAGa--- 4   -1,0

----CUGUAAACAUCCUCGACUGGAAGC-- 37 -1,1
----CUGUAAACAUCCUCGACUGGAAGCa--- 4 -1,1
----CUGUAAACAUCCUCGACUGGAAGCU--- 38 -1,2
-----UGUAAACAUCCUCGACUG--- 104 0,-4
-----UGUAAACAUCCUCGACUGa-- 2 0,-4
-----UGUAAACAUCCUCGACUGG-- 135 0,-3
-----UGUAAACAUCCUCGACUGGu--- 7 0,-3
-----UGUAAACAUCCUCGACUGGuu-- 2 0,-3
-----UGUAAACAUCCUCGACUGGA--- 516 0,-2
-----UGUAAACAUCCUCGACUGGAu-- 2 0,-2
-----UGUAAACAUCCUCGACUGGAA-- 990 0,-1
-----UGUAAACAUCCUCGACUGGAAa--- 438 0,-1
-----UGUAAACAUCCUCGACUGGAAaa-- 21 0,-1
-----UGUAAACAUCCUCGACUGGAAau-- 6 0,-1
-----UGUAAACAUCCUCGACUGGAAu--- 12 0,-1
-----UGUAAACAUCCUCGACUGGAAG--- 4535
0,0
-----UGUAAACAUCCUCGACUGGAAGa-- 2752
0,0
-----UGUAAACAUCCUCGACUGGAAGaa--- 63 0,0
-----UGUAAACAUCCUCGACUGGAAGau--- 74 0,0
-----UGUAAACAUCCUCGACUGGAAGu-- 173 0,0
-----UGUAAACAUCCUCGACUGGAAGuu--- 11 0,0
-----UGUAAACAUCCUCGACUGGAAGC-- 9863
0,1
-----UGUAAACAUCCUCGACUGGAAGCa--- 2099
0,1
-----UGUAAACAUCCUCGACUGGAAGCaa-- 35 0,1
-----UGUAAACAUCCUCGACUGGAAGCau-- 60 0,1
-----UGUAAACAUCCUCGACUGGAAGCU--- 14495
0,2
-----UGUAAACAUCCUCGACUGGAAGCUa-- 128 0,2
-----UGUAAACAUCCUCGACUGGAAGCUu-- 473 0,2
-----UGUAAACAUCCUCGACUGGAAGCUG-- 5 0,3
------GUAAACAUCCUCGACUGGAA-- 6 1,-1
------GUAAACAUCCUCGACUGGAAa--- 2 1,-1
------GUAAACAUCCUCGACUGGAAG--- 40 1,0
------GUAAACAUCCUCGACUGGAAGa-- 9 1,0

------GUAAACAUCCUCGACUGGAAGC--- 86 1,1
------GUAAACAUCCUCGACUGGAAGCa-- 3 1,1
------GUAAACAUCCUCGACUGGAAGCau--- 3 1,1
------GUAAACAUCCUCGACUGGAAGCU-- 34 1,2
-------UAAACAUCCUCGACUGGAAGCU-- 6 2,2
--CUUUCAGUCGGAUGUUUG------- 3 0,-4
--CUUUCAGUCGGAUGUUUGC------ 3 0,-3
--CUUUCAGUCGGAUGUUUGCA----- 2 0,-2
--CUUUCAGUCGGAUGUUUGCAa---- 12 0,-2
--CUUUCAGUCGGAUGUUUGCAG---- 83 0,-1
--CUUUCAGUCGGAUGUUUGCAGa--- 4 0,-1
--CUUUCAGUCGGAUGUUUGCAGu--- 20 0,-1
--CUUUCAGUCGGAUGUUUGCAGC--- 143 0,0
--CUUUCAGUCGGAUGUUUGCAGCa-- 36 0,0
--CUUUCAGUCGGAUGUUUGCAGCaa- 6 0,0
--CUUUCAGUCGGAUGUUUGCAGCau- 42 0,0
--CUUUCAGUCGGAUGUUUGCAGCU-- 4 0,1
---UUUCAGUCGGAUGUUUGCA----- 3 1,-2
---UUUCAGUCGGAUGUUUGCAG---- 33 1,-1
---UUUCAGUCGGAUGUUUGCAGu--- 2 1,-1
---UUUCAGUCGGAUGUUUGCAGuu-- 5 1,-1
---UUUCAGUCGGAUGUUUGCAGC--- 60 1,0
---UUUCAGUCGGAUGUUUGCAGCa-- 12 1,0
---UUUCAGUCGGAUGUUUGCAGCaa- 5 1,0
---UUUCAGUCGGAUGUUUGCAGCau- 13 1,0
---UUUCAGUCGGAUGUUUGCAGCU-- 4 1,1
--UUCAGUCGGAUGUUUGCAGC--- 2 2,0

>hsa-mir-30b MA:17-38;mi:55-76 SRX018957
accaaguuucaguucaUGUAAACAUCCUACACUCAGCUguaauacauggauuggCUGGGAGGUGGAUGUUUACUUCagcu
gacuugga
.(((((..((((((...(((((((((.((.((((((((..............))))))))..)))))))))))...))))
))))))).
----------------UGUAAACAUCCUACACUC--
-------- 33 0,-4
----------------UGUAAACAUCCUACACUCAG--
-------- 9 0,-2
----------------UGUAAACAUCCUACACUCAGau--

-------- 2 0,-2

----------------UGUAAACAUCCUACACUCAGC---

-------- 41 0,-1

----------------UGUAAACAUCCUACACUCAGCa--

-------- 4 0,-1

----------------UGUAAACAUCCUACACUCAGCau---

-------- 5 0,-1

----------------UGUAAACAUCCUACACUCAGCU--

-------- 210 0,0

----------------UGUAAACAUCCUACACUCAGCUa---

-------- 27 0,0

----------------UGUAAACAUCCUACACUCAGCUau--

-------- 4 0,0

----------------UGUAAACAUCCUACACUCAGCUu---

-------- 14 0,0

----------------UGUAAACAUCCUACACUCAGCUuu--

-------- 4 0,0

-----------------GUAAACAUCCUACACUCAGCU--

-------- 2 1,0

--CUGGGAGGUGGAUGUUUA--------

-------- 15 0,-4

--CUGGGAGGUGGAUGUUUAa-------

-------- 2 0,-4

--CUGGGAGGUGGAUGUUUAu-------

-------- 2 0,-4

--CUGGGAGGUGGAUGUUUAC-------

-------- 14 0,-3

--CUGGGAGGUGGAUGUUUACa------

-------- 13 0,-3

--CUGGGAGGUGGAUGUUUACaa-----

-------- 3 0,-3

--CUGGGAGGUGGAUGUUUACU------

-------- 142 0,-2

--CUGGGAGGUGGAUGUUUACUa-----

-------- 13 0,-2

--CUGGGAGGUGGAUGUUUACUaa----

-------- 8 0,-2

--CUGGGAGGUGGAUGUUUACUU-----

--CUGGGAGGUGGAUGUUUACUUa----
--------	235	0,-1
--CUGGGAGGUGGAUGUUUACUUaa---
--------	75	0,-1
--CUGGGAGGUGGAUGUUUACUUau---
--------	37	0,-1
--CUGGGAGGUGGAUGUUUACUUu----
--------	5	0,-1
--CUGGGAGGUGGAUGUUUACUUC----
--------	25	0,-1
--CUGGGAGGUGGAUGUUUACUUCu---
--------	20	0,0
--CUGGGAGGUGGAUGUUUACUUCA---
--------	4	0,0
---UGGGAGGUGGAUGUUUACU------
--------	3	0,1
---UGGGAGGUGGAUGUUUACUU-----
--------	4	1,-2
---UGGGAGGUGGAUGUUUACUUa----
--------	11	1,-1
--------	2	1,-1

>hsa-mir-30c-1	MA:17-39;mi:56-77	SRX018957
accaugcuguagugugUGUAAACAUCCUACACUCUCAGCugugagcucaagguggCUGGGAGAGGGUUGUUUACUCCuuc
ugccaugga
.(((((..((((...(.(((((((.(((...(((((((((.((....))....))))))))).))).))))))).)...)
)))))))).
----------------UGUAAACAUCCUACACUC--
---------	33	0,-5
----------------UGUAAACAUCCUACACUCU---
---------	2	0,-4
----------------UGUAAACAUCCUACACUCUC--
---------	12	0,-3
----------------UGUAAACAUCCUACACUCUCA---
---------	36	0,-2
----------------UGUAAACAUCCUACACUCUCAG--
---------	70	0,-1
----------------UGUAAACAUCCUACACUCUCAGa---

--------- 28 0,-1

----------------UGUAAACAUCCUACACUCUCAGaa--

--------- 2 0,-1

----------------UGUAAACAUCCUACACUCUCAGau--

--------- 3 0,-1

----------------UGUAAACAUCCUACACUCUCAGu---

--------- 5 0,-1

----------------UGUAAACAUCCUACACUCUCAGC---

--------- 183 0,0

----------------UGUAAACAUCCUACACUCUCAGCa--

--------- 55 0,0

----------------UGUAAACAUCCUACACUCUCAGCaa---------------------------------------

--------- 8 0,0

----------------UGUAAACAUCCUACACUCUCAGCau---------------------------------------

--------- 7 0,0

----------------UGUAAACAUCCUACACUCUCAGCU--

--------- 648 0,1

----------------UGUAAACAUCCUACACUCUCAGCUa---------------------------------------

--------- 18 0,1

----------------UGUAAACAUCCUACACUCUCAGCUu---------------------------------------

--------- 24 0,1

-----------------GUAAACAUCCUACACUCUCAGCU--

--------- 4 1,1

---CUGGGAGAGGGUUGUUUA-------

--------- 7 0,-4

---CUGGGAGAGGGUUGUUUAa------

--------- 2 0,-4

---CUGGGAGAGGGUUGUUUAC------

--------- 5 0,-3

---CUGGGAGAGGGUUGUUUACa-----

--------- 11 0,-3

---CUGGGAGAGGGUUGUUUACU-----

--------- 264 0,-2

---CUGGGAGAGGGUUGUUUACUa----

--------- 18 0,-2

---CUGGGAGAGGGUUGUUUACUaa---

--------- 13 0,-2

---CUGGGAGAGGGUUGUUUACUau---

--------- 4 0,-2

---CUGGGAGAGGGUUGUUUACUu----

--------- 7 0,-2

---CUGGGAGAGGGUUGUUUACUC----

--------- 62 0,-1

---CUGGGAGAGGGUUGUUUACUCa---

--------- 6 0,-1

---CUGGGAGAGGGUUGUUUACUCaa--

--------- 3 0,-1

---CUGGGAGAGGGUUGUUUACUCC---

--------- 6 0,0

---CUGGGAGAGGGUUGUUUACUCCa--

--------- 12 0,0

---CUGGGAGAGGGUUGUUUACUCCau-

--------- 5 0,0

---CUGGGAGAGGGUUGUUUACUCCU--

--------- 16 0,1

--UGGGAGAGGGUUGUUUAC------

--------- 3 1,-3

--UGGGAGAGGGUUGUUUACau----

--------- 2 1,-3

--UGGGAGAGGGUUGUUUACU-----

--------- 33 1,-2

--UGGGAGAGGGUUGUUUACUaa---

--------- 3 1,-2

--UGGGAGAGGGUUGUUUACUC----

--------- 10 1,-1

--UGGGAGAGGGUUGUUUACUCa---

--------- 4 1,-1

--UGGGAGAGGGUUGUUUACUCaa--

--------- 2 1,-1

--UGGGAGAGGGUUGUUUACUCC---

--------- 3 1,0

--UGGGAGAGGGUUGUUUACUCCa--

--------- 4 1,0

--UGGGAGAGGGUUGUUUACUCCau-

--------- 3 1,0

--UGGGAGAGGGUUGUUUACUCCU--

--------- 6 1,1

>hsa-mir-30c-2 MA:7-29;mi:47-68 SRX018957
agauacUGUAAACAUCCUACACUCUCAGCuguggaaaguaagaaagCUGGGAGAAGGCUGUUUACUCUuucu
(((....(((((((.(((...(((((((((.............))))))))).))).)))))))....)))
------UGUAAACAUCCUACACUC-- 33
0,-5
------UGUAAACAUCCUACACUCU--- 2
0,-4
------UGUAAACAUCCUACACUCUC-- 12
0,-3
------UGUAAACAUCCUACACUCUCA--- 36
0,-2
------UGUAAACAUCCUACACUCUCAG-- 70
0,-1
------UGUAAACAUCCUACACUCUCAGa--- 28
0,-1
------UGUAAACAUCCUACACUCUCAGaa-- 2
0,-1
------UGUAAACAUCCUACACUCUCAGau-- 3
0,-1
------UGUAAACAUCCUACACUCUCAGu--- 5
0,-1
------UGUAAACAUCCUACACUCUCAGC--- 183
0,0
------UGUAAACAUCCUACACUCUCAGCa-- 55
0,0
------UGUAAACAUCCUACACUCUCAGCaa--- 8
0,0
------UGUAAACAUCCUACACUCUCAGCau--- 7
0,0
------UGUAAACAUCCUACACUCUCAGCU-- 648
0,1
------UGUAAACAUCCUACACUCUCAGCUa--- 18
0,1
------UGUAAACAUCCUACACUCUCAGCUu--- 24
0,1
-------GUAAACAUCCUACACUCUCAGCU-- 4

1,1

--CUGGGAGAAGGCUGUUUA-------- 16

0,-4

--CUGGGAGAAGGCUGUUUAa------- 4

0,-4

--CUGGGAGAAGGCUGUUUAC------- 11

0,-3

--CUGGGAGAAGGCUGUUUACa------ 8

0,-3

--CUGGGAGAAGGCUGUUUACU------ 410

0,-2

--CUGGGAGAAGGCUGUUUACUa----- 22

0,-2

--CUGGGAGAAGGCUGUUUACUaa---- 5

0,-2

--CUGGGAGAAGGCUGUUUACUau---- 2

0,-2

--CUGGGAGAAGGCUGUUUACUu----- 2

0,-2

--CUGGGAGAAGGCUGUUUACUC----- 423

0,-1

--CUGGGAGAAGGCUGUUUACUCa---- 24

0,-1

--CUGGGAGAAGGCUGUUUACUCU---- 87

0,0

--CUGGGAGAAGGCUGUUUACUCUU--- 4

0,1

---UGGGAGAAGGCUGUUUACU------ 23

1,-2

---UGGGAGAAGGCUGUUUACUC----- 34

1,-1

---UGGGAGAAGGCUGUUUACUCU---- 9

1,0

---UGGGAGAAGGCUGUUUACUCUU--- 8

1,1

--GGGAGAAGGCUGUUUACU------ 2

2,-2

>hsa-mir-30d MA:6-27;mi:46-67 SRX018957

guuguUGUAAACAUCCCCGACUGGAAGcuguaagacacagcuaagCUUUCAGUCAGAUGUUUGCUGCuac

...((.(((((((((...(((((((((((...((......)).))))))))))).))))))))).))...

----UUGUAAACAUCCCCGACUGGAAGC-- 5 -1,1

----UUGUAAACAUCCCCGACUGGAAGCU--- 5 -1,2

-----UGUAAACAUCCCCGACUG--- 84 0,-4

-----UGUAAACAUCCCCGACUGa-- 3 0,-4

-----UGUAAACAUCCCCGACUGG-- 177 0,-3

-----UGUAAACAUCCCCGACUGGA--- 359 0,-2

-----UGUAAACAUCCCCGACUGGAu-- 2 0,-2

-----UGUAAACAUCCCCGACUGGAA-- 1500

0,-1

-----UGUAAACAUCCCCGACUGGAAa--- 654 0,-1

-----UGUAAACAUCCCCGACUGGAAaa-- 42 0,-1

-----UGUAAACAUCCCCGACUGGAAau-- 10 0,-1

-----UGUAAACAUCCCCGACUGGAAu--- 60 0,-1

-----UGUAAACAUCCCCGACUGGAAuu-- 3 0,-1

-----UGUAAACAUCCCCGACUGGAAG--- 2348

0,0

-----UGUAAACAUCCCCGACUGGAAGa-- 1258

0,0

-----UGUAAACAUCCCCGACUGGAAGaa--- 182 0,0

-----UGUAAACAUCCCCGACUGGAAGau--- 131 0,0

-----UGUAAACAUCCCCGACUGGAAGu-- 164 0,0

-----UGUAAACAUCCCCGACUGGAAGuu--- 19 0,0

-----UGUAAACAUCCCCGACUGGAAGC-- 6528

0,1

-----UGUAAACAUCCCCGACUGGAAGCa--- 1947

0,1

-----UGUAAACAUCCCCGACUGGAAGCaa-- 58 0,1

-----UGUAAACAUCCCCGACUGGAAGCau-- 53 0,1

-----UGUAAACAUCCCCGACUGGAAGCU--- 36201

0,2

-----UGUAAACAUCCCCGACUGGAAGCUa-- 551 0,2

-----UGUAAACAUCCCCGACUGGAAGCUu-- 1458

0,2

-----UGUAAACAUCCCCGACUGGAAGCUG-- 8 0,3

------GUAAACAUCCCCGACUGGA--- 4 1,-2

------GUAAACAUCCCCGACUGGAA-- 6 1,-1
------GUAAACAUCCCCGACUGGAAa--- 5 1,-1
------GUAAACAUCCCCGACUGGAAG--- 21 1,0
------GUAAACAUCCCCGACUGGAAGa-- 12 1,0
------GUAAACAUCCCCGACUGGAAGu-- 4 1,0
------GUAAACAUCCCCGACUGGAAGC-- 66 1,1
------GUAAACAUCCCCGACUGGAAGCa--- 16 1,1
------GUAAACAUCCCCGACUGGAAGCU--- 277 1,2
------GUAAACAUCCCCGACUGGAAGCUa-- 8 1,2
------GUAAACAUCCCCGACUGGAAGCUu-- 11 1,2
------GUAAACAUCCCCGACUGGAAGCUuu--------------------------------------- 12 1,2
-------UAAACAUCCCCGACUGGAAGC-- 2 2,1
-------UAAACAUCCCCGACUGGAAGCU--- 9 2,2

>hsa-mir-30e MA:17-38;mi:59-80 SRX018957
gggcagucuuugcuacUGUAAACAUCCUUGACUGGAAGcuguaagguguucagaggagCUUUCAGUCGGAUGUUUACAGC
ggcaggcugcca
.(((((((..((..((((((((((((..((((((((((((.(...(....)...).))))))))))))))))))))))).
.)))))))))).
---------------CUGUAAACAUCCUUGACUGGAAa---
----------- 3 -1,-1
---------------CUGUAAACAUCCUUGACUGGAAG---
----------- 3 -1,0
---------------CUGUAAACAUCCUUGACUGGAAGC--
----------- 14 -1,1
---------------CUGUAAACAUCCUUGACUGGAAGCa---
----------- 2 -1,1
---------------CUGUAAACAUCCUUGACUGGAAGCU---
----------- 5 -1,2
----------------UGUAAACAUCCUUGACUG---
----------- 13 0,-4
----------------UGUAAACAUCCUUGACUGG--
----------- 25 0,-3
----------------UGUAAACAUCCUUGACUGGA---
----------- 26 0,-2
----------------UGUAAACAUCCUUGACUGGAA--
----------- 44 0,-1
----------------UGUAAACAUCCUUGACUGGAAa---

-----------   28   0,-1
----------------UGUAAACAUCCUUGACUGGAAaa---------------------------------------
-----------   3   0,-1
----------------UGUAAACAUCCUUGACUGGAAu--
-----------   5   0,-1
----------------UGUAAACAUCCUUGACUGGAAG--
-----------   255 0,0
----------------UGUAAACAUCCUUGACUGGAAGa---------------------------------------
-----------   262 0,0
----------------UGUAAACAUCCUUGACUGGAAGaa--------------------------------------
-----------   66   0,0
----------------UGUAAACAUCCUUGACUGGAAGau--------------------------------------
-----------   15   0,0
----------------UGUAAACAUCCUUGACUGGAAGu---------------------------------------
-----------   11   0,0
----------------UGUAAACAUCCUUGACUGGAAGC---------------------------------------
-----------   1153   0,1
----------------UGUAAACAUCCUUGACUGGAAGCa--------------------------------------
-----------   246 0,1
----------------UGUAAACAUCCUUGACUGGAAGCau-------------------------------------
-----------   2   0,1
----------------UGUAAACAUCCUUGACUGGAAGCU--------------------------------------
-----------   2058   0,2
----------------UGUAAACAUCCUUGACUGGAAGCUa-------------------------------------
-----------   59   0,2
----------------UGUAAACAUCCUUGACUGGAAGCUu-------------------------------------
-----------   468 0,2
----------------UGUAAACAUCCUUGACUGGAAGCUG-------------------------------------
-----------   8   0,3
-----------------GUAAACAUCCUUGACUGGAAG--
-----------   11   1,0
-----------------GUAAACAUCCUUGACUGGAAGa---------------------------------------
-----------   16   1,0
-----------------GUAAACAUCCUUGACUGGAAGaa--------------------------------------
-----------   3   1,0
-----------------GUAAACAUCCUUGACUGGAAGC---------------------------------------
-----------   39   1,1
-----------------GUAAACAUCCUUGACUGGAAGCa--------------------------------------

------------ 16 1,1
-----------------GUAAACAUCCUUGACUGGAAGCU--
------------ 57 1,2
-----------------GUAAACAUCCUUGACUGGAAGCUa---------------------------------------
------------ 4 1,2
-----------------GUAAACAUCCUUGACUGGAAGCUu---------------------------------------
------------ 9 1,2
-----------------GUAAACAUCCUUGACUGGAAGCUuu--------------------------------------
------------ 2 1,2
-----------------GUAAACAUCCUUGACUGGAAGCUG---------------------------------------
------------ 4 1,3
------------------UAAACAUCCUUGACUGGAAGCU--
------------ 6 2,2
--CUUUCAGUCGGAUGUUUA----
------------ 4 0,-4
--CUUUCAGUCGGAUGUUUAC---
------------ 4 0,-3
--CUUUCAGUCGGAUGUUUACA--
------------ 5 0,-2
--CUUUCAGUCGGAUGUUUACAa-
------------ 2 0,-2
--CUUUCAGUCGGAUGUUUACAG-
------------ 28 0,-1
--CUUUCAGUCGGAUGUUUACAGa
------------ 8 0,-1
--CUUUCAGUCGGAUGUUUACAGu
------------ 47 0,-1
--CUUUCAGUCGGAUGUUUACAGC
------------ 83 0,0
--CUUUCAGUCGGAUGUUUACAGC
a----------- 193 0,0
--CUUUCAGUCGGAUGUUUACAGC
aa---------- 5 0,0
--CUUUCAGUCGGAUGUUUACAGC
au---------- 8 0,0
--CUUUCAGUCGGAUGUUUACAGC
u----------- 4 0,0
---UUUCAGUCGGAUGUUUACA--

----------- 3 1,-2
--UUUCAGUCGGAUGUUUACAGa
----------- 2 1,-1
--UUUCAGUCGGAUGUUUACAGu
----------- 2 1,-1
--UUUCAGUCGGAUGUUUACAGC
----------- 7 1,0
--UUUCAGUCGGAUGUUUACAGC
a---------- 9 1,0
--UUUCAGUCGGAUGUUUACAGC
au--------- 3 1,0

>hsa-mir-31 MA:8-28;mi:44-65 SRX018957
ggagaggAGGCAAGAUGCUGGCAUAGCUguugaacugggaaccUGCUAUGCCAACAUAUUGCCAUcuuucc
((((((..(((((.(((.(((((((((.(((........)))..))))))))).))).)))))..))))))
-------AGGCAAGAUGCUGGCAUAGCU--- 14 0,0
-------AGGCAAGAUGCUGGCAUAGCUG-- 3 0,1
-------AGGCAAGAUGCUGGCAUAGCUGU--- 4 0,2

>hsa-mir-3117 3p:46-66 SRX018957
cccuaaagggccagacacuauacgagucauauaagggaaggcauuAUAGGACUCAUAUAGUGCCAGguguuuguggg
((((((((.(((.(.(((((((.(((((.(((((........))))).))))).))))))).).))).))))).)))
---AUAGGACUCAUAUAGUGCCAG-----------
2 0,0

>hsa-mir-3124 5p:7-27 SRX018957
gcgggcUUCGCGGGCGAAGGCAAAGUCgauuuccaaaagugacuuuccucacucccgugaagucggc
((.(((((((((((.(.(((.((((((.((((....)))))))))))))..).))))))))))).))
------UUCGCGGGCGAAGGCAAAGUCG--------------------------------------- 2 0,1

>hsa-mir-3127 5p:11-33 SRX018957
ggccaggcccAUCAGGGCUUGUGGAAUGGGAAGgagaagggacgcuuccccuucugcaggccugcugggguguggcu
(((((.(((((...(((((((..(((.(((((((........).)))))).)))..)))))))..))))).)))))
----------AUCAGGGCUUGUGGAAUGGGAA--
2 0,-1

>hsa-mir-3129 5p:9-30 SRX018957
guacuuggGCAGUAGUGUAGAGAUUGGUUUgccuguuaaugaauucaaacuaaucucuacacugcugcccaagagc

...((((((((((((((((((((((((((((...(((....))).))))))))))))))))))))))))))))...
----------AGUAGUGUAGAGAUUGGUUUG---
3 2,1

>hsa-mir-3130-1 5p:13-33;3p:44-64 SRX018957
cuugucaugucuUACCCAGUCUCCGGUGCAGCCuguugucaagGCUGCACCGGAGACUGGGUAAgacaugacaag
((((((((((((((((((((((((((((((((((.......))))))))))))))))))))))))))))))))))
---GCUGCACCGGAGACUGGGUA------------ 3
0,-1
---GCUGCACCGGAGACUGGGUAA----------- 25
0,0

>hsa-mir-3130-2 5p:13-33;3p:44-64 SRX018957
cuugucaugucuUACCCAGUCUCCGGUGCAGCCuugacaacagGCUGCACCGGAGACUGGGUAAgacaugacaag
((((((((((((((((((((((((((((((((((.......))))))))))))))))))))))))))))))))))
---GCUGCACCGGAGACUGGGUA------------ 3
0,-1
---GCUGCACCGGAGACUGGGUAA----------- 25
0,0

>hsa-mir-3140 3p:60-81 SRX018957
ccucuugagguaccugaauuaccaaaagcuuuauguauucugaaguuauugaaaauaagAGCUUUUGGGAAUUCAGGUAG
Uucaggagug
.((((((((.((((((((((.(((((((((((.(((.(((..........))).))))))))))))))).)))))))))).
))))))))..
---AGAGCUUUUGGGAAUUCAGGUAa
---------- 2 -2,-2

>hsa-mir-3154 3p:54-75 SRX018957
ggccccuccuucucagccccagcucccgcucaccccugccacgucaaaggaggCAGAAGGGGAGUUGGGAGCAGAgaggg
gacc
((((((((..(((..((((((((((((.((.....(((((.(......)..))))).))))))))))))).))))))))))
).))
---CAGAAGGGGAGUUGGGAGCAG------
---- 6 0,-1

>hsa-mir-3163 5p:10-31 SRX018957
uuccucaucUAUAAAAUGAGGGCAGUAAGACcuuccuuccuugucuuacuacccccauuuuauagaugaggaa

.((((((((((((((((.(((.((((((((...........)))))))).))).)))))))))))))))).
---------UAUAAAAUGAGGGCAGUAAGA---   3
0,-1

>hsa-mir-3164   5p:10-31   SRX018957
cuuggaaacUGUGACUUUAAGGGAAAUGGCGcacagcagacccugcaaucaugccguuuugcuugaagucgcaguuuccc
agg
((.((((((((((((((((((.(((((((((....((((...)))).....))))))))).)))))))))))))))))).
)).
---------UGUGACUUUAAGGGAAAUGGCG---
--- 2   0,0

>hsa-mir-3173   3p:43-64   SRX018957
ucccugcccugccuguuuucuccuuugugauuuuaugagaacAAAGGAGGAAAUAGGCAGGCCAggga
((((((.(((((((((((((((((((((..((....))..))))))))))))))))))))).))))))
--AAGGAGGAAAUAGGCAGGCCAaa--   2   1,0

>hsa-mir-3174   5p:10-32   SRX018957
guuaccuggUAGUGAGUUAGAGAUGCAGAGCCcugggcuccucagcaaaccuacuggaucugcauuuuaauucacaugca
ugguaau
((((((..((((((((((.((((((((((..((.(((((....)))...))....)).))))))))))))))))).))).
.))))))
---------UAGUGAGUUAGAGAUGCAG--
------- 2   0,-4

>hsa-mir-3179-1 3p:52-73   SRX018957
caggaucacagacguuuaaauuacacuccuucugcugugccuuacagcaguAGAAGGGGUGAAAUUUAAACGUcugugau
ccug
(((((((((((((((((((((.((((((((((((((((........)))))))))))))))).)))))))))))))))))
))))
--UAGAAGGGGUGAAAUUUAAACGU-------
----   2   -1,0

>hsa-mir-3179-2 3p:52-73   SRX018957
caggaucacagacguuuaaauuacacuccuucugcugugccuuacagcaguAGAAGGGGUGAAAUUUAAACGUcugugau
ccug
(((((((((((((((((((((.((((((((((((((((........)))))))))))))))).)))))))))))))))))
))))

--UAGAAGGGGUGAAAUUUAAACGU-------
----	2	-1,0

>hsa-mir-3179-3 3p:52-73	SRX018957
caggaucacagacguuuaaauuacacuccuucugcugugccuuacagcaguAGAAGGGGUGAAAUUUAAACGUcugugau
ccug
((((((((((((((((((((((.(((((((((((((((........))))))))))))))).))))))))))))))))))
))))
--UAGAAGGGGUGAAAUUUAAACGU-------
----	2	-1,0

>hsa-mir-3190	3p:47-69	SRX018957
cuggggucaccugucuggccagcuacguccccacggcccuugucagUGUGGAAGGUAGACGGCCAGAGAggugaccccgg
((((((((((((.(((((((..((((.(((.((((((....))).))).)))..))))..))))))).))))))))))))
--UGGAAGGUAGACGGCCAGAGAG----------
	2	2,1
--UGGAAGGUAGACGGCCAGAGAGa---------
	2	2,1

>hsa-mir-3191	3p:46-68	SRX018957
ggggucaccucucuggccgucuaccuuccacacugacaagggccgUGGGGACGUAGCUGGCCAGACAGgugacccc
((((((((((.((((((((.((((.((((.(((.(.......).))))))).)))).)))))))).))))))))))
---UGGGGACGUAGCUGGCCAGAC----------
	2	0,-2

>hsa-mir-32 MA:6-27;mi:47-68	SRX018957
ggagaUAUUGCACAUUACUAAGUUGCAuguugucacggccucaaugCAAUUUAGUGUGUGUGAUAUUUuc
((((((((..(((((.(((((((((((((..((....))..).)))))))))))))))))..))))))))
-----UAUUGCACAUUACUAAGUUG---	12	0,-2
-----UAUUGCACAUUACUAAGUUGC--	22	0,-1

>hsa-mir-3200	5p:13-34;3p:54-75	SRX018957
gguggucgagggAAUCUGAGAAGGCGCACAAGGUuuguguccaauacaguccaCACCUUGCGCUACUCAGGUCUGcucgu
gcccu
((.(((((((.(.(((((((..((((((..((((..(((..(......)..))))))))))))).))))))).).)))).
)))))
---CACCUUGCGCUACUCAGGUCU------
-----	2	0,-1

>hsa-mir-320a 3p:48-69 SRX018957
gcuucgcuccccuccgccuucucuucccgguucuucccggagucgggAAAAGCUGGGUUGAGAGGGCGAaaaaggaugag
gu
((((((.(((....(((((((((..(((((((.((((((....)))))).)))))))..))))))))).....)))))))
))
--GAAAAGCUGGGUUGAGAGG---------------
-- 2 -1,-4
--GAAAAGCUGGGUUGAGAGGGC-------------
-- 7 -1,-2
--GAAAAGCUGGGUUGAGAGGGCG------------
-- 19 -1,-1
--GAAAAGCUGGGUUGAGAGGGCGu-----------
-- 34 -1,-1
--GAAAAGCUGGGUUGAGAGGGCGuu----------
-- 4 -1,-1
--GAAAAGCUGGGUUGAGAGGGCGA-----------
-- 108 -1,0
--GAAAAGCUGGGUUGAGAGGGCGAu----------
-- 6 -1,0
--GAAAAGCUGGGUUGAGAGGGCGAAA---------
-- 2 -1,2
---AAAAGCUGGGUUGAGAGG---------------
-- 95 0,-4
---AAAAGCUGGGUUGAGAGGG--------------
-- 181 0,-3
---AAAAGCUGGGUUGAGAGGGa-------------
-- 17 0,-3
---AAAAGCUGGGUUGAGAGGGaa------------
-- 15 0,-3
---AAAAGCUGGGUUGAGAGGGau------------
-- 3 0,-3
---AAAAGCUGGGUUGAGAGGGC-------------
-- 565 0,-2
---AAAAGCUGGGUUGAGAGGGCu------------
-- 11 0,-2
---AAAAGCUGGGUUGAGAGGGCuu-----------
-- 2 0,-2

---AAAAGCUGGGUUGAGAGGGCG------------
-- 2225 0,-1
---AAAAGCUGGGUUGAGAGGGCGu-----------
-- 1966 0,-1
---AAAAGCUGGGUUGAGAGGGCGuu----------
-- 515 0,-1
---AAAAGCUGGGUUGAGAGGGCGA-----------
-- 13706 0,0
---AAAAGCUGGGUUGAGAGGGCGAu----------
-- 3140 0,0
---AAAAGCUGGGUUGAGAGGGCGAuu---------
-- 831 0,0
---AAAAGCUGGGUUGAGAGGGCGAA----------
-- 5488 0,1
---AAAAGCUGGGUUGAGAGGGCGAAu---------
-- 1719 0,1
---AAAAGCUGGGUUGAGAGGGCGAAuu--------
-- 227 0,1
---AAAAGCUGGGUUGAGAGGGCGAAA---------
-- 1285 0,2
---AAAAGCUGGGUUGAGAGGGCGAAAu--------
-- 445 0,2
---AAAAGCUGGGUUGAGAGGGCGAAAA--------
-- 189 0,3
--AAAGCUGGGUUGAGAGGG--------------
-- 9 1,-3
--AAAGCUGGGUUGAGAGGGC-------------
-- 23 1,-2
--AAAGCUGGGUUGAGAGGGCG------------
-- 101 1,-1
--AAAGCUGGGUUGAGAGGGCGu-----------
-- 45 1,-1
--AAAGCUGGGUUGAGAGGGCGuu----------
-- 23 1,-1
--AAAGCUGGGUUGAGAGGGCGA-----------
-- 560 1,0
--AAAGCUGGGUUGAGAGGGCGAu----------
-- 131 1,0

--AAAGCUGGGUUGAGAGGGCGAuu---------
-- 43 1,0
--AAAGCUGGGUUGAGAGGGCGAA----------
-- 458 1,1
--AAAGCUGGGUUGAGAGGGCGAAu---------
-- 220 1,1
--AAAGCUGGGUUGAGAGGGCGAAuu--------
-- 46 1,1
--AAAGCUGGGUUGAGAGGGCGAAA---------
-- 262 1,2
--AAAGCUGGGUUGAGAGGGCGAAAu--------
-- 99 1,2
--AAAGCUGGGUUGAGAGGGCGAAAuu-------
-- 4 1,2
--AAAGCUGGGUUGAGAGGGCGAAAA--------
-- 48 1,3
---AAGCUGGGUUGAGAGGGC-------------
-- 2 2,-2
---AAGCUGGGUUGAGAGGGCG------------
-- 6 2,-1
---AAGCUGGGUUGAGAGGGCGA-----------
-- 23 2,0
---AAGCUGGGUUGAGAGGGCGAu----------
-- 5 2,0
---AAGCUGGGUUGAGAGGGCGAuu---------
-- 2 2,0
---AAGCUGGGUUGAGAGGGCGAA----------
-- 44 2,1
---AAGCUGGGUUGAGAGGGCGAAu---------
-- 23 2,1
---AAGCUGGGUUGAGAGGGCGAAuu--------
-- 3 2,1
---AAGCUGGGUUGAGAGGGCGAAA---------
-- 76 2,2
---AAGCUGGGUUGAGAGGGCGAAAu--------
-- 32 2,2
---AAGCUGGGUUGAGAGGGCGAAAA--------
-- 16 2,3

>hsa-mir-320b-1 3p:39-60   SRX018957

aauuaaucccucucuuucuaguucuucccuagagugaggAAAAGCUGGGUUGAGAGGGCAAacaaauuaacuaauuaauu

........((((...((((((......)))))).))))...

--------------------------------------GAAAAGCUGGGUUGAGAGG-----------------------

2   -1,-4

--------------------------------------GAAAAGCUGGGUUGAGAGGGC---------------------

7   -1,-2

--------------------------------------GAAAAGCUGGGUUGAGAGGGCAu-------------------

3   -1,-1

---------------------------------------AAAAGCUGGGUUGAGAGG-----------------------

95   0,-4

---------------------------------------AAAAGCUGGGUUGAGAGGG----------------------

181 0,-3

---------------------------------------AAAAGCUGGGUUGAGAGGGa---------------------

17   0,-3

---------------------------------------AAAAGCUGGGUUGAGAGGGaa--------------------

15   0,-3

---------------------------------------AAAAGCUGGGUUGAGAGGGau--------------------

3   0,-3

---------------------------------------AAAAGCUGGGUUGAGAGGGC---------------------

565 0,-2

---------------------------------------AAAAGCUGGGUUGAGAGGGCu--------------------

11   0,-2

---------------------------------------AAAAGCUGGGUUGAGAGGGCuu-------------------

2   0,-2

---------------------------------------AAAAGCUGGGUUGAGAGGGCA--------------------

75   0,-1

---------------------------------------AAAAGCUGGGUUGAGAGGGCAu-------------------

115 0,-1

---------------------------------------AAAAGCUGGGUUGAGAGGGCAuu------------------

38   0,-1

---------------------------------------AAAAGCUGGGUUGAGAGGGCAA-------------------

167 0,0

---------------------------------------AAAAGCUGGGUUGAGAGGGCAAu------------------

34   0,0

---------------------------------------AAAAGCUGGGUUGAGAGGGCAAuu-----------------

6   0,0

--------------------------------------AAAAGCUGGGUUGAGAGGGCAAA------------------
    19 0,1
--------------------------------------AAAAGCUGGGUUGAGAGGGCAAAu-----------------
    7   0,1
---------------------------------------AAAGCUGGGUUGAGAGGG----------------------
    9   1,-3
---------------------------------------AAAGCUGGGUUGAGAGGGC---------------------
    23 1,-2
---------------------------------------AAAGCUGGGUUGAGAGGGCA--------------------
    6   1,-1
---------------------------------------AAAGCUGGGUUGAGAGGGCAu-------------------
    8   1,-1
---------------------------------------AAAGCUGGGUUGAGAGGGCAuu------------------
    2   1,-1
---------------------------------------AAAGCUGGGUUGAGAGGGCAA-------------------
    5   1,0
---------------------------------------AAAGCUGGGUUGAGAGGGCAAu------------------
    4   1,0
---------------------------------------AAAGCUGGGUUGAGAGGGCAAA------------------
    2   1,1
--AAGCUGGGUUGAGAGGGC---------------------
    2   2,-2

>hsa-mir-320b-2 3p:72-93   SRX018957
uguuauuuuuugucuucuaccuaagaauucugucucuuaggcuuucucuucccagauuucccaaaguugggAAAAGCUGG
GUUGAGAGGGCAAaaggaaaaaaaaagaauucugucucugacauaauuagauagggaa
.......................((((((((.((.(((..((((((((..(((((.(((((((....)))))))..))))
)..))))))))..)))))......)))))))).........................
--GAAAAGCUGG
GUUGAGAGG--- 2   -1,-4
--GAAAAGCUGG
GUUGAGAGGGC--- 7   -1,-2
--GAAAAGCUGG
GUUGAGAGGGCAu--- 3   -1,-1
---AAAAGCUGG
GUUGAGAGG--- 95 0,-4
---AAAAGCUGG
GUUGAGAGGG-- 181 0,-3

--AAAAGCUGG
GUUGAGAGGGa--- 17 0,-3
--AAAAGCUGG
GUUGAGAGGGaa-- 15 0,-3
--AAAAGCUGG
GUUGAGAGGGau-- 3   0,-3
--AAAAGCUGG
GUUGAGAGGGC--- 565 0,-2
--AAAAGCUGG
GUUGAGAGGGCu-- 11 0,-2
--AAAAGCUGG
GUUGAGAGGGCuu--- 2   0,-2
--AAAAGCUGG
GUUGAGAGGGCA-- 75 0,-1
--AAAAGCUGG
GUUGAGAGGGCAu--- 115 0,-1
--AAAAGCUGG
GUUGAGAGGGCAuu-- 38 0,-1
--AAAAGCUGG
GUUGAGAGGGCAA--- 167 0,0
--AAAAGCUGG
GUUGAGAGGGCAAu-- 34 0,0
--AAAAGCUGG
GUUGAGAGGGCAAuu--- 6   0,0
--AAAAGCUGG
GUUGAGAGGGCAAA-- 19 0,1
--AAAAGCUGG
GUUGAGAGGGCAAAu--- 7   0,1
--AAAAGCUGG
GUUGAGAGGGCAAAA--- 6   0,2
--AAAAGCUGG
GUUGAGAGGGCAAAAa-- 2   0,2
---AAAGCUGG
GUUGAGAGGG-- 9   1,-3
---AAAGCUGG
GUUGAGAGGGC--- 23 1,-2
---AAAGCUGG
GUUGAGAGGGCA-- 6   1,-1

---AAAGCUGG
GUUGAGAGGGCAu-- 8   1,-1
---AAAGCUGG
GUUGAGAGGGCAuu--- 2   1,-1
---AAAGCUGG
GUUGAGAGGGCAA-- 5   1,0
---AAAGCUGG
GUUGAGAGGGCAAu--- 4   1,0
---AAAGCUGG
GUUGAGAGGGCAAA--- 2   1,1
---AAAGCUGG
GUUGAGAGGGCAAAA-- 2   1,2
--AAGCUGG
GUUGAGAGGGC-- 2   2,-2

>hsa-mir-320c-1 3p:50-69   SRX018957
uuugcauuaaaaaugaggccuucucuucccaguucuucccagagucaggAAAAGCUGGGUUGAGAGGGUagaaaaaaau
gauguagg
.((((((((........((((((((..(((((((.((((........)))).)))))))..)))))))).........)
)))))))).
--GAAAAGCUGGGUUGAGAGG------------
--------   2   -1,-2
---AAAAGCUGGGUUGAGAGG------------
--------   95   0,-2
---AAAAGCUGGGUUGAGAGGG-----------
--------   181 0,-1
---AAAAGCUGGGUUGAGAGGGa----------
--------   17   0,-1
---AAAAGCUGGGUUGAGAGGGaa---------
--------   15   0,-1
---AAAAGCUGGGUUGAGAGGGau---------
--------   3   0,-1
---AAAAGCUGGGUUGAGAGGGU----------
--------   77   0,0
---AAAAGCUGGGUUGAGAGGGUu---------
--------   5   0,0
---AAAAGCUGGGUUGAGAGGGUuu--------
--------   3   0,0

---AAAAGCUGGGUUGAGAGGGUA----------
-------- 8 0,1
---AAAAGCUGGGUUGAGAGGGUAa---------
-------- 13 0,1
---AAAAGCUGGGUUGAGAGGGUAau--------
-------- 2 0,1
---AAAAGCUGGGUUGAGAGGGUAu---------
-------- 16 0,1
--AAAGCUGGGUUGAGAGGG------------
-------- 9 1,-1
--AAAGCUGGGUUGAGAGGGUA----------
-------- 3 1,1

>hsa-mir-320c-2 3p:31-50 SRX018957
cuucucuuuccaguucuucccagaauugggAAAAGCUGGGUUGAGAGGGU
((((((..(((((((.(((((......))))).)))))))..))))))..
-----------------------------GAAAAGCUGGGUUGAGAGG-- 2 -1,-2
------------------------------AAAAGCUGGGUUGAGAGG-- 95 0,-2
------------------------------AAAAGCUGGGUUGAGAGGG- 181 0,-1
------------------------------AAAAGCUGGGUUGAGAGGGa 17 0,-1
------------------------------AAAAGCUGGGUUGAGAGGGaa 15 0,-1
------------------------------AAAAGCUGGGUUGAGAGGGau 3 0,-1
------------------------------AAAAGCUGGGUUGAGAGGGU 77 0,0
------------------------------AAAAGCUGGGUUGAGAGGGUu 5 0,0
------------------------------AAAAGCUGGGUUGAGAGGGUuu 3 0,0
-------------------------------AAAGCUGGGUUGAGAGGG- 9 1,-1

>hsa-mir-320d-1 3p:30-48 SRX018957
uucucgucccaguucuucccaaaguugagAAAAGCUGGGUUGAGAGGA
((((((.(((((((.(((.((....)).))).))))))).))))))..
----------------------------GAAAAGCUGGGUUGAGAGG- 2 -1,-1
-----------------------------AAAAGCUGGGUUGAGAGG- 95 0,-1
-----------------------------AAAAGCUGGGUUGAGAGGA 4 0,0
-----------------------------AAAAGCUGGGUUGAGAGGAa 7 0,0
-----------------------------AAAAGCUGGGUUGAGAGGAaa 6 0,0

>hsa-mir-320d-2 3p:30-48 SRX018957
uucucuucccaguucuucuuggagucaggAAAAGCUGGGUUGAGAGGA

(((((..(((((((.((((((....)))))).)))))))..)))))..
--------------------------GAAAAGCUGGGUUGAGAGG- 2 -1,-1
---------------------------AAAAGCUGGGUUGAGAGG- 95 0,-1
---------------------------AAAAGCUGGGUUGAGAGGA 4 0,0
---------------------------AAAAGCUGGGUUGAGAGGAa 7 0,0
---------------------------AAAAGCUGGGUUGAGAGGAaa 6 0,0

>hsa-mir-320e 3p:35-52 SRX018957
gccuucucuucccaguucuuccuggagucggggaAAAGCUGGGUUGAGAAGGu
((((((((..(((((((.(((((.(...).))))).)))))))..))))))))
---------------------------------AAAAGCUGGGUUGAGAAGG- 14 -1,0

>hsa-mir-323 5p:16-37;3p:51-71 SRX018957
uugguacuuggagagAGGUGGUCCGUGGCGCGUUCGCuuuauuuauggcgCACAUUACACGGUCGACCUCUuugcaguau
cuaauc
..((((((.(.((((((((.(.(((((....((.((((........)))).))....))))).).)))))))).))))))
).....
---------------AGGUGGUCCGUGGCGCGUUC---
------ 2 0,-2
--CGCACAUUACACGGUCGACCU-----------
------ 2 -2,-2
---GCACAUUACACGGUCGAC-------------
------ 3 -1,-4
---GCACAUUACACGGUCGACC------------
------ 3 -1,-3
---GCACAUUACACGGUCGACCU-----------
------ 23 -1,-2
---GCACAUUACACGGUCGACCUC----------
------ 6 -1,-1
---GCACAUUACACGGUCGACCUCU---------
------ 4 -1,0
--CACAUUACACGGUCGACC------------
------ 7 0,-3
--CACAUUACACGGUCGACCU-----------
------ 106 0,-2
--CACAUUACACGGUCGACCUC----------
------ 8 0,-1

>hsa-mir-323b	5p:15-37;3p:51-72	SRX018957
ugguacucggagggAGGUUGUCCGUGGUGAGUUCGCAuuauuuaaugaugCCCAAUACACGGUCGACCUCUUuucgguau
ca
.(((((.(((((((((((((.(((((........(((((((...)))))))......))))).)))))))))))))))))
).
--CCCAAUACACGGUCGACCU-----------
--	44	0,-3
---CCAAUACACGGUCGACCU-----------
--	2	1,-3

>hsa-mir-324	5p:16-38;3p:53-72	SRX018957
cugacuaugccucccCGCAUCCCCUAGGGCAUUGGUGUaaagcuggagacccACUGCCCCAGGUGCUGCUGGggguugua
guc
..((((((..(((((.(((.(.(((.(((((.(((.((..........))))).))))).))).).))).)))))..)))
)))
---------------CGCAUCCCCUAGGGCAUUG--
---	3	0,-4
---------------CGCAUCCCCUAGGGCAUUGG---
---	3	0,-3
---------------CGCAUCCCCUAGGGCAUUGGU--
---	7	0,-2
---------------CGCAUCCCCUAGGGCAUUGGUG---
---	2	0,-1
---------------CGCAUCCCCUAGGGCAUUGGUGU--
---	10	0,0
---------------CGCAUCCCCUAGGGCAUUGGUGUu---
---	2	0,0
--CCACUGCCCCAGGUGCUGCUG---------
---	6	-2,-1
--CCACUGCCCCAGGUGCUGCUGGau------
---	2	-2,0
--ACUGCCCCAGGUGCUGCU----------
---	2	0,-2
--ACUGCCCCAGGUGCUGCUG---------
---	10	0,-1
--ACUGCCCCAGGUGCUGCUGG--------
---	23	0,0

>hsa-mir-326 3p:60-79 SRX018957
cucaucugucuguugggcuggaggcagggccuuugugaaggcggguggugcucagaucgCCUCUGGGCCCUUCCUCCAGc
cccgaggcggauuca
...((((((((...((((((((((.(((((((....((.(((((.((.....))..))))))).))))))).))))))))
))..))))))))...
---CCUCUGGGCCCUUCCUCCAGa
--------------- 5 0,0
---CCUCUGGGCCCUUCCUCCAGu
--------------- 3 0,0

>hsa-mir-328 3p:48-69 SRX018957
uggagugggggggcaggaggggcucagggagaaagugcauacagcccCUGGCCCUCUCUGCCCUUCCGUccccug
.((..(((((((((((..(((((.(((((.(...((....))..)))))))))))..)))))))))))..))...
---CUGGCCCUCUCUGCCCUUCCGU------ 3
0,0
---CUGGCCCUCUCUGCCCUUCCGUa----- 3
0,0
---CUGGCCCUCUCUGCCCUUCCGUau---- 2
0,0
---CUGGCCCUCUCUGCCCUUCCGUu----- 3
0,0
---CUGGCCCUCUCUGCCCUUCCGUuu---- 3
0,0

>hsa-mir-330 5p:18-39;3p:57-79 SRX018957
cuuuggcgaucacugccUCUCUGGGCCUGUGUCUUAGGCucugcaagaucaaccgaGCAAAGCACACGGCCUGCAGAGAg
gcagcgcucugccc
....(((((.(.(((((((((((((((((((.(((..((((.............)))).))))))).))))..)))))))
)))).).)).))).
-----------------UCUCUGGGCCUGUGUCUUAGGCau---------------------------------------
-------------- 2 0,0
--GCAAAGCACACGGCCUGCA-----
-------------- 2 0,-4
--GCAAAGCACACGGCCUGCAGAGA-
-------------- 10 0,0
---CAAAGCACACGGCCUGCAGAGA-
-------------- 8 1,0
---CAAAGCACACGGCCUGCAGAGAG

-------------- 13 1,1
---CAAAGCACACGGCCUGCAGAGAG
a------------- 6   1,1
---CAAAGCACACGGCCUGCAGAGAG
u------------- 4   1,1
---CAAAGCACACGGCCUGCAGAGAG
G------------- 6   1,2
---CAAAGCACACGGCCUGCAGAGAG
Gu------------ 2   1,2
--AAAGCACACGGCCUGCAGAGA-
-------------- 8   2,0
--AAAGCACACGGCCUGCAGAGAG
-------------- 5   2,1
--AAAGCACACGGCCUGCAGAGAG
G------------- 4   2,2
--AAAGCACACGGCCUGCAGAGAG
Gu------------ 3   2,2

>hsa-mir-331   5p:26-47;3p:61-81   SRX018957
gaguuugguuuuguuuggguuuguuCUAGGUAUGGUCCCAGGGAUCCcagaucaaaccagGCCCCUGGGCCUAUCCUAGA
Accaaccuaagcuc
............((((((((.((((((((.((((.(((((((..(.............)..))))))).))))))))))
)).)))))))))..
--GCCCCUGGGCCUAUCCUA--
-------------- 2   0,-3
--GCCCCUGGGCCUAUCCUAG-
-------------- 4   0,-2
--GCCCCUGGGCCUAUCCUAGA
-------------- 16 0,-1
--GCCCCUGGGCCUAUCCUAGA
u------------- 3   0,-1
--GCCCCUGGGCCUAUCCUAGA
A------------- 50 0,0
--GCCCCUGGGCCUAUCCUAGA
Aa------------ 11 0,0
--GCCCCUGGGCCUAUCCUAGA
Aaa----------- 2   0,0
--GCCCCUGGGCCUAUCCUAGA

Au------------ 4 0,0

>hsa-mir-335 MA:16-38;mi:52-73 SRX018957
uguuuugagcgggggUCAAGAGCAAUAACGAAAAAUGUuugucauaaaccgUUUUUCAUUAUUGCUCCUGACCuccucuc
auuugcuauauuca
.((..((((.((((((((.(((((((((.((((((((.((......)).)))))))).))))))))).)))))))).)))
)...))........
---------------UCAAGAGCAAUAACGAAAA--
-------------- 3 0,-4
---------------UCAAGAGCAAUAACGAAAAA---
-------------- 8 0,-3
---------------UCAAGAGCAAUAACGAAAAAa--
-------------- 3 0,-3
---------------UCAAGAGCAAUAACGAAAAAU--
-------------- 54 0,-2
---------------UCAAGAGCAAUAACGAAAAAUa---
-------------- 2 0,-2
---------------UCAAGAGCAAUAACGAAAAAUG---
-------------- 194 0,-1
---------------UCAAGAGCAAUAACGAAAAAUGa--
-------------- 5 0,-1
---------------UCAAGAGCAAUAACGAAAAAUGU--
-------------- 70 0,0
-----------------AAGAGCAAUAACGAAAAAUG---
-------------- 5 2,-1
---UUUUUCAUUAUUGCUCCUGAC--------
-------------- 4 0,-1
---UUUUUCAUUAUUGCUCCUGACC-------
-------------- 3 0,0

>hsa-mir-339 5p:15-37;3p:50-72 SRX018957
cggggcggccgcucUCCCUGUCCUCCAGGAGCUCACGugugccugccugUGAGCGCCUCGACGACAGAGCCGgcgccugc
cccagugucugcgc
.(((((((.((((..(.(((((.((.(((.(((((((.((....)).))))))).))).)).))))).)..)))).))))
)))...........
--------------UCCCUGUCCUCCAGGAGC--
-------------- 4 0,-5
--------------UCCCUGUCCUCCAGGAGCU---

------------- 9 0,-4
--------------UCCCUGUCCUCCAGGAGCUCA--
------------- 10 0,-2
--------------UCCCUGUCCUCCAGGAGCUCAu---
------------- 4 0,-2
--------------UCCCUGUCCUCCAGGAGCUCAuu--
------------- 4 0,-2
--------------UCCCUGUCCUCCAGGAGCUCAC---
------------- 5 0,-1
--------------UCCCUGUCCUCCAGGAGCUCACG--
------------- 16 0,0
--------------UCCCUGUCCUCCAGGAGCUCACGU---
------------- 6 0,1
--------------UCCCUGUCCUCCAGGAGCUCACGUu--
------------- 3 0,1
----------------CCUGUCCUCCAGGAGCUCACG--
------------- 4 2,0
----------------CCUGUCCUCCAGGAGCUCACGUa--
------------- 2 2,1
--UGAGCGCCUCGACGACAG-------------
------------- 7 0,-5
--UGAGCGCCUCGACGACAGA------------
------------- 12 0,-4
--UGAGCGCCUCGACGACAGAG-----------
------------- 36 0,-3
--UGAGCGCCUCGACGACAGAGC----------
------------- 15 0,-2
--UGAGCGCCUCGACGACAGAGCa---------
------------- 3 0,-2
--UGAGCGCCUCGACGACAGAGCau--------
------------- 4 0,-2
--UGAGCGCCUCGACGACAGAGCC---------
------------- 28 0,-1
--UGAGCGCCUCGACGACAGAGCCa--------
------------- 37 0,-1
--UGAGCGCCUCGACGACAGAGCCaa-------
------------- 4 0,-1
--UGAGCGCCUCGACGACAGAGCCau-------

------------- 20 0,-1
---UGAGCGCCUCGACGACAGAGCCu--------
------------- 24 0,-1
---UGAGCGCCUCGACGACAGAGCCuu-------
------------- 17 0,-1
---UGAGCGCCUCGACGACAGAGCCG--------
------------- 18 0,0
---UGAGCGCCUCGACGACAGAGCCGa-------
------------- 2 0,0
---UGAGCGCCUCGACGACAGAGCCGau------
------------- 3 0,0
---UGAGCGCCUCGACGACAGAGCCGu-------
------------- 5 0,0
---UGAGCGCCUCGACGACAGAGCCGuu------
------------- 5 0,0
--GAGCGCCUCGACGACAGAG-----------
------------- 2 1,-3
---AGCGCCUCGACGACAGAG-----------
------------- 24 2,-3
---AGCGCCUCGACGACAGAGCC---------
------------- 2 2,-1
---AGCGCCUCGACGACAGAGCCG--------
------------- 8 2,0
---AGCGCCUCGACGACAGAGCCGuu------
------------- 2 2,0

>hsa-mir-33a   MA:6-26;mi:46-67   SRX018957
cugugGUGCAUUGUAGUUGCAUUGCAuguucuggugguacccaugCAAUGUUUCCACAGUGCAUCACag
((((((((((((((.(..((((((((((....((.....))))))))))))..).))))))))))))))
-----GUGCAUUGUAGUUGCAUU-- 121 0,-3
-----GUGCAUUGUAGUUGCAUUG--- 6 0,-2
-----GUGCAUUGUAGUUGCAUUGa-- 2 0,-2
-----GUGCAUUGUAGUUGCAUUGC-- 35 0,-1
-----GUGCAUUGUAGUUGCAUUGCA--- 45 0,0
-----GUGCAUUGUAGUUGCAUUGCAa-- 41 0,0
-----GUGCAUUGUAGUUGCAUUGCAau--- 3 0,0
---CAAUGUUUCCACAGUGCAUCA--- 2 0,-1

>hsa-mir-33b MA:16-35;mi:54-75 SRX018957
gcgggcggccccgcgGUGCAUUGCUGUUGCAUUGCacgugugugaggcgggugCAGUGCCUCGGCAGUGCAGCCCggagc
cggccccuggcaccac
(((((((((.(((.((((((((((((..(((((((((.(((.....))).)))))))))..)))))))))).))))).))
).))))...)).....
---------------GUGCAUUGCUGUUGCAUU---
---------------- 11 0,-2
---------------GUGCAUUGCUGUUGCAUUG--
---------------- 3 0,-1
---------------GUGCAUUGCUGUUGCAUUGa---
---------------- 2 0,-1
---------------GUGCAUUGCUGUUGCAUUGCA--
---------------- 2 0,1
---------------GUGCAUUGCUGUUGCAUUGCAa---
---------------- 2 0,1
---CAGUGCCUCGGCAGUGCAGCC------
---------------- 2 0,-1

>hsa-mir-340 MA:16-37;mi:58-79 SRX018957
uuguaccuggugugaUUAUAAAGCAAUGAGACUGAUUgucauaugucguuuguggga UCCGUCUCAGUUACUUUAUAGCc
auaccugguaucuua
..(((((.((((((..(((((((...((((((.((((.(((((.......))))))))).))))))....)))))))..)
))))).)))))....
---------------UUAUAAAGCAAUGAGACU---
--------------- 40 0,-4
---------------UUAUAAAGCAAUGAGACUGA---
--------------- 8 0,-2
---------------UUAUAAAGCAAUGAGACUGAa--
--------------- 4 0,-2
---------------UUAUAAAGCAAUGAGACUGAU--
--------------- 19 0,-1
---------------UUAUAAAGCAAUGAGACUGAUa---
--------------- 15 0,-1
---------------UUAUAAAGCAAUGAGACUGAUU---
--------------- 89 0,0
---------------UUAUAAAGCAAUGAGACUGAUUa--
--------------- 2 0,0
---------------UUAUAAAGCAAUGAGACUGAUUG--

--------------- 2   0,1

>hsa-mir-342   5p:19-39;3p:61-83   SRX018957
gaaacugggcucaaggugAGGGGUGCUAUCUGUGAUUGAgggacaugguuaauggaauugUCUCACACAGAAAUCGCACC
CGUcaccuuggccuacuua
.....(((((.((((((((.((((((..((((((.....((((((...((....))..)))))).))))))....)))))
).)))))))))))))....
------------------AGGGGUGCUAUCUGUGAUa---
------------------- 2   0,-3
------------------AGGGGUGCUAUCUGUGAUU---
------------------- 26   0,-2
------------------AGGGGUGCUAUCUGUGAUUG--
------------------- 6   0,-1
------------------AGGGGUGCUAUCUGUGAUUGA---
------------------- 26   0,0
------------------AGGGGUGCUAUCUGUGAUUGAaa---------------------------------------
------------------- 2   0,0
------------------AGGGGUGCUAUCUGUGAUUGAG--
------------------- 36   0,1
------------------AGGGGUGCUAUCUGUGAUUGAGa---------------------------------------
------------------- 6   0,1
------------------AGGGGUGCUAUCUGUGAUUGAGu---------------------------------------
------------------- 3   0,1
------------------AGGGGUGCUAUCUGUGAUUGAGG---------------------------------------
------------------- 25   0,2
------------------AGGGGUGCUAUCUGUGAUUGAGGG--------------------------------------
------------------- 2   0,3
------------------AGGGGUGCUAUCUGUGAUUGAGGGA-------------------------------------
------------------- 4   0,4
-------------------GGGGUGCUAUCUGUGAUUGAGGa--------------------------------------
------------------- 2   1,2
-------------------GGGGUGCUAUCUGUGAUUGAGGGA-------------------------------------
------------------- 5   1,4
-------------------GGGGUGCUAUCUGUGAUUGAGGGAC------------------------------------
------------------- 2   1,5
--UCUCACACAGAAAUCGCA--
------------------- 12   0,-5
--UCUCACACAGAAAUCGCAC-

------------------- 32 0,-4

---UCUCACACAGAAAUCGCACC

------------------- 18 0,-3

---UCUCACACAGAAAUCGCACC

C------------------ 15 0,-2

---UCUCACACAGAAAUCGCACC

CG----------------- 59 0,-1

---UCUCACACAGAAAUCGCACC

CGU---------------- 217 0,0

---UCUCACACAGAAAUCGCACC

CGUa--------------- 8 0,0

---UCUCACACAGAAAUCGCACC

CGUu--------------- 10 0,0

---UCUCACACAGAAAUCGCACC

CGUC--------------- 114 0,1

---UCUCACACAGAAAUCGCACC

CGUCu-------------- 49 0,1

---UCUCACACAGAAAUCGCACC

CGUCA-------------- 32 0,2

--CUCACACAGAAAUCGCACC

CG----------------- 3 1,-1

---UCACACAGAAAUCGCACC

C------------------ 4 2,-2

---UCACACAGAAAUCGCACC

CG----------------- 2 2,-1

---UCACACAGAAAUCGCACC

CGU---------------- 7 2,0

---UCACACAGAAAUCGCACC

CGUaa-------------- 2 2,0

---UCACACAGAAAUCGCACC

CGUC--------------- 3 2,1

---UCACACAGAAAUCGCACC

CGUCu-------------- 5 2,1

---UCACACAGAAAUCGCACC

CGUCA-------------- 42 2,2

---UCACACAGAAAUCGCACC

CGUCAu------------- 5 2,2

>hsa-mir-345   5p:18-39   SRX018957
acccaaacccuaggucuGCUGACUCCUAGUCCAGGGCUCgugauggcugguggggcccugaacgaggggucuggaggccug
gguuugaauaucgacagc
...(((((((.((((((.(.(((((((.((.(((((((((..(....)..))))))))).)).))))))).).)))))))
))))))............
-----------------GCUGACUCCUAGUCCAGG--
------------------ 57 0,-4
-----------------GCUGACUCCUAGUCCAGGG---
------------------ 26 0,-3
-----------------GCUGACUCCUAGUCCAGGGC--
------------------ 24 0,-2
-----------------GCUGACUCCUAGUCCAGGGCU---
------------------ 46 0,-1
-----------------GCUGACUCCUAGUCCAGGGCUa--
------------------ 4   0,-1
-----------------GCUGACUCCUAGUCCAGGGCUaa---
------------------ 8   0,-1
-----------------GCUGACUCCUAGUCCAGGGCUau---
------------------ 14 0,-1
-----------------GCUGACUCCUAGUCCAGGGCUu--
------------------ 4   0,-1
-----------------GCUGACUCCUAGUCCAGGGCUuu---
------------------ 12 0,-1
-----------------GCUGACUCCUAGUCCAGGGCUC--
------------------ 50 0,0
-----------------GCUGACUCCUAGUCCAGGGCUCa---
------------------ 4   0,0
-----------------GCUGACUCCUAGUCCAGGGCUCaa--
------------------ 10 0,0
-----------------GCUGACUCCUAGUCCAGGGCUCau--
------------------ 24 0,0
-----------------GCUGACUCCUAGUCCAGGGCUCuu--
------------------ 16 0,0
-----------------GCUGACUCCUAGUCCAGGGCUCGa--
------------------ 3   0,1

>hsa-mir-34a   MA:22-43;mi:64-85   SRX018957
ggccagcugugaguguuucuuUGGCAGUGUCUUAGCUGGUUGUugugagcaauaguaaggaagCAAUCAGCAAGUAUACU

GCCCUagaagugcugcacguuguggggccc
((((.((((((((..(((((..(((((((((((.((((((((((....((....)).....))))))))))))).)))))
)))..)))))..)).))))..))..)))).
---------------------UGGCAGUGUCUUAGCUGGUUGU-------------------------------------
------------------------------ 2 0,0
--AAUCAGCAAGUAUACU
GCCCUA------------------------ 15 1,1

>hsa-mir-3605 5p:22-44;3p:66-88 SRX018957
acuuuauacguguaauugugaUGAGGAUGGAUAGCAAGGAAGCCgcucccaccugacccucacggCCUCCGUGUUACCUG
UCCUCUAGgugggacgcucg
.........((((.........((((((((.(((((.(((.((((.................)))).))).))))).)))
))))).........))))...
-------------------GAUGAGGAUGGAUAGCAAGG---
-------------------- 3 -2,-5
-------------------GAUGAGGAUGGAUAGCAAGGA--
-------------------- 13 -2,-4
-------------------GAUGAGGAUGGAUAGCAAGGAA---------------------------------------
-------------------- 10 -2,-3
-------------------GAUGAGGAUGGAUAGCAAGGAAa--------------------------------------
-------------------- 2 -2,-3
-------------------GAUGAGGAUGGAUAGCAAGGAAG--------------------------------------
-------------------- 5 -2,-2
-------------------GAUGAGGAUGGAUAGCAAGGAAGC-------------------------------------
-------------------- 3 -2,-1
-------------------GAUGAGGAUGGAUAGCAAGGAAGCC------------------------------------
-------------------- 3 -2,0
--------------------AUGAGGAUGGAUAGCAAGGAA---------------------------------------
-------------------- 2 -1,-3
--------------------AUGAGGAUGGAUAGCAAGGAAa--------------------------------------
-------------------- 2 -1,-3
--------------------AUGAGGAUGGAUAGCAAGGAAG--------------------------------------
-------------------- 2 -1,-2
---------------------UGAGGAUGGAUAGCAAGG---
-------------------- 3 0,-5
---------------------UGAGGAUGGAUAGCAAGGAA---------------------------------------
-------------------- 3 0,-3
---------------------UGAGGAUGGAUAGCAAGGAAG--------------------------------------

------------------- 5 0,-2
--------------------UGAGGAUGGAUAGCAAGGAAGC-----------------------------------
------------------- 5 0,-1
--------------------UGAGGAUGGAUAGCAAGGAAGCC----------------------------------
------------------- 10 0,0
--------------------UGAGGAUGGAUAGCAAGGAAGCCu---------------------------------
------------------- 2 0,0

>hsa-mir-361 5p:6-27;3p:45-67 SRX018957
ggagcUUAUCAGAAUCUCCAGGGGUACuuuauaauuucaaaaagUCCCCCAGGUGUGAUUCUGAUUUgcuuc
(((((..(((((((((.((.((((.(((((..........))))).)))).))...)))))))))..)))))
-----UUAUCAGAAUCUCCAGGG-- 3
0,-4
-----UUAUCAGAAUCUCCAGGGG--- 11
0,-3
-----UUAUCAGAAUCUCCAGGGGU-- 11
0,-2
-----UUAUCAGAAUCUCCAGGGGUA--- 39
0,-1
-----UUAUCAGAAUCUCCAGGGGUAa-- 23
0,-1
-----UUAUCAGAAUCUCCAGGGGUAu-- 53
0,-1
-----UUAUCAGAAUCUCCAGGGGUAuu--- 4
0,-1
-----UUAUCAGAAUCUCCAGGGGUAC-- 51
0,0
-----UUAUCAGAAUCUCCAGGGGUACa--- 32
0,0
-----UUAUCAGAAUCUCCAGGGGUACaa-- 4
0,0
-----UUAUCAGAAUCUCCAGGGGUACau-- 4
0,0
-----UUAUCAGAAUCUCCAGGGGUACU--- 9
0,1
---UCCCCCAGGUGUGAUUCU---------- 2
0,-5
---UCCCCCAGGUGUGAUUCUGA-------- 2

0,-3

--UCCCCCAGGUGUGAUUCUGAa------- 6

0,-3

--UCCCCCAGGUGUGAUUCUGAU------- 2

0,-2

--UCCCCCAGGUGUGAUUCUGAUa------ 2

0,-2

--UCCCCCAGGUGUGAUUCUGAUU------ 3

0,-1

--UCCCCCAGGUGUGAUUCUGAUUU----- 7

0,0

--UCCCCCAGGUGUGAUUCUGAUUUG---- 2

0,1

---CCCCCAGGUGUGAUUCUGAUUUGC--- 3

1,2

>hsa-mir-3613 5p:16-37;3p:53-76 SRX018957

ugguuggguuuggauUGUUGUACUUUUUUUUUGUUCguugcauuuuuaggaACAAAAAAAAAAGCCCAACCCUUCacac
cacuuca

.((...(((.((((..((((..((((((((((((((.(.........).)))))))))))))))..))))..)))).))
)...)).

--ACAAAAAAAAAAGCCCAACCCUUu----
------- 2 0,-1

>hsa-mir-3614 5p:15-37;3p:53-75 SRX018957

gguucugucuugggCCACUUGGAUCUGAAGGCUGCCCcuuugcucucuggggUAGCCUUCAGAUCUUGGUGUUUUgaauu
cuuacu

.........(..(..((((.(((((((((((((((((.........))))))))))))))))).))))..)..)...
......

--UAGCCUUCAGAUCUUGGUGUU-------
------ 3 0,-2

>hsa-mir-3615 3p:51-71 SRX018957

gacucugggacgcucagacgccgcgcggggcggggauuggucuguggucccUCUCUCGGCUCCUCGCGGCUCgcggcggcc
gacgguu

((((.(.((.((((..((.((((((.(((((.((((..((.(....).))..)))).))))).))))))))..)))).))
.).))))

--UCUCUCGGCUCCUCGCGGC-----------

------- 11 0,-2
--UCUCUCGGCUCCUCGCGGCU----------
------- 25 0,-1
--UCUCUCGGCUCCUCGCGGCUuu--------
------- 4 0,-1
--UCUCUCGGCUCCUCGCGGCUC---------
------- 2 0,0

>hsa-mir-362 5p:5-28;3p:42-63 SRX018957
cuugAAUCCUUGGAACCUAGGUGUGAGUgcuauuucagugcAACACACCUAUUCAAGGAUUCAaa
.(((((((((((((...((((((((.(..((.....))..)..))))))))))))))))))))).
----AAUCCUUGGAACCUAGGUGUG-- 5 0,-3
----AAUCCUUGGAACCUAGGUGUGA--------------------------------------- 4 0,-2
----AAUCCUUGGAACCUAGGUGUGAGU------------------------------------- 2 0,0
---AACACACCUAUUCAAGGAUU---- 3 0,-2
---AACACACCUAUUCAAGGAUUC--- 2 0,-1
---AACACACCUAUUCAAGGAUUCA-- 5 0,0
---AACACACCUAUUCAAGGAUUCAA- 2 0,1

>hsa-mir-363 mi:7-28;MA:50-71 SRX018957
uguuguCGGGUGGAUCACGAUGCAAUUUugaugaguaucauaggagaaaAAUUGCACGGUAUCCAUCUGUAaacc
.(((..(((((((((..((.(((((((((.(((.....)))......)))))))))))..)))))))))..))).
---AAUUGCACGGUAUCCAUC-------- 3
0,-4
---AAUUGCACGGUAUCCAUCU------- 10
0,-3
---AAUUGCACGGUAUCCAUCUG------ 3
0,-2
---AAUUGCACGGUAUCCAUCUGU----- 8
0,-1
---AAUUGCACGGUAUCCAUCUGUA---- 17
0,0
---AAUUGCACGGUAUCCAUCUGUAuu-- 3
0,0
---AAUUGCACGGUAUCCAUCUGUAA--- 14
0,1
--AUUGCACGGUAUCCAUCUGUA---- 3
1,0

--AUUGCACGGUAUCCAUCUGUAu--- 2
1,0

>hsa-mir-3647 5p:15-37;3p:62-84 SRX018957
uugcuuucaucagcCUGAAGUGAUGAUUCACAUUCAUgucucuucucugauaaauucuugaAGAAAAUUUUUGUGUGUCU
GAUCaggccucuagagggcag
.(((((((....((((((......(((.((((........(((((...((.....))..)))))......)))).))).
..))))))).....))))))).
--AAGAAAAUUUUUGUGUGUCU
GA------------------- 2 -1,-2

>hsa-mir-365-1 3p:56-77 SRX018957
accgcagggaaaaugagggacuuuugggggcagauguguuuccauuccacuaucaUAAUGCCCCUAAAAAUCCUUAUugc
ucuugca
...((((((..((((((((..(((((((((((((((((.........))).)))....))))))))))).))))))))..
)))))).
---------------AGGGACUUUUGGGGGCAGA---
------- 2
---------------AGGGACUUUUGGGGGCAGAU--
------- 2
---------------AGGGACUUUUGGGGGCAGAUu---
------- 2
---------------AGGGACUUUUGGGGGCAGAUG---
------- 6
---------------AGGGACUUUUGGGGGCAGAUGU--
------- 23
---------------AGGGACUUUUGGGGGCAGAUGUa---
------- 2
---------------AGGGACUUUUGGGGGCAGAUGUaa--
------- 3
---------------AGGGACUUUUGGGGGCAGAUGUau--
------- 3
---------------AGGGACUUUUGGGGGCAGAUGUu---
------- 2
---------------AGGGACUUUUGGGGGCAGAUGUuu--
------- 3
---------------AGGGACUUUUGGGGGCAGAUGUG---
------- 65

---------------AGGGACUUUUGGGGGCAGAUGUGa---
------- 4
---------------AGGGACUUUUGGGGGCAGAUGUGU---
------- 59
---------------AGGGACUUUUGGGGGCAGAUGUGUU--
------- 2
----------------GGGACUUUUGGGGGCAGAUGUG--
------- 2
----------------GGGACUUUUGGGGGCAGAUGUGU---
------- 3
---UAAUGCCCCUAAAAAUCC-------
------- 3   0,-4
---UAAUGCCCCUAAAAAUCCUU-----
------- 2   0,-2
---UAAUGCCCCUAAAAAUCCUUA----
------- 3   0,-1
---UAAUGCCCCUAAAAAUCCUUAU---
------- 10   0,0

>hsa-mir-365-2 mi:29-50;MA:68-89   SRX018957
agaguguucaaggacagcaagaaaaaugAGGGACUUUCAGGGGCAGCUGUguuuucugacucagucaUAAUGCCCCUAAA
AAUCCUUAUuguucuugcagugugcaucggg
...........(.((((((((((.((((((((..(((.(((((((((((.(((....))).)))).....))))))))))
..)))))))).)))))))..))).)......
---------------------------GAGGGACUUUCAGGGGCAGCU--------------------------------
------------------------------- 18   -1,-2
---------------------------GAGGGACUUUCAGGGGCAGCUa-------------------------------
------------------------------- 2   -1,-2
---------------------------GAGGGACUUUCAGGGGCAGCUG-------------------------------
------------------------------- 5   -1,-1
---------------------------GAGGGACUUUCAGGGGCAGCUGU------------------------------
------------------------------- 31   -1,0
---------------------------GAGGGACUUUCAGGGGCAGCUGUau----------------------------
------------------------------- 2   -1,0
---------------------------GAGGGACUUUCAGGGGCAGCUGUu-----------------------------
------------------------------- 7   -1,0
---------------------------GAGGGACUUUCAGGGGCAGCUGUG-----------------------------
------------------------------- 3   -1,1

---------------------------GAGGGACUUUCAGGGGCAGCUGUGU----------------------------
------------------------------- 3 -1,2
----------------------------AGGGACUUUCAGGGGCAGCU--------------------------------
------------------------------- 8 0,-2
----------------------------AGGGACUUUCAGGGGCAGCUG-------------------------------
------------------------------- 7 0,-1
----------------------------AGGGACUUUCAGGGGCAGCUGU------------------------------
------------------------------- 12 0,0
----------------------------AGGGACUUUCAGGGGCAGCUGUa-----------------------------
------------------------------- 2 0,0
----------------------------AGGGACUUUCAGGGGCAGCUGUau----------------------------
------------------------------- 4 0,0
----------------------------AGGGACUUUCAGGGGCAGCUGUG-----------------------------
------------------------------- 78 0,1
----------------------------AGGGACUUUCAGGGGCAGCUGUGa----------------------------
------------------------------- 8 0,1
----------------------------AGGGACUUUCAGGGGCAGCUGUGU---------------------------
------------------------------- 35 0,2
----------------------------AGGGACUUUCAGGGGCAGCUGUGUU--------------------------
------------------------------- 3 0,3
---UAAUGCCCCUAAA
AAUCC-------------------------- 3 0,-4
---UAAUGCCCCUAAA
AAUCCUU------------------------ 2 0,-2
---UAAUGCCCCUAAA
AAUCCUUA----------------------- 3 0,-1
---UAAUGCCCCUAAA
AAUCCUUAU---------------------- 10 0,0

>hsa-mir-3656 3p:49-65 SRX018957
cuuucggccagcgggacggcauccgagguggcuaggcucgggcccguGGCGGGUGCGGGGGUGGgagg
((((.(((.......(.(((((((..(((((((.......)))))))..))))))).).))).)))))
--GGCGGGUGCGGGGGUGGG--- 10 0,1

>hsa-mir-3664 5p:21-42 SRX018957
cuguaaacuugaagguagggAACUCUGUCUUCACUCAUGAGUaccuuccaacacgagcucucaggaguaaagacagaguu
cccuaccuucaauguggau
......((((((((((((((((((((((.((((.((((...(((.......)))..)))).)))).)))))))))))

))))))))))))).))....
--UCUCAGGAGUAAAGACAGAG--
------------------ 3
--UCUCAGGAGUAAAGACAGAGUU
------------------ 11
---CAGGAGUAAAGACAGAGUU
------------------ 6
---CAGGAGUAAAGACAGAGUU
C----------------- 2
---CAGGAGUAAAGACAGAGUU
CCC--------------- 2

>hsa-mir-3676   3p:60-79   SRX018957
uugguuaaagcgccugucuaguaaacaggagauccuggguucgaaucccagcggugccuCCGUGUUUCCCCCACGCUUUu
gccaa
.((((.((((((..((....(.(((((((((((((((((.......))))).)))..)))).))))).)..))))))))).
)))).
--------------------GUAAACAGGAGAUCCUGG--
-----   2

>hsa-mir-3679   5p:6-28;3p:44-65   SRX018957
cguggUGAGGAUAUGGCAGGGAAGGGGAguuucccucuauuccCUUCCCCCCAGUAAUCUUCAUCaug
((((((((((((.(((..((((((((((...........)))))))))).)))...))))))))))))
-----UGAGGAUAUGGCAGGGAAGGGGA--   3   0,0
-----UGAGGAUAUGGCAGGGAAGGGGAa---------------------------------------   2   0,0

>hsa-mir-3687   3p:35-58   SRX018957
cgcgcgugcgcccgagcgcggcccgguggucccuCCCGGACAGGCGUUCGUGCGACGUgug
(((((((.(((.(((((((.(.((((..(...)..)))).)..))))))).))))))))))
-----------------------------------CCGGACAGGCGUUCGUGCGAC-----   3   1,-2

>hsa-mir-369   5p:9-30;3p:44-64   SRX018957
uugaagggAGAUCGACCGUGUUAUAUUCGCuuuauugacuucgAAUAAUACAUGGUUGAUCUUUucucag
.(((..(((((((((((((((..((((((............))))))..)))))))))))))))..))).
--------AGAUCGACCGUGUUAUAUU---   3   0,-3
--------AGAUCGACCGUGUUAUAUUC--   21   0,-2
--------AGAUCGACCGUGUUAUAUUCG---   6   0,-1
--------AGAUCGACCGUGUUAUAUUCGCU---------------------------------------   4   0,1

--------AGAUCGACCGUGUUAUAUUCGCUU--------------------------------------- 4 0,2
--AAUAAUACAUGGUUGAUCUU------- 3 0,-1
--AAUAAUACAUGGUUGAUCUUU------ 14 0,0
--AAUAAUACAUGGUUGAUCUUUU----- 3 0,1

>hsa-mir-3690 5p:10-32 SRX018957
cccaucuccACCUGGACCCAGCGUAGACAAAGagguguuucuacuccauaucuaccuggacccaguguagauggg
(((((((.(((.(((..((((.(((((....(((.........)))....)))))))))..)))))).)))))))
---------ACCUGGACCCAGCGUAGACAA--- 2
0,-2
---------ACCUGGACCCAGCGUAGACAAAGA--- 2
0,1

>hsa-mir-3691 5p:15-37 SRX018957
uugaggcacuggguAGUGGAUGAUGGAGACUCGGUACccacugcugaggguggggaccaagucugcgucauccucuccuc
agugccucaa
.((((((((((((.((.((((((((.(((((.(((.((((((......)))))).))).))))).)))))))).)).)))
))))))))).
---ACCAAGUCUGCGUCAUCCUCUCa--
---------- 2

>hsa-mir-370 3p:48-69 SRX018957
agacagagaagccaggucacgucucugcaguuacacagcucacgaguGCCUGCUGGGGUGGAACCUGGUcugucu
((((((....(((((((..(..(((.((((.(((.(.......).))).)))).)))..)..)))))))))))))
---GCCUGCUGGGGUGGAACCUGa------- 3
0,-2
---GCCUGCUGGGGUGGAACCUGaa------ 2
0,-2
---GCCUGCUGGGGUGGAACCUGGaa----- 2
0,-1

>hsa-mir-372 3p:42-64 SRX018957
gugggccucaaauguggagcacuauucugauguccaaguggAAAGUGCUGCGACAUUUGAGCGUcac
...((((((((((((.(((((((.((((...........)))))))))).).))))))))).)))..
---AAAGUGCUGCGACAUUUGAG------ 4 0,-3
---AAAGUGCUGCGACAUUUGAGC----- 3 0,-2
--AAGUGCUGCGACAUUUGAGCGU--- 2 1,0

>hsa-mir-374a MA:12-33;mi:42-63 SRX018957

uacaucggccaUUAUAAUACAACCUGAUAAGUGuuauagcaCUUAUCAGAUUGUAUUGUAAUUgucugugua

(((((.(((.((((((((((((.(((((((((((....))))))))))).)))))))))))).))).)))))

-----------UUAUAAUACAACCUGAUAA-- 76

0,-3

-----------UUAUAAUACAACCUGAUAAG--- 20

0,-2

-----------UUAUAAUACAACCUGAUAAGa-- 2

0,-2

-----------UUAUAAUACAACCUGAUAAGU-- 182

0,-1

-----------UUAUAAUACAACCUGAUAAGUa--------------------------------------- 21

0,-1

-----------UUAUAAUACAACCUGAUAAGUaa-------------------------------------- 9

0,-1

-----------UUAUAAUACAACCUGAUAAGUG--------------------------------------- 462

0,0

-----------UUAUAAUACAACCUGAUAAGUGa-------------------------------------- 142

0,0

-----------UUAUAAUACAACCUGAUAAGUGaa------------------------------------- 5

0,0

-----------UUAUAAUACAACCUGAUAAGUGU-------------------------------------- 23

0,1

------------UAUAAUACAACCUGAUAAGU-- 2

1,-1

------------UAUAAUACAACCUGAUAAGUG--------------------------------------- 2

1,0

---CUUAUCAGAUUGUAUUGU------------- 4

0,-4

---CUUAUCAGAUUGUAUUGUAA----------- 7

0,-2

---CUUAUCAGAUUGUAUUGUAAU---------- 31

0,-1

---CUUAUCAGAUUGUAUUGUAAUa--------- 2

0,-1

---CUUAUCAGAUUGUAUUGUAAUU--------- 72

0,0

---CUUAUCAGAUUGUAUUGUAAUUa-------- 55

0,0

>hsa-mir-374b MA:11-32;mi:41-62 SRX018957
acucggauggAUAUAAUACAACCUGCUAAGUGuccuagcaCUUAGCAGGUUGUAUUAUCAUUguccgugucu
((.((((..(..((((((((((((((((((((....))))))))))))))))))))..)..)))).))..
----------AUAUAAUACAACCUGCUAAaa--- 2
0,-3
----------AUAUAAUACAACCUGCUAAGU--- 37
0,-1
----------AUAUAAUACAACCUGCUAAGUa-- 4
0,-1
----------AUAUAAUACAACCUGCUAAGUG-- 107
0,0
----------AUAUAAUACAACCUGCUAAGUGa--------------------------------------- 39
0,0
----------AUAUAAUACAACCUGCUAAGUGU--------------------------------------- 10
0,1
--CUUAGCAGGUUGUAUUAUCAU----------- 2
0,-1

>hsa-mir-375 3p:40-61 SRX018957
ccccgcgacgagccccucgcacaaaccggaccugagcguUUUGUUCGUUCGGCUCGCGUGAggc
.((((((.((((((...((.(((((.((........)).))))).))...))))))))).)).
---------------------------------------UUUUGUUCGUUCGGCUCGCGU----- 6 -1,-2
---------------------------------------UUUUGUUCGUUCGGCUCGCGUu---- 2 -1,-2
---------------------------------------UUUUGUUCGUUCGGCUCGCGUG---- 10 -1,-1
---------------------------------------UUUUGUUCGUUCGGCUCGCGUGu--- 2 -1,-1
---------------------------------------UUUUGUUCGUUCGGCUCGCGUGA--- 6 -1,0
--UUUGUUCGUUCGGCUCGC------- 8 0,-4
--UUUGUUCGUUCGGCUCGCG------ 14 0,-3
--UUUGUUCGUUCGGCUCGCGU----- 107 0,-2
--UUUGUUCGUUCGGCUCGCGUu---- 3 0,-2
--UUUGUUCGUUCGGCUCGCGUG---- 549 0,-1
--UUUGUUCGUUCGGCUCGCGUGu--- 23 0,-1
--UUUGUUCGUUCGGCUCGCGUGA--- 472 0,0
--UUUGUUCGUUCGGCUCGCGUGAa-- 18 0,0
--UUUGUUCGUUCGGCUCGCGUGAaa- 9 0,0
--UUUGUUCGUUCGGCUCGCGUGAau- 2 0,0

---------------------------------------UUUGUUCGUUCGGCUCGCGUGAu--   36   0,0
---------------------------------------UUUGUUCGUUCGGCUCGCGUGAuu-   3   0,0
---------------------------------------UUUGUUCGUUCGGCUCGCGUGAG--   3   0,1
--UUGUUCGUUCGGCUCGCGUG----   11   1,-1
--UUGUUCGUUCGGCUCGCGUGA---   6   1,0
--UUGUUCGUUCGGCUCGCGUGAaa-   2   1,0

>hsa-mir-376c   3p:43-63   SRX018957
aaaagguggauauuccuucuauguuuauguuauuuaugguuaAACAUAGAGGAAAUUCCACGUuuu
((((.(((((..(((((.(((((((((.............))))))))))))))..))))).))))
--AACAUAGAGGAAAUUCCAC----- 3   0,-2
--AACAUAGAGGAAAUUCCACG---- 10   0,-1
--AACAUAGAGGAAAUUCCACGU--- 71   0,0
--AACAUAGAGGAAAUUCCACGUa-- 3   0,0
--AACAUAGAGGAAAUUCCACGUU-- 9   0,1
--AACAUAGAGGAAAUUCCACGUUa- 2   0,1
---ACAUAGAGGAAAUUCCACGUUU- 2   1,2

>hsa-mir-377   mi:7-28;MA:45-66   SRX018957
uugagcAGAGGUUGCCCUUGGUGAAUUCgcuuuauuuauguugaAUCACACAAAGGCAACUUUUGUuug
..((((((((((((((.(((((((.(((((.........).)))))))).))).)))))))))))))).
------AGAGGUUGCCCUUGGUGAA--   2   0,-3
------AGAGGUUGCCCUUGGUGAAUU--   32   0,-1
------AGAGGUUGCCCUUGGUGAAUUC---   12   0,0
------AGAGGUUGCCCUUGGUGAAUUCa--   2   0,0
--AUCACACAAAGGCAACUUUUGU---   5   0,0

>hsa-mir-378   mi:5-26;MA:43-63   SRX018957
agggCUCCUGACUCCAGGUCCUGUGUguuaccuagaaauagcACUGGACUUGGAGUCAGAAGGccu
(((.((.((((((((((((((...((((((........)))))).)))))))))))))).)).)))
----CUCCUGACUCCAGGUCCUG--- 2   0,-3
----CUCCUGACUCCAGGUCCUGUGU-- 4   0,0
-----UCCUGACUCCAGGUCCUGUGU-- 27   1,0
-----UCCUGACUCCAGGUCCUGUGUa--------------------------------------- 8   1,0
-----UCCUGACUCCAGGUCCUGUGUaa-------------------------------------- 4   1,0
-----UCCUGACUCCAGGUCCUGUGUau-------------------------------------- 5   1,0
-----UCCUGACUCCAGGUCCUGUGUu--------------------------------------- 3   1,0
-----UCCUGACUCCAGGUCCUGUGUGU-------------------------------------- 4   1,2

--GCACUGGACUUGGAGUCAGAAG----	5	-2,-1
--GCACUGGACUUGGAGUCAGAAGa---	2	-2,-1
---CACUGGACUUGGAGUCAGAAG----	3	-1,-1
---CACUGGACUUGGAGUCAGAAGG---	30	-1,0
---CACUGGACUUGGAGUCAGAAGGa--	5	-1,0
---CACUGGACUUGGAGUCAGAAGGaa-	3	-1,0
---CACUGGACUUGGAGUCAGAAGGu--	2	-1,0
---CACUGGACUUGGAGUCAGAAGGC--	19	-1,1
---CACUGGACUUGGAGUCAGAAGGCa-	20	-1,1
---CACUGGACUUGGAGUCAGAAGGCau	6	-1,1
---CACUGGACUUGGAGUCAGAAGGCu-	7	-1,1
---CACUGGACUUGGAGUCAGAAGGCuu	3	-1,1
--ACUGGACUUGGAGUCAGA------	110	0,-3
--ACUGGACUUGGAGUCAGAu-----	2	0,-3
--ACUGGACUUGGAGUCAGAA-----	340	0,-2
--ACUGGACUUGGAGUCAGAAa----	36	0,-2
--ACUGGACUUGGAGUCAGAAaa---	18	0,-2
--ACUGGACUUGGAGUCAGAAu----	4	0,-2
--ACUGGACUUGGAGUCAGAAG----	1646	0,-1
--ACUGGACUUGGAGUCAGAAGu---	101	0,-1
--ACUGGACUUGGAGUCAGAAGG---	17900	0,0
--ACUGGACUUGGAGUCAGAAGGa--	3653	0,0
--ACUGGACUUGGAGUCAGAAGGaa-	403	0,0
--ACUGGACUUGGAGUCAGAAGGau-	122	0,0
--ACUGGACUUGGAGUCAGAAGGu--	960	0,0
--ACUGGACUUGGAGUCAGAAGGuu-	142	0,0
--ACUGGACUUGGAGUCAGAAGGC--	25487	0,1
--ACUGGACUUGGAGUCAGAAGGCa-	18130	0,1
--ACUGGACUUGGAGUCAGAAGGCaa	1742	0,1
--ACUGGACUUGGAGUCAGAAGGCau	4771	0,1
--ACUGGACUUGGAGUCAGAAGGCu-	10424	0,1
--ACUGGACUUGGAGUCAGAAGGCuu	2987	0,1
--ACUGGACUUGGAGUCAGAAGGCC-	40	0,2
--ACUGGACUUGGAGUCAGAAGGCCa	5	0,2
--ACUGGACUUGGAGUCAGAAGGCCau	10	0,2
--ACUGGACUUGGAGUCAGAAGGCCU	12	0,3
--ACUGGACUUGGAGUCAGAAGGCCUu	5	0,3
---CUGGACUUGGAGUCAGAA-----	2	1,-2

---CUGGACUUGGAGUCAGAAa---- 2 1,-2
---CUGGACUUGGAGUCAGAAG---- 22 1,-1
---CUGGACUUGGAGUCAGAAGG--- 277 1,0
---CUGGACUUGGAGUCAGAAGGa-- 56 1,0
---CUGGACUUGGAGUCAGAAGGaa- 18 1,0
---CUGGACUUGGAGUCAGAAGGau- 3 1,0
---CUGGACUUGGAGUCAGAAGGu-- 23 1,0
---CUGGACUUGGAGUCAGAAGGuu- 3 1,0
---CUGGACUUGGAGUCAGAAGGC-- 1503 1,1
---CUGGACUUGGAGUCAGAAGGCa- 515 1,1
---CUGGACUUGGAGUCAGAAGGCaa 165 1,1
---CUGGACUUGGAGUCAGAAGGCau 144 1,1
---CUGGACUUGGAGUCAGAAGGCu- 640 1,1
---CUGGACUUGGAGUCAGAAGGCuu 478 1,1
---CUGGACUUGGAGUCAGAAGGCC- 10 1,2
---CUGGACUUGGAGUCAGAAGGCCa 5 1,2
---CUGGACUUGGAGUCAGAAGGCCau 7 1,2
---CUGGACUUGGAGUCAGAAGGCCU 3 1,3
---CUGGACUUGGAGUCAGAAGGCCUau 2 1,3
---CUGGACUUGGAGUCAGAAGGCCUu 2 1,3
--UGGACUUGGAGUCAGAAG---- 4 2,-1
--UGGACUUGGAGUCAGAAGG--- 12 2,0
--UGGACUUGGAGUCAGAAGGa-- 5 2,0
--UGGACUUGGAGUCAGAAGGaa- 5 2,0
--UGGACUUGGAGUCAGAAGGC-- 56 2,1
--UGGACUUGGAGUCAGAAGGCa- 18 2,1
--UGGACUUGGAGUCAGAAGGCaa 5 2,1
--UGGACUUGGAGUCAGAAGGCau 6 2,1
--UGGACUUGGAGUCAGAAGGCu- 20 2,1
--UGGACUUGGAGUCAGAAGGCuu 22 2,1

>hsa-mir-378b 3p:34-52 SRX018957
ggucauugagucuucaaggcuaguggaaagagcACUGGACUUGGAGGCAGAAagacc
((((.((..(((((((((.((((((.......)))))).)))))))))..)).))))
---------------------------------ACUGGACUUGGAGGCAGAA----- 199 0,0

>hsa-mir-378c 5p:11-35 SRX018957
ggaggccaucACUGGACUUGGAGUCAGAAGAGUGGagucgggucagacuucaacucugacuuugaagguggugagugccu

c
.((((((((((((....(..((((((((....(((((((......)))))))..))))))))..).)))))))...))))
)
---------CACUGGACUUGGAGUCAGAAG--
-	3	-1,-5
----------ACUGGACUUGGAGUCAGAAG--
-	1646	0,-5
----------ACUGGACUUGGAGUCAGAAGu---
-	101	0,-5
----------ACUGGACUUGGAGUCAGAAGA---
-	810	0,-4
----------ACUGGACUUGGAGUCAGAAGAa--
-	54	0,-4
----------ACUGGACUUGGAGUCAGAAGAaa---
-	11	0,-4
----------ACUGGACUUGGAGUCAGAAGAu--
-	8	0,-4
----------ACUGGACUUGGAGUCAGAAGAuu---
-	2	0,-4
-----------CUGGACUUGGAGUCAGAAG--
-	22	1,-5
-----------CUGGACUUGGAGUCAGAAGA---
-	10	1,-4
------------UGGACUUGGAGUCAGAAG--
-	4	2,-5
------------UGGACUUGGAGUCAGAAGA---
-	3	2,-4

>hsa-mir-379	MA:6-26;mi:44-65	SRX018957
agagaUGGUAGACUAUGGAACGUAGGcguuaugauuucugaccUAUGUAACAUGGUCCACUAACUcu
((((.((((.(((((((..(((((((((..(....)..)).)))))))..))))))).)))).))))
-----UGGUAGACUAUGGAACGUA---	4	0,-2
-----UGGUAGACUAUGGAACGUAG--	39	0,-1
-----UGGUAGACUAUGGAACGUAGG---	119	0,0
-----UGGUAGACUAUGGAACGUAGGa--	27	0,0
-----UGGUAGACUAUGGAACGUAGGaa---------------------------------------	35	0,0
-----UGGUAGACUAUGGAACGUAGGu--	12	0,0

>hsa-mir-381 3p:49-70 SRX018957
uacuuaaagcgagguugcccuuuguauauucgguuuauugacauggaaUAUACAAGGGCAAGCUCUCUGUgagua
((((((.((.(((.((((((((.((((((((.(((....)))...))))))))))))))))).))).)).))))))
-------AGCGAGGUUGCCCUUUGUAU-- 2

>hsa-mir-382 5p:11-32 SRX018957
uacuugaagaGAAGUUGUUCGUGGUGGAUUCGcuuuacuuaugacgaaucauucacggacaacacuuuuuucagua
((((.(((((((.(((((((((((..(((((((.........).))))))..)))))))))))..)))))))))))
----------GAAGUUGUUCGUGGUGGA--
2 0,-4
----------GAAGUUGUUCGUGGUGGAU---
9 0,-3
----------GAAGUUGUUCGUGGUGGAUU--
9 0,-2
----------GAAGUUGUUCGUGGUGGAUUC---
11 0,-1
-----------AAGUUGUUCGUGGUGGAUUCGu---
2 1,0
------------AGUUGUUCGUGGUGGAUUCGCU--
2 2,2
--AAUCAUUCACGGACAACAC-----------
3
--AAUCAUUCACGGACAACACU----------
42
--AAUCAUUCACGGACAACACUU---------
5
--AAUCAUUCACGGACAACACUUU--------
4
--AAUCAUUCACGGACAACACUUUa-------
3

>hsa-mir-383 5p:7-28 SRX018957
cuccucAGAUCAGAAGGUGAUUGUGGCUuuggguggauauuaaucagccacagcacugccuggucagaaagag
(((.((.((((((..((((.((((((((...(((........)))))))))))))))..)))))).))..)))
-----CAGAUCAGAAGGUGAUUG-- 2
-1,-5
-----CAGAUCAGAAGGUGAUUGUGGCa--- 2
-1,-1

-----CAGAUCAGAAGGUGAUUGUGGCU---   5
-1,0
-----CAGAUCAGAAGGUGAUUGUGGCUU--   2
-1,1
-----CAGAUCAGAAGGUGAUUGUGGCUUU---   2
-1,2
------AGAUCAGAAGGUGAUUGUGGC--   2
0,-1
------AGAUCAGAAGGUGAUUGUGGCau--   2
0,-1
------AGAUCAGAAGGUGAUUGUGGCU---   84
0,0
------AGAUCAGAAGGUGAUUGUGGCUau---   20
0,0
------AGAUCAGAAGGUGAUUGUGGCUU--   100
0,1
------AGAUCAGAAGGUGAUUGUGGCUUa---   18
0,1
------AGAUCAGAAGGUGAUUGUGGCUUaa--   3
0,1
------AGAUCAGAAGGUGAUUGUGGCUUU---   81
0,2
------AGAUCAGAAGGUGAUUGUGGCUUUu--   5
0,2
-------GAUCAGAAGGUGAUUGUGGCU---   5
1,0
-------GAUCAGAAGGUGAUUGUGGCUU--   18
1,1
-------GAUCAGAAGGUGAUUGUGGCUUU---   13
1,2

>hsa-mir-3910-1 3p:63-82   SRX018957
cuuuugcugucaguuuuucuguugcuugucuugguuuuaugccuuuuauaucaaggcacauaAAAGGCAUAAAACCAAGA
CAagcaacaaaaaaaggauugaucacagaag
((((((..(((((((((.(((((((((((((((((((((((((((((((..........)))))))))))))))))))))
)))))))))....))))))))))..))))))
--AAAGGCAUAAAACCAAGA
CA----------------------------- 2   0,0

>hsa-mir-3910-2 3p:50-69   SRX018957
uuuuuuuguugcuugucuugguuuuaugccuuuuaugugccuugauauaAAAGGCAUAAAACCAAGACAagcaacagaaa
aa
.(((((((((((((((((((((((((((((((((((((......))))))))))))))))))))))))))))))))))))
).
---AAAGGCAUAAAACCAAGACA-----------
-- 2   0,0

>hsa-mir-3928   3p:37-58   SRX018957
gcugaagcucuaagguuccgccugcgggcaggaagcGGAGGAACCUUGGAGCUUCGGC
(((((((((((((((((((..((((.........)))).)))))))))))))))))))
------------------------------------GGAGGAACCUUGGAGCUUCGG- 7   0,-1
------------------------------------GGAGGAACCUUGGAGCUUCGGC 9   0,0

>hsa-mir-3936   3p:66-87   SRX018957
augauucagagcaucuguccagugucugcuguagaucccucaaauccguguuuggacgcuucuggUAAGGGGUGUAUGGC
AGAUGCAcccgacagaugcacuuggcagca
.((.(..((.(((((((((..((((((((((((.((((((.....((((((....))))....))..)))))).))))))
))))))....))))))))).))..)))...
---UAAGGGGUGUAUGGC
AGAUGCA----------------------- 2   0,0

>hsa-mir-409   5p:15-37;3p:47-68   SRX018957
ugguacucggggagAGGUUACCCGAGCAACUUUGCAUcuggacgacGAAUGUUGCUCGGUGAACCCCUuuucgguauca
.((((.((..(((.((((..(((((((((((((((........).)))).))))))))))..)))).)))..)))))).
--------------AGGUUACCCGAGCAACUU---
    4   0,-5
--------------AGGUUACCCGAGCAACUUU--
    18 0,-4
--------------AGGUUACCCGAGCAACUUUG---
    4   0,-3
--------------AGGUUACCCGAGCAACUUUGC--
    2   0,-2
--------------AGGUUACCCGAGCAACUUUGCA---
    72 0,-1
--------------AGGUUACCCGAGCAACUUUGCAa--
    2   0,-1

--------------AGGUUACCCGAGCAACUUUGCAU--
102 0,0
--------------AGGUUACCCGAGCAACUUUGCAUC---
9 0,1
--------------AGGUUACCCGAGCAACUUUGCAUCa--
2 0,1
--------------AGGUUACCCGAGCAACUUUGCAUCU--
2 0,2
---CGAAUGUUGCUCGGUGAA----------------
13 -1,-5
---CGAAUGUUGCUCGGUGAAC---------------
5 -1,-4
---CGAAUGUUGCUCGGUGAACCC-------------
2 -1,-2
---CGAAUGUUGCUCGGUGAACCCC------------
2 -1,-1
--GAAUGUUGCUCGGUGAAC---------------
5 0,-4
--GAAUGUUGCUCGGUGAACCCC------------
3 0,-1
--GAAUGUUGCUCGGUGAACCCCU-----------
7 0,0
--GAAUGUUGCUCGGUGAACCCCUU----------
2 0,1
---AAUGUUGCUCGGUGAACCCCU-----------
2 1,0

>hsa-mir-410 3p:50-70 SRX018957
gguaccugagaagagguugucugugaugaguucgcuuuuauuaaugacgAAUAUAACACAGAUGGCCUGUuuucaguacc
(((((.((((((.((((..((((((.((.((((((..........).))))).)).))))))..)))).)))))))))))
---AAUAUAACACAGAUGGCCU------------
4 0,-2
---AAUAUAACACAGAUGGCCUGU----------
4 0,0
---AAUAUAACACAGAUGGCCUGUaa--------
2 0,0

>hsa-mir-411 MA:16-36;mi:51-72 SRX018957

ugguacuuggagagaUAGUAGACCGUAUAGCGUACGcuuuaucugugacgUAUGUAACACGGUCCACUAACCcucaguau
caaauccauccccgag
((((((..(((.(.((((.((((((.(.((((((((.........).))))))).).)))))).)))).).))))))))
))..............
--------------AUAGUAGACCGUAUAGCGU--
----------------   31 -1,-3
--------------AUAGUAGACCGUAUAGCGUA---
----------------   19 -1,-2
--------------AUAGUAGACCGUAUAGCGUAa--
----------------   8   -1,-2
--------------AUAGUAGACCGUAUAGCGUAaa---
----------------   2   -1,-2
--------------AUAGUAGACCGUAUAGCGUAC--
----------------   67 -1,-1
--------------AUAGUAGACCGUAUAGCGUACa---
----------------   5   -1,-1
--------------AUAGUAGACCGUAUAGCGUACG---
----------------   85 -1,0
---------------UAGUAGACCGUAUAGCGU--
----------------   29 0,-3
---------------UAGUAGACCGUAUAGCGUA---
----------------   25 0,-2
---------------UAGUAGACCGUAUAGCGUAa--
----------------   2   0,-2
---------------UAGUAGACCGUAUAGCGUAC--
----------------   81 0,-1
---------------UAGUAGACCGUAUAGCGUACa---
----------------   3   0,-1
---------------UAGUAGACCGUAUAGCGUACaa--
----------------   2   0,-1
---------------UAGUAGACCGUAUAGCGUACG---
----------------   61 0,0

>hsa-mir-421   3p:48-70   SRX018957
gcacauuguaggccucauuaaauguuuguugaaugaaaaaaugaaucAUCAACAGACAUUAAUUGGGCGCcugcucugug
aucuc
.((((..((((((((((...(((((((((((.(((.........))))))))))))))...)))).))))))..))))
.....

---AUCAACAGACAUUAAUUG---------------
-----   7   0,-5
---AUCAACAGACAUUAAUUGG--------------
-----   11 0,-4
---AUCAACAGACAUUAAUUGGG-------------
-----   3   0,-3
---AUCAACAGACAUUAAUUGGGCG----------
-----   4   0,-1
---AUCAACAGACAUUAAUUGGGCGCa---------
-----   2   0,0

>hsa-mir-423   5p:17-39;3p:53-75   SRX018957
auaaaggaaguuaggcUGAGGGGCAGAGAGCGAGACUUUucuauuuuccaaaAGCUCGGUCUGAGGCCCCUCAGUcuugc
uuccuaacccgcgc
....(((((((.((((((((((((..(((.((((.(((((.........))))))))).)))...)))))))))))).))
))))).........
---------------CUGAGGGGCAGAGAGCGAGACUUU---
-------------- 3   -1,0
----------------UGAGGGGCAGAGAGCGAG--
-------------- 41 0,-5
----------------UGAGGGGCAGAGAGCGAGu---
-------------- 5   0,-5
----------------UGAGGGGCAGAGAGCGAGA---
-------------- 123 0,-4
----------------UGAGGGGCAGAGAGCGAGAa--
-------------- 20 0,-4
----------------UGAGGGGCAGAGAGCGAGAaa---
-------------- 19 0,-4
----------------UGAGGGGCAGAGAGCGAGAau---
-------------- 2   0,-4
----------------UGAGGGGCAGAGAGCGAGAC--
-------------- 68 0,-3
----------------UGAGGGGCAGAGAGCGAGACa---
-------------- 8   0,-3
----------------UGAGGGGCAGAGAGCGAGACaa--
-------------- 3   0,-3
----------------UGAGGGGCAGAGAGCGAGACU---
-------------- 71 0,-2

----------------UGAGGGGCAGAGAGCGAGACUa--
-------------- 7 0,-2
----------------UGAGGGGCAGAGAGCGAGACUaa---
-------------- 13 0,-2
----------------UGAGGGGCAGAGAGCGAGACUau---
-------------- 11 0,-2
----------------UGAGGGGCAGAGAGCGAGACUU--
-------------- 147 0,-1
----------------UGAGGGGCAGAGAGCGAGACUUa---
-------------- 76 0,-1
----------------UGAGGGGCAGAGAGCGAGACUUaa--
-------------- 12 0,-1
----------------UGAGGGGCAGAGAGCGAGACUUau--
-------------- 24 0,-1
----------------UGAGGGGCAGAGAGCGAGACUUU---
-------------- 1563 0,0
----------------UGAGGGGCAGAGAGCGAGACUUUa--
-------------- 72 0,0
----------------UGAGGGGCAGAGAGCGAGACUUUaa---------------------------------------
-------------- 12 0,0
----------------UGAGGGGCAGAGAGCGAGACUUUau---------------------------------------
-------------- 24 0,0
----------------UGAGGGGCAGAGAGCGAGACUUUU--
-------------- 254 0,1
----------------UGAGGGGCAGAGAGCGAGACUUUUa---------------------------------------
-------------- 4 0,1
----------------UGAGGGGCAGAGAGCGAGACUUUUu---------------------------------------
-------------- 2 0,1
----------------UGAGGGGCAGAGAGCGAGACUUUUC---------------------------------------
-------------- 2 0,2
-----------------GAGGGGCAGAGAGCGAGACUUU---
-------------- 11 1,0
------------------AGGGGCAGAGAGCGAGACUUUU--
-------------- 3 2,1
---AAGCUCGGUCUGAGGCCCCUC--------
-------------- 3 -1,-3
---AAGCUCGGUCUGAGGCCCCUCAa------
-------------- 3 -1,-2

--AAGCUCGGUCUGAGGCCCCUCAu------
-------------- 3 -1,-2
--AAGCUCGGUCUGAGGCCCCUCAG------
-------------- 5 -1,-1
--AAGCUCGGUCUGAGGCCCCUCAGa-----
-------------- 5 -1,-1
--AAGCUCGGUCUGAGGCCCCUCAGU-----
-------------- 37 -1,0
--AAGCUCGGUCUGAGGCCCCUCAGUu----
-------------- 2 -1,0
---AGCUCGGUCUGAGGCCCC----------
-------------- 5 0,-5
---AGCUCGGUCUGAGGCCCCUC--------
-------------- 7 0,-3
---AGCUCGGUCUGAGGCCCCUCA-------
-------------- 10 0,-2
---AGCUCGGUCUGAGGCCCCUCAa------
-------------- 2 0,-2
---AGCUCGGUCUGAGGCCCCUCAaa-----
-------------- 4 0,-2
---AGCUCGGUCUGAGGCCCCUCAau-----
-------------- 4 0,-2
---AGCUCGGUCUGAGGCCCCUCAu------
-------------- 3 0,-2
---AGCUCGGUCUGAGGCCCCUCAG------
-------------- 30 0,-1
---AGCUCGGUCUGAGGCCCCUCAGa-----
-------------- 10 0,-1
---AGCUCGGUCUGAGGCCCCUCAGU-----
-------------- 170 0,0
---AGCUCGGUCUGAGGCCCCUCAGUa----
-------------- 20 0,0
---AGCUCGGUCUGAGGCCCCUCAGUaa---
-------------- 5 0,0
---AGCUCGGUCUGAGGCCCCUCAGUau---
-------------- 30 0,0
---AGCUCGGUCUGAGGCCCCUCAGUu----
-------------- 23 0,0

--AGCUCGGUCUGAGGCCCCUCAGUuu---
-------------- 9 0,0
---GCUCGGUCUGAGGCCCCUCAGU-----
-------------- 4 1,0

>hsa-mir-424 MA:11-32;mi:48-68 SRX018957
cgaggggauaCAGCAGCAAUUCAUGUUUUGAAguguucuaaaugguuCAAAACGUGAGGCGCUGCUAUacccccucgugg
ggaagguagaaggugggg
((((((.((.((((((..((((((((((((.(((.....))).)))))))))))))..)))))).)).)))))))...
....
----------CAGCAGCAAUUCAUGUUU--
------------------ 8 0,-4
----------CAGCAGCAAUUCAUGUUUU---
------------------ 12 0,-3
----------CAGCAGCAAUUCAUGUUUUG--
------------------ 223 0,-2
----------CAGCAGCAAUUCAUGUUUUGA---
------------------ 315 0,-1
----------CAGCAGCAAUUCAUGUUUUGAu--
------------------ 2 0,-1
----------CAGCAGCAAUUCAUGUUUUGAA--
------------------ 71 0,0
----------CAGCAGCAAUUCAUGUUUUGAAa---
------------------ 16 0,0
----------CAGCAGCAAUUCAUGUUUUGAAu---
------------------ 4 0,0
-----------AGCAGCAAUUCAUGUUUUG--
------------------ 8 1,-2
-----------AGCAGCAAUUCAUGUUUUGA---
------------------ 9 1,-1
---CAAAACGUGAGGCGCUGCU--------------
------------------ 5 0,-2
---CAAAACGUGAGGCGCUGCUA-------------
------------------ 44 0,-1
---CAAAACGUGAGGCGCUGCUAa------------
------------------ 4 0,-1
---CAAAACGUGAGGCGCUGCUAU------------
------------------ 430 0,0

---CAAAACGUGAGGCGCUGCUAUu-----------
------------------ 20 0,0
---CAAAACGUGAGGCGCUGCUAUA-----------
------------------ 261 0,1
---CAAAACGUGAGGCGCUGCUAUAa----------
------------------ 70 0,1
---CAAAACGUGAGGCGCUGCUAUAaa---------
------------------ 4   0,1
---CAAAACGUGAGGCGCUGCUAUAu----------
------------------ 35 0,1
--AAAACGUGAGGCGCUGCUAU------------
------------------ 11 1,0
--AAAACGUGAGGCGCUGCUAUA-----------
------------------ 11 1,1
--AAAACGUGAGGCGCUGCUAUAu----------
------------------ 9   1,1
---AAACGUGAGGCGCUGCUAUAu----------
------------------ 3   2,1

>hsa-mir-425   MA:14-36;mi:55-76   SRX018957
gaaagcgcuuuggAAUGACACGAUCACUCCCGUUGAgugggcacccgagaagccAUCGGGAAUGUCGUGUCCGCCCagug
cucuuuc
(((((.((.((((...(((((((.((.(((((.((.((((....)).....)))).))))).)))))))))...)))).)
).)))))
-------------AAUGACACGAUCACUCCCGU---
------- 2   0,-3
-------------AAUGACACGAUCACUCCCGUUG---
------- 10 0,-1
-------------AAUGACACGAUCACUCCCGUUGA--
------- 31 0,0
-------------AAUGACACGAUCACUCCCGUUGAG---
------- 6   0,1
--------------AUGACACGAUCACUCCCGUUGA--
------- 9   1,0
--------------AUGACACGAUCACUCCCGUUGAG---
------- 5   1,1
---CAUCGGGAAUGUCGUGUC---------
------- 2   -1,-5

---CAUCGGGAAUGUCGUGUCC--------
------- 5   -1,-4
---CAUCGGGAAUGUCGUGUCCGu------
------- 2   -1,-3
---CAUCGGGAAUGUCGUGUCCGC------
------- 3   -1,-2
---CAUCGGGAAUGUCGUGUCCGCa-----
------- 2   -1,-2
---CAUCGGGAAUGUCGUGUCCGCC-----
------- 15   -1,-1
--AUCGGGAAUGUCGUGUCCGCC-----
------- 7   0,-1
--AUCGGGAAUGUCGUGUCCGCCC----
------- 2   0,0

>hsa-mir-4286   5p:11-27   SRX018957
uacuuauggcACCCCACUCCUGGUACCauagucauaaguuaggagauguuagagcugugaguaccaugacuuaagugugg
uggcuuaaacaug
...(((.(.((((.(((((.((((((((((((..((((((....))).)))..)))))).)))))).)).....))).))
)).).))).....
----------ACCCCACUCCUGGUACCA--
-------------   7   0,1
----------ACCCCACUCCUGGUACCAa---
-------------   3   0,1
----------ACCCCACUCCUGGUACCAU---
-------------   6   0,2
----------ACCCCACUCCUGGUACCAUu--
-------------   2   0,2

>hsa-mir-429   3p:51-72   SRX018957
cgccggccgaugggcgucuuaccagacaugguuagaccuggcccucugucUAAUACUGUCUGGUAAAACCGUccauccgc
ugc
...((((.((((((((..((((((((((..(((((((..........)))))))..))))))))))...)))))))).))
)).
--UAAUACUGUCUGGUAAAACCG---------
--- 14   0,-1
--UAAUACUGUCUGGUAAAACCGU--------
--- 33   0,0

>hsa-mir-432 MA:14-36;mi:62-82 SRX018957
ugacuccuccaggUCUUGGAGUAGGUCAUUGGGUGGauccucuauuuccuuacgugggccaCUGGAUGGCUCCUCCAUGU
CUuggaguagauca
.((((.(((((((.(.(((((..(((((((.(((((.(((.(((......)).).)))))))).))))))).))))).).
))))))).)).)).
-------------UCUUGGAGUAGGUCAUUG---
-------------- 6 0,-5
-------------UCUUGGAGUAGGUCAUUGaa---
-------------- 2 0,-5
-------------UCUUGGAGUAGGUCAUUGG--
-------------- 67 0,-4
-------------UCUUGGAGUAGGUCAUUGGa---
-------------- 4 0,-4
-------------UCUUGGAGUAGGUCAUUGGaa--
-------------- 7 0,-4
-------------UCUUGGAGUAGGUCAUUGGG---
-------------- 142 0,-3
-------------UCUUGGAGUAGGUCAUUGGGa--
-------------- 9 0,-3
-------------UCUUGGAGUAGGUCAUUGGGaa---
-------------- 5 0,-3
-------------UCUUGGAGUAGGUCAUUGGGU--
-------------- 45 0,-2
-------------UCUUGGAGUAGGUCAUUGGGUa---
-------------- 13 0,-2
-------------UCUUGGAGUAGGUCAUUGGGUaa--
-------------- 15 0,-2
-------------UCUUGGAGUAGGUCAUUGGGUau--
-------------- 26 0,-2
-------------UCUUGGAGUAGGUCAUUGGGUuu--
-------------- 3 0,-2
-------------UCUUGGAGUAGGUCAUUGGGUG---
-------------- 48 0,-1
-------------UCUUGGAGUAGGUCAUUGGGUGa--
-------------- 30 0,-1
-------------UCUUGGAGUAGGUCAUUGGGUGaa---
-------------- 3 0,-1

-------------UCUUGGAGUAGGUCAUUGGGUGau---
-------------- 2   0,-1
-------------UCUUGGAGUAGGUCAUUGGGUGu--
-------------- 19 0,-1
-------------UCUUGGAGUAGGUCAUUGGGUGG--
-------------- 23 0,0
-------------UCUUGGAGUAGGUCAUUGGGUGGu---
-------------- 2   0,0

>hsa-mir-4326   5p:11-30   SRX018957
gcugcucugcUGUUCCUCUGUCUCCCAGACucuggguggauggagcaggucgggggcca
(((.((...(((((((...(((((((((...))))).)))))))))))...)).)))..
----------UGUUCCUCUGUCUCCCAGACU---------------------------- 4   0,1

>hsa-mir-433   3p:64-85   SRX018957
ccggggagaaguacggugagccugucauuauucagagaggcuagauccucuguguugagaaggAUCAUGAUGGGCUCCUC
GGUGUucuccagg
((..((((((...(((.(((((((((((.(((((.(((((......))))).))........))).))))))))))).))
)...)))))).))
---AUCAUGAUGGGCUCCUC
G------------   2   0,-4
---AUCAUGAUGGGCUCCUC
GG-----------   2   0,-3
---AUCAUGAUGGGCUCCUC
GGUG---------   7   0,-1
---AUCAUGAUGGGCUCCUC
GGUGa--------   6   0,-1
---AUCAUGAUGGGCUCCUC
GGUGU--------   72 0,0
---AUCAUGAUGGGCUCCUC
GGUGUa-------   8   0,0
---AUCAUGAUGGGCUCCUC
GGUGUaa------   2   0,0
---AUCAUGAUGGGCUCCUC
GGUGUau------   3   0,0
---AUCAUGAUGGGCUCCUC
GGUGUU-------   6   0,1
--UCAUGAUGGGCUCCUC

GGUG---------   2   1,-1
--UCAUGAUGGGCUCCUC
GGUGU--------   6   1,0

>hsa-mir-449c   MA:17-41;mi:57-79   SRX018957
gcugggaugugucaggUAGGCAGUGUAUUGCUAGCGGCUGUuaaugauuuuaacagUUGCUAGUUGCACUCCUCUCUGUu
gcauucagaagc
(((.(((((((.(((..(((.((((((..((((((((((((((.....)))))))))))))))))))))))))..))).)
))))))...)))
-----------------AGGCAGUGUAUUGCUAGCGGCU---
------------   3   1,-2

>hsa-mir-450a-1 5p:18-39   SRX018957
aaacgauacuaaacuguUUUUGCGAUGUGUUCCUAAUAUgcacuauaaauauauugggaacauuuugcauguauaguuuu
guaucaauaua
....(((((.(((((((...(((((.((((((((((((((.........))))))))))))).)))))...))))))).
)))))......
-----------------UUUUGCGAUGUGUUCCUAAU---
----------- 4   0,-2
-----------------UUUUGCGAUGUGUUCCUAAUA--
----------- 3   0,-1
-----------------UUUUGCGAUGUGUUCCUAAUAU---
----------- 12   0,0
---UUGGGAACAUUUUGCAUGUAUu-----
----------- 2

>hsa-mir-450a-2 5p:23-44   SRX018957
ccaaagaaagaugcuaaacuauUUUUGCGAUGUGUUCCUAAUAUguaauauaaauguauuggggacauuuugcauucaua
guuuuguaucaauaauaugg
.........(((((.(((((((...(((((.(((((((((((((((.......))))))))))))))).)))))...)))
)))).))))).........
----------------------UUUUGCGAUGUGUUCCUAAU--------------------------------------
--------------------   4   0,-2
----------------------UUUUGCGAUGUGUUCCUAAUA-------------------------------------
--------------------   3   0,-1
----------------------UUUUGCGAUGUGUUCCUAAUAU------------------------------------
--------------------   12   0,0
---AUUGGGGACAUUUUGCAUU----

-------------------- 9
--AUUGGGGACAUUUUGCAUUCA--
-------------------- 3
--AUUGGGGACAUUUUGCAUUCAU-
-------------------- 7
---UUGGGGACAUUUUGCAUU----
-------------------- 2
---UUGGGGACAUUUUGCAUUCAU-
-------------------- 3

>hsa-mir-450b 5p:11-32;3p:48-69 SRX018957
gcagaauuauUUUUGCAAUAUGUUCCUGAAUAuguaauauaaguguaUUGGGAUCAUUUUGCAUCCAUAguuuuguau
((((((((((...(((((.(((.((((.((((..(.......)..)))))))).))).)))))...))))))))))..
----------UUUUGCAAUAUGUUCCUG--
10 0,-4
----------UUUUGCAAUAUGUUCCUGA---
6 0,-3
----------UUUUGCAAUAUGUUCCUGAA--
16 0,-2
----------UUUUGCAAUAUGUUCCUGAAa---
3 0,-2
----------UUUUGCAAUAUGUUCCUGAAU---
6 0,-1
----------UUUUGCAAUAUGUUCCUGAAUA--
2 0,0
----------UUUUGCAAUAUGUUCCUGAAUAa---
8 0,0

>hsa-mir-451 5p:17-38 SRX018957
cuugggaauggcaaggAAACCGUUACCAUUACUGAGUUuaguaaugguaaugguucucuugcuauacccaga
.(((((.((((((((..(((((((((((((((((....)))))))))))))))))..)))))))).))))).
----------------AAACCGUUACCAUUACUG-------------------------------------- 2
0,-4
----------------AAACCGUUACCAUUACUGAG------------------------------------ 19
0,-2
----------------AAACCGUUACCAUUACUGAGU----------------------------------- 5
0,-1
----------------AAACCGUUACCAUUACUGAGUU---------------------------------- 126

0,0
----------------AAACCGUUACCAUUACUGAGUUU---------------------------------   27
0,1
----------------AAACCGUUACCAUUACUGAGUUUA--------------------------------   4
0,2
----------------AAACCGUUACCAUUACUGAGUUUAG-------------------------------   5
0,3
-----------------AACCGUUACCAUUACUGAGUU----------------------------------   2
1,0

>hsa-mir-452   MA:14-35;mi:58-79   SRX018957
gcuaagcacuuacAACUGUUUGCAGAGGAAACUGAgacuuuguaacuaugucucaguCUCAUCUGCAAAGAAGUAAGUGc
uuugc
((.((((((((((...(.((((((((.((.((((((((...........)))))))).)).)))))))).).))))))))
)).))
-------------AACUGUUUGCAGAGGAAAC--
-----   7   0,-3
-------------AACUGUUUGCAGAGGAAACU---
-----   23   0,-2
-------------AACUGUUUGCAGAGGAAACUG--
-----   3   0,-1
-------------AACUGUUUGCAGAGGAAACUGA---
-----   2   0,0
-------------AACUGUUUGCAGAGGAAACUGAa--
-----   2   0,0
-------------AACUGUUUGCAGAGGAAACUGAG--
-----   5   0,1
-------------AACUGUUUGCAGAGGAAACUGAGA---
-----   4   0,2

>hsa-mir-455   5p:16-37;3p:54-74   SRX018957
ucccuggcgugagggUAUGUGCCUUUGGACUACAUCGuggaagccagcaccauGCAGUCCAUGGGCAUAUACACuugccu
caaggccuaugucauc
.....(((.(((((((((((((((.((((((.(((.(((........))).))).)))))).)))))))))).....)))
))..))).........
--------------GUAUGUGCCUUUGGACUACA--
----------------   4   -1,-3
--------------GUAUGUGCCUUUGGACUACAU---

--------------- 18 -1,-2

--------------GUAUGUGCCUUUGGACUACAUC---

--------------- 4 -1,-1

--------------GUAUGUGCCUUUGGACUACAUCG--

--------------- 7 -1,0

--------------GUAUGUGCCUUUGGACUACAUCGU---------------------------------------

--------------- 5 -1,1

---------------UAUGUGCCUUUGGACUACA---

--------------- 3 0,-3

---------------UAUGUGCCUUUGGACUACAU--

--------------- 5 0,-2

---------------UAUGUGCCUUUGGACUACAUC---

--------------- 39 0,-1

---------------UAUGUGCCUUUGGACUACAUCa--

--------------- 2 0,-1

---------------UAUGUGCCUUUGGACUACAUCG--

--------------- 127 0,0

---------------UAUGUGCCUUUGGACUACAUCGa---------------------------------------

--------------- 9 0,0

---------------UAUGUGCCUUUGGACUACAUCGU---------------------------------------

--------------- 48 0,1

---------------UAUGUGCCUUUGGACUACAUCGUG--------------------------------------

--------------- 3 0,2

----------------AUGUGCCUUUGGACUACAUCGU---------------------------------------

--------------- 2 1,1

-----------------UGUGCCUUUGGACUACAUCGU---------------------------------------

--------------- 2 2,1

-----------------UGUGCCUUUGGACUACAUCGUG--------------------------------------

--------------- 4 2,2

-----------------UGUGCCUUUGGACUACAUCGUGa-------------------------------------

--------------- 3 2,2

-----------------UGUGCCUUUGGACUACAUCGUGu-------------------------------------

--------------- 5 2,2

---AUGCAGUCCAUGGGCAUA----------

--------------- 3 -2,-5

---AUGCAGUCCAUGGGCAUAU---------

--------------- 11 -2,-4

---AUGCAGUCCAUGGGCAUAUA--------

--------------- 3 -2,-3

---AUGCAGUCCAUGGGCAUAUAa--------

--------------- 2 -2,-3

---AUGCAGUCCAUGGGCAUAUAu--------

--------------- 3 -2,-3

---AUGCAGUCCAUGGGCAUAUAC--------

--------------- 32 -2,-2

---AUGCAGUCCAUGGGCAUAUACA-------

--------------- 56 -2,-1

---AUGCAGUCCAUGGGCAUAUACAa------

--------------- 2 -2,-1

---AUGCAGUCCAUGGGCAUAUACAC------

--------------- 28 -2,0

---AUGCAGUCCAUGGGCAUAUACACa-----

--------------- 2 -2,0

---AUGCAGUCCAUGGGCAUAUACACU-----

--------------- 12 -2,1

--UGCAGUCCAUGGGCAUAU----------

--------------- 2 -1,-4

--UGCAGUCCAUGGGCAUAUAC--------

--------------- 5 -1,-2

--UGCAGUCCAUGGGCAUAUACA-------

--------------- 23 -1,-1

--UGCAGUCCAUGGGCAUAUACAC------

--------------- 13 -1,0

--UGCAGUCCAUGGGCAUAUACACa-----

--------------- 3 -1,0

--UGCAGUCCAUGGGCAUAUACACU-----

--------------- 3 -1,1

---GCAGUCCAUGGGCAUAUAa--------

--------------- 4 0,-3

---GCAGUCCAUGGGCAUAUAu--------

--------------- 3 0,-3

---GCAGUCCAUGGGCAUAUAC--------

--------------- 29 0,-2

---GCAGUCCAUGGGCAUAUACu-------

--------------- 5 0,-2

---GCAGUCCAUGGGCAUAUACA-------

---------------- 186 0,-1

--GCAGUCCAUGGGCAUAUACAa------

---------------- 12 0,-1

--GCAGUCCAUGGGCAUAUACAu------

---------------- 3 0,-1

--GCAGUCCAUGGGCAUAUACAuu-----

---------------- 4 0,-1

--GCAGUCCAUGGGCAUAUACAC------

---------------- 254 0,0

--GCAGUCCAUGGGCAUAUACACa-----

---------------- 65 0,0

--GCAGUCCAUGGGCAUAUACACaa----

---------------- 4 0,0

--GCAGUCCAUGGGCAUAUACACU-----

---------------- 286 0,1

--GCAGUCCAUGGGCAUAUACACUa----

---------------- 9 0,1

--GCAGUCCAUGGGCAUAUACACUau---

---------------- 3 0,1

--GCAGUCCAUGGGCAUAUACACUU----

---------------- 14 0,2

--GCAGUCCAUGGGCAUAUACACUUa---

---------------- 2 0,2

--GCAGUCCAUGGGCAUAUACACUUu---

---------------- 6 0,2

---CAGUCCAUGGGCAUAUACA-------

---------------- 7 1,-1

---CAGUCCAUGGGCAUAUACAC------

---------------- 62 1,0

---CAGUCCAUGGGCAUAUACACU-----

---------------- 61 1,1

---CAGUCCAUGGGCAUAUACACUa----

---------------- 3 1,1

---CAGUCCAUGGGCAUAUACACUU----

---------------- 38 1,2

---CAGUCCAUGGGCAUAUACACUUa---

---------------- 18 1,2

---CAGUCCAUGGGCAUAUACACUUu---

---------------- 36 1,2
--CAGUCCAUGGGCAUAUACACUUuu--
---------------- 3 1,2
---AGUCCAUGGGCAUAUACAC------
---------------- 2 2,0
---AGUCCAUGGGCAUAUACACU-----
---------------- 2 2,1
---AGUCCAUGGGCAUAUACACUU----
---------------- 2 2,2

>hsa-mir-483 5p:8-29;3p:48-68 SRX018957
gaggggggAAGACGGGAGGAAAGAAGGGAGugguuccaucacgccuccUCACUCCUCUCCUCCCGUCUUcuccucuc
(((((((((((((((((((.((.((.(((.(((........)))..))).)).)).))))))))))))).))))))
-------AAGACGGGAGGAAAGAAG---
66 0,-4
-------AAGACGGGAGGAAAGAAGa--
2 0,-4
-------AAGACGGGAGGAAAGAAGG--
175 0,-3
-------AAGACGGGAGGAAAGAAGGa---
13 0,-3
-------AAGACGGGAGGAAAGAAGGaa--
12 0,-3
-------AAGACGGGAGGAAAGAAGGu---
2 0,-3
-------AAGACGGGAGGAAAGAAGGG---
437 0,-2
-------AAGACGGGAGGAAAGAAGGGu--
3 0,-2
-------AAGACGGGAGGAAAGAAGGGuu---
5 0,-2
-------AAGACGGGAGGAAAGAAGGGA--
863 0,-1
-------AAGACGGGAGGAAAGAAGGGAa---
303 0,-1
-------AAGACGGGAGGAAAGAAGGGAaa--
162 0,-1
-------AAGACGGGAGGAAAGAAGGGAau--

62 0,-1

-------AAGACGGGAGGAAAGAAGGGAu--

64 0,-1

-------AAGACGGGAGGAAAGAAGGGAuu---

48 0,-1

-------AAGACGGGAGGAAAGAAGGGAG--

1587 0,0

-------AAGACGGGAGGAAAGAAGGGAGa---

436 0,0

-------AAGACGGGAGGAAAGAAGGGAGaa--

56 0,0

-------AAGACGGGAGGAAAGAAGGGAGau--

33 0,0

-------AAGACGGGAGGAAAGAAGGGAGU---

282 0,1

-------AAGACGGGAGGAAAGAAGGGAGUa--

14 0,1

-------AAGACGGGAGGAAAGAAGGGAGUaa---

13 0,1

-------AAGACGGGAGGAAAGAAGGGAGUau---

26 0,1

-------AAGACGGGAGGAAAGAAGGGAGUu--

37 0,1

-------AAGACGGGAGGAAAGAAGGGAGUuu---

24 0,1

-------AAGACGGGAGGAAAGAAGGGAGUG--

4 0,2

-------AAGACGGGAGGAAAGAAGGGAGUGG---

10 0,3

--------AGACGGGAGGAAAGAAGG---

12 1,-3

--------AGACGGGAGGAAAGAAGGG--

18 1,-2

--------AGACGGGAGGAAAGAAGGGA---

36 1,-1

--------AGACGGGAGGAAAGAAGGGAG--

68 1,0

--------AGACGGGAGGAAAGAAGGGAGa---

3   1,0
--------AGACGGGAGGAAAGAAGGGAGU---
46   1,1
--------AGACGGGAGGAAAGAAGGGAGUu--
2   1,1
--------AGACGGGAGGAAAGAAGGGAGUG--
14   1,2
--------AGACGGGAGGAAAGAAGGGAGUGG---
89   1,3
--------AGACGGGAGGAAAGAAGGGAGUGGU--
4   1,4
--UCACUCCUCUCCUCCCGUCUUC-------
2   0,1
---CACUCCUCUCCUCCCGUCUUC-------
3   1,1
---CACUCCUCUCCUCCCGUCUUCU------
2   1,2

>hsa-mir-484   5p:8-29 SRX018957
agccucgUCAGGCUCAGUCCCCUCCCGAUaaaccccuaaauagggacuuucccggggggugacccuggcuuuuuggcg
.(((..((((((....(((.(((((((..(((.((((....))))..)))..))))))).)))))))))......))).
-------UCAGGCUCAGUCCCCUCCCGuu---
2   0,-2
-------UCAGGCUCAGUCCCCUCCCGA--
7   0,-1
-------UCAGGCUCAGUCCCCUCCCGAa---
15   0,-1
-------UCAGGCUCAGUCCCCUCCCGAaa--
2   0,-1
-------UCAGGCUCAGUCCCCUCCCGAU---
40   0,0
-------UCAGGCUCAGUCCCCUCCCGAUu--
8   0,0
-------UCAGGCUCAGUCCCCUCCCGAUA--
11   0,1
-------UCAGGCUCAGUCCCCUCCCGAUAu---
3   0,1
-------UCAGGCUCAGUCCCCUCCCGAUAA---

3   0,2
---------AGGCUCAGUCCCCUCCCGA---
2   2,-1
---------AGGCUCAGUCCCCUCCCGAU--
2   2,0

>hsa-mir-485   5p:9-30;3p:46-67   SRX018957
acuuggagAGAGGCUGGCCGUGAUGAAUUCgauucaucaaagcgaGUCAUACACGGCUCUCCUCUCUuuuagu
(((..((((((((..(((((((((((.((((...........)))))))).)))))))..))))))))..)))
--------AGAGGCUGGCCGUGAUGA---   4
0,-4
--------AGAGGCUGGCCGUGAUGAA--   13
0,-3
--------AGAGGCUGGCCGUGAUGAAU---   12
0,-2
--------AGAGGCUGGCCGUGAUGAAUU--   276
0,-1
--------AGAGGCUGGCCGUGAUGAAUUa---   6
0,-1
--------AGAGGCUGGCCGUGAUGAAUUu---   6
0,-1
--------AGAGGCUGGCCGUGAUGAAUUC---   86
0,0
--------AGAGGCUGGCCGUGAUGAAUUCu--   4
0,0
--------AGAGGCUGGCCGUGAUGAAUUCG--   121
0,1
--------AGAGGCUGGCCGUGAUGAAUUCGu---   15
0,1
--------AGAGGCUGGCCGUGAUGAAUUCGA---   10
0,2
---GUCAUACACGGCUCUCCUCUCa------   4
0,-1
---GUCAUACACGGCUCUCCUCUCU------   16
0,0

>hsa-mir-486   5p:4-25;3p:46-66   SRX018957
gcaUCCUGUACUGAGCUGCCCCGAGgcccuucaugcugcccagcuCGGGGCAGCUCAGUACAGGAUac

..((((((((((((((((((((((((.........)))....))))))))))))))))))))))..
---UCCUGUACUGAGCUGCCC--- 2 0,-4
---UCCUGUACUGAGCUGCCCC-- 6 0,-3
---UCCUGUACUGAGCUGCCCCG--- 4 0,-2
---UCCUGUACUGAGCUGCCCCGA-- 22 0,-1
---UCCUGUACUGAGCUGCCCCGAa--------------------------------------- 5 0,-1
---UCCUGUACUGAGCUGCCCCGAG--------------------------------------- 61 0,0
---UCCUGUACUGAGCUGCCCCGAGa-------------------------------------- 11 0,0
---UCCUGUACUGAGCUGCCCCGAGaa------------------------------------- 3 0,0
---UCCUGUACUGAGCUGCCCCGAGu-------------------------------------- 15 0,0
-----CUGUACUGAGCUGCCCCGAG--------------------------------------- 2 2,0

>hsa-mir-487a 3p:49-70 SRX018957
gguacuugaagagugguuaucccugcuguguucgcuuaauuuaugacgAAUCAUACAGGGACAUCCAGUUuuucaguauc
(((((.(((((.(((...((((((.((((((((.(((.....))))))).))))))))))...))).))))).))))))
--AAUCAUACAGGGACAUCCAGUU----------
3 0,0

>hsa-mir-487b 3p:51-72 SRX018957
uugguacuuggagagugguuaucccuguccuguucguuuugcucaugucgAAUCGUACAGGGUCAUCCACUUuuucagua
ucaa
.(((((((..((((((((....((((((.(.(((((...........))))).).))))))....))))))))..))))
))).
--AAUCGUACAGGGUCAUCCACU---------
---- 8 0,-1
--AAUCGUACAGGGUCAUCCACUU--------
---- 13 0,0
--AAUCGUACAGGGUCAUCCACUUU-------
---- 2 0,1
---AUCGUACAGGGUCAUCCACUUU-------
---- 4 1,1

>hsa-mir-488 mi:14-34;MA:52-72 SRX018957
gagaaucaucucuCCCAGAUAAUGGCACUCUCAAacaaguuuccaaauuguUUGAAAGGCUAUUUCUUGGUCagaugacu
cuc
((((.((((((..((.(((.((((((.((.((((((((........)))))))).))))))))))).))..)))))).)
)))
-------------CCCAGAUAAUGGCACUCUCA---

--- 5 0,-1
-------------CCCAGAUAAUGGCACUCUCAA--
--- 3 0,0

>hsa-mir-490 5p:39-58;3p:76-97 SRX018957
uggaggccuugcugguuuggaaaguucauuguucgacaCCAUGGAUCUCCAGGUGGGUcaaguuuagagaugcacCAACC
UGGAGGACUCCAUGCUGuugagcuguucacaagcagcggacacuucca
.(((((..((((((.(((((((........((((((((.(((((((((((((((.(((...(((....)))..))).)))
))))))..)))))).))))))))..))).))))))))))...))))).
---CAACC
UGGAGGACUCCAUG---------------------------------- 3 0,-3
---CAACC
UGGAGGACUCCAUGC--------------------------------- 2 0,-2
---CAACC
UGGAGGACUCCAUGCa-------------------------------- 2 0,-2
---CAACC
UGGAGGACUCCAUGCU-------------------------------- 9 0,-1
---CAACC
UGGAGGACUCCAUGCUa------------------------------- 5 0,-1
---CAACC
UGGAGGACUCCAUGCUu------------------------------- 5 0,-1
---CAACC
UGGAGGACUCCAUGCUG------------------------------- 79 0,0
---CAACC
UGGAGGACUCCAUGCUGa------------------------------ 18 0,0
---CAACC
UGGAGGACUCCAUGCUGU------------------------------ 263 0,1
---CAACC
UGGAGGACUCCAUGCUGUa----------------------------- 9 0,1
---CAACC
UGGAGGACUCCAUGCUGUU----------------------------- 20 0,2
---CAACC
UGGAGGACUCCAUGCUGUUu---------------------------- 3 0,2
--AACC
UGGAGGACUCCAUGCUG------------------------------- 2 1,0
--AACC
UGGAGGACUCCAUGCUGU------------------------------ 10 1,1

>hsa-mir-491   5p:16-37;3p:50-71   SRX018957
uugacuuagcugggguAGUGGGGAACCCUUCCAUGAGGaguagaacacucCUUAUGCAAGAUUCCCUUCUACcuggcuggg
uugg
(..(((((((..(((((.((((((..(((.((((((((((.....)))))))))).))).)))))).)))))..))))))
)..)
---------------AGUGGGGAACCCUUCCAUGAG--
----   2   0,-1
---------------AGUGGGGAACCCUUCCAUGAGGA--
----   5   0,1

>hsa-mir-493   mi:16-37;MA:57-78   SRX018957
cuggccuccagggcuUUGUACAUGGUAGGCUUUCAUUcauucguuugcacauucggUGAAGGUCUACUGUGUGCCAGGcc
cugugccag
((((...((((((((.((((((((((((((((((((.....((....))....))))))))))))))))))).)))))
))).)))))
---------------UUGUACAUGGUAGGCUUUCA---
---------   2   0,-2
---------------UUGUACAUGGUAGGCUUUCAU--
---------   2   0,-1
---------------UUGUACAUGGUAGGCUUUCAUU---
---------   12   0,0
-----------------GUACAUGGUAGGCUUUCAUU---
---------   2   2,0
--UGAAGGUCUACUGUGUGCC-----
---------   2   0,-3
--UGAAGGUCUACUGUGUGCCAa---
---------   3   0,-2
--UGAAGGUCUACUGUGUGCCAG---
---------   6   0,-1
--UGAAGGUCUACUGUGUGCCAGa--
---------   9   0,-1
--UGAAGGUCUACUGUGUGCCAGaa-
---------   2   0,-1
--UGAAGGUCUACUGUGUGCCAGG--
---------   8   0,0
--UGAAGGUCUACUGUGUGCCAGGa-
---------   4   0,0
--UGAAGGUCUACUGUGUGCCAGGu-

---------   2   0,0

>hsa-mir-494   3p:48-69   SRX018957
gauacucgaaggagagguuguccguguugucuucucuuuauuuaugaUGAAACAUACACGGGAAACCUCuuuuuuaguau
c
((((((.((((((((((((..(((((((((.(((((.........)).)))))).))))))..)))))))))))))))))
)
---UGAAACAUACACGGGAAAC--------------
-   2   0,-3
---UGAAACAUACACGGGAAACCU------------
-   6   0,-1
---UGAAACAUACACGGGAAACCUC-----------
-   5   0,0
---UGAAACAUACACGGGAAACCUCU----------
-   29   0,1
--GAAACAUACACGGGAAACCU------------
-   2   1,-1

>hsa-mir-495   3p:50-71   SRX018957
ugguaccugaaaagaaguugcccauguuauuuucgcuuuauaugugacgAAACAAACAUGGUGCACUUCUUuuucgguau
ca
.((((((.((((((((((.(((((((((..((((((.........).)))))..))))))).))))))))))))).)))))
).
---AAACAAACAUGGUGCACUU------------
--   2   0,-3
---AAACAAACAUGGUGCACUUCUU---------
--   13   0,0
---AAACAAACAUGGUGCACUUCUUa--------
--   3   0,0
---AAACAAACAUGGUGCACUUCUUU--------
--   6   0,1
--AACAAACAUGGUGCACUUCUUa--------
--   2   1,0
--AACAAACAUGGUGCACUUCUUU--------
--   5   1,1

>hsa-mir-496   3p:56-77   SRX018957
cccaagucagguacucgaauggagguuguccauggugugguucauuuuauuuaugaUGAGUAUUACAUGGCCAAUCUCcuu

ucgguacucaauucuucuuggg
((((((...(((((.((((.((((((((.(((((..(((((((((........)))))))))..))))).)))))))).)
))))))))........))))))
---UGAGUAUUACAUGGCCAAU------
---------------------- 2 0,-3
---UGAGUAUUACAUGGCCAAUCU----
---------------------- 3 0,-1

>hsa-mir-497 MA:24-44;mi:64-85 SRX018957
ccaccccgguccugcucccgcccCAGCAGCACACUGUGGUUUGUacggcacuguggccacgucCAAACCACACUGUGGUG
UUAGAgcgagggugggggaggcaccgccgagg
((.(..(((((((.((((((((((((((.((((.(((((((((.(((((......)))..)).))))))))).)))).))
)).....).)))))))))))).))))..).))
-----------------------CAGCAGCACACUGUGGUU---------------------------------------
-------------------------------- 2 0,-3
-----------------------CAGCAGCACACUGUGGUUa--------------------------------------
-------------------------------- 2 0,-3
-----------------------CAGCAGCACACUGUGGUUU--------------------------------------
-------------------------------- 17 0,-2
-----------------------CAGCAGCACACUGUGGUUUG-------------------------------------
-------------------------------- 25 0,-1
-----------------------CAGCAGCACACUGUGGUUUGa------------------------------------
-------------------------------- 2 0,-1
-----------------------CAGCAGCACACUGUGGUUUGU------------------------------------
-------------------------------- 68 0,0
-----------------------CAGCAGCACACUGUGGUUUGUA-----------------------------------
-------------------------------- 61 0,1
-----------------------CAGCAGCACACUGUGGUUUGUAa----------------------------------
-------------------------------- 26 0,1
-----------------------CAGCAGCACACUGUGGUUUGUAu----------------------------------
-------------------------------- 15 0,1
------------------------AGCAGCACACUGUGGUUU--------------------------------------
-------------------------------- 9 1,-2
------------------------AGCAGCACACUGUGGUUUG-------------------------------------
-------------------------------- 23 1,-1
------------------------AGCAGCACACUGUGGUUUGU------------------------------------
-------------------------------- 6 1,0
------------------------AGCAGCACACUGUGGUUUGUA-----------------------------------

--------------------------------   15   1,1
------------------------AGCAGCACACUGUGGUUUGUAa----------------------------------
--------------------------------   14   1,1
------------------------AGCAGCACACUGUGGUUUGUAu----------------------------------
--------------------------------   5   1,1
------------------------AGCAGCACACUGUGGUUUGUAC----------------------------------
--------------------------------   11   1,2
------------------------AGCAGCACACUGUGGUUUGUACa---------------------------------
--------------------------------   4   1,2

>hsa-mir-499   5p:33-53;3p:70-91   SRX018957
gcccuguccccugugccuugggcgggcggcugUUAAGACUUGCAGUGAUGUUUaacuccucuccacgugAACAUCACAGC
AAGUCUGUGCUgcuucccgucccuacgcugccugggcagggu
(((((((((.....((...(((((((.(((.((..((((((((.((((((((((.............)))))))))).))
))))))..)).))).)))).)))...)).....)))))))))
--------------------------------UUAAGACUUGCAGUGAUGU-----------------------------
-- 2   0,-2
--------------------------------UUAAGACUUGCAGUGAUGUU----------------------------
-- 19   0,-1
--------------------------------UUAAGACUUGCAGUGAUGUUa---------------------------
-- 8   0,-1
--------------------------------UUAAGACUUGCAGUGAUGUUU---------------------------
-- 419 0,0
--------------------------------UUAAGACUUGCAGUGAUGUUUu--------------------------
-- 6   0,0
--------------------------------UUAAGACUUGCAGUGAUGUUUA--------------------------
-- 237 0,1
--------------------------------UUAAGACUUGCAGUGAUGUUUAu-------------------------
-- 3   0,1
--------------------------------UUAAGACUUGCAGUGAUGUUUAA-------------------------
-- 14   0,2

>hsa-mir-500a   MA:13-35;mi:52-73   SRX018957
gcucccccucucUAAUCCUUGCUACCUGGGUGAGAgugcugucugaaugcaAUGCACCUGGGCAAGGAUUCUGagagcga
gagc
((((.(.(((((.(((((((((...(..((((....(((.........)))...))))..))))))))))..))))).).
))))
--AAUGCACCUGGGCAAGGA------------

----   9   -1,-5
--AAUGCACCUGGGCAAGGAU-----------
----   14 -1,-4
--AAUGCACCUGGGCAAGGAUU----------
----   40 -1,-3
--AAUGCACCUGGGCAAGGAUUC---------
----   6   -1,-2
---AUGCACCUGGGCAAGGAU-----------
----   25 0,-4
---AUGCACCUGGGCAAGGAUU----------
----   166 0,-3
---AUGCACCUGGGCAAGGAUUC---------
----   21 0,-2
---AUGCACCUGGGCAAGGAUUCU--------
----   6   0,-1
---AUGCACCUGGGCAAGGAUUCUGu------
----   5   0,0
---AUGCACCUGGGCAAGGAUUCUGA------
----   36 0,1

>hsa-mir-501   5p:14-35;3p:51-72   SRX018957
gcucuuccucucuAAUCCUUUGUCCCUGGGUGAGAgugcuuucugaaugcAAUGCACCCGGGCAAGGAUUCUgagagggu
gagc
((((.(((((((.(((((((.(.(((.(((((....(((.........)))...))))))))))))))))..))))))).
))))
--AAUGCACCCGGGCAAGGA------------
----   5   0,-4
--AAUGCACCCGGGCAAGGAU-----------
----   3   0,-3
--AAUGCACCCGGGCAAGGAUU----------
----   21 0,-2
--AAUGCACCCGGGCAAGGAUUC---------
----   3   0,-1
---AUGCACCCGGGCAAGGAUU----------
----   3   1,-2

>hsa-mir-502   5p:16-36;3p:52-73   SRX018957
ugcucccccucucuaAUCCUUGCUAUCUGGGUGCUAgugcuggcucaaugcAAUGCACCUGGGCAAGGAUUCAgagaggg

ggagcu
.((((((((((((((((((((((...(..(((((...(((.........)))..)))))..)))))))))).))))))))
))))).
---AAUGCACCUGGGCAAGGA-----------
------ 9 0,-4
---AAUGCACCUGGGCAAGGAU----------
------ 14 0,-3
---AAUGCACCUGGGCAAGGAUU---------
------ 40 0,-2
---AAUGCACCUGGGCAAGGAUUC--------
------ 6 0,-1
---AAUGCACCUGGGCAAGGAUUCA-------
------ 13 0,0
---AAUGCACCUGGGCAAGGAUUCAG------
------ 8 0,1
--AUGCACCUGGGCAAGGAU----------
------ 25 1,-3
--AUGCACCUGGGCAAGGAUU---------
------ 166 1,-2
--AUGCACCUGGGCAAGGAUUC--------
------ 21 1,-1

>hsa-mir-503 5p:6-28 SRX018957
ugcccUAGCAGCGGGAACAGUUCUGCAGugagcgaucggugcucugggguauuguuuccgcugccagggua
((((((.((((((((((((((.((.((..(((((.....))))))).)).)))))))))))))).))))))
-----UAGCAGCGGGAACAGUUC-- 257 0,-5
-----UAGCAGCGGGAACAGUUCa--- 27 0,-5
-----UAGCAGCGGGAACAGUUCaa-- 18 0,-5
-----UAGCAGCGGGAACAGUUCU--- 314 0,-4
-----UAGCAGCGGGAACAGUUCUa-- 47 0,-4
-----UAGCAGCGGGAACAGUUCUaa--- 95 0,-4
-----UAGCAGCGGGAACAGUUCUau--- 4 0,-4
-----UAGCAGCGGGAACAGUUCUG-- 21 0,-3
-----UAGCAGCGGGAACAGUUCUGa--- 3 0,-3
-----UAGCAGCGGGAACAGUUCUGaa-- 9 0,-3
-----UAGCAGCGGGAACAGUUCUGCA-- 4 0,-1
-----UAGCAGCGGGAACAGUUCUGCAa--- 5 0,-1
-----UAGCAGCGGGAACAGUUCUGCAu--- 2 0,-1

-----UAGCAGCGGGAACAGUUCUGCAG--- 55 0,0
-----UAGCAGCGGGAACAGUUCUGCAGa-- 2 0,0
------AGCAGCGGGAACAGUUCU-- 2 1,-4
------AGCAGCGGGAACAGUUCUGCAG--- 3 1,0

>hsa-mir-504 5p:13-34 SRX018957
gcugcuguugggAGACCCUGGUCUGCACUCUAUCuguauucuuacugaagggagugcagggcaggguuucccauacagag
ggc
(((.((((((((((((((((.((((((((((.((.(((....))).))..)))))))))).)))))))))))).))))..
)))
------------AGACCCUGGUCUGCACUCUAU---
--- 13 0,-1
------------AGACCCUGGUCUGCACUCUAUC--
--- 8 0,0
------------AGACCCUGGUCUGCACUCUAUCa---
--- 2 0,0
-------------GACCCUGGUCUGCACUCUAUC--
--- 3 1,0

>hsa-mir-505 mi:15-36;MA:51-72 SRX018957
gaugcacccaguggGGGAGCCAGGAAGUAUUGAUGUuucugccaguuuagCGUCAACACUUGCUGGUUUCCUcucuggag
cauc
(((((..((((.(((((((((((.((((.((((((((...........)))))))).)))).))))))))))).)))).)
))))
--------------GGGAGCCAGGAAGUAUUGAU--
---- 5 0,-2
--------------GGGAGCCAGGAAGUAUUGAUa---
---- 5 0,-2
--------------GGGAGCCAGGAAGUAUUGAUG---
---- 19 0,-1
--------------GGGAGCCAGGAAGUAUUGAUGa--
---- 2 0,-1
--------------GGGAGCCAGGAAGUAUUGAUGU--
---- 124 0,0
--------------GGGAGCCAGGAAGUAUUGAUGUa---
---- 11 0,0
--------------GGGAGCCAGGAAGUAUUGAUGUU---
---- 317 0,1

--------------GGGAGCCAGGAAGUAUUGAUGUUa--
----	11	0,1
--------------GGGAGCCAGGAAGUAUUGAUGUUU--
----	28	0,2
---GUCAACACUUGCUGGUUUCCUC-------
----	3	1,1
--UCAACACUUGCUGGUUUCCUC-------
----	2	2,1

>hsa-mir-509-3	5p:10-31;3p:44-65	SRX018957
gugguacccUACUGCAGACGUGGCAAUCAUGuauaauuaaaaaUGAUUGGUACGUCUGUGGGUAGaguacugcau
(..((((.((((..((((((((.(((((((.((....))...))))))).))))))))..).))).))))..)..
---------UACUGCAGACGUGGCAAUCAUG-- 2
	0,0

>hsa-mir-511-1	5p:16-36	SRX018957
caauagacacccaucGUGUCUUUUGCUCUGCAGUCAguaaauauuuuuuugugaauguguagcaaaagacagaauggugg
uccauug
((((.(((..((((..((((((((((((..(((.(((.((((......)))))).)))..)))))))))))..))))..)
)).))))
---------------GUGUCUUUUGCUCUGCAGUCA--
------- 7	0,0
---UGAAUGUGUAGCAAAAGACAGA-------
------- 5
---AAUGUGUAGCAAAAGACAG--------
------- 2
---AAUGUGUAGCAAAAGACAGA-------
------- 45
---AAUGUGUAGCAAAAGACAGAA------
------- 14
---AAUGUGUAGCAAAAGACAGAAa-----
------- 2
---AAUGUGUAGCAAAAGACAGAAU-----
------- 7

>hsa-mir-511-2	5p:16-36	SRX018957
caauagacacccaucGUGUCUUUUGCUCUGCAGUCAguaaauauuuuuuugugaauguguagcaaaagacagaauggugg
uccauug

((((.(((..((((..((((((((((..(((.(((.((((......)))))).)))..))))))))))..)))).)
)).))))
---------------GUGUCUUUUGCUCUGCAGUCA---
------- 7   0,0
--UGAAUGUGUAGCAAAAGACAGA-------
------- 5
--AAUGUGUAGCAAAAGACAG--------
------- 2
--AAUGUGUAGCAAAAGACAGA-------
------- 45
--AAUGUGUAGCAAAAGACAGAA------
------- 14
--AAUGUGUAGCAAAAGACAGAAa-----
------- 2
--AAUGUGUAGCAAAAGACAGAAU-----
------- 7

>hsa-mir-532   5p:20-41;3p:57-78   SRX018957
cgacuugcuuucucuccucCAUGCCUUGAGUGUAGGACCGUuggcaucuuaauuacCCUCCCACACCCAAGGCUUGCAaa
aaagcgagccu
.(.((((((((.......((.((((((.((((.(((..((.............))..))).)))).)))))).))....
)))))))).).
-------------------CAUGCCUUGAGUGUAGGA--
----------- 3   0,-4
-------------------CAUGCCUUGAGUGUAGGACCGU--
----------- 54   0,0
-------------------CAUGCCUUGAGUGUAGGACCGUa---
----------- 11   0,0
-------------------CAUGCCUUGAGUGUAGGACCGUU---
----------- 4   0,1
---CCUCCCACACCCAAGGCUU-----
----------- 5   0,-3
---CCUCCCACACCCAAGGCUUGC---
----------- 8   0,-1
---CCUCCCACACCCAAGGCUUGCA--
----------- 40   0,0
---CCUCCCACACCCAAGGCUUGCAu-
----------- 4   0,0

--CCUCCCACACCCAAGGCUUGCAA-
----------- 6 0,1

>hsa-mir-539 5p:9-30 SRX018957
auacuugaGGAGAAAUUAUCCUUGGUGUGUucgcuuuauuuaugaugaaucauacaaggacaauuucuuuugaguau
(((((..((((((((((.((((((.(((((((((.........).)))).)))))))))).))))))))))..)))))
--------GGAGAAAUUAUCCUUGGUGUGU---
2 0,0

>hsa-mir-542 5p:16-38;3p:53-74 SRX018957
cagaucucagacaucUCGGGGAUCAUCAUGUCACGAGAuaccagugugcacuUGUGACAGAUUGAUAACUGAAAggucug
ggagccacucaucuuca
....((((((((...((((..((((...(((((((((.(((....)))..)))))))))...))))..))))...)))))
)))..............
---------------UCGGGGAUCAUCAUGUCACG---
----------------- 2 0,-3
---UUGUGACAGAUUGAUAACU----------
----------------- 2 -1,-4
---UUGUGACAGAUUGAUAACUG---------
----------------- 2 -1,-3
---UUGUGACAGAUUGAUAACUGA--------
----------------- 2 -1,-2
--UGUGACAGAUUGAUAACU----------
----------------- 16 0,-4
--UGUGACAGAUUGAUAACUG---------
----------------- 14 0,-3
--UGUGACAGAUUGAUAACUGA--------
----------------- 11 0,-2
--UGUGACAGAUUGAUAACUGAA-------
----------------- 24 0,-1
--UGUGACAGAUUGAUAACUGAAA------
----------------- 26 0,0

>hsa-mir-543 3p:47-68 SRX018957
uacuuaaugagaaguugcccguguuuuuuucgcuuuauuugugacgAAACAUUCGCGGUGCACUUCUUuuucaguauc
((((.((.(((((((.(((((((.((((.((((.......)))).))))....))))).))))))))).)).))))..
--AAACAUUCGCGGUGCACUU-------------
2 0,-3

--AAACAUUCGCGGUGCACUUCUU----------
    5   0,0
--AAACAUUCGCGGUGCACUUCUUU---------
    3   0,1

>hsa-mir-548a-1 3p:61-82   SRX018957
ugcagggagguauuaaguuggugcaaaaguaauugugauuuuugccauuaaaaguaacgaCAAAACUGGCAAUUACUUUU
GCaccaaaccugguauu
..((((...........(((((((((((((((((((.(.(((((.(.(((....))).).))))).).))))))))))))
))))))).)))).....
--CAAAACUGGCAAUUACUUUU
G----------------   4   0,-1

>hsa-mir-548a-2 3p:61-82   SRX018957
ugugauguguauuagguuugugcaaaaguaauugggguuuuugccguuaaaaguaauggCAAAACUGGCAAUUACUUUU
GCaccaaacuaauauaa
.......((((((((....(((((((((((((((.(((((..((((((((....)))))))))))))..)))))))))))
))))....)))))))).
--CAAAACUGGCAAUUACUUUU
G----------------   4   0,-1

>hsa-mir-548a-3 5p:25-46;3p:61-82   SRX018957
ccuagaauguuauuaggucggugcAAAAGUAAUUGCGAGUUUUACCauuacuuucaauggCAAAACUGGCAAUUACUUUU
GCaccaacguaauacuu
..........(((((.((.(((((((((((((((((.((((((.(((((......))))).)))))).))))))))))))
))))).)).)))))...
--CAAAACUGGCAAUUACUUUU
G----------------   4   0,-1

>hsa-mir-548b   5p:25-46;3p:61-82   SRX018957
cagacuauauauuuagguuggcgcAAAAGUAAUUGUGGUUUUGGCCuuuauuuucaauggCAAGAACCUCAGUUGCUUUU
GUgccaaccuaauacuu
............(((((((((((((((((((((((.((((((.(((............))).)))))).)))))))))))
))))))))))))).....
------------------------AAAAGUAAUUGUGGUUUUGGCa----------------------------------
-----------------   5   0,-1
------------------------AAAAGUAAUUGUGGUUUUGGCau---------------------------------
-----------------   2   0,-1

>hsa-mir-548c   5p:25-46;3p:61-82   SRX018957
cauuggcaucuauuagguuggugcAAAAGUAAUUGCGGUUUUUGCCauuacuuucaguagCAAAAAUCUCAAUUACUUUU
GCaccaacuuaauacuu
..........(((((((((((((((((((((((((.(((((((((...(((.....))))))))))).))))))))))))
))))))))))))))...
------------------------AAAAGUAAUUGCGGUUUUUG------------------------------------
-----------------   2   0,-2

>hsa-mir-548e   3p:53-74   SRX018957
uuauuagguugguacaaaagcaaucgcgguuuuugcuauuacuuuuaaaggcAAAAACUGAGACUACUUUUGCAccaacc
ugauagaa
(((((((((((((.((((((...((.(((((((((((.(((....))).))))))))))).))...)))))).)))))))
))))))..
--AAAAACUGAGACUACUUUUGC-------
--------   3   0,-1
--AAAAACUGAGACUACUUUUGCA------
--------   20   0,0
--AAAAACUGAGACUACUUUUGCAa-----
--------   5   0,0

>hsa-mir-548f-1 3p:51-69   SRX018957
auuagguuggugcaaaaguaaucacaguuuuugacauuacuuucaaagacAAAAACUGUAAUUACUUUUggaccaaccua
auag
(((((((((((.((((((((((.((((((((((................)))))))))).)))))))))).)))))))))
))..
-UUAGGUUGGUGCAAAAGUAAU--
----   2

>hsa-mir-548f-2 3p:59-77   SRX018957
uaauaacuauuagguuggugcgaacauaauugcaguuuuuaucauuacuuuuaauggcAAAAACUGUAAUUACUUUUgca
ccaaccuaauauuuuagu
.......(((((((((((((((((..((((((((((((((..(((((....)))))..))))))))))))))..))))))
))))))))))).......
---CAAAAACUGUAAUUACUUUUGC-
------------------   4   -1,2

>hsa-mir-548f-3 3p:54-72   SRX018957

auuagguuggugcaaaccuaauugcaauuuuugcaguuuuuuuaaguaauugcAAAAACUGUAAUUACUUUUgcaccaac
cuaauac
((((((((((((((((..((((((((.(((((((((((.........))))))))))).))))))))..)))))))))))
)))))..
--CAAAAACUGUAAUUACUUUUGC------
------- 4   -1,2

>hsa-mir-548f-4 3p:63-81   SRX018957
gaguucuaacguauuagguuggugcaaaaguaauagugguuuuugccauuaaaaguaaugacAAAAACUGUAAUUACUUU
Uggaacaauauuaauagaauuucag
((((((((...((....((((.(.((((((((((.(..(((((((.(((((....))))).)))))))..).))))))))
)).).))))..)).))))))))...
-------------UUAGGUUGGUGCAAAAGUAAU--
-------------------------   2

>hsa-mir-548h-1 5p:21-42   SRX018957
ucuguccauuaggugggugcAAAAGUAAUCGCGGUUUUUGUCauuacuuuuaaugguaaaaacuggaauuacuuuugcac
ugaccuaauauuaagccagaua
((((.(.((((((.(((((((((((((((..((((((((..(((((....)))))..))))))))..)))))))))))))
).))))))).....).))))..
--------------------AAAAGUAAUCGCGGUUUUUG--
---------------------- 2   0,-2
----------------------AAGUAAUCGCGGUUUUUG--
---------------------- 2   2,-2

>hsa-mir-548h-2 5p:16-37   SRX018957
guauuagguuggugcAAAAGUAAUCGCGGUUUUUGUCauuacuuucaauggcaaacaccacaauuacuuuugcaccaacc
uaauauaa
((((((((((((((((((((((((.(.(((.(((((((((......))))))))).))).).))))))))))))))))))
))))))..
---------------AAAAGUAAUCGCGGUUUUUG---
--------   2   0,-2
-----------------AAGUAAUCGCGGUUUUUG---
--------   2   2,-2

>hsa-mir-548h-3 5p:29-50   SRX018957
ucugauucugcauguauuagguuggugcAAAAGUAAUCGCGGUUUUUGUCauugaaaguaauagcaaaaacugcaauuac
uuuugcaccaaccuaaaaguagucacugucuucagaua

(((((..((((.....(((((((((((((((((((((.((((((((((..((((....))))..)))))))))).)))))
))))))))))))))))..)))).........)))))..
----------------------------AAAAGUAAUCGCGGUUUUUG--------------------------------
-------------------------------------- 2   0,-2
------------------------------AAGUAAUCGCGGUUUUUG--------------------------------
-------------------------------------- 2   2,-2
--CAAAAACUGCAAUUAC
UUU----------------------------------- 5
--CAAAAACUGCAAUUAC
UUUU---------------------------------- 7
--CAAAAACUGCAAUUAC
UUUUG--------------------------------- 20
--CAAAAACUGCAAUUAC
UUUUGa-------------------------------- 15
--CAAAAACUGCAAUUAC
UUUUGaa------------------------------- 2
--CAAAAACUGCAAUUAC
UUUUGu-------------------------------- 2
--CAAAAACUGCAAUUAC
UUUUGC-------------------------------- 3
---AAAAACUGCAAUUAC
UUUU---------------------------------- 4
---AAAAACUGCAAUUAC
UUUUG--------------------------------- 21
---AAAAACUGCAAUUAC
UUUUGaa------------------------------- 2
---AAAAACUGCAAUUAC
UUUUGC-------------------------------- 3

>hsa-mir-548h-4 5p:17-38   SRX018957
gcuauuagguuggugcAAAAGUAAUCGCGGUUUUUGUCauuacuuuaauuacuuuacguuucauuaaugacaaaaaccgc
aauuacuuuugcaccaaccuaauacuugcua
(((((((((((((((((((((((((.((((((((((((((((....(((........)))....))))))))))))))))
.)))))))))))))))))))))))...))..
----------------AAAAGUAAUCGCGGUUUUUG--
------------------------------- 2   0,-2
------------------AAGUAAUCGCGGUUUUUG--
------------------------------- 2   2,-2

>hsa-mir-548j   5p:29-50   SRX018957
gggcagccagugaauaguuagcuggugcAAAAGUAAUUGCGGUCUUUGGUauuacuuucaguggcaaaaacugcauuacu
uuugcaccagccuacuagaacgcugaguucag
((((...(((((..((((..(((((((((((((((((.(((((.((((.((((......)))).)))).)))))))))))))))
)))))))))))..))))...))))).))))..
---------------------------CAAAAGUAAUUGCGGUCUUU---------------------------------
--------------------------------   3   -1,-3
---------------------------CAAAAGUAAUUGCGGUCUUUGG-------------------------------
--------------------------------   5   -1,-1
---------------------------CAAAAGUAAUUGCGGUCUUUGGU------------------------------
--------------------------------   3   -1,0
----------------------------AAAAGUAAUUGCGGUCUUUGG-------------------------------
--------------------------------   15   0,-1
----------------------------AAAAGUAAUUGCGGUCUUUGGU------------------------------
--------------------------------   11   0,0
----------------------------AAAAGUAAUUGCGGUCUUUGGUA-----------------------------
--------------------------------   2   0,1
--CAAAAACUGCAUUACU
UUUG----------------------------   2
--CAAAAACUGCAUUACU
UUUGC---------------------------   5

>hsa-mir-548k   5p:32-53   SRX018957
cuuuucucaaguauugcuguuagguuggugcAAAAGUACUUGCGGAUUUUGCUuuacuuuuaauggcaaaaaccgcaauu
auuuuugcuucaaccuaauaugaugcaaaauuggcu
..........((((((.(((((((((((.(((((((((.((((((.(((((((...........))))))).)))))).)
)))))))).)))))))))))))))))..........
-------------------------------AAAAGUACUUGCGGAUUUUGC----------------------------
------------------------------------   3   0,-1
-------------------------------AAAAGUACUUGCGGAUUUUGCU---------------------------
------------------------------------   10   0,0

>hsa-mir-548o   3p:87-108   SRX018957
uggugaaaauguguugauuguaaugguuccuauucugaucaauaaacaugguuugagccuaguuacaaugaucuaaaauu
cacgguCCAAAACUGCAGUUACUUUUGCaccaac
(((((.(((...((.(((((((.(((..((.(((..(((((...(((..(((....)))..)))....)))))...))).
...)).)))....))))))))).))).)))))..

------CCAAAACUGCAGUUACUUUUGC------ 7 0,0

>hsa-mir-548q 5p:8-29 SRX018957
auauuagGCUGGUGCAAAAGUAAUGGCGGuuuuugccauuacuuuucauuuuuaccauuaaaaguaauggcaaaaagcau
gauuacuuuuucaccaaccu
.....(((.(((((.((((((((.(.(.(((((((((((((((((.((.......)).))))))))))))))))).).)
.))))))))).))))).)))
-----AGGCUGGUGCAAAAGUAAUGa--
-------------------- 2 -2,-4
-----AGGCUGGUGCAAAAGUAAUGGCa--
-------------------- 2 -2,-2
-------GCUGGUGCAAAAGUAAUG---
-------------------- 2 0,-4

>hsa-mir-548s 3p:52-74 SRX018957
uugcugcaaaaauaauugcaguuuuugccauuauuuuuaauaauuauaauaAUGGCCAAAACUGCAGUUAUUUUugcacc
aa
.((.((((((((((((((((((((.(((((((((...........))))))))).))))))))))))))))))))).)
).
---UAAUGGCCAAAACUGCAGUUA----------
-- 2 -2,-4

>hsa-mir-548v 3p:49-70 SRX018957
aauacuagguuugagcaaaaguaauugcgguuuugccaucaugccaaaAGCUACAGUUACUUUUGCACCAgccuaauauu
((((.((((((.(.((((((((((((((..(((((((......))..)))))..)))))))))))))).).)))))).))))
--AGCUACAGUUACUUUUGCACC-----------
 2 0,-1

>hsa-mir-548w 5p:10-32 SRX018957
gguuggugcAAAAGUAACUGCGGUUUUUGCCUuucaacauaauggcaaaacccacaauuacuuuugcaccaauc
((((((((((((((((.((.((.(((((((............))))))).)).)).))))))))))))))))
---------AAAAGUAACUGCGGUUUUUG--- 3
 0,-3
---------AAAAGUAACUGCGGUUUUUGCC--- 3
 0,-1

>hsa-mir-550a-1 MA:22-44;mi:61-82 SRX018957

ugaugcuuugcuggcuggugcAGUGCCUGAGGGAGUAAGAGCCCuguuguuguaagauagUGUCUUACUCCCUCAGGCAC
AUcuccaacaagucucu
.((.((((.(.(((..(((...((((((((((((((((((.(.(((((.......))))).)))))))))))))))))))
))).))).))))).)).
---------------------AGUGCCUGAGGGAGUAAGA---
----------------- 2 0,-4
---------------------AGUGCCUGAGGGAGUAAGAG--
----------------- 2 0,-3

>hsa-mir-550a-2 MA:22-44;mi:61-82 SRX018957
ugaugcuuugcuggcuggugcAGUGCCUGAGGGAGUAAGAGCCCuguuguugucagauagUGUCUUACUCCCUCAGGCAC
AUcuccagcgagucucu
.........(((.(((((.(..((((((((((((((((((.(.(((((.......))))).)))))))))))))))))))
..).))))).)))....
---------------------AGUGCCUGAGGGAGUAAGA---
----------------- 2 0,-4
---------------------AGUGCCUGAGGGAGUAAGAG--
----------------- 2 0,-3

>hsa-mir-552 3p:61-81 SRX018957
aaccauucaaauauaccacaguuuguuuaaccuuuugccuguugguugaagaugccuuucAACAGGUGACUGGUUAGACA
Aacugguauauaca
..........((((((((((((((((((((((..(..((((((((..(.......)..))))))))..)..)))))))))
)))))))))))))...
--AACAGGUGACUGGUUAGACA
---------------- 3 0,-1
--AACAGGUGACUGGUUAGACA
A--------------- 2 0,0
--AACAGGUGACUGGUUAGACA
AAa------------- 2 0,1

>hsa-mir-556 5p:16-37;3p:55-76 SRX018957
gauaguaauaagaaaGAUGAGCUCAUUGUAAUAUGAGcuucauuuauacauuucAUAUUACCAUUAGCUCAUCUUUuuua
uuacuaccuucaaca
..(((((((((((((((((((((.((.((((((((((..............)))))))))).)).)))))))))))))))
)))))).........
--AUAUUACCAUUAGCUCAUCU------
--------------- 2 0,-2

>hsa-mir-558   3p:61-79   SRX018957
gugugugugugugugugugugguuauuuugguauaguagcucuagacucuauuauaguuuccUGAGCUGCUGUACCAAAAUa
ccacaaacgggcug
........((.(((.((((((.((((((((((((((((((..((..(((...)))..))..)))))))))))))))))))
))))).))).))..

>hsa-mir-574   5p:25-47;3p:61-82   SRX018957
gggaccugcgugggugcgggcgugUGAGUGUGUGUGUGAGUGUGUgucgcuccgggucCACGCUCAUGCACACACCCA
CAcgcccacacucagg
........(.((((((.((((((((.(.((((((((((((((((((..((......))..))))))))))))))))))))
))))))).)))))).)
------------------------UGAGUGUGUGUGUGAG-------------------------------------
----------------   40 0,-5
------------------------UGAGUGUGUGUGUGAGU------------------------------------
----------------   72 0,-4
------------------------UGAGUGUGUGUGUGAGUaa----------------------------------
----------------   3   0,-4
------------------------UGAGUGUGUGUGUGAGUG-----------------------------------
----------------   3   0,-3
------------------------UGAGUGUGUGUGUGAGUGU----------------------------------
----------------   20 0,-2
------------------------UGAGUGUGUGUGUGAGUGUG---------------------------------
----------------   2   0,-1
------------------------UGAGUGUGUGUGUGAGUGUGa--------------------------------
----------------   6   0,-1
------------------------UGAGUGUGUGUGUGAGUGUGaa-------------------------------
----------------   3   0,-1
------------------------UGAGUGUGUGUGUGAGUGUGU--------------------------------
----------------   14 0,0
------------------------UGAGUGUGUGUGUGAGUGUGUau------------------------------
----------------   3   0,0
------------------------UGAGUGUGUGUGUGAGUGUGUG-------------------------------
----------------   7   0,1
------------------------UGAGUGUGUGUGUGAGUGUGUGa------------------------------
----------------   6   0,1
------------------------UGAGUGUGUGUGUGAGUGUGUGU------------------------------
----------------   18 0,2

--CACGCUCAUGCACACACC--
----------------   8   0,-4
--CACGCUCAUGCACACACCC-
----------------   15 0,-3
--CACGCUCAUGCACACACCCA
----------------   16 0,-2
--CACGCUCAUGCACACACCCA
a---------------   2   0,-2
--CACGCUCAUGCACACACCCA
u---------------   5   0,-2
--CACGCUCAUGCACACACCCA
C---------------   12 0,-1
--CACGCUCAUGCACACACCCA
Cu--------------   3   0,-1
--CACGCUCAUGCACACACCCA
CA--------------   95 0,0
--CACGCUCAUGCACACACCCA
CAa-------------   7   0,0
--CACGCUCAUGCACACACCCA
CAu-------------   8   0,0
---ACGCUCAUGCACACACCCA
C---------------   2   1,-1
---ACGCUCAUGCACACACCCA
CA--------------   2   1,0

>hsa-mir-576   5p:16-37;3p:55-76   SRX018957
uacaauccaacgaggAUUCUAAUUUCUCCACGUCUUUgguaauaagguuuggcaAAGAUGUGGAAAAAUUGGAAUCcuca
uucgauugguuauaacca
..(((((.((.((((((((((((((.((((((((((((.(((......))).)))))))))))).)))))))))))))).
)).))))).........
--AAGAUGUGGAAAAAUUGGAA------
------------------ 2   0,-2
--AAGAUGUGGAAAAAUUGGAAU-----
------------------ 2   0,-1
---AGAUGUGGAAAAAUUGGAAUC----
------------------ 2   1,0

>hsa-mir-582   5p:16-38;3p:53-74   SRX018957

aucugugcucuuugaUUACAGUUGUUCAACCAGUUACUaaucuaacuaauugUAACUGGUUGAACAACUGAACCcaaagg
gugcaaaguagaaacauu
...((..((((((((....(((((((((((((((((((..............)))))))))))))))))))....))))))
)..)).............
--UAACUGGUUGAACAACUGA---------
------------------ 3   0,-3
--UAACUGGUUGAACAACUGAA--------
------------------ 10 0,-2
--UAACUGGUUGAACAACUGAAC-------
------------------ 3   0,-1
--UAACUGGUUGAACAACUGAACC------
------------------ 3   0,0
--UAACUGGUUGAACAACUGAACCC-----
------------------ 4   0,1

>hsa-mir-584   5p:16-37   SRX018957
uagggugaccagccaUUAUGGUUUGCCUGGGACUGAGgaauuugcugggauaugucaguuccaggccaaccaggcugguu
ggucucccugaagcaac
(((((.(((((((((...(((((.((((((((((((.(.(((......))).).)))))))))))).)))))...)))))
)))).)))))).......
---------------UUAUGGUUUGCCUGGGAC---
-----------------   6   0,-4

>hsa-mir-589   MA:24-45;mi:61-84   SRX018957
uccagccugugcccagcagccccUGAGAACCACGUCUGCUCUGAGcuggguacugccuguUCAGAACAAAUGCCGGUUCC
CAGAcgcugccagcuggcc
....(((...((...(((((..(((.(((((.(((.((.(((((((.(((.....)))))))))).)).))).))))).
)))..)))))..)).))).
-----------------------UGAGAACCACGUCUGCUC---------------------------------------
------------------- 3   0,-4
-----------------------UGAGAACCACGUCUGCUCU--------------------------------------
------------------- 2   0,-3

>hsa-mir-590   5p:16-37;3p:56-76   SRX018957
uagccagucagaaauGAGCUUAUUCAUAAAAGUGCAGuauggugaagucaaucugUAAUUUUAUGUAUAAGCUAGUcucu
gauugaaacaugcagca
....((((((((.((.(((((((.(((((((.(((((..(((.....)))..))))).))))))).))))))).)).)))
))))).............

---------------GAGCUUAUUCAUAAAAGUGCA--
-----------------   3   0,-1
---------------GAGCUUAUUCAUAAAAGUGCAG---
-----------------   7   0,0
--GUAAUUUUAUGUAUAAGCUAGU----
-----------------   6   -1,0

>hsa-mir-598   3p:61-82   SRX018957
gcuugaugaugcugcugaugcuggcggugaucccgauggugugagcuggaaauggggugcUACGUCAUCGUUGUCAUCGU
CAucaucaucauccgag
......................(((((((((..(((((..((.(((............)))))..)))))..))))))))
)................
--UACGUCAUCGUUGUCAUC--
-----------------   13   0,-4
--UACGUCAUCGUUGUCAUCG-
-----------------   9   0,-3
--UACGUCAUCGUUGUCAUCGU
-----------------   13   0,-2
--UACGUCAUCGUUGUCAUCGU
C----------------   8   0,-1
--UACGUCAUCGUUGUCAUCGU
CA---------------   64   0,0
--UACGUCAUCGUUGUCAUCGU
CAa--------------   7   0,0
--UACGUCAUCGUUGUCAUCGU
CAU--------------   24   0,1
--UACGUCAUCGUUGUCAUCGU
CAUu-------------   6   0,1

>hsa-mir-600   3p:61-83   SRX018957
aagucacgugcuguggcuccagcuucauaggaaggcucuugucugucaggcaguggaguuACUUACAGACAAGAGCCUUG
CUCaggccagcccugccc
.........((.(.((((...((((...((.(((((((((((((((.((............)).))))))))))))))).
)).)))).))))).))..
-------------------------CAUAGGAAGGCUCUUGUCU------------------------------------
------------------   5

>hsa-mir-605   5p:16-38   SRX018957

gcccuagcuugguucUAAAUCCCAUGGUGCCUUCUCCUugggaaaaacagagaaggcacuaugagauuuagaaucaaguu
agg
..(((((((((((((((((((.((((((((((((((..((.......)))))))))))))))).))))))))))))))))
)))
--AGAAGGCACUAUGAGAUUUAGA--------
--- 3

>hsa-mir-618   5p:16-38   SRX018957
cucuuguucacagccAAACUCUACUUGUCCUUCUGAGUguaauuacguacaugcaguagcucaggagacaagcagguuua
cccuguggaugagucuga
..((..(((((((..((((.((.((((((.((((((((.....(((((....)).))))))))))))))))).)))))).
..)))))))..)).....
---------------AAACUCUACUUGUCCUUCU--
------------------ 2   0,-4
---------------AAACUCUACUUGUCCUUCUGA--
------------------ 2   0,-2
---------------AAACUCUACUUGUCCUUCUGAG---
------------------ 2   0,-1

>hsa-mir-619   3p:61-84   SRX018957
cgcccaccucagccucccaaaaugcugggauuacaggcaugagccacugcggucgaccauGACCUGGACAUGUUUGUGCC
CAGUacugucaguuugcag
.((..((..(((..........(((((((..((((((((((..(((....((((......))))))).))))))))))))
))))))))...))..))..
-----------------------GCUGGGAUUACAGGCAUGAG-------------------------------------
------------------- 7
-----------------------GCUGGGAUUACAGGCAUGAGCC-----------------------------------
------------------- 4
------------------------CUGGGAUUACAGGCAUGA--------------------------------------
------------------- 2
------------------------CUGGGAUUACAGGCAUGAG-------------------------------------
------------------- 11
------------------------CUGGGAUUACAGGCAUGAGC------------------------------------
------------------- 2
------------------------CUGGGAUUACAGGCAUGAGCCAC---------------------------------
------------------- 4
-------------------------UGGGAUUACAGGCAUGAG-------------------------------------
------------------- 4

-------------------------UGGGAUUACAGGCAUGAGCCA----------------------------------
------------------- 4
-------------------------UGGGAUUACAGGCAUGAGCCAC---------------------------------
------------------- 2
--------------------------GGGAUUACAGGCAUGAGCCAC---------------------------------
------------------- 7

>hsa-mir-624 mi:16-37;MA:54-74 SRX018957
aaugcuguuucaaggUAGUACCAGUACCUUGUGUUCAguggaaccaagguaaaCACAAGGUAUUGGUAUUACCUugagau
agcauuacaccuaagug
((((((((((((((((((((((((((((((((((((..((....))..)..)))))))))))))))))))))))))))))
))))))...........
---------------UAGUACCAGUACCUUGUGUUC--
----------------- 5 0,-1
---------------UAGUACCAGUACCUUGUGUUCA---
----------------- 15 0,0

>hsa-mir-625 MA:15-35;mi:52-73 SRX018957
aggguagagggaugAGGGGGAAAGUUCUAUAGUCCuguaauuagaucucagGACUAUAGAACUUUCCCCCUCAucccucu
gcccu
(((((((((((((((((((((((((((((((((((((...........))))))))))))))))))))))))))))))))
)))))
--------------AGGGGGAAAGUUCUAUAGUC--
----- 18 0,-1
--------------AGGGGGAAAGUUCUAUAGUCC---
----- 3 0,0
--------------AGGGGGAAAGUUCUAUAGUCCU--
----- 5 0,1

>hsa-mir-628 5p:23-44;3p:61-81 SRX018957
auagcuguugugucacuuccucAUGCUGACAUAUUUACUAGAGGguaaaauuaauaaccuUCUAGUAAGAGUGGCAGUCG
Aagggaagggcucau
........((.(((.((((((..(((((.(((.(((((((((((((..........))))))))))))).))).))).))
..))))))))).)).
----------------------AUGCUGACAUAUUUACUA--
--------------- 2 0,-4
----------------------AUGCUGACAUAUUUACUAGA--------------------------------------
--------------- 3 0,-2

----------------------AUGCUGACAUAUUUACUAGAG--------------------------------------
-------------- 13 0,-1
----------------------AUGCUGACAUAUUUACUAGAGa-------------------------------------
-------------- 2   0,-1
----------------------AUGCUGACAUAUUUACUAGAGG-------------------------------------
-------------- 8   0,0
----------------------AUGCUGACAUAUUUACUAGAGGa------------------------------------
-------------- 3   0,0
----------------------AUGCUGACAUAUUUACUAGAGGGa-----------------------------------
-------------- 2   0,1

>hsa-mir-629   MA:22-42;mi:61-82   SRX018957
ucccuuucccaggggaggggcUGGGUUUACGUUGGGAGAACUuuuacggugaaccaggagGUUCUCCCAACGUAAGCCCA
GCcccucccccucugccu
..........((((((((((((((((((((((((((((((((((((..((....)).))))))))))))))))))))))))
))))))))))))......
---------------------UGGGUUUACGUUGGGAGA--
-----------------   76 0,-3
---------------------UGGGUUUACGUUGGGAGAA---
-----------------   51 0,-2

>hsa-mir-641   5p:16-39   SRX018957
ugggugaaaggaaggAAAGACAUAGGAUAGAGUCACCUCuguccucuguccucuaccuauagaggugacuguccuauguc
uuuccuuccucuuaccccu
.(((((.((((((((((((((((((((..((((((((((((((.....(....).....))))))))))))))))))))))
))))))))))).)))))).
---------------AAAGACAUAGGAUAGAGUCA--
------------------- 3   0,-4
---------------AAAGACAUAGGAUAGAGUCAC---
------------------- 10 0,-3
---------------AAAGACAUAGGAUAGAGUCACC--
------------------- 12 0,-2
---------------AAAGACAUAGGAUAGAGUCACCU---
------------------- 22 0,-1
---------------AAAGACAUAGGAUAGAGUCACCUC--
------------------- 4   0,0

>hsa-mir-652   3p:61-81   SRX018957

acgaauggcuaugcacugcacaacccuaggagagggugccauucacauagacuauaauugAAUGGCGCCACUAGGGUUGU
Gcagugcacaaccuacac
......((...((((((((((((((((((.....(((((((((((..((.....))..))))))))))).))))))))))
))))))))...)).....
-------------------ACAACCCUAGGAGAGGGUGCCAU--------------------------------------
------------------ 3
--AAUGGCGCCACUAGGGUU--
------------------ 10 0,-3
--AAUGGCGCCACUAGGGUUGU
Ga---------------- 5   0,0
--AAUGGCGCCACUAGGGUUGU
Gu---------------- 11 0,0
--AAUGGCGCCACUAGGGUUGU
Guu--------------- 2   0,0
--AAUGGCGCCACUAGGGUUGU
GC---------------- 2   0,1

>hsa-mir-654   5p:16-37;3p:51-72   SRX018957
ggguaaguggaaagaUGGUGGGCCGCAGAACAUGUGCugaguucgugccaUAUGUCUGCUGACCAUCACCUUuagaagcc
c
((((......((((.((((((.(.(((((.(((((((((....)).).))))))))))).).)))))).))))....)))
)
--UAUGUCUGCUGACCAUCACCa---------
-   6   0,-2
--UAUGUCUGCUGACCAUCACCU---------
-   3   0,-1
--UAUGUCUGCUGACCAUCACCUU--------
-   2   0,0

>hsa-mir-656   3p:43-63   SRX018957
cugaaauagguugccugugagguguucacuuucuauaugaugAAUAUUAUACAGUCAACCUCUuuccgauaucgaauc
..((((.((((((.(((((..(((((((...((.....)))))))))..))))).)))))).))))............
--AAUAUUAUACAGUCAACCU-----------------
    2   0,-2

>hsa-mir-659   3p:61-82   SRX018957
uaccgacccucgauuugguucaggaccuucccugaaccaaggaagagucacagucucuucCUUGGUUCAGGGAGGGUCCC
CAacaauguccucaugg

...........(((...(((..(((((((((((((((((((((((((.......))))))))))))))))))))))))).
.)))...))).......
---------------------AGGACCUUCCCUGAACCAAGGA-------------------------------------
-----------------   2

>hsa-mir-660   5p:16-37   SRX018957
cugcuccuucucccaUACCCAUUGCAUAUCGGAGUUGugaauucucaaaacaccuccugugugcauggauuacaggaggg
ugagccuugucaucgug
..((((.((((((.....((((.((((((.((((.(((...........))).)))).))))))))))......))))))
.))))............
---------------UACCCAUUGCAUAUCGGAGUU--
-----------------   11   0,-1
---------------UACCCAUUGCAUAUCGGAGUUG---
-----------------   18   0,0
---------------UACCCAUUGCAUAUCGGAGUUGU--
-----------------   6   0,1
---ACCUCCUGUGUGCAUGGAUU---------
-----------------   3
---ACCUCCUGUGUGCAUGGAUUA--------
-----------------   3

>hsa-mir-663b   3p:90-111   SRX018957
ggugccgagggccguccggcauccuaggcgggucgcugcgguaccucccuccugucuguggcggugggaucccguggccg
uguuuuccuGGUGGCCCGGCCGUGCCUGAGGuuuc
...(((..((((((.(((((...(((((..((..((..(((....((((.((.((.....)))).))))..)))..))..
..))..)))))..).)))).)).))))..)))...

---------GGUGGCCCGGCCGUGCCU-------- 16   0,-4

---------GGUGGCCCGGCCGUGCCUG------- 3   0,-3

---------GGUGGCCCGGCCGUGCCUGA------ 4   0,-2

---------GGUGGCCCGGCCGUGCCUGAG----- 8   0,-1

---------GGUGGCCCGGCCGUGCCUGAGG---- 5   0,0

>hsa-mir-664   mi:11-34;MA:49-71   SRX018957

gaacauugaaACUGGCUAGGGAAAAUGAUUGGAUagaaacuauuauucUAUUCAUUUAUCCCCAGCCUACAaaaugaaaa
aa
...((((......((((.((((...(((.(((((((((.......))))))))).))))))).)))).....)))).....
..
----------ACUGGCUAGGGAAAAUGAUUG---
-- 4 0,-3
----------ACUGGCUAGGGAAAAUGAUUGG--
-- 77 0,-2
----------ACUGGCUAGGGAAAAUGAUUGGu---
-- 36 0,-2
----------ACUGGCUAGGGAAAAUGAUUGGA---
-- 119 0,-1
----------ACUGGCUAGGGAAAAUGAUUGGAa--
-- 7 0,-1
----------ACUGGCUAGGGAAAAUGAUUGGAU--
-- 2 0,0
-----------CUGGCUAGGGAAAAUGAUU--
-- 2 1,-4
-----------CUGGCUAGGGAAAAUGAUUG---
-- 4 1,-3
-----------CUGGCUAGGGAAAAUGAUUGG--
-- 6 1,-2
-----------CUGGCUAGGGAAAAUGAUUGGA---
-- 39 1,-1
-----------CUGGCUAGGGAAAAUGAUUGGAa--
-- 4 1,-1
------------UGGCUAGGGAAAAUGAUUGGA---
-- 2 2,-1
------------UGGCUAGGGAAAAUGAUUGGAa--
-- 2 2,-1
------------UGGCUAGGGAAAAUGAUUGGAaa---
-- 2 2,-1
--UAUUCAUUUAUCCCCAGCCUA-----------
-- 2 0,-2
--UAUUCAUUUAUCCCCAGCCUAC----------
-- 4 0,-1
--UAUUCAUUUAUCCCCAGCCUACA---------
-- 8 0,0

>hsa-mir-665 3p:43-62 SRX018957
ucuccucgaggggucucugccucuacccaggacucuuucaugACCAGGAGGCUGAGGCCCCUcacaggcggc
((.(((.((((((((((.((((((...((.((.....)).))....)))))).))))))))))..))).)).
--ACCAGGAGGCUGAGGCCCCUCA-------- 3
0,2
--ACCAGGAGGCUGAGGCCCCUCAu------- 2
0,2

>hsa-mir-671 5p:29-51;3p:68-88 SRX018957
gcaggugaacuggcaggccaggaagaggAGGAAGCCCUGGAGGGGCUGGAGgugauggauguuuuccUCCGGUUCUCAGG
GCUCCACCucuuucgggccguagagccagggcuggugc
(((...(..(((((.((((.((((((((.(((.((((((..((((((((((.(((....)))..))))))))))))))
))))).)))))))).)))).....)))))..)...)))
---UCCGGUUCUCAGG
GCUCCACC------------------------------ 2 0,0

>hsa-mir-675 MA:10-32;mi:45-64 SRX018957
cccagggucUGGUGCGGAGAGGGCCCACAGUGgacuuggugacgCUGUAUGCCCUCACCGCUCAgccccuggg
((((((.((((.((((.((((((..((((((.........))))))..)))))).)))))))).)))))))
---------UGGUGCGGAGAGGGCCCACAG--- 5
0,-2
---------UGGUGCGGAGAGGGCCCACAGU-- 14
0,-1
---------UGGUGCGGAGAGGGCCCACAGUa--------------------------------------- 2
0,-1
---------UGGUGCGGAGAGGGCCCACAGUG--------------------------------------- 14
0,0
---------UGGUGCGGAGAGGGCCCACAGUGa-------------------------------------- 2
0,0
---------UGGUGCGGAGAGGGCCCACAGUGu-------------------------------------- 16
0,0
--CUGUAUGCCCUCACCGCUCAGu------- 2
0,1
--CUGUAUGCCCUCACCGCUCAGC------- 7
0,2

>hsa-mir-676 mi:8-28;MA:43-63 SRX018957

gcaugacUCUUCAACCUCAGGACUUGCAgaauuaauggaaugCUGUCCUAAGGUUGUUGAGUUgugc
(((..((((..((((((.(((((..(((............))).))))).))))))..))))..)))
--CUGUCCUAAGGUUGUUGAGUU---- 2   0,0
--CUGUCCUAAGGUUGUUGAGUUG--- 2   0,1

>hsa-mir-7-1   MA:24-46;mi:66-87   SRX018957
uuggauguuggccuaguucugugUGGAAGACUAGUGAUUUUGUUGUuuuuagauaacuaaaucgaCAACAAAUCACAGUC
UGCCAUAuggcacaggccaugccucuacag
.((((.((((((((.((.((((((((.(((((.(((((((.(((((((((((....)))))..)))))))))))))))))
).)))))))).)))))))).)).))))...
-----------------------UGGAAGACUAGUGAUUUUGUU------------------------------------
------------------------------ 2   0,-2
-----------------------UGGAAGACUAGUGAUUUUGUUG-----------------------------------
------------------------------ 7   0,-1
-----------------------UGGAAGACUAGUGAUUUUGUUGU----------------------------------
------------------------------ 48   0,0
-----------------------UGGAAGACUAGUGAUUUUGUUGUU---------------------------------
------------------------------ 117 0,1
-----------------------UGGAAGACUAGUGAUUUUGUUGUUa--------------------------------
------------------------------ 6   0,1
-----------------------UGGAAGACUAGUGAUUUUGUUGUUU--------------------------------
------------------------------ 3   0,2
---CAACAAAUCACAGUC
UGCCAU------------------------ 4   0,-1
---CAACAAAUCACAGUC
UGCCAUu----------------------- 2   0,-1
---CAACAAAUCACAGUC
UGCCAUA----------------------- 8   0,0
---CAACAAAUCACAGUC
UGCCAUAa---------------------- 3   0,0
---CAACAAAUCACAGUC
UGCCAUAU---------------------- 2   0,1
--AACAAAUCACAGUC
UGCCAUA----------------------- 3   1,0
--AACAAAUCACAGUC
UGCCAUAU---------------------- 2   1,1

>hsa-mir-7-2   MA:32-54;mi:72-93   SRX018957

cuggauacagaguggaccggcuggccccaucUGGAAGACUAGUGAUUUUGUUGUugucuuacugcgcucaaCAACAAAUC
CCAGUCUACCUAAuggugccagccaucgca
...........((((...(((((((.((((..((.(((((.(.(((((.(((((((............))))))))))))
).))))).))..)))).))))))).)))).
-------------------------------UGGAAGACUAGUGAUUUUGUU----------------------------
------------------------------ 2 0,-2
-------------------------------UGGAAGACUAGUGAUUUUGUUG---------------------------
------------------------------ 7 0,-1
-------------------------------UGGAAGACUAGUGAUUUUGUUGU--------------------------
------------------------------ 48 0,0
-------------------------------UGGAAGACUAGUGAUUUUGUUGUU-------------------------
------------------------------ 117 0,1
-------------------------------UGGAAGACUAGUGAUUUUGUUGUUa------------------------
------------------------------ 6 0,1
-------------------------------UGGAAGACUAGUGAUUUUGUUGUUG------------------------
------------------------------ 3 0,2

>hsa-mir-7-3 5p:31-53 SRX018957
agauuagaguggcuguggucuagugcugugUGGAAGACUAGUGAUUUUGUUGUucugauguacuacgacaacaagucaca
gccggccucauagcgcagacucccuucgac
......(((.((....(((((.((((((((.((..(.((.(((((((.((((((.((......)).))))))))))))))
).)..)).))))))))))))))).)))...
------------------------------UGGAAGACUAGUGAUUUUGUU-----------------------------
------------------------------ 2 0,-2
------------------------------UGGAAGACUAGUGAUUUUGUUG----------------------------
------------------------------ 7 0,-1
------------------------------UGGAAGACUAGUGAUUUUGUUGU---------------------------
------------------------------ 48 0,0
------------------------------UGGAAGACUAGUGAUUUUGUUGUU--------------------------
------------------------------ 117 0,1
------------------------------UGGAAGACUAGUGAUUUUGUUGUUa-------------------------
------------------------------ 6 0,1

>hsa-mir-708 MA:11-33;mi:57-78 SRX018957
aacugcccucAAGGAGCUUACAAUCUAGCUGGGgguaaaugacuugcacaugaacaCAACUAGACUGUGAGCUUCUAGag
ggcaggga
..((((((((.(((((((((((.(((((.((...((..(((.......)))..)).)).))))).))))))))))).)))
)))))...

----------AAGGAGCUUACAAUCUAGCU---
--------   7   0,-3
----------AAGGAGCUUACAAUCUAGCUGa--
--------   3   0,-2
----------AAGGAGCUUACAAUCUAGCUGG--
--------   13 0,-1
----------AAGGAGCUUACAAUCUAGCUGGa---
--------   6   0,-1
----------AAGGAGCUUACAAUCUAGCUGGu---
--------   2   0,-1
----------AAGGAGCUUACAAUCUAGCUGGG---
--------   6   0,0
--AACUAGACUGUGAGCUUCUAGA-
--------   3   1,1

>hsa-mir-720   5p:27-43   SRX018957
ccggaucucacacggugguguuaaauaUCUCGCUGGGGCCUCCAaaauguugugcccagggguguuagagaaaacaccaca
cuuugagaugaauuaagaguccuuuauuag
....((((((....(((((((((((((((((....(((((..(.....)..).)))).))))))))))......))))))).
...)))))).....................
-------------------------AUCUCGCUGGGGCCUCCA--------------------------------------
------------------------------ 112 -1,0

>hsa-mir-744   MA:11-32;mi:68-89   SRX018957
uugggcaaggUGCGGGGCUAGGGCUAACAGCAgucuuacugaagguuuccuggaaaccacgcacaugCUGUUGCCACUAA
CCUCAACCUuacucgguc
(((((.(((((..((((.((((((.(((((((.................................)))))))))).))).
)))).))))).)))))..
----------UGCGGGGCUAGGGCUAACA---
------------------ 5   0,-3
----------UGCGGGGCUAGGGCUAACAa--
------------------ 2   0,-3
----------UGCGGGGCUAGGGCUAACAG--
------------------ 63 0,-2
----------UGCGGGGCUAGGGCUAACAGa---
------------------ 24 0,-2
----------UGCGGGGCUAGGGCUAACAGaa--
------------------ 7   0,-2

----------UGCGGGGCUAGGGCUAACAGu--
------------------ 6 0,-2
----------UGCGGGGCUAGGGCUAACAGC--
------------------ 183 0,-1
----------UGCGGGGCUAGGGCUAACAGCu---
------------------ 3 0,-1
----------UGCGGGGCUAGGGCUAACAGCA--
------------------ 360 0,0
----------UGCGGGGCUAGGGCUAACAGCAa---
------------------ 128 0,0
----------UGCGGGGCUAGGGCUAACAGCAaa--
------------------ 12 0,0
----------UGCGGGGCUAGGGCUAACAGCAau--
------------------ 21 0,0
----------UGCGGGGCUAGGGCUAACAGCAu---
------------------ 428 0,0
----------UGCGGGGCUAGGGCUAACAGCAuu--
------------------ 43 0,0
----------UGCGGGGCUAGGGCUAACAGCAG---
------------------ 11 0,1
-----------GCGGGGCUAGGGCUAACAG---
------------------ 3 1,-2
-----------GCGGGGCUAGGGCUAACAGC--
------------------ 9 1,-1
-----------GCGGGGCUAGGGCUAACAGCA---
------------------ 22 1,0
-----------GCGGGGCUAGGGCUAACAGCAa--
------------------ 7 1,0
-----------GCGGGGCUAGGGCUAACAGCAau---
------------------ 2 1,0
-----------GCGGGGCUAGGGCUAACAGCAu--
------------------ 13 1,0

>hsa-mir-758 3p:52-73 SRX018957
gccuggauacaugagaugguugaccagagagcacacgcuuuauuugugccgUUUGUGACCUGGUCCACUAACCcucagua
ucuaaugc
...(((((((.((((.((((.((((((...(((.((((.........).))).))).))...)))))).)))).)))...)))))))
)))).....

---------------AUGGUUGACCAGAGAGCACA---
-------- 11
---------------AUGGUUGACCAGAGAGCACAC--
-------- 18
---------------AUGGUUGACCAGAGAGCACACG---
-------- 2

>hsa-mir-765 3p:69-89 SRX018957
uuuaggcgcugaugaaaguggaguucaguagacagcccuuuucaagcccuacgagaaacugggguuucUGGAGGAGAAGG
AAGGUGAUGaaggaucuguucucgugagccugaa
.((((((....((((.((..((.((((....((...(((((((((((((((........))))))))......)))))))
...))..))))...))..)).))))..)))))).
--UCUGGAGGAGAAGG
AAGGUGAUG------------------------- 2 -2,0
--UGGAGGAGAAGG
AAGGUGAUGA------------------------ 2 0,1

>hsa-mir-766 3p:65-86 SRX018957
gcauccucaggaccugggcuuggguggguaggaggaauuggugcuggucuuucauuuuggauuugACUCCAGCCCCACAGC
CUCAGCcaccccagccaauugucauaggagc
((.((((...(((...((((.(((((((..((((...(((.(((((....(((.........)))..))))).)))...)
))).))))))).))))....)))..))))))
----------------------------AGGAGGAAUUGGUGCUGGUCU-------------------------------
------------------------------- 5
----------------------------AGGAGGAAUUGGUGCUGGUCUU------------------------------
------------------------------- 10
----------------------------AGGAGGAAUUGGUGCUGGUCUUa-----------------------------
------------------------------- 7
----------------------------AGGAGGAAUUGGUGCUGGUCUUU----------------------------
------------------------------- 11

>hsa-mir-769 5p:30-51;3p:69-91 SRX018957
gccuuggugcugauuccugggcucugaccUGAGACCUCUGGGUUCUGAGCUgugauguugcucucgagCUGGGAUCUCCG
GGGUCUUGGUUcagggccggggccucuggguuccaagc
..(((((.(((((..(((.((((((((.(..(((((((.(((.(((.((((.(((........))))))).))).))).)
))))))..).)))))))).)))..))..))).))))).
-----------------------------UGAGACCUCUGGGUUCUGAG-------------------------------
-------------------------------------- 7 0,-2

-----------------------------UGAGACCUCUGGGUUCUGAGC--- 3   0,-1
-----------------------------UGAGACCUCUGGGUUCUGAGCU-- 3   0,0
-----------------------------UGAGACCUCUGGGUUCUGAGCUa--- 2   0,0
-----------------------------UGAGACCUCUGGGUUCUGAGCUau-- 4   0,0
-----------------------------UGAGACCUCUGGGUUCUGAGCUu--- 2   0,0
---CUGGGAUCUCCGGGGUCUUGGUU--------------------------- 2   0,0

>hsa-mir-874   3p:47-68   SRX018957
uuagcccugcggccccacgcaccaggguaagagagacucucgcuucCUGCCCUGGCCCGAGGGACCGAcuggcugggc
((((((...(((.(((.((..((((((((.(((.(.....)..))).))))))))..)).))).)))...))))))..
--CUGCCCUGGCCCGAGGGACCGA---------- 3   0,0
--CUGCCCUGGCCCGAGGGACCGAC--------- 2   0,1

>hsa-mir-877   MA:1-20;mi:66-86   SRX018957
GUAGAGGAGAUGGCGCAGGGgacacgggcaaagacuuggggguuccugggacccucagacgugugUCCUCUUCUCCCUCCUCCCAG
...((((((..((.(.((((((((((.(.......(((((((((.....))))))))).).))))))))))).).))))))))....
GUAGAGGAGAUGGCGCAGGGGACA-- 13 0,4
GUAGAGGAGAUGGCGCAGGGGACAa--- 4   0,4

>hsa-mir-885   5p:11-32;3p:43-64   SRX018957
ccgcacucucUCCAUUACACUACCCUGCCUCUucuccaugagAGGCAGCGGGGUGUAGUGGAUAgagcacgggu
(((..((((.(((((((((((.((((((((((......))))))))).)))))))))))).))))..)))..
----------UCCAUUACACUACCCUGCCU-- 5   0,-2
----------UCCAUUACACUACCCUGCCUC--- 12 0,-1

----------UCCAUUACACUACCCUGCCUCa--- 2

0,-1

----------UCCAUUACACUACCCUGCCUCU--- 31

0,0

----------UCCAUUACACUACCCUGCCUCUa-- 2

0,0

----------UCCAUUACACUACCCUGCCUCUau--- 3

0,0

------------CAUUACACUACCCUGCCUCU--- 2

2,0

--GAGGCAGCGGGGUGUAGUGGAUA---------- 2

-1,0

---AGGCAGCGGGGUGUAGUGa------------- 2

0,-4

---AGGCAGCGGGGUGUAGUGG------------- 61

0,-3

---AGGCAGCGGGGUGUAGUGGA------------ 25

0,-2

---AGGCAGCGGGGUGUAGUGGAa----------- 4

0,-2

---AGGCAGCGGGGUGUAGUGGAaa---------- 4

0,-2

---AGGCAGCGGGGUGUAGUGGAU----------- 33

0,-1

---AGGCAGCGGGGUGUAGUGGAUu---------- 15

0,-1

---AGGCAGCGGGGUGUAGUGGAUA---------- 72

0,0

---AGGCAGCGGGGUGUAGUGGAUAa--------- 33

0,0

---AGGCAGCGGGGUGUAGUGGAUAaa-------- 15

0,0

---AGGCAGCGGGGUGUAGUGGAUAau-------- 15

0,0

---AGGCAGCGGGGUGUAGUGGAUAu--------- 40

0,0

---AGGCAGCGGGGUGUAGUGGAUAuu-------- 5

0,0

---GGCAGCGGGGUGUAGUGGAUAa--------- 4
1,0
---GGCAGCGGGGUGUAGUGGAUAaa-------- 2
1,0

>hsa-mir-889 3p:49-69 SRX018957
gugcuuaaagaauggcuguccguaguauggucucuauauuuaugaugaUUAAUAUCGGACAACCAUUGUuuuaguaucc
(((((.(((.(((((.((((((.....(((((((.........)).)))))....)))))).))))).))).)))))..
--UUAAUAUCGGACAACCAU-------------
8 0,-3
--UUAAUAUCGGACAACCAUU------------
3 0,-2
--UUAAUAUCGGACAACCAUUG-----------
4 0,-1

>hsa-mir-9-1 MA:16-38;mi:55-76 SRX018957
cgggguugguuguuaUCUUUGGUUAUCUAGCUGUAUGAguggugugguggagucuucAUAAAGCUAGAUAACCGAAAGUaaaa
auaacccca
.(((((((....(((..(((((((((((((((.((((((.((.......)))))))).)))))))))))))))..)))..
.))))))).
---------------UCUUUGGUUAUCUAGCUGUAU--
--------- 3 0,-2
--AUAAAGCUAGAUAACCGAAAGU----
--------- 4 0,0

>hsa-mir-9-2 MA:16-38;mi:53-74 SRX018957
ggaagcgaguuguuaUCUUUGGUUAUCUAGCUGUAUGAguguauuggucuucAUAAAGCUAGAUAACCGAAAGUaaaaac
uccuuca
.((((.(((((.(((..(((((((((((((((.((((((..(....)..)))))).)))))))))))))))..))).)))
)))))).
---------------UCUUUGGUUAUCUAGCUGUAU--
------- 3 0,-2
--AUAAAGCUAGAUAACCGAAAGU------
------- 4 0,0

>hsa-mir-9-3 MA:16-38;mi:55-76 SRX018957
ggaggcccguuucucUCUUUGGUUAUCUAGCUGUAUGAgugccacagagccgucAUAAAGCUAGAUAACCGAAAGUagaa
augauucuca

.((((..(((((((((.(((((((((((((((.(((((..((......))..))))).))))))))))))))))).))))
))))..)))).
---------------UCUUUGGUUAUCUAGCUGUAU--
---------- 3 0,-2
--AUAAAGCUAGAUAACCGAAAGU----
---------- 4 0,0

>hsa-mir-92a-1 mi:11-33;MA:48-69 SRX018957
cuuucuacacAGGUUGGGAUCGGUUGCAAUGCUguguuucuguauggUAUUGCACUUGUCCCGGCCUGUugaguuugg
..(((...((((((((((((.(((.(((((((((((......)))))))))))))).)))))))))))).))).....
----------AGGUUGGGAUCGGUUGCAAU--
2 0,-3
----------AGGUUGGGAUCGGUUGCAAUG---
9 0,-2
----------AGGUUGGGAUCGGUUGCAAUGC--
14 0,-1
----------AGGUUGGGAUCGGUUGCAAUGCa---
4 0,-1
----------AGGUUGGGAUCGGUUGCAAUGCU---
196 0,0
----------AGGUUGGGAUCGGUUGCAAUGCUu--
8 0,0
----------AGGUUGGGAUCGGUUGCAAUGCUuu---
2 0,0
---UAUUGCACUUGUCCCGGC-------------
91 0,-4
---UAUUGCACUUGUCCCGGCuu-----------
2 0,-4
---UAUUGCACUUGUCCCGGCC------------
195 0,-3
---UAUUGCACUUGUCCCGGCCU-----------
2651 0,-2
---UAUUGCACUUGUCCCGGCCUau---------
2 0,-2
---UAUUGCACUUGUCCCGGCCUu----------
3 0,-2
---UAUUGCACUUGUCCCGGCCUG----------
633 0,-1

---UAUUGCACUUGUCCCGGCCUGa---------

8 0,-1

---UAUUGCACUUGUCCCGGCCUGaa--------

16 0,-1

---UAUUGCACUUGUCCCGGCCUGau--------

4 0,-1

---UAUUGCACUUGUCCCGGCCUGU---------

889 0,0

---UAUUGCACUUGUCCCGGCCUGUa--------

188 0,0

---UAUUGCACUUGUCCCGGCCUGUaa-------

87 0,0

---UAUUGCACUUGUCCCGGCCUGUau-------

131 0,0

---UAUUGCACUUGUCCCGGCCUGUU--------

74 0,1

---UAUUGCACUUGUCCCGGCCUGUUa-------

9 0,1

---UAUUGCACUUGUCCCGGCCUGUUau------

6 0,1

---UAUUGCACUUGUCCCGGCCUGUUu-------

24 0,1

---UAUUGCACUUGUCCCGGCCUGUUuu------

5 0,1

--AUUGCACUUGUCCCGGCC------------

4 1,-3

--AUUGCACUUGUCCCGGCCU-----------

54 1,-2

--AUUGCACUUGUCCCGGCCUG----------

5 1,-1

--AUUGCACUUGUCCCGGCCUGU---------

9 1,0

--AUUGCACUUGUCCCGGCCUGUa--------

2 1,0

--AUUGCACUUGUCCCGGCCUGUau-------

3 1,0

--AUUGCACUUGUCCCGGCCUGUUu-------

2 1,1

---UUGCACUUGUCCCGGCCU-----------
2 2,-2

>hsa-mir-92a-2 mi:9-30;MA:48-69 SRX018957
ucaucccuGGGUGGGGAUUUGUUGCAUUACuuguguucuauauaaagUAUUGCACUUGUCCCGGCCUGUggaaga
((.(((.(((((.(((((..((.(((.(((((((((...)))).))))).)))))..))))).))))).))).))
---UAUUGCACUUGUCCCGGC---------- 91
0,-4
---UAUUGCACUUGUCCCGGCuu-------- 2
0,-4
---UAUUGCACUUGUCCCGGCC--------- 195
0,-3
---UAUUGCACUUGUCCCGGCCU-------- 2651
0,-2
---UAUUGCACUUGUCCCGGCCUau------ 2
0,-2
---UAUUGCACUUGUCCCGGCCUu------- 3
0,-2
---UAUUGCACUUGUCCCGGCCUG------- 633
0,-1
---UAUUGCACUUGUCCCGGCCUGa------ 8
0,-1
---UAUUGCACUUGUCCCGGCCUGaa----- 16
0,-1
---UAUUGCACUUGUCCCGGCCUGau----- 4
0,-1
---UAUUGCACUUGUCCCGGCCUGU------ 889
0,0
---UAUUGCACUUGUCCCGGCCUGUa----- 188
0,0
---UAUUGCACUUGUCCCGGCCUGUaa---- 87
0,0
---UAUUGCACUUGUCCCGGCCUGUau---- 131
0,0
---UAUUGCACUUGUCCCGGCCUGUG----- 7
0,1
--AUUGCACUUGUCCCGGCC--------- 4
1,-3

---AUUGCACUUGUCCCGGCCU-------- 54
1,-2
---AUUGCACUUGUCCCGGCCUG------- 5
1,-1
---AUUGCACUUGUCCCGGCCUGU------ 9
1,0
---AUUGCACUUGUCCCGGCCUGUa----- 2
1,0
---AUUGCACUUGUCCCGGCCUGUau---- 3
1,0
---AUUGCACUUGUCCCGGCCUGUG----- 2
1,1
--UUGCACUUGUCCCGGCCU-------- 2
2,-2

>hsa-mir-92b   mi:20-41;MA:61-82   SRX018957
cgggccccgggcgggcgggAGGGACGGGACGCGGUGCAGUGuuguuuuuucccccgccaaUAUUGCACUCGUCCCGGCCU
CCggcccccccggccc
.(((((..(((.((((.(((((..(((((((.((((((((((((.............))))))))))))))))))).)))
)).))))))).)))))
-------------------AGGGACGGGACGCGGUGCAGUGU--------------------------------------
----------------   3   0,1
-------------------AGGGACGGGACGCGGUGCAGUGUU-------------------------------------
----------------   2   0,2
--UAUUGCACUCGUCCCGGCC-
----------------   20   0,-3
--UAUUGCACUCGUCCCGGCCU
----------------   17   0,-2
--UAUUGCACUCGUCCCGGCCU
aa--------------   2   0,-2
--UAUUGCACUCGUCCCGGCCU
C---------------   12   0,-1
--UAUUGCACUCGUCCCGGCCU
Ca--------------   4   0,-1
--UAUUGCACUCGUCCCGGCCU
Caa-------------   4   0,-1
--UAUUGCACUCGUCCCGGCCU
CC--------------   8   0,0

--UAUUGCACUCGUCCCGGCCU
CCa-------------   5   0,0
--UAUUGCACUCGUCCCGGCCU
CCaa------------   6   0,0
--UAUUGCACUCGUCCCGGCCU
CCau------------   2   0,0
--UAUUGCACUCGUCCCGGCCU
CCu-------------   6   0,0
--UAUUGCACUCGUCCCGGCCU
CCuu------------   2   0,0

>hsa-mir-93 MA:11-33;mi:50-71   SRX018957
cugggggcucCAAAGUGCUGUUCGUGCAGGUAGugugauuacccaaccuACUGCUGAGCUAGCACUUCCCGagcccccgg
((((((((((..((((((((((((.((((.(((..((......))..)))))))))))).)))))))...))))))))))
----------CAAAGUGCUGUUCGUGCA--
    20 0,-5
----------CAAAGUGCUGUUCGUGCAG---
    33 0,-4
----------CAAAGUGCUGUUCGUGCAGG--
    42 0,-3
----------CAAAGUGCUGUUCGUGCAGGU---
    86 0,-2
----------CAAAGUGCUGUUCGUGCAGGUA--
    215 0,-1
----------CAAAGUGCUGUUCGUGCAGGUAa---
    26 0,-1
----------CAAAGUGCUGUUCGUGCAGGUAu---
    15 0,-1
----------CAAAGUGCUGUUCGUGCAGGUAG---
    54 0,0
----------CAAAGUGCUGUUCGUGCAGGUAGa--
    2   0,0
-----------AAAGUGCUGUUCGUGCAG---
    3   1,-4
-----------AAAGUGCUGUUCGUGCAGG--
    2   1,-3
-----------AAAGUGCUGUUCGUGCAGGU---
    7   1,-2

-----------AAAGUGCUGUUCGUGCAGGUA--
     7   1,-1

>hsa-mir-940   3p:60-80   SRX018957
gugaggugugggcccggccccaggagcggggccugggcagccccguguguugaggaaggAAGGCAGGGCCCCCGCUCCCC
gggccugaccccac
(((.(((.(((((((((.....((((((((((((..((..((((.........))..))...)).))).)))))))))))
)))))))))).)))
---AAGGCAGGGCCCCCGCUCCCC
-------------- 2   0,0

>hsa-mir-941-1 3p:71-93   SRX018957
cacggaagaggacacacccggcuguguggacaugugcccagggcccgggacagcgccacggaagaggacgCACCCGGCUG
UGUGCACAUGUGCccagggcccgggacagcgccacgg
.........((.....(((((((.((.((((((((((.((.((((.(((...(((((.(....).)).))).))))))).
)).)))))))).)))).)).)))))......))....
--CACCCGGCUG
UGUGCACAUGUG-------------------------   3   0,-1
--CACCCGGCUG
UGUGCACAUGUGa------------------------   6   0,-1
--CACCCGGCUG
UGUGCACAUGUGu------------------------   3   0,-1
--CACCCGGCUG
UGUGCACAUGUGC------------------------   35 0,0
--CACCCGGCUG
UGUGCACAUGUGCa-----------------------   3   0,0

>hsa-mir-941-2 3p:71-93   SRX018957
cacggaagaggacgcacccggcugugugcacaugugcccagggcccgggacagcgccacggaagaggacgCACCCGGCUG
UGUGCACAUGUGCccagggcccgggacagcgccacgg
.........((.(((.(((((((.((.((((((((((.((.((((.(((...(((((.(....).)).))).))))))).
)).)))))))))).)).)).)))))...))))).....
--------------CACCCGGCUGUGUGCACAUGUG--
-------------------------------------   3
--------------CACCCGGCUGUGUGCACAUGUGa---
-------------------------------------   6
--------------CACCCGGCUGUGUGCACAUGUGu---
-------------------------------------   3

--------------CACCCGGCUGUGUGCACAUGUGC---
-------------------------------------   35
--------------CACCCGGCUGUGUGCACAUGUGCa--
-------------------------------------   3
--CACCCGGCUG
UGUGCACAUGUG-------------------------   3   0,-1
--CACCCGGCUG
UGUGCACAUGUGa------------------------   6   0,-1
--CACCCGGCUG
UGUGCACAUGUGu------------------------   3   0,-1
--CACCCGGCUG
UGUGCACAUGUGC------------------------   35   0,0
--CACCCGGCUG
UGUGCACAUGUGCa-----------------------   3   0,0

>hsa-mir-941-3 3p:71-93   SRX018957
cacggaagaggacgcacccggcugugugcacaugugcccagggcccgggacagcgccacggaagaggacgCACCCGGCUG
UGUGCACAUGUGCccagggcccgggacagcgccaugg
.........((.(((.(((((((.((.((((((((((.((.((((.(((...(((((.(....).)).))).))))))).
)).)))))))))).)).)).)))))...)))))....
--------------CACCCGGCUGUGUGCACAUGUG--
-------------------------------------   3
--------------CACCCGGCUGUGUGCACAUGUGa---
-------------------------------------   6
--------------CACCCGGCUGUGUGCACAUGUGu---
-------------------------------------   3
--------------CACCCGGCUGUGUGCACAUGUGC---
-------------------------------------   35
--------------CACCCGGCUGUGUGCACAUGUGCa--
-------------------------------------   3
--CACCCGGCUG
UGUGCACAUGUG-------------------------   3   0,-1
--CACCCGGCUG
UGUGCACAUGUGa------------------------   6   0,-1
--CACCCGGCUG
UGUGCACAUGUGu------------------------   3   0,-1
--CACCCGGCUG
UGUGCACAUGUGC------------------------   35   0,0

--CACCCGGCUG
UGUGCACAUGUGCa-----------------------   3   0,0

>hsa-mir-941-4 3p:71-93   SRX018957
cauggaagaggacgcacccggcugugugcacaugugcccagggcccgggacagcgccacggaagaggacgCACCCGGCUG
UGUGCACAUGUGCccagggcccgggacagcgccacgg
.........((.(((.(((((((.((.((((((((((.((.((((.(((...(((((.(....).)).))).))))))).
)).)))))))))).)).)).)))))...)))))....
--------------CACCCGGCUGUGUGCACAUGUG--
-------------------------------------   3
--------------CACCCGGCUGUGUGCACAUGUGa---
-------------------------------------   6
--------------CACCCGGCUGUGUGCACAUGUGu---
-------------------------------------   3
--------------CACCCGGCUGUGUGCACAUGUGC---
-------------------------------------   35
--------------CACCCGGCUGUGUGCACAUGUGCa--
-------------------------------------   3
--CACCCGGCUG
UGUGCACAUGUG-------------------------   3   0,-1
--CACCCGGCUG
UGUGCACAUGUGa------------------------   6   0,-1
--CACCCGGCUG
UGUGCACAUGUGu------------------------   3   0,-1
--CACCCGGCUG
UGUGCACAUGUGC------------------------   35 0,0
--CACCCGGCUG
UGUGCACAUGUGCa-----------------------   3   0,0

>hsa-mir-944   3p:54-75   SRX018957
guuccagacacaucucaucugauauacaauauuuucuuaaauuguauaaagagAAAUUAUUGUACAUCGGAUGAGcugug
ucugggau
((((((((((((.((((((((((.(((((((.((((((............)))))).))))))).)))))))))).))))
))))))))
---AAAUUAUUGUACAUCGGAUGAG-----
--------   4   0,0

>hsa-mir-95 3p:49-70   SRX018957

aacacagugggcacucaauaaaugucuguugaauugaaaugcguuacaUUCAACGGGUAUUUAUUGAGCAcccacucugu
g
.(((.((((((..((((((((((..((((((((((((......))).)))))))))..))))))))))..)))))).)))
.
-------------CUCAAUAAAUGUCUGUUGAAU--
-    2
--UUCAACGGGUAUUUAUUG--------------
-    112 0,-4
--UUCAACGGGUAUUUAUUGA-------------
-    67 0,-3
--UUCAACGGGUAUUUAUUGAG------------
-    268 0,-2
--UUCAACGGGUAUUUAUUGAGC-----------
-    18 0,-1
--UUCAACGGGUAUUUAUUGAGCA----------
-    20 0,0
--UUCAACGGGUAUUUAUUGAGCAa---------
-    32 0,0
--UUCAACGGGUAUUUAUUGAGCAu---------
-    4   0,0
---UCAACGGGUAUUUAUUGA-------------
-    2   1,-3
---UCAACGGGUAUUUAUUGAG------------
-    3   1,-2
---UCAACGGGUAUUUAUUGAGC-----------
-    2   1,-1
---UCAACGGGUAUUUAUUGAGCA----------
-    4   1,0
---UCAACGGGUAUUUAUUGAGCAa---------
-    9   1,0
---UCAACGGGUAUUUAUUGAGCAu---------
-    4   1,0
--CAACGGGUAUUUAUUGAGCAa---------
-    2   2,0
--CAACGGGUAUUUAUUGAGCAu---------
-    5   2,0

>hsa-mir-98 5p:22-43   SRX018957

aggauucugcucaugccagggUGAGGUAGUAAGUUGUAUUGUUguggguagggauauuaggccccaauuagaagauaac
uauacaacuuacuacuuucccugguguguggcauauuca
.((((..(((((((((((((.((((((((((((((((((.((((((((((..........))))))).........))))
.))))))))))))))))))))))))))).)))).)))).
---------------------UGAGGUAGUAAGUUGUAU--
--------------------------------------- 89 0,-4
---------------------UGAGGUAGUAAGUUGUAUa---------------------------------------
--------------------------------------- 2 0,-4
---------------------UGAGGUAGUAAGUUGUAUU---------------------------------------
--------------------------------------- 2 0,-3
---------------------UGAGGUAGUAAGUUGUAUUG--------------------------------------
--------------------------------------- 8 0,-2
---------------------UGAGGUAGUAAGUUGUAUUGU-------------------------------------
--------------------------------------- 21 0,-1
---------------------UGAGGUAGUAAGUUGUAUUGUU------------------------------------
--------------------------------------- 17 0,0
---------------------UGAGGUAGUAAGUUGUAUUGUUu-----------------------------------
--------------------------------------- 5 0,0

>hsa-mir-99a MA:13-34;mi:50-71 SRX018957
cccauuggcauaAACCCGUAGAUCCGAUCUUGUGgugaaguggaccgcaCAAGCUCGCUUCUAUGGGUCUGugucagugu
g
..((((((((((.(((((((((..(((.((((((.((.........)))))))).)))..))))))))).)))))))))).
.
-----------AAACCCGUAGAUCCGAUCUU--
- 5 -1,-3
-----------AAACCCGUAGAUCCGAUCUUG---
- 40 -1,-2
-----------AAACCCGUAGAUCCGAUCUUGU--
- 207 -1,-1
-----------AAACCCGUAGAUCCGAUCUUGUa---
- 4 -1,-1
-----------AAACCCGUAGAUCCGAUCUUGUG---
- 17 -1,0
-----------AAACCCGUAGAUCCGAUCUUGUGa--
- 2 -1,0
-----------AAACCCGUAGAUCCGAUCUUGUGu--
- 8 -1,0

------------AACCCGUAGAUCCGAUCU--

-	531	0,-4

------------AACCCGUAGAUCCGAUCUa---

-	9	0,-4

------------AACCCGUAGAUCCGAUCUU---

-	1979	0,-3

------------AACCCGUAGAUCCGAUCUUa--

-	44	0,-3

------------AACCCGUAGAUCCGAUCUUau---

-	12	0,-3

------------AACCCGUAGAUCCGAUCUUu--

-	33	0,-3

------------AACCCGUAGAUCCGAUCUUuu---

-	17	0,-3

------------AACCCGUAGAUCCGAUCUUG--

-	7817	0,-2

------------AACCCGUAGAUCCGAUCUUGa---

-	131	0,-2

------------AACCCGUAGAUCCGAUCUUGaa--

-	8	0,-2

------------AACCCGUAGAUCCGAUCUUGU---

-	45842	0,-1

------------AACCCGUAGAUCCGAUCUUGUa--

-	567	0,-1

------------AACCCGUAGAUCCGAUCUUGUaa---

-	124	0,-1

------------AACCCGUAGAUCCGAUCUUGUau---

-	18	0,-1

------------AACCCGUAGAUCCGAUCUUGUu--

-	113	0,-1

------------AACCCGUAGAUCCGAUCUUGUuu---

-	24	0,-1

------------AACCCGUAGAUCCGAUCUUGUG--

-	56791	0,0

------------AACCCGUAGAUCCGAUCUUGUGa---

-	15497	0,0

------------AACCCGUAGAUCCGAUCUUGUGaa--

-	374	0,0

------------AACCCGUAGAUCCGAUCUUGUGau--
- 29 0,0
------------AACCCGUAGAUCCGAUCUUGUGu---
- 16634 0,0
------------AACCCGUAGAUCCGAUCUUGUGuu---
- 67 0,0
------------AACCCGUAGAUCCGAUCUUGUGG---
- 43 0,1
-------------ACCCGUAGAUCCGAUCUU---
- 5 1,-3
-------------ACCCGUAGAUCCGAUCUUG--
- 4 1,-2
-------------ACCCGUAGAUCCGAUCUUGU---
- 41 1,-1
-------------ACCCGUAGAUCCGAUCUUGUG--
- 49 1,0
-------------ACCCGUAGAUCCGAUCUUGUGa---
- 14 1,0
-------------ACCCGUAGAUCCGAUCUUGUGaa--
- 2 1,0
-------------ACCCGUAGAUCCGAUCUUGUGu---
- 23 1,0
---CAAGCUCGCUUCUAUGGG-------------
- 6 0,-4
---CAAGCUCGCUUCUAUGGGU------------
- 5 0,-3
---CAAGCUCGCUUCUAUGGGUCU----------
- 17 0,-1
---CAAGCUCGCUUCUAUGGGUCUG---------
- 12 0,0
---CAAGCUCGCUUCUAUGGGUCUGa--------
- 12 0,0
---CAAGCUCGCUUCUAUGGGUCUGU--------
- 29 0,1
---CAAGCUCGCUUCUAUGGGUCUGUu-------
- 2 0,1
--AAGCUCGCUUCUAUGGGU------------
- 2 1,-3

--AAGCUCGCUUCUAUGGGUC-----------
- 2 1,-2
--AAGCUCGCUUCUAUGGGUCUGU--------
- 3 1,1

>hsa-mir-99b MA:7-28;mi:45-66 SRX018957
ggcaccCACCCGUAGAACCGACCUUGCGgggccuucgccgcacaCAAGCUCGUGUCUGUGGGUCCGuguc
((((..(((((((((..(((.((((.(((((....))).).).)))).)))..)))))))))..)))))
------CACCCGUAGAACCGACCUU-- 2 0,-3
------CACCCGUAGAACCGACCUUG--- 11 0,-2
------CACCCGUAGAACCGACCUUGC-- 123 0,-1
------CACCCGUAGAACCGACCUUGCa--------------------------------------- 4 0,-1
------CACCCGUAGAACCGACCUUGCaa-------------------------------------- 2 0,-1
------CACCCGUAGAACCGACCUUGCau-------------------------------------- 3 0,-1
------CACCCGUAGAACCGACCUUGCG--------------------------------------- 195 0,0
------CACCCGUAGAACCGACCUUGCGa-------------------------------------- 86 0,0
------CACCCGUAGAACCGACCUUGCGaa------------------------------------- 3 0,0
------CACCCGUAGAACCGACCUUGCGau------------------------------------- 2 0,0
------CACCCGUAGAACCGACCUUGCGu-------------------------------------- 73 0,0
------CACCCGUAGAACCGACCUUGCGG-------------------------------------- 18 0,1
------CACCCGUAGAACCGACCUUGCGGu------------------------------------- 2 0,1
-------ACCCGUAGAACCGACCUUGC-- 3 1,-1
-------ACCCGUAGAACCGACCUUGCG--------------------------------------- 4 1,0
-------ACCCGUAGAACCGACCUUGCGa-------------------------------------- 2 1,0
-------ACCCGUAGAACCGACCUUGCGu-------------------------------------- 2 1,0
--CAAGCUCGUGUCUGUGGGUC------ 4 0,-2
--CAAGCUCGUGUCUGUGGGUCu----- 2 0,-2
--CAAGCUCGUGUCUGUGGGUCC----- 2 0,-1
--CAAGCUCGUGUCUGUGGGUCCGa--- 7 0,0
--CAAGCUCGUGUCUGUGGGUCCGU--- 2 0,1

>hsa-let-7a-1 MA:6-27;mi:57-77 SRX018958
ugggaUGAGGUAGUAGGUUGUAUAGUUuuagggucacacccaccacugggagauaaCUAUACAAUCUACUGUCUUUCcua
(((((.(((..((((((((((((((((.........................)))))))))))))))).))))))))
----AUGAGGUAGUAGGUUGUAU--
14 -1,-4
----AUGAGGUAGUAGGUUGUAUAGU---
4 -1,-1

----AUGAGGUAGUAGGUUGUAUAGUU--

20 -1,0

----AUGAGGUAGUAGGUUGUAUAGUUa---

3 -1,0

----AUGAGGUAGUAGGUUGUAUAGUUU---

2 -1,1

-----UGAGGUAGUAGGUUGUAU--

1382 0,-4

-----UGAGGUAGUAGGUUGUAUu---

90 0,-4

-----UGAGGUAGUAGGUUGUAUuu--

88 0,-4

-----UGAGGUAGUAGGUUGUAUA---

8212 0,-3

-----UGAGGUAGUAGGUUGUAUAa--

406 0,-3

-----UGAGGUAGUAGGUUGUAUAaa---

95 0,-3

-----UGAGGUAGUAGGUUGUAUAau---

15 0,-3

-----UGAGGUAGUAGGUUGUAUAu--

148 0,-3

-----UGAGGUAGUAGGUUGUAUAuu---

20 0,-3

-----UGAGGUAGUAGGUUGUAUAG--

15057 0,-2

-----UGAGGUAGUAGGUUGUAUAGa---

361 0,-2

-----UGAGGUAGUAGGUUGUAUAGaa--

66 0,-2

-----UGAGGUAGUAGGUUGUAUAGau--

191 0,-2

-----UGAGGUAGUAGGUUGUAUAGU---

49093 0,-1

-----UGAGGUAGUAGGUUGUAUAGUa--

2283 0,-1

-----UGAGGUAGUAGGUUGUAUAGUaa---

3110 0,-1

-----UGAGGUAGUAGGUUGUAUAGUau---

680 0,-1

-----UGAGGUAGUAGGUUGUAUAGUU--

233014 0,0

-----UGAGGUAGUAGGUUGUAUAGUUa---

35001 0,0

-----UGAGGUAGUAGGUUGUAUAGUUaa--

4564 0,0

-----UGAGGUAGUAGGUUGUAUAGUUau--

1317 0,0

-----UGAGGUAGUAGGUUGUAUAGUUU---

36370 0,1

-----UGAGGUAGUAGGUUGUAUAGUUUU--

895 0,2

-----UGAGGUAGUAGGUUGUAUAGUUUUu---

15 0,2

-----UGAGGUAGUAGGUUGUAUAGUUUUA---

11 0,3

------GAGGUAGUAGGUUGUAUA---

21 1,-3

------GAGGUAGUAGGUUGUAUAa--

2 1,-3

------GAGGUAGUAGGUUGUAUAG--

58 1,-2

------GAGGUAGUAGGUUGUAUAGau--

2 1,-2

------GAGGUAGUAGGUUGUAUAGU---

177 1,-1

------GAGGUAGUAGGUUGUAUAGUa--

4 1,-1

------GAGGUAGUAGGUUGUAUAGUaa---

12 1,-1

------GAGGUAGUAGGUUGUAUAGUau---

5 1,-1

------GAGGUAGUAGGUUGUAUAGUU--

759 1,0

------GAGGUAGUAGGUUGUAUAGUUa---

125 1,0

------GAGGUAGUAGGUUGUAUAGUUaa--

30 1,0

------GAGGUAGUAGGUUGUAUAGUUau--

6 1,0

------GAGGUAGUAGGUUGUAUAGUUU---

134 1,1

------GAGGUAGUAGGUUGUAUAGUUUU--

7 1,2

-------AGGUAGUAGGUUGUAUAG--

3 2,-2

-------AGGUAGUAGGUUGUAUAGU---

11 2,-1

-------AGGUAGUAGGUUGUAUAGUU--

110 2,0

-------AGGUAGUAGGUUGUAUAGUUa---

13 2,0

-------AGGUAGUAGGUUGUAUAGUUU---

28 2,1

-------AGGUAGUAGGUUGUAUAGUUUU--

2 2,2

---CUAUACAAUCUACUGUCUU-----

2 0,-2

---CUAUACAAUCUACUGUCUUU----

2 0,-1

---CUAUACAAUCUACUGUCUUUC---

8 0,0

---CUAUACAAUCUACUGUCUUUCa--

2 0,0

---CUAUACAAUCUACUGUCUUUCu--

10 0,0

---CUAUACAAUCUACUGUCUUUCCa-

2 0,1

---AUACAAUCUACUGUCUUU----

2 2,-1

---AUACAAUCUACUGUCUUUCCU-

7 2,2

>hsa-let-7a-2 MA:5-26;mi:50-71 SRX018958

agguUGAGGUAGUAGGUUGUAUAGUUuagaauuacaucaagggagauaaCUGUACAGCCUCCUAGCUUUCCu
(((..(((.(((.(((((((((((((.....(..(.....)..)...))))))))))))).))).))).)))
---UUGAGGUAGUAGGUUGUAUA--- 2
-1,-3
---UUGAGGUAGUAGGUUGUAUAG-- 3
-1,-2
---UUGAGGUAGUAGGUUGUAUAGU--- 9
-1,-1
---UUGAGGUAGUAGGUUGUAUAGUU-- 6
-1,0
---UUGAGGUAGUAGGUUGUAUAGUUa--- 2
-1,0
----UGAGGUAGUAGGUUGUAU-- 1382
0,-4
----UGAGGUAGUAGGUUGUAUu--- 90
0,-4
----UGAGGUAGUAGGUUGUAUuu-- 88
0,-4
----UGAGGUAGUAGGUUGUAUA--- 8212
0,-3
----UGAGGUAGUAGGUUGUAUAa-- 406
0,-3
----UGAGGUAGUAGGUUGUAUAaa--- 95
0,-3
----UGAGGUAGUAGGUUGUAUAau--- 15
0,-3
----UGAGGUAGUAGGUUGUAUAu-- 148
0,-3
----UGAGGUAGUAGGUUGUAUAuu--- 20
0,-3
----UGAGGUAGUAGGUUGUAUAG--
15057 0,-2
----UGAGGUAGUAGGUUGUAUAGa--- 361
0,-2
----UGAGGUAGUAGGUUGUAUAGaa-- 66
0,-2
----UGAGGUAGUAGGUUGUAUAGau-- 191
0,-2

----UGAGGUAGUAGGUUGUAUAGU--
49093 0,-1
----UGAGGUAGUAGGUUGUAUAGUa-- 2283
0,-1
----UGAGGUAGUAGGUUGUAUAGUaa--- 3110
0,-1
----UGAGGUAGUAGGUUGUAUAGUau--- 680
0,-1
----UGAGGUAGUAGGUUGUAUAGUU--
233014 0,0
----UGAGGUAGUAGGUUGUAUAGUUa---
35001 0,0
----UGAGGUAGUAGGUUGUAUAGUUaa-- 4564
0,0
----UGAGGUAGUAGGUUGUAUAGUUau-- 1317
0,0
----UGAGGUAGUAGGUUGUAUAGUUU---
36370 0,1
----UGAGGUAGUAGGUUGUAUAGUUUA-- 402
0,2
----UGAGGUAGUAGGUUGUAUAGUUUAa--- 11
0,2
----UGAGGUAGUAGGUUGUAUAGUUUAu--- 10
0,2
-----GAGGUAGUAGGUUGUAUA--- 21
1,-3
-----GAGGUAGUAGGUUGUAUAa-- 2
1,-3
-----GAGGUAGUAGGUUGUAUAG-- 58
1,-2
-----GAGGUAGUAGGUUGUAUAGau-- 2
1,-2
-----GAGGUAGUAGGUUGUAUAGU--- 177
1,-1
-----GAGGUAGUAGGUUGUAUAGUa-- 4
1,-1
-----GAGGUAGUAGGUUGUAUAGUaa--- 12
1,-1

-----GAGGUAGUAGGUUGUAUAGUau--- 5
 1,-1
-----GAGGUAGUAGGUUGUAUAGUU-- 759
 1,0
-----GAGGUAGUAGGUUGUAUAGUUa--- 125
 1,0
-----GAGGUAGUAGGUUGUAUAGUUaa-- 30
 1,0
-----GAGGUAGUAGGUUGUAUAGUUau-- 6
 1,0
-----GAGGUAGUAGGUUGUAUAGUUU--- 134
 1,1
-----GAGGUAGUAGGUUGUAUAGUUUA-- 2
 1,2
------AGGUAGUAGGUUGUAUAG-- 3
 2,-2
------AGGUAGUAGGUUGUAUAGU--- 11
 2,-1
------AGGUAGUAGGUUGUAUAGUU-- 110
 2,0
------AGGUAGUAGGUUGUAUAGUUa--- 13
 2,0
------AGGUAGUAGGUUGUAUAGUUU--- 28
 2,1

>hsa-let-7a-3 MA:4-25;mi:52-72 SRX018958
gggUGAGGUAGUAGGUUGUAUAGUUuggggcucugcccugcuaugggauaaCUAUACAAUCUACUGUCUUUCcu
(((.(((..((((((((((((((((((((((...)))))).........))))))))))))))))..))).)))
--GUGAGGUAGUAGGUUGUAUA-- 2
 -1,-3
--GUGAGGUAGUAGGUUGUAUAG--- 32
 -1,-2
--GUGAGGUAGUAGGUUGUAUAGU-- 42
 -1,-1
--GUGAGGUAGUAGGUUGUAUAGUU--- 25
 -1,0
---UGAGGUAGUAGGUUGUAU-- 1382
 0,-4

---UGAGGUAGUAGGUUGUAUu-- 90
0,-4
---UGAGGUAGUAGGUUGUAUuu--- 88
0,-4
---UGAGGUAGUAGGUUGUAUA-- 8212
0,-3
---UGAGGUAGUAGGUUGUAUAa--- 406
0,-3
---UGAGGUAGUAGGUUGUAUAaa-- 95
0,-3
---UGAGGUAGUAGGUUGUAUAau-- 15
0,-3
---UGAGGUAGUAGGUUGUAUAu--- 148
0,-3
---UGAGGUAGUAGGUUGUAUAuu-- 20
0,-3
---UGAGGUAGUAGGUUGUAUAG---
15057 0,-2
---UGAGGUAGUAGGUUGUAUAGa-- 361
0,-2
---UGAGGUAGUAGGUUGUAUAGaa--- 66
0,-2
---UGAGGUAGUAGGUUGUAUAGau--- 191
0,-2
---UGAGGUAGUAGGUUGUAUAGU--
49093 0,-1
---UGAGGUAGUAGGUUGUAUAGUa--- 2283
0,-1
---UGAGGUAGUAGGUUGUAUAGUaa-- 3110
0,-1
---UGAGGUAGUAGGUUGUAUAGUau-- 680
0,-1
---UGAGGUAGUAGGUUGUAUAGUU---
233014 0,0
---UGAGGUAGUAGGUUGUAUAGUUa--
35001 0,0
---UGAGGUAGUAGGUUGUAUAGUUaa--- 4564
0,0

---UGAGGUAGUAGGUUGUAUAGUUau-- 1317
0,0
---UGAGGUAGUAGGUUGUAUAGUUU--
36370 0,1
---UGAGGUAGUAGGUUGUAUAGUUUG-- 11
0,2
---UGAGGUAGUAGGUUGUAUAGUUUGG--- 2
0,3
----GAGGUAGUAGGUUGUAUA-- 21
1,-3
----GAGGUAGUAGGUUGUAUAa--- 2
1,-3
----GAGGUAGUAGGUUGUAUAG--- 58
1,-2
----GAGGUAGUAGGUUGUAUAGau--- 2
1,-2
----GAGGUAGUAGGUUGUAUAGU-- 177
1,-1
----GAGGUAGUAGGUUGUAUAGUa--- 4
1,-1
----GAGGUAGUAGGUUGUAUAGUaa-- 12
1,-1
----GAGGUAGUAGGUUGUAUAGUau-- 5
1,-1
----GAGGUAGUAGGUUGUAUAGUU--- 759
1,0
----GAGGUAGUAGGUUGUAUAGUUa-- 125
1,0
----GAGGUAGUAGGUUGUAUAGUUaa--- 30
1,0
----GAGGUAGUAGGUUGUAUAGUUau--- 6
1,0
----GAGGUAGUAGGUUGUAUAGUUU-- 134
1,1
-----AGGUAGUAGGUUGUAUAG--- 3
2,-2
-----AGGUAGUAGGUUGUAUAGU-- 11
2,-1

-----AGGUAGUAGGUUGUAUAGUU--- 110 2,0
-----AGGUAGUAGGUUGUAUAGUUa-- 13 2,0
-----AGGUAGUAGGUUGUAUAGUUU-- 28 2,1
---CUAUACAAUCUACUGUCUU---- 2 0,-2
---CUAUACAAUCUACUGUCUUU--- 2 0,-1
---CUAUACAAUCUACUGUCUUUC-- 8 0,0
---CUAUACAAUCUACUGUCUUUCa- 2 0,0
---CUAUACAAUCUACUGUCUUUCu- 10 0,0
---CUAUACAAUCUACUGUCUUUCCa 2 0,1
---AUACAAUCUACUGUCUUU--- 2 2,-1
---AUACAAUCUACUGUCUUUCCU 7 2,2

>hsa-let-7b MA:6-27;mi:60-81 SRX018958
cggggUGAGGUAGUAGGUUGUGUGGUUucagggcagugauguugccccucggaagauaaCUAUACAACCUACUGCCUUCCCug
(((((.(((((((((((((((((((((..............................))))))))))))))))))))))))))
-----UGAGGUAGUAGGUUGUGU-- 4011 0,-4
-----UGAGGUAGUAGGUUGUGUa--- 25 0,-4
-----UGAGGUAGUAGGUUGUGUaa-- 158 0,-4
-----UGAGGUAGUAGGUUGUGUau-- 15 0,-4
-----UGAGGUAGUAGGUUGUGUu--- 19 0,-4

-----UGAGGUAGUAGGUUGUGUuu--
--- 11 0,-4
-----UGAGGUAGUAGGUUGUGUG---
--- 14742 0,-3
-----UGAGGUAGUAGGUUGUGUGa--
--- 115 0,-3
-----UGAGGUAGUAGGUUGUGUGaa---
--- 18 0,-3
-----UGAGGUAGUAGGUUGUGUGau---
--- 3 0,-3
-----UGAGGUAGUAGGUUGUGUGu--
--- 158 0,-3
-----UGAGGUAGUAGGUUGUGUGuu---
--- 40 0,-3
-----UGAGGUAGUAGGUUGUGUGG--
--- 3473 0,-2
-----UGAGGUAGUAGGUUGUGUGGa---
--- 78 0,-2
-----UGAGGUAGUAGGUUGUGUGGaa--
--- 46 0,-2
-----UGAGGUAGUAGGUUGUGUGGau--
--- 38 0,-2
-----UGAGGUAGUAGGUUGUGUGGU---
--- 4839 0,-1
-----UGAGGUAGUAGGUUGUGUGGUa--
--- 758 0,-1
-----UGAGGUAGUAGGUUGUGUGGUaa---
--- 3556 0,-1
-----UGAGGUAGUAGGUUGUGUGGUau---
--- 2978 0,-1
-----UGAGGUAGUAGGUUGUGUGGUU--
--- 18370 0,0
-----UGAGGUAGUAGGUUGUGUGGUUa---
--- 11628 0,0
-----UGAGGUAGUAGGUUGUGUGGUUaa--
--- 4231 0,0
-----UGAGGUAGUAGGUUGUGUGGUUau--
--- 8343 0,0

-----UGAGGUAGUAGGUUGUGUGGUUU---
--- 32546 0,1
-----UGAGGUAGUAGGUUGUGUGGUUUa--
--- 1872 0,1
-----UGAGGUAGUAGGUUGUGUGGUUUaa---
--- 276 0,1
-----UGAGGUAGUAGGUUGUGUGGUUUau---
--- 345 0,1
-----UGAGGUAGUAGGUUGUGUGGUUUu--
--- 4734 0,1
-----UGAGGUAGUAGGUUGUGUGGUUUuu---
--- 193 0,1
-----UGAGGUAGUAGGUUGUGUGGUUUC--
--- 49 0,2
-----UGAGGUAGUAGGUUGUGUGGUUUCA---
--- 2 0,3
------GAGGUAGUAGGUUGUGUG---
--- 66 1,-3
------GAGGUAGUAGGUUGUGUGu--
--- 2 1,-3
------GAGGUAGUAGGUUGUGUGG--
--- 17 1,-2
------GAGGUAGUAGGUUGUGUGGU---
--- 23 1,-1
------GAGGUAGUAGGUUGUGUGGUa--
--- 5 1,-1
------GAGGUAGUAGGUUGUGUGGUaa---
--- 32 1,-1
------GAGGUAGUAGGUUGUGUGGUau---
--- 27 1,-1
------GAGGUAGUAGGUUGUGUGGUU--
--- 123 1,0
------GAGGUAGUAGGUUGUGUGGUUa---
--- 72 1,0
------GAGGUAGUAGGUUGUGUGGUUaa--
--- 19 1,0
------GAGGUAGUAGGUUGUGUGGUUau--
--- 52 1,0

------GAGGUAGUAGGUUGUGUGGUUU---
--- 209 1,1
------GAGGUAGUAGGUUGUGUGGUUUa--
--- 12 1,1
------GAGGUAGUAGGUUGUGUGGUUUau---
--- 4 1,1
------GAGGUAGUAGGUUGUGUGGUUUu--
--- 43 1,1
------GAGGUAGUAGGUUGUGUGGUUUuu---
--- 4 1,1
-------AGGUAGUAGGUUGUGUGGUau---
--- 2 2,-1
-------AGGUAGUAGGUUGUGUGGUU--
--- 4 2,0
-------AGGUAGUAGGUUGUGUGGUUa---
--- 5 2,0
-------AGGUAGUAGGUUGUGUGGUUaa--
--- 2 2,0
-------AGGUAGUAGGUUGUGUGGUUau--
--- 9 2,0
-------AGGUAGUAGGUUGUGUGGUUU---
--- 24 2,1
-------AGGUAGUAGGUUGUGUGGUUUu--
--- 6 2,1
--CUAUACAACCUACUGCCUU--
--- 3 0,-3
--CUAUACAACCUACUGCCUUC-
--- 3 0,-2
--CUAUACAACCUACUGCCUUCu
--- 3 0,-2
--CUAUACAACCUACUGCCUUCC
--- 22 0,-1
--CUAUACAACCUACUGCCUUCC
a-- 5 0,-1
--CUAUACAACCUACUGCCUUCC
u-- 45 0,-1
--CUAUACAACCUACUGCCUUCC
uu- 9 0,-1

>hsa-let-7c MA:11-32;mi:33-54 SRX018958
gcauccggguUGAGGUAGUAGGUUGUAUGGUUUAGAGUUACACCCUGGGAGUUAacuguacaaccuucuagcuuuccuug
gagc
((.((((((..(((.(((.(((((((((((((..((.(..(.....)..).)))))))))))))).))).))).)..))))
))))
--------GUUGAGGUAGUAGGUUGUAUGGU---
---- 5 -2,-1
--------GUUGAGGUAGUAGGUUGUAUGGUU--
---- 7 -2,0
---------UUGAGGUAGUAGGUUGUAUG---
---- 15 -1,-3
---------UUGAGGUAGUAGGUUGUAUGG--
---- 48 -1,-2
---------UUGAGGUAGUAGGUUGUAUGGU---
---- 94 -1,-1
---------UUGAGGUAGUAGGUUGUAUGGUa--
---- 61 -1,-1
---------UUGAGGUAGUAGGUUGUAUGGUaa---
---- 4 -1,-1
---------UUGAGGUAGUAGGUUGUAUGGUau---
---- 25 -1,-1
---------UUGAGGUAGUAGGUUGUAUGGUU--
---- 331 -1,0
---------UUGAGGUAGUAGGUUGUAUGGUUa---
---- 7 -1,0
---------UUGAGGUAGUAGGUUGUAUGGUUau--
---- 5 -1,0
---------UUGAGGUAGUAGGUUGUAUGGUUU---
---- 20 -1,1
---------UUGAGGUAGUAGGUUGUAUGGUUUu--
---- 2 -1,1
----------UGAGGUAGUAGGUUGUAU--
---- 1382 0,-4
----------UGAGGUAGUAGGUUGUAUu---
---- 90 0,-4
----------UGAGGUAGUAGGUUGUAUuu--
---- 88 0,-4

----------UGAGGUAGUAGGUUGUAUG--

----   3853   0,-3

----------UGAGGUAGUAGGUUGUAUGa--

----   20 0,-3

----------UGAGGUAGUAGGUUGUAUGau--

----   4   0,-3

----------UGAGGUAGUAGGUUGUAUGu--

----   38 0,-3

----------UGAGGUAGUAGGUUGUAUGuu--

----   26 0,-3

----------UGAGGUAGUAGGUUGUAUGG--

----   6596   0,-2

----------UGAGGUAGUAGGUUGUAUGGa--

----   102 0,-2

----------UGAGGUAGUAGGUUGUAUGGaa--

----   148 0,-2

----------UGAGGUAGUAGGUUGUAUGGau--

----   124 0,-2

----------UGAGGUAGUAGGUUGUAUGGU--

----   15826   0,-1

----------UGAGGUAGUAGGUUGUAUGGUa--

----   4152   0,-1

----------UGAGGUAGUAGGUUGUAUGGUaa--

----   3286   0,-1

----------UGAGGUAGUAGGUUGUAUGGUau--

----   2815   0,-1

----------UGAGGUAGUAGGUUGUAUGGUU--

----   113282 0,0

----------UGAGGUAGUAGGUUGUAUGGUUa--

----   12076   0,0

----------UGAGGUAGUAGGUUGUAUGGUUaa--

----   2351   0,0

----------UGAGGUAGUAGGUUGUAUGGUUau--

----   2543   0,0

----------UGAGGUAGUAGGUUGUAUGGUUU--

----   23601   0,1

----------UGAGGUAGUAGGUUGUAUGGUUUu--

----   1291   0,1

----------UGAGGUAGUAGGUUGUAUGGUUUuu--
----   30   0,1
----------UGAGGUAGUAGGUUGUAUGGUUUA---
----   642 0,2
----------UGAGGUAGUAGGUUGUAUGGUUUAa--
----   59   0,2
----------UGAGGUAGUAGGUUGUAUGGUUUAu--
----   45   0,2
-----------GAGGUAGUAGGUUGUAUG--
----   56   1,-3
-----------GAGGUAGUAGGUUGUAUGG---
----   54   1,-2
-----------GAGGUAGUAGGUUGUAUGGU--
----   73   1,-1
-----------GAGGUAGUAGGUUGUAUGGUa---
----   51   1,-1
-----------GAGGUAGUAGGUUGUAUGGUaa--
----   34   1,-1
-----------GAGGUAGUAGGUUGUAUGGUau--
----   24   1,-1
-----------GAGGUAGUAGGUUGUAUGGUU---
----   938 1,0
-----------GAGGUAGUAGGUUGUAUGGUUa--
----   108 1,0
-----------GAGGUAGUAGGUUGUAUGGUUaa---
----   27   1,0
-----------GAGGUAGUAGGUUGUAUGGUUau---
----   38   1,0
-----------GAGGUAGUAGGUUGUAUGGUUU--
----   217 1,1
-----------GAGGUAGUAGGUUGUAUGGUUUu---
----   17   1,1
-----------GAGGUAGUAGGUUGUAUGGUUUA---
----   4   1,2
------------AGGUAGUAGGUUGUAUGGU--
----   6   2,-1
------------AGGUAGUAGGUUGUAUGGUaa--
----   3   2,-1

------------AGGUAGUAGGUUGUAUGGUU--
----   41   2,0
------------AGGUAGUAGGUUGUAUGGUUa---
----   5   2,0
------------AGGUAGUAGGUUGUAUGGUUau--
----   2   2,0
------------AGGUAGUAGGUUGUAUGGUUU---
----   23   2,1
------------AGGUAGUAGGUUGUAUGGUUUA--
----   3   2,2

>hsa-let-7d MA:8-29;mi:62-83   SRX018958
ccuaggaAGAGGUAGUAGGUUGCAUAGUUuuagggcagggauuuugcccacaaggagguaaCUAUACGACCUGCUGCCUU
UCUuagg
(((((((.(((((((((((((.((((((................................)))))).)))))))))))))
)))))))
------AAGAGGUAGUAGGUUGCAU---
------- 2   -1,-4
------AAGAGGUAGUAGGUUGCAUA--
------- 2   -1,-3
------AAGAGGUAGUAGGUUGCAUAG---
------- 2   -1,-2
------AAGAGGUAGUAGGUUGCAUAGU--
------- 14   -1,-1
------AAGAGGUAGUAGGUUGCAUAGUU---
------- 32   -1,0
-------AGAGGUAGUAGGUUGCAU---
------- 1595   0,-4
-------AGAGGUAGUAGGUUGCAUu--
------- 47   0,-4
-------AGAGGUAGUAGGUUGCAUuu---
------- 85   0,-4
-------AGAGGUAGUAGGUUGCAUA--
------- 1393   0,-3
-------AGAGGUAGUAGGUUGCAUAa---
------- 116 0,-3
-------AGAGGUAGUAGGUUGCAUAaa--
------- 10   0,-3

-------AGAGGUAGUAGGUUGCAUAau--
------- 7 0,-3
-------AGAGGUAGUAGGUUGCAUAu---
------- 9 0,-3
-------AGAGGUAGUAGGUUGCAUAuu--
------- 13 0,-3
-------AGAGGUAGUAGGUUGCAUAG---
------- 3375 0,-2
-------AGAGGUAGUAGGUUGCAUAGa--
------- 119 0,-2
-------AGAGGUAGUAGGUUGCAUAGaa---
------- 38 0,-2
-------AGAGGUAGUAGGUUGCAUAGau---
------- 153 0,-2
-------AGAGGUAGUAGGUUGCAUAGU--
------- 6070 0,-1
-------AGAGGUAGUAGGUUGCAUAGUa---
------- 1244 0,-1
-------AGAGGUAGUAGGUUGCAUAGUaa--
------- 364 0,-1
-------AGAGGUAGUAGGUUGCAUAGUau--
------- 235 0,-1
-------AGAGGUAGUAGGUUGCAUAGUU---
------- 92404 0,0
-------AGAGGUAGUAGGUUGCAUAGUUa--
------- 9176 0,0
-------AGAGGUAGUAGGUUGCAUAGUUaa---
------- 480 0,0
-------AGAGGUAGUAGGUUGCAUAGUUau---
------- 311 0,0
-------AGAGGUAGUAGGUUGCAUAGUUU--
------- 15598 0,1
-------AGAGGUAGUAGGUUGCAUAGUUUa---
------- 152 0,1
-------AGAGGUAGUAGGUUGCAUAGUUUaa--
------- 5 0,1
-------AGAGGUAGUAGGUUGCAUAGUUUau--
------- 5 0,1

-------AGAGGUAGUAGGUUGCAUAGUUUU--
------- 694 0,2
-------AGAGGUAGUAGGUUGCAUAGUUUUu---
------- 29 0,2
-------AGAGGUAGUAGGUUGCAUAGUUUUA---
------- 2 0,3
--------GAGGUAGUAGGUUGCAUA---
------- 7 1,-3
--------GAGGUAGUAGGUUGCAUAG--
------- 5 1,-2
--------GAGGUAGUAGGUUGCAUAGU---
------- 21 1,-1
--------GAGGUAGUAGGUUGCAUAGUa--
------- 3 1,-1
--------GAGGUAGUAGGUUGCAUAGUau---
------- 5 1,-1
--------GAGGUAGUAGGUUGCAUAGUU--
------- 221 1,0
--------GAGGUAGUAGGUUGCAUAGUUa---
------- 46 1,0
--------GAGGUAGUAGGUUGCAUAGUUaa--
------- 4 1,0
--------GAGGUAGUAGGUUGCAUAGUUU---
------- 86 1,1
--------GAGGUAGUAGGUUGCAUAGUUUa--
------- 2 1,1
--------GAGGUAGUAGGUUGCAUAGUUUU--
------- 4 1,2
---------AGGUAGUAGGUUGCAUAGUU--
------- 25 2,0
---------AGGUAGUAGGUUGCAUAGUUa---
------- 5 2,0
---------AGGUAGUAGGUUGCAUAGUUU---
------- 3 2,1
--CUAUACGACCUGCUGCCUU
U------ 2 0,-2
--CUAUACGACCUGCUGCCUU
Uu----- 2 0,-2

---CUAUACGACCUGCUGCCUU
UC----- 14 0,-1
---CUAUACGACCUGCUGCCUU
UCa---- 12 0,-1
---CUAUACGACCUGCUGCCUU
UCU---- 57 0,0
---CUAUACGACCUGCUGCCUU
UCUa--- 2 0,0
--UAUACGACCUGCUGCCUU
UC----- 2 1,-1
--UAUACGACCUGCUGCCUU
UCU---- 8 1,0

>hsa-let-7e MA:8-29;mi:53-74 SRX018958
cccgggcUGAGGUAGGAGGUUGUAUAGUUgaggaggacacccaaggagaucaCUAUACGGCCUCCUAGCUUUCCccagg
((.(((..(((.(((((((((((((((((.((...((....)).......))))))))))))))))).)))..))).))
------CUGAGGUAGGAGGUUGUAa--
2 -1,-5
------CUGAGGUAGGAGGUUGUAUA---
2 -1,-3
------CUGAGGUAGGAGGUUGUAUAG--
5 -1,-2
------CUGAGGUAGGAGGUUGUAUAGa---
2 -1,-2
------CUGAGGUAGGAGGUUGUAUAGU---
17 -1,-1
------CUGAGGUAGGAGGUUGUAUAGUaa---
2 -1,-1
------CUGAGGUAGGAGGUUGUAUAGUau---
3 -1,-1
------CUGAGGUAGGAGGUUGUAUAGUU--
57 -1,0
------CUGAGGUAGGAGGUUGUAUAGUUa---
12 -1,0
------CUGAGGUAGGAGGUUGUAUAGUUaa--
2 -1,0
------CUGAGGUAGGAGGUUGUAUAGUUu---
25 -1,0

-------UGAGGUAGGAGGUUGUAU--
1303 0,-4
-------UGAGGUAGGAGGUUGUAUu---
6 0,-4
-------UGAGGUAGGAGGUUGUAUuu--
2 0,-4
-------UGAGGUAGGAGGUUGUAUA---
852 0,-3
-------UGAGGUAGGAGGUUGUAUAa--
55 0,-3
-------UGAGGUAGGAGGUUGUAUAaa---
28 0,-3
-------UGAGGUAGGAGGUUGUAUAu--
11 0,-3
-------UGAGGUAGGAGGUUGUAUAuu---
5 0,-3
-------UGAGGUAGGAGGUUGUAUAG--
1527 0,-2
-------UGAGGUAGGAGGUUGUAUAGa---
58 0,-2
-------UGAGGUAGGAGGUUGUAUAGaa--
24 0,-2
-------UGAGGUAGGAGGUUGUAUAGau--
38 0,-2
-------UGAGGUAGGAGGUUGUAUAGU---
2921 0,-1
-------UGAGGUAGGAGGUUGUAUAGUa--
304 0,-1
-------UGAGGUAGGAGGUUGUAUAGUaa---
811 0,-1
-------UGAGGUAGGAGGUUGUAUAGUau---
258 0,-1
-------UGAGGUAGGAGGUUGUAUAGUU--
10806 0,0
-------UGAGGUAGGAGGUUGUAUAGUUa---
4066 0,0
-------UGAGGUAGGAGGUUGUAUAGUUaa--
752 0,0

-------UGAGGUAGGAGGUUGUAUAGUUau---

236 0,0

-------UGAGGUAGGAGGUUGUAUAGUUu--

3201 0,0

-------UGAGGUAGGAGGUUGUAUAGUUuu---

75 0,0

-------UGAGGUAGGAGGUUGUAUAGUUG--

470 0,1

-------UGAGGUAGGAGGUUGUAUAGUUGu---

61 0,1

-------UGAGGUAGGAGGUUGUAUAGUUGA---

24 0,2

--------GAGGUAGGAGGUUGUAUAG---

5 1,-2

--------GAGGUAGGAGGUUGUAUAGU--

8 1,-1

--------GAGGUAGGAGGUUGUAUAGUaa--

4 1,-1

--------GAGGUAGGAGGUUGUAUAGUU---

19 1,0

--------GAGGUAGGAGGUUGUAUAGUUa--

9 1,0

--------GAGGUAGGAGGUUGUAUAGUUaa---

2 1,0

--------GAGGUAGGAGGUUGUAUAGUUau---

3 1,0

--------GAGGUAGGAGGUUGUAUAGUUu--

13 1,0

---------AGGUAGGAGGUUGUAUAGUU---

3 2,0

---------AGGUAGGAGGUUGUAUAGUUa--

2 2,0

---CUAUACGGCCUCCUAGCUUU-------

2 0,-2

---CUAUACGGCCUCCUAGCUUUCC-----

2 0,0

--UAUACGGCCUCCUAGCUUUCCa----

2 1,0

>hsa-let-7f-1	MA:7-28;mi:63-84	SRX018958
ucagagUGAGGUAGUAGAUUGUAUAGUUgugggguagugauuuuacccuguucaggagauaaCUAUACAAUCUAUUGCCU
UCCCuga
((((.(..(((((((((((((((((((((((((((((.....))))))).........))))))))))))))))))))))
..)))))
------UGAGGUAGUAGAUUGUAU--
------- 1297	0,-4
------UGAGGUAGUAGAUUGUAUu---
------- 45	0,-4
------UGAGGUAGUAGAUUGUAUuu--
------- 81	0,-4
------UGAGGUAGUAGAUUGUAUA---
------- 11759	0,-3
------UGAGGUAGUAGAUUGUAUAa--
------- 178	0,-3
------UGAGGUAGUAGAUUGUAUAaa---
------- 64	0,-3
------UGAGGUAGUAGAUUGUAUAau---
------- 20	0,-3
------UGAGGUAGUAGAUUGUAUAu--
------- 29	0,-3
------UGAGGUAGUAGAUUGUAUAuu---
------- 17	0,-3
------UGAGGUAGUAGAUUGUAUAG--
------- 8334	0,-2
------UGAGGUAGUAGAUUGUAUAGa---
------- 405	0,-2
------UGAGGUAGUAGAUUGUAUAGaa--
------- 62	0,-2
------UGAGGUAGUAGAUUGUAUAGau--
------- 140	0,-2
------UGAGGUAGUAGAUUGUAUAGU---
------- 46467	0,-1
------UGAGGUAGUAGAUUGUAUAGUa--
------- 1506	0,-1
------UGAGGUAGUAGAUUGUAUAGUaa---
------- 4938	0,-1

------UGAGGUAGUAGAUUGUAUAGUau---
------- 743 0,-1
------UGAGGUAGUAGAUUGUAUAGUU--
------- 153565 0,0
------UGAGGUAGUAGAUUGUAUAGUUa---
------- 63702 0,0
------UGAGGUAGUAGAUUGUAUAGUUaa--
------- 6331 0,0
------UGAGGUAGUAGAUUGUAUAGUUau--
------- 1341 0,0
------UGAGGUAGUAGAUUGUAUAGUUG---
------- 1711 0,1
------UGAGGUAGUAGAUUGUAUAGUUGa--
------- 70 0,1
------UGAGGUAGUAGAUUGUAUAGUUGaa---
------- 3 0,1
------UGAGGUAGUAGAUUGUAUAGUUGU--
------- 95 0,2
-------GAGGUAGUAGAUUGUAUA---
------- 26 1,-3
-------GAGGUAGUAGAUUGUAUAa--
------- 2 1,-3
-------GAGGUAGUAGAUUGUAUAG--
------- 44 1,-2
-------GAGGUAGUAGAUUGUAUAGU---
------- 217 1,-1
-------GAGGUAGUAGAUUGUAUAGUa--
------- 10 1,-1
-------GAGGUAGUAGAUUGUAUAGUaa---
------- 32 1,-1
-------GAGGUAGUAGAUUGUAUAGUau---
------- 4 1,-1
-------GAGGUAGUAGAUUGUAUAGUU--
------- 822 1,0
-------GAGGUAGUAGAUUGUAUAGUUa---
------- 369 1,0
-------GAGGUAGUAGAUUGUAUAGUUaa--
------- 44 1,0

-------GAGGUAGUAGAUUGUAUAGUUau--
------- 20 1,0
-------GAGGUAGUAGAUUGUAUAGUUG---
------- 14 1,1
--------AGGUAGUAGAUUGUAUAGU---
------- 12 2,-1
--------AGGUAGUAGAUUGUAUAGUU--
------- 65 2,0
--------AGGUAGUAGAUUGUAUAGUUa---
------- 19 2,0
--------AGGUAGUAGAUUGUAUAGUUau--
------- 2   2,0
--------AGGUAGUAGAUUGUAUAGUUG---
------- 23 2,1
--------AGGUAGUAGAUUGUAUAGUUGU--
------- 33 2,2
--------AGGUAGUAGAUUGUAUAGUUGUa---
------- 3   2,2
---CUAUACAAUCUAUUGCCU
UCCu--- 7   0,-1

>hsa-let-7f-2   MA:8-29;mi:58-79   SRX018958
ugugggaUGAGGUAGUAGAUUGUAUAGUUuuagggucauaccccaucuuggagauaaCUAUACAGUCUACUGUCUUUCCc
acg
.((((((.(((..((((((((((((((((..........................))))))))))))))))..)))))))
)).
------AUGAGGUAGUAGAUUGUA--
--- 2   -1,-5
------AUGAGGUAGUAGAUUGUAa---
--- 3   -1,-5
------AUGAGGUAGUAGAUUGUAUA--
--- 3   -1,-3
------AUGAGGUAGUAGAUUGUAUAa---
--- 2   -1,-3
------AUGAGGUAGUAGAUUGUAUAG---
--- 3   -1,-2
------AUGAGGUAGUAGAUUGUAUAGU--
--- 44 -1,-1

------AUGAGGUAGUAGAUUGUAUAGUU---
--- 64 -1,0
------AUGAGGUAGUAGAUUGUAUAGUUa--
--- 5 -1,0
------AUGAGGUAGUAGAUUGUAUAGUUU--
--- 13 -1,1
-------UGAGGUAGUAGAUUGUAU---
--- 1297 0,-4
-------UGAGGUAGUAGAUUGUAUu--
--- 45 0,-4
-------UGAGGUAGUAGAUUGUAUuu---
--- 81 0,-4
-------UGAGGUAGUAGAUUGUAUA--
--- 11759 0,-3
-------UGAGGUAGUAGAUUGUAUAa---
--- 178 0,-3
-------UGAGGUAGUAGAUUGUAUAaa--
--- 64 0,-3
-------UGAGGUAGUAGAUUGUAUAau--
--- 20 0,-3
-------UGAGGUAGUAGAUUGUAUAu---
--- 29 0,-3
-------UGAGGUAGUAGAUUGUAUAuu--
--- 17 0,-3
-------UGAGGUAGUAGAUUGUAUAG---
--- 8334 0,-2
-------UGAGGUAGUAGAUUGUAUAGa--
--- 405 0,-2
-------UGAGGUAGUAGAUUGUAUAGaa---
--- 62 0,-2
-------UGAGGUAGUAGAUUGUAUAGau---
--- 140 0,-2
-------UGAGGUAGUAGAUUGUAUAGU--
--- 46467 0,-1
-------UGAGGUAGUAGAUUGUAUAGUa---
--- 1506 0,-1
-------UGAGGUAGUAGAUUGUAUAGUaa--
--- 4938 0,-1

-------UGAGGUAGUAGAUUGUAUAGUau---
--- 743 0,-1
-------UGAGGUAGUAGAUUGUAUAGUU--
--- 153565 0,0
-------UGAGGUAGUAGAUUGUAUAGUUa---
--- 63702 0,0
-------UGAGGUAGUAGAUUGUAUAGUUaa--
--- 6331 0,0
-------UGAGGUAGUAGAUUGUAUAGUUau--
--- 1341 0,0
-------UGAGGUAGUAGAUUGUAUAGUUU---
--- 38768 0,1
-------UGAGGUAGUAGAUUGUAUAGUUUa--
--- 137 0,1
-------UGAGGUAGUAGAUUGUAUAGUUUaa---
--- 9 0,1
-------UGAGGUAGUAGAUUGUAUAGUUUU--
--- 354 0,2
-------UGAGGUAGUAGAUUGUAUAGUUUUu---
--- 4 0,2
--------GAGGUAGUAGAUUGUAUA---
--- 26 1,-3
--------GAGGUAGUAGAUUGUAUAa--
--- 2 1,-3
--------GAGGUAGUAGAUUGUAUAG--
--- 44 1,-2
--------GAGGUAGUAGAUUGUAUAGU---
--- 217 1,-1
--------GAGGUAGUAGAUUGUAUAGUa--
--- 10 1,-1
--------GAGGUAGUAGAUUGUAUAGUaa---
--- 32 1,-1
--------GAGGUAGUAGAUUGUAUAGUau---
--- 4 1,-1
--------GAGGUAGUAGAUUGUAUAGUU--
--- 822 1,0
--------GAGGUAGUAGAUUGUAUAGUUa---
--- 369 1,0

--------GAGGUAGUAGAUUGUAUAGUUaa---
--- 44 1,0
--------GAGGUAGUAGAUUGUAUAGUUau---
--- 20 1,0
--------GAGGUAGUAGAUUGUAUAGUUU--
--- 210 1,1
--------GAGGUAGUAGAUUGUAUAGUUUU---
--- 2   1,2
---------AGGUAGUAGAUUGUAUAGU--
--- 12 2,-1
---------AGGUAGUAGAUUGUAUAGUU---
--- 65 2,0
---------AGGUAGUAGAUUGUAUAGUUa--
--- 19 2,0
---------AGGUAGUAGAUUGUAUAGUUau---
--- 2   2,0
---------AGGUAGUAGAUUGUAUAGUUU--
--- 27 2,1

>hsa-let-7g MA:5-26;mi:62-82   SRX018958
aggcUGAGGUAGUAGUUUGUACAGUUugagggucuaugauaccacccgguacaggagauaaCUGUACAGGCCACUGCCUU
GCca
.(((.((((((((.((((((((((((.....((((.((.((((....))))))..)))))))))))))))).))))))))
))).
---CUGAGGUAGUAGUUUGUA---
----   4   -1,-5
---CUGAGGUAGUAGUUUGUACAG--
----   7   -1,-2
---CUGAGGUAGUAGUUUGUACAGU---
----   65 -1,-1
---CUGAGGUAGUAGUUUGUACAGUU--
----   77 -1,0
---CUGAGGUAGUAGUUUGUACAGUUa---
----   6   -1,0
---CUGAGGUAGUAGUUUGUACAGUUU---
----   10 -1,1
----UGAGGUAGUAGUUUGUAC--
----   91 0,-4

----UGAGGUAGUAGUUUGUACA--
---- 197 0,-3
----UGAGGUAGUAGUUUGUACAa---
---- 42 0,-3
----UGAGGUAGUAGUUUGUACAaa--
---- 8 0,-3
----UGAGGUAGUAGUUUGUACAau--
---- 2 0,-3
----UGAGGUAGUAGUUUGUACAu---
---- 3 0,-3
----UGAGGUAGUAGUUUGUACAG---
---- 828 0,-2
----UGAGGUAGUAGUUUGUACAGa--
---- 52 0,-2
----UGAGGUAGUAGUUUGUACAGaa---
---- 6 0,-2
----UGAGGUAGUAGUUUGUACAGau---
---- 16 0,-2
----UGAGGUAGUAGUUUGUACAGU--
---- 5535 0,-1
----UGAGGUAGUAGUUUGUACAGUa---
---- 96 0,-1
----UGAGGUAGUAGUUUGUACAGUaa--
---- 67 0,-1
----UGAGGUAGUAGUUUGUACAGUau--
---- 28 0,-1
----UGAGGUAGUAGUUUGUACAGUU---
---- 13941 0,0
----UGAGGUAGUAGUUUGUACAGUUa--
---- 2018 0,0
----UGAGGUAGUAGUUUGUACAGUUaa---
---- 134 0,0
----UGAGGUAGUAGUUUGUACAGUUau---
---- 56 0,0
----UGAGGUAGUAGUUUGUACAGUUU--
---- 1835 0,1
----UGAGGUAGUAGUUUGUACAGUUUa---
---- 32 0,1

----UGAGGUAGUAGUUUGUACAGUUUau--
----   2   0,1

----UGAGGUAGUAGUUUGUACAGUUUu---
----   77 0,1

----UGAGGUAGUAGUUUGUACAGUUUG---
----   2   0,2

-----GAGGUAGUAGUUUGUACA---
----   2   1,-3

-----GAGGUAGUAGUUUGUACAG--
----   23 1,-2

-----GAGGUAGUAGUUUGUACAGau--
----   2   1,-2

-----GAGGUAGUAGUUUGUACAGU---
----   68 1,-1

-----GAGGUAGUAGUUUGUACAGUa--
----   6   1,-1

-----GAGGUAGUAGUUUGUACAGUU--
----   321 1,0

-----GAGGUAGUAGUUUGUACAGUUa---
----   18 1,0

-----GAGGUAGUAGUUUGUACAGUUaa--
----   3   1,0

-----GAGGUAGUAGUUUGUACAGUUU---
----   20 1,1

------AGGUAGUAGUUUGUACAG--
----   2   2,-2

------AGGUAGUAGUUUGUACAGU---
----   3   2,-1

------AGGUAGUAGUUUGUACAGUU--
----   6   2,0

------AGGUAGUAGUUUGUACAGUUU---
----   8   2,1

---CUGUACAGGCCACUGCCUU
GCu-   3   0,0

>hsa-let-7i MA:6-27;mi:62-83   SRX018958

cuggcUGAGGUAGUAGUUUGUGCUGUUggucggguugugacauugcccgcuguggagauaaCUGCGCAAGCUACUGCCUU
GCUa

.((((.((((((((((((((((.(((((.(((((.........))))))).......))).))))))))))))))))
))))
----CUGAGGUAGUAGUUUGUGCU--
----	3	-1,-3
----CUGAGGUAGUAGUUUGUGCUGU--
----	2	-1,-1
----CUGAGGUAGUAGUUUGUGCUGUUa--
----	2	-1,0
-----UGAGGUAGUAGUUUGUGC---
----	519	0,-4
-----UGAGGUAGUAGUUUGUGCaa---
----	3	0,-4
-----UGAGGUAGUAGUUUGUGCU--
----	2175	0,-3
-----UGAGGUAGUAGUUUGUGCUu---
----	2	0,-3
-----UGAGGUAGUAGUUUGUGCUuu--
----	2	0,-3
-----UGAGGUAGUAGUUUGUGCUG---
----	319	0,-2
-----UGAGGUAGUAGUUUGUGCUGa--
----	4	0,-2
-----UGAGGUAGUAGUUUGUGCUGaa---
----	3	0,-2
-----UGAGGUAGUAGUUUGUGCUGU--
----	216	0,-1
-----UGAGGUAGUAGUUUGUGCUGUa---
----	7	0,-1
-----UGAGGUAGUAGUUUGUGCUGUaa--
----	13	0,-1
-----UGAGGUAGUAGUUUGUGCUGUau--
----	3	0,-1
-----UGAGGUAGUAGUUUGUGCUGUU---
----	303	0,0
-----UGAGGUAGUAGUUUGUGCUGUUa--
----	142	0,0
-----UGAGGUAGUAGUUUGUGCUGUUaa---
----	22	0,0

-----UGAGGUAGUAGUUUGUGCUGUUau---
----	3	0,0
-----UGAGGUAGUAGUUUGUGCUGUUu--
----	276	0,0
-----UGAGGUAGUAGUUUGUGCUGUUuu---
----	44	0,0
-----UGAGGUAGUAGUUUGUGCUGUUG--
----	8	0,1
------GAGGUAGUAGUUUGUGCU--
----	16	1,-3
------GAGGUAGUAGUUUGUGCUG---
----	5	1,-2
------GAGGUAGUAGUUUGUGCUGU--
----	5	1,-1
------GAGGUAGUAGUUUGUGCUGUU---
----	14	1,0
------GAGGUAGUAGUUUGUGCUGUUa--
----	3	1,0
------GAGGUAGUAGUUUGUGCUGUUaa---
----	2	1,0
------GAGGUAGUAGUUUGUGCUGUUu--
----	9	1,0
---CUGCGCAAGCUACUGCCUU
----	6	0,-3
---CUGCGCAAGCUACUGCCUU
G---	8	0,-2
---CUGCGCAAGCUACUGCCUU
GC--	2	0,-1

>hsa-mir-1-1	3p:46-67	SRX018958
ugggaaacauacuucuuuauaugcccauauggaccugcuaagcuaUGGAAUGUAAAGAAGUAUGUAUcuca
.((((.((((((((((((((((..((((((((.....)))...))))).))))))))))))))))).)))).
---UGGAAUGUAAAGAAGUAU--------	56	0,-4
---UGGAAUGUAAAGAAGUAUa-------	3	0,-4
---UGGAAUGUAAAGAAGUAUG-------	45	0,-3
---UGGAAUGUAAAGAAGUAUGU------	49	0,-2
---UGGAAUGUAAAGAAGUAUGUuu----	3	0,-2
---UGGAAUGUAAAGAAGUAUGUA-----	156	0,-1

---UGGAAUGUAAAGAAGUAUGUAa---- 102 0,-1
---UGGAAUGUAAAGAAGUAUGUAaa--- 9 0,-1
---UGGAAUGUAAAGAAGUAUGUAau--- 2 0,-1
---UGGAAUGUAAAGAAGUAUGUAU---- 2084
0,0
---UGGAAUGUAAAGAAGUAUGUAUa--- 53 0,0
---UGGAAUGUAAAGAAGUAUGUAUaa-- 2 0,0
--GGAAUGUAAAGAAGUAUGUAU---- 32 1,0
---GAAUGUAAAGAAGUAUGUAU---- 5 2,0

>hsa-mir-1-2 3p:53-74 SRX018958
accuacucagaguacauacuucuuuauguacccauaugaacauacaaugcuaUGGAAUGUAAAGAAGUAUGUAUuuuugg
uaggc
.((((((.(((((((((((((((((((((..(((((..............))))).(((((((((((((((((((((.))
)))).
--UGGAAUGUAAAGAAGUAU----------
----- 56 0,-4
--UGGAAUGUAAAGAAGUAUa---------
----- 3 0,-4
--UGGAAUGUAAAGAAGUAUG---------
----- 45 0,-3
--UGGAAUGUAAAGAAGUAUGU--------
----- 49 0,-2
--UGGAAUGUAAAGAAGUAUGUuu------
----- 3 0,-2
--UGGAAUGUAAAGAAGUAUGUA-------
----- 156 0,-1
--UGGAAUGUAAAGAAGUAUGUAa------
----- 102 0,-1
--UGGAAUGUAAAGAAGUAUGUAaa-----
----- 9 0,-1
--UGGAAUGUAAAGAAGUAUGUAau-----
----- 2 0,-1
--UGGAAUGUAAAGAAGUAUGUAU------
----- 2084 0,0
--UGGAAUGUAAAGAAGUAUGUAUa-----
----- 53 0,0
--UGGAAUGUAAAGAAGUAUGUAUaa----

-----	2	0,0
--UGGAAUGUAAAGAAGUAUGUAUU-----
-----	56	0,1
---GGAAUGUAAAGAAGUAUGUAU------
-----	32	1,0
---GGAAUGUAAAGAAGUAUGUAUU-----
-----	3	1,1
--GAAUGUAAAGAAGUAUGUAU------
-----	5	2,0

>hsa-mir-100	MA:13-34;mi:48-69	SRX018958
ccuguugccacaAACCCGUAGAUCCGAACUUGUGguauuaguccgcaCAAGCUUGUAUCUAUAGGUAUGugucuguuagg
((((..(.((((.(((..(((((.(((.(((((((........).)))))).))).)))))..))).)))).)...))))
------------AACCCGUAGAUCCGAACU--
	1017	0,-4
------------AACCCGUAGAUCCGAACUa---
	12	0,-4
------------AACCCGUAGAUCCGAACUU---
	499	0,-3
------------AACCCGUAGAUCCGAACUUa--
	3	0,-3
------------AACCCGUAGAUCCGAACUUau---
	2	0,-3
------------AACCCGUAGAUCCGAACUUu--
	2	0,-3
------------AACCCGUAGAUCCGAACUUuu---
	10	0,-3
------------AACCCGUAGAUCCGAACUUG--
	2109	0,-2
------------AACCCGUAGAUCCGAACUUGa---
	57	0,-2
------------AACCCGUAGAUCCGAACUUGaa--
	3	0,-2
------------AACCCGUAGAUCCGAACUUGU---
	10290	0,-1
------------AACCCGUAGAUCCGAACUUGUa--
	94	0,-1
------------AACCCGUAGAUCCGAACUUGUaa---

87 0,-1

------------AACCCGUAGAUCCGAACUUGUau---

19 0,-1

------------AACCCGUAGAUCCGAACUUGUu--

76 0,-1

------------AACCCGUAGAUCCGAACUUGUuu---

26 0,-1

------------AACCCGUAGAUCCGAACUUGUG--

13858 0,0

------------AACCCGUAGAUCCGAACUUGUGa---

13174 0,0

------------AACCCGUAGAUCCGAACUUGUGaa--

839 0,0

------------AACCCGUAGAUCCGAACUUGUGau--

58 0,0

------------AACCCGUAGAUCCGAACUUGUGu---

8993 0,0

------------AACCCGUAGAUCCGAACUUGUGuu--

72 0,0

------------AACCCGUAGAUCCGAACUUGUGG---

42 0,1

------------AACCCGUAGAUCCGAACUUGUGGU--

2 0,2

-------------ACCCGUAGAUCCGAACUUG--

2 1,-2

-------------ACCCGUAGAUCCGAACUUGU---

9 1,-1

-------------ACCCGUAGAUCCGAACUUGUG--

17 1,0

-------------ACCCGUAGAUCCGAACUUGUGa---

28 1,0

-------------ACCCGUAGAUCCGAACUUGUGaa--

12 1,0

-------------ACCCGUAGAUCCGAACUUGUGu---

9 1,0

>hsa-mir-101-1 mi:11-32;MA:47-67 SRX018958

ugcccuggcuCAGUUAUCACAGUGCUGAUGCUgucuauucuaaaggUACAGUACUGUGAUAACUGAAggauggca

.(((.....(((((((((((((((((.((((...........)))))))))))))))))))))).....))).
---GGUACAGUACUGUGAUAACU----------- 3
-2,-3
--GUACAGUACUGUGAUAAC------------ 1948
-1,-4
--GUACAGUACUGUGAUAACa----------- 16
-1,-4
--GUACAGUACUGUGAUAACaa---------- 3
-1,-4
--GUACAGUACUGUGAUAACU-----------
63242 -1,-3
--GUACAGUACUGUGAUAACUa---------- 29
-1,-3
--GUACAGUACUGUGAUAACUaa--------- 44
-1,-3
--GUACAGUACUGUGAUAACUau--------- 2
-1,-3
--GUACAGUACUGUGAUAACUu---------- 25
-1,-3
--GUACAGUACUGUGAUAACUG---------- 4265
-1,-2
--GUACAGUACUGUGAUAACUGu--------- 130
-1,-2
--GUACAGUACUGUGAUAACUGuu-------- 10
-1,-2
--GUACAGUACUGUGAUAACUGA---------
51714 -1,-1
--GUACAGUACUGUGAUAACUGAu-------- 502
-1,-1
--GUACAGUACUGUGAUAACUGAuu------- 10
-1,-1
--GUACAGUACUGUGAUAACUGAA--------
73289 -1,0
--GUACAGUACUGUGAUAACUGAAa-------
18127 -1,0
--GUACAGUACUGUGAUAACUGAAaa------ 696
-1,0
--GUACAGUACUGUGAUAACUGAAau------ 38

-1,0

---GUACAGUACUGUGAUAACUGAAu------- 261

-1,0

---GUACAGUACUGUGAUAACUGAAuu------ 6

-1,0

---GUACAGUACUGUGAUAACUGAAG------- 100

-1,1

---GUACAGUACUGUGAUAACUGAAGu------ 5

-1,1

---GUACAGUACUGUGAUAACUGAAGG------ 9

-1,2

---GUACAGUACUGUGAUAACUGAAGGu----- 2

-1,2

--UACAGUACUGUGAUAACU-----------

75423 0,-3

--UACAGUACUGUGAUAACUa---------- 18

0,-3

--UACAGUACUGUGAUAACUaa--------- 66

0,-3

--UACAGUACUGUGAUAACUau--------- 9

0,-3

--UACAGUACUGUGAUAACUu---------- 36

0,-3

--UACAGUACUGUGAUAACUG---------- 6904

0,-2

--UACAGUACUGUGAUAACUGu--------- 121

0,-2

--UACAGUACUGUGAUAACUGuu-------- 5

0,-2

--UACAGUACUGUGAUAACUGA---------

93514 0,-1

--UACAGUACUGUGAUAACUGAu-------- 564

0,-1

--UACAGUACUGUGAUAACUGAuu------- 448

0,-1

--UACAGUACUGUGAUAACUGAA--------

192208 0,0

--UACAGUACUGUGAUAACUGAAa-------

53831 0,0

--UACAGUACUGUGAUAACUGAAaa------ 1949

0,0

--UACAGUACUGUGAUAACUGAAau------ 226

0,0

--UACAGUACUGUGAUAACUGAAu------- 8877

0,0

--UACAGUACUGUGAUAACUGAAuu------ 318

0,0

--UACAGUACUGUGAUAACUGAAG-------

14629 0,1

--UACAGUACUGUGAUAACUGAAGu------ 2019

0,1

--UACAGUACUGUGAUAACUGAAGuu----- 42

0,1

--UACAGUACUGUGAUAACUGAAGG------ 41

0,2

--UACAGUACUGUGAUAACUGAAGGu----- 5

0,2

--UACAGUACUGUGAUAACUGAAGGA----- 25

0,3

---ACAGUACUGUGAUAACUG---------- 54

1,-2

---ACAGUACUGUGAUAACUGA--------- 527

1,-1

---ACAGUACUGUGAUAACUGAu-------- 2

1,-1

---ACAGUACUGUGAUAACUGAuu------- 8

1,-1

---ACAGUACUGUGAUAACUGAA-------- 1334

1,0

---ACAGUACUGUGAUAACUGAAa------- 516

1,0

---ACAGUACUGUGAUAACUGAAaa------ 39

1,0

---ACAGUACUGUGAUAACUGAAau------ 23

1,0

---ACAGUACUGUGAUAACUGAAu------- 127

1,0
---ACAGUACUGUGAUAACUGAAuu------ 8
1,0
---ACAGUACUGUGAUAACUGAAG------- 169
1,1
---ACAGUACUGUGAUAACUGAAGu------ 48
1,1
--CAGUACUGUGAUAACUGA--------- 57
2,-1
--CAGUACUGUGAUAACUGAA-------- 203
2,0
--CAGUACUGUGAUAACUGAAa------- 54
2,0
--CAGUACUGUGAUAACUGAAaa------ 9
2,0
--CAGUACUGUGAUAACUGAAau------ 6
2,0
--CAGUACUGUGAUAACUGAAu------- 12
2,0
--CAGUACUGUGAUAACUGAAuu------ 6
2,0
--CAGUACUGUGAUAACUGAAG------- 28
2,1
--CAGUACUGUGAUAACUGAAGu------ 30
2,1
--CAGUACUGUGAUAACUGAAGuu----- 4
2,1

>hsa-mir-101-2 3p:49-69 SRX018958
acuguccuuuuucgguuaucaugguaccgaugcuguauaucugaaaggUACAGUACUGUGAUAACUGAAgaaugguggu
((..((.((((((((((((((((((((.(.((((...........))))).))))))))))))))))))))).))..))
-------------GGUUAUCAUGGUACCGAUGCU---
2
--GGUACAGUACUGUGAUAACU-------------
3 -2,-3
---GUACAGUACUGUGAUAAC--------------
1948 -1,-4
---GUACAGUACUGUGAUAACa-------------

16 -1,-4

--GUACAGUACUGUGAUAACaa------------

3 -1,-4

--GUACAGUACUGUGAUAACU-------------

63242 -1,-3

--GUACAGUACUGUGAUAACUa------------

29 -1,-3

--GUACAGUACUGUGAUAACUaa-----------

44 -1,-3

--GUACAGUACUGUGAUAACUau-----------

2 -1,-3

--GUACAGUACUGUGAUAACUu------------

25 -1,-3

--GUACAGUACUGUGAUAACUG------------

4265 -1,-2

--GUACAGUACUGUGAUAACUGu-----------

130 -1,-2

--GUACAGUACUGUGAUAACUGuu----------

10 -1,-2

--GUACAGUACUGUGAUAACUGA-----------

51714 -1,-1

--GUACAGUACUGUGAUAACUGAu----------

502 -1,-1

--GUACAGUACUGUGAUAACUGAuu---------

10 -1,-1

--GUACAGUACUGUGAUAACUGAA----------

73289 -1,0

--GUACAGUACUGUGAUAACUGAAa---------

18127 -1,0

--GUACAGUACUGUGAUAACUGAAaa--------

696 -1,0

--GUACAGUACUGUGAUAACUGAAau--------

38 -1,0

--GUACAGUACUGUGAUAACUGAAu---------

261 -1,0

--GUACAGUACUGUGAUAACUGAAuu--------

6 -1,0

--GUACAGUACUGUGAUAACUGAAG---------

100 -1,1

---GUACAGUACUGUGAUAACUGAAGu--------

5 -1,1

---GUACAGUACUGUGAUAACUGAAGA--------

50 -1,2

---GUACAGUACUGUGAUAACUGAAGAA-------

2 -1,3

--UACAGUACUGUGAUAACU-------------

75423 0,-3

--UACAGUACUGUGAUAACUa------------

18 0,-3

--UACAGUACUGUGAUAACUaa-----------

66 0,-3

--UACAGUACUGUGAUAACUau-----------

9 0,-3

--UACAGUACUGUGAUAACUu------------

36 0,-3

--UACAGUACUGUGAUAACUG------------

6904 0,-2

--UACAGUACUGUGAUAACUGu-----------

121 0,-2

--UACAGUACUGUGAUAACUGuu----------

5 0,-2

--UACAGUACUGUGAUAACUGA-----------

93514 0,-1

--UACAGUACUGUGAUAACUGAu----------

564 0,-1

--UACAGUACUGUGAUAACUGAuu---------

448 0,-1

--UACAGUACUGUGAUAACUGAA----------

192208 0,0

--UACAGUACUGUGAUAACUGAAa---------

53831 0,0

--UACAGUACUGUGAUAACUGAAaa--------

1949 0,0

--UACAGUACUGUGAUAACUGAAau--------

226 0,0

--UACAGUACUGUGAUAACUGAAu---------

8877 0,0

---UACAGUACUGUGAUAACUGAAuu--------

318 0,0

---UACAGUACUGUGAUAACUGAAG---------

14629 0,1

---UACAGUACUGUGAUAACUGAAGu--------

2019 0,1

---UACAGUACUGUGAUAACUGAAGuu-------

42 0,1

---UACAGUACUGUGAUAACUGAAGA--------

3522 0,2

---UACAGUACUGUGAUAACUGAAGAu-------

19 0,2

---UACAGUACUGUGAUAACUGAAGAA-------

207 0,3

---UACAGUACUGUGAUAACUGAAGAAa------

41 0,3

---UACAGUACUGUGAUAACUGAAGAAU------

3 0,4

--ACAGUACUGUGAUAACUG------------

54 1,-2

--ACAGUACUGUGAUAACUGA-----------

527 1,-1

--ACAGUACUGUGAUAACUGAu----------

2 1,-1

--ACAGUACUGUGAUAACUGAuu---------

8 1,-1

--ACAGUACUGUGAUAACUGAA----------

1334 1,0

--ACAGUACUGUGAUAACUGAAa---------

516 1,0

--ACAGUACUGUGAUAACUGAAaa--------

39 1,0

--ACAGUACUGUGAUAACUGAAau--------

23 1,0

--ACAGUACUGUGAUAACUGAAu---------

127 1,0

--ACAGUACUGUGAUAACUGAAuu--------

8 1,0

--ACAGUACUGUGAUAACUGAAG---------

169 1,1

--ACAGUACUGUGAUAACUGAAGu--------

48 1,1

--ACAGUACUGUGAUAACUGAAGA--------

56 1,2

--ACAGUACUGUGAUAACUGAAGAA-------

4 1,3

--ACAGUACUGUGAUAACUGAAGAAa------

5 1,3

---CAGUACUGUGAUAACUGA-----------

57 2,-1

---CAGUACUGUGAUAACUGAA----------

203 2,0

---CAGUACUGUGAUAACUGAAa---------

54 2,0

---CAGUACUGUGAUAACUGAAaa--------

9 2,0

---CAGUACUGUGAUAACUGAAau--------

6 2,0

---CAGUACUGUGAUAACUGAAu---------

12 2,0

---CAGUACUGUGAUAACUGAAuu--------

6 2,0

---CAGUACUGUGAUAACUGAAG---------

28 2,1

---CAGUACUGUGAUAACUGAAGu--------

30 2,1

---CAGUACUGUGAUAACUGAAGuu-------

4 2,1

---CAGUACUGUGAUAACUGAAGA--------

57 2,2

---CAGUACUGUGAUAACUGAAGAu-------

4 2,2

---CAGUACUGUGAUAACUGAAGAA-------

37 2,3

---CAGUACUGUGAUAACUGAAGAAa------

45	2,3
--CAGUACUGUGAUAACUGAAGAAU------
8	2,4

>hsa-mir-103-1 3p:48-70	SRX018958
uacugcccucggcuucuuuacagugcugccuuguugcauauggaucaAGCAGCAUUGUACAGGGCUAUGAaggcauug
...((((.((((((.((.(((((((((((.(((.(.(....).).))))))))))))))).))))))..)).))))...
----------GGCUUCUUUACAGUGCUGCCUUG---
3
--AAGCAGCAUUGUACAGGGCUAUau--------
2	-1,-2
---AGCAGCAUUGUACAGGGC-------------
4252	0,-5
---AGCAGCAUUGUACAGGGCa------------
2	0,-5
---AGCAGCAUUGUACAGGGCaa-----------
4	0,-5
---AGCAGCAUUGUACAGGGCau-----------
6	0,-5
---AGCAGCAUUGUACAGGGCU------------
1844	0,-4
---AGCAGCAUUGUACAGGGCUu-----------
27	0,-4
---AGCAGCAUUGUACAGGGCUuu----------
22	0,-4
---AGCAGCAUUGUACAGGGCUA-----------
2674	0,-3
---AGCAGCAUUGUACAGGGCUAa----------
206	0,-3
---AGCAGCAUUGUACAGGGCUAaa---------
39	0,-3
---AGCAGCAUUGUACAGGGCUAau---------
56	0,-3
---AGCAGCAUUGUACAGGGCUAU----------
14288	0,-2
---AGCAGCAUUGUACAGGGCUAUa---------
5914	0,-2
---AGCAGCAUUGUACAGGGCUAUaa--------

429 0,-2

---AGCAGCAUUGUACAGGGCUAUau--------

452 0,-2

---AGCAGCAUUGUACAGGGCUAUu---------

1891 0,-2

---AGCAGCAUUGUACAGGGCUAUuu--------

673 0,-2

---AGCAGCAUUGUACAGGGCUAUG---------

10728 0,-1

---AGCAGCAUUGUACAGGGCUAUGu--------

3060 0,-1

---AGCAGCAUUGUACAGGGCUAUGuu-------

344 0,-1

---AGCAGCAUUGUACAGGGCUAUGA--------

13902 0,0

---AGCAGCAUUGUACAGGGCUAUGAu-------

917 0,0

---AGCAGCAUUGUACAGGGCUAUGAuu------

32 0,0

---AGCAGCAUUGUACAGGGCUAUGAA-------

291 0,1

---AGCAGCAUUGUACAGGGCUAUGAAu------

8 0,1

---AGCAGCAUUGUACAGGGCUAUGAAG------

2 0,2

--GCAGCAUUGUACAGGGCU------------

6 1,-4

--GCAGCAUUGUACAGGGCUA-----------

9 1,-3

--GCAGCAUUGUACAGGGCUAU----------

52 1,-2

--GCAGCAUUGUACAGGGCUAUa---------

3 1,-2

--GCAGCAUUGUACAGGGCUAUu---------

3 1,-2

--GCAGCAUUGUACAGGGCUAUG---------

27 1,-1

--GCAGCAUUGUACAGGGCUAUGu--------

--GCAGCAUUGUACAGGGCUAUGA--------
11 1,-1
--GCAGCAUUGUACAGGGCUAUGAu-------
94 1,0
--GCAGCAUUGUACAGGGCUAUGAA-------
2 1,0
---CAGCAUUGUACAGGGCUA-----------
4 1,1
---CAGCAUUGUACAGGGCUAU----------
3 2,-3
---CAGCAUUGUACAGGGCUAUa---------
9 2,-2
---CAGCAUUGUACAGGGCUAUG---------
2 2,-2
---CAGCAUUGUACAGGGCUAUGu--------
20 2,-1
---CAGCAUUGUACAGGGCUAUGA--------
3 2,-1
---CAGCAUUGUACAGGGCUAUGAu-------
38 2,0
2 2,0

>hsa-mir-103-2 mi:11-33;MA:48-70 SRX018958
uugugcuuucAGCUUCUUUACAGUGCUGCCUUGuagcauucaggucaAGCAGCAUUGUACAGGGCUAUGAaagaacca
..((.(((((((((.((.(((((((((((((((.......))))....))))))))))).)))))..)))))).))..
----------AGCUUCUUUACAGUGCUGCCUUG---
3 0,0
----------AGCUUCUUUACAGUGCUGCCUUGU--
5 0,1
--AAGCAGCAUUGUACAGGGCUAUau--------
2 -1,-2
---AGCAGCAUUGUACAGGGC-------------
4252 0,-5
---AGCAGCAUUGUACAGGGCa------------
2 0,-5
---AGCAGCAUUGUACAGGGCaa-----------
4 0,-5
---AGCAGCAUUGUACAGGGCau-----------

6 0,-5

---AGCAGCAUUGUACAGGGCU------------

1844 0,-4

---AGCAGCAUUGUACAGGGCUu-----------

27 0,-4

---AGCAGCAUUGUACAGGGCUuu----------

22 0,-4

---AGCAGCAUUGUACAGGGCUA-----------

2674 0,-3

---AGCAGCAUUGUACAGGGCUAa----------

206 0,-3

---AGCAGCAUUGUACAGGGCUAaa---------

39 0,-3

---AGCAGCAUUGUACAGGGCUAau---------

56 0,-3

---AGCAGCAUUGUACAGGGCUAU----------

14288 0,-2

---AGCAGCAUUGUACAGGGCUAUa---------

5914 0,-2

---AGCAGCAUUGUACAGGGCUAUaa--------

429 0,-2

---AGCAGCAUUGUACAGGGCUAUau--------

452 0,-2

---AGCAGCAUUGUACAGGGCUAUu---------

1891 0,-2

---AGCAGCAUUGUACAGGGCUAUuu--------

673 0,-2

---AGCAGCAUUGUACAGGGCUAUG---------

10728 0,-1

---AGCAGCAUUGUACAGGGCUAUGu--------

3060 0,-1

---AGCAGCAUUGUACAGGGCUAUGuu-------

344 0,-1

---AGCAGCAUUGUACAGGGCUAUGA--------

13902 0,0

---AGCAGCAUUGUACAGGGCUAUGAu-------

917 0,0

---AGCAGCAUUGUACAGGGCUAUGAuu------

32 0,0
--AGCAGCAUUGUACAGGGCUAUGAA-------
291 0,1
--AGCAGCAUUGUACAGGGCUAUGAAu------
8 0,1
--AGCAGCAUUGUACAGGGCUAUGAAA------
33 0,2
---GCAGCAUUGUACAGGGCU------------
6 1,-4
---GCAGCAUUGUACAGGGCUA-----------
9 1,-3
---GCAGCAUUGUACAGGGCUAU----------
52 1,-2
---GCAGCAUUGUACAGGGCUAUa---------
3 1,-2
---GCAGCAUUGUACAGGGCUAUu---------
3 1,-2
---GCAGCAUUGUACAGGGCUAUG---------
27 1,-1
---GCAGCAUUGUACAGGGCUAUGu--------
11 1,-1
---GCAGCAUUGUACAGGGCUAUGA--------
94 1,0
---GCAGCAUUGUACAGGGCUAUGAu-------
2 1,0
---GCAGCAUUGUACAGGGCUAUGAA-------
4 1,1
--CAGCAUUGUACAGGGCUA-----------
3 2,-3
--CAGCAUUGUACAGGGCUAU----------
9 2,-2
--CAGCAUUGUACAGGGCUAUa---------
2 2,-2
--CAGCAUUGUACAGGGCUAUG---------
20 2,-1
--CAGCAUUGUACAGGGCUAUGu--------
3 2,-1
--CAGCAUUGUACAGGGCUAUGA--------

38 2,0
--CAGCAUUGUACAGGGCUAUGAu-------
2 2,0

>hsa-mir-106a MA:13-35;mi:50-71 SRX018958
ccuuggccauguAAAAGUGCUUACAGUGCAGGUAGcuuuuugagaucuaCUGCAAUGUAAGCACUUCUUACauuaccaug
g
((.(((..(((((((((((((((((.(((((.(((.((.....)).)))))))).))))))))))).))))))..))).)
)
------------AAAAGUGCUUACAGUGCAG---
- 4 0,-4
------------AAAAGUGCUUACAGUGCAGGU---
- 7 0,-2
-------------AAAGUGCUUACAGUGCAGG--
- 8 1,-3
-------------AAAGUGCUUACAGUGCAGGU---
- 8 1,-2
-------------AAAGUGCUUACAGUGCAGGUA--
- 7 1,-1
-------------AAAGUGCUUACAGUGCAGGUAa---
- 6 1,-1
-------------AAAGUGCUUACAGUGCAGGUAu---
- 2 1,-1
-------------AAAGUGCUUACAGUGCAGGUAG---
- 2 1,0
-------------AAAGUGCUUACAGUGCAGGUAGa--
- 3 1,0

>hsa-mir-106b MA:12-32;mi:52-73 SRX018958
ccugccggggcUAAAGUGCUGACAGUGCAGAUagugguccucuccgugcuaCCGCACUGUGGGUACUUGCUGCuccagca
gg
(((((.(((((..(((((((.(((((((.(.((((((......))..))))).))))))).)))))))...))))).)))
))
-----------UAAAGUGCUGACAGUGCA---
-- 11 0,-3
-----------UAAAGUGCUGACAGUGCAG--
-- 47 0,-2
-----------UAAAGUGCUGACAGUGCAGu---

```
--  2    0,-2
-----------UAAAGUGCUGACAGUGCAGA-------------------------------------------------
--  25   0,-1
-----------UAAAGUGCUGACAGUGCAGAaa-----------------------------------------------
--  8    0,-1
-----------UAAAGUGCUGACAGUGCAGAU------------------------------------------------
--  49   0,0
-----------UAAAGUGCUGACAGUGCAGAUu-----------------------------------------------
--  11   0,0
-----------UAAAGUGCUGACAGUGCAGAUuu----------------------------------------------
--  2    0,0
-----------UAAAGUGCUGACAGUGCAGAUA-----------------------------------------------
--  102  0,1
-----------UAAAGUGCUGACAGUGCAGAUAa----------------------------------------------
--  55   0,1
-----------UAAAGUGCUGACAGUGCAGAUAaa---------------------------------------------
--  2    0,1
-----------UAAAGUGCUGACAGUGCAGAUAu----------------------------------------------
--  25   0,1
-----------UAAAGUGCUGACAGUGCAGAUAuu---------------------------------------------
--  3    0,1
-----------UAAAGUGCUGACAGUGCAGAUAG----------------------------------------------
--  2    0,2
-----------UAAAGUGCUGACAGUGCAGAUAGU---------------------------------------------
--  3    0,3
-------------------------------------------------UACCGCACUGUGGGUACUUGu----------
--  2    -2,-4
-------------------------------------------------UACCGCACUGUGGGUACUUGC----------
--  3    -2,-3
-------------------------------------------------UACCGCACUGUGGGUACUUGCU---------
--  31   -2,-2
--------------------------------------------------ACCGCACUGUGGGUACUUGCU---------
--  2    -1,-2
---------------------------------------------------CCGCACUGUGGGUACUUGC----------
--  2    0,-3
---------------------------------------------------CCGCACUGUGGGUACUUGCU---------
--  13   0,-2
---------------------------------------------------CCGCACUGUGGGUACUUGCUG--------
```

---CCGCACUGUGGGUACUUGCUGa-------
-- 55 0,-1
---CCGCACUGUGGGUACUUGCUGa-------
-- 30 0,-1
---CCGCACUGUGGGUACUUGCUGaa------
-- 9 0,-1
---CCGCACUGUGGGUACUUGCUGu-------
-- 12 0,-1
---CCGCACUGUGGGUACUUGCUGC-------
-- 87 0,0
---CCGCACUGUGGGUACUUGCUGCau-----
-- 5 0,0

>hsa-mir-107 3p:50-72 SRX018958
cucucugcuuucagcuucuuuacaguguugccuuguggcauggaguucaAGCAGCAUUGUACAGGGCUAUCAaagcacag
a
.(((.((((((.((((.((.(((((((((((.(((.(((.....))))))))))))))))).))))))...)))))).))
)
--AAGCAGCAUUGUACAGGGCUAUau--------
- 2 -1,-2
---AGCAGCAUUGUACAGGGC-------------
- 4252 0,-5
---AGCAGCAUUGUACAGGGCa------------
- 2 0,-5
---AGCAGCAUUGUACAGGGCaa-----------
- 4 0,-5
---AGCAGCAUUGUACAGGGCau-----------
- 6 0,-5
---AGCAGCAUUGUACAGGGCU------------
- 1844 0,-4
---AGCAGCAUUGUACAGGGCUu-----------
- 27 0,-4
---AGCAGCAUUGUACAGGGCUuu----------
- 22 0,-4
---AGCAGCAUUGUACAGGGCUA-----------
- 2674 0,-3
---AGCAGCAUUGUACAGGGCUAa----------
- 206 0,-3
---AGCAGCAUUGUACAGGGCUAaa---------

- 39 0,-3
---AGCAGCAUUGUACAGGGCUAau---------
- 56 0,-3
---AGCAGCAUUGUACAGGGCUAU----------
- 14288 0,-2
---AGCAGCAUUGUACAGGGCUAUa---------
- 5914 0,-2
---AGCAGCAUUGUACAGGGCUAUaa--------
- 429 0,-2
---AGCAGCAUUGUACAGGGCUAUau--------
- 452 0,-2
---AGCAGCAUUGUACAGGGCUAUu---------
- 1891 0,-2
---AGCAGCAUUGUACAGGGCUAUuu--------
- 673 0,-2
---AGCAGCAUUGUACAGGGCUAUC---------
- 1913 0,-1
---AGCAGCAUUGUACAGGGCUAUCu--------
- 1407 0,-1
---AGCAGCAUUGUACAGGGCUAUCuu-------
- 31 0,-1
---AGCAGCAUUGUACAGGGCUAUCA--------
- 4615 0,0
---AGCAGCAUUGUACAGGGCUAUCAu-------
- 148 0,0
---AGCAGCAUUGUACAGGGCUAUCAuu------
- 5 0,0
---AGCAGCAUUGUACAGGGCUAUCAA-------
- 37 0,1
--GCAGCAUUGUACAGGGCU------------
- 6 1,-4
--GCAGCAUUGUACAGGGCUA-----------
- 9 1,-3
--GCAGCAUUGUACAGGGCUAU----------
- 52 1,-2
--GCAGCAUUGUACAGGGCUAUa---------
- 3 1,-2
--GCAGCAUUGUACAGGGCUAUu---------

- 3 1,-2
--GCAGCAUUGUACAGGGCUAUCuu-------
- 4 1,-1
---CAGCAUUGUACAGGGCUA-----------
- 3 2,-3
---CAGCAUUGUACAGGGCUAU----------
- 9 2,-2
---CAGCAUUGUACAGGGCUAUa---------
- 2 2,-2
---CAGCAUUGUACAGGGCUAUC---------
- 3 2,-1

>hsa-mir-10a MA:22-44;mi:63-84 SRX018958
gaucugucugucuucuguauaUACCCUGUAGAUCCGAAUUUGUGuaaggaauuuuguggucaCAAAUUCGUAUCUAGGGG
AAUAuguaguugacauaaacacuccgcucu
....(((.((((..((((((((.((((.(((((.((((((((((................)))))))))).)))))))))
.))))))))..))))...))).........
--------------------AUACCCUGUAGAUCCGAAUUUG--------------------------------------
------------------------------ 4 -1,-2
--------------------AUACCCUGUAGAUCCGAAUUUGU-------------------------------------
------------------------------ 21 -1,-1
---------------------UACCCUGUAGAUCCGAAU---
------------------------------ 74 0,-5
---------------------UACCCUGUAGAUCCGAAUa--
------------------------------ 71 0,-5
---------------------UACCCUGUAGAUCCGAAUaa---------------------------------------
------------------------------ 6 0,-5
---------------------UACCCUGUAGAUCCGAAUU--
------------------------------ 479 0,-4
---------------------UACCCUGUAGAUCCGAAUUaa--------------------------------------
------------------------------ 4 0,-4
---------------------UACCCUGUAGAUCCGAAUUU---------------------------------------
------------------------------ 281 0,-3
---------------------UACCCUGUAGAUCCGAAUUUuu-------------------------------------
------------------------------ 2 0,-3
---------------------UACCCUGUAGAUCCGAAUUUG--------------------------------------
------------------------------ 1653 0,-2
---------------------UACCCUGUAGAUCCGAAUUUGa-------------------------------------

----------------------------- 32   0,-2
--------------------UACCCUGUAGAUCCGAAUUUGaa-----------------------------------
----------------------------- 4   0,-2
--------------------UACCCUGUAGAUCCGAAUUUGU------------------------------------
----------------------------- 3978   0,-1
--------------------UACCCUGUAGAUCCGAAUUUGUa-----------------------------------
----------------------------- 15   0,-1
--------------------UACCCUGUAGAUCCGAAUUUGUu-----------------------------------
----------------------------- 6   0,-1
--------------------UACCCUGUAGAUCCGAAUUUGUG-----------------------------------
----------------------------- 363 0,0
--------------------UACCCUGUAGAUCCGAAUUUGUGa----------------------------------
----------------------------- 144 0,0
--------------------UACCCUGUAGAUCCGAAUUUGUGaa---------------------------------
----------------------------- 2   0,0
--------------------UACCCUGUAGAUCCGAAUUUGUGU---------------------------------
----------------------------- 34   0,1
---------------------ACCCUGUAGAUCCGAAUU---------------------------------------
----------------------------- 4   1,-4
---------------------ACCCUGUAGAUCCGAAUUaa-------------------------------------
----------------------------- 2   1,-4
---------------------ACCCUGUAGAUCCGAAUUU--------------------------------------
----------------------------- 16   1,-3
---------------------ACCCUGUAGAUCCGAAUUUG-------------------------------------
----------------------------- 28   1,-2
---------------------ACCCUGUAGAUCCGAAUUUGa------------------------------------
----------------------------- 3   1,-2
---------------------ACCCUGUAGAUCCGAAUUUGaa-----------------------------------
----------------------------- 3   1,-2
---------------------ACCCUGUAGAUCCGAAUUUGU------------------------------------
----------------------------- 413 1,-1
---------------------ACCCUGUAGAUCCGAAUUUGUaa----------------------------------
----------------------------- 5   1,-1
---------------------ACCCUGUAGAUCCGAAUUUGUG-----------------------------------
----------------------------- 265 1,0
---------------------ACCCUGUAGAUCCGAAUUUGUGa----------------------------------
----------------------------- 478 1,0
---------------------ACCCUGUAGAUCCGAAUUUGUGaa---------------------------------

------------------------------ 49 1,0
----------------------ACCCUGUAGAUCCGAAUUUGUGau----------------------------------
------------------------------ 3   1,0
----------------------ACCCUGUAGAUCCGAAUUUGUGU-----------------------------------
------------------------------ 127 1,1
----------------------ACCCUGUAGAUCCGAAUUUGUGUA----------------------------------
------------------------------ 5   1,2
-----------------------CCCUGUAGAUCCGAAUUUGU-------------------------------------
------------------------------ 6   2,-1
-----------------------CCCUGUAGAUCCGAAUUUGUGa-----------------------------------
------------------------------ 3   2,0
--CAAAUUCGUAUCUAGGGG
------------------------------ 3   0,-4
--CAAAUUCGUAUCUAGGGG
A----------------------------- 4   0,-3
--CAAAUUCGUAUCUAGGGG
AAUAa------------------------- 2   0,0
--CAAAUUCGUAUCUAGGGG
AAUAU------------------------- 4   0,1
---AAAUUCGUAUCUAGGGG
A----------------------------- 2   1,-3
---AAAUUCGUAUCUAGGGG
AAU--------------------------- 2   1,-1
---AAAUUCGUAUCUAGGGG
AAUu-------------------------- 3   1,-1
---AAAUUCGUAUCUAGGGG
AAUA-------------------------- 2   1,0
---AAAUUCGUAUCUAGGGG
AAUAa------------------------- 7   1,0
---AAAUUCGUAUCUAGGGG
AAUAU------------------------- 2   1,1
--AAUUCGUAUCUAGGGG
AAUAa------------------------- 2   2,0

>hsa-mir-10b   MA:27-49;mi:66-87   SRX018958
ccagagguuguaacguugucuauauaUACCCUGUAGAACCGAAUUUGUGugguauccguauagucACAGAUUCGAUUCUA
GGGGAAUauauggucgaugcaaaaacuuca
...(((((....((((.((((((((.((((.(((((.((((((((((..(((....)))...)))))))))).)))))

)))).)))))))).))))).....)))))).

--------------------------UACCCUGUAGAACCGAAU------------------------------------
------------------------------ 2   0,-5
--------------------------UACCCUGUAGAACCGAAUUU----------------------------------
------------------------------ 2   0,-3
--------------------------UACCCUGUAGAACCGAAUUUG---------------------------------
------------------------------ 26   0,-2
--------------------------UACCCUGUAGAACCGAAUUUGa--------------------------------
------------------------------ 5   0,-2
--------------------------UACCCUGUAGAACCGAAUUUGU--------------------------------
------------------------------ 570 0,-1
--------------------------UACCCUGUAGAACCGAAUUUGUa-------------------------------
------------------------------ 2   0,-1
--------------------------UACCCUGUAGAACCGAAUUUGUG-------------------------------
------------------------------ 36   0,0
--------------------------UACCCUGUAGAACCGAAUUUGUGa------------------------------
------------------------------ 26   0,0
--------------------------UACCCUGUAGAACCGAAUUUGUGU------------------------------
------------------------------ 15   0,1
---------------------------ACCCUGUAGAACCGAAUUUG---------------------------------
------------------------------ 3   1,-2
---------------------------ACCCUGUAGAACCGAAUUUGU--------------------------------
------------------------------ 19   1,-1
---------------------------ACCCUGUAGAACCGAAUUUGUG-------------------------------
------------------------------ 11   1,0
---------------------------ACCCUGUAGAACCGAAUUUGUGa------------------------------
------------------------------ 19   1,0
---------------------------ACCCUGUAGAACCGAAUUUGUGaa-----------------------------
------------------------------ 3   1,0
---------------------------ACCCUGUAGAACCGAAUUUGUGU------------------------------
------------------------------ 21   1,1
---AGAUUCGAUUCUA
GGGGAAUA---------------------- 2   2,1

>hsa-mir-1181   5p:14-34   SRX018958

uccacugcugccgCCGUCGCCGCCACCCGAGCCGgagcgggcugggccgccaaggcaagaugguggacuacagcgugugg
g

.((((((((((((((((((((..(((.((((......)))))))..))...)))..))))))))....))))).))))

.
--AGGCAAGAUGGUGGACUA-----------
-    2

>hsa-mir-1185-1 5p:15-35   SRX018958
uuugguacuugaagAGAGGAUACCCUUUGUAUGUUcacuugauuaauggcgaauauacagggggagacucuuauuugcgu
aucaaa
.((((((.(((((((((..(.((((((((((((((.((........)).)))))))))))))))..))))).)))).))
))))).
--------------AGAGGAUACCCUUUGUAUGU--
------ 5   0,-1
--------------AGAGGAUACCCUUUGUAUGUU---
------ 23 0,0
--------------AGAGGAUACCCUUUGUAUGUUa--
------ 7   0,0
--------------AGAGGAUACCCUUUGUAUGUUC--
------ 24 0,1
--------------AGAGGAUACCCUUUGUAUGUUCA---
------ 5   0,2
--AUAUACAGGGGGAGACUCUUA-------
------ 5
--AUAUACAGGGGGAGACUCUUAa------
------ 2
--AUAUACAGGGGGAGACUCUUAU------
------ 6

>hsa-mir-1185-2 5p:15-35   SRX018958
uuugguacuuaaagAGAGGAUACCCUUUGUAUGUUcacuugauuaauggcgaauauacagggggagacucucauuugcgu
aucaaa
.((((((.(((((((((..(.((((((((((((((.((........)).)))))))))))))))..))))).)))).))
))))).
--------------AGAGGAUACCCUUUGUAUGU--
------ 5   0,-1
--------------AGAGGAUACCCUUUGUAUGUU---
------ 23 0,0
--------------AGAGGAUACCCUUUGUAUGUUa--
------ 7   0,0
--------------AGAGGAUACCCUUUGUAUGUUC--

------ 24 0,1
--------------AGAGGAUACCCUUUGUAUGUUCA---
------ 5   0,2

>hsa-mir-1193   5p:10-32   SRX018958
guagcugagGGGAUGGUAGACCGGUGACGUGCacuucauuuacgauguaggucacccguuugacuauccaccagcgcc
...((((.(.(((((((((((.((((((.((((..((......)))))).)))))).)))).))))))).)))))...
---------GGGAUGGUAGACCGGUGACGUGCAu--
    4   0,1

>hsa-mir-122   MA:15-36;mi:51-72   SRX018958
ccuuagcagagcugUGGAGUGUGACAAUGGUGUUUGugucuaaacuaucaAACGCCAUUAUCACACUAAAUAgcuacugc
uaggc
.((((((((((((((..(((((((.(((((((((((.((....))...))))))))))).)))))))..)))))).))))
)))).
------------UGUGGAGUGUGACAAUGGUG--
-----   2   -2,-4
------------UGUGGAGUGUGACAAUGGUGU---
-----   17 -2,-3
------------UGUGGAGUGUGACAAUGGUGUU--
-----   88 -2,-2
------------UGUGGAGUGUGACAAUGGUGUUa---
-----   10 -2,-2
------------UGUGGAGUGUGACAAUGGUGUUU---
-----   12 -2,-1
------------UGUGGAGUGUGACAAUGGUGUUUGU---
-----   2   -2,1
-------------GUGGAGUGUGACAAUGGUG--
-----   3   -1,-4
-------------GUGGAGUGUGACAAUGGUGU---
-----   17 -1,-3
-------------GUGGAGUGUGACAAUGGUGUU--
-----   47 -1,-2
-------------GUGGAGUGUGACAAUGGUGUUU---
-----   200 -1,-1
-------------GUGGAGUGUGACAAUGGUGUUUG--
-----   9   -1,0
-------------GUGGAGUGUGACAAUGGUGUUUGa---

----- 9 -1,0

-------------GUGGAGUGUGACAAUGGUGUUUGU--

----- 9 -1,1

--------------UGGAGUGUGACAAUGGUG---

----- 13832 0,-4

--------------UGGAGUGUGACAAUGGUGa--

----- 466 0,-4

--------------UGGAGUGUGACAAUGGUGaa---

----- 309 0,-4

--------------UGGAGUGUGACAAUGGUGau---

----- 28 0,-4

--------------UGGAGUGUGACAAUGGUGU--

----- 48610 0,-3

--------------UGGAGUGUGACAAUGGUGUa---

----- 1625 0,-3

--------------UGGAGUGUGACAAUGGUGUaa--

----- 4243 0,-3

--------------UGGAGUGUGACAAUGGUGUau--

----- 513 0,-3

--------------UGGAGUGUGACAAUGGUGUU---

----- 104956 0,-2

--------------UGGAGUGUGACAAUGGUGUUa--

----- 11270 0,-2

--------------UGGAGUGUGACAAUGGUGUUaa---

----- 5383 0,-2

--------------UGGAGUGUGACAAUGGUGUUau---

----- 1007 0,-2

--------------UGGAGUGUGACAAUGGUGUUU--

----- 601368 0,-1

--------------UGGAGUGUGACAAUGGUGUUUa---

----- 43157 0,-1

--------------UGGAGUGUGACAAUGGUGUUUaa--

----- 40792 0,-1

--------------UGGAGUGUGACAAUGGUGUUUau--

----- 20396 0,-1

--------------UGGAGUGUGACAAUGGUGUUUu---

----- 19389 0,-1

--------------UGGAGUGUGACAAUGGUGUUUuu--

----- 2518 0,-1
--------------UGGAGUGUGACAAUGGUGUUUG--
----- 1354051 0,0
--------------UGGAGUGUGACAAUGGUGUUUGa---
----- 1589016 0,0
--------------UGGAGUGUGACAAUGGUGUUUGaa--
----- 32292 0,0
--------------UGGAGUGUGACAAUGGUGUUUGau--
----- 16436 0,0
--------------UGGAGUGUGACAAUGGUGUUUGU---
----- 1001990 0,1
--------------UGGAGUGUGACAAUGGUGUUUGUa--
----- 3352 0,1
--------------UGGAGUGUGACAAUGGUGUUUGUaa---
----- 2754 0,1
--------------UGGAGUGUGACAAUGGUGUUUGUau---
----- 1822 0,1
--------------UGGAGUGUGACAAUGGUGUUUGUu--
----- 3463 0,1
--------------UGGAGUGUGACAAUGGUGUUUGUuu---
----- 942 0,1
--------------UGGAGUGUGACAAUGGUGUUUGUG--
----- 326 0,2
--------------UGGAGUGUGACAAUGGUGUUUGUGa---
----- 68 0,2
--------------UGGAGUGUGACAAUGGUGUUUGUGU---
----- 145 0,3
---------------GGAGUGUGACAAUGGUGU---
----- 646 1,-3
---------------GGAGUGUGACAAUGGUGUa--
----- 13 1,-3
---------------GGAGUGUGACAAUGGUGUaa---
----- 64 1,-3
---------------GGAGUGUGACAAUGGUGUau---
----- 6 1,-3
---------------GGAGUGUGACAAUGGUGUU--
----- 1419 1,-2
---------------GGAGUGUGACAAUGGUGUUa---

----- 130 1,-2
---------------GGAGUGUGACAAUGGUGUUaa--
----- 93 1,-2
---------------GGAGUGUGACAAUGGUGUUau--
----- 19 1,-2
---------------GGAGUGUGACAAUGGUGUUU---
----- 5090 1,-1
---------------GGAGUGUGACAAUGGUGUUUa--
----- 618 1,-1
---------------GGAGUGUGACAAUGGUGUUUaa---
----- 689 1,-1
---------------GGAGUGUGACAAUGGUGUUUau---
----- 246 1,-1
---------------GGAGUGUGACAAUGGUGUUUu--
----- 153 1,-1
---------------GGAGUGUGACAAUGGUGUUUuu---
----- 16 1,-1
---------------GGAGUGUGACAAUGGUGUUUG--
----- 22662 1,0
---------------GGAGUGUGACAAUGGUGUUUGa---
----- 24016 1,0
---------------GGAGUGUGACAAUGGUGUUUGaa--
----- 631 1,0
---------------GGAGUGUGACAAUGGUGUUUGau--
----- 331 1,0
---------------GGAGUGUGACAAUGGUGUUUGU---
----- 9001 1,1
---------------GGAGUGUGACAAUGGUGUUUGUa--
----- 75 1,1
---------------GGAGUGUGACAAUGGUGUUUGUaa---
----- 50 1,1
---------------GGAGUGUGACAAUGGUGUUUGUau---
----- 62 1,1
---------------GGAGUGUGACAAUGGUGUUUGUu--
----- 84 1,1
---------------GGAGUGUGACAAUGGUGUUUGUuu---
----- 29 1,1
---------------GGAGUGUGACAAUGGUGUUUGUG--

----- 17 1,2

---------------GGAGUGUGACAAUGGUGUUUGUGa---

----- 2 1,2

---------------GGAGUGUGACAAUGGUGUUUGUGU---

----- 7 1,3

----------------GAGUGUGACAAUGGUGUU--

----- 147 2,-2

----------------GAGUGUGACAAUGGUGUUU---

----- 105 2,-1

----------------GAGUGUGACAAUGGUGUUUa--

----- 8 2,-1

----------------GAGUGUGACAAUGGUGUUUaa---

----- 15 2,-1

----------------GAGUGUGACAAUGGUGUUUau---

----- 2 2,-1

----------------GAGUGUGACAAUGGUGUUUu--

----- 3 2,-1

----------------GAGUGUGACAAUGGUGUUUuu---

----- 2 2,-1

----------------GAGUGUGACAAUGGUGUUUG--

----- 106 2,0

----------------GAGUGUGACAAUGGUGUUUGa---

----- 133 2,0

----------------GAGUGUGACAAUGGUGUUUGaa--

----- 8 2,0

----------------GAGUGUGACAAUGGUGUUUGU---

----- 106 2,1

----------------GAGUGUGACAAUGGUGUUUGUGa---

----- 2 2,2

--CAAACGCCAUUAUCACACUAA-----------

----- 3 -2,-3

---AAACGCCAUUAUCACACUA------------

----- 30 -1,-4

---AAACGCCAUUAUCACACUAu-----------

----- 11 -1,-4

---AAACGCCAUUAUCACACUAuu----------

----- 17 -1,-4

---AAACGCCAUUAUCACACUAA-----------

----- 100 -1,-3
--AAACGCCAUUAUCACACUAAu----------
----- 25 -1,-3
--AAACGCCAUUAUCACACUAAuu---------
----- 16 -1,-3
--AAACGCCAUUAUCACACUAAA----------
----- 82 -1,-2
--AAACGCCAUUAUCACACUAAAa---------
----- 11 -1,-2
--AAACGCCAUUAUCACACUAAAaa--------
----- 6 -1,-2
--AAACGCCAUUAUCACACUAAAU---------
----- 410 -1,-1
--AAACGCCAUUAUCACACUAAAUu--------
----- 33 -1,-1
--AAACGCCAUUAUCACACUAAAUA--------
----- 12 -1,0
--AAACGCCAUUAUCACACUAAAUAa-------
----- 3 -1,0
---AACGCCAUUAUCACACUA------------
----- 67 0,-4
---AACGCCAUUAUCACACUAu-----------
----- 14 0,-4
---AACGCCAUUAUCACACUAuu----------
----- 2 0,-4
---AACGCCAUUAUCACACUAA-----------
----- 305 0,-3
---AACGCCAUUAUCACACUAAu----------
----- 37 0,-3
---AACGCCAUUAUCACACUAAuu---------
----- 59 0,-3
---AACGCCAUUAUCACACUAAA----------
----- 314 0,-2
---AACGCCAUUAUCACACUAAAa---------
----- 64 0,-2
---AACGCCAUUAUCACACUAAAaa--------
----- 7 0,-2
---AACGCCAUUAUCACACUAAAau--------

-----	2	0,-2
---AACGCCAUUAUCACACUAAAU---------
-----	3975	0,-1
---AACGCCAUUAUCACACUAAAUu--------
-----	391	0,-1
---AACGCCAUUAUCACACUAAAUuu-------
-----	36	0,-1
---AACGCCAUUAUCACACUAAAUA--------
-----	1243	0,0
---AACGCCAUUAUCACACUAAAUAa-------
-----	259	0,0
---AACGCCAUUAUCACACUAAAUAaa------
-----	4	0,0
---AACGCCAUUAUCACACUAAAUAu-------
-----	811	0,0
---AACGCCAUUAUCACACUAAAUAuu------
-----	12	0,0
---AACGCCAUUAUCACACUAAAUAG-------
-----	5	0,1
--ACGCCAUUAUCACACUAA-----------
-----	2	1,-3
--ACGCCAUUAUCACACUAAuu---------
-----	4	1,-3
--ACGCCAUUAUCACACUAAA----------
-----	3	1,-2
--ACGCCAUUAUCACACUAAAU---------
-----	14	1,-1
--ACGCCAUUAUCACACUAAAUu--------
-----	5	1,-1
--ACGCCAUUAUCACACUAAAUAa-------
-----	2	1,0
--ACGCCAUUAUCACACUAAAUAu-------
-----	3	1,0

>hsa-mir-1228	mi:1-21;MA:52-71	SRX018958
GUGGGCGGGGGCAGGUGUGUGgugggugguggccugcggugagcagggcccUCACACCUGCCUCGCCCCCCag
..(((((((.((((((((....((((.....(((((.....))))))))).))))))))))))))).....
GUGGGCGGGGGCAGGUGUGUG--	3

0,0
GUGGGCGGGGGCAGGUGUGUGG--- 2
0,1

>hsa-mir-124-1 mi:14-35;MA:53-72 SRX018958
aggccucucucucCGUGUUCACAGCGGACCUUGAUuuaaauguccauacaauUAAGGCACGCGGUGAAUGCCaagaaugg
ggcug
.((((((..(((..((((((((.(((..((((((((.............))))))))..))).))))))))..)))..))
)))).
--UAAGGCACGCGGUGAAUGCCAA------
----- 7 0,2

>hsa-mir-124-2 mi:25-46;MA:62-81 SRX018958
aucaagauuagaggcucugcucucCGUGUUCACAGCGGACCUUGAUuuaaugucauacaauUAAGGCACGCGGUGAAUGC
Caagagcggagccuacggcugcacuugaa
.((((..((((((((((((((..((((((((.(((..((((((((...........))))))))..))).))))))))
..)))))))))))))....)))..))))).
---UAAGGCACGCGGUGAAUGC
CAA------------------------ 7 0,2

>hsa-mir-124-3 mi:15-36;MA:53-72 SRX018958
ugagggccccucugCGUGUUCACAGCGGACCUUGAUuuaaugucuauacaauUAAGGCACGCGGUGAAUGCCaagagagg
cgccucc
.(((((((.(((((.((((((((.(((..((((((((............))))))))..))).))))))))).)))).))
).)))).
--UAAGGCACGCGGUGAAUGCCAA------
------- 7 0,2

>hsa-mir-1246 5p:11-29 SRX018958
uguauccuugAAUGGAUUUUUGGAGCAGGaguggacaccugacccaaaggaaaucaauccauaggcuagcaau
(((..(((...((((((((((((..((((.(....).))))..))))).......))))))))))...)))..
----------AAUGGAUUUUUGGAGCAGG-- 15
0,0
-----------AUGGAUUUUUGGAGCAGG-- 3
1,0

>hsa-mir-1247 5p:40-61 SRX018958
ccgcuugccucgcccagcgcagccccggccgcugggcgcACCCGUCCCGUUCGUCCCCGGAcguugcucucuaccccggg

aacgucgagacuggagcgcccgaacugagccaccuucgcggaccccgagagcggcg
.((((.((((((.((.(((.((....(((((.(((((((.((.(((.((..(((.(((((..((........)).)))))
.))).)).))).)).)))))))...)).)))..)).)))))....)))).))))))
---------------------------------------ACCCGUCCCGUUCGUCCCCGG--------------------
--   2   0,-1

>hsa-mir-1248   5p:4-30 SRX018958
uuuACCUUCUUGUAUAAGCACUGUGCUAAAauugcagacacuaggaccaugucuugguuuuugcaauaaugcuagcagag
uacacacaagaagaaaaguaacagca
.....((((((((....(.(((.(((((..((((((((.((((((((...)))))))).))))))))).....))))).))
).)..)))))))).............
---ACCUUCUUGUAUAAGCACUGUGCU---
-------------------------- 2   0,-3

>hsa-mir-1249   3p:41-62   SRX018958
gggaggagggaggagaugggccaaguucccucuggcuggaACGCCCUUCCCCCCCUUCUUCAccug
.((((((((.((.((.((((...(((((........))))))))).)).)))))))))))).....
--ACGCCCUUCCCCCCCUUCUUCA----   2   0,0

>hsa-mir-1252   5p:5-26 SRX018958
agaaAGAAGGAAAUUGAAUUCAUUUAgaaaagagaauuccaaaugagcuuaauuuccuuuuuucu
((((((((((((((((.(((((((.(((.......))).))))))).))))))))))))))))
----AGAAGGAAAUUGAAUUCAUU---   3   0,-2
----AGAAGGAAAUUGAAUUCAUUU--   3   0,-1

>hsa-mir-1254   5p:18-41   SRX018958
ggugggaggauugcuugAGCCUGGAAGCUGGAGCCUGCAGUgaacuaucauugugccacuguacuccagccuaggcaaca
aaaugaaauccugucua
...((.((((((..(((.((((((..(((((((..(((((((.((.......))..))))))))))))))))))))..))
).....)))))).))..
-----------------AGCCUGGAAGCUGGAGCCUGCAGU---------------------------------------
-----------------   4   0,0

>hsa-mir-1255a   5p:28-50   SRX018958
auuggaaauccuuugaguugcuucucaAGGAUGAGCAAAGAAAGUAGAUUuuuuagauucuaaagaaacuaucuucuuug
cucauccuugagaagcaacuccuuauccauuaa
..((((........((((((((((((((((((((((((((..(((.((((((((...)))))))).)))..)))))))
))))))))))))))))))))....)))).....

---------------------------AGGAUGAGCAAAGAAAGUAGAUU-- 5 0,0

>hsa-mir-1255b-1 5p:3-24 SRX018958
uaCGGAUGAGCAAAGAAAGUGGUUucuuaaaauggaaucuacucuuugugaagaugcugugaa
((((.(..(((((((..((((.((((......)))).)))))))))))..)....)))))..
--CGGAUGAGCAAAGAAAGUGGUa-------------------------------------- 2 0,-1
--CGGAUGAGCAAAGAAAGUGGUU-------------------------------------- 17 0,0
--CGGAUGAGCAAAGAAAGUGGUUa------------------------------------- 8 0,0
--CGGAUGAGCAAAGAAAGUGGUUU------------------------------------- 7 0,1
--CGGAUGAGCAAAGAAAGUGGUUUu------------------------------------ 2 0,1

>hsa-mir-1255b-2 5p:6-27 SRX018958
ucuuaCGGAUGAGCAAAGAAAGUGGUUugcgccucaagaaaccacuuucuuugcucauccauaagga
(((((.((((((((((((((((((((((.(.......)))))))))))))))))))))))).))))).
-----CGGAUGAGCAAAGAAAGUGGUa--------------------------------------- 2 0,-1
-----CGGAUGAGCAAAGAAAGUGGUU--------------------------------------- 17 0,0
-----CGGAUGAGCAAAGAAAGUGGUUa-------------------------------------- 8 0,0
-----CGGAUGAGCAAAGAAAGUGGUUU-------------------------------------- 7 0,1
-----CGGAUGAGCAAAGAAAGUGGUUUu------------------------------------- 2 0,1

>hsa-mir-1256 5p:35-56 SRX018958
agucagccuguugaagcuuugaagcuuugaugccAGGCAUUGACUUCUCACUAGCUgugaaaguccuagcuaaagagaag
ucaaugcaugacaucuuguuucaauagauggcuguuuca
(((((..((((((((((..........((((.((.(((((((((((((..((((((.(.....).))))))..))))))
)))))).)).)))))..))))))))))).))))))......
-----------------------------------AGGCAUUGACUUCUCACUAGC---6 0,-1
-----------------------------------AGGCAUUGACUUCUCACUAGCU--6 0,0

>hsa-mir-1258 3p:44-64 SRX018958
cuguggcuuccacgaccuaauccuaacuccugcgagucccuggAGUUAGGAUUAGGUCGUGGAAgccacagga
(((((((((((((((((((((((((((((...........))))))))))))))))))))))))))))))..
---AGUUAGGAUUAGGUCGUGGAAa-------- 2
0,0

>hsa-mir-125a 5p:15-38;3p:53-74 SRX018958

ugccagucucuaggUCCCUGAGACCCUUUAACCUGUGAggacauccagggucACAGGUGAGGUUCUUGGGAGCCuggcgucuggcc
.(((((.(.(((((..((..(((.((((..((((((((((....))....))))))))))).)))..))..))))).).)))))).
--------------UCCCUGAGACCCUUUAACC-- 893 0,-5
--------------UCCCUGAGACCCUUUAACCa--- 5 0,-5
--------------UCCCUGAGACCCUUUAACCU--- 8494 0,-4
--------------UCCCUGAGACCCUUUAACCUa-- 6 0,-4
--------------UCCCUGAGACCCUUUAACCUG-- 821 0,-3
--------------UCCCUGAGACCCUUUAACCUGa--- 35 0,-3
--------------UCCCUGAGACCCUUUAACCUGaa-- 9 0,-3
--------------UCCCUGAGACCCUUUAACCUGU--- 907 0,-2
--------------UCCCUGAGACCCUUUAACCUGUa-- 10 0,-2
--------------UCCCUGAGACCCUUUAACCUGUaa--- 2 0,-2
--------------UCCCUGAGACCCUUUAACCUGUu-- 2 0,-2
--------------UCCCUGAGACCCUUUAACCUGUG-- 239 0,-1
--------------UCCCUGAGACCCUUUAACCUGUGu--- 9 0,-1
--------------UCCCUGAGACCCUUUAACCUGUGA--- 40 0,0
--------------UCCCUGAGACCCUUUAACCUGUGAa-- 5 0,0
---------------CCCUGAGACCCUUUAACCU--- 5 1,-4
----------------CCUGAGACCCUUUAACCU--- 25 2,-4

----------------CCUGAGACCCUUUAACCUG---
------ 6 2,-3
----------------CCUGAGACCCUUUAACCUGU--
------ 27 2,-2
----------------CCUGAGACCCUUUAACCUGUG---
------ 5 2,-1
---CAGGUGAGGUUCUUGGGAGC-------
------ 2 1,-1

>hsa-mir-125b-1 MA:15-36;mi:55-76 SRX018958
ugcgcuccucucagUCCCUGAGACCCUAACUUGUGAuguuuaccguuuaaauccACGGGUUAGGCUCUUGGGAGCUgcga
gucgugcu
.((((..(((.(((..((..(((.(((((((((((..(((((.....))))).))))))))))).)))..))..))).))
)..)))).
--------------UCCCUGAGACCCUAACUU--
-------- 235 0,-4
--------------UCCCUGAGACCCUAACUUa---
-------- 15 0,-4
--------------UCCCUGAGACCCUAACUUaa--
-------- 10 0,-4
--------------UCCCUGAGACCCUAACUUau--
-------- 4 0,-4
--------------UCCCUGAGACCCUAACUUu---
-------- 5 0,-4
--------------UCCCUGAGACCCUAACUUuu--
-------- 2 0,-4
--------------UCCCUGAGACCCUAACUUG---
-------- 737 0,-3
--------------UCCCUGAGACCCUAACUUGa--
-------- 43 0,-3
--------------UCCCUGAGACCCUAACUUGaa---
-------- 5 0,-3
--------------UCCCUGAGACCCUAACUUGU--
-------- 2151 0,-2
--------------UCCCUGAGACCCUAACUUGUa---
-------- 12 0,-2
--------------UCCCUGAGACCCUAACUUGUaa--
-------- 27 0,-2

--------------UCCCUGAGACCCUAACUUGUau--
--------	4	0,-2
--------------UCCCUGAGACCCUAACUUGUu---
--------	16	0,-2
--------------UCCCUGAGACCCUAACUUGUG---
--------	4296	0,-1
--------------UCCCUGAGACCCUAACUUGUGu--
--------	319	0,-1
--------------UCCCUGAGACCCUAACUUGUGA--
--------	12593	0,0
--------------UCCCUGAGACCCUAACUUGUGAa---
--------	3669	0,0
--------------UCCCUGAGACCCUAACUUGUGAaa--
--------	450	0,0
--------------UCCCUGAGACCCUAACUUGUGAau--
--------	29	0,0
--------------UCCCUGAGACCCUAACUUGUGAU---
--------	242	0,1
--------------UCCCUGAGACCCUAACUUGUGAUa--
--------	46	0,1
--------------UCCCUGAGACCCUAACUUGUGAUu--
--------	5	0,1
--------------UCCCUGAGACCCUAACUUGUGAUG--
--------	3	0,2
---------------CCCUGAGACCCUAACUUG---
--------	4	1,-3
---------------CCCUGAGACCCUAACUUGUGA--
--------	3	1,0
---------------CCCUGAGACCCUAACUUGUGAa---
--------	3	1,0
---------------CCCUGAGACCCUAACUUGUGAU---
--------	4	1,1
----------------CCUGAGACCCUAACUUGU--
--------	13	2,-2
----------------CCUGAGACCCUAACUUGUG---
--------	9	2,-1
----------------CCUGAGACCCUAACUUGUGu--
--------	5	2,-1

----------------CCUGAGACCCUAACUUGUGA--
--------   95 2,0
----------------CCUGAGACCCUAACUUGUGAa---
--------   27 2,0
----------------CCUGAGACCCUAACUUGUGAaa--
--------   7   2,0
----------------CCUGAGACCCUAACUUGUGAU---
--------   4   2,1
---CACGGGUUAGGCUCUUGGGAGa-----
--------   2   -1,-2
--ACGGGUUAGGCUCUUGGGAG------
--------   2   0,-2
--ACGGGUUAGGCUCUUGGGAGu-----
--------   8   0,-2
--ACGGGUUAGGCUCUUGGGAGC-----
--------   5   0,-1

>hsa-mir-125b-2 MA:17-38;mi:54-75   SRX018958
accagacuuuuccuagUCCCUGAGACCCUAACUUGUGAgguauuuuaguaacaUCACAAGUCAGGCUCUUGGGACcuagg
cggaggga
.((...(((..(((((..((..(((.(((.((((((((..(((....)))...)))))))).))).)))..))..)))))
..))).)).
----------------UCCCUGAGACCCUAACUU--
---------   235 0,-4
----------------UCCCUGAGACCCUAACUUa---
---------   15 0,-4
----------------UCCCUGAGACCCUAACUUaa--
---------   10 0,-4
----------------UCCCUGAGACCCUAACUUau--
---------   4   0,-4
----------------UCCCUGAGACCCUAACUUu---
---------   5   0,-4
----------------UCCCUGAGACCCUAACUUuu--
---------   2   0,-4
----------------UCCCUGAGACCCUAACUUG---
---------   737 0,-3
----------------UCCCUGAGACCCUAACUUGa--
---------   43 0,-3

----------------UCCCUGAGACCCUAACUUGaa--
---------	5	0,-3
----------------UCCCUGAGACCCUAACUUGU---
---------	2151	0,-2
----------------UCCCUGAGACCCUAACUUGUa--
---------	12	0,-2
----------------UCCCUGAGACCCUAACUUGUaa---
---------	27	0,-2
----------------UCCCUGAGACCCUAACUUGUau---
---------	4	0,-2
----------------UCCCUGAGACCCUAACUUGUu--
---------	16	0,-2
----------------UCCCUGAGACCCUAACUUGUG--
---------	4296	0,-1
----------------UCCCUGAGACCCUAACUUGUGu---
---------	319	0,-1
----------------UCCCUGAGACCCUAACUUGUGA---
---------	12593	0,0
----------------UCCCUGAGACCCUAACUUGUGAa--
---------	3669	0,0
----------------UCCCUGAGACCCUAACUUGUGAaa---
---------	450	0,0
----------------UCCCUGAGACCCUAACUUGUGAau---
---------	29	0,0
----------------UCCCUGAGACCCUAACUUGUGAG--
---------	28	0,1
----------------UCCCUGAGACCCUAACUUGUGAGa---
---------	11	0,1
----------------UCCCUGAGACCCUAACUUGUGAGu---
---------	3	0,1
-----------------CCCUGAGACCCUAACUUG--
---------	4	1,-3
-----------------CCCUGAGACCCUAACUUGUGA---
---------	3	1,0
-----------------CCCUGAGACCCUAACUUGUGAa--
---------	3	1,0
------------------CCUGAGACCCUAACUUGU---
---------	13	2,-2

------------------CCUGAGACCCUAACUUGUG--

---------   9   2,-1

------------------CCUGAGACCCUAACUUGUGu---

---------   5   2,-1

------------------CCUGAGACCCUAACUUGUGA---

---------   95   2,0

------------------CCUGAGACCCUAACUUGUGAa--

---------   27   2,0

------------------CCUGAGACCCUAACUUGUGAaa---

---------   7   2,0

---AUCACAAGUCAGGCUCUU----------

---------   2   -1,-5

---AUCACAAGUCAGGCUCUUG---------

---------   10   -1,-4

---AUCACAAGUCAGGCUCUUGG--------

---------   16   -1,-3

---AUCACAAGUCAGGCUCUUGGG-------

---------   7   -1,-2

---AUCACAAGUCAGGCUCUUGGGA------

---------   2   -1,-1

--UCACAAGUCAGGCUCUUG---------

---------   28   0,-4

--UCACAAGUCAGGCUCUUGG--------

---------   7   0,-3

--UCACAAGUCAGGCUCUUGGG-------

---------   2   0,-2

--UCACAAGUCAGGCUCUUGGGA------

---------   3   0,-1

--ACAAGUCAGGCUCUUGGG-------

---------   52   2,-2

--ACAAGUCAGGCUCUUGGGA------

---------   36   2,-1

--ACAAGUCAGGCUCUUGGGAC-----

---------   24   2,0

--ACAAGUCAGGCUCUUGGGACau---

---------   3   2,0

--ACAAGUCAGGCUCUUGGGACu----

---------   2   2,0

---ACAAGUCAGGCUCUUGGGACC----
---------   2   2,1
---ACAAGUCAGGCUCUUGGGACCaa--
---------   2   2,1
---ACAAGUCAGGCUCUUGGGACCau--
---------   2   2,1
---ACAAGUCAGGCUCUUGGGACCU---
---------   5   2,2
---ACAAGUCAGGCUCUUGGGACCUu--
---------   8   2,2
---ACAAGUCAGGCUCUUGGGACCUuu-
---------   10   2,2
---ACAAGUCAGGCUCUUGGGACCUA--
---------   2   2,3
---ACAAGUCAGGCUCUUGGGACCUAu-
---------   4   2,3

>hsa-mir-126   mi:15-35;MA:52-73   SRX018958
cgcuggcgacgggaCAUUAUUACUUUUGGUACGCGcugugacacuucaaacUCGUACCGUGAGUAAUAAUGCGccgucca
cggca
.((((..((((((.(((((((((((.(((((((.(...(((....)))..).))))))).))))))))))).))))))..
)))).
-------------ACAUUAUUACUUUUGGUACG---
-----   2   -1,-2
--------------CAUUAUUACUUUUGGUAC--
-----   136 0,-3
--------------CAUUAUUACUUUUGGUACG---
-----   79   0,-2
--------------CAUUAUUACUUUUGGUACGa--
-----   2   0,-2
--------------CAUUAUUACUUUUGGUACGC--
-----   76   0,-1
--------------CAUUAUUACUUUUGGUACGCa---
-----   7   0,-1
--------------CAUUAUUACUUUUGGUACGCaa--
-----   4   0,-1
--------------CAUUAUUACUUUUGGUACGCau--
-----   2   0,-1

--------------CAUUAUUACUUUUGGUACGCu--
----- 2 0,-1
--------------CAUUAUUACUUUUGGUACGCG--
----- 649 0,0
--------------CAUUAUUACUUUUGGUACGCGa---
----- 239 0,0
--------------CAUUAUUACUUUUGGUACGCGaa--
----- 9 0,0
--------------CAUUAUUACUUUUGGUACGCGu---
----- 83 0,0
--------------CAUUAUUACUUUUGGUACGCGuu--
----- 3 0,0
--------------CAUUAUUACUUUUGGUACGCGC---
----- 13 0,1
--------------CAUUAUUACUUUUGGUACGCGCa--
----- 4 0,1
--------------CAUUAUUACUUUUGGUACGCGCU--
----- 5 0,2
---------------AUUAUUACUUUUGGUACG--
----- 9 1,-2
---------------AUUAUUACUUUUGGUACGC---
----- 9 1,-1
---------------AUUAUUACUUUUGGUACGCG--
----- 27 1,0
---------------AUUAUUACUUUUGGUACGCGa---
----- 11 1,0
---------------AUUAUUACUUUUGGUACGCGaa--
----- 3 1,0
---------------AUUAUUACUUUUGGUACGCGCU--
----- 7 1,2
---CUCGUACCGUGAGUAAUAAU----------
----- 4 -1,-3
---CUCGUACCGUGAGUAAUAAUa---------
----- 3 -1,-3
---CUCGUACCGUGAGUAAUAAUG---------
----- 9 -1,-2
---CUCGUACCGUGAGUAAUAAUGa--------
----- 12 -1,-2

--CUCGUACCGUGAGUAAUAAUGC--------
-----   57   -1,-1

--CUCGUACCGUGAGUAAUAAUGCG-------
-----   27   -1,0

---UCGUACCGUGAGUAAUAAU----------
-----   37   0,-3

---UCGUACCGUGAGUAAUAAUa---------
-----   19   0,-3

---UCGUACCGUGAGUAAUAAUG---------
-----   72   0,-2

---UCGUACCGUGAGUAAUAAUGa--------
-----   30   0,-2

---UCGUACCGUGAGUAAUAAUGaa-------
-----   25   0,-2

---UCGUACCGUGAGUAAUAAUGC--------
-----   348 0,-1

---UCGUACCGUGAGUAAUAAUGCa-------
-----   3   0,-1

---UCGUACCGUGAGUAAUAAUGCG-------
-----   232 0,0

---UCGUACCGUGAGUAAUAAUGCGa------
-----   180 0,0

---UCGUACCGUGAGUAAUAAUGCGaa-----
-----   5   0,0

---UCGUACCGUGAGUAAUAAUGCGu------
-----   10   0,0

--CGUACCGUGAGUAAUAAU----------
-----   11   1,-3

--CGUACCGUGAGUAAUAAUa---------
-----   4   1,-3

--CGUACCGUGAGUAAUAAUG---------
-----   44   1,-2

--CGUACCGUGAGUAAUAAUGa--------
-----   3   1,-2

--CGUACCGUGAGUAAUAAUGaa-------
-----   4   1,-2

--CGUACCGUGAGUAAUAAUGC--------
-----   100 1,-1

---CGUACCGUGAGUAAUAAUGCG------
-----	96	1,0
---CGUACCGUGAGUAAUAAUGCGa-----
-----	53	1,0
---CGUACCGUGAGUAAUAAUGCGu-----
-----	19	1,0
--GUACCGUGAGUAAUAAUG--------
-----	8	2,-2
--GUACCGUGAGUAAUAAUGa-------
-----	6	2,-2
--GUACCGUGAGUAAUAAUGC-------
-----	41	2,-1
--GUACCGUGAGUAAUAAUGCaa-----
-----	2	2,-1
--GUACCGUGAGUAAUAAUGCG------
-----	17	2,0
--GUACCGUGAGUAAUAAUGCGa-----
-----	5	2,0

>hsa-mir-1262	5p:14-35	SRX018958
aucuacaauggugAUGGGUGAAUUUGUAGAAGGAUgaaagucaaagaauccuucugggaacuaauuuuuggccuucaaca
agaauugugauau
(((.(((((.((...((((.(((((.(((((((((............))))))))).)))........)).))))...)).
...))))))))..
-------------AUGGGUGAAUUUGUAGAAGGAU---
-------------	8	0,0

>hsa-mir-1265	5p:14-35	SRX018958
augguuugggacuCAGGAUGUGGUCAAGUGUUGUUaaggcauguucaggaacaauacuugaccacauuuugaauuccaaa
ccauau
((((((((((.((((((((((((((((((((((((...(......)...)))))))))))))))))))))))).)))))))
))))..
-------------CAGGAUGUGGUCAAGUGUU--
------	2	0,-3

>hsa-mir-127	5p:23-44;3p:57-78	SRX018958
ugugaucacugucuccagccugCUGAAGCUCAGAGGGCUCUGAUucagaaagaucaUCGGAUCCGUCUGAGCUUGGCUgg
ucggaagucucaucauc

((.(((..(((...((((((.....(((((((((.((.((((((((.....))..)))))).)).)))))))))))))))
.)))..))).)).....

--------------------UGCUGAAGCUCAGAGGGCUC--
-----------------   2   -2,-4

----------------------CUGAAGCUCAGAGGGCUC--
-----------------   13   0,-4

----------------------CUGAAGCUCAGAGGGCUCU---------------------------------------
-----------------   8   0,-3

----------------------CUGAAGCUCAGAGGGCUCUGA-------------------------------------
-----------------   7   0,-1

----------------------CUGAAGCUCAGAGGGCUCUGAU------------------------------------
-----------------   3   0,0

-----------------------UGAAGCUCAGAGGGCUCU---------------------------------------
-----------------   2   1,-3

------------------------GAAGCUCAGAGGGCUCUGAUU-----------------------------------
-----------------   3   2,1

--UCGGAUCCGUCUGAGCUU------
-----------------   2   0,-4

--UCGGAUCCGUCUGAGCUUG-----
-----------------   4   0,-3

--UCGGAUCCGUCUGAGCUUGG----
-----------------   38   0,-2

--UCGGAUCCGUCUGAGCUUGGa---
-----------------   2   0,-2

--UCGGAUCCGUCUGAGCUUGGaa--
-----------------   4   0,-2

--UCGGAUCCGUCUGAGCUUGGau--
-----------------   2   0,-2

--UCGGAUCCGUCUGAGCUUGGu---
-----------------   2   0,-2

--UCGGAUCCGUCUGAGCUUGGuu--
-----------------   2   0,-2

--UCGGAUCCGUCUGAGCUUGGC---
-----------------   77   0,-1

--UCGGAUCCGUCUGAGCUUGGCa--
-----------------   95   0,-1

--UCGGAUCCGUCUGAGCUUGGCaa-
-----------------   92   0,-1

--UCGGAUCCGUCUGAGCUUGGCau-
-----------------   404 0,-1
--UCGGAUCCGUCUGAGCUUGGCU--
-----------------   531 0,0
--UCGGAUCCGUCUGAGCUUGGCUa-
-----------------   28 0,0
--UCGGAUCCGUCUGAGCUUGGCUaa
-----------------   48 0,0
--UCGGAUCCGUCUGAGCUUGGCUau
-----------------   223 0,0
--UCGGAUCCGUCUGAGCUUGGCUu-
-----------------   202 0,0
--UCGGAUCCGUCUGAGCUUGGCUuu
-----------------   60 0,0
--UCGGAUCCGUCUGAGCUUGGCUGa
-----------------   6   0,1
---CGGAUCCGUCUGAGCUUGGCau-
-----------------   2   1,-1
---CGGAUCCGUCUGAGCUUGGCU--
-----------------   4   1,0
---CGGAUCCGUCUGAGCUUGGCUau
-----------------   7   1,0
--GGAUCCGUCUGAGCUUGGCaa-
-----------------   2   2,-1
--GGAUCCGUCUGAGCUUGGCau-
-----------------   6   2,-1
--GGAUCCGUCUGAGCUUGGCU--
-----------------   10 2,0
--GGAUCCGUCUGAGCUUGGCUau
-----------------   7   2,0
--GGAUCCGUCUGAGCUUGGCUu-
-----------------   4   2,0
--GGAUCCGUCUGAGCUUGGCUuu
-----------------   2   2,0

>hsa-mir-1270-1 5p:13-35   SRX018958
cacagaguuauaCUGGAGAUAUGGAAGAGCUGUGUugggguauaaguaacaggcuuuucuuuaucuucuauguggcucuuu
gca

((.(((((((((..(((((((.(((((((((.(((((........))))))))))))))).)))))))..))))))))).)
)..
------------CUGGAGAUAUGGAAGAGC--
--- 3	0,-5
------------CUGGAGAUAUGGAAGAGCU---
--- 2	0,-4
------------CUGGAGAUAUGGAAGAGCUGU---
--- 2	0,-2

>hsa-mir-1270-2 5p:13-35	SRX018958
cacagaguuauaCUGGAGAUAUGGAAGAGCUGUGUuggguauaaguaacaggcuuuucuuuaucuucuauguggcucuuu
gca
((.(((((((((..(((((((.(((((((((.(((((........))))))))))))))).)))))))..))))))))).)
)..
------------CUGGAGAUAUGGAAGAGC--
--- 3	0,-5
------------CUGGAGAUAUGGAAGAGCU---
--- 2	0,-4
------------CUGGAGAUAUGGAAGAGCUGU---
--- 2	0,-2

>hsa-mir-1271	5p:15-36	SRX018958
cacccagaucagugCUUGGCACCUAGCAAGCACUCAguaaauauuuguugagugccugcuaugugccaggcauugugcug
agggcu
..(((((..((((((((((((.(((((.((((((((((((....))))))))))).))))).)))))))))))))..)))
.))...
--------------CUUGGCACCUAGCAAGCAC---
------	42	0,-3
--------------CUUGGCACCUAGCAAGCACU--
------	25	0,-2
--------------CUUGGCACCUAGCAAGCACUC---
------	3	0,-1
--------------CUUGGCACCUAGCAAGCACUCA--
------	4	0,0
--AGUGCCUGCUAUGUGCCA------------
------	4
--AGUGCCUGCUAUGUGCCAG-----------
------	2

>hsa-mir-1273   3p:76-100   SRX018958
ugaggcaggagaauugcuugaacccgggugguggagguugcagugagccaagauugcgccacugcacuccagccuGGGCG
ACAAAGCAAGACUCUUUCUUgga
.((((.(((((..((((((...(((((((..(((((..(((((((.((((....)).)))))))))))))))))))))))..
...))))))..)))))))))...
----GCAGGAGAAUUGCUUGAACCCGu---
---------------------- 2

>hsa-mir-1273c   5p:10-31   SRX018958
ugcagccugGGCGACAAAACGAGACCCUGUCuuuuuuuuuucugagacagagucucguucuguugcccaagcugga
..((((.(((((((((.((((((((.(((((((..........))))))).)))))))).))))))))).))))..
---------GGCGACAAAACGAGACCC---
    6   0,-4
---------GGCGACAAAACGAGACCCU--
    6   0,-3
---------GGCGACAAAACGAGACCCUGU--
    2   0,-1
---------GGCGACAAAACGAGACCCUGUa---
    2   0,-1
---------GGCGACAAAACGAGACCCUGUaa--
    2   0,-1
---------GGCGACAAAACGAGACCCUGUCa--
    10   0,0
---------GGCGACAAAACGAGACCCUGUCU--
    2   0,1

>hsa-mir-1277   3p:47-68   SRX018958
accucccaaauauauauauauauguacguauguguauauaaauguaUACGUAGAUAUAUAUGUAUUUUugguggguuu
(((..(((((.((((((((((((.((((((((..(......)..)))))))).)))))))))))).))))).)))..
--UACGUAGAUAUAUAUGUAU-------------
    2   0,-3
--UACGUAGAUAUAUAUGUAUa------------
    3   0,-3
--UACGUAGAUAUAUAUGUAUU------------
    5   0,-2
--UACGUAGAUAUAUAUGUAUUU-----------
    38   0,-1

--UACGUAGAUAUAUAUGUAUUUU----------
    95 0,0

>hsa-mir-1278   3p:50-71   SRX018958
auuugcucauagaugauaugcauaguacucccagaacucauuaaguuggUAGUACUGUGCAUAUCAUCUAUgagcgaaua
g
(((((((((((((((((((((((((((((.(((..(((.....)))))).))))))))))))))))))))))))))))).
.
--GUAGUACUGUGCAUAUCAUCU-----------
-   4   -1,-2
---UAGUACUGUGCAUAUCAU-------------
-   5   0,-4
---UAGUACUGUGCAUAUCAUa------------
-   10 0,-4
---UAGUACUGUGCAUAUCAUCa-----------
-   2   0,-3
---UAGUACUGUGCAUAUCAUCU-----------
-   25 0,-2
---UAGUACUGUGCAUAUCAUCUA----------
-   11 0,-1
---UAGUACUGUGCAUAUCAUCUAa---------
-   5   0,-1
---UAGUACUGUGCAUAUCAUCUAU---------
-   138 0,0
---UAGUACUGUGCAUAUCAUCUAUa--------
-   11 0,0
--AGUACUGUGCAUAUCAUCUAU---------
-   2   1,0

>hsa-mir-128-1 3p:50-70   SRX018958
ugagcuguuggauucggggccguagcacugucugagagguuuacauuucUCACAGUGAACCGGUCUCUUUuucagcugcu
uc
.((((.((((((...(((((((...((((((..(((((((....)))))))))))))...)))))))...)))))).)))
).
--CUCACAGUGAACCGGUCUCUUUU---------
-- 2   -1,1
---UCACAGUGAACCGGUCUC-------------
-- 47 0,-3

--UCACAGUGAACCGGUCUCaa-----------
-- 2 0,-3
--UCACAGUGAACCGGUCUCU------------
-- 173 0,-2
--UCACAGUGAACCGGUCUCUaa----------
-- 6 0,-2
--UCACAGUGAACCGGUCUCUau----------
-- 3 0,-2
--UCACAGUGAACCGGUCUCUU-----------
-- 299 0,-1
--UCACAGUGAACCGGUCUCUUa----------
-- 67 0,-1
--UCACAGUGAACCGGUCUCUUaa---------
-- 34 0,-1
--UCACAGUGAACCGGUCUCUUau---------
-- 29 0,-1
--UCACAGUGAACCGGUCUCUUU----------
-- 2930 0,0
--UCACAGUGAACCGGUCUCUUUa---------
-- 770 0,0
--UCACAGUGAACCGGUCUCUUUaa--------
-- 141 0,0
--UCACAGUGAACCGGUCUCUUUau--------
-- 98 0,0
--UCACAGUGAACCGGUCUCUUUU---------
-- 1511 0,1
--UCACAGUGAACCGGUCUCUUUUa--------
-- 62 0,1
--UCACAGUGAACCGGUCUCUUUUaa-------
-- 3 0,1
--UCACAGUGAACCGGUCUCUUUUau-------
-- 7 0,1
--UCACAGUGAACCGGUCUCUUUUU--------
-- 80 0,2
--UCACAGUGAACCGGUCUCUUUUUa-------
-- 7 0,2
--UCACAGUGAACCGGUCUCUUUUUu-------
-- 4 0,2

--CACAGUGAACCGGUCUCUUUU---------
-- 2   1,1
---ACAGUGAACCGGUCUCUUa----------
-- 2   2,-1
---ACAGUGAACCGGUCUCUUU----------
-- 10 2,0
---ACAGUGAACCGGUCUCUUUa---------
-- 2   2,0
---ACAGUGAACCGGUCUCUUUU---------
-- 10 2,1
---ACAGUGAACCGGUCUCUUUUa--------
-- 3   2,1
---ACAGUGAACCGGUCUCUUUUU--------
-- 2   2,2

>hsa-mir-128-2 3p:52-72   SRX018958
ugugcagugggaaggggggccgauacacuguacgagagugaguagcaggucUCACAGUGAACCGGUCUCUUUcccuacug
uguc
.(..(((((((.((((((((((...((((((..((((.(........).))))))))))...)))))))))).)))))))
..).
---UCACAGUGAACCGGUCUC-----------
----   47 0,-3
---UCACAGUGAACCGGUCUCaa---------
----   2   0,-3
---UCACAGUGAACCGGUCUCU----------
----   173 0,-2
---UCACAGUGAACCGGUCUCUaa--------
----   6   0,-2
---UCACAGUGAACCGGUCUCUau--------
----   3   0,-2
---UCACAGUGAACCGGUCUCUU---------
----   299 0,-1
---UCACAGUGAACCGGUCUCUUa--------
----   67 0,-1
---UCACAGUGAACCGGUCUCUUaa-------
----   34 0,-1
---UCACAGUGAACCGGUCUCUUau-------
----   29 0,-1

---UCACAGUGAACCGGUCUCUUU--------
----	2930	0,0
---UCACAGUGAACCGGUCUCUUUa-------
----	770	0,0
---UCACAGUGAACCGGUCUCUUUaa------
----	141	0,0
---UCACAGUGAACCGGUCUCUUUau------
----	98	0,0
---UCACAGUGAACCGGUCUCUUUC-------
----	79	0,1
---UCACAGUGAACCGGUCUCUUUCa------
----	17	0,1
---UCACAGUGAACCGGUCUCUUUCu------
----	35	0,1
---ACAGUGAACCGGUCUCUUa--------
----	2	2,-1
---ACAGUGAACCGGUCUCUUU--------
----	10	2,0
---ACAGUGAACCGGUCUCUUUa-------
----	2	2,0

>hsa-mir-1284	5p:30-51	SRX018958
auuuugauauauaagccaguuuaauguuuUCUAUACAGACCCUGGCUUUUCuuaaauuuuauauauuggaaagcccaugu
uuguauuggaaacugcugguuucuuucauacugaaaaucu
....(((.....((((((((.....((((((.((((((((...((((((((................))))))))...))
)))))).)))))).))))))))...)))............
-----------------------------UCUAUACAGACCCUGGCUUU-------------------------------
--	3	0,-2
-----------------------------UCUAUACAGACCCUGGCUUUU------------------------------
--	4	0,-1
-----------------------------UCUAUACAGACCCUGGCUUUUC-----------------------------
--	3	0,0

>hsa-mir-1285-1	3p:51-72	SRX018958
uguagagauaggaucucacuuuguugcccaggcuggucucaaacuccuggUCUGGGCAACAAAGUGAGACCUuaucucua
caag
(((((((((((.(((((((((((((((((((((..(..........)..))))))))))))))))))))).)))))))))
))..

--UCUGGGCAACAAAGUGAG------------
----   2   0,-4
--UCUGGGCAACAAAGUGAGA-----------
----   5   0,-3
--UCUGGGCAACAAAGUGAGAC----------
----   33   0,-2
--UCUGGGCAACAAAGUGAGACa---------
----   7   0,-2
--UCUGGGCAACAAAGUGAGACaa--------
----   11   0,-2
--UCUGGGCAACAAAGUGAGACau--------
----   2   0,-2
--UCUGGGCAACAAAGUGAGACC---------
----   20   0,-1
--UCUGGGCAACAAAGUGAGACCaa-------
----   9   0,-1
--UCUGGGCAACAAAGUGAGACCau-------
----   11   0,-1
--UCUGGGCAACAAAGUGAGACCU--------
----   78   0,0
--UCUGGGCAACAAAGUGAGACCUa-------
----   30   0,0
--UCUGGGCAACAAAGUGAGACCUaa------
----   10   0,0
--UCUGGGCAACAAAGUGAGACCUau------
----   64   0,0
--UCUGGGCAACAAAGUGAGACCUU-------
----   72   0,1
--UCUGGGCAACAAAGUGAGACCUUu------
----   26   0,1
--UCUGGGCAACAAAGUGAGACCUUuu-----
----   3   0,1
--UCUGGGCAACAAAGUGAGACCUUA------
----   12   0,2
--UCUGGGCAACAAAGUGAGACCUUAU-----
----   3   0,3
---CUGGGCAACAAAGUGAGACC---------
----   2   1,-1

>hsa-mir-1285-2 3p:52-73 SRX018958
uuugggaggccgaggcuggugcaucacuugagcccagcaauuugagaccaaUCUGGGCAACAAAGUGAGACCUccgucuc
uacaaaga
((((((((((.((((........((((((..((((((....(((....))).))))))....))))))..)))).)))))
).)))))..
---UCUGGGCAACAAAGUGAG-----------
-------- 2 0,-4
---UCUGGGCAACAAAGUGAGA----------
-------- 5 0,-3
---UCUGGGCAACAAAGUGAGAC---------
-------- 33 0,-2
---UCUGGGCAACAAAGUGAGACa--------
-------- 7 0,-2
---UCUGGGCAACAAAGUGAGACaa-------
-------- 11 0,-2
---UCUGGGCAACAAAGUGAGACau-------
-------- 2 0,-2
---UCUGGGCAACAAAGUGAGACC--------
-------- 20 0,-1
---UCUGGGCAACAAAGUGAGACCaa------
-------- 9 0,-1
---UCUGGGCAACAAAGUGAGACCau------
-------- 11 0,-1
---UCUGGGCAACAAAGUGAGACCU-------
-------- 78 0,0
---UCUGGGCAACAAAGUGAGACCUa------
-------- 30 0,0
---UCUGGGCAACAAAGUGAGACCUaa-----
-------- 10 0,0
---UCUGGGCAACAAAGUGAGACCUau-----
-------- 64 0,0
--CUGGGCAACAAAGUGAGACC--------
-------- 2 1,-1

>hsa-mir-1287 5p:16-37 SRX018958
guugugcuguccaggUGCUGGAUCAGUGGUUCGAGUCugagccuuuaaaagccacucuagccacagaugcagugauugga
gccaugacaa

((..((...(((((..((((.(((.((((((.((((..(.((........))))))).)))))).))).))))..)))))
..))..))..
---------------UGCUGGAUCAGUGGUUCGAG--
---------- 3 0,-2

>hsa-mir-129-2 5p:15-35;3p:57-78 SRX018958
ugcccuucgcgaauCUUUUUGCGGUCUGGGCUUGCuguacauaacucaauagccggAAGCCCUUACCCCAAAAAGCAUuu
gcggagggcg
.((((((((((((((((((.(((..(((((((((((.........)))))...))))))..))).))))))).))))
))))))))).
--AAGCCCUUACCCCAAAAAGCAU--
---------- 3 0,0

>hsa-mir-1291 5p:14-37 SRX018958
gguagaauuccagUGGCCCUGACUGAAGACCAGCAGUuguacuguggcuguugguuucaagcagaggccuaaaggacugu
cuuccug
((.(((..(((...((((((.((..(((((((((((..(...)..)))))))))))..))))).))))....)))...)
)).)))..
------------GUGGCCCUGACUGAAGACCA--
------- 3 -1,-5
------------GUGGCCCUGACUGAAGACCAGC--
------- 8 -1,-3
------------GUGGCCCUGACUGAAGACCAGCA---
------- 203 -1,-2
-------------UGGCCCUGACUGAAGACCAGCA---
------- 17 0,-2

>hsa-mir-1301 3p:48-71 SRX018958
ggauugugggggucgcucuaggcaccgcagcacugugcuggggaugUUGCAGCUGCCUGGGAGUGACUUCacacagucc
uc
(((((((.(((((((((((((((((.((((((((.......)).)))))).).))))).))))))))))).)))))))
..
---UUGCAGCUGCCUGGGAGUGAC------------
-- 3 0,-3

>hsa-mir-1303 3p:52-73 SRX018958
ggcugggcaacauagcgagaccucaacucuacaauuuuuuuuuuuuuaaauUUUAGAGACGGGGUCUUGCUCUguugcca
ggcuuu

((((.(((((((.(((((((((((..(((((.(((((..........))))).)))))..))))))))))).))))))).
))))..
-------------AGCGAGACCUCAACUCUACAAU---
------ 3
--UUUUAGAGACGGGGUCUUGC----------
------ 2   -1,-3
--UUUUAGAGACGGGGUCUUGCU---------
------ 16 -1,-2
---UUUAGAGACGGGGUCUUGCU---------
------ 13 0,-2
---UUUAGAGACGGGGUCUUGCUaa-------
------ 2   0,-2
--UUAGAGACGGGGUCUUGCU---------
------ 3   1,-2

>hsa-mir-1306   3p:55-72   SRX018958
gugagcagucuccaccaccucccccugcaaacguccaguggugcagagguaauggACGUUGGCUCUGGUGGUGauggacag
uccga
.((..(.((((.(((((((......((.((((((((....(((....))).)))))))).))...)))))))..)))).)
..)).
--------------CCACCUCCCCUGCAAACGUCCAa---
-----   3
--ACGUUGGCUCUGGUGGUGAUG-----
-----   2   0,3
--ACGUUGGCUCUGGUGGUGAUGu----
-----   3   0,3

>hsa-mir-1307   3p:80-101   SRX018958
caucaagacccagcugagucacugucacugccuaccaaucucgaccggaccucgaccggcucgucuguguugccaaucgA
CUCGGCGUGGCGUCGGUCGUGguagauaggcggucaugcauacgaauuucagcucuuguucuggugac
(((((..((..(((((((((..(((.((((((((.(.(((.(((((((.((.((.(((..(((..((......))..)))
..))))).))..))))))).))).).))))))))...)))...))...)))))))...))..)))))..
--UCGACCGGACCUCGACCG----------------------
---   4
--UCGACCGGACCUCGACCGaa--------------------
---   2
--UCGACCGGACCUCGACCGG---------------------
---   12

--UCGACCGGACCUCGACCGGC--------------------
-- 16
--UCGACCGGACCUCGACCGGCaa------------------
-- 5
--UCGACCGGACCUCGACCGGCau------------------
-- 2
--UCGACCGGACCUCGACCGGCU-------------------
-- 409
--UCGACCGGACCUCGACCGGCUa------------------
-- 4
--UCGACCGGACCUCGACCGGCUaa-----------------
-- 11
--UCGACCGGACCUCGACCGGCUau-----------------
-- 5
--UCGACCGGACCUCGACCGGCUu------------------
-- 74
--UCGACCGGACCUCGACCGGCUuu-----------------
-- 21
--UCGACCGGACCUCGACCGGCUC------------------
-- 151
--UCGACCGGACCUCGACCGGCUCa-----------------
-- 18
--UCGACCGGACCUCGACCGGCUCaa----------------
-- 2
--UCGACCGGACCUCGACCGGCUCau----------------
-- 3
--UCGACCGGACCUCGACCGGCUCu-----------------
-- 17
--UCGACCGGACCUCGACCGGCUCG----------------
-- 12
---CGACCGGACCUCGACCGGCU-------------------
-- 2
---CGACCGGACCUCGACCGGCUu------------------
-- 2
--GACCGGACCUCGACCGGCaa------------------
-- 2
--GACCGGACCUCGACCGGCU-------------------
-- 2

--GACCGGACCUCGACCGGCUu------------------
-- 2

--GACCGGACCUCGACCGGCUC------------------
-- 5

---A
CUCGGCGUGGCGUCGGU--- 4 0,-4

---A
CUCGGCGUGGCGUCGGUCG--- 11 0,-2

---A
CUCGGCGUGGCGUCGGUCGU-- 15 0,-1

---A
CUCGGCGUGGCGUCGGUCGUu--- 4 0,-1

---A
CUCGGCGUGGCGUCGGUCGUG--- 59 0,0

---A
CUCGGCGUGGCGUCGGUCGUGu-- 4 0,0

CUCGGCGUGGCGUCGGUCGUG--- 35 1,0

>hsa-mir-130a mi:21-42;MA:55-76 SRX018958

ugcugcuggccagagcucuuUUCACAUUGUGCUACUGUCUGCaccugucacuagCAGUGCAAUGUUAAAAGGGCAUuggc
cguguagug

.((((((((((((.((((((((.((((((..((.(((...((....))...))).))..)))))).)))))))).)))))
)).))))).

---GCAGUGCAAUGUUAAAAGGGCAUU---
--------- 3 -1,1

--CAGUGCAAUGUUAAAAGG--------
--------- 3398 0,-4

--CAGUGCAAUGUUAAAAGGu-------
--------- 4 0,-4

--CAGUGCAAUGUUAAAAGGG-------
--------- 471 0,-3

--CAGUGCAAUGUUAAAAGGGa------
--------- 3 0,-3

--CAGUGCAAUGUUAAAAGGGu------
--------- 5 0,-3

--CAGUGCAAUGUUAAAAGGGC------
--------- 831 0,-2

--CAGUGCAAUGUUAAAAGGGCA-----
---------	196	0,-1
--CAGUGCAAUGUUAAAAGGGCAa----
---------	26	0,-1
--CAGUGCAAUGUUAAAAGGGCAaa---
---------	10	0,-1
--CAGUGCAAUGUUAAAAGGGCAau---
---------	7	0,-1
--CAGUGCAAUGUUAAAAGGGCAU----
---------	1072	0,0
--CAGUGCAAUGUUAAAAGGGCAUa---
---------	339	0,0
--CAGUGCAAUGUUAAAAGGGCAUaa--
---------	19	0,0
--CAGUGCAAUGUUAAAAGGGCAUau--
---------	6	0,0
--CAGUGCAAUGUUAAAAGGGCAUU---
---------	375	0,1
--CAGUGCAAUGUUAAAAGGGCAUUa--
---------	12	0,1
--CAGUGCAAUGUUAAAAGGGCAUUau-
---------	7	0,1
--CAGUGCAAUGUUAAAAGGGCAUUu--
---------	7	0,1
--CAGUGCAAUGUUAAAAGGGCAUUuu-
---------	4	0,1
---AGUGCAAUGUUAAAAGGG-------
---------	12	1,-3
---AGUGCAAUGUUAAAAGGGC------
---------	11	1,-2
---AGUGCAAUGUUAAAAGGGCA-----
---------	8	1,-1
---AGUGCAAUGUUAAAAGGGCAa----
---------	2	1,-1
---AGUGCAAUGUUAAAAGGGCAU----
---------	19	1,0
---AGUGCAAUGUUAAAAGGGCAUa---
---------	6	1,0

---AGUGCAAUGUUAAAAGGGCAUU---
---------   2   1,1

>hsa-mir-130b   mi:13-33;MA:51-72   SRX018958
ggccugcccgacACUCUUUCCCUGUUGCACUACuauaggccgcugggaagCAGUGCAAUGAUGAAAGGGCAUcggucagg
uc
((((((.((((..(((((((..(((((((((.((.(((....)))...)).)))))))))..)))))))..)))).))))
))
------------ACUCUUUCCCUGUUGCACUACU--
-- 9   0,1
--CAGUGCAAUGAUGAAAGG------------
-- 83 0,-4
--CAGUGCAAUGAUGAAAGGG-----------
-- 18 0,-3
--CAGUGCAAUGAUGAAAGGGC----------
-- 12 0,-2
--CAGUGCAAUGAUGAAAGGGCA---------
-- 14 0,-1
--CAGUGCAAUGAUGAAAGGGCAa--------
-- 11 0,-1
--CAGUGCAAUGAUGAAAGGGCAau-------
-- 2   0,-1
--CAGUGCAAUGAUGAAAGGGCAU--------
-- 365 0,0
--CAGUGCAAUGAUGAAAGGGCAUa-------
-- 54 0,0
--CAGUGCAAUGAUGAAAGGGCAUaa------
-- 2   0,0
--CAGUGCAAUGAUGAAAGGGCAUu-------
-- 91 0,0
--CAGUGCAAUGAUGAAAGGGCAUuu------
-- 13 0,0
--CAGUGCAAUGAUGAAAGGGCAUC-------
-- 7   0,1
---AGUGCAAUGAUGAAAGGGCA---------
-- 2   1,-1
---AGUGCAAUGAUGAAAGGGCAU--------
-- 7   1,0

>hsa-mir-132   mi:23-44;MA:59-80   SRX018958
ccgcccccgcgucuccagggcaACCGUGGCUUUCGAUUGUUACUgugggaacuggaggUAACAGUCUACAGCCAUGGUCG
ccccgcagcacgcccacgcgc
.(((....((((((.(.((((.(((((((((...((((((((((..(....)....))))))))))...))))))))).)
))).).)).))))....))).
---------------------AACCGUGGCUUUCGAUUGUU---------------------------------------
--------------------   2   -1,-3
---------------------AACCGUGGCUUUCGAUUGUUA--------------------------------------
--------------------   14 -1,-2
----------------------ACCGUGGCUUUCGAUUGUUAC-------------------------------------
--------------------   3   0,-1
----------------------ACCGUGGCUUUCGAUUGUUACU------------------------------------
--------------------   8   0,0
--UAACAGUCUACAGCCAUG----
--------------------   8   0,-4
--UAACAGUCUACAGCCAUGG---
--------------------   11 0,-3
--UAACAGUCUACAGCCAUGGU--
--------------------   8   0,-2
--UAACAGUCUACAGCCAUGGUC-
--------------------   4   0,-1
--UAACAGUCUACAGCCAUGGUCG
--------------------   12 0,0
--UAACAGUCUACAGCCAUGGUCG
u-------------------   8   0,0
---AACAGUCUACAGCCAUGG---
--------------------   2   1,-3
---AACAGUCUACAGCCAUGGU--
--------------------   3   1,-2
--ACAGUCUACAGCCAUGGUCG
--------------------   4   2,0

>hsa-mir-133a-1 3p:53-74   SRX018958
acaaugcuuugcuagagcugguaaaauggaaccaaaucgccucuucaauggaUUUGGUCCCCUUCAACCAGCUGuagcua
ugcauuga
.((((((...((((.(((((((..((.((.((((((((...........)))))))).)).))..))))))).))))..
.)))))).

---------------AGCUGGUAAAAUGGAACCAA-- 3
--UUGGUCCCCUUCAACCAGCUGU------------- 2 1,1

>hsa-mir-133a-2 3p:59-80 SRX018958
gggagccaaaugcuuugcuagagcugguaaaauggaaccaaaucgacuguccaauggaUUUGGUCCCCUUCAACCAGCUGuagcugugcauugauggcgccg
.((.(((((((((...((((.(((((((..((.((.((((((((.(........).)))))))).)).))..))))))).))))...)))))..)))).)).
---------------------AGCUGGUAAAAUGGAACCAA--- 3
---UUGGUCCCCUUCAACCAGCUGU--------------------- 2 1,1

>hsa-mir-134 5p:8-29 SRX018958
cagggugUGUGACUGGUUGACCAGAGGGGcaugcacuguguucacccugugggccaccuagucaccaacccuc
.((((..((((((((.((.(((.((((((((.....))))...)))).)))..)).))))))))..))))).
-------UGUGACUGGUUGACCAGAGGG--- 4 0,-1
-------UGUGACUGGUUGACCAGAGGGa-- 7 0,-1
-------UGUGACUGGUUGACCAGAGGGaa--- 3 0,-1
-------UGUGACUGGUUGACCAGAGGGau--- 2 0,-1
-------UGUGACUGGUUGACCAGAGGGuu--- 3 0,-1
-------UGUGACUGGUUGACCAGAGGGG-- 12 0,0
-------UGUGACUGGUUGACCAGAGGGGa--- 2 0,0
-------UGUGACUGGUUGACCAGAGGGGau-- 3 0,0

>hsa-mir-135a-2 5p:23-45 SRX018958
agauaaauucacucuagugcuuUAUGGCUUUUUAUUCCUAUGUGAuaguaauaaagucucauguagggauggaagccaugaaauacauugugaaaaauca

.(((...(((((....(((.((((((((((((.((((((((((((.(.(.....).).))))))))))))))))))))))
)).)))...)))))..))).
---UGUAGGGAUGGAAGCCAUG
AAA-----------------   2

>hsa-mir-135b   MA:16-38;mi:55-76   SRX018958
cacucugcuguggccUAUGGCUUUUCAUUCCUAUGUGAuugcugucccaaacucAUGUAGGGCUAAAAGCCAUGGGcuac
agugaggggcgagcucc
..(((((((((((((((((((((((...(((((((((((((......)))..)))))))))..)))))))))))))))))
))).)))).........
---------------UAUGGCUUUUCAUUCCUAUGU--
-----------------   5   0,-2
---------------UAUGGCUUUUCAUUCCUAUGUG---
-----------------   3   0,-1
---------------UAUGGCUUUUCAUUCCUAUGUGA--
-----------------   4   0,0

>hsa-mir-136   MA:15-37;mi:49-70   SRX018958
ugagcccucggaggACUCCAUUUGUUUUGAUGAUGGAuucuuaugcucCAUCAUCGUCUCAAAUGAGUCUucagaggguu
cu
.((((((((.((((((((.(((((...((((((((((.........))))))))))...))))))))))))).)))))))
).
--------------ACUCCAUUUGUUUUGAUG--
--   4   0,-5
--------------ACUCCAUUUGUUUUGAUGA---
--   21 0,-4
--------------ACUCCAUUUGUUUUGAUGAU--
--   52 0,-3
--------------ACUCCAUUUGUUUUGAUGAUG---
--   3   0,-2
--------------ACUCCAUUUGUUUUGAUGAUGG--
--   2   0,-1
--------------ACUCCAUUUGUUUUGAUGAUGGA---
--   9   0,0
--CAUCAUCGUCUCAAAUGA--------------
--   12 0,-4
--CAUCAUCGUCUCAAAUGAG-------------
--   3   0,-3

--CAUCAUCGUCUCAAAUGAGUC-----------
-- 2   0,-1
--CAUCAUCGUCUCAAAUGAGUCU----------
-- 6   0,0
---AUCAUCGUCUCAAAUGAG-------------
-- 3   1,-3

>hsa-mir-138-1 MA:23-45;mi:63-84   SRX018958
cccuggcauggugugguggggcAGCUGGUGUUGUGAAUCAGGCCGuugccaaucagagaacgGCUACUUCACAACACCAG
GGCCacaccacacuacagg
.((((...((((((((((.(((..(((((((((((((...(((((((..(.....)..)))))))..)))))))))))))
.))).))))))))))))))
----------------------AGCUGGUGUUGUGAAUCA--
------------------- 3   0,-5
----------------------AGCUGGUGUUGUGAAUCAGGC-------------------------------------
------------------- 2   0,-2
----------------------AGCUGGUGUUGUGAAUCAGGCC------------------------------------
------------------- 3   0,-1

>hsa-mir-138-2 MA:10-32;mi:57-78   SRX018958
cguugcugcAGCUGGUGUUGUGAAUCAGGCCGacgagcagcgcauccucuuacccgGCUAUUUCACGACACCAGGGUUgc
auca
.(.(((.((..(((((((((((((...(((((..(((.((......)))))...)))))..))))))))))))).)).))
).).
---------AGCUGGUGUUGUGAAUCA---
----   3   0,-5
---------AGCUGGUGUUGUGAAUCAGGC--
----   2   0,-2
---------AGCUGGUGUUGUGAAUCAGGCC---
----   3   0,-1

>hsa-mir-139   5p:7-28;3p:44-65   SRX018958
guguauUCUACAGUGCACGUGUCUCCAGuguggcucggaggcuGGAGACGCGGCCCUGUUGGAGUaac
...((((((((((.((.((((((((((((.(.......).)))))))))))))).))).)))))))..
------UCUACAGUGCACGUGUCU--   11   0,-4
------UCUACAGUGCACGUGUCUC---   49   0,-3
------UCUACAGUGCACGUGUCUCa--   3   0,-3
------UCUACAGUGCACGUGUCUCC--   39   0,-2

------UCUACAGUGCACGUGUCUCCA--------------------------------------- 196 0,-1
------UCUACAGUGCACGUGUCUCCAa-------------------------------------- 26 0,-1
------UCUACAGUGCACGUGUCUCCAaa------------------------------------- 2 0,-1
------UCUACAGUGCACGUGUCUCCAau------------------------------------- 3 0,-1
------UCUACAGUGCACGUGUCUCCAu-------------------------------------- 25 0,-1
------UCUACAGUGCACGUGUCUCCAG-------------------------------------- 261 0,0
------UCUACAGUGCACGUGUCUCCAGa------------------------------------- 46 0,0
------UCUACAGUGCACGUGUCUCCAGaa------------------------------------ 3 0,0
------UCUACAGUGCACGUGUCUCCAGU------------------------------------- 470 0,1
------UCUACAGUGCACGUGUCUCCAGUa------------------------------------ 8 0,1
------UCUACAGUGCACGUGUCUCCAGUau----------------------------------- 3 0,1
------UCUACAGUGCACGUGUCUCCAGUu------------------------------------ 18 0,1
--UGGAGACGCGGCCCUGUU-------- 12
-1,-5
--UGGAGACGCGGCCCUGUUG------- 19
-1,-4
--UGGAGACGCGGCCCUGUUGG------ 20
-1,-3
--UGGAGACGCGGCCCUGUUGGA----- 25
-1,-2
--UGGAGACGCGGCCCUGUUGGAa---- 3
-1,-2
--UGGAGACGCGGCCCUGUUGGAaa--- 6
-1,-2
--UGGAGACGCGGCCCUGUUGGAau--- 2
-1,-2
--UGGAGACGCGGCCCUGUUGGAu---- 2
-1,-2
--UGGAGACGCGGCCCUGUUGGAG---- 54
-1,-1
--UGGAGACGCGGCCCUGUUGGAGa--- 20
-1,-1
--UGGAGACGCGGCCCUGUUGGAGU--- 110 -1,0
--UGGAGACGCGGCCCUGUUGGAGUu-- 3 -1,0
--UGGAGACGCGGCCCUGUUGGAGUuu- 2 -1,0
--UGGAGACGCGGCCCUGUUGGAGUAu- 5 -1,1
---GGAGACGCGGCCCUGUUG------- 3 0,-4
---GGAGACGCGGCCCUGUUGG------ 3 0,-3

---GGAGACGCGGCCCUGUUGGA-----   4   0,-2
---GGAGACGCGGCCCUGUUGGAG----   10   0,-1
---GGAGACGCGGCCCUGUUGGAGa---   2   0,-1
---GGAGACGCGGCCCUGUUGGAGU---   32   0,0
---GGAGACGCGGCCCUGUUGGAGUu--   2   0,0
---AGACGCGGCCCUGUUGGAGU---   5   2,0
---AGACGCGGCCCUGUUGGAGUu--   5   2,0
---AGACGCGGCCCUGUUGGAGUuu-   9   2,0

>hsa-mir-140   5p:23-44;3p:62-82   SRX018958
ugugucucucucuguguccugcCAGUGGUUUUACCCUAUGGUAGguuacgucaugcuguucUACCACAGGGUAGAACCAC
GGacaggauaccggggcacc
.(((((((.....((((((((((.(((((((((((((.(((((((..(((......)))))))))).)))))))))))))
)).)))))))).))))))).
----------------------CAGUGGUUUUACCCUAUGGUA-------------------------------------
--------------------   2   0,-1
----------------------CAGUGGUUUUACCCUAUGGUAa------------------------------------
--------------------   5   0,-1
----------------------CAGUGGUUUUACCCUAUGGUAGa-----------------------------------
--------------------   2   0,0
--CUACCACAGGGUAGAACCAC
G-------------------   4   -1,-1
--CUACCACAGGGUAGAACCAC
GGA-----------------   11   -1,1
--CUACCACAGGGUAGAACCAC
GGAa----------------   2   -1,1
--CUACCACAGGGUAGAACCAC
GGAC----------------   4   -1,2
---UACCACAGGGUAGAACCA-
--------------------   83   0,-3
---UACCACAGGGUAGAACCAC
--------------------   162 0,-2
---UACCACAGGGUAGAACCAC
a-------------------   2   0,-2
---UACCACAGGGUAGAACCAC
G-------------------   1899   0,-1
---UACCACAGGGUAGAACCAC
Ga------------------   15   0,-1

--UACCACAGGGUAGAACCAC
Gaa-----------------   3   0,-1
--UACCACAGGGUAGAACCAC
Gu------------------   3   0,-1
--UACCACAGGGUAGAACCAC
GG------------------   3373   0,0
--UACCACAGGGUAGAACCAC
GGu-----------------   75   0,0
--UACCACAGGGUAGAACCAC
GGuu----------------   34   0,0
--UACCACAGGGUAGAACCAC
GGA-----------------   3617   0,1
--UACCACAGGGUAGAACCAC
GGAa----------------   1302   0,1
--UACCACAGGGUAGAACCAC
GGAaa---------------   657 0,1
--UACCACAGGGUAGAACCAC
GGAau---------------   248 0,1
--UACCACAGGGUAGAACCAC
GGAu----------------   266 0,1
--UACCACAGGGUAGAACCAC
GGAuu---------------   20   0,1
--UACCACAGGGUAGAACCAC
GGAC----------------   1173   0,2
--UACCACAGGGUAGAACCAC
GGACu---------------   23   0,2
--UACCACAGGGUAGAACCAC
GGACA---------------   155 0,3
--UACCACAGGGUAGAACCAC
GGACAa--------------   19   0,3
--UACCACAGGGUAGAACCAC
GGACAu--------------   9   0,3
---ACCACAGGGUAGAACCAC
--------------------   57   1,-2
---ACCACAGGGUAGAACCAC
u-------------------   3   1,-2
---ACCACAGGGUAGAACCAC
G-------------------   128 1,-1

--ACCACAGGGUAGAACCAC

Gaa-----------------   5   1,-1

--ACCACAGGGUAGAACCAC

GG------------------   2916   1,0

--ACCACAGGGUAGAACCAC

GGu-----------------   182 1,0

--ACCACAGGGUAGAACCAC

GGuu----------------   110 1,0

--ACCACAGGGUAGAACCAC

GGA-----------------   17362   1,1

--ACCACAGGGUAGAACCAC

GGAa----------------   6393   1,1

--ACCACAGGGUAGAACCAC

GGAaa---------------   2325   1,1

--ACCACAGGGUAGAACCAC

GGAau---------------   509 1,1

--ACCACAGGGUAGAACCAC

GGAu----------------   3146   1,1

--ACCACAGGGUAGAACCAC

GGAuu---------------   238 1,1

--ACCACAGGGUAGAACCAC

GGAC----------------   3941   1,2

--ACCACAGGGUAGAACCAC

GGACu---------------   96   1,2

--ACCACAGGGUAGAACCAC

GGACuu--------------   12   1,2

--ACCACAGGGUAGAACCAC

GGACA---------------   409 1,3

--ACCACAGGGUAGAACCAC

GGACAa--------------   273 1,3

--ACCACAGGGUAGAACCAC

GGACAaa-------------   21   1,3

--ACCACAGGGUAGAACCAC

GGACAau-------------   8   1,3

--ACCACAGGGUAGAACCAC

GGACAu--------------   55   1,3

--ACCACAGGGUAGAACCAC

GGACAG--------------   17   1,4

--ACCACAGGGUAGAACCAC
GGACAGu-------------   3   1,4
---CCACAGGGUAGAACCAC
GG------------------   61   2,0
---CCACAGGGUAGAACCAC
GGu-----------------   4   2,0
---CCACAGGGUAGAACCAC
GGuu----------------   4   2,0
---CCACAGGGUAGAACCAC
GGA-----------------   308 2,1
---CCACAGGGUAGAACCAC
GGAa----------------   130 2,1
---CCACAGGGUAGAACCAC
GGAaa---------------   60   2,1
---CCACAGGGUAGAACCAC
GGAau---------------   13   2,1
---CCACAGGGUAGAACCAC
GGAu----------------   61   2,1
---CCACAGGGUAGAACCAC
GGAuu---------------   4   2,1
---CCACAGGGUAGAACCAC
GGAC----------------   98   2,2
---CCACAGGGUAGAACCAC
GGACu---------------   5   2,2
---CCACAGGGUAGAACCAC
GGACA---------------   26   2,3
---CCACAGGGUAGAACCAC
GGACAa--------------   12   2,3
---CCACAGGGUAGAACCAC
GGACAaa-------------   5   2,3
---CCACAGGGUAGAACCAC
GGACAu--------------   3   2,3
---CCACAGGGUAGAACCAC
GGACAG--------------   3   2,4

>hsa-mir-141   mi:17-38;MA:59-80   SRX018958
cggccggcccugggucCAUCUUCCAGUACAGUGUUGGAuggucuaauugugaagcuccUAACACUGUCUGGUAAAGAUGG
cucccggguggguuc

.((((.((((.(((.((((((((((.(((((((((((...((.......))...))).)))))))))))..)))))))
..)))))))).)))).
--UAACACUGUCUGGUAAAGAU--
--------------- 3   0,-2
--UAACACUGUCUGGUAAAGAUG-
--------------- 2   0,-1
--UAACACUGUCUGGUAAAGAUGu
--------------- 3   0,-1
--UAACACUGUCUGGUAAAGAUGu
u-------------- 2   0,-1

>hsa-mir-142   5p:16-36;3p:52-74   SRX018958
gacagugcagucaccCAUAAAGUAGAAAGCACUACUaacagcacuggagggUGUAGUGUUUCCUACUUUAUGGAugagug
uacugug
.((((((((.(((.(((((((((((.(((((((((...(..(....)..)..))))))))).))))))))))).))).))
)))))).
-------------CCCAUAAAGUAGAAAGCA---
------- 50 -2,-5
-------------CCCAUAAAGUAGAAAGCAa--
------- 31 -2,-5
-------------CCCAUAAAGUAGAAAGCAaa---
------- 2   -2,-5
-------------CCCAUAAAGUAGAAAGCAuu---
------- 7   -2,-5
-------------CCCAUAAAGUAGAAAGCAC--
------- 59 -2,-4
-------------CCCAUAAAGUAGAAAGCACa---
------- 3   -2,-4
-------------CCCAUAAAGUAGAAAGCACU---
------- 375 -2,-3
-------------CCCAUAAAGUAGAAAGCACUA--
------- 159 -2,-2
-------------CCCAUAAAGUAGAAAGCACUAa---
------- 36 -2,-2
-------------CCCAUAAAGUAGAAAGCACUAaa--
------- 9   -2,-2
-------------CCCAUAAAGUAGAAAGCACUAu---
------- 28 -2,-2

-------------CCCAUAAAGUAGAAAGCACUAC---

------- 33 -2,-1

-------------CCCAUAAAGUAGAAAGCACUACa--

------- 2 -2,-1

--------------CCAUAAAGUAGAAAGCACU---

------- 5 -1,-3

--------------CCAUAAAGUAGAAAGCACUA--

------- 6 -1,-2

--------------CCAUAAAGUAGAAAGCACUAu---

------- 2 -1,-2

---------------CAUAAAGUAGAAAGCACU---

------- 16 0,-3

---------------CAUAAAGUAGAAAGCACUA--

------- 102 0,-2

---------------CAUAAAGUAGAAAGCACUAa---

------- 44 0,-2

---------------CAUAAAGUAGAAAGCACUAaa--

------- 34 0,-2

---------------CAUAAAGUAGAAAGCACUAu---

------- 4 0,-2

---------------CAUAAAGUAGAAAGCACUAuu--

------- 2 0,-2

---------------CAUAAAGUAGAAAGCACUAC---

------- 90 0,-1

---------------CAUAAAGUAGAAAGCACUACU--

------- 88 0,0

---------------CAUAAAGUAGAAAGCACUACUA---

------- 64 0,1

---------------CAUAAAGUAGAAAGCACUACUAu--

------- 10 0,1

---------------CAUAAAGUAGAAAGCACUACUAA--

------- 25 0,2

----------------AUAAAGUAGAAAGCACUA--

------- 19 1,-2

----------------AUAAAGUAGAAAGCACUAa---

------- 2 1,-2

----------------AUAAAGUAGAAAGCACUAaa--

------- 2 1,-2

----------------AUAAAGUAGAAAGCACUAC---
------- 10 1,-1
----------------AUAAAGUAGAAAGCACUACU--
------- 18 1,0
----------------AUAAAGUAGAAAGCACUACUA---
------- 18 1,1
----------------AUAAAGUAGAAAGCACUACUAu--
------- 15 1,1
----------------AUAAAGUAGAAAGCACUACUAA--
------- 211 1,2
-----------------UAAAGUAGAAAGCACUAC---
------- 3   2,-1
---UGUAGUGUUUCCUACUUUAUG--------
------- 3   0,-2
---UGUAGUGUUUCCUACUUUAUGG-------
------- 5   0,-1
--GUAGUGUUUCCUACUUUAU---------
------- 2   1,-3
--GUAGUGUUUCCUACUUUAUG--------
------- 4   1,-2
--GUAGUGUUUCCUACUUUAUGa-------
------- 2   1,-2
--GUAGUGUUUCCUACUUUAUGG-------
------- 36 1,-1

>hsa-mir-143   mi:27-48;MA:61-81   SRX018958
gcgcagcgcccugucucccagccugaGGUGCAGUGCUGCAUCUCUGGUcaguugggagucUGAGAUGAAGCACUGUAGCU
Caggaagagagaaguuguucugcagc
..((((.((....((((.(..((((((.(((((((((.((((((.((((......)).)).))))))).))))))))).))
))))..).))))....)).))))...
-------------------------AGGUGCAGUGCUGCAUCUCU-----------------------------------
-------------------------- 12 -1,-3
--------------------------GGUGCAGUGCUGCAUCUC------------------------------------
-------------------------- 305 0,-4
--------------------------GGUGCAGUGCUGCAUCUCU-----------------------------------
-------------------------- 2650   0,-3
--------------------------GGUGCAGUGCUGCAUCUCUu----------------------------------
-------------------------- 4   0,-3

--------------------------GGUGCAGUGCUGCAUCUCUG----------------------------------
------------------------- 57 0,-2
--------------------------GGUGCAGUGCUGCAUCUCUGa---------------------------------
------------------------- 6 0,-2
--------------------------GGUGCAGUGCUGCAUCUCUGaa--------------------------------
------------------------- 6 0,-2
--------------------------GGUGCAGUGCUGCAUCUCUGG---------------------------------
------------------------- 62 0,-1
--------------------------GGUGCAGUGCUGCAUCUCUGGa--------------------------------
------------------------- 41 0,-1
--------------------------GGUGCAGUGCUGCAUCUCUGGaa-------------------------------
------------------------- 14 0,-1
--------------------------GGUGCAGUGCUGCAUCUCUGGau-------------------------------
------------------------- 3 0,-1
--------------------------GGUGCAGUGCUGCAUCUCUGGU--------------------------------
------------------------- 8 0,0
--------------------------GGUGCAGUGCUGCAUCUCUGGUa-------------------------------
------------------------- 14 0,0
--------------------------GGUGCAGUGCUGCAUCUCUGGUaa------------------------------
------------------------- 10 0,0
--------------------------GGUGCAGUGCUGCAUCUCUGGUau------------------------------
------------------------- 23 0,0
--------------------------GGUGCAGUGCUGCAUCUCUGGUu-------------------------------
------------------------- 3 0,0
--------------------------GGUGCAGUGCUGCAUCUCUGGUuu------------------------------
------------------------- 3 0,0
--------------------------GGUGCAGUGCUGCAUCUCUGGUC-------------------------------
------------------------- 2 0,1
--------------------------GGUGCAGUGCUGCAUCUCUGGUCA------------------------------
------------------------- 4 0,2
--------------------------GGUGCAGUGCUGCAUCUCUGGUCAa-----------------------------
------------------------- 2 0,2
--UCUGAGAUGAAGCACUGUAGC-
------------------------- 3 -2,-2
--UCUGAGAUGAAGCACUGUAGCa
u------------------------ 3 -2,-2
--UCUGAGAUGAAGCACUGUAGCU
------------------------- 7 -2,-1

---UCUGAGAUGAAGCACUGUAGCU
a------------------------ 3 -2,-1
---UCUGAGAUGAAGCACUGUAGCU
au----------------------- 2 -2,-1
---UCUGAGAUGAAGCACUGUAGCU
C------------------------ 5 -2,0
--CUGAGAUGAAGCACUGUA---
------------------------- 10 -1,-4
--CUGAGAUGAAGCACUGUAG--
------------------------- 5 -1,-3
--CUGAGAUGAAGCACUGUAGa-
------------------------- 2 -1,-3
--CUGAGAUGAAGCACUGUAGC-
------------------------- 15 -1,-2
--CUGAGAUGAAGCACUGUAGCU
------------------------- 95 -1,-1
--CUGAGAUGAAGCACUGUAGCU
a------------------------ 4 -1,-1
--CUGAGAUGAAGCACUGUAGCU
aa----------------------- 2 -1,-1
--CUGAGAUGAAGCACUGUAGCU
au----------------------- 2 -1,-1
--CUGAGAUGAAGCACUGUAGCU
u------------------------ 3 -1,-1
--CUGAGAUGAAGCACUGUAGCU
C------------------------ 86 -1,0
--CUGAGAUGAAGCACUGUAGCU
Cu----------------------- 29 -1,0
--CUGAGAUGAAGCACUGUAGCU
Cuu---------------------- 3 -1,0
--CUGAGAUGAAGCACUGUAGCU
CA----------------------- 4 -1,1
---UGAGAUGAAGCACUGUAG--
------------------------- 418 0,-3
---UGAGAUGAAGCACUGUAGa-
------------------------- 5 0,-3
---UGAGAUGAAGCACUGUAGaa
------------------------- 7 0,-3

---UGAGAUGAAGCACUGUAGau
-------------------------- 2   0,-3
---UGAGAUGAAGCACUGUAGu-
-------------------------- 2   0,-3
---UGAGAUGAAGCACUGUAGuu
-------------------------- 8   0,-3
---UGAGAUGAAGCACUGUAGC-
-------------------------- 385 0,-2
---UGAGAUGAAGCACUGUAGCa
-------------------------- 56   0,-2
---UGAGAUGAAGCACUGUAGCa
a------------------------- 73   0,-2
---UGAGAUGAAGCACUGUAGCa
u------------------------- 5   0,-2
---UGAGAUGAAGCACUGUAGCU
-------------------------- 4959   0,-1
---UGAGAUGAAGCACUGUAGCU
a------------------------- 302 0,-1
---UGAGAUGAAGCACUGUAGCU
aa------------------------ 99   0,-1
---UGAGAUGAAGCACUGUAGCU
au------------------------ 56   0,-1
---UGAGAUGAAGCACUGUAGCU
u------------------------- 111 0,-1
---UGAGAUGAAGCACUGUAGCU
uu------------------------ 26   0,-1
---UGAGAUGAAGCACUGUAGCU
C------------------------- 7883   0,0
---UGAGAUGAAGCACUGUAGCU
Cu------------------------ 2173   0,0
---UGAGAUGAAGCACUGUAGCU
Cuu----------------------- 326 0,0
---UGAGAUGAAGCACUGUAGCU
CA------------------------ 492 0,1
---UGAGAUGAAGCACUGUAGCU
CAa----------------------- 66   0,1
---UGAGAUGAAGCACUGUAGCU
CAaa---------------------- 12   0,1

--UGAGAUGAAGCACUGUAGCU
CAau---------------------- 3 0,1
--UGAGAUGAAGCACUGUAGCU
CAu----------------------- 11 0,1
--UGAGAUGAAGCACUGUAGCU
CAG----------------------- 4 0,2
---GAGAUGAAGCACUGUAGC-
-------------------------- 7 1,-2
---GAGAUGAAGCACUGUAGCU
-------------------------- 32 1,-1
---GAGAUGAAGCACUGUAGCU
C------------------------- 94 1,0
---GAGAUGAAGCACUGUAGCU
Cu------------------------ 22 1,0
---GAGAUGAAGCACUGUAGCU
Cuu----------------------- 3 1,0
---GAGAUGAAGCACUGUAGCU
CA------------------------ 3 1,1
--AGAUGAAGCACUGUAGCU
-------------------------- 20 2,-1
--AGAUGAAGCACUGUAGCU
C------------------------- 18 2,0
--AGAUGAAGCACUGUAGCU
Cu------------------------ 3 2,0

>hsa-mir-144 mi:15-36;MA:52-71 SRX018958
uggggcccuggcugGGAUAUCAUCAUAUACUGUAAGuuugcgaugagacacUACAGUAUAGAUGAUGUACUaguccgggc
accccc
.((((((.((((((.((((((((.(((((((((.((((......))))..)))))))))))))))))).)))))).))))
.)))..
--------------GGAUAUCAUCAUAUACUG--
------ 26 0,-4
--------------GGAUAUCAUCAUAUACUGU---
------ 8 0,-3
--------------GGAUAUCAUCAUAUACUGUA--
------ 7 0,-2
--------------GGAUAUCAUCAUAUACUGUAA---
------ 11 0,-1

--------------GGAUAUCAUCAUAUACUGUAAG--
------ 65 0,0
--------------GGAUAUCAUCAUAUACUGUAAGU---
------ 31 0,1
--------------GGAUAUCAUCAUAUACUGUAAGUa--
------ 3   0,1
--------------GGAUAUCAUCAUAUACUGUAAGUU--
------ 15 0,2
--CUACAGUAUAGAUGAUGU------------
------ 3   -1,-3
--CUACAGUAUAGAUGAUGUA-----------
------ 5   -1,-2
---UACAGUAUAGAUGAUGUA-----------
------ 3   0,-2
---UACAGUAUAGAUGAUGUAa----------
------ 2   0,-2
---UACAGUAUAGAUGAUGUAC----------
------ 10 0,-1
---UACAGUAUAGAUGAUGUACU---------
------ 11 0,0
---UACAGUAUAGAUGAUGUACUuu-------
------ 2   0,0
---UACAGUAUAGAUGAUGUACUAa-------
------ 7   0,1
---UACAGUAUAGAUGAUGUACUAG-------
------ 3   0,2

>hsa-mir-145   MA:16-38;mi:54-75   SRX018958
caccuuguccucacgGUCCAGUUUUCCCAGGAAUCCCUuagaugcuaagauggGGAUUCCUGGAAAUACUGUUCUugagg
ucaugguu
.(((.((.(((((.((..((((.((.((((((((((((............)))))))))))).)).))))..)))))))
.)).))).
---------------GUCCAGUUUUCCCAGGAA---
--------   8   0,-5
---------------GUCCAGUUUUCCCAGGAAa--
--------   12   0,-5
---------------GUCCAGUUUUCCCAGGAAaa---
--------   3   0,-5

---------------GUCCAGUUUUCCCAGGAAU---
-------- 91 0,-4
---------------GUCCAGUUUUCCCAGGAAUa--
-------- 86 0,-4
---------------GUCCAGUUUUCCCAGGAAUaa---
-------- 17 0,-4
---------------GUCCAGUUUUCCCAGGAAUu--
-------- 3 0,-4
---------------GUCCAGUUUUCCCAGGAAUC--
-------- 37 0,-3
---------------GUCCAGUUUUCCCAGGAAUCa---
-------- 47 0,-3
---------------GUCCAGUUUUCCCAGGAAUCaa--
-------- 3 0,-3
---------------GUCCAGUUUUCCCAGGAAUCu---
-------- 5 0,-3
---------------GUCCAGUUUUCCCAGGAAUCC---
-------- 173 0,-2
---------------GUCCAGUUUUCCCAGGAAUCCa--
-------- 26 0,-2
---------------GUCCAGUUUUCCCAGGAAUCCu--
-------- 4 0,-2
---------------GUCCAGUUUUCCCAGGAAUCCuu---
-------- 2 0,-2
---------------GUCCAGUUUUCCCAGGAAUCCC--
-------- 379 0,-1
---------------GUCCAGUUUUCCCAGGAAUCCCa---
-------- 27 0,-1
---------------GUCCAGUUUUCCCAGGAAUCCCU---
-------- 621 0,0
---------------GUCCAGUUUUCCCAGGAAUCCCUa--
-------- 10 0,0
---------------GUCCAGUUUUCCCAGGAAUCCCUU--
-------- 48 0,1
---------------GUCCAGUUUUCCCAGGAAUCCCUUu---
-------- 7 0,1
----------------UCCAGUUUUCCCAGGAAUCCCU---
-------- 2 1,0

---GGAUUCCUGGAAAUACUGUUC------
--------	12	0,-1
---GGAUUCCUGGAAAUACUGUUCa-----
--------	2	0,-1
---GGAUUCCUGGAAAUACUGUUCU-----
--------	38	0,0
--GAUUCCUGGAAAUACUGUUCU-----
--------	5	1,0
--GAUUCCUGGAAAUACUGUUCUU----
--------	4	1,1
---AUUCCUGGAAAUACUGUU-------
--------	3	2,-2
---AUUCCUGGAAAUACUGUUC------
--------	13	2,-1
---AUUCCUGGAAAUACUGUUCa-----
--------	4	2,-1
---AUUCCUGGAAAUACUGUUCU-----
--------	93	2,0
---AUUCCUGGAAAUACUGUUCUa----
--------	9	2,0
---AUUCCUGGAAAUACUGUUCUau---
--------	2	2,0
---AUUCCUGGAAAUACUGUUCUU----
--------	73	2,1
---AUUCCUGGAAAUACUGUUCUUa---
--------	2	2,1
---AUUCCUGGAAAUACUGUUCUUaa--
--------	2	2,1
---AUUCCUGGAAAUACUGUUCUUu---
--------	3	2,1
---AUUCCUGGAAAUACUGUUCUUG---
--------	4	2,2

>hsa-mir-1468	5p:13-33	SRX018958
ggugggugguuuCUCCGUUUGCCUGUUUCGCUGaugugcauucaacucauucucagcaaaauaagcaaauggaaaauucg
uccauc
(((((..(..((.(((((((((.(((((.(((((.(((.........)))..))))).))))).))))))))).))..).
.)))))

------------CUCCGUUUGCCUGUUUCGC--
------ 3 0,-2
------------CUCCGUUUGCCUGUUUCGCU---
------ 11 0,-1
------------CUCCGUUUGCCUGUUUCGCUGA---
------ 10 0,1

>hsa-mir-146a MA:21-42;mi:57-78 SRX018958
ccgauguguauccucagcuuUGAGAACUGAAUUCCAUGGGUUgugucagugucagaCCUCUGAAAUUCAGUUCUUCAGcu
gggauaucucugucaucgu
.(((((.((((((.(((((..(((((((((((.((.(((((.((.......))))))).)).)))))))))))).))))
)))))))......))))).
-------------------UUGAGAACUGAAUUCCAUGGGUU--------------------------------------
------------------- 2 -1,0
--------------------UGAGAACUGAAUUCCAUG--
------------------- 5 0,-4
--------------------UGAGAACUGAAUUCCAUGG---
------------------- 19 0,-3
--------------------UGAGAACUGAAUUCCAUGGa--
------------------- 2 0,-3
--------------------UGAGAACUGAAUUCCAUGGG--
------------------- 17 0,-2
--------------------UGAGAACUGAAUUCCAUGGGau--------------------------------------
------------------- 2 0,-2
--------------------UGAGAACUGAAUUCCAUGGGU---------------------------------------
------------------- 59 0,-1
--------------------UGAGAACUGAAUUCCAUGGGUa--------------------------------------
------------------- 17 0,-1
--------------------UGAGAACUGAAUUCCAUGGGUaa-------------------------------------
------------------- 21 0,-1
--------------------UGAGAACUGAAUUCCAUGGGUau-------------------------------------
------------------- 71 0,-1
--------------------UGAGAACUGAAUUCCAUGGGUU--------------------------------------
------------------- 277 0,0
--------------------UGAGAACUGAAUUCCAUGGGUUa-------------------------------------
------------------- 168 0,0
--------------------UGAGAACUGAAUUCCAUGGGUUaa------------------------------------
------------------- 51 0,0

--------------------UGAGAACUGAAUUCCAUGGGUUau------------------------------------
------------------- 88 0,0
--------------------UGAGAACUGAAUUCCAUGGGUUu-------------------------------------
------------------- 107 0,0
--------------------UGAGAACUGAAUUCCAUGGGUUuu------------------------------------
------------------- 22 0,0
--------------------UGAGAACUGAAUUCCAUGGGUUG-------------------------------------
------------------- 69 0,1
--------------------UGAGAACUGAAUUCCAUGGGUUGa------------------------------------
------------------- 22 0,1
--------------------UGAGAACUGAAUUCCAUGGGUUGU------------------------------------
------------------- 36 0,2
---------------------GAGAACUGAAUUCCAUGGGU---------------------------------------
------------------- 2   1,-1
---------------------GAGAACUGAAUUCCAUGGGUa--------------------------------------
------------------- 2   1,-1
---------------------GAGAACUGAAUUCCAUGGGUU--------------------------------------
------------------- 19 1,0
---------------------GAGAACUGAAUUCCAUGGGUUa-------------------------------------
------------------- 8   1,0
---------------------GAGAACUGAAUUCCAUGGGUUau------------------------------------
------------------- 4   1,0
---------------------GAGAACUGAAUUCCAUGGGUUu-------------------------------------
------------------- 8   1,0
---------------------GAGAACUGAAUUCCAUGGGUUG-------------------------------------
------------------- 4   1,1
---------------------GAGAACUGAAUUCCAUGGGUUGa------------------------------------
------------------- 2   1,1
----------------------AGAACUGAAUUCCAUGGG--
------------------- 3   2,-2
----------------------AGAACUGAAUUCCAUGGGUU--------------------------------------
------------------- 17 2,0
----------------------AGAACUGAAUUCCAUGGGUUG-------------------------------------
------------------- 9   2,1

>hsa-mir-146b   5p:9-30;3p:45-66   SRX018958
ccuggcacUGAGAACUGAAUUCCAUAGGCUgugagcucuagcaaUGCCCUGUGGACUCAGUUCUGGugcccgg
((.((((((.((((((((..((((((((..((..((....)).))..)))))))).))))))))))))))).))

-------CUGAGAACUGAAUUCCAU--- 4

-1,-5

-------CUGAGAACUGAAUUCCAUAGG-- 4

-1,-2

-------CUGAGAACUGAAUUCCAUAGGC--- 5

-1,-1

-------CUGAGAACUGAAUUCCAUAGGCU-- 111

-1,0

--------UGAGAACUGAAUUCCAUA-- 2

0,-4

--------UGAGAACUGAAUUCCAUAG--- 40

0,-3

--------UGAGAACUGAAUUCCAUAGG-- 186

0,-2

--------UGAGAACUGAAUUCCAUAGGau-- 2

0,-2

--------UGAGAACUGAAUUCCAUAGGC--- 90

0,-1

--------UGAGAACUGAAUUCCAUAGGCa-- 100

0,-1

--------UGAGAACUGAAUUCCAUAGGCaa--- 46

0,-1

--------UGAGAACUGAAUUCCAUAGGCau--- 27

0,-1

--------UGAGAACUGAAUUCCAUAGGCU-- 2300

0,0

--------UGAGAACUGAAUUCCAUAGGCUa--- 19

0,0

--------UGAGAACUGAAUUCCAUAGGCUaa-- 38

0,0

--------UGAGAACUGAAUUCCAUAGGCUau-- 120

0,0

--------UGAGAACUGAAUUCCAUAGGCUu--- 260

0,0

--------UGAGAACUGAAUUCCAUAGGCUuu-- 147

0,0

--------UGAGAACUGAAUUCCAUAGGCUG--- 119

0,1

--------UGAGAACUGAAUUCCAUAGGCUGa-- 483
0,1
--------UGAGAACUGAAUUCCAUAGGCUGaa--------------------------------------- 11
0,1
--------UGAGAACUGAAUUCCAUAGGCUGau--------------------------------------- 14
0,1
--------UGAGAACUGAAUUCCAUAGGCUGU-- 21
0,2
--------UGAGAACUGAAUUCCAUAGGCUGUa--------------------------------------- 4
0,2
---------GAGAACUGAAUUCCAUAGG-- 4
1,-2
---------GAGAACUGAAUUCCAUAGGCU-- 37
1,0
---------GAGAACUGAAUUCCAUAGGCUu--- 7
1,0
---------GAGAACUGAAUUCCAUAGGCUG--- 7
1,1
---------GAGAACUGAAUUCCAUAGGCUGa-- 7
1,1
----------AGAACUGAAUUCCAUAGGCU-- 3
2,0
---UGCCCUGUGGACUCAGUUCUGGUau---- 3
0,1
--GCCCUGUGGACUCAGUUCUG-------- 5
1,-1
--GCCCUGUGGACUCAGUUCUGG------- 18
1,0
--GCCCUGUGGACUCAGUUCUGGU------ 16
1,1
--GCCCUGUGGACUCAGUUCUGGUa----- 15
1,1
--GCCCUGUGGACUCAGUUCUGGUaa---- 2
1,1
--GCCCUGUGGACUCAGUUCUGGUau---- 75
1,1
--GCCCUGUGGACUCAGUUCUGGUu----- 9
1,1

--GCCCUGUGGACUCAGUUCUGGUuu----   4
1,1
--GCCCUGUGGACUCAGUUCUGGUG-----   2
1,2
---CCCUGUGGACUCAGUUCUGG-------   2
2,0
---CCCUGUGGACUCAGUUCUGGUa-----   2
2,1
---CCCUGUGGACUCAGUUCUGGUau----   5
2,1

>hsa-mir-148a   mi:6-27;MA:44-65   SRX018958
gaggcAAAGUUCUGAGACACUCCGACUcugaguaugauagaagUCAGUGCACUACAGAACUUUGUcuc
((((((((((((((.((((..(((((((((.......))).))))))))..)).))))))))))))))
----CAAAGUUCUGAGACACUCCGA---   6
-1,-2
-----AAAGUUCUGAGACACUCC---   3   0,-4
-----AAAGUUCUGAGACACUCCGA---   151   0,-2
-----AAAGUUCUGAGACACUCCGAa--   6   0,-2
-----AAAGUUCUGAGACACUCCGAaa---   3   0,-2
-----AAAGUUCUGAGACACUCCGAC--   8   0,-1
-----AAAGUUCUGAGACACUCCGACU---   57   0,0
-----AAAGUUCUGAGACACUCCGACUa--   4   0,0
-----AAAGUUCUGAGACACUCCGACUu--   4   0,0
------AAGUUCUGAGACACUCCGA---   2   1,-2
---AGUCAGUGCACUACAGAACUUUG----   2
-2,-1
--GUCAGUGCACUACAGAACUUUG----   2
-1,-1
--GUCAGUGCACUACAGAACUUUGa---   2
-1,-1
--GUCAGUGCACUACAGAACUUUGU---   12   -1,0
---UCAGUGCACUACAGAACU-------   12983
0,-4
---UCAGUGCACUACAGAACUa------   8   0,-4
---UCAGUGCACUACAGAACUaa-----   10   0,-4
---UCAGUGCACUACAGAACUau-----   5   0,-4
---UCAGUGCACUACAGAACUU------   818   0,-3

---UCAGUGCACUACAGAACUUa----- 15 0,-3
---UCAGUGCACUACAGAACUUaa---- 8 0,-3
---UCAGUGCACUACAGAACUUau---- 7 0,-3
---UCAGUGCACUACAGAACUUU----- 4586
0,-2
---UCAGUGCACUACAGAACUUUa---- 80 0,-2
---UCAGUGCACUACAGAACUUUaa--- 19 0,-2
---UCAGUGCACUACAGAACUUUau--- 22 0,-2
---UCAGUGCACUACAGAACUUUu---- 55 0,-2
---UCAGUGCACUACAGAACUUUuu--- 16 0,-2
---UCAGUGCACUACAGAACUUUG---- 6471
0,-1
---UCAGUGCACUACAGAACUUUGa--- 6205
0,-1
---UCAGUGCACUACAGAACUUUGaa-- 705 0,-1
---UCAGUGCACUACAGAACUUUGau-- 14 0,-1
---UCAGUGCACUACAGAACUUUGU--- 58520
0,0
---UCAGUGCACUACAGAACUUUGUa-- 479 0,0
---UCAGUGCACUACAGAACUUUGUaa- 109 0,0
---UCAGUGCACUACAGAACUUUGUau- 41 0,0
---UCAGUGCACUACAGAACUUUGUu-- 697 0,0
---UCAGUGCACUACAGAACUUUGUuu- 34 0,0
---UCAGUGCACUACAGAACUUUGUC-- 933 0,1
---UCAGUGCACUACAGAACUUUGUCa- 55 0,1
---UCAGUGCACUACAGAACUUUGUCaa 2 0,1
---UCAGUGCACUACAGAACUUUGUCau 4 0,1
---UCAGUGCACUACAGAACUUUGUCU- 186 0,2
---UCAGUGCACUACAGAACUUUGUCUu 3 0,2
---UCAGUGCACUACAGAACUUUGUCUC 3 0,3
--CAGUGCACUACAGAACUUU----- 6 1,-2
--CAGUGCACUACAGAACUUUG---- 2 1,-1
--CAGUGCACUACAGAACUUUGU--- 74 1,0
--CAGUGCACUACAGAACUUUGUu-- 3 1,0
---AGUGCACUACAGAACUUU----- 112 2,-2
---AGUGCACUACAGAACUUUG---- 99 2,-1
---AGUGCACUACAGAACUUUGa--- 68 2,-1
---AGUGCACUACAGAACUUUGaa-- 16 2,-1

---AGUGCACUACAGAACUUUGU--- 885 2,0
---AGUGCACUACAGAACUUUGUa-- 14 2,0
---AGUGCACUACAGAACUUUGUaa- 2 2,0
---AGUGCACUACAGAACUUUGUu-- 15 2,0
---AGUGCACUACAGAACUUUGUC-- 22 2,1
---AGUGCACUACAGAACUUUGUCa- 4 2,1
---AGUGCACUACAGAACUUUGUCU- 7 2,2

>hsa-mir-148b mi:25-46;MA:63-84 SRX018958
caagcacgauuagcauuugaggugAAGUUCUGUUAUACACUCAGGCuguggcucucugaaagUCAGUGCAUCACAGAACU
UUGUcucgaaagcuuucua
.......((..(((.(((((((..((((((((.((.((((..((((....(.....)..)))))))).)).))))))))
)..))))))).))).))..
----------------------UGAAGUUCUGUUAUACACU---------------------------------------
------------------- 2 -2,-5
-----------------------GAAGUUCUGUUAUACACU---------------------------------------
------------------- 19 -1,-5
-----------------------GAAGUUCUGUUAUACACUC--------------------------------------
------------------- 8 -1,-4
-----------------------GAAGUUCUGUUAUACACUCAG------------------------------------
------------------- 6 -1,-2
-----------------------GAAGUUCUGUUAUACACUCAGG-----------------------------------
------------------- 5 -1,-1
-----------------------GAAGUUCUGUUAUACACUCAGGC----------------------------------
------------------- 3 -1,0
-----------------------GAAGUUCUGUUAUACACUCAGGCa---------------------------------
------------------- 2 -1,0
-----------------------GAAGUUCUGUUAUACACUCAGGCau--------------------------------
------------------- 5 -1,0
-----------------------GAAGUUCUGUUAUACACUCAGGCU---------------------------------
------------------- 45 -1,1
-----------------------GAAGUUCUGUUAUACACUCAGGCUa--------------------------------
------------------- 7 -1,1
-----------------------GAAGUUCUGUUAUACACUCAGGCUu--------------------------------
------------------- 4 -1,1
--UCAGUGCAUCACAGAACU
------------------- 1456 0,-4
--UCAGUGCAUCACAGAACU

U------------------ 112 0,-3
--UCAGUGCAUCACAGAACU
UU----------------- 528 0,-2
--UCAGUGCAUCACAGAACU
UUa---------------- 3 0,-2
--UCAGUGCAUCACAGAACU
UUu---------------- 6 0,-2
--UCAGUGCAUCACAGAACU
UUG---------------- 126 0,-1
--UCAGUGCAUCACAGAACU
UUGa--------------- 92 0,-1
--UCAGUGCAUCACAGAACU
UUGaa-------------- 8 0,-1
--UCAGUGCAUCACAGAACU
UUGU--------------- 1014 0,0
--UCAGUGCAUCACAGAACU
UUGUa-------------- 37 0,0
--UCAGUGCAUCACAGAACU
UUGUaa------------- 11 0,0
--UCAGUGCAUCACAGAACU
UUGUau------------- 11 0,0
--UCAGUGCAUCACAGAACU
UUGUu-------------- 370 0,0
--UCAGUGCAUCACAGAACU
UUGUuu------------- 33 0,0
--UCAGUGCAUCACAGAACU
UUGUC-------------- 2 0,1
---CAGUGCAUCACAGAACU
UUGUuu------------- 2 1,0
--AGUGCAUCACAGAACU
UU----------------- 9 2,-2
--AGUGCAUCACAGAACU
UUGa--------------- 2 2,-1
--AGUGCAUCACAGAACU
UUGU--------------- 18 2,0
--AGUGCAUCACAGAACU
UUGUu-------------- 12 2,0

>hsa-mir-150 MA:16-37;mi:51-72 SRX018958
cuccccauggcccugUCUCCCAACCCUUGUACCAGUGcugggcucagaccCUGGUACAGGCCUGGGGGACAGggaccugg
ggac
.(((((.((((((((((((((..(((.(((((((.((((....))).).))))))))))..))))))))))))).)))))
))).
---------------UCUCCCAACCCUUGUACC--
----	11	0,-4
---------------UCUCCCAACCCUUGUACCA---
----	41	0,-3
---------------UCUCCCAACCCUUGUACCAG--
----	15	0,-2
---------------UCUCCCAACCCUUGUACCAGU---
----	61	0,-1
---------------UCUCCCAACCCUUGUACCAGUa--
----	8	0,-1
---------------UCUCCCAACCCUUGUACCAGUaa---
----	2	0,-1
---------------UCUCCCAACCCUUGUACCAGUG--
----	170	0,0
---------------UCUCCCAACCCUUGUACCAGUGa---
----	86	0,0
---------------UCUCCCAACCCUUGUACCAGUGaa--
----	2	0,0
---------------UCUCCCAACCCUUGUACCAGUGu---
----	30	0,0

>hsa-mir-151 5p:11-31;3p:47-67 SRX018958
uuuccugcccUCGAGGAGCUCACAGUCUAGUaugucucauccccuaCUAGACUGAAGCUCCUUGAGGacagggaugguca
uacucaccuc
.(((((.((((((((((((..((((((((((.(........).)))))))))).)))))))))))).))))).......
....
---------CUCGAGGAGCUCACAGUCUA--
----------	5	-1,-2
---------CUCGAGGAGCUCACAGUCUAG---
----------	9	-1,-1
---------CUCGAGGAGCUCACAGUCUAGU--
----------	63	-1,0
---------CUCGAGGAGCUCACAGUCUAGUA---

---------- 15 -1,1

---------CUCGAGGAGCUCACAGUCUAGUAU--

---------- 7 -1,2

----------UCGAGGAGCUCACAGUCU---

---------- 4 0,-3

----------UCGAGGAGCUCACAGUCUA--

---------- 145 0,-2

----------UCGAGGAGCUCACAGUCUAuu--

---------- 2 0,-2

----------UCGAGGAGCUCACAGUCUAG---

---------- 120 0,-1

----------UCGAGGAGCUCACAGUCUAGa--

---------- 18 0,-1

----------UCGAGGAGCUCACAGUCUAGaa---

---------- 10 0,-1

----------UCGAGGAGCUCACAGUCUAGU--

---------- 944 0,0

----------UCGAGGAGCUCACAGUCUAGUu---

---------- 5 0,0

----------UCGAGGAGCUCACAGUCUAGUA---

---------- 479 0,1

----------UCGAGGAGCUCACAGUCUAGUAa--

---------- 112 0,1

----------UCGAGGAGCUCACAGUCUAGUAaa---

---------- 5 0,1

----------UCGAGGAGCUCACAGUCUAGUAU--

---------- 71 0,2

----------UCGAGGAGCUCACAGUCUAGUAUa---

---------- 4 0,2

----------UCGAGGAGCUCACAGUCUAGUAUG---

---------- 2 0,3

-----------CGAGGAGCUCACAGUCUAG---

---------- 2 1,-1

-----------CGAGGAGCUCACAGUCUAGUA---

---------- 2 1,1

-----------CGAGGAGCUCACAGUCUAGUAU--

---------- 2 1,2

-----------CGAGGAGCUCACAGUCUAGUAUG---

---------- 2 1,3

------------GAGGAGCUCACAGUCUAGU--

---------- 30 2,0

------------GAGGAGCUCACAGUCUAGUA---

---------- 7 2,1

------------GAGGAGCUCACAGUCUAGUAa--

---------- 2 2,1

------------GAGGAGCUCACAGUCUAGUAU--

---------- 4 2,2

---UACUAGACUGAAGCUCCUUG----------------

---------- 3 -2,-3

---UACUAGACUGAAGCUCCUUGA---------------

---------- 59 -2,-2

---UACUAGACUGAAGCUCCUUGAa--------------

---------- 16 -2,-2

---UACUAGACUGAAGCUCCUUGAuu-------------

---------- 3 -2,-2

---UACUAGACUGAAGCUCCUUGAG--------------

---------- 140 -2,-1

---UACUAGACUGAAGCUCCUUGAGa-------------

---------- 46 -2,-1

---UACUAGACUGAAGCUCCUUGAGaa------------

---------- 2 -2,-1

---UACUAGACUGAAGCUCCUUGAGu-------------

---------- 4 -2,-1

---UACUAGACUGAAGCUCCUUGAGG-------------

---------- 75 -2,0

---UACUAGACUGAAGCUCCUUGAGGu------------

---------- 7 -2,0

---UACUAGACUGAAGCUCCUUGAGGA------------

---------- 22 -2,1

---UACUAGACUGAAGCUCCUUGAGGAa-----------

---------- 4 -2,1

--ACUAGACUGAAGCUCCUUGAG--------------

---------- 17 -1,-1

--ACUAGACUGAAGCUCCUUGAGa-------------

---------- 8 -1,-1

--ACUAGACUGAAGCUCCUUGAGu-------------

--------- 7 -1,-1
--ACUAGACUGAAGCUCCUUGAGG-------------
--------- 26 -1,0
--ACUAGACUGAAGCUCCUUGAGGA------------
--------- 45 -1,1
--ACUAGACUGAAGCUCCUUGAGGAa-----------
--------- 10 -1,1
--ACUAGACUGAAGCUCCUUGAGGAau----------
--------- 3 -1,1
---CUAGACUGAAGCUCCUUG----------------
--------- 6 0,-3
---CUAGACUGAAGCUCCUUGA---------------
--------- 61 0,-2
---CUAGACUGAAGCUCCUUGAa--------------
--------- 22 0,-2
---CUAGACUGAAGCUCCUUGAaa-------------
--------- 14 0,-2
---CUAGACUGAAGCUCCUUGAau-------------
--------- 2 0,-2
---CUAGACUGAAGCUCCUUGAu--------------
--------- 2 0,-2
---CUAGACUGAAGCUCCUUGAG--------------
--------- 422 0,-1
---CUAGACUGAAGCUCCUUGAGa-------------
--------- 109 0,-1
---CUAGACUGAAGCUCCUUGAGaa------------
--------- 48 0,-1
---CUAGACUGAAGCUCCUUGAGau------------
--------- 3 0,-1
---CUAGACUGAAGCUCCUUGAGu-------------
--------- 31 0,-1
---CUAGACUGAAGCUCCUUGAGG-------------
--------- 1305 0,0
---CUAGACUGAAGCUCCUUGAGGu------------
--------- 54 0,0
---CUAGACUGAAGCUCCUUGAGGuu-----------
--------- 6 0,0
---CUAGACUGAAGCUCCUUGAGGA------------

---------- 2449 0,1

--CUAGACUGAAGCUCCUUGAGGAa-----------

---------- 2559 0,1

--CUAGACUGAAGCUCCUUGAGGAaa----------

---------- 1468 0,1

--CUAGACUGAAGCUCCUUGAGGAau----------

---------- 405 0,1

--CUAGACUGAAGCUCCUUGAGGAu-----------

---------- 394 0,1

--CUAGACUGAAGCUCCUUGAGGAuu----------

---------- 54 0,1

--CUAGACUGAAGCUCCUUGAGGAC-----------

---------- 43 0,2

--CUAGACUGAAGCUCCUUGAGGACu----------

---------- 13 0,2

--CUAGACUGAAGCUCCUUGAGGACA----------

---------- 6 0,3

---UAGACUGAAGCUCCUUGAG--------------

---------- 14 1,-1

---UAGACUGAAGCUCCUUGAGa-------------

---------- 3 1,-1

---UAGACUGAAGCUCCUUGAGaa------------

---------- 2 1,-1

---UAGACUGAAGCUCCUUGAGG-------------

---------- 37 1,0

---UAGACUGAAGCUCCUUGAGGu------------

---------- 8 1,0

---UAGACUGAAGCUCCUUGAGGA------------

---------- 70 1,1

---UAGACUGAAGCUCCUUGAGGAa-----------

---------- 76 1,1

---UAGACUGAAGCUCCUUGAGGAaa----------

---------- 47 1,1

---UAGACUGAAGCUCCUUGAGGAau----------

---------- 16 1,1

---UAGACUGAAGCUCCUUGAGGAu-----------

---------- 11 1,1

---UAGACUGAAGCUCCUUGAGGAuu----------

---------- 2 1,1
---UAGACUGAAGCUCCUUGAGGAC-----------
---------- 10 1,2
---UAGACUGAAGCUCCUUGAGGACu----------
---------- 5 1,2
--AGACUGAAGCUCCUUGAGGA------------
---------- 2 2,1
--AGACUGAAGCUCCUUGAGGAC-----------
---------- 8 2,2
--AGACUGAAGCUCCUUGAGGACu----------
---------- 10 2,2
--AGACUGAAGCUCCUUGAGGACA----------
---------- 2 2,3
--AGACUGAAGCUCCUUGAGGACAa---------
---------- 2 2,3

>hsa-mir-152 3p:54-74 SRX018958
uguccccccggcccagguucugugauacacuccgacucgggcucuggagcagUCAGUGCAUGACAGAACUUGGgcccgg
aaggacc
.((((..((.((((((((((((((.((.((((..((((...(((....))))))))))).)).)))))))))))))).))
..)))).
--GUCAGUGCAUGACAGAACUUGG------
------- 4 -1,0
---UCAGUGCAUGACAGAACU---------
------- 902 0,-3
---UCAGUGCAUGACAGAACUU--------
------- 94 0,-2
---UCAGUGCAUGACAGAACUUa-------
------- 3 0,-2
---UCAGUGCAUGACAGAACUUG-------
------- 274 0,-1
---UCAGUGCAUGACAGAACUUGa------
------- 10 0,-1
---UCAGUGCAUGACAGAACUUGaa-----
------- 4 0,-1
---UCAGUGCAUGACAGAACUUGu------
------- 11 0,-1
---UCAGUGCAUGACAGAACUUGuu-----

------- 3 0,-1
--UCAGUGCAUGACAGAACUUGG------
------- 1083 0,0
--UCAGUGCAUGACAGAACUUGGa-----
------- 248 0,0
--UCAGUGCAUGACAGAACUUGGaa----
------- 65 0,0
--UCAGUGCAUGACAGAACUUGGau----
------- 5 0,0
--UCAGUGCAUGACAGAACUUGGu-----
------- 1106 0,0
--UCAGUGCAUGACAGAACUUGGuu----
------- 126 0,0
--UCAGUGCAUGACAGAACUUGGG-----
------- 867 0,1
--UCAGUGCAUGACAGAACUUGGGa----
------- 280 0,1
--UCAGUGCAUGACAGAACUUGGGaa---
------- 90 0,1
--UCAGUGCAUGACAGAACUUGGGau---
------- 13 0,1
--UCAGUGCAUGACAGAACUUGGGu----
------- 665 0,1
--UCAGUGCAUGACAGAACUUGGGuu---
------- 54 0,1
--AGUGCAUGACAGAACUUG-------
------- 5 2,-1
--AGUGCAUGACAGAACUUGG------
------- 22 2,0
--AGUGCAUGACAGAACUUGGa-----
------- 3 2,0
--AGUGCAUGACAGAACUUGGaa----
------- 2 2,0
--AGUGCAUGACAGAACUUGGu-----
------- 15 2,0
--AGUGCAUGACAGAACUUGGuu----
------- 4 2,0
--AGUGCAUGACAGAACUUGGG-----

------- 14 2,1
---AGUGCAUGACAGAACUUGGGa----
------- 7 2,1
---AGUGCAUGACAGAACUUGGGaa---
------- 3 2,1
---AGUGCAUGACAGAACUUGGGu----
------- 10 2,1
---AGUGCAUGACAGAACUUGGGuu---
------- 2 2,1

>hsa-mir-154 MA:15-36;mi:51-72 SRX018958
gugguacuugaagaUAGGUUAUCCGUGUUGCCUUCGcuuuauuugugacgAAUCAUACACGGUUGACCUAUUuuucagua
ccaa
.((((((.(((((((((((((.((((((....(((((.........).))))....)))))).))))))))))))).))))
))).
--------------UAGGUUAUCCGUGUUGCCU---
---- 2 0,-3
--------------UAGGUUAUCCGUGUUGCCUU--
---- 4 0,-2
--------------UAGGUUAUCCGUGUUGCCUUC---
---- 7 0,-1
--------------UAGGUUAUCCGUGUUGCCUUCG--
---- 15 0,0
--AAUCAUACACGGUUGACCUAU---------
---- 3 0,-1
--AAUCAUACACGGUUGACCUAUa--------
---- 2 0,-1
--AAUCAUACACGGUUGACCUAUU--------
---- 24 0,0
--AAUCAUACACGGUUGACCUAUUU-------
---- 4 0,1

>hsa-mir-155 MA:4-26;mi:43-64 SRX018958
cugUUAAUGCUAAUCGUGAUAGGGGUuuuugccuccaacugaCUCCUACAUAUUAGCAUUAACAg
((((((((((((((.(((.((((((((.(((....)))..)))))))))))))))))))))))))
---UUAAUGCUAAUCGUGAUA-- 53 0,-5
---UUAAUGCUAAUCGUGAUAG--- 4 0,-4
---UUAAUGCUAAUCGUGAUAGG-- 2 0,-3

---UUAAUGCUAAUCGUGAUAGGGG-------------------------------------- 3 0,-1
---UUAAUGCUAAUCGUGAUAGGGGU------------------------------------- 4 0,0
---UUAAUGCUAAUCGUGAUAGGGGUU------------------------------------ 3 0,1
----UAAUGCUAAUCGUGAUAGGGGUUU------------------------------------ 3 1,2

>hsa-mir-15a MA:14-35;mi:51-72 SRX018958
ccuuggaguaaagUAGCAGCACAUAAUGGUUUGUGgauuuugaaaaggugCAGGCCAUAUUGUGCUGCCUCAaaaauacaagg
(((((.......(..((((((((..((((((((((..((.....))..))))))))))..))))))))..).......)))))
-------------UAGCAGCACAUAAUGGUU-- 104 0,-4
-------------UAGCAGCACAUAAUGGUUU--- 1039 0,-3
-------------UAGCAGCACAUAAUGGUUUa-- 6 0,-3
-------------UAGCAGCACAUAAUGGUUUaa--- 2 0,-3
-------------UAGCAGCACAUAAUGGUUUu-- 15 0,-3
-------------UAGCAGCACAUAAUGGUUUG-- 490 0,-2
-------------UAGCAGCACAUAAUGGUUUGa--- 2 0,-2
-------------UAGCAGCACAUAAUGGUUUGU--- 389 0,-1
-------------UAGCAGCACAUAAUGGUUUGUa-- 5 0,-1
-------------UAGCAGCACAUAAUGGUUUGUau--- 2 0,-1
-------------UAGCAGCACAUAAUGGUUUGUu-- 2 0,-1
-------------UAGCAGCACAUAAUGGUUUGUG-- 42 0,0
-------------UAGCAGCACAUAAUGGUUUGUGa--- 11 0,0
--------------AGCAGCACAUAAUGGUUU--- 3 1,-3

--------------AGCAGCACAUAAUGGUUUG---
--- 2   1,-2
--------------AGCAGCACAUAAUGGUUUGU--
--- 3   1,-1

>hsa-mir-15b   MA:20-41;mi:58-79   SRX018958
uugaggccuuaaaguacugUAGCAGCACAUCAUGGUUUACAugcuacagucaagaugCGAAUCAUUAUUUGCUGCUCUAg
aaauuuaaggaaauucau
.((((.(((((((...(((.(((((((.((.((((((..(((.((.......))))).))))))).)).))))))).)))
...)))))))...)))).
-------------------UAGCAGCACAUCAUGGUU---
------------------ 13 0,-4
-------------------UAGCAGCACAUCAUGGUUU--
------------------ 85 0,-3
-------------------UAGCAGCACAUCAUGGUUUu---
------------------ 14 0,-3
-------------------UAGCAGCACAUCAUGGUUUA---
------------------ 352 0,-2
-------------------UAGCAGCACAUCAUGGUUUAa--
------------------ 19 0,-2
-------------------UAGCAGCACAUCAUGGUUUAu--
------------------ 8   0,-2
-------------------UAGCAGCACAUCAUGGUUUAC--
------------------ 172 0,-1
-------------------UAGCAGCACAUCAUGGUUUACu---------------------------------------
------------------ 2   0,-1
-------------------UAGCAGCACAUCAUGGUUUACA---------------------------------------
------------------ 463 0,0
-------------------UAGCAGCACAUCAUGGUUUACAa--------------------------------------
------------------ 13 0,0
-------------------UAGCAGCACAUCAUGGUUUACAU--------------------------------------
------------------ 5   0,1
--------------------AGCAGCACAUCAUGGUUUA---
------------------ 2   1,-2
--------------------AGCAGCACAUCAUGGUUUAC--
------------------ 4   1,-1
---------------------GCAGCACAUCAUGGUUUACA---------------------------------------
------------------ 2   2,0

---CGAAUCAUUAUUUGCUGCUCUAu
------------------ 3 0,0

>hsa-mir-16-1 MA:14-35;mi:56-77 SRX018958
gucagcagugccuUAGCAGCACGUAAAUAUUGGCGuuaagauucuaaaauuaucuCCAGUAUUAACUGUGCUGCUGAagu
aagguugac
((((((..(((.((((((((((((.((((((((.(.(((..........))).).)))))))).)).)))))))))).))
)..))))))
-------------UAGCAGCACGUAAAUAUU---
--------- 331 0,-4
-------------UAGCAGCACGUAAAUAUUa--
--------- 17 0,-4
-------------UAGCAGCACGUAAAUAUUaa---
--------- 3 0,-4
-------------UAGCAGCACGUAAAUAUUu--
--------- 2 0,-4
-------------UAGCAGCACGUAAAUAUUG--
--------- 1125 0,-3
-------------UAGCAGCACGUAAAUAUUGa---
--------- 11 0,-3
-------------UAGCAGCACGUAAAUAUUGaa--
--------- 9 0,-3
-------------UAGCAGCACGUAAAUAUUGu---
--------- 5 0,-3
-------------UAGCAGCACGUAAAUAUUGG---
--------- 1720 0,-2
-------------UAGCAGCACGUAAAUAUUGGa--
--------- 35 0,-2
-------------UAGCAGCACGUAAAUAUUGGaa---
--------- 16 0,-2
-------------UAGCAGCACGUAAAUAUUGGu--
--------- 12 0,-2
-------------UAGCAGCACGUAAAUAUUGGC--
--------- 661 0,-1
-------------UAGCAGCACGUAAAUAUUGGCa---
--------- 247 0,-1
-------------UAGCAGCACGUAAAUAUUGGCaa--
--------- 146 0,-1

-------------UAGCAGCACGUAAAUAUUGGCau--

--------- 72 0,-1

-------------UAGCAGCACGUAAAUAUUGGCu---

--------- 12 0,-1

-------------UAGCAGCACGUAAAUAUUGGCG---

--------- 3076 0,0

-------------UAGCAGCACGUAAAUAUUGGCGa--

--------- 98 0,0

-------------UAGCAGCACGUAAAUAUUGGCGaa---

--------- 20 0,0

-------------UAGCAGCACGUAAAUAUUGGCGau---

--------- 27 0,0

-------------UAGCAGCACGUAAAUAUUGGCGU--

--------- 519 0,1

-------------UAGCAGCACGUAAAUAUUGGCGUU---

--------- 42 0,2

-------------UAGCAGCACGUAAAUAUUGGCGUUu--

--------- 9 0,2

--------------AGCAGCACGUAAAUAUUG--

--------- 8 1,-3

--------------AGCAGCACGUAAAUAUUGG---

--------- 7 1,-2

--------------AGCAGCACGUAAAUAUUGGC--

--------- 2 1,-1

--------------AGCAGCACGUAAAUAUUGGCa---

--------- 2 1,-1

--------------AGCAGCACGUAAAUAUUGGCuu--

--------- 2 1,-1

--------------AGCAGCACGUAAAUAUUGGCG---

--------- 37 1,0

--------------AGCAGCACGUAAAUAUUGGCGU--

--------- 5 1,1

--------------AGCAGCACGUAAAUAUUGGCGUU---

--------- 2 1,2

---------------GCAGCACGUAAAUAUUGGCG---

--------- 4 2,0

---CCAGUAUUAACUGUGCUGCU-----

--------- 2 0,-2

>hsa-mir-16-2   MA:10-31;mi:53-74   SRX018958
guuccacucUAGCAGCACGUAAAUAUUGGCGuagugaaauauauauuaaacaCCAAUAUUACUGUGCUGCUUUAguguga
c
((..((((..(((((((((..((((((((.((..(((........))).)).))))))))..)))))))))..))))..)
)
--------CUAGCAGCACGUAAAUAUU---
-   2   -1,-4
--------CUAGCAGCACGUAAAUAUUG--
-   5   -1,-3
--------CUAGCAGCACGUAAAUAUUGG---
-   4   -1,-2
--------CUAGCAGCACGUAAAUAUUGGC--
-   2   -1,-1
--------CUAGCAGCACGUAAAUAUUGGCG---
-   4   -1,0
---------UAGCAGCACGUAAAUAUU---
-   331 0,-4
---------UAGCAGCACGUAAAUAUUa--
-   17 0,-4
---------UAGCAGCACGUAAAUAUUaa---
-   3   0,-4
---------UAGCAGCACGUAAAUAUUu--
-   2   0,-4
---------UAGCAGCACGUAAAUAUUG--
-   1125   0,-3
---------UAGCAGCACGUAAAUAUUGa---
-   11 0,-3
---------UAGCAGCACGUAAAUAUUGaa--
-   9   0,-3
---------UAGCAGCACGUAAAUAUUGu---
-   5   0,-3
---------UAGCAGCACGUAAAUAUUGG---
-   1720   0,-2
---------UAGCAGCACGUAAAUAUUGGa--
-   35 0,-2
---------UAGCAGCACGUAAAUAUUGGaa---
-   16 0,-2

---------UAGCAGCACGUAAAUAUUGGu--
-    12   0,-2
---------UAGCAGCACGUAAAUAUUGGC--
-    661 0,-1
---------UAGCAGCACGUAAAUAUUGGCa---
-    247 0,-1
---------UAGCAGCACGUAAAUAUUGGCaa--
-    146 0,-1
---------UAGCAGCACGUAAAUAUUGGCau--
-    72   0,-1
---------UAGCAGCACGUAAAUAUUGGCu---
-    12   0,-1
---------UAGCAGCACGUAAAUAUUGGCG---
-    3076   0,0
---------UAGCAGCACGUAAAUAUUGGCGa--
-    98   0,0
---------UAGCAGCACGUAAAUAUUGGCGaa---
-    20   0,0
---------UAGCAGCACGUAAAUAUUGGCGau---
-    27   0,0
---------UAGCAGCACGUAAAUAUUGGCGU--
-    519 0,1
---------UAGCAGCACGUAAAUAUUGGCGUA---
-    8   0,2
---------UAGCAGCACGUAAAUAUUGGCGUAu--
-    2   0,2
----------AGCAGCACGUAAAUAUUG--
-    8   1,-3
----------AGCAGCACGUAAAUAUUGG---
-    7   1,-2
----------AGCAGCACGUAAAUAUUGGC--
-    2   1,-1
----------AGCAGCACGUAAAUAUUGGCa---
-    2   1,-1
----------AGCAGCACGUAAAUAUUGGCuu--
-    2   1,-1
----------AGCAGCACGUAAAUAUUGGCG---
-    37   1,0

```
----------AGCAGCACGUAAAUAUUGGCGU------------------------------------------------
-    5    1,1
-----------GCAGCACGUAAAUAUUGGCG-------------------------------------------------
-    4    2,0
---------------------------------------------------ACCAAUAUUACUGUGCUGCU---------
-    8    -1,-3
---------------------------------------------------ACCAAUAUUACUGUGCUGCUU--------
-    20   -1,-2
---------------------------------------------------ACCAAUAUUACUGUGCUGCUUU-------
-    96   -1,-1
---------------------------------------------------ACCAAUAUUACUGUGCUGCUUUu------
-    25   -1,-1
---------------------------------------------------ACCAAUAUUACUGUGCUGCUUUA------
-    15   -1,0
---------------------------------------------------ACCAAUAUUACUGUGCUGCUUUAu-----
-    4    -1,0
----------------------------------------------------CCAAUAUUACUGUGCUGCUUU-------
-    2    0,-1

>hsa-mir-17 MA:14-36;mi:51-72   SRX018958
gucagaauaauguCAAAGUGCUUACAGUGCAGGUAGugauaugugcaucuACUGCAGUGAAGGCACUUGUAGcauuaugg
ugac
((((..(((((((..((((((((.((.(((((.(((((.......)).)))))))).)).))))))))...)))))))..
))))
------------UCAAAGUGCUUACAGUGCAG------------------------------------------------
----     10  -1,-4
------------UCAAAGUGCUUACAGUGCAGGU----------------------------------------------
----     8   -1,-2
------------UCAAAGUGCUUACAGUGCAGGUAa--------------------------------------------
----     2   -1,-1
------------UCAAAGUGCUUACAGUGCAGGUAu--------------------------------------------
----     2   -1,-1
-------------CAAAGUGCUUACAGUGCA-------------------------------------------------
----     40  0,-5
-------------CAAAGUGCUUACAGUGCAG------------------------------------------------
----     268 0,-4
-------------CAAAGUGCUUACAGUGCAGG-----------------------------------------------
----     217 0,-3
```

-------------CAAAGUGCUUACAGUGCAGGa---
---- 4 0,-3
-------------CAAAGUGCUUACAGUGCAGGU---
---- 140 0,-2
-------------CAAAGUGCUUACAGUGCAGGUu--
---- 3 0,-2
-------------CAAAGUGCUUACAGUGCAGGUuu---
---- 4 0,-2
-------------CAAAGUGCUUACAGUGCAGGUA--
---- 204 0,-1
-------------CAAAGUGCUUACAGUGCAGGUAa---
---- 56 0,-1
-------------CAAAGUGCUUACAGUGCAGGUAau--
---- 3 0,-1
-------------CAAAGUGCUUACAGUGCAGGUAu---
---- 80 0,-1
-------------CAAAGUGCUUACAGUGCAGGUAuu--
---- 5 0,-1
-------------CAAAGUGCUUACAGUGCAGGUAG---
---- 270 0,0
-------------CAAAGUGCUUACAGUGCAGGUAGa--
---- 2 0,0
-------------CAAAGUGCUUACAGUGCAGGUAGU--
---- 16 0,1
--------------AAAGUGCUUACAGUGCAGG--
---- 8 1,-3
--------------AAAGUGCUUACAGUGCAGGU---
---- 8 1,-2
--------------AAAGUGCUUACAGUGCAGGUA--
---- 7 1,-1
--------------AAAGUGCUUACAGUGCAGGUAa---
---- 6 1,-1
--------------AAAGUGCUUACAGUGCAGGUAu---
---- 2 1,-1
--------------AAAGUGCUUACAGUGCAGGUAG---
---- 2 1,0
--------------AAAGUGCUUACAGUGCAGGUAGa--
---- 3 1,0

---UACUGCAGUGAAGGCACUUGU----------
----   3   -1,-2
--ACUGCAGUGAAGGCACUU------------
----   243 0,-4
--ACUGCAGUGAAGGCACUUuu----------
----   2   0,-4
--ACUGCAGUGAAGGCACUUG-----------
----   443 0,-3
--ACUGCAGUGAAGGCACUUGU----------
----   768 0,-2
--ACUGCAGUGAAGGCACUUGUu---------
----   2   0,-2
--ACUGCAGUGAAGGCACUUGUA---------
----   73   0,-1
--ACUGCAGUGAAGGCACUUGUAa--------
----   7   0,-1
--ACUGCAGUGAAGGCACUUGUAu--------
----   9   0,-1
--ACUGCAGUGAAGGCACUUGUAG--------
----   25   0,0
--ACUGCAGUGAAGGCACUUGUAGa-------
----   3   0,0
--ACUGCAGUGAAGGCACUUGUAGaa------
----   3   0,0
--ACUGCAGUGAAGGCACUUGUAGC-------
----   5   0,1
--ACUGCAGUGAAGGCACUUGUAGCu------
----   5   0,1
--ACUGCAGUGAAGGCACUUGUAGCA------
----   5   0,2
---CUGCAGUGAAGGCACUUG-----------
----   5   1,-3
---CUGCAGUGAAGGCACUUGU----------
----   12   1,-2
---CUGCAGUGAAGGCACUUGUA---------
----   7   1,-1
---CUGCAGUGAAGGCACUUGUAG--------
----   5   1,0

---CUGCAGUGAAGGCACUUGUAGa-------
----   2   1,0
---CUGCAGUGAAGGCACUUGUAGCu------
----   2   1,1
--UGCAGUGAAGGCACUUGU----------
----   9   2,-2
--UGCAGUGAAGGCACUUGUA---------
----   2   2,-1

>hsa-mir-181a-1 MA:24-46;mi:64-85   SRX018958
ugaguuuugagguugcuucagugAACAUUCAACGCUGUCGGUGAGUuuggaauuaaaaucaaaACCAUCGACCGUUGAUU
GUACCcuauggcuaaccaucaucuacucca
.((((..((((((((((..((.(.(((.((((((..(((((((.((((.((.......)).))))))))))))))))).)
)).).))..))).)))).)))...))))..
-----------------------AACAUUCAACGCUGUCGG---------------------------------------
------------------------------ 98 0,-5
-----------------------AACAUUCAACGCUGUCGGU--------------------------------------
------------------------------ 5   0,-4
-----------------------AACAUUCAACGCUGUCGGUaa------------------------------------
------------------------------ 9   0,-4
-----------------------AACAUUCAACGCUGUCGGUau------------------------------------
------------------------------ 2   0,-4
-----------------------AACAUUCAACGCUGUCGGUG-------------------------------------
------------------------------ 13 0,-3
-----------------------AACAUUCAACGCUGUCGGUGu------------------------------------
------------------------------ 4   0,-3
-----------------------AACAUUCAACGCUGUCGGUGA------------------------------------
------------------------------ 120 0,-2
-----------------------AACAUUCAACGCUGUCGGUGAa-----------------------------------
------------------------------ 2   0,-2
-----------------------AACAUUCAACGCUGUCGGUGAu-----------------------------------
------------------------------ 2   0,-2
-----------------------AACAUUCAACGCUGUCGGUGAG-----------------------------------
------------------------------ 600 0,-1
-----------------------AACAUUCAACGCUGUCGGUGAGa----------------------------------
------------------------------ 9   0,-1
-----------------------AACAUUCAACGCUGUCGGUGAGU----------------------------------
------------------------------ 73 0,0

-----------------------AACAUUCAACGCUGUCGGUGAGUa---------------------------------
------------------------------ 9 0,0
-----------------------AACAUUCAACGCUGUCGGUGAGUau--------------------------------
------------------------------ 2 0,0
-----------------------AACAUUCAACGCUGUCGGUGAGUU---------------------------------
------------------------------ 116 0,1
-----------------------AACAUUCAACGCUGUCGGUGAGUUa--------------------------------
------------------------------ 5 0,1
-----------------------AACAUUCAACGCUGUCGGUGAGUUU--------------------------------
------------------------------ 49 0,2
------------------------ACAUUCAACGCUGUCGGUGAGUU---------------------------------
------------------------------ 4 1,1
---AAACCAUCGACCGUUGAUU
------------------------------ 5 -2,-5
---ACCAUCGACCGUUGAUU
G----------------------------- 14 0,-4
---ACCAUCGACCGUUGAUU
GU---------------------------- 4 0,-3
---ACCAUCGACCGUUGAUU
GUA--------------------------- 2 0,-2
---ACCAUCGACCGUUGAUU
GUAa-------------------------- 2 0,-2
---ACCAUCGACCGUUGAUU
GUAC-------------------------- 3 0,-1
---ACCAUCGACCGUUGAUU
GUACu------------------------- 2 0,-1
---ACCAUCGACCGUUGAUU
GUACC------------------------- 13 0,0

>hsa-mir-181a-2 MA:39-61;mi:77-98 SRX018958
agaagggcuaucaggccagccuucagaggacuccaaggAACAUUCAACGCUGUCGGUGAGUuugggauuugaaaaaACCA
CUGACCGUUGACUGUACCuuggggguccuua
.........................(((((((((((((.(((.((((((..(((((((.((((...........))))))
))))))))))).))).))))))))))))).
--------------------------------------AACAUUCAACGCUGUCGG------------------------
------------------------------ 98 0,-5
--------------------------------------AACAUUCAACGCUGUCGGU-----------------------
------------------------------ 5 0,-4

------------------------------------AACAUUCAACGCUGUCGGUaa--------------------
----------------------------- 9 0,-4
------------------------------------AACAUUCAACGCUGUCGGUau--------------------
----------------------------- 2 0,-4
------------------------------------AACAUUCAACGCUGUCGGUG---------------------
----------------------------- 13 0,-3
------------------------------------AACAUUCAACGCUGUCGGUGu--------------------
----------------------------- 4 0,-3
------------------------------------AACAUUCAACGCUGUCGGUGA--------------------
----------------------------- 120 0,-2
------------------------------------AACAUUCAACGCUGUCGGUGAa-------------------
----------------------------- 2 0,-2
------------------------------------AACAUUCAACGCUGUCGGUGAu-------------------
----------------------------- 2 0,-2
------------------------------------AACAUUCAACGCUGUCGGUGAG-------------------
----------------------------- 600 0,-1
------------------------------------AACAUUCAACGCUGUCGGUGAGa------------------
----------------------------- 9 0,-1
------------------------------------AACAUUCAACGCUGUCGGUGAGU------------------
----------------------------- 73 0,0
------------------------------------AACAUUCAACGCUGUCGGUGAGUa-----------------
----------------------------- 9 0,0
------------------------------------AACAUUCAACGCUGUCGGUGAGUau----------------
----------------------------- 2 0,0
------------------------------------AACAUUCAACGCUGUCGGUGAGUU-----------------
----------------------------- 116 0,1
------------------------------------AACAUUCAACGCUGUCGGUGAGUUa----------------
----------------------------- 5 0,1
------------------------------------AACAUUCAACGCUGUCGGUGAGUUU----------------
----------------------------- 49 0,2
-------------------------------------ACAUUCAACGCUGUCGGUGAGUU-----------------
----------------------------- 4 1,1
--AACCA
CUGACCGUUGACU---------------- 5 -1,-5
---ACCA
CUGACCGUUGACUG--------------- 33 0,-4
---ACCA
CUGACCGUUGACUGU-------------- 7 0,-3

--ACCA
CUGACCGUUGACUGUA-------------- 10 0,-2
--ACCA
CUGACCGUUGACUGUAC------------- 14 0,-1
--ACCA
CUGACCGUUGACUGUACu------------ 5   0,-1
--ACCA
CUGACCGUUGACUGUACC----------- 22 0,0
--ACCA
CUGACCGUUGACUGUACCU---------- 3   0,1

>hsa-mir-181b-1 5p:36-58   SRX018958
ccugugcagagauuauuuuuuaaaaggucacaaucAACAUUCAUUGCUGUCGGUGGGUugaacuguguggacaagcucac
ugaacaaugaaugcaacuguggccccgcuu
.........................(((((((.....(((((((((...((((((((((...((....))...)))))))
))).)))))))))....)))))))......
-----------------------------------AACAUUCAUUGCUGUCGG---------------------------
------------------------------ 35 0,-5
-----------------------------------AACAUUCAUUGCUGUCGGU--------------------------
------------------------------ 3   0,-4
-----------------------------------AACAUUCAUUGCUGUCGGUG-------------------------
------------------------------ 24 0,-3
-----------------------------------AACAUUCAUUGCUGUCGGUGGG-----------------------
------------------------------ 79 0,-1
-----------------------------------AACAUUCAUUGCUGUCGGUGGGU----------------------
------------------------------ 10 0,0
-----------------------------------AACAUUCAUUGCUGUCGGUGGGUaa--------------------
------------------------------ 3   0,0
-----------------------------------AACAUUCAUUGCUGUCGGUGGGUau--------------------
------------------------------ 13 0,0
-----------------------------------AACAUUCAUUGCUGUCGGUGGGUU---------------------
------------------------------ 25 0,1
-----------------------------------AACAUUCAUUGCUGUCGGUGGGUUa--------------------
------------------------------ 9   0,1
------------------------------------ACAUUCAUUGCUGUCGGUGGGUU---------------------
------------------------------ 3   1,1
---CUCAC
UGAACAAUGAAUGCA--------------- 2

---CUCAC
UGAACAAUGAAUGCAuu------------- 3
---CUCAC
UGAACAAUGAAUGCAA-------------- 2

>hsa-mir-181b-2 5p:16-38   SRX018958
cugauggcugcacucAACAUUCAUUGCUGUCGGUGGGUuugagucugaaucaacucacugaucaaugaaugcaaacugcg
gaccaaaca
....((((((((.....(((((((((..(((((((((((.((.......)))))))))))))))))))))).....))))
).)))....
---------------AACAUUCAUUGCUGUCGG---
---------   35 0,-5
---------------AACAUUCAUUGCUGUCGGU--
---------   3   0,-4
---------------AACAUUCAUUGCUGUCGGUG---
---------   24 0,-3
---------------AACAUUCAUUGCUGUCGGUGGG---
---------   79 0,-1
---------------AACAUUCAUUGCUGUCGGUGGGU--
---------   10 0,0
---------------AACAUUCAUUGCUGUCGGUGGGUaa--
---------   3   0,0
---------------AACAUUCAUUGCUGUCGGUGGGUau--
---------   13 0,0
---------------AACAUUCAUUGCUGUCGGUGGGUU---
---------   25 0,1
---------------AACAUUCAUUGCUGUCGGUGGGUUa--
---------   9   0,1
---------------AACAUUCAUUGCUGUCGGUGGGUUU--
---------   10 0,2
----------------ACAUUCAUUGCUGUCGGUGGGUU---
---------   3   1,1

>hsa-mir-181c   MA:27-48;mi:65-86   SRX018958
cggaaaauuugccaaggguuugggggAACAUUCAACCUGUCGGUGAGUuugggcagcucaggcaAACCAUCGACCGUUGA
GUGGACccugaggccuggaauugccauccu
.(((.......(((.((.((..(((...((((((((..((((((.((((((...........)))))))))))).)))))
)))..)))..)).)))))........))).

--------------------------AACAUUCAACCUGUCGGUGAG---------------------------------
------------------------------ 13 0,-1
--------------------------AACAUUCAACCUGUCGGUGAGUU-------------------------------
------------------------------ 12 0,1
--------------------------AACAUUCAACCUGUCGGUGAGUUU------------------------------
------------------------------ 6 0,2
---ACCAUCGACCGUUGA
GUG--------------------------- 11 1,-3
---ACCAUCGACCGUUGA
GUGG-------------------------- 2 1,-2

>hsa-mir-181d 5p:36-58 SRX018958
guccccuccccuaggccacagccgaggucacaaucAACAUUCAUUGUUGUCGGUGGGUugugaggacugaggccagaccc
accgggggaugaaugucacuguggcugggccagacacggcuuaaggggaauggggac
(((((((((((((((((...(((..(((((((....(((((((((.(..((((((((((....((.(....))).)))))
)))))).)))))))))...))))))).))).......))))).))))))..))))))
-----------------------------------AACAUUCAUUGUUGUCGGUG-------------------------
--- 5 0,-3
-----------------------------------AACAUUCAUUGUUGUCGGUGG------------------------
--- 2 0,-2
-----------------------------------AACAUUCAUUGUUGUCGGUGGG-----------------------
--- 19 0,-1
-----------------------------------AACAUUCAUUGUUGUCGGUGGGa----------------------
--- 2 0,-1
-----------------------------------AACAUUCAUUGUUGUCGGUGGGau---------------------
--- 2 0,-1
-----------------------------------AACAUUCAUUGUUGUCGGUGGGU----------------------
--- 11 0,0
-----------------------------------AACAUUCAUUGUUGUCGGUGGGUaa--------------------
--- 2 0,0
-----------------------------------AACAUUCAUUGUUGUCGGUGGGUau--------------------
--- 5 0,0
-----------------------------------AACAUUCAUUGUUGUCGGUGGGUU---------------------
--- 17 0,1
-----------------------------------AACAUUCAUUGUUGUCGGUGGGUUu--------------------
--- 3 0,1
------------------------------------ACAUUCAUUGUUGUCGGUGGGUU---------------------
--- 2 1,1

>hsa-mir-182   MA:23-46;mi:67-87   SRX018958
gagcugcuugccucccccgguuUUUGGCAAUGGUAGAACUCACACUggugagguaacaggauccggUGGUUCUAGACUUG
CCAACUAuggggcgaggacucagccggcac
..((((((..((((.((((((..(((((((.(.((((((...((((((.............))))))))))))..))))
))))..)))))).))))....)).)))))..
----------------------UUUGGCAAUGGUAGAACUCA--------------------------------------
------------------------------ 7   0,-4
----------------------UUUGGCAAUGGUAGAACUCACA-------------------------------------
------------------------------ 3   0,-2
----------------------UUUGGCAAUGGUAGAACUCACACU----------------------------------
------------------------------ 7   0,0

>hsa-mir-1827   3p:49-66   SRX018958
ucagcagcacagccuucagccuaaagcaaugagaagccucugaaaggcUGAGGCAGUAGAUUGAAU
((((.((...((((.((((((....((..((((....))))))..)))))))))).)).).)))))..
--UGAGGCAGUAGAUUGAAU 2   0,0

>hsa-mir-183   MA:27-48;mi:66-87   SRX018958
ccgcagagugugacuccuguucugugUAUGGCACUGGUAGAAUUCACUgugaacagucucagucaGUGAAUUACCGAAGG
GCCAUAAacagagcagagacagauccacga
.......(((.((.((((((((((.((((((..(((((..(((((((((((......)))..)))))))))))))....
)))))).))))))))).))....)))))..
--------------------------UAUGGCACUGGUAGAAUUCACU--------------------------------
------------------------------ 5   0,0

>hsa-mir-185   MA:15-36;mi:50-71   SRX018958
agggggcgagggauUGGAGAGAAAGGCAGUUCCUGAugguccccuccccAGGGGCUGGCUUUCCUCUGGUCcuucccucc
ca
.((((.((((((((..(((.(((((.(((((((((..((......)).))))))))).))))))))..)))))))).))))
).
-------------UUGGAGAGAAAGGCAGUUCCU--
-- 5   -1,-2
--------------UGGAGAGAAAGGCAGUUC--
-- 60 0,-4
--------------UGGAGAGAAAGGCAGUUCau--
-- 2   0,-4
--------------UGGAGAGAAAGGCAGUUCC---

-- 127 0,-3
--------------UGGAGAGAAAGGCAGUUCCU---
-- 1942 0,-2
--------------UGGAGAGAAAGGCAGUUCCUa--
-- 2 0,-2
--------------UGGAGAGAAAGGCAGUUCCUaa---
-- 4 0,-2
--------------UGGAGAGAAAGGCAGUUCCUau---
-- 5 0,-2
--------------UGGAGAGAAAGGCAGUUCCUG--
-- 839 0,-1
--------------UGGAGAGAAAGGCAGUUCCUGu---
-- 72 0,-1
--------------UGGAGAGAAAGGCAGUUCCUGuu--
-- 18 0,-1
--------------UGGAGAGAAAGGCAGUUCCUGA---
-- 6750 0,0
--------------UGGAGAGAAAGGCAGUUCCUGAa--
-- 1337 0,0
--------------UGGAGAGAAAGGCAGUUCCUGAaa---
-- 55 0,0
--------------UGGAGAGAAAGGCAGUUCCUGAau---
-- 49 0,0
--------------UGGAGAGAAAGGCAGUUCCUGAU--
-- 1880 0,1
--------------UGGAGAGAAAGGCAGUUCCUGAUa---
-- 123 0,1
--------------UGGAGAGAAAGGCAGUUCCUGAUau--
-- 7 0,1
--------------UGGAGAGAAAGGCAGUUCCUGAUu---
-- 106 0,1
--------------UGGAGAGAAAGGCAGUUCCUGAUuu--
-- 9 0,1
--------------UGGAGAGAAAGGCAGUUCCUGAUG---
-- 6 0,2
---------------GGAGAGAAAGGCAGUUCC--
-- 6 1,-3
---------------GGAGAGAAAGGCAGUUCCU---

--   15   1,-2
---------------GGAGAGAAAGGCAGUUCCUG--
--   6   1,-1
---------------GGAGAGAAAGGCAGUUCCUGA---
--   62   1,0
---------------GGAGAGAAAGGCAGUUCCUGAa--
--   13   1,0
---------------GGAGAGAAAGGCAGUUCCUGAU--
--   6   1,1
---------------GGAGAGAAAGGCAGUUCCUGAUu---
--   2   1,1
----------------GAGAGAAAGGCAGUUCCU---
--   4   2,-2
---CAGGGGCUGGCUUUCCUC--------------
--   4   -1,-5
--AGGGGCUGGCUUUCCUCU-------------
--   23   0,-4
--AGGGGCUGGCUUUCCUCUGGU----------
--   10   0,-1
--AGGGGCUGGCUUUCCUCUGGUaa--------
--   3   0,-1
--AGGGGCUGGCUUUCCUCUGGUau--------
--   2   0,-1

>hsa-mir-186   MA:15-36;mi:54-75   SRX018958
ugcuuguaacuuucCAAAGAAUUCUCCUUUUGGGCUuucugguuuuauuuuaaGCCCAAAGGUGAAUUUUUGGGaaguu
ugagcu
.((((.(((((((((((((((((.(((((.((((((...............))))))))))).))))))))))))))))
))))).
-------------CCAAAGAAUUCUCCUUUUG---
------   2   -1,-4
-------------CCAAAGAAUUCUCCUUUUGGGC--
------   2   -1,-1
-------------CCAAAGAAUUCUCCUUUUGGGCU---
------   10   -1,0
--------------CAAAGAAUUCUCCUUUUG---
------   13   0,-4
--------------CAAAGAAUUCUCCUUUUGG--

------ 21 0,-3
--------------CAAAGAAUUCUCCUUUUGGa---
------ 4 0,-3
--------------CAAAGAAUUCUCCUUUUGGG---
------ 26 0,-2
--------------CAAAGAAUUCUCCUUUUGGGa--
------ 8 0,-2
--------------CAAAGAAUUCUCCUUUUGGGaa---
------ 2 0,-2
--------------CAAAGAAUUCUCCUUUUGGGC--
------ 53 0,-1
--------------CAAAGAAUUCUCCUUUUGGGCa---
------ 74 0,-1
--------------CAAAGAAUUCUCCUUUUGGGCaa--
------ 55 0,-1
--------------CAAAGAAUUCUCCUUUUGGGCau--
------ 192 0,-1
--------------CAAAGAAUUCUCCUUUUGGGCU---
------ 281 0,0
--------------CAAAGAAUUCUCCUUUUGGGCUa--
------ 52 0,0
--------------CAAAGAAUUCUCCUUUUGGGCUaa---
------ 15 0,0
--------------CAAAGAAUUCUCCUUUUGGGCUau---
------ 37 0,0
--------------CAAAGAAUUCUCCUUUUGGGCUU--
------ 578 0,1
--------------CAAAGAAUUCUCCUUUUGGGCUUa---
------ 33 0,1
--------------CAAAGAAUUCUCCUUUUGGGCUUau--
------ 7 0,1
--------------CAAAGAAUUCUCCUUUUGGGCUUU---
------ 36 0,2
---------------AAAGAAUUCUCCUUUUGGG---
------ 3 1,-2
---------------AAAGAAUUCUCCUUUUGGGCa---
------ 3 1,-1
---------------AAAGAAUUCUCCUUUUGGGCaa--

------ 2 1,-1
---------------AAAGAAUUCUCCUUUUGGGCau---
------ 7 1,-1
---------------AAAGAAUUCUCCUUUUGGGCU--
------ 26 1,0
---------------AAAGAAUUCUCCUUUUGGGCUa---
------ 4 1,0
---------------AAAGAAUUCUCCUUUUGGGCUau--
------ 7 1,0
---------------AAAGAAUUCUCCUUUUGGGCUU---
------ 57 1,1
---------------AAAGAAUUCUCCUUUUGGGCUUa--
------ 2 1,1
---------------AAAGAAUUCUCCUUUUGGGCUUU--
------ 3 1,2
---------------AAAGAAUUCUCCUUUUGGGCUUUa---
------ 2 1,2

>hsa-mir-187 mi:35-56;MA:71-92 SRX018958
ggucgggcucaccaugacacagugugagaccucgGGCUACAACACAGGACCCGGGCgcugcucugaccccUCGUGUCUUG
UGUUGCAGCCGGagggacgcagguccgca
.(.((((((............((((...((((.((((.((((((((((.((((.((......).).)).)).))))))
))))).)))).)))).)).))))))))).
--UCGUGUCUUG
UGUUGCAGCCGG---------------- 8 0,0
--UCGUGUCUUG
UGUUGCAGCCGGu--------------- 15 0,0
--UCGUGUCUUG
UGUUGCAGCCGGuu-------------- 6 0,0

>hsa-mir-188 5p:15-35;3p:54-74 SRX018958
ugcucccucucucaCAUCCCUUGCAUGGUGGAGGGugagcuuucugaaaacccCUCCCACAUGCAGGGUUUGCAggaugg
cgagcc
.((((((..(((..((..(((((((((..((((((....(.....).....))))))..)))))))))..))..))).))
.)))).
--------------CAUCCCUUGCAUGGUGGAG---
------ 2 0,-2
--------------CAUCCCUUGCAUGGUGGAGGGUu---

------ 2 0,1

>hsa-mir-18a MA:6-28;mi:47-69 SRX018958
uguucUAAGGUGCAUCUAGUGCAGAUAGugaaguagauuagcaucuACUGCCCUAAGUGCUCCUUCUGGca
.(((..((((.(((..(((.((((.(((((..(.....)..)).))))))).)))..))).))))..))).
-----UAAGGUGCAUCUAGUGCA-- 3 0,-5
-----UAAGGUGCAUCUAGUGCAGA-- 7 0,-3
-----UAAGGUGCAUCUAGUGCAGAU--- 35 0,-2
-----UAAGGUGCAUCUAGUGCAGAUA-- 20 0,-1
------AAGGUGCAUCUAGUGCAGAUAG--- 3 1,0
-------AGGUGCAUCUAGUGCAGAU--- 2 2,-2
--ACUGCCCUAAGUGCUCCUUCU---- 2 0,-2

>hsa-mir-18b MA:6-28;mi:49-70 SRX018958
uguguUAAGGUGCAUCUAGUGCAGUUAGugaagcagcuuagaaucuacUGCCCUAAAUGCCCCUUCUGGCa
..((((((((.((((.(((.(((((..((.(((...)))...))..))))).))).)))).)))..)))))
-----UAAGGUGCAUCUAGUGCA-- 3 0,-5

>hsa-mir-190 5p:15-36 SRX018958
ugcaggccucugugUGAUAUGUUUGAUAUAUUAGGUuguuauuuaauccaacuauauaucaaacauauuccuacagugucuugcc
.((((.(.(((((..(((((((((((((((..(((((.........))))))))))))))))))))..).)))).).)
)))).
---------------GAUAUGUUUGAUAUAUUAGGUU---
----- 3 1,1
---ACUAUAUAUCAAACAUAUUCCU--------
----- 5

>hsa-mir-191 MA:16-38;mi:58-79 SRX018958
cggcuggacagcgggCAACGGAAUCCCAAAAGCAGCUGuugucuccagagcauuccaGCUGCGCUUGGAUUUCGUCCCCugcucuccugccu
.(((.(((.((((((..((((((((.((..(((((((..((((....)).))..)))))))..))))))))))..)))
))).))).))).
---------------CAACGGAAUCCCAAAAGC---
----------- 340 0,-5
---------------CAACGGAAUCCCAAAAGCu--
----------- 5 0,-5
---------------CAACGGAAUCCCAAAAGCA--

------------ 1133 0,-4

---------------CAACGGAAUCCCAAAAGCAa---

------------ 20 0,-4

---------------CAACGGAAUCCCAAAAGCAu---

------------ 7 0,-4

---------------CAACGGAAUCCCAAAAGCAuu--

------------ 2 0,-4

---------------CAACGGAAUCCCAAAAGCAG---

------------ 4179 0,-3

---------------CAACGGAAUCCCAAAAGCAGa--

------------ 21 0,-3

---------------CAACGGAAUCCCAAAAGCAGau---

------------ 22 0,-3

---------------CAACGGAAUCCCAAAAGCAGu--

------------ 22 0,-3

---------------CAACGGAAUCCCAAAAGCAGuu---

------------ 36 0,-3

---------------CAACGGAAUCCCAAAAGCAGC--

------------ 13436 0,-2

---------------CAACGGAAUCCCAAAAGCAGCa---

------------ 180 0,-2

---------------CAACGGAAUCCCAAAAGCAGCaa--

------------ 29 0,-2

---------------CAACGGAAUCCCAAAAGCAGCau--

------------ 11 0,-2

---------------CAACGGAAUCCCAAAAGCAGCU---

------------ 27534 0,-1

---------------CAACGGAAUCCCAAAAGCAGCUa--

------------ 392 0,-1

---------------CAACGGAAUCCCAAAAGCAGCUaa---

------------ 46 0,-1

---------------CAACGGAAUCCCAAAAGCAGCUau---

------------ 168 0,-1

---------------CAACGGAAUCCCAAAAGCAGCUu--

------------ 513 0,-1

---------------CAACGGAAUCCCAAAAGCAGCUuu---

------------ 187 0,-1

---------------CAACGGAAUCCCAAAAGCAGCUG--

-----------	3702	0,0

---------------CAACGGAAUCCCAAAAGCAGCUGa---

-----------	251	0,0

---------------CAACGGAAUCCCAAAAGCAGCUGaa--

-----------	13	0,0

---------------CAACGGAAUCCCAAAAGCAGCUGau--

-----------	3	0,0

---------------CAACGGAAUCCCAAAAGCAGCUGU---

-----------	2358	0,1

---------------CAACGGAAUCCCAAAAGCAGCUGUa--

-----------	22	0,1

---------------CAACGGAAUCCCAAAAGCAGCUGUU--

-----------	105	0,2

----------------AACGGAAUCCCAAAAGCA--

-----------	128	1,-4

----------------AACGGAAUCCCAAAAGCAG---

-----------	506	1,-3

----------------AACGGAAUCCCAAAAGCAGu--

-----------	6	1,-3

----------------AACGGAAUCCCAAAAGCAGuu---

-----------	2	1,-3

----------------AACGGAAUCCCAAAAGCAGC--

-----------	1653	1,-2

----------------AACGGAAUCCCAAAAGCAGCa---

-----------	12	1,-2

----------------AACGGAAUCCCAAAAGCAGCU---

-----------	3669	1,-1

----------------AACGGAAUCCCAAAAGCAGCUa--

-----------	29	1,-1

----------------AACGGAAUCCCAAAAGCAGCUaa---

-----------	6	1,-1

----------------AACGGAAUCCCAAAAGCAGCUau---

-----------	38	1,-1

----------------AACGGAAUCCCAAAAGCAGCUu--

-----------	35	1,-1

----------------AACGGAAUCCCAAAAGCAGCUuu---

-----------	23	1,-1

----------------AACGGAAUCCCAAAAGCAGCUG--

----------- 530 1,0
----------------AACGGAAUCCCAAAAGCAGCUGa---------------------------------------
----------- 89 1,0
----------------AACGGAAUCCCAAAAGCAGCUGaa--------------------------------------
----------- 3 1,0
----------------AACGGAAUCCCAAAAGCAGCUGU---------------------------------------
----------- 697 1,1
----------------AACGGAAUCCCAAAAGCAGCUGUa--------------------------------------
----------- 6 1,1
----------------AACGGAAUCCCAAAAGCAGCUGUau-------------------------------------
----------- 5 1,1
----------------AACGGAAUCCCAAAAGCAGCUGUU--------------------------------------
----------- 27 1,2
----------------AACGGAAUCCCAAAAGCAGCUGUUa-------------------------------------
----------- 4 1,2
----------------AACGGAAUCCCAAAAGCAGCUGUUu-------------------------------------
----------- 5 1,2
-----------------ACGGAAUCCCAAAAGCAG---
----------- 2 2,-3
-----------------ACGGAAUCCCAAAAGCAGC--
----------- 9 2,-2
-----------------ACGGAAUCCCAAAAGCAGCU---
----------- 27 2,-1
-----------------ACGGAAUCCCAAAAGCAGCUG--
----------- 5 2,0
-----------------ACGGAAUCCCAAAAGCAGCUGU---------------------------------------
----------- 2 2,1
---GCUGCGCUUGGAUUUCGUCCCC-
----------- 2 0,0

>hsa-mir-1910 5p:12-32 SRX018958
ugucccuucagCCAGUCCUGUGCCUGCCGCCUuugugcuguccuuggagggaggcagaagcaggaugacaaugagggcaa
((.((((.((....(((((((..(((((.(((((...........))))).)))))..)))))))).....)))))))).
--GAGGCAGAAGCAGGAUGACA----------
2

>hsa-mir-192 MA:24-44;mi:67-88 SRX018958
gccgagaccgagugcacagggcuCUGACCUAUGAAUUGACAGCCagugcucucgucuccccucuggCUGCCAAUUCCAUA

GGUCACAGguauguucgccucaaugccagc

.........(((.(((((.(.((.((((((((((((((.(((((((................))))))).))))).))))
))))).)).).))))..))))).........

---------------------CUCUGACCUAUGAAUUGACAGC--------------------------------------
------------------------------ 8 -2,-1

---------------------CUCUGACCUAUGAAUUGACAGCC-------------------------------------
------------------------------ 12 -2,0

----------------------UCUGACCUAUGAAUUGAC---
------------------------------ 3 -1,-4

----------------------UCUGACCUAUGAAUUGACA--
------------------------------ 41 -1,-3

----------------------UCUGACCUAUGAAUUGACAG---------------------------------------
------------------------------ 128 -1,-2

----------------------UCUGACCUAUGAAUUGACAGa--------------------------------------
------------------------------ 5 -1,-2

----------------------UCUGACCUAUGAAUUGACAGaa-------------------------------------
------------------------------ 3 -1,-2

----------------------UCUGACCUAUGAAUUGACAGu--------------------------------------
------------------------------ 2 -1,-2

----------------------UCUGACCUAUGAAUUGACAGC--------------------------------------
------------------------------ 753 -1,-1

----------------------UCUGACCUAUGAAUUGACAGCa-------------------------------------
------------------------------ 98 -1,-1

----------------------UCUGACCUAUGAAUUGACAGCaa------------------------------------
------------------------------ 7 -1,-1

----------------------UCUGACCUAUGAAUUGACAGCau------------------------------------
------------------------------ 5 -1,-1

----------------------UCUGACCUAUGAAUUGACAGCu-------------------------------------
------------------------------ 18 -1,-1

----------------------UCUGACCUAUGAAUUGACAGCC-------------------------------------
------------------------------ 3531 -1,0

----------------------UCUGACCUAUGAAUUGACAGCCu------------------------------------
------------------------------ 339 -1,0

----------------------UCUGACCUAUGAAUUGACAGCCuu-----------------------------------
------------------------------ 59 -1,0

----------------------UCUGACCUAUGAAUUGACAGCCA------------------------------------
------------------------------ 604 -1,1

----------------------UCUGACCUAUGAAUUGACAGCCAa-----------------------------------

------------------------------ 12 -1,1
----------------------UCUGACCUAUGAAUUGACAGCCAau---------------------------------
------------------------------ 2 -1,1
----------------------UCUGACCUAUGAAUUGACAGCCAu----------------------------------
------------------------------ 191 -1,1
----------------------UCUGACCUAUGAAUUGACAGCCAuu---------------------------------
------------------------------ 97 -1,1
----------------------UCUGACCUAUGAAUUGACAGCCAG----------------------------------
------------------------------ 3 -1,2
----------------------UCUGACCUAUGAAUUGACAGCCAGU---------------------------------
------------------------------ 2 -1,3
-----------------------CUGACCUAUGAAUUGACA---------------------------------------
------------------------------ 8210 0,-3
-----------------------CUGACCUAUGAAUUGACAa--------------------------------------
------------------------------ 33 0,-3
-----------------------CUGACCUAUGAAUUGACAaa-------------------------------------
------------------------------ 26 0,-3
-----------------------CUGACCUAUGAAUUGACAau-------------------------------------
------------------------------ 3 0,-3
-----------------------CUGACCUAUGAAUUGACAu--------------------------------------
------------------------------ 8 0,-3
-----------------------CUGACCUAUGAAUUGACAuu-------------------------------------
------------------------------ 16 0,-3
-----------------------CUGACCUAUGAAUUGACAG--------------------------------------
------------------------------ 20917 0,-2
-----------------------CUGACCUAUGAAUUGACAGa-------------------------------------
------------------------------ 568 0,-2
-----------------------CUGACCUAUGAAUUGACAGaa------------------------------------
------------------------------ 107 0,-2
-----------------------CUGACCUAUGAAUUGACAGau------------------------------------
------------------------------ 13 0,-2
-----------------------CUGACCUAUGAAUUGACAGu-------------------------------------
------------------------------ 815 0,-2
-----------------------CUGACCUAUGAAUUGACAGuu------------------------------------
------------------------------ 158 0,-2
-----------------------CUGACCUAUGAAUUGACAGC-------------------------------------
------------------------------ 185649 0,-1
-----------------------CUGACCUAUGAAUUGACAGCa------------------------------------

------------------------------ 22438 0,-1
-----------------------CUGACCUAUGAAUUGACAGCaa-----------------------------------
------------------------------ 3724 0,-1
-----------------------CUGACCUAUGAAUUGACAGCau-----------------------------------
------------------------------ 1398 0,-1
-----------------------CUGACCUAUGAAUUGACAGCu------------------------------------
------------------------------ 5061 0,-1
-----------------------CUGACCUAUGAAUUGACAGCuu-----------------------------------
------------------------------ 498 0,-1
-----------------------CUGACCUAUGAAUUGACAGCC------------------------------------
------------------------------ 585089 0,0
-----------------------CUGACCUAUGAAUUGACAGCCu-----------------------------------
------------------------------ 38430 0,0
-----------------------CUGACCUAUGAAUUGACAGCCuu----------------------------------
------------------------------ 12309 0,0
-----------------------CUGACCUAUGAAUUGACAGCCA-----------------------------------
------------------------------ 316113 0,1
-----------------------CUGACCUAUGAAUUGACAGCCAa----------------------------------
------------------------------ 12857 0,1
-----------------------CUGACCUAUGAAUUGACAGCCAaa---------------------------------
------------------------------ 1167 0,1
-----------------------CUGACCUAUGAAUUGACAGCCAau---------------------------------
------------------------------ 2330 0,1
-----------------------CUGACCUAUGAAUUGACAGCCAu----------------------------------
------------------------------ 152778 0,1
-----------------------CUGACCUAUGAAUUGACAGCCAuu---------------------------------
------------------------------ 77339 0,1
-----------------------CUGACCUAUGAAUUGACAGCCAG----------------------------------
------------------------------ 3310 0,2
-----------------------CUGACCUAUGAAUUGACAGCCAGa---------------------------------
------------------------------ 327 0,2
-----------------------CUGACCUAUGAAUUGACAGCCAGaa--------------------------------
------------------------------ 18 0,2
-----------------------CUGACCUAUGAAUUGACAGCCAGau--------------------------------
------------------------------ 21 0,2
-----------------------CUGACCUAUGAAUUGACAGCCAGU---------------------------------
------------------------------ 3089 0,3
-----------------------CUGACCUAUGAAUUGACAGCCAGUa--------------------------------

------------------------------- 243 0,3
-----------------------CUGACCUAUGAAUUGACAGCCAGUu---------------------------------
------------------------------- 1010 0,3
-----------------------CUGACCUAUGAAUUGACAGCCAGUG---------------------------------
------------------------------- 14 0,4
------------------------UGACCUAUGAAUUGACAG---------------------------------------
------------------------------- 3927 1,-2
------------------------UGACCUAUGAAUUGACAGu--------------------------------------
------------------------------- 34 1,-2
------------------------UGACCUAUGAAUUGACAGuu-------------------------------------
------------------------------- 4 1,-2
------------------------UGACCUAUGAAUUGACAGC--------------------------------------
------------------------------- 37848 1,-1
------------------------UGACCUAUGAAUUGACAGCa-------------------------------------
------------------------------- 637 1,-1
------------------------UGACCUAUGAAUUGACAGCaa------------------------------------
------------------------------- 224 1,-1
------------------------UGACCUAUGAAUUGACAGCau------------------------------------
------------------------------- 38 1,-1
------------------------UGACCUAUGAAUUGACAGCu-------------------------------------
------------------------------- 146 1,-1
------------------------UGACCUAUGAAUUGACAGCuu------------------------------------
------------------------------- 7 1,-1
------------------------UGACCUAUGAAUUGACAGCC-------------------------------------
------------------------------- 16871 1,0
------------------------UGACCUAUGAAUUGACAGCCu------------------------------------
------------------------------- 633 1,0
------------------------UGACCUAUGAAUUGACAGCCuu-----------------------------------
------------------------------- 187 1,0
------------------------UGACCUAUGAAUUGACAGCCA------------------------------------
------------------------------- 44394 1,1
------------------------UGACCUAUGAAUUGACAGCCAa-----------------------------------
------------------------------- 2767 1,1
------------------------UGACCUAUGAAUUGACAGCCAaa----------------------------------
------------------------------- 1374 1,1
------------------------UGACCUAUGAAUUGACAGCCAau----------------------------------
------------------------------- 1314 1,1
------------------------UGACCUAUGAAUUGACAGCCAu-----------------------------------

------------------------------ 12403 1,1

------------------------UGACCUAUGAAUUGACAGCCAuu---------------------------------

------------------------------ 11395 1,1

------------------------UGACCUAUGAAUUGACAGCCAG----------------------------------

------------------------------ 10483 1,2

------------------------UGACCUAUGAAUUGACAGCCAGa---------------------------------

------------------------------ 2378 1,2

------------------------UGACCUAUGAAUUGACAGCCAGaa--------------------------------

------------------------------ 153 1,2

------------------------UGACCUAUGAAUUGACAGCCAGau--------------------------------

------------------------------ 136 1,2

------------------------UGACCUAUGAAUUGACAGCCAGU---------------------------------

------------------------------ 15994 1,3

------------------------UGACCUAUGAAUUGACAGCCAGUa--------------------------------

------------------------------ 2942 1,3

------------------------UGACCUAUGAAUUGACAGCCAGUaa-------------------------------

------------------------------ 217 1,3

------------------------UGACCUAUGAAUUGACAGCCAGUau-------------------------------

------------------------------ 250 1,3

------------------------UGACCUAUGAAUUGACAGCCAGUu--------------------------------

------------------------------ 6142 1,3

------------------------UGACCUAUGAAUUGACAGCCAGUuu-------------------------------

------------------------------ 710 1,3

------------------------UGACCUAUGAAUUGACAGCCAGUG--------------------------------

------------------------------ 116 1,4

------------------------UGACCUAUGAAUUGACAGCCAGUGa-------------------------------

------------------------------ 6 1,4

------------------------UGACCUAUGAAUUGACAGCCAGUGu-------------------------------

------------------------------ 10 1,4

-------------------------GACCUAUGAAUUGACAGC-------------------------------------

------------------------------ 705 2,-1

-------------------------GACCUAUGAAUUGACAGCa------------------------------------

------------------------------ 9 2,-1

-------------------------GACCUAUGAAUUGACAGCaa-----------------------------------

------------------------------ 5 2,-1

-------------------------GACCUAUGAAUUGACAGCau-----------------------------------

------------------------------ 2 2,-1

-------------------------GACCUAUGAAUUGACAGCu------------------------------------

------------------------------ 2 2,-1
-------------------------GACCUAUGAAUUGACAGCC------------------------------------
------------------------------ 423 2,0
-------------------------GACCUAUGAAUUGACAGCCu-----------------------------------
------------------------------ 19 2,0
-------------------------GACCUAUGAAUUGACAGCCuu----------------------------------
------------------------------ 9 2,0
-------------------------GACCUAUGAAUUGACAGCCA-----------------------------------
------------------------------ 559 2,1
-------------------------GACCUAUGAAUUGACAGCCAa----------------------------------
------------------------------ 63 2,1
-------------------------GACCUAUGAAUUGACAGCCAaa---------------------------------
------------------------------ 27 2,1
-------------------------GACCUAUGAAUUGACAGCCAau---------------------------------
------------------------------ 25 2,1
-------------------------GACCUAUGAAUUGACAGCCAu----------------------------------
------------------------------ 174 2,1
-------------------------GACCUAUGAAUUGACAGCCAuu---------------------------------
------------------------------ 159 2,1
-------------------------GACCUAUGAAUUGACAGCCAG----------------------------------
------------------------------ 230 2,2
-------------------------GACCUAUGAAUUGACAGCCAGa---------------------------------
------------------------------ 59 2,2
-------------------------GACCUAUGAAUUGACAGCCAGaa--------------------------------
------------------------------ 2 2,2
-------------------------GACCUAUGAAUUGACAGCCAGau--------------------------------
------------------------------ 2 2,2
-------------------------GACCUAUGAAUUGACAGCCAGU---------------------------------
------------------------------ 399 2,3
-------------------------GACCUAUGAAUUGACAGCCAGUa--------------------------------
------------------------------ 66 2,3
-------------------------GACCUAUGAAUUGACAGCCAGUaa-------------------------------
------------------------------ 7 2,3
-------------------------GACCUAUGAAUUGACAGCCAGUau-------------------------------
------------------------------ 4 2,3
-------------------------GACCUAUGAAUUGACAGCCAGUu--------------------------------
------------------------------ 120 2,3
-------------------------GACCUAUGAAUUGACAGCCAGUuu-------------------------------

----------------------------- 19 2,3
-------------------------GACCUAUGAAUUGACAGCCAGUG--------------------------------
----------------------------- 2   2,4
-------------------------GACCUAUGAAUUGACAGCCAGUGu-------------------------------
----------------------------- 2   2,4
--GGCUGCCAAUUCCAUA
GGUC------------------------- 2   -2,-4
--GGCUGCCAAUUCCAUA
GGUCACu---------------------- 2   -2,-2
--GGCUGCCAAUUCCAUA
GGUCACA---------------------- 3   -2,-1
---GCUGCCAAUUCCAUA
GGUCACA---------------------- 3   -1,-1
---GCUGCCAAUUCCAUA
GGUCACAG--------------------- 3   -1,0
--CUGCCAAUUCCAUA
GGUCA------------------------ 2   0,-3
--CUGCCAAUUCCAUA
GGUCACA---------------------- 9   0,-1
--CUGCCAAUUCCAUA
GGUCACAG--------------------- 5   0,0
--CUGCCAAUUCCAUA
GGUCACAGu-------------------- 5   0,0

>hsa-mir-193a   5p:21-42;3p:55-76   SRX018958
cgaggaugggagcugagggcUGGGUCUUUGCGGGCGAGAUGAgggugucggaucAACUGGCCUACAAAGUCCCAGUucuc
ggcccccg
.......(((.((((((((((((.(((((..(((.((.(((..........))).)).)))..))))).))))))))))
))).))).
--------------------UGGGUCUUUGCGGGCGAG--
--------   2   0,-4
--------------------UGGGUCUUUGCGGGCGAGA---
--------   2   0,-3
--------------------UGGGUCUUUGCGGGCGAGAUa---------------------------------------
--------   5   0,-2
--------------------UGGGUCUUUGCGGGCGAGAUG---------------------------------------
--------   34   0,-1
--------------------UGGGUCUUUGCGGGCGAGAUGu--------------------------------------

--------   23 0,-1

--------------------UGGGUCUUUGCGGGCGAGAUGuu---------------------------------------

--------   2   0,-1

--------------------UGGGUCUUUGCGGGCGAGAUGA--

--------   129 0,0

--------------------UGGGUCUUUGCGGGCGAGAUGAa---------------------------------------

--------   2   0,0

--------------------UGGGUCUUUGCGGGCGAGAUGAu---------------------------------------

--------   12 0,0

--------------------UGGGUCUUUGCGGGCGAGAUGAuu--------------------------------------

--------   2   0,0

---------------------GGGUCUUUGCGGGCGAGAUGu--

--------   3   1,-1

---------------------GGGUCUUUGCGGGCGAGAUGuu---------------------------------------

--------   2   1,-1

---------------------GGGUCUUUGCGGGCGAGAUGAa---------------------------------------

--------   3   1,0

--AACUGGCCUACAAAGUCC--------

--------   212 0,-4

--AACUGGCCUACAAAGUCCaa------

--------   4   0,-4

--AACUGGCCUACAAAGUCCC-------

--------   339 0,-3

--AACUGGCCUACAAAGUCCCA------

--------   3105   0,-2

--AACUGGCCUACAAAGUCCCAa-----

--------   3   0,-2

--AACUGGCCUACAAAGUCCCAaa----

--------   2   0,-2

--AACUGGCCUACAAAGUCCCAu-----

--------   24 0,-2

--AACUGGCCUACAAAGUCCCAuu----

--------   5   0,-2

--AACUGGCCUACAAAGUCCCAG-----

--------   78 0,-1

--AACUGGCCUACAAAGUCCCAGa----

--------   10 0,-1

--AACUGGCCUACAAAGUCCCAGaa---

--------   3   0,-1
--AACUGGCCUACAAAGUCCCAGau---
--------   2   0,-1
--AACUGGCCUACAAAGUCCCAGU----
--------   88 0,0
--AACUGGCCUACAAAGUCCCAGUa---
--------   23 0,0
--AACUGGCCUACAAAGUCCCAGUaa--
--------   4   0,0
--AACUGGCCUACAAAGUCCCAGUau--
--------   3   0,0
--AACUGGCCUACAAAGUCCCAGUU---
--------   2   0,1
---ACUGGCCUACAAAGUCCCA------
--------   4   1,-2

>hsa-mir-193b   mi:14-35;MA:51-72   SRX018958
guggucucagaauCGGGGUUUUGAGGGCGAGAUGAguuuauguuuuauccAACUGGCCCUCAAAGUCCCGCUuuuggggu
cau
(((..(((((((.((((..(((((((((.((.((..(.........)..)).)).)))))))))..)))).)))))))..
)))
------------UCGGGGUUUUGAGGGCGAG---
--- 2   -1,-4
------------UCGGGGUUUUGAGGGCGAGAUG--
--- 2   -1,-1
-------------CGGGGUUUUGAGGGCGAG---
--- 45 0,-4
-------------CGGGGUUUUGAGGGCGAGA--
--- 6   0,-3
-------------CGGGGUUUUGAGGGCGAGAaa--
--- 2   0,-3
-------------CGGGGUUUUGAGGGCGAGAU---
--- 17 0,-2
-------------CGGGGUUUUGAGGGCGAGAUa--
--- 30 0,-2
-------------CGGGGUUUUGAGGGCGAGAUaa---
--- 3   0,-2
-------------CGGGGUUUUGAGGGCGAGAUau---

--- 6 0,-2

-------------CGGGGUUUUGAGGGCGAGAUu---

--- 8 0,-2

-------------CGGGGUUUUGAGGGCGAGAUG---

--- 116 0,-1

-------------CGGGGUUUUGAGGGCGAGAUGu--

--- 114 0,-1

-------------CGGGGUUUUGAGGGCGAGAUGuu---

--- 7 0,-1

-------------CGGGGUUUUGAGGGCGAGAUGA--

--- 433 0,0

-------------CGGGGUUUUGAGGGCGAGAUGAa---

--- 19 0,0

-------------CGGGGUUUUGAGGGCGAGAUGAu---

--- 39 0,0

--------------GGGGUUUUGAGGGCGAGAUG---

--- 6 1,-1

--------------GGGGUUUUGAGGGCGAGAUGu--

--- 7 1,-1

--------------GGGGUUUUGAGGGCGAGAUGA--

--- 20 1,0

--------------GGGGUUUUGAGGGCGAGAUGAa---

--- 3 1,0

---AACUGGCCCUCAAAGUCC------------

--- 48 0,-4

---AACUGGCCCUCAAAGUCCC-----------

--- 46 0,-3

---AACUGGCCCUCAAAGUCCCG----------

--- 12 0,-2

---AACUGGCCCUCAAAGUCCCGuu--------

--- 3 0,-2

---AACUGGCCCUCAAAGUCCCGC---------

--- 15 0,-1

---AACUGGCCCUCAAAGUCCCGCa--------

--- 2 0,-1

---AACUGGCCCUCAAAGUCCCGCaa-------

--- 2 0,-1

---AACUGGCCCUCAAAGUCCCGCau-------

--- 8   0,-1
--AACUGGCCCUCAAAGUCCCGCU--------
--- 73   0,0
--AACUGGCCCUCAAAGUCCCGCUa-------
--- 6   0,0
--AACUGGCCCUCAAAGUCCCGCUau------
--- 22   0,0
--AACUGGCCCUCAAAGUCCCGCUU-------
--- 48   0,1
--AACUGGCCCUCAAAGUCCCGCUUa------
--- 6   0,1
--AACUGGCCCUCAAAGUCCCGCUUau-----
--- 5   0,1
--AACUGGCCCUCAAAGUCCCGCUUU------
--- 33   0,2
--AACUGGCCCUCAAAGUCCCGCUUUU-----
--- 12   0,3
---ACUGGCCCUCAAAGUCCCGCUau------
--- 7   1,0
---ACUGGCCCUCAAAGUCCCGCUUU------
--- 4   1,2
---ACUGGCCCUCAAAGUCCCGCUUUa-----
--- 2   1,2

>hsa-mir-194-1 5p:15-36   SRX018958
augguguuaucaagUGUAACAGCAACUCCAUGUGGAcuguguaccaauuuccaguggagaugcuguuacuuuugaugguu
accaa
.(((((..((((((.(((((((((.((((((.((((..((......)).)))))))))).))))))))).))))))..).
)))).
--------------UGUAACAGCAACUCCAUG--
-----   15   0,-4
--------------UGUAACAGCAACUCCAUGU---
-----   25   0,-3
--------------UGUAACAGCAACUCCAUGUaa---
-----   2   0,-3
--------------UGUAACAGCAACUCCAUGUG--
-----   27   0,-2
--------------UGUAACAGCAACUCCAUGUGa---

----- 4 0,-2
--------------UGUAACAGCAACUCCAUGUGaa--
----- 4 0,-2
--------------UGUAACAGCAACUCCAUGUGG---
----- 365 0,-1
--------------UGUAACAGCAACUCCAUGUGGu--
----- 14 0,-1
--------------UGUAACAGCAACUCCAUGUGGA--
----- 1017 0,0
--------------UGUAACAGCAACUCCAUGUGGAu---
----- 216 0,0
--------------UGUAACAGCAACUCCAUGUGGAuu--
----- 15 0,0
--------------UGUAACAGCAACUCCAUGUGGAC---
----- 12 0,1
--------------UGUAACAGCAACUCCAUGUGGACa--
----- 4 0,1
---------------GUAACAGCAACUCCAUGU---
----- 3 1,-3
---------------GUAACAGCAACUCCAUGUG--
----- 2 1,-2
---------------GUAACAGCAACUCCAUGUGG---
----- 5 1,-1
---------------GUAACAGCAACUCCAUGUGGu--
----- 2 1,-1
---------------GUAACAGCAACUCCAUGUGGA--
----- 2 1,0
---------------GUAACAGCAACUCCAUGUGGAu---
----- 3 1,0

>hsa-mir-194-2 MA:15-36;mi:51-72 SRX018958
ugguucccgcccccUGUAACAGCAACUCCAUGUGGAagugcccacugguuCCAGUGGGGCUGCUGUUAUCUGgggcgagg
gccag
((((((.((((((..(((((((((.((((((.((((...(((....))))))))))))).)))))))))..)))))).))
)))).
------------CCUGUAACAGCAACUCCAUGUGG---
----- 12 -2,-1
------------CCUGUAACAGCAACUCCAUGUGGAu---

----- 2 -2,0
-------------CUGUAACAGCAACUCCAUGUGG---
----- 31 -1,-1
-------------CUGUAACAGCAACUCCAUGUGGu--
----- 3 -1,-1
-------------CUGUAACAGCAACUCCAUGUGGA--
----- 72 -1,0
-------------CUGUAACAGCAACUCCAUGUGGAu---------------------------------------
----- 5 -1,0
-------------CUGUAACAGCAACUCCAUGUGGAA---------------------------------------
----- 21 -1,1
-------------CUGUAACAGCAACUCCAUGUGGAAa--------------------------------------
----- 6 -1,1
--------------UGUAACAGCAACUCCAUG--
----- 15 0,-4
--------------UGUAACAGCAACUCCAUGU---
----- 25 0,-3
--------------UGUAACAGCAACUCCAUGUaa---
----- 2 0,-3
--------------UGUAACAGCAACUCCAUGUG--
----- 27 0,-2
--------------UGUAACAGCAACUCCAUGUGa---
----- 4 0,-2
--------------UGUAACAGCAACUCCAUGUGaa--
----- 4 0,-2
--------------UGUAACAGCAACUCCAUGUGG---
----- 365 0,-1
--------------UGUAACAGCAACUCCAUGUGGu--
----- 14 0,-1
--------------UGUAACAGCAACUCCAUGUGGA--
----- 1017 0,0
--------------UGUAACAGCAACUCCAUGUGGAu---------------------------------------
----- 216 0,0
--------------UGUAACAGCAACUCCAUGUGGAuu--------------------------------------
----- 15 0,0
--------------UGUAACAGCAACUCCAUGUGGAA---------------------------------------
----- 2875 0,1
--------------UGUAACAGCAACUCCAUGUGGAAa--------------------------------------

----- 537 0,1

--------------UGUAACAGCAACUCCAUGUGGAAaa--

----- 24 0,1

--------------UGUAACAGCAACUCCAUGUGGAAau--

----- 26 0,1

--------------UGUAACAGCAACUCCAUGUGGAAu---

----- 191 0,1

--------------UGUAACAGCAACUCCAUGUGGAAuu--

----- 47 0,1

--------------UGUAACAGCAACUCCAUGUGGAAG---

----- 6 0,2

---------------GUAACAGCAACUCCAUGU--

----- 3 1,-3

---------------GUAACAGCAACUCCAUGUG---

----- 2 1,-2

---------------GUAACAGCAACUCCAUGUGG--

----- 5 1,-1

---------------GUAACAGCAACUCCAUGUGGu---

----- 2 1,-1

---------------GUAACAGCAACUCCAUGUGGA---

----- 2 1,0

---------------GUAACAGCAACUCCAUGUGGAu--

----- 3 1,0

---------------GUAACAGCAACUCCAUGUGGAA--

----- 11 1,1

---------------GUAACAGCAACUCCAUGUGGAAa---

----- 4 1,1

---CCAGUGGGGCUGCUGUUAaa----------

----- 3 0,-4

---CCAGUGGGGCUGCUGUUAU-----------

----- 2 0,-3

---CCAGUGGGGCUGCUGUUAUa----------

----- 29 0,-3

---CCAGUGGGGCUGCUGUUAUC----------

----- 17 0,-2

---CCAGUGGGGCUGCUGUUAUCU---------

----- 108 0,-1

---CCAGUGGGGCUGCUGUUAUCUG--------

----- 7 0,0
--CCAGUGGGGCUGCUGUUAUCUGa-------
----- 8 0,0
--CCAGUGGGGCUGCUGUUAUCUGu-------
----- 15 0,0
--CCAGUGGGGCUGCUGUUAUCUGuu------
----- 2 0,0
---CAGUGGGGCUGCUGUUAUC----------
----- 2 1,-2
---CAGUGGGGCUGCUGUUAUCU---------
----- 3 1,-1

>hsa-mir-195 MA:15-35;mi:53-74 SRX018958
agcuucccuggcucUAGCAGCACAGAAAUAUUGGCacagggaagcgagucugCCAAUAUUGGCUGUGCUGCUCCaggcag
gguggug
.(((.(((((.((..((((((((((.((((((((((.............))))))))))..))))))))))..)).)))
)).))).
-------------CUAGCAGCACAGAAAUAUUGG---
------- 3 -1,-1
--------------UAGCAGCACAGAAAUAUU---
------- 37 0,-3
--------------UAGCAGCACAGAAAUAUUa--
------- 8 0,-3
--------------UAGCAGCACAGAAAUAUUaa---
------- 3 0,-3
--------------UAGCAGCACAGAAAUAUUG--
------- 124 0,-2
--------------UAGCAGCACAGAAAUAUUGa---
------- 4 0,-2
--------------UAGCAGCACAGAAAUAUUGaa--
------- 9 0,-2
--------------UAGCAGCACAGAAAUAUUGu---
------- 5 0,-2
--------------UAGCAGCACAGAAAUAUUGG---
------- 763 0,-1
--------------UAGCAGCACAGAAAUAUUGGa--
------- 49 0,-1
--------------UAGCAGCACAGAAAUAUUGGaa---

------- 36 0,-1
--------------UAGCAGCACAGAAAUAUUGGu---
------- 11 0,-1
--------------UAGCAGCACAGAAAUAUUGGC---
------- 82 0,0
--------------UAGCAGCACAGAAAUAUUGGCA--
------- 1481   0,1
--------------UAGCAGCACAGAAAUAUUGGCAa---
------- 1127   0,1
--------------UAGCAGCACAGAAAUAUUGGCAaa--
------- 15 0,1
--------------UAGCAGCACAGAAAUAUUGGCAau--
------- 28 0,1
--------------UAGCAGCACAGAAAUAUUGGCAu---
------- 667 0,1
--------------UAGCAGCACAGAAAUAUUGGCAuu--
------- 36 0,1
--------------UAGCAGCACAGAAAUAUUGGCAC---
------- 4   0,2
--------------UAGCAGCACAGAAAUAUUGGCACA--
------- 2   0,3
---------------AGCAGCACAGAAAUAUUG---
------- 3   1,-2
---------------AGCAGCACAGAAAUAUUGG--
------- 5   1,-1
---------------AGCAGCACAGAAAUAUUGGCA--
------- 14 1,1
---------------AGCAGCACAGAAAUAUUGGCAa---
------- 9   1,1
---------------AGCAGCACAGAAAUAUUGGCAu---
------- 6   1,1
---------------AGCAGCACAGAAAUAUUGGCAuu--
------- 2   1,1
----------------GCAGCACAGAAAUAUUGGCAa---
------- 3   2,1

>hsa-mir-197   3p:48-69   SRX018958
ggcugugccggguagagagggcagugggagguaagagcucuucacccUUCACCACCUUCUCCACCCAGCauggcc

((((((((.((((.(((((((..((((..((((((....))).)))..))))..))))))).)))).))))))))
--------CGGGUAGAGAGGGCAGUG--- 2
--------CGGGUAGAGAGGGCAGUGGGAGG-- 3
---UUCACCACCUUCUCCACCC--------- 2
0,-3
---UUCACCACCUUCUCCACCCAG------- 2
0,-1
---UUCACCACCUUCUCCACCCAGC------ 4
0,0
---UUCACCACCUUCUCCACCCAGCA----- 3
0,1

>hsa-mir-199a-1 5p:6-28;3p:47-68 SRX018958
gccaaCCCAGUGUUCAGACUACCUGUUCaggaggcucucaauguguACAGUAGUCUGCACAUUGGUUAggc
(((...(((((((.((((((((.(((.((.(((...)))....)).))))))))))).)))))))...)))
-----CCCAGUGUUCAGACUACC-- 236 0,-5
-----CCCAGUGUUCAGACUACCU--- 2340
0,-4
-----CCCAGUGUUCAGACUACCUG-- 107 0,-3
-----CCCAGUGUUCAGACUACCUGU--- 51 0,-2
-----CCCAGUGUUCAGACUACCUGUa-- 3 0,-2
-----CCCAGUGUUCAGACUACCUGUU-- 659 0,-1
-----CCCAGUGUUCAGACUACCUGUUa--- 45 0,-1
-----CCCAGUGUUCAGACUACCUGUUu--- 64 0,-1
-----CCCAGUGUUCAGACUACCUGUUuu-- 2 0,-1
-----CCCAGUGUUCAGACUACCUGUUC--- 181 0,0
-----CCCAGUGUUCAGACUACCUGUUCu-- 3 0,0
-----CCCAGUGUUCAGACUACCUGUUCA-- 4 0,1
-----CCCAGUGUUCAGACUACCUGUUCAa--- 3 0,1
------CCAGUGUUCAGACUACCU--- 28 1,-4
------CCAGUGUUCAGACUACCUGUa-- 2 1,-2
------CCAGUGUUCAGACUACCUGUU-- 10 1,-1
------CCAGUGUUCAGACUACCUGUUu--- 3 1,-1
------CCAGUGUUCAGACUACCUGUUC--- 3 1,0
---UACAGUAGUCUGCACAUU-------- 111
-1,-5
---UACAGUAGUCUGCACAUUaa------ 2
-1,-5

---UACAGUAGUCUGCACAUUG------- 313
-1,-4
---UACAGUAGUCUGCACAUUGaa----- 4
-1,-4
---UACAGUAGUCUGCACAUUGu------ 4
-1,-4
---UACAGUAGUCUGCACAUUGuu----- 4
-1,-4
---UACAGUAGUCUGCACAUUGG------ 578
-1,-3
---UACAGUAGUCUGCACAUUGGa----- 33
-1,-3
---UACAGUAGUCUGCACAUUGGaa---- 6
-1,-3
---UACAGUAGUCUGCACAUUGGau---- 43
-1,-3
---UACAGUAGUCUGCACAUUGGU----- 1280
-1,-2
---UACAGUAGUCUGCACAUUGGUa---- 597
-1,-2
---UACAGUAGUCUGCACAUUGGUaa--- 479
-1,-2
---UACAGUAGUCUGCACAUUGGUau--- 398
-1,-2
---UACAGUAGUCUGCACAUUGGUU---- 13202
-1,-1
---UACAGUAGUCUGCACAUUGGUUu--- 2109
-1,-1
---UACAGUAGUCUGCACAUUGGUUuu-- 77
-1,-1
---UACAGUAGUCUGCACAUUGGUUA--- 2564
-1,0
---UACAGUAGUCUGCACAUUGGUUAa-- 293 -1,0
---UACAGUAGUCUGCACAUUGGUUAaa- 2 -1,0
---UACAGUAGUCUGCACAUUGGUUAu-- 173 -1,0
---UACAGUAGUCUGCACAUUGGUUAG-- 6 -1,1
--ACAGUAGUCUGCACAUUG------- 1209
0,-4

--ACAGUAGUCUGCACAUUGa------ 31 0,-4
--ACAGUAGUCUGCACAUUGaa----- 10 0,-4
--ACAGUAGUCUGCACAUUGau----- 3 0,-4
--ACAGUAGUCUGCACAUUGu------ 35 0,-4
--ACAGUAGUCUGCACAUUGuu----- 24 0,-4
--ACAGUAGUCUGCACAUUGG------ 5039
0,-3
--ACAGUAGUCUGCACAUUGGa----- 38 0,-3
--ACAGUAGUCUGCACAUUGGaa---- 37 0,-3
--ACAGUAGUCUGCACAUUGGau---- 102 0,-3
--ACAGUAGUCUGCACAUUGGU----- 4523
0,-2
--ACAGUAGUCUGCACAUUGGUa---- 1363
0,-2
--ACAGUAGUCUGCACAUUGGUaa--- 2547
0,-2
--ACAGUAGUCUGCACAUUGGUau--- 812 0,-2
--ACAGUAGUCUGCACAUUGGUU---- 74446
0,-1
--ACAGUAGUCUGCACAUUGGUUu--- 11560
0,-1
--ACAGUAGUCUGCACAUUGGUUuu-- 498 0,-1
--ACAGUAGUCUGCACAUUGGUUA--- 51924
0,0
--ACAGUAGUCUGCACAUUGGUUAa-- 25332
0,0
--ACAGUAGUCUGCACAUUGGUUAaa- 152 0,0
--ACAGUAGUCUGCACAUUGGUUAau- 100 0,0
--ACAGUAGUCUGCACAUUGGUUAu-- 3895
0,0
--ACAGUAGUCUGCACAUUGGUUAuu- 60 0,0
--ACAGUAGUCUGCACAUUGGUUAG-- 100 0,1
--ACAGUAGUCUGCACAUUGGUUAGu- 5 0,1
--ACAGUAGUCUGCACAUUGGUUAGG- 2 0,2
---CAGUAGUCUGCACAUUGG------ 611 1,-3
---CAGUAGUCUGCACAUUGGa----- 5 1,-3
---CAGUAGUCUGCACAUUGGaa---- 2 1,-3
---CAGUAGUCUGCACAUUGGau---- 3 1,-3

---CAGUAGUCUGCACAUUGGU----- 474 1,-2
---CAGUAGUCUGCACAUUGGUa---- 143 1,-2
---CAGUAGUCUGCACAUUGGUaa--- 283 1,-2
---CAGUAGUCUGCACAUUGGUau--- 55 1,-2
---CAGUAGUCUGCACAUUGGUU---- 5499 1,-1
---CAGUAGUCUGCACAUUGGUUu--- 1054 1,-1
---CAGUAGUCUGCACAUUGGUUuu-- 130 1,-1
---CAGUAGUCUGCACAUUGGUUA--- 9473 1,0
---CAGUAGUCUGCACAUUGGUUAa-- 4457 1,0
---CAGUAGUCUGCACAUUGGUUAaa- 146 1,0
---CAGUAGUCUGCACAUUGGUUAau- 29 1,0
---CAGUAGUCUGCACAUUGGUUAu-- 924 1,0
---CAGUAGUCUGCACAUUGGUUAuu- 14 1,0
---CAGUAGUCUGCACAUUGGUUAG-- 41 1,1
---CAGUAGUCUGCACAUUGGUUAGu- 2 1,1
--AGUAGUCUGCACAUUGGU----- 8 2,-2
--AGUAGUCUGCACAUUGGUa---- 3 2,-2
--AGUAGUCUGCACAUUGGUaa--- 2 2,-2
--AGUAGUCUGCACAUUGGUU---- 145 2,-1
--AGUAGUCUGCACAUUGGUUu--- 23 2,-1
--AGUAGUCUGCACAUUGGUUuu-- 2 2,-1
--AGUAGUCUGCACAUUGGUUA--- 113 2,0
--AGUAGUCUGCACAUUGGUUAa-- 79 2,0
--AGUAGUCUGCACAUUGGUUAaa- 5 2,0
--AGUAGUCUGCACAUUGGUUAau- 2 2,0
--AGUAGUCUGCACAUUGGUUAu-- 23 2,0

>hsa-mir-199a-2 5p:31-53;3p:70-91 SRX018958
aggaagcuucuggagauccugcuccgucgcCCCAGUGUUCAGACUACCUGUUCaggacaaugccguuguACAGUAGUCUGCACAUUGGUUAgacugggcaagggagagca
.....(((.((.....(((((((.(((...(((((((.((((((((.(((...((......)).....))))))))))).)))))))...))).)))).))))))))).
-----------------------------CCCCAGUGUUCAGACUACCU--- 2 -1,-4

------------------------------CCCAGUGUUCAGACUACC-------------------------------
------------------------------ 236 0,-5
------------------------------CCCAGUGUUCAGACUACCU------------------------------
------------------------------ 2340 0,-4
------------------------------CCCAGUGUUCAGACUACCUG-----------------------------
------------------------------ 107 0,-3
------------------------------CCCAGUGUUCAGACUACCUGU----------------------------
------------------------------ 51 0,-2
------------------------------CCCAGUGUUCAGACUACCUGUa---------------------------
------------------------------ 3 0,-2
------------------------------CCCAGUGUUCAGACUACCUGUU---------------------------
------------------------------ 659 0,-1
------------------------------CCCAGUGUUCAGACUACCUGUUa--------------------------
------------------------------ 45 0,-1
------------------------------CCCAGUGUUCAGACUACCUGUUu--------------------------
------------------------------ 64 0,-1
------------------------------CCCAGUGUUCAGACUACCUGUUuu-------------------------
------------------------------ 2 0,-1
------------------------------CCCAGUGUUCAGACUACCUGUUC--------------------------
------------------------------ 181 0,0
------------------------------CCCAGUGUUCAGACUACCUGUUCu-------------------------
------------------------------ 3 0,0
------------------------------CCCAGUGUUCAGACUACCUGUUCA-------------------------
------------------------------ 4 0,1
------------------------------CCCAGUGUUCAGACUACCUGUUCAa------------------------
------------------------------ 3 0,1
-------------------------------CCAGUGUUCAGACUACCU------------------------------
------------------------------ 28 1,-4
-------------------------------CCAGUGUUCAGACUACCUGUa---------------------------
------------------------------ 2 1,-2
-------------------------------CCAGUGUUCAGACUACCUGUU---------------------------
------------------------------ 10 1,-1
-------------------------------CCAGUGUUCAGACUACCUGUUu--------------------------
------------------------------ 3 1,-1
-------------------------------CCAGUGUUCAGACUACCUGUUC--------------------------
------------------------------ 3 1,0
---UACAGUAGUCUG
CACAUU------------------------ 111 -1,-5

---UACAGUAGUCUG
CACAUUaa----------------------   2   -1,-5
---UACAGUAGUCUG
CACAUUG-----------------------   313 -1,-4
---UACAGUAGUCUG
CACAUUGaa---------------------   4   -1,-4
---UACAGUAGUCUG
CACAUUGu----------------------   4   -1,-4
---UACAGUAGUCUG
CACAUUGuu---------------------   4   -1,-4
---UACAGUAGUCUG
CACAUUGG----------------------   578 -1,-3
---UACAGUAGUCUG
CACAUUGGa---------------------   33 -1,-3
---UACAGUAGUCUG
CACAUUGGaa--------------------   6   -1,-3
---UACAGUAGUCUG
CACAUUGGau--------------------   43 -1,-3
---UACAGUAGUCUG
CACAUUGGU---------------------   1280   -1,-2
---UACAGUAGUCUG
CACAUUGGUa--------------------   597 -1,-2
---UACAGUAGUCUG
CACAUUGGUaa-------------------   479 -1,-2
---UACAGUAGUCUG
CACAUUGGUau-------------------   398 -1,-2
---UACAGUAGUCUG
CACAUUGGUU--------------------   13202   -1,-1
---UACAGUAGUCUG
CACAUUGGUUu-------------------   2109   -1,-1
---UACAGUAGUCUG
CACAUUGGUUuu------------------   77 -1,-1
---UACAGUAGUCUG
CACAUUGGUUA-------------------   2564   -1,0
---UACAGUAGUCUG
CACAUUGGUUAa------------------   293 -1,0
---UACAGUAGUCUG
CACAUUGGUUAaa-----------------   2   -1,0

--UACAGUAGUCUG

CACAUUGGUUAu------------------ 173 -1,0

--UACAGUAGUCUG

CACAUUGGUUAG------------------ 6 -1,1

---ACAGUAGUCUG

CACAUUG----------------------- 1209 0,-4

---ACAGUAGUCUG

CACAUUGa---------------------- 31 0,-4

---ACAGUAGUCUG

CACAUUGaa--------------------- 10 0,-4

---ACAGUAGUCUG

CACAUUGau--------------------- 3 0,-4

---ACAGUAGUCUG

CACAUUGu---------------------- 35 0,-4

---ACAGUAGUCUG

CACAUUGuu--------------------- 24 0,-4

---ACAGUAGUCUG

CACAUUGG---------------------- 5039 0,-3

---ACAGUAGUCUG

CACAUUGGa--------------------- 38 0,-3

---ACAGUAGUCUG

CACAUUGGaa-------------------- 37 0,-3

---ACAGUAGUCUG

CACAUUGGau-------------------- 102 0,-3

---ACAGUAGUCUG

CACAUUGGU--------------------- 4523 0,-2

---ACAGUAGUCUG

CACAUUGGUa-------------------- 1363 0,-2

---ACAGUAGUCUG

CACAUUGGUaa------------------- 2547 0,-2

---ACAGUAGUCUG

CACAUUGGUau------------------- 812 0,-2

---ACAGUAGUCUG

CACAUUGGUU-------------------- 74446 0,-1

---ACAGUAGUCUG

CACAUUGGUUu------------------- 11560 0,-1

---ACAGUAGUCUG

CACAUUGGUUuu------------------ 498 0,-1

--ACAGUAGUCUG
CACAUUGGUUA------------------- 51924 0,0
--ACAGUAGUCUG
CACAUUGGUUAa------------------ 25332 0,0
--ACAGUAGUCUG
CACAUUGGUUAaa----------------- 152 0,0
--ACAGUAGUCUG
CACAUUGGUUAau----------------- 100 0,0
--ACAGUAGUCUG
CACAUUGGUUAu------------------ 3895 0,0
--ACAGUAGUCUG
CACAUUGGUUAuu----------------- 60 0,0
--ACAGUAGUCUG
CACAUUGGUUAG------------------ 100 0,1
--ACAGUAGUCUG
CACAUUGGUUAGu----------------- 5 0,1
--ACAGUAGUCUG
CACAUUGGUUAGA----------------- 3 0,2
--ACAGUAGUCUG
CACAUUGGUUAGAC---------------- 2 0,3
---CAGUAGUCUG
CACAUUGG---------------------- 611 1,-3
---CAGUAGUCUG
CACAUUGGa--------------------- 5 1,-3
---CAGUAGUCUG
CACAUUGGaa-------------------- 2 1,-3
---CAGUAGUCUG
CACAUUGGau-------------------- 3 1,-3
---CAGUAGUCUG
CACAUUGGU--------------------- 474 1,-2
---CAGUAGUCUG
CACAUUGGUa-------------------- 143 1,-2
---CAGUAGUCUG
CACAUUGGUaa------------------- 283 1,-2
---CAGUAGUCUG
CACAUUGGUau------------------- 55 1,-2
---CAGUAGUCUG
CACAUUGGUU-------------------- 5499 1,-1

---CAGUAGUCUG
CACAUUGGUUu------------------- 1054 1,-1
---CAGUAGUCUG
CACAUUGGUUuu------------------ 130 1,-1
---CAGUAGUCUG
CACAUUGGUUA------------------- 9473 1,0
---CAGUAGUCUG
CACAUUGGUUAa------------------ 4457 1,0
---CAGUAGUCUG
CACAUUGGUUAaa----------------- 146 1,0
---CAGUAGUCUG
CACAUUGGUUAau----------------- 29 1,0
---CAGUAGUCUG
CACAUUGGUUAu------------------ 924 1,0
---CAGUAGUCUG
CACAUUGGUUAuu----------------- 14 1,0
---CAGUAGUCUG
CACAUUGGUUAG------------------ 41 1,1
---CAGUAGUCUG
CACAUUGGUUAGu----------------- 2 1,1
--AGUAGUCUG
CACAUUGGU--------------------- 8 2,-2
--AGUAGUCUG
CACAUUGGUa-------------------- 3 2,-2
--AGUAGUCUG
CACAUUGGUaa------------------- 2 2,-2
--AGUAGUCUG
CACAUUGGUU-------------------- 145 2,-1
--AGUAGUCUG
CACAUUGGUUu------------------- 23 2,-1
--AGUAGUCUG
CACAUUGGUUuu------------------ 2 2,-1
--AGUAGUCUG
CACAUUGGUUA------------------- 113 2,0
--AGUAGUCUG
CACAUUGGUUAa------------------ 79 2,0
--AGUAGUCUG
CACAUUGGUUAaa----------------- 5 2,0

---AGUAGUCUG
CACAUUGGUUAau----------------- 2   2,0
---AGUAGUCUG
CACAUUGGUUAu------------------ 23 2,0

>hsa-mir-199b   5p:26-48;3p:65-86   SRX018958
ccagaggacaccuccacuccgucuaCCCAGUGUUUAGACUAUCUGUUCaggacucccaaauuguACAGUAGUCUGCACAU
UGGUUAggcugggcuggguuagacccucgg
...((((.....((((..(((((((.(((((((.(((((((.((((.((((....))....)).))))))))))).))))
))).))))).))..))))......))))..
-------------------------CCCAGUGUUUAGACUAUC-------------------------------------
------------------------------ 48 0,-5
-------------------------CCCAGUGUUUAGACUAUCU------------------------------------
------------------------------ 383 0,-4
-------------------------CCCAGUGUUUAGACUAUCUG-----------------------------------
------------------------------ 6   0,-3
-------------------------CCCAGUGUUUAGACUAUCUGa----------------------------------
------------------------------ 3   0,-3
-------------------------CCCAGUGUUUAGACUAUCUGU----------------------------------
------------------------------ 6   0,-2
-------------------------CCCAGUGUUUAGACUAUCUGUU---------------------------------
------------------------------ 30 0,-1
-------------------------CCCAGUGUUUAGACUAUCUGUUC--------------------------------
------------------------------ 10 0,0
--------------------------CCAGUGUUUAGACUAUCU------------------------------------
------------------------------ 3   1,-4
--------------------------CCAGUGUUUAGACUAUCUGUUC--------------------------------
------------------------------ 2   1,0
---UACAGUAGUCUGCACAU
U----------------------------- 111 -1,-5
---UACAGUAGUCUGCACAU
Uaa--------------------------- 2   -1,-5
---UACAGUAGUCUGCACAU
UG---------------------------- 313 -1,-4
---UACAGUAGUCUGCACAU
UGaa-------------------------- 4   -1,-4
---UACAGUAGUCUGCACAU
UGu--------------------------- 4   -1,-4

--UACAGUAGUCUGCACAU
UGuu------------------------- 4 -1,-4
--UACAGUAGUCUGCACAU
UGG-------------------------- 578 -1,-3
--UACAGUAGUCUGCACAU
UGGa------------------------- 33 -1,-3
--UACAGUAGUCUGCACAU
UGGaa------------------------ 6 -1,-3
--UACAGUAGUCUGCACAU
UGGau------------------------ 43 -1,-3
--UACAGUAGUCUGCACAU
UGGU------------------------- 1280 -1,-2
--UACAGUAGUCUGCACAU
UGGUa------------------------ 597 -1,-2
--UACAGUAGUCUGCACAU
UGGUaa----------------------- 479 -1,-2
--UACAGUAGUCUGCACAU
UGGUau----------------------- 398 -1,-2
--UACAGUAGUCUGCACAU
UGGUU------------------------ 13202 -1,-1
--UACAGUAGUCUGCACAU
UGGUUu----------------------- 2109 -1,-1
--UACAGUAGUCUGCACAU
UGGUUuu---------------------- 77 -1,-1
--UACAGUAGUCUGCACAU
UGGUUA----------------------- 2564 -1,0
--UACAGUAGUCUGCACAU
UGGUUAa---------------------- 293 -1,0
--UACAGUAGUCUGCACAU
UGGUUAaa--------------------- 2 -1,0
--UACAGUAGUCUGCACAU
UGGUUAu---------------------- 173 -1,0
--UACAGUAGUCUGCACAU
UGGUUAG---------------------- 6 -1,1
---ACAGUAGUCUGCACAU
UG--------------------------- 1209 0,-4
---ACAGUAGUCUGCACAU
UGa-------------------------- 31 0,-4

--ACAGUAGUCUGCACAU
UGaa-------------------------- 10 0,-4
--ACAGUAGUCUGCACAU
UGau-------------------------- 3 0,-4
--ACAGUAGUCUGCACAU
UGu--------------------------- 35 0,-4
--ACAGUAGUCUGCACAU
UGuu-------------------------- 24 0,-4
--ACAGUAGUCUGCACAU
UGG--------------------------- 5039 0,-3
--ACAGUAGUCUGCACAU
UGGa-------------------------- 38 0,-3
--ACAGUAGUCUGCACAU
UGGaa------------------------- 37 0,-3
--ACAGUAGUCUGCACAU
UGGau------------------------- 102 0,-3
--ACAGUAGUCUGCACAU
UGGU-------------------------- 4523 0,-2
--ACAGUAGUCUGCACAU
UGGUa------------------------- 1363 0,-2
--ACAGUAGUCUGCACAU
UGGUaa------------------------ 2547 0,-2
--ACAGUAGUCUGCACAU
UGGUau------------------------ 812 0,-2
--ACAGUAGUCUGCACAU
UGGUU------------------------- 74446 0,-1
--ACAGUAGUCUGCACAU
UGGUUu------------------------ 11560 0,-1
--ACAGUAGUCUGCACAU
UGGUUuu----------------------- 498 0,-1
--ACAGUAGUCUGCACAU
UGGUUA------------------------ 51924 0,0
--ACAGUAGUCUGCACAU
UGGUUAa----------------------- 25332 0,0
--ACAGUAGUCUGCACAU
UGGUUAaa---------------------- 152 0,0
--ACAGUAGUCUGCACAU
UGGUUAau---------------------- 100 0,0

---ACAGUAGUCUGCACAU
UGGUUAu----------------------- 3895   0,0
---ACAGUAGUCUGCACAU
UGGUUAuu---------------------- 60   0,0
---ACAGUAGUCUGCACAU
UGGUUAG----------------------- 100 0,1
---ACAGUAGUCUGCACAU
UGGUUAGu---------------------- 5   0,1
---ACAGUAGUCUGCACAU
UGGUUAGG---------------------- 2   0,2
--CAGUAGUCUGCACAU
UGG--------------------------- 611 1,-3
--CAGUAGUCUGCACAU
UGGa-------------------------- 5   1,-3
--CAGUAGUCUGCACAU
UGGaa------------------------- 2   1,-3
--CAGUAGUCUGCACAU
UGGau------------------------- 3   1,-3
--CAGUAGUCUGCACAU
UGGU-------------------------- 474 1,-2
--CAGUAGUCUGCACAU
UGGUa------------------------- 143 1,-2
--CAGUAGUCUGCACAU
UGGUaa------------------------ 283 1,-2
--CAGUAGUCUGCACAU
UGGUau------------------------ 55   1,-2
--CAGUAGUCUGCACAU
UGGUU------------------------- 5499   1,-1
--CAGUAGUCUGCACAU
UGGUUu------------------------ 1054   1,-1
--CAGUAGUCUGCACAU
UGGUUuu----------------------- 130 1,-1
--CAGUAGUCUGCACAU
UGGUUA------------------------ 9473   1,0
--CAGUAGUCUGCACAU
UGGUUAa----------------------- 4457   1,0
--CAGUAGUCUGCACAU
UGGUUAaa---------------------- 146 1,0

--CAGUAGUCUGCACAU
UGGUUAau--------------------- 29 1,0
--CAGUAGUCUGCACAU
UGGUUAu---------------------- 924 1,0
--CAGUAGUCUGCACAU
UGGUUAuu--------------------- 14 1,0
--CAGUAGUCUGCACAU
UGGUUAG---------------------- 41 1,1
--CAGUAGUCUGCACAU
UGGUUAGu--------------------- 2 1,1
---AGUAGUCUGCACAU
UGGU------------------------- 8 2,-2
---AGUAGUCUGCACAU
UGGUa------------------------ 3 2,-2
---AGUAGUCUGCACAU
UGGUaa----------------------- 2 2,-2
---AGUAGUCUGCACAU
UGGUU------------------------ 145 2,-1
---AGUAGUCUGCACAU
UGGUUu----------------------- 23 2,-1
---AGUAGUCUGCACAU
UGGUUuu---------------------- 2 2,-1
---AGUAGUCUGCACAU
UGGUUA----------------------- 113 2,0
---AGUAGUCUGCACAU
UGGUUAa---------------------- 79 2,0
---AGUAGUCUGCACAU
UGGUUAaa--------------------- 5 2,0
---AGUAGUCUGCACAU
UGGUUAau--------------------- 2 2,0
---AGUAGUCUGCACAU
UGGUUAu---------------------- 23 2,0

>hsa-mir-19a mi:14-35;MA:49-71 SRX018958
gcaguccucuguuAGUUUUGCAUAGUUGCACUACAagaagaauguaguUGUGCAAAUCUAUGCAAAACUGAugguggccu
gc
((((.((.((((((((((((((((((((((((((.......))))...))))))..))))))))))))))))).)).))
))

-------------AGUUUUGCAUAGUUGCACUA---
-- 4   0,-2
---UUGUGCAAAUCUAUGCAAAACU-----------
-- 5   -1,-2
--UGUGCAAAUCUAUGCAAAAC------------
-- 2   0,-3
--UGUGCAAAUCUAUGCAAAACU-----------
-- 9   0,-2
--UGUGCAAAUCUAUGCAAAACUG----------
-- 5   0,-1
--UGUGCAAAUCUAUGCAAAACUGA---------
-- 4   0,0

>hsa-mir-19b-1 mi:16-38;MA:54-76   SRX018958
cacuguucuaugguuAGUUUUGCAGGUUUGCAUCCAGCugugugauauucugcUGUGCAAAUCCAUGCAAAACUGAcugu
gguagug
(((((..(((((((((((((((((((((((((..((((.............))))))))))).)).)))))))))))))))
)))))))
---------------AGUUUUGCAGGUUUGCAUCCA--
------- 3   0,-2
---------------AGUUUUGCAGGUUUGCAUCCAG---
------- 2   0,-1
---UGUGCAAAUCCAUGCAAAAC-------
------- 4   0,-3
---UGUGCAAAUCCAUGCAAAACU------
------- 169 0,-2
---UGUGCAAAUCCAUGCAAAACUG-----
------- 31 0,-1
---UGUGCAAAUCCAUGCAAAACUGu----
------- 2   0,-1
---UGUGCAAAUCCAUGCAAAACUGA----
------- 14 0,0
--GUGCAAAUCCAUGCAAAACU------
------- 3   1,-2

>hsa-mir-19b-2 mi:19-40;MA:62-84   SRX018958
acauugcuacuuacaauuAGUUUUGCAGGUUUGCAUUUCAgcguauauauguauauguggcUGUGCAAAUCCAUGCAAAA
CUGAuugugauaaugu

(((((....(((((((((((((((((((((((....((((((....))))))....)))))).)).)))))))
)))))))))))))))
--UGUGCAAAUCCAUGCAAAA
C---------------   4   0,-3
--UGUGCAAAUCCAUGCAAAA
CU--------------   169 0,-2
--UGUGCAAAUCCAUGCAAAA
CUG-------------   31 0,-1
--UGUGCAAAUCCAUGCAAAA
CUGu------------   2   0,-1
--UGUGCAAAUCCAUGCAAAA
CUGA------------   14 0,0
---GUGCAAAUCCAUGCAAAA
CU--------------   3   1,-2

>hsa-mir-200a   mi:16-37;MA:54-75   SRX018958
ccgggccccugugagCAUCUUACCGGACAGUGCUGGAuuucccagcuugacucUAACACUGUCUGGUAACGAUGUucaaa
ggugacccgc
.(((((.(((.(((((((((((((((((((((((.....))))).........)))))))))))).)))))))).)
)).).))))).
--CUAACACUGUCUGGUAACGAUGUU----
---------- 2   -1,1
---UAACACUGUCUGGUAACG---------
---------- 12 0,-4
---UAACACUGUCUGGUAACGA--------
---------- 22 0,-3
---UAACACUGUCUGGUAACGAU-------
---------- 187 0,-2
---UAACACUGUCUGGUAACGAUa------
---------- 8   0,-2
---UAACACUGUCUGGUAACGAUG------
---------- 48 0,-1
---UAACACUGUCUGGUAACGAUGa-----
---------- 17 0,-1
---UAACACUGUCUGGUAACGAUGaa----
---------- 7   0,-1
---UAACACUGUCUGGUAACGAUGU-----
---------- 64 0,0

---UAACACUGUCUGGUAACGAUGUa----
---------- 14 0,0
---UAACACUGUCUGGUAACGAUGUaa---
---------- 2   0,0
---UAACACUGUCUGGUAACGAUGUU----
---------- 205 0,1
---UAACACUGUCUGGUAACGAUGUUa---
---------- 20 0,1
---UAACACUGUCUGGUAACGAUGUUaa--
---------- 4   0,1
---UAACACUGUCUGGUAACGAUGUUau--
---------- 2   0,1
---UAACACUGUCUGGUAACGAUGUUu---
---------- 220 0,1
---UAACACUGUCUGGUAACGAUGUUuu--
---------- 8   0,1
---UAACACUGUCUGGUAACGAUGUUC---
---------- 4   0,2
--AACACUGUCUGGUAACGAU-------
---------- 3   1,-2
--AACACUGUCUGGUAACGAUa------
---------- 2   1,-2
--AACACUGUCUGGUAACGAUGUU----
---------- 11 1,1
--AACACUGUCUGGUAACGAUGUUa---
---------- 22 1,1
--AACACUGUCUGGUAACGAUGUUaa--
---------- 2   1,1
--AACACUGUCUGGUAACGAUGUUu---
---------- 29 1,1
--AACACUGUCUGGUAACGAUGUUuu--
---------- 2   1,1

>hsa-mir-200b mi:21-42;MA:57-78 SRX018958
ccagcucgggcagccguggcCAUCUUACUGGGCAGCAUUGGAuggagucaggucucUAAUACUGCCUGGUAAUGAUGAcg
gcggagcccugcacg
...((..((((..((((.(.((((((((..(((((.((((((.((.......)))))))).)))))..)))).)))).).
)))).)))).))...

--------------------CAUCUUACUGGGCAGCAUa--

--------------- 5 0,-4

--------------------CAUCUUACUGGGCAGCAUU--

--------------- 85 0,-3

--------------------CAUCUUACUGGGCAGCAUUG---

--------------- 13 0,-2

--------------------CAUCUUACUGGGCAGCAUUGG--

--------------- 40 0,-1

--------------------CAUCUUACUGGGCAGCAUUGGu---------------------------------------

--------------- 4 0,-1

--------------------CAUCUUACUGGGCAGCAUUGGA---------------------------------------

--------------- 13 0,0

--------------------CAUCUUACUGGGCAGCAUUGGAa--------------------------------------

--------------- 4 0,0

--------------------CAUCUUACUGGGCAGCAUUGGAaa-------------------------------------

--------------- 4 0,0

---------------------AUCUUACUGGGCAGCAUU--

--------------- 8 1,-3

---------------------AUCUUACUGGGCAGCAUUGG--

--------------- 5 1,-1

---------------------AUCUUACUGGGCAGCAUUGGA---------------------------------------

--------------- 2 1,0

---------------------AUCUUACUGGGCAGCAUUGGAa--------------------------------------

--------------- 4 1,0

---------------------AUCUUACUGGGCAGCAUUGGAaa-------------------------------------

--------------- 4 1,0

---------------------AUCUUACUGGGCAGCAUUGGAU--------------------------------------

--------------- 2 1,1

---UAAUACUGCCUGGUAAUG------

--------------- 5 0,-4

---UAAUACUGCCUGGUAAUGA-----

--------------- 19 0,-3

---UAAUACUGCCUGGUAAUGAa----

--------------- 3 0,-3

---UAAUACUGCCUGGUAAUGAU----

--------------- 24 0,-2

---UAAUACUGCCUGGUAAUGAUa---

--------------- 18 0,-2

---UAAUACUGCCUGGUAAUGAUaa--
--------------- 2 0,-2
---UAAUACUGCCUGGUAAUGAUG---
--------------- 44 0,-1
---UAAUACUGCCUGGUAAUGAUGu--
--------------- 15 0,-1
---UAAUACUGCCUGGUAAUGAUGuu-
--------------- 5 0,-1
---UAAUACUGCCUGGUAAUGAUGA--
--------------- 202 0,0
---UAAUACUGCCUGGUAAUGAUGAa-
--------------- 50 0,0
---UAAUACUGCCUGGUAAUGAUGAaa
--------------- 7 0,0
---UAAUACUGCCUGGUAAUGAUGAu-
--------------- 10 0,0
---UAAUACUGCCUGGUAAUGAUGAuu
--------------- 2 0,0
---UAAUACUGCCUGGUAAUGAUGAC-
--------------- 166 0,1
---UAAUACUGCCUGGUAAUGAUGACa
--------------- 32 0,1
---UAAUACUGCCUGGUAAUGAUGACa
u-------------- 2 0,1
---UAAUACUGCCUGGUAAUGAUGACu
--------------- 100 0,1
---UAAUACUGCCUGGUAAUGAUGACu
u-------------- 6 0,1
---UAAUACUGCCUGGUAAUGAUGACG
--------------- 2 0,2
--AAUACUGCCUGGUAAUGAUGA--
--------------- 10 1,0
--AAUACUGCCUGGUAAUGAUGAC-
--------------- 20 1,1
--AAUACUGCCUGGUAAUGAUGACa
--------------- 18 1,1
--AAUACUGCCUGGUAAUGAUGACu
--------------- 35 1,1

--AUACUGCCUGGUAAUGAUG---
--------------- 3   2,-1
--AUACUGCCUGGUAAUGAUGA--
--------------- 10 2,0
--AUACUGCCUGGUAAUGAUGAC-
--------------- 55 2,1
--AUACUGCCUGGUAAUGAUGACa
--------------- 7   2,1
--AUACUGCCUGGUAAUGAUGACu
--------------- 60 2,1
--AUACUGCCUGGUAAUGAUGACu
u-------------- 15 2,1
--AUACUGCCUGGUAAUGAUGACG
--------------- 3   2,2
--AUACUGCCUGGUAAUGAUGACG
G-------------- 2   2,3

>hsa-mir-200c   mi:5-26;MA:44-66   SRX018958
cccuCGUCUUACCCAGCAGUGUUUGGgugcgguugggagucucUAAUACUGCCGGGUAAUGAUGGAgg
((..((((((((((.((((((((.(((.((........))))).)))))))).)))))).))))..))
---UAAUACUGCCGGGUAAUGAU-----   3   0,-3
---UAAUACUGCCGGGUAAUGAUGG---   16 0,-1
---UAAUACUGCCGGGUAAUGAUGGu--   5   0,-1
---UAAUACUGCCGGGUAAUGAUGGA--   19 0,0

>hsa-mir-202   mi:28-49;MA:64-83   SRX018958
cgccucagagccgcccgccguuccuuuUUCCUAUGCAUAUACUUCUUUGaggaucuggccuaaAGAGGUAUAGGGCAUGG
GAAaacggggcggucggguccucccccagcg
(((....(((..((((((((((((.(((((((((((.(((((((((((.(((......))))))))))))))..))))))
))))).))))))).))))).)))....)))
---------------------------UUCCUAUGCAUAUACUUCUUU-------------------------------
------------------------------ 5   0,-1

>hsa-mir-203   3p:65-86   SRX018958
guguuggggacucgcgcgcuggguccagugguucuuaacaguucaacaguucuguagcgcaauuGUGAAAUGUUUAGGAC
CACUAGacccggcgggcgcggcgacagcga
.(((((..(.(.(((.(((((((((.(((((((((.((((.((((.((((((......).)))))))))).)))).)))))
)))).))))))))).))).).)..)))))).

--UGUGAAAUGUUUAGGAC
CACUAG------------------------ 2 -1,0
--GUGAAAUGUUUAGGAC
CAC--------------------------- 3 0,-3
--GUGAAAUGUUUAGGAC
CACU-------------------------- 14 0,-2
--GUGAAAUGUUUAGGAC
CACUA------------------------- 32 0,-1
--GUGAAAUGUUUAGGAC
CACUAu------------------------ 5 0,-1
--GUGAAAUGUUUAGGAC
CACUAG------------------------ 132 0,0
--GUGAAAUGUUUAGGAC
CACUAGu----------------------- 29 0,0
--GUGAAAUGUUUAGGAC
CACUAGA----------------------- 20 0,1
---UGAAAUGUUUAGGAC
CACUA------------------------- 9 1,-1
---UGAAAUGUUUAGGAC
CACUAG------------------------ 22 1,0
---UGAAAUGUUUAGGAC
CACUAGu----------------------- 13 1,0
---UGAAAUGUUUAGGAC
CACUAGuu---------------------- 5 1,0
---UGAAAUGUUUAGGAC
CACUAGA----------------------- 2 1,1
---UGAAAUGUUUAGGAC
CACUAGAa---------------------- 2 1,1
--GAAAUGUUUAGGAC
CACUAG------------------------ 2 2,0

>hsa-mir-204 5p:33-54 SRX018958
ggcuacagucuuucuucaugugacucguggacUUCCCUUUGUCAUCCUAUGCCUgagaauauaugaaggaggcugggaag
gcaaagggacguucaauugucaucacuggc
...............(((((((((...(((((.((((((((((.(((((.((((...............))))))))).)
))))))))).)))))...)))).)).))).
--------------------------------UUCCCUUUGUCAUCCUAUGCC---------------------------
------------------------------ 5 0,-1

--------------------------------UUCCCUUUGUCAUCCUAUGCCU--------------------------
------------------------------ 22 0,0
---GCUGGGAAG
GCAAAGGGA--------------------- 11
---GCUGGGAAG
GCAAAGGGAC-------------------- 7
---GCUGGGAAG
GCAAAGGGACGU------------------ 11

>hsa-mir-206 3p:53-74 SRX018958
ugcuucccgaggccacaugcuucuuuauauccccauauggauuacuuugcuaUGGAAUGUAAGGAAGUGUGUGGuuucgg
caagug
.((((.((((((((((((((((((((((((..(((((.(.(......).)))))).))))))))))))))))))))))))
.)))).
--UGGAAUGUAAGGAAGUGUGUG-------
------ 2 0,-1
--UGGAAUGUAAGGAAGUGUGUGG------
------ 12 0,0
--UGGAAUGUAAGGAAGUGUGUGGU-----
------ 2 0,1

>hsa-mir-208b 3p:46-67 SRX018958
ccucucagggaagcuuuuugcucgaauuauguuucugauccgaauAUAAGACGAACAAAAGGUUUGUcugagggcag
.(((((((.((((((((((.(((..(((((((((.......)))))))))..))).)))))))))).)))))))...
---AUAAGACGAACAAAAGGUUU------------
3 0,-2

>hsa-mir-20a MA:8-30;mi:44-65 SRX018958
guagcacUAAAGUGCUUAUAGUGCAGGUAGuguuuaguuaucuACUGCAUUAUGAGCACUUAAAGuacugc
((((.(((.(((((((((((((((((.(((((......)).))))))))))))))))))))..))).))))
-------UAAAGUGCUUAUAGUGCA-- 11 0,-5
-------UAAAGUGCUUAUAGUGCAG--- 162 0,-4
-------UAAAGUGCUUAUAGUGCAGG-- 77 0,-3
-------UAAAGUGCUUAUAGUGCAGGa--- 2 0,-3
-------UAAAGUGCUUAUAGUGCAGGU--- 65 0,-2
-------UAAAGUGCUUAUAGUGCAGGUu-- 5 0,-2
-------UAAAGUGCUUAUAGUGCAGGUA-- 223 0,-1
-------UAAAGUGCUUAUAGUGCAGGUAa--- 81 0,-1

-------UAAAGUGCUUAUAGUGCAGGUAau-- 2 0,-1
-------UAAAGUGCUUAUAGUGCAGGUAu--- 84 0,-1
-------UAAAGUGCUUAUAGUGCAGGUAuu-- 2 0,-1
-------UAAAGUGCUUAUAGUGCAGGUAG--- 94 0,0
-------UAAAGUGCUUAUAGUGCAGGUAGa-- 8 0,0
-------UAAAGUGCUUAUAGUGCAGGUAGU-- 3 0,1
-------UAAAGUGCUUAUAGUGCAGGUAGUa--------------------------------------- 2 0,1
---------AAGUGCUUAUAGUGCAGGUA-- 2 2,-1
---ACUGCAUUAUGAGCACUUA--------- 5 0,-3
---ACUGCAUUAUGAGCACUUAA-------- 2 0,-2
---ACUGCAUUAUGAGCACUUAAA------- 3 0,-1
---ACUGCAUUAUGAGCACUUAAAG------ 3 0,0
---ACUGCAUUAUGAGCACUUAAAGU----- 8 0,1
---ACUGCAUUAUGAGCACUUAAAGUA---- 4 0,2
--CUGCAUUAUGAGCACUUAAAGU----- 2 1,1

>hsa-mir-20b MA:6-28;mi:44-65 SRX018958
aguacCAAAGUGCUCAUAGUGCAGGUAGuuuuggcaugacucuACUGUAGUAUGGGCACUUCCAGuacu
(((((..(((((((((((.(((((..((((.......))))...))))).)))))))))))...)))))
-----CAAAGUGCUCAUAGUGCAGGU--- 2 0,-2
-----CAAAGUGCUCAUAGUGCAGGUAu--- 2 0,-1
---ACUGUAGUAUGGGCACUUCC------ 2 0,-2
---ACUGUAGUAUGGGCACUUCCA----- 2 0,-1
---ACUGUAGUAUGGGCACUUCCAGU--- 3 0,1

>hsa-mir-21 MA:8-29;mi:46-66 SRX018958
ugucgggUAGCUUAUCAGACUGAUGUUGAcuguugaaucucauggCAACACCAGUCGAUGGGCUGUcugaca
.(((((..((((((((.(((((.(((((.((((.(.....)))))))))).)))))))))))))..))))).
------GUAGCUUAUCAGACUGAUGU-- 2
-1,-3
------GUAGCUUAUCAGACUGAUGUU--- 27
-1,-2
------GUAGCUUAUCAGACUGAUGUUG-- 45
-1,-1
------GUAGCUUAUCAGACUGAUGUUGA--- 51
-1,0
------GUAGCUUAUCAGACUGAUGUUGAC-- 3
-1,1

------GUAGCUUAUCAGACUGAUGUUGACa--- 5
-1,1
------GUAGCUUAUCAGACUGAUGUUGACU--- 3
-1,2
-------UAGCUUAUCAGACUGAUG--- 1802
0,-4
-------UAGCUUAUCAGACUGAUGa-- 3
0,-4
-------UAGCUUAUCAGACUGAUGau--- 13
0,-4
-------UAGCUUAUCAGACUGAUGU-- 5753
0,-3
-------UAGCUUAUCAGACUGAUGUa--- 54
0,-3
-------UAGCUUAUCAGACUGAUGUaa-- 20
0,-3
-------UAGCUUAUCAGACUGAUGUU---
39996 0,-2
-------UAGCUUAUCAGACUGAUGUUa-- 244
0,-2
-------UAGCUUAUCAGACUGAUGUUaa--- 34
0,-2
-------UAGCUUAUCAGACUGAUGUUu-- 44
0,-2
-------UAGCUUAUCAGACUGAUGUUG--
23613 0,-1
-------UAGCUUAUCAGACUGAUGUUGu--- 79
0,-1
-------UAGCUUAUCAGACUGAUGUUGA---
18367 0,0
-------UAGCUUAUCAGACUGAUGUUGAa-- 1053
0,0
-------UAGCUUAUCAGACUGAUGUUGAaa--- 125
0,0
-------UAGCUUAUCAGACUGAUGUUGAau--- 7
0,0
-------UAGCUUAUCAGACUGAUGUUGAu-- 46
0,0

-------UAGCUUAUCAGACUGAUGUUGAuu-- 13
0,0
-------UAGCUUAUCAGACUGAUGUUGAC-- 7182
0,1
-------UAGCUUAUCAGACUGAUGUUGACa--- 3612
0,1
-------UAGCUUAUCAGACUGAUGUUGACaa-- 30
0,1
-------UAGCUUAUCAGACUGAUGUUGACau-- 14
0,1
-------UAGCUUAUCAGACUGAUGUUGACU--- 2000
0,2
-------UAGCUUAUCAGACUGAUGUUGACUa-- 7
0,2
-------UAGCUUAUCAGACUGAUGUUGACUu-- 3
0,2
--------AGCUUAUCAGACUGAUGU-- 23
1,-3
--------AGCUUAUCAGACUGAUGUU--- 178
1,-2
--------AGCUUAUCAGACUGAUGUUa-- 2
1,-2
--------AGCUUAUCAGACUGAUGUUG-- 102
1,-1
--------AGCUUAUCAGACUGAUGUUGu--- 14
1,-1
--------AGCUUAUCAGACUGAUGUUGA--- 260
1,0
--------AGCUUAUCAGACUGAUGUUGAa-- 13
1,0
--------AGCUUAUCAGACUGAUGUUGAau--- 4
1,0
--------AGCUUAUCAGACUGAUGUUGAu-- 3
1,0
--------AGCUUAUCAGACUGAUGUUGAC-- 92
1,1
--------AGCUUAUCAGACUGAUGUUGACa--- 63
1,1

--------AGCUUAUCAGACUGAUGUUGACau-- 4

1,1

--------AGCUUAUCAGACUGAUGUUGACU--- 16

1,2

---------GCUUAUCAGACUGAUGUU--- 48

2,-2

---------GCUUAUCAGACUGAUGUUG-- 48

2,-1

---------GCUUAUCAGACUGAUGUUGA--- 58

2,0

---------GCUUAUCAGACUGAUGUUGAC-- 25

2,1

---------GCUUAUCAGACUGAUGUUGACa--- 6

2,1

---------GCUUAUCAGACUGAUGUUGACau-- 3

2,1

---------GCUUAUCAGACUGAUGUUGACU--- 4

2,2

---------GCUUAUCAGACUGAUGUUGACUa-- 4

2,2

---CAACACCAGUCGAUGGGC--------- 2

0,-3

---CAACACCAGUCGAUGGGCU-------- 30

0,-2

---CAACACCAGUCGAUGGGCUG------- 11

0,-1

---CAACACCAGUCGAUGGGCUGU------ 49

0,0

---CAACACCAGUCGAUGGGCUGUaa---- 82

0,0

---CAACACCAGUCGAUGGGCUGUau---- 48

0,0

---CAACACCAGUCGAUGGGCUGUuu---- 13

0,0

---CAACACCAGUCGAUGGGCUGUC----- 16

0,1

---CAACACCAGUCGAUGGGCUGUCa---- 84

0,1

--CAACACCAGUCGAUGGGCUGUCaa---   4   0,1
--CAACACCAGUCGAUGGGCUGUCau---   19   0,1
--CAACACCAGUCGAUGGGCUGUCU----   66   0,2
--CAACACCAGUCGAUGGGCUGUCUa---   2   0,2
--CAACACCAGUCGAUGGGCUGUCUu---   15   0,2
--CAACACCAGUCGAUGGGCUGUCUuu--   15   0,2
---AACACCAGUCGAUGGGCUG-------   2   1,-1
---AACACCAGUCGAUGGGCUGUCa----   3   1,1
---AACACCAGUCGAUGGGCUGUCU----   11   1,2
---AACACCAGUCGAUGGGCUGUCUau--   3   1,2
---AACACCAGUCGAUGGGCUGUCUu---   3   1,2
---AACACCAGUCGAUGGGCUGUCUuu--   4   1,2

>hsa-mir-210   3p:66-87   SRX018958
acccggcagugccuccaggcgcagggcagccccugcccaccgcacacugcgcugccccagacccaCUGUGCGUGUGACAGCGGCUGAucugugccugggcagcgcgaccc
....((..((((.(((((((((((..(((((.(((..(((((((((.((.((((...))).).)).)))))).))).))).)))))..)))))))))))..))))..)).
---------------------------AGCCCCUGCCCACCGCACACU--   2
--ACUGUGCGUGUGACAGCGG---------------------------   2   -1,-4
--ACUGUGCGUGUGACAGCGGCU-------------------------   6   -1,-2
--ACUGUGCGUGUGACAGCGGCUGAU----------------------   2   -1,1

---CUGUGCGUGUGACAG

CGG-------------------------- 2   0,-4

---CUGUGCGUGUGACAG

CGGCU------------------------ 88 0,-2

---CUGUGCGUGUGACAG

CGGCUGA---------------------- 21 0,0

---CUGUGCGUGUGACAG

CGGCUGAa--------------------- 11 0,0

---CUGUGCGUGUGACAG

CGGCUGAU--------------------- 2   0,1

---CUGUGCGUGUGACAG

CGGCUGAUa-------------------- 2   0,1

---CUGUGCGUGUGACAG

CGGCUGAUu-------------------- 3   0,1

---CUGUGCGUGUGACAG

CGGCUGAUuu------------------- 4   0,1

>hsa-mir-2110   5p:8-29 SRX018958

cagggguUUGGGGAAACGGCCGCUGAGUGaggcgucggcuguguuucucaccgcggucuuuuccucccacucuug

(((((((..(((((((.((((((((((.((.(((.....))).)).))))..)))))).)))))))..)))))))

-------UUGGGGAAACGGCCGCUGAGU--- 2

0,-1

-------UUGGGGAAACGGCCGCUGAGUG-- 8

0,0

-------UUGGGGAAACGGCCGCUGAGUGu--- 12

0,0

-------UUGGGGAAACGGCCGCUGAGUGA--- 29

0,1

-------UUGGGGAAACGGCCGCUGAGUGAa-- 2

0,1

-------UUGGGGAAACGGCCGCUGAGUGAu-- 2

0,1

-------UUGGGGAAACGGCCGCUGAGUGAG-- 10

0,2

-------UUGGGGAAACGGCCGCUGAGUGAGu--- 2

0,2

>hsa-mir-212   3p:71-91   SRX018958

cggggcaccccgcccggacagcgcgccggcaccuuggcucuagacugcuuacugcccgggccgcccucagUAACAGUCUC
CAGUCACGGCCaccgacgccuggccccgcc
..................(((.(((.(((..((.(((((..((((((.((((((...(((...))).)))))))))))).
.))))).))...))).))))))........
------------------------------ACCUUGGCUCUAGACUGCUUACU---------------------------
------------------------------ 2
--UAACAGUCUC
CAGUCACGGCCA------------------ 2   0,1

>hsa-mir-214   mi:30-51;MA:71-92   SRX018958
ggccuggcuggacagaguugucaugugucUGCCUGUCUACACUUGCUGUGCagaacauccgcucaccuguACAGCAGGCA
CAGACAGGCAGUcacaugacaacccagccu
.....((((((.....(((((((((((.((((((((((...((((((((((((.............))))))))))))..
.)))))))))).))))))))))))))))).
--GUACAGCAGGCA
CAGACAGG---------------------- 5   -2,-4
--GUACAGCAGGCA
CAGACAGGCA-------------------- 4   -2,-2
--GUACAGCAGGCA
CAGACAGGCAu------------------- 3   -2,-2
---UACAGCAGGCA
CAGACAG----------------------- 13 -1,-5
---UACAGCAGGCA
CAGACAGa---------------------- 6   -1,-5
---UACAGCAGGCA
CAGACAGaa--------------------- 6   -1,-5
---UACAGCAGGCA
CAGACAGu---------------------- 5   -1,-5
---UACAGCAGGCA
CAGACAGG---------------------- 63 -1,-4
---UACAGCAGGCA
CAGACAGGa--------------------- 10 -1,-4
---UACAGCAGGCA
CAGACAGGaa-------------------- 5   -1,-4
---UACAGCAGGCA
CAGACAGGCA-------------------- 51 -1,-2
---UACAGCAGGCA
CAGACAGGCAa------------------- 81 -1,-2

--UACAGCAGGCA

CAGACAGGCAaa------------------ 18 -1,-2

--UACAGCAGGCA

CAGACAGGCAau------------------ 23 -1,-2

--UACAGCAGGCA

CAGACAGGCAu------------------- 64 -1,-2

--UACAGCAGGCA

CAGACAGGCAuu------------------ 26 -1,-2

--UACAGCAGGCA

CAGACAGGCAG------------------- 10 -1,-1

--UACAGCAGGCA

CAGACAGGCAGa------------------ 36 -1,-1

--UACAGCAGGCA

CAGACAGGCAGaa----------------- 3 -1,-1

--UACAGCAGGCA

CAGACAGGCAGau----------------- 2 -1,-1

--UACAGCAGGCA

CAGACAGGCAGU------------------ 3 -1,0

---ACAGCAGGCA

CAGACAGG---------------------- 62 0,-4

---ACAGCAGGCA

CAGACAGGa--------------------- 4 0,-4

---ACAGCAGGCA

CAGACAGGaa-------------------- 5 0,-4

---ACAGCAGGCA

CAGACAGGCA-------------------- 143 0,-2

---ACAGCAGGCA

CAGACAGGCAa------------------- 63 0,-2

---ACAGCAGGCA

CAGACAGGCAaa------------------ 42 0,-2

---ACAGCAGGCA

CAGACAGGCAau------------------ 18 0,-2

---ACAGCAGGCA

CAGACAGGCAu------------------- 38 0,-2

---ACAGCAGGCA

CAGACAGGCAuu------------------ 9 0,-2

---ACAGCAGGCA

CAGACAGGCAG------------------- 67 0,-1

--ACAGCAGGCA
CAGACAGGCAGa------------------ 87 0,-1
--ACAGCAGGCA
CAGACAGGCAGaa----------------- 19 0,-1
--ACAGCAGGCA
CAGACAGGCAGau----------------- 10 0,-1
--ACAGCAGGCA
CAGACAGGCAGU------------------ 46 0,0
--ACAGCAGGCA
CAGACAGGCAGUa----------------- 12 0,0

>hsa-mir-215 5p:27-47 SRX018958
aucauucagaaaugguauacaggaaaAUGACCUAUGAAUUGACAGACaauauagcugaguuugucugucauuucuuuagg
ccaauauucuguaugacugugcuacuucaa
........(((.((((((((((((.(.((.((((.(((.((((((((((...........)))))))))).)))..))))
.)).).))))))......)))))).)))..
--------------------------AUGACCUAUGAAUUGACAG-----------------------------------
------------------------------ 295 0,-2
--------------------------AUGACCUAUGAAUUGACAGAC---------------------------------
------------------------------ 9 0,0
--------------------------AUGACCUAUGAAUUGACAGACA--------------------------------
------------------------------ 7 0,1
---------------------------UGACCUAUGAAUUGACAG-----------------------------------
------------------------------ 3927 1,-2
---------------------------UGACCUAUGAAUUGACAGu----------------------------------
------------------------------ 34 1,-2
---------------------------UGACCUAUGAAUUGACAGuu---------------------------------
------------------------------ 4 1,-2
---------------------------UGACCUAUGAAUUGACAGA----------------------------------
------------------------------ 98 1,-1
---------------------------UGACCUAUGAAUUGACAGAaa--------------------------------
------------------------------ 6 1,-1
---------------------------UGACCUAUGAAUUGACAGAC---------------------------------
------------------------------ 11 1,0
---------------------------UGACCUAUGAAUUGACAGACA--------------------------------
------------------------------ 81 1,1
---------------------------UGACCUAUGAAUUGACAGACAu-------------------------------
------------------------------ 17 1,1

---------------------------UGACCUAUGAAUUGACAGACAuu-------------------------------
------------------------------ 16 1,1
---------------------------UGACCUAUGAAUUGACAGACAA--------------------------------
------------------------------ 5   1,2
---------------------------UGACCUAUGAAUUGACAGACAAU-------------------------------
------------------------------ 2   1,3
----------------------------GACCUAUGAAUUGACAGA-----------------------------------
------------------------------ 4   2,-1

>hsa-mir-216b   5p:11-32   SRX018958
gcagacuggaAAAUCUCUGCAGGCAAAUGUGAugucacugaggaaaucacacacuuacccguagagauucuacagucuga
ca
.(((((((...(((((((((.((....((((((.((......)).))))))......)).)))))))))...))))))).
..
---------AAAAUCUCUGCAGGCAAAUGU--
-- 4   -1,-2
---------AAAAUCUCUGCAGGCAAAUGUG---
-- 2   -1,-1
----------AAAUCUCUGCAGGCAAAUGUG---
-- 13 0,-1
----------AAAUCUCUGCAGGCAAAUGUGu--
-- 2   0,-1
----------AAAUCUCUGCAGGCAAAUGUGA--
-- 17 0,0

>hsa-mir-217   5p:35-57   SRX018958
aguauaauuauuacauaguuuuugaugucgcagaUACUGCAUCAGGAACUGAUUGGAuaagaaucagucaccaucaguuc
cuaaugcauugccuucagcaucuaaacaag
.................((((..(((((.(.((.((.(((((.((((((((((.((((.........))).).))))))))
)).))))).)).)).).))))).))))...
---------------------------------AUACUGCAUCAGGAACUGAU----------------------------
------------------------------ 3   -1,-4
---------------------------------AUACUGCAUCAGGAACUGAUa---------------------------
------------------------------ 4   -1,-4
---------------------------------AUACUGCAUCAGGAACUGAUU---------------------------
------------------------------ 16 -1,-3
---------------------------------AUACUGCAUCAGGAACUGAUUG--------------------------
------------------------------ 34 -1,-2

---------------------------------AUACUGCAUCAGGAACUGAUUGa------------------------
------------------------------ 2   -1,-2
---------------------------------AUACUGCAUCAGGAACUGAUUGG------------------------
------------------------------ 4   -1,-1
----------------------------------UACUGCAUCAGGAACUGAUUGG------------------------
------------------------------ 10 0,-1
----------------------------------UACUGCAUCAGGAACUGAUUGGA-----------------------
------------------------------ 3   0,0

>hsa-mir-218-1 MA:25-45;mi:68-89   SRX018958
gugauaauguagcgagauuuucugUUGUGCUUGAUCUAACCAUGUgguugcgagguaugaguaaaacAUGGUUCCGUCAA
GCACCAUGGaacgucacgcagcuuucuaca
(((..((.((.(((.(((.((((((.(((((((((..((((((((..((((.........)))).))))))))..)))))
)))).)))))).))).))).))))..))).
------------------------UUGUGCUUGAUCUAACCAUGU-----------------------------------
------------------------------ 4   0,0

>hsa-mir-218-2 MA:25-45;mi:67-88   SRX018958
gaccagucgcugcggggcuuuccuUUGUGCUUGAUCUAACCAUGUgguggaacgauggaaacggaaCAUGGUUCUGUCAA
GCACCGCGgaaagcaccgugcucuccugca
...(((..((.((((.(((((((...(((((((((..((((((((..........((....))..))))))))..)))))
))))...))))))).))))))....)))..
------------------------UUGUGCUUGAUCUAACCAUGU-----------------------------------
------------------------------ 4   0,0

>hsa-mir-219-1 5p:21-41;3p:62-83   SRX018958
ccgccccgggccgcggcuccUGAUUGUCCAAACGCAAUUCUcgagucuauggcuccggccgAGAGUUGAGUCUGGACGUC
CCGagccgccgcccccaaaccucgagcggg
((((.(.((((.(((((((..(((.(((((.((.(((((((((.(((.........)))))))))))).)).))))))))
..))))))).)))).........).)))).
--------------------UGAUUGUCCAAACGCAAU--
------------------------------ 3   0,-3
--------------------UGAUUGUCCAAACGCAAUU---
------------------------------ 3   0,-2
--------------------UGAUUGUCCAAACGCAAUUCUCG-------------------------------------
------------------------------ 2   0,2
---AGAGUUGAGUCUGGACGUC
CCa--------------------------- 3   0,-1

---AGAGUUGAGUCUGGACGUC
CCGu------------------------- 4   0,0

>hsa-mir-219-2 5p:19-39;3p:62-83   SRX018958
acucaggggcuucgccacUGAUUGUCCAAACGCAAUUCUuguacgagucugcggccaaccgAGAAUUGUGGCUGGACAUC
UGUggcugagcuccggg
.(((.(((((((.(((((.(((.(((((..(((((((((((...(.((.....)))...)))))))))))..))))))))
.))))).))))))))))
------------------UGAUUGUCCAAACGCAAU--
-----------------   3   0,-3
------------------UGAUUGUCCAAACGCAAUU---
-----------------   3   0,-2

>hsa-mir-22 mi:15-36;MA:53-74   SRX018958
ggcugagccgcaguAGUUCUUCAGUGGCAAGCUUUAuguccugacccagcuaAAGCUGCCAGUUGAAGAACUGUugcccu
cugcc
(((.(((..((((((((((((((((((((.((((((.((.........))))))))))))).))))))))))))))).))
).)))
--------------AGUUCUUCAGUGGCAAGC--
-----   10 0,-4
--------------AGUUCUUCAGUGGCAAGCU---
-----   71 0,-3
--------------AGUUCUUCAGUGGCAAGCUU--
-----   22 0,-2
--------------AGUUCUUCAGUGGCAAGCUUa---
-----   8   0,-2
--------------AGUUCUUCAGUGGCAAGCUUU---
-----   509 0,-1
--------------AGUUCUUCAGUGGCAAGCUUUu--
-----   32 0,-1
--------------AGUUCUUCAGUGGCAAGCUUUA--
-----   238 0,0
--------------AGUUCUUCAGUGGCAAGCUUUAa---
-----   35 0,0
--------------AGUUCUUCAGUGGCAAGCUUUAU---
-----   32 0,1
---AAAGCUGCCAGUUGAAGAA----------
-----   3   -1,-4

---AAAGCUGCCAGUUGAAGAACU--------
-----	2	-1,-2
---AAAGCUGCCAGUUGAAGAACUGU------
-----	8	-1,0
--AAGCUGCCAGUUGAAGAA----------
-----	2982	0,-4
--AAGCUGCCAGUUGAAGAAa---------
-----	19	0,-4
--AAGCUGCCAGUUGAAGAAaa--------
-----	7	0,-4
--AAGCUGCCAGUUGAAGAAau--------
-----	7	0,-4
--AAGCUGCCAGUUGAAGAAu---------
-----	6	0,-4
--AAGCUGCCAGUUGAAGAAuu--------
-----	166	0,-4
--AAGCUGCCAGUUGAAGAAC---------
-----	1227	0,-3
--AAGCUGCCAGUUGAAGAACa--------
-----	207	0,-3
--AAGCUGCCAGUUGAAGAACaa-------
-----	42	0,-3
--AAGCUGCCAGUUGAAGAACau-------
-----	7	0,-3
--AAGCUGCCAGUUGAAGAACU--------
-----	4072	0,-2
--AAGCUGCCAGUUGAAGAACUa-------
-----	47	0,-2
--AAGCUGCCAGUUGAAGAACUaa------
-----	39	0,-2
--AAGCUGCCAGUUGAAGAACUau------
-----	56	0,-2
--AAGCUGCCAGUUGAAGAACUu-------
-----	67	0,-2
--AAGCUGCCAGUUGAAGAACUuu------
-----	224	0,-2
--AAGCUGCCAGUUGAAGAACUG-------
-----	690	0,-1

--AAGCUGCCAGUUGAAGAACUGa------
-----	38	0,-1
--AAGCUGCCAGUUGAAGAACUGaa-----
-----	4	0,-1
--AAGCUGCCAGUUGAAGAACUGau-----
-----	2	0,-1
--AAGCUGCCAGUUGAAGAACUGU------
-----	3331	0,0
--AAGCUGCCAGUUGAAGAACUGUa-----
-----	72	0,0
--AAGCUGCCAGUUGAAGAACUGUaa----
-----	36	0,0
--AAGCUGCCAGUUGAAGAACUGUau----
-----	35	0,0
--AAGCUGCCAGUUGAAGAACUGUU-----
-----	140	0,1
--AAGCUGCCAGUUGAAGAACUGUUa----
-----	13	0,1
--AAGCUGCCAGUUGAAGAACUGUUau---
-----	4	0,1
--AAGCUGCCAGUUGAAGAACUGUUu----
-----	161	0,1
--AAGCUGCCAGUUGAAGAACUGUUuu---
-----	12	0,1
---AGCUGCCAGUUGAAGAAC---------
-----	14	1,-3
---AGCUGCCAGUUGAAGAACU--------
-----	25	1,-2
---AGCUGCCAGUUGAAGAACUG-------
-----	9	1,-1
---AGCUGCCAGUUGAAGAACUGU------
-----	34	1,0
---AGCUGCCAGUUGAAGAACUGUU-----
-----	3	1,1
---AGCUGCCAGUUGAAGAACUGUUu----
-----	2	1,1
--GCUGCCAGUUGAAGAACUGUUu----
-----	2	2,1

>hsa-mir-221 mi:25-46;MA:65-87 SRX018958
ugaacauccaggucuggggcaugaACCUGGCAUACAAUGUAGAUUUcuguguucguuaggcaacAGCUACAUUGUCUGCU
GGGUUUCaggcuaccuggaaacauguucuc
.((((((((((((....(((.((((((..(((.(((((((((....(((((((.....))).))))))))))))).))).
.)).)))).))))))))))....)))))..
-----------------------AACCUGGCAUACAAUGUAGAUU-----------------------------------
------------------------------ 10 -1,-1
-----------------------AACCUGGCAUACAAUGUAGAUUa----------------------------------
------------------------------ 5 -1,-1
-----------------------AACCUGGCAUACAAUGUAGAUUU----------------------------------
------------------------------ 15 -1,0
-----------------------AACCUGGCAUACAAUGUAGAUUUC---------------------------------
------------------------------ 2 -1,1
------------------------ACCUGGCAUACAAUGUAG--------------------------------------
------------------------------ 7 0,-4
------------------------ACCUGGCAUACAAUGUAGA-------------------------------------
------------------------------ 2 0,-3
------------------------ACCUGGCAUACAAUGUAGAa------------------------------------
------------------------------ 2 0,-3
------------------------ACCUGGCAUACAAUGUAGAaa-----------------------------------
------------------------------ 2 0,-3
------------------------ACCUGGCAUACAAUGUAGAU------------------------------------
------------------------------ 27 0,-2
------------------------ACCUGGCAUACAAUGUAGAUa-----------------------------------
------------------------------ 33 0,-2
------------------------ACCUGGCAUACAAUGUAGAUaa----------------------------------
------------------------------ 3 0,-2
------------------------ACCUGGCAUACAAUGUAGAUau----------------------------------
------------------------------ 2 0,-2
------------------------ACCUGGCAUACAAUGUAGAUU-----------------------------------
------------------------------ 143 0,-1
------------------------ACCUGGCAUACAAUGUAGAUUa----------------------------------
------------------------------ 13 0,-1
------------------------ACCUGGCAUACAAUGUAGAUUU----------------------------------
------------------------------ 1137 0,0
------------------------ACCUGGCAUACAAUGUAGAUUUa---------------------------------
------------------------------ 18 0,0

-----------------------ACCUGGCAUACAAUGUAGAUUUu---------------------------------
----------------------------- 23   0,0
-----------------------ACCUGGCAUACAAUGUAGAUUUC---------------------------------
----------------------------- 170 0,1
-----------------------ACCUGGCAUACAAUGUAGAUUUCU--------------------------------
----------------------------- 97   0,2
-----------------------ACCUGGCAUACAAUGUAGAUUUCUG-------------------------------
----------------------------- 2   0,3
------------------------CCUGGCAUACAAUGUAGAUUU----------------------------------
----------------------------- 10   1,0
-------------------------CUGGCAUACAAUGUAGAUU-----------------------------------
----------------------------- 2   2,-1
---AGCUACAUUGUCUGCU
GG--------------------------- 8   0,-5
---AGCUACAUUGUCUGCU
GGG-------------------------- 118 0,-4
---AGCUACAUUGUCUGCU
GGGU------------------------- 41   0,-3
---AGCUACAUUGUCUGCU
GGGUa------------------------ 3   0,-3
---AGCUACAUUGUCUGCU
GGGUaa----------------------- 9   0,-3
---AGCUACAUUGUCUGCU
GGGUau----------------------- 11   0,-3
---AGCUACAUUGUCUGCU
GGGUU------------------------ 116 0,-2
---AGCUACAUUGUCUGCU
GGGUUa----------------------- 54   0,-2
---AGCUACAUUGUCUGCU
GGGUUaa---------------------- 18   0,-2
---AGCUACAUUGUCUGCU
GGGUUau---------------------- 47   0,-2
---AGCUACAUUGUCUGCU
GGGUUU----------------------- 1326   0,-1
---AGCUACAUUGUCUGCU
GGGUUUa---------------------- 161 0,-1
---AGCUACAUUGUCUGCU
GGGUUUaa--------------------- 17   0,-1

--AGCUACAUUGUCUGCU
GGGUUUau---------------------- 30 0,-1
--AGCUACAUUGUCUGCU
GGGUUUu----------------------- 324 0,-1
--AGCUACAUUGUCUGCU
GGGUUUuu---------------------- 23 0,-1
--AGCUACAUUGUCUGCU
GGGUUUC----------------------- 524 0,0
--AGCUACAUUGUCUGCU
GGGUUUCu---------------------- 42 0,0
--AGCUACAUUGUCUGCU
GGGUUUCA---------------------- 21 0,1
---GCUACAUUGUCUGCU
GGGUUa------------------------ 2   1,-2
---GCUACAUUGUCUGCU
GGGUUU------------------------ 49 1,-1
---GCUACAUUGUCUGCU
GGGUUUu----------------------- 7   1,-1
---GCUACAUUGUCUGCU
GGGUUUC----------------------- 10 1,0

>hsa-mir-222   mi:31-52;MA:69-89   SRX018958
gcugcuggaagguguagguacccucaauggCUCAGUAGCCAGUGUAGAUCCUgucuuucguaaucagcAGCUACAUCUGG
CUACUGGGUcucugauggcaucuucuagcu
...((((((((((......((.(((..((((((((((((((((((((...(((...........)))...))))).))))
)))))))))))..))).)))))))))))).
--AGCUACAUCUGG
CUACUG------------------------ 389 0,-3
--AGCUACAUCUGG
CUACUGG----------------------- 518 0,-2
--AGCUACAUCUGG
CUACUGGu---------------------- 2   0,-2
--AGCUACAUCUGG
CUACUGGG---------------------- 509 0,-1
--AGCUACAUCUGG
CUACUGGGa--------------------- 6   0,-1
--AGCUACAUCUGG
CUACUGGGU--------------------- 607 0,0

---AGCUACAUCUGG

CUACUGGGUa------------------- 35 0,0

---AGCUACAUCUGG

CUACUGGGUaa------------------ 51 0,0

---AGCUACAUCUGG

CUACUGGGUau------------------ 56 0,0

---AGCUACAUCUGG

CUACUGGGUu------------------- 36 0,0

---AGCUACAUCUGG

CUACUGGGUuu------------------ 18 0,0

---AGCUACAUCUGG

CUACUGGGUC------------------- 2471 0,1

---AGCUACAUCUGG

CUACUGGGUCa------------------ 24 0,1

---AGCUACAUCUGG

CUACUGGGUCaa----------------- 5 0,1

---AGCUACAUCUGG

CUACUGGGUCau----------------- 3 0,1

---AGCUACAUCUGG

CUACUGGGUCU------------------ 2074 0,2

---AGCUACAUCUGG

CUACUGGGUCUa----------------- 16 0,2

---AGCUACAUCUGG

CUACUGGGUCUaa---------------- 5 0,2

---AGCUACAUCUGG

CUACUGGGUCUau---------------- 12 0,2

---AGCUACAUCUGG

CUACUGGGUCUu----------------- 79 0,2

---AGCUACAUCUGG

CUACUGGGUCUuu---------------- 59 0,2

---AGCUACAUCUGG

CUACUGGGUCUC----------------- 234 0,3

---AGCUACAUCUGG

CUACUGGGUCUCa---------------- 35 0,3

---AGCUACAUCUGG

CUACUGGGUCUCU---------------- 90 0,4

--GCUACAUCUGG

CUACUGG---------------------- 2 1,-2

---GCUACAUCUGG
CUACUGGG---------------------- 2   1,-1
---GCUACAUCUGG
CUACUGGGU--------------------- 2   1,0
---GCUACAUCUGG
CUACUGGGUC-------------------- 8   1,1
---GCUACAUCUGG
CUACUGGGUCU------------------- 13 1,2
---GCUACAUCUGG
CUACUGGGUCUuu----------------- 2   1,2

>hsa-mir-223   mi:26-47;MA:68-89   SRX018958
ccuggccuccugcagugccacgcucCGUGUAUUUGACAAGCUGAGUUggacacuccaugugguagagUGUCAGUUUGUCA
AAUACCCCAagugcggcacaugcuuaccag
.((((......(((((((.((((..(.((((((((((((((......((((((((.........))))))))))))))))
))))).)..)))).))))).)))...))))
-------------------------GUGUAUUUGACAAGCUGAGUUG---------------------------------
------------------------------ 2   1,1
---UGUCAGUUUGUCA
AAUAC------------------------- 3   0,-4
---UGUCAGUUUGUCA
AAUACC------------------------ 4   0,-3
---UGUCAGUUUGUCA
AAUACCC----------------------- 3   0,-2
---UGUCAGUUUGUCA
AAUACCCC---------------------- 2   0,-1
---UGUCAGUUUGUCA
AAUACCCCA--------------------- 18 0,0
---UGUCAGUUUGUCA
AAUACCCCAu-------------------- 4   0,0
---UGUCAGUUUGUCA
AAUACCCCAA-------------------- 14 0,1
---UGUCAGUUUGUCA
AAUACCCCAAa------------------- 3   0,1
--GUCAGUUUGUCA
AAUACC------------------------ 8   1,-3
--GUCAGUUUGUCA
AAUACCC----------------------- 5   1,-2

--GUCAGUUUGUCA
AAUACCCC--------------------- 4 1,-1
--GUCAGUUUGUCA
AAUACCCCA-------------------- 16 1,0
--GUCAGUUUGUCA
AAUACCCCAA------------------- 23 1,1
--GUCAGUUUGUCA
AAUACCCCAAa------------------ 2 1,1
--GUCAGUUUGUCA
AAUACCCCAAu------------------ 5 1,1

>hsa-mir-224 MA:8-28;mi:53-75 SRX018958
gggcuuuCAAGUCACUAGUGGUUCCGUUuaguagaugauugugcauuguuucAAAAUGGUGCCCUAGUGACUACAaagcc
c
(((((((..(((((((((.(((.((((((.(.(((..((.....))..)))).)))))).))))))))))))..))))))
)
-------CAAGUCACUAGUGGUUCCGUUU---
- 16 0,1
-------CAAGUCACUAGUGGUUCCGUUUA--
- 6 0,2
-------CAAGUCACUAGUGGUUCCGUUUAa---
- 2 0,2
-------CAAGUCACUAGUGGUUCCGUUUAG---
- 39 0,3
--------AAGUCACUAGUGGUUCCGUUUAG---
- 2 1,3
---AAAUGGUGCCCUAGUGAC---------
- 2 1,-4
---AAAUGGUGCCCUAGUGACUAC------
- 4 1,-1

>hsa-mir-2355 5p:11-31;3p:54-75 SRX018958
cagacgugucAUCCCCAGAUACAAUGGACAAuaugcuauuauaaucguauggcAUUGUCCUUGCUGUUUGGAGAUaauac
ugcugac
(((..((((.(((.((((((((((.(((((..(((((((.........))))))))))))))).))))))).))).))))
..)))..
----------AUCCCCAGAUACAAUGGACAAau---
------- 2 0,0

----------AUCCCCAGAUACAAUGGACAAU---
------- 15 0,1

>hsa-mir-23a mi:9-30;MA:45-65 SRX018958
ggccggcuGGGGUUCCUGGGGAUGGGAUUUgcuuccugucacaaAUCACAUUGCCAGGGAUUUCCaaccgacc
((.(((.((((((((((((.((((.((((((..........)))))).)))).))))))).))))).))).))
---AAUCACAUUGCCAGGGAUU----------- 4
-1,-3
---AAUCACAUUGCCAGGGAUUU---------- 6
-1,-2
---AAUCACAUUGCCAGGGAUUUC--------- 3
-1,-1
---AAUCACAUUGCCAGGGAUUUCC-------- 5
-1,0
---AAUCACAUUGCCAGGGAUUUCCA------- 4
-1,1
--AUCACAUUGCCAGGGAUU----------- 145
0,-3
--AUCACAUUGCCAGGGAUUU---------- 1279
0,-2
--AUCACAUUGCCAGGGAUUUa--------- 181
0,-2
--AUCACAUUGCCAGGGAUUUaa-------- 50
0,-2
--AUCACAUUGCCAGGGAUUUau-------- 9
0,-2
--AUCACAUUGCCAGGGAUUUu--------- 106
0,-2
--AUCACAUUGCCAGGGAUUUC--------- 1005
0,-1
--AUCACAUUGCCAGGGAUUUCa-------- 218
0,-1
--AUCACAUUGCCAGGGAUUUCaa------- 24
0,-1
--AUCACAUUGCCAGGGAUUUCu-------- 25
0,-1
--AUCACAUUGCCAGGGAUUUCC-------- 651
0,0

--AUCACAUUGCCAGGGAUUUCCu------- 9

0,0

--AUCACAUUGCCAGGGAUUUCCA------- 2981

0,1

--AUCACAUUGCCAGGGAUUUCCAu------ 319

0,1

--AUCACAUUGCCAGGGAUUUCCAuu----- 20

0,1

--AUCACAUUGCCAGGGAUUUCCAA------ 437

0,2

--AUCACAUUGCCAGGGAUUUCCAAa----- 8

0,2

--AUCACAUUGCCAGGGAUUUCCAAu----- 35

0,2

--AUCACAUUGCCAGGGAUUUCCAAuu---- 5

0,2

--AUCACAUUGCCAGGGAUUUCCAAC----- 2

0,3

---UCACAUUGCCAGGGAUUUa--------- 2

1,-2

---UCACAUUGCCAGGGAUUUu--------- 4

1,-2

---UCACAUUGCCAGGGAUUUC--------- 7

1,-1

---UCACAUUGCCAGGGAUUUCa-------- 4

1,-1

---UCACAUUGCCAGGGAUUUCC-------- 2

1,0

---UCACAUUGCCAGGGAUUUCCA------- 9

1,1

---UCACAUUGCCAGGGAUUUCCAu------ 3

1,1

---UCACAUUGCCAGGGAUUUCCAA------ 7

1,2

---UCACAUUGCCAGGGAUUUCCAAu----- 4

1,2

---UCACAUUGCCAGGGAUUUCCAAC----- 2

1,3

--UCACAUUGCCAGGGAUUUCCAACu----   3
1,3

>hsa-mir-23b   mi:20-41;MA:58-78   SRX018958
cucaggugcucuggcugcuUGGGUUCCUGGCAUGCUGAUUUgugacuuaagauuaaaAUCACAUUGCCAGGGAUUACCac
gcaaccacgaccuuggc
.(((((.(.(((((.(((.(((((((((((((((.((((((..............)))))))).))))))))))..))).
))).))).)))))))).
------------------UUGGGUUCCUGGCAUGCUGAU---
-----------------   4   -1,-2
------------------UUGGGUUCCUGGCAUGCUGAUU--
-----------------   13   -1,-1
------------------UUGGGUUCCUGGCAUGCUGAUUa---------------------------------------
-----------------   3   -1,-1
------------------UUGGGUUCCUGGCAUGCUGAUUU---------------------------------------
-----------------   26   -1,0
------------------UUGGGUUCCUGGCAUGCUGAUUUu--------------------------------------
-----------------   4   -1,0
-------------------UGGGUUCCUGGCAUGCUGA--
-----------------   2   0,-3
-------------------UGGGUUCCUGGCAUGCUGAU---
-----------------   18   0,-2
-------------------UGGGUUCCUGGCAUGCUGAUa--
-----------------   25   0,-2
-------------------UGGGUUCCUGGCAUGCUGAUU--
-----------------   84   0,-1
-------------------UGGGUUCCUGGCAUGCUGAUUa---------------------------------------
-----------------   15   0,-1
-------------------UGGGUUCCUGGCAUGCUGAUUaa--------------------------------------
-----------------   2   0,-1
-------------------UGGGUUCCUGGCAUGCUGAUUau--------------------------------------
-----------------   2   0,-1
-------------------UGGGUUCCUGGCAUGCUGAUUU---------------------------------------
-----------------   607   0,0
-------------------UGGGUUCCUGGCAUGCUGAUUUa--------------------------------------
-----------------   42   0,0
-------------------UGGGUUCCUGGCAUGCUGAUUUau-------------------------------------
-----------------   2   0,0

-------------------UGGGUUCCUGGCAUGCUGAUUUu---------------------------------------

----------------- 84 0,0

--------------------GGGUUCCUGGCAUGCUGA---

----------------- 5 1,-3

--------------------GGGUUCCUGGCAUGCUGAU--

----------------- 27 1,-2

--------------------GGGUUCCUGGCAUGCUGAUa---

----------------- 6 1,-2

--------------------GGGUUCCUGGCAUGCUGAUU---

----------------- 73 1,-1

--------------------GGGUUCCUGGCAUGCUGAUUa--

----------------- 3 1,-1

--------------------GGGUUCCUGGCAUGCUGAUUaa---------------------------------------

----------------- 2 1,-1

--------------------GGGUUCCUGGCAUGCUGAUUU--

----------------- 537 1,0

--------------------GGGUUCCUGGCAUGCUGAUUUa---------------------------------------

----------------- 22 1,0

--------------------GGGUUCCUGGCAUGCUGAUUUaa--------------------------------------

----------------- 9 1,0

--------------------GGGUUCCUGGCAUGCUGAUUUau--------------------------------------

----------------- 9 1,0

--------------------GGGUUCCUGGCAUGCUGAUUUu---------------------------------------

----------------- 174 1,0

--------------------GGGUUCCUGGCAUGCUGAUUUuu--------------------------------------

----------------- 22 1,0

--------------------GGGUUCCUGGCAUGCUGAUUUGU--------------------------------------

----------------- 9 1,2

---AAUCACAUUGCCAGGGAUU-----

----------------- 4 -1,-3

---AAUCACAUUGCCAGGGAUUA----

----------------- 10 -1,-2

---AAUCACAUUGCCAGGGAUUAC---

----------------- 6 -1,-1

---AAUCACAUUGCCAGGGAUUACa--

----------------- 2 -1,-1

---AAUCACAUUGCCAGGGAUUACC--

----------------- 24 -1,0

---AAUCACAUUGCCAGGGAUUACCA-

-----------------   7   -1,1

---AAUCACAUUGCCAGGGAUUACCAC

-----------------   3   -1,2

--AUCACAUUGCCAGGGAUU-----

-----------------   145 0,-3

--AUCACAUUGCCAGGGAUUA----

-----------------   927 0,-2

--AUCACAUUGCCAGGGAUUAa---

-----------------   237 0,-2

--AUCACAUUGCCAGGGAUUAaa--

-----------------   40   0,-2

--AUCACAUUGCCAGGGAUUAau--

-----------------   4   0,-2

--AUCACAUUGCCAGGGAUUAu---

-----------------   83   0,-2

--AUCACAUUGCCAGGGAUUAuu--

-----------------   12   0,-2

--AUCACAUUGCCAGGGAUUAC---

-----------------   919 0,-1

--AUCACAUUGCCAGGGAUUACa--

-----------------   291 0,-1

--AUCACAUUGCCAGGGAUUACaa-

-----------------   34   0,-1

--AUCACAUUGCCAGGGAUUACau-

-----------------   2   0,-1

--AUCACAUUGCCAGGGAUUACu--

-----------------   55   0,-1

--AUCACAUUGCCAGGGAUUACuu-

-----------------   3   0,-1

--AUCACAUUGCCAGGGAUUACC--

-----------------   1412   0,0

--AUCACAUUGCCAGGGAUUACCu-

-----------------   48   0,0

--AUCACAUUGCCAGGGAUUACCuu

-----------------   14   0,0

--AUCACAUUGCCAGGGAUUACCA-

-----------------   5120   0,1

--AUCACAUUGCCAGGGAUUACCAa
-----------------   190 0,1
--AUCACAUUGCCAGGGAUUACCAa
u----------------   11   0,1
--AUCACAUUGCCAGGGAUUACCAu
-----------------   945 0,1
--AUCACAUUGCCAGGGAUUACCAu
u----------------   249 0,1
--AUCACAUUGCCAGGGAUUACCAC
-----------------   1288   0,2
--AUCACAUUGCCAGGGAUUACCAC
a----------------   230 0,2
--AUCACAUUGCCAGGGAUUACCAC
aa---------------   6   0,2
--AUCACAUUGCCAGGGAUUACCAC
au---------------   12   0,2
--AUCACAUUGCCAGGGAUUACCAC
u----------------   3536   0,2
--AUCACAUUGCCAGGGAUUACCAC
uu---------------   51   0,2
--AUCACAUUGCCAGGGAUUACCAC
G----------------   15   0,3
--AUCACAUUGCCAGGGAUUACCAC
Gu---------------   3   0,3
---UCACAUUGCCAGGGAUUA----
-----------------   14   1,-2
---UCACAUUGCCAGGGAUUAa---
-----------------   3   1,-2
---UCACAUUGCCAGGGAUUAu---
-----------------   3   1,-2
---UCACAUUGCCAGGGAUUAC---
-----------------   20   1,-1
---UCACAUUGCCAGGGAUUACa--
-----------------   7   1,-1
---UCACAUUGCCAGGGAUUACC--
-----------------   28   1,0
---UCACAUUGCCAGGGAUUACCA-
-----------------   86   1,1

--UCACAUUGCCAGGGAUUACCAa
-----------------	8	1,1
--UCACAUUGCCAGGGAUUACCAu
-----------------	6	1,1
--UCACAUUGCCAGGGAUUACCAu
u----------------	45	1,1
--UCACAUUGCCAGGGAUUACCAC
-----------------	54	1,2
--UCACAUUGCCAGGGAUUACCAC
a----------------	34	1,2
--UCACAUUGCCAGGGAUUACCAC
aa---------------	3	1,2
--UCACAUUGCCAGGGAUUACCAC
u----------------	278	1,2
--UCACAUUGCCAGGGAUUACCAC
uu---------------	47	1,2
---CACAUUGCCAGGGAUUACCAC
u----------------	2	2,2

>hsa-mir-23c	3p:66-87	SRX018958
agugacuuuccaggugucacacagugaguggcauaaucagaguacaauuugagucaugcccauacAUCACAUUGCCAGUG
AUUACCCaaggaaagugacg
.((.(((((((.((((((((.(((((((((((((..(((((......)))))...)))))...)).)))))..))...)))
).))))...))))))).)).
---AUCACAUUGCCAGUG
AUUACCa-------------	4	0,-1

>hsa-mir-24-1	mi:7-28;MA:44-65	SRX018958
cuccggUGCCUACUGAGCUGAUAUCAGUucucauuuuacacacUGGCUCAGUUCAGCAGGAACAGgag
((((.((.(((.(((((((((..(((((...............))))).))))))))).))).)).))))
----GGUGCCUACUGAGCUGAUAUCA--	5
-2,-2
-----GUGCCUACUGAGCUGAUAU--	2
-1,-4
-----GUGCCUACUGAGCUGAUAUa---	4
-1,-4
-----GUGCCUACUGAGCUGAUAUC---	2
-1,-3

-----GUGCCUACUGAGCUGAUAUCA-- 27
-1,-2
-----GUGCCUACUGAGCUGAUAUCAG--------------------------------------- 9
-1,-1
--CUGGCUCAGUUCAGCAGGAAC----- 2
-1,-2
---UGGCUCAGUUCAGCAGGA------- 8 0,-4
---UGGCUCAGUUCAGCAGGAA------ 61 0,-3
---UGGCUCAGUUCAGCAGGAAa----- 4 0,-3
---UGGCUCAGUUCAGCAGGAAC----- 32 0,-2
---UGGCUCAGUUCAGCAGGAACA---- 110 0,-1
---UGGCUCAGUUCAGCAGGAACAa--- 42 0,-1
---UGGCUCAGUUCAGCAGGAACAaa-- 7 0,-1
---UGGCUCAGUUCAGCAGGAACAau-- 2 0,-1
---UGGCUCAGUUCAGCAGGAACAu--- 21 0,-1
---UGGCUCAGUUCAGCAGGAACAuu-- 20 0,-1
---UGGCUCAGUUCAGCAGGAACAG--- 1019
0,0
---UGGCUCAGUUCAGCAGGAACAGa-- 28 0,0
---UGGCUCAGUUCAGCAGGAACAGu-- 1619
0,0
---UGGCUCAGUUCAGCAGGAACAGuu- 325 0,0
---UGGCUCAGUUCAGCAGGAACAGG-- 6 0,1
---UGGCUCAGUUCAGCAGGAACAGGu- 2 0,1
--GGCUCAGUUCAGCAGGAA------ 11 1,-3
--GGCUCAGUUCAGCAGGAAC----- 2 1,-2
--GGCUCAGUUCAGCAGGAACA---- 5 1,-1
--GGCUCAGUUCAGCAGGAACAa--- 7 1,-1
--GGCUCAGUUCAGCAGGAACAu--- 4 1,-1
--GGCUCAGUUCAGCAGGAACAG--- 43 1,0
--GGCUCAGUUCAGCAGGAACAGa-- 5 1,0
--GGCUCAGUUCAGCAGGAACAGu-- 107 1,0
--GGCUCAGUUCAGCAGGAACAGuu- 16 1,0
---GCUCAGUUCAGCAGGAACAG--- 32 2,0
---GCUCAGUUCAGCAGGAACAGa-- 4 2,0
---GCUCAGUUCAGCAGGAACAGu-- 18 2,0
---GCUCAGUUCAGCAGGAACAGuu- 15 2,0
---GCUCAGUUCAGCAGGAACAGG-- 18 2,1

---GCUCAGUUCAGCAGGAACAGGA- 3 2,2

>hsa-mir-24-2 mi:13-34;MA:50-71 SRX018958
cucugccucccgUGCCUACUGAGCUGAAACACAGuugguuuguguacacUGGCUCAGUUCAGCAGGAACAGgg
(((((..(((..(((..(((((((((..((((((.....))))))....)))))))))...)))))).)))))
-----------GUGCCUACUGAGCUGAAACA-- 3
-1,-3
-----------GUGCCUACUGAGCUGAAACAC--- 37
-1,-2
-----------GUGCCUACUGAGCUGAAACACA-- 28
-1,-1
-----------GUGCCUACUGAGCUGAAACACAG--------------------------------------- 28
-1,0
-----------GUGCCUACUGAGCUGAAACACAGU-------------------------------------- 4
-1,1
--CUGGCUCAGUUCAGCAGGAAC---- 2
-1,-2
---UGGCUCAGUUCAGCAGGA------ 8
0,-4
---UGGCUCAGUUCAGCAGGAA----- 61
0,-3
---UGGCUCAGUUCAGCAGGAAa---- 4
0,-3
---UGGCUCAGUUCAGCAGGAAC---- 32
0,-2
---UGGCUCAGUUCAGCAGGAACA--- 110
0,-1
---UGGCUCAGUUCAGCAGGAACAa-- 42
0,-1
---UGGCUCAGUUCAGCAGGAACAaa- 7
0,-1
---UGGCUCAGUUCAGCAGGAACAau- 2
0,-1
---UGGCUCAGUUCAGCAGGAACAu-- 21
0,-1
---UGGCUCAGUUCAGCAGGAACAuu- 20
0,-1
---UGGCUCAGUUCAGCAGGAACAG-- 1019

0,0
---UGGCUCAGUUCAGCAGGAACAGa- 28
0,0
---UGGCUCAGUUCAGCAGGAACAGu- 1619
0,0
---UGGCUCAGUUCAGCAGGAACAGuu 325
0,0
---UGGCUCAGUUCAGCAGGAACAGG- 6
0,1
---UGGCUCAGUUCAGCAGGAACAGGu 2
0,1
--GGCUCAGUUCAGCAGGAA----- 11
1,-3
--GGCUCAGUUCAGCAGGAAC---- 2
1,-2
--GGCUCAGUUCAGCAGGAACA--- 5
1,-1
--GGCUCAGUUCAGCAGGAACAa-- 7
1,-1
--GGCUCAGUUCAGCAGGAACAu-- 4
1,-1
--GGCUCAGUUCAGCAGGAACAG-- 43
1,0
--GGCUCAGUUCAGCAGGAACAGa- 5
1,0
--GGCUCAGUUCAGCAGGAACAGu- 107
1,0
--GGCUCAGUUCAGCAGGAACAGuu 16
1,0
---GCUCAGUUCAGCAGGAACAG-- 32
2,0
---GCUCAGUUCAGCAGGAACAGa- 4
2,0
---GCUCAGUUCAGCAGGAACAGu- 18
2,0
---GCUCAGUUCAGCAGGAACAGuu 15
2,0
---GCUCAGUUCAGCAGGAACAGG- 18

2,1

>hsa-mir-25 mi:14-34;MA:52-73 SRX018958
ggccaguguugagAGGCGGAGACUUGGGCAAUUGcuggacgcugcccugggCAUUGCACUUGUCUCGGUCUGAcagugcc
ggcc
((((.(..(((..((((.(((((..(.(((((.(((((.......))..)))))))).)..))))).))))..)))..).
))))
-------------AGGCGGAGACUUGGGCAAUa---
---- 2 0,-2
-------------AGGCGGAGACUUGGGCAAUU---
---- 12 0,-1
-------------AGGCGGAGACUUGGGCAAUUG--
---- 24 0,0
-------------AGGCGGAGACUUGGGCAAUUGa---
---- 3 0,0
-------------AGGCGGAGACUUGGGCAAUUGC---
---- 50 0,1
-------------AGGCGGAGACUUGGGCAAUUGCa--
---- 2 0,1
-------------AGGCGGAGACUUGGGCAAUUGCU--
---- 228 0,2
-------------AGGCGGAGACUUGGGCAAUUGCUa---
---- 4 0,2
-------------AGGCGGAGACUUGGGCAAUUGCUu---
---- 13 0,2
-------------AGGCGGAGACUUGGGCAAUUGCUuu--
---- 2 0,2
---CAUUGCACUUGUCUCGGU-----------
---- 88 0,-4
---CAUUGCACUUGUCUCGGUuu---------
---- 2 0,-4
---CAUUGCACUUGUCUCGGUC----------
---- 233 0,-3
---CAUUGCACUUGUCUCGGUCU---------
---- 503 0,-2
---CAUUGCACUUGUCUCGGUCUa--------
---- 3 0,-2
---CAUUGCACUUGUCUCGGUCUu--------

```
----    2    0,-2
-----------------------------------------------------CAUUGCACUUGUCUCGGUCUG--------
----    293  0,-1
-----------------------------------------------------CAUUGCACUUGUCUCGGUCUGu-------
----    73   0,-1
-----------------------------------------------------CAUUGCACUUGUCUCGGUCUGuu------
----    14   0,-1
-----------------------------------------------------CAUUGCACUUGUCUCGGUCUGA-------
----    983  0,0
-----------------------------------------------------CAUUGCACUUGUCUCGGUCUGAa------
----    48   0,0
-----------------------------------------------------CAUUGCACUUGUCUCGGUCUGAaa-----
----    4    0,0
-----------------------------------------------------CAUUGCACUUGUCUCGGUCUGAu------
----    31   0,0
-----------------------------------------------------CAUUGCACUUGUCUCGGUCUGAC------
----    3    0,1
------------------------------------------------------AUUGCACUUGUCUCGGUC----------
----    9    1,-3
------------------------------------------------------AUUGCACUUGUCUCGGUCU---------
----    33   1,-2
------------------------------------------------------AUUGCACUUGUCUCGGUCUG--------
----    19   1,-1
------------------------------------------------------AUUGCACUUGUCUCGGUCUGu-------
----    3    1,-1
------------------------------------------------------AUUGCACUUGUCUCGGUCUGA-------
----    30   1,0
------------------------------------------------------AUUGCACUUGUCUCGGUCUGAu------
----    2    1,0

>hsa-mir-26a-1  MA:10-31;mi:49-70   SRX018958
guggccucgUUCAAGUAAUCCAGGAUAGGCUgugcaggucccaaugggCCUAUUCUUGGUUACUUGCACGgggacgc
(((.((((((.(((((((((.((((((((((.((.......))...)))))))))).))))))))).)))))).)))
---------UUCAAGUAAUCCAGGAUA-------------------------------------------------
    1095    0,-4
---------UUCAAGUAAUCCAGGAUAa------------------------------------------------
    37  0,-4
---------UUCAAGUAAUCCAGGAUAaa-----------------------------------------------
```

5 0,-4

---------UUCAAGUAAUCCAGGAUAu---

7 0,-4

---------UUCAAGUAAUCCAGGAUAG---

1910 0,-3

---------UUCAAGUAAUCCAGGAUAGa--

6 0,-3

---------UUCAAGUAAUCCAGGAUAGu--

5 0,-3

---------UUCAAGUAAUCCAGGAUAGG--

1853 0,-2

---------UUCAAGUAAUCCAGGAUAGGa---

8 0,-2

---------UUCAAGUAAUCCAGGAUAGGaa--

6 0,-2

---------UUCAAGUAAUCCAGGAUAGGau--

18 0,-2

---------UUCAAGUAAUCCAGGAUAGGu---

10 0,-2

---------UUCAAGUAAUCCAGGAUAGGuu--

44 0,-2

---------UUCAAGUAAUCCAGGAUAGGC---

1081 0,-1

---------UUCAAGUAAUCCAGGAUAGGCa--

1671 0,-1

---------UUCAAGUAAUCCAGGAUAGGCaa---

990 0,-1

---------UUCAAGUAAUCCAGGAUAGGCau---

651 0,-1

---------UUCAAGUAAUCCAGGAUAGGCU--

13236 0,0

---------UUCAAGUAAUCCAGGAUAGGCUa---

356 0,0

---------UUCAAGUAAUCCAGGAUAGGCUaa--

451 0,0

---------UUCAAGUAAUCCAGGAUAGGCUau--

1436 0,0

---------UUCAAGUAAUCCAGGAUAGGCUu---

645 0,0

---------UUCAAGUAAUCCAGGAUAGGCUuu--

2064 0,0

---------UUCAAGUAAUCCAGGAUAGGCUGa--

4 0,1

---------UUCAAGUAAUCCAGGAUAGGCUGU--

2 0,2

----------UCAAGUAAUCCAGGAUAG---

25 1,-3

----------UCAAGUAAUCCAGGAUAGG--

28 1,-2

----------UCAAGUAAUCCAGGAUAGGau--

2 1,-2

----------UCAAGUAAUCCAGGAUAGGuu--

2 1,-2

----------UCAAGUAAUCCAGGAUAGGCa--

36 1,-1

----------UCAAGUAAUCCAGGAUAGGCaa---

12 1,-1

----------UCAAGUAAUCCAGGAUAGGCau---

12 1,-1

----------UCAAGUAAUCCAGGAUAGGCU--

186 1,0

----------UCAAGUAAUCCAGGAUAGGCUa---

11 1,0

----------UCAAGUAAUCCAGGAUAGGCUaa--

8 1,0

----------UCAAGUAAUCCAGGAUAGGCUau--

6 1,0

----------UCAAGUAAUCCAGGAUAGGCUu---

15 1,0

----------UCAAGUAAUCCAGGAUAGGCUuu--

19 1,0

-----------CAAGUAAUCCAGGAUAGG--

4 2,-2

-----------CAAGUAAUCCAGGAUAGGCa--

2 2,-1

-----------CAAGUAAUCCAGGAUAGGCaa---

6	2,-1
-----------CAAGUAAUCCAGGAUAGGCU--
14	2,0
-----------CAAGUAAUCCAGGAUAGGCUau--
3	2,0
-----------CAAGUAAUCCAGGAUAGGCUu---
2	2,0
-----------CAAGUAAUCCAGGAUAGGCUuu--
5	2,0
--CCUAUUCUUGGUUACUUGCAC--------
2	0,-1
--CCUAUUCUUGGUUACUUGCACG-------
2	0,0

>hsa-mir-26a-2	MA:14-35;mi:52-73	SRX018958
ggcuguggcuggaUUCAAGUAAUCCAGGAUAGGCUguuuccaucugugaggCCUAUUCUUGAUUACUUGUUUCuggaggc
agcu
((((((..(..((..(((((((((.((((((((((...............)))))))))).)))))))))..))..)..))
))))
------------AUUCAAGUAAUCCAGGAUA--
----	2	-1,-4
------------AUUCAAGUAAUCCAGGAUAGG--
----	4	-1,-2
------------AUUCAAGUAAUCCAGGAUAGGC---
----	2	-1,-1
------------AUUCAAGUAAUCCAGGAUAGGCU--
----	3	-1,0
-------------UUCAAGUAAUCCAGGAUA--
----	1095	0,-4
-------------UUCAAGUAAUCCAGGAUAa---
----	37	0,-4
-------------UUCAAGUAAUCCAGGAUAaa--
----	5	0,-4
-------------UUCAAGUAAUCCAGGAUAu---
----	7	0,-4
-------------UUCAAGUAAUCCAGGAUAG---
----	1910	0,-3
-------------UUCAAGUAAUCCAGGAUAGa--

---- 6 0,-3
-------------UUCAAGUAAUCCAGGAUAGu--
---- 5 0,-3
-------------UUCAAGUAAUCCAGGAUAGG--
---- 1853 0,-2
-------------UUCAAGUAAUCCAGGAUAGGa---
---- 8 0,-2
-------------UUCAAGUAAUCCAGGAUAGGaa--
---- 6 0,-2
-------------UUCAAGUAAUCCAGGAUAGGau--
---- 18 0,-2
-------------UUCAAGUAAUCCAGGAUAGGu---
---- 10 0,-2
-------------UUCAAGUAAUCCAGGAUAGGuu--
---- 44 0,-2
-------------UUCAAGUAAUCCAGGAUAGGC---
---- 1081 0,-1
-------------UUCAAGUAAUCCAGGAUAGGCa--
---- 1671 0,-1
-------------UUCAAGUAAUCCAGGAUAGGCaa---
---- 990 0,-1
-------------UUCAAGUAAUCCAGGAUAGGCau---
---- 651 0,-1
-------------UUCAAGUAAUCCAGGAUAGGCU--
---- 13236 0,0
-------------UUCAAGUAAUCCAGGAUAGGCUa---
---- 356 0,0
-------------UUCAAGUAAUCCAGGAUAGGCUaa--
---- 451 0,0
-------------UUCAAGUAAUCCAGGAUAGGCUau--
---- 1436 0,0
-------------UUCAAGUAAUCCAGGAUAGGCUu---
---- 645 0,0
-------------UUCAAGUAAUCCAGGAUAGGCUuu--
---- 2064 0,0
-------------UUCAAGUAAUCCAGGAUAGGCUGa--
---- 4 0,1
-------------UUCAAGUAAUCCAGGAUAGGCUGU--

---- 2 0,2
--------------UCAAGUAAUCCAGGAUAG--
---- 25 1,-3
--------------UCAAGUAAUCCAGGAUAGG---
---- 28 1,-2
--------------UCAAGUAAUCCAGGAUAGGau---
---- 2 1,-2
--------------UCAAGUAAUCCAGGAUAGGuu---
---- 2 1,-2
--------------UCAAGUAAUCCAGGAUAGGCa---
---- 36 1,-1
--------------UCAAGUAAUCCAGGAUAGGCaa--
---- 12 1,-1
--------------UCAAGUAAUCCAGGAUAGGCau--
---- 12 1,-1
--------------UCAAGUAAUCCAGGAUAGGCU---
---- 186 1,0
--------------UCAAGUAAUCCAGGAUAGGCUa--
---- 11 1,0
--------------UCAAGUAAUCCAGGAUAGGCUaa---
---- 8 1,0
--------------UCAAGUAAUCCAGGAUAGGCUau---
---- 6 1,0
--------------UCAAGUAAUCCAGGAUAGGCUu--
---- 15 1,0
--------------UCAAGUAAUCCAGGAUAGGCUuu---
---- 19 1,0
---------------CAAGUAAUCCAGGAUAGG---
---- 4 2,-2
---------------CAAGUAAUCCAGGAUAGGCa---
---- 2 2,-1
---------------CAAGUAAUCCAGGAUAGGCaa--
---- 6 2,-1
---------------CAAGUAAUCCAGGAUAGGCU---
---- 14 2,0
---------------CAAGUAAUCCAGGAUAGGCUau---
---- 3 2,0
---------------CAAGUAAUCCAGGAUAGGCUu--

----   2   2,0
---------------CAAGUAAUCCAGGAUAGGCUuu---
----   5   2,0
---CCUAUUCUUGAUUACUUGUUUC-------
----   2   0,0

>hsa-mir-26b   MA:12-32;mi:47-68   SRX018958
ccgggacccagUUCAAGUAAUUCAGGAUAGGUugugugcuguccagCCUGUUCUCCAUUACUUGGCUCggggaccgg
((((..((((((.((((((((..(((((((((((.(......)))))))))))).)))))))))).))).))))
-----------UUCAAGUAAUUCAGGAUA---
    111 0,-3
-----------UUCAAGUAAUUCAGGAUAa--
    2   0,-3
-----------UUCAAGUAAUUCAGGAUAG--
    790 0,-2
-----------UUCAAGUAAUUCAGGAUAGa---
    3   0,-2
-----------UUCAAGUAAUUCAGGAUAGu---
    2   0,-2
-----------UUCAAGUAAUUCAGGAUAGG---
    1115   0,-1
-----------UUCAAGUAAUUCAGGAUAGGa--
    6   0,-1
-----------UUCAAGUAAUUCAGGAUAGGau---
    12   0,-1
-----------UUCAAGUAAUUCAGGAUAGGU--
    1064   0,0
-----------UUCAAGUAAUUCAGGAUAGGUa---
    340 0,0
-----------UUCAAGUAAUUCAGGAUAGGUaa--
    660 0,0
-----------UUCAAGUAAUUCAGGAUAGGUau--
    378 0,0
-----------UUCAAGUAAUUCAGGAUAGGUU---
    5274   0,1
-----------UUCAAGUAAUUCAGGAUAGGUUa--
    972 0,1
-----------UUCAAGUAAUUCAGGAUAGGUUaa---------------------------------------

65 0,1
-----------UUCAAGUAAUUCAGGAUAGGUUau---
20 0,1
-----------UUCAAGUAAUUCAGGAUAGGUUu--
1825 0,1
-----------UUCAAGUAAUUCAGGAUAGGUUuu---
41 0,1
-----------UUCAAGUAAUUCAGGAUAGGUUG--
13 0,2
-----------UUCAAGUAAUUCAGGAUAGGUUGa---
2 0,2
------------UCAAGUAAUUCAGGAUAG--
9 1,-2
------------UCAAGUAAUUCAGGAUAGG---
22 1,-1
------------UCAAGUAAUUCAGGAUAGGU--
12 1,0
------------UCAAGUAAUUCAGGAUAGGUaa--
2 1,0
------------UCAAGUAAUUCAGGAUAGGUau--
3 1,0
------------UCAAGUAAUUCAGGAUAGGUU---
63 1,1
------------UCAAGUAAUUCAGGAUAGGUUa--
2 1,1
------------UCAAGUAAUUCAGGAUAGGUUu--
32 1,1
------------UCAAGUAAUUCAGGAUAGGUUuu---
4 1,1
-------------CAAGUAAUUCAGGAUAGG---
4 2,-1
-------------CAAGUAAUUCAGGAUAGGU--
3 2,0
-------------CAAGUAAUUCAGGAUAGGUU---
6 2,1
-------------CAAGUAAUUCAGGAUAGGUUu--
9 2,1
---CCUGUUCUCCAUUACUUGGCU----------

3 0,-1
--CCUGUUCUCCAUUACUUGGCUC---------
2 0,0

>hsa-mir-27a mi:10-31;MA:51-71 SRX018958
cugaggagcAGGGCUUAGCUGCUUGUGAGCAgggucccacaccaagucgugUUCACAGUGGCUAAGUUCCGCccccccag
(((.((.((.(((((((((..((.(((((((.((.(........))).)))))))))..))))))))).)).)).)))
---------AGGGCUUAGCUGCUUGUGAGCA---
6 0,0
---GUUCACAGUGGCUAAGUU-----------
14 -1,-4
---GUUCACAGUGGCUAAGUUC----------
56 -1,-3
--UUCACAGUGGCUAAGUUC----------
7689 0,-3
--UUCACAGUGGCUAAGUUCa---------
25 0,-3
--UUCACAGUGGCUAAGUUCaa--------
5 0,-3
--UUCACAGUGGCUAAGUUCC---------
142 0,-2
--UUCACAGUGGCUAAGUUCCa--------
2 0,-2
--UUCACAGUGGCUAAGUUCCG--------
398 0,-1
--UUCACAGUGGCUAAGUUCCGa-------
32 0,-1
--UUCACAGUGGCUAAGUUCCGau------
2 0,-1
--UUCACAGUGGCUAAGUUCCGu-------
38 0,-1
--UUCACAGUGGCUAAGUUCCGuu------
5 0,-1
--UUCACAGUGGCUAAGUUCCGC-------
25 0,0
--UUCACAGUGGCUAAGUUCCGCaa-----
8 0,0
--UUCACAGUGGCUAAGUUCCGCau-----

4   0,0
--UUCACAGUGGCUAAGUUCCGCC------
3   0,1
---UCACAGUGGCUAAGUUCCG--------
2   1,-1

>hsa-mir-27b   mi:19-40;MA:61-81   SRX018958
accucucuaacaaggugcAGAGCUUAGCUGAUUGGUGAACagugauugguuuccgcuuugUUCACAGUGGCUAAGUUCUG
Caccugaagagaaggug
(((((((....(((((((((((((((((((....((((((((....(((...)))..))))))))..)))))))))))))
)))))..)))).)))).
-----------------CAGAGCUUAGCUGAUUGGUGA--
-----------------   7   -1,-2
-----------------CAGAGCUUAGCUGAUUGGUGAA---
-----------------   13   -1,-1
-----------------CAGAGCUUAGCUGAUUGGUGAAC--
-----------------   3   -1,0
------------------AGAGCUUAGCUGAUUGGU--
-----------------   5   0,-4
------------------AGAGCUUAGCUGAUUGGUG---
-----------------   38   0,-3
------------------AGAGCUUAGCUGAUUGGUGA--
-----------------   358 0,-2
------------------AGAGCUUAGCUGAUUGGUGAA---
-----------------   181 0,-1
------------------AGAGCUUAGCUGAUUGGUGAAC--
-----------------   14   0,0
------------------AGAGCUUAGCUGAUUGGUGAACA---------------------------------------
-----------------   24   0,1
------------------AGAGCUUAGCUGAUUGGUGAACAa--------------------------------------
-----------------   4   0,1
------------------AGAGCUUAGCUGAUUGGUGAACAG--------------------------------------
-----------------   14   0,2
------------------AGAGCUUAGCUGAUUGGUGAACAGU-------------------------------------
-----------------   7   0,3
-------------------GAGCUUAGCUGAUUGGUGAACA---------------------------------------
-----------------   6   1,1
---GUUCACAGUGGCUAAGUU---

----------------   14   -1,-4
--GUUCACAGUGGCUAAGUUC--
----------------   56   -1,-3
--GUUCACAGUGGCUAAGUUCU-
----------------   6   -1,-2
--GUUCACAGUGGCUAAGUUCUG
CAa-------------   2   -1,1
---UUCACAGUGGCUAAGUUC--
----------------   7689   0,-3
---UUCACAGUGGCUAAGUUCa-
----------------   25   0,-3
---UUCACAGUGGCUAAGUUCaa
----------------   5   0,-3
---UUCACAGUGGCUAAGUUCU-
----------------   1703   0,-2
---UUCACAGUGGCUAAGUUCUa
----------------   16   0,-2
---UUCACAGUGGCUAAGUUCUa
a---------------   14   0,-2
---UUCACAGUGGCUAAGUUCUa
u---------------   3   0,-2
---UUCACAGUGGCUAAGUUCUu
----------------   13   0,-2
---UUCACAGUGGCUAAGUUCUu
u---------------   18   0,-2
---UUCACAGUGGCUAAGUUCUG
----------------   155 0,-1
---UUCACAGUGGCUAAGUUCUG
a---------------   37   0,-1
---UUCACAGUGGCUAAGUUCUG
aa--------------   61   0,-1
---UUCACAGUGGCUAAGUUCUG
au--------------   2   0,-1
---UUCACAGUGGCUAAGUUCUG
u---------------   14   0,-1
---UUCACAGUGGCUAAGUUCUG
uu--------------   14   0,-1
---UUCACAGUGGCUAAGUUCUG

C----------------   296 0,0
--UUCACAGUGGCUAAGUUCUG
Cu---------------   3   0,0
--UUCACAGUGGCUAAGUUCUG
CA---------------   83 0,1
--UUCACAGUGGCUAAGUUCUG
CAa--------------   70 0,1
--UUCACAGUGGCUAAGUUCUG
CAaa-------------   3   0,1
--UUCACAGUGGCUAAGUUCUG
CAau-------------   5   0,1
--UUCACAGUGGCUAAGUUCUG
CAu--------------   86 0,1
--UUCACAGUGGCUAAGUUCUG
CAuu-------------   15 0,1
---UCACAGUGGCUAAGUUCU-
-----------------   20 1,-2
---UCACAGUGGCUAAGUUCUG
Cu---------------   2   1,0
---UCACAGUGGCUAAGUUCUG
CAa--------------   5   1,1
---UCACAGUGGCUAAGUUCUG
CAu--------------   3   1,1

>hsa-mir-28 5p:14-35;3p:54-75   SRX018958
gguccuugcccucAAGGAGCUCACAGUCUAUUGAGuuaccuuucugacuuuccCACUAGAUUGUGAGCUCCUGGAgggca
ggcacu
(((.(((((((((.((((((((((((((((.(((((((......)))))....)).)))))))))))))))).)))))))
)).)))
------------CAAGGAGCUCACAGUCUAUU--
------   2   -1,-3
------------CAAGGAGCUCACAGUCUAUUG---
------   7   -1,-2
------------CAAGGAGCUCACAGUCUAUUGA--
------   5   -1,-1
-------------AAGGAGCUCACAGUCUAU---
------   4   0,-4
-------------AAGGAGCUCACAGUCUAUa--

------ 16 0,-4

-------------AAGGAGCUCACAGUCUAUaa---

------ 3 0,-4

-------------AAGGAGCUCACAGUCUAUU--

------ 135 0,-3

-------------AAGGAGCUCACAGUCUAUUa---

------ 14 0,-3

-------------AAGGAGCUCACAGUCUAUUau--

------ 2 0,-3

-------------AAGGAGCUCACAGUCUAUUG---

------ 124 0,-2

-------------AAGGAGCUCACAGUCUAUUGu--

------ 10 0,-2

-------------AAGGAGCUCACAGUCUAUUGA--

------ 343 0,-1

-------------AAGGAGCUCACAGUCUAUUGAa---

------ 19 0,-1

-------------AAGGAGCUCACAGUCUAUUGAaa--

------ 6 0,-1

-------------AAGGAGCUCACAGUCUAUUGAG---

------ 203 0,0

-------------AAGGAGCUCACAGUCUAUUGAGa--

------ 59 0,0

-------------AAGGAGCUCACAGUCUAUUGAGU--

------ 9 0,1

--------------AGGAGCUCACAGUCUAUUG---

------ 4 1,-2

--------------AGGAGCUCACAGUCUAUUGAGa--

------ 4 1,0

---CACUAGAUUGUGAGCUCC---------

------ 4 0,-4

---CACUAGAUUGUGAGCUCCU--------

------ 15 0,-3

---CACUAGAUUGUGAGCUCCUG-------

------ 38 0,-2

---CACUAGAUUGUGAGCUCCUGa------

------ 9 0,-2

---CACUAGAUUGUGAGCUCCUGaa-----

------ 5 0,-2

--CACUAGAUUGUGAGCUCCUGG------

------ 516 0,-1

--CACUAGAUUGUGAGCUCCUGGu-----

------ 19 0,-1

--CACUAGAUUGUGAGCUCCUGGuu----

------ 3 0,-1

--CACUAGAUUGUGAGCUCCUGGA-----

------ 2562 0,0

--CACUAGAUUGUGAGCUCCUGGAa----

------ 1498 0,0

--CACUAGAUUGUGAGCUCCUGGAaa---

------ 193 0,0

--CACUAGAUUGUGAGCUCCUGGAau---

------ 52 0,0

--CACUAGAUUGUGAGCUCCUGGAu----

------ 126 0,0

--CACUAGAUUGUGAGCUCCUGGAuu---

------ 10 0,0

--CACUAGAUUGUGAGCUCCUGGAG----

------ 24 0,1

--CACUAGAUUGUGAGCUCCUGGAGa---

------ 6 0,1

--CACUAGAUUGUGAGCUCCUGGAGu---

------ 20 0,1

---ACUAGAUUGUGAGCUCCUGG------

------ 16 1,-1

---ACUAGAUUGUGAGCUCCUGGA-----

------ 113 1,0

---ACUAGAUUGUGAGCUCCUGGAa----

------ 68 1,0

---ACUAGAUUGUGAGCUCCUGGAaa---

------ 20 1,0

---ACUAGAUUGUGAGCUCCUGGAau---

------ 14 1,0

---ACUAGAUUGUGAGCUCCUGGAu----

------ 5 1,0

---ACUAGAUUGUGAGCUCCUGGAG----

------ 67 1,1
--ACUAGAUUGUGAGCUCCUGGAGa---
------ 64 1,1
--ACUAGAUUGUGAGCUCCUGGAGaa--
------ 9 1,1
--ACUAGAUUGUGAGCUCCUGGAGu---
------ 30 1,1
---CUAGAUUGUGAGCUCCUGG------
------ 3 2,-1
---CUAGAUUGUGAGCUCCUGGA-----
------ 9 2,0
---CUAGAUUGUGAGCUCCUGGAa----
------ 4 2,0
---CUAGAUUGUGAGCUCCUGGAG----
------ 20 2,1
---CUAGAUUGUGAGCUCCUGGAGa---
------ 27 2,1
---CUAGAUUGUGAGCUCCUGGAGaa--
------ 5 2,1
---CUAGAUUGUGAGCUCCUGGAGau--
------ 6 2,1
---CUAGAUUGUGAGCUCCUGGAGu---
------ 13 2,1

>hsa-mir-296 5p:14-34;3p:48-69 SRX018958
aggacccuuccagAGGGCCCCCCCUCAAUCCUGUugugccuaauucaGAGGGUUGGGUGGAGGCUCUCCugaagggcucu
((..((((((..((((((.((.(((((((((.(((...........))))))))))).)).)))))))..))))))..))
---GAGGGUUGGGUGGAGGCU---------------
2 0,-4

>hsa-mir-299 5p:7-28;3p:39-60 SRX018958
aagaaaUGGUUUACCGUCCCACAUACAUuuugaauaugUAUGUGGGAUGGUAAACCGCUUcuu
((((.(((((((((((((((((((((((.......)))))))))))))))))))))).)))))
-----AUGGUUUACCGUCCCACAUACA------------------------------------- 2 -1,-1
------UGGUUUACCGUCCCACAUACAU----------------------------------- 8 0,0
-------------------------------------GUAUGUGGGAUGGUAAACCGCUa--- 2 -1,-1
--------------------------------------UAUGUGGGAUGGUAAACCGCUU--- 15 0,0

```
>hsa-mir-29a    mi:4-25;MA:42-63    SRX018958
augACUGAUUUCUUUUGGUGUUCAGagucaauauaauuuucUAGCACCAUCUGAAAUCGGUUAu
((((((((((((...(((((((.((((...........)))))))))))...))))))))))))
---ACUGAUUUCUUUUGGUGUUCA----------------------------------------    12  0,-1
---ACUGAUUUCUUUUGGUGUUCAG---------------------------------------    10  0,0
---ACUGAUUUCUUUUGGUGUUCAGA--------------------------------------    4   0,1
---ACUGAUUUCUUUUGGUGUUCAGAG-------------------------------------    2   0,2
---------------------------------------UCUAGCACCAUCUGAAAUCGGU---    3   -2,-2
---------------------------------------UCUAGCACCAUCUGAAAUCGGUUA-    2   -2,0
----------------------------------------CUAGCACCAUCUGAAAUC------    10  -1,-5
----------------------------------------CUAGCACCAUCUGAAAUCG-----    13  -1,-4
----------------------------------------CUAGCACCAUCUGAAAUCGG----    82  -1,-3
----------------------------------------CUAGCACCAUCUGAAAUCGGa---    3   -1,-3
----------------------------------------CUAGCACCAUCUGAAAUCGGau--    2   -1,-3
----------------------------------------CUAGCACCAUCUGAAAUCGGU---    86  -1,-2
----------------------------------------CUAGCACCAUCUGAAAUCGGUa--    27  -1,-2
----------------------------------------CUAGCACCAUCUGAAAUCGGUaa-    46  -1,-2
----------------------------------------CUAGCACCAUCUGAAAUCGGUau-    7   -1,-2
----------------------------------------CUAGCACCAUCUGAAAUCGGUU--    833 -1,-1
----------------------------------------CUAGCACCAUCUGAAAUCGGUUu-    302 -1,-1
----------------------------------------CUAGCACCAUCUGAAAUCGGUUA-    645 -1,0
----------------------------------------CUAGCACCAUCUGAAAUCGGUUAa    48  -1,0
----------------------------------------CUAGCACCAUCUGAAAUCGGUUAU    39  -1,1
-----------------------------------------UAGCACCAUCUGAAAUCG-----    393 0,-4
-----------------------------------------UAGCACCAUCUGAAAUCGa----    4   0,-4
-----------------------------------------UAGCACCAUCUGAAAUCGuu---    10  0,-4
-----------------------------------------UAGCACCAUCUGAAAUCGG----    7495    0,-3
-----------------------------------------UAGCACCAUCUGAAAUCGGa---    2   0,-3
-----------------------------------------UAGCACCAUCUGAAAUCGGaa--    5   0,-3
-----------------------------------------UAGCACCAUCUGAAAUCGGau--    35  0,-3
-----------------------------------------UAGCACCAUCUGAAAUCGGU---    1363    0,-2
-----------------------------------------UAGCACCAUCUGAAAUCGGUa--    122 0,-2
-----------------------------------------UAGCACCAUCUGAAAUCGGUaa-    1654    0,-2
-----------------------------------------UAGCACCAUCUGAAAUCGGUau-    171 0,-2
-----------------------------------------UAGCACCAUCUGAAAUCGGUU--    18634   0,-1
-----------------------------------------UAGCACCAUCUGAAAUCGGUUu-    12915   0,-1
-----------------------------------------UAGCACCAUCUGAAAUCGGUUuu    403 0,-1
-----------------------------------------UAGCACCAUCUGAAAUCGGUUA-    122371  0,0
```

---UAGCACCAUCUGAAAUCGGUUAa 5880 0,0
---UAGCACCAUCUGAAAUCGGUUAaa 40 0,0
---UAGCACCAUCUGAAAUCGGUUAau 19 0,0
---UAGCACCAUCUGAAAUCGGUUAU 3433 0,1
---UAGCACCAUCUGAAAUCGGUUAUa 33 0,1
---UAGCACCAUCUGAAAUCGGUUAUu 25 0,1
---UAGCACCAUCUGAAAUCGGUUAUuu 2 0,1
--AGCACCAUCUGAAAUCGG---- 78 1,-3
--AGCACCAUCUGAAAUCGGU--- 16 1,-2
--AGCACCAUCUGAAAUCGGUa-- 2 1,-2
--AGCACCAUCUGAAAUCGGUaa- 11 1,-2
--AGCACCAUCUGAAAUCGGUU-- 135 1,-1
--AGCACCAUCUGAAAUCGGUUu- 150 1,-1
--AGCACCAUCUGAAAUCGGUUuu 4 1,-1
--AGCACCAUCUGAAAUCGGUUA- 1157 1,0
--AGCACCAUCUGAAAUCGGUUAa 71 1,0
--AGCACCAUCUGAAAUCGGUUAU 38 1,1
---GCACCAUCUGAAAUCGGU--- 2 2,-2
---GCACCAUCUGAAAUCGGUU-- 9 2,-1
---GCACCAUCUGAAAUCGGUUu- 7 2,-1
---GCACCAUCUGAAAUCGGUUA- 62 2,0
---GCACCAUCUGAAAUCGGUUAa 3 2,0
---GCACCAUCUGAAAUCGGUUAU 7 2,1

>hsa-mir-29b-1 mi:10-33;MA:51-73 SRX018958
cuucaggaaGCUGGUUUCAUAUGGUGGUUUAGAuuuaaauagugauugucUAGCACCAUUUGAAAUCAGUGUUcuugggg
g
(((((((((((((((((((.((((((..((((((.............)))))))))))).)))))))))).)))))))))
.
---CUAGCACCAUUUGAAAUCA------------
- 4 -1,-5
---CUAGCACCAUUUGAAAUCAG-----------
- 12 -1,-4
---CUAGCACCAUUUGAAAUCAGU----------
- 79 -1,-3
---CUAGCACCAUUUGAAAUCAGUG---------
- 7 -1,-2
--UAGCACCAUUUGAAAUCA------------

- 162 0,-5

--UAGCACCAUUUGAAAUCAa-----------

- 2 0,-5

--UAGCACCAUUUGAAAUCAau----------

- 2 0,-5

--UAGCACCAUUUGAAAUCAuu----------

- 3 0,-5

--UAGCACCAUUUGAAAUCAG-----------

- 346 0,-4

--UAGCACCAUUUGAAAUCAGa----------

- 14 0,-4

--UAGCACCAUUUGAAAUCAGU----------

- 8715 0,-3

--UAGCACCAUUUGAAAUCAGUa---------

- 12 0,-3

--UAGCACCAUUUGAAAUCAGUaa--------

- 6 0,-3

--UAGCACCAUUUGAAAUCAGUu---------

- 17 0,-3

--UAGCACCAUUUGAAAUCAGUuu--------

- 10 0,-3

--UAGCACCAUUUGAAAUCAGUG---------

- 278 0,-2

--UAGCACCAUUUGAAAUCAGUGa--------

- 11 0,-2

--UAGCACCAUUUGAAAUCAGUGU--------

- 142 0,-1

--UAGCACCAUUUGAAAUCAGUGUa-------

- 34 0,-1

--UAGCACCAUUUGAAAUCAGUGUaa------

- 3 0,-1

--UAGCACCAUUUGAAAUCAGUGUU-------

- 274 0,0

--UAGCACCAUUUGAAAUCAGUGUUa------

- 13 0,0

---AGCACCAUUUGAAAUCAGU----------

- 56 1,-3

---AGCACCAUUUGAAAUCAGUG---------

- 7 1,-2
--GCACCAUUUGAAAUCAGU----------
- 8 2,-3

>hsa-mir-29b-2 mi:11-32;MA:52-74 SRX018958
cuucuggaagCUGGUUUCACAUGGUGGCUUAGauuuuuccaucuuuguaucUAGCACCAUUUGAAAUCAGUGUUuuagga
g
(((((((((((((((((((.((((((.((.((((..............)))))))))))).)))))))))).))))))))
)
----------CUGGUUUCACAUGGUGGCUUA---
- 4 0,-1
--CUAGCACCAUUUGAAAUCA-----------
- 4 -1,-5
--CUAGCACCAUUUGAAAUCAG----------
- 12 -1,-4
--CUAGCACCAUUUGAAAUCAGU---------
- 79 -1,-3
--CUAGCACCAUUUGAAAUCAGUG--------
- 7 -1,-2
---UAGCACCAUUUGAAAUCA-----------
- 162 0,-5
---UAGCACCAUUUGAAAUCAa----------
- 2 0,-5
---UAGCACCAUUUGAAAUCAau---------
- 2 0,-5
---UAGCACCAUUUGAAAUCAuu---------
- 3 0,-5
---UAGCACCAUUUGAAAUCAG----------
- 346 0,-4
---UAGCACCAUUUGAAAUCAGa---------
- 14 0,-4
---UAGCACCAUUUGAAAUCAGU---------
- 8715 0,-3
---UAGCACCAUUUGAAAUCAGUa--------
- 12 0,-3
---UAGCACCAUUUGAAAUCAGUaa-------
- 6 0,-3
---UAGCACCAUUUGAAAUCAGUu--------

- 17 0,-3
--UAGCACCAUUUGAAAUCAGUuu-------
- 10 0,-3
--UAGCACCAUUUGAAAUCAGUG--------
- 278 0,-2
--UAGCACCAUUUGAAAUCAGUGa-------
- 11 0,-2
--UAGCACCAUUUGAAAUCAGUGU-------
- 142 0,-1
--UAGCACCAUUUGAAAUCAGUGUa------
- 34 0,-1
--UAGCACCAUUUGAAAUCAGUGUaa-----
- 3 0,-1
--UAGCACCAUUUGAAAUCAGUGUU------
- 274 0,0
--UAGCACCAUUUGAAAUCAGUGUUa-----
- 13 0,0
--UAGCACCAUUUGAAAUCAGUGUUU-----
- 38 0,1
---AGCACCAUUUGAAAUCAGU---------
- 56 1,-3
---AGCACCAUUUGAAAUCAGUG--------
- 7 1,-2
--GCACCAUUUGAAAUCAGU---------
- 8 2,-3

>hsa-mir-29c mi:16-37;MA:54-75 SRX018958
aucucuuacacaggcUGACCGAUUUCUCCUGGUGUUCagagucuguuuugucUAGCACCAUUUGAAAUCGGUUAugaug
uaggggga
.(((((((((((...(((((((((((...(((((((((((.....)))))...)))))))...))))))))))))).))
))))))).
---------------UGACCGAUUUCUCCUGGUGUa---
-------- 2 0,-2
---------------UGACCGAUUUCUCCUGGUGUU---
-------- 5 0,-1
---------------UGACCGAUUUCUCCUGGUGUUa--
-------- 5 0,-1
---------------UGACCGAUUUCUCCUGGUGUUu--

-------- 2 0,-1

----------------GACCGAUUUCUCCUGGUGUU--

-------- 12 1,-1

----------------GACCGAUUUCUCCUGGUGUUu---

-------- 4 1,-1

----------------GACCGAUUUCUCCUGGUGUUC---

-------- 2 1,0

----------------GACCGAUUUCUCCUGGUGUUCA--

-------- 4 1,1

-----------------ACCGAUUUCUCCUGGUGUUC---

-------- 2 2,0

-----------------ACCGAUUUCUCCUGGUGUUCA--

-------- 6 2,1

-----------------ACCGAUUUCUCCUGGUGUUCAa---

-------- 2 2,1

-----------------ACCGAUUUCUCCUGGUGUUCAG---

-------- 10 2,2

-----------------ACCGAUUUCUCCUGGUGUUCAGA--

-------- 6 2,3

--CUAGCACCAUUUGAAAUCGG--------

-------- 2 -1,-3

--CUAGCACCAUUUGAAAUCGGUUu-----

-------- 2 -1,-1

--CUAGCACCAUUUGAAAUCGGUUA-----

-------- 16 -1,0

---UAGCACCAUUUGAAAUCG---------

-------- 187 0,-4

---UAGCACCAUUUGAAAUCGa--------

-------- 2 0,-4

---UAGCACCAUUUGAAAUCGuu-------

-------- 7 0,-4

---UAGCACCAUUUGAAAUCGG--------

-------- 1101 0,-3

---UAGCACCAUUUGAAAUCGGau------

-------- 4 0,-3

---UAGCACCAUUUGAAAUCGGU-------

-------- 290 0,-2

---UAGCACCAUUUGAAAUCGGUa------

-------- 8 0,-2
---UAGCACCAUUUGAAAUCGGUaa-----
-------- 128 0,-2
---UAGCACCAUUUGAAAUCGGUau-----
-------- 9 0,-2
---UAGCACCAUUUGAAAUCGGUU------
-------- 1206 0,-1
---UAGCACCAUUUGAAAUCGGUUu-----
-------- 892 0,-1
---UAGCACCAUUUGAAAUCGGUUuu----
-------- 39 0,-1
---UAGCACCAUUUGAAAUCGGUUA-----
-------- 11690 0,0
---UAGCACCAUUUGAAAUCGGUUAa----
-------- 670 0,0
---UAGCACCAUUUGAAAUCGGUUAaa---
-------- 11 0,0
---UAGCACCAUUUGAAAUCGGUUAau---
-------- 2 0,0
---UAGCACCAUUUGAAAUCGGUUAU----
-------- 292 0,1
---UAGCACCAUUUGAAAUCGGUUAUu---
-------- 5 0,1
---UAGCACCAUUUGAAAUCGGUUAUuu--
-------- 2 0,1
--AGCACCAUUUGAAAUCGG--------
-------- 2 1,-3
--AGCACCAUUUGAAAUCGGU-------
-------- 4 1,-2
--AGCACCAUUUGAAAUCGGUa------
-------- 2 1,-2
--AGCACCAUUUGAAAUCGGUU------
-------- 10 1,-1
--AGCACCAUUUGAAAUCGGUUu-----
-------- 4 1,-1
--AGCACCAUUUGAAAUCGGUUA-----
-------- 64 1,0
--AGCACCAUUUGAAAUCGGUUAa----

-------- 2 1,0
--AGCACCAUUUGAAAUCGGUUAU----
-------- 4 1,1
---GCACCAUUUGAAAUCGGUU------
-------- 2 2,-1
---GCACCAUUUGAAAUCGGUUA-----
-------- 13 2,0
---GCACCAUUUGAAAUCGGUUAa----
-------- 2 2,0
---GCACCAUUUGAAAUCGGUUAUa---
-------- 2 2,1
---GCACCAUUUGAAAUCGGUUAUu---
-------- 5 2,1

>hsa-mir-301a 3p:51-73 SRX018958
acugcuaacgaaugcucugacuuuauugcacuacuguacuuuacagcuagCAGUGCAAUAGUAUUGUCAAAGCaucugaa
agcagg
.(((((..((.(((((.((((.((((((((((.(((((...))))).....))))))))))....)))).))))).))..
))))).
-------------GCUCUGACUUUAUUGCACUAC--
------ 12
-------------GCUCUGACUUUAUUGCACUACU---
------ 17
-------------GCUCUGACUUUAUUGCACUACUuu---
------ 4
--CAGUGCAAUAGUAUUGUCAA----------
------ 3 0,-3
--CAGUGCAAUAGUAUUGUCAAAGCAU-----
------ 2 0,2

>hsa-mir-3065 5p:10-32;3p:50-72 SRX018958
cugcccucuUCAACAAAAUCACUGAUGCUGGAgucgccugagucaucacUCAGCACCAGGAUAUUGUUGGAGaggacag
(((.((((((((((((.(((.(((.(((((((.((....)).))......))))).)))))).))))))))))))).)))
---------UCAACAAAAUCACUGAUGCU--
17 0,-3
---------UCAACAAAAUCACUGAUGCUGGA---
7 0,0
---UCAGCACCAGGAUAUUGUUG----------

7 0,-3
--UCAGCACCAGGAUAUUGUUGGAGu------
2 0,0

>hsa-mir-30a MA:6-27;mi:47-68 SRX018958
gcgacUGUAAACAUCCUCGACUGGAAGcugugaagccacagaugggCUUUCAGUCGGAUGUUUGCAGCugc
(((.((((((((((((..((((((((((((((....))).....))))))))))))))))))))))).)))
----CUGUAAACAUCCUCGACU--- 5 -1,-5
----CUGUAAACAUCCUCGACUGGA-- 2 -1,-2
----CUGUAAACAUCCUCGACUGGAA--- 9 -1,-1
----CUGUAAACAUCCUCGACUGGAAa-- 4 -1,-1
----CUGUAAACAUCCUCGACUGGAAG-- 18 -1,0
----CUGUAAACAUCCUCGACUGGAAGa--- 10 -1,0
----CUGUAAACAUCCUCGACUGGAAGC--- 61 -1,1
----CUGUAAACAUCCUCGACUGGAAGCa-- 3 -1,1
----CUGUAAACAUCCUCGACUGGAAGCU-- 65 -1,2
-----UGUAAACAUCCUCGACUG-- 139 0,-4
-----UGUAAACAUCCUCGACUGa--- 2 0,-4
-----UGUAAACAUCCUCGACUGG--- 143 0,-3
-----UGUAAACAUCCUCGACUGGu-- 6 0,-3
-----UGUAAACAUCCUCGACUGGA-- 360 0,-2
-----UGUAAACAUCCUCGACUGGAA--- 1096 0,-1
-----UGUAAACAUCCUCGACUGGAAa-- 583 0,-1
-----UGUAAACAUCCUCGACUGGAAaa--- 41 0,-1
-----UGUAAACAUCCUCGACUGGAAau--- 8 0,-1
-----UGUAAACAUCCUCGACUGGAAu-- 14 0,-1
-----UGUAAACAUCCUCGACUGGAAuu--- 4 0,-1
-----UGUAAACAUCCUCGACUGGAAG-- 3972 0,0
-----UGUAAACAUCCUCGACUGGAAGa--- 3284 0,0
-----UGUAAACAUCCUCGACUGGAAGaa-- 163 0,0
-----UGUAAACAUCCUCGACUGGAAGau-- 151 0,0

-----UGUAAACAUCCUCGACUGGAAGu-- 265 0,0
-----UGUAAACAUCCUCGACUGGAAGuu--- 38 0,0
-----UGUAAACAUCCUCGACUGGAAGC-- 18904
0,1
-----UGUAAACAUCCUCGACUGGAAGCa--- 2421
0,1
-----UGUAAACAUCCUCGACUGGAAGCaa-- 30 0,1
-----UGUAAACAUCCUCGACUGGAAGCau-- 71 0,1
-----UGUAAACAUCCUCGACUGGAAGCU--- 26590
0,2
-----UGUAAACAUCCUCGACUGGAAGCUa-- 145 0,2
-----UGUAAACAUCCUCGACUGGAAGCUu-- 1164
0,2
-----UGUAAACAUCCUCGACUGGAAGCUG-- 10 0,3
------GUAAACAUCCUCGACUGGA--- 2 1,-2
------GUAAACAUCCUCGACUGGAA-- 6 1,-1
------GUAAACAUCCUCGACUGGAAa--- 2 1,-1
------GUAAACAUCCUCGACUGGAAau-- 2 1,-1
------GUAAACAUCCUCGACUGGAAu--- 2 1,-1
------GUAAACAUCCUCGACUGGAAG--- 69 1,0
------GUAAACAUCCUCGACUGGAAGa-- 15 1,0
------GUAAACAUCCUCGACUGGAAGaa--- 2 1,0
------GUAAACAUCCUCGACUGGAAGu-- 3 1,0
------GUAAACAUCCUCGACUGGAAGuu--- 2 1,0
------GUAAACAUCCUCGACUGGAAGC-- 165 1,1
------GUAAACAUCCUCGACUGGAAGCa--- 7 1,1
------GUAAACAUCCUCGACUGGAAGCU--- 100 1,2
------GUAAACAUCCUCGACUGGAAGCUu-- 5 1,2
-------UAAACAUCCUCGACUGGAAGC-- 5 2,1
-------UAAACAUCCUCGACUGGAAGCa--- 3 2,1
-------UAAACAUCCUCGACUGGAAGCU--- 5 2,2
--CUUUCAGUCGGAUGUUUG------- 13 0,-4
--CUUUCAGUCGGAUGUUUGC------ 8 0,-3
--CUUUCAGUCGGAUGUUUGCA----- 31 0,-2
--CUUUCAGUCGGAUGUUUGCAa---- 10 0,-2
--CUUUCAGUCGGAUGUUUGCAu---- 7 0,-2
--CUUUCAGUCGGAUGUUUGCAuu--- 4 0,-2
--CUUUCAGUCGGAUGUUUGCAG---- 197 0,-1

--CUUUCAGUCGGAUGUUUGCAGu--- 26 0,-1
--CUUUCAGUCGGAUGUUUGCAGuu-- 4 0,-1
--CUUUCAGUCGGAUGUUUGCAGC--- 111 0,0
--CUUUCAGUCGGAUGUUUGCAGCa-- 24 0,0
--CUUUCAGUCGGAUGUUUGCAGCaa- 5 0,0
--CUUUCAGUCGGAUGUUUGCAGCau- 47 0,0
--CUUUCAGUCGGAUGUUUGCAGCU-- 4 0,1
---UUUCAGUCGGAUGUUUGCAG---- 49 1,-1
---UUUCAGUCGGAUGUUUGCAGu--- 7 1,-1
---UUUCAGUCGGAUGUUUGCAGC--- 23 1,0
---UUUCAGUCGGAUGUUUGCAGCa-- 5 1,0
---UUUCAGUCGGAUGUUUGCAGCau- 6 1,0
---UUUCAGUCGGAUGUUUGCAGCU-- 2 1,1

>hsa-mir-30b MA:17-38;mi:55-76 SRX018958
accaaguuucaguucaUGUAAACAUCCUACACUCAGCUguaauacauggauuggCUGGGAGGUGGAUGUUUACUUCagcu
gacuugga
.(((((..((((((...((((((((((.((.((((((((............))))))))..))))))))))))...))))
))))))).
----------------UGUAAACAUCCUACACUC--
-------- 16 0,-4
----------------UGUAAACAUCCUACACUCA---
-------- 5 0,-3
----------------UGUAAACAUCCUACACUCAG--
-------- 29 0,-2
----------------UGUAAACAUCCUACACUCAGC---
-------- 68 0,-1
----------------UGUAAACAUCCUACACUCAGCa--
-------- 8 0,-1
----------------UGUAAACAUCCUACACUCAGCau---
-------- 4 0,-1
----------------UGUAAACAUCCUACACUCAGCU--
-------- 290 0,0
----------------UGUAAACAUCCUACACUCAGCUa---
-------- 38 0,0
----------------UGUAAACAUCCUACACUCAGCUau--
-------- 18 0,0
----------------UGUAAACAUCCUACACUCAGCUu---

--------	39	0,0
----------------UGUAAACAUCCUACACUCAGCUuu--
--------	7	0,0
-----------------GUAAACAUCCUACACUCAGCU--
--------	6	1,0
--CUGGGAGGUGGAUGUUUA--------
--------	19	0,-4
--CUGGGAGGUGGAUGUUUAa-------
--------	2	0,-4
--CUGGGAGGUGGAUGUUUAu-------
--------	3	0,-4
--CUGGGAGGUGGAUGUUUAC-------
--------	17	0,-3
--CUGGGAGGUGGAUGUUUACa------
--------	6	0,-3
--CUGGGAGGUGGAUGUUUACaa-----
--------	5	0,-3
--CUGGGAGGUGGAUGUUUACU------
--------	216	0,-2
--CUGGGAGGUGGAUGUUUACUa-----
--------	16	0,-2
--CUGGGAGGUGGAUGUUUACUaa----
--------	25	0,-2
--CUGGGAGGUGGAUGUUUACUau----
--------	3	0,-2
--CUGGGAGGUGGAUGUUUACUU-----
--------	232	0,-1
--CUGGGAGGUGGAUGUUUACUUa----
--------	69	0,-1
--CUGGGAGGUGGAUGUUUACUUaa---
--------	56	0,-1
--CUGGGAGGUGGAUGUUUACUUau---
--------	7	0,-1
--CUGGGAGGUGGAUGUUUACUUu----
--------	39	0,-1
--CUGGGAGGUGGAUGUUUACUUuu---
--------	7	0,-1
--CUGGGAGGUGGAUGUUUACUUC----

--------   31 0,0
---CUGGGAGGUGGAUGUUUACUUCu---
--------   4   0,0
---CUGGGAGGUGGAUGUUUACUUCA---
--------   4   0,1
--UGGGAGGUGGAUGUUUACU------
--------   7   1,-2
--UGGGAGGUGGAUGUUUACUU-----
--------   5   1,-1
--UGGGAGGUGGAUGUUUACUUC----
--------   3   1,0
--UGGGAGGUGGAUGUUUACUUCu---
--------   2   1,0

>hsa-mir-30c-1 MA:17-39;mi:56-77   SRX018958
accaugcuguagugugUGUAAACAUCCUACACUCUCAGCugugagcucaagguggCUGGGAGAGGGUUGUUUACUCCuuc
ugccaugga
.((((..((((...(.(((((((.(((...(((((((((.((....))....))))))))).))).))))))).)...)
)))))))).
----------------UGUAAACAUCCUACACUC--
---------   16 0,-5
----------------UGUAAACAUCCUACACUCU---
---------   6   0,-4
----------------UGUAAACAUCCUACACUCUC--
---------   18 0,-3
----------------UGUAAACAUCCUACACUCUCA---
---------   60 0,-2
----------------UGUAAACAUCCUACACUCUCAG--
---------   105 0,-1
----------------UGUAAACAUCCUACACUCUCAGa---
---------   23 0,-1
----------------UGUAAACAUCCUACACUCUCAGaa--
---------   13 0,-1
----------------UGUAAACAUCCUACACUCUCAGu---
---------   2   0,-1
----------------UGUAAACAUCCUACACUCUCAGuu--
---------   2   0,-1
----------------UGUAAACAUCCUACACUCUCAGC---

--------- 181 0,0

----------------UGUAAACAUCCUACACUCUCAGCa--

--------- 43 0,0

----------------UGUAAACAUCCUACACUCUCAGCaa---

--------- 3 0,0

----------------UGUAAACAUCCUACACUCUCAGCau---

--------- 9 0,0

----------------UGUAAACAUCCUACACUCUCAGCU--

--------- 634 0,1

----------------UGUAAACAUCCUACACUCUCAGCUa---

--------- 28 0,1

----------------UGUAAACAUCCUACACUCUCAGCUu---

--------- 38 0,1

-----------------GUAAACAUCCUACACUCUCA---

--------- 2 1,-2

-----------------GUAAACAUCCUACACUCUCAG--

--------- 2 1,-1

-----------------GUAAACAUCCUACACUCUCAGC---

--------- 5 1,0

-----------------GUAAACAUCCUACACUCUCAGCau---

--------- 2 1,0

-----------------GUAAACAUCCUACACUCUCAGCU--

--------- 9 1,1

------------------UAAACAUCCUACACUCUCAGCU--

--------- 4 2,1

---CUGGGAGAGGGUUGUUUA-------

--------- 3 0,-4

---CUGGGAGAGGGUUGUUUAa------

--------- 2 0,-4

---CUGGGAGAGGGUUGUUUAau-----

--------- 2 0,-4

---CUGGGAGAGGGUUGUUUAC------

--------- 17 0,-3

---CUGGGAGAGGGUUGUUUACa-----

--------- 6 0,-3

---CUGGGAGAGGGUUGUUUACU-----

--------- 254 0,-2

---CUGGGAGAGGGUUGUUUACUa----

--------- 16 0,-2

---CUGGGAGAGGGUUGUUUACUaa---

--------- 22 0,-2

---CUGGGAGAGGGUUGUUUACUau---

--------- 5 0,-2

---CUGGGAGAGGGUUGUUUACUu----

--------- 3 0,-2

---CUGGGAGAGGGUUGUUUACUC----

--------- 41 0,-1

---CUGGGAGAGGGUUGUUUACUCaa--

--------- 4 0,-1

---CUGGGAGAGGGUUGUUUACUCau--

--------- 4 0,-1

---CUGGGAGAGGGUUGUUUACUCC---

--------- 6 0,0

---CUGGGAGAGGGUUGUUUACUCCa--

--------- 2 0,0

---CUGGGAGAGGGUUGUUUACUCCaa-

--------- 3 0,0

---CUGGGAGAGGGUUGUUUACUCCau-

--------- 7 0,0

---CUGGGAGAGGGUUGUUUACUCCU--

--------- 11 0,1

--UGGGAGAGGGUUGUUUAC------

--------- 2 1,-3

--UGGGAGAGGGUUGUUUACU-----

--------- 22 1,-2

--UGGGAGAGGGUUGUUUACUC----

--------- 6 1,-1

--UGGGAGAGGGUUGUUUACUCCa--

--------- 2 1,0

--UGGGAGAGGGUUGUUUACUCCaa-

--------- 2 1,0

--UGGGAGAGGGUUGUUUACUCCau-

--------- 2 1,0

--UGGGAGAGGGUUGUUUACUCCU--

--------- 12 1,1

```
>hsa-mir-30c-2  MA:7-29;mi:47-68    SRX018958
agauacUGUAAACAUCCUACACUCUCAGCuguggaaaguaagaaagCUGGGAGAAGGCUGUUUACUCUuucu
(((....(((((((.(((...(((((((((..............))))))))).))).)))))))....)))
-----CUGUAAACAUCCUACACUC------------------------------------------------	3
	-1,-5
------UGUAAACAUCCUACACUC------------------------------------------------	16
	0,-5
------UGUAAACAUCCUACACUCU-----------------------------------------------	6
	0,-4
------UGUAAACAUCCUACACUCUC----------------------------------------------	18
	0,-3
------UGUAAACAUCCUACACUCUCA---------------------------------------------	60
	0,-2
------UGUAAACAUCCUACACUCUCAG--------------------------------------------	105
	0,-1
------UGUAAACAUCCUACACUCUCAGa-------------------------------------------	23
	0,-1
------UGUAAACAUCCUACACUCUCAGaa------------------------------------------	13
	0,-1
------UGUAAACAUCCUACACUCUCAGu-------------------------------------------	2
	0,-1
------UGUAAACAUCCUACACUCUCAGuu------------------------------------------	2
	0,-1
------UGUAAACAUCCUACACUCUCAGC-------------------------------------------	181
	0,0
------UGUAAACAUCCUACACUCUCAGCa------------------------------------------	43
	0,0
------UGUAAACAUCCUACACUCUCAGCaa-----------------------------------------	3
	0,0
------UGUAAACAUCCUACACUCUCAGCau-----------------------------------------	9
	0,0
------UGUAAACAUCCUACACUCUCAGCU------------------------------------------	634
	0,1
------UGUAAACAUCCUACACUCUCAGCUa-----------------------------------------	28
	0,1
------UGUAAACAUCCUACACUCUCAGCUu-----------------------------------------	38
	0,1
-------GUAAACAUCCUACACUCUCA---------------------------------------------	2
```

1,-2

-------GUAAACAUCCUACACUCUCAG-- 2

1,-1

-------GUAAACAUCCUACACUCUCAGC--- 5

1,0

-------GUAAACAUCCUACACUCUCAGCau-- 2

1,0

-------GUAAACAUCCUACACUCUCAGCU-- 9

1,1

--------UAAACAUCCUACACUCUCAGCU-- 4

2,1

--CUGGGAGAAGGCUGUUUA-------- 24

0,-4

--CUGGGAGAAGGCUGUUUAa------- 7

0,-4

--CUGGGAGAAGGCUGUUUAau------ 5

0,-4

--CUGGGAGAAGGCUGUUUAu------- 2

0,-4

--CUGGGAGAAGGCUGUUUAuu------ 3

0,-4

--CUGGGAGAAGGCUGUUUAC------- 77

0,-3

--CUGGGAGAAGGCUGUUUACa------ 5

0,-3

--CUGGGAGAAGGCUGUUUACU------ 1584

0,-2

--CUGGGAGAAGGCUGUUUACUa----- 17

0,-2

--CUGGGAGAAGGCUGUUUACUaa---- 15

0,-2

--CUGGGAGAAGGCUGUUUACUau---- 4

0,-2

--CUGGGAGAAGGCUGUUUACUu----- 14

0,-2

--CUGGGAGAAGGCUGUUUACUuu---- 3

0,-2

--CUGGGAGAAGGCUGUUUACUC----- 604

0,-1

--CUGGGAGAAGGCUGUUUACUCa---- 13

0,-1

--CUGGGAGAAGGCUGUUUACUCaa--- 3

0,-1

--CUGGGAGAAGGCUGUUUACUCau--- 2

0,-1

--CUGGGAGAAGGCUGUUUACUCU---- 138

0,0

--CUGGGAGAAGGCUGUUUACUCUa--- 4

0,0

--CUGGGAGAAGGCUGUUUACUCUU--- 7

0,1

---UGGGAGAAGGCUGUUUAC------- 2

1,-3

---UGGGAGAAGGCUGUUUACU------ 32

1,-2

---UGGGAGAAGGCUGUUUACUa----- 2

1,-2

---UGGGAGAAGGCUGUUUACUC----- 30

1,-1

---UGGGAGAAGGCUGUUUACUCU---- 5

1,0

---UGGGAGAAGGCUGUUUACUCUU--- 6

1,1

--GGGAGAAGGCUGUUUACU------ 2

2,-2

>hsa-mir-30d MA:6-27;mi:46-67 SRX018958

guuguUGUAAACAUCCCCGACUGGAAGcuguaagacacagcuaagCUUUCAGUCAGAUGUUUGCUGCuac

...((.(((((((((...(((((((((((...((......)).))))))))))).))))))))).))...

----UUGUAAACAUCCCCGACUGGAAGC-- 3 -1,1

----UUGUAAACAUCCCCGACUGGAAGCU--- 5 -1,2

-----UGUAAACAUCCCCGACUG--- 101 0,-4

-----UGUAAACAUCCCCGACUGG-- 94 0,-3

-----UGUAAACAUCCCCGACUGGA--- 259 0,-2

-----UGUAAACAUCCCCGACUGGAA-- 762 0,-1

-----UGUAAACAUCCCCGACUGGAAa--- 649 0,-1

-----UGUAAACAUCCCCGACUGGAAaa--- 67 0,-1
-----UGUAAACAUCCCCGACUGGAAau--- 12 0,-1
-----UGUAAACAUCCCCGACUGGAAu-- 37 0,-1
-----UGUAAACAUCCCCGACUGGAAG-- 1194 0,0
-----UGUAAACAUCCCCGACUGGAAGa--- 1754 0,0
-----UGUAAACAUCCCCGACUGGAAGaa-- 165 0,0
-----UGUAAACAUCCCCGACUGGAAGau-- 168 0,0
-----UGUAAACAUCCCCGACUGGAAGu--- 208 0,0
-----UGUAAACAUCCCCGACUGGAAGuu-- 52 0,0
-----UGUAAACAUCCCCGACUGGAAGC--- 5213 0,1
-----UGUAAACAUCCCCGACUGGAAGCa-- 1334 0,1
-----UGUAAACAUCCCCGACUGGAAGCaa--------------------------------------- 18 0,1
-----UGUAAACAUCCCCGACUGGAAGCau--------------------------------------- 43 0,1
-----UGUAAACAUCCCCGACUGGAAGCU-- 43988 0,2
-----UGUAAACAUCCCCGACUGGAAGCUa--------------------------------------- 320 0,2
-----UGUAAACAUCCCCGACUGGAAGCUu--------------------------------------- 2359 0,2
-----UGUAAACAUCCCCGACUGGAAGCUG--------------------------------------- 2 0,3
------GUAAACAUCCCCGACUGGAA--- 10 1,-1
------GUAAACAUCCCCGACUGGAAa-- 14 1,-1
------GUAAACAUCCCCGACUGGAAau--- 4 1,-1
------GUAAACAUCCCCGACUGGAAG-- 69 1,0
------GUAAACAUCCCCGACUGGAAGa--- 31 1,0
------GUAAACAUCCCCGACUGGAAGaa-- 3 1,0
------GUAAACAUCCCCGACUGGAAGau-- 3 1,0
------GUAAACAUCCCCGACUGGAAGu--- 7 1,0
------GUAAACAUCCCCGACUGGAAGC--- 111 1,1
------GUAAACAUCCCCGACUGGAAGCa-- 22 1,1
------GUAAACAUCCCCGACUGGAAGCU-- 743 1,2
------GUAAACAUCCCCGACUGGAAGCUa--------------------------------------- 7 1,2
------GUAAACAUCCCCGACUGGAAGCUaa-------------------------------------- 3 1,2
------GUAAACAUCCCCGACUGGAAGCUau-------------------------------------- 6 1,2
------GUAAACAUCCCCGACUGGAAGCUu--------------------------------------- 57 1,2

------GUAAACAUCCCCGACUGGAAGCUuu--------------------------------------- 16 1,2
-------UAAACAUCCCCGACUGGAAGCU--- 9   2,2
---CUUUCAGUCAGAUGUUUGCU----- 8   0,-2
---CUUUCAGUCAGAUGUUUGCUGCU-- 2   0,1
--UUUCAGUCAGAUGUUUGCU----- 2   1,-2

>hsa-mir-30e   MA:17-38;mi:59-80   SRX018958
gggcagucuuugcuacUGUAAACAUCCUUGACUGGAAGcuguaagguguucagaggagCUUUCAGUCGGAUGUUUACAGC
ggcaggcugcca
.((((((((..((..(((((((((((..((((((((((.(...(....)...).))))))))))))))))))))))))).
.)))))))))).
---------------CUGUAAACAUCCUUGACUG--
------------   2   -1,-4
---------------CUGUAAACAUCCUUGACUGGAAG--
------------   3   -1,0
---------------CUGUAAACAUCCUUGACUGGAAGa---
------------   7   -1,0
---------------CUGUAAACAUCCUUGACUGGAAGau--
------------   2   -1,0
---------------CUGUAAACAUCCUUGACUGGAAGC---
------------   21   -1,1
---------------CUGUAAACAUCCUUGACUGGAAGCa--
------------   2   -1,1
---------------CUGUAAACAUCCUUGACUGGAAGCU--
------------   24   -1,2
----------------UGUAAACAUCCUUGACUG--
------------   28   0,-4
----------------UGUAAACAUCCUUGACUGG---
------------   32   0,-3
----------------UGUAAACAUCCUUGACUGGu--
------------   3   0,-3
----------------UGUAAACAUCCUUGACUGGA--
------------   22   0,-2
----------------UGUAAACAUCCUUGACUGGAA---
------------   107 0,-1
----------------UGUAAACAUCCUUGACUGGAAa--
------------   41   0,-1
----------------UGUAAACAUCCUUGACUGGAAaa---

------------ 7 0,-1

----------------UGUAAACAUCCUUGACUGGAAu--

------------ 8 0,-1

----------------UGUAAACAUCCUUGACUGGAAG--

------------ 210 0,0

----------------UGUAAACAUCCUUGACUGGAAGa---------------------------------------

------------ 482 0,0

----------------UGUAAACAUCCUUGACUGGAAGaa--------------------------------------

------------ 136 0,0

----------------UGUAAACAUCCUUGACUGGAAGau--------------------------------------

------------ 17 0,0

----------------UGUAAACAUCCUUGACUGGAAGu---------------------------------------

------------ 10 0,0

----------------UGUAAACAUCCUUGACUGGAAGuu--------------------------------------

------------ 3 0,0

----------------UGUAAACAUCCUUGACUGGAAGC---------------------------------------

------------ 2795 0,1

----------------UGUAAACAUCCUUGACUGGAAGCa--------------------------------------

------------ 249 0,1

----------------UGUAAACAUCCUUGACUGGAAGCaa-------------------------------------

------------ 2 0,1

----------------UGUAAACAUCCUUGACUGGAAGCau-------------------------------------

------------ 7 0,1

----------------UGUAAACAUCCUUGACUGGAAGCU--------------------------------------

------------ 3171 0,2

----------------UGUAAACAUCCUUGACUGGAAGCUa-------------------------------------

------------ 31 0,2

----------------UGUAAACAUCCUUGACUGGAAGCUu-------------------------------------

------------ 524 0,2

----------------UGUAAACAUCCUUGACUGGAAGCUG-------------------------------------

------------ 2 0,3

-----------------GUAAACAUCCUUGACUGGAA---

------------ 10 1,-1

-----------------GUAAACAUCCUUGACUGGAAG--

------------ 54 1,0

-----------------GUAAACAUCCUUGACUGGAAGa---------------------------------------

------------ 18 1,0

-----------------GUAAACAUCCUUGACUGGAAGaa--------------------------------------

```
-----------     2    1,0
----------------GUAAACAUCCUUGACUGGAAGC---------------------------------------
-----------     223  1,1
----------------GUAAACAUCCUUGACUGGAAGCa--------------------------------------
-----------     47   1,1
----------------GUAAACAUCCUUGACUGGAAGCU--------------------------------------
-----------     207  1,2
----------------GUAAACAUCCUUGACUGGAAGCUau------------------------------------
-----------     5    1,2
----------------GUAAACAUCCUUGACUGGAAGCUu-------------------------------------
-----------     21   1,2
----------------GUAAACAUCCUUGACUGGAAGCUuu------------------------------------
-----------     11   1,2
-----------------UAAACAUCCUUGACUGGAAGC---------------------------------------
-----------     11   2,1
-----------------UAAACAUCCUUGACUGGAAGCU--------------------------------------
-----------     9    2,2
-------------------------------------------------------CUUUCAGUCGGAUGUUUA----
-----------     14   0,-4
-------------------------------------------------------CUUUCAGUCGGAUGUUUAa---
-----------     7    0,-4
-------------------------------------------------------CUUUCAGUCGGAUGUUUAC---
-----------     13   0,-3
-------------------------------------------------------CUUUCAGUCGGAUGUUUACA--
-----------     30   0,-2
-------------------------------------------------------CUUUCAGUCGGAUGUUUACAa-
-----------     3    0,-2
-------------------------------------------------------CUUUCAGUCGGAUGUUUACAG-
-----------     109  0,-1
-------------------------------------------------------CUUUCAGUCGGAUGUUUACAGa
-----------     12   0,-1
-------------------------------------------------------CUUUCAGUCGGAUGUUUACAGu
-----------     158  0,-1
-------------------------------------------------------CUUUCAGUCGGAUGUUUACAGu
u----------     18   0,-1
-------------------------------------------------------CUUUCAGUCGGAUGUUUACAGC
-----------     130  0,0
-------------------------------------------------------CUUUCAGUCGGAUGUUUACAGC
```

a----------   122 0,0
--CUUUCAGUCGGAUGUUUACAGC
aa---------   9   0,0
--CUUUCAGUCGGAUGUUUACAGC
au---------   8   0,0
--CUUUCAGUCGGAUGUUUACAGC
u----------   3   0,0
---UUUCAGUCGGAUGUUUACAGu
-----------   2   1,-1
---UUUCAGUCGGAUGUUUACAGC
-----------   5   1,0
---UUUCAGUCGGAUGUUUACAGC
a----------   7   1,0

>hsa-mir-31 MA:8-28;mi:44-65   SRX018958
ggagaggAGGCAAGAUGCUGGCAUAGCUguugaacugggaaccUGCUAUGCCAACAUAUUGCCAUcuuucc
((((((..(((((.(((.(((((((((.(((........)))..))))))))).))).)))))..))))))
-------AGGCAAGAUGCUGGCAUAGaa--- 3   0,-2
-------AGGCAAGAUGCUGGCAUAGCU--- 12 0,0
-------AGGCAAGAUGCUGGCAUAGCUGa--- 3   0,1
-------AGGCAAGAUGCUGGCAUAGCUGU--- 3   0,2

>hsa-mir-3121   3p:47-68   SRX018958
aaaugguuauguccuuugccuauucuauuuaagacacccuguaccuUAAAUAGAGUAGGCAAAGGACAgaaacauuu
(((((....((((((((((((((((((((((((((.....))..))))))))))))))))))))))))....)))))
--UAAAUAGAGUAGGCAAAGGACAa--------
    2   0,0

>hsa-mir-3129   5p:9-30 SRX018958
guacuuggGCAGUAGUGUAGAGAUUGGUUUgccuguuaaugaauucaaacuaaucucuacacugcugcccaagagc
...((((((((((((((((((((((((((((...(((....))).))))))))))))))))))))))))))))...
--------GCAGUAGUGUAGAGAUUGGUU---
    3   0,-1
---------CAGUAGUGUAGAGAUUGGUUUG---
    2   1,1
----------AGUAGUGUAGAGAUUGGUUU--
    2   2,0
----------AGUAGUGUAGAGAUUGGUUUG---

8   2,1
----------AGUAGUGUAGAGAUUGGUUUGC---
7   2,2

>hsa-mir-3130-1 5p:13-33;3p:44-64   SRX018958
cuugucaugucuUACCCAGUCUCCGGUGCAGCCuguugucaagGCUGCACCGGAGACUGGGUAAgacaugacaag
((((((((((((((((((((((((((((((((((.......))))))))))))))))))))))))))))))))))
---GCUGCACCGGAGACUGGGUAA----------- 21
0,0

>hsa-mir-3130-2 5p:13-33;3p:44-64   SRX018958
cuugucaugucuUACCCAGUCUCCGGUGCAGCCuugacaacagGCUGCACCGGAGACUGGGUAAgacaugacaag
((((((((((((((((((((((((((((((((((.......))))))))))))))))))))))))))))))))))
---GCUGCACCGGAGACUGGGUAA----------- 21
0,0

>hsa-mir-3133   5p:10-31   SRX018958
cagaaauugUAAAGAACUCUUAAAACCCAAUaguaaaaagacaaccuguugaguuuuaagaguucuuuauauauucug
(((((..(((((((((((((((((((.((((((............)))))).)))))))))))))))))))..)))))
---------UAAAGAACUCUUAAAACCCAAU---
2   0,0
----------AAAGAACUCUUAAAACCCAAU---
3   1,0
----------AAAGAACUCUUAAAACCCAAUA--
3   1,1

>hsa-mir-3140   3p:60-81   SRX018958
ccucuugagguaccugaauuaccaaaagcuuuauguauucugaaguuauugaaaauaagAGCUUUUGGGAAUUCAGGUAG
Uucaggagug
.((((((((.((((((((((.(((((((((((.(((.(((..........))).)))))))))))))).)))))))))).
))))))))..
---AGCUUUUGGGAAUUCAGGU--
----------   2   0,-3

>hsa-mir-3144   5p:13-34;3p:48-69   SRX018958
aacuacacuuuaAGGGGACCAAAGAGAUAUAUAGauaucagcuaccuAUAUACCUGUUCGGUCUCUUUAaaguguaguu
((((((((((((((((((((.((.((.(((((((...........))))))).)).)).))))))))))))))))))))
------------AGGGGACCAAAGAGAUAUAUAG---

3   0,0

>hsa-mir-3145   3p:49-72   SRX018958
uauaugaguucaacuccaaacacucaaaacucauuguugaauggaaugAGAUAUUUUGAGUGUUUGGAAUUGaacucgua
ua
((((((((((((.((((((((((((((((((((((.(.....).))))))...))))))))))))))))).))))))))))
))
--AGAUAUUUUGAGUGUUUGGAAUUG--------
--   2   0,0

>hsa-mir-3154   3p:54-75   SRX018958
ggccccuccuucucagccccagcucccgcucaccccugccacgucaaaggaggCAGAAGGGGAGUUGGGAGCAGAgaggg
gacc
((((((((..(((..((((((((((((.((.....(((((.(......)..))))).))))))))))))).))))))))))
).))
---CAGAAGGGGAGUUGGGAG---------
----   2   0,-4
--AGAAGGGGAGUUGGGAGCAau-----
----   2   1,-2

>hsa-mir-3160-1 3p:54-75   SRX018958
ggaccugcccugggcuuucuagucucagcucuccuccagcucagcuggucaggAGAGCUGAGACUAGAAAGCCCAgggca
gguuc
((...)))))..))))))))))))))))))))))))))))))
)))))
---AGAGCUGAGACUAGAAAGCCC------
-----   4   0,-1

>hsa-mir-3160-2 3p:52-73   SRX018958
accugcccugggcuuucuagucucagcucuccugaccagcugagcuggaggAGAGCUGAGACUAGAAAGCCCAgggcagg
u
(((((((((((((((((((((((((((((((((..(((((...)))))))))))))))))))))))))))))))))))))
)
---AGAGCUGAGACUAGAAAGCCC--------
-    4   0,-1

>hsa-mir-3163   5p:10-31   SRX018958
uuccucaucUAUAAAAUGAGGGCAGUAAGACcuuccuuccuugucuuacuacccccauuuuauagaugaggaa

.((((((((((((((((.(((.((((((((..........)))))))).))).)))))))))))))))).
---------UAUAAAAUGAGGGCAGUAAG--- 3
0,-2

>hsa-mir-3165 5p:10-31 SRX018958
ccgguggcaAGGUGGAUGCAAUGUGACCUCAacucuugguccucugaggucacauuguauccaccuuaccacugg
((((((.((((((((((((((((((((((((.....))....)))))))))))))))))))))).)))))))
---------AGGUGGAUGCAAUGUGACCU--- 5
0,-2

>hsa-mir-3170 5p:10-31 SRX018958
cugguaacaCUGGGGUUCUGAGACAGACAGUguuagcuccagaagcauugccugucuuagaaccccuauguuaccag
(((((((((..((((((((((((((.(((((((.........))))))).))))))))))))))..)))))))))
---------CUGGGGUUCUGAGACAGACAGU--
4 0,0

>hsa-mir-3173 3p:43-64 SRX018958
ucccugcccugccuguuuucuccuuugugauuuuaugagaacAAAGGAGGAAAUAGGCAGGCCAggga
((((((.((((((((((((((((((((((..((....))..)))))))))))))))))))))).))))))
--AAAGGAGGAAAUAGGCAGGCCAuu-- 3 0,0
---AAGGAGGAAAUAGGCAGGCCAu--- 2 1,0

>hsa-mir-3174 5p:10-32 SRX018958
guuaccuggUAGUGAGUUAGAGAUGCAGAGCCcugggcuccucagcaaaccuacuggaucugcauuuuaauucacaugca
ugguaau
(((((..(((((((((.((((((((((..((.(((((....)))...))....)).)))))))))))))))).))).
.))))))
---------UAGUGAGUUAGAGAUGCAGAGCCC---
------- 2 0,1

>hsa-mir-3179-1 3p:52-73 SRX018958
caggaucacagacguuuaaauuacacuccuucugcugugccuuacagcaguAGAAGGGGUGAAAUUUAAACGUcugugau
ccug
((((((((((((((((((((.((((((((((((((.......)))))))))))))).))))))))))))))))))
))))
---UAGAAGGGGUGAAAUUUAAACG--------
---- 2 -1,-1
---UAGAAGGGGUGAAAUUUAAACGU-------

---- 3 -1,0
---AGAAGGGGUGAAAUUUAAACG--------
---- 2 0,-1
---AGAAGGGGUGAAAUUUAAACGU-------
---- 5 0,0

>hsa-mir-3179-2 3p:52-73 SRX018958
caggaucacagacguuuaaauuacacuccuucugcugugccuuacagcaguAGAAGGGGUGAAAUUUAAACGUcugugau
ccug
((((((((((((((((((((.(((((((((((((((((........))))))))))))))))).))))))))))))))))
))))
--UAGAAGGGGUGAAAUUUAAACG--------
---- 2 -1,-1
--UAGAAGGGGUGAAAUUUAAACGU-------
---- 3 -1,0
---AGAAGGGGUGAAAUUUAAACG--------
---- 2 0,-1
---AGAAGGGGUGAAAUUUAAACGU-------
---- 5 0,0

>hsa-mir-3179-3 3p:52-73 SRX018958
caggaucacagacguuuaaauuacacuccuucugcugugccuuacagcaguAGAAGGGGUGAAAUUUAAACGUcugugau
ccug
((((((((((((((((((((.(((((((((((((((((........))))))))))))))))).))))))))))))))))
))))
--UAGAAGGGGUGAAAUUUAAACG--------
---- 2 -1,-1
--UAGAAGGGGUGAAAUUUAAACGU-------
---- 3 -1,0
---AGAAGGGGUGAAAUUUAAACG--------
---- 2 0,-1
---AGAAGGGGUGAAAUUUAAACGU-------
---- 5 0,0

>hsa-mir-3188 3p:53-75 SRX018958
ggcgccuccugcucugcugugccgccagggccuccccuagcgcgccuucuggAGAGGCUUUGUGCGGAUACGGGGcugga
ggccu
...((((((.((((((.(((.(((((((((((((..((((........)))).))))))))).))))))))))))).)))

))))..
---AGAGGCUUUGUGCGGAUACaa-------
-----   2   0,-4
---AGAGGCUUUGUGCGGAUACGGGGau---
-----   2   0,0

>hsa-mir-3190   3p:47-69   SRX018958
cuggggucaccugucuggccagcuacguccccacggcccuugucagUGUGGAAGGUAGACGGCCAGAGAggugaccccgg
((((((((((.((((((..((((.(((.(((((....))).))).)))..))))..))))))).)))))))))))
---GUGGAAGGUAGACGGCCAGAGA-----------
    4   1,0

>hsa-mir-3192   5p:10-32   SRX018958
ggaagggauUCUGGGAGGUUGUAGCAGUGGAAaaaguucuuuucuucccucugaucgcccucucagcucuuuccuucu
(((((((..((((((((.((...(((.((((.(((....))).)))).)))..)).))))))))....))))))))
---------UCUGGGAGGUUGUAGCAGU--
    2   0,-4

>hsa-mir-3193   5p:1-22 SRX018958
UCCUGCGUAGGAUCUGAGGAGUggacgagucucauuacccagcuccugagcagga
(((((.(((((.(((.((((((((....)).)))).))))).))))).))))))
UCCUGCGUAGGAUCUGAGGAGU--------------------------------- 2   0,0

>hsa-mir-3194   5p:10-30   SRX018958
agguggcagGGCCAGCCACCAGGAGGGCUGcgugccacccgggcagcucugcugcucacuggcagugucaccu
(((((((..(((((....(((.((((((((.((.....)).)))))))).)))....)))))..))))))))
--AGCUCUGCUGCUCACUGGCAuu-------   3

>hsa-mir-32 MA:6-27;mi:47-68   SRX018958
ggagaUAUUGCACAUUACUAAGUUGCAuguugucacggccucaaugCAAUUUAGUGUGUGUGAUAUUUuc
(((((((..(((((.((((((((((((((..((....))..).))))))))))))))))..))))))))
----AUAUUGCACAUUACUAAGUUG--- 2
    -1,-2
-----UAUUGCACAUUACUAAGU--- 12 0,-4
-----UAUUGCACAUUACUAAGUU-- 27 0,-3
-----UAUUGCACAUUACUAAGUUG--- 65 0,-2
-----UAUUGCACAUUACUAAGUUGa-- 2   0,-2
-----UAUUGCACAUUACUAAGUUGC-- 29 0,-1

-----UAUUGCACAUUACUAAGUUGCA--- 4   0,0
-----UAUUGCACAUUACUAAGUUGCAa-- 3   0,0
-----UAUUGCACAUUACUAAGUUGCAU-- 4   0,1
-----UAUUGCACAUUACUAAGUUGCAUu--- 2   0,1
------AUUGCACAUUACUAAGUUG--- 5   1,-2
--CAAUUUAGUGUGUGUGAUAUU--- 9   0,-1

>hsa-mir-320a   3p:48-69   SRX018958
gcuucgcuccccuccgccuucucuucccgguucuucccggagucgggAAAAGCUGGGUUGAGAGGGCGAaaaaggaugag
gu
((((((.(((....(((((((((..(((((((.((((((....)))))).)))))))..))))))))).....)))))))
))
--GAAAAGCUGGGUUGAGAGG---------------
-- 3   -1,-4
--GAAAAGCUGGGUUGAGAGGG--------------
-- 3   -1,-3
--GAAAAGCUGGGUUGAGAGGGC-------------
-- 15 -1,-2
--GAAAAGCUGGGUUGAGAGGGCG------------
-- 14 -1,-1
--GAAAAGCUGGGUUGAGAGGGCGu-----------
-- 21 -1,-1
--GAAAAGCUGGGUUGAGAGGGCGuu----------
-- 2   -1,-1
--GAAAAGCUGGGUUGAGAGGGCGA-----------
-- 60 -1,0
--GAAAAGCUGGGUUGAGAGGGCGAu----------
-- 11 -1,0
--GAAAAGCUGGGUUGAGAGGGCGAA----------
-- 3   -1,1
---AAAAGCUGGGUUGAGAGG---------------
-- 224 0,-4
---AAAAGCUGGGUUGAGAGGu--------------
-- 3   0,-4
---AAAAGCUGGGUUGAGAGGG--------------
-- 296 0,-3
---AAAAGCUGGGUUGAGAGGGa-------------
-- 18 0,-3

---AAAAGCUGGGUUGAGAGGGaa------------
-- 16 0,-3
---AAAAGCUGGGUUGAGAGGGau------------
-- 7 0,-3
---AAAAGCUGGGUUGAGAGGGC-------------
-- 1049 0,-2
---AAAAGCUGGGUUGAGAGGGCu------------
-- 3 0,-2
---AAAAGCUGGGUUGAGAGGGCG------------
-- 1280 0,-1
---AAAAGCUGGGUUGAGAGGGCGu-----------
-- 1100 0,-1
---AAAAGCUGGGUUGAGAGGGCGuu----------
-- 476 0,-1
---AAAAGCUGGGUUGAGAGGGCGA-----------
-- 9463 0,0
---AAAAGCUGGGUUGAGAGGGCGAu----------
-- 2918 0,0
---AAAAGCUGGGUUGAGAGGGCGAuu---------
-- 525 0,0
---AAAAGCUGGGUUGAGAGGGCGAA----------
-- 4279 0,1
---AAAAGCUGGGUUGAGAGGGCGAAu---------
-- 778 0,1
---AAAAGCUGGGUUGAGAGGGCGAAuu--------
-- 143 0,1
---AAAAGCUGGGUUGAGAGGGCGAAA---------
-- 611 0,2
---AAAAGCUGGGUUGAGAGGGCGAAAu--------
-- 180 0,2
---AAAAGCUGGGUUGAGAGGGCGAAAA--------
-- 54 0,3
--AAAGCUGGGUUGAGAGGG-------------
-- 7 1,-3
--AAAGCUGGGUUGAGAGGGa------------
-- 2 1,-3
--AAAGCUGGGUUGAGAGGGC------------
-- 55 1,-2

--AAAGCUGGGUUGAGAGGGCG------------
-- 89 1,-1
--AAAGCUGGGUUGAGAGGGCGu-----------
-- 32 1,-1
--AAAGCUGGGUUGAGAGGGCGuu----------
-- 25 1,-1
--AAAGCUGGGUUGAGAGGGCGA-----------
-- 466 1,0
--AAAGCUGGGUUGAGAGGGCGAu----------
-- 168 1,0
--AAAGCUGGGUUGAGAGGGCGAuu---------
-- 47 1,0
--AAAGCUGGGUUGAGAGGGCGAA----------
-- 428 1,1
--AAAGCUGGGUUGAGAGGGCGAAu---------
-- 129 1,1
--AAAGCUGGGUUGAGAGGGCGAAuu--------
-- 50 1,1
--AAAGCUGGGUUGAGAGGGCGAAA---------
-- 145 1,2
--AAAGCUGGGUUGAGAGGGCGAAAu--------
-- 45 1,2
--AAAGCUGGGUUGAGAGGGCGAAAuu-------
-- 2 1,2
--AAAGCUGGGUUGAGAGGGCGAAAA--------
-- 20 1,3
---AAGCUGGGUUGAGAGGGC-------------
-- 2 2,-2
---AAGCUGGGUUGAGAGGGCG------------
-- 3 2,-1
---AAGCUGGGUUGAGAGGGCGA-----------
-- 26 2,0
---AAGCUGGGUUGAGAGGGCGAu----------
-- 3 2,0
---AAGCUGGGUUGAGAGGGCGAuu---------
-- 13 2,0
---AAGCUGGGUUGAGAGGGCGAA----------
-- 35 2,1

---AAGCUGGGUUGAGAGGGCGAAu---------
--   17   2,1
---AAGCUGGGUUGAGAGGGCGAAuu--------
--   6   2,1
---AAGCUGGGUUGAGAGGGCGAAA---------
--   46   2,2
---AAGCUGGGUUGAGAGGGCGAAAu--------
--   22   2,2
---AAGCUGGGUUGAGAGGGCGAAAA--------
--   3   2,3
---AAGCUGGGUUGAGAGGGCGAAAAu-------
--   3   2,3
---AAGCUGGGUUGAGAGGGCGAAAAAu------
--   2   2,4

>hsa-mir-320b-1 3p:39-60   SRX018958
aauuaaucccucucuuucuaguucuuccuagagugaggAAAAGCUGGGUUGAGAGGGCAAacaaauuaacuaauuaauu
........((((...((((((......)))))).))))...
-------------------------------------GAAAAGCUGGGUUGAGAGG-----------------------
     3   -1,-4
-------------------------------------GAAAAGCUGGGUUGAGAGGG----------------------
     3   -1,-3
-------------------------------------GAAAAGCUGGGUUGAGAGGGC---------------------
     15   -1,-2
-------------------------------------GAAAAGCUGGGUUGAGAGGGCA--------------------
     2   -1,-1
-------------------------------------GAAAAGCUGGGUUGAGAGGGCAu-------------------
     2   -1,-1
-------------------------------------GAAAAGCUGGGUUGAGAGGGCAA-------------------
     2   -1,0
--------------------------------------AAAAGCUGGGUUGAGAGG-----------------------
     224 0,-4
--------------------------------------AAAAGCUGGGUUGAGAGGu----------------------
     3   0,-4
--------------------------------------AAAAGCUGGGUUGAGAGGG----------------------
     296 0,-3
--------------------------------------AAAAGCUGGGUUGAGAGGGa---------------------
     18 0,-3

--------------------------------------AAAAGCUGGGUUGAGAGGGaa--------------------

16 0,-3

--------------------------------------AAAAGCUGGGUUGAGAGGGau--------------------

7 0,-3

--------------------------------------AAAAGCUGGGUUGAGAGGGC---------------------

1049 0,-2

--------------------------------------AAAAGCUGGGUUGAGAGGGCu--------------------

3 0,-2

--------------------------------------AAAAGCUGGGUUGAGAGGGCA--------------------

128 0,-1

--------------------------------------AAAAGCUGGGUUGAGAGGGCAu-------------------

206 0,-1

--------------------------------------AAAAGCUGGGUUGAGAGGGCAuu------------------

67 0,-1

--------------------------------------AAAAGCUGGGUUGAGAGGGCAA-------------------

163 0,0

--------------------------------------AAAAGCUGGGUUGAGAGGGCAAu------------------

34 0,0

--------------------------------------AAAAGCUGGGUUGAGAGGGCAAuu-----------------

2 0,0

--------------------------------------AAAAGCUGGGUUGAGAGGGCAAA------------------

23 0,1

--------------------------------------AAAAGCUGGGUUGAGAGGGCAAAu-----------------

9 0,1

---------------------------------------AAAGCUGGGUUGAGAGGG----------------------

7 1,-3

---------------------------------------AAAGCUGGGUUGAGAGGGa---------------------

2 1,-3

---------------------------------------AAAGCUGGGUUGAGAGGGC---------------------

55 1,-2

---------------------------------------AAAGCUGGGUUGAGAGGGCA--------------------

3 1,-1

---------------------------------------AAAGCUGGGUUGAGAGGGCAu-------------------

6 1,-1

---------------------------------------AAAGCUGGGUUGAGAGGGCAA-------------------

4 1,0

---------------------------------------AAAGCUGGGUUGAGAGGGCAAu------------------

2 1,0

--------------------------------------AAAGCUGGGUUGAGAGGGCAAA-----------------
3 1,1
---------------------------------------AAGCUGGGUUGAGAGGGC--------------------
2 2,-2

>hsa-mir-320b-2 3p:72-93 SRX018958
uguuauuuuuugucuucuaccuaagaauucugucucuuaggcuuucucuucccagauuucccaaaguugggAAAAGCUGG
GUUGAGAGGGCAAaaggaaaaaaaaaagaauucugucucugacauaauuagauagggaa
......................((((((((.((.(((..((((((((..(((((.(((((((....)))))))..))))
)..))))))))..)))))......))))))))...........................
---GAAAAGCUGG
GUUGAGAGG-- 3 -1,-4
---GAAAAGCUGG
GUUGAGAGGG--- 3 -1,-3
---GAAAAGCUGG
GUUGAGAGGGC-- 15 -1,-2
---GAAAAGCUGG
GUUGAGAGGGCA--- 2 -1,-1
---GAAAAGCUGG
GUUGAGAGGGCAu-- 2 -1,-1
---GAAAAGCUGG
GUUGAGAGGGCAA-- 2 -1,0
--AAAAGCUGG
GUUGAGAGG-- 224 0,-4
--AAAAGCUGG
GUUGAGAGGu--- 3 0,-4
--AAAAGCUGG
GUUGAGAGGG--- 296 0,-3
--AAAAGCUGG
GUUGAGAGGGa-- 18 0,-3
--AAAAGCUGG
GUUGAGAGGGaa--- 16 0,-3
--AAAAGCUGG
GUUGAGAGGGau--- 7 0,-3
--AAAAGCUGG
GUUGAGAGGGC-- 1049 0,-2
--AAAAGCUGG
GUUGAGAGGGCu--- 3 0,-2

---AAAAGCUGG
GUUGAGAGGGCA--- 128 0,-1
---AAAAGCUGG
GUUGAGAGGGCAu-- 206 0,-1
---AAAAGCUGG
GUUGAGAGGGCAuu--- 67 0,-1
---AAAAGCUGG
GUUGAGAGGGCAA-- 163 0,0
---AAAAGCUGG
GUUGAGAGGGCAAu--- 34 0,0
---AAAAGCUGG
GUUGAGAGGGCAAuu-- 2 0,0
---AAAAGCUGG
GUUGAGAGGGCAAA--- 23 0,1
---AAAAGCUGG
GUUGAGAGGGCAAAu-- 9 0,1
---AAAAGCUGG
GUUGAGAGGGCAAAA-- 5 0,2
---AAAAGCUGG
GUUGAGAGGGCAAAAu--- 3 0,2
--AAAGCUGG
GUUGAGAGGG--- 7 1,-3
--AAAGCUGG
GUUGAGAGGGa-- 2 1,-3
--AAAGCUGG
GUUGAGAGGGC-- 55 1,-2
--AAAGCUGG
GUUGAGAGGGCA--- 3 1,-1
--AAAGCUGG
GUUGAGAGGGCAu-- 6 1,-1
--AAAGCUGG
GUUGAGAGGGCAA-- 4 1,0
--AAAGCUGG
GUUGAGAGGGCAAu--- 2 1,0
--AAAGCUGG
GUUGAGAGGGCAAA--- 3 1,1
---AAGCUGG
GUUGAGAGGGC-- 2 2,-2

>hsa-mir-320c-1 3p:50-69   SRX018958
uuugcauuaaaaaugaggccuucucuucccaguucuucccagagucaggAAAAGCUGGGUUGAGAGGGUagaaaaaaaau
gauguagg
.((((((((........((((((((..(((((((.((((........)))).)))))))..))))))))..........)
))))))).
--GAAAAGCUGGGUUGAGAGG-------------
--------   3   -1,-2
--GAAAAGCUGGGUUGAGAGGG------------
--------   3   -1,-1
--GAAAAGCUGGGUUGAGAGGGU-----------
--------   4   -1,0
--GAAAAGCUGGGUUGAGAGGGUAa---------
--------   2   -1,1
---AAAAGCUGGGUUGAGAGG-------------
--------   224 0,-2
---AAAAGCUGGGUUGAGAGGu------------
--------   3   0,-2
---AAAAGCUGGGUUGAGAGGG------------
--------   296 0,-1
---AAAAGCUGGGUUGAGAGGGa-----------
--------   18   0,-1
---AAAAGCUGGGUUGAGAGGGaa----------
--------   16   0,-1
---AAAAGCUGGGUUGAGAGGGau----------
--------   7   0,-1
---AAAAGCUGGGUUGAGAGGGU-----------
--------   202 0,0
---AAAAGCUGGGUUGAGAGGGUu----------
--------   10   0,0
---AAAAGCUGGGUUGAGAGGGUuu---------
--------   7   0,0
---AAAAGCUGGGUUGAGAGGGUA----------
--------   3   0,1
---AAAAGCUGGGUUGAGAGGGUAa---------
--------   14   0,1
---AAAAGCUGGGUUGAGAGGGUAaa--------
--------   3   0,1

---AAAAGCUGGGUUGAGAGGGUAau--------
--------   4   0,1
---AAAAGCUGGGUUGAGAGGGUAu---------
--------   20   0,1
---AAAAGCUGGGUUGAGAGGGUAuu--------
--------   3   0,1
--AAAGCUGGGUUGAGAGGG------------
--------   7   1,-1
--AAAGCUGGGUUGAGAGGGa-----------
--------   2   1,-1

>hsa-mir-320c-2 3p:31-50   SRX018958
cuucucuuuccaguucuucccagaauugggAAAAGCUGGGUUGAGAGGGU
((((((..(((((((.(((((......))))).)))))))..))))))..
-----------------------------GAAAAGCUGGGUUGAGAGG-- 3   -1,-2
-----------------------------GAAAAGCUGGGUUGAGAGGG- 3   -1,-1
-----------------------------GAAAAGCUGGGUUGAGAGGGU 4   -1,0
------------------------------AAAAGCUGGGUUGAGAGG-- 224 0,-2
------------------------------AAAAGCUGGGUUGAGAGGu- 3   0,-2
------------------------------AAAAGCUGGGUUGAGAGGG- 296 0,-1
------------------------------AAAAGCUGGGUUGAGAGGGa 18   0,-1
------------------------------AAAAGCUGGGUUGAGAGGGaa 16   0,-1
------------------------------AAAAGCUGGGUUGAGAGGGau 7   0,-1
------------------------------AAAAGCUGGGUUGAGAGGGU 202 0,0
------------------------------AAAAGCUGGGUUGAGAGGGUu 10   0,0
------------------------------AAAAGCUGGGUUGAGAGGGUuu   7   0,0
-------------------------------AAAGCUGGGUUGAGAGGG- 7   1,-1
-------------------------------AAAGCUGGGUUGAGAGGGa 2   1,-1

>hsa-mir-320d-1 3p:30-48   SRX018958
uucucgucccaguucuucccaaaguugagAAAAGCUGGGUUGAGAGGA
((((((.(((((((.(((.((....)).))).))))))).))))))..
----------------------------GAAAAGCUGGGUUGAGAGG-   3   -1,-1
-----------------------------AAAAGCUGGGUUGAGAGG-   224 0,-1
-----------------------------AAAAGCUGGGUUGAGAGGu   3   0,-1
-----------------------------AAAAGCUGGGUUGAGAGGA   15   0,0
-----------------------------AAAAGCUGGGUUGAGAGGAa   8   0,0
-----------------------------AAAAGCUGGGUUGAGAGGAaa 4   0,0

-----------------------------AAAAGCUGGGUUGAGAGGAau 3 0,0
------------------------------AAAGCUGGGUUGAGAGGA 3 1,0

>hsa-mir-320d-2 3p:30-48 SRX018958
uucucuucccaguucuucuuggagucaggAAAAGCUGGGUUGAGAGGA
(((((..(((((((.((((((....)))))).)))))))..)))))..
----------------------------GAAAAGCUGGGUUGAGAGG- 3 -1,-1
-----------------------------AAAAGCUGGGUUGAGAGG- 224 0,-1
-----------------------------AAAAGCUGGGUUGAGAGGu 3 0,-1
-----------------------------AAAAGCUGGGUUGAGAGGA 15 0,0
-----------------------------AAAAGCUGGGUUGAGAGGAa 8 0,0
-----------------------------AAAAGCUGGGUUGAGAGGAaa 4 0,0
-----------------------------AAAAGCUGGGUUGAGAGGAau 3 0,0
------------------------------AAAGCUGGGUUGAGAGGA 3 1,0

>hsa-mir-320e 3p:35-52 SRX018958
gccuucucuucccaguucuuccuggagucggggaAAAGCUGGGUUGAGAAGGu
((((((((..(((((((.(((((.(...).))))).)))))))..))))))))
---------------------------------AAAAGCUGGGUUGAGAAGG- 2 -1,0

>hsa-mir-323 5p:16-37;3p:51-71 SRX018958
uugguacuuggagagAGGUGGUCCGUGGCGCGUUCGCuuuauuuauggcgCACAUUACACGGUCGACCUCUuugcaguau
cuaauc
..((((((.(.((((((((.(.(((((....((.((((........)))).))....))))).).)))))))).))))))
).....
---GCACAUUACACGGUCGACC------------
------ 8 -1,-3
---GCACAUUACACGGUCGACCU-----------
------ 43 -1,-2
---GCACAUUACACGGUCGACCUC----------
------ 8 -1,-1
--CACAUUACACGGUCGACC------------
------ 8 0,-3
--CACAUUACACGGUCGACCU-----------
------ 79 0,-2
--CACAUUACACGGUCGACCUC----------
------ 4 0,-1
--CACAUUACACGGUCGACCUCa---------

------ 2 0,-1
--CACAUUACACGGUCGACCUCU---------
------ 5 0,0
--CACAUUACACGGUCGACCUCUaa-------
------ 2 0,0
---ACAUUACACGGUCGACCU-----------
------ 2 1,-2

>hsa-mir-323b 5p:15-37;3p:51-72 SRX018958
ugguacucggagggAGGUUGUCCGUGGUGAGUUCGCAuuauuuaaugaugCCCAAUACACGGUCGACCUCUUuucgguau
ca
.((((.((((((((((((((.(((((........(((((((...)))))))......))))).))))))))))))))))))
).
--CCCAAUACACGGUCGACCU-----------
-- 11 0,-3
--CCCAAUACACGGUCGACCUCU---------
-- 2 0,-1

>hsa-mir-324 5p:16-38;3p:53-72 SRX018958
cugacuaugccucccCGCAUCCCCUAGGGCAUUGGUGUaaagcuggagacccACUGCCCCAGGUGCUGCUGGggguugua
guc
..((((((..(((((.(((.(.(((.(((((.(((.((..........))))).))))).))).).))).)))))..)))
)))
---------------CGCAUCCCCUAGGGCAUU---
--- 6 0,-5
---------------CGCAUCCCCUAGGGCAUUG--
--- 29 0,-4
---------------CGCAUCCCCUAGGGCAUUGG---
--- 8 0,-3
---------------CGCAUCCCCUAGGGCAUUGGU--
--- 7 0,-2
---------------CGCAUCCCCUAGGGCAUUGGUu---
--- 2 0,-2
---------------CGCAUCCCCUAGGGCAUUGGUG---
--- 2 0,-1
---------------CGCAUCCCCUAGGGCAUUGGUGa--
--- 3 0,-1
---------------CGCAUCCCCUAGGGCAUUGGUGU--

--- 25 0,0
--CCACUGCCCCAGGUGCUGCU----------
--- 2 -2,-2
--ACUGCCCCAGGUGCUGCU----------
--- 5 0,-2
--ACUGCCCCAGGUGCUGCUG---------
--- 5 0,-1
--ACUGCCCCAGGUGCUGCUGG--------
--- 16 0,0

>hsa-mir-326 3p:60-79 SRX018958
cucaucugucuguugggcuggaggcagggccuuugugaaggcggguggugcucagaucgCCUCUGGGCCCUUCCUCCAGc
cccgaggcggauuca
...((((((((...((((((((((.(((((((....((.(((((.((.....))..))))))).))))))).))))))))
))..))))))))...
---CCUCUGGGCCCUUCCUCCAG-
--------------- 8 0,0
---CCUCUGGGCCCUUCCUCCAGu
--------------- 5 0,0
---CCUCUGGGCCCUUCCUCCAGu
u-------------- 4 0,0

>hsa-mir-328 3p:48-69 SRX018958
uggaguggggggggcaggaggggcucagggagaaagugcauacagcccCUGGCCCUCUCUGCCCUUCCGUccccug
.((..(((((((((((..(((((.(((((.(...((....))..)))))))))))..)))))))))))..))...
---CUGGCCCUCUCUGCCCUUCC-------- 3
0,-2
---CUGGCCCUCUCUGCCCUUCCG------- 2
0,-1
---CUGGCCCUCUCUGCCCUUCCGU------ 10
0,0
---CUGGCCCUCUCUGCCCUUCCGUu----- 2
0,0

>hsa-mir-329-1 3p:50-71 SRX018958
gguaccugaagagagguuuucuggguuucuguuucuuuaaugaggacgaAACACACCUGGUUAACCUCUUUuccaguauc
(((((..(((((((((((..(..(((...((((((.((......)).)))))).)))..)..)))))))))))..)))))
-----------AGAGGUUUUCUGGGUUUCUGUUU--

3
---AACACACCUGGUUAACCU-------------
2 0,-4
---AACACACCUGGUUAACCUC------------
8 0,-3
---AACACACCUGGUUAACCUCU-----------
6 0,-2
---AACACACCUGGUUAACCUCUau---------
3 0,-2
---AACACACCUGGUUAACCUCUUa---------
2 0,-1
---AACACACCUGGUUAACCUCUUau--------
2 0,-1
---AACACACCUGGUUAACCUCUUU---------
7 0,0
--ACACACCUGGUUAACCUCUUaa--------
2 1,-1

>hsa-mir-329-2 3p:52-73 SRX018958
gugguaccugaagagagguuuucuggguuucuguuucuuuauugaggacgaAACACACCUGGUUAACCUCUUUuccagua
ucaa
.((((((..(((((((((((..(..(((...((((((.((......)).)))))).)))..)..)))))))))))..)))
))).
-------------AGAGGUUUUCUGGGUUUCUGUUU--
---- 3
---AACACACCUGGUUAACCU-----------
---- 2 0,-4
---AACACACCUGGUUAACCUC----------
---- 8 0,-3
---AACACACCUGGUUAACCUCU---------
---- 6 0,-2
---AACACACCUGGUUAACCUCUau-------
---- 3 0,-2
---AACACACCUGGUUAACCUCUUa-------
---- 2 0,-1
---AACACACCUGGUUAACCUCUUau------
---- 2 0,-1
---AACACACCUGGUUAACCUCUUU-------

----   7   0,0
--ACACACCUGGUUAACCUCUUaa------
----   2   1,-1

>hsa-mir-330   5p:18-39;3p:57-79   SRX018958
cuuuggcgaucacugccUCUCUGGGCCUGUGUCUUAGGCucugcaagaucaaccgaGCAAAGCACACGGCCUGCAGAGAg
gcagcgcucugccc
....(((((.(.(((((((((((((((((((.(((..((((.............)))).))))))).))))..)))))))
)))).).)).))).
--GCAAAGCACACGGCCUGCA-----
--------------   3   0,-4
--GCAAAGCACACGGCCUGCAGAGu-
--------------   5   0,-1
--GCAAAGCACACGGCCUGCAGAGA-
--------------   16   0,0
--GCAAAGCACACGGCCUGCAGAGAu
--------------   4   0,0
--GCAAAGCACACGGCCUGCAGAGAG
--------------   2   0,1
--GCAAAGCACACGGCCUGCAGAGAG
u-------------   2   0,1
--GCAAAGCACACGGCCUGCAGAGAG
G-------------   3   0,2
---CAAAGCACACGGCCUGCAGAGA-
--------------   10   1,0
---CAAAGCACACGGCCUGCAGAGAu
--------------   2   1,0
---CAAAGCACACGGCCUGCAGAGAG
--------------   8   1,1
---CAAAGCACACGGCCUGCAGAGAG
G-------------   2   1,2
--AAAGCACACGGCCUGCAGAGA-
--------------   4   2,0
--AAAGCACACGGCCUGCAGAGAa
--------------   3   2,0
--AAAGCACACGGCCUGCAGAGAG
--------------   3   2,1
--AAAGCACACGGCCUGCAGAGAG

a------------- 5 2,1

--AAAGCACACGGCCUGCAGAGAG

G------------- 6 2,2

>hsa-mir-331 5p:26-47;3p:61-81 SRX018958

gaguuugguuuuguuuggguuuguuCUAGGUAUGGUCCCAGGGAUCCcagaucaaaccagGCCCCUGGGCCUAUCCUAGA

Accaaccuaagcuc

............(((((((((.((((((((.((((.(((((((..(.............)..))))))).))))))))))

)).)))))))))..

--GCCCCUGGGCCUAUCCUA--

-------------- 9 0,-3

--GCCCCUGGGCCUAUCCUAG-

-------------- 4 0,-2

--GCCCCUGGGCCUAUCCUAGA

-------------- 16 0,-1

--GCCCCUGGGCCUAUCCUAGA

u------------- 4 0,-1

--GCCCCUGGGCCUAUCCUAGA

A------------- 50 0,0

--GCCCCUGGGCCUAUCCUAGA

Aa------------ 4 0,0

--GCCCCUGGGCCUAUCCUAGA

Aaa----------- 2 0,0

--GCCCCUGGGCCUAUCCUAGA

Au------------ 10 0,0

--GCCCCUGGGCCUAUCCUAGA

Auu----------- 3 0,0

---CCCCUGGGCCUAUCCUAG-

-------------- 2 1,-2

>hsa-mir-335 MA:16-38;mi:52-73 SRX018958

uguuuugagcgggggUCAAGAGCAAUAACGAAAAAUGUuugucauaaaccgUUUUCAUUAUUGCUCCUGACCuccucuc

auuugcuauauuca

.((..((((.((((((((.(((((((((.((((((((.((......)).)))))))).))))))))).)))))))).)))

)...))........

---------------UCAAGAGCAAUAACGAAA---

-------------- 9 0,-5

---------------UCAAGAGCAAUAACGAAAA--

-------------- 17 0,-4
---------------UCAAGAGCAAUAACGAAAAA---
-------------- 42 0,-3
---------------UCAAGAGCAAUAACGAAAAAa--
-------------- 19 0,-3
---------------UCAAGAGCAAUAACGAAAAAaa---
-------------- 4 0,-3
---------------UCAAGAGCAAUAACGAAAAAU--
-------------- 358 0,-2
---------------UCAAGAGCAAUAACGAAAAAUa---
-------------- 184 0,-2
---------------UCAAGAGCAAUAACGAAAAAUaa--
-------------- 2 0,-2
---------------UCAAGAGCAAUAACGAAAAAUu---
-------------- 2 0,-2
---------------UCAAGAGCAAUAACGAAAAAUG---
-------------- 978 0,-1
---------------UCAAGAGCAAUAACGAAAAAUGa--
-------------- 75 0,-1
---------------UCAAGAGCAAUAACGAAAAAUGU--
-------------- 308 0,0
---------------UCAAGAGCAAUAACGAAAAAUGUa---
-------------- 2 0,0
---------------UCAAGAGCAAUAACGAAAAAUGUU---
-------------- 3 0,1
----------------CAAGAGCAAUAACGAAAAAU--
-------------- 2 1,-2
----------------CAAGAGCAAUAACGAAAAAUG---
-------------- 3 1,-1
-----------------AAGAGCAAUAACGAAAAAU--
-------------- 2 2,-2
-----------------AAGAGCAAUAACGAAAAAUG---
-------------- 7 2,-1
-----------------AAGAGCAAUAACGAAAAAUGU--
-------------- 4 2,0
---UUUUUCAUUAUUGCUCCU-----------
-------------- 4 0,-4
---UUUUUCAUUAUUGCUCCUGA---------

------------- 3 0,-2
---UUUUUCAUUAUUGCUCCUGACC-------
------------- 3 0,0

>hsa-mir-337 5p:23-43;3p:61-82 SRX018958
guagucaguaguuggggggugGGAACGGCUUCAUACAGGAGUUgaugcacaguuauccagCUCCUAUAUGAUGCCUUUCU
UCauccccuucaa
..........(..((((((((((((.(((.(((((.(((((((((((......))).)))))))).))))).))).))).
))))))))).)..
----------------------GAACGGCUUCAUACAGGAG---------------------------------------
------------- 2 0,-2
----------------------GAACGGCUUCAUACAGGAGU--------------------------------------
------------- 4 0,-1
----------------------GAACGGCUUCAUACAGGAGUU-------------------------------------
------------- 2 0,0
----------------------GAACGGCUUCAUACAGGAGUUa------------------------------------
------------- 2 0,0
----------------------GAACGGCUUCAUACAGGAGUUaa-----------------------------------
------------- 5 0,0
----------------------GAACGGCUUCAUACAGGAGUUu------------------------------------
------------- 2 0,0
-----------------------AACGGCUUCAUACAGGAGUU-------------------------------------
------------- 2 1,0
--CUCCUAUAUGAUGCCUUUCU
U------------ 3 0,-1
--CUCCUAUAUGAUGCCUUUCU
UC----------- 4 0,0
---UCCUAUAUGAUGCCUUUCU
------------- 2 1,-2
---UCCUAUAUGAUGCCUUUCU
UC----------- 6 1,0

>hsa-mir-338 5p:6-27;3p:42-63 SRX018958
ucuccAACAAUAUCCUGGUGCUGAGUGaugacucaggcgacUCCAGCAUCAGUGAUUUUGUUGaaga
(((.((((((.(((((((((((((((.............))).))))))))).))).)))))).)))
---UCCAGCAUCAGUGAUUUU-------- 7 0,-4
---UCCAGCAUCAGUGAUUUUG------- 15 0,-3
---UCCAGCAUCAGUGAUUUUGU------ 5 0,-2

---UCCAGCAUCAGUGAUUUUGUU----- 8 0,-1
---UCCAGCAUCAGUGAUUUUGUUGA--- 4 0,1

>hsa-mir-339 5p:15-37;3p:50-72 SRX018958
cggggcggccgcucUCCCUGUCCUCCAGGAGCUCACGugugccugccugUGAGCGCCUCGACGACAGAGCCGgcgccugc
cccagugucugcgc
.(((((((.((((..(.(((((.((.(((.(((((((.((....)).))))))).))).)).))))).)..)))).))))
)))...........
--------------UCCCUGUCCUCCAGGAGCU---
-------------- 10 0,-4
--------------UCCCUGUCCUCCAGGAGCUCA---
-------------- 14 0,-2
--------------UCCCUGUCCUCCAGGAGCUCAa--
-------------- 3 0,-2
--------------UCCCUGUCCUCCAGGAGCUCAau---
-------------- 4 0,-2
--------------UCCCUGUCCUCCAGGAGCUCAu--
-------------- 7 0,-2
--------------UCCCUGUCCUCCAGGAGCUCAuu---
-------------- 2 0,-2
--------------UCCCUGUCCUCCAGGAGCUCAC--
-------------- 11 0,-1
--------------UCCCUGUCCUCCAGGAGCUCACG---
-------------- 25 0,0
--------------UCCCUGUCCUCCAGGAGCUCACGa--
-------------- 3 0,0
--------------UCCCUGUCCUCCAGGAGCUCACGaa---
-------------- 2 0,0
--------------UCCCUGUCCUCCAGGAGCUCACGU--
-------------- 11 0,1
----------------CCUGUCCUCCAGGAGCUCAC--
-------------- 4 2,-1
---UGAGCGCCUCGACGACAG-------------
-------------- 10 0,-5
---UGAGCGCCUCGACGACAGA------------
-------------- 7 0,-4
---UGAGCGCCUCGACGACAGAG-----------
-------------- 9 0,-3

---UGAGCGCCUCGACGACAGAGC----------
-------------- 7 0,-2
---UGAGCGCCUCGACGACAGAGCa---------
-------------- 2 0,-2
---UGAGCGCCUCGACGACAGAGCu---------
-------------- 3 0,-2
---UGAGCGCCUCGACGACAGAGCuu--------
-------------- 2 0,-2
---UGAGCGCCUCGACGACAGAGCC---------
-------------- 7 0,-1
---UGAGCGCCUCGACGACAGAGCCa--------
-------------- 9 0,-1
---UGAGCGCCUCGACGACAGAGCCau-------
-------------- 12 0,-1
---UGAGCGCCUCGACGACAGAGCCu--------
-------------- 9 0,-1
---UGAGCGCCUCGACGACAGAGCCuu-------
-------------- 9 0,-1
---UGAGCGCCUCGACGACAGAGCCG--------
-------------- 3 0,0
---UGAGCGCCUCGACGACAGAGCCGu-------
-------------- 3 0,0
--GAGCGCCUCGACGACAGAG-----------
-------------- 2 1,-3
---AGCGCCUCGACGACAGAG-----------
-------------- 17 2,-3
---AGCGCCUCGACGACAGAGCCau-------
-------------- 2 2,-1
---AGCGCCUCGACGACAGAGCCG--------
-------------- 7 2,0
---AGCGCCUCGACGACAGAGCCGu-------
-------------- 3 2,0
---AGCGCCUCGACGACAGAGCCGG-------
-------------- 2 2,1

>hsa-mir-33a MA:6-26;mi:46-67 SRX018958
cugugGUGCAUUGUAGUUGCAUUGCAuguucuggugguacccaugCAAUGUUUCCACAGUGCAUCACag
(((((((((((((.(..((((((((((....((.....)))))))))))..).))))))))))))))

-----GUGCAUUGUAGUUGCAUU--   898 0,-3
-----GUGCAUUGUAGUUGCAUUG---   25 0,-2
-----GUGCAUUGUAGUUGCAUUGa--   11 0,-2
-----GUGCAUUGUAGUUGCAUUGu--   5 0,-2
-----GUGCAUUGUAGUUGCAUUGC--   215 0,-1
-----GUGCAUUGUAGUUGCAUUGCu---   2 0,-1
-----GUGCAUUGUAGUUGCAUUGCA---   474 0,0
-----GUGCAUUGUAGUUGCAUUGCAa--   117 0,0
-----GUGCAUUGUAGUUGCAUUGCAU--   25 0,1
---CAAUGUUUCCACAGUGCA------   2 0,-4
---CAAUGUUUCCACAGUGCAU-----   4 0,-3
---CAAUGUUUCCACAGUGCAUa----   2 0,-3

>hsa-mir-33b MA:16-35;mi:54-75 SRX018958
gcgggcggccccgcgGUGCAUUGCUGUUGCAUUGCacgugugugaggcgggugCAGUGCCUCGGCAGUGCAGCCCggagc
cggccccuggcaccac
((((((((.(((.((((((((((((..(((((((((.(((.....))).)))))))))..)))))))))).))))).))
).))))...)).....
---------------GUGCAUUGCUGUUGCAUU---
----------------   24 0,-2
---------------GUGCAUUGCUGUUGCAUUaa---
----------------   2 0,-2
---------------GUGCAUUGCUGUUGCAUUG--
----------------   14 0,-1
---------------GUGCAUUGCUGUUGCAUUGa---
----------------   9 0,-1
---------------GUGCAUUGCUGUUGCAUUGu---
----------------   2 0,-1
---------------GUGCAUUGCUGUUGCAUUGC---
----------------   6 0,0
---------------GUGCAUUGCUGUUGCAUUGCA--
----------------   5 0,1
---------------GUGCAUUGCUGUUGCAUUGCAa---
----------------   9 0,1

>hsa-mir-340 MA:16-37;mi:58-79 SRX018958
uuguaccugguguga UUAUAAAGCAAUGAGACUGAUUgucauaugucguuugugggaUCCGUCUCAGUUACUUUAUAGCc
auaccugguaucuua

```
..(((((.((((((..(((((((...((((((.((((.(((((.......))))))))).))))))....)))))))..)
))))).)))))....
--------------AUUAUAAAGCAAUGAGAC------------------------------------------------
--------------- 2    -1,-5
--------------AUUAUAAAGCAAUGAGACU-----------------------------------------------
--------------- 4    -1,-4
---------------UUAUAAAGCAAUGAGACU-----------------------------------------------
--------------- 17   0,-4
---------------UUAUAAAGCAAUGAGACUG----------------------------------------------
--------------- 3    0,-3
---------------UUAUAAAGCAAUGAGACUGA---------------------------------------------
--------------- 6    0,-2
---------------UUAUAAAGCAAUGAGACUGAU--------------------------------------------
--------------- 31   0,-1
---------------UUAUAAAGCAAUGAGACUGAUa-------------------------------------------
--------------- 44   0,-1
---------------UUAUAAAGCAAUGAGACUGAUaa------------------------------------------
--------------- 5    0,-1
---------------UUAUAAAGCAAUGAGACUGAUau------------------------------------------
--------------- 3    0,-1
---------------UUAUAAAGCAAUGAGACUGAUU-------------------------------------------
--------------- 185  0,0
---------------UUAUAAAGCAAUGAGACUGAUUa------------------------------------------
--------------- 12   0,0
---------------UUAUAAAGCAAUGAGACUGAUUu------------------------------------------
--------------- 4    0,0
----------------UAUAAAGCAAUGAGACUGAUU-------------------------------------------
--------------- 9    1,0

>hsa-mir-342      5p:19-39;3p:61-83    SRX018958
gaaacugggcucaaggugAGGGGUGCUAUCUGUGAUUGAgggacaugguuaauggaauugUCUCACACAGAAAUCGCACC
CGUcaccuuggccuacuua
.....(((((.((((((((.((((((..((((((.....((((((...((....))..)))))).))))))....)))))
).)))))))))))))....
------------------AGGGGUGCUAUCUGUGAU--------------------------------------------
------------------- 5    0,-3
------------------AGGGGUGCUAUCUGUGAUa-------------------------------------------
------------------- 4    0,-3
```

------------------AGGGGUGCUAUCUGUGAUU---
------------------- 19 0,-2
------------------AGGGGUGCUAUCUGUGAUUG--
------------------- 5   0,-1
------------------AGGGGUGCUAUCUGUGAUUGA---
------------------- 34 0,0
------------------AGGGGUGCUAUCUGUGAUUGAa--
------------------- 6   0,0
------------------AGGGGUGCUAUCUGUGAUUGAG--
------------------- 37 0,1
------------------AGGGGUGCUAUCUGUGAUUGAGa---------------------------------------
------------------- 3   0,1
------------------AGGGGUGCUAUCUGUGAUUGAGG---------------------------------------
------------------- 26 0,2
------------------AGGGGUGCUAUCUGUGAUUGAGGG--------------------------------------
------------------- 2   0,3
------------------AGGGGUGCUAUCUGUGAUUGAGGGA-------------------------------------
------------------- 3   0,4
-------------------GGGGUGCUAUCUGUGAUUGAGGGAC------------------------------------
------------------- 4   1,5
--UCUCACACAGAAAUCGCA--
------------------- 78 0,-5
--UCUCACACAGAAAUCGCAC-
------------------- 162 0,-4
--UCUCACACAGAAAUCGCACC
------------------- 157 0,-3
--UCUCACACAGAAAUCGCACC
C------------------ 205 0,-2
--UCUCACACAGAAAUCGCACC
Ca----------------- 2   0,-2
--UCUCACACAGAAAUCGCACC
Cuu---------------- 2   0,-2
--UCUCACACAGAAAUCGCACC
CG----------------- 270 0,-1
--UCUCACACAGAAAUCGCACC
CGa---------------- 23 0,-1
--UCUCACACAGAAAUCGCACC
CGU---------------- 952 0,0

--UCUCACACAGAAAUCGCACC
CGUa--------------- 11 0,0
--UCUCACACAGAAAUCGCACC
CGUaa-------------- 3   0,0
--UCUCACACAGAAAUCGCACC
CGUau-------------- 3   0,0
--UCUCACACAGAAAUCGCACC
CGUu--------------- 40 0,0
--UCUCACACAGAAAUCGCACC
CGUuu-------------- 7   0,0
--UCUCACACAGAAAUCGCACC
CGUC--------------- 331 0,1
--UCUCACACAGAAAUCGCACC
CGUCu-------------- 169 0,1
--UCUCACACAGAAAUCGCACC
CGUCA-------------- 61 0,2
---CUCACACAGAAAUCGCACC
CGU---------------- 2   1,0
--UCACACAGAAAUCGCACC
------------------- 4   2,-3
--UCACACAGAAAUCGCACC
C------------------ 7   2,-2
--UCACACAGAAAUCGCACC
CG----------------- 9   2,-1
--UCACACAGAAAUCGCACC
CGU---------------- 18 2,0
--UCACACAGAAAUCGCACC
CGUC--------------- 12 2,1
--UCACACAGAAAUCGCACC
CGUCu-------------- 6   2,1
--UCACACAGAAAUCGCACC
CGUCA-------------- 76 2,2
--UCACACAGAAAUCGCACC
CGUCAa------------- 3   2,2
--UCACACAGAAAUCGCACC
CGUCAu------------- 4   2,2

>hsa-mir-345   5p:18-39   SRX018958

acccaaacccuaggucuGCUGACUCCUAGUCCAGGGCUCgugauggcugguggggcccugaacgaggggucuggaggccug
gguuugaauaucgacagc
...(((((((.((((((.(.(((((((.((.(((((((((..(....)..))))))))).)).))))))).).)))))))
)))))).............
-----------------GCUGACUCCUAGUCCAGG--
------------------ 83 0,-4
-----------------GCUGACUCCUAGUCCAGGG---
------------------ 45 0,-3
-----------------GCUGACUCCUAGUCCAGGGC--
------------------ 39 0,-2
-----------------GCUGACUCCUAGUCCAGGGCaa--
------------------ 6 0,-2
-----------------GCUGACUCCUAGUCCAGGGCau--
------------------ 5 0,-2
-----------------GCUGACUCCUAGUCCAGGGCU---
------------------ 78 0,-1
-----------------GCUGACUCCUAGUCCAGGGCUa--
------------------ 9 0,-1
-----------------GCUGACUCCUAGUCCAGGGCUaa---
------------------ 9 0,-1
-----------------GCUGACUCCUAGUCCAGGGCUau---
------------------ 36 0,-1
-----------------GCUGACUCCUAGUCCAGGGCUu--
------------------ 10 0,-1
-----------------GCUGACUCCUAGUCCAGGGCUuu---
------------------ 19 0,-1
-----------------GCUGACUCCUAGUCCAGGGCUC--
------------------ 81 0,0
-----------------GCUGACUCCUAGUCCAGGGCUCa---
------------------ 22 0,0
-----------------GCUGACUCCUAGUCCAGGGCUCaa--
------------------ 13 0,0
-----------------GCUGACUCCUAGUCCAGGGCUCau--
------------------ 56 0,0
-----------------GCUGACUCCUAGUCCAGGGCUCu---
------------------ 28 0,0
-----------------GCUGACUCCUAGUCCAGGGCUCuu--
------------------ 13 0,0

-----------------GCUGACUCCUAGUCCAGGGCUCGa---------------------------------------
------------------ 4 0,1

>hsa-mir-34a MA:22-43;mi:64-85 SRX018958
ggccagcugugaguguuucuuUGGCAGUGUCUUAGCUGGUUGUugugagcaauaguaaggaagCAAUCAGCAAGUAUACU
GCCCUagaagugcugcacguuguggggccc
((((.((((((((..(((((..(((((((((((.((((((((((....((....)).....))))))))))))).)))))
)))..)))))..)).))))..))..)))).
---------------------UGGCAGUGUCUUAGCUGGUUGU-------------------------------------
------------------------------ 3 0,0
---------------------UGGCAGUGUCUUAGCUGGUUGUU------------------------------------
------------------------------ 2 0,1
---------------------UGGCAGUGUCUUAGCUGGUUGUUu-----------------------------------
------------------------------ 2 0,1
---CAAUCAGCAAGUAUACU
GC---------------------------- 2 0,-3
---CAAUCAGCAAGUAUACU
GCCCU------------------------- 2 0,0
---CAAUCAGCAAGUAUACU
GCCCUA------------------------ 5 0,1
---CAAUCAGCAAGUAUACU
GCCCUAu----------------------- 2 0,1
--AAUCAGCAAGUAUACU
GCC--------------------------- 6 1,-2
--AAUCAGCAAGUAUACU
GCCCU------------------------- 4 1,0
--AAUCAGCAAGUAUACU
GCCCUA------------------------ 23 1,1

>hsa-mir-34c 5p:13-35;3p:46-67 SRX018958
agucuaguuacuAGGCAGUGUAGUUAGCUGAUUGCuaauaguaccAAUCACUAACCACACGGCCAGGuaaaaagauu
(((((..(((((.(((.((((.(((((.((((((.((....)).))))))))))).)))).))).)))))..)))))
------------AGGCAGUGUAGUUAGCUGAUUGu--
 2 0,-1
------------AGGCAGUGUAGUUAGCUGAUUGC--
 2 0,0

>hsa-mir-3605 5p:22-44;3p:66-88 SRX018958

acuuuauacguguaauugugaUGAGGAUGGAUAGCAAGGAAGCCgcucccaccugacccucacggCCUCCGUGUUACCUG
UCCUCUAGgugggacgcucg
.........((((.........((((((((.(((((.(((.((((.................)))).))).))))).)))
)))))........))))...
-------------------GAUGAGGAUGGAUAGCAAGG---
--------------------   7   -2,-5
-------------------GAUGAGGAUGGAUAGCAAGGA--
--------------------   8   -2,-4
-------------------GAUGAGGAUGGAUAGCAAGGAA---------------------------------------
--------------------   7   -2,-3
-------------------GAUGAGGAUGGAUAGCAAGGAAG--------------------------------------
--------------------   10   -2,-2
-------------------GAUGAGGAUGGAUAGCAAGGAAGC-------------------------------------
--------------------   5   -2,-1
--------------------AUGAGGAUGGAUAGCAAGGAA---------------------------------------
--------------------   3   -1,-3
--------------------AUGAGGAUGGAUAGCAAGGAAG--------------------------------------
--------------------   2   -1,-2
--------------------AUGAGGAUGGAUAGCAAGGAAGC-------------------------------------
--------------------   2   -1,-1
--------------------AUGAGGAUGGAUAGCAAGGAAGCC------------------------------------
--------------------   3   -1,0
--------------------AUGAGGAUGGAUAGCAAGGAAGCCu-----------------------------------
--------------------   2   -1,0
---------------------UGAGGAUGGAUAGCAAGG---
--------------------   2   0,-5
---------------------UGAGGAUGGAUAGCAAGGAAG--------------------------------------
--------------------   5   0,-2
---------------------UGAGGAUGGAUAGCAAGGAAGC-------------------------------------
--------------------   3   0,-1
---------------------UGAGGAUGGAUAGCAAGGAAGCC------------------------------------
--------------------   4   0,0
---------------------UGAGGAUGGAUAGCAAGGAAGCCuu----------------------------------
--------------------   2   0,0

>hsa-mir-3609   3p:51-74   SRX018958
guaacaguaacuuuuauucucauuuuccuuuucucuaccuuguagagaagCAAAGUGAUGAGUAAUACUGGCUGgagccc
((..((((.....(((((((((((((...(((((((((...))))))))).))))))).)))))).....))))..))..

---GCAAAGUGAUGAGUAAUACU-----------
3 -1,-5
--CAAAGUGAUGAGUAAUACU-----------
7 0,-5

>hsa-mir-361 5p:6-27;3p:45-67 SRX018958
ggagcUUAUCAGAAUCUCCAGGGGUACuuuauaauuucaaaaagUCCCCCAGGUGUGAUUCUGAUUUgcuuc
(((((..(((((((((.((.((((.(((((..........))))).)))).))...)))))))))..)))))
----CUUAUCAGAAUCUCCAGGGGUAu--- 2
-1,-1
-----UUAUCAGAAUCUCCAGGG--- 6
0,-4
-----UUAUCAGAAUCUCCAGGGG-- 7
0,-3
-----UUAUCAGAAUCUCCAGGGGU--- 21
0,-2
-----UUAUCAGAAUCUCCAGGGGUA-- 39
0,-1
-----UUAUCAGAAUCUCCAGGGGUAa--- 23
0,-1
-----UUAUCAGAAUCUCCAGGGGUAaa-- 5
0,-1
-----UUAUCAGAAUCUCCAGGGGUAu--- 47
0,-1
-----UUAUCAGAAUCUCCAGGGGUAuu-- 8
0,-1
-----UUAUCAGAAUCUCCAGGGGUAC--- 71
0,0
-----UUAUCAGAAUCUCCAGGGGUACa-- 43
0,0
-----UUAUCAGAAUCUCCAGGGGUACaa--- 4
0,0
-----UUAUCAGAAUCUCCAGGGGUACau--- 5
0,0
-----UUAUCAGAAUCUCCAGGGGUACU-- 9
0,1
-------AUCAGAAUCUCCAGGGGUACUUU-- 2
2,3

--UCCCCCAGGUGUGAUUCU---------- 11 0,-5
--UCCCCCAGGUGUGAUUCUGA-------- 5 0,-3
--UCCCCCAGGUGUGAUUCUGAa------- 4 0,-3
--UCCCCCAGGUGUGAUUCUGAU------- 8 0,-2
--UCCCCCAGGUGUGAUUCUGAUa------ 5 0,-2
--UCCCCCAGGUGUGAUUCUGAUaa----- 2 0,-2
--UCCCCCAGGUGUGAUUCUGAUU------ 19 0,-1
--UCCCCCAGGUGUGAUUCUGAUUa----- 2 0,-1
--UCCCCCAGGUGUGAUUCUGAUUU----- 64 0,0
--UCCCCCAGGUGUGAUUCUGAUUUu---- 4 0,0
--UCCCCCAGGUGUGAUUCUGAUUUG---- 5 0,1
--UCCCCCAGGUGUGAUUCUGAUUUGu--- 2 0,1
--UCCCCCAGGUGUGAUUCUGAUUUGC--- 3 0,2
---CCCCCAGGUGUGAUUCUGAUUUG---- 4 1,1
--CCCCAGGUGUGAUUCUGA-------- 2 2,-3

>hsa-mir-3613 5p:16-37;3p:53-76 SRX018958
ugguuggguuuggauUGUUGUACUUUUUUUUUUGUUCguugcauuuuuaggaACAAAAAAAAAAGCCCAACCCUUCacaccacuuca
.((...(((.((((..((((..(((((((((((((((.(.........).)))))))))))))))..))))..)))).)))...)).
---------------UGUUGUACUUUUUUUUUUGUU--- 2 0,-1

---------------UGUUGUACUUUUUUUUUGUUC---
------- 3   0,0
---ACAAAAAAAAAAGCCCAAC---------
------- 8   0,-5
---ACAAAAAAAAAAGCCCAACC--------
------- 4   0,-4
---ACAAAAAAAAAAGCCCAACCC-------
------- 2   0,-3

>hsa-mir-3614   5p:15-37;3p:53-75   SRX018958
gguucugucuugggCCACUUGGAUCUGAAGGCUGCCCcuuugcucucuggggUAGCCUUCAGAUCUUGGUGUUUUgaauu
cuuacu
.........(..(..((((.(((((((((((((((((..........))))))))))))))))).))))..)..)...
......
--UAGCCUUCAGAUCUUGGUG---------
------   2   0,-4
--UAGCCUUCAGAUCUUGGUGU--------
------   2   0,-3
--UAGCCUUCAGAUCUUGGUGUU-------
------   4   0,-2
--UAGCCUUCAGAUCUUGGUGUUa------
------   4   0,-2
--UAGCCUUCAGAUCUUGGUGUUU------
------   22   0,-1
--UAGCCUUCAGAUCUUGGUGUUUU-----
------   3   0,0

>hsa-mir-362   5p:5-28;3p:42-63   SRX018958
cuugAAUCCUUGGAACCUAGGUGUGAGUgcuauuucagugcAACACACCUAUUCAAGGAUUCAaa
.((((((((((((...((((((((.(..((.....))..)..))))))))))))))))))))).
----AAUCCUUGGAACCUAGGUGU---   4   0,-4
----AAUCCUUGGAACCUAGGUGUG--   3   0,-3
----AAUCCUUGGAACCUAGGUGUGA---------------------------------------   11   0,-2
----AAUCCUUGGAACCUAGGUGUGAG--------------------------------------   9   0,-1
----AAUCCUUGGAACCUAGGUGUGAGU-------------------------------------   8   0,0
---AACACACCUAUUCAAGGAU-----   3   0,-3
---AACACACCUAUUCAAGGAUU----   6   0,-2
---AACACACCUAUUCAAGGAUUa---   2   0,-2

---AACACACCUAUUCAAGGAUUC--- 16 0,-1
---AACACACCUAUUCAAGGAUUCA-- 22 0,0
---AACACACCUAUUCAAGGAUUCAA- 2 0,1

>hsa-mir-363 mi:7-28;MA:50-71 SRX018958
uguuguCGGGUGGAUCACGAUGCAAUUUugaugaguaucauaggagaaaAAUUGCACGGUAUCCAUCUGUAaacc
.(((..(((((((((..((.(((((((((.(((.....)))......)))))))))))..)))))))))..))).
---AAUUGCACGGUAUCCAUC-------- 8
0,-4
---AAUUGCACGGUAUCCAUCU------- 50
0,-3
---AAUUGCACGGUAUCCAUCUG------ 6
0,-2
---AAUUGCACGGUAUCCAUCUGU----- 11
0,-1
---AAUUGCACGGUAUCCAUCUGUA---- 9
0,0
---AAUUGCACGGUAUCCAUCUGUAu--- 4
0,0
---AAUUGCACGGUAUCCAUCUGUAA--- 11
0,1
--AUUGCACGGUAUCCAUCU------- 9
1,-3

>hsa-mir-3647 5p:15-37;3p:62-84 SRX018958
uugcuuucaucagcCUGAAGUGAUGAUUCACAUUCAUgucucuucucugauaaauucuugaAGAAAAUUUUUGUGUGUCU
GAUCaggccucuagagggcag
.(((((((....((((((......(((.((((........(((((...((.....))..))))).......)))).))).
..))))))).....))))))).
---GAAGAAAAUUUUUGUGUGUCU
--------------------- 3 -2,-4
---AGAAAAUUUUUGUGUGUC-
--------------------- 2 0,-5
---AGAAAAUUUUUGUGUGUCU
--------------------- 2 0,-4

>hsa-mir-365-1 3p:56-77 SRX018958
accgcagggaaaaugagggacuuuuggggcagauguguuuccauuccacuaucaUAAUGCCCCUAAAAAUCCUUAUugc

ucuugca
...((((((..((((((((..(((((((((((((((((.........))).)))....))))))))))).))))))))..
)))))).
---------------AGGGACUUUUGGGGGCAG---
------- 2
---------------AGGGACUUUUGGGGGCAGA--
------- 3
---------------AGGGACUUUUGGGGGCAGAU---
------- 6
---------------AGGGACUUUUGGGGGCAGAUG--
------- 3
---------------AGGGACUUUUGGGGGCAGAUGU---
------- 28
---------------AGGGACUUUUGGGGGCAGAUGUa--
------- 4
---------------AGGGACUUUUGGGGGCAGAUGUau---
------- 4
---------------AGGGACUUUUGGGGGCAGAUGUu--
------- 6
---------------AGGGACUUUUGGGGGCAGAUGUuu---
------- 2
---------------AGGGACUUUUGGGGGCAGAUGUG--
------- 97
---------------AGGGACUUUUGGGGGCAGAUGUGa---
------- 5
---------------AGGGACUUUUGGGGGCAGAUGUGau--
------- 2
---------------AGGGACUUUUGGGGGCAGAUGUGU---
------- 73
---------------AGGGACUUUUGGGGGCAGAUGUGUU--
------- 7
----------------GGGACUUUUGGGGGCAGAUGUGU---
------- 9
---UAAUGCCCCUAAAAAUCC-------
------- 10 0,-4
---UAAUGCCCCUAAAAAUCCU------
------- 4 0,-3
---UAAUGCCCCUAAAAAUCCUU-----

------- 22 0,-2
---UAAUGCCCCUAAAAAUCCUUu----
------- 6   0,-2
---UAAUGCCCCUAAAAAUCCUUuu---
------- 4   0,-2
---UAAUGCCCCUAAAAAUCCUUA----
------- 60 0,-1
---UAAUGCCCCUAAAAAUCCUUAa---
------- 33 0,-1
---UAAUGCCCCUAAAAAUCCUUAU---
------- 316 0,0
---UAAUGCCCCUAAAAAUCCUUAUa--
------- 20 0,0
---UAAUGCCCCUAAAAAUCCUUAUU--
------- 22 0,1
---UAAUGCCCCUAAAAAUCCUUAUUu-
------- 2   0,1
---AUGCCCCUAAAAAUCCUUA----
------- 2   2,-1
---AUGCCCCUAAAAAUCCUUAU---
------- 3   2,0

>hsa-mir-365-2 mi:29-50;MA:68-89   SRX018958
agaguguucaaggacagcaagaaaaaugAGGGACUUUCAGGGGCAGCUGUguuuucugacucagucaUAAUGCCCCUAAA
AAUCCUUAUuguucuugcagugugcaucggg
...........(.((((((((((.((((((((..(((.(((((((((((.(((....))).)))).....))))))))))
..)))))))).)))))))..))).)......
---------------------------GAGGGACUUUCAGGGGCAGC---------------------------------
------------------------------- 2   -1,-3
---------------------------GAGGGACUUUCAGGGGCAGCU--------------------------------
------------------------------- 16 -1,-2
---------------------------GAGGGACUUUCAGGGGCAGCUG-------------------------------
------------------------------- 3   -1,-1
---------------------------GAGGGACUUUCAGGGGCAGCUGU------------------------------
------------------------------- 3   -1,0
---------------------------GAGGGACUUUCAGGGGCAGCUGUuu----------------------------
------------------------------- 2   -1,0
----------------------------AGGGACUUUCAGGGGCAGC---------------------------------

------------------------------ 4   0,-3
---------------------------AGGGACUUUCAGGGGCAGCU---------------------------------
------------------------------ 10 0,-2
---------------------------AGGGACUUUCAGGGGCAGCUG--------------------------------
------------------------------ 4   0,-1
---------------------------AGGGACUUUCAGGGGCAGCUGUa------------------------------
------------------------------ 2   0,0
---------------------------AGGGACUUUCAGGGGCAGCUGUau-----------------------------
------------------------------ 6   0,0
---------------------------AGGGACUUUCAGGGGCAGCUGUG------------------------------
------------------------------ 43 0,1
---------------------------AGGGACUUUCAGGGGCAGCUGUGU-----------------------------
------------------------------ 11 0,2
---UAAUGCCCCUAAA
AAUCC------------------------- 10 0,-4
---UAAUGCCCCUAAA
AAUCCU------------------------ 4   0,-3
---UAAUGCCCCUAAA
AAUCCUU----------------------- 22 0,-2
---UAAUGCCCCUAAA
AAUCCUUu---------------------- 6   0,-2
---UAAUGCCCCUAAA
AAUCCUUuu--------------------- 4   0,-2
---UAAUGCCCCUAAA
AAUCCUUA---------------------- 60 0,-1
---UAAUGCCCCUAAA
AAUCCUUAa--------------------- 33 0,-1
---UAAUGCCCCUAAA
AAUCCUUAU--------------------- 316 0,0
---UAAUGCCCCUAAA
AAUCCUUAUa-------------------- 20 0,0
---UAAUGCCCCUAAA
AAUCCUUAUU-------------------- 22 0,1
---UAAUGCCCCUAAA
AAUCCUUAUUu------------------- 2   0,1
---AUGCCCCUAAA
AAUCCUUA---------------------- 2   2,-1
---AUGCCCCUAAA

AAUCCUUAU--------------------- 3 2,0

>hsa-mir-3656 3p:49-65 SRX018958
cuuucggccagcgggacggcauccgaggugggcuaggcucgggcccguGGCGGGUGCGGGGGUGGgagg
(((((.(((.......(.(((((((..(((((((.......)))))))..))))))).).))).)))))
--GGCGGGUGCGGGGGUGGG--- 2 0,1

>hsa-mir-3664 5p:21-42 SRX018958
cuguaaacuugaagguagggAACUCUGUCUUCACUCAUGAGUaccuuccaacacgagcucucaggaguaaagacagaguu
cccuaccuucaauguggau
......(((((((((((((((((((((((((.((((.((((...(((.......)))..)))).)))).)))))))))))
))))))))))))).))....
--UCUCAGGAGUAAAGACAG----
------------------- 10
--UCUCAGGAGUAAAGACAGA---
------------------- 13
--UCUCAGGAGUAAAGACAGAG--
------------------- 18
--UCUCAGGAGUAAAGACAGAGUU
------------------- 20
--UCUCAGGAGUAAAGACAGAGUU
a------------------ 3
--UCUCAGGAGUAAAGACAGAGUU
u------------------ 2
--UCAGGAGUAAAGACAGAGUU
------------------- 6
---CAGGAGUAAAGACAGAGUU
------------------- 6
---CAGGAGUAAAGACAGAGUU
CCC---------------- 3
---AGGAGUAAAGACAGAGUU
CCCU--------------- 4

>hsa-mir-3676 3p:60-79 SRX018958
uugguuaaagcgccugucuaguaaacaggagauccuggguucgaaucccagcggugccuCCGUGUUUCCCCCACGCUUUu
gccaa
.((((.((((((..((....(.(((((((((((((((((.......))))).)))..)))).))))).)..)))))))).
)))).

----------------------AAACAGGAGAUCCUGGGUU---------------------------------------

----- 2

----------------------AAACAGGAGAUCCUGGGUUC--------------------------------------

----- 2

>hsa-mir-369 5p:9-30;3p:44-64 SRX018958

uugaagggAGAUCGACCGUGUUAUAUUCGCuuuauugacuucgAAUAAUACAUGGUUGAUCUUUucucag

.(((..(((((((((((((((..((((((............))))))..)))))))))))))))..))).

--------AGAUCGACCGUGUUAUAUa--- 2 0,-4

--------AGAUCGACCGUGUUAUAUU--- 29 0,-3

--------AGAUCGACCGUGUUAUAUUC-- 37 0,-2

--------AGAUCGACCGUGUUAUAUUCG--- 12 0,-1

--------AGAUCGACCGUGUUAUAUUCGa-- 2 0,-1

--------AGAUCGACCGUGUUAUAUUCGCa--------------------------------------- 3 0,0

--------AGAUCGACCGUGUUAUAUUCGCU--------------------------------------- 10 0,1

--------AGAUCGACCGUGUUAUAUUCGCUU-------------------------------------- 2 0,2

--GAAUAAUACAUGGUUGAUCUUU------ 2 -1,0

---AAUAAUACAUGGUUGAUCUU------- 3 0,-1

---AAUAAUACAUGGUUGAUCUUa------ 2 0,-1

---AAUAAUACAUGGUUGAUCUUU------ 78 0,0

---AAUAAUACAUGGUUGAUCUUUa----- 3 0,0

---AAUAAUACAUGGUUGAUCUUUU----- 7 0,1

>hsa-mir-3690 5p:10-32 SRX018958

cccaucuccACCUGGACCCAGCGUAGACAAAGagguguuucuacuccauaucuaccuggacccaguguagauggg

(((((((.(((.(((..((((.(((((....(((.........)))....)))))))))..)))))).)))))))

---------ACCUGGACCCAGCGUAGA-- 2

0,-5

---------ACCUGGACCCAGCGUAGACA-- 2

0,-3

---------ACCUGGACCCAGCGUAGACAA--- 4

0,-2

---------ACCUGGACCCAGCGUAGACAAAG--- 5

0,0

---------ACCUGGACCCAGCGUAGACAAAGA-- 2

0,1

>hsa-mir-3692 mi:6-29;MA:44-67 SRX018958

ccauuCCUGCUGGUCAGGAGUGGAUACUGgagcaauagauacaGUUCCACACUGACACUGCAGAAGUgg
(((((.((((..(((((..(((((.((((...(....)...))))))))).)))))...)))).)))))
---GUUCCACACUGACACUGCAGAAG--- 2 0,-1

>hsa-mir-370 3p:48-69 SRX018958
agacagagaagccaggucacgucucugcaguuacacagcucacgaguGCCUGCUGGGGUGGAACCUGGUcugucu
((((((....(((((((..(..(((.((((.(((.(.......).))).)))).)))..)..)))))))))))))
---GCCUGCUGGGGUGGAACCUGG------- 3
0,-1
---GCCUGCUGGGGUGGAACCUGGUaa---- 3
0,0

>hsa-mir-371 5p:6-25;3p:42-64 SRX018958
guggcACUCAAACUGUGGGGGCACUuucugcucucuggugaAAGUGCCGCCAUCUUUUGAGUGUuac
((((((((((((..((((.(((((((((.(((....)))))))))))).))))..))))))))))))
-----ACUCAAACUGUGGGGGCACU-- 2 0,0
-----ACUCAAACUGUGGGGGCACUUau-- 2 0,1

>hsa-mir-372 3p:42-64 SRX018958
gugggccucaaauguggagcacuauucugauguccaaguggAAAGUGCUGCGACAUUUGAGCGUcac
...((((((((((((.(((((((.((((...........))))))))))).).)))))))).)))..
---AAAGUGCUGCGACAUUUGAG------ 11 0,-3

>hsa-mir-374a MA:12-33;mi:42-63 SRX018958
uacaucggccaUUAUAAUACAACCUGAUAAGUGuuauagcaCUUAUCAGAUUGUAUUGUAAUUgucugugua
(((((.(((.((((((((((((.(((((((((((....))))))))))).)))))))))))).))).)))))
-----------UUAUAAUACAACCUGAUA--- 31
0,-4
-----------UUAUAAUACAACCUGAUAA-- 257
0,-3
-----------UUAUAAUACAACCUGAUAAa--- 10
0,-3
-----------UUAUAAUACAACCUGAUAAaa-- 5
0,-3
-----------UUAUAAUACAACCUGAUAAuu-- 3
0,-3
-----------UUAUAAUACAACCUGAUAAG--- 71
0,-2

-----------UUAUAAUACAACCUGAUAAGa--------------------------------------- 8
0,-2
-----------UUAUAAUACAACCUGAUAAGU--------------------------------------- 705
0,-1
-----------UUAUAAUACAACCUGAUAAGUa-------------------------------------- 37
0,-1
-----------UUAUAAUACAACCUGAUAAGUaa------------------------------------- 22
0,-1
-----------UUAUAAUACAACCUGAUAAGUG-------------------------------------- 823
0,0
-----------UUAUAAUACAACCUGAUAAGUGa------------------------------------- 355
0,0
-----------UUAUAAUACAACCUGAUAAGUGaa------------------------------------ 7
0,0
-----------UUAUAAUACAACCUGAUAAGUGU------------------------------------- 62
0,1
------------UAUAAUACAACCUGAUAA--- 2
1,-3
------------UAUAAUACAACCUGAUAAGU--------------------------------------- 7
1,-1
------------UAUAAUACAACCUGAUAAGUG-------------------------------------- 2
1,0
------------UAUAAUACAACCUGAUAAGUGa------------------------------------- 3
1,0
---------------------------------------ACUUAUCAGAUUGUAUUGUAAU---------- 11
-1,-1
--CUUAUCAGAUUGUAUUGU------------- 17
0,-4
--CUUAUCAGAUUGUAUUGUA------------ 2
0,-3
--CUUAUCAGAUUGUAUUGUAuu---------- 2
0,-3
--CUUAUCAGAUUGUAUUGUAA----------- 18
0,-2
--CUUAUCAGAUUGUAUUGUAAa---------- 12
0,-2
--CUUAUCAGAUUGUAUUGUAAaa--------- 7
0,-2

---CUUAUCAGAUUGUAUUGUAAU---------- 529
0,-1
---CUUAUCAGAUUGUAUUGUAAUa--------- 231
0,-1
---CUUAUCAGAUUGUAUUGUAAUaa-------- 4
0,-1
---CUUAUCAGAUUGUAUUGUAAUU--------- 1229
0,0
---CUUAUCAGAUUGUAUUGUAAUUa-------- 131
0,0
---CUUAUCAGAUUGUAUUGUAAUUu-------- 25
0,0
---CUUAUCAGAUUGUAUUGUAAUUG-------- 2
0,1
--UUAUCAGAUUGUAUUGUAAU---------- 4
1,-1
--UUAUCAGAUUGUAUUGUAAUa--------- 2
1,-1
--UUAUCAGAUUGUAUUGUAAUU--------- 13
1,0
---UAUCAGAUUGUAUUGUAAU---------- 2
2,-1
---UAUCAGAUUGUAUUGUAAUU--------- 2
2,0
---UAUCAGAUUGUAUUGUAAUUa-------- 5
2,0
---UAUCAGAUUGUAUUGUAAUUu-------- 4
2,0
---UAUCAGAUUGUAUUGUAAUUGU------- 2
2,2

>hsa-mir-374b MA:11-32;mi:41-62 SRX018958
acucggauggAUAUAAUACAACCUGCUAAGUGuccuagcaCUUAGCAGGUUGUAUUAUCAUUguccgugucu
((.((((..(..(((((((((((((((((((((....)))))))))))))))))))))..)..)))).))..
----------AUAUAAUACAACCUGCUAA--- 10
0,-3
----------AUAUAAUACAACCUGCUAAG-- 38
0,-2

----------AUAUAAUACAACCUGCUAAGa--- 2
0,-2
----------AUAUAAUACAACCUGCUAAGU--- 389
0,-1
----------AUAUAAUACAACCUGCUAAGUa-- 15
0,-1
----------AUAUAAUACAACCUGCUAAGUaa--------------------------------------- 5
0,-1
----------AUAUAAUACAACCUGCUAAGUu-- 3
0,-1
----------AUAUAAUACAACCUGCUAAGUG-- 729
0,0
----------AUAUAAUACAACCUGCUAAGUGa--------------------------------------- 196
0,0
----------AUAUAAUACAACCUGCUAAGUGaa-------------------------------------- 4
0,0
----------AUAUAAUACAACCUGCUAAGUGU--------------------------------------- 64
0,1
-----------UAUAAUACAACCUGCUAAGUGaa-------------------------------------- 2
1,0
------------AUAAUACAACCUGCUAAGU--- 2
2,-1
------------AUAAUACAACCUGCUAAGUG-- 5
2,0
--CUUAGCAGGUUGUAUUAUCA------------ 3
0,-2
--CUUAGCAGGUUGUAUUAUCAU----------- 2
0,-1
--CUUAGCAGGUUGUAUUAUCAUa---------- 2
0,-1
--CUUAGCAGGUUGUAUUAUCAUU---------- 8
0,0
--CUUAGCAGGUUGUAUUAUCAUUu--------- 2
0,0

>hsa-mir-374c 5p:13-34 SRX018958
acacggacaaugAUAAUACAACCUGCUAAGUGCUaggacacuuagcagguuguauuauauccauccgagu
...((((.....((((((((((((((((((((......)))))))))))))))))))).....))))...

------------AUAAUACAACCUGCUAAGU--------------------------------------- 2 0,-3
------------AUAAUACAACCUGCUAAGUG-------------------------------------- 5 0,-2
--CUUAGCAGGUUGUAUUAUA----------- 2

>hsa-mir-375 3p:40-61 SRX018958
ccccgcgacgagccccucgcacaaaccggaccugagcguUUUGUUCGUUCGGCUCGCGUGAggc
.((((((.((((((...((.(((((.((........)).))))).))...)))))))))).)).
--------------------------------------UUUUGUUCGUUCGGCUCGCGU----- 2 -1,-2
--------------------------------------UUUUGUUCGUUCGGCUCGCGUG---- 3 -1,-1
--------------------------------------UUUUGUUCGUUCGGCUCGCGUGA--- 2 -1,0
---------------------------------------UUUGUUCGUUCGGCUCGCG------ 4 0,-3
---------------------------------------UUUGUUCGUUCGGCUCGCGU----- 27 0,-2
---------------------------------------UUUGUUCGUUCGGCUCGCGUG---- 111 0,-1
---------------------------------------UUUGUUCGUUCGGCUCGCGUGu--- 11 0,-1
---------------------------------------UUUGUUCGUUCGGCUCGCGUGA--- 126 0,0
---------------------------------------UUUGUUCGUUCGGCUCGCGUGAa-- 5 0,0
---------------------------------------UUUGUUCGUUCGGCUCGCGUGAaa- 5 0,0
---------------------------------------UUUGUUCGUUCGGCUCGCGUGAau- 2 0,0
---------------------------------------UUUGUUCGUUCGGCUCGCGUGAu-- 11 0,0
---------------------------------------UUUGUUCGUUCGGCUCGCGUGAuu- 4 0,0
--UUGUUCGUUCGGCUCGCGUGA--- 4 1,0

>hsa-mir-376a-1 mi:7-28;MA:44-64 SRX018958
uaaaagGUAGAUUCUCCUUCUAUGAGUAcauuauuuaugauuaAUCAUAGAGGAAAAUCCACGUuuuc
.((((.((.((((.((((.((((((...(((.....))).....)))))))))).)))).)).)))).
-------UAGAUUCUCCUUCUAUGAGUAC--------------------------------------- 2 1,1
---AUCAUAGAGGAAAAUCCA------- 2 0,-3
---AUCAUAGAGGAAAAUCCACGU---- 15 0,0

>hsa-mir-376a-2 3p:50-70 SRX018958
gguauuuaaaagguagauuuuccuucuaugguuacguguuugaugguuaAUCAUAGAGGAAAAUCCACGUuuucaguauc
((((((.((((.((.(((((((((.((((((((((((.....)))..)))))))))))))))))).)).)))).))))))
---AUCAUAGAGGAAAAUCCA-------------
2 0,-3
---AUCAUAGAGGAAAAUCCACGU----------
15 0,0

>hsa-mir-376b 3p:62-83 SRX018958

caguccuucuuugguauuuuaaaacguggauauuccuucuauguuuacgugauuccugguuaAUCAUAGAGGAAAAUCCAU
GUUuucaguaucaaaugcug
((((.....(((((((((.(((((((((((.(((((.(((((......((((.....))))..)))))))))).))))))
))))).)))))))))).))))
--AUCAUAGAGGAAAAUCCA-
--------------------   2   0,-4
--AUCAUAGAGGAAAAUCCAU
--------------------   43   0,-3
--AUCAUAGAGGAAAAUCCAU
G-------------------   2   0,-2
--AUCAUAGAGGAAAAUCCAU
GU------------------   2   0,-1
--AUCAUAGAGGAAAAUCCAU
GUU-----------------   3   0,0

>hsa-mir-376c   3p:43-63   SRX018958
aaaagguggauauuccuucuauguuuauguuauuuaugguuaAACAUAGAGGAAAUUCCACGUuuu
((((.(((((..(((((.(((((((((.................))))))))))))))..))))).))))
--AACAUAGAGGAAAUUCCA------   5   0,-3
--AACAUAGAGGAAAUUCCAC-----   52   0,-2
--AACAUAGAGGAAAUUCCACG----   60   0,-1
--AACAUAGAGGAAAUUCCACGU---   292   0,0
--AACAUAGAGGAAAUUCCACGUa--   11   0,0
--AACAUAGAGGAAAUUCCACGUU--   27   0,1
---ACAUAGAGGAAAUUCCACGUaa-   2   1,0
---ACAUAGAGGAAAUUCCACGUUU-   2   1,2

>hsa-mir-377   mi:7-28;MA:45-66   SRX018958
uugagcAGAGGUUGCCCUUGGUGAAUUCgcuuuauuuauguugaAUCACACAAAGGCAACUUUUGUuug
..((((((((((((((.(((((((.(((((.............).)))))))).))).)))))))))))))).
------AGAGGUUGCCCUUGGUGAAU---   2   0,-2
------AGAGGUUGCCCUUGGUGAAUa--   12   0,-2
------AGAGGUUGCCCUUGGUGAAUU--   84   0,-1
------AGAGGUUGCCCUUGGUGAAUUC---   6   0,0
--AUCACACAAAGGCAACUUUU-----   6   0,-2
--AUCACACAAAGGCAACUUUUG----   6   0,-1
--AUCACACAAAGGCAACUUUUGU---   11   0,0

```
>hsa-mir-378    mi:5-26;MA:43-63    SRX018958
agggCUCCUGACUCCAGGUCCUGUGUguuaccuagaaauagcACUGGACUUGGAGUCAGAAGGccu
(((.((.((((((((((((((...((((((........)))))).))))))))))))))).)).)))
----CUCCUGACUCCAGGUCCU--------------------------------------------  7    0,-4
----CUCCUGACUCCAGGUCCUGUGU----------------------------------------  5    0,0
-----UCCUGACUCCAGGUCCUGUGU----------------------------------------  2    1,0
-----UCCUGACUCCAGGUCCUGUGUau--------------------------------------  3    1,0
-----UCCUGACUCCAGGUCCUGUGUu---------------------------------------  2    1,0
-----UCCUGACUCCAGGUCCUGUGUuu--------------------------------------  2    1,0
----------------------------------------GCACUGGACUUGGAGUCAGAA-----  2    -2,-2
----------------------------------------GCACUGGACUUGGAGUCAGAAG----  4    -2,-1
----------------------------------------GCACUGGACUUGGAGUCAGAAGa---  3    -2,-1
----------------------------------------GCACUGGACUUGGAGUCAGAAGG---  3    -2,0
-----------------------------------------CACUGGACUUGGAGUCAGA------  2    -1,-3
-----------------------------------------CACUGGACUUGGAGUCAGAA-----  2    -1,-2
-----------------------------------------CACUGGACUUGGAGUCAGAAG----  3    -1,-1
-----------------------------------------CACUGGACUUGGAGUCAGAAGG---  16   -1,0
-----------------------------------------CACUGGACUUGGAGUCAGAAGGa--  3    -1,0
-----------------------------------------CACUGGACUUGGAGUCAGAAGGaa-  2    -1,0
-----------------------------------------CACUGGACUUGGAGUCAGAAGGu--  4    -1,0
-----------------------------------------CACUGGACUUGGAGUCAGAAGGC--  33   -1,1
-----------------------------------------CACUGGACUUGGAGUCAGAAGGCa-  23   -1,1
-----------------------------------------CACUGGACUUGGAGUCAGAAGGCaa  2    -1,1
-----------------------------------------CACUGGACUUGGAGUCAGAAGGCau  5    -1,1
-----------------------------------------CACUGGACUUGGAGUCAGAAGGCu-  7    -1,1
-----------------------------------------CACUGGACUUGGAGUCAGAAGGCuu  2    -1,1
------------------------------------------ACUGGACUUGGAGUCAGA------  190  0,-3
------------------------------------------ACUGGACUUGGAGUCAGAu-----  4    0,-3
------------------------------------------ACUGGACUUGGAGUCAGAA-----  434  0,-2
------------------------------------------ACUGGACUUGGAGUCAGAAa----  53   0,-2
------------------------------------------ACUGGACUUGGAGUCAGAAaa---  14   0,-2
------------------------------------------ACUGGACUUGGAGUCAGAAu----  4    0,-2
------------------------------------------ACUGGACUUGGAGUCAGAAG----  1614     0,-1
------------------------------------------ACUGGACUUGGAGUCAGAAGu---  138  0,-1
------------------------------------------ACUGGACUUGGAGUCAGAAGuu--  4    0,-1
------------------------------------------ACUGGACUUGGAGUCAGAAGG---  11456    0,0
------------------------------------------ACUGGACUUGGAGUCAGAAGGa--  3130     0,0
------------------------------------------ACUGGACUUGGAGUCAGAAGGaa-  432  0,0
```

--ACUGGACUUGGAGUCAGAAGGau- 78 0,0
--ACUGGACUUGGAGUCAGAAGGu-- 888 0,0
--ACUGGACUUGGAGUCAGAAGGuu- 182 0,0
--ACUGGACUUGGAGUCAGAAGGC-- 25656 0,1
--ACUGGACUUGGAGUCAGAAGGCa- 14904 0,1
--ACUGGACUUGGAGUCAGAAGGCaa 1314 0,1
--ACUGGACUUGGAGUCAGAAGGCau 6175 0,1
--ACUGGACUUGGAGUCAGAAGGCu- 8292 0,1
--ACUGGACUUGGAGUCAGAAGGCuu 2994 0,1
--ACUGGACUUGGAGUCAGAAGGCC- 29 0,2
--ACUGGACUUGGAGUCAGAAGGCCa 3 0,2
--ACUGGACUUGGAGUCAGAAGGCCau 7 0,2
--ACUGGACUUGGAGUCAGAAGGCCU 22 0,3
--ACUGGACUUGGAGUCAGAAGGCCUu 9 0,3
---CUGGACUUGGAGUCAGAA----- 15 1,-2
---CUGGACUUGGAGUCAGAAG---- 27 1,-1
---CUGGACUUGGAGUCAGAAGG--- 229 1,0
---CUGGACUUGGAGUCAGAAGGa-- 29 1,0
---CUGGACUUGGAGUCAGAAGGaa- 21 1,0
---CUGGACUUGGAGUCAGAAGGu-- 25 1,0
---CUGGACUUGGAGUCAGAAGGuu- 5 1,0
---CUGGACUUGGAGUCAGAAGGC-- 2342 1,1
---CUGGACUUGGAGUCAGAAGGCa- 557 1,1
---CUGGACUUGGAGUCAGAAGGCaa 207 1,1
---CUGGACUUGGAGUCAGAAGGCau 257 1,1
---CUGGACUUGGAGUCAGAAGGCu- 738 1,1
---CUGGACUUGGAGUCAGAAGGCuu 649 1,1
---CUGGACUUGGAGUCAGAAGGCC- 9 1,2
---CUGGACUUGGAGUCAGAAGGCCa 8 1,2
---CUGGACUUGGAGUCAGAAGGCCau 22 1,2
---CUGGACUUGGAGUCAGAAGGCCU 9 1,3
---CUGGACUUGGAGUCAGAAGGCCUau 3 1,3
---CUGGACUUGGAGUCAGAAGGCCUu 3 1,3
---CUGGACUUGGAGUCAGAAGGCCUuu 5 1,3
--UGGACUUGGAGUCAGAAGGC-- 55 2,1
--UGGACUUGGAGUCAGAAGGCa- 16 2,1
--UGGACUUGGAGUCAGAAGGCau 3 2,1
--UGGACUUGGAGUCAGAAGGCu- 6 2,1

--UGGACUUGGAGUCAGAAGGCuu 12 2,1

>hsa-mir-378b 3p:34-52 SRX018958
ggucauugagucuucaaggcuaguggaaagagcACUGGACUUGGAGGCAGAAagacc
((((.((..(((((((((.((((((.......)))))).)))))))))..)).))))
---------------------------------ACUGGACUUGGAGGCAGAA----- 363 0,0

>hsa-mir-378c 5p:11-35 SRX018958
ggaggccaucACUGGACUUGGAGUCAGAAGAGUGGagucgggucagacuucaacucugacuuugaagguggugagugccu
c
.((((((((((((....(..((((((((....(((((((......)))))))..))))))))..).)))))))...))))
)
---------CACUGGACUUGGAGUCAGAAG--
- 3 -1,-5
---------CACUGGACUUGGAGUCAGAAGA---
- 3 -1,-4
----------ACUGGACUUGGAGUCAGAAG--
- 1614 0,-5
----------ACUGGACUUGGAGUCAGAAGu---
- 138 0,-5
----------ACUGGACUUGGAGUCAGAAGuu--
- 4 0,-5
----------ACUGGACUUGGAGUCAGAAGA---
- 715 0,-4
----------ACUGGACUUGGAGUCAGAAGAa--
- 65 0,-4
----------ACUGGACUUGGAGUCAGAAGAaa---
- 18 0,-4
----------ACUGGACUUGGAGUCAGAAGAau---
- 2 0,-4
----------ACUGGACUUGGAGUCAGAAGAu--
- 5 0,-4
----------ACUGGACUUGGAGUCAGAAGAuu---
- 3 0,-4
----------ACUGGACUUGGAGUCAGAAGAGa---
- 4 0,-3
-----------CUGGACUUGGAGUCAGAAG--
- 27 1,-5

-----------CUGGACUUGGAGUCAGAAGA---
-   9   1,-4
------------UGGACUUGGAGUCAGAAGA---
-   2   2,-4

>hsa-mir-379   MA:6-26;mi:44-65   SRX018958
agagaUGGUAGACUAUGGAACGUAGGcguuaugauuucugaccUAUGUAACAUGGUCCACUAACUcu
((((.((((.(((((((..((((((((((..(....)..)).))))))))..))))))).)))).))))
-----UGGUAGACUAUGGAACGUAG-- 35   0,-1
-----UGGUAGACUAUGGAACGUAGa--- 4   0,-1
-----UGGUAGACUAUGGAACGUAGG--- 114 0,0
-----UGGUAGACUAUGGAACGUAGGa-- 34   0,0
-----UGGUAGACUAUGGAACGUAGGaa--- 29   0,0
-----UGGUAGACUAUGGAACGUAGGau--- 4   0,0
-----UGGUAGACUAUGGAACGUAGGu-- 24   0,0
-----UGGUAGACUAUGGAACGUAGGuu--- 5   0,0
-----UGGUAGACUAUGGAACGUAGGC-- 2   0,1
-----UGGUAGACUAUGGAACGUAGGCa--- 3   0,1
-----UGGUAGACUAUGGAACGUAGGCau-- 2   0,1
-----UGGUAGACUAUGGAACGUAGGCu--- 2   0,1
-----UGGUAGACUAUGGAACGUAGGCGU-- 2   0,3

>hsa-mir-380   mi:5-26;MA:40-61   SRX018958
aagaUGGUUGACCAUAGAACAUGCGCuaucucugugucgUAUGUAAUAUGGUCCACAUCUU
((((((...(((((((..(((((((.(((....))).)))))))..)))))))..))))))
---------------------------------------UAUGUAAUAUGGUCCACAUCU-   2   0,-1

>hsa-mir-381   3p:49-70   SRX018958
uacuuaaagcgagguugcccuuuguauauucgguuuauugacauggaaUAUACAAGGGCAAGCUCUCUGUgagua
((((((.((.(((.((((((((.((((((((.(((....)))...)))))))))))))))))).))).)).))))))
-------AGCGAGGUUGCCCUUUGUAUAUa--- 3
-------AGCGAGGUUGCCCUUUGUAUAUU--- 4
--UAUACAAGGGCAAGCUCUCU------- 2
    0,-2
--UAUACAAGGGCAAGCUCUCUGa----- 2
    0,-1
--UAUACAAGGGCAAGCUCUCUGUa---- 3
    0,0

>hsa-mir-382 5p:11-32 SRX018958
uacuugaagaGAAGUUGUUCGUGGUGGAUUCGcuuuacuuaugacgaaucauucacggacaacacuuuuuucagua
((((.(((((((.(((((((((((..(((((((.........).)))))))..))))))))))))..))))))))))))
----------GAAGUUGUUCGUGGUGGAUU--
21 0,-2
----------GAAGUUGUUCGUGGUGGAUUa---
2 0,-2
----------GAAGUUGUUCGUGGUGGAUUC---
6 0,-1
----------GAAGUUGUUCGUGGUGGAUUCG--
9 0,0
----------GAAGUUGUUCGUGGUGGAUUCGu---
4 0,0
-----------AAGUUGUUCGUGGUGGAUU--
3 1,-2
-----------AAGUUGUUCGUGGUGGAUUC---
2 1,-1
------------AGUUGUUCGUGGUGGAUUCGCU--
2 2,2
---AAUCAUUCACGGACAACAC-----------
122
---AAUCAUUCACGGACAACACU----------
155
---AAUCAUUCACGGACAACACUU---------
38
---AAUCAUUCACGGACAACACUUU--------
15
--AUCAUUCACGGACAACAC-----------
2
--AUCAUUCACGGACAACACU----------
2

>hsa-mir-383 5p:7-28 SRX018958
cuccucAGAUCAGAAGGUGAUUGUGGCUuugggugggauauuaaucagccacagcacugccuggucagaaagag
(((.((.((((((..((((.((((((((...(((........))))))))))))))..)))))).))..)))
-----CAGAUCAGAAGGUGAUUG--- 3
-1,-5

-----CAGAUCAGAAGGUGAUUGUGGCU-- 3
-1,0
------AGAUCAGAAGGUGAUUGU-- 4
0,-4
------AGAUCAGAAGGUGAUUGUG--- 4
0,-3
------AGAUCAGAAGGUGAUUGUGG-- 16
0,-2
------AGAUCAGAAGGUGAUUGUGGC--- 8
0,-1
------AGAUCAGAAGGUGAUUGUGGCa-- 4
0,-1
------AGAUCAGAAGGUGAUUGUGGCaa--- 5
0,-1
------AGAUCAGAAGGUGAUUGUGGCau--- 3
0,-1
------AGAUCAGAAGGUGAUUGUGGCU-- 116
0,0
------AGAUCAGAAGGUGAUUGUGGCUaa-- 17
0,0
------AGAUCAGAAGGUGAUUGUGGCUU--- 81
0,1
------AGAUCAGAAGGUGAUUGUGGCUUa-- 26
0,1
------AGAUCAGAAGGUGAUUGUGGCUUau--- 5
0,1
------AGAUCAGAAGGUGAUUGUGGCUUU-- 78
0,2
------AGAUCAGAAGGUGAUUGUGGCUUUa--- 2
0,2
------AGAUCAGAAGGUGAUUGUGGCUUUu--- 4
0,2
-------GAUCAGAAGGUGAUUGUGGCU-- 2
1,0
-------GAUCAGAAGGUGAUUGUGGCUUU-- 2
1,2

>hsa-mir-3910-1 3p:63-82 SRX018958

cuuuugcugucaguuuuucuguugcuugucuugguuuuaugccuuuuauaucaaggcacauaAAAGGCAUAAAACCAAGA
CAagcaacaaaaaaaggauugaucacagaag
((((((..((((((((((.((((((((((((((((((((((((((((((..........)))))))))))))))))))))
)))))))))....))))))))))..))))))
---AAAAGGCAUAAAACCAAGA
------------------------------- 2   -1,-2
---AAAAGGCAUAAAACCAAGA
CA----------------------------- 5   -1,0

>hsa-mir-3910-2 3p:50-69   SRX018958
uuuuuuuguugcuugucuugguuuuaugccuuuuaugugccuugauauaAAAGGCAUAAAACCAAGACAagcaacagaaa
aa
.(((((((((((((((((((((((((((((((((((((......))))))))))))))))))))))))))))))))))))
).
--AAAAGGCAUAAAACCAAGA-------------
--   2   -1,-2
--AAAAGGCAUAAAACCAAGACA-----------
--   5   -1,0

>hsa-mir-3912   3p:63-83   SRX018958
agagaggaaugaacaguuaaauuauaacauguccauauuauggguuaguuguggacacauacUAACGCAUAAUAUGGACA
UGUuauaauuuaacuguuccuuucu
(((((((....(((((((((((((((((((((((((((((((.((((((.(((....))))))))).)))))))))))))
)))))))))))))))))))))))))
--UAACGCAUAAUAUGGACA
UG-----------------------   2   0,-1
---AACGCAUAAUAUGGACA
UG-----------------------   3   1,-1
---AACGCAUAAUAUGGACA
UGUUA--------------------   2   1,2

>hsa-mir-3913-1 5p:23-44   SRX018958
uuguuuauaauaaacugaaauaUUUGGGACUGAUCUUGAUGUCUgccaaaaccuuggcagacaucaagaucagucccaaa
uauuucaguuuauuauagacag
.((....)))))))))))))))))))))))))))
))))))))))))))))))))).
---GACAUCAAGAUCAGUCCCAA-
----------------------   2

>hsa-mir-3913-2 5p:22-43 SRX018958

ugucuauaauaaacugaaauaUUUGGGACUGAUCUUGAUGUCUgccaagguuuuggcagacaucaagaucagucccaaau
auuucaguuuauuauaaaca

.((.((....))))))))))))))))))))))))))))
)))))))))))))))).)).

--GACAUCAAGAUCAGUCCCAA--
-------------------- 2

>hsa-mir-3928 3p:37-58 SRX018958

gcugaagcucuaagguuccgccugcgggcaggaagcGGAGGAACCUUGGAGCUUCGGC

(((((((((((((((((((..((((.........)))).)))))))))))))))))))

------------------------------------GGAGGAACCUUGGAGCUUCGG- 11 0,-1

------------------------------------GGAGGAACCUUGGAGCUUCGGu 5 0,-1

------------------------------------GGAGGAACCUUGGAGCUUCGGC 6 0,0

------------------------------------GGAGGAACCUUGGAGCUUCGGCa 15 0,0

------------------------------------GGAGGAACCUUGGAGCUUCGGCaa 2 0,0

------------------------------------GGAGGAACCUUGGAGCUUCGGCau 8 0,0

------------------------------------GGAGGAACCUUGGAGCUUCGGCu 6 0,0

-------------------------------------GAGGAACCUUGGAGCUUCGGCaa 3 1,0

>hsa-mir-3929 3p:33-55 SRX018958

aguggcucacaccaguaaucccagcacuuuggGAGGCUGAUGUGAGUAGACCACU

(((((((((((.((((..((((((....)))))).)))).))))))....)))))

>hsa-mir-3942 5p:23-44 SRX018958

ucuucaguaugacaccucaaagAAGCAAUACUGUUACCUGAAAUaggcugcgaagauaacaguauuucagauaacaguau
uacaucuuugaagugucauauucacugac

...((((((((((((.(((((((.(.((((((((((.(((((((..((((.........))))))))))).)))))))))
).).))))))).)))))))....))))).

-----------------------AGCAAUACUGUUACCUGAAA-------------------------------------
----------------------------- 2 1,-1

>hsa-mir-409 5p:15-37;3p:47-68 SRX018958

ugguacucggggagAGGUUACCCGAGCAACUUUGCAUcuggacgacGAAUGUUGCUCGGUGAACCCCUuuucgguauca

.(((((.((..(((.((((..((((((((((((((........).)))).)))))))))..)))).)))..))))))).

--------------AGGUUACCCGAGCAACUU---
4 0,-5

--------------AGGUUACCCGAGCAACUUU--

44   0,-4

--------------AGGUUACCCGAGCAACUUUG---

5   0,-3

--------------AGGUUACCCGAGCAACUUUGC--

4   0,-2

--------------AGGUUACCCGAGCAACUUUGCA---

67   0,-1

--------------AGGUUACCCGAGCAACUUUGCAU--

64   0,0

--------------AGGUUACCCGAGCAACUUUGCAUa---

7   0,0

--------------AGGUUACCCGAGCAACUUUGCAUC---

2   0,1

--------------AGGUUACCCGAGCAACUUUGCAUCU--

3   0,2

---CGAAUGUUGCUCGGUGAA----------------

27   -1,-5

---CGAAUGUUGCUCGGUGAAC---------------

4   -1,-4

---CGAAUGUUGCUCGGUGAACaa-------------

2   -1,-4

---CGAAUGUUGCUCGGUGAACCCCU-----------

9   -1,0

---CGAAUGUUGCUCGGUGAACCCCUU----------

6   -1,1

--GAAUGUUGCUCGGUGAAC---------------

4   0,-4

--GAAUGUUGCUCGGUGAACC--------------

2   0,-3

--GAAUGUUGCUCGGUGAACCCC------------

13   0,-1

--GAAUGUUGCUCGGUGAACCCCU-----------

17   0,0

--GAAUGUUGCUCGGUGAACCCCUau---------

3   0,0

--GAAUGUUGCUCGGUGAACCCCUU----------

5   0,1

--GAAUGUUGCUCGGUGAACCCCUUU---------
    8   0,2
---AAUGUUGCUCGGUGAACCCCU-----------
    3   1,0
---AAUGUUGCUCGGUGAACCCCUa----------
    2   1,0
---AAUGUUGCUCGGUGAACCCCUU----------
    2   1,1
---AAUGUUGCUCGGUGAACCCCUUU---------
    2   1,2

>hsa-mir-410   3p:50-70   SRX018958
gguaccugagaagagguugucugugaugaguucgcuuuuauuaaugacgAAUAUAACACAGAUGGCCUGUuuucaguacc
(((((.((((((.((((..((((((.((.((((((..........).))))).)).))))))..)))).)))))))))))
---AAUAUAACACAGAUGGCCU------------
    12 0,-2

>hsa-mir-411   MA:16-36;mi:51-72   SRX018958
ugguacuuggagagaUAGUAGACCGUAUAGCGUACGcuuuaucugugacgUAUGUAACACGGUCCACUAACCcucaguau
caaauccauccccgag
(((((((..(((.(.((((.((((((.(.((((((((.........).))))))).).)))))).)))).).))))))))
))..............
--------------AUAGUAGACCGUAUAGCG--
----------------   11 -1,-4
--------------AUAGUAGACCGUAUAGCGU---
----------------   248 -1,-3
--------------AUAGUAGACCGUAUAGCGUA--
----------------   144 -1,-2
--------------AUAGUAGACCGUAUAGCGUAa---
----------------   20 -1,-2
--------------AUAGUAGACCGUAUAGCGUAaa--
----------------   4   -1,-2
--------------AUAGUAGACCGUAUAGCGUAu---
----------------   9   -1,-2
--------------AUAGUAGACCGUAUAGCGUAC---
----------------   470 -1,-1
--------------AUAGUAGACCGUAUAGCGUACa--
----------------   3   -1,-1

--------------AUAGUAGACCGUAUAGCGUACaa---
----------------   3   -1,-1
--------------AUAGUAGACCGUAUAGCGUACG--
----------------   83   -1,0
--------------AUAGUAGACCGUAUAGCGUACGa---
----------------   4   -1,0
---------------UAGUAGACCGUAUAGCGU---
----------------   235 0,-3
---------------UAGUAGACCGUAUAGCGUA--
----------------   201 0,-2
---------------UAGUAGACCGUAUAGCGUAa---
----------------   13   0,-2
---------------UAGUAGACCGUAUAGCGUAaa--
----------------   3   0,-2
---------------UAGUAGACCGUAUAGCGUAu---
----------------   4   0,-2
---------------UAGUAGACCGUAUAGCGUAC---
----------------   432 0,-1
---------------UAGUAGACCGUAUAGCGUACa--
----------------   8   0,-1
---------------UAGUAGACCGUAUAGCGUACaa---
----------------   2   0,-1
---------------UAGUAGACCGUAUAGCGUACG--
----------------   70   0,0
--UAUGUAACACGGUCCACUAAC---------
----------------   7   0,-1
--UAUGUAACACGGUCCACUAACa--------
----------------   4   0,-1
--UAUGUAACACGGUCCACUAACu--------
----------------   4   0,-1

>hsa-mir-421   3p:48-70   SRX018958
gcacauuguaggccucauuaaauguuuguugaaugaaaaaaugaaucAUCAACAGACAUUAAUUGGGCGCcugcucugug
aucuc
.((((..((((((((((...((((((((((((.(((.........)))))))))))))))...)))).))))))..))))
.....
---AUCAACAGACAUUAAUUG---------------
-----   47   0,-5

---AUCAACAGACAUUAAUUGG--------------
-----   16 0,-4
---AUCAACAGACAUUAAUUGGG-------------
-----   2   0,-3
---AUCAACAGACAUUAAUUGGGCGa----------
-----   2   0,-1
---AUCAACAGACAUUAAUUGGGCGau---------
-----   3   0,-1
---AUCAACAGACAUUAAUUGGGCGC---------
-----   3   0,0

>hsa-mir-423   5p:17-39;3p:53-75   SRX018958
auaaaggaaguuaggcUGAGGGGCAGAGAGCGAGACUUUucuauuuuccaaaAGCUCGGUCUGAGGCCCCUCAGUcuugc
uuccuaacccgcgc
....(((((((.((((((((((((..(((.((((.(((((.........))))))))).)))...)))))))))))).))
))))).........
---------------CUGAGGGGCAGAGAGCGAGAu--
-------------- 2   -1,-4
---------------CUGAGGGGCAGAGAGCGAGACUUU---
-------------- 9   -1,0
----------------UGAGGGGCAGAGAGCGAG--
-------------- 9   0,-5
----------------UGAGGGGCAGAGAGCGAGu---
-------------- 2   0,-5
----------------UGAGGGGCAGAGAGCGAGA---
-------------- 36 0,-4
----------------UGAGGGGCAGAGAGCGAGAa--
-------------- 3   0,-4
----------------UGAGGGGCAGAGAGCGAGAaa---
-------------- 7   0,-4
----------------UGAGGGGCAGAGAGCGAGAC--
-------------- 30 0,-3
----------------UGAGGGGCAGAGAGCGAGACaa--
-------------- 4   0,-3
----------------UGAGGGGCAGAGAGCGAGACU---
-------------- 50 0,-2
----------------UGAGGGGCAGAGAGCGAGACUa--
-------------- 2   0,-2

----------------UGAGGGGCAGAGAGCGAGACUaa--
-------------- 4 0,-2
----------------UGAGGGGCAGAGAGCGAGACUau--
-------------- 4 0,-2
----------------UGAGGGGCAGAGAGCGAGACUU---
-------------- 91 0,-1
----------------UGAGGGGCAGAGAGCGAGACUUa--
-------------- 31 0,-1
----------------UGAGGGGCAGAGAGCGAGACUUaa---
-------------- 6 0,-1
----------------UGAGGGGCAGAGAGCGAGACUUau---
-------------- 16 0,-1
----------------UGAGGGGCAGAGAGCGAGACUUU--
-------------- 918 0,0
----------------UGAGGGGCAGAGAGCGAGACUUUa---
-------------- 33 0,0
----------------UGAGGGGCAGAGAGCGAGACUUUaa--
-------------- 7 0,0
----------------UGAGGGGCAGAGAGCGAGACUUUau--
-------------- 8 0,0
----------------UGAGGGGCAGAGAGCGAGACUUUU---
-------------- 208 0,1
----------------UGAGGGGCAGAGAGCGAGACUUUUa--
-------------- 3 0,1
----------------UGAGGGGCAGAGAGCGAGACUUUUu--
-------------- 5 0,1
-----------------GAGGGGCAGAGAGCGAGAC---
-------------- 2 1,-3
-----------------GAGGGGCAGAGAGCGAGACUU---
-------------- 4 1,-1
-----------------GAGGGGCAGAGAGCGAGACUUU--
-------------- 55 1,0
-----------------GAGGGGCAGAGAGCGAGACUUUa---
-------------- 2 1,0
-----------------GAGGGGCAGAGAGCGAGACUUUU---
-------------- 9 1,1
------------------AGGGGCAGAGAGCGAGACUUU--
-------------- 8 2,0

------------------AGGGGCAGAGAGCGAGACUUUU--
-------------- 35 2,1
------------------AGGGGCAGAGAGCGAGACUUUUa---------------------------------------
-------------- 5 2,1
------------------AGGGGCAGAGAGCGAGACUUUUu---------------------------------------
-------------- 2 2,1
---AAGCUCGGUCUGAGGCCCC----------
-------------- 4 -1,-5
---AAGCUCGGUCUGAGGCCCCU---------
-------------- 2 -1,-4
---AAGCUCGGUCUGAGGCCCCUaa-------
-------------- 2 -1,-4
---AAGCUCGGUCUGAGGCCCCUu--------
-------------- 3 -1,-4
---AAGCUCGGUCUGAGGCCCCUuu-------
-------------- 2 -1,-4
---AAGCUCGGUCUGAGGCCCCUCA-------
-------------- 5 -1,-2
---AAGCUCGGUCUGAGGCCCCUCAu------
-------------- 7 -1,-2
---AAGCUCGGUCUGAGGCCCCUCAuu-----
-------------- 2 -1,-2
---AAGCUCGGUCUGAGGCCCCUCAG------
-------------- 11 -1,-1
---AAGCUCGGUCUGAGGCCCCUCAGa-----
-------------- 2 -1,-1
---AAGCUCGGUCUGAGGCCCCUCAGU-----
-------------- 47 -1,0
---AAGCUCGGUCUGAGGCCCCUCAGUu----
-------------- 5 -1,0
--AGCUCGGUCUGAGGCCCC----------
-------------- 3 0,-5
--AGCUCGGUCUGAGGCCCCU---------
-------------- 3 0,-4
--AGCUCGGUCUGAGGCCCCUC--------
-------------- 9 0,-3
--AGCUCGGUCUGAGGCCCCUCA-------
-------------- 23 0,-2

---AGCUCGGUCUGAGGCCCCUCAa------
-------------- 10 0,-2
---AGCUCGGUCUGAGGCCCCUCAaa-----
-------------- 10 0,-2
---AGCUCGGUCUGAGGCCCCUCAG------
-------------- 49 0,-1
---AGCUCGGUCUGAGGCCCCUCAGa-----
-------------- 9 0,-1
---AGCUCGGUCUGAGGCCCCUCAGU-----
-------------- 213 0,0
---AGCUCGGUCUGAGGCCCCUCAGUa----
-------------- 21 0,0
---AGCUCGGUCUGAGGCCCCUCAGUaa---
-------------- 13 0,0
---AGCUCGGUCUGAGGCCCCUCAGUau---
-------------- 31 0,0
---AGCUCGGUCUGAGGCCCCUCAGUu----
-------------- 44 0,0
---AGCUCGGUCUGAGGCCCCUCAGUuu---
-------------- 17 0,0
--GCUCGGUCUGAGGCCCCUCAGUaa---
-------------- 2 1,0

>hsa-mir-424 MA:11-32;mi:48-68 SRX018958
cgaggggauaCAGCAGCAAUUCAUGUUUUGAAguguucuaaaugguuCAAAACGUGAGGCGCUGCUAUacccccucgugg
ggaagguagaaggugggg
(((((((.((.((((((..((((((((((((((.(((.....))).))))))))))))))..)))))).)).)))))))...
....
---------ACAGCAGCAAUUCAUGUUUU---
------------------ 7 -1,-3
---------ACAGCAGCAAUUCAUGUUUUG--
------------------ 8 -1,-2
---------ACAGCAGCAAUUCAUGUUUUGA---
------------------ 2 -1,-1
---------ACAGCAGCAAUUCAUGUUUUGAA--
------------------ 5 -1,0
----------CAGCAGCAAUUCAUGUUU--
------------------ 54 0,-4

----------CAGCAGCAAUUCAUGUUUa---
------------------ 3 0,-4

----------CAGCAGCAAUUCAUGUUUU---
------------------ 332 0,-3

----------CAGCAGCAAUUCAUGUUUUa--
------------------ 9 0,-3

----------CAGCAGCAAUUCAUGUUUUaa---
------------------ 3 0,-3

----------CAGCAGCAAUUCAUGUUUUG--
------------------ 1113 0,-2

----------CAGCAGCAAUUCAUGUUUUGu---
------------------ 4 0,-2

----------CAGCAGCAAUUCAUGUUUUGA---
------------------ 3546 0,-1

----------CAGCAGCAAUUCAUGUUUUGAu--
------------------ 6 0,-1

----------CAGCAGCAAUUCAUGUUUUGAA--
------------------ 1565 0,0

----------CAGCAGCAAUUCAUGUUUUGAAa---
------------------ 209 0,0

----------CAGCAGCAAUUCAUGUUUUGAAaa--
------------------ 14 0,0

----------CAGCAGCAAUUCAUGUUUUGAAu---
------------------ 31 0,0

----------CAGCAGCAAUUCAUGUUUUGAAG---
------------------ 7 0,1

-----------AGCAGCAAUUCAUGUUUU---
------------------ 7 1,-3

-----------AGCAGCAAUUCAUGUUUUG--
------------------ 17 1,-2

-----------AGCAGCAAUUCAUGUUUUGA---
------------------ 46 1,-1

-----------AGCAGCAAUUCAUGUUUUGAA--
------------------ 40 1,0

-----------AGCAGCAAUUCAUGUUUUGAAa---
------------------ 5 1,0

--CAAAACGUGAGGCGCUGCUA--------------
------------------ 19 0,-1

---CAAAACGUGAGGCGCUGCUAa------------
------------------ 5 0,-1

---CAAAACGUGAGGCGCUGCUAaa-----------
------------------ 2 0,-1

---CAAAACGUGAGGCGCUGCUAU------------
------------------ 369 0,0

---CAAAACGUGAGGCGCUGCUAUu-----------
------------------ 24 0,0

---CAAAACGUGAGGCGCUGCUAUuu----------
------------------ 2 0,0

---CAAAACGUGAGGCGCUGCUAUA-----------
------------------ 209 0,1

---CAAAACGUGAGGCGCUGCUAUAa----------
------------------ 83 0,1

---CAAAACGUGAGGCGCUGCUAUAaa---------
------------------ 3 0,1

---CAAAACGUGAGGCGCUGCUAUAu----------
------------------ 36 0,1

---CAAAACGUGAGGCGCUGCUAUAuu---------
------------------ 2 0,1

--AAAACGUGAGGCGCUGCUAU------------
------------------ 15 1,0

--AAAACGUGAGGCGCUGCUAUA-----------
------------------ 19 1,1

--AAAACGUGAGGCGCUGCUAUAa----------
------------------ 6 1,1

--AAAACGUGAGGCGCUGCUAUAu----------
------------------ 17 1,1

--AAAACGUGAGGCGCUGCUAUAuu---------
------------------ 2 1,1

---AAACGUGAGGCGCUGCUAUAa----------
------------------ 3 2,1

---AAACGUGAGGCGCUGCUAUAu----------
------------------ 3 2,1

>hsa-mir-425 MA:14-36;mi:55-76 SRX018958

gaaagcgcuuuggAAUGACACGAUCACUCCCGUUGAgugggcacccgagaagccAUCGGGAAUGUCGUGUCCGCCCagug
cucuuuc

(((((.((.((((...(((((((.((.(((((.((.((((....))....)))).))))).)))))))))...)))).)
).)))))
-------------AAUGACACGAUCACUCCC---
------- 6 0,-5
-------------AAUGACACGAUCACUCCCG--
------- 2 0,-4
-------------AAUGACACGAUCACUCCCGU---
------- 2 0,-3
-------------AAUGACACGAUCACUCCCGUU--
------- 8 0,-2
-------------AAUGACACGAUCACUCCCGUUG---
------- 6 0,-1
-------------AAUGACACGAUCACUCCCGUUGA--
------- 49 0,0
-------------AAUGACACGAUCACUCCCGUUGAu---
------- 3 0,0
-------------AAUGACACGAUCACUCCCGUUGAG---
------- 14 0,1
--------------AUGACACGAUCACUCCCGUUGA--
------- 6 1,0
--------------AUGACACGAUCACUCCCGUUGAG---
------- 5 1,1
--------------AUGACACGAUCACUCCCGUUGAGU--
------- 6 1,2
---CAUCGGGAAUGUCGUGUCC--------
------- 3 -1,-4
---CAUCGGGAAUGUCGUGUCCGC------
------- 2 -1,-2
---CAUCGGGAAUGUCGUGUCCGCu-----
------- 5 -1,-2
---CAUCGGGAAUGUCGUGUCCGCC-----
------- 10 -1,-1
--AUCGGGAAUGUCGUGUCC--------
------- 2 0,-4
--AUCGGGAAUGUCGUGUCCGCa-----
------- 2 0,-2
--AUCGGGAAUGUCGUGUCCGCC-----
------- 9 0,-1

---AUCGGGAAUGUCGUGUCCGCCu----
------- 4   0,-1
---AUCGGGAAUGUCGUGUCCGCCC----
------- 3   0,0

>hsa-mir-4284   5p:11-28   SRX018958
guucugugagGGGCUCACAUCACCCCAUcaaaguggggacucauggggagagggggguaguuaggagcuuugauagaggcg
g
.((((((..(((((((..((.(((((.((...((((....))))...))...))))).))...))))))).))))))...
.
----------GGGCUCACAUCACCCCAU---
-   14 0,0

>hsa-mir-4286   5p:11-27   SRX018958
uacuuauggcACCCCACUCCUGGUACCauagucauaaguuaggagauguuagagcugugaguaccaugacuuaagugugg
uggcuuaaacaug
...(((.(.((((.(((((.((((((((((((..((((((....))).)))..)))))).)))))).))......))).))
)).).))))....
----------ACCCCACUCCUGGUACCA---
-------------   32 0,1
----------ACCCCACUCCUGGUACCAa--
-------------   6   0,1
----------ACCCCACUCCUGGUACCAaa---
-------------   2   0,1
----------ACCCCACUCCUGGUACCAU--
-------------   10 0,2
----------ACCCCACUCCUGGUACCAUu---
-------------   10 0,2
----------ACCCCACUCCUGGUACCAUuu--
-------------   3   0,2
----------ACCCCACUCCUGGUACCAUA---
-------------   2   0,3

>hsa-mir-429   3p:51-72   SRX018958
cgccggccgaugggcgucuuaccagacaugguuagaccuggcccucugucUAAUACUGUCUGGUAAAACCGUccauccgc
ugc
...((((.((((((((..((((((((((..(((((((.........)))))))..))))))))))...)))))))).))
)).

---UAAUACUGUCUGGUAAAA------------
--- 11 0,-4
---UAAUACUGUCUGGUAAAAC-----------
--- 2   0,-3
---UAAUACUGUCUGGUAAAACC----------
--- 5   0,-2
---UAAUACUGUCUGGUAAAACCG---------
--- 26 0,-1
---UAAUACUGUCUGGUAAAACCGU--------
--- 73 0,0
--AAUACUGUCUGGUAAAAC-----------
--- 3   1,-3

>hsa-mir-4306   3p:65-81   SRX018958
aagcugcuuagugucccuuagagucuccagaggcaucccuaacccagaaucuuuugacugucccucUGGAGAGAAAGGCAGU
Aggucuguacc
..((.(((((.((.((((....((((((((((((((.................)).)).))))))))))..)))))).)
))))..))...
--UCUGGAGAGAAAGGCAGU
----------- 4   -2,-1

>hsa-mir-432   MA:14-36;mi:62-82   SRX018958
ugacucccuccaggUCUUGGAGUAGGUCAUUGGGUGGauccucuauuuccuuacgugggccaCUGGAUGGCUCCUCCAUGU
CUuggaguagauca
.((((.(((((((.(.(((((..(((((((.(((((.(((.(((......)).).)))))))).))))))).))))).).
))))))).)).)).
-------------UCUUGGAGUAGGUCAUUG--
-------------- 56 0,-5
-------------UCUUGGAGUAGGUCAUUGa---
-------------- 3   0,-5
-------------UCUUGGAGUAGGUCAUUGG--
-------------- 91 0,-4
-------------UCUUGGAGUAGGUCAUUGGa---
-------------- 2   0,-4
-------------UCUUGGAGUAGGUCAUUGGaa--
-------------- 7   0,-4
-------------UCUUGGAGUAGGUCAUUGGG---
-------------- 77 0,-3

-------------UCUUGGAGUAGGUCAUUGGGa---
-------------- 6 0,-3
-------------UCUUGGAGUAGGUCAUUGGGaa--
-------------- 4 0,-3
-------------UCUUGGAGUAGGUCAUUGGGU---
-------------- 28 0,-2
-------------UCUUGGAGUAGGUCAUUGGGUa--
-------------- 7 0,-2
-------------UCUUGGAGUAGGUCAUUGGGUaa---
-------------- 23 0,-2
-------------UCUUGGAGUAGGUCAUUGGGUau---
-------------- 32 0,-2
-------------UCUUGGAGUAGGUCAUUGGGUu--
-------------- 2 0,-2
-------------UCUUGGAGUAGGUCAUUGGGUG--
-------------- 28 0,-1
-------------UCUUGGAGUAGGUCAUUGGGUGa---
-------------- 14 0,-1
-------------UCUUGGAGUAGGUCAUUGGGUGau--
-------------- 6 0,-1
-------------UCUUGGAGUAGGUCAUUGGGUGu---
-------------- 12 0,-1
-------------UCUUGGAGUAGGUCAUUGGGUGG---
-------------- 2 0,0
-------------UCUUGGAGUAGGUCAUUGGGUGGu--
-------------- 4 0,0

>hsa-mir-4326 5p:11-30 SRX018958
gcugcucugcUGUUCCUCUGUCUCCCAGACucuggguggauggagcaggucgggggcca
(((.((...(((((((...((((((((...))))).)))))))))))...)).)))..
----------UGUUCCUCUGUCUCCCAGACU---------------------------- 2 0,1

>hsa-mir-433 3p:64-85 SRX018958
ccggggagaaguacggugagccugucauuauucagagaggcuagauccucuguguugagaaggAUCAUGAUGGGCUCCUC
GGUGUucuccagg
((..((((((...(((.(((((((((((.(((((.(((((......))))).)).......))).))))))))))).))
)...)))))).))
-----------UACGGUGAGCCUGUCAUUAUa---

------------ 2
--AUCAUGAUGGGCUCCUC
G----------- 2 0,-4
--AUCAUGAUGGGCUCCUC
GGUaa-------- 2 0,-2
--AUCAUGAUGGGCUCCUC
GGUau-------- 2 0,-2
--AUCAUGAUGGGCUCCUC
GGUG--------- 5 0,-1
--AUCAUGAUGGGCUCCUC
GGUGa-------- 2 0,-1
--AUCAUGAUGGGCUCCUC
GGUGU-------- 43 0,0
--AUCAUGAUGGGCUCCUC
GGUGUa------- 4 0,0
--AUCAUGAUGGGCUCCUC
GGUGUau------ 2 0,0
--AUCAUGAUGGGCUCCUC
GGUGUU------- 6 0,1
--AUCAUGAUGGGCUCCUC
GGUGUUu------ 4 0,1

>hsa-mir-450a-1 5p:18-39 SRX018958
aaacgauacuaaacuguUUUUGCGAUGUGUUCCUAAUAUgcacuauaaauauauugggaacauuuugcauguauaguuuu
guaucaauaua
....(((((.(((((((...(((((.((((((((((((((.........)))))))))))))).)))))...))))))).
))))).......
-----------------UUUUGCGAUGUGUUCCUAAU--
---------- 16 0,-2
-----------------UUUUGCGAUGUGUUCCUAAUA---
---------- 19 0,-1
-----------------UUUUGCGAUGUGUUCCUAAUAa--
---------- 5 0,-1
-----------------UUUUGCGAUGUGUUCCUAAUAU--
---------- 32 0,0
--AUUGGGAACAUUUUGCAUGUAU------
---------- 8
--AUUGGGAACAUUUUGCAUGUAUA-----

----------- 4

>hsa-mir-450a-2 5p:23-44   SRX018958
ccaaagaaagaugcuaaacuauUUUUGCGAUGUGUUCCUAAUAUguaauauaaauguauuggggacauuuugcauucaua
guuuuguaucaauaaauaugg
.........(((((.(((((((...(((((.(((((((((((((((.......))))))))))))))).)))))...)))
)))).))))).........
----------------------UUUUGCGAUGUGUUCCUAAU--------------------------------------
---------------------   16   0,-2
----------------------UUUUGCGAUGUGUUCCUAAUA-------------------------------------
---------------------   19   0,-1
----------------------UUUUGCGAUGUGUUCCUAAUAa------------------------------------
---------------------   5   0,-1
----------------------UUUUGCGAUGUGUUCCUAAUAU------------------------------------
---------------------   32   0,0
--UAUUGGGGACAUUUUGCAUU----
---------------------   2
---AUUGGGGACAUUUUGCAU-----
---------------------   2
---AUUGGGGACAUUUUGCAUa----
---------------------   4
---AUUGGGGACAUUUUGCAUU----
---------------------   23
---AUUGGGGACAUUUUGCAUUCA--
---------------------   7
---AUUGGGGACAUUUUGCAUUCAU-
---------------------   22
--UUGGGGACAUUUUGCAUU----
---------------------   3
--UUGGGGACAUUUUGCAUUCAU-
---------------------   4

>hsa-mir-450b   5p:11-32;3p:48-69   SRX018958
gcagaauuauUUUUGCAAUAUGUUCCUGAAUAuguaauauaaguguaUUGGGAUCAUUUUGCAUCCAUAguuuuguau
((((((((((...(((((.(((.((((.((((..(.......)..)))))))).))).)))))...))))))))))..
----------UUUUGCAAUAUGUUCCUG--
    4   0,-4
----------UUUUGCAAUAUGUUCCUGA---

4 0,-3
----------UUUUGCAAUAUGUUCCUGAA--
14 0,-2
----------UUUUGCAAUAUGUUCCUGAAU---
12 0,-1
----------UUUUGCAAUAUGUUCCUGAAUA--
7 0,0
----------UUUUGCAAUAUGUUCCUGAAUAa---
15 0,0
----------UUUUGCAAUAUGUUCCUGAAUAU---
2 0,1
--AUUGGGAUCAUUUUGCAUC-------------
2 -1,-4

>hsa-mir-451 5p:17-38 SRX018958
cuugggaauggcaaggAAACCGUUACCAUUACUGAGUUuaguaaugguaaugguucucuugcuauacccaga
.(((((.((((((((..(((((((((((((((((....)))))))))))))))))..)))))))).))))).
----------------AAACCGUUACCAUUACUG-------------------------------------- 98
0,-4
----------------AAACCGUUACCAUUACUGA------------------------------------- 34
0,-3
----------------AAACCGUUACCAUUACUGAG------------------------------------ 228
0,-2
----------------AAACCGUUACCAUUACUGAGau---------------------------------- 4
0,-2
----------------AAACCGUUACCAUUACUGAGU----------------------------------- 245
0,-1
----------------AAACCGUUACCAUUACUGAGUU---------------------------------- 2126
0,0
----------------AAACCGUUACCAUUACUGAGUUa--------------------------------- 4
0,0
----------------AAACCGUUACCAUUACUGAGUUU--------------------------------- 548
0,1
----------------AAACCGUUACCAUUACUGAGUUUA-------------------------------- 71
0,2
----------------AAACCGUUACCAUUACUGAGUUUAa------------------------------- 3
0,2
----------------AAACCGUUACCAUUACUGAGUUUAG------------------------------- 45

0,3
-----------------AACCGUUACCAUUACUGAG------------------------------------ 2
1,-2
-----------------AACCGUUACCAUUACUGAGUU---------------------------------- 10
1,0
-----------------AACCGUUACCAUUACUGAGUUa--------------------------------- 3
1,0
-----------------AACCGUUACCAUUACUGAGUUU--------------------------------- 12
1,1

>hsa-mir-452 MA:14-35;mi:58-79 SRX018958
gcuaagcacuuacAACUGUUUGCAGAGGAAACUGAgacuuuguaacuaugucucaguCUCAUCUGCAAAGAAGUAAGUGc
uuugc
((.((((((((((...(.((((((((.((.((((((((...........)))))))).)).)))))))).).))))))))
)).))
-------------AACUGUUUGCAGAGGAAA---
----- 22 0,-4
-------------AACUGUUUGCAGAGGAAAC--
----- 97 0,-3
-------------AACUGUUUGCAGAGGAAACU---
----- 128 0,-2
-------------AACUGUUUGCAGAGGAAACUG--
----- 2 0,-1
-------------AACUGUUUGCAGAGGAAACUGA---
----- 8 0,0
-------------AACUGUUUGCAGAGGAAACUGAa--
----- 5 0,0
-------------AACUGUUUGCAGAGGAAACUGAG--
----- 16 0,1

>hsa-mir-454 mi:24-45;MA:64-86 SRX018958
ucuguuuaucaccagauccuagaACCCUAUCAAUAUUGUCUCUGCuguguaaauaguucugagUAGUGCAAUAUUGCUUA
UAGGGUuuugguguuuggaaagaacaaugggcagg
.(((((((((.(((((..((((((((((((((((((((((((.(((((....)))))...)))....)))))))))...)
))))))))))).)))))...))....))))))).
---UAGUGCAAUAUUGCUUA
UA--------------------------------- 6 0,-4
---UAGUGCAAUAUUGCUUA

UAGG------------------------------- 2 0,-2
---UAGUGCAAUAUUGCUUA
UAGGGU----------------------------- 2 0,0
---UAGUGCAAUAUUGCUUA
UAGGGUUU--------------------------- 2 0,2

>hsa-mir-455 5p:16-37;3p:54-74 SRX018958
ucccuggcgugagggUAUGUGCCUUUGGACUACAUCGuggaagccagcaccauGCAGUCCAUGGGCAUAUACACuugccu
caaggccuaugucauc
.....(((.((((((((((((((.((((((.(((.(((........))).))).)))))).)))))))))).....)))
))..))).........
--------------GUAUGUGCCUUUGGACUA--
---------------- 5 -1,-5
--------------GUAUGUGCCUUUGGACUACA--
---------------- 14 -1,-3
--------------GUAUGUGCCUUUGGACUACAa---
---------------- 2 -1,-3
--------------GUAUGUGCCUUUGGACUACAU---
---------------- 153 -1,-2
--------------GUAUGUGCCUUUGGACUACAUa--
---------------- 74 -1,-2
--------------GUAUGUGCCUUUGGACUACAUaa---
---------------- 9 -1,-2
--------------GUAUGUGCCUUUGGACUACAUu--
---------------- 36 -1,-2
--------------GUAUGUGCCUUUGGACUACAUC--
---------------- 28 -1,-1
--------------GUAUGUGCCUUUGGACUACAUCa---
---------------- 2 -1,-1
--------------GUAUGUGCCUUUGGACUACAUCG---
---------------- 48 -1,0
--------------GUAUGUGCCUUUGGACUACAUCGa--
---------------- 3 -1,0
--------------GUAUGUGCCUUUGGACUACAUCGU--
---------------- 39 -1,1
---------------UAUGUGCCUUUGGACUACA--
---------------- 15 0,-3
---------------UAUGUGCCUUUGGACUACAa---

--------------- 2 0,-3
---------------UAUGUGCCUUUGGACUACAU---
--------------- 26 0,-2
---------------UAUGUGCCUUUGGACUACAUa--
--------------- 177 0,-2
---------------UAUGUGCCUUUGGACUACAUu--
--------------- 2 0,-2
---------------UAUGUGCCUUUGGACUACAUC--
--------------- 100 0,-1
---------------UAUGUGCCUUUGGACUACAUCG---
--------------- 196 0,0
---------------UAUGUGCCUUUGGACUACAUCGa--
--------------- 9 0,0
---------------UAUGUGCCUUUGGACUACAUCGU--
--------------- 67 0,1
---------------UAUGUGCCUUUGGACUACAUCGUG---
--------------- 5 0,2
---------------UAUGUGCCUUUGGACUACAUCGUGu--
--------------- 2 0,2
----------------AUGUGCCUUUGGACUACAUa--
--------------- 2 1,-2
----------------AUGUGCCUUUGGACUACAUCG---
--------------- 4 1,0
----------------AUGUGCCUUUGGACUACAUCGU--
--------------- 4 1,1
-----------------UGUGCCUUUGGACUACAUa--
--------------- 2 2,-2
-----------------UGUGCCUUUGGACUACAUC--
--------------- 2 2,-1
-----------------UGUGCCUUUGGACUACAUCGU--
--------------- 2 2,1
-----------------UGUGCCUUUGGACUACAUCGUG---
--------------- 6 2,2
-----------------UGUGCCUUUGGACUACAUCGUGa--
--------------- 12 2,2
-----------------UGUGCCUUUGGACUACAUCGUGu--
--------------- 4 2,2
---AUGCAGUCCAUGGGCAUA-----------

--------------- 9 -2,-5
---AUGCAGUCCAUGGGCAUAU----------
--------------- 16 -2,-4
---AUGCAGUCCAUGGGCAUAUA---------
--------------- 64 -2,-3
---AUGCAGUCCAUGGGCAUAUAa--------
--------------- 18 -2,-3
---AUGCAGUCCAUGGGCAUAUAaa-------
--------------- 2 -2,-3
---AUGCAGUCCAUGGGCAUAUAu--------
--------------- 12 -2,-3
---AUGCAGUCCAUGGGCAUAUAC--------
--------------- 58 -2,-2
---AUGCAGUCCAUGGGCAUAUACu-------
--------------- 5 -2,-2
---AUGCAGUCCAUGGGCAUAUACA-------
--------------- 149 -2,-1
---AUGCAGUCCAUGGGCAUAUACAa------
--------------- 10 -2,-1
---AUGCAGUCCAUGGGCAUAUACAu------
--------------- 3 -2,-1
---AUGCAGUCCAUGGGCAUAUACAC------
--------------- 78 -2,0
---AUGCAGUCCAUGGGCAUAUACACa-----
--------------- 11 -2,0
---AUGCAGUCCAUGGGCAUAUACACU-----
--------------- 13 -2,1
--UGCAGUCCAUGGGCAUAUA---------
--------------- 3 -1,-3
--UGCAGUCCAUGGGCAUAUAC--------
--------------- 5 -1,-2
--UGCAGUCCAUGGGCAUAUACA-------
--------------- 25 -1,-1
--UGCAGUCCAUGGGCAUAUACAC------
--------------- 18 -1,0
--UGCAGUCCAUGGGCAUAUACACa-----
--------------- 7 -1,0
--UGCAGUCCAUGGGCAUAUACACU-----

--------------- 5 -1,1

--GCAGUCCAUGGGCAUAUA---------

--------------- 61 0,-3

--GCAGUCCAUGGGCAUAUAa--------

--------------- 8 0,-3

--GCAGUCCAUGGGCAUAUAu--------

--------------- 7 0,-3

--GCAGUCCAUGGGCAUAUAC--------

--------------- 47 0,-2

--GCAGUCCAUGGGCAUAUACA-------

--------------- 389 0,-1

--GCAGUCCAUGGGCAUAUACAa------

--------------- 34 0,-1

--GCAGUCCAUGGGCAUAUACAu------

--------------- 8 0,-1

--GCAGUCCAUGGGCAUAUACAuu-----

--------------- 10 0,-1

--GCAGUCCAUGGGCAUAUACAC------

--------------- 672 0,0

--GCAGUCCAUGGGCAUAUACACa-----

--------------- 98 0,0

--GCAGUCCAUGGGCAUAUACACaa----

--------------- 16 0,0

--GCAGUCCAUGGGCAUAUACACU-----

--------------- 796 0,1

--GCAGUCCAUGGGCAUAUACACUa----

--------------- 9 0,1

--GCAGUCCAUGGGCAUAUACACUU----

--------------- 28 0,2

--GCAGUCCAUGGGCAUAUACACUUa---

--------------- 5 0,2

--GCAGUCCAUGGGCAUAUACACUUu---

--------------- 4 0,2

--GCAGUCCAUGGGCAUAUACACUUuu--

--------------- 2 0,2

---CAGUCCAUGGGCAUAUACA-------

--------------- 13 1,-1

---CAGUCCAUGGGCAUAUACAuu-----

--------------- 2 1,-1
--CAGUCCAUGGGCAUAUACAC------
--------------- 114 1,0
--CAGUCCAUGGGCAUAUACACa-----
--------------- 3 1,0
--CAGUCCAUGGGCAUAUACACU-----
--------------- 200 1,1
--CAGUCCAUGGGCAUAUACACUa----
--------------- 7 1,1
--CAGUCCAUGGGCAUAUACACUaa---
--------------- 2 1,1
--CAGUCCAUGGGCAUAUACACUU----
--------------- 77 1,2
--CAGUCCAUGGGCAUAUACACUUa---
--------------- 30 1,2
--CAGUCCAUGGGCAUAUACACUUu---
--------------- 44 1,2
--CAGUCCAUGGGCAUAUACACUUuu--
--------------- 5 1,2
---AGUCCAUGGGCAUAUACAC------
--------------- 4 2,0
---AGUCCAUGGGCAUAUACACU-----
--------------- 6 2,1
---AGUCCAUGGGCAUAUACACUU----
--------------- 2 2,2

>hsa-mir-483 5p:8-29;3p:48-68 SRX018958
gagggggAAGACGGGAGGAAAGAAGGGAGugguuccaucacgccuccUCACUCCUCUCCUCCCGUCUUcuccucuc
(((((((((((((((((.((.((.(((.(((........)))..))).)).)).))))))))))))).))))))
-------AAGACGGGAGGAAAGAAG---
154 0,-4
-------AAGACGGGAGGAAAGAAGa--
2 0,-4
-------AAGACGGGAGGAAAGAAGaa---
3 0,-4
-------AAGACGGGAGGAAAGAAGu--
3 0,-4
-------AAGACGGGAGGAAAGAAGG--

309 0,-3

-------AAGACGGGAGGAAAGAAGGa---

21 0,-3

-------AAGACGGGAGGAAAGAAGGaa--

21 0,-3

-------AAGACGGGAGGAAAGAAGGu---

5 0,-3

-------AAGACGGGAGGAAAGAAGGG---

743 0,-2

-------AAGACGGGAGGAAAGAAGGGu--

17 0,-2

-------AAGACGGGAGGAAAGAAGGGuu---

7 0,-2

-------AAGACGGGAGGAAAGAAGGGA--

1701 0,-1

-------AAGACGGGAGGAAAGAAGGGAa---

548 0,-1

-------AAGACGGGAGGAAAGAAGGGAaa--

306 0,-1

-------AAGACGGGAGGAAAGAAGGGAau--

70 0,-1

-------AAGACGGGAGGAAAGAAGGGAu---

130 0,-1

-------AAGACGGGAGGAAAGAAGGGAuu--

66 0,-1

-------AAGACGGGAGGAAAGAAGGGAG---

3161 0,0

-------AAGACGGGAGGAAAGAAGGGAGa--

918 0,0

-------AAGACGGGAGGAAAGAAGGGAGaa---

77 0,0

-------AAGACGGGAGGAAAGAAGGGAGau---

67 0,0

-------AAGACGGGAGGAAAGAAGGGAGU--

604 0,1

-------AAGACGGGAGGAAAGAAGGGAGUa---

18 0,1

-------AAGACGGGAGGAAAGAAGGGAGUaa--

12 0,1

-------AAGACGGGAGGAAAGAAGGGAGUau--

19 0,1

-------AAGACGGGAGGAAAGAAGGGAGUu---

47 0,1

-------AAGACGGGAGGAAAGAAGGGAGUuu--

36 0,1

-------AAGACGGGAGGAAAGAAGGGAGUG---

5 0,2

-------AAGACGGGAGGAAAGAAGGGAGUGu--

2 0,2

-------AAGACGGGAGGAAAGAAGGGAGUGG--

15 0,3

--------AGACGGGAGGAAAGAAGG--

26 1,-3

--------AGACGGGAGGAAAGAAGGG---

61 1,-2

--------AGACGGGAGGAAAGAAGGGA--

77 1,-1

--------AGACGGGAGGAAAGAAGGGAa---

5 1,-1

--------AGACGGGAGGAAAGAAGGGAaa--

3 1,-1

--------AGACGGGAGGAAAGAAGGGAu---

2 1,-1

--------AGACGGGAGGAAAGAAGGGAG---

166 1,0

--------AGACGGGAGGAAAGAAGGGAGU--

136 1,1

--------AGACGGGAGGAAAGAAGGGAGUa---

2 1,1

--------AGACGGGAGGAAAGAAGGGAGUu---

6 1,1

--------AGACGGGAGGAAAGAAGGGAGUG---

35 1,2

--------AGACGGGAGGAAAGAAGGGAGUGG--

107 1,3

--------AGACGGGAGGAAAGAAGGGAGUGGU---

6 1,4

---------GACGGGAGGAAAGAAGGGAG--

2 2,0

---------GACGGGAGGAAAGAAGGGAGU---

2 2,1

--UCACUCCUCUCCUCCCGU-----------

2 0,-3

--UCACUCCUCUCCUCCCGUCUau-------

2 0,-1

--UCACUCCUCUCCUCCCGUCUU--------

3 0,0

--UCACUCCUCUCCUCCCGUCUUa-------

3 0,0

--UCACUCCUCUCCUCCCGUCUUaa------

2 0,0

--UCACUCCUCUCCUCCCGUCUUau------

3 0,0

--UCACUCCUCUCCUCCCGUCUUu-------

10 0,0

--UCACUCCUCUCCUCCCGUCUUuu------

2 0,0

--UCACUCCUCUCCUCCCGUCUUC-------

7 0,1

---CACUCCUCUCCUCCCGUC----------

3 1,-2

---CACUCCUCUCCUCCCGUCUUCa------

2 1,1

---CACUCCUCUCCUCCCGUCUUCU------

15 1,2

>hsa-mir-484 5p:8-29 SRX018958

agccucgUCAGGCUCAGUCCCCUCCCGAUaaaccccuaaauagggacuuucccgggggugacccuggcuuuuuuggcg

.(((..((((((....(((.(((((((..(((.((((....))))..)))..))))))).)))))))))......))).

-------UCAGGCUCAGUCCCCUCC---

7 0,-4

-------UCAGGCUCAGUCCCCUCCC--

6 0,-3

-------UCAGGCUCAGUCCCCUCCCG---

3 0,-2

-------UCAGGCUCAGUCCCCUCCCGuu---

4 0,-2

-------UCAGGCUCAGUCCCCUCCCGA--

31 0,-1

-------UCAGGCUCAGUCCCCUCCCGAa---

19 0,-1

-------UCAGGCUCAGUCCCCUCCCGAaa--

10 0,-1

-------UCAGGCUCAGUCCCCUCCCGAU---

100 0,0

-------UCAGGCUCAGUCCCCUCCCGAUu--

23 0,0

-------UCAGGCUCAGUCCCCUCCCGAUuu---

2 0,0

-------UCAGGCUCAGUCCCCUCCCGAUA--

24 0,1

-------UCAGGCUCAGUCCCCUCCCGAUAu---

16 0,1

-------UCAGGCUCAGUCCCCUCCCGAUAA---

9 0,2

---------AGGCUCAGUCCCCUCCCGAau--

2 2,-1

---------AGGCUCAGUCCCCUCCCGAU---

3 2,0

>hsa-mir-485 5p:9-30;3p:46-67 SRX018958

acuuggagAGAGGCUGGCCGUGAUGAAUUCgauucaucaaagcgaGUCAUACACGGCUCUCCUCUCUuuuagu

(((..((((((((..((((((((((.((((...........)))))))).)))))))..))))))))..)))

--------AGAGGCUGGCCGUGAUGAA-- 2

0,-3

--------AGAGGCUGGCCGUGAUGAAau-- 6

0,-3

--------AGAGGCUGGCCGUGAUGAAU--- 4

0,-2

--------AGAGGCUGGCCGUGAUGAAUa-- 15

0,-2

--------AGAGGCUGGCCGUGAUGAAUU--- 147

0,-1
--------AGAGGCUGGCCGUGAUGAAUUa-- 2
0,-1
--------AGAGGCUGGCCGUGAUGAAUUu-- 15
0,-1
--------AGAGGCUGGCCGUGAUGAAUUC-- 43
0,0
--------AGAGGCUGGCCGUGAUGAAUUCa--- 2
0,0
--------AGAGGCUGGCCGUGAUGAAUUCu--- 3
0,0
--------AGAGGCUGGCCGUGAUGAAUUCG--- 52
0,1
--------AGAGGCUGGCCGUGAUGAAUUCGu-- 10
0,1
--------AGAGGCUGGCCGUGAUGAAUUCGA-- 3
0,2
---GUCAUACACGGCUCUCCUC--------- 3
0,-3
---GUCAUACACGGCUCUCCUCU-------- 2
0,-2
---GUCAUACACGGCUCUCCUCUC------- 15
0,-1
---GUCAUACACGGCUCUCCUCUCa------ 7
0,-1
---GUCAUACACGGCUCUCCUCUCU------ 71
0,0
---GUCAUACACGGCUCUCCUCUCUa----- 3
0,0
---GUCAUACACGGCUCUCCUCUCUau---- 4
0,0
---GUCAUACACGGCUCUCCUCUCUU----- 2
0,1

>hsa-mir-486 5p:4-25;3p:46-66 SRX018958
gcaUCCUGUACUGAGCUGCCCCGAGgcccuucaugcugcccagcuCGGGGCAGCUCAGUACAGGAUac
..((((((((((((((((((((((((..........)))....)))))))))))))))))))))))..
---UCCUGUACUGAGCUGCCC-- 26 0,-4

---UCCUGUACUGAGCUGCCCC--   62 0,-3
---UCCUGUACUGAGCUGCCCCG---   16 0,-2
---UCCUGUACUGAGCUGCCCCGA--   131 0,-1
---UCCUGUACUGAGCUGCCCCGAa---   16 0,-1
---UCCUGUACUGAGCUGCCCCGAu---   2   0,-1
---UCCUGUACUGAGCUGCCCCGAG---   343 0,0
---UCCUGUACUGAGCUGCCCCGAGa--   83 0,0
---UCCUGUACUGAGCUGCCCCGAGaa---   4   0,0
---UCCUGUACUGAGCUGCCCCGAGau---   2   0,0
---UCCUGUACUGAGCUGCCCCGAGu--   10 0,0
-----CUGUACUGAGCUGCCCCGAG---   3   2,0
-----CUGUACUGAGCUGCCCCGAGa--   2   2,0
---CGGGGCAGCUCAGUACAGGAUA-   2   0,1

>hsa-mir-487a   3p:49-70   SRX018958
gguacuugaagaggugguuaucccugcuguguucgcuuaauuuaugacgAAUCAUACAGGGACAUCCAGUUuuucaguauc
(((((.(((((.(((...((((((.((((((((.(((.....))))))).))))))))))...))).))))).))))))
---AAUCAUACAGGGACAUCCAGUU----------
    6   0,0
---AAUCAUACAGGGACAUCCAGUUU---------
    3   0,1

>hsa-mir-487b   3p:51-72   SRX018958
uugguacuuggagaggugguuaucccuguccuguucguuuugcucaugucgAAUCGUACAGGGUCAUCCACUUuuucagua
ucaa
.((((((..((((((((....((((((.(.(((((...........))))).).))))))....))))))))..))))
))).
---AAUCGUACAGGGUCAUCCAC----------
----   5   0,-2
---AAUCGUACAGGGUCAUCCACU---------
----   87 0,-1
---AAUCGUACAGGGUCAUCCACUU--------
----   42 0,0
---AAUCGUACAGGGUCAUCCACUUU-------
----   3   0,1
---AAUCGUACAGGGUCAUCCACUUUU------
----   2   0,2
--AUCGUACAGGGUCAUCCACUU--------

----   5   1,0
---AUCGUACAGGGUCAUCCACUUU-------
----   7   1,1
--UCGUACAGGGUCAUCCACUU--------
----   2   2,0
--UCGUACAGGGUCAUCCACUUau------
----   2   2,0
--UCGUACAGGGUCAUCCACUUU-------
----   3   2,1
--UCGUACAGGGUCAUCCACUUUa------
----   2   2,1

>hsa-mir-488   mi:14-34;MA:52-72   SRX018958
gagaaucaucucuCCCAGAUAAUGGCACUCUCAAacaaguuuccaaauuguUUGAAAGGCUAUUUCUUGGUCagaugacu
cuc
((((.((((((..((.(((.((((((.((.((((((((.........)))))))).))))))))))).))..)))))).)
)))
-------------CCCAGAUAAUGGCACUCUCAAAC--
--- 3   0,2

>hsa-mir-489   3p:52-73   SRX018958
guggcagcuugguggucguaugugugacgccauuuacuugaaccuuuaggaGUGACAUCACAUAUACGGCAGCuaaacug
cuac
(((((((.(((((.(((((((((((((.(.((((..(((((....))))))))).).))))))))))))).))))).)))
))))
---GUGACAUCACAUAUACGGCAGC-------
----   4   0,0

>hsa-mir-490   5p:39-58;3p:76-97   SRX018958
uggaggccuugcugguuuggaaaguucauuguucgacaCCAUGGAUCUCCAGGUGGGUcaaguuuagagaugcacCAACC
UGGAGGACUCCAUGCUGuugagcuguucacaagcagcggacacuucca
.(((((..((((((.(((((((........((((((((.(((((((((((((((.(((...(((....)))..))).)))
))))))..)))))).))))))))..))).))))))))))...))))).
---CAACC
UGGAGGACUCCAUG----------------------------------   7   0,-3
---CAACC
UGGAGGACUCCAUGC---------------------------------   9   0,-2
---CAACC

UGGAGGACUCCAUGCU-------------------------------- 21 0,-1
--CAACC
UGGAGGACUCCAUGCUu------------------------------- 6 0,-1
--CAACC
UGGAGGACUCCAUGCUG------------------------------- 38 0,0
--CAACC
UGGAGGACUCCAUGCUGa------------------------------ 33 0,0
--CAACC
UGGAGGACUCCAUGCUGaa----------------------------- 2 0,0
--CAACC
UGGAGGACUCCAUGCUGU------------------------------ 164 0,1
--CAACC
UGGAGGACUCCAUGCUGUa----------------------------- 8 0,1
--CAACC
UGGAGGACUCCAUGCUGUU----------------------------- 11 0,2
---AACC
UGGAGGACUCCAUGCUGU------------------------------ 3 1,1

>hsa-mir-491 5p:16-37;3p:50-71 SRX018958
uugacuuagcuggguAGUGGGGAACCCUUCCAUGAGGaguagaacacucCUUAUGCAAGAUUCCCUUCUACcuggcuggg
uugg
(..(((((((..(((((.((((((..(((.((((((((((.....)))))))))).))).)))))).))))).))))))
)..)
---------------AGUGGGGAACCCUUCCAUGAGa---
---- 4 0,-1
---------------AGUGGGGAACCCUUCCAUGAGG---
---- 2 0,0
---------------AGUGGGGAACCCUUCCAUGAGGA--
---- 15 0,1

>hsa-mir-493 mi:16-37;MA:57-78 SRX018958
cuggccuccagggcuUUGUACAUGGUAGGCUUUCAUUcauucguuugcacauucggUGAAGGUCUACUGUGUGCCAGGcc
cugugccag
(((((...((((((((.((((((((((((((((((((.....((....))....))))))))))))))))))).)))))
))).)))))
---------------UUGUACAUGGUAGGCUUUCA---
--------- 2 0,-2
---------------UUGUACAUGGUAGGCUUUCAU--

---------   5   0,-1
---------------UUGUACAUGGUAGGCUUUCAUU---
---------   19   0,0
---------------UUGUACAUGGUAGGCUUUCAUUa--
---------   2   0,0
--UGAAGGUCUACUGUGUGCC-----
---------   2   0,-3
--UGAAGGUCUACUGUGUGCCAa---
---------   2   0,-2
--UGAAGGUCUACUGUGUGCCAG---
---------   2   0,-1
--UGAAGGUCUACUGUGUGCCAGa--
---------   3   0,-1

>hsa-mir-494   3p:48-69   SRX018958
gauacucgaaggagagguuguccguguugucuucucuuuauuuaugaUGAAACAUACACGGGAAACCUCuuuuuuaguau
c
(((((.((((((((((((..((((((((((.(((((.........)).)))))).))))))..)))))))))))))))))
)
---UGAAACAUACACGGGAAACCU------------
-    7   0,-1
---UGAAACAUACACGGGAAACCUC-----------
-    17   0,0
---UGAAACAUACACGGGAAACCUCa----------
-    2   0,0
---UGAAACAUACACGGGAAACCUCU----------
-    89   0,1
--GAAACAUACACGGGAAACCU------------
-    3   1,-1
--GAAACAUACACGGGAAACCUC-----------
-    2   1,0
--GAAACAUACACGGGAAACCUCU----------
-    2   1,1

>hsa-mir-495   3p:50-71   SRX018958
ugguaccugaaaagaaguugcccauguuauuuucgcuuuauaugugacgAAACAAACAUGGUGCACUUCUUuuucgguau
ca
.(((((.((((((((((.((((((((((..((((((.........).)))))..)))))))).))))))))))))).)))))

).
---AAACAAACAUGGUGCACUU------------
-- 9   0,-3
---AAACAAACAUGGUGCACUUuu----------
-- 2   0,-3
---AAACAAACAUGGUGCACUUCU----------
-- 15 0,-1
---AAACAAACAUGGUGCACUUCUU---------
-- 145 0,0
---AAACAAACAUGGUGCACUUCUUa--------
-- 12 0,0
---AAACAAACAUGGUGCACUUCUUU--------
-- 158 0,1
--AACAAACAUGGUGCACUUCU----------
-- 5   1,-1
--AACAAACAUGGUGCACUUCUU---------
-- 5   1,0
--AACAAACAUGGUGCACUUCUUa--------
-- 6   1,0
--AACAAACAUGGUGCACUUCUUau-------
-- 2   1,0
--AACAAACAUGGUGCACUUCUUU--------
-- 30 1,1
---ACAAACAUGGUGCACUUCUUUU-------
-- 2   2,2

>hsa-mir-496   3p:56-77   SRX018958
cccaagucagguacucgaauggagguuguccaugguguguucauuuuauuuaugaUGAGUAUUACAUGGCCAAUCUCcuu
ucgguacucaauucuucuuggg
((((((...(((((.((((.((((((((.(((((..(((((((((........)))))))))..))))).)))))))).)
)))))))........))))))
---UGAGUAUUACAUGGCCAAUCU----
---------------------- 3   0,-1
---AGUAUUACAUGGCCAAUCUCC--
---------------------- 3   2,1

>hsa-mir-497   MA:24-44;mi:64-85   SRX018958
ccaccccgguccugcucccgccccCAGCAGCACACUGUGGUUUGUacggcacuguggccacgucCAAACCACACUGUGGUG

UUAGAgcgaggugggggaggcaccgccgagg
((.(..(((((((.((((((((((((((.((((.(((((((((((((.(((((......)))..)).))))))))).)))).))
)).....).)))))))))))).))))..).))
----------------------CCAGCAGCACACUGUGGUUUGU------------------------------------
--------------------------------	3	-1,0
-----------------------CAGCAGCACACUGUGGUU---------------------------------------
--------------------------------	2	0,-3
-----------------------CAGCAGCACACUGUGGUUU--------------------------------------
--------------------------------	62	0,-2
-----------------------CAGCAGCACACUGUGGUUUG-------------------------------------
--------------------------------	35	0,-1
-----------------------CAGCAGCACACUGUGGUUUGU------------------------------------
--------------------------------	163	0,0
-----------------------CAGCAGCACACUGUGGUUUGUA-----------------------------------
--------------------------------	85	0,1
-----------------------CAGCAGCACACUGUGGUUUGUAa----------------------------------
--------------------------------	37	0,1
-----------------------CAGCAGCACACUGUGGUUUGUAu----------------------------------
--------------------------------	30	0,1
------------------------AGCAGCACACUGUGGUUU--------------------------------------
--------------------------------	25	1,-2
------------------------AGCAGCACACUGUGGUUUG-------------------------------------
--------------------------------	7	1,-1
------------------------AGCAGCACACUGUGGUUUGU------------------------------------
--------------------------------	20	1,0
------------------------AGCAGCACACUGUGGUUUGUA-----------------------------------
--------------------------------	21	1,1
------------------------AGCAGCACACUGUGGUUUGUAa----------------------------------
--------------------------------	16	1,1
------------------------AGCAGCACACUGUGGUUUGUAu----------------------------------
--------------------------------	4	1,1
------------------------AGCAGCACACUGUGGUUUGUAC----------------------------------
--------------------------------	20	1,2
------------------------AGCAGCACACUGUGGUUUGUACa---------------------------------
--------------------------------	3	1,2

>hsa-mir-499	5p:33-53;3p:70-91	SRX018958
gcccuguccccugugccuugggcgggcggcugUUAAGACUUGCAGUGAUGUUUaacuccucuccacgugAACAUCACAGC

AAGUCUGUGCUgcuucccgucccuacgcugccugggcagggu
(((((((((.....((...(((((((.(((.((..((((((((.((((((((((.............)))))))))).))
))))))..)).))).)))).)))...)).....)))))))))
--------------------------------UUAAGACUUGCAGUGAUGU-----------------------------
-- 13 0,-2
--------------------------------UUAAGACUUGCAGUGAUGUU----------------------------
-- 12 0,-1
--------------------------------UUAAGACUUGCAGUGAUGUUa---------------------------
-- 2 0,-1
--------------------------------UUAAGACUUGCAGUGAUGUUaa--------------------------
-- 2 0,-1
--------------------------------UUAAGACUUGCAGUGAUGUUU---------------------------
-- 404 0,0
--------------------------------UUAAGACUUGCAGUGAUGUUUu--------------------------
-- 5 0,0
--------------------------------UUAAGACUUGCAGUGAUGUUUA--------------------------
-- 195 0,1
--------------------------------UUAAGACUUGCAGUGAUGUUUAu-------------------------
-- 2 0,1
--------------------------------UUAAGACUUGCAGUGAUGUUUAA-------------------------
-- 15 0,2
---------------------------------UAAGACUUGCAGUGAUGUUU---------------------------
-- 4 1,0

>hsa-mir-500a MA:13-35;mi:52-73 SRX018958
gcucccccucucUAAUCCUUGCUACCUGGGUGAGAgugcugucugaaugcaAUGCACCUGGGCAAGGAUUCUGagagcga
gagc
((((.(.(((((.(((((((((...(..((((....(((.........)))...))))..))))))))))..))))).).
))))
---AAUGCACCUGGGCAAGGA-----------
---- 21 -1,-5
---AAUGCACCUGGGCAAGGAU----------
---- 12 -1,-4
---AAUGCACCUGGGCAAGGAUa---------
---- 4 -1,-4
---AAUGCACCUGGGCAAGGAUU---------
---- 59 -1,-3
---AAUGCACCUGGGCAAGGAUUC--------

----   5   -1,-2
---AUGCACCUGGGCAAGGAU-----------
----   45   0,-4
---AUGCACCUGGGCAAGGAUa----------
----   22   0,-4
---AUGCACCUGGGCAAGGAUU----------
----   224 0,-3
---AUGCACCUGGGCAAGGAUUC---------
----   15   0,-2
---AUGCACCUGGGCAAGGAUUCU--------
----   21   0,-1
---AUGCACCUGGGCAAGGAUUCUGu------
----   5   0,0
---AUGCACCUGGGCAAGGAUUCUGA------
----   25   0,1
---AUGCACCUGGGCAAGGAUUCUGAa-----
----   2   0,1

>hsa-mir-500b   5p:11-28   SRX018958
cccccucucuAAUCCUUGCUACCUGGGUgagagugcuuucugaaugcagugcacccaggcaaggauucugcaaggggga
(((((..(.(((((((((...(((((((..(.(((.........))).).)))))))))))))))..)..)))))).
---AGUGCACCCAGGCAAGGA--------------
    2
---AGUGCACCCAGGCAAGGAUU------------
    2

>hsa-mir-501   5p:14-35;3p:51-72   SRX018958
gcucuuccucucuAAUCCUUUGUCCCUGGGUGAGAgugcuuucugaaugcAAUGCACCCGGGCAAGGAUUCUgagagggu
gagc
((((.(((((((.(((((((.(.(((.(((((....(((.........)))...)))))))))))))))..))))))).
))))
--AAUGCACCCGGGCAAGGAUa----------
----   2   0,-3
--AAUGCACCCGGGCAAGGAUU----------
----   21   0,-2
--AAUGCACCCGGGCAAGGAUUC---------
----   2   0,-1
--AAUGCACCCGGGCAAGGAUUCU--------

----   6   0,0
--AAUGCACCCGGGCAAGGAUUCUu-------
----   2   0,0
--AAUGCACCCGGGCAAGGAUUCUG-------
----   2   0,1

>hsa-mir-502   5p:16-36;3p:52-73   SRX018958
ugcucccccucucuaAUCCUUGCUAUCUGGGUGCUAgugcuggcucaaugcAAUGCACCUGGGCAAGGAUUCAgagaggg
ggagcu
.((((((((((((((((((((...(..(((((...(((.........)))..)))))..)))))))))).))))))))
))))).
--------------AAUCCUUGCUAUCUGGGUGCU---
------   2   -1,-1
---AAUGCACCUGGGCAAGGA-----------
------   21   0,-4
---AAUGCACCUGGGCAAGGAU----------
------   12   0,-3
---AAUGCACCUGGGCAAGGAUa---------
------   4   0,-3
---AAUGCACCUGGGCAAGGAUU---------
------   59   0,-2
---AAUGCACCUGGGCAAGGAUUC--------
------   5   0,-1
---AAUGCACCUGGGCAAGGAUUCA-------
------   7   0,0
---AAUGCACCUGGGCAAGGAUUCAa------
------   4   0,0
---AAUGCACCUGGGCAAGGAUUCAu------
------   5   0,0
---AAUGCACCUGGGCAAGGAUUCAG------
------   20   0,1
---AAUGCACCUGGGCAAGGAUUCAGu-----
------   2   0,1
--AUGCACCUGGGCAAGGAU----------
------   45   1,-3
--AUGCACCUGGGCAAGGAUa---------
------   22   1,-3
--AUGCACCUGGGCAAGGAUU---------

------ 224 1,-2
--AUGCACCUGGGCAAGGAUUC--------
------ 15 1,-1

>hsa-mir-503 5p:6-28 SRX018958
ugcccUAGCAGCGGGAACAGUUCUGCAGugagcgaucggugcucuggggguauuguuuccgcugccagggua
(((((.((((((((((((((.((.((..(((((.....))))))).)).))))))))))))))).))))))
-----UAGCAGCGGGAACAGUUC--- 533 0,-5
-----UAGCAGCGGGAACAGUUCa-- 16 0,-5
-----UAGCAGCGGGAACAGUUCaa--- 40 0,-5
-----UAGCAGCGGGAACAGUUCau--- 2 0,-5
-----UAGCAGCGGGAACAGUUCU-- 440 0,-4
-----UAGCAGCGGGAACAGUUCUa--- 24 0,-4
-----UAGCAGCGGGAACAGUUCUaa-- 181 0,-4
-----UAGCAGCGGGAACAGUUCUau-- 5 0,-4
-----UAGCAGCGGGAACAGUUCUG--- 60 0,-3
-----UAGCAGCGGGAACAGUUCUGa-- 6 0,-3
-----UAGCAGCGGGAACAGUUCUGaa--- 18 0,-3
-----UAGCAGCGGGAACAGUUCUGCA--- 8 0,-1
-----UAGCAGCGGGAACAGUUCUGCAa-- 6 0,-1
-----UAGCAGCGGGAACAGUUCUGCAuu--- 4 0,-1
-----UAGCAGCGGGAACAGUUCUGCAG-- 77 0,0
-----UAGCAGCGGGAACAGUUCUGCAGa--- 3 0,0
------AGCAGCGGGAACAGUUCUG--- 4 1,-3
------AGCAGCGGGAACAGUUCUGCAa-- 3 1,-1
------AGCAGCGGGAACAGUUCUGCAG-- 11 1,0
------AGCAGCGGGAACAGUUCUGCAGU--- 2 1,1

>hsa-mir-504 5p:13-34 SRX018958
gcugcuguugggAGACCCUGGUCUGCACUCUAUCuguauucuuacugaagggagugcagggcaggguuucccauacagag
ggc
(((.((((((((((((((((.((((((((((.((.(((....))).))..))))))))))).)))))))))))).))))..
)))
------------AGACCCUGGUCUGCACUCUAU---
--- 30 0,-1
------------AGACCCUGGUCUGCACUCUAUa--
--- 9 0,-1
------------AGACCCUGGUCUGCACUCUAUu--

--- 3 0,-1
------------AGACCCUGGUCUGCACUCUAUC--
--- 5 0,0
------------AGACCCUGGUCUGCACUCUAUCa---
--- 8 0,0
------------AGACCCUGGUCUGCACUCUAUCU---
--- 2 0,1
-------------GACCCUGGUCUGCACUCUAU---
--- 2 1,-1
-------------GACCCUGGUCUGCACUCUAUa--
--- 2 1,-1

>hsa-mir-505 mi:15-36;MA:51-72 SRX018958
gaugcacccaguggGGGAGCCAGGAAGUAUUGAUGUuucugccaguuuagCGUCAACACUUGCUGGUUUCCUcucuggag
cauc
(((((..((((.(((((((((((.((((.((((((((..........)))))))).)))).)))))))))))).)))).)
))))
--------------GGGAGCCAGGAAGUAUUG--
---- 3 0,-4
--------------GGGAGCCAGGAAGUAUUGAU--
---- 22 0,-2
--------------GGGAGCCAGGAAGUAUUGAUa---
---- 11 0,-2
--------------GGGAGCCAGGAAGUAUUGAUaa--
---- 3 0,-2
--------------GGGAGCCAGGAAGUAUUGAUu---
---- 5 0,-2
--------------GGGAGCCAGGAAGUAUUGAUG---
---- 63 0,-1
--------------GGGAGCCAGGAAGUAUUGAUGa--
---- 10 0,-1
--------------GGGAGCCAGGAAGUAUUGAUGU--
---- 281 0,0
--------------GGGAGCCAGGAAGUAUUGAUGUa---
---- 27 0,0
--------------GGGAGCCAGGAAGUAUUGAUGUau--
---- 2 0,0
--------------GGGAGCCAGGAAGUAUUGAUGUU---

---- 822 0,1
--------------GGGAGCCAGGAAGUAUUGAUGUUa--
---- 36 0,1
--------------GGGAGCCAGGAAGUAUUGAUGUUU--
---- 200 0,2
--------------GGGAGCCAGGAAGUAUUGAUGUUUu---
---- 3 0,2
---------------GGAGCCAGGAAGUAUUGAUGUU---
---- 3 1,1
--CGUCAACACUUGCUGGUUUCC---------
---- 4 0,-1
--CGUCAACACUUGCUGGUUUCCU--------
---- 7 0,0
---GUCAACACUUGCUGGUUUC----------
---- 2 1,-2
---GUCAACACUUGCUGGUUUCC---------
---- 2 1,-1
---GUCAACACUUGCUGGUUUCCU--------
---- 31 1,0
---GUCAACACUUGCUGGUUUCCUu-------
---- 2 1,0
---GUCAACACUUGCUGGUUUCCUC-------
---- 12 1,1
---GUCAACACUUGCUGGUUUCCUCU------
---- 7 1,2
--UCAACACUUGCUGGUUUCC---------
---- 2 2,-1
--UCAACACUUGCUGGUUUCCU--------
---- 4 2,0
--UCAACACUUGCUGGUUUCCUC-------
---- 5 2,1
--UCAACACUUGCUGGUUUCCUCU------
---- 3 2,2

>hsa-mir-509-3 5p:10-31;3p:44-65 SRX018958
gugguacccUACUGCAGACGUGGCAAUCAUGuauaauuaaaaaUGAUUGGUACGUCUGUGGGUAGaguacugcau
(..((((.((((..((((((((.(((((((.((....))...))))))).))))))))..).))).))))..)..
---------UACUGCAGACGUGGCAAUCAUG-- 2

0,0

>hsa-mir-511-1 5p:16-36 SRX018958
caauagacacccaucGUGUCUUUUGCUCUGCAGUCAguaaauauuuuuuugugaauguguagcaaaagacagaauggugg
uccauug
((((.(((..((((..(((((((((((..(((.(((.((((......))))))).)))..)))))))))))..))))..)
)).))))
--------------CGUGUCUUUUGCUCUGCAGUCAu---
------- 2 -1,0
---------------GUGUCUUUUGCUCUGCAGUCA--
------- 20 0,0
---------------GUGUCUUUUGCUCUGCAGUCAGa--
------- 2 0,1
---UGAAUGUGUAGCAAAAGACAGA-------
------- 17
--GAAUGUGUAGCAAAAGACAGA-------
------- 2
--GAAUGUGUAGCAAAAGACAGAA------
------- 4
---AAUGUGUAGCAAAAGACA---------
------- 14
---AAUGUGUAGCAAAAGACAG--------
------- 8
---AAUGUGUAGCAAAAGACAGA-------
------- 182
---AAUGUGUAGCAAAAGACAGAu------
------- 2
---AAUGUGUAGCAAAAGACAGAA------
------- 109
---AAUGUGUAGCAAAAGACAGAAa-----
------- 8
---AAUGUGUAGCAAAAGACAGAAU-----
------- 45
---AAUGUGUAGCAAAAGACAGAAUa----
------- 3
---AAUGUGUAGCAAAAGACAGAAUu----
------- 4
--AUGUGUAGCAAAAGACAGAA------

------- 3
---AUGUGUAGCAAAAGACAGAAa-----
------- 2

>hsa-mir-511-2 5p:16-36 SRX018958
caauagacacccaucGUGUCUUUUGCUCUGCAGUCAguaaauauuuuuuugugaauguguagcaaaagacagaauggugg
uccauug
((((.(((..((((..(((((((((((..(((.(((.((((......))))))).)))..)))))))))))..))))..)
)).))))
--------------CGUGUCUUUUGCUCUGCAGUCAu---
------- 2 -1,0
---------------GUGUCUUUUGCUCUGCAGUCA---
------- 20 0,0
---------------GUGUCUUUUGCUCUGCAGUCAGa---------------------------------------
------- 2 0,1
--UGAAUGUGUAGCAAAAGACAGA-------
------- 17
---GAAUGUGUAGCAAAAGACAGA-------
------- 2
---GAAUGUGUAGCAAAAGACAGAA------
------- 4
--AAUGUGUAGCAAAAGACA---------
------- 14
--AAUGUGUAGCAAAAGACAG--------
------- 8
--AAUGUGUAGCAAAAGACAGA-------
------- 182
--AAUGUGUAGCAAAAGACAGAu------
------- 2
--AAUGUGUAGCAAAAGACAGAA------
------- 109
--AAUGUGUAGCAAAAGACAGAAa-----
------- 8
--AAUGUGUAGCAAAAGACAGAAU-----
------- 45
--AAUGUGUAGCAAAAGACAGAAUa----
------- 3
--AAUGUGUAGCAAAAGACAGAAUu----

------- 4
--AUGUGUAGCAAAAGACAGAA------
------- 3
--AUGUGUAGCAAAAGACAGAAa-----
------- 2

>hsa-mir-532   5p:20-41;3p:57-78   SRX018958
cgacuugcuuucucuccucCAUGCCUUGAGUGUAGGACCGUuggcaucuuaauuacCCUCCCACACCCAAGGCUUGCAaa
aaagcgagccu
.(.((((((((.......((.((((((.((((.(((..((..............))..))).)))).)))))).))....
)))))))).).
-------------------CAUGCCUUGAGUGUAGGAC--
----------- 3   0,-3
-------------------CAUGCCUUGAGUGUAGGACC---
----------- 4   0,-2
-------------------CAUGCCUUGAGUGUAGGACCG--
----------- 7   0,-1
-------------------CAUGCCUUGAGUGUAGGACCGa---------------------------------------
----------- 5   0,-1
-------------------CAUGCCUUGAGUGUAGGACCGU---------------------------------------
----------- 154 0,0
-------------------CAUGCCUUGAGUGUAGGACCGUa--------------------------------------
----------- 7   0,0
-------------------CAUGCCUUGAGUGUAGGACCGUU--------------------------------------
----------- 10 0,1
--------------------AUGCCUUGAGUGUAGGACCGU---------------------------------------
----------- 4   1,0
--CCUCCCACACCCAAGGCU------
----------- 3   0,-4
--CCUCCCACACCCAAGGCUUG----
----------- 10 0,-2
--CCUCCCACACCCAAGGCUUGC---
----------- 11 0,-1
--CCUCCCACACCCAAGGCUUGCA--
----------- 61 0,0
--CCUCCCACACCCAAGGCUUGCAu-
----------- 10 0,0
--CCUCCCACACCCAAGGCUUGCAuu

----------- 2   0,0
---CCUCCCACACCCAAGGCUUGCAA-
----------- 12   0,1
--CUCCCACACCCAAGGCUUGCA--
----------- 3   1,0

>hsa-mir-539   5p:9-30 SRX018958
auacuugaGGAGAAAUUAUCCUUGGUGUGUucgcuuuauuuaugaugaaucauacaaggacaauuucuuuugaguau
(((((..((((((((((.((((((.(((((((((.........).)))).))))))))))).))))))))))..)))))
--------GGAGAAAUUAUCCUUGGUGUGU---
    2   0,0
--AUCAUACAAGGACAAUUU-----------
    2
--AUCAUACAAGGACAAUUUCU---------
    3
--AUCAUACAAGGACAAUUUCUU--------
    2
--AUCAUACAAGGACAAUUUCUUU-------
    13
---AUACAAGGACAAUUUCUUUUa-----
    2
---AUACAAGGACAAUUUCUUUUU-----
    2
---AUACAAGGACAAUUUCUUUUUa----
    3
---AUACAAGGACAAUUUCUUUUUG----
    15
---AUACAAGGACAAUUUCUUUUUGA---
    4

>hsa-mir-542   5p:16-38;3p:53-74   SRX018958
cagaucucagacaucUCGGGGAUCAUCAUGUCACGAGAuaccagugugcacuUGUGACAGAUUGAUAACUGAAAggucug
ggagccacucaucuuca
....((((((((...((((..((((...((((((((((.(((....)))..)))))))))))...))))..))))...)))))
))).............
---------------UCGGGGAUCAUCAUGUCAC---
------------------   3   0,-4
---------------UCGGGGAUCAUCAUGUCACG--

---------------- 2 0,-3
--------------UCGGGGAUCAUCAUGUCACGAGA---
---------------- 4 0,0
---UUGUGACAGAUUGAUAAC-----------
---------------- 6 -1,-5
---UUGUGACAGAUUGAUAACU----------
---------------- 6 -1,-4
---UUGUGACAGAUUGAUAACUGAA-------
---------------- 2 -1,-1
---UUGUGACAGAUUGAUAACUGAAA------
---------------- 3 -1,0
--UGUGACAGAUUGAUAACU----------
---------------- 40 0,-4
--UGUGACAGAUUGAUAACUG---------
---------------- 2 0,-3
--UGUGACAGAUUGAUAACUGA--------
---------------- 8 0,-2
--UGUGACAGAUUGAUAACUGAA-------
---------------- 23 0,-1
--UGUGACAGAUUGAUAACUGAAA------
---------------- 17 0,0
--UGUGACAGAUUGAUAACUGAAAa-----
---------------- 8 0,0

>hsa-mir-543 3p:47-68 SRX018958
uacuuaaugagaaguugcccguguuuuuuucgcuuuauuugugacgAAACAUUCGCGGUGCACUUCUUuuucaguauc
((((.((.(((((((.(((((((.((((.((((.......)))).))))....))))).))))))))).)).))))..
--AAACAUUCGCGGUGCACUU-------------
  4 0,-3
--AAACAUUCGCGGUGCACUUu------------
  4 0,-3
--AAACAUUCGCGGUGCACUUC------------
  2 0,-2
--AAACAUUCGCGGUGCACUUCU-----------
  3 0,-1
--AAACAUUCGCGGUGCACUUCUU----------
  24 0,0
--AAACAUUCGCGGUGCACUUCUUa---------

7 0,0
--AAACAUUCGCGGUGCACUUCUUU---------
41 0,1

>hsa-mir-544 3p:55-76 SRX018958
auuuucaucaccuagggaucuuguuaaaaagcagauucugauucagggaccaagAUUCUGCAUUUUUAGCAAGUUCucaa
gugaugcuaau
.....((((((...((((.((((((((((((((((.(((..((....))...))).))))).))))))))))).))))..
)))))).....
------------------UCUUGUUAAAAAGCAGAUUCU---
----------- 2

>hsa-mir-548a-1 3p:61-82 SRX018958
ugcagggagguauuaaguuggugcaaaaguaauugugauuuuugccauuaaaaguaacgaCAAAACUGGCAAUUACUUUU
GCaccaaaccugguauu
..((((...........(((((((((((((((((((.(.(((((.(.(((....))).).))))).).))))))))))))
))))))).)))).....
--CAAAACUGGCAAUUACUUUU
----------------- 9 0,-2
--CAAAACUGGCAAUUACUUUU
G---------------- 10 0,-1
--CAAAACUGGCAAUUACUUUU
Ga--------------- 2 0,-1

>hsa-mir-548a-2 3p:61-82 SRX018958
ugugauguguauuagguuugugcaaaaguaauuggguuuuuugccguuaaaaguaauggCAAAACUGGCAAUUACUUUU
GCaccaaacuaauauaa
.......((((((((....(((((((((((((((.(((((..((((((((....)))))))))))))..)))))))))))
))))....)))))))).
--CAAAACUGGCAAUUACUUUU
----------------- 9 0,-2
--CAAAACUGGCAAUUACUUUU
G---------------- 10 0,-1
--CAAAACUGGCAAUUACUUUU
Ga--------------- 2 0,-1

>hsa-mir-548a-3 5p:25-46;3p:61-82 SRX018958
ccuagaauguuauuaggucggugcAAAAGUAAUUGCGAGUUUUACCauuacuuucaauggCAAAACUGGCAAUUACUUUU

GCaccaacguaauacuu
..........(((((.((.(((((((((((((((((.((((((.(((((......))))).)))))).))))))))))))
))))).)).)))))...
--CAAAACUGGCAAUUACUUUU
-----------------   9   0,-2
--CAAAACUGGCAAUUACUUUU
G----------------   10   0,-1
--CAAAACUGGCAAUUACUUUU
Ga---------------   2   0,-1

>hsa-mir-548aa-1   3p:55-79   SRX018958
cuuuauuagucuggugcaaaagaaacugugguuuuugccauuacuuuuacaggcAAAAACCACAAUUACUUUUGCACCAa
ccuaauauaacuuguuu
...((((((..(((((((((((.((.(((((((((((((............))))))))))))).)).))))))))))).
.)))))).........
--AAAAACCACAAUUACUUUUGCA----
-----------------   2   0,-3

>hsa-mir-548aa-2   3p:55-79   SRX018958
uuuuauuagguuggugcaaaagaaacugugguuuuugccauuacuuucaauggcAAAAACCACAAUUACUUUUGCACCAa
ccuaaaucuucccucuc
.....((((((((((((((((.((.(((((((((((((((((......))))))))))))))))).)).)))))))))))
))))).............
--AAAAACCACAAUUACUUUUGCA----
-----------------   2   0,-3

>hsa-mir-548b   5p:25-46;3p:61-82   SRX018958
cagacuauauauuuagguuggcgcAAAAGUAAUUGUGGUUUUGGCCuuuauuuucaauggCAAGAACCUCAGUUGCUUUU
GUgccaaccuaauacuu
............(((((((((((((((((((((((.((((((.(((............))).)))))).)))))))))))
))))))))))))).....
-----------------------CAAAAGUAAUUGUGGUUUUG-------------------------------------
-----------------   2   -1,-3
------------------------AAAAGUAAUUGUGGUUUU--------------------------------------
-----------------   3   0,-4
------------------------AAAAGUAAUUGUGGUUUUGGCa----------------------------------
-----------------   2   0,-1
------------------------AAAAGUAAUUGUGGUUUUGGCau---------------------------------

-----------------   3   0,-1
-------------------------AAAGUAAUUGUGGUUUUGGCaa---------------------------------
-----------------   2   1,-1
--------------------------AAGUAAUUGUGGUUUUGGCCUUU-------------------------------
-----------------   2   2,3
--CAAGAACCUCAGUUGCUUUU
G----------------   4   0,-1
--CAAGAACCUCAGUUGCUUUU
GU---------------   4   0,0

>hsa-mir-548c   5p:25-46;3p:61-82   SRX018958
cauuggcaucuauuagguuggugcAAAAGUAAUUGCGGUUUUUGCCauuacuuucaguagCAAAAAUCUCAAUUACUUUU
GCaccaacuuaauacuu
..........(((((((((((((((((((((((((.(((((((((...(((.....)))))))))))).)))))))))))
))))))))))))))...
------------------------AAAAGUAAUUGCGGUUUUU-------------------------------------
-----------------   2   0,-3
------------------------AAAAGUAAUUGCGGUUUUUG------------------------------------
-----------------   3   0,-2
------------------------AAAAGUAAUUGCGGUUUUUGC-----------------------------------
-----------------   5   0,-1
-------------------------AAAGUAAUUGCGGUUUUUGC-----------------------------------
-----------------   2   1,-1
--------------------------AAGUAAUUGCGGUUUUUGCCA---------------------------------
-----------------   2   2,1

>hsa-mir-548d-1 5p:25-46;3p:61-82   SRX018958
aaacaaguuauauuagguuggugcAAAAGUAAUUGUGGUUUUUGCCuguaaaaguaauggCAAAAACCACAGUUUCUUUU
GCaccagacuaauaaag
..........((((((.((((((((((((.((((((((((((((((............)))))))))))))))).)))))
))))))).))))))...
-----------------------CAAAAGUAAUUGUGGUUUUU-------------------------------------
-----------------   4   -1,-3
-----------------------CAAAAGUAAUUGUGGUUUUUGa-----------------------------------
-----------------   3   -1,-2
------------------------AAAAGUAAUUGUGGUUUU--------------------------------------
-----------------   3   0,-4
------------------------AAAAGUAAUUGUGGUUUUU-------------------------------------

----------------- 2 0,-3
------------------------AAAAGUAAUUGUGGUUUUUG------------------------------------
----------------- 2 0,-2
------------------------AAAAGUAAUUGUGGUUUUUGu-----------------------------------
----------------- 2 0,-2
------------------------AAAAGUAAUUGUGGUUUUUGuu----------------------------------
----------------- 6 0,-2
-------------------------AAAGUAAUUGUGGUUUUUGa-----------------------------------
----------------- 2 1,-2
-------------------------AAAGUAAUUGUGGUUUUUGCu----------------------------------
----------------- 3 1,-1

>hsa-mir-548d-2 5p:25-46;3p:61-82 SRX018958
gagagggaagauuuagguuggugcAAAAGUAAUUGUGGUUUUUGCCauugaaaguaauggCAAAAACCACAGUUUCUUUU
GCaccaaccuaauaaaa
............((((((((((((((((.(((((((((((((((((((((....)))))))))))))))))))).)))))
))))))))))))).....
-----------------------CAAAAGUAAUUGUGGUUUUU-------------------------------------
----------------- 4 -1,-3
-----------------------CAAAAGUAAUUGUGGUUUUUGa-----------------------------------
----------------- 3 -1,-2
------------------------AAAAGUAAUUGUGGUUUU--------------------------------------
----------------- 3 0,-4
------------------------AAAAGUAAUUGUGGUUUUU-------------------------------------
----------------- 2 0,-3
------------------------AAAAGUAAUUGUGGUUUUUG------------------------------------
----------------- 2 0,-2
------------------------AAAAGUAAUUGUGGUUUUUGu-----------------------------------
----------------- 2 0,-2
------------------------AAAAGUAAUUGUGGUUUUUGuu----------------------------------
----------------- 6 0,-2
-------------------------AAAGUAAUUGUGGUUUUUGa-----------------------------------
----------------- 2 1,-2
-------------------------AAAGUAAUUGUGGUUUUUGCu----------------------------------
----------------- 3 1,-1

>hsa-mir-548e 3p:53-74 SRX018958
uuauuagguugguacaaaagcaaucgcgguuuuugcuauuacuuuuaaaggcAAAAACUGAGACUACUUUUGCAccaacc

ugauagaa
((((((((((((((.((((((...((.(((((((((((.(((....))).))))))))))).))...)))))).)))))))
))))))..
---CAAAAACUGAGACUACUUUUG--------
--------	2	-1,-2
---CAAAAACUGAGACUACUUUUGC-------
--------	3	-1,-1
--AAAAACUGAGACUACUUU----------
--------	3	0,-4
--AAAAACUGAGACUACUUUU---------
--------	5	0,-3
--AAAAACUGAGACUACUUUUG--------
--------	6	0,-2
--AAAAACUGAGACUACUUUUGC-------
--------	29	0,-1
--AAAAACUGAGACUACUUUUGCu------
--------	2	0,-1
--AAAAACUGAGACUACUUUUGCA------
--------	59	0,0
--AAAAACUGAGACUACUUUUGCAa-----
--------	27	0,0
--AAAAACUGAGACUACUUUUGCAu-----
--------	4	0,0
--AAACUGAGACUACUUUUGCA------
--------	2	2,0
--AAACUGAGACUACUUUUGCACCAu--
--------	2	2,3

>hsa-mir-548f-2 3p:59-77	SRX018958
uaauaacuauuagguuggugcgaacauaauugcaguuuuuaucauuacuuuuaauggcAAAAACUGUAAUUACUUUUgca
ccaaccuaauauuuuagu
.......(((((((((((((((((..((((((((((((((..(((((....)))))..))))))))))))))..))))))
))))))))))).......
---CAAAAACUGUAAUUACUUUUGC-
------------------	4	-1,2

>hsa-mir-548f-3 3p:54-72	SRX018958
auuagguuggugcaaaccuaauugcaauuuuugcaguuuuuuuaaguaauugcAAAAACUGUAAUUACUUUUgcaccaac

cuaauac
((((((((((((((((..((((((((.(((((((((((.........))))))))))).))))))))..)))))))))))
)))))..
--CAAAAACUGUAAUUACUUUUGC------
------- 4   -1,2

>hsa-mir-548g   3p:54-75   SRX018958
aguuauuagauuagugcaaaaguaauugcaguuuuugcauuacguucuauggcAAAACUGUAAUUACUUUUGUACcaaca
uaauacuuc
...(((((..((.((((((((((((((((((((((.((.............)))))))))))))))))))))))).))..
)))))....
-----------------AAAAGUAAUUGCAGUUUU---
---------   2

>hsa-mir-548h-1 5p:21-42   SRX018958
ucuguccauuaggugggugcAAAAGUAAUCGCGGUUUUUGUCauuacuuuuaaugguaaaaacuggaauuacuuuugcac
ugaccuaauauuaagccagaua
((((.(.(((((((.((((((((((((((..((((((((..(((((....)))))..))))))))..)))))))))))))
).))))))).....).))))..
--------------------AAAAGUAAUCGCGGUUUUU---
--------------------- 2   0,-3
--------------------AAAAGUAAUCGCGGUUUUUGU---------------------------------------
--------------------- 5   0,-1
----------------------AAGUAAUCGCGGUUUUUG--
--------------------- 3   2,-2

>hsa-mir-548h-2 5p:16-37   SRX018958
guauuagguuggugcAAAAGUAAUCGCGGUUUUUGUCauuacuuucaauggcaaacaccacaauuacuuuugcaccaacc
uaauauaa
((((((((((((((((((((((((.(.(((.(((((((((......))))))))).))).).))))))))))))))))))
))))))..
---------------AAAAGUAAUCGCGGUUUUU--
--------   2   0,-3
---------------AAAAGUAAUCGCGGUUUUUGU--
--------   5   0,-1
-----------------AAGUAAUCGCGGUUUUUG---
--------   3   2,-2
---ACAAUUACUUUUGCACCAACu

u-------   2

>hsa-mir-548h-3 5p:29-50   SRX018958
ucugauucugcauguauuagguuggugcAAAAGUAAUCGCGGUUUUUGUCauugaaaguaauagcaaaaacugcaauuac
uuuugcaccaaccuaaaaguagucacugucuucagaua
((((..(((.....(((((((((((((((((((((((.((((((((((..((((....))))..))))))))))).)))))
))))))))))))))))..)))).........)))))..
----------------------------AAAAGUAAUCGCGGUUUUU---------------------------------
-------------------------------------- 2   0,-3
----------------------------AAAAGUAAUCGCGGUUUUUGU-------------------------------
-------------------------------------- 5   0,-1
------------------------------AAGUAAUCGCGGUUUUUG--------------------------------
-------------------------------------- 3   2,-2
--CAAAAACUGCAAUUAC
UU------------------------------------ 8
--CAAAAACUGCAAUUAC
UUU----------------------------------- 15
--CAAAAACUGCAAUUAC
UUUU---------------------------------- 69
--CAAAAACUGCAAUUAC
UUUUG--------------------------------- 35
--CAAAAACUGCAAUUAC
UUUUGa-------------------------------- 7
--CAAAAACUGCAAUUAC
UUUUGu-------------------------------- 10
--CAAAAACUGCAAUUAC
UUUUGC-------------------------------- 7
---AAAAACUGCAAUUAC
UUU----------------------------------- 14
---AAAAACUGCAAUUAC
UUUU---------------------------------- 39
---AAAAACUGCAAUUAC
UUUUa--------------------------------- 2
---AAAAACUGCAAUUAC
UUUUG--------------------------------- 30
---AAAAACUGCAAUUAC
UUUUGa-------------------------------- 3

>hsa-mir-548h-4 5p:17-38   SRX018958
gcuauuagguuggugcAAAAGUAAUCGCGGUUUUUGUCauuacuuuaauuacuuuacguuucauuaaugacaaaaaccgc
aauuacuuuugcaccaaccuaauacuugcua
(((((((((((((((((((((((((.((((((((((((((((....(((........)))....))))))))))))))))
.)))))))))))))))))))))))...))..
----------------AAAAGUAAUCGCGGUUUUU---
------------------------------- 2   0,-3
----------------AAAAGUAAUCGCGGUUUUUGU---
------------------------------- 5   0,-1
------------------AAGUAAUCGCGGUUUUUG--
------------------------------- 3   2,-2
--CAAAAACCGC
AAUUACUUUU--------------------- 3
---AAAAACCGC
AAUUACUUUU--------------------- 2

>hsa-mir-548j   5p:29-50   SRX018958
gggcagccagugaauaguuagcuggugcAAAAGUAAUUGCGGUCUUUGGUauuacuuucaguggcaaaaacugcauuacu
uuugcaccagccuacuagaacgcugaguucag
((((...(((((..((((..(((((((((((((((((.(((((.((((.((((......)))).)))).)))))))))))
)))))))))))..))))...))))).))))..
---------------------------CAAAAGUAAUUGCGGUCUUUG--------------------------------
--------------------------------   7   -1,-2
---------------------------CAAAAGUAAUUGCGGUCUUUGG-------------------------------
--------------------------------   2   -1,-1
---------------------------CAAAAGUAAUUGCGGUCUUUGGU------------------------------
--------------------------------   6   -1,0
----------------------------AAAAGUAAUUGCGGUCUUU---------------------------------
--------------------------------   15   0,-3
----------------------------AAAAGUAAUUGCGGUCUUUG--------------------------------
--------------------------------   3   0,-2
----------------------------AAAAGUAAUUGCGGUCUUUGG-------------------------------
--------------------------------   12   0,-1
----------------------------AAAAGUAAUUGCGGUCUUUGGU------------------------------
--------------------------------   22   0,0
----------------------------AAAAGUAAUUGCGGUCUUUGGUA-----------------------------
--------------------------------   5   0,1
-----------------------------AAAGUAAUUGCGGUCUUU---------------------------------

-------------------------------- 3 1,-3
--CAAAAACUGCAUUACU
UUU----------------------------- 5
--CAAAAACUGCAUUACU
UUUG---------------------------- 24
--CAAAAACUGCAUUACU
UUUGa--------------------------- 3
---AAAAACUGCAUUACU
UUUGCA-------------------------- 2

>hsa-mir-548k 5p:32-53 SRX018958
cuuuucucaaguauugcuguuagguuggugcAAAAGUACUUGCGGAUUUUGCUuuacuuuuaauggcaaaaaccgcaauu
auuuuugcuucaaccuaauaugaugcaaaauuggcu
..........((((((.(((((((((((.(((((((((.((((((.(((((((...........))))))).)))))).)
)))))))).)))))))))))))))))..........
-------------------------------AAAAGUACUUGCGGAUUU-------------------------------
------------------------------------ 2 0,-4
-------------------------------AAAAGUACUUGCGGAUUUU------------------------------
------------------------------------ 8 0,-3
-------------------------------AAAAGUACUUGCGGAUUUUGC----------------------------
------------------------------------ 4 0,-1
-------------------------------AAAAGUACUUGCGGAUUUUGCU---------------------------
------------------------------------ 20 0,0

>hsa-mir-548n 5p:10-31 SRX018958
agguuggugCAAAAGUAAUUGUGGAUUUUGUcguuaaaaauagcaaaacccgcaauuacuuuugcaccaaccuaa
((((((((((((((((((((((((.((((((..(.....)..)))))).))))))))))))))))))))))))..
---------CAAAAGUAAUUGUGGAUUUU-- 5
0,-2

>hsa-mir-548o 3p:87-108 SRX018958
uggugaaaauguguugauuguaaugguuccuauucugaucaauaaacaugguuugagccuaguuacaaugaucuaaaauu
cacgguCCAAAACUGCAGUUACUUUUGCaccaac
(((((.(((...((.(((((((.(((..((.(((..(((((...(((..(((....)))..)))....)))))...))).
...)).)))....))))))))).))).)))))..

------CCAAAACUGCAGUUACUUUU-------- 2 0,-2

------CCAAAACUGCAGUUACUUUUG------- 5 0,-1

------CCAAAACUGCAGUUACUUUUGa------ 3 0,-1

------CCAAAACUGCAGUUACUUUUGC------ 4 0,0

>hsa-mir-548q 5p:8-29 SRX018958
auauuagGCUGGUGCAAAAGUAAUGGCGGuuuuugccauuacuuuucauuuuuaccauuaaaaguaauggcaaaaagcau
gauuacuuuuucaccaaccu
.....(((.(((((.(((((((((.(.(.(((((((((((((((((.((.......)).))))))))))))))))).).)
.))))))))).))))).)))
-----AGGCUGGUGCAAAAGUAAUG---
-------------------- 2 -2,-4
-----AGGCUGGUGCAAAAGUAAUGGCa--
-------------------- 3 -2,-2

>hsa-mir-548s 3p:52-74 SRX018958
uugcugcaaaaauaauugcaguuuuugccauuauuuuuaauaauuauaauaAUGGCCAAAACUGCAGUUAUUUUugcacc
aa
.((.(((((((((((((((((((((.(((((((((............))))))))).))))))))))))))))))))).)
).
-----------AUAAUUGCAGUUUUUGCCAU---
-- 2

>hsa-mir-548t 5p:10-30 SRX018958
aggguggugCAAAAGUGAUCGUGGUUUUUGcaauuuuuuaaugacaaaaaccacaauuacuuuugcaccaaccu
(((.(((((((((((((((.((((((((((..(((....)))..)))))))))).))))))))))))))).)))
---AAAAACCACAAUUACUUUU---------- 2
---AAAAACCACAAUUACUUUUGCA------- 2

>hsa-mir-548u 3p:49-71 SRX018958
auuaggauggugcaaaaguaaugugguuuuuuucuuuacuuuuaauggCAAAGACUGCAAUUACUUUUGCGccaaccuaa
u
(((((.(((((((((((((((((..((((((.((.(((....))).)).))))))..).))))))))))))))).)))))
)
--CAAAGACUGCAAUUACUUUUGCG---------
- 4 0,0

>hsa-mir-548v 3p:49-70 SRX018958
aauacuagguuugagcaaaaguaauugcgguuuugccaucaugccaaaAGCUACAGUUACUUUUGCACCAgccuaauauu
((((.((((((.(.(((((((((((((..(((((((......))..)))))..))))))))))))).).)))))).))))
---AAGCUACAGUUACUUUUGCACC-----------
2 -1,-1
--AGCUACAGUUACUUUUGCACC-----------
3 0,-1
--AGCUACAGUUACUUUUGCACCA----------
7 0,0

>hsa-mir-548w 5p:10-32 SRX018958
gguuggugcAAAAGUAACUGCGGUUUUUGCCUuucaacauaauggcaaaacccacaauuacuuuugcaccaauc
((((((((((((((((.((.((.(((((((...........))))))).)).)).))))))))))))))))
---------AAAAGUAACUGCGGUUUUU-- 11
0,-4
---------AAAAGUAACUGCGGUUUUUG--- 4
0,-3

>hsa-mir-548x 3p:46-66 SRX018958
agguuagugcaaaaguaauugcaguuuuugcguuacuuucaaucgUAAAAACUGCAAUUACUUUCAcaccaaucu
(((((.(((..((((((((((((((((((((..........))))))))))))))))))))..))).)))))
----------AAAAGUAAUUGCAGUUUU--- 2
--AAAAACUGCAAUUACUUU----------- 14
1,-2

>hsa-mir-548z 3p:54-76 SRX018958
aaguauuaaguuggugcaaaaguaauugagauuuuugcuacugaaaguaauggCAAAAACCGCAAUUACUUUUGCAccaa
ccuaauagaugccaaug
...(((((.((((((((((((((((((.(.(((((((((.((....)).))))))))).).))))))))))))))))))
).))))).........
---CAAAAACCGCAAUUACUUUU-------
----------------- 3 0,-3
--AAAAACCGCAAUUACUUUU-------
----------------- 2 1,-3

>hsa-mir-550a-1 MA:22-44;mi:61-82 SRX018958
ugaugcuuugcuggcugguggcAGUGCCUGAGGGAGUAAGAGCCCuguuguuguaagauagUGUCUUACUCCCUCAGGCAC
AUcuccaacaagucucu

.((.((((.(.(((..(((...((((((((((((((((((.(.(((((.......))))).)))))))))))))))))))
))).))).))))).)).
---------------------AGUGCCUGAGGGAGUAAG---
-----------------   3   0,-5

>hsa-mir-550a-2 MA:22-44;mi:61-82   SRX018958
ugaugcuuugcuggcuggugcAGUGCCUGAGGGAGUAAGAGCCCuguuguugucagauagUGUCUUACUCCCUCAGGCAC
AUcuccagcgagucucu
.........(((.(((((.(..((((((((((((((((((.(.(((((.......))))).)))))))))))))))))))
..).))))).)))....
---------------------AGUGCCUGAGGGAGUAAG---
-----------------   3   0,-5

>hsa-mir-551b   mi:22-43;MA:61-81   SRX018958
agaugugcucuccuggcccauGAAAUCAAGCGUGGGUGAGACCuggugcagaacgggaagGCGACCCAUACUUGGUUUCA
Gaggcugugagaauaa
........((((.(((((..((((((((((..((((((...((((........))))....).)))))..))))))))))
..))))).))))....
--GCGACCCAUACUUGGUUU--
----------------   2   0,-3
--GCGACCCAUACUUGGUUUCA
----------------   4   0,-1
--GCGACCCAUACUUGGUUUCA
Gu--------------   7   0,0

>hsa-mir-556   5p:16-37;3p:55-76   SRX018958
gauaguaauaagaaaGAUGAGCUCAUUGUAAUAUGAGcuucauuuauacauuucAUAUUACCAUUAGCUCAUCUUUuuua
uuacuaccuucaaca
..(((((((((((((((((((((.((.((((((((((..............)))))))))).)).)))))))))))))))
)))))).........
--AUAUUACCAUUAGCUCAUCUU-----
--------------- 18 0,-1
--AUAUUACCAUUAGCUCAUCUUa----
--------------- 5   0,-1
--AUAUUACCAUUAGCUCAUCUUU----
--------------- 4   0,0

>hsa-mir-570   3p:60-81   SRX018958

cuagauaaguuauuaggugggugcaaagguaauugcaguuuuucccauuauuuuaauugCGAAAACAGCAAUUACCUUUG
Caccaaccugauggagu
.........(((((((((.(((((((((((((((((.((((((.(.((((...)))).).)))))).)))))))))))))
)))).)))))))))...
------------------------AAAGGUAAUUGCAGUUUU--------------------------------------
----------------- 2
------------------------AAAGGUAAUUGCAGUUUUUC------------------------------------
----------------- 2
------------------------AAAGGUAAUUGCAGUUUUUCC-----------------------------------
----------------- 3
------------------------AAAGGUAAUUGCAGUUUUUCCCA---------------------------------
----------------- 10
---CGAAAACAGCAAUUACCUUUG
----------------- 5 0,-1

>hsa-mir-574 5p:25-47;3p:61-82 SRX018958
gggaccugcgugggugcgggcgugUGAGUGUGUGUGUGAGUGUGUgucgcuccgggucCACGCUCAUGCACACACCCA
CAcgcccacacucagg
........(.((((((.(((((((((.(.((((((((((((((((..((......))..)))))))))))))))))))
))))))).)))))).)
------------------------UGAGUGUGUGUGUGAG--------------------------------------
---------------- 14 0,-5
------------------------UGAGUGUGUGUGUGAGU-------------------------------------
---------------- 22 0,-4
------------------------UGAGUGUGUGUGUGAGUG------------------------------------
---------------- 2 0,-3
------------------------UGAGUGUGUGUGUGAGUGU-----------------------------------
---------------- 4 0,-2
------------------------UGAGUGUGUGUGUGAGUGUGaa--------------------------------
---------------- 2 0,-1
------------------------UGAGUGUGUGUGUGAGUGUGU---------------------------------
---------------- 5 0,0
------------------------UGAGUGUGUGUGUGAGUGUGUG--------------------------------
---------------- 2 0,1
------------------------UGAGUGUGUGUGUGAGUGUGUGa-------------------------------
---------------- 2 0,1
------------------------UGAGUGUGUGUGUGAGUGUGUGU-------------------------------
---------------- 5 0,2

---CACGCUCAUGCACACACC--
----------------   22 0,-4
---CACGCUCAUGCACACACCC-
----------------   33 0,-3
---CACGCUCAUGCACACACCCA
----------------   76 0,-2
---CACGCUCAUGCACACACCCA
a---------------   3   0,-2
---CACGCUCAUGCACACACCCA
u---------------   3   0,-2
---CACGCUCAUGCACACACCCA
uu--------------   4   0,-2
---CACGCUCAUGCACACACCCA
C---------------   45 0,-1
---CACGCUCAUGCACACACCCA
Cu--------------   15 0,-1
---CACGCUCAUGCACACACCCA
CA--------------   206 0,0
---CACGCUCAUGCACACACCCA
CAa-------------   28 0,0
---CACGCUCAUGCACACACCCA
CAu-------------   32 0,0
---CACGCUCAUGCACACACCCA
CAuu------------   7   0,0
--ACGCUCAUGCACACACCCA
----------------   2   1,-2
--ACGCUCAUGCACACACCCA
C---------------   3   1,-1
--ACGCUCAUGCACACACCCA
CA--------------   9   1,0
--ACGCUCAUGCACACACCCA
CAu-------------   2   1,0

>hsa-mir-576   5p:16-37;3p:55-76   SRX018958
uacaauccaacgaggAUUCUAAUUUCUCCACGUCUUUgguaauaagguuuggcaAAGAUGUGGAAAAAUUGGAAUCcuca
uucgauugguuauaacca
..(((((.((.((((((((((((((.((((((((((((.(((......))).)))))))))))).)))))))))))))).
)).))))).........

---------------AUUCUAAUUUCUCCACGU---
------------------ 2 0,-4
---------------AUUCUAAUUUCUCCACGUCUU--
------------------ 6 0,-1
---------------AUUCUAAUUUCUCCACGUCUUU---
------------------ 10 0,0
---------------AUUCUAAUUUCUCCACGUCUUUG--
------------------ 2 0,1
--AAGAUGUGGAAAAAUUGG--------
------------------ 13 0,-4
--AAGAUGUGGAAAAAUUGGA-------
------------------ 5 0,-3
--AAGAUGUGGAAAAAUUGGAA------
------------------ 8 0,-2
--AAGAUGUGGAAAAAUUGGAAU-----
------------------ 23 0,-1
--AAGAUGUGGAAAAAUUGGAAUa----
------------------ 13 0,-1
--AAGAUGUGGAAAAAUUGGAAUC----
------------------ 6 0,0
--AAGAUGUGGAAAAAUUGGAAUCa---
------------------ 2 0,0
--AAGAUGUGGAAAAAUUGGAAUCC---
------------------ 13 0,1

>hsa-mir-579 3p:62-84 SRX018958
cauauuagguuaaugcaaaaguaaucgcgguuugugccagaugacgauuugaauuaauaaaUUCAUUUGGUAUAAACCGC
GAUUauuuuugcaucaac
........(((.((((((((((((((((((((((((((((((((..(((((......)))))))))))))))))))))))
)))))))))))))).)))
------------------------UCGCGGUUUGUGCCAGAUGACaa---------------------------------
------------------ 2
--AUUCAUUUGGUAUAAACCGC
GA---------------- 3 -1,-2

>hsa-mir-582 5p:16-38;3p:53-74 SRX018958
aucugugcucuuugaUUACAGUUGUUCAACCAGUUACUaaucuaacuaauugUAACUGGUUGAACAACUGAACCcaaagg
gugcaaaguagaaacauu

...((..((((((....(((((((((((((((((((..............))))))))))))))))))....))))))
)..)).............
---------------UACAGUUGUUCAACCAGUUAC--
------------------ 2   1,-1
---------------UACAGUUGUUCAACCAGUUACU---
------------------ 4   1,0
--UAACUGGUUGAACAACUGA---------
------------------ 3   0,-3
--UAACUGGUUGAACAACUGAA--------
------------------ 35 0,-2
--UAACUGGUUGAACAACUGAAC-------
------------------ 6   0,-1
--UAACUGGUUGAACAACUGAACC------
------------------ 4   0,0
--UAACUGGUUGAACAACUGAACCC-----
------------------ 4   0,1
---AACUGGUUGAACAACUGAA--------
------------------ 2   1,-2

>hsa-mir-584   5p:16-37   SRX018958
uagggugaccagccaUUAUGGUUUGCCUGGGACUGAGgaauuugcugggauaugucaguuccaggccaaccaggcugguu
ggucucccugaagcaac
(((((.(((((((((...(((((.((((((((((((.(.(((......))).).)))))))))))).)))))...)))))
)))).)))))......
---------------UUAUGGUUUGCCUGGGAC---
-----------------   2   0,-4
---------------UUAUGGUUUGCCUGGGACUGAa---
-----------------   2   0,-1

>hsa-mir-589   MA:24-45;mi:61-84   SRX018958
uccagccugugcccagcagccccUGAGAACCACGUCUGCUCUGAGcuggguacugccuguUCAGAACAAAUGCCGGUUCC
CAGAcgcugccagcuggcc
....(((...((...(((((..(((.(((((.(((.((.(((((((.(((.....)))))))))).)).)))..))))).
)))..)))))..)).))).
-----------------------UGAGAACCACGUCUGCUCU--------------------------------------
------------------- 2   0,-3
--UCAGAACAAAUGCCGGUUCC
CAG---------------- 2   0,-1

>hsa-mir-590 5p:16-37;3p:56-76 SRX018958
uagccagucagaaauGAGCUUAUUCAUAAAAGUGCAGuauggugaagucaaucugUAAUUUUAUGUAUAAGCUAGUcucu
gauugaaacaugcagca
....((((((((.((.(((((((.(((((((.(((((..(((.....)))..))))).))))))).))))))).)).)))
))))).............
---------------GAGCUUAUUCAUAAAAGU---
----------------- 3 0,-4
---------------GAGCUUAUUCAUAAAAGUG--
----------------- 8 0,-3
---------------GAGCUUAUUCAUAAAAGUGCA--
----------------- 14 0,-1
---------------GAGCUUAUUCAUAAAAGUGCAa---
----------------- 4 0,-1
---------------GAGCUUAUUCAUAAAAGUGCAG---
----------------- 51 0,0
--GUAAUUUUAUGUAUAAGCUAG-----
----------------- 7 -1,-1
--GUAAUUUUAUGUAUAAGCUAGU----
----------------- 11 -1,0
--GUAAUUUUAUGUAUAAGCUAGUa---
----------------- 3 -1,0
---UAAUUUUAUGUAUAAGCU-------
----------------- 24 0,-3
---UAAUUUUAUGUAUAAGCUA------
----------------- 2 0,-2
---UAAUUUUAUGUAUAAGCUAG-----
----------------- 2 0,-1
---UAAUUUUAUGUAUAAGCUAGU----
----------------- 7 0,0
---UAAUUUUAUGUAUAAGCUAGUu---
----------------- 2 0,0
---UAAUUUUAUGUAUAAGCUAGUC---
----------------- 4 0,1

>hsa-mir-597 5p:16-37 SRX018958
uacuuacucuacgugUGUGUCACUCGAUGACCACUGUgaagacaguaaaauguacagugguucucuuguggcucaagcgu
aauguagaguacugguc

.(((((((((((((.((.(((((..((.(((((((((....(((......)))))))))))).))..))))).)).....
.))))))))))..))).
---AGUGGUUCUCUUGUGGCUCAA----
-----------------   2
---AGUGGUUCUCUUGUGGCUCAAG---
-----------------   3

>hsa-mir-598   3p:61-82   SRX018958
gcuugaugaugcugcugaugcuggcggugaucccgauggugugagcuggaaaugggugcUACGUCAUCGUUGUCAUCGU
CAucaucaucauccgag
......................(((((((((..(((((..((.(((............)))))..)))))..))))))))
)................
--UACGUCAUCGUUGUCAUC--
-----------------   21   0,-4
--UACGUCAUCGUUGUCAUCG-
-----------------   4   0,-3
--UACGUCAUCGUUGUCAUCGU
-----------------   22   0,-2
--UACGUCAUCGUUGUCAUCGU
C----------------   6   0,-1
--UACGUCAUCGUUGUCAUCGU
CA---------------   27   0,0
--UACGUCAUCGUUGUCAUCGU
CAa--------------   5   0,0
--UACGUCAUCGUUGUCAUCGU
CAU--------------   36   0,1

>hsa-mir-616   mi:16-37;MA:56-77   SRX018958
uuagguaauuccuccACUCAAAACCCUUCAGUGACUUccaugacaugaaauaggaAGUCAUUGGAGGGUUUGAGCAGagg
aaugaccuguuuuaaaa
.(((((.(((((((..((((((.((((((((((((((((((........)).))))))))))))))))))))))..))))
))).)))))........
---------------ACUCAAAACCCUUCAGUGACUU---
-----------------   2   0,0
--AAGUCAUUGGAGGGUUUGAGCA----
-----------------   3   -1,-1

>hsa-mir-618   5p:16-38   SRX018958

cucuuguucacagccAAACUCUACUUGUCCUUCUGAGUguaauuacguacaugcaguagcucaggagacaagcagguuua
cccuguggaugagucuga
..((..(((((((..((((.((.((((((.((((((((.....(((((....)).))))))))))))))))).)))))).
..)))))))..)).....
---------------AAACUCUACUUGUCCUUCU--
------------------ 10 0,-4
---------------AAACUCUACUUGUCCUUCUGA---
------------------ 2   0,-2
---------------AAACUCUACUUGUCCUUCUGAG---
------------------ 2   0,-1

>hsa-mir-619   3p:61-84   SRX018958
cgcccaccucagccucccaaaaugcugggauuacaggcaugagccacugcggucgaccauGACCUGGACAUGUUUGUGCC
CAGUacugucaguuugcag
.((..((..(((..........(((((((..((((((((((..(((....((((......))))))).))))))))))))
))))))))...))..))..
-----------------------GCUGGGAUUACAGGCAUG---------------------------------------
------------------- 3
-----------------------GCUGGGAUUACAGGCAUGAG-------------------------------------
------------------- 2

>hsa-mir-624   mi:16-37;MA:54-74   SRX018958
aaugcuguuucaaggUAGUACCAGUACCUUGUGUUCAguggaaccaagguaaaCACAAGGUAUUGGUAUUACCUugagau
agcauuacaccuaagug
((((((((((((((((((((((((((((((((((((..((....))..)..)))))))))))))))))))))))))))))
)))))).........
---------------UAGUACCAGUACCUUGUGUU---
-----------------   2   0,-2
---------------UAGUACCAGUACCUUGUGUUC--
-----------------   9   0,-1
---------------UAGUACCAGUACCUUGUGUUCA---
-----------------   9   0,0
---AACACAAGGUAUUGGUAUUACu-------
-----------------   2   -2,-2

>hsa-mir-625   MA:15-35;mi:52-73   SRX018958
aggguagagggaugAGGGGGAAAGUUCUAUAGUCCuguaauuagaucucagGACUAUAGAACUUUCCCCCUCAucccucu
gcccu

(((((((((((((((((((((((((((((((((((((...........))))))))))))))))))))))))))))))))
)))))
--------------AGGGGGAAAGUUCUAUAGU---
----- 5 0,-2
--------------AGGGGGAAAGUUCUAUAGUC--
----- 12 0,-1
--------------AGGGGGAAAGUUCUAUAGUCa---
----- 2 0,-1
--------------AGGGGGAAAGUUCUAUAGUCC---
----- 11 0,0
--------------AGGGGGAAAGUUCUAUAGUCCU--
----- 10 0,1
--------------AGGGGGAAAGUUCUAUAGUCCUa---
----- 2 0,1
---GACUAUAGAACUUUCCCCC----------
----- 3 0,-3
---GACUAUAGAACUUUCCCCCUC--------
----- 9 0,-1
---GACUAUAGAACUUUCCCCCUCu-------
----- 2 0,-1
---GACUAUAGAACUUUCCCCCUCA-------
----- 10 0,0
---GACUAUAGAACUUUCCCCCUCAa------
----- 8 0,0
---GACUAUAGAACUUUCCCCCUCAU------
----- 6 0,1
--ACUAUAGAACUUUCCCCCUCA-------
----- 4 1,0
--ACUAUAGAACUUUCCCCCUCAaa-----
----- 3 1,0

>hsa-mir-628 5p:23-44;3p:61-81 SRX018958
auagcuguugugucacuuccucAUGCUGACAUAUUUACUAGAGGguaaaauuaauaaccuUCUAGUAAGAGUGGCAGUCG
Aagggaagggcucau
........((.(((.((((((..(((((.(((.(((((((((((((..........))))))))))))).))).))).))
..))))))))).)).
----------------------AUGCUGACAUAUUUACUA--
--------------- 6 0,-4

----------------------AUGCUGACAUAUUUACUAG---------------------------------------
--------------- 5   0,-3
----------------------AUGCUGACAUAUUUACUAGA--------------------------------------
--------------- 7   0,-2
----------------------AUGCUGACAUAUUUACUAGAG-------------------------------------
--------------- 18 0,-1
----------------------AUGCUGACAUAUUUACUAGAGa------------------------------------
--------------- 4   0,-1
----------------------AUGCUGACAUAUUUACUAGAGG------------------------------------
--------------- 20 0,0
----------------------AUGCUGACAUAUUUACUAGAGGa-----------------------------------
--------------- 17 0,0
----------------------AUGCUGACAUAUUUACUAGAGGaa----------------------------------
--------------- 8   0,0
----------------------AUGCUGACAUAUUUACUAGAGGu-----------------------------------
--------------- 2   0,0
----------------------AUGCUGACAUAUUUACUAGAGGG-----------------------------------
--------------- 5   0,1
----------------------AUGCUGACAUAUUUACUAGAGGGa----------------------------------
--------------- 2   0,1

>hsa-mir-629   MA:22-42;mi:61-82   SRX018958
ucccuuucccaggggaggggcUGGGUUUACGUUGGGAGAACUuuuacggugaaccaggagGUUCUCCCAACGUAAGCCCA
GCcccuccccucugccu
..........(((((((((((((((((((((((((((((((((((..((....)).))))))))))))))))))))))))
))))))))))).......
---------------------UGGGUUUACGUUGGGAGA---
-----------------   13 0,-3
---------------------UGGGUUUACGUUGGGAGAA--
-----------------   8   0,-2

>hsa-mir-641   5p:16-39   SRX018958
ugggugaaaggaaggAAAGACAUAGGAUAGAGUCACCUCuguccucuguccucuaccuauagaggugacuguccuauguc
uuuccuuccucuuaccccu
.((((((.((((((((((((((((((((..((((((((((((.....(....).....))))))))))))))))))))))
)))))))))).))))))..
---------------AAAGACAUAGGAUAGAGUCA---
------------------- 21 0,-4

---------------AAAGACAUAGGAUAGAGUCAa---
------------------- 3   0,-4
---------------AAAGACAUAGGAUAGAGUCAC---
------------------- 30 0,-3
---------------AAAGACAUAGGAUAGAGUCACC--
------------------- 50 0,-2
---------------AAAGACAUAGGAUAGAGUCACCa---
------------------- 3   0,-2
---------------AAAGACAUAGGAUAGAGUCACCU---
------------------- 67 0,-1
---------------AAAGACAUAGGAUAGAGUCACCUu--
------------------- 2   0,-1
---------------AAAGACAUAGGAUAGAGUCACCUC--
------------------- 6   0,0
---------------AAAGACAUAGGAUAGAGUCACCUCU---------------------------------------
------------------- 2   0,1
----------------AAGACAUAGGAUAGAGUCACC--
------------------- 3   1,-2
----------------AAGACAUAGGAUAGAGUCACCU---
------------------- 2   1,-1
---AGGUGACUGUCCUAUGUC
UUU---------------- 2

>hsa-mir-642a   5p:16-37   SRX018958
aucugaguugggaggGUCCCUCUCCAAAUGUGUCUUGgggugggggaucaagacacauuuggagagggaaccucccaacu
cggccucugccaucauu
..(((((((((((((.((((((((((((((((((((((.........))))))))))))))))))))))).))))))))))
))).............
---AGACACAUUUGGAGAGGG-----------
-----------------   4
---AGACACAUUUGGAGAGGGA----------
-----------------   3
---AGACACAUUUGGAGAGGGAA---------
-----------------   3
---AGACACAUUUGGAGAGGGAAuu-------
-----------------   2

>hsa-mir-642b   3p:47-68   SRX018958

gaguugggagguucccucuccaaaugugucuugaucccccaccccaAGACACAUUUGGAGAGGGACCCucccaacuc
(((((((((((.(((((((((((((((((((((...........))))))))))))))))))))).)))))))))))
--AGACACAUUUGGAGAGGG-------------
4 0,-4
--AGACACAUUUGGAGAGGGA------------
3 0,-3

>hsa-mir-643 3p:61-82 SRX018958
accaagugauauucauugucuaccugagcuagaauacaaguaguuggcgucuucagagacACUUGUAUGCUAGCUCAGGU
AGauauugaaugaaaaa
.........((((((.((((((((((((((((.((((((((.(((..(.......).))))))))))).)))))))))))
))))).)))))).....
--ACACUUGUAUGCUAGCUCAGG-
----------------- 2 -2,-3
--ACACUUGUAUGCUAGCUCAGGU
A---------------- 3 -2,-1

>hsa-mir-652 3p:61-81 SRX018958
acgaauggcuaugcacugcacaacccuaggagagggugccauucacauagacuauaauugAAUGGCGCCACUAGGGUUGU
Gcagugcacaaccuacac
......((...(((((((((((((((((((.....((((((((((..((.....))..))))))))))).))))))))))
))))))))...)).....
--AAUGGCGCCACUAGGGUU--
------------------ 20 0,-3
--AAUGGCGCCACUAGGGUUGU
------------------ 3 0,-1
--AAUGGCGCCACUAGGGUUGU
Ga---------------- 3 0,0
--AAUGGCGCCACUAGGGUUGU
Gu---------------- 7 0,0

>hsa-mir-654 5p:16-37;3p:51-72 SRX018958
ggguaaguggaaagaUGGUGGGCCGCAGAACAUGUGCugaguucgugccaUAUGUCUGCUGACCAUCACCUUuagaagcc
c
((((......((((.((((((.(.(((((.(((((((((....)).).))))))))))).).)))))).))))....)))
)
--UAUGUCUGCUGACCAUCACC----------
- 11 0,-2

--UAUGUCUGCUGACCAUCACCa---------
-	2	0,-2
--UAUGUCUGCUGACCAUCACCaa--------
-	2	0,-2
--UAUGUCUGCUGACCAUCACCU---------
-	11	0,-1
--UAUGUCUGCUGACCAUCACCUau-------
-	2	0,-1
--UAUGUCUGCUGACCAUCACCUU--------
-	2	0,0
--UAUGUCUGCUGACCAUCACCUUU-------
-	3	0,1

>hsa-mir-655	3p:61-82	SRX018958
aacuaugcaaggauauuugaggagagguuauccguguuauguucgcuucauucaucaugaAUAAUACAUGGUUAACCUCU
UUuugaaauaucagacuc
...........((((((..(((((((((((.((((((..((((((............))))))..)))))).))))))))
)))..))))))......
--AUAAUACAUGGUUAACCUC-
-----------------	4	0,-3
--AUAAUACAUGGUUAACCUCU
UU---------------	11	0,0

>hsa-mir-656	3p:43-63	SRX018958
cugaaauagguugccugugagguguucacuuucuauaugaugAAUAUUAUACAGUCAACCUCUuuccgauaucgaauc
..((((.((((((.(((((..(((((((...((.....)))))))))..))))).)))))).))))............
--AAUAUUAUACAGUCAACCU-----------------
	4	0,-2
--AAUAUUAUACAGUCAACCUC----------------
	5	0,-1
--AAUAUUAUACAGUCAACCUCU---------------
	2	0,0

>hsa-mir-659	3p:61-82	SRX018958
uaccgacccucgauuugguucaggaccuucccugaaccaaggaagagucacagucucuucCUUGGUUCAGGGAGGGUCCC
CAacaauguccucaugg
...........(((...(((..(((((((((((((((((((((((((.......))))))))))))))))))))))))).
.)))...)))......

--------------------CAGGACCUUCCCUGAACCAAGGA-------------------------------------
-----------------   2

---------------------AGGACCUUCCCUGAACCAAGGA-------------------------------------
-----------------   3

---------------------AGGACCUUCCCUGAACCAAGGAA------------------------------------
-----------------   5

----------------------GGACCUUCCCUGAACCAAGGAu------------------------------------
-----------------   2

>hsa-mir-660   5p:16-37   SRX018958

cugcuccuucucccaUACCCAUUGCAUAUCGGAGUUGugaauucucaaaacaccuccugugugcauggauuacaggaggg
ugagccuugucaucgug

..((((.((((((.....((((.((((((.((((.(((...........))).)))).))))))))))......))))))
.)))).............

---------------UACCCAUUGCAUAUCGGAG--
-----------------   8   0,-3

---------------UACCCAUUGCAUAUCGGAGU---
-----------------   14   0,-2

---------------UACCCAUUGCAUAUCGGAGUU--
-----------------   119 0,-1

---------------UACCCAUUGCAUAUCGGAGUUa---
-----------------   6   0,-1

---------------UACCCAUUGCAUAUCGGAGUUu---
-----------------   2   0,-1

---------------UACCCAUUGCAUAUCGGAGUUG---
-----------------   25   0,0

---------------UACCCAUUGCAUAUCGGAGUUGU--
-----------------   17   0,1

---ACCUCCUGUGUGCAUGGAUU---------
-----------------   5

---ACCUCCUGUGUGCAUGGAUUu--------
-----------------   2

---ACCUCCUGUGUGCAUGGAUUA--------
-----------------   5

>hsa-mir-663b   3p:90-111   SRX018958

ggugccgagggccguccggcauccuaggcgggucgcugcgguaccucccuccugucuguggcggugggaucccguggccg
uguuuuccuGGUGGCCCGGCCGUGCCUGAGGuuuc

...(((..((((((.(((((...(((((..((..((..(((....((((.((.((.....)))).)))).)))..))..))..
..))..)))))..).)))).)).)))).)))...

---------GGUGGCCCGGCCGUGCCU-------- 2 0,-4

>hsa-mir-664 mi:11-34;MA:49-71 SRX018958
gaacauugaaACUGGCUAGGGAAAAUGAUUGGAUagaaacuauuauucUAUUCAUUUAUCCCCAGCCUACAaaaugaaaaaa
...((((......((((.((((...(((.(((((((((.......))))))))).))))))).)))).....))))......
..
---------AACUGGCUAGGGAAAAUGAUUGG--
-- 4 -1,-2
----------ACUGGCUAGGGAAAAUGAUU--
-- 2 0,-4
----------ACUGGCUAGGGAAAAUGAUUG---
-- 7 0,-3
----------ACUGGCUAGGGAAAAUGAUUGu--
-- 2 0,-3
----------ACUGGCUAGGGAAAAUGAUUGG--
-- 131 0,-2
----------ACUGGCUAGGGAAAAUGAUUGGu---
-- 45 0,-2
----------ACUGGCUAGGGAAAAUGAUUGGuu--
-- 2 0,-2
----------ACUGGCUAGGGAAAAUGAUUGGA---
-- 181 0,-1
----------ACUGGCUAGGGAAAAUGAUUGGAa--
-- 12 0,-1
----------ACUGGCUAGGGAAAAUGAUUGGAU--
-- 6 0,0
-----------CUGGCUAGGGAAAAUGAUU--
-- 2 1,-4
-----------CUGGCUAGGGAAAAUGAUUGG--
-- 2 1,-2
-----------CUGGCUAGGGAAAAUGAUUGGu---
-- 2 1,-2
-----------CUGGCUAGGGAAAAUGAUUGGA---
-- 24 1,-1

-----------CUGGCUAGGGAAAAUGAUUGGAa--
-- 2 1,-1
-----------CUGGCUAGGGAAAAUGAUUGGAU--
-- 4 1,0
------------UGGCUAGGGAAAAUGAUUGGA---
-- 16 2,-1
------------UGGCUAGGGAAAAUGAUUGGAa--
-- 2 2,-1
--UAUUCAUUUAUCCCCAGC--------------
-- 5 0,-5
--UAUUCAUUUAUCCCCAGCCU------------
-- 2 0,-3
--UAUUCAUUUAUCCCCAGCCUA-----------
-- 8 0,-2
--UAUUCAUUUAUCCCCAGCCUAu----------
-- 3 0,-2
--UAUUCAUUUAUCCCCAGCCUAC----------
-- 7 0,-1
--UAUUCAUUUAUCCCCAGCCUACA---------
-- 30 0,0
--UAUUCAUUUAUCCCCAGCCUACAu--------
-- 3 0,0

>hsa-mir-665 3p:43-62 SRX018958
ucuccucgaggggucucugccucuacccaggacucuuucaugACCAGGAGGCUGAGGCCCCUcacaggcggc
((.(((.((((((((((.((((((...((.((.....)).))....)))))).))))))))))..))).)).
--ACCAGGAGGCUGAGGCCCCUC--------- 4
0,1
--ACCAGGAGGCUGAGGCCCCUCu-------- 2
0,1

>hsa-mir-671 5p:29-51;3p:68-88 SRX018958
gcaggugaacuggcaggccaggaagaggAGGAAGCCCUGGAGGGGCUGGAGgugauggauguuuuccUCCGGUUCUCAGG
GCUCCACCucuuucgggccguagagccagggcuggugc
(((...(..(((((.((((.((((((((.(((.((((((..(((((((((((.(((....)))..)))))))))))))))
))))).)))))))).)))).....)))))..)...)))
---UCCGGUUCUCAGG
GCUCCACu------------------------------ 3 0,-1

--UCCGGUUCUCAGG
GCUCCACC------------------------------ 2 0,0

>hsa-mir-675 MA:10-32;mi:45-64 SRX018958
cccagggucUGGUGCGGAGAGGGCCCACAGUGgacuuggugacgCUGUAUGCCCUCACCGCUCAgccccuggg
(((((((.((((.((((.((((((..((((((.........))))))..)))))).)))))))).)))))))
---------UGGUGCGGAGAGGGCCCACAGU-- 2
0,-1
---------UGGUGCGGAGAGGGCCCACAGUGu-- 2
0,0
--CUGUAUGCCCUCACCGCUCAG-------- 2
0,1
--CUGUAUGCCCUCACCGCUCAGC------- 2
0,2
--CUGUAUGCCCUCACCGCUCAGCa------ 2
0,2

>hsa-mir-676 mi:8-28;MA:43-63 SRX018958
gcaugacUCUUCAACCUCAGGACUUGCAgaauuaauggaaugCUGUCCUAAGGUUGUUGAGUUgugc
(((..((((..((((((.(((((..(((...........))).))))).))))))..))))..)))
--CUGUCCUAAGGUUGUUGAGU----- 2 0,-1
--CUGUCCUAAGGUUGUUGAGUU---- 4 0,0

>hsa-mir-7-1 MA:24-46;mi:66-87 SRX018958
uggauguuggccuaguucugugUGGAAGACUAGUGAUUUUGUUGUuuuuagauaacuaaaucgaCAACAAAUCACAGUC
UGCCAUAuggcacaggccaugccucuacag
.((((.((((((((.((.((((((((.(((((.(((((((.((((((((((((....)))))..))))))))))))))))
).))))))).)))))))).)).))))...
-----------------------UGGAAGACUAGUGAUUUU---------------------------------------
------------------------------ 2 0,-5
-----------------------UGGAAGACUAGUGAUUUUGUU------------------------------------
------------------------------ 7 0,-2
-----------------------UGGAAGACUAGUGAUUUUGUUG-----------------------------------
------------------------------ 13 0,-1
-----------------------UGGAAGACUAGUGAUUUUGUUGU----------------------------------
------------------------------ 40 0,0
-----------------------UGGAAGACUAGUGAUUUUGUUGUU---------------------------------
------------------------------ 235 0,1

-----------------------UGGAAGACUAGUGAUUUUGUUGUUa--------------------------------
------------------------------ 2 0,1
------------------------GGAAGACUAGUGAUUUUGUUGU----------------------------------
------------------------------ 3 1,0
---CAACAAAUCACAGUC
UGCCA------------------------- 2 0,-2
---CAACAAAUCACAGUC
UGCCAU------------------------ 5 0,-1
---CAACAAAUCACAGUC
UGCCAUu----------------------- 3 0,-1
---CAACAAAUCACAGUC
UGCCAUA----------------------- 17 0,0
---CAACAAAUCACAGUC
UGCCAUAa---------------------- 7 0,0
---CAACAAAUCACAGUC
UGCCAUAU---------------------- 2 0,1
--AACAAAUCACAGUC
UGCCAUA----------------------- 11 1,0
--AACAAAUCACAGUC
UGCCAUAa---------------------- 4 1,0
--AACAAAUCACAGUC
UGCCAUAU---------------------- 4 1,1

>hsa-mir-7-2 MA:32-54;mi:72-93 SRX018958
cuggauacagaguggaccggcuggccccaucUGGAAGACUAGUGAUUUUGUUGUugucuuacugcgcucaaCAACAAAUC
CCAGUCUACCUAAuggugccagccaucgca
..........((((...(((((((.((((..((.(((((.(.(((((.(((((((...........))))))))))))
).)))))).))..)))).))))))).)))).
-------------------------------UGGAAGACUAGUGAUUUU-------------------------------
------------------------------ 2 0,-5
-------------------------------UGGAAGACUAGUGAUUUUGUU----------------------------
------------------------------ 7 0,-2
-------------------------------UGGAAGACUAGUGAUUUUGUUG---------------------------
------------------------------ 13 0,-1
-------------------------------UGGAAGACUAGUGAUUUUGUUGU--------------------------
------------------------------ 40 0,0
-------------------------------UGGAAGACUAGUGAUUUUGUUGUU-------------------------
------------------------------ 235 0,1

-------------------------------UGGAAGACUAGUGAUUUUGUUGUUa------------------------
------------------------------ 2   0,1
--------------------------------GGAAGACUAGUGAUUUUGUUGU--------------------------
------------------------------ 3   1,0

>hsa-mir-7-3   5p:31-53   SRX018958
agauuagaguggcuguggucuagugcugugUGGAAGACUAGUGAUUUUGUUGUucugauguacuacgacaacaagucaca
gccggccucauagcgcagacucccuucgac
......(((.((....(((((.((((((((.((..(.((.(((((((.((((((.((......)).))))))))))))))
).)..)).))))))))))))))).)))...
------------------------------UGGAAGACUAGUGAUUUU--------------------------------
------------------------------ 2   0,-5
------------------------------UGGAAGACUAGUGAUUUUGUU-----------------------------
------------------------------ 7   0,-2
------------------------------UGGAAGACUAGUGAUUUUGUUG----------------------------
------------------------------ 13 0,-1
------------------------------UGGAAGACUAGUGAUUUUGUUGU---------------------------
------------------------------ 40 0,0
------------------------------UGGAAGACUAGUGAUUUUGUUGUU--------------------------
------------------------------ 235 0,1
------------------------------UGGAAGACUAGUGAUUUUGUUGUUa-------------------------
------------------------------ 2   0,1
-------------------------------GGAAGACUAGUGAUUUUGUUGU---------------------------
------------------------------ 3   1,0

>hsa-mir-708   MA:11-33;mi:57-78   SRX018958
aacugcccucAAGGAGCUUACAAUCUAGCUGGGgguaaaugacuugcacaugaacaCAACUAGACUGUGAGCUUCUAGag
ggcaggga
..((((((((.(((((((((((.(((((.((...((..(((.......)))..)).)).))))).))))))))))).)))
)))))...
----------AAGGAGCUUACAAUCUAG--
--------   7   0,-5
----------AAGGAGCUUACAAUCUAGC---
--------   4   0,-4
----------AAGGAGCUUACAAUCUAGCU--
--------   27 0,-3
----------AAGGAGCUUACAAUCUAGCUG---
--------   4   0,-2

----------AAGGAGCUUACAAUCUAGCUGG--
--------   33 0,-1
----------AAGGAGCUUACAAUCUAGCUGGa---
--------   3   0,-1
----------AAGGAGCUUACAAUCUAGCUGGu---
--------   7   0,-1
----------AAGGAGCUUACAAUCUAGCUGGG---
--------   24 0,0
--CAACUAGACUGUGAGCUUCUAG--
--------   3   0,0
---AACUAGACUGUGAGCUUCUAGA-
--------   4   1,1
---AACUAGACUGUGAGCUUCUAGAa
--------   4   1,1
--ACUAGACUGUGAGCUUCUAGA-
--------   4   2,1

>hsa-mir-720   5p:27-43   SRX018958
ccggaucucacacggugguguuaaauaUCUCGCUGGGGCCUCCAaaauguugugcccagggguguuagagaaaacaccaca
cuuugagaugaauuaagaguccuuuauuag
....((((((....((((((((((((((....(((((..(.....)..).)))).)))))))))......))))))).
...))))))......................
-------------------------AUCUCGCUGGGGCCUCCA--------------------------------------
------------------------------ 71 -1,0

>hsa-mir-744   MA:11-32;mi:68-89   SRX018958
uugggcaaggUGCGGGGCUAGGGCUAACAGCAgucuuacugaagguuuccuggaaaccacgcacaugCUGUUGCCACUAA
CCUCAACCUuacucgguc
(((((.(((((..((((.((((((.(((((((..............................))))))))).)))).
)))).))))).)))))..
----------UGCGGGGCUAGGGCUAACAG--
------------------ 11 0,-2
----------UGCGGGGCUAGGGCUAACAGa---
------------------ 3   0,-2
----------UGCGGGGCUAGGGCUAACAGu---
------------------ 4   0,-2
----------UGCGGGGCUAGGGCUAACAGC---
------------------ 11 0,-1

----------UGCGGGGCUAGGGCUAACAGCA--
------------------ 121 0,0
----------UGCGGGGCUAGGGCUAACAGCAa---
------------------ 49 0,0
----------UGCGGGGCUAGGGCUAACAGCAau--
------------------ 6 0,0
----------UGCGGGGCUAGGGCUAACAGCAu---
------------------ 136 0,0
----------UGCGGGGCUAGGGCUAACAGCAuu--
------------------ 12 0,0
-----------GCGGGGCUAGGGCUAACAG--
------------------ 2 1,-2
-----------GCGGGGCUAGGGCUAACAGC---
------------------ 4 1,-1
-----------GCGGGGCUAGGGCUAACAGCA--
------------------ 28 1,0
-----------GCGGGGCUAGGGCUAACAGCAa---
------------------ 10 1,0
-----------GCGGGGCUAGGGCUAACAGCAaa--
------------------ 2 1,0
-----------GCGGGGCUAGGGCUAACAGCAu---
------------------ 25 1,0
-----------GCGGGGCUAGGGCUAACAGCAuu--
------------------ 5 1,0

>hsa-mir-758 3p:52-73 SRX018958
gccuggauacaugagaugguugaccagagagcacacgcuuuauuugugccgUUUGUGACCUGGUCCACUAACCcucagua
ucuaaugc
...(((((((.((((.((((.((((((...(((.((((.........).))).)))...)))))).))))...)))))))
))))....
---------------AUGGUUGACCAGAGAGCACA---
-------- 18
---------------AUGGUUGACCAGAGAGCACAC--
-------- 14
---------------AUGGUUGACCAGAGAGCACACGu--
-------- 2
----------------UGGUUGACCAGAGAGCACAC--
-------- 2

>hsa-mir-760 3p:49-68 SRX018958
ggcgcgucgcccccucaguccaccagagcccggauaccucagaaauuCGGCUCUGGGUCUGUGGGGAgcgaaaugcaac
.(((..((((.((((.(((....((((((((.((((.........))))))))))))..))).)))).)))).)))...
--CGGCUCUGGGUCUGUGGGGAG-----------
2 0,1

>hsa-mir-765 3p:69-89 SRX018958
uuuaggcgcugaugaaaguggaguucaguagacagcccuuuucaagcccuacgagaaacugggguuucUGGAGGAGAAGG
AAGGUGAUGaaggaucuguucucgugagccugaa
.((((((....((((.((..((.((((....((...(((((((((((((((........))))))))......)))))))
...))..))))...))..)).))))..)))))).
--UGGAGGAGAAGG
AAGGUGAUGA------------------------ 2 0,1
--UGGAGGAGAAGG
AAGGUGAUGAA----------------------- 2 0,2

>hsa-mir-766 3p:65-86 SRX018958
gcauccucaggaccugggcuugggugguaggaggaauuggugcuggucuuucauuuuggauuugACUCCAGCCCCACAGC
CUCAGCcaccccagccaauugucauaggagc
((.((((...(((...((((.(((((((..((((...(((.(((((....(((.........)))..))))).)))...)
))).))))))).))))....)))..))))))
----------------------------AGGAGGAAUUGGUGCUGGUa--------------------------------
------------------------------- 2
----------------------------AGGAGGAAUUGGUGCUGGUC--------------------------------
------------------------------- 2
----------------------------AGGAGGAAUUGGUGCUGGUCUU------------------------------
------------------------------- 12
----------------------------AGGAGGAAUUGGUGCUGGUCUUa-----------------------------
------------------------------- 11
----------------------------AGGAGGAAUUGGUGCUGGUCUUau----------------------------
------------------------------- 4
----------------------------AGGAGGAAUUGGUGCUGGUCUUU-----------------------------
------------------------------- 64
----------------------------AGGAGGAAUUGGUGCUGGUCUUUa----------------------------
------------------------------- 5
----------------------------AGGAGGAAUUGGUGCUGGUCUUUaa---------------------------
------------------------------- 2

----------------------------AGGAGGAAUUGGUGCUGGUCUUUu----------------------------
------------------------------- 5
----------------------------AGGAGGAAUUGGUGCUGGUCUUUC----------------------------
------------------------------- 4
----------------------------AGGAGGAAUUGGUGCUGGUCUUUCA---------------------------
------------------------------- 6
-----------------------------GGAGGAAUUGGUGCUGGUCUU------------------------------
------------------------------- 2
-----------------------------GGAGGAAUUGGUGCUGGUCUUU-----------------------------
------------------------------- 3
-----------------------------GGAGGAAUUGGUGCUGGUCUUUC----------------------------
------------------------------- 2
--ACUCCAGCCCCACAGC
CUCAGCau----------------------- 2 0,0

>hsa-mir-769 5p:30-51;3p:69-91 SRX018958
gccuuggugcugauuccugggcucugaccUGAGACCUCUGGGUUCUGAGCUgugauguugcucucgagCUGGGAUCUCCG
GGGUCUUGGUUcagggccggggccucuggguuccaagc
..(((((.(((((..(((.((((((((.(..(((((((.(((.(((.((((.(((........))))))).))).))).)
))))))..).)))))))).)))..))..))).))))).
-----------------------------UGAGACCUCUGGGUUCUGAG-------------------------------
-------------------------------------- 4 0,-2
-----------------------------UGAGACCUCUGGGUUCUGAGa------------------------------
-------------------------------------- 2 0,-2
-----------------------------UGAGACCUCUGGGUUCUGAGCU-----------------------------
-------------------------------------- 4 0,0
-----------------------------UGAGACCUCUGGGUUCUGAGCUuu---------------------------
-------------------------------------- 3 0,0

>hsa-mir-873 5p:11-31 SRX018958
gugugcauuuGCAGGAACUUGUGAGUCUCCUauugaaaaugaacaggagacugaugaguucccgggaacacccacaa
(((.(..(((.(.((((((..(.((((((((.((.......)).)))))))).)..)))))).).)))..).)))..
----------GCAGGAACUUGUGAGUCUCCU--
2 0,0

>hsa-mir-874 3p:47-68 SRX018958
uuagcccugcggccccacgcaccaggguaagagagacucucgcuucCUGCCCUGGCCCGAGGGACCGAcuggcugggc
((((((...(((.(((.((..((((((((.(((.(.....)..))).))))))))..)).))).)))...))))))..

--CUGCCCUGGCCCGAGGGACCGACU--------
    2   0,2

>hsa-mir-877   MA:1-20;mi:66-86   SRX018958
GUAGAGGAGAUGGCGCAGGGgacacgggcaaagacuugggggguuccugggacccucagacgugugUCCUCUUCUCCCUCC
UCCCAG
...((((((..((.(.((((((((((.(.......(((((((((.....))))))))).).)))))))))).).))))))
))....
GUAGAGGAGAUGGCGCAGGG--
------ 2   0,0
GUAGAGGAGAUGGCGCAGGGGA---
------ 6   0,2
GUAGAGGAGAUGGCGCAGGGGAC--
------ 4   0,3
GUAGAGGAGAUGGCGCAGGGGACA---
------ 17   0,4
-UAGAGGAGAUGGCGCAGGGGACA---
------ 2   1,4

>hsa-mir-885   5p:11-32;3p:43-64   SRX018958
ccgcacucucUCCAUUACACUACCCUGCCUCUucuccaugagAGGCAGCGGGGUGUAGUGGAUAgagcacgggu
(((..((((.(((((((((((.(((((((((((......))))))))).))))))))))))).)))).)))..
----------UCCAUUACACUACCCUGC--   15
    0,-4
----------UCCAUUACACUACCCUGCC---   14
    0,-3
----------UCCAUUACACUACCCUGCCU--   39
    0,-2
----------UCCAUUACACUACCCUGCCUC---   44
    0,-1
----------UCCAUUACACUACCCUGCCUCa--   4
    0,-1
----------UCCAUUACACUACCCUGCCUCU--   154
    0,0
----------UCCAUUACACUACCCUGCCUCUa---   5
    0,0
----------UCCAUUACACUACCCUGCCUCUau--   3
    0,0

----------UCCAUUACACUACCCUGCCUCUU--- 3
0,1
------------CAUUACACUACCCUGCCUC--- 7
2,-1
------------CAUUACACUACCCUGCCUCU-- 12
2,0
------------CAUUACACUACCCUGCCUCUU--- 2
2,1
--AGGCAGCGGGGUGUAGUG-------------- 4
0,-4
--AGGCAGCGGGGUGUAGUGG------------- 11
0,-3
--AGGCAGCGGGGUGUAGUGGA------------ 6
0,-2
--AGGCAGCGGGGUGUAGUGGAa----------- 5
0,-2
--AGGCAGCGGGGUGUAGUGGAaa---------- 7
0,-2
--AGGCAGCGGGGUGUAGUGGAU----------- 27
0,-1
--AGGCAGCGGGGUGUAGUGGAUu---------- 24
0,-1
--AGGCAGCGGGGUGUAGUGGAUuu--------- 2
0,-1
--AGGCAGCGGGGUGUAGUGGAUA---------- 39
0,0
--AGGCAGCGGGGUGUAGUGGAUAa--------- 23
0,0
--AGGCAGCGGGGUGUAGUGGAUAaa-------- 17
0,0
--AGGCAGCGGGGUGUAGUGGAUAau-------- 10
0,0
--AGGCAGCGGGGUGUAGUGGAUAu--------- 12
0,0
---GGCAGCGGGGUGUAGUGGAU----------- 2
1,-1

>hsa-mir-887 3p:48-69 SRX018958

gugcagauccuugggagcccuguuagacucuggauuuuacacuuggaGUGAACGGGCGCCAUCCCGAGGcuuugcacag
((((((.(((((((((((((((((..((((..(.........)..)))).)))))).))..)))))))).)))))))..
---GUGAACGGGCGCCAUCCCGAG-----------
3 0,-1

>hsa-mir-889 3p:49-69 SRX018958
gugcuuaaagaauggcuguccguaguauggucucuauauuuaugaugaUUAAUAUCGGACAACCAUUGUuuuaguaucc
(((((.(((.(((((.((((((.....(((((((.........)).)))))....)))))).))))).))).)))))..
--UUAAUAUCGGACAACCAU-------------
2 0,-3
--UUAAUAUCGGACAACCAUa------------
4 0,-3
--UUAAUAUCGGACAACCAUU------------
27 0,-2
--UUAAUAUCGGACAACCAUUG-----------
3 0,-1
--UUAAUAUCGGACAACCAUUGa----------
2 0,-1
--UUAAUAUCGGACAACCAUUGU----------
19 0,0

>hsa-mir-9-1 MA:16-38;mi:55-76 SRX018958
cgggguugguuguuaUCUUUGGUUAUCUAGCUGUAUGAgugguguggagucuucAUAAAGCUAGAUAACCGAAAGUaaaa
auaacccca
.(((((((....(((..(((((((((((((((.((((((.((.......))))))))).)))))))))))))))..)))..
.))))))).
---------------UCUUUGGUUAUCUAGCUGUAUG---
--------- 4 0,-1
---------------UCUUUGGUUAUCUAGCUGUAUGA--
--------- 6 0,0
--AUAAAGCUAGAUAACCGAAAGU----
--------- 8 0,0
---UAAAGCUAGAUAACCGAAAG-----
--------- 2 1,-1
---UAAAGCUAGAUAACCGAAAGU----
--------- 9 1,0
---UAAAGCUAGAUAACCGAAAGUAu--
--------- 2 1,1

>hsa-mir-9-2   MA:16-38;mi:53-74   SRX018958
ggaagcgaguuguuaUCUUUGGUUAUCUAGCUGUAUGAguguauuggucuucAUAAAGCUAGAUAACCGAAAGUaaaaac
uccuuca
.((((.(((((.(((..(((((((((((((((.((((((..(....)..)))))).)))))))))))))))..))).)))
)))))).
---------------UCUUUGGUUAUCUAGCUGUAUG---
------- 4   0,-1
---------------UCUUUGGUUAUCUAGCUGUAUGA--
------- 6   0,0
--AUAAAGCUAGAUAACCGAAAGU------
------- 8   0,0
---UAAAGCUAGAUAACCGAAAG-------
------- 2   1,-1
---UAAAGCUAGAUAACCGAAAGU------
------- 9   1,0
---UAAAGCUAGAUAACCGAAAGUAu----
------- 2   1,1

>hsa-mir-9-3   MA:16-38;mi:55-76   SRX018958
ggaggcccguuucucUCUUUGGUUAUCUAGCUGUAUGAgugccacagagccgucAUAAAGCUAGAUAACCGAAAGUagaa
augauucuca
.((((..((((((((.((((((((((((((((.(((((..((......))..))))).)))))))))))))))))).))))
)))..)))).
---------------UCUUUGGUUAUCUAGCUGUAUG---
----------   4   0,-1
---------------UCUUUGGUUAUCUAGCUGUAUGA--
----------   6   0,0
--AUAAAGCUAGAUAACCGAAAGU----
----------   8   0,0
---UAAAGCUAGAUAACCGAAAG-----
----------   2   1,-1
---UAAAGCUAGAUAACCGAAAGU----
----------   9   1,0
---UAAAGCUAGAUAACCGAAAGUAu--
----------   2   1,1

>hsa-mir-92a-1 mi:11-33;MA:48-69   SRX018958

cuuucuacacAGGUUGGGAUCGGUUGCAAUGCUguguuucuguauggUAUUGCACUUGUCCCGGCCUGUugaguuugg
..(((...((((((((((((.(((.((((((((((((......))))))))))))))).))))))))))))).))).....
----------AGGUUGGGAUCGGUUGCAAU--
21 0,-3
----------AGGUUGGGAUCGGUUGCAAUa---
7 0,-3
----------AGGUUGGGAUCGGUUGCAAUG---
54 0,-2
----------AGGUUGGGAUCGGUUGCAAUGuu---
2 0,-2
----------AGGUUGGGAUCGGUUGCAAUGC--
35 0,-1
----------AGGUUGGGAUCGGUUGCAAUGCU---
193 0,0
----------AGGUUGGGAUCGGUUGCAAUGCUu--
3 0,0
-----------GGUUGGGAUCGGUUGCAAUGCU---
4 1,0
---GUAUUGCACUUGUCCCGGCCU----------
2 -1,-2
--UAUUGCACUUGUCCCGGC------------
66 0,-4
--UAUUGCACUUGUCCCGGCau----------
2 0,-4
--UAUUGCACUUGUCCCGGCuu----------
5 0,-4
--UAUUGCACUUGUCCCGGCC-----------
388 0,-3
--UAUUGCACUUGUCCCGGCCa----------
4 0,-3
--UAUUGCACUUGUCCCGGCCU----------
5225 0,-2
--UAUUGCACUUGUCCCGGCCUa---------
6 0,-2
--UAUUGCACUUGUCCCGGCCUaa--------
15 0,-2
--UAUUGCACUUGUCCCGGCCUau--------
8 0,-2

---UAUUGCACUUGUCCCGGCCUu----------

7 0,-2

---UAUUGCACUUGUCCCGGCCUuu---------

4 0,-2

---UAUUGCACUUGUCCCGGCCUG----------

200 0,-1

---UAUUGCACUUGUCCCGGCCUGa---------

9 0,-1

---UAUUGCACUUGUCCCGGCCUGaa--------

12 0,-1

---UAUUGCACUUGUCCCGGCCUGau--------

5 0,-1

---UAUUGCACUUGUCCCGGCCUGU---------

467 0,0

---UAUUGCACUUGUCCCGGCCUGUa--------

65 0,0

---UAUUGCACUUGUCCCGGCCUGUaa-------

90 0,0

---UAUUGCACUUGUCCCGGCCUGUau-------

118 0,0

---UAUUGCACUUGUCCCGGCCUGUU--------

54 0,1

---UAUUGCACUUGUCCCGGCCUGUUa-------

14 0,1

---UAUUGCACUUGUCCCGGCCUGUUau------

6 0,1

---UAUUGCACUUGUCCCGGCCUGUUu-------

49 0,1

---UAUUGCACUUGUCCCGGCCUGUUuu------

13 0,1

--AUUGCACUUGUCCCGGCC------------

9 1,-3

--AUUGCACUUGUCCCGGCCU-----------

56 1,-2

--AUUGCACUUGUCCCGGCCUG----------

2 1,-1

--AUUGCACUUGUCCCGGCCUGU---------

3 1,0

--AUUGCACUUGUCCCGGCCUGUaa-------
3 1,0
--AUUGCACUUGUCCCGGCCUGUau-------
3 1,0
--AUUGCACUUGUCCCGGCCUGUU--------
2 1,1
---UUGCACUUGUCCCGGCCUGUUu-------
2 2,1

>hsa-mir-92a-2 mi:9-30;MA:48-69 SRX018958
ucaucccuGGGUGGGGAUUUGUUGCAUUACuuguguucuauauaaagUAUUGCACUUGUCCCGGCCUGUggaaga
((.(((.(((((.(((((..((.(((.(((((((((...)))).))))).)))))..))))).))))).))).))
--GUAUUGCACUUGUCCCGGCCU-------- 2
-1,-2
---UAUUGCACUUGUCCCGGC---------- 66
0,-4
---UAUUGCACUUGUCCCGGCau-------- 2
0,-4
---UAUUGCACUUGUCCCGGCuu-------- 5
0,-4
---UAUUGCACUUGUCCCGGCC--------- 388
0,-3
---UAUUGCACUUGUCCCGGCCa-------- 4
0,-3
---UAUUGCACUUGUCCCGGCCU-------- 5225
0,-2
---UAUUGCACUUGUCCCGGCCUa------- 6
0,-2
---UAUUGCACUUGUCCCGGCCUaa------ 15
0,-2
---UAUUGCACUUGUCCCGGCCUau------ 8
0,-2
---UAUUGCACUUGUCCCGGCCUu------- 7
0,-2
---UAUUGCACUUGUCCCGGCCUuu------ 4
0,-2
---UAUUGCACUUGUCCCGGCCUG------- 200
0,-1

--UAUUGCACUUGUCCCGGCCUGa------ 9
0,-1
--UAUUGCACUUGUCCCGGCCUGaa----- 12
0,-1
--UAUUGCACUUGUCCCGGCCUGau----- 5
0,-1
--UAUUGCACUUGUCCCGGCCUGU------ 467
0,0
--UAUUGCACUUGUCCCGGCCUGUa----- 65
0,0
--UAUUGCACUUGUCCCGGCCUGUaa---- 90
0,0
--UAUUGCACUUGUCCCGGCCUGUau---- 118
0,0
--UAUUGCACUUGUCCCGGCCUGUG----- 2
0,1
---AUUGCACUUGUCCCGGCC--------- 9
1,-3
---AUUGCACUUGUCCCGGCCU-------- 56
1,-2
---AUUGCACUUGUCCCGGCCUG------- 2
1,-1
---AUUGCACUUGUCCCGGCCUGU------ 3
1,0
---AUUGCACUUGUCCCGGCCUGUaa---- 3
1,0
---AUUGCACUUGUCCCGGCCUGUau---- 3
1,0

>hsa-mir-92b mi:20-41;MA:61-82 SRX018958
cgggccccgggcgggcgggAGGGACGGGACGCGGUGCAGUGuuguuuuuccccccgccaaUAUUGCACUCGUCCCGGCCUCCggcccccccggccc
.(((((..(((.((((.(((((..(((((((.((((((((((((............))))))))))))))))))).))))).))))))).)))))
-------------------AGGGACGGGACGCGGUGCAGU--- 3 0,-1
-------------------AGGGACGGGACGCGGUGCAGUGU--- 8 0,1

-------------------AGGGACGGGACGCGGUGCAGUGUU-------------------------------------
----------------   5   0,2

--UAUUGCACUCGUCCCGGCC-
----------------   8   0,-3

--UAUUGCACUCGUCCCGGCCU
----------------   31 0,-2

--UAUUGCACUCGUCCCGGCCU
a---------------   6   0,-2

--UAUUGCACUCGUCCCGGCCU
aa--------------   5   0,-2

--UAUUGCACUCGUCCCGGCCU
au--------------   4   0,-2

--UAUUGCACUCGUCCCGGCCU
uu--------------   5   0,-2

--UAUUGCACUCGUCCCGGCCU
C---------------   11 0,-1

--UAUUGCACUCGUCCCGGCCU
Caa-------------   4   0,-1

--UAUUGCACUCGUCCCGGCCU
Cau-------------   4   0,-1

--UAUUGCACUCGUCCCGGCCU
CC--------------   3   0,0

--UAUUGCACUCGUCCCGGCCU
CCa-------------   5   0,0

--UAUUGCACUCGUCCCGGCCU
CCau------------   9   0,0

--UAUUGCACUCGUCCCGGCCU
CCu-------------   7   0,0

---AUUGCACUCGUCCCGGCCU
CCu-------------   2   1,0

>hsa-mir-93 MA:11-33;mi:50-71   SRX018958
cugggggcucCAAAGUGCUGUUCGUGCAGGUAGugugauuacccaaccuACUGCUGAGCUAGCACUUCCCGagcccccgg
((((((((((..((((((((((((.((((.(((..((......))..)))))))))))))).)))))))...))))))))))

----------CAAAGUGCUGUUCGUGCA--
    114 0,-5

----------CAAAGUGCUGUUCGUGCAG---
    191 0,-4

----------CAAAGUGCUGUUCGUGCAGG---

94 0,-3

----------CAAAGUGCUGUUCGUGCAGGaa---

6 0,-3

----------CAAAGUGCUGUUCGUGCAGGU--

100 0,-2

----------CAAAGUGCUGUUCGUGCAGGUA---

289 0,-1

----------CAAAGUGCUGUUCGUGCAGGUAa--

77 0,-1

----------CAAAGUGCUGUUCGUGCAGGUAaa---

2 0,-1

----------CAAAGUGCUGUUCGUGCAGGUAu--

69 0,-1

----------CAAAGUGCUGUUCGUGCAGGUAG--

41 0,0

----------CAAAGUGCUGUUCGUGCAGGUAGa---

3 0,0

----------CAAAGUGCUGUUCGUGCAGGUAGU---

3 0,1

-----------AAAGUGCUGUUCGUGCAG--

12 1,-4

-----------AAAGUGCUGUUCGUGCAGG---

5 1,-3

-----------AAAGUGCUGUUCGUGCAGGU--

4 1,-2

-----------AAAGUGCUGUUCGUGCAGGUA---

19 1,-1

-----------AAAGUGCUGUUCGUGCAGGUAu--

4 1,-1

--ACUGCUGAGCUAGCACUUCCC----------

5 0,-1

--ACUGCUGAGCUAGCACUUCCCG---------

2 0,0

--ACUGCUGAGCUAGCACUUCCCGA--------

6 0,1

--ACUGCUGAGCUAGCACUUCCCGAu-------

3 0,1

>hsa-mir-934   5p:15-36   SRX018958

agaaauaaggcuucUGUCUACUACUGGAGACACUGGuaguauaaaacccagagucuccaguaauggacgggagccuuauu

ucu

((((((((((((((((((((.((((((((((.((((...........)))).)))))))))).)))))))))))))))))

)))

------------UCUGUCUACUACUGGAGACACU--

--- 2   -2,-2

--------------UGUCUACUACUGGAGACACU--

--- 2   0,-2

>hsa-mir-940   3p:60-80   SRX018958

gugaggugugggcccggccccaggagcggggccugggcagccccguguguugaggaaggAAGGCAGGGCCCCCGCUCCCC

gggccugaccccac

(((.(((.(((((((((.....((((((((((((..((..((((.......))..))...)).))).)))))))))))

)))))))))).)))

---AAGGCAGGGCCCCCGCUCCCC

--------------   8   0,0

---AAGGCAGGGCCCCCGCUCCCC

u-------------   3   0,0

---AAGGCAGGGCCCCCGCUCCCC

uu------------   3   0,0

>hsa-mir-941-1   3p:71-93   SRX018958

cacggaagaggacacacccggcuguguggacaugugcccagggcccgggacagcgccacggaagaggacgCACCCGGCUG

UGUGCACAUGUGCccagggcccgggacagcgccacgg

.........((.....(((((((.((.((((((((((.((.((((.(((...(((((.(....).)).))).))))))).

)).)))))))).)))).)).)))))......))....

--CACCCGGCUG

UGUGCACAUGU--------------------------   2   0,-2

--CACCCGGCUG

UGUGCACAUGUG-------------------------   5   0,-1

--CACCCGGCUG

UGUGCACAUGUGa------------------------   8   0,-1

--CACCCGGCUG

UGUGCACAUGUGu------------------------   3   0,-1

--CACCCGGCUG

UGUGCACAUGUGC------------------------   14   0,0

---ACCCGGCUG
UGUGCACAUGUGC------------------------ 2 1,0

>hsa-mir-941-2 3p:71-93 SRX018958
cacggaagaggacgcacccggcugugugcacaugugcccagggcccgggacagcgccacggaagaggacgCACCCGGCUG
UGUGCACAUGUGCccagggcccgggacagcgccacgg
.........((.(((.(((((((.((.((((((((((.((.((((.(((...(((((.(....).)).))).))))))).
)).)))))))))).)).)).)))))...)))))....
--------------CACCCGGCUGUGUGCACAUGU---
------------------------------------- 2
--------------CACCCGGCUGUGUGCACAUGUG--
------------------------------------- 5
--------------CACCCGGCUGUGUGCACAUGUGa---
------------------------------------- 8
--------------CACCCGGCUGUGUGCACAUGUGu---
------------------------------------- 3
--------------CACCCGGCUGUGUGCACAUGUGC---
------------------------------------- 14
---------------ACCCGGCUGUGUGCACAUGUGC---
------------------------------------- 2
--CACCCGGCUG
UGUGCACAUGU-------------------------- 2 0,-2
--CACCCGGCUG
UGUGCACAUGUG------------------------- 5 0,-1
--CACCCGGCUG
UGUGCACAUGUGa------------------------ 8 0,-1
--CACCCGGCUG
UGUGCACAUGUGu------------------------ 3 0,-1
--CACCCGGCUG
UGUGCACAUGUGC------------------------ 14 0,0
---ACCCGGCUG
UGUGCACAUGUGC------------------------ 2 1,0

>hsa-mir-941-3 3p:71-93 SRX018958
cacggaagaggacgcacccggcugugugcacaugugcccagggcccgggacagcgccacggaagaggacgCACCCGGCUG
UGUGCACAUGUGCccagggcccgggacagcgccaugg
.........((.(((.(((((((.((.((((((((((.((.((((.(((...(((((.(....).)).))).))))))).
)).)))))))))).)).)).)))))...)))))....

--------------CACCCGGCUGUGUGCACAUGU---
------------------------------------- 2
--------------CACCCGGCUGUGUGCACAUGUG--
------------------------------------- 5
--------------CACCCGGCUGUGUGCACAUGUGa---
------------------------------------- 8
--------------CACCCGGCUGUGUGCACAUGUGu---
------------------------------------- 3
--------------CACCCGGCUGUGUGCACAUGUGC---
------------------------------------- 14
---------------ACCCGGCUGUGUGCACAUGUGC---
------------------------------------- 2
--CACCCGGCUG
UGUGCACAUGU-------------------------- 2 0,-2
--CACCCGGCUG
UGUGCACAUGUG------------------------- 5 0,-1
--CACCCGGCUG
UGUGCACAUGUGa------------------------ 8 0,-1
--CACCCGGCUG
UGUGCACAUGUGu------------------------ 3 0,-1
--CACCCGGCUG
UGUGCACAUGUGC------------------------ 14 0,0
---ACCCGGCUG
UGUGCACAUGUGC------------------------ 2 1,0

>hsa-mir-941-4 3p:71-93 SRX018958
cauggaagaggacgcacccggcugugugcacaugugcccagggcccgggacagcgccacggaagaggacgCACCCGGCUG
UGUGCACAUGUGCccagggcccgggacagcgccacgg
.........((.(((.(((((((.((.((((((((((.((.((((.(((...(((((.(....).)).))).))))))).
)).)))))))))).)).)).))))))...)))))....
--------------CACCCGGCUGUGUGCACAUGU---
------------------------------------- 2
--------------CACCCGGCUGUGUGCACAUGUG--
------------------------------------- 5
--------------CACCCGGCUGUGUGCACAUGUGa---
------------------------------------- 8
--------------CACCCGGCUGUGUGCACAUGUGu---
------------------------------------- 3

--------------CACCCGGCUGUGUGCACAUGUGC---
-------------------------------------   14
---------------ACCCGGCUGUGUGCACAUGUGC---
-------------------------------------   2
--CACCCGGCUG
UGUGCACAUGU--------------------------   2   0,-2
--CACCCGGCUG
UGUGCACAUGUG-------------------------   5   0,-1
--CACCCGGCUG
UGUGCACAUGUGa------------------------   8   0,-1
--CACCCGGCUG
UGUGCACAUGUGu------------------------   3   0,-1
--CACCCGGCUG
UGUGCACAUGUGC------------------------   14 0,0
---ACCCGGCUG
UGUGCACAUGUGC------------------------   2   1,0

>hsa-mir-942   5p:13-34   SRX018958
auuaggagaguaUCUUCUCUGUUUUGGCCAUGUGuguacucacagccccucacacauggccgaaacagagaaguuacuuu
ccuaau
((((((((((((.(((((((((((((((((((((((..............))))))))))))))))))))))).))))))
))))))
--------------UUCUCUGUUUUGGCCAUGUGUG--
------ 2   2,2

>hsa-mir-944   3p:54-75   SRX018958
guuccagacacaucucaucugauauacaauauuuucuuaaauuguauaaagagAAAUUAUUGUACAUCGGAUGAGcugug
ucugggau
((((((((((((.((((((((((.(((((((.((((((............)))))).))))))).)))))))))).))))
))))))))
--GAAAUUAUUGUACAUCGGAUGAG-----
--------   3   -1,0
---AAAUUAUUGUACAUCGGAUGAG-----
--------   12   0,0
--AAUUAUUGUACAUCGGAUGAG-----
--------   4   1,0

>hsa-mir-95 3p:49-70   SRX018958

aacacagugggcacucaauaaaugucuguugaauugaaaugcguuacaUUCAACGGGUAUUUAUUGAGCAcccacucugu
g

.(((.((((((..((((((((((..((((((((((((......))).)))))))))..))))))))))..)))))).)))
.

--------------UCAAUAAAUGUCUGUUGAAUUa--
-    2

---AUUCAACGGGUAUUUAUUGAGa-----------
-    2   -1,-2

--UUCAACGGGUAUUUAUUG--------------
-    383 0,-4

--UUCAACGGGUAUUUAUUGA-------------
-    334 0,-3

--UUCAACGGGUAUUUAUUGAG------------
-    329 0,-2

--UUCAACGGGUAUUUAUUGAGC-----------
-    23   0,-1

--UUCAACGGGUAUUUAUUGAGCA----------
-    16   0,0

--UUCAACGGGUAUUUAUUGAGCAa---------
-    30   0,0

---UCAACGGGUAUUUAUUGA-------------
-    19   1,-3

---UCAACGGGUAUUUAUUGAG------------
-    2   1,-2

---UCAACGGGUAUUUAUUGAGC-----------
-    3   1,-1

---UCAACGGGUAUUUAUUGAGCA----------
-    6   1,0

---UCAACGGGUAUUUAUUGAGCAa---------
-    10   1,0

---UCAACGGGUAUUUAUUGAGCAu---------
-    4   1,0

--CAACGGGUAUUUAUUGAG------------
-    8   2,-2

--CAACGGGUAUUUAUUGAGCAa---------
-    4   2,0

--CAACGGGUAUUUAUUGAGCAu---------
-    4   2,0

>hsa-mir-98 5p:22-43 SRX018958
aggauucugcucaugccagggUGAGGUAGUAAGUUGUAUUGUUgugggguagggauauuaggccccaauuagaagauaac
uauacaacuuacuacuuucccugguguguggcauauuca
.((((..((((((((((((((.(((((((((((((((((.((((((((((...........)))))).........))))
.))))))))))))))))))))))))))).)))).)))).
---------------------UGAGGUAGUAAGUUGUAU---
--------------------------------------- 98 0,-4
---------------------UGAGGUAGUAAGUUGUAUa--
--------------------------------------- 10 0,-4
---------------------UGAGGUAGUAAGUUGUAUU--
--------------------------------------- 47 0,-3
---------------------UGAGGUAGUAAGUUGUAUUG---------------------------------------
--------------------------------------- 44 0,-2
---------------------UGAGGUAGUAAGUUGUAUUGU--------------------------------------
--------------------------------------- 53 0,-1
---------------------UGAGGUAGUAAGUUGUAUUGUa-------------------------------------
--------------------------------------- 3 0,-1
---------------------UGAGGUAGUAAGUUGUAUUGUaa------------------------------------
--------------------------------------- 2 0,-1
---------------------UGAGGUAGUAAGUUGUAUUGUU-------------------------------------
--------------------------------------- 73 0,0
---------------------UGAGGUAGUAAGUUGUAUUGUUa------------------------------------
--------------------------------------- 2 0,0
---------------------UGAGGUAGUAAGUUGUAUUGUUu------------------------------------
--------------------------------------- 12 0,0
----------------------GAGGUAGUAAGUUGUAUUG---------------------------------------
--------------------------------------- 2 1,-2
---C
UAUACAACUUACUACUUUCCu------------------ 6

UAUACAACUUACUACUUUCCa------------------ 2

>hsa-mir-99a MA:13-34;mi:50-71 SRX018958
cccauuggcauaAACCCGUAGAUCCGAUCUUGUGgugaaguggaccgcaCAAGCUCGCUUCUAUGGGUCUGgucagugu
g
..((((((((((.(((((((((..(((.((((((.((........)))))))).)))..))))))))).)))))))))).
.

-----------AAACCCGUAGAUCCGAUC---
- 3 -1,-5
-----------AAACCCGUAGAUCCGAUCU--
- 30 -1,-4
-----------AAACCCGUAGAUCCGAUCUU---
- 42 -1,-3
-----------AAACCCGUAGAUCCGAUCUUau---
- 2 -1,-3
-----------AAACCCGUAGAUCCGAUCUUG--
- 128 -1,-2
-----------AAACCCGUAGAUCCGAUCUUGU---
- 730 -1,-1
-----------AAACCCGUAGAUCCGAUCUUGUa--
- 6 -1,-1
-----------AAACCCGUAGAUCCGAUCUUGUaa---
- 7 -1,-1
-----------AAACCCGUAGAUCCGAUCUUGUuu---
- 2 -1,-1
-----------AAACCCGUAGAUCCGAUCUUGUG--
- 30 -1,0
-----------AAACCCGUAGAUCCGAUCUUGUGa---
- 10 -1,0
------------AACCCGUAGAUCCGAUCU--
- 1833 0,-4
------------AACCCGUAGAUCCGAUCUa---
- 6 0,-4
------------AACCCGUAGAUCCGAUCUU---
- 3995 0,-3
------------AACCCGUAGAUCCGAUCUUa--
- 79 0,-3
------------AACCCGUAGAUCCGAUCUUau---
- 15 0,-3
------------AACCCGUAGAUCCGAUCUUu--
- 125 0,-3
------------AACCCGUAGAUCCGAUCUUuu---
- 95 0,-3
------------AACCCGUAGAUCCGAUCUUG--
- 18253 0,-2

------------AACCCGUAGAUCCGAUCUUGa--

- 265 0,-2

------------AACCCGUAGAUCCGAUCUUGaa---

- 12 0,-2

------------AACCCGUAGAUCCGAUCUUGau---

- 7 0,-2

------------AACCCGUAGAUCCGAUCUUGU--

- 70183 0,-1

------------AACCCGUAGAUCCGAUCUUGUa---

- 397 0,-1

------------AACCCGUAGAUCCGAUCUUGUaa--

- 143 0,-1

------------AACCCGUAGAUCCGAUCUUGUau--

- 25 0,-1

------------AACCCGUAGAUCCGAUCUUGUu---

- 546 0,-1

------------AACCCGUAGAUCCGAUCUUGUuu--

- 55 0,-1

------------AACCCGUAGAUCCGAUCUUGUG---

- 59683 0,0

------------AACCCGUAGAUCCGAUCUUGUGa--

- 20906 0,0

------------AACCCGUAGAUCCGAUCUUGUGaa---

- 440 0,0

------------AACCCGUAGAUCCGAUCUUGUGau---

- 72 0,0

------------AACCCGUAGAUCCGAUCUUGUGu--

- 8930 0,0

------------AACCCGUAGAUCCGAUCUUGUGuu---

- 89 0,0

------------AACCCGUAGAUCCGAUCUUGUGG--

- 26 0,1

-------------ACCCGUAGAUCCGAUCUU--

- 2 1,-3

-------------ACCCGUAGAUCCGAUCUUG---

- 9 1,-2

-------------ACCCGUAGAUCCGAUCUUGU--

- 64 1,-1

```
-------------ACCCGUAGAUCCGAUCUUGUG----------------------------------------------
-    86   1,0
-------------ACCCGUAGAUCCGAUCUUGUGa---------------------------------------------
-    36   1,0
-------------ACCCGUAGAUCCGAUCUUGUGu---------------------------------------------
-    26   1,0
-------------------------------------------------CAAGCUCGCUUCUAUGGGU------------
-    2    0,-3
-------------------------------------------------CAAGCUCGCUUCUAUGGGUCU----------
-    16   0,-1
-------------------------------------------------CAAGCUCGCUUCUAUGGGUCUG---------
-    16   0,0
-------------------------------------------------CAAGCUCGCUUCUAUGGGUCUGa--------
-    2    0,0
-------------------------------------------------CAAGCUCGCUUCUAUGGGUCUGU--------
-    19   0,1
-------------------------------------------------CAAGCUCGCUUCUAUGGGUCUGUu-------
-    5    0,1
--------------------------------------------------AAGCUCGCUUCUAUGGGUC-----------
-    2    1,-2

>hsa-mir-99b     MA:7-28;mi:45-66     SRX018958
ggcaccCACCCGUAGAACCGACCUUGCGgggccuucgccgcacaCAAGCUCGUGUCUGUGGGUCCGuguc
(((((..(((((((((..(((.((((.(((((....))).).).)))).)))..)))))))))..)))))
------CACCCGUAGAACCGACCU----------------------------------------------  3    0,-4
------CACCCGUAGAACCGACCUU---------------------------------------------  12   0,-3
------CACCCGUAGAACCGACCUUG--------------------------------------------  22   0,-2
------CACCCGUAGAACCGACCUUGa-------------------------------------------  6    0,-2
------CACCCGUAGAACCGACCUUGaa------------------------------------------  3    0,-2
------CACCCGUAGAACCGACCUUGu-------------------------------------------  3    0,-2
------CACCCGUAGAACCGACCUUGC-------------------------------------------  285  0,-1
------CACCCGUAGAACCGACCUUGCa------------------------------------------  14   0,-1
------CACCCGUAGAACCGACCUUGCaa-----------------------------------------  3    0,-1
------CACCCGUAGAACCGACCUUGCau-----------------------------------------  3    0,-1
------CACCCGUAGAACCGACCUUGCG------------------------------------------  300  0,0
------CACCCGUAGAACCGACCUUGCGa-----------------------------------------  119  0,0
------CACCCGUAGAACCGACCUUGCGaa----------------------------------------  2    0,0
------CACCCGUAGAACCGACCUUGCGau----------------------------------------  7    0,0
```

------CACCCGUAGAACCGACCUUGCGu-------------------------------------- 99 0,0
------CACCCGUAGAACCGACCUUGCGuu------------------------------------- 4 0,0
------CACCCGUAGAACCGACCUUGCGG-------------------------------------- 14 0,1
-------ACCCGUAGAACCGACCUUGC-- 5 1,-1
-------ACCCGUAGAACCGACCUUGCG--------------------------------------- 6 1,0
-------ACCCGUAGAACCGACCUUGCGa-------------------------------------- 2 1,0
-------ACCCGUAGAACCGACCUUGCGu-------------------------------------- 5 1,0
--CAAGCUCGUGUCUGUGGGUC------ 4 0,-2
--CAAGCUCGUGUCUGUGGGUCC----- 3 0,-1
--CAAGCUCGUGUCUGUGGGUCCG---- 2 0,0
--CAAGCUCGUGUCUGUGGGUCCGa--- 5 0,0
--CAAGCUCGUGUCUGUGGGUCCGU--- 8 0,1
--CAAGCUCGUGUCUGUGGGUCCGUu-- 4 0,1

>hsa-let-7a-1 MA:6-27;mi:57-77 SRX018959
ugggaUGAGGUAGUAGGUUGUAUAGUUuuagggucacacccaccacugggagauaaCUAUACAAUCUACUGUCUUUCcua
(((((.(((..(((((((((((((((.........................)))))))))))))))..))))))))
----AUGAGGUAGUAGGUUGUAU--
12 -1,-4
----AUGAGGUAGUAGGUUGUAUAGU---
2 -1,-1
----AUGAGGUAGUAGGUUGUAUAGUU--
11 -1,0
----AUGAGGUAGUAGGUUGUAUAGUUU---
2 -1,1
-----UGAGGUAGUAGGUUGUAU--
1223 0,-4
-----UGAGGUAGUAGGUUGUAUu---
109 0,-4
-----UGAGGUAGUAGGUUGUAUuu--
74 0,-4
-----UGAGGUAGUAGGUUGUAUA---
6825 0,-3
-----UGAGGUAGUAGGUUGUAUAa--
312 0,-3
-----UGAGGUAGUAGGUUGUAUAaa---
101 0,-3
-----UGAGGUAGUAGGUUGUAUAau---

11 0,-3

-----UGAGGUAGUAGGUUGUAUAu---

128 0,-3

-----UGAGGUAGUAGGUUGUAUAuu--

20 0,-3

-----UGAGGUAGUAGGUUGUAUAG---

12652 0,-2

-----UGAGGUAGUAGGUUGUAUAGa--

322 0,-2

-----UGAGGUAGUAGGUUGUAUAGaa---

44 0,-2

-----UGAGGUAGUAGGUUGUAUAGau---

137 0,-2

-----UGAGGUAGUAGGUUGUAUAGU--

48817 0,-1

-----UGAGGUAGUAGGUUGUAUAGUa---

1812 0,-1

-----UGAGGUAGUAGGUUGUAUAGUaa--

2410 0,-1

-----UGAGGUAGUAGGUUGUAUAGUau--

558 0,-1

-----UGAGGUAGUAGGUUGUAUAGUU---

210971 0,0

-----UGAGGUAGUAGGUUGUAUAGUUa--

28624 0,0

-----UGAGGUAGUAGGUUGUAUAGUUaa---

3725 0,0

-----UGAGGUAGUAGGUUGUAUAGUUau---

1190 0,0

-----UGAGGUAGUAGGUUGUAUAGUUU--

30598 0,1

-----UGAGGUAGUAGGUUGUAUAGUUUU---

634 0,2

-----UGAGGUAGUAGGUUGUAUAGUUUUu--

11 0,2

-----UGAGGUAGUAGGUUGUAUAGUUUUA--

5 0,3

------GAGGUAGUAGGUUGUAUA--

11 1,-3

------GAGGUAGUAGGUUGUAUAG---

31 1,-2

------GAGGUAGUAGGUUGUAUAGau---

2 1,-2

------GAGGUAGUAGGUUGUAUAGU--

133 1,-1

------GAGGUAGUAGGUUGUAUAGUa---

4 1,-1

------GAGGUAGUAGGUUGUAUAGUaa--

11 1,-1

------GAGGUAGUAGGUUGUAUAGUau--

2 1,-1

------GAGGUAGUAGGUUGUAUAGUU---

608 1,0

------GAGGUAGUAGGUUGUAUAGUUa--

72 1,0

------GAGGUAGUAGGUUGUAUAGUUaa---

14 1,0

------GAGGUAGUAGGUUGUAUAGUUau---

4 1,0

------GAGGUAGUAGGUUGUAUAGUUU--

100 1,1

------GAGGUAGUAGGUUGUAUAGUUUU---

3 1,2

-------AGGUAGUAGGUUGUAUAGUU---

45 2,0

-------AGGUAGUAGGUUGUAUAGUUa--

8 2,0

-------AGGUAGUAGGUUGUAUAGUUU--

6 2,1

--CUAUACAAUCUACUGUCUUU----

4 0,-1

--CUAUACAAUCUACUGUCUUUC---

16 0,0

--CUAUACAAUCUACUGUCUUUCa--

2 0,0

--CUAUACAAUCUACUGUCUUUCu--

20 0,0
--CUAUACAAUCUACUGUCUUUCC--
3 0,1
---UAUACAAUCUACUGUCUUUCu--
2 1,0
---UAUACAAUCUACUGUCUUUCC--
2 1,1
---UAUACAAUCUACUGUCUUUCCUu
3 1,2
--AUACAAUCUACUGUCUUUCCU-
3 2,2

>hsa-let-7a-2 MA:5-26;mi:50-71 SRX018959
agguUGAGGUAGUAGGUUGUAUAGUUuagaauuacaucaagggagauaaCUGUACAGCCUCCUAGCUUUCCu
(((..(((.(((.(((((((((((((.....(..(.....)..)...))))))))))))).))).))).)))
--GUUGAGGUAGUAGGUUGUAUA--- 2
-2,-3
---UUGAGGUAGUAGGUUGUAUA--- 2
-1,-3
---UUGAGGUAGUAGGUUGUAUAG-- 7
-1,-2
---UUGAGGUAGUAGGUUGUAUAGU--- 8
-1,-1
---UUGAGGUAGUAGGUUGUAUAGUU-- 5
-1,0
----UGAGGUAGUAGGUUGUAU-- 1223
0,-4
----UGAGGUAGUAGGUUGUAUu--- 109
0,-4
----UGAGGUAGUAGGUUGUAUuu-- 74
0,-4
----UGAGGUAGUAGGUUGUAUA--- 6825
0,-3
----UGAGGUAGUAGGUUGUAUAa-- 312
0,-3
----UGAGGUAGUAGGUUGUAUAaa--- 101
0,-3
----UGAGGUAGUAGGUUGUAUAau--- 11

0,-3
----UGAGGUAGUAGGUUGUAUAu-- 128
0,-3
----UGAGGUAGUAGGUUGUAUAuu--- 20
0,-3
----UGAGGUAGUAGGUUGUAUAG--
12652 0,-2
----UGAGGUAGUAGGUUGUAUAGa--- 322
0,-2
----UGAGGUAGUAGGUUGUAUAGaa-- 44
0,-2
----UGAGGUAGUAGGUUGUAUAGau-- 137
0,-2
----UGAGGUAGUAGGUUGUAUAGU---
48817 0,-1
----UGAGGUAGUAGGUUGUAUAGUa-- 1812
0,-1
----UGAGGUAGUAGGUUGUAUAGUaa--- 2410
0,-1
----UGAGGUAGUAGGUUGUAUAGUau--- 558
0,-1
----UGAGGUAGUAGGUUGUAUAGUU--
210971 0,0
----UGAGGUAGUAGGUUGUAUAGUUa---
28624 0,0
----UGAGGUAGUAGGUUGUAUAGUUaa-- 3725
0,0
----UGAGGUAGUAGGUUGUAUAGUUau-- 1190
0,0
----UGAGGUAGUAGGUUGUAUAGUUU---
30598 0,1
----UGAGGUAGUAGGUUGUAUAGUUUA-- 290
0,2
----UGAGGUAGUAGGUUGUAUAGUUUAa--- 20
0,2
----UGAGGUAGUAGGUUGUAUAGUUUAu--- 5
0,2
-----GAGGUAGUAGGUUGUAUA--- 11

1,-3
-----GAGGUAGUAGGUUGUAUAG-- 31
1,-2
-----GAGGUAGUAGGUUGUAUAGau-- 2
1,-2
-----GAGGUAGUAGGUUGUAUAGU--- 133
1,-1
-----GAGGUAGUAGGUUGUAUAGUa-- 4
1,-1
-----GAGGUAGUAGGUUGUAUAGUaa--- 11
1,-1
-----GAGGUAGUAGGUUGUAUAGUau--- 2
1,-1
-----GAGGUAGUAGGUUGUAUAGUU-- 608
1,0
-----GAGGUAGUAGGUUGUAUAGUUa--- 72
1,0
-----GAGGUAGUAGGUUGUAUAGUUaa-- 14
1,0
-----GAGGUAGUAGGUUGUAUAGUUau-- 4
1,0
-----GAGGUAGUAGGUUGUAUAGUUU--- 100
1,1
------AGGUAGUAGGUUGUAUAGUU-- 45
2,0
------AGGUAGUAGGUUGUAUAGUUa--- 8
2,0
------AGGUAGUAGGUUGUAUAGUUU--- 6
2,1

>hsa-let-7a-3 MA:4-25;mi:52-72 SRX018959
gggUGAGGUAGUAGGUUGUAUAGUUuggggcucugcccugcuaugggauaaCUAUACAAUCUACUGUCUUUCcu
(((.(((..((((((((((((((((((((((...)))))).........))))))))))))))))..))).)))
--GUGAGGUAGUAGGUUGUAU--- 2
-1,-4
--GUGAGGUAGUAGGUUGUAUA-- 4
-1,-3
--GUGAGGUAGUAGGUUGUAUAG--- 8

-1,-2

--GUGAGGUAGUAGGUUGUAUAGa-- 2

-1,-2

--GUGAGGUAGUAGGUUGUAUAGU-- 41

-1,-1

--GUGAGGUAGUAGGUUGUAUAGUU--- 37

-1,0

---UGAGGUAGUAGGUUGUAU--- 1223

0,-4

---UGAGGUAGUAGGUUGUAUu-- 109

0,-4

---UGAGGUAGUAGGUUGUAUuu--- 74

0,-4

---UGAGGUAGUAGGUUGUAUA-- 6825

0,-3

---UGAGGUAGUAGGUUGUAUAa--- 312

0,-3

---UGAGGUAGUAGGUUGUAUAaa-- 101

0,-3

---UGAGGUAGUAGGUUGUAUAau-- 11

0,-3

---UGAGGUAGUAGGUUGUAUAu--- 128

0,-3

---UGAGGUAGUAGGUUGUAUAuu-- 20

0,-3

---UGAGGUAGUAGGUUGUAUAG---

12652 0,-2

---UGAGGUAGUAGGUUGUAUAGa-- 322

0,-2

---UGAGGUAGUAGGUUGUAUAGaa--- 44

0,-2

---UGAGGUAGUAGGUUGUAUAGau--- 137

0,-2

---UGAGGUAGUAGGUUGUAUAGU--

48817 0,-1

---UGAGGUAGUAGGUUGUAUAGUa--- 1812

0,-1

---UGAGGUAGUAGGUUGUAUAGUaa-- 2410

0,-1

---UGAGGUAGUAGGUUGUAUAGUau-- 558

0,-1

---UGAGGUAGUAGGUUGUAUAGUU---

210971 0,0

---UGAGGUAGUAGGUUGUAUAGUUa--

28624 0,0

---UGAGGUAGUAGGUUGUAUAGUUaa--- 3725

0,0

---UGAGGUAGUAGGUUGUAUAGUUau--- 1190

0,0

---UGAGGUAGUAGGUUGUAUAGUUU--

30598 0,1

---UGAGGUAGUAGGUUGUAUAGUUUG--- 7

0,2

----GAGGUAGUAGGUUGUAUA-- 11

1,-3

----GAGGUAGUAGGUUGUAUAG--- 31

1,-2

----GAGGUAGUAGGUUGUAUAGau--- 2

1,-2

----GAGGUAGUAGGUUGUAUAGU-- 133

1,-1

----GAGGUAGUAGGUUGUAUAGUa--- 4

1,-1

----GAGGUAGUAGGUUGUAUAGUaa-- 11

1,-1

----GAGGUAGUAGGUUGUAUAGUau-- 2

1,-1

----GAGGUAGUAGGUUGUAUAGUU--- 608

1,0

----GAGGUAGUAGGUUGUAUAGUUa-- 72

1,0

----GAGGUAGUAGGUUGUAUAGUUaa--- 14

1,0

----GAGGUAGUAGGUUGUAUAGUUau--- 4

1,0

----GAGGUAGUAGGUUGUAUAGUUU-- 100

1,1
-----AGGUAGUAGGUUGUAUAGUU--- 45
2,0
-----AGGUAGUAGGUUGUAUAGUUa-- 8
2,0
-----AGGUAGUAGGUUGUAUAGUUU-- 6
2,1
-----AGGUAGUAGGUUGUAUAGUUUG--- 3
2,2
---CUAUACAAUCUACUGUCUUU--- 4
0,-1
---CUAUACAAUCUACUGUCUUUC-- 16
0,0
---CUAUACAAUCUACUGUCUUUCa- 2
0,0
---CUAUACAAUCUACUGUCUUUCu- 20
0,0
---CUAUACAAUCUACUGUCUUUCC- 3
0,1
--UAUACAAUCUACUGUCUUUCu- 2
1,0
--UAUACAAUCUACUGUCUUUCC- 2
1,1
--UAUACAAUCUACUGUCUUUCCUu 3
1,2
---AUACAAUCUACUGUCUUUCCU 3
2,2

>hsa-let-7b MA:6-27;mi:60-81 SRX018959
cggggUGAGGUAGUAGGUUGUGUGGUUucagggcagugauguugccccucggaagauaaCUAUACAACCUACUGCCUUCC
Cug
(((((.(((((((((((((((((((((..............................)))))))))))))))))))))))
)))
-----UGAGGUAGUAGGUUGUGU---
--- 5239 0,-4
-----UGAGGUAGUAGGUUGUGUa--
--- 28 0,-4
-----UGAGGUAGUAGGUUGUGUaa---

--- 165 0,-4
-----UGAGGUAGUAGGUUGUGUau--
--- 17 0,-4
-----UGAGGUAGUAGGUUGUGUu---
--- 31 0,-4
-----UGAGGUAGUAGGUUGUGUuu--
--- 16 0,-4
-----UGAGGUAGUAGGUUGUGUG---
--- 18866 0,-3
-----UGAGGUAGUAGGUUGUGUGa--
--- 135 0,-3
-----UGAGGUAGUAGGUUGUGUGaa---
--- 14 0,-3
-----UGAGGUAGUAGGUUGUGUGau---
--- 8 0,-3
-----UGAGGUAGUAGGUUGUGUGu--
--- 150 0,-3
-----UGAGGUAGUAGGUUGUGUGuu---
--- 38 0,-3
-----UGAGGUAGUAGGUUGUGUGG--
--- 4468 0,-2
-----UGAGGUAGUAGGUUGUGUGGa---
--- 62 0,-2
-----UGAGGUAGUAGGUUGUGUGGaa--
--- 39 0,-2
-----UGAGGUAGUAGGUUGUGUGGau--
--- 20 0,-2
-----UGAGGUAGUAGGUUGUGUGGU---
--- 6090 0,-1
-----UGAGGUAGUAGGUUGUGUGGUa--
--- 856 0,-1
-----UGAGGUAGUAGGUUGUGUGGUaa---
--- 3546 0,-1
-----UGAGGUAGUAGGUUGUGUGGUau---
--- 3049 0,-1
-----UGAGGUAGUAGGUUGUGUGGUU--
--- 23759 0,0
-----UGAGGUAGUAGGUUGUGUGGUUa---

--- 10784 0,0
-----UGAGGUAGUAGGUUGUGUGGUUaa---
--- 4401 0,0
-----UGAGGUAGUAGGUUGUGUGGUUau---
--- 9419 0,0
-----UGAGGUAGUAGGUUGUGUGGUUU--
--- 39898 0,1
-----UGAGGUAGUAGGUUGUGUGGUUUa---
--- 1904 0,1
-----UGAGGUAGUAGGUUGUGUGGUUUaa--
--- 314 0,1
-----UGAGGUAGUAGGUUGUGUGGUUUau--
--- 510 0,1
-----UGAGGUAGUAGGUUGUGUGGUUUu---
--- 7278 0,1
-----UGAGGUAGUAGGUUGUGUGGUUUuu--
--- 284 0,1
-----UGAGGUAGUAGGUUGUGUGGUUUC---
--- 51 0,2
-----UGAGGUAGUAGGUUGUGUGGUUUCA--
--- 3 0,3
------GAGGUAGUAGGUUGUGUG--
--- 73 1,-3
------GAGGUAGUAGGUUGUGUGG---
--- 23 1,-2
------GAGGUAGUAGGUUGUGUGGU--
--- 16 1,-1
------GAGGUAGUAGGUUGUGUGGUa---
--- 3 1,-1
------GAGGUAGUAGGUUGUGUGGUaa--
--- 20 1,-1
------GAGGUAGUAGGUUGUGUGGUau--
--- 17 1,-1
------GAGGUAGUAGGUUGUGUGGUU---
--- 126 1,0
------GAGGUAGUAGGUUGUGUGGUUa--
--- 70 1,0
------GAGGUAGUAGGUUGUGUGGUUaa---

--- 23 1,0
------GAGGUAGUAGGUUGUGUGGUUau--
--- 35 1,0
------GAGGUAGUAGGUUGUGUGGUUU---
--- 232 1,1
------GAGGUAGUAGGUUGUGUGGUUUa--
--- 6 1,1
------GAGGUAGUAGGUUGUGUGGUUUau---
--- 4 1,1
------GAGGUAGUAGGUUGUGUGGUUUu--
--- 24 1,1
------GAGGUAGUAGGUUGUGUGGUUUuu---
--- 2 1,1
-------AGGUAGUAGGUUGUGUGGUU--
--- 3 2,0
-------AGGUAGUAGGUUGUGUGGUUa---
--- 2 2,0
-------AGGUAGUAGGUUGUGUGGUUaa--
--- 2 2,0
-------AGGUAGUAGGUUGUGUGGUUU---
--- 10 2,1
-------AGGUAGUAGGUUGUGUGGUUUu--
--- 6 2,1
--CUAUACAACCUACUGCCUU--
--- 3 0,-3
--CUAUACAACCUACUGCCUUC-
--- 5 0,-2
--CUAUACAACCUACUGCCUUCC
--- 19 0,-1
--CUAUACAACCUACUGCCUUCC
a-- 9 0,-1
--CUAUACAACCUACUGCCUUCC
au- 2 0,-1
--CUAUACAACCUACUGCCUUCC
u-- 62 0,-1
--CUAUACAACCUACUGCCUUCC
uu- 3 0,-1
---UAUACAACCUACUGCCUUCC

--- 2 1,-1
--UAUACAACCUACUGCCUUCC
u-- 4 1,-1

>hsa-let-7c MA:11-32;mi:33-54 SRX018959
gcauccggguUGAGGUAGUAGGUUGUAUGGUUUAGAGUUACACCCUGGGAGUUAacuguacaaccuucuagcuuuccuug
gagc
((.((((((..(((.(((.((((((((((((..((.(..(.....)..).)))))))))))))))).))).))).))))
))))
--------GUUGAGGUAGUAGGUUGUAUGGUU--
---- 13 -2,0
---------UUGAGGUAGUAGGUUGUAUG---
---- 17 -1,-3
---------UUGAGGUAGUAGGUUGUAUGG--
---- 59 -1,-2
---------UUGAGGUAGUAGGUUGUAUGGaa--
---- 2 -1,-2
---------UUGAGGUAGUAGGUUGUAUGGU---
---- 106 -1,-1
---------UUGAGGUAGUAGGUUGUAUGGUa--
---- 38 -1,-1
---------UUGAGGUAGUAGGUUGUAUGGUaa---
---- 8 -1,-1
---------UUGAGGUAGUAGGUUGUAUGGUau---
---- 21 -1,-1
---------UUGAGGUAGUAGGUUGUAUGGUU--
---- 348 -1,0
---------UUGAGGUAGUAGGUUGUAUGGUUa---
---- 8 -1,0
---------UUGAGGUAGUAGGUUGUAUGGUUau--
---- 4 -1,0
---------UUGAGGUAGUAGGUUGUAUGGUUU---
---- 29 -1,1
---------UUGAGGUAGUAGGUUGUAUGGUUUu--
---- 2 -1,1
----------UGAGGUAGUAGGUUGUAU--
---- 1223 0,-4
----------UGAGGUAGUAGGUUGUAUu---

---- 109 0,-4

----------UGAGGUAGUAGGUUGUAUuu--

---- 74 0,-4

----------UGAGGUAGUAGGUUGUAUG---

---- 3459 0,-3

----------UGAGGUAGUAGGUUGUAUGa--

---- 13 0,-3

----------UGAGGUAGUAGGUUGUAUGaa---

---- 3 0,-3

----------UGAGGUAGUAGGUUGUAUGau---

---- 2 0,-3

----------UGAGGUAGUAGGUUGUAUGu--

---- 28 0,-3

----------UGAGGUAGUAGGUUGUAUGuu---

---- 17 0,-3

----------UGAGGUAGUAGGUUGUAUGG--

---- 6724 0,-2

----------UGAGGUAGUAGGUUGUAUGGa---

---- 97 0,-2

----------UGAGGUAGUAGGUUGUAUGGaa--

---- 127 0,-2

----------UGAGGUAGUAGGUUGUAUGGau--

---- 94 0,-2

----------UGAGGUAGUAGGUUGUAUGGU---

---- 17597 0,-1

----------UGAGGUAGUAGGUUGUAUGGUa--

---- 3987 0,-1

----------UGAGGUAGUAGGUUGUAUGGUaa---

---- 2728 0,-1

----------UGAGGUAGUAGGUUGUAUGGUau---

---- 2099 0,-1

----------UGAGGUAGUAGGUUGUAUGGUU--

---- 121174 0,0

----------UGAGGUAGUAGGUUGUAUGGUUa---

---- 9963 0,0

----------UGAGGUAGUAGGUUGUAUGGUUaa--

---- 1897 0,0

----------UGAGGUAGUAGGUUGUAUGGUUau--

---- 2060 0,0

----------UGAGGUAGUAGGUUGUAUGGUUU--

---- 18961 0,1

----------UGAGGUAGUAGGUUGUAUGGUUUu---

---- 1139 0,1

----------UGAGGUAGUAGGUUGUAUGGUUUuu--

---- 26 0,1

----------UGAGGUAGUAGGUUGUAUGGUUUA---

---- 476 0,2

----------UGAGGUAGUAGGUUGUAUGGUUUAa--

---- 49 0,2

----------UGAGGUAGUAGGUUGUAUGGUUUAu--

---- 29 0,2

----------UGAGGUAGUAGGUUGUAUGGUUUAG--

---- 3 0,3

-----------GAGGUAGUAGGUUGUAUG--

---- 61 1,-3

-----------GAGGUAGUAGGUUGUAUGG---

---- 46 1,-2

-----------GAGGUAGUAGGUUGUAUGGU--

---- 110 1,-1

-----------GAGGUAGUAGGUUGUAUGGUa---

---- 28 1,-1

-----------GAGGUAGUAGGUUGUAUGGUaa--

---- 21 1,-1

-----------GAGGUAGUAGGUUGUAUGGUau--

---- 19 1,-1

-----------GAGGUAGUAGGUUGUAUGGUU---

---- 892 1,0

-----------GAGGUAGUAGGUUGUAUGGUUa--

---- 86 1,0

-----------GAGGUAGUAGGUUGUAUGGUUaa---

---- 29 1,0

-----------GAGGUAGUAGGUUGUAUGGUUau---

---- 26 1,0

-----------GAGGUAGUAGGUUGUAUGGUUU--

---- 183 1,1

-----------GAGGUAGUAGGUUGUAUGGUUUu---

----   11 1,1
-----------GAGGUAGUAGGUUGUAUGGUUUA--
----   2   1,2
------------AGGUAGUAGGUUGUAUGG--
----   2   2,-2
------------AGGUAGUAGGUUGUAUGGU---
----   2   2,-1
------------AGGUAGUAGGUUGUAUGGUU--
----   17 2,0
------------AGGUAGUAGGUUGUAUGGUUa---
----   2   2,0
------------AGGUAGUAGGUUGUAUGGUUU---
----   13 2,1

>hsa-let-7d MA:8-29;mi:62-83   SRX018959
ccuaggaAGAGGUAGUAGGUUGCAUAGUUuuagggcagggauuuugcccacaaggagguaaCUAUACGACCUGCUGCCUU
UCUuagg
((((((.(((((((((((((.((((((............................)))))).)))))))))))))
)))))))
------AAGAGGUAGUAGGUUGCAU---
------- 3   -1,-4
------AAGAGGUAGUAGGUUGCAUA--
------- 2   -1,-3
------AAGAGGUAGUAGGUUGCAUAG---
------- 2   -1,-2
------AAGAGGUAGUAGGUUGCAUAGU--
------- 10   -1,-1
------AAGAGGUAGUAGGUUGCAUAGUU---
------- 31   -1,0
------AAGAGGUAGUAGGUUGCAUAGUUa--
------- 4   -1,0
-------AGAGGUAGUAGGUUGCAU---
------- 1192   0,-4
-------AGAGGUAGUAGGUUGCAUu--
------- 58   0,-4
-------AGAGGUAGUAGGUUGCAUuu---
------- 62   0,-4
-------AGAGGUAGUAGGUUGCAUA--

------- 939 0,-3

-------AGAGGUAGUAGGUUGCAUAa---

------- 78 0,-3

-------AGAGGUAGUAGGUUGCAUAaa--

------- 6 0,-3

-------AGAGGUAGUAGGUUGCAUAau--

------- 3 0,-3

-------AGAGGUAGUAGGUUGCAUAu---

------- 6 0,-3

-------AGAGGUAGUAGGUUGCAUAuu--

------- 9 0,-3

-------AGAGGUAGUAGGUUGCAUAG---

------- 2441 0,-2

-------AGAGGUAGUAGGUUGCAUAGa--

------- 70 0,-2

-------AGAGGUAGUAGGUUGCAUAGaa---

------- 35 0,-2

-------AGAGGUAGUAGGUUGCAUAGau---

------- 129 0,-2

-------AGAGGUAGUAGGUUGCAUAGU--

------- 4632 0,-1

-------AGAGGUAGUAGGUUGCAUAGUa---

------- 891 0,-1

-------AGAGGUAGUAGGUUGCAUAGUaa--

------- 212 0,-1

-------AGAGGUAGUAGGUUGCAUAGUau--

------- 151 0,-1

-------AGAGGUAGUAGGUUGCAUAGUU---

------- 75040 0,0

-------AGAGGUAGUAGGUUGCAUAGUUa--

------- 6183 0,0

-------AGAGGUAGUAGGUUGCAUAGUUaa---

------- 302 0,0

-------AGAGGUAGUAGGUUGCAUAGUUau---

------- 189 0,0

-------AGAGGUAGUAGGUUGCAUAGUUU--

------- 11217 0,1

-------AGAGGUAGUAGGUUGCAUAGUUUa---

------- 92 0,1
-------AGAGGUAGUAGGUUGCAUAGUUUaa--
------- 3 0,1
-------AGAGGUAGUAGGUUGCAUAGUUUau--
------- 2 0,1
-------AGAGGUAGUAGGUUGCAUAGUUUU---
------- 487 0,2
-------AGAGGUAGUAGGUUGCAUAGUUUUu--
------- 22 0,2
-------AGAGGUAGUAGGUUGCAUAGUUUUA--
------- 6 0,3
--------GAGGUAGUAGGUUGCAUA--
------- 6 1,-3
--------GAGGUAGUAGGUUGCAUAG---
------- 2 1,-2
--------GAGGUAGUAGGUUGCAUAGU--
------- 15 1,-1
--------GAGGUAGUAGGUUGCAUAGUa---
------- 3 1,-1
--------GAGGUAGUAGGUUGCAUAGUaa--
------- 2 1,-1
--------GAGGUAGUAGGUUGCAUAGUU---
------- 188 1,0
--------GAGGUAGUAGGUUGCAUAGUUa--
------- 41 1,0
--------GAGGUAGUAGGUUGCAUAGUUaa---
------- 3 1,0
--------GAGGUAGUAGGUUGCAUAGUUU--
------- 52 1,1
--------GAGGUAGUAGGUUGCAUAGUUUU---
------- 4 1,2
---------AGGUAGUAGGUUGCAUAGUU---
------- 35 2,0
---------AGGUAGUAGGUUGCAUAGUUa--
------- 5 2,0
---------AGGUAGUAGGUUGCAUAGUUU--
------- 6 2,1
---CUAUACGACCUGCUGCCUU

UC----- 30 0,-1
---CUAUACGACCUGCUGCCUU
UCa---- 19 0,-1
---CUAUACGACCUGCUGCCUU
UCU---- 93 0,0
---CUAUACGACCUGCUGCCUU
UCUa--- 2   0,0
---CUAUACGACCUGCUGCCUU
UCUU--- 2   0,1
--UAUACGACCUGCUGCCUU
UC----- 4   1,-1
--UAUACGACCUGCUGCCUU
UCa---- 3   1,-1
--UAUACGACCUGCUGCCUU
UCU---- 14 1,0

>hsa-let-7e MA:8-29;mi:53-74   SRX018959
cccgggcUGAGGUAGGAGGUUGUAUAGUUgaggaggacacccaaggagaucaCUAUACGGCCUCCUAGCUUUCCccagg
((.(((..(((.((((((((((((((((.((...((....)).......)))))))))))))))))).)))..))).))
------CUGAGGUAGGAGGUUGUAUAG--
    4   -1,-2
------CUGAGGUAGGAGGUUGUAUAGU---
    15 -1,-1
------CUGAGGUAGGAGGUUGUAUAGUaa---
    2   -1,-1
------CUGAGGUAGGAGGUUGUAUAGUU--
    32 -1,0
------CUGAGGUAGGAGGUUGUAUAGUUa---
    15 -1,0
------CUGAGGUAGGAGGUUGUAUAGUUu---
    10 -1,0
------CUGAGGUAGGAGGUUGUAUAGUUG---
    2   -1,1
-------UGAGGUAGGAGGUUGUAU--
    1070   0,-4
-------UGAGGUAGGAGGUUGUAUu---
    6   0,-4
-------UGAGGUAGGAGGUUGUAUuu--

7 0,-4

-------UGAGGUAGGAGGUUGUAUA---

710 0,-3

-------UGAGGUAGGAGGUUGUAUAa--

69 0,-3

-------UGAGGUAGGAGGUUGUAUAaa---

24 0,-3

-------UGAGGUAGGAGGUUGUAUAau---

3 0,-3

-------UGAGGUAGGAGGUUGUAUAu--

5 0,-3

-------UGAGGUAGGAGGUUGUAUAuu---

3 0,-3

-------UGAGGUAGGAGGUUGUAUAG--

1408 0,-2

-------UGAGGUAGGAGGUUGUAUAGa---

91 0,-2

-------UGAGGUAGGAGGUUGUAUAGaa--

17 0,-2

-------UGAGGUAGGAGGUUGUAUAGau--

26 0,-2

-------UGAGGUAGGAGGUUGUAUAGU---

3410 0,-1

-------UGAGGUAGGAGGUUGUAUAGUa--

239 0,-1

-------UGAGGUAGGAGGUUGUAUAGUaa---

732 0,-1

-------UGAGGUAGGAGGUUGUAUAGUau---

255 0,-1

-------UGAGGUAGGAGGUUGUAUAGUU--

10849 0,0

-------UGAGGUAGGAGGUUGUAUAGUUa---

4189 0,0

-------UGAGGUAGGAGGUUGUAUAGUUaa--

710 0,0

-------UGAGGUAGGAGGUUGUAUAGUUau--

244 0,0

-------UGAGGUAGGAGGUUGUAUAGUUu---

3296 0,0

-------UGAGGUAGGAGGUUGUAUAGUUuu--

84 0,0

-------UGAGGUAGGAGGUUGUAUAGUUG--

569 0,1

-------UGAGGUAGGAGGUUGUAUAGUUGu---

81 0,1

-------UGAGGUAGGAGGUUGUAUAGUUGuu--

2 0,1

-------UGAGGUAGGAGGUUGUAUAGUUGA---

34 0,2

--------GAGGUAGGAGGUUGUAUAG---

3 1,-2

--------GAGGUAGGAGGUUGUAUAGU--

5 1,-1

--------GAGGUAGGAGGUUGUAUAGUU---

22 1,0

--------GAGGUAGGAGGUUGUAUAGUUa--

10 1,0

--------GAGGUAGGAGGUUGUAUAGUUaa---

2 1,0

--------GAGGUAGGAGGUUGUAUAGUUu--

6 1,0

--ACUAUACGGCCUCCUAGCUUUCCa----

2 -1,0

---CUAUACGGCCUCCUAGCUUUC------

3 0,-1

---CUAUACGGCCUCCUAGCUUUCC-----

7 0,0

>hsa-let-7f-1 MA:7-28;mi:63-84 SRX018959

ucagagUGAGGUAGUAGAUUGUAUAGUUgugggguagugauuuuacccuguucaggagauaaCUAUACAAUCUAUUGCCUUCCCuga

((((.(..((((((((((((((((((((((((((((.....))))))).........)))))))))))))))))))))..)))))

------UGAGGUAGUAGAUUGUAU--- 771 0,-4

------UGAGGUAGUAGAUUGUAUu---

------- 24 0,-4

------UGAGGUAGUAGAUUGUAUuu---

------- 54 0,-4

------UGAGGUAGUAGAUUGUAUA--

------- 8549 0,-3

------UGAGGUAGUAGAUUGUAUAa---

------- 102 0,-3

------UGAGGUAGUAGAUUGUAUAaa--

------- 44 0,-3

------UGAGGUAGUAGAUUGUAUAau--

------- 11 0,-3

------UGAGGUAGUAGAUUGUAUAu---

------- 23 0,-3

------UGAGGUAGUAGAUUGUAUAuu--

------- 8 0,-3

------UGAGGUAGUAGAUUGUAUAG---

------- 5817 0,-2

------UGAGGUAGUAGAUUGUAUAGa--

------- 225 0,-2

------UGAGGUAGUAGAUUGUAUAGaa---

------- 34 0,-2

------UGAGGUAGUAGAUUGUAUAGau---

------- 67 0,-2

------UGAGGUAGUAGAUUGUAUAGU--

------- 34436 0,-1

------UGAGGUAGUAGAUUGUAUAGUa---

------- 948 0,-1

------UGAGGUAGUAGAUUGUAUAGUaa--

------- 3310 0,-1

------UGAGGUAGUAGAUUGUAUAGUau--

------- 472 0,-1

------UGAGGUAGUAGAUUGUAUAGUU---

------- 111267 0,0

------UGAGGUAGUAGAUUGUAUAGUUa--

------- 44260 0,0

------UGAGGUAGUAGAUUGUAUAGUUaa---

------- 4090 0,0

------UGAGGUAGUAGAUUGUAUAGUUau---

------- 1082 0,0
------UGAGGUAGUAGAUUGUAUAGUUG---
------- 1299 0,1
------UGAGGUAGUAGAUUGUAUAGUUGa--
------- 58 0,1
------UGAGGUAGUAGAUUGUAUAGUUGaa---
------- 2 0,1
------UGAGGUAGUAGAUUGUAUAGUUGU--
------- 74 0,2
-------GAGGUAGUAGAUUGUAUA---
------- 17 1,-3
-------GAGGUAGUAGAUUGUAUAu--
------- 2 1,-3
-------GAGGUAGUAGAUUGUAUAG--
------- 40 1,-2
-------GAGGUAGUAGAUUGUAUAGU---
------- 117 1,-1
-------GAGGUAGUAGAUUGUAUAGUaa---
------- 16 1,-1
-------GAGGUAGUAGAUUGUAUAGUU--
------- 558 1,0
-------GAGGUAGUAGAUUGUAUAGUUa---
------- 197 1,0
-------GAGGUAGUAGAUUGUAUAGUUaa--
------- 31 1,0
-------GAGGUAGUAGAUUGUAUAGUUau--
------- 10 1,0
-------GAGGUAGUAGAUUGUAUAGUUG---
------- 7 1,1
--------AGGUAGUAGAUUGUAUAG--
------- 2 2,-2
--------AGGUAGUAGAUUGUAUAGU---
------- 3 2,-1
--------AGGUAGUAGAUUGUAUAGUU--
------- 22 2,0
--------AGGUAGUAGAUUGUAUAGUUa---
------- 4 2,0
--------AGGUAGUAGAUUGUAUAGUUG---

------- 10 2,1
--------AGGUAGUAGAUUGUAUAGUUGU---
------- 27 2,2
---CUAUACAAUCUAUUGCCU
U------ 6   0,-3
---CUAUACAAUCUAUUGCCU
UCC---- 2   0,-1
---CUAUACAAUCUAUUGCCU
UCCu--- 4   0,-1

>hsa-let-7f-2   MA:8-29;mi:58-79   SRX018959
ugugggaUGAGGUAGUAGAUUGUAUAGUUuuagggucauaccccaucuuggagauaaCUAUACAGUCUACUGUCUUUCCc
acg
.((((((.(((..((((((((((((((((.........................))))))))))))))))..)))))))
)).
------AUGAGGUAGUAGAUUGUAUA---
--- 2   -1,-3
------AUGAGGUAGUAGAUUGUAUAG--
--- 2   -1,-2
------AUGAGGUAGUAGAUUGUAUAGU---
--- 48 -1,-1
------AUGAGGUAGUAGAUUGUAUAGUU--
--- 31 -1,0
------AUGAGGUAGUAGAUUGUAUAGUUa---
--- 3   -1,0
------AUGAGGUAGUAGAUUGUAUAGUUU---
--- 4   -1,1
-------UGAGGUAGUAGAUUGUAU--
--- 771 0,-4
-------UGAGGUAGUAGAUUGUAUu---
--- 24 0,-4
-------UGAGGUAGUAGAUUGUAUuu--
--- 54 0,-4
-------UGAGGUAGUAGAUUGUAUA---
--- 8549   0,-3
-------UGAGGUAGUAGAUUGUAUAa--
--- 102 0,-3
-------UGAGGUAGUAGAUUGUAUAaa---

--- 44	0,-3
-------UGAGGUAGUAGAUUGUAUAau---
--- 11	0,-3
-------UGAGGUAGUAGAUUGUAUAu--
--- 23	0,-3
-------UGAGGUAGUAGAUUGUAUAuu---
--- 8	0,-3
-------UGAGGUAGUAGAUUGUAUAG--
--- 5817	0,-2
-------UGAGGUAGUAGAUUGUAUAGa---
--- 225	0,-2
-------UGAGGUAGUAGAUUGUAUAGaa--
--- 34	0,-2
-------UGAGGUAGUAGAUUGUAUAGau--
--- 67	0,-2
-------UGAGGUAGUAGAUUGUAUAGU---
--- 34436	0,-1
-------UGAGGUAGUAGAUUGUAUAGUa--
--- 948	0,-1
-------UGAGGUAGUAGAUUGUAUAGUaa---
--- 3310	0,-1
-------UGAGGUAGUAGAUUGUAUAGUau---
--- 472	0,-1
-------UGAGGUAGUAGAUUGUAUAGUU--
--- 111267	0,0
-------UGAGGUAGUAGAUUGUAUAGUUa---
--- 44260	0,0
-------UGAGGUAGUAGAUUGUAUAGUUaa--
--- 4090	0,0
-------UGAGGUAGUAGAUUGUAUAGUUau--
--- 1082	0,0
-------UGAGGUAGUAGAUUGUAUAGUUU---
--- 26371	0,1
-------UGAGGUAGUAGAUUGUAUAGUUUa--
--- 89	0,1
-------UGAGGUAGUAGAUUGUAUAGUUUaa---
--- 2	0,1
-------UGAGGUAGUAGAUUGUAUAGUUUau---

--- 4   0,1

-------UGAGGUAGUAGAUUGUAUAGUUUU---

--- 310 0,2

-------UGAGGUAGUAGAUUGUAUAGUUUUu--

--- 6   0,2

-------UGAGGUAGUAGAUUGUAUAGUUUUA--

--- 2   0,3

--------GAGGUAGUAGAUUGUAUA--

--- 17   1,-3

--------GAGGUAGUAGAUUGUAUAu---

--- 2   1,-3

--------GAGGUAGUAGAUUGUAUAG---

--- 40   1,-2

--------GAGGUAGUAGAUUGUAUAGU--

--- 117 1,-1

--------GAGGUAGUAGAUUGUAUAGUaa--

--- 16   1,-1

--------GAGGUAGUAGAUUGUAUAGUU---

--- 558 1,0

--------GAGGUAGUAGAUUGUAUAGUUa--

--- 197 1,0

--------GAGGUAGUAGAUUGUAUAGUUaa---

--- 31   1,0

--------GAGGUAGUAGAUUGUAUAGUUau---

--- 10   1,0

--------GAGGUAGUAGAUUGUAUAGUUU--

--- 155 1,1

--------GAGGUAGUAGAUUGUAUAGUUUU---

--- 2   1,2

---------AGGUAGUAGAUUGUAUAG---

--- 2   2,-2

---------AGGUAGUAGAUUGUAUAGU--

--- 3   2,-1

---------AGGUAGUAGAUUGUAUAGUU---

--- 22   2,0

---------AGGUAGUAGAUUGUAUAGUUa--

--- 4   2,0

---------AGGUAGUAGAUUGUAUAGUUU--

--- 8	2,1

>hsa-let-7g MA:5-26;mi:62-82	SRX018959
aggcUGAGGUAGUAGUUUGUACAGUUugagggucuaugauaccacccgguacaggagauaaCUGUACAGGCCACUGCCUU
GCca
.(((.((((((((.(((((((((((.....((((.((.((((....))))))..))))))))))))))))).))))))))
))).
---CUGAGGUAGUAGUUUGUA---
----	2	-1,-5
---CUGAGGUAGUAGUUUGUACA---
----	3	-1,-3
---CUGAGGUAGUAGUUUGUACAG--
----	3	-1,-2
---CUGAGGUAGUAGUUUGUACAGU---
----	37	-1,-1
---CUGAGGUAGUAGUUUGUACAGUU--
----	63	-1,0
---CUGAGGUAGUAGUUUGUACAGUUa---
----	3	-1,0
---CUGAGGUAGUAGUUUGUACAGUUU---
----	8	-1,1
----UGAGGUAGUAGUUUGUAC--
----	56	0,-4
----UGAGGUAGUAGUUUGUACA---
----	132	0,-3
----UGAGGUAGUAGUUUGUACAa--
----	23	0,-3
----UGAGGUAGUAGUUUGUACAaa---
----	3	0,-3
----UGAGGUAGUAGUUUGUACAu--
----	5	0,-3
----UGAGGUAGUAGUUUGUACAG--
----	573	0,-2
----UGAGGUAGUAGUUUGUACAGa---
----	35	0,-2
----UGAGGUAGUAGUUUGUACAGaa--
----	8	0,-2
----UGAGGUAGUAGUUUGUACAGau--

---- 3 0,-2

----UGAGGUAGUAGUUUGUACAGU--

---- 4133 0,-1

----UGAGGUAGUAGUUUGUACAGUa---

---- 70 0,-1

----UGAGGUAGUAGUUUGUACAGUaa--

---- 56 0,-1

----UGAGGUAGUAGUUUGUACAGUau--

---- 6 0,-1

----UGAGGUAGUAGUUUGUACAGUU---

---- 9411 0,0

----UGAGGUAGUAGUUUGUACAGUUa--

---- 1472 0,0

----UGAGGUAGUAGUUUGUACAGUUaa---

---- 102 0,0

----UGAGGUAGUAGUUUGUACAGUUau---

---- 67 0,0

----UGAGGUAGUAGUUUGUACAGUUU--

---- 1187 0,1

----UGAGGUAGUAGUUUGUACAGUUUa---

---- 13 0,1

----UGAGGUAGUAGUUUGUACAGUUUu---

---- 37 0,1

----UGAGGUAGUAGUUUGUACAGUUUG---

---- 3 0,2

-----GAGGUAGUAGUUUGUACA--

---- 2 1,-3

-----GAGGUAGUAGUUUGUACAa---

---- 2 1,-3

-----GAGGUAGUAGUUUGUACAG---

---- 9 1,-2

-----GAGGUAGUAGUUUGUACAGU--

---- 50 1,-1

-----GAGGUAGUAGUUUGUACAGUa---

---- 4 1,-1

-----GAGGUAGUAGUUUGUACAGUaa--

---- 2 1,-1

-----GAGGUAGUAGUUUGUACAGUau--

----   3   1,-1
-----GAGGUAGUAGUUUGUACAGUU--
----   146 1,0
-----GAGGUAGUAGUUUGUACAGUUa---
----   13   1,0
-----GAGGUAGUAGUUUGUACAGUUaa--
----   4   1,0
-----GAGGUAGUAGUUUGUACAGUUU---
----   16   1,1
-----GAGGUAGUAGUUUGUACAGUUUu--
----   2   1,1
------AGGUAGUAGUUUGUACAGU---
----   2   2,-1
------AGGUAGUAGUUUGUACAGUU--
----   3   2,0

>hsa-let-7i MA:6-27;mi:62-83   SRX018959
cuggcUGAGGUAGUAGUUUGUGCUGUUggucggguugugacauugcccgcuguggagauaaCUGCGCAAGCUACUGCCUU
GCUa
.((((.(((((((((((((((((.(((((.(((((.........))))))).........))).)))))))))))))))))
))))
----CUGAGGUAGUAGUUUGUGC---
----   4   -1,-4
----CUGAGGUAGUAGUUUGUGCU--
----   6   -1,-3
-----UGAGGUAGUAGUUUGUGC---
----   540 0,-4
-----UGAGGUAGUAGUUUGUGCaa---
----   2   0,-4
-----UGAGGUAGUAGUUUGUGCau---
----   3   0,-4
-----UGAGGUAGUAGUUUGUGCU--
----   2038   0,-3
-----UGAGGUAGUAGUUUGUGCUG---
----   305 0,-2
-----UGAGGUAGUAGUUUGUGCUGa--
----   7   0,-2
-----UGAGGUAGUAGUUUGUGCUGU--

----   188 0,-1
-----UGAGGUAGUAGUUUGUGCUGUa---
----   5   0,-1
-----UGAGGUAGUAGUUUGUGCUGUaa--
----   8   0,-1
-----UGAGGUAGUAGUUUGUGCUGUau--
----   4   0,-1
-----UGAGGUAGUAGUUUGUGCUGUU---
----   293 0,0
-----UGAGGUAGUAGUUUGUGCUGUUa--
----   101 0,0
-----UGAGGUAGUAGUUUGUGCUGUUaa---
----   27 0,0
-----UGAGGUAGUAGUUUGUGCUGUUau---
----   8   0,0
-----UGAGGUAGUAGUUUGUGCUGUUu--
----   192 0,0
-----UGAGGUAGUAGUUUGUGCUGUUuu---
----   28 0,0
------GAGGUAGUAGUUUGUGCU--
----   10 1,-3
------GAGGUAGUAGUUUGUGCUGU--
----   5   1,-1
------GAGGUAGUAGUUUGUGCUGUU---
----   3   1,0
------GAGGUAGUAGUUUGUGCUGUUu--
----   2   1,0
---CUGCGCAAGCUACUGCCUU
----   3   0,-3

>hsa-mir-1-1   3p:46-67   SRX018959
ugggaaacauacuucuuuauaugcccauauggaccugcuaagcuaUGGAAUGUAAAGAAGUAUGUAUcuca
.((((.((((((((((((((((..((((((((.....)))...))))).)))))))))))))))).)))).
---UGGAAUGUAAAGAAGUAU-------- 44 0,-4
---UGGAAUGUAAAGAAGUAUa------- 5   0,-4
---UGGAAUGUAAAGAAGUAUG------- 34 0,-3
---UGGAAUGUAAAGAAGUAUGU------ 29 0,-2
---UGGAAUGUAAAGAAGUAUGUA----- 99 0,-1

---UGGAAUGUAAAGAAGUAUGUAa---- 65 0,-1
---UGGAAUGUAAAGAAGUAUGUAaa--- 7 0,-1
---UGGAAUGUAAAGAAGUAUGUAU---- 1689
0,0
---UGGAAUGUAAAGAAGUAUGUAUa--- 36 0,0
---UGGAAUGUAAAGAAGUAUGUAUaa-- 2 0,0
---UGGAAUGUAAAGAAGUAUGUAUC--- 3 0,1
--GGAAUGUAAAGAAGUAUGUAa---- 2 1,-1
--GGAAUGUAAAGAAGUAUGUAU---- 20 1,0
---GAAUGUAAAGAAGUAUGUAU---- 3 2,0

>hsa-mir-1-2 3p:53-74 SRX018959
accuacucagaguacauacuucuuuauguacccauaugaacauacaaugcuaUGGAAUGUAAAGAAGUAUGUAUuuuugg
uaggc
.((((((.(((((((((((((((((((((..(((((..............))))).))))))))))))))))))))).))
)))).
--UGGAAUGUAAAGAAGUAU----------
----- 44 0,-4
--UGGAAUGUAAAGAAGUAUa---------
----- 5 0,-4
--UGGAAUGUAAAGAAGUAUG---------
----- 34 0,-3
--UGGAAUGUAAAGAAGUAUGU--------
----- 29 0,-2
--UGGAAUGUAAAGAAGUAUGUA-------
----- 99 0,-1
--UGGAAUGUAAAGAAGUAUGUAa------
----- 65 0,-1
--UGGAAUGUAAAGAAGUAUGUAaa-----
----- 7 0,-1
--UGGAAUGUAAAGAAGUAUGUAU------
----- 1689 0,0
--UGGAAUGUAAAGAAGUAUGUAUa-----
----- 36 0,0
--UGGAAUGUAAAGAAGUAUGUAUaa----
----- 2 0,0
--UGGAAUGUAAAGAAGUAUGUAUU-----
----- 42 0,1

--UGGAAUGUAAAGAAGUAUGUAUUU----
-----	2	0,2
---GGAAUGUAAAGAAGUAUGUAa------
-----	2	1,-1
---GGAAUGUAAAGAAGUAUGUAU------
-----	20	1,0
--GAAUGUAAAGAAGUAUGUAU------
-----	3	2,0

>hsa-mir-100	MA:13-34;mi:48-69	SRX018959
ccuguugccacaAACCCGUAGAUCCGAACUUGUGguauuaguccgcaCAAGCUUGUAUCUAUAGGUAUGugucuguuagg
((((..(.((((.(((..(((((.(((.(((((((........).)))))).))).)))))..))).)))).)...))))
------------AACCCGUAGAUCCGAACU--
	610	0,-4
------------AACCCGUAGAUCCGAACUa---
	3	0,-4
------------AACCCGUAGAUCCGAACUU---
	284	0,-3
------------AACCCGUAGAUCCGAACUUa--
	2	0,-3
------------AACCCGUAGAUCCGAACUUau---
	2	0,-3
------------AACCCGUAGAUCCGAACUUu--
	3	0,-3
------------AACCCGUAGAUCCGAACUUuu---
	10	0,-3
------------AACCCGUAGAUCCGAACUUG--
	1222	0,-2
------------AACCCGUAGAUCCGAACUUGa---
	28	0,-2
------------AACCCGUAGAUCCGAACUUGaa--
	3	0,-2
------------AACCCGUAGAUCCGAACUUGau--
	2	0,-2
------------AACCCGUAGAUCCGAACUUGU---
	6694	0,-1
------------AACCCGUAGAUCCGAACUUGUa--
	55	0,-1

------------AACCCGUAGAUCCGAACUUGUaa--
49 0,-1
------------AACCCGUAGAUCCGAACUUGUau--
18 0,-1
------------AACCCGUAGAUCCGAACUUGUu---
44 0,-1
------------AACCCGUAGAUCCGAACUUGUuu--
16 0,-1
------------AACCCGUAGAUCCGAACUUGUG---
8799 0,0
------------AACCCGUAGAUCCGAACUUGUGa--
7640 0,0
------------AACCCGUAGAUCCGAACUUGUGaa---
472 0,0
------------AACCCGUAGAUCCGAACUUGUGau---
26 0,0
------------AACCCGUAGAUCCGAACUUGUGu--
5573 0,0
------------AACCCGUAGAUCCGAACUUGUGuu---
27 0,0
------------AACCCGUAGAUCCGAACUUGUGG--
33 0,1
------------AACCCGUAGAUCCGAACUUGUGGa---
2 0,1
-------------ACCCGUAGAUCCGAACUUGU--
4 1,-1
-------------ACCCGUAGAUCCGAACUUGUG---
4 1,0
-------------ACCCGUAGAUCCGAACUUGUGa--
16 1,0
-------------ACCCGUAGAUCCGAACUUGUGu--
5 1,0

>hsa-mir-101-1 mi:11-32;MA:47-67 SRX018959
ugcccuggcuCAGUUAUCACAGUGCUGAUGCUgucuauucuaaaggUACAGUACUGUGAUAACUGAAggaugca
.(((.....(((((((((((((((((.((((.............))))))))))))))))))))).....))).
--GGUACAGUACUGUGAUAACUG--------- 2
-2,-2

--GGUACAGUACUGUGAUAACUGAA-------- 3
-2,0
---GUACAGUACUGUGAUAAC------------ 1019
-1,-4
---GUACAGUACUGUGAUAACa----------- 8
-1,-4
---GUACAGUACUGUGAUAACU-----------
37264 -1,-3
---GUACAGUACUGUGAUAACUa---------- 19
-1,-3
---GUACAGUACUGUGAUAACUaa--------- 19
-1,-3
---GUACAGUACUGUGAUAACUau--------- 2
-1,-3
---GUACAGUACUGUGAUAACUu---------- 16
-1,-3
---GUACAGUACUGUGAUAACUuu--------- 2
-1,-3
---GUACAGUACUGUGAUAACUG---------- 2760
-1,-2
---GUACAGUACUGUGAUAACUGu--------- 76
-1,-2
---GUACAGUACUGUGAUAACUGuu-------- 2
-1,-2
---GUACAGUACUGUGAUAACUGA---------
31898 -1,-1
---GUACAGUACUGUGAUAACUGAu-------- 296
-1,-1
---GUACAGUACUGUGAUAACUGAuu------- 9
-1,-1
---GUACAGUACUGUGAUAACUGAA--------
43674 -1,0
---GUACAGUACUGUGAUAACUGAAa-------
10276 -1,0
---GUACAGUACUGUGAUAACUGAAaa------ 354
-1,0
---GUACAGUACUGUGAUAACUGAAau------ 22
-1,0

--GUACAGUACUGUGAUAACUGAAu------- 105

-1,0

--GUACAGUACUGUGAUAACUGAAG------- 56

-1,1

--GUACAGUACUGUGAUAACUGAAGu------ 3

-1,1

--GUACAGUACUGUGAUAACUGAAGG------ 2

-1,2

---UACAGUACUGUGAUAACU-----------

39178 0,-3

---UACAGUACUGUGAUAACUa---------- 10

0,-3

---UACAGUACUGUGAUAACUaa--------- 31

0,-3

---UACAGUACUGUGAUAACUu---------- 16

0,-3

---UACAGUACUGUGAUAACUG---------- 4005

0,-2

---UACAGUACUGUGAUAACUGu--------- 50

0,-2

---UACAGUACUGUGAUAACUGA---------

53281 0,-1

---UACAGUACUGUGAUAACUGAu-------- 235

0,-1

---UACAGUACUGUGAUAACUGAuu------- 219

0,-1

---UACAGUACUGUGAUAACUGAA--------

115605 0,0

---UACAGUACUGUGAUAACUGAAa-------

31911 0,0

---UACAGUACUGUGAUAACUGAAaa------ 1030

0,0

---UACAGUACUGUGAUAACUGAAau------ 119

0,0

---UACAGUACUGUGAUAACUGAAu------- 3595

0,0

---UACAGUACUGUGAUAACUGAAuu------ 128

0,0

--UACAGUACUGUGAUAACUGAAG------- 8492
0,1
--UACAGUACUGUGAUAACUGAAGu------ 1004
0,1
--UACAGUACUGUGAUAACUGAAGuu----- 24
0,1
--UACAGUACUGUGAUAACUGAAGG------ 9
0,2
--UACAGUACUGUGAUAACUGAAGGA----- 9
0,3
---ACAGUACUGUGAUAACUG---------- 27
1,-2
---ACAGUACUGUGAUAACUGA--------- 299
1,-1
---ACAGUACUGUGAUAACUGAA-------- 779
1,0
---ACAGUACUGUGAUAACUGAAa------- 298
1,0
---ACAGUACUGUGAUAACUGAAaa------ 18
1,0
---ACAGUACUGUGAUAACUGAAau------ 22
1,0
---ACAGUACUGUGAUAACUGAAu------- 35
1,0
---ACAGUACUGUGAUAACUGAAuu------ 11
1,0
---ACAGUACUGUGAUAACUGAAG------- 102
1,1
---ACAGUACUGUGAUAACUGAAGu------ 44
1,1
--CAGUACUGUGAUAACUGA--------- 29
2,-1
--CAGUACUGUGAUAACUGAA-------- 119
2,0
--CAGUACUGUGAUAACUGAAa------- 29
2,0
--CAGUACUGUGAUAACUGAAaa------ 9
2,0

--CAGUACUGUGAUAACUGAAu------- 4
2,0
--CAGUACUGUGAUAACUGAAuu------ 2
2,0
--CAGUACUGUGAUAACUGAAG------- 15
2,1
--CAGUACUGUGAUAACUGAAGu------ 5
2,1

>hsa-mir-101-2 3p:49-69 SRX018959
acuguccuuuuucgguuaucaugguaccgaugcuguauaucugaaaggUACAGUACUGUGAUAACUGAAgaaugguggu
((..((.((((((((((((((((((((.(.((((...........))))).))))))))))))))))))))).))..))
--GGUACAGUACUGUGAUAACUG------------
2 -2,-2
--GGUACAGUACUGUGAUAACUGAA----------
3 -2,0
---GUACAGUACUGUGAUAAC--------------
1019 -1,-4
---GUACAGUACUGUGAUAACa-------------
8 -1,-4
---GUACAGUACUGUGAUAACU-------------
37264 -1,-3
---GUACAGUACUGUGAUAACUa------------
19 -1,-3
---GUACAGUACUGUGAUAACUaa-----------
19 -1,-3
---GUACAGUACUGUGAUAACUau-----------
2 -1,-3
---GUACAGUACUGUGAUAACUu------------
16 -1,-3
---GUACAGUACUGUGAUAACUuu-----------
2 -1,-3
---GUACAGUACUGUGAUAACUG------------
2760 -1,-2
---GUACAGUACUGUGAUAACUGu-----------
76 -1,-2
---GUACAGUACUGUGAUAACUGuu----------
2 -1,-2

---GUACAGUACUGUGAUAACUGA-----------

31898 -1,-1

---GUACAGUACUGUGAUAACUGAu----------

296 -1,-1

---GUACAGUACUGUGAUAACUGAuu---------

9 -1,-1

---GUACAGUACUGUGAUAACUGAA----------

43674 -1,0

---GUACAGUACUGUGAUAACUGAAa---------

10276 -1,0

---GUACAGUACUGUGAUAACUGAAaa--------

354 -1,0

---GUACAGUACUGUGAUAACUGAAau--------

22 -1,0

---GUACAGUACUGUGAUAACUGAAu---------

105 -1,0

---GUACAGUACUGUGAUAACUGAAG---------

56 -1,1

---GUACAGUACUGUGAUAACUGAAGu--------

3 -1,1

---GUACAGUACUGUGAUAACUGAAGA--------

17 -1,2

--UACAGUACUGUGAUAACU-------------

39178 0,-3

--UACAGUACUGUGAUAACUa------------

10 0,-3

--UACAGUACUGUGAUAACUaa-----------

31 0,-3

--UACAGUACUGUGAUAACUu------------

16 0,-3

--UACAGUACUGUGAUAACUG------------

4005 0,-2

--UACAGUACUGUGAUAACUGu-----------

50 0,-2

--UACAGUACUGUGAUAACUGA-----------

53281 0,-1

--UACAGUACUGUGAUAACUGAu----------

235 0,-1

--UACAGUACUGUGAUAACUGAuu---------

219 0,-1

--UACAGUACUGUGAUAACUGAA----------

115605 0,0

--UACAGUACUGUGAUAACUGAAa---------

31911 0,0

--UACAGUACUGUGAUAACUGAAaa--------

1030 0,0

--UACAGUACUGUGAUAACUGAAau--------

119 0,0

--UACAGUACUGUGAUAACUGAAu---------

3595 0,0

--UACAGUACUGUGAUAACUGAAuu--------

128 0,0

--UACAGUACUGUGAUAACUGAAG---------

8492 0,1

--UACAGUACUGUGAUAACUGAAGu--------

1004 0,1

--UACAGUACUGUGAUAACUGAAGuu-------

24 0,1

--UACAGUACUGUGAUAACUGAAGA--------

2077 0,2

--UACAGUACUGUGAUAACUGAAGAu-------

6 0,2

--UACAGUACUGUGAUAACUGAAGAA-------

89 0,3

--UACAGUACUGUGAUAACUGAAGAAa------

20 0,3

---ACAGUACUGUGAUAACUG------------

27 1,-2

---ACAGUACUGUGAUAACUGA-----------

299 1,-1

---ACAGUACUGUGAUAACUGAA----------

779 1,0

---ACAGUACUGUGAUAACUGAAa---------

298 1,0

---ACAGUACUGUGAUAACUGAAaa--------

18 1,0

---ACAGUACUGUGAUAACUGAAau--------

22 1,0

---ACAGUACUGUGAUAACUGAAu---------

35 1,0

---ACAGUACUGUGAUAACUGAAuu--------

11 1,0

---ACAGUACUGUGAUAACUGAAG---------

102 1,1

---ACAGUACUGUGAUAACUGAAGu--------

44 1,1

---ACAGUACUGUGAUAACUGAAGA--------

36 1,2

---ACAGUACUGUGAUAACUGAAGAA-------

4 1,3

---ACAGUACUGUGAUAACUGAAGAAa------

2 1,3

--CAGUACUGUGAUAACUGA-----------

29 2,-1

--CAGUACUGUGAUAACUGAA----------

119 2,0

--CAGUACUGUGAUAACUGAAa---------

29 2,0

--CAGUACUGUGAUAACUGAAaa--------

9 2,0

--CAGUACUGUGAUAACUGAAu---------

4 2,0

--CAGUACUGUGAUAACUGAAuu--------

2 2,0

--CAGUACUGUGAUAACUGAAG---------

15 2,1

--CAGUACUGUGAUAACUGAAGu--------

5 2,1

--CAGUACUGUGAUAACUGAAGA--------

35 2,2

--CAGUACUGUGAUAACUGAAGAu-------

3 2,2

--CAGUACUGUGAUAACUGAAGAA-------

21 2,3

--CAGUACUGUGAUAACUGAAGAAa------
28	2,3
--CAGUACUGUGAUAACUGAAGAAU------
2	2,4

>hsa-mir-103-1	3p:48-70	SRX018959
uacugcccucggcuucuuuacagugcugccuuguugcauauggaucaAGCAGCAUUGUACAGGGCUAUGAaggcauug
...((((.((((((.((.(((((((((((.(((.(.(....).).)))))))))))))).))))))..)).))))...
----------GGCUUCUUUACAGUGCUGCCUU--
2
---AGCAGCAUUGUACAGGGC-------------
4102	0,-5
---AGCAGCAUUGUACAGGGCa------------
4	0,-5
---AGCAGCAUUGUACAGGGCaa-----------
3	0,-5
---AGCAGCAUUGUACAGGGCau-----------
4	0,-5
---AGCAGCAUUGUACAGGGCU------------
1983	0,-4
---AGCAGCAUUGUACAGGGCUu-----------
26	0,-4
---AGCAGCAUUGUACAGGGCUuu----------
26	0,-4
---AGCAGCAUUGUACAGGGCUA-----------
2933	0,-3
---AGCAGCAUUGUACAGGGCUAa----------
148	0,-3
---AGCAGCAUUGUACAGGGCUAaa---------
34	0,-3
---AGCAGCAUUGUACAGGGCUAau---------
28	0,-3
---AGCAGCAUUGUACAGGGCUAU----------
14923	0,-2
---AGCAGCAUUGUACAGGGCUAUa---------
4958	0,-2
---AGCAGCAUUGUACAGGGCUAUaa--------
306	0,-2

--AGCAGCAUUGUACAGGGCUAUau--------

304 0,-2

--AGCAGCAUUGUACAGGGCUAUu---------

1299 0,-2

--AGCAGCAUUGUACAGGGCUAUuu--------

403 0,-2

--AGCAGCAUUGUACAGGGCUAUG---------

9556 0,-1

--AGCAGCAUUGUACAGGGCUAUGu--------

2270 0,-1

--AGCAGCAUUGUACAGGGCUAUGuu-------

231 0,-1

--AGCAGCAUUGUACAGGGCUAUGA--------

12145 0,0

--AGCAGCAUUGUACAGGGCUAUGAu-------

804 0,0

--AGCAGCAUUGUACAGGGCUAUGAuu------

26 0,0

--AGCAGCAUUGUACAGGGCUAUGAA-------

225 0,1

--AGCAGCAUUGUACAGGGCUAUGAAu------

4 0,1

---GCAGCAUUGUACAGGGCU------------

4 1,-4

---GCAGCAUUGUACAGGGCUA-----------

8 1,-3

---GCAGCAUUGUACAGGGCUAU----------

40 1,-2

---GCAGCAUUGUACAGGGCUAUa---------

8 1,-2

---GCAGCAUUGUACAGGGCUAUG---------

27 1,-1

---GCAGCAUUGUACAGGGCUAUGu--------

10 1,-1

---GCAGCAUUGUACAGGGCUAUGA--------

82 1,0

---GCAGCAUUGUACAGGGCUAUGAu-------

6 1,0

---CAGCAUUGUACAGGGCUAU----------
11 2,-2
---CAGCAUUGUACAGGGCUAUa---------
5 2,-2
---CAGCAUUGUACAGGGCUAUG---------
16 2,-1
---CAGCAUUGUACAGGGCUAUGu--------
6 2,-1
---CAGCAUUGUACAGGGCUAUGA--------
49 2,0

>hsa-mir-103-2 mi:11-33;MA:48-70 SRX018959
uugugcuuucAGCUUCUUUACAGUGCUGCCUUGuagcauucaggucaAGCAGCAUUGUACAGGGCUAUGAaagaacca
..((.(((((((((.((.((((((((((((((((.......))))....))))))))))))).))))).))))))).))..
----------AGCUUCUUUACAGUGCUGCCUUG---
2 0,0
---AGCAGCAUUGUACAGGGC-------------
4102 0,-5
---AGCAGCAUUGUACAGGGCa------------
4 0,-5
---AGCAGCAUUGUACAGGGCaa-----------
3 0,-5
---AGCAGCAUUGUACAGGGCau-----------
4 0,-5
---AGCAGCAUUGUACAGGGCU------------
1983 0,-4
---AGCAGCAUUGUACAGGGCUu-----------
26 0,-4
---AGCAGCAUUGUACAGGGCUuu----------
26 0,-4
---AGCAGCAUUGUACAGGGCUA-----------
2933 0,-3
---AGCAGCAUUGUACAGGGCUAa----------
148 0,-3
---AGCAGCAUUGUACAGGGCUAaa---------
34 0,-3
---AGCAGCAUUGUACAGGGCUAau---------
28 0,-3

---AGCAGCAUUGUACAGGGCUAU----------

14923 0,-2

---AGCAGCAUUGUACAGGGCUAUa---------

4958 0,-2

---AGCAGCAUUGUACAGGGCUAUaa--------

306 0,-2

---AGCAGCAUUGUACAGGGCUAUau--------

304 0,-2

---AGCAGCAUUGUACAGGGCUAUu---------

1299 0,-2

---AGCAGCAUUGUACAGGGCUAUuu--------

403 0,-2

---AGCAGCAUUGUACAGGGCUAUG---------

9556 0,-1

---AGCAGCAUUGUACAGGGCUAUGu--------

2270 0,-1

---AGCAGCAUUGUACAGGGCUAUGuu-------

231 0,-1

---AGCAGCAUUGUACAGGGCUAUGA--------

12145 0,0

---AGCAGCAUUGUACAGGGCUAUGAu-------

804 0,0

---AGCAGCAUUGUACAGGGCUAUGAuu------

26 0,0

---AGCAGCAUUGUACAGGGCUAUGAA-------

225 0,1

---AGCAGCAUUGUACAGGGCUAUGAAu------

4 0,1

---AGCAGCAUUGUACAGGGCUAUGAAA------

46 0,2

--GCAGCAUUGUACAGGGCU------------

4 1,-4

--GCAGCAUUGUACAGGGCUA-----------

8 1,-3

--GCAGCAUUGUACAGGGCUAU----------

40 1,-2

--GCAGCAUUGUACAGGGCUAUa---------

8 1,-2

--GCAGCAUUGUACAGGGCUAUG---------
    27 1,-1
--GCAGCAUUGUACAGGGCUAUGu--------
    10 1,-1
--GCAGCAUUGUACAGGGCUAUGA--------
    82 1,0
--GCAGCAUUGUACAGGGCUAUGAu-------
    6   1,0
---CAGCAUUGUACAGGGCUAU----------
    11 2,-2
---CAGCAUUGUACAGGGCUAUa---------
    5   2,-2
---CAGCAUUGUACAGGGCUAUG---------
    16 2,-1
---CAGCAUUGUACAGGGCUAUGu--------
    6   2,-1
---CAGCAUUGUACAGGGCUAUGA--------
    49 2,0

>hsa-mir-106a   MA:13-35;mi:50-71   SRX018959
ccuuggccauguAAAAGUGCUUACAGUGCAGGUAGcuuuugagaucuaCUGCAAUGUAAGCACUUCUUACauuaccaug
g
((.(((..((((((((((((((((.(((((.(((.((.....)).)))))))).)))))))))).))))))..))).)
)
------------AAAAGUGCUUACAGUGCAG--
-   2   0,-4
------------AAAAGUGCUUACAGUGCAGGU--
-   2   0,-2
------------AAAAGUGCUUACAGUGCAGGUA---
-   2   0,-1
-------------AAAGUGCUUACAGUGCAG--
-   4   1,-4
-------------AAAGUGCUUACAGUGCAGG---
-   4   1,-3
-------------AAAGUGCUUACAGUGCAGGU--
-   3   1,-2
-------------AAAGUGCUUACAGUGCAGGUA---
-   2   1,-1

-------------AAAGUGCUUACAGUGCAGGUAu---
-   3   1,-1
-------------AAAGUGCUUACAGUGCAGGUAG---
-   8   1,0

>hsa-mir-106b   MA:12-32;mi:52-73   SRX018959
ccugccggggcUAAAGUGCUGACAGUGCAGAUagugguccucuccgugcuaCCGCACUGUGGGUACUUGCUGCuccagca
gg
(((((.(((((..(((((((.(((((((.(.((((((......))..))))).))))))).)))))))...))))).)))
))
-----------UAAAGUGCUGACAGUGCA---
-- 10 0,-3
-----------UAAAGUGCUGACAGUGCAG--
-- 35 0,-2
-----------UAAAGUGCUGACAGUGCAGA---
-- 10 0,-1
-----------UAAAGUGCUGACAGUGCAGAU--
-- 37 0,0
-----------UAAAGUGCUGACAGUGCAGAUu---
-- 6   0,0
-----------UAAAGUGCUGACAGUGCAGAUA---
-- 73 0,1
-----------UAAAGUGCUGACAGUGCAGAUAa--
-- 31 0,1
-----------UAAAGUGCUGACAGUGCAGAUAu--
-- 25 0,1
-----------UAAAGUGCUGACAGUGCAGAUAG--
-- 3   0,2
---UACCGCACUGUGGGUACUUGC----------
-- 3   -2,-3
---UACCGCACUGUGGGUACUUGCU---------
-- 17 -2,-2
---CCGCACUGUGGGUACUUGCU---------
-- 20 0,-2
---CCGCACUGUGGGUACUUGCUa--------
-- 4   0,-2
---CCGCACUGUGGGUACUUGCUG--------
-- 44 0,-1

---CCGCACUGUGGGUACUUGCUGa-------
--	44	0,-1
---CCGCACUGUGGGUACUUGCUGaa------
--	11	0,-1
---CCGCACUGUGGGUACUUGCUGu-------
--	11	0,-1
---CCGCACUGUGGGUACUUGCUGC-------
--	78	0,0
---CCGCACUGUGGGUACUUGCUGCa------
--	2	0,0
---CCGCACUGUGGGUACUUGCUGCU------
--	3	0,1
--CGCACUGUGGGUACUUGCUGC-------
--	3	1,0

>hsa-mir-107	3p:50-72	SRX018959
cucucugcuuucagcuucuuuacaguguugccuuguggcauggaguucaAGCAGCAUUGUACAGGGCUAUCAaagcacag
a
.(((.((((((.((((.((.(((((((((((.(((.(((.....))))))))))))))))).))))))...)))))).))
)
------------AGCUUCUUUACAGUGUUGCCUUG---
-	2
---AGCAGCAUUGUACAGGGC-------------
-	4102	0,-5
---AGCAGCAUUGUACAGGGCa------------
-	4	0,-5
---AGCAGCAUUGUACAGGGCaa-----------
-	3	0,-5
---AGCAGCAUUGUACAGGGCau-----------
-	4	0,-5
---AGCAGCAUUGUACAGGGCU------------
-	1983	0,-4
---AGCAGCAUUGUACAGGGCUu-----------
-	26	0,-4
---AGCAGCAUUGUACAGGGCUuu----------
-	26	0,-4
---AGCAGCAUUGUACAGGGCUA-----------
-	2933	0,-3

---AGCAGCAUUGUACAGGGCUAa----------
-    148 0,-3
---AGCAGCAUUGUACAGGGCUAaa---------
-    34 0,-3
---AGCAGCAUUGUACAGGGCUAau---------
-    28 0,-3
---AGCAGCAUUGUACAGGGCUAU----------
-    14923   0,-2
---AGCAGCAUUGUACAGGGCUAUa---------
-    4958   0,-2
---AGCAGCAUUGUACAGGGCUAUaa--------
-    306 0,-2
---AGCAGCAUUGUACAGGGCUAUau--------
-    304 0,-2
---AGCAGCAUUGUACAGGGCUAUu---------
-    1299   0,-2
---AGCAGCAUUGUACAGGGCUAUuu--------
-    403 0,-2
---AGCAGCAUUGUACAGGGCUAUC---------
-    1474   0,-1
---AGCAGCAUUGUACAGGGCUAUCu--------
-    871 0,-1
---AGCAGCAUUGUACAGGGCUAUCuu-------
-    24 0,-1
---AGCAGCAUUGUACAGGGCUAUCA--------
-    3636   0,0
---AGCAGCAUUGUACAGGGCUAUCAu-------
-    94 0,0
---AGCAGCAUUGUACAGGGCUAUCAuu------
-    4   0,0
---AGCAGCAUUGUACAGGGCUAUCAA-------
-    24 0,1
--GCAGCAUUGUACAGGGCU------------
-    4   1,-4
--GCAGCAUUGUACAGGGCUA-----------
-    8   1,-3
--GCAGCAUUGUACAGGGCUAU----------
-    40 1,-2

--GCAGCAUUGUACAGGGCUAUa---------
-    8   1,-2
---CAGCAUUGUACAGGGCUAU----------
-    11   2,-2
---CAGCAUUGUACAGGGCUAUa---------
-    5   2,-2
---CAGCAUUGUACAGGGCUAUC---------
-    2   2,-1
---CAGCAUUGUACAGGGCUAUCA--------
-    3   2,0

>hsa-mir-10a   MA:22-44;mi:63-84   SRX018959
gaucugucugucuucuguauaUACCCUGUAGAUCCGAAUUUGUGuaaggaauuuuguggucaCAAAUUCGUAUCUAGGGG
AAUAuguaguugacauaaacacuccgcucu
....(((.((((..((((((((.((((.(((((.((((((((((................)))))))))).)))))))))
.))))))))..))))...))).........
--------------------AUACCCUGUAGAUCCGAAUUUG--------------------------------------
------------------------------   5   -1,-2
--------------------AUACCCUGUAGAUCCGAAUUUGa-------------------------------------
------------------------------   2   -1,-2
--------------------AUACCCUGUAGAUCCGAAUUUGU-------------------------------------
------------------------------   6   -1,-1
---------------------UACCCUGUAGAUCCGAAU---
------------------------------   41   0,-5
---------------------UACCCUGUAGAUCCGAAUa--
------------------------------   38   0,-5
---------------------UACCCUGUAGAUCCGAAUaa---------------------------------------
------------------------------   6   0,-5
---------------------UACCCUGUAGAUCCGAAUU--
------------------------------   271 0,-4
---------------------UACCCUGUAGAUCCGAAUUa---------------------------------------
------------------------------   4   0,-4
---------------------UACCCUGUAGAUCCGAAUUU---------------------------------------
------------------------------   110 0,-3
---------------------UACCCUGUAGAUCCGAAUUUa--------------------------------------
------------------------------   4   0,-3
---------------------UACCCUGUAGAUCCGAAUUUuu-------------------------------------
------------------------------   2   0,-3

---------------------UACCCUGUAGAUCCGAAUUUG--------------------------------------
------------------------------ 763 0,-2
---------------------UACCCUGUAGAUCCGAAUUUGa-------------------------------------
------------------------------ 9   0,-2
---------------------UACCCUGUAGAUCCGAAUUUGau------------------------------------
------------------------------ 3   0,-2
---------------------UACCCUGUAGAUCCGAAUUUGU-------------------------------------
------------------------------ 2053   0,-1
---------------------UACCCUGUAGAUCCGAAUUUGUa------------------------------------
------------------------------ 7   0,-1
---------------------UACCCUGUAGAUCCGAAUUUGUaa-----------------------------------
------------------------------ 2   0,-1
---------------------UACCCUGUAGAUCCGAAUUUGUu------------------------------------
------------------------------ 5   0,-1
---------------------UACCCUGUAGAUCCGAAUUUGUG------------------------------------
------------------------------ 164 0,0
---------------------UACCCUGUAGAUCCGAAUUUGUGa-----------------------------------
------------------------------ 45 0,0
---------------------UACCCUGUAGAUCCGAAUUUGUGU-----------------------------------
------------------------------ 23 0,1
----------------------ACCCUGUAGAUCCGAAUUU---------------------------------------
------------------------------ 4   1,-3
----------------------ACCCUGUAGAUCCGAAUUUG--------------------------------------
------------------------------ 8   1,-2
----------------------ACCCUGUAGAUCCGAAUUUGa-------------------------------------
------------------------------ 3   1,-2
----------------------ACCCUGUAGAUCCGAAUUUGU-------------------------------------
------------------------------ 230 1,-1
----------------------ACCCUGUAGAUCCGAAUUUGUG------------------------------------
------------------------------ 150 1,0
----------------------ACCCUGUAGAUCCGAAUUUGUGa-----------------------------------
------------------------------ 205 1,0
----------------------ACCCUGUAGAUCCGAAUUUGUGaa----------------------------------
------------------------------ 26 1,0
----------------------ACCCUGUAGAUCCGAAUUUGUGU-----------------------------------
------------------------------ 75 1,1
-----------------------CCCUGUAGAUCCGAAUUUGU-------------------------------------
------------------------------ 2   2,-1

-----------------------CCCUGUAGAUCCGAAUUUGUGa-----------------------------------
------------------------------ 4   2,0
-----------------------CCCUGUAGAUCCGAAUUUGUGU-----------------------------------
------------------------------ 2   2,1
---AAAUUCGUAUCUAGGGG
A----------------------------- 2   1,-3
---AAAUUCGUAUCUAGGGG
AAUA-------------------------- 2   1,0

>hsa-mir-10b   MA:27-49;mi:66-87   SRX018959
ccagagguuguaacguugucuauauaUACCCUGUAGAACCGAAUUUGUGugguauccguauagucACAGAUUCGAUUCUA
GGGGAAUauauggucgaugcaaaaacuuca
...((((((....(((((.((((((((.((((.(((((.((((((((((..(((....)))...)))))))))).)))))
)))).)))))))).)))))....)))))).
--------------------------UACCCUGUAGAACCGAAUUU----------------------------------
------------------------------ 2   0,-3
--------------------------UACCCUGUAGAACCGAAUUUG---------------------------------
------------------------------ 3   0,-2
--------------------------UACCCUGUAGAACCGAAUUUGa--------------------------------
------------------------------ 2   0,-2
--------------------------UACCCUGUAGAACCGAAUUUGU--------------------------------
------------------------------ 81 0,-1
--------------------------UACCCUGUAGAACCGAAUUUGUG-------------------------------
------------------------------ 8   0,0
--------------------------UACCCUGUAGAACCGAAUUUGUGa------------------------------
------------------------------ 5   0,0
--------------------------UACCCUGUAGAACCGAAUUUGUGU------------------------------
------------------------------ 2   0,1
---------------------------ACCCUGUAGAACCGAAUUUGU--------------------------------
------------------------------ 5   1,-1
---------------------------ACCCUGUAGAACCGAAUUUGUG-------------------------------
------------------------------ 4   1,0
---------------------------ACCCUGUAGAACCGAAUUUGUGa------------------------------
------------------------------ 3   1,0
---------------------------ACCCUGUAGAACCGAAUUUGUGU------------------------------
------------------------------ 5   1,1

>hsa-mir-1181   5p:14-34   SRX018959

uccacugcugccgCCGUCGCCGCCACCCGAGCCGgagcgggcugggccgccaaggcaagaugguggacuacagcgugugg
g
.((((((((((((((((((((((..(((.((((......)))))))..))...)))..))))))))....))))).))))
.
--AGGCAAGAUGGUGGACUA----------
-    4

>hsa-mir-1185-1 5p:15-35   SRX018959
uuugguacuugaagAGAGGAUACCCUUUGUAUGUUcacuugauuaauggcgaauauacagggggagacucuuauuugcgu
aucaaa
.(((((((.(((((((((..(.((((((((((((((.((........)).)))))))))))))))..))))).)))).))
))))).
--------------AGAGGAUACCCUUUGUAU--
------ 2   0,-3
--------------AGAGGAUACCCUUUGUAUGUU---
------ 18   0,0
--------------AGAGGAUACCCUUUGUAUGUUa--
------ 2   0,0
--------------AGAGGAUACCCUUUGUAUGUUC--
------ 9   0,1
--------------AGAGGAUACCCUUUGUAUGUUCA---
------ 5   0,2
--AUAUACAGGGGGAGACUCUUAU------
------ 4

>hsa-mir-1185-2 5p:15-35   SRX018959
uuugguacuuaaagAGAGGAUACCCUUUGUAUGUUcacuugauuaauggcgaauauacagggggagacucucauuugcgu
aucaaa
.(((((((.(((((((((..(.((((((((((((((.((........)).)))))))))))))))..))))).)))).))
))))).
--------------AGAGGAUACCCUUUGUAU--
------ 2   0,-3
--------------AGAGGAUACCCUUUGUAUGUU---
------ 18   0,0
--------------AGAGGAUACCCUUUGUAUGUUa--
------ 2   0,0
--------------AGAGGAUACCCUUUGUAUGUUC--
------ 9   0,1

--------------AGAGGAUACCCUUUGUAUGUUCA---
------ 5 0,2
--AUAUACAGGGGGAGACUCUCA-------
------ 2
--AUAUACAGGGGGAGACUCUCAU------
------ 3

>hsa-mir-122 MA:15-36;mi:51-72 SRX018959
ccuuagcagagcugUGGAGUGUGACAAUGGUGUUUGugucuaaacuaucaAACGCCAUUAUCACACUAAAUAgcuacugc
uaggc
.((((((((((((((..(((((((.(((((((((((.((....))...))))))))))).))))))).)))))).))))
)))).
------------UGUGGAGUGUGACAAUGGUGU---
----- 14 -2,-3
------------UGUGGAGUGUGACAAUGGUGUU--
----- 109 -2,-2
------------UGUGGAGUGUGACAAUGGUGUUa---
----- 12 -2,-2
------------UGUGGAGUGUGACAAUGGUGUUU---
----- 15 -2,-1
-------------GUGGAGUGUGACAAUGGUG--
----- 4 -1,-4
-------------GUGGAGUGUGACAAUGGUGU---
----- 7 -1,-3
-------------GUGGAGUGUGACAAUGGUGUU--
----- 48 -1,-2
-------------GUGGAGUGUGACAAUGGUGUUU---
----- 169 -1,-1
-------------GUGGAGUGUGACAAUGGUGUUUa--
----- 2 -1,-1
-------------GUGGAGUGUGACAAUGGUGUUUG--
----- 9 -1,0
-------------GUGGAGUGUGACAAUGGUGUUUGa---
----- 5 -1,0
-------------GUGGAGUGUGACAAUGGUGUUUGU---
----- 6 -1,1
--------------UGGAGUGUGACAAUGGUG--
----- 15922 0,-4

--------------UGGAGUGUGACAAUGGUGa---
-----	416	0,-4
--------------UGGAGUGUGACAAUGGUGaa--
-----	240	0,-4
--------------UGGAGUGUGACAAUGGUGau--
-----	14	0,-4
--------------UGGAGUGUGACAAUGGUGU---
-----	57427	0,-3
--------------UGGAGUGUGACAAUGGUGUa--
-----	1356	0,-3
--------------UGGAGUGUGACAAUGGUGUaa---
-----	3396	0,-3
--------------UGGAGUGUGACAAUGGUGUau---
-----	433	0,-3
--------------UGGAGUGUGACAAUGGUGUU--
-----	119783	0,-2
--------------UGGAGUGUGACAAUGGUGUUa---
-----	11885	0,-2
--------------UGGAGUGUGACAAUGGUGUUaa--
-----	4739	0,-2
--------------UGGAGUGUGACAAUGGUGUUau--
-----	805	0,-2
--------------UGGAGUGUGACAAUGGUGUUU---
-----	766530	0,-1
--------------UGGAGUGUGACAAUGGUGUUUa--
-----	54095	0,-1
--------------UGGAGUGUGACAAUGGUGUUUaa---
-----	36921	0,-1
--------------UGGAGUGUGACAAUGGUGUUUau---
-----	18234	0,-1
--------------UGGAGUGUGACAAUGGUGUUUu--
-----	22122	0,-1
--------------UGGAGUGUGACAAUGGUGUUUuu---
-----	2779	0,-1
--------------UGGAGUGUGACAAUGGUGUUUG--
-----	1676822	0,0
--------------UGGAGUGUGACAAUGGUGUUUGa---
-----	1542903	0,0

--------------UGGAGUGUGACAAUGGUGUUUGaa---
-----	31836	0,0
--------------UGGAGUGUGACAAUGGUGUUUGau---
-----	14742	0,0
--------------UGGAGUGUGACAAUGGUGUUUGU--
-----	932474	0,1
--------------UGGAGUGUGACAAUGGUGUUUGUa---
-----	2580	0,1
--------------UGGAGUGUGACAAUGGUGUUUGUaa--
-----	2456	0,1
--------------UGGAGUGUGACAAUGGUGUUUGUau--
-----	1410	0,1
--------------UGGAGUGUGACAAUGGUGUUUGUu---
-----	2617	0,1
--------------UGGAGUGUGACAAUGGUGUUUGUuu--
-----	781	0,1
--------------UGGAGUGUGACAAUGGUGUUUGUG---
-----	142	0,2
--------------UGGAGUGUGACAAUGGUGUUUGUGa--
-----	32	0,2
--------------UGGAGUGUGACAAUGGUGUUUGUGU--
-----	114	0,3
---------------GGAGUGUGACAAUGGUGU--
-----	718	1,-3
---------------GGAGUGUGACAAUGGUGUa---
-----	20	1,-3
---------------GGAGUGUGACAAUGGUGUaa--
-----	37	1,-3
---------------GGAGUGUGACAAUGGUGUau--
-----	7	1,-3
---------------GGAGUGUGACAAUGGUGUU---
-----	1493	1,-2
---------------GGAGUGUGACAAUGGUGUUa--
-----	103	1,-2
---------------GGAGUGUGACAAUGGUGUUaa---
-----	79	1,-2
---------------GGAGUGUGACAAUGGUGUUau---
-----	14	1,-2

---------------GGAGUGUGACAAUGGUGUUU---
-----   7321   1,-1
---------------GGAGUGUGACAAUGGUGUUUa--
-----   653 1,-1
---------------GGAGUGUGACAAUGGUGUUUaa--
-----   516 1,-1
---------------GGAGUGUGACAAUGGUGUUUau--
-----   208 1,-1
---------------GGAGUGUGACAAUGGUGUUUu--
-----   175 1,-1
---------------GGAGUGUGACAAUGGUGUUUuu--
-----   18 1,-1
---------------GGAGUGUGACAAUGGUGUUUG--
-----   23633   1,0
---------------GGAGUGUGACAAUGGUGUUUGa--
-----   20580   1,0
---------------GGAGUGUGACAAUGGUGUUUGaa---
-----   522 1,0
---------------GGAGUGUGACAAUGGUGUUUGau---
-----   276 1,0
---------------GGAGUGUGACAAUGGUGUUUGU--
-----   10155   1,1
---------------GGAGUGUGACAAUGGUGUUUGUa---
-----   39 1,1
---------------GGAGUGUGACAAUGGUGUUUGUaa--
-----   56 1,1
---------------GGAGUGUGACAAUGGUGUUUGUau--
-----   29 1,1
---------------GGAGUGUGACAAUGGUGUUUGUu---
-----   68 1,1
---------------GGAGUGUGACAAUGGUGUUUGUuu--
-----   20 1,1
---------------GGAGUGUGACAAUGGUGUUUGUG---
-----   5   1,2
---------------GGAGUGUGACAAUGGUGUUUGUGU--
-----   9   1,3
----------------GAGUGUGACAAUGGUGUU---
-----   181 2,-2

----------------GAGUGUGACAAUGGUGUUU--
-----   199 2,-1
----------------GAGUGUGACAAUGGUGUUUa---
-----   10   2,-1
----------------GAGUGUGACAAUGGUGUUUaa--
-----   6   2,-1
----------------GAGUGUGACAAUGGUGUUUau--
-----   2   2,-1
----------------GAGUGUGACAAUGGUGUUUu---
-----   5   2,-1
----------------GAGUGUGACAAUGGUGUUUG---
-----   133 2,0
----------------GAGUGUGACAAUGGUGUUUGa--
-----   189 2,0
----------------GAGUGUGACAAUGGUGUUUGaa---
-----   6   2,0
----------------GAGUGUGACAAUGGUGUUUGau---
-----   2   2,0
----------------GAGUGUGACAAUGGUGUUUGU--
-----   124 2,1
---CAAACGCCAUUAUCACACUAA-----------
-----   5   -2,-3
--AAACGCCAUUAUCACACU-------------
-----   4   -1,-5
--AAACGCCAUUAUCACACUA------------
-----   25   -1,-4
--AAACGCCAUUAUCACACUAu-----------
-----   12   -1,-4
--AAACGCCAUUAUCACACUAuu----------
-----   22   -1,-4
--AAACGCCAUUAUCACACUAA-----------
-----   176 -1,-3
--AAACGCCAUUAUCACACUAAu----------
-----   45   -1,-3
--AAACGCCAUUAUCACACUAAuu---------
-----   27   -1,-3
--AAACGCCAUUAUCACACUAAA----------
-----   88   -1,-2

---AAACGCCAUUAUCACACUAAAa---------
-----   9   -1,-2
---AAACGCCAUUAUCACACUAAAU---------
-----   848 -1,-1
---AAACGCCAUUAUCACACUAAAUu--------
-----   42   -1,-1
---AAACGCCAUUAUCACACUAAAUA--------
-----   38   -1,0
---AAACGCCAUUAUCACACUAAAUAa-------
-----   4   -1,0
--AACGCCAUUAUCACACUA------------
-----   153 0,-4
--AACGCCAUUAUCACACUAu-----------
-----   5   0,-4
--AACGCCAUUAUCACACUAuu----------
-----   29   0,-4
--AACGCCAUUAUCACACUAA-----------
-----   150 0,-3
--AACGCCAUUAUCACACUAAu----------
-----   46   0,-3
--AACGCCAUUAUCACACUAAuu---------
-----   141 0,-3
--AACGCCAUUAUCACACUAAA----------
-----   407 0,-2
--AACGCCAUUAUCACACUAAAa---------
-----   78   0,-2
--AACGCCAUUAUCACACUAAAaa--------
-----   25   0,-2
--AACGCCAUUAUCACACUAAAau--------
-----   2   0,-2
--AACGCCAUUAUCACACUAAAU---------
-----   5861   0,-1
--AACGCCAUUAUCACACUAAAUu--------
-----   540 0,-1
--AACGCCAUUAUCACACUAAAUuu-------
-----   55   0,-1
--AACGCCAUUAUCACACUAAAUA--------
-----   1564   0,0

--AACGCCAUUAUCACACUAAAUAa-------
-----	308	0,0
--AACGCCAUUAUCACACUAAAUAau------
-----	2	0,0
--AACGCCAUUAUCACACUAAAUAu-------
-----	911	0,0
--AACGCCAUUAUCACACUAAAUAuu------
-----	19	0,0
---ACGCCAUUAUCACACUAAA----------
-----	2	1,-2
---ACGCCAUUAUCACACUAAAa---------
-----	5	1,-2
---ACGCCAUUAUCACACUAAAU---------
-----	23	1,-1
---ACGCCAUUAUCACACUAAAUA--------
-----	11	1,0
---ACGCCAUUAUCACACUAAAUAa-------
-----	3	1,0

>hsa-mir-1228	mi:1-21;MA:52-71	SRX018959
GUGGGCGGGGGCAGGUGUGUGgugggugguggccugcggugagcagggcccUCACACCUGCCUCGCCCCCCag
..((((((((.((((((((....((((.....(((((.....))))))))).))))))))))))))))).....
GUGGGCGGGGGCAGGUGUGUG---	9
	0,0

>hsa-mir-124-1	mi:14-35;MA:53-72	SRX018959
aggccucucucucCGUGUUCACAGCGGACCUUGAUuuaaauguccauacaauUAAGGCACGCGGUGAAUGCCaagaaugg
ggcug
.((((((..(((..((((((((.(((..((((((((............))))))))..))).))))))))..)))..))
)))).
---UAAGGCACGCGGUGAAUGCCAu------
-----	2	0,1
---UAAGGCACGCGGUGAAUGCCAA------
-----	3	0,2

>hsa-mir-124-2	mi:25-46;MA:62-81	SRX018959
aucaagauuagaggcucugcucucCGUGUUCACAGCGGACCUUGAUuuaaaugucauacaauUAAGGCACGCGGUGAAUGC
Caagagcggagccuacggcugcacuugaa

.(((((..((((((((((((((..((((((((.(((..((((((((..........))))))))..))).))))))))
..))))))))))))....)))..))))).
---UAAGGCACGCGGUGAAUGC
CAu-------------------------- 2 0,1
---UAAGGCACGCGGUGAAUGC
CAA-------------------------- 3 0,2

>hsa-mir-124-3 mi:15-36;MA:53-72 SRX018959
ugagggccccucugCGUGUUCACAGCGGACCUUGAUuuaaugucuauacaauUAAGGCACGCGGUGAAUGCCaagagagg
cgccucc
.(((((((.(((((.((((((((.(((..((((((((...........))))))))..))).))))))))).)))).))
).)))).
--UAAGGCACGCGGUGAAUGCCAu------
------- 2 0,1
--UAAGGCACGCGGUGAAUGCCAA------
------- 3 0,2

>hsa-mir-1245 3p:45-65 SRX018959
auuuauguauaggccuuuagaucaucugauguugaauacucuuuAAGUGAUCUAAAGGCCUACAUauaaa
.(((((((.((((((((((((((..(((.((.....))...)))..)))))))))))))).)))))))
------GUAUAGGCCUUUAGAUCAUCUGA--- 3

>hsa-mir-1246 5p:11-29 SRX018959
uguauccuugAAUGGAUUUUUGGAGCAGGaguggacaccugacccaaaggaaaucaauccauaggcuagcaau
(((..(((...((((((((((((..((((.(....).))))..)))))).......))))))))))...)))..
----------AAUGGAUUUUUGGAGCAGG-- 10
0,0
-----------AUGGAUUUUUGGAGCAGG-- 8
1,0

>hsa-mir-1247 5p:40-61 SRX018959
ccgcuugccucgcccagcgcagccccggccgcugggcgcACCCGUCCCGUUCGUCCCCGGAcguugcucucuaccccggg
aacgucgagacuggagcgcccgaacugagccaccuucgcggaccccgagagcggcg
.((((.((((((.((.(((.((....(((((.(((((((.((.(((.((..(((.(((((..((........)).)))))
.))).)).))).)).))))))))...)).)))..)).)))))....)))).))))))
---------------------------------------ACCCGUCCCGUUCGUCCCCGGAa------------------
-- 2 0,0

>hsa-mir-1252   5p:5-26 SRX018959
agaaAGAAGGAAAUUGAAUUCAUUUAgaaaagagaauuccaaaugagcuuaauuuccuuuuuucu
(((((((((((((((((.(((((((.(((.......))).))))))).)))))))))))))))))
----AGAAGGAAAUUGAAUUCAUUU--   2   0,-1

>hsa-mir-1254   5p:18-41   SRX018959
ggugggaggauugcuugAGCCUGGAAGCUGGAGCCUGCAGUgaacuaucauugugccacuguacuccagccuaggcaaca
aaaugaaauccugucua
...((.((((((..(((.((((((..(((((((..(((((((.((.......))..))))))))))))))))))))..))
).....)))))).))..
-----------------AGCCUGGAAGCUGGAGCCUGCAGU---------------------------------------
-----------------   2   0,0

>hsa-mir-1255b-1   5p:3-24 SRX018959
uaCGGAUGAGCAAAGAAAGUGGUUucuuaaaauggaaucuacucuuugugaagaugcugugaa
(((((.(..(((((((..((((.((((......)))).)))))))))))..)....)))))..
--CGGAUGAGCAAAGAAAGUGGUU--------------------------------------- 17   0,0
--CGGAUGAGCAAAGAAAGUGGUUa-------------------------------------- 2   0,0
--CGGAUGAGCAAAGAAAGUGGUUU-------------------------------------- 3   0,1

>hsa-mir-1255b-2   5p:6-27 SRX018959
ucuuaCGGAUGAGCAAAGAAAGUGGUUugcgccucaagaaaccacuuucuuugcucauccauaagga
(((((.((((((((((((((((((((((.(.......))))))))))))))))))))))).))))).
-----CGGAUGAGCAAAGAAAGUGGUU-- 17   0,0
-----CGGAUGAGCAAAGAAAGUGGUUa--------------------------------------- 2   0,0
-----CGGAUGAGCAAAGAAAGUGGUUU--------------------------------------- 3   0,1

>hsa-mir-1256   5p:35-56   SRX018959
agucagccuguugaagcuuugaagcuuugaugccAGGCAUUGACUUCUCACUAGCUgugaaaguccuagcuaaagagaag
ucaaugcaugacaucuuguuucaauagauggcuguuuca
(((((..((((((((((..........((((.((.(((((((((((((..((((((.(.....).))))))..))))))
))))))).)).))))..)))))))))).)))))......
----------------------------------AGGCAUUGACUUCUCACUAGCU------------------------
--------------------------------------- 5   0,0
--UAAAGAGAAG
UCAAUGCAUG----------------------------- 3

>hsa-mir-1258   3p:44-64   SRX018959

cuguggcuuccacgaccuaauccuaacuccugcgagucccuggAGUUAGGAUUAGGUCGUGGAAgccacagga
((((((((((((((((((((((((((((((...........))))))))))))))))))))))))))))))..
----------CACGACCUAAUCCUAACU---	2
--GAGUUAGGAUUAGGUCGU-------------	2
-1,-4
---AGUUAGGAUUAGGUCGUG------------	2
0,-3

>hsa-mir-125a	5p:15-38;3p:53-74	SRX018959
ugccagucucuaggUCCCUGAGACCCUUUAACCUGUGAggacauccagggucACAGGUGAGGUUCUUGGGAGCCuggcgu
cuggcc
.((((.(.(((((..((..(((.((((..(((((((((((....))....)))))))))))).)))..))..))))).).
))))).
--------------UCCCUGAGACCCUUUAACC---
------	700	0,-5
--------------UCCCUGAGACCCUUUAACCa--
------	3	0,-5
--------------UCCCUGAGACCCUUUAACCU--
------	7411	0,-4
--------------UCCCUGAGACCCUUUAACCUu---
------	6	0,-4
--------------UCCCUGAGACCCUUUAACCUG---
------	724	0,-3
--------------UCCCUGAGACCCUUUAACCUGa--
------	29	0,-3
--------------UCCCUGAGACCCUUUAACCUGaa---
------	9	0,-3
--------------UCCCUGAGACCCUUUAACCUGU--
------	721	0,-2
--------------UCCCUGAGACCCUUUAACCUGUa---
------	10	0,-2
--------------UCCCUGAGACCCUUUAACCUGUu---
------	12	0,-2
--------------UCCCUGAGACCCUUUAACCUGUG---
------	221	0,-1
--------------UCCCUGAGACCCUUUAACCUGUGu--
------	10	0,-1
--------------UCCCUGAGACCCUUUAACCUGUGA--

------ 30 0,0
----------------CCUGAGACCCUUUAACCU--
------ 16 2,-4
----------------CCUGAGACCCUUUAACCUG---
------ 5 2,-3
----------------CCUGAGACCCUUUAACCUGU--
------ 18 2,-2
----------------CCUGAGACCCUUUAACCUGUG---
------ 6 2,-1
----------------CCUGAGACCCUUUAACCUGUGA--
------ 2 2,0
---CAGGUGAGGUUCUUGGGAGCaa-----
------ 2 1,-1

>hsa-mir-125b-1 MA:15-36;mi:55-76 SRX018959
ugcgcuccucucagUCCCUGAGACCCUAACUUGUGAuguuuaccguuuaaauccACGGGUUAGGCUCUUGGGAGCUgcga
gucgugcu
.((((..(((.(((..((..(((.(((((((((((..(((((.....))))).))))))))))).)))..))..))).))
)..)))).
--------------UCCCUGAGACCCUAACUU--
-------- 158 0,-4
--------------UCCCUGAGACCCUAACUUa---
-------- 3 0,-4
--------------UCCCUGAGACCCUAACUUaa--
-------- 5 0,-4
--------------UCCCUGAGACCCUAACUUG---
-------- 498 0,-3
--------------UCCCUGAGACCCUAACUUGa--
-------- 16 0,-3
--------------UCCCUGAGACCCUAACUUGaa---
-------- 6 0,-3
--------------UCCCUGAGACCCUAACUUGU--
-------- 1668 0,-2
--------------UCCCUGAGACCCUAACUUGUa---
-------- 17 0,-2
--------------UCCCUGAGACCCUAACUUGUaa--
-------- 21 0,-2
--------------UCCCUGAGACCCUAACUUGUu---

-------- 11 0,-2

--------------UCCCUGAGACCCUAACUUGUG---

-------- 3526 0,-1

--------------UCCCUGAGACCCUAACUUGUGu--

-------- 209 0,-1

--------------UCCCUGAGACCCUAACUUGUGA--

-------- 10487 0,0

--------------UCCCUGAGACCCUAACUUGUGAa---

-------- 2570 0,0

--------------UCCCUGAGACCCUAACUUGUGAaa--

-------- 338 0,0

--------------UCCCUGAGACCCUAACUUGUGAau--

-------- 14 0,0

--------------UCCCUGAGACCCUAACUUGUGAU---

-------- 122 0,1

--------------UCCCUGAGACCCUAACUUGUGAUa--

-------- 30 0,1

--------------UCCCUGAGACCCUAACUUGUGAUG--

-------- 2 0,2

---------------CCCUGAGACCCUAACUUGUG---

-------- 2 1,-1

---------------CCCUGAGACCCUAACUUGUGA--

-------- 2 1,0

---------------CCCUGAGACCCUAACUUGUGAa---

-------- 3 1,0

---------------CCCUGAGACCCUAACUUGUGAaa--

-------- 2 1,0

---------------CCCUGAGACCCUAACUUGUGAU---

-------- 6 1,1

----------------CCUGAGACCCUAACUUGU--

-------- 16 2,-2

----------------CCUGAGACCCUAACUUGUG---

-------- 19 2,-1

----------------CCUGAGACCCUAACUUGUGu--

-------- 3 2,-1

----------------CCUGAGACCCUAACUUGUGA--

-------- 71 2,0

----------------CCUGAGACCCUAACUUGUGAa---

--------   12 2,0
----------------CCUGAGACCCUAACUUGUGAaa--
--------   4   2,0
----------------CCUGAGACCCUAACUUGUGAU---
--------   5   2,1
--ACGGGUUAGGCUCUUGGGAGCU----
--------   2   0,0

>hsa-mir-125b-2 MA:17-38;mi:54-75   SRX018959
accagacuuuuccuagUCCCUGAGACCCUAACUUGUGAgguauuuuaguaacaUCACAAGUCAGGCUCUUGGGACcuagg
cggaggga
.((...(((..(((((..((..(((.(((.((((((((..(((....)))...)))))))).))).)))..))..)))))
..))).)).
----------------UCCCUGAGACCCUAACUU--
---------   158 0,-4
----------------UCCCUGAGACCCUAACUUa---
---------   3   0,-4
----------------UCCCUGAGACCCUAACUUaa--
---------   5   0,-4
----------------UCCCUGAGACCCUAACUUG---
---------   498 0,-3
----------------UCCCUGAGACCCUAACUUGa--
---------   16 0,-3
----------------UCCCUGAGACCCUAACUUGaa---
---------   6   0,-3
----------------UCCCUGAGACCCUAACUUGU--
---------   1668   0,-2
----------------UCCCUGAGACCCUAACUUGUa---
---------   17 0,-2
----------------UCCCUGAGACCCUAACUUGUaa--
---------   21 0,-2
----------------UCCCUGAGACCCUAACUUGUu---
---------   11 0,-2
----------------UCCCUGAGACCCUAACUUGUG---
---------   3526   0,-1
----------------UCCCUGAGACCCUAACUUGUGu--
---------   209 0,-1
----------------UCCCUGAGACCCUAACUUGUGA--

--------- 10487 0,0

----------------UCCCUGAGACCCUAACUUGUGAa---

--------- 2570 0,0

----------------UCCCUGAGACCCUAACUUGUGAaa--

--------- 338 0,0

----------------UCCCUGAGACCCUAACUUGUGAau--

--------- 14 0,0

----------------UCCCUGAGACCCUAACUUGUGAG---

--------- 14 0,1

-----------------CCCUGAGACCCUAACUUGUG---

--------- 2 1,-1

-----------------CCCUGAGACCCUAACUUGUGA--

--------- 2 1,0

-----------------CCCUGAGACCCUAACUUGUGAa---

--------- 3 1,0

-----------------CCCUGAGACCCUAACUUGUGAaa--

--------- 2 1,0

------------------CCUGAGACCCUAACUUGU--

--------- 16 2,-2

------------------CCUGAGACCCUAACUUGUG---

--------- 19 2,-1

------------------CCUGAGACCCUAACUUGUGu--

--------- 3 2,-1

------------------CCUGAGACCCUAACUUGUGA--

--------- 71 2,0

------------------CCUGAGACCCUAACUUGUGAa---

--------- 12 2,0

------------------CCUGAGACCCUAACUUGUGAaa--

--------- 4 2,0

--AUCACAAGUCAGGCUCUU----------

--------- 2 -1,-5

--AUCACAAGUCAGGCUCUUG---------

--------- 14 -1,-4

--AUCACAAGUCAGGCUCUUGG--------

--------- 19 -1,-3

--AUCACAAGUCAGGCUCUUGGG-------

--------- 3 -1,-2

--AUCACAAGUCAGGCUCUUGGGAC-----

---------   5   -1,0
---UCACAAGUCAGGCUCUUG---------
---------   9   0,-4
---UCACAAGUCAGGCUCUUGG--------
---------   7   0,-3
---UCACAAGUCAGGCUCUUGGG-------
---------   5   0,-2
---ACAAGUCAGGCUCUUGGG-------
---------   42   2,-2
---ACAAGUCAGGCUCUUGGGA------
---------   27   2,-1
---ACAAGUCAGGCUCUUGGGAa-----
---------   6   2,-1
---ACAAGUCAGGCUCUUGGGAC-----
---------   32   2,0
---ACAAGUCAGGCUCUUGGGACC----
---------   8   2,1
---ACAAGUCAGGCUCUUGGGACCU---
---------   8   2,2
---ACAAGUCAGGCUCUUGGGACCUu--
---------   4   2,2
---ACAAGUCAGGCUCUUGGGACCUuu-
---------   2   2,2
---ACAAGUCAGGCUCUUGGGACCUAu-
---------   3   2,3

>hsa-mir-126   mi:15-35;MA:52-73   SRX018959
cgcuggcgacgggaCAUUAUUACUUUUGGUACGCGcugugacacuucaaacUCGUACCGUGAGUAAUAAUGCGccgucca
cggca
.((((..(((((.((((((((((((.(((((((.(...(((....)))..).))))))).))))))))))))).)))))..
)))).
--------------CAUUAUUACUUUUGGUAC--
-----   113 0,-3
--------------CAUUAUUACUUUUGGUACG---
-----   51   0,-2
--------------CAUUAUUACUUUUGGUACGC--
-----   52   0,-1
--------------CAUUAUUACUUUUGGUACGCG---

-----   509 0,0
--------------CAUUAUUACUUUUGGUACGCGa--
-----   167 0,0
--------------CAUUAUUACUUUUGGUACGCGu--
-----   72   0,0
--------------CAUUAUUACUUUUGGUACGCGuu---
-----   3   0,0
--------------CAUUAUUACUUUUGGUACGCGC--
-----   7   0,1
--------------CAUUAUUACUUUUGGUACGCGCa---
-----   3   0,1
--------------CAUUAUUACUUUUGGUACGCGCU---
-----   3   0,2
---------------AUUAUUACUUUUGGUACGC--
-----   9   1,-1
---------------AUUAUUACUUUUGGUACGCG---
-----   20   1,0
---------------AUUAUUACUUUUGGUACGCGa--
-----   6   1,0
---------------AUUAUUACUUUUGGUACGCGaa---
-----   3   1,0
---------------AUUAUUACUUUUGGUACGCGu--
-----   4   1,0
---------------AUUAUUACUUUUGGUACGCGC--
-----   4   1,1
---------------AUUAUUACUUUUGGUACGCGCU---
-----   14   1,2
---------------AUUAUUACUUUUGGUACGCGCUa--
-----   3   1,2
--CUCGUACCGUGAGUAAUAAU----------
-----   3   -1,-3
--CUCGUACCGUGAGUAAUAAUaa--------
-----   2   -1,-3
--CUCGUACCGUGAGUAAUAAUG---------
-----   14   -1,-2
--CUCGUACCGUGAGUAAUAAUGa--------
-----   4   -1,-2
--CUCGUACCGUGAGUAAUAAUGaa-------

-----   2   -1,-2
--CUCGUACCGUGAGUAAUAAUGC--------
-----   41   -1,-1
--CUCGUACCGUGAGUAAUAAUGCG-------
-----   14   -1,0
---UCGUACCGUGAGUAAUAA-----------
-----   2   0,-4
---UCGUACCGUGAGUAAUAAU----------
-----   23   0,-3
---UCGUACCGUGAGUAAUAAUa---------
-----   5   0,-3
---UCGUACCGUGAGUAAUAAUG---------
-----   24   0,-2
---UCGUACCGUGAGUAAUAAUGa--------
-----   21   0,-2
---UCGUACCGUGAGUAAUAAUGaa-------
-----   13   0,-2
---UCGUACCGUGAGUAAUAAUGC--------
-----   232   0,-1
---UCGUACCGUGAGUAAUAAUGCa-------
-----   4   0,-1
---UCGUACCGUGAGUAAUAAUGCG-------
-----   121   0,0
---UCGUACCGUGAGUAAUAAUGCGa------
-----   82   0,0
---UCGUACCGUGAGUAAUAAUGCGu------
-----   6   0,0
--CGUACCGUGAGUAAUAAU----------
-----   4   1,-3
--CGUACCGUGAGUAAUAAUG---------
-----   17   1,-2
--CGUACCGUGAGUAAUAAUGaa-------
-----   3   1,-2
--CGUACCGUGAGUAAUAAUGC--------
-----   70   1,-1
--CGUACCGUGAGUAAUAAUGCa-------
-----   3   1,-1
--CGUACCGUGAGUAAUAAUGCu-------

-----   2   1,-1
--CGUACCGUGAGUAAUAAUGCG-------
-----   84   1,0
--CGUACCGUGAGUAAUAAUGCGa------
-----   29   1,0
--CGUACCGUGAGUAAUAAUGCGu------
-----   11   1,0
---GUACCGUGAGUAAUAAUG---------
-----   5   2,-2
---GUACCGUGAGUAAUAAUGC--------
-----   26   2,-1
---GUACCGUGAGUAAUAAUGCG-------
-----   19   2,0
---GUACCGUGAGUAAUAAUGCGa------
-----   11   2,0

>hsa-mir-1260b 5p:10-28   SRX018959
ucuccguuuAUCCCACCACUGCCACCAUuauugcuacuguucagcaggugcugcugguggugauggugauagucuggugg
gggcggugg
.(.((((...(((((((((((.(((((((((..(((.....((((....)))).)))..))))))))).)))).)))))
)))))).).
---------AUCCCACCACUGCCACCA---
---------   2   0,-1

>hsa-mir-127   5p:23-44;3p:57-78   SRX018959
ugugaucacugucuccagccugCUGAAGCUCAGAGGGCUCUGAUucagaaagaucaUCGGAUCCGUCUGAGCUUGGCUgg
ucggaagucucaucauc
((.(((..(((...((((((.....((((((((.((.(((((((((.....))..)))))).)).)))))))))))))))
.)))..))).)).....
----------------------CUGAAGCUCAGAGGGCUC--
-----------------   14   0,-4
----------------------CUGAAGCUCAGAGGGCUCU---------------------------------------
-----------------   8   0,-3
----------------------CUGAAGCUCAGAGGGCUCUG--------------------------------------
-----------------   2   0,-2
----------------------CUGAAGCUCAGAGGGCUCUGA-------------------------------------
-----------------   10   0,-1
----------------------CUGAAGCUCAGAGGGCUCUGAU------------------------------------

----------------- 6 0,0
--UCGGAUCCGUCUGAGCUU------
----------------- 3 0,-4
--UCGGAUCCGUCUGAGCUUG-----
----------------- 2 0,-3
--UCGGAUCCGUCUGAGCUUGG----
----------------- 18 0,-2
--UCGGAUCCGUCUGAGCUUGGa---
----------------- 3 0,-2
--UCGGAUCCGUCUGAGCUUGGaa--
----------------- 2 0,-2
--UCGGAUCCGUCUGAGCUUGGuu--
----------------- 3 0,-2
--UCGGAUCCGUCUGAGCUUGGC---
----------------- 60 0,-1
--UCGGAUCCGUCUGAGCUUGGCa--
----------------- 69 0,-1
--UCGGAUCCGUCUGAGCUUGGCaa-
----------------- 49 0,-1
--UCGGAUCCGUCUGAGCUUGGCau-
----------------- 255 0,-1
--UCGGAUCCGUCUGAGCUUGGCU--
----------------- 440 0,0
--UCGGAUCCGUCUGAGCUUGGCUa-
----------------- 21 0,0
--UCGGAUCCGUCUGAGCUUGGCUaa
----------------- 36 0,0
--UCGGAUCCGUCUGAGCUUGGCUau
----------------- 192 0,0
--UCGGAUCCGUCUGAGCUUGGCUu-
----------------- 174 0,0
--UCGGAUCCGUCUGAGCUUGGCUuu
----------------- 63 0,0
---CGGAUCCGUCUGAGCUUGGCUaa
----------------- 4 1,0
---CGGAUCCGUCUGAGCUUGGCUau
----------------- 2 1,0
---CGGAUCCGUCUGAGCUUGGCUu-

---------------- 2   1,0
--GGAUCCGUCUGAGCUUGG----
---------------- 2   2,-2
--GGAUCCGUCUGAGCUUGGCau-
---------------- 4   2,-1
--GGAUCCGUCUGAGCUUGGCU--
---------------- 10 2,0
--GGAUCCGUCUGAGCUUGGCUau
---------------- 2   2,0
--GGAUCCGUCUGAGCUUGGCUu-
---------------- 11 2,0
--GGAUCCGUCUGAGCUUGGCUuu
---------------- 2   2,0

>hsa-mir-1270-1 5p:13-35   SRX018959
cacagaguuauaCUGGAGAUAUGGAAGAGCUGUGUuggguauaaguaacaggcuuuucuuuaucuucuauguggcucuuu
gca
((.(((((((((..(((((((.(((((((((.(((((.........)))))))))))))).)))))))..))))))))).)
)..
------------CUGGAGAUAUGGAAGAGC---
--- 3   0,-5

>hsa-mir-1270-2 5p:13-35   SRX018959
cacagaguuauaCUGGAGAUAUGGAAGAGCUGUGUuggguauaaguaacaggcuuuucuuuaucuucuauguggcucuuu
gca
((.(((((((((..(((((((.(((((((((.(((((.........)))))))))))))).)))))))..))))))))).)
)..
------------CUGGAGAUAUGGAAGAGC---
--- 3   0,-5

>hsa-mir-1271   5p:15-36   SRX018959
cacccagaucagugCUUGGCACCUAGCAAGCACUCAguaaauauuuguugagugccugcuaugugccaggcauugugcug
agggcu
..(((((..(((((((((((((.(((((.(((((((((((....))))))))))).))))).)))))))))))))..)))
.))...
--------------CUUGGCACCUAGCAAGCA--
------ 2   0,-4
--------------CUUGGCACCUAGCAAGCAC---

------ 37 0,-3
--------------CUUGGCACCUAGCAAGCACU---
------ 36 0,-2
--------------CUUGGCACCUAGCAAGCACUCA---
------ 2 0,0

>hsa-mir-1273c 5p:10-31 SRX018959
ugcagccugGGCGACAAAACGAGACCCUGUCuuuuuuuuuucugagacagagucucguucuguugcccaagcugga
..((((.(((((((((.((((((((.(((((((..........))))))).)))))))).))))))))).))))..
---------GGCGACAAAACGAGACCC---
 2 0,-4
---------GGCGACAAAACGAGACCCU--
 10 0,-3
---------GGCGACAAAACGAGACCCUGU--
 3 0,-1
---------GGCGACAAAACGAGACCCUGUa---
 2 0,-1
---------GGCGACAAAACGAGACCCUGUu---
 4 0,-1
---------GGCGACAAAACGAGACCCUGUCa--
 7 0,0
---------GGCGACAAAACGAGACCCUGUCaa---
 3 0,0
---------GGCGACAAAACGAGACCCUGUCau---
 2 0,0

>hsa-mir-1273e 5p:14-35 SRX018959
ugaggcaggagaaUUGCUUGAACCCAGGAAGUGGAggcugcagugagccgagaucgagccacuguacuccagccugggug
acacagcgagacuccagucuca
.((((.((((..((((((.((((((...(((((..(((((((.((((....)).))))))))))))).))))))).
.)).)))))..)))).)))))).
--UGGGUG
ACACAGCGAGAC---------- 3

>hsa-mir-1277 3p:47-68 SRX018959
accucccaaauauauauauauauguacguauguguauauaaauguaUACGUAGAUAUAUAUGUAUUUUugguggguuu
(((..(((((.((((((((((((.((((((((..(......)..)))))))).))))))))))))).))))).)))..
---AUACGUAGAUAUAUAUGUAUUU-----------

2   -1,-1
--UACGUAGAUAUAUAUGUAU-------------
2   0,-3
--UACGUAGAUAUAUAUGUAUa------------
2   0,-3
--UACGUAGAUAUAUAUGUAUU------------
7   0,-2
--UACGUAGAUAUAUAUGUAUUU-----------
19   0,-1
--UACGUAGAUAUAUAUGUAUUUU----------
96   0,0
---ACGUAGAUAUAUAUGUAUUUU----------
2   1,0

>hsa-mir-1278   3p:50-71   SRX018959
auuugcucauagaugauaugcauaguacucccagaacucauuaaguuggUAGUACUGUGCAUAUCAUCUAUgagcgaaua
g
(((((((((((((((((((((((((((((.(((..(((.....)))))).))))))))))))))))))))))))))))).
.
--GUAGUACUGUGCAUAUCAUCU-----------
-    2   -1,-2
--GUAGUACUGUGCAUAUCAUCUA----------
-    4   -1,-1
---UAGUACUGUGCAUAUCAU-------------
-    2   0,-4
---UAGUACUGUGCAUAUCAUa------------
-    4   0,-4
---UAGUACUGUGCAUAUCAUCU-----------
-    25   0,-2
---UAGUACUGUGCAUAUCAUCUA----------
-    14   0,-1
---UAGUACUGUGCAUAUCAUCUAU---------
-    94   0,0
---UAGUACUGUGCAUAUCAUCUAUa--------
-    4   0,0

>hsa-mir-128-1   3p:50-70   SRX018959
ugagcuguuggauucggggccguagcacugucugagagguuuacauuucUCACAGUGAACCGGUCUCUUUuucagcugcu

uc
.((((.((((((...(((((((...((((((..(((((((....)))))))))))))...)))))))...)))))).)))
).
--CUCACAGUGAACCGGUCUCUUa----------
-- 3 -1,-1
--CUCACAGUGAACCGGUCUCUUU----------
-- 5 -1,0
---UCACAGUGAACCGGUCUC-------------
-- 43 0,-3
---UCACAGUGAACCGGUCUCU------------
-- 117 0,-2
---UCACAGUGAACCGGUCUCUaa----------
-- 6 0,-2
---UCACAGUGAACCGGUCUCUau----------
-- 3 0,-2
---UCACAGUGAACCGGUCUCUU-----------
-- 260 0,-1
---UCACAGUGAACCGGUCUCUUa----------
-- 73 0,-1
---UCACAGUGAACCGGUCUCUUaa---------
-- 38 0,-1
---UCACAGUGAACCGGUCUCUUau---------
-- 37 0,-1
---UCACAGUGAACCGGUCUCUUU----------
-- 2691 0,0
---UCACAGUGAACCGGUCUCUUUa---------
-- 579 0,0
---UCACAGUGAACCGGUCUCUUUaa--------
-- 97 0,0
---UCACAGUGAACCGGUCUCUUUau--------
-- 84 0,0
---UCACAGUGAACCGGUCUCUUUU---------
-- 1427 0,1
---UCACAGUGAACCGGUCUCUUUUa--------
-- 73 0,1
---UCACAGUGAACCGGUCUCUUUUaa-------
-- 5 0,1
---UCACAGUGAACCGGUCUCUUUUau-------

-- 4   0,1
---UCACAGUGAACCGGUCUCUUUUU--------
-- 66 0,2
---UCACAGUGAACCGGUCUCUUUUUa-------
-- 3   0,2
---UCACAGUGAACCGGUCUCUUUUUu-------
-- 6   0,2
--CACAGUGAACCGGUCUCUUU----------
-- 5   1,0
---ACAGUGAACCGGUCUCUU-----------
-- 2   2,-1
---ACAGUGAACCGGUCUCUUU----------
-- 17 2,0
---ACAGUGAACCGGUCUCUUUa---------
-- 6   2,0
---ACAGUGAACCGGUCUCUUUU---------
-- 13 2,1

>hsa-mir-128-2 3p:52-72   SRX018959
ugugcagugggaagggggggccgauacacuguacgagagugaguagcaggucUCACAGUGAACCGGUCUCUUUcccuacug
uguc
.(..(((((((.((((((((((...((((((..((((.(........).))))))))))...)))))))))).)))))))
..).
--CUCACAGUGAACCGGUCUCUUa--------
----   3   -1,-1
--CUCACAGUGAACCGGUCUCUUU--------
----   5   -1,0
---UCACAGUGAACCGGUCUC-----------
----   43 0,-3
---UCACAGUGAACCGGUCUCU----------
----   117 0,-2
---UCACAGUGAACCGGUCUCUaa--------
----   6   0,-2
---UCACAGUGAACCGGUCUCUau--------
----   3   0,-2
---UCACAGUGAACCGGUCUCUU---------
----   260 0,-1
---UCACAGUGAACCGGUCUCUUa--------

---- 73 0,-1

---UCACAGUGAACCGGUCUCUUaa-------

---- 38 0,-1

---UCACAGUGAACCGGUCUCUUau-------

---- 37 0,-1

---UCACAGUGAACCGGUCUCUUU--------

---- 2691 0,0

---UCACAGUGAACCGGUCUCUUUa-------

---- 579 0,0

---UCACAGUGAACCGGUCUCUUUaa------

---- 97 0,0

---UCACAGUGAACCGGUCUCUUUau------

---- 84 0,0

---UCACAGUGAACCGGUCUCUUUC-------

---- 24 0,1

---UCACAGUGAACCGGUCUCUUUCa------

---- 4 0,1

---UCACAGUGAACCGGUCUCUUUCu------

---- 6 0,1

--CACAGUGAACCGGUCUCUUU--------

---- 5 1,0

---ACAGUGAACCGGUCUCUU---------

---- 2 2,-1

---ACAGUGAACCGGUCUCUUU--------

---- 17 2,0

---ACAGUGAACCGGUCUCUUUa-------

---- 6 2,0

>hsa-mir-1284 5p:30-51 SRX018959

auuuugauauauaagccaguuuaauguuuUCUAUACAGACCCUGGCUUUUCuuaaauuuuauauauuggaaagcccaugu

uuguauuggaaacugcugguuucuuucauacugaaaaucu

....(((.....((((((((.....((((((.((((((((...((((((((................))))))))...))

)))))).)))))).))))))))...)))............

-----------------------------UCUAUACAGACCCUGGCUUUUC-----------------------------

-- 4 0,0

>hsa-mir-1285-1 3p:51-72 SRX018959

uguagagauaggaucucacuuuguugcccaggcuggucucaaacuccuggUCUGGGCAACAAAGUGAGACCUuaucucua

caag
((((((((((((.((((((((((((((((((((..(..........)..)))))))))))))))))))).))))))))))
))..
--UCUGGGCAACAAAGUGAGA-----------
----	4	0,-3
--UCUGGGCAACAAAGUGAGAC----------
----	29	0,-2
--UCUGGGCAACAAAGUGAGACa---------
----	5	0,-2
--UCUGGGCAACAAAGUGAGACaa--------
----	6	0,-2
--UCUGGGCAACAAAGUGAGACau--------
----	3	0,-2
--UCUGGGCAACAAAGUGAGACC---------
----	13	0,-1
--UCUGGGCAACAAAGUGAGACCaa-------
----	4	0,-1
--UCUGGGCAACAAAGUGAGACCau-------
----	12	0,-1
--UCUGGGCAACAAAGUGAGACCU--------
----	46	0,0
--UCUGGGCAACAAAGUGAGACCUa-------
----	33	0,0
--UCUGGGCAACAAAGUGAGACCUaa------
----	11	0,0
--UCUGGGCAACAAAGUGAGACCUau------
----	41	0,0
--UCUGGGCAACAAAGUGAGACCUU-------
----	33	0,1
--UCUGGGCAACAAAGUGAGACCUUu------
----	18	0,1
--UCUGGGCAACAAAGUGAGACCUUuu-----
----	5	0,1
--UCUGGGCAACAAAGUGAGACCUUA------
----	2	0,2
---CUGGGCAACAAAGUGAGACC---------
----	2	1,-1
---CUGGGCAACAAAGUGAGACCUUu------

----   2   1,1

>hsa-mir-1285-2 3p:52-73   SRX018959
uuugggaggccgaggcuggugcaucacuugagcccagcaauuugagaccaaUCUGGGCAACAAAGUGAGACCUccgucuc
uacaaaga
((((((((((.((((........((((((..((((((....(((....))).))))))....))))))..)))).)))))
).))))..
---UCUGGGCAACAAAGUGAGA----------
--------   4   0,-3
---UCUGGGCAACAAAGUGAGAC---------
--------   29 0,-2
---UCUGGGCAACAAAGUGAGACa--------
--------   5   0,-2
---UCUGGGCAACAAAGUGAGACaa-------
--------   6   0,-2
---UCUGGGCAACAAAGUGAGACau-------
--------   3   0,-2
---UCUGGGCAACAAAGUGAGACC--------
--------   13 0,-1
---UCUGGGCAACAAAGUGAGACCaa------
--------   4   0,-1
---UCUGGGCAACAAAGUGAGACCau------
--------   12 0,-1
---UCUGGGCAACAAAGUGAGACCU-------
--------   46 0,0
---UCUGGGCAACAAAGUGAGACCUa------
--------   33 0,0
---UCUGGGCAACAAAGUGAGACCUaa-----
--------   11 0,0
---UCUGGGCAACAAAGUGAGACCUau-----
--------   41 0,0
--CUGGGCAACAAAGUGAGACC--------
--------   2   1,-1

>hsa-mir-1286   3p:47-67   SRX018959
uguccucuggggacucagcuugcucuggcugcuggauugaauuagcUGCAGGACCAAGAUGAGCCCUugguggagaca
..(((.(..(((.((((.((((.(((.((.(((((......))))).)).))).)))).)))).)))..).)))....
--UGCAGGACCAAGAUGAGCCCUu----------

2 0,1

>hsa-mir-1287 5p:16-37 SRX018959
guugugcuguccaggUGCUGGAUCAGUGGUUCGAGUCugagccuuuaaaagccacucuagccacagaugcagugauugga
gccaugacaa
((..((...(((((..((((.(((.((((((.((((..(.((........))))))).)))))).))).)))))..)))))
..))..))..
---------------UGCUGGAUCAGUGGUUCGAGUu---
---------- 2 0,-1

>hsa-mir-129-2 5p:15-35;3p:57-78 SRX018959
ugcccuucgcgaauCUUUUUGCGGUCUGGGCUUGCuguacauaacucaauagccggAAGCCCUUACCCCAAAAAGCAUuu
gcggagggcg
.(((((((((((((((((((.(((..((((((((((.........)))))...))))))..))).))))))).))))
)))))))).
--AAGCCCUUACCCCAAAAAGCAU--
---------- 2 0,0

>hsa-mir-1291 5p:14-37 SRX018959
gguagaauuccagUGGCCCUGACUGAAGACCAGCAGUuguacuguggcuguugguuucaagcagaggccuaaaggacugu
cuuccug
((.(((..(((...((((((.((..((((((((((..(...)..))))))))))..))))).))))....)))...)
)).))..
------------GUGGCCCUGACUGAAGACCA---
------- 5 -1,-5
------------GUGGCCCUGACUGAAGACCAG--
------- 2 -1,-4
------------GUGGCCCUGACUGAAGACCAGC---
------- 3 -1,-3
------------GUGGCCCUGACUGAAGACCAGCA--
------- 91 -1,-2
-------------UGGCCCUGACUGAAGACCAGCA--
------- 16 0,-2

>hsa-mir-1301 3p:48-71 SRX018959
ggauugugggggggucgcucuaggcaccgcagcacugugcuggggaugUUGCAGCUGCCUGGGAGUGACUUCacacagucc
uc
((((((((.(((((((((((((((((.((((((((.......)).)))))).).))))).))))))))))).))))))))

..
---UUGCAGCUGCCUGGGAGUGACU-----------
-- 4 0,-2
---UUGCAGCUGCCUGGGAGUGACUUC---------
-- 2 0,0
--UGCAGCUGCCUGGGAGUGACU-----------
-- 3 1,-2

>hsa-mir-1303 3p:52-73 SRX018959
ggcugggcaacauagcgagaccucaacucuacaauuuuuuuuuuuuuaaauUUUAGAGACGGGGUCUUGCUCUguugcca
ggcuuu
((((.(((((((.(((((((((((..(((((.(((((..........))))).)))))..))))))))))).))))))).
))))..
----GGGCAACAUAGCGAGACC--
------ 2
--UUUUAGAGACGGGGUCUUGCU---------
------ 14 -1,-2
---UUUAGAGACGGGGUCUUGC----------
------ 2 0,-3
---UUUAGAGACGGGGUCUUGCU---------
------ 19 0,-2

>hsa-mir-1306 3p:55-72 SRX018959
gugagcagucuccaccaccuccccugcaaacguccaguggugcagagguaauggACGUUGGCUCUGGUGGUGauggacag
uccga
.((..(.((((.(((((((......((.((((((((....(((....))).)))))))).))...)))))))..)))).)
..)).
--------------CCACCUCCCCUGCAAACGUCCA--
----- 2
---GACGUUGGCUCUGGUGGUGAU------
----- 2 -1,2
--ACGUUGGCUCUGGUGGUGAU------
----- 4 0,2
--ACGUUGGCUCUGGUGGUGAUG-----
----- 3 0,3
--ACGUUGGCUCUGGUGGUGAUGu----
----- 4 0,3

>hsa-mir-1307 3p:80-101 SRX018959

caucaagacccagcugagucacugucacugccuaccaaucucgaccggaccucgaccggcucgucuguguugccaaucgA
CUCGGCGUGGCGUCGGUCGUGguagauaggcggucaugcauacgaauuuucagcucuuguucuggugac
(((((..((..(((((((((..(((.((((((((.(.(((.(((((((.((.((.(((..(((..((......))..)))
..))))).))..))))))).))).).))))))))...)))...))...)))))))...))..)))))..
--UCGACCGGACCUCGACCGGuu-------------------
--- 2
--UCGACCGGACCUCGACCGGC--------------------
--- 2
--UCGACCGGACCUCGACCGGCU-------------------
--- 145
--UCGACCGGACCUCGACCGGCUaa-----------------
--- 3
--UCGACCGGACCUCGACCGGCUuu-----------------
--- 13
--UCGACCGGACCUCGACCGGCUC------------------
--- 24
--UCGACCGGACCUCGACCGGCUCa-----------------
--- 5
--UCGACCGGACCUCGACCGGCUCau----------------
--- 2
--UCGACCGGACCUCGACCGGCUCu-----------------
--- 7
--UCGACCGGACCUCGACCGGCUCG-----------------
--- 3
---A
CUCGGCGUGGCGUCGGUCG-- 18 0,-2
---A
CUCGGCGUGGCGUCGGUCGU--- 23 0,-1
---A
CUCGGCGUGGCGUCGGUCGUu-- 5 0,-1
---A
CUCGGCGUGGCGUCGGUCGUG-- 79 0,0
---A
CUCGGCGUGGCGUCGGUCGUGu--- 7 0,0

CUCGGCGUGGCGUCGGUCG-- 2 1,-2

CUCGGCGUGGCGUCGGUCGU-- 4 1,-1

CUCGGCGUGGCGUCGGUCGUG-- 58 1,0

CUCGGCGUGGCGUCGGUCGUGG--- 2 1,1

-UCGGCGUGGCGUCGGUCG-- 2 2,-2

>hsa-mir-130a mi:21-42;MA:55-76 SRX018959
ugcugcuggccagagcucuuUUCACAUUGUGCUACUGUCUGCaccugucacuagCAGUGCAAUGUUAAAAGGGCAUuggc
cguguagug
.((((((((((((.((((((((.((((((..((.(((...((....))...))).))..)))))).)))))))).)))))
)).))))).
--CAGUGCAAUGUUAAAAGG--------
--------- 2255 0,-4
--CAGUGCAAUGUUAAAAGGa-------
--------- 2 0,-4
--CAGUGCAAUGUUAAAAGGu-------
--------- 2 0,-4
--CAGUGCAAUGUUAAAAGGG-------
--------- 388 0,-3
--CAGUGCAAUGUUAAAAGGGaa-----
--------- 2 0,-3
--CAGUGCAAUGUUAAAAGGGC------
--------- 664 0,-2
--CAGUGCAAUGUUAAAAGGGCA-----
--------- 161 0,-1
--CAGUGCAAUGUUAAAAGGGCAa----
--------- 20 0,-1
--CAGUGCAAUGUUAAAAGGGCAaa---
--------- 6 0,-1
--CAGUGCAAUGUUAAAAGGGCAau---
--------- 10 0,-1
--CAGUGCAAUGUUAAAAGGGCAU----
--------- 794 0,0
--CAGUGCAAUGUUAAAAGGGCAUa---
--------- 206 0,0
--CAGUGCAAUGUUAAAAGGGCAUaa--

---------   15 0,0
--CAGUGCAAUGUUAAAAGGGCAUU---
---------   261 0,1
--CAGUGCAAUGUUAAAAGGGCAUUa--
---------   7   0,1
--CAGUGCAAUGUUAAAAGGGCAUUaa-
---------   3   0,1
--CAGUGCAAUGUUAAAAGGGCAUUau-
---------   3   0,1
--CAGUGCAAUGUUAAAAGGGCAUUu--
---------   9   0,1
---AGUGCAAUGUUAAAAGGG-------
---------   9   1,-3
---AGUGCAAUGUUAAAAGGGC------
---------   10 1,-2
---AGUGCAAUGUUAAAAGGGCA-----
---------   2   1,-1
---AGUGCAAUGUUAAAAGGGCAU----
---------   18 1,0
---AGUGCAAUGUUAAAAGGGCAUa---
---------   5   1,0
---AGUGCAAUGUUAAAAGGGCAUU---
---------   7   1,1

>hsa-mir-130b   mi:13-33;MA:51-72   SRX018959
ggccugcccgacACUCUUUCCCUGUUGCACUACuauaggccgcugggaagCAGUGCAAUGAUGAAAGGGCAUcggucagg
uc
((((((.((((..(((((((..(((((((((.((.(((....)))...)).)))))))))..)))))))..)))).))))
))
------------ACUCUUUCCCUGUUGCACUA--
-- 2   0,-1
------------ACUCUUUCCCUGUUGCACUAC---
-- 2   0,0
------------ACUCUUUCCCUGUUGCACUACU--
-- 9   0,1
--CAGUGCAAUGAUGAAAGG------------
-- 48 0,-4
--CAGUGCAAUGAUGAAAGGG-----------

-- 24 0,-3
--CAGUGCAAUGAUGAAAGGGC----------
-- 26 0,-2
--CAGUGCAAUGAUGAAAGGGCA---------
-- 11 0,-1
--CAGUGCAAUGAUGAAAGGGCAa--------
-- 26 0,-1
--CAGUGCAAUGAUGAAAGGGCAaa-------
-- 2 0,-1
--CAGUGCAAUGAUGAAAGGGCAau-------
-- 3 0,-1
--CAGUGCAAUGAUGAAAGGGCAU--------
-- 425 0,0
--CAGUGCAAUGAUGAAAGGGCAUa-------
-- 57 0,0
--CAGUGCAAUGAUGAAAGGGCAUaa------
-- 2 0,0
--CAGUGCAAUGAUGAAAGGGCAUau------
-- 4 0,0
--CAGUGCAAUGAUGAAAGGGCAUu-------
-- 88 0,0
--CAGUGCAAUGAUGAAAGGGCAUuu------
-- 15 0,0
---AGUGCAAUGAUGAAAGGGCAU--------
-- 13 1,0

>hsa-mir-132 mi:23-44;MA:59-80 SRX018959
ccgcccccgcgucuccagggcaACCGUGGCUUUCGAUUGUUACUgugggaacuggaggUAACAGUCUACAGCCAUGGUCG
ccccgcagcacgcccacgcgc
.(((....((((((.(.((((.(((((((((...((((((((((..(....)....))))))))))...))))))))).)
))).).)).))))....))).
---------------------AACCGUGGCUUUCGAUUGUU---------------------------------------
--------------------- 3 -1,-3
---------------------AACCGUGGCUUUCGAUUGUUA--------------------------------------
--------------------- 12 -1,-2
----------------------ACCGUGGCUUUCGAUUGUUA--------------------------------------
--------------------- 3 0,-2
----------------------ACCGUGGCUUUCGAUUGUUAC-------------------------------------

--------------------   2   0,-1
----------------------ACCGUGGCUUUCGAUUGUUACU------------------------------------
--------------------   3   0,0
--UAACAGUCUACAGCCAUG----
--------------------   23   0,-4
--UAACAGUCUACAGCCAUGG---
--------------------   17   0,-3
--UAACAGUCUACAGCCAUGGU--
--------------------   17   0,-2
--UAACAGUCUACAGCCAUGGUC-
--------------------   21   0,-1
--UAACAGUCUACAGCCAUGGUCa
--------------------   3   0,-1
--UAACAGUCUACAGCCAUGGUCG
--------------------   21   0,0
--UAACAGUCUACAGCCAUGGUCG
a-------------------   4   0,0
--UAACAGUCUACAGCCAUGGUCG
u-------------------   6   0,0
---AACAGUCUACAGCCAUGGUCG
u-------------------   2   1,0
--ACAGUCUACAGCCAUGGU--
--------------------   2   2,-2
--ACAGUCUACAGCCAUGGUCG
uu------------------   2   2,0

>hsa-mir-133a-1 3p:53-74   SRX018959
acaaugcuuugcuagagcugguaaaauggaaccaaaucgccucuucaauggaUUUGGUCCCCUUCAACCAGCUGuagcua
ugcauuga
.((((((...((((.(((((((..((.((.((((((((............)))))))).)).))..))))))).))))..
.)))))).
---UUGGUCCCCUUCAACCAGCUGU-----
--------   2   1,1

>hsa-mir-133a-2 3p:59-80   SRX018959
gggagccaaaugcuuugcuagagcugguaaaauggaaccaaaucgacuguccaauggaUUUGGUCCCCUUCAACCAGCUG
uagcugugcauugauggcgccg
.((.((((((((...((((.(((((((..((.((.((((((((.(........).)))))))).)).))..))))))).

))))...)))))..)))).)).
---UUGGUCCCCUUCAACCAGCUG
U-------------------- 2   1,1

>hsa-mir-134   5p:8-29 SRX018959
cagggugUGUGACUGGUUGACCAGAGGGGcaugcacuguguucacccugugggccaccuagucaccaacccuc
.(((((..((((((((.((.(((.((((((((.....))))...)))).))).)).))))))))..))))).
-------UGUGACUGGUUGACCAGAGGG--   4
    0,-1
-------UGUGACUGGUUGACCAGAGGGG---   6
    0,0
-------UGUGACUGGUUGACCAGAGGGGau---   2
    0,0

>hsa-mir-135a-2 5p:23-45   SRX018959
agauaaauucacucuagugcuuUAUGGCUUUUUAUUCCUAUGUGAuaguaauaaagucucauguagggauggaagccaug
aaauacauugugaaaaauca
.(((...((((....(((.(((((((((((.(((((((((((.(.(.....).).)))))))))))))))))))))
)).)))...)))))..))).
---UGUAGGGAUGGAAGCCAUG
AAA----------------   2

>hsa-mir-136   MA:15-37;mi:49-70   SRX018959
ugagcccucggaggACUCCAUUUGUUUUGAUGAUGGAuucuuaugcucCAUCAUCGUCUCAAAUGAGUCUucagaggguu
cu
.(((((((.((((((((.(((((...(((((((((.........)))))))))...))))))))))))).)))))))
).
--------------ACUCCAUUUGUUUUGAUG---
-- 3   0,-5
--------------ACUCCAUUUGUUUUGAUGA--
-- 19 0,-4
--------------ACUCCAUUUGUUUUGAUGAU---
-- 47 0,-3
--------------ACUCCAUUUGUUUUGAUGAUGGA--
-- 6   0,0
--------------ACUCCAUUUGUUUUGAUGAUGGAa---
-- 3   0,0
---------------CUCCAUUUGUUUUGAUGA--

-- 3   1,-4
---CAUCAUCGUCUCAAAUGA---------------
-- 15 0,-4
---CAUCAUCGUCUCAAAUGAG--------------
-- 3   0,-3
---CAUCAUCGUCUCAAAUGAGU-------------
-- 2   0,-2
---CAUCAUCGUCUCAAAUGAGUC------------
-- 5   0,-1
---CAUCAUCGUCUCAAAUGAGUCU-----------
-- 3   0,0
--AUCAUCGUCUCAAAUGAGU-------------
-- 2   1,-2

>hsa-mir-138-1 MA:23-45;mi:63-84   SRX018959
cccuggcauggugugguggggcAGCUGGUGUUGUGAAUCAGGCCGuugccaaucagagaacgGCUACUUCACAACACCAG
GGCCacaccacacuacagg
.((((...((((((((((.(((..(((((((((((((...(((((((..(.....)..)))))))..)))))))))))))
.))).))))))))))))))
----------------------AGCUGGUGUUGUGAAUCAGGCCGU----------------------------------
------------------- 3   0,1

>hsa-mir-139   5p:7-28;3p:44-65   SRX018959
guguauUCUACAGUGCACGUGUCUCCAGuguggcucggaggcuGGAGACGCGGCCCUGUUGGAGUaac
...(((((((((.((.(((((((((((((.(.......).))))))))))))).))).))))))))..
------UCUACAGUGCACGUGUCU--   8   0,-4
------UCUACAGUGCACGUGUCUC---   27 0,-3
------UCUACAGUGCACGUGUCUCa--   2   0,-3
------UCUACAGUGCACGUGUCUCC--   40 0,-2
------UCUACAGUGCACGUGUCUCCA---   152 0,-1
------UCUACAGUGCACGUGUCUCCAa--   9   0,-1
------UCUACAGUGCACGUGUCUCCAu--   17 0,-1
------UCUACAGUGCACGUGUCUCCAuu---------------------------------------   5   0,-1
------UCUACAGUGCACGUGUCUCCAG--   130 0,0
------UCUACAGUGCACGUGUCUCCAGa---------------------------------------   20 0,0
------UCUACAGUGCACGUGUCUCCAGU---------------------------------------   301 0,1
------UCUACAGUGCACGUGUCUCCAGUa--------------------------------------   4   0,1
------UCUACAGUGCACGUGUCUCCAGUu--------------------------------------   17 0,1

--UGGAGACGCGGCCCUGUU--------   9
-1,-5
--UGGAGACGCGGCCCUGUUG-------   20
-1,-4
--UGGAGACGCGGCCCUGUUGG------   21
-1,-3
--UGGAGACGCGGCCCUGUUGGA-----   22
-1,-2
--UGGAGACGCGGCCCUGUUGGAa----   5
-1,-2
--UGGAGACGCGGCCCUGUUGGAaa---   6
-1,-2
--UGGAGACGCGGCCCUGUUGGAG----   63
-1,-1
--UGGAGACGCGGCCCUGUUGGAGa---   10
-1,-1
--UGGAGACGCGGCCCUGUUGGAGU---   116 -1,0
--UGGAGACGCGGCCCUGUUGGAGUu--   3   -1,0
--UGGAGACGCGGCCCUGUUGGAGUuu-   2   -1,0
---GGAGACGCGGCCCUGUUGG------   4   0,-3
---GGAGACGCGGCCCUGUUGGAG----   9   0,-1
---GGAGACGCGGCCCUGUUGGAGa---   6   0,-1
---GGAGACGCGGCCCUGUUGGAGU---   33   0,0
---GGAGACGCGGCCCUGUUGGAGUAu-   2   0,1
---AGACGCGGCCCUGUUGGAGU---   2   2,0
---AGACGCGGCCCUGUUGGAGUu--   7   2,0
---AGACGCGGCCCUGUUGGAGUuu-   4   2,0
---AGACGCGGCCCUGUUGGAGUAu-   2   2,1

>hsa-mir-140   5p:23-44;3p:62-82   SRX018959
ugugucucucucuguguccugcCAGUGGUUUUACCCUAUGGUAGguuacgucaugcuguucUACCACAGGGUAGAACCAC
GGacaggauaccggggcacc
.((((((.....(((((((((.((((((((((((.(((((((..(((......)))))))))).))))))))))))
)).)))))))).))))))).
----------------------CAGUGGUUUUACCCUAUGGUAG--
--------------------   4   0,0
--CUACCACAGGGUAGAACCAC
G-------------------   3   -1,-1

--CUACCACAGGGUAGAACCAC
GG------------------ 3 -1,0
--CUACCACAGGGUAGAACCAC
GGA----------------- 4 -1,1
--CUACCACAGGGUAGAACCAC
GGAa---------------- 2 -1,1
--CUACCACAGGGUAGAACCAC
GGAC---------------- 2 -1,2
---UACCACAGGGUAGAACCA-
-------------------- 48 0,-3
---UACCACAGGGUAGAACCAC
-------------------- 104 0,-2
---UACCACAGGGUAGAACCAC
G------------------- 1135 0,-1
---UACCACAGGGUAGAACCAC
Ga------------------ 5 0,-1
---UACCACAGGGUAGAACCAC
Gu------------------ 5 0,-1
---UACCACAGGGUAGAACCAC
GG------------------ 2456 0,0
---UACCACAGGGUAGAACCAC
GGu----------------- 55 0,0
---UACCACAGGGUAGAACCAC
GGuu---------------- 12 0,0
---UACCACAGGGUAGAACCAC
GGA----------------- 2315 0,1
---UACCACAGGGUAGAACCAC
GGAa---------------- 689 0,1
---UACCACAGGGUAGAACCAC
GGAaa--------------- 474 0,1
---UACCACAGGGUAGAACCAC
GGAau--------------- 146 0,1
---UACCACAGGGUAGAACCAC
GGAu---------------- 131 0,1
---UACCACAGGGUAGAACCAC
GGAuu--------------- 6 0,1
---UACCACAGGGUAGAACCAC
GGAC---------------- 917 0,2

```
-------------------------------------------------------------UACCACAGGGUAGAACCAC
GGACu---------------	11	0,2
-------------------------------------------------------------UACCACAGGGUAGAACCAC
GGACA---------------	90	0,3
-------------------------------------------------------------UACCACAGGGUAGAACCAC
GGACAa--------------	7	0,3
-------------------------------------------------------------UACCACAGGGUAGAACCAC
GGACAu--------------	4	0,3
--------------------------------------------------------------ACCACAGGGUAGAACCAC
--------------------	48	1,-2
--------------------------------------------------------------ACCACAGGGUAGAACCAC
G-------------------	52	1,-1
--------------------------------------------------------------ACCACAGGGUAGAACCAC
GG------------------	1301	1,0
--------------------------------------------------------------ACCACAGGGUAGAACCAC
GGu-----------------	57	1,0
--------------------------------------------------------------ACCACAGGGUAGAACCAC
GGuu----------------	31	1,0
--------------------------------------------------------------ACCACAGGGUAGAACCAC
GGA-----------------	10290	1,1
--------------------------------------------------------------ACCACAGGGUAGAACCAC
GGAa----------------	3262	1,1
--------------------------------------------------------------ACCACAGGGUAGAACCAC
GGAaa---------------	1072	1,1
--------------------------------------------------------------ACCACAGGGUAGAACCAC
GGAau---------------	266	1,1
--------------------------------------------------------------ACCACAGGGUAGAACCAC
GGAu----------------	1529	1,1
--------------------------------------------------------------ACCACAGGGUAGAACCAC
GGAuu---------------	80	1,1
--------------------------------------------------------------ACCACAGGGUAGAACCAC
GGAC----------------	2834	1,2
--------------------------------------------------------------ACCACAGGGUAGAACCAC
GGACu---------------	69	1,2
--------------------------------------------------------------ACCACAGGGUAGAACCAC
GGACuu--------------	3	1,2
--------------------------------------------------------------ACCACAGGGUAGAACCAC
GGACA---------------	178	1,3
```

--ACCACAGGGUAGAACCAC

GGACAa--------------   159 1,3

--ACCACAGGGUAGAACCAC

GGACAaa-------------   13 1,3

--ACCACAGGGUAGAACCAC

GGACAau-------------   2   1,3

--ACCACAGGGUAGAACCAC

GGACAu--------------   22 1,3

--ACCACAGGGUAGAACCAC

GGACAG--------------   8   1,4

---CCACAGGGUAGAACCAC

GG------------------   28 2,0

---CCACAGGGUAGAACCAC

GGuu----------------   2   2,0

---CCACAGGGUAGAACCAC

GGA-----------------   184 2,1

---CCACAGGGUAGAACCAC

GGAa----------------   78 2,1

---CCACAGGGUAGAACCAC

GGAaa---------------   35 2,1

---CCACAGGGUAGAACCAC

GGAau---------------   8   2,1

---CCACAGGGUAGAACCAC

GGAu----------------   29 2,1

---CCACAGGGUAGAACCAC

GGAuu---------------   3   2,1

---CCACAGGGUAGAACCAC

GGAC----------------   68 2,2

---CCACAGGGUAGAACCAC

GGACu---------------   3   2,2

---CCACAGGGUAGAACCAC

GGACA---------------   3   2,3

---CCACAGGGUAGAACCAC

GGACAa--------------   8   2,3

---CCACAGGGUAGAACCAC

GGACAaa-------------   2   2,3

>hsa-mir-141   mi:17-38;MA:59-80   SRX018959

cggccggcccuggguc CAUCUUCCAGUACAGUGUUGGAuggucuaauugugaagcuccUAACACUGUCUGGUAAAGAUGG
cucccggguggguuc
.((((.((((.(((.(((((((((((.(((((((((((...((.......))...))).))))))))))))..)))))))
..))))))).)))).
--UAACACUGUCUGGUAAAGAUG-
--------------- 2 0,-1
--UAACACUGUCUGGUAAAGAUGu
u-------------- 2 0,-1

>hsa-mir-142 5p:16-36;3p:52-74 SRX018959
gacagugcagucaccCAUAAAGUAGAAAGCACUACUaacagcacuggagggUGUAGUGUUUCCUACUUUAUGGAugagug
uacugug
.(((((((.(((.(((((((((((.((((((((((...(..(....)..)..))))))))).))))))))))).))).))
)))))).
-------------CCCAUAAAGUAGAAAGCA---
------- 24 -2,-5
-------------CCCAUAAAGUAGAAAGCAa--
------- 4 -2,-5
-------------CCCAUAAAGUAGAAAGCAuu---
------- 2 -2,-5
-------------CCCAUAAAGUAGAAAGCAC--
------- 34 -2,-4
-------------CCCAUAAAGUAGAAAGCACU---
------- 268 -2,-3
-------------CCCAUAAAGUAGAAAGCACUA--
------- 146 -2,-2
-------------CCCAUAAAGUAGAAAGCACUAa---
------- 36 -2,-2
-------------CCCAUAAAGUAGAAAGCACUAaa--
------- 5 -2,-2
-------------CCCAUAAAGUAGAAAGCACUAu---
------- 13 -2,-2
-------------CCCAUAAAGUAGAAAGCACUAC---
------- 42 -2,-1
-------------CCCAUAAAGUAGAAAGCACUACa--
------- 2 -2,-1
-------------CCCAUAAAGUAGAAAGCACUACU--
------- 2 -2,0

--------------CCAUAAAGUAGAAAGCACU--
------- 3   -1,-3
--------------CCAUAAAGUAGAAAGCACUA---
------- 3   -1,-2
--------------CCAUAAAGUAGAAAGCACUAa--
------- 3   -1,-2
--------------CCAUAAAGUAGAAAGCACUAC--
------- 2   -1,-1
---------------CAUAAAGUAGAAAGCACU--
------- 3   0,-3
---------------CAUAAAGUAGAAAGCACUA---
------- 77   0,-2
---------------CAUAAAGUAGAAAGCACUAa--
------- 36   0,-2
---------------CAUAAAGUAGAAAGCACUAaa---
------- 17   0,-2
---------------CAUAAAGUAGAAAGCACUAu--
------- 6   0,-2
---------------CAUAAAGUAGAAAGCACUAuu---
------- 5   0,-2
---------------CAUAAAGUAGAAAGCACUAC--
------- 73   0,-1
---------------CAUAAAGUAGAAAGCACUACU---
------- 84   0,0
---------------CAUAAAGUAGAAAGCACUACUA--
------- 66   0,1
---------------CAUAAAGUAGAAAGCACUACUAu---
------- 8   0,1
---------------CAUAAAGUAGAAAGCACUACUAA---
------- 17   0,2
---------------CAUAAAGUAGAAAGCACUACUAAa--
------- 3   0,2
----------------AUAAAGUAGAAAGCACUAa--
------- 2   1,-2
----------------AUAAAGUAGAAAGCACUAC--
------- 4   1,-1
----------------AUAAAGUAGAAAGCACUACU---
------- 12   1,0

```
----------------AUAAAGUAGAAAGCACUACUA-------------------------------------------
------- 12  1,1
----------------AUAAAGUAGAAAGCACUACUAu------------------------------------------
------- 12  1,1
----------------AUAAAGUAGAAAGCACUACUAA------------------------------------------
------- 158 1,2
----------------AUAAAGUAGAAAGCACUACUAAa-----------------------------------------
------- 2   1,2
----------------AUAAAGUAGAAAGCACUACUAAu-----------------------------------------
------- 2   1,2
---------------------------------------------------UGUAGUGUUUCCUACUUUAUG--------
------- 6   0,-2
---------------------------------------------------UGUAGUGUUUCCUACUUUAUGG-------
------- 4   0,-1
----------------------------------------------------GUAGUGUUUCCUACUUUAUG--------
------- 2   1,-2
----------------------------------------------------GUAGUGUUUCCUACUUUAUGG-------
------- 31  1,-1
----------------------------------------------------GUAGUGUUUCCUACUUUAUGGA------
------- 5   1,0

>hsa-mir-143    mi:27-48;MA:61-81   SRX018959
gcgcagcgcccugucucccagccugaGGUGCAGUGCUGCAUCUCUGGUcaguugggagucUGAGAUGAAGCACUGUAGCU
Caggaagagagaaguuguucugcagc
..((((.((....((((.(..((((((.(((((((((.((((((.((((......)).)).)))))).))))))))).))
))))..).))))....)).))))...
-------------------------AGGUGCAGUGCUGCAUCUCU-----------------------------------
--------------------------  2   -1,-3
--------------------------GGUGCAGUGCUGCAUCUC------------------------------------
--------------------------  126 0,-4
--------------------------GGUGCAGUGCUGCAUCUCU-----------------------------------
--------------------------  1038    0,-3
--------------------------GGUGCAGUGCUGCAUCUCUG----------------------------------
--------------------------  23  0,-2
--------------------------GGUGCAGUGCUGCAUCUCUGaa--------------------------------
--------------------------  2   0,-2
--------------------------GGUGCAGUGCUGCAUCUCUGG---------------------------------
--------------------------  13  0,-1
```

-------------------------GGUGCAGUGCUGCAUCUCUGGa-------------------------------
------------------------- 13 0,-1
-------------------------GGUGCAGUGCUGCAUCUCUGGaa------------------------------
------------------------- 14 0,-1
-------------------------GGUGCAGUGCUGCAUCUCUGGU-------------------------------
------------------------- 4 0,0
-------------------------GGUGCAGUGCUGCAUCUCUGGUa------------------------------
------------------------- 7 0,0
-------------------------GGUGCAGUGCUGCAUCUCUGGUaa-----------------------------
------------------------- 2 0,0
-------------------------GGUGCAGUGCUGCAUCUCUGGUau-----------------------------
------------------------- 8 0,0
-------------------------GGUGCAGUGCUGCAUCUCUGGUC------------------------------
------------------------- 2 0,1
-------------------------GGUGCAGUGCUGCAUCUCUGGUCA-----------------------------
------------------------- 4 0,2
--UCUGAGAUGAAGCACUGUAG--
------------------------- 2 -2,-3
--UCUGAGAUGAAGCACUGUAGCa
u------------------------ 2 -2,-2
--UCUGAGAUGAAGCACUGUAGCU
------------------------- 13 -2,-1
--UCUGAGAUGAAGCACUGUAGCU
C------------------------ 3 -2,0
---CUGAGAUGAAGCACUGUA---
------------------------- 3 -1,-4
---CUGAGAUGAAGCACUGUAaa-
------------------------- 3 -1,-4
---CUGAGAUGAAGCACUGUAGC-
------------------------- 3 -1,-2
---CUGAGAUGAAGCACUGUAGCU
------------------------- 40 -1,-1
---CUGAGAUGAAGCACUGUAGCU
a------------------------ 5 -1,-1
---CUGAGAUGAAGCACUGUAGCU
C------------------------ 34 -1,0
---CUGAGAUGAAGCACUGUAGCU
Cu----------------------- 9 -1,0

--CUGAGAUGAAGCACUGUAGCU
CA------------------------ 8 -1,1
---UGAGAUGAAGCACUGUAG--
-------------------------- 222 0,-3
---UGAGAUGAAGCACUGUAGaa
-------------------------- 2 0,-3
---UGAGAUGAAGCACUGUAGau
-------------------------- 3 0,-3
---UGAGAUGAAGCACUGUAGuu
-------------------------- 2 0,-3
---UGAGAUGAAGCACUGUAGC-
-------------------------- 191 0,-2
---UGAGAUGAAGCACUGUAGCa
-------------------------- 29 0,-2
---UGAGAUGAAGCACUGUAGCa
a------------------------- 40 0,-2
---UGAGAUGAAGCACUGUAGCa
u------------------------- 3 0,-2
---UGAGAUGAAGCACUGUAGCU
-------------------------- 2544 0,-1
---UGAGAUGAAGCACUGUAGCU
a------------------------- 160 0,-1
---UGAGAUGAAGCACUGUAGCU
aa------------------------ 51 0,-1
---UGAGAUGAAGCACUGUAGCU
au------------------------ 22 0,-1
---UGAGAUGAAGCACUGUAGCU
u------------------------- 63 0,-1
---UGAGAUGAAGCACUGUAGCU
uu------------------------ 11 0,-1
---UGAGAUGAAGCACUGUAGCU
C------------------------- 4387 0,0
---UGAGAUGAAGCACUGUAGCU
Cu------------------------ 1223 0,0
---UGAGAUGAAGCACUGUAGCU
Cuu----------------------- 163 0,0
---UGAGAUGAAGCACUGUAGCU
CA------------------------ 274 0,1

---UGAGAUGAAGCACUGUAGCU
CAa------------------------ 34 0,1
---UGAGAUGAAGCACUGUAGCU
CAaa----------------------- 6 0,1
---UGAGAUGAAGCACUGUAGCU
CAu------------------------ 4 0,1
---UGAGAUGAAGCACUGUAGCU
CAG------------------------ 2 0,2
--GAGAUGAAGCACUGUAGC-
--------------------------- 3 1,-2
--GAGAUGAAGCACUGUAGCU
--------------------------- 7 1,-1
--GAGAUGAAGCACUGUAGCU
C-------------------------- 52 1,0
--GAGAUGAAGCACUGUAGCU
Cu------------------------- 11 1,0
--GAGAUGAAGCACUGUAGCU
Cuu------------------------ 3 1,0
---AGAUGAAGCACUGUAGCU
--------------------------- 2 2,-1
---AGAUGAAGCACUGUAGCU
C-------------------------- 3 2,0
---AGAUGAAGCACUGUAGCU
Cu------------------------- 2 2,0

>hsa-mir-144 mi:15-36;MA:52-71 SRX018959
ugggcccuggcugGGAUAUCAUCAUAUACUGUAAGuuugcgaugagacacUACAGUAUAGAUGAUGUACUaguccgggc
accccc
.(((((((.((((((.((((((((.(((((((((.((((......))))..)))))))))))))))))).))))))).))))
.)))..
-------------GGAUAUCAUCAUAUACUG--
------ 5 0,-4
-------------GGAUAUCAUCAUAUACUGUAAG--
------ 23 0,0
-------------GGAUAUCAUCAUAUACUGUAAGU---
------ 12 0,1
---CUACAGUAUAGAUGAUGUA-----------
------ 2 -1,-2

---UACAGUAUAGAUGAUGUA-----------
------ 6 0,-2
---UACAGUAUAGAUGAUGUAC----------
------ 5 0,-1
---UACAGUAUAGAUGAUGUACU---------
------ 3 0,0
---UACAGUAUAGAUGAUGUACUu--------
------ 2 0,0
---UACAGUAUAGAUGAUGUACUuu-------
------ 3 0,0
---UACAGUAUAGAUGAUGUACUAa-------
------ 3 0,1

>hsa-mir-145 MA:16-38;mi:54-75 SRX018959
caccuuguccucacgGUCCAGUUUUCCCAGGAAUCCCUuagaugcuaagauggGGAUUCCUGGAAAUACUGUUCUugagg
ucaugguu
.(((.((.(((((.((..((((.((.((((((((((((.............))))))))))))).)).))))..)))))))
.)).))).
---------------GUCCAGUUUUCCCAGGAA---
-------- 5 0,-5
---------------GUCCAGUUUUCCCAGGAAU--
-------- 12 0,-4
---------------GUCCAGUUUUCCCAGGAAUa---
-------- 26 0,-4
---------------GUCCAGUUUUCCCAGGAAUaa--
-------- 3 0,-4
---------------GUCCAGUUUUCCCAGGAAUu---
-------- 4 0,-4
---------------GUCCAGUUUUCCCAGGAAUC---
-------- 12 0,-3
---------------GUCCAGUUUUCCCAGGAAUCa--
-------- 23 0,-3
---------------GUCCAGUUUUCCCAGGAAUCaa---
-------- 4 0,-3
---------------GUCCAGUUUUCCCAGGAAUCC--
-------- 30 0,-2
---------------GUCCAGUUUUCCCAGGAAUCCa---
-------- 4 0,-2

---------------GUCCAGUUUUCCCAGGAAUCCC--
--------   110 0,-1
---------------GUCCAGUUUUCCCAGGAAUCCCa---
--------   17 0,-1
---------------GUCCAGUUUUCCCAGGAAUCCCU---
--------   186 0,0
---------------GUCCAGUUUUCCCAGGAAUCCCUa--
--------   3   0,0
---------------GUCCAGUUUUCCCAGGAAUCCCUU--
--------   19 0,1
--GGAUUCCUGGAAAUACUGUUC------
--------   4   0,-1
--GGAUUCCUGGAAAUACUGUUCU-----
--------   25 0,0
---GAUUCCUGGAAAUACUGUUC------
--------   3   1,-1
---GAUUCCUGGAAAUACUGUUCUU----
--------   5   1,1
--AUUCCUGGAAAUACUGUU-------
--------   3   2,-2
--AUUCCUGGAAAUACUGUUC------
--------   6   2,-1
--AUUCCUGGAAAUACUGUUCU-----
--------   60 2,0
--AUUCCUGGAAAUACUGUUCUa----
--------   14 2,0
--AUUCCUGGAAAUACUGUUCUaa---
--------   5   2,0
--AUUCCUGGAAAUACUGUUCUU----
--------   45 2,1
--AUUCCUGGAAAUACUGUUCUUa---
--------   5   2,1
--AUUCCUGGAAAUACUGUUCUUu---
--------   2   2,1

>hsa-mir-1468   5p:13-33   SRX018959
ggugggugguuuCUCCGUUUGCCUGUUUCGCUGaugugcauucaacucauucucagcaaaauaagcaaauggaaaauucg
uccauc

((((..(..((.((((((((.(((((.(((((.(((.........)))..))))).))))).))))))))).))..).
.)))))
------------CUCCGUUUGCCUGUUUCGC---
------ 2 0,-2
------------CUCCGUUUGCCUGUUUCGCU--
------ 12 0,-1
------------CUCCGUUUGCCUGUUUCGCUGA--
------ 3 0,1
------------CUCCGUUUGCCUGUUUCGCUGAa---
------ 3 0,1

>hsa-mir-146a MA:21-42;mi:57-78 SRX018959
ccgauguguauccucagcuuUGAGAACUGAAUUCCAUGGGUUgugucagugucagaCCUCUGAAAUUCAGUUCUUCAGcu
gggauaucucugucaucgu
.(((((.((((((.(((((..((((((((((((.((.(((((.((.......))))))).)).)))))))))))).))))
)))))))......))))).
-------------------UUGAGAACUGAAUUCCAU--
------------------- 2 -1,-5
-------------------UUGAGAACUGAAUUCCAUGGGUau--------------------------------------
------------------- 2 -1,-1
-------------------UUGAGAACUGAAUUCCAUGGGUU---------------------------------------
------------------- 2 -1,0
--------------------UGAGAACUGAAUUCCAUGG--
------------------- 12 0,-3
--------------------UGAGAACUGAAUUCCAUGGG---
------------------- 13 0,-2
--------------------UGAGAACUGAAUUCCAUGGGU--
------------------- 31 0,-1
--------------------UGAGAACUGAAUUCCAUGGGUa---------------------------------------
------------------- 9 0,-1
--------------------UGAGAACUGAAUUCCAUGGGUaa--------------------------------------
------------------- 16 0,-1
--------------------UGAGAACUGAAUUCCAUGGGUau--------------------------------------
------------------- 39 0,-1
--------------------UGAGAACUGAAUUCCAUGGGUU---------------------------------------
------------------- 208 0,0
--------------------UGAGAACUGAAUUCCAUGGGUUa--------------------------------------
------------------- 163 0,0

--------------------UGAGAACUGAAUUCCAUGGGUUaa------------------------------------
------------------- 43   0,0
--------------------UGAGAACUGAAUUCCAUGGGUUau------------------------------------
------------------- 72   0,0
--------------------UGAGAACUGAAUUCCAUGGGUUu-------------------------------------
------------------- 59   0,0
--------------------UGAGAACUGAAUUCCAUGGGUUuu------------------------------------
------------------- 11   0,0
--------------------UGAGAACUGAAUUCCAUGGGUUG-------------------------------------
------------------- 40   0,1
--------------------UGAGAACUGAAUUCCAUGGGUUGa------------------------------------
------------------- 22   0,1
--------------------UGAGAACUGAAUUCCAUGGGUUGU------------------------------------
------------------- 23   0,2
--------------------UGAGAACUGAAUUCCAUGGGUUGUa-----------------------------------
------------------- 6   0,2
---------------------GAGAACUGAAUUCCAUGGGUU--------------------------------------
------------------- 9   1,0
---------------------GAGAACUGAAUUCCAUGGGUUa-------------------------------------
------------------- 3   1,0
---------------------GAGAACUGAAUUCCAUGGGUUaa------------------------------------
------------------- 3   1,0
----------------------AGAACUGAAUUCCAUGGGUU--------------------------------------
------------------- 3   2,0
----------------------AGAACUGAAUUCCAUGGGUUG-------------------------------------
------------------- 2   2,1
---ACCUCUGAAAUUCAGUUCUUC----
------------------- 3   -1,-2

>hsa-mir-146b   5p:9-30;3p:45-66   SRX018959
ccuggcacUGAGAACUGAAUUCCAUAGGCUgugagcucuagcaaUGCCCUGUGGACUCAGUUCUGGugcccgg
((.((((((.((((((((..((((((((..((..((....)).))..)))))))).))))))))))))))).))
-------CUGAGAACUGAAUUCCAUAG--   2
    -1,-3
-------CUGAGAACUGAAUUCCAUAGG---   8
    -1,-2
-------CUGAGAACUGAAUUCCAUAGGC--   7
    -1,-1

-------CUGAGAACUGAAUUCCAUAGGCU--   83
-1,0
-------CUGAGAACUGAAUUCCAUAGGCUu---   3
-1,0
--------UGAGAACUGAAUUCCAUA--   5
0,-4
--------UGAGAACUGAAUUCCAUAG---   28
0,-3
--------UGAGAACUGAAUUCCAUAGG--   163
0,-2
--------UGAGAACUGAAUUCCAUAGGau--   4
0,-2
--------UGAGAACUGAAUUCCAUAGGuu--   3
0,-2
--------UGAGAACUGAAUUCCAUAGGC---   72
0,-1
--------UGAGAACUGAAUUCCAUAGGCa--   88
0,-1
--------UGAGAACUGAAUUCCAUAGGCaa---   41
0,-1
--------UGAGAACUGAAUUCCAUAGGCau---   41
0,-1
--------UGAGAACUGAAUUCCAUAGGCU--   1805
0,0
--------UGAGAACUGAAUUCCAUAGGCUa---   8
0,0
--------UGAGAACUGAAUUCCAUAGGCUaa--   30
0,0
--------UGAGAACUGAAUUCCAUAGGCUau--   68
0,0
--------UGAGAACUGAAUUCCAUAGGCUu---   216
0,0
--------UGAGAACUGAAUUCCAUAGGCUuu--   72
0,0
--------UGAGAACUGAAUUCCAUAGGCUG---   54
0,1
--------UGAGAACUGAAUUCCAUAGGCUGa--   429
0,1

--------UGAGAACUGAAUUCCAUAGGCUGaa-- 17
0,1
--------UGAGAACUGAAUUCCAUAGGCUGau-- 12
0,1
--------UGAGAACUGAAUUCCAUAGGCUGU--- 12
0,2
---------GAGAACUGAAUUCCAUAGG--- 4
1,-2
---------GAGAACUGAAUUCCAUAGGCU--- 23
1,0
---------GAGAACUGAAUUCCAUAGGCUaa--- 3
1,0
---------GAGAACUGAAUUCCAUAGGCUu-- 6
1,0
---------GAGAACUGAAUUCCAUAGGCUuu--- 3
1,0
---------GAGAACUGAAUUCCAUAGGCUG-- 6
1,1
---------GAGAACUGAAUUCCAUAGGCUGa--- 11
1,1
----------AGAACUGAAUUCCAUAGGCU--- 3
2,0
--UGCCCUGUGGACUCAGUUCUGGUau---- 4
0,1
---GCCCUGUGGACUCAGUUCUG-------- 2
1,-1
---GCCCUGUGGACUCAGUUCUGG------- 12
1,0
---GCCCUGUGGACUCAGUUCUGGU------ 20
1,1
---GCCCUGUGGACUCAGUUCUGGUa----- 21
1,1
---GCCCUGUGGACUCAGUUCUGGUaa---- 9
1,1
---GCCCUGUGGACUCAGUUCUGGUau---- 57
1,1
---GCCCUGUGGACUCAGUUCUGGUu----- 13
1,1

--CCCUGUGGACUCAGUUCUGGUau----   11
2,1

>hsa-mir-148a   mi:6-27;MA:44-65   SRX018959
gaggcAAAGUUCUGAGACACUCCGACUcugaguaugauagaagUCAGUGCACUACAGAACUUUGUcuc
((((((((((((((((.((((..(((((((.......))).))))))))..)).))))))))))))))
----CAAAGUUCUGAGACACUCCGA---   9
-1,-2
-----AAAGUUCUGAGACACUCC---   5   0,-4
-----AAAGUUCUGAGACACUCCGA---   53   0,-2
-----AAAGUUCUGAGACACUCCGAa--   4   0,-2
-----AAAGUUCUGAGACACUCCGAaa---   2   0,-2
-----AAAGUUCUGAGACACUCCGAC--   3   0,-1
-----AAAGUUCUGAGACACUCCGACa---   5   0,-1
-----AAAGUUCUGAGACACUCCGACU---   15   0,0
-----AAAGUUCUGAGACACUCCGACUa--   3   0,0
------AAGUUCUGAGACACUCCGACU---   3   1,0
--GUCAGUGCACUACAGAACUUUGa---   2
-1,-1
--GUCAGUGCACUACAGAACUUUGU---   3   -1,0
---UCAGUGCACUACAGAACU-------   9673
0,-4
---UCAGUGCACUACAGAACUa------   5   0,-4
---UCAGUGCACUACAGAACUaa-----   9   0,-4
---UCAGUGCACUACAGAACUau-----   2   0,-4
---UCAGUGCACUACAGAACUU------   498   0,-3
---UCAGUGCACUACAGAACUUa-----   9   0,-3
---UCAGUGCACUACAGAACUUU-----   2518
0,-2
---UCAGUGCACUACAGAACUUUa----   45   0,-2
---UCAGUGCACUACAGAACUUUaa---   9   0,-2
---UCAGUGCACUACAGAACUUUau---   5   0,-2
---UCAGUGCACUACAGAACUUUu----   37   0,-2
---UCAGUGCACUACAGAACUUUuu---   15   0,-2
---UCAGUGCACUACAGAACUUUG----   3646
0,-1
---UCAGUGCACUACAGAACUUUGa---   3055
0,-1

---UCAGUGCACUACAGAACUUUGaa--   261 0,-1
---UCAGUGCACUACAGAACUUUGU---   32418
    0,0
---UCAGUGCACUACAGAACUUUGUa--   219 0,0
---UCAGUGCACUACAGAACUUUGUaa-   49   0,0
---UCAGUGCACUACAGAACUUUGUau-   15   0,0
---UCAGUGCACUACAGAACUUUGUu--   313 0,0
---UCAGUGCACUACAGAACUUUGUuu-   15   0,0
---UCAGUGCACUACAGAACUUUGUC--   521 0,1
---UCAGUGCACUACAGAACUUUGUCa-   44   0,1
---UCAGUGCACUACAGAACUUUGUCaa   3   0,1
---UCAGUGCACUACAGAACUUUGUCU-   96   0,2
---UCAGUGCACUACAGAACUUUGUCUa   2   0,2
---UCAGUGCACUACAGAACUUUGUCUu   3   0,2
--CAGUGCACUACAGAACUUU-----   4   1,-2
--CAGUGCACUACAGAACUUUG----   2   1,-1
--CAGUGCACUACAGAACUUUGa---   3   1,-1
--CAGUGCACUACAGAACUUUGU---   29   1,0
---AGUGCACUACAGAACUUU-----   78   2,-2
---AGUGCACUACAGAACUUUa----   2   2,-2
---AGUGCACUACAGAACUUUu----   3   2,-2
---AGUGCACUACAGAACUUUG----   48   2,-1
---AGUGCACUACAGAACUUUGa---   19   2,-1
---AGUGCACUACAGAACUUUGaa--   5   2,-1
---AGUGCACUACAGAACUUUGU---   486 2,0
---AGUGCACUACAGAACUUUGUa--   7   2,0
---AGUGCACUACAGAACUUUGUaa-   2   2,0
---AGUGCACUACAGAACUUUGUu--   11   2,0
---AGUGCACUACAGAACUUUGUC--   15   2,1
---AGUGCACUACAGAACUUUGUCa-   2   2,1
---AGUGCACUACAGAACUUUGUCU-   3   2,2

>hsa-mir-148b   mi:25-46;MA:63-84   SRX018959
caagcacgauuagcauuugaggugAAGUUCUGUUAUACACUCAGGCuguggcucucugaaagUCAGUGCAUCACAGAACU
UUGUcucgaaagcuuucua
.......((..(((.(((((((..(((((((((.((.((((..((((....(.....)..)))))))).)).))))))))
)..))))))).))).))..
-----------------------GAAGUUCUGUUAUACACU--

------------------ 4   -1,-5

-----------------------GAAGUUCUGUUAUACACUC--------------------------------------

------------------ 6   -1,-4

-----------------------GAAGUUCUGUUAUACACUCAG------------------------------------

------------------ 10   -1,-2

-----------------------GAAGUUCUGUUAUACACUCAGG-----------------------------------

------------------ 9   -1,-1

-----------------------GAAGUUCUGUUAUACACUCAGGCau--------------------------------

------------------ 2   -1,0

-----------------------GAAGUUCUGUUAUACACUCAGGCU---------------------------------

------------------ 25   -1,1

-----------------------GAAGUUCUGUUAUACACUCAGGCUa--------------------------------

------------------ 2   -1,1

-----------------------GAAGUUCUGUUAUACACUCAGGCUu--------------------------------

------------------ 4   -1,1

--UCAGUGCAUCACAGAACU

------------------ 1877   0,-4

--UCAGUGCAUCACAGAACU

U----------------- 83   0,-3

--UCAGUGCAUCACAGAACU

Ua---------------- 3   0,-3

--UCAGUGCAUCACAGAACU

UU---------------- 491 0,-2

--UCAGUGCAUCACAGAACU

UUa--------------- 2   0,-2

--UCAGUGCAUCACAGAACU

UUau-------------- 2   0,-2

--UCAGUGCAUCACAGAACU

UUu--------------- 4   0,-2

--UCAGUGCAUCACAGAACU

UUuu-------------- 2   0,-2

--UCAGUGCAUCACAGAACU

UUG--------------- 106 0,-1

--UCAGUGCAUCACAGAACU

UUGa-------------- 59   0,-1

--UCAGUGCAUCACAGAACU

UUGaa------------- 6   0,-1

--UCAGUGCAUCACAGAACU

UUGU--------------- 908 0,0
--UCAGUGCAUCACAGAACU
UUGUa-------------- 24 0,0
--UCAGUGCAUCACAGAACU
UUGUaa------------- 4   0,0
--UCAGUGCAUCACAGAACU
UUGUu-------------- 290 0,0
--UCAGUGCAUCACAGAACU
UUGUuu------------- 33 0,0
--UCAGUGCAUCACAGAACU
UUGUC-------------- 4   0,1
--UCAGUGCAUCACAGAACU
UUGUCU------------- 3   0,2
--AGUGCAUCACAGAACU
UU----------------- 6   2,-2
--AGUGCAUCACAGAACU
UUGU--------------- 12 2,0
--AGUGCAUCACAGAACU
UUGUu-------------- 6   2,0
--AGUGCAUCACAGAACU
UUGUuu------------- 2   2,0

>hsa-mir-150   MA:16-37;mi:51-72   SRX018959
cuccccauggcccugUCUCCCAACCCUUGUACCAGUGcugggcucagaccCUGGUACAGGCCUGGGGGACAGggaccugg
ggac
.((((((.((((((((((((((..(((.(((((((.((((....))).).))))))))))..))))))))))))).)))))
))).
---------------UCUCCCAACCCUUGUACC---
----   14 0,-4
---------------UCUCCCAACCCUUGUACCA--
----   26 0,-3
---------------UCUCCCAACCCUUGUACCAG---
----   8   0,-2
---------------UCUCCCAACCCUUGUACCAGU--
----   19 0,-1
---------------UCUCCCAACCCUUGUACCAGUa---
----   5   0,-1
---------------UCUCCCAACCCUUGUACCAGUaa--

---- 4 0,-1
---------------UCUCCCAACCCUUGUACCAGUG--
---- 98 0,0
---------------UCUCCCAACCCUUGUACCAGUGa---
---- 53 0,0
---------------UCUCCCAACCCUUGUACCAGUGu---
---- 13 0,0

>hsa-mir-151 5p:11-31;3p:47-67 SRX018959
uuuccugcccUCGAGGAGCUCACAGUCUAGUaugucucauccccuaCUAGACUGAAGCUCCUUGAGGacagggaugguca
uacucaccuc
.(((((.(((((((((((..(((((((((.(........).)))))))))).))))))))))).))))))......
....
---------CUCGAGGAGCUCACAGUCUAG--
--------- 7 -1,-1
---------CUCGAGGAGCUCACAGUCUAGU---
--------- 35 -1,0
---------CUCGAGGAGCUCACAGUCUAGUA--
--------- 11 -1,1
---------CUCGAGGAGCUCACAGUCUAGUAa---
--------- 2 -1,1
----------UCGAGGAGCUCACAGUCU--
--------- 2 0,-3
----------UCGAGGAGCUCACAGUCUA---
--------- 80 0,-2
----------UCGAGGAGCUCACAGUCUAa--
--------- 2 0,-2
----------UCGAGGAGCUCACAGUCUAuu---
--------- 2 0,-2
----------UCGAGGAGCUCACAGUCUAG--
--------- 75 0,-1
----------UCGAGGAGCUCACAGUCUAGa---
--------- 9 0,-1
----------UCGAGGAGCUCACAGUCUAGaa--
--------- 2 0,-1
----------UCGAGGAGCUCACAGUCUAGU---
--------- 666 0,0
----------UCGAGGAGCUCACAGUCUAGUu--

--------- 4   0,0
----------UCGAGGAGCUCACAGUCUAGUA---
--------- 339 0,1
----------UCGAGGAGCUCACAGUCUAGUAa--
--------- 91   0,1
----------UCGAGGAGCUCACAGUCUAGUAaa---
--------- 7   0,1
----------UCGAGGAGCUCACAGUCUAGUAU--
--------- 25   0,2
----------UCGAGGAGCUCACAGUCUAGUAUG---
--------- 2   0,3
-----------CGAGGAGCUCACAGUCUAGU--
--------- 2   1,0
------------GAGGAGCUCACAGUCUAGU--
--------- 24   2,0
------------GAGGAGCUCACAGUCUAGUA---
--------- 7   2,1
------------GAGGAGCUCACAGUCUAGUAa--
--------- 2   2,1
---UACUAGACUGAAGCUCCUUGA---------------
--------- 47   -2,-2
---UACUAGACUGAAGCUCCUUGAa--------------
--------- 22   -2,-2
---UACUAGACUGAAGCUCCUUGAaa-------------
--------- 4   -2,-2
---UACUAGACUGAAGCUCCUUGAu--------------
--------- 2   -2,-2
---UACUAGACUGAAGCUCCUUGAG--------------
--------- 57   -2,-1
---UACUAGACUGAAGCUCCUUGAGa-------------
--------- 26   -2,-1
---UACUAGACUGAAGCUCCUUGAGu-------------
--------- 5   -2,-1
---UACUAGACUGAAGCUCCUUGAGG-------------
--------- 47   -2,0
---UACUAGACUGAAGCUCCUUGAGGA------------
--------- 15   -2,1
---UACUAGACUGAAGCUCCUUGAGGAa-----------

--------- 2 -2,1

--ACUAGACUGAAGCUCCUUG---------------

--------- 2 -1,-3

--ACUAGACUGAAGCUCCUUGAG-------------

--------- 8 -1,-1

--ACUAGACUGAAGCUCCUUGAGa------------

--------- 2 -1,-1

--ACUAGACUGAAGCUCCUUGAGG------------

--------- 12 -1,0

--ACUAGACUGAAGCUCCUUGAGGu-----------

--------- 3 -1,0

--ACUAGACUGAAGCUCCUUGAGGA-----------

--------- 29 -1,1

--ACUAGACUGAAGCUCCUUGAGGAa----------

--------- 3 -1,1

--ACUAGACUGAAGCUCCUUGAGGAaa---------

--------- 3 -1,1

---CUAGACUGAAGCUCCUUG---------------

--------- 2 0,-3

---CUAGACUGAAGCUCCUUGA--------------

--------- 48 0,-2

---CUAGACUGAAGCUCCUUGAa-------------

--------- 17 0,-2

---CUAGACUGAAGCUCCUUGAaa------------

--------- 9 0,-2

---CUAGACUGAAGCUCCUUGAu-------------

--------- 2 0,-2

---CUAGACUGAAGCUCCUUGAG-------------

--------- 249 0,-1

---CUAGACUGAAGCUCCUUGAGa------------

--------- 67 0,-1

---CUAGACUGAAGCUCCUUGAGaa-----------

--------- 20 0,-1

---CUAGACUGAAGCUCCUUGAGau-----------

--------- 3 0,-1

---CUAGACUGAAGCUCCUUGAGu------------

--------- 22 0,-1

---CUAGACUGAAGCUCCUUGAGG------------

---------- 806 0,0
--CUAGACUGAAGCUCCUUGAGGu-------------
---------- 29 0,0
--CUAGACUGAAGCUCCUUGAGGA-------------
---------- 1592 0,1
--CUAGACUGAAGCUCCUUGAGGAa------------
---------- 1549 0,1
--CUAGACUGAAGCUCCUUGAGGAaa-----------
---------- 906 0,1
--CUAGACUGAAGCUCCUUGAGGAau-----------
---------- 206 0,1
--CUAGACUGAAGCUCCUUGAGGAu------------
---------- 199 0,1
--CUAGACUGAAGCUCCUUGAGGAuu-----------
---------- 29 0,1
--CUAGACUGAAGCUCCUUGAGGAC------------
---------- 22 0,2
--CUAGACUGAAGCUCCUUGAGGACu-----------
---------- 8 0,2
--CUAGACUGAAGCUCCUUGAGGACA-----------
---------- 4 0,3
---UAGACUGAAGCUCCUUGA----------------
---------- 2 1,-2
---UAGACUGAAGCUCCUUGAGG--------------
---------- 11 1,0
---UAGACUGAAGCUCCUUGAGGA-------------
---------- 29 1,1
---UAGACUGAAGCUCCUUGAGGAa------------
---------- 43 1,1
---UAGACUGAAGCUCCUUGAGGAaa-----------
---------- 24 1,1
---UAGACUGAAGCUCCUUGAGGAau-----------
---------- 8 1,1
---UAGACUGAAGCUCCUUGAGGAu------------
---------- 9 1,1
---UAGACUGAAGCUCCUUGAGGAC------------
---------- 6 1,2
---UAGACUGAAGCUCCUUGAGGACu-----------

---------- 3 1,2
--AGACUGAAGCUCCUUGAGGAaa----------
---------- 2 2,1
--AGACUGAAGCUCCUUGAGGAC-----------
---------- 11 2,2
--AGACUGAAGCUCCUUGAGGACu----------
---------- 5 2,2

>hsa-mir-152 3p:54-74 SRX018959
ugucccccccggcccagguucugugauacacuccgacucgggcucuggagcagUCAGUGCAUGACAGAACUUGGgcccgg
aaggacc
.((((..((.((((((((((((((.((.((((..((((...(((....))))))))))).)).)))))))))))))).))
..)))).
---UCAGUGCAUGACAGAACU---------
------- 957 0,-3
---UCAGUGCAUGACAGAACUU--------
------- 90 0,-2
---UCAGUGCAUGACAGAACUUa-------
------- 3 0,-2
---UCAGUGCAUGACAGAACUUuu------
------- 3 0,-2
---UCAGUGCAUGACAGAACUUG-------
------- 240 0,-1
---UCAGUGCAUGACAGAACUUGa------
------- 2 0,-1
---UCAGUGCAUGACAGAACUUGaa-----
------- 3 0,-1
---UCAGUGCAUGACAGAACUUGu------
------- 3 0,-1
---UCAGUGCAUGACAGAACUUGG------
------- 1125 0,0
---UCAGUGCAUGACAGAACUUGGa-----
------- 208 0,0
---UCAGUGCAUGACAGAACUUGGaa----
------- 55 0,0
---UCAGUGCAUGACAGAACUUGGau----
------- 2 0,0
---UCAGUGCAUGACAGAACUUGGu-----

------- 1001 0,0

--UCAGUGCAUGACAGAACUUGGuu----

------- 107 0,0

--UCAGUGCAUGACAGAACUUGGG-----

------- 1006 0,1

--UCAGUGCAUGACAGAACUUGGGa----

------- 263 0,1

--UCAGUGCAUGACAGAACUUGGGaa---

------- 86 0,1

--UCAGUGCAUGACAGAACUUGGGau---

------- 8 0,1

--UCAGUGCAUGACAGAACUUGGGu----

------- 647 0,1

--UCAGUGCAUGACAGAACUUGGGuu---

------- 45 0,1

--UCAGUGCAUGACAGAACUUGGGC----

------- 2 0,2

--AGUGCAUGACAGAACUUG-------

------- 9 2,-1

--AGUGCAUGACAGAACUUGG------

------- 20 2,0

--AGUGCAUGACAGAACUUGGa-----

------- 3 2,0

--AGUGCAUGACAGAACUUGGaa----

------- 2 2,0

--AGUGCAUGACAGAACUUGGu-----

------- 18 2,0

--AGUGCAUGACAGAACUUGGuu----

------- 4 2,0

--AGUGCAUGACAGAACUUGGG-----

------- 22 2,1

--AGUGCAUGACAGAACUUGGGa----

------- 9 2,1

--AGUGCAUGACAGAACUUGGGu----

------- 9 2,1

--AGUGCAUGACAGAACUUGGGuu---

------- 2 2,1

>hsa-mir-1537 3p:40-61 SRX018959
acagcuguaauuagucaguuuucuguccuguccacacagAAAACCGUCUAGUUACAGUUGU
((((((((((((((.(.(((((((((.........))))))))).).))))))))))))))
---------------------------------------AAAACCGUCUAGUUACAG---- 4 0,-4

>hsa-mir-154 MA:15-36;mi:51-72 SRX018959
gugguacuugaagaUAGGUUAUCCGUGUUGCCUUCGcuuuauuugugacgAAUCAUACACGGUUGACCUAUUuuucagua
ccaa
.(((((((.(((((((((((((.((((((....(((((.........).))))....)))))).))))))))))))).))))
))).
--------------UAGGUUAUCCGUGUUGCCU---
---- 4 0,-3
--------------UAGGUUAUCCGUGUUGCCUU--
---- 2 0,-2
--------------UAGGUUAUCCGUGUUGCCUUC---
---- 10 0,-1
--------------UAGGUUAUCCGUGUUGCCUUCG--
---- 29 0,0
--AAUCAUACACGGUUGACCUAU---------
---- 2 0,-1
--AAUCAUACACGGUUGACCUAUU--------
---- 12 0,0
---AUCAUACACGGUUGACCUAUU--------
---- 2 1,0

>hsa-mir-155 MA:4-26;mi:43-64 SRX018959
cugUUAAUGCUAAUCGUGAUAGGGGUuuuugccuccaacugaCUCCUACAUAUUAGCAUUAACAg
((((((((((((((.(((.((((((((.(((....)))..)))))))))))))))))))))))))
---UUAAUGCUAAUCGUGAUA-- 64 0,-5
---UUAAUGCUAAUCGUGAUAG--- 15 0,-4
---UUAAUGCUAAUCGUGAUAGG-- 7 0,-3
---UUAAUGCUAAUCGUGAUAGGG--- 6 0,-2
---UUAAUGCUAAUCGUGAUAGGGa-- 2 0,-2
---UUAAUGCUAAUCGUGAUAGGGG-- 2 0,-1
---UUAAUGCUAAUCGUGAUAGGGGU--------------------------------------- 4 0,0
---UUAAUGCUAAUCGUGAUAGGGGUU-------------------------------------- 5 0,1
----UAAUGCUAAUCGUGAUAGGGGU--------------------------------------- 2 1,0

>hsa-mir-15a   MA:14-35;mi:51-72   SRX018959
ccuuggaguaaagUAGCAGCACAUAAUGGUUUGUGgauuuugaaaaggugCAGGCCAUAUUGUGCUGCCUCAaaaauaca
agg
(((((.......(..((((((((..((((((((((..((.....))..))))))))))..))))))))..).......))
)))
-------------UAGCAGCACAUAAUGGUU---
--- 40 0,-4
-------------UAGCAGCACAUAAUGGUUU--
--- 679 0,-3
-------------UAGCAGCACAUAAUGGUUUau--
--- 2   0,-3
-------------UAGCAGCACAUAAUGGUUUu---
--- 8   0,-3
-------------UAGCAGCACAUAAUGGUUUG---
--- 388 0,-2
-------------UAGCAGCACAUAAUGGUUUGa--
--- 2   0,-2
-------------UAGCAGCACAUAAUGGUUUGU--
--- 279 0,-1
-------------UAGCAGCACAUAAUGGUUUGUa---
--- 5   0,-1
-------------UAGCAGCACAUAAUGGUUUGUaa--
--- 3   0,-1
-------------UAGCAGCACAUAAUGGUUUGUG---
--- 32 0,0
--------------AGCAGCACAUAAUGGUUU--
--- 2   1,-3

>hsa-mir-15b   MA:20-41;mi:58-79   SRX018959
uugaggccuuaaaguacugUAGCAGCACAUCAUGGUUUACAugcuacagucaagaugCGAAUCAUUAUUUGCUGCUCUAg
aaauuuaaggaaauucau
.((((.(((((((...(((.(((((((.((.((((((..(((.((.......)))))..)))))).)).))))))).)))
...)))))))...)))).
-------------------UAGCAGCACAUCAUGGUU---
------------------ 6   0,-4
-------------------UAGCAGCACAUCAUGGUUa--
------------------ 2   0,-4
-------------------UAGCAGCACAUCAUGGUUU--

------------------ 56 0,-3
-------------------UAGCAGCACAUCAUGGUUUu---
------------------ 2   0,-3
-------------------UAGCAGCACAUCAUGGUUUA---
------------------ 297 0,-2
-------------------UAGCAGCACAUCAUGGUUUAa--
------------------ 16 0,-2
-------------------UAGCAGCACAUCAUGGUUUAu--
------------------ 3   0,-2
-------------------UAGCAGCACAUCAUGGUUUAC--
------------------ 130 0,-1
-------------------UAGCAGCACAUCAUGGUUUACA---------------------------------------
------------------ 336 0,0
-------------------UAGCAGCACAUCAUGGUUUACAa--------------------------------------
------------------ 17 0,0
--------------------AGCAGCACAUCAUGGUUUA---
------------------ 3   1,-2
---CGAAUCAUUAUUUGCUGCU----
------------------ 9   0,-3
---CGAAUCAUUAUUUGCUGCUCUAu
------------------ 2   0,0

>hsa-mir-16-1   MA:14-35;mi:56-77   SRX018959
gucagcagugccuUAGCAGCACGUAAAUAUUGGCGuuaagauucuaaaauuaucuCCAGUAUUAACUGUGCUGCUGAagu
aagguugac
((((((..(((.((((((((((((.((((((((.(.(((..........))).).)))))))).)).)))))))))).))
)..))))))
-------------UAGCAGCACGUAAAUAUU---
---------   167 0,-4
-------------UAGCAGCACGUAAAUAUUa--
---------   6   0,-4
-------------UAGCAGCACGUAAAUAUUaa---
---------   2   0,-4
-------------UAGCAGCACGUAAAUAUUG--
---------   501 0,-3
-------------UAGCAGCACGUAAAUAUUGa---
---------   3   0,-3
-------------UAGCAGCACGUAAAUAUUGu---

---------   2   0,-3
-------------UAGCAGCACGUAAAUAUUGG---
---------   1003   0,-2
-------------UAGCAGCACGUAAAUAUUGGa--
---------   14   0,-2
-------------UAGCAGCACGUAAAUAUUGGaa---
---------   7   0,-2
-------------UAGCAGCACGUAAAUAUUGGu--
---------   5   0,-2
-------------UAGCAGCACGUAAAUAUUGGC--
---------   365   0,-1
-------------UAGCAGCACGUAAAUAUUGGCa---
---------   151   0,-1
-------------UAGCAGCACGUAAAUAUUGGCaa--
---------   79   0,-1
-------------UAGCAGCACGUAAAUAUUGGCau--
---------   50   0,-1
-------------UAGCAGCACGUAAAUAUUGGCu---
---------   4   0,-1
-------------UAGCAGCACGUAAAUAUUGGCG---
---------   2243   0,0
-------------UAGCAGCACGUAAAUAUUGGCGa--
---------   59   0,0
-------------UAGCAGCACGUAAAUAUUGGCGaa---
---------   9   0,0
-------------UAGCAGCACGUAAAUAUUGGCGau---
---------   9   0,0
-------------UAGCAGCACGUAAAUAUUGGCGU--
---------   388   0,1
-------------UAGCAGCACGUAAAUAUUGGCGUU---
---------   20   0,2
-------------UAGCAGCACGUAAAUAUUGGCGUUu--
---------   2   0,2
--------------AGCAGCACGUAAAUAUUGG---
---------   3   1,-2
--------------AGCAGCACGUAAAUAUUGGCa---
---------   2   1,-1
--------------AGCAGCACGUAAAUAUUGGCG---

---------	19	1,0
--------------AGCAGCACGUAAAUAUUGGCGU--
---------	4	1,1
---------------GCAGCACGUAAAUAUUGGCG---
---------	4	2,0

>hsa-mir-16-2	MA:10-31;mi:53-74	SRX018959
guuccacucUAGCAGCACGUAAAUAUUGGCGuagugaaauauauauuaaacaCCAAUAUUACUGUGCUGCUUUAguguga
c
((..((((..(((((((((..((((((((.((..(((........))).)).))))))))..)))))))))..))))..)
)
--------CUAGCAGCACGUAAAUAUUGGC--
-	2	-1,-1
---------UAGCAGCACGUAAAUAUU---
-	167	0,-4
---------UAGCAGCACGUAAAUAUUa--
-	6	0,-4
---------UAGCAGCACGUAAAUAUUaa---
-	2	0,-4
---------UAGCAGCACGUAAAUAUUG--
-	501	0,-3
---------UAGCAGCACGUAAAUAUUGa---
-	3	0,-3
---------UAGCAGCACGUAAAUAUUGu---
-	2	0,-3
---------UAGCAGCACGUAAAUAUUGG---
-	1003	0,-2
---------UAGCAGCACGUAAAUAUUGGa--
-	14	0,-2
---------UAGCAGCACGUAAAUAUUGGaa---
-	7	0,-2
---------UAGCAGCACGUAAAUAUUGGu--
-	5	0,-2
---------UAGCAGCACGUAAAUAUUGGC--
-	365	0,-1
---------UAGCAGCACGUAAAUAUUGGCa---
-	151	0,-1
---------UAGCAGCACGUAAAUAUUGGCaa--

- 79 0,-1
---------UAGCAGCACGUAAAUAUUGGCau--
- 50 0,-1
---------UAGCAGCACGUAAAUAUUGGCu---
- 4 0,-1
---------UAGCAGCACGUAAAUAUUGGCG---
- 2243 0,0
---------UAGCAGCACGUAAAUAUUGGCGa--
- 59 0,0
---------UAGCAGCACGUAAAUAUUGGCGaa---
- 9 0,0
---------UAGCAGCACGUAAAUAUUGGCGau---
- 9 0,0
---------UAGCAGCACGUAAAUAUUGGCGU--
- 388 0,1
---------UAGCAGCACGUAAAUAUUGGCGUA---
- 3 0,2
----------AGCAGCACGUAAAUAUUGG---
- 3 1,-2
----------AGCAGCACGUAAAUAUUGGCa---
- 2 1,-1
----------AGCAGCACGUAAAUAUUGGCG---
- 19 1,0
----------AGCAGCACGUAAAUAUUGGCGU--
- 4 1,1
-----------GCAGCACGUAAAUAUUGGCG---
- 4 2,0
--CACCAAUAUUACUGUGCUGCUUU-------
- 2 -2,-1
---ACCAAUAUUACUGUGCUGC----------
- 3 -1,-4
---ACCAAUAUUACUGUGCUGCU---------
- 8 -1,-3
---ACCAAUAUUACUGUGCUGCUU--------
- 14 -1,-2
---ACCAAUAUUACUGUGCUGCUUa-------
- 2 -1,-2
---ACCAAUAUUACUGUGCUGCUUU-------

-    71 -1,-1
---ACCAAUAUUACUGUGCUGCUUUu------
-    36 -1,-1
---ACCAAUAUUACUGUGCUGCUUUA------
-    13 -1,0
---ACCAAUAUUACUGUGCUGCUUUAa-----
-    2   -1,0
--CCAAUAUUACUGUGCUGCUU--------
-    2   0,-2
--CCAAUAUUACUGUGCUGCUUU-------
-    10 0,-1
--CCAAUAUUACUGUGCUGCUUUA------
-    4   0,0
--CCAAUAUUACUGUGCUGCUUUAa-----
-    2   0,0

>hsa-mir-17 MA:14-36;mi:51-72   SRX018959
gucagaauaauguCAAAGUGCUUACAGUGCAGGUAGugauaugugcaucuACUGCAGUGAAGGCACUUGUAGcauuaugg
ugac
((((..(((((((..((((((((.((.(((((.(((((.......)).)))))))).)).))))))))...)))))))..
))))
------------UCAAAGUGCUUACAGUGCAG--
----   8   -1,-4
------------UCAAAGUGCUUACAGUGCAGGa--
----   4   -1,-3
------------UCAAAGUGCUUACAGUGCAGGU--
----   13   -1,-2
------------UCAAAGUGCUUACAGUGCAGGUAa--
----   2   -1,-1
-------------CAAAGUGCUUACAGUGCA---
----   21   0,-5
-------------CAAAGUGCUUACAGUGCAG--
----   193 0,-4
-------------CAAAGUGCUUACAGUGCAGG---
----   195 0,-3
-------------CAAAGUGCUUACAGUGCAGGU--
----   189 0,-2
-------------CAAAGUGCUUACAGUGCAGGUu---

---- 5 0,-2
-------------CAAAGUGCUUACAGUGCAGGUA---
---- 182 0,-1
-------------CAAAGUGCUUACAGUGCAGGUAa--
---- 43 0,-1
-------------CAAAGUGCUUACAGUGCAGGUAu--
---- 45 0,-1
-------------CAAAGUGCUUACAGUGCAGGUAG--
---- 198 0,0
-------------CAAAGUGCUUACAGUGCAGGUAGa---
---- 8 0,0
-------------CAAAGUGCUUACAGUGCAGGUAGU---
---- 12 0,1
--------------AAAGUGCUUACAGUGCAG--
---- 4 1,-4
--------------AAAGUGCUUACAGUGCAGG---
---- 4 1,-3
--------------AAAGUGCUUACAGUGCAGGU--
---- 3 1,-2
--------------AAAGUGCUUACAGUGCAGGUA---
---- 2 1,-1
--------------AAAGUGCUUACAGUGCAGGUAu--
---- 3 1,-1
--------------AAAGUGCUUACAGUGCAGGUAG--
---- 8 1,0
---UACUGCAGUGAAGGCACUUGU----------
---- 3 -1,-2
---UACUGCAGUGAAGGCACUUGUA---------
---- 2 -1,-1
--ACUGCAGUGAAGGCACUU------------
---- 107 0,-4
--ACUGCAGUGAAGGCACUUG-----------
---- 258 0,-3
--ACUGCAGUGAAGGCACUUGU----------
---- 487 0,-2
--ACUGCAGUGAAGGCACUUGUu---------
---- 2 0,-2
--ACUGCAGUGAAGGCACUUGUA---------

----   57 0,-1
--ACUGCAGUGAAGGCACUUGUAa--------
----   3   0,-1
--ACUGCAGUGAAGGCACUUGUAG--------
----   15 0,0
--ACUGCAGUGAAGGCACUUGUAGa-------
----   3   0,0
--ACUGCAGUGAAGGCACUUGUAGC-------
----   8   0,1
--ACUGCAGUGAAGGCACUUGUAGCu------
----   3   0,1
--ACUGCAGUGAAGGCACUUGUAGCA------
----   4   0,2
--ACUGCAGUGAAGGCACUUGUAGCAU-----
----   2   0,3
---CUGCAGUGAAGGCACUUG-----------
----   3   1,-3
---CUGCAGUGAAGGCACUUGU----------
----   15 1,-2
---CUGCAGUGAAGGCACUUGUA---------
----   9   1,-1
---CUGCAGUGAAGGCACUUGUAGC-------
----   5   1,1
--UGCAGUGAAGGCACUUGU----------
----   5   2,-2
--UGCAGUGAAGGCACUUGUA---------
----   2   2,-1

>hsa-mir-181a-1 MA:24-46;mi:64-85   SRX018959
ugaguuuugagguugcuucagugAACAUUCAACGCUGUCGGUGAGUuuggaauuaaaaucaaaACCAUCGACCGUUGAUU
GUACCcuauggcuaaccaucaucuacucca
.((((..((((((((((..((.(.(((.((((((..(((((((.((((.((.......)).))))))))))))))))).)
)).).))..))).)))).)))...))))..
----------------------GAACAUUCAACGCUGUCGGUGA------------------------------------
------------------------------   2   -1,-2
-----------------------AACAUUCAACGCUGUCGG---------------------------------------
------------------------------   144 0,-5
-----------------------AACAUUCAACGCUGUCGGU--------------------------------------

------------------------------ 5 0,-4
------------------------AACAUUCAACGCUGUCGGUaa------------------------------------
------------------------------ 2 0,-4
------------------------AACAUUCAACGCUGUCGGUau------------------------------------
------------------------------ 2 0,-4
------------------------AACAUUCAACGCUGUCGGUG-------------------------------------
------------------------------ 22 0,-3
------------------------AACAUUCAACGCUGUCGGUGu------------------------------------
------------------------------ 2 0,-3
------------------------AACAUUCAACGCUGUCGGUGA------------------------------------
------------------------------ 139 0,-2
------------------------AACAUUCAACGCUGUCGGUGAa-----------------------------------
------------------------------ 4 0,-2
------------------------AACAUUCAACGCUGUCGGUGAG-----------------------------------
------------------------------ 733 0,-1
------------------------AACAUUCAACGCUGUCGGUGAGa----------------------------------
------------------------------ 5 0,-1
------------------------AACAUUCAACGCUGUCGGUGAGU----------------------------------
------------------------------ 80 0,0
------------------------AACAUUCAACGCUGUCGGUGAGUa---------------------------------
------------------------------ 2 0,0
------------------------AACAUUCAACGCUGUCGGUGAGUau--------------------------------
------------------------------ 3 0,0
------------------------AACAUUCAACGCUGUCGGUGAGUU---------------------------------
------------------------------ 111 0,1
------------------------AACAUUCAACGCUGUCGGUGAGUUa--------------------------------
------------------------------ 4 0,1
------------------------AACAUUCAACGCUGUCGGUGAGUUU--------------------------------
------------------------------ 56 0,2
-------------------------ACAUUCAACGCUGUCGGUGAG-----------------------------------
------------------------------ 5 1,-1
-------------------------ACAUUCAACGCUGUCGGUGAGUU---------------------------------
------------------------------ 2 1,1
---AACCAUCGACCGUUGAUU
GUAu--------------------------- 3 -1,-2
--ACCAUCGACCGUUGAUU
G----------------------------- 13 0,-4
--ACCAUCGACCGUUGAUU

GU----------------------------- 2 0,-3
---ACCAUCGACCGUUGAUU
GUA---------------------------- 4 0,-2
---ACCAUCGACCGUUGAUU
GUAC--------------------------- 6 0,-1
---ACCAUCGACCGUUGAUU
GUACC-------------------------- 8 0,0

>hsa-mir-181a-2 MA:39-61;mi:77-98 SRX018959
agaagggcuaucaggccagccuucagaggacuccaaggAACAUUCAACGCUGUCGGUGAGUuugggauuugaaaaaACCA
CUGACCGUUGACUGUACCuuggggguccuua
.......................((((((((((((.(((.((((((..(((((((.((((..........))))))
))))))))))).))).))))))))))))).
-------------------------------------GAACAUUCAACGCUGUCGGUGA---------------------
------------------------------- 2 -1,-2
--------------------------------------AACAUUCAACGCUGUCGG------------------------
------------------------------- 144 0,-5
--------------------------------------AACAUUCAACGCUGUCGGU-----------------------
------------------------------- 5 0,-4
--------------------------------------AACAUUCAACGCUGUCGGUaa---------------------
------------------------------- 2 0,-4
--------------------------------------AACAUUCAACGCUGUCGGUau---------------------
------------------------------- 2 0,-4
--------------------------------------AACAUUCAACGCUGUCGGUG----------------------
------------------------------- 22 0,-3
--------------------------------------AACAUUCAACGCUGUCGGUGu---------------------
------------------------------- 2 0,-3
--------------------------------------AACAUUCAACGCUGUCGGUGA---------------------
------------------------------- 139 0,-2
--------------------------------------AACAUUCAACGCUGUCGGUGAa--------------------
------------------------------- 4 0,-2
--------------------------------------AACAUUCAACGCUGUCGGUGAG--------------------
------------------------------- 733 0,-1
--------------------------------------AACAUUCAACGCUGUCGGUGAGa-------------------
------------------------------- 5 0,-1
--------------------------------------AACAUUCAACGCUGUCGGUGAGU-------------------
------------------------------- 80 0,0
--------------------------------------AACAUUCAACGCUGUCGGUGAGUa------------------

------------------------------ 2   0,0
--------------------------------------AACAUUCAACGCUGUCGGUGAGUau-----------------
------------------------------ 3   0,0
--------------------------------------AACAUUCAACGCUGUCGGUGAGUU------------------
------------------------------ 111 0,1
--------------------------------------AACAUUCAACGCUGUCGGUGAGUUa-----------------
------------------------------ 4   0,1
--------------------------------------AACAUUCAACGCUGUCGGUGAGUUU-----------------
------------------------------ 56 0,2
---------------------------------------ACAUUCAACGCUGUCGGUGAG--------------------
------------------------------ 5   1,-1
---------------------------------------ACAUUCAACGCUGUCGGUGAGUU------------------
------------------------------ 2   1,1
---AACCA
CUGACCGUUGACU----------------- 4   -1,-5
---AACCA
CUGACCGUUGACUG---------------- 2   -1,-4
--ACCA
CUGACCGUUGACUG---------------- 39 0,-4
--ACCA
CUGACCGUUGACUGU--------------- 9   0,-3
--ACCA
CUGACCGUUGACUGUA-------------- 6   0,-2
--ACCA
CUGACCGUUGACUGUAa------------- 3   0,-2
--ACCA
CUGACCGUUGACUGUAaa------------ 3   0,-2
--ACCA
CUGACCGUUGACUGUAC------------- 6   0,-1
--ACCA
CUGACCGUUGACUGUACu------------ 2   0,-1
--ACCA
CUGACCGUUGACUGUACC------------ 19 0,0
--ACCA
CUGACCGUUGACUGUACCU----------- 2   0,1

>hsa-mir-181b-1 5p:36-58   SRX018959
ccugugcagagauuauuuuuuaaaaggucacaaucAACAUUCAUUGCUGUCGGUGGGUugaacuguguggacaagcucac

ugaacaaugaaugcaacuguggccccgcuu
.......................((((((.....(((((((((...((((((((((...((....))...)))))))
))).))))))))))....)))))))......
-----------------------------------AACAUUCAUUGCUGUCGG---------------------------
------------------------------ 27 0,-5
-----------------------------------AACAUUCAUUGCUGUCGGU--------------------------
------------------------------ 2 0,-4
-----------------------------------AACAUUCAUUGCUGUCGGUG-------------------------
------------------------------ 19 0,-3
-----------------------------------AACAUUCAUUGCUGUCGGUGu------------------------
------------------------------ 2 0,-3
-----------------------------------AACAUUCAUUGCUGUCGGUGGG-----------------------
------------------------------ 72 0,-1
-----------------------------------AACAUUCAUUGCUGUCGGUGGGU----------------------
------------------------------ 8 0,0
-----------------------------------AACAUUCAUUGCUGUCGGUGGGUaa--------------------
------------------------------ 3 0,0
-----------------------------------AACAUUCAUUGCUGUCGGUGGGUau--------------------
------------------------------ 4 0,0
-----------------------------------AACAUUCAUUGCUGUCGGUGGGUU---------------------
------------------------------ 26 0,1
-----------------------------------AACAUUCAUUGCUGUCGGUGGGUUa--------------------
------------------------------ 5 0,1
------------------------------------ACAUUCAUUGCUGUCGGUGGGU----------------------
------------------------------ 2 1,0
------------------------------------ACAUUCAUUGCUGUCGGUGGGUU---------------------
------------------------------ 2 1,1
---CUCAC
UGAACAAUGAAUGCA--------------- 2
---CUCAC
UGAACAAUGAAUGCAu-------------- 2

>hsa-mir-181b-2 5p:16-38 SRX018959
cugauggcugcacucAACAUUCAUUGCUGUCGGUGGGUuugagucugaaucaacucacugaucaaugaaugcaaacugcg
gaccaaaca
....(((((((.....(((((((((..(((((((((((.((.......))))))))))))))))))))))).....))))
).)))....
---------------AACAUUCAUUGCUGUCGG---

---------   27   0,-5
---------------AACAUUCAUUGCUGUCGGU--
---------   2   0,-4
---------------AACAUUCAUUGCUGUCGGUG---
---------   19   0,-3
---------------AACAUUCAUUGCUGUCGGUGu--
---------   2   0,-3
---------------AACAUUCAUUGCUGUCGGUGGG---
---------   72   0,-1
---------------AACAUUCAUUGCUGUCGGUGGGU--
---------   8   0,0
---------------AACAUUCAUUGCUGUCGGUGGGUaa--
---------   3   0,0
---------------AACAUUCAUUGCUGUCGGUGGGUau--
---------   4   0,0
---------------AACAUUCAUUGCUGUCGGUGGGUU---
---------   26   0,1
---------------AACAUUCAUUGCUGUCGGUGGGUUa--
---------   5   0,1
---------------AACAUUCAUUGCUGUCGGUGGGUUU--
---------   2   0,2
----------------ACAUUCAUUGCUGUCGGUGGGU--
---------   2   1,0
----------------ACAUUCAUUGCUGUCGGUGGGUU---
---------   2   1,1
---CUCACUGAUCAAUGAAUGCA-------
---------   2

>hsa-mir-181c   MA:27-48;mi:65-86   SRX018959
cggaaaauuugccaaggguuugggggAACAUUCAACCUGUCGGUGAGUuugggcagcucaggcaAACCAUCGACCGUUGA
GUGGACccugaggccuggaauugccauccu
.(((.......(((.((.((..(((...((((((((..(((((((.(((((...........))))))))))))).)))))
)))..)))..)).)))))........))).
--------------------------AACAUUCAACCUGUCGGUGA----------------------------------
------------------------------   2   0,-2
--------------------------AACAUUCAACCUGUCGGUGAG---------------------------------
------------------------------   9   0,-1
--------------------------AACAUUCAACCUGUCGGUGAGUU-------------------------------

------------------------------ 6   0,1
--------------------------AACAUUCAACCUGUCGGUGAGUUU------------------------------
------------------------------ 5   0,2
---ACCAUCGACCGUUGA
GUG--------------------------- 2   1,-3

>hsa-mir-181d   5p:36-58   SRX018959
guccccuccccuaggccacagccgaggucacaaucAACAUUCAUUGUUGUCGGUGGGUugugaggacugaggccagaccc
accgggggaugaaugucacuguggcugggccagacacggcuuaaggggaauggggac
(((((((((((((((((...(((..(((((((....(((((((((.(..((((((((((....((.(....))).)))))
)))))).)))))))))...))))))).))).......))))).))))))..))))))
-----------------------------------AACAUUCAUUGUUGUCGGUG-------------------------
---   4   0,-3
-----------------------------------AACAUUCAUUGUUGUCGGUGG------------------------
---   2   0,-2
-----------------------------------AACAUUCAUUGUUGUCGGUGGG-----------------------
---   6   0,-1
-----------------------------------AACAUUCAUUGUUGUCGGUGGGa----------------------
---   2   0,-1
-----------------------------------AACAUUCAUUGUUGUCGGUGGGU----------------------
---   5   0,0
-----------------------------------AACAUUCAUUGUUGUCGGUGGGUa---------------------
---   4   0,0
-----------------------------------AACAUUCAUUGUUGUCGGUGGGUaa--------------------
---   2   0,0
-----------------------------------AACAUUCAUUGUUGUCGGUGGGUau--------------------
---   4   0,0
-----------------------------------AACAUUCAUUGUUGUCGGUGGGUU---------------------
---   16 0,1
-----------------------------------AACAUUCAUUGUUGUCGGUGGGUUu--------------------
---   5   0,1

>hsa-mir-185   MA:15-36;mi:50-71   SRX018959
agggggcgagggauUGGAGAGAAAGGCAGUUCCUGAugguccccuccccAGGGGCUGGCUUUCCUCUGGUCcuucccucc
ca
.(((((.(((((((..(((.(((((.(((((((((..((......)).))))))))).))))))))..))))))).))))
).
--------------UGGAGAGAAAGGCAGUUC--

-- 53 0,-4

--------------UGGAGAGAAAGGCAGUUCC---

-- 104 0,-3

--------------UGGAGAGAAAGGCAGUUCCU--

-- 1413 0,-2

--------------UGGAGAGAAAGGCAGUUCCUa---

-- 3 0,-2

--------------UGGAGAGAAAGGCAGUUCCUaa--

-- 3 0,-2

--------------UGGAGAGAAAGGCAGUUCCUau--

-- 3 0,-2

--------------UGGAGAGAAAGGCAGUUCCUu---

-- 2 0,-2

--------------UGGAGAGAAAGGCAGUUCCUG---

-- 570 0,-1

--------------UGGAGAGAAAGGCAGUUCCUGu--

-- 70 0,-1

--------------UGGAGAGAAAGGCAGUUCCUGuu---

-- 19 0,-1

--------------UGGAGAGAAAGGCAGUUCCUGA--

-- 6298 0,0

--------------UGGAGAGAAAGGCAGUUCCUGAa---

-- 1059 0,0

--------------UGGAGAGAAAGGCAGUUCCUGAaa--

-- 38 0,0

--------------UGGAGAGAAAGGCAGUUCCUGAau--

-- 41 0,0

--------------UGGAGAGAAAGGCAGUUCCUGAU---

-- 1479 0,1

--------------UGGAGAGAAAGGCAGUUCCUGAUa--

-- 94 0,1

--------------UGGAGAGAAAGGCAGUUCCUGAUaa---

-- 2 0,1

--------------UGGAGAGAAAGGCAGUUCCUGAUau---

-- 4 0,1

--------------UGGAGAGAAAGGCAGUUCCUGAUu--

-- 95 0,1

---------------GGAGAGAAAGGCAGUUCC---

-- 2 1,-3
---------------GGAGAGAAAGGCAGUUCCU--
-- 13 1,-2
---------------GGAGAGAAAGGCAGUUCCUG---
-- 2 1,-1
---------------GGAGAGAAAGGCAGUUCCUGA--
-- 45 1,0
---------------GGAGAGAAAGGCAGUUCCUGAa---
-- 10 1,0
---------------GGAGAGAAAGGCAGUUCCUGAU---
-- 24 1,1
---------------GGAGAGAAAGGCAGUUCCUGAUu--
-- 2 1,1
---AGGGGCUGGCUUUCCUCU-------------
-- 17 0,-4
---AGGGGCUGGCUUUCCUCUG------------
-- 2 0,-3
---AGGGGCUGGCUUUCCUCUGuu----------
-- 2 0,-3
---AGGGGCUGGCUUUCCUCUGG-----------
-- 4 0,-2
---AGGGGCUGGCUUUCCUCUGGU----------
-- 11 0,-1
---AGGGGCUGGCUUUCCUCUGGUa---------
-- 2 0,-1
---AGGGGCUGGCUUUCCUCUGGUaa--------
-- 3 0,-1
---AGGGGCUGGCUUUCCUCUGGUu---------
-- 2 0,-1

>hsa-mir-186 MA:15-36;mi:54-75 SRX018959
ugcuuguaacuuucCAAAGAAUUCUCCUUUUGGGCUuucugguuuuauuuuaaGCCCAAAGGUGAAUUUUUUGGGaaguu
ugagcu
.((((.((((((((((((((((((.(((((.((((((..............))))))))))).)))))))))))))))))
))))).
-------------CCAAAGAAUUCUCCUUUUGGGCU--
------ 4 -1,0
--------------CAAAGAAUUCUCCUUUUG--

------ 15 0,-4
--------------CAAAGAAUUCUCCUUUUGG--
------ 9 0,-3
--------------CAAAGAAUUCUCCUUUUGGG---
------ 42 0,-2
--------------CAAAGAAUUCUCCUUUUGGGa--
------ 2 0,-2
--------------CAAAGAAUUCUCCUUUUGGGaa---
------ 2 0,-2
--------------CAAAGAAUUCUCCUUUUGGGC--
------ 30 0,-1
--------------CAAAGAAUUCUCCUUUUGGGCa---
------ 68 0,-1
--------------CAAAGAAUUCUCCUUUUGGGCaa--
------ 40 0,-1
--------------CAAAGAAUUCUCCUUUUGGGCau--
------ 162 0,-1
--------------CAAAGAAUUCUCCUUUUGGGCU---
------ 246 0,0
--------------CAAAGAAUUCUCCUUUUGGGCUa--
------ 52 0,0
--------------CAAAGAAUUCUCCUUUUGGGCUaa---
------ 7 0,0
--------------CAAAGAAUUCUCCUUUUGGGCUau---
------ 27 0,0
--------------CAAAGAAUUCUCCUUUUGGGCUU--
------ 531 0,1
--------------CAAAGAAUUCUCCUUUUGGGCUUa---
------ 17 0,1
--------------CAAAGAAUUCUCCUUUUGGGCUUau--
------ 6 0,1
--------------CAAAGAAUUCUCCUUUUGGGCUUU---
------ 32 0,2
--------------CAAAGAAUUCUCCUUUUGGGCUUUa--
------ 2 0,2
---------------AAAGAAUUCUCCUUUUGG--
------ 3 1,-3
---------------AAAGAAUUCUCCUUUUGGG---

------ 3 1,-2
---------------AAAGAAUUCUCCUUUUGGGC---
------ 4 1,-1
---------------AAAGAAUUCUCCUUUUGGGCa--
------ 9 1,-1
---------------AAAGAAUUCUCCUUUUGGGCaa---
------ 2 1,-1
---------------AAAGAAUUCUCCUUUUGGGCau---
------ 2 1,-1
---------------AAAGAAUUCUCCUUUUGGGCU--
------ 13 1,0
---------------AAAGAAUUCUCCUUUUGGGCUa---
------ 4 1,0
---------------AAAGAAUUCUCCUUUUGGGCUaa--
------ 2 1,0
---------------AAAGAAUUCUCCUUUUGGGCUau--
------ 7 1,0
---------------AAAGAAUUCUCCUUUUGGGCUU---
------ 46 1,1
---------------AAAGAAUUCUCCUUUUGGGCUUa--
------ 6 1,1
---------------AAAGAAUUCUCCUUUUGGGCUUU--
------ 4 1,2

>hsa-mir-187 mi:35-56;MA:71-92 SRX018959
ggucgggcucaccaugacacagugugagaccucgGGCUACAACACAGGACCCGGGCgcugcucugaccccUCGUGUCUUG
UGUUGCAGCCGGagggacgcagguccgca
.(.((((((.............((((...((((.((((.(((((((((((.((((.((......).).)).)).))))))
))))).)))).))))..))))))))))).
----------------------------------GCUACAACACAGGACCCGGGCGu-----------------------
----------------------------- 2 1,1
--UCGUGUCUUG
UGUUGCAGC-------------------- 2 0,-3
--UCGUGUCUUG
UGUUGCAGCC------------------- 2 0,-2
--UCGUGUCUUG
UGUUGCAGCCGG----------------- 8 0,0
--UCGUGUCUUG

UGUUGCAGCCGGu---------------- 3 0,0
--UCGUGUCUUG
UGUUGCAGCCGGuu--------------- 10 0,0

>hsa-mir-18a MA:6-28;mi:47-69 SRX018959
uguucUAAGGUGCAUCUAGUGCAGAUAGugaaguagauuagcaucuACUGCCCUAAGUGCUCCUUCUGGca
.(((..((((.(((..(((.((((.(((((..(.....)..)).))))))).)))..))).)))).))).
-----UAAGGUGCAUCUAGUGCAGA-- 5 0,-3
-----UAAGGUGCAUCUAGUGCAGAU--- 23 0,-2
-----UAAGGUGCAUCUAGUGCAGAUA-- 19 0,-1
-----UAAGGUGCAUCUAGUGCAGAUAa--- 3 0,-1
-----UAAGGUGCAUCUAGUGCAGAUAG--- 5 0,0
---ACUGCCCUAAGUGCUCCUUCU---- 6 0,-2

>hsa-mir-190 5p:15-36 SRX018959
ugcaggccucugugUGAUAUGUUUGAUAUAUUAGGUuguuauuuaauccaacuauauaucaaacauauuccuacagugucuugcc
.((((.(.(((((..((((((((((((((..(((((.........))))))))))))))))))..).)))).).)
)))).
--------------UGAUAUGUUUGAUAUAUUAGGUUG--- 2 0,2

>hsa-mir-1908 5p:12-32 SRX018959
cgggaaugccgCGGCGGGGACGGCGAUUGGUCcguauguguggugccaccggccgccggcuccgccccggcccccgcccc
((((...((((.(((((((.(((((..((((..((((.....)))).))))..))))).))))))).)))).)))).....
-----------CGGCGGGGACGGCGAUUGGUC--- 3 0,0
-----------CGGCGGGGACGGCGAUUGGUCu-- 2 0,0

>hsa-mir-190b 5p:11-31 SRX018959
ugcuucugugUGAUAUGUUUGAUAUUGGGUUguuuaauuaggaaccaacuaaaugucaaacauauucuuacagcagcag
((((.(((((.((((((((((((((.((((((((......))).))))).)))))))))))))))..))))).)))).
----------UGAUAUGUUUGAUAUUGGGUUGU-- 2 0,2

>hsa-mir-191 MA:16-38;mi:58-79 SRX018959
cggcuggacagcgggCAACGGAAUCCCAAAAGCAGCUGuugucuccagagcauuccaGCUGCGCUUGGAUUUCGUCCCCu

gcucuccugccu
.(((.(((.((((((..(((((((((.((..(((((((..((((....)).)).)).)))))))..)))))))))))..)))
))).))).))).
----------------CAACGGAAUCCCAAAAGC---
------------ 200 0,-5
----------------CAACGGAAUCCCAAAAGCu--
------------ 3 0,-5
----------------CAACGGAAUCCCAAAAGCA--
------------ 694 0,-4
----------------CAACGGAAUCCCAAAAGCAa---
------------ 8 0,-4
----------------CAACGGAAUCCCAAAAGCAu---
------------ 3 0,-4
----------------CAACGGAAUCCCAAAAGCAG---
------------ 2551 0,-3
----------------CAACGGAAUCCCAAAAGCAGa--
------------ 11 0,-3
----------------CAACGGAAUCCCAAAAGCAGau---
------------ 29 0,-3
----------------CAACGGAAUCCCAAAAGCAGu--
------------ 15 0,-3
----------------CAACGGAAUCCCAAAAGCAGuu---
------------ 16 0,-3
----------------CAACGGAAUCCCAAAAGCAGC--
------------ 8192 0,-2
----------------CAACGGAAUCCCAAAAGCAGCa---
------------ 85 0,-2
----------------CAACGGAAUCCCAAAAGCAGCaa--
------------ 12 0,-2
----------------CAACGGAAUCCCAAAAGCAGCau--
------------ 5 0,-2
----------------CAACGGAAUCCCAAAAGCAGCU---
------------ 19298 0,-1
----------------CAACGGAAUCCCAAAAGCAGCUa--
------------ 232 0,-1
----------------CAACGGAAUCCCAAAAGCAGCUaa---
------------ 27 0,-1
----------------CAACGGAAUCCCAAAAGCAGCUau---

----------- 98 0,-1
---------------CAACGGAAUCCCAAAAGCAGCUu--
----------- 358 0,-1
---------------CAACGGAAUCCCAAAAGCAGCUuu---
----------- 111 0,-1
---------------CAACGGAAUCCCAAAAGCAGCUG--
----------- 2703 0,0
---------------CAACGGAAUCCCAAAAGCAGCUGa---
----------- 203 0,0
---------------CAACGGAAUCCCAAAAGCAGCUGaa--
----------- 13 0,0
---------------CAACGGAAUCCCAAAAGCAGCUGau--
----------- 6 0,0
---------------CAACGGAAUCCCAAAAGCAGCUGU---
----------- 1735 0,1
---------------CAACGGAAUCCCAAAAGCAGCUGUa--
----------- 6 0,1
---------------CAACGGAAUCCCAAAAGCAGCUGUU--
----------- 73 0,2
----------------AACGGAAUCCCAAAAGCA--
----------- 68 1,-4
----------------AACGGAAUCCCAAAAGCAG---
----------- 342 1,-3
----------------AACGGAAUCCCAAAAGCAGa--
----------- 4 1,-3
----------------AACGGAAUCCCAAAAGCAGu--
----------- 4 1,-3
----------------AACGGAAUCCCAAAAGCAGuu---
----------- 4 1,-3
----------------AACGGAAUCCCAAAAGCAGC--
----------- 1177 1,-2
----------------AACGGAAUCCCAAAAGCAGCa---
----------- 2 1,-2
----------------AACGGAAUCCCAAAAGCAGCU---
----------- 2873 1,-1
----------------AACGGAAUCCCAAAAGCAGCUa--
----------- 20 1,-1
----------------AACGGAAUCCCAAAAGCAGCUaa---

----------- 4 1,-1
----------------AACGGAAUCCCAAAAGCAGCUau---
----------- 28 1,-1
----------------AACGGAAUCCCAAAAGCAGCUu--
----------- 19 1,-1
----------------AACGGAAUCCCAAAAGCAGCUuu---
----------- 11 1,-1
----------------AACGGAAUCCCAAAAGCAGCUG--
----------- 405 1,0
----------------AACGGAAUCCCAAAAGCAGCUGa---
----------- 39 1,0
----------------AACGGAAUCCCAAAAGCAGCUGaa--
----------- 2 1,0
----------------AACGGAAUCCCAAAAGCAGCUGU---
----------- 466 1,1
----------------AACGGAAUCCCAAAAGCAGCUGUa--
----------- 7 1,1
----------------AACGGAAUCCCAAAAGCAGCUGUU--
----------- 10 1,2
----------------AACGGAAUCCCAAAAGCAGCUGUUa---------------------------------------
----------- 2 1,2
----------------AACGGAAUCCCAAAAGCAGCUGUUu---------------------------------------
----------- 4 1,2
-----------------ACGGAAUCCCAAAAGCAG---
----------- 3 2,-3
-----------------ACGGAAUCCCAAAAGCAGC--
----------- 7 2,-2
-----------------ACGGAAUCCCAAAAGCAGCU---
----------- 14 2,-1
-----------------ACGGAAUCCCAAAAGCAGCUG--
----------- 3 2,0

>hsa-mir-1910 5p:12-32 SRX018959
ugucccuucagCCAGUCCUGUGCCUGCCGCCUuugugcuguccuuggagggaggcagaagcaggaugacaaugagggcaa
((.((((.((....(((((((..(((((.(((((............))))).)))))..))))))).....)))))))).
--GAGGCAGAAGCAGGAUGACA----------
2

>hsa-mir-192   MA:24-44;mi:67-88   SRX018959
gccgagaccgagugcacagggcuCUGACCUAUGAAUUGACAGCCagugcucucgucuccccucuggCUGCCAAUUCCAUA
GGUCACAGguauguucgccucaaugccagc
.........(((.(((((.(.((.((((((((((((((.(((((((................))))))).))))).))))
))))).)).).)))..)))))).........
---------------------CUCUGACCUAUGAAUUGACAGC-------------------------------------
------------------------------ 6   -2,-1
---------------------CUCUGACCUAUGAAUUGACAGCC------------------------------------
------------------------------ 3   -2,0
----------------------UCUGACCUAUGAAUUGAC--
------------------------------ 5   -1,-4
----------------------UCUGACCUAUGAAUUGACA---------------------------------------
------------------------------ 25   -1,-3
----------------------UCUGACCUAUGAAUUGACAG--------------------------------------
------------------------------ 96   -1,-2
----------------------UCUGACCUAUGAAUUGACAGu-------------------------------------
------------------------------ 6   -1,-2
----------------------UCUGACCUAUGAAUUGACAGC-------------------------------------
------------------------------ 461 -1,-1
----------------------UCUGACCUAUGAAUUGACAGCa------------------------------------
------------------------------ 44   -1,-1
----------------------UCUGACCUAUGAAUUGACAGCaa-----------------------------------
------------------------------ 4   -1,-1
----------------------UCUGACCUAUGAAUUGACAGCu------------------------------------
------------------------------ 7   -1,-1
----------------------UCUGACCUAUGAAUUGACAGCC------------------------------------
------------------------------ 2196   -1,0
----------------------UCUGACCUAUGAAUUGACAGCCu-----------------------------------
------------------------------ 158 -1,0
----------------------UCUGACCUAUGAAUUGACAGCCuu----------------------------------
------------------------------ 41   -1,0
----------------------UCUGACCUAUGAAUUGACAGCCA-----------------------------------
------------------------------ 313 -1,1
----------------------UCUGACCUAUGAAUUGACAGCCAa----------------------------------
------------------------------ 9   -1,1
----------------------UCUGACCUAUGAAUUGACAGCCAu----------------------------------
------------------------------ 94   -1,1
----------------------UCUGACCUAUGAAUUGACAGCCAuu---------------------------------

----------------------------- 41 -1,1
---------------------UCUGACCUAUGAAUUGACAGCCAG---------------------------------
----------------------------- 3 -1,2
---------------------UCUGACCUAUGAAUUGACAGCCAGU--------------------------------
----------------------------- 2 -1,3
----------------------CUGACCUAUGAAUUGACA--------------------------------------
----------------------------- 5889 0,-3
----------------------CUGACCUAUGAAUUGACAa-------------------------------------
----------------------------- 19 0,-3
----------------------CUGACCUAUGAAUUGACAaa------------------------------------
----------------------------- 18 0,-3
----------------------CUGACCUAUGAAUUGACAu-------------------------------------
----------------------------- 2 0,-3
----------------------CUGACCUAUGAAUUGACAuu------------------------------------
----------------------------- 6 0,-3
----------------------CUGACCUAUGAAUUGACAG-------------------------------------
----------------------------- 14715 0,-2
----------------------CUGACCUAUGAAUUGACAGa------------------------------------
----------------------------- 364 0,-2
----------------------CUGACCUAUGAAUUGACAGaa-----------------------------------
----------------------------- 42 0,-2
----------------------CUGACCUAUGAAUUGACAGau-----------------------------------
----------------------------- 7 0,-2
----------------------CUGACCUAUGAAUUGACAGu------------------------------------
----------------------------- 540 0,-2
----------------------CUGACCUAUGAAUUGACAGuu-----------------------------------
----------------------------- 94 0,-2
----------------------CUGACCUAUGAAUUGACAGC------------------------------------
----------------------------- 150469 0,-1
----------------------CUGACCUAUGAAUUGACAGCa-----------------------------------
----------------------------- 14206 0,-1
----------------------CUGACCUAUGAAUUGACAGCaa----------------------------------
----------------------------- 2173 0,-1
----------------------CUGACCUAUGAAUUGACAGCau----------------------------------
----------------------------- 708 0,-1
----------------------CUGACCUAUGAAUUGACAGCu-----------------------------------
----------------------------- 3031 0,-1
----------------------CUGACCUAUGAAUUGACAGCuu----------------------------------

----------------------------- 204 0,-1

----------------------CUGACCUAUGAAUUGACAGCC-----------------------------------

----------------------------- 460348 0,0

----------------------CUGACCUAUGAAUUGACAGCCu----------------------------------

----------------------------- 26208 0,0

----------------------CUGACCUAUGAAUUGACAGCCuu---------------------------------

----------------------------- 6096 0,0

----------------------CUGACCUAUGAAUUGACAGCCA----------------------------------

----------------------------- 219691 0,1

----------------------CUGACCUAUGAAUUGACAGCCAa---------------------------------

----------------------------- 7645 0,1

----------------------CUGACCUAUGAAUUGACAGCCAaa--------------------------------

----------------------------- 627 0,1

----------------------CUGACCUAUGAAUUGACAGCCAau--------------------------------

----------------------------- 1230 0,1

----------------------CUGACCUAUGAAUUGACAGCCAu---------------------------------

----------------------------- 88113 0,1

----------------------CUGACCUAUGAAUUGACAGCCAuu--------------------------------

----------------------------- 36071 0,1

----------------------CUGACCUAUGAAUUGACAGCCAG---------------------------------

----------------------------- 1947 0,2

----------------------CUGACCUAUGAAUUGACAGCCAGa--------------------------------

----------------------------- 173 0,2

----------------------CUGACCUAUGAAUUGACAGCCAGaa-------------------------------

----------------------------- 9 0,2

----------------------CUGACCUAUGAAUUGACAGCCAGau-------------------------------

----------------------------- 5 0,2

----------------------CUGACCUAUGAAUUGACAGCCAGU--------------------------------

----------------------------- 1592 0,3

----------------------CUGACCUAUGAAUUGACAGCCAGUa-------------------------------

----------------------------- 96 0,3

----------------------CUGACCUAUGAAUUGACAGCCAGUu-------------------------------

----------------------------- 396 0,3

----------------------CUGACCUAUGAAUUGACAGCCAGUG-------------------------------

----------------------------- 3 0,4

-----------------------UGACCUAUGAAUUGACAG-------------------------------------

----------------------------- 3441 1,-2

-----------------------UGACCUAUGAAUUGACAGu------------------------------------

------------------------------ 33 1,-2
------------------------UGACCUAUGAAUUGACAGC------------------------------------
------------------------------ 37051 1,-1
------------------------UGACCUAUGAAUUGACAGCa-----------------------------------
------------------------------ 496 1,-1
------------------------UGACCUAUGAAUUGACAGCaa----------------------------------
------------------------------ 148 1,-1
------------------------UGACCUAUGAAUUGACAGCau----------------------------------
------------------------------ 26 1,-1
------------------------UGACCUAUGAAUUGACAGCu-----------------------------------
------------------------------ 88 1,-1
------------------------UGACCUAUGAAUUGACAGCuu----------------------------------
------------------------------ 5 1,-1
------------------------UGACCUAUGAAUUGACAGCC-----------------------------------
------------------------------ 16524 1,0
------------------------UGACCUAUGAAUUGACAGCCu----------------------------------
------------------------------ 529 1,0
------------------------UGACCUAUGAAUUGACAGCCuu---------------------------------
------------------------------ 118 1,0
------------------------UGACCUAUGAAUUGACAGCCA----------------------------------
------------------------------ 36514 1,1
------------------------UGACCUAUGAAUUGACAGCCAa---------------------------------
------------------------------ 2135 1,1
------------------------UGACCUAUGAAUUGACAGCCAaa--------------------------------
------------------------------ 862 1,1
------------------------UGACCUAUGAAUUGACAGCCAau--------------------------------
------------------------------ 935 1,1
------------------------UGACCUAUGAAUUGACAGCCAu---------------------------------
------------------------------ 10025 1,1
------------------------UGACCUAUGAAUUGACAGCCAuu--------------------------------
------------------------------ 6881 1,1
------------------------UGACCUAUGAAUUGACAGCCAG---------------------------------
------------------------------ 8053 1,2
------------------------UGACCUAUGAAUUGACAGCCAGa--------------------------------
------------------------------ 1514 1,2
------------------------UGACCUAUGAAUUGACAGCCAGaa-------------------------------
------------------------------ 79 1,2
------------------------UGACCUAUGAAUUGACAGCCAGau-------------------------------

------------------------------ 74 1,2
------------------------UGACCUAUGAAUUGACAGCCAGU---------------------------------
------------------------------ 9806 1,3
------------------------UGACCUAUGAAUUGACAGCCAGUa--------------------------------
------------------------------ 1281 1,3
------------------------UGACCUAUGAAUUGACAGCCAGUaa-------------------------------
------------------------------ 86 1,3
------------------------UGACCUAUGAAUUGACAGCCAGUau-------------------------------
------------------------------ 66 1,3
------------------------UGACCUAUGAAUUGACAGCCAGUu--------------------------------
------------------------------ 2966 1,3
------------------------UGACCUAUGAAUUGACAGCCAGUuu-------------------------------
------------------------------ 231 1,3
------------------------UGACCUAUGAAUUGACAGCCAGUG--------------------------------
------------------------------ 40 1,4
------------------------UGACCUAUGAAUUGACAGCCAGUGu-------------------------------
------------------------------ 6 1,4
-------------------------GACCUAUGAAUUGACAGC-------------------------------------
------------------------------ 598 2,-1
-------------------------GACCUAUGAAUUGACAGCa------------------------------------
------------------------------ 7 2,-1
-------------------------GACCUAUGAAUUGACAGCaa-----------------------------------
------------------------------ 5 2,-1
-------------------------GACCUAUGAAUUGACAGCu------------------------------------
------------------------------ 2 2,-1
-------------------------GACCUAUGAAUUGACAGCC------------------------------------
------------------------------ 346 2,0
-------------------------GACCUAUGAAUUGACAGCCu-----------------------------------
------------------------------ 15 2,0
-------------------------GACCUAUGAAUUGACAGCCuu----------------------------------
------------------------------ 3 2,0
-------------------------GACCUAUGAAUUGACAGCCA-----------------------------------
------------------------------ 310 2,1
-------------------------GACCUAUGAAUUGACAGCCAa----------------------------------
------------------------------ 16 2,1
-------------------------GACCUAUGAAUUGACAGCCAaa---------------------------------
------------------------------ 12 2,1
-------------------------GACCUAUGAAUUGACAGCCAau---------------------------------

----------------------------- 11 2,1
-------------------------GACCUAUGAAUUGACAGCCAu----------------------------------
----------------------------- 142 2,1
-------------------------GACCUAUGAAUUGACAGCCAuu---------------------------------
----------------------------- 77 2,1
-------------------------GACCUAUGAAUUGACAGCCAG----------------------------------
----------------------------- 129 2,2
-------------------------GACCUAUGAAUUGACAGCCAGa---------------------------------
----------------------------- 31 2,2
-------------------------GACCUAUGAAUUGACAGCCAGau--------------------------------
----------------------------- 3   2,2
-------------------------GACCUAUGAAUUGACAGCCAGU---------------------------------
----------------------------- 174 2,3
-------------------------GACCUAUGAAUUGACAGCCAGUa--------------------------------
----------------------------- 17 2,3
-------------------------GACCUAUGAAUUGACAGCCAGUaa-------------------------------
----------------------------- 2   2,3
-------------------------GACCUAUGAAUUGACAGCCAGUau-------------------------------
----------------------------- 2   2,3
-------------------------GACCUAUGAAUUGACAGCCAGUu--------------------------------
----------------------------- 54 2,3
-------------------------GACCUAUGAAUUGACAGCCAGUuu-------------------------------
----------------------------- 11 2,3
---GGCUGCCAAUUCCAUA
GGUCAC----------------------- 3   -2,-2
---GGCUGCCAAUUCCAUA
GGUCACA---------------------- 4   -2,-1
--GCUGCCAAUUCCAUA
GGUCA------------------------ 3   -1,-3
---CUGCCAAUUCCAUA
GGUCA------------------------ 3   0,-3
---CUGCCAAUUCCAUA
GGUCAC----------------------- 3   0,-2
---CUGCCAAUUCCAUA
GGUCACA---------------------- 6   0,-1
---CUGCCAAUUCCAUA
GGUCACAu--------------------- 2   0,-1
---CUGCCAAUUCCAUA

GGUCACAG---------------------- 6   0,0

>hsa-mir-193a   5p:21-42;3p:55-76   SRX018959
cgaggaugggagcugagggcUGGGUCUUUGCGGGCGAGAUGAgggugucggaucAACUGGCCUACAAAGUCCCAGUucuc
ggcccccg
.......(((.(((((((((((((.(((((..(((.((.(((..........))).)).)))..))))).))))))))))
))).))).
--------------------UGGGUCUUUGCGGGCGAG--
--------   3   0,-4
--------------------UGGGUCUUUGCGGGCGAGAU--
--------   5   0,-2
--------------------UGGGUCUUUGCGGGCGAGAUa---------------------------------------
--------   14   0,-2
--------------------UGGGUCUUUGCGGGCGAGAUG---------------------------------------
--------   52   0,-1
--------------------UGGGUCUUUGCGGGCGAGAUGu--------------------------------------
--------   24   0,-1
--------------------UGGGUCUUUGCGGGCGAGAUGuu-------------------------------------
--------   2   0,-1
--------------------UGGGUCUUUGCGGGCGAGAUGA--------------------------------------
--------   168 0,0
--------------------UGGGUCUUUGCGGGCGAGAUGAa-------------------------------------
--------   6   0,0
--------------------UGGGUCUUUGCGGGCGAGAUGAu-------------------------------------
--------   13   0,0
--------------------UGGGUCUUUGCGGGCGAGAUGAG-------------------------------------
--------   2   0,1
---------------------GGGUCUUUGCGGGCGAGAUa---------------------------------------
--------   2   1,-2
---------------------GGGUCUUUGCGGGCGAGAUGA--------------------------------------
--------   7   1,0
--AACUGGCCUACAAAGUCC--------
--------   91   0,-4
--AACUGGCCUACAAAGUCCa-------
--------   2   0,-4
--AACUGGCCUACAAAGUCCC-------
--------   201 0,-3
--AACUGGCCUACAAAGUCCCA------

--------	2637	0,-2
--AACUGGCCUACAAAGUCCCAa-----
--------	3	0,-2
--AACUGGCCUACAAAGUCCCAaa----
--------	2	0,-2
--AACUGGCCUACAAAGUCCCAau----
--------	2	0,-2
--AACUGGCCUACAAAGUCCCAu-----
--------	19	0,-2
--AACUGGCCUACAAAGUCCCAuu----
--------	6	0,-2
--AACUGGCCUACAAAGUCCCAG-----
--------	79	0,-1
--AACUGGCCUACAAAGUCCCAGa----
--------	9	0,-1
--AACUGGCCUACAAAGUCCCAGau---
--------	2	0,-1
--AACUGGCCUACAAAGUCCCAGU----
--------	87	0,0
--AACUGGCCUACAAAGUCCCAGUa---
--------	11	0,0
---ACUGGCCUACAAAGUCCCA------
--------	14	1,-2

>hsa-mir-193b	mi:14-35;MA:51-72	SRX018959
guggucucagaauCGGGGUUUUGAGGGCGAGAUGAguuuauguuuuauccAACUGGCCCUCAAAGUCCCGCUuuuggggu
cau
(((..((((((.((((..(((((((((.((.((..(.........)..)).)).)))))))))..)))).)))))))..
)))
------------UCGGGGUUUUGAGGGCGAG---
--- 3	-1,-4
------------UCGGGGUUUUGAGGGCGAGAU---
--- 2	-1,-2
------------UCGGGGUUUUGAGGGCGAGAUGu---
--- 8	-1,-1
------------UCGGGGUUUUGAGGGCGAGAUGA---
--- 2	-1,0
-------------CGGGGUUUUGAGGGCGAG---

--- 44 0,-4

-------------CGGGGUUUUGAGGGCGAGA---

--- 6 0,-3

-------------CGGGGUUUUGAGGGCGAGAaa---

--- 7 0,-3

-------------CGGGGUUUUGAGGGCGAGAU--

--- 34 0,-2

-------------CGGGGUUUUGAGGGCGAGAUa---

--- 38 0,-2

-------------CGGGGUUUUGAGGGCGAGAUaa--

--- 3 0,-2

-------------CGGGGUUUUGAGGGCGAGAUau--

--- 4 0,-2

-------------CGGGGUUUUGAGGGCGAGAUu---

--- 10 0,-2

-------------CGGGGUUUUGAGGGCGAGAUuu--

--- 3 0,-2

-------------CGGGGUUUUGAGGGCGAGAUG---

--- 134 0,-1

-------------CGGGGUUUUGAGGGCGAGAUGu--

--- 129 0,-1

-------------CGGGGUUUUGAGGGCGAGAUGuu---

--- 9 0,-1

-------------CGGGGUUUUGAGGGCGAGAUGA--

--- 661 0,0

-------------CGGGGUUUUGAGGGCGAGAUGAa---

--- 24 0,0

-------------CGGGGUUUUGAGGGCGAGAUGAu---

--- 41 0,0

-------------CGGGGUUUUGAGGGCGAGAUGAG---

--- 2 0,1

--------------GGGGUUUUGAGGGCGAGAUG---

--- 2 1,-1

--------------GGGGUUUUGAGGGCGAGAUGu--

--- 7 1,-1

--------------GGGGUUUUGAGGGCGAGAUGA--

--- 19 1,0

--------------GGGGUUUUGAGGGCGAGAUGAu---

--- 4 1,0
---AACUGGCCCUCAAAGUCC------------
--- 72 0,-4
---AACUGGCCCUCAAAGUCCC-----------
--- 59 0,-3
---AACUGGCCCUCAAAGUCCCG----------
--- 8 0,-2
---AACUGGCCCUCAAAGUCCCGC---------
--- 34 0,-1
---AACUGGCCCUCAAAGUCCCGCau-------
--- 7 0,-1
---AACUGGCCCUCAAAGUCCCGCU--------
--- 71 0,0
---AACUGGCCCUCAAAGUCCCGCUa-------
--- 12 0,0
---AACUGGCCCUCAAAGUCCCGCUaa------
--- 3 0,0
---AACUGGCCCUCAAAGUCCCGCUau------
--- 8 0,0
---AACUGGCCCUCAAAGUCCCGCUU-------
--- 51 0,1
---AACUGGCCCUCAAAGUCCCGCUUa------
--- 2 0,1
---AACUGGCCCUCAAAGUCCCGCUUau-----
--- 3 0,1
---AACUGGCCCUCAAAGUCCCGCUUU------
--- 24 0,2
---AACUGGCCCUCAAAGUCCCGCUUUU-----
--- 4 0,3
--ACUGGCCCUCAAAGUCCC-----------
--- 2 1,-3
--ACUGGCCCUCAAAGUCCCGCUau------
--- 5 1,0
--ACUGGCCCUCAAAGUCCCGCUU-------
--- 7 1,1
--ACUGGCCCUCAAAGUCCCGCUUUU-----
--- 2 1,3

>hsa-mir-194-1 5p:15-36   SRX018959
augguguuaucaagUGUAACAGCAACUCCAUGUGGAcuguguaccaauuuccaguggagaugcuguuacuuuugaugguu
accaa
.(((((..((((((.(((((((((.((((((.((((..((......)).))))))))))).))))))))).))))))..).
)))).
--------------UGUAACAGCAACUCCAUG--
-----   11 0,-4
--------------UGUAACAGCAACUCCAUGa---
-----   2   0,-4
--------------UGUAACAGCAACUCCAUGU---
-----   11 0,-3
--------------UGUAACAGCAACUCCAUGUG--
-----   31 0,-2
--------------UGUAACAGCAACUCCAUGUGa---
-----   2   0,-2
--------------UGUAACAGCAACUCCAUGUGG---
-----   437 0,-1
--------------UGUAACAGCAACUCCAUGUGGu--
-----   7   0,-1
--------------UGUAACAGCAACUCCAUGUGGA--
-----   966 0,0
--------------UGUAACAGCAACUCCAUGUGGAu---
-----   131 0,0
--------------UGUAACAGCAACUCCAUGUGGAuu--
-----   5   0,0
--------------UGUAACAGCAACUCCAUGUGGAC---
-----   9   0,1
--------------UGUAACAGCAACUCCAUGUGGACa--
-----   3   0,1
---------------GUAACAGCAACUCCAUGUG--
-----   4   1,-2
---------------GUAACAGCAACUCCAUGUGG---
-----   5   1,-1
---------------GUAACAGCAACUCCAUGUGGA--
-----   4   1,0

>hsa-mir-194-2 MA:15-36;mi:51-72   SRX018959
ugguucccgcccccUGUAACAGCAACUCCAUGUGGAagugcccacugguuCCAGUGGGGCUGCUGUUAUCUGgggcgagg

gccag
((((((.((((((..(((((((((.((((((.((((...(((....))))))))))))).)))))))))..)))))).))
)))).
------------CCUGUAACAGCAACUCCAUGUGG---
-----   12 -2,-1
-------------CUGUAACAGCAACUCCAUGU---
-----   2   -1,-3
-------------CUGUAACAGCAACUCCAUGUGG---
-----   22 -1,-1
-------------CUGUAACAGCAACUCCAUGUGGA--
-----   36 -1,0
-------------CUGUAACAGCAACUCCAUGUGGAu---
-----   3   -1,0
-------------CUGUAACAGCAACUCCAUGUGGAA---
-----   8   -1,1
-------------CUGUAACAGCAACUCCAUGUGGAAu--
-----   2   -1,1
--------------UGUAACAGCAACUCCAUG--
-----   11 0,-4
--------------UGUAACAGCAACUCCAUGa---
-----   2   0,-4
--------------UGUAACAGCAACUCCAUGU---
-----   11 0,-3
--------------UGUAACAGCAACUCCAUGUG--
-----   31 0,-2
--------------UGUAACAGCAACUCCAUGUGa---
-----   2   0,-2
--------------UGUAACAGCAACUCCAUGUGG---
-----   437 0,-1
--------------UGUAACAGCAACUCCAUGUGGu--
-----   7   0,-1
--------------UGUAACAGCAACUCCAUGUGGA--
-----   966 0,0
--------------UGUAACAGCAACUCCAUGUGGAu---
-----   131 0,0
--------------UGUAACAGCAACUCCAUGUGGAuu--
-----   5   0,0
--------------UGUAACAGCAACUCCAUGUGGAA---

----- 2543 0,1

--------------UGUAACAGCAACUCCAUGUGGAAa--

----- 482 0,1

--------------UGUAACAGCAACUCCAUGUGGAAaa---

----- 20 0,1

--------------UGUAACAGCAACUCCAUGUGGAAau---

----- 14 0,1

--------------UGUAACAGCAACUCCAUGUGGAAu--

----- 144 0,1

--------------UGUAACAGCAACUCCAUGUGGAAuu---

----- 15 0,1

--------------UGUAACAGCAACUCCAUGUGGAAG--

----- 3 0,2

---------------GUAACAGCAACUCCAUGUG--

----- 4 1,-2

---------------GUAACAGCAACUCCAUGUGG---

----- 5 1,-1

---------------GUAACAGCAACUCCAUGUGGA--

----- 4 1,0

---------------GUAACAGCAACUCCAUGUGGAA---

----- 11 1,1

---------------GUAACAGCAACUCCAUGUGGAAa--

----- 3 1,1

---------------GUAACAGCAACUCCAUGUGGAAau---

----- 3 1,1

--CCAGUGGGGCUGCUGUUAU-----------

----- 5 0,-3

--CCAGUGGGGCUGCUGUUAUa----------

----- 24 0,-3

--CCAGUGGGGCUGCUGUUAUuu---------

----- 2 0,-3

--CCAGUGGGGCUGCUGUUAUC----------

----- 9 0,-2

--CCAGUGGGGCUGCUGUUAUCU---------

----- 126 0,-1

--CCAGUGGGGCUGCUGUUAUCUu--------

----- 2 0,-1

--CCAGUGGGGCUGCUGUUAUCUG--------

----- 9 0,0
--CCAGUGGGGCUGCUGUUAUCUGa-------
----- 3 0,0
--CCAGUGGGGCUGCUGUUAUCUGu-------
----- 8 0,0
---CAGUGGGGCUGCUGUUAU-----------
----- 2 1,-3
---CAGUGGGGCUGCUGUUAUa----------
----- 2 1,-3
---CAGUGGGGCUGCUGUUAUCU---------
----- 7 1,-1
---CAGUGGGGCUGCUGUUAUCUG--------
----- 2 1,0
---CAGUGGGGCUGCUGUUAUCUGGu------
----- 2 1,1

>hsa-mir-195 MA:15-35;mi:53-74 SRX018959
agcuucccuggcucUAGCAGCACAGAAAUAUUGGCacagggaagcgagucugCCAAUAUUGGCUGUGCUGCUCCaggcag
gguggug
.(((.(((((.((..((((((((((.((((((((((..............))))))))))..))))))))))..)).)))
)).))).
--------------UAGCAGCACAGAAAUAUU--
------- 2 0,-3
--------------UAGCAGCACAGAAAUAUUG---
------- 38 0,-2
--------------UAGCAGCACAGAAAUAUUGa--
------- 8 0,-2
--------------UAGCAGCACAGAAAUAUUGG--
------- 301 0,-1
--------------UAGCAGCACAGAAAUAUUGGa---
------- 9 0,-1
--------------UAGCAGCACAGAAAUAUUGGaa--
------- 8 0,-1
--------------UAGCAGCACAGAAAUAUUGGau--
------- 2 0,-1
--------------UAGCAGCACAGAAAUAUUGGC---
------- 27 0,0
--------------UAGCAGCACAGAAAUAUUGGCu--

------- 2 0,0
--------------UAGCAGCACAGAAAUAUUGGCA--- 734 0,1
--------------UAGCAGCACAGAAAUAUUGGCAa-- 412 0,1
--------------UAGCAGCACAGAAAUAUUGGCAaa--- 6 0,1
--------------UAGCAGCACAGAAAUAUUGGCAau--- 10 0,1
--------------UAGCAGCACAGAAAUAUUGGCAu-- 289 0,1
--------------UAGCAGCACAGAAAUAUUGGCAuu--- 41 0,1
---------------AGCAGCACAGAAAUAUUGG--- 4 1,-1
---------------AGCAGCACAGAAAUAUUGGCA--- 8 1,1
---------------AGCAGCACAGAAAUAUUGGCAa-- 5 1,1
---------------AGCAGCACAGAAAUAUUGGCAu-- 2 1,1

>hsa-mir-197 3p:48-69 SRX018959
ggcugugccggguagagagggcagugggagguaagagcucuucacccUUCACCACCUUCUCCACCCAGCauggcc
((((((((.((((.((((((((..((((..((((((....))).)))..))))..)))))))).)))).))))))))
--------CGGGUAGAGAGGGCAGUGGGAGG-- 2
---UUCACCACCUUCUCCACCCAG------- 4 0,-1
---UUCACCACCUUCUCCACCCAGC------ 2 0,0

>hsa-mir-199a-1 5p:6-28;3p:47-68 SRX018959
gccaaCCCAGUGUUCAGACUACCUGUUCaggaggcucucaauguguACAGUAGUCUGCACAUUGGUUAggc
(((...(((((((.((((((((.(((.((.(((...)))....)).))))))))))).)))))))...)))
-----CCCAGUGUUCAGACUACC-- 110 0,-5
-----CCCAGUGUUCAGACUACCU--- 1261 0,-4
-----CCCAGUGUUCAGACUACCUu-- 2 0,-4

-----CCCAGUGUUCAGACUACCUG-- 42 0,-3
-----CCCAGUGUUCAGACUACCUGa--- 2 0,-3
-----CCCAGUGUUCAGACUACCUGU--- 21 0,-2
-----CCCAGUGUUCAGACUACCUGUa-- 4 0,-2
-----CCCAGUGUUCAGACUACCUGUU-- 319 0,-1
-----CCCAGUGUUCAGACUACCUGUUa--------------------------------------- 26 0,-1
-----CCCAGUGUUCAGACUACCUGUUu--------------------------------------- 26 0,-1
-----CCCAGUGUUCAGACUACCUGUUC--------------------------------------- 101 0,0
-----CCCAGUGUUCAGACUACCUGUUCu-------------------------------------- 4 0,0
-----CCCAGUGUUCAGACUACCUGUUCA-------------------------------------- 4 0,1
------CCAGUGUUCAGACUACCU--- 16 1,-4
------CCAGUGUUCAGACUACCUGUU-- 4 1,-1
------CCAGUGUUCAGACUACCUGUUC--------------------------------------- 2 1,0
-------CAGUGUUCAGACUACCUGUU-- 2 2,-1
---UACAGUAGUCUGCACAUU-------- 32
-1,-5
---UACAGUAGUCUGCACAUUaa------ 2
-1,-5
---UACAGUAGUCUGCACAUUG------- 129
-1,-4
---UACAGUAGUCUGCACAUUGa------ 4
-1,-4
---UACAGUAGUCUGCACAUUGu------ 2
-1,-4
---UACAGUAGUCUGCACAUUGuu----- 3
-1,-4
---UACAGUAGUCUGCACAUUGG------ 284
-1,-3
---UACAGUAGUCUGCACAUUGGa----- 10
-1,-3
---UACAGUAGUCUGCACAUUGGaa---- 4
-1,-3
---UACAGUAGUCUGCACAUUGGau---- 21
-1,-3
---UACAGUAGUCUGCACAUUGGU----- 694
-1,-2
---UACAGUAGUCUGCACAUUGGUa---- 265
-1,-2

---UACAGUAGUCUGCACAUUGGUaa--- 212
-1,-2
---UACAGUAGUCUGCACAUUGGUau--- 189
-1,-2
---UACAGUAGUCUGCACAUUGGUU---- 7005
-1,-1
---UACAGUAGUCUGCACAUUGGUUu--- 1046
-1,-1
---UACAGUAGUCUGCACAUUGGUUuu-- 51
-1,-1
---UACAGUAGUCUGCACAUUGGUUA--- 1193
-1,0
---UACAGUAGUCUGCACAUUGGUUAa-- 135 -1,0
---UACAGUAGUCUGCACAUUGGUUAu-- 113 -1,0
---UACAGUAGUCUGCACAUUGGUUAG-- 3 -1,1
--ACAGUAGUCUGCACAUUG------- 588 0,-4
--ACAGUAGUCUGCACAUUGa------ 15 0,-4
--ACAGUAGUCUGCACAUUGaa----- 11 0,-4
--ACAGUAGUCUGCACAUUGu------ 12 0,-4
--ACAGUAGUCUGCACAUUGuu----- 10 0,-4
--ACAGUAGUCUGCACAUUGG------ 3077
0,-3
--ACAGUAGUCUGCACAUUGGa----- 19 0,-3
--ACAGUAGUCUGCACAUUGGaa---- 10 0,-3
--ACAGUAGUCUGCACAUUGGau---- 57 0,-3
--ACAGUAGUCUGCACAUUGGU----- 2777
0,-2
--ACAGUAGUCUGCACAUUGGUa---- 781 0,-2
--ACAGUAGUCUGCACAUUGGUaa--- 1375
0,-2
--ACAGUAGUCUGCACAUUGGUau--- 379 0,-2
--ACAGUAGUCUGCACAUUGGUU---- 45778
0,-1
--ACAGUAGUCUGCACAUUGGUUu--- 5563
0,-1
--ACAGUAGUCUGCACAUUGGUUuu-- 257 0,-1
--ACAGUAGUCUGCACAUUGGUUA--- 29339
0,0

--ACAGUAGUCUGCACAUUGGUUAa-- 14645 0,0
--ACAGUAGUCUGCACAUUGGUUAaa- 70 0,0
--ACAGUAGUCUGCACAUUGGUUAau- 54 0,0
--ACAGUAGUCUGCACAUUGGUUAu-- 2281 0,0
--ACAGUAGUCUGCACAUUGGUUAuu- 44 0,0
--ACAGUAGUCUGCACAUUGGUUAG-- 74 0,1
--ACAGUAGUCUGCACAUUGGUUAGu- 5 0,1
---CAGUAGUCUGCACAUUGG------ 395 1,-3
---CAGUAGUCUGCACAUUGGaa---- 2 1,-3
---CAGUAGUCUGCACAUUGGU----- 279 1,-2
---CAGUAGUCUGCACAUUGGUa---- 86 1,-2
---CAGUAGUCUGCACAUUGGUaa--- 160 1,-2
---CAGUAGUCUGCACAUUGGUau--- 20 1,-2
---CAGUAGUCUGCACAUUGGUU---- 3667 1,-1
---CAGUAGUCUGCACAUUGGUUu--- 637 1,-1
---CAGUAGUCUGCACAUUGGUUuu-- 38 1,-1
---CAGUAGUCUGCACAUUGGUUA--- 5913 1,0
---CAGUAGUCUGCACAUUGGUUAa-- 2753 1,0
---CAGUAGUCUGCACAUUGGUUAaa- 71 1,0
---CAGUAGUCUGCACAUUGGUUAau- 12 1,0
---CAGUAGUCUGCACAUUGGUUAu-- 521 1,0
---CAGUAGUCUGCACAUUGGUUAG-- 14 1,1
---CAGUAGUCUGCACAUUGGUUAGu- 2 1,1
--AGUAGUCUGCACAUUGGU----- 9 2,-2
--AGUAGUCUGCACAUUGGUa---- 3 2,-2
--AGUAGUCUGCACAUUGGUaa--- 3 2,-2
--AGUAGUCUGCACAUUGGUau--- 2 2,-2
--AGUAGUCUGCACAUUGGUU---- 89 2,-1
--AGUAGUCUGCACAUUGGUUu--- 6 2,-1
--AGUAGUCUGCACAUUGGUUA--- 75 2,0
--AGUAGUCUGCACAUUGGUUAa-- 57 2,0
--AGUAGUCUGCACAUUGGUUAu-- 9 2,0

>hsa-mir-199a-2 5p:31-53;3p:70-91   SRX018959
aggaagcuucuggagauccugcuccgucgcCCCAGUGUUCAGACUACCUGUUCaggacaaugccguuguACAGUAGUCUG
CACAUUGGUUAgacugggcaagggagagca
.....(((.((.....(((((((.(((...((((((.((((((((.(((...((......)).....)))))))))))))
.)))))))...))).)))).))))))))).
------------------------------CCCAGUGUUCAGACUACC--------------------------------
------------------------------ 110 0,-5
------------------------------CCCAGUGUUCAGACUACCU-------------------------------
------------------------------ 1261   0,-4
------------------------------CCCAGUGUUCAGACUACCUu------------------------------
------------------------------ 2   0,-4
------------------------------CCCAGUGUUCAGACUACCUG------------------------------
------------------------------ 42   0,-3
------------------------------CCCAGUGUUCAGACUACCUGa-----------------------------
------------------------------ 2   0,-3
------------------------------CCCAGUGUUCAGACUACCUGU-----------------------------
------------------------------ 21   0,-2
------------------------------CCCAGUGUUCAGACUACCUGUa----------------------------
------------------------------ 4   0,-2
------------------------------CCCAGUGUUCAGACUACCUGUU----------------------------
------------------------------ 319 0,-1
------------------------------CCCAGUGUUCAGACUACCUGUUa---------------------------
------------------------------ 26   0,-1
------------------------------CCCAGUGUUCAGACUACCUGUUu---------------------------
------------------------------ 26   0,-1
------------------------------CCCAGUGUUCAGACUACCUGUUC---------------------------
------------------------------ 101 0,0
------------------------------CCCAGUGUUCAGACUACCUGUUCu--------------------------
------------------------------ 4   0,0
------------------------------CCCAGUGUUCAGACUACCUGUUCA--------------------------
------------------------------ 4   0,1
-------------------------------CCAGUGUUCAGACUACCU-------------------------------
------------------------------ 16   1,-4
-------------------------------CCAGUGUUCAGACUACCUGUU----------------------------
------------------------------ 4   1,-1
-------------------------------CCAGUGUUCAGACUACCUGUUC---------------------------
------------------------------ 2   1,0
--------------------------------CAGUGUUCAGACUACCUGUU----------------------------

----------------------------- 2 2,-1

---UACAGUAGUCUG

CACAUU----------------------- 32 -1,-5

---UACAGUAGUCUG

CACAUUaa--------------------- 2 -1,-5

---UACAGUAGUCUG

CACAUUG---------------------- 129 -1,-4

---UACAGUAGUCUG

CACAUUGa--------------------- 4 -1,-4

---UACAGUAGUCUG

CACAUUGu--------------------- 2 -1,-4

---UACAGUAGUCUG

CACAUUGuu-------------------- 3 -1,-4

---UACAGUAGUCUG

CACAUUGG--------------------- 284 -1,-3

---UACAGUAGUCUG

CACAUUGGa-------------------- 10 -1,-3

---UACAGUAGUCUG

CACAUUGGaa------------------- 4 -1,-3

---UACAGUAGUCUG

CACAUUGGau------------------- 21 -1,-3

---UACAGUAGUCUG

CACAUUGGU-------------------- 694 -1,-2

---UACAGUAGUCUG

CACAUUGGUa------------------- 265 -1,-2

---UACAGUAGUCUG

CACAUUGGUaa------------------ 212 -1,-2

---UACAGUAGUCUG

CACAUUGGUau------------------ 189 -1,-2

---UACAGUAGUCUG

CACAUUGGUU------------------- 7005 -1,-1

---UACAGUAGUCUG

CACAUUGGUUu------------------ 1046 -1,-1

---UACAGUAGUCUG

CACAUUGGUUuu----------------- 51 -1,-1

---UACAGUAGUCUG

CACAUUGGUUA------------------ 1193 -1,0

---UACAGUAGUCUG

CACAUUGGUUAa----------------- 135 -1,0
---UACAGUAGUCUG
CACAUUGGUUAu----------------- 113 -1,0
---UACAGUAGUCUG
CACAUUGGUUAG----------------- 3   -1,1
--ACAGUAGUCUG
CACAUUG---------------------- 588 0,-4
--ACAGUAGUCUG
CACAUUGa--------------------- 15   0,-4
--ACAGUAGUCUG
CACAUUGaa-------------------- 11   0,-4
--ACAGUAGUCUG
CACAUUGu--------------------- 12   0,-4
--ACAGUAGUCUG
CACAUUGuu-------------------- 10   0,-4
--ACAGUAGUCUG
CACAUUGG--------------------- 3077   0,-3
--ACAGUAGUCUG
CACAUUGGa-------------------- 19   0,-3
--ACAGUAGUCUG
CACAUUGGaa------------------- 10   0,-3
--ACAGUAGUCUG
CACAUUGGau------------------- 57   0,-3
--ACAGUAGUCUG
CACAUUGGU-------------------- 2777   0,-2
--ACAGUAGUCUG
CACAUUGGUa------------------- 781 0,-2
--ACAGUAGUCUG
CACAUUGGUaa------------------ 1375   0,-2
--ACAGUAGUCUG
CACAUUGGUau------------------ 379 0,-2
--ACAGUAGUCUG
CACAUUGGUU------------------- 45778   0,-1
--ACAGUAGUCUG
CACAUUGGUUu------------------ 5563   0,-1
--ACAGUAGUCUG
CACAUUGGUUuu----------------- 257 0,-1
--ACAGUAGUCUG

CACAUUGGUUA------------------- 29339 0,0

---ACAGUAGUCUG

CACAUUGGUUAa------------------ 14645 0,0

---ACAGUAGUCUG

CACAUUGGUUAaa----------------- 70 0,0

---ACAGUAGUCUG

CACAUUGGUUAau----------------- 54 0,0

---ACAGUAGUCUG

CACAUUGGUUAu------------------ 2281 0,0

---ACAGUAGUCUG

CACAUUGGUUAuu----------------- 44 0,0

---ACAGUAGUCUG

CACAUUGGUUAG------------------ 74 0,1

---ACAGUAGUCUG

CACAUUGGUUAGu----------------- 5 0,1

--CAGUAGUCUG

CACAUUGG---------------------- 395 1,-3

--CAGUAGUCUG

CACAUUGGaa-------------------- 2 1,-3

--CAGUAGUCUG

CACAUUGGU--------------------- 279 1,-2

--CAGUAGUCUG

CACAUUGGUa-------------------- 86 1,-2

--CAGUAGUCUG

CACAUUGGUaa------------------- 160 1,-2

--CAGUAGUCUG

CACAUUGGUau------------------- 20 1,-2

--CAGUAGUCUG

CACAUUGGUU-------------------- 3667 1,-1

--CAGUAGUCUG

CACAUUGGUUu------------------- 637 1,-1

--CAGUAGUCUG

CACAUUGGUUuu------------------ 38 1,-1

--CAGUAGUCUG

CACAUUGGUUA------------------- 5913 1,0

--CAGUAGUCUG

CACAUUGGUUAa------------------ 2753 1,0

--CAGUAGUCUG

CACAUUGGUUAaa----------------- 71 1,0
--CAGUAGUCUG
CACAUUGGUUAau----------------- 12 1,0
--CAGUAGUCUG
CACAUUGGUUAu------------------ 521 1,0
--CAGUAGUCUG
CACAUUGGUUAG------------------ 14 1,1
--CAGUAGUCUG
CACAUUGGUUAGu----------------- 2   1,1
---AGUAGUCUG
CACAUUGGU--------------------- 9   2,-2
---AGUAGUCUG
CACAUUGGUa-------------------- 3   2,-2
---AGUAGUCUG
CACAUUGGUaa------------------- 3   2,-2
---AGUAGUCUG
CACAUUGGUau------------------- 2   2,-2
---AGUAGUCUG
CACAUUGGUU-------------------- 89 2,-1
---AGUAGUCUG
CACAUUGGUUu------------------- 6   2,-1
---AGUAGUCUG
CACAUUGGUUA------------------- 75 2,0
---AGUAGUCUG
CACAUUGGUUAa------------------ 57 2,0
---AGUAGUCUG
CACAUUGGUUAu------------------ 9   2,0

>hsa-mir-199b   5p:26-48;3p:65-86   SRX018959
ccagaggacaccuccacuccgucuaCCCAGUGUUUAGACUAUCUGUUCaggacucccaaauuguACAGUAGUCUGCACAU
UGGUUAggcugggcuggguuagacccucgg
...((((.....((((..(((((((.(((((((.(((((((.((((.((((....))....)).))))))))))).))))
))).))))).))..))))......))))..
-------------------------CCCAGUGUUUAGACUAUC-------------------------------------
------------------------------ 18 0,-5
-------------------------CCCAGUGUUUAGACUAUCU------------------------------------
------------------------------ 129 0,-4
-------------------------CCCAGUGUUUAGACUAUCUG-----------------------------------

----------------------------- 2   0,-3
-------------------------CCCAGUGUUUAGACUAUCUGUU---------------------------------
----------------------------- 5   0,-1
-------------------------CCCAGUGUUUAGACUAUCUGUUC--------------------------------
----------------------------- 2   0,0
--------------------------CCAGUGUUUAGACUAUCU------------------------------------
----------------------------- 3   1,-4
--UACAGUAGUCUGCACAU
U---------------------------- 32   -1,-5
--UACAGUAGUCUGCACAU
Uaa-------------------------- 2   -1,-5
--UACAGUAGUCUGCACAU
UG--------------------------- 129 -1,-4
--UACAGUAGUCUGCACAU
UGa-------------------------- 4   -1,-4
--UACAGUAGUCUGCACAU
UGu-------------------------- 2   -1,-4
--UACAGUAGUCUGCACAU
UGuu------------------------- 3   -1,-4
--UACAGUAGUCUGCACAU
UGG-------------------------- 284 -1,-3
--UACAGUAGUCUGCACAU
UGGa------------------------- 10   -1,-3
--UACAGUAGUCUGCACAU
UGGaa------------------------ 4   -1,-3
--UACAGUAGUCUGCACAU
UGGau------------------------ 21   -1,-3
--UACAGUAGUCUGCACAU
UGGU------------------------- 694 -1,-2
--UACAGUAGUCUGCACAU
UGGUa------------------------ 265 -1,-2
--UACAGUAGUCUGCACAU
UGGUaa----------------------- 212 -1,-2
--UACAGUAGUCUGCACAU
UGGUau----------------------- 189 -1,-2
--UACAGUAGUCUGCACAU
UGGUU------------------------ 7005   -1,-1
--UACAGUAGUCUGCACAU

UGGUUu------------------------ 1046 -1,-1

--UACAGUAGUCUGCACAU

UGGUUuu----------------------- 51 -1,-1

--UACAGUAGUCUGCACAU

UGGUUA------------------------ 1193 -1,0

--UACAGUAGUCUGCACAU

UGGUUAa----------------------- 135 -1,0

--UACAGUAGUCUGCACAU

UGGUUAu----------------------- 113 -1,0

--UACAGUAGUCUGCACAU

UGGUUAG----------------------- 3 -1,1

---ACAGUAGUCUGCACAU

UG---------------------------- 588 0,-4

---ACAGUAGUCUGCACAU

UGa--------------------------- 15 0,-4

---ACAGUAGUCUGCACAU

UGaa-------------------------- 11 0,-4

---ACAGUAGUCUGCACAU

UGu--------------------------- 12 0,-4

---ACAGUAGUCUGCACAU

UGuu-------------------------- 10 0,-4

---ACAGUAGUCUGCACAU

UGG--------------------------- 3077 0,-3

---ACAGUAGUCUGCACAU

UGGa-------------------------- 19 0,-3

---ACAGUAGUCUGCACAU

UGGaa------------------------- 10 0,-3

---ACAGUAGUCUGCACAU

UGGau------------------------- 57 0,-3

---ACAGUAGUCUGCACAU

UGGU-------------------------- 2777 0,-2

---ACAGUAGUCUGCACAU

UGGUa------------------------- 781 0,-2

---ACAGUAGUCUGCACAU

UGGUaa------------------------ 1375 0,-2

---ACAGUAGUCUGCACAU

UGGUau------------------------ 379 0,-2

---ACAGUAGUCUGCACAU

UGGUU------------------------- 45778 0,-1

---ACAGUAGUCUGCACAU

UGGUUu------------------------ 5563 0,-1

---ACAGUAGUCUGCACAU

UGGUUuu----------------------- 257 0,-1

---ACAGUAGUCUGCACAU

UGGUUA------------------------ 29339 0,0

---ACAGUAGUCUGCACAU

UGGUUAa----------------------- 14645 0,0

---ACAGUAGUCUGCACAU

UGGUUAaa---------------------- 70 0,0

---ACAGUAGUCUGCACAU

UGGUUAau---------------------- 54 0,0

---ACAGUAGUCUGCACAU

UGGUUAu----------------------- 2281 0,0

---ACAGUAGUCUGCACAU

UGGUUAuu---------------------- 44 0,0

---ACAGUAGUCUGCACAU

UGGUUAG----------------------- 74 0,1

---ACAGUAGUCUGCACAU

UGGUUAGu---------------------- 5 0,1

--CAGUAGUCUGCACAU

UGG--------------------------- 395 1,-3

--CAGUAGUCUGCACAU

UGGaa------------------------- 2 1,-3

--CAGUAGUCUGCACAU

UGGU-------------------------- 279 1,-2

--CAGUAGUCUGCACAU

UGGUa------------------------- 86 1,-2

--CAGUAGUCUGCACAU

UGGUaa------------------------ 160 1,-2

--CAGUAGUCUGCACAU

UGGUau------------------------ 20 1,-2

--CAGUAGUCUGCACAU

UGGUU------------------------- 3667 1,-1

--CAGUAGUCUGCACAU

UGGUUu------------------------ 637 1,-1

--CAGUAGUCUGCACAU

UGGUUuu----------------------- 38 1,-1
---CAGUAGUCUGCACAU
UGGUUA------------------------ 5913   1,0
---CAGUAGUCUGCACAU
UGGUUAa----------------------- 2753   1,0
---CAGUAGUCUGCACAU
UGGUUAaa---------------------- 71 1,0
---CAGUAGUCUGCACAU
UGGUUAau---------------------- 12 1,0
---CAGUAGUCUGCACAU
UGGUUAu----------------------- 521 1,0
---CAGUAGUCUGCACAU
UGGUUAG----------------------- 14 1,1
---CAGUAGUCUGCACAU
UGGUUAGu---------------------- 2   1,1
--AGUAGUCUGCACAU
UGGU-------------------------- 9   2,-2
--AGUAGUCUGCACAU
UGGUa------------------------- 3   2,-2
--AGUAGUCUGCACAU
UGGUaa------------------------ 3   2,-2
--AGUAGUCUGCACAU
UGGUau------------------------ 2   2,-2
--AGUAGUCUGCACAU
UGGUU------------------------- 89 2,-1
--AGUAGUCUGCACAU
UGGUUu------------------------ 6   2,-1
--AGUAGUCUGCACAU
UGGUUA------------------------ 75 2,0
--AGUAGUCUGCACAU
UGGUUAa----------------------- 57 2,0
--AGUAGUCUGCACAU
UGGUUAu----------------------- 9   2,0

>hsa-mir-19a   mi:14-35;MA:49-71   SRX018959
gcaguccucuguuAGUUUUGCAUAGUUGCACUACAagaagaauguaguUGUGCAAAUCUAUGCAAAACUGAugguggccu
gc
((((.((.(((((((((((((((((((((((((((.......))))...))))))..))))))))))))))))).)).))

))
-------------AGUUUUGCAUAGUUGCACUA---
-- 3 0,-2
--UUGUGCAAAUCUAUGCAAAACU----------
-- 4 -1,-2
---UGUGCAAAUCUAUGCAAAAC-----------
-- 2 0,-3
---UGUGCAAAUCUAUGCAAAACU----------
-- 7 0,-2
---UGUGCAAAUCUAUGCAAAACUGA--------
-- 2 0,0

>hsa-mir-19b-1 mi:16-38;MA:54-76 SRX018959
cacuguucuaugguuAGUUUUGCAGGUUUGCAUCCAGCugugugauauucugcUGUGCAAAUCCAUGCAAAACUGAcugu
gguagug
(((((..(((((((((((((((((((((((((..((((.............)))))))))).)).)))))))))))))))
)))))))
---------------AGUUUUGCAGGUUUGCAUCCA--
------- 3 0,-2
---UGUGCAAAUCCAUGCAAAA--------
------- 2 0,-4
---UGUGCAAAUCCAUGCAAAAC-------
------- 4 0,-3
---UGUGCAAAUCCAUGCAAAACU------
------- 118 0,-2
---UGUGCAAAUCCAUGCAAAACUG-----
------- 21 0,-1
---UGUGCAAAUCCAUGCAAAACUGA----
------- 8 0,0
--GUGCAAAUCCAUGCAAAACU------
------- 2 1,-2

>hsa-mir-19b-2 mi:19-40;MA:62-84 SRX018959
acauugcuacuuacaauuAGUUUUGCAGGUUUGCAUUUCAgcguauauauguauauguggcUGUGCAAAUCCAUGCAAAA
CUGAuugugauaaugu
((((((....((((((((((((((((((((((((((....(((((((....)))))))....))))))).)).)))))))
))))))))))))))))
---UGUGCAAAUCCAUGCAAAA

---------------- 2 0,-4
---UGUGCAAAUCCAUGCAAAA
C--------------- 4 0,-3
---UGUGCAAAUCCAUGCAAAA
CU-------------- 118 0,-2
---UGUGCAAAUCCAUGCAAAA
CUG------------- 21 0,-1
---UGUGCAAAUCCAUGCAAAA
CUGA------------ 8 0,0
--GUGCAAAUCCAUGCAAAA
CU-------------- 2 1,-2

>hsa-mir-200a mi:16-37;MA:54-75 SRX018959
ccgggccccugugagCAUCUUACCGGACAGUGCUGGAuuucccagcuugacucUAACACUGUCUGGUAACGAUGUucaaa
ggugacccgc
.((((.(((.((((((((((((((((((((((((.....))))).........))))))))))).)))))))).)
)).).))))).
---UAACACUGUCUGGUAACG---------
---------- 2 0,-4
---UAACACUGUCUGGUAACGA--------
---------- 19 0,-3
---UAACACUGUCUGGUAACGAU-------
---------- 103 0,-2
---UAACACUGUCUGGUAACGAUa------
---------- 6 0,-2
---UAACACUGUCUGGUAACGAUG------
---------- 32 0,-1
---UAACACUGUCUGGUAACGAUGa-----
---------- 6 0,-1
---UAACACUGUCUGGUAACGAUGU-----
---------- 40 0,0
---UAACACUGUCUGGUAACGAUGUa----
---------- 8 0,0
---UAACACUGUCUGGUAACGAUGUaa---
---------- 2 0,0
---UAACACUGUCUGGUAACGAUGUU----
---------- 114 0,1
---UAACACUGUCUGGUAACGAUGUUa---

---------- 9 0,1
--UAACACUGUCUGGUAACGAUGUUu---
---------- 116 0,1
---AACACUGUCUGGUAACGAUG------
---------- 2 1,-1
---AACACUGUCUGGUAACGAUGU-----
---------- 2 1,0
---AACACUGUCUGGUAACGAUGUU----
---------- 4 1,1
---AACACUGUCUGGUAACGAUGUUa---
---------- 5 1,1
---AACACUGUCUGGUAACGAUGUUu---
---------- 14 1,1

>hsa-mir-200b mi:21-42;MA:57-78 SRX018959
ccagcucgggcagccguggcCAUCUUACUGGGCAGCAUUGGAuggagucaggucucUAAUACUGCCUGGUAAUGAUGAcg
gcggagcccugcacg
...((..((((..((((.(.((((((((..(((((.((((((.((.......)))))))).)))))..)))).)))).).
)))).))))).))...
--------------------CAUCUUACUGGGCAGCAUa--
--------------- 3 0,-4
--------------------CAUCUUACUGGGCAGCAUU--
--------------- 46 0,-3
--------------------CAUCUUACUGGGCAGCAUUG---
--------------- 11 0,-2
--------------------CAUCUUACUGGGCAGCAUUGG--
--------------- 26 0,-1
--------------------CAUCUUACUGGGCAGCAUUGGA---------------------------------------
--------------- 12 0,0
--------------------CAUCUUACUGGGCAGCAUUGGAa--------------------------------------
--------------- 11 0,0
--------------------CAUCUUACUGGGCAGCAUUGGAaa-------------------------------------
--------------- 3 0,0
--------------------CAUCUUACUGGGCAGCAUUGGAau-------------------------------------
--------------- 2 0,0
---------------------AUCUUACUGGGCAGCAUU--
--------------- 5 1,-3
---------------------AUCUUACUGGGCAGCAUUGG--

--------------------AUCUUACUGGGCAGCAUUGGA-------------------------------------
-------------- 5 1,-1
--------------------AUCUUACUGGGCAGCAUUGGAa------------------------------------
-------------- 2 1,0
---CUAAUACUGCCUGGUAAUGAUGACa
-------------- 7 1,0
--UAAUACUGCCUGGUAAUG------
-------------- 2 -1,1
--UAAUACUGCCUGGUAAUGA-----
-------------- 2 0,-4
--UAAUACUGCCUGGUAAUGAU----
-------------- 17 0,-3
--UAAUACUGCCUGGUAAUGAUa---
-------------- 24 0,-2
--UAAUACUGCCUGGUAAUGAUG---
-------------- 3 0,-2
--UAAUACUGCCUGGUAAUGAUGu--
-------------- 26 0,-1
--UAAUACUGCCUGGUAAUGAUGA--
-------------- 9 0,-1
--UAAUACUGCCUGGUAAUGAUGAa-
-------------- 133 0,0
--UAAUACUGCCUGGUAAUGAUGAu-
-------------- 32 0,0
--UAAUACUGCCUGGUAAUGAUGAC-
-------------- 2 0,0
--UAAUACUGCCUGGUAAUGAUGACa
-------------- 103 0,1
--UAAUACUGCCUGGUAAUGAUGACa
u------------- 30 0,1
--UAAUACUGCCUGGUAAUGAUGACu
-------------- 4 0,1
--UAAUACUGCCUGGUAAUGAUGACu
u------------- 59 0,1
--UAAUACUGCCUGGUAAUGAUGACG
-------------- 2 0,1
---AAUACUGCCUGGUAAUGAUGA--
-------------- 2 0,2

--------------- 3 1,0
---AAUACUGCCUGGUAAUGAUGAC-
--------------- 12 1,1
---AAUACUGCCUGGUAAUGAUGACa
--------------- 8 1,1
---AAUACUGCCUGGUAAUGAUGACu
--------------- 18 1,1
--AUACUGCCUGGUAAUGAUGA--
--------------- 6 2,0
--AUACUGCCUGGUAAUGAUGAa-
--------------- 2 2,0
--AUACUGCCUGGUAAUGAUGAC-
--------------- 43 2,1
--AUACUGCCUGGUAAUGAUGACa
u-------------- 4 2,1
--AUACUGCCUGGUAAUGAUGACu
--------------- 69 2,1
--AUACUGCCUGGUAAUGAUGACu
u-------------- 13 2,1
--AUACUGCCUGGUAAUGAUGACG
--------------- 2 2,2
--AUACUGCCUGGUAAUGAUGACG
G-------------- 3 2,3

>hsa-mir-200c mi:5-26;MA:44-66 SRX018959
cccuCGUCUUACCCAGCAGUGUUUGGgugcgguugggagucucUAAUACUGCCGGGUAAUGAUGGAgg
((..((((((((((.((((((((.(((.((........))))).)))))))).)))))).))))..))
---UAAUACUGCCGGGUAAUGAUa---- 2 0,-3
---UAAUACUGCCGGGUAAUGAUGG--- 8 0,-1

>hsa-mir-202 mi:28-49;MA:64-83 SRX018959
cgccucagagccgcccgccguuccuuuUUCCUAUGCAUAUACUUCUUUGaggaucuggccuaaAGAGGUAUAGGGCAUGG
GAAaacggggcggucggguccuccccagcg
(((....(((..((((((((((((.(((((((((((.(((((((((((.(((......))))))))))))))..))))))
))))).))))))).))))).)))....)))
---------------------------UUCCUAUGCAUAUACUUCUUU--------------------------------
------------------------------ 6 0,-1
---AGAGGUAUAGGGCAUGG

GAAA-------------------------- 2 0,1

>hsa-mir-203 3p:65-86 SRX018959
guguuggggacucgcgcgcuggguccagugguucuuaacaguucaacaguucuguagcgcaauuGUGAAAUGUUUAGGAC
CACUAGacccggcgggcgcggcgacagcga
.(((((..(.(.(((.(((((((((.(((((((((.((((.((((.((((((......).))))))))).)))).)))))
)))).))))))))).))).).)..))))).
--UUGUGAAAUGUUUAGGAC
CACU-------------------------- 2 -2,-2
---UGUGAAAUGUUUAGGAC
CACU-------------------------- 2 -1,-2
---UGUGAAAUGUUUAGGAC
CACUA------------------------- 2 -1,-1
---UGUGAAAUGUUUAGGAC
CACUAG------------------------ 2 -1,0
--GUGAAAUGUUUAGGAC
CA---------------------------- 4 0,-4
--GUGAAAUGUUUAGGAC
CACU-------------------------- 19 0,-2
--GUGAAAUGUUUAGGAC
CACUA------------------------- 49 0,-1
--GUGAAAUGUUUAGGAC
CACUAu------------------------ 13 0,-1
--GUGAAAUGUUUAGGAC
CACUAuu----------------------- 4 0,-1
--GUGAAAUGUUUAGGAC
CACUAG------------------------ 157 0,0
--GUGAAAUGUUUAGGAC
CACUAGu----------------------- 22 0,0
--GUGAAAUGUUUAGGAC
CACUAGA----------------------- 18 0,1
---UGAAAUGUUUAGGAC
CAC--------------------------- 2 1,-3
---UGAAAUGUUUAGGAC
CACUA------------------------- 4 1,-1
---UGAAAUGUUUAGGAC
CACUAG------------------------ 36 1,0
---UGAAAUGUUUAGGAC

CACUAGu----------------------- 16 1,0
---UGAAAUGUUUAGGAC
CACUAGuu---------------------- 4 1,0
---UGAAAUGUUUAGGAC
CACUAGA----------------------- 4 1,1

>hsa-mir-204 5p:33-54 SRX018959
ggcuacagucuuucuucaugugacucguggacUUCCCUUUGUCAUCCUAUGCCUgagaauauaugaaggaggcugggaag
gcaaagggacguucaauugucaucacuggc
...............((((((((...(((((.(((((((((((.(((((.((((...............))))))))).)
))))))))).)))))...)))).)).))).
--------------------------------UUCCCUUUGUCAUCCUAUGCC---------------------------
------------------------------ 4 0,-1
--------------------------------UUCCCUUUGUCAUCCUAUGCCU--------------------------
------------------------------ 14 0,0
---GCUGGGAAG
GCAAAGGGA--------------------- 14
---GCUGGGAAG
GCAAAGGGAC-------------------- 2
---GCUGGGAAG
GCAAAGGGACG------------------- 2
---GCUGGGAAG
GCAAAGGGACGU------------------ 17
---GCUGGGAAG
GCAAAGGGACGUa----------------- 2
---GCUGGGAAG
GCAAAGGGACGUau---------------- 2

>hsa-mir-206 3p:53-74 SRX018959
ugcuucccgaggccacaugcuucuuuauauccccauauggauuacuuugcuaUGGAAUGUAAGGAAGUGUGUGGuuucgg
caagug
.((((.((((((((((((((((((((((((..(((((.(.(......).)))))).))))))))))))))))))))))))
.)))).
--UGGAAUGUAAGGAAGUGUGU--------
------ 2 0,-2
--UGGAAUGUAAGGAAGUGUGUG-------
------ 2 0,-1
--UGGAAUGUAAGGAAGUGUGUGu------

------ 2 0,-1

---UGGAAUGUAAGGAAGUGUGUGG------

------ 19 0,0

>hsa-mir-20a MA:8-30;mi:44-65 SRX018959

guagcacUAAAGUGCUUAUAGUGCAGGUAGuguuuaguuaucuACUGCAUUAUGAGCACUUAAAGuacugc

((((.(((.(((((((((((((((((.(((((.......)).))))))))))))))))))))..))).))))

-------UAAAGUGCUUAUAGUGCA--- 12 0,-5

-------UAAAGUGCUUAUAGUGCAG-- 162 0,-4

-------UAAAGUGCUUAUAGUGCAGG--- 65 0,-3

-------UAAAGUGCUUAUAGUGCAGGU-- 81 0,-2

-------UAAAGUGCUUAUAGUGCAGGUA--- 171 0,-1

-------UAAAGUGCUUAUAGUGCAGGUAa-- 42 0,-1

-------UAAAGUGCUUAUAGUGCAGGUAu-- 72 0,-1

-------UAAAGUGCUUAUAGUGCAGGUAG-- 64 0,0

-------UAAAGUGCUUAUAGUGCAGGUAGa--- 4 0,0

-------UAAAGUGCUUAUAGUGCAGGUAGU--- 6 0,1

--------AAAGUGCUUAUAGUGCAG-- 2 1,-4

--------AAAGUGCUUAUAGUGCAGGUA--- 2 1,-1

---------AAGUGCUUAUAGUGCAGGU-- 2 2,-2

--ACUGCAUUAUGAGCACUUAA-------- 3 0,-2

--ACUGCAUUAUGAGCACUUAAAG------ 3 0,0

--ACUGCAUUAUGAGCACUUAAAGU----- 8 0,1

---CUGCAUUAUGAGCACUUAAAGU----- 2 1,1

>hsa-mir-20b MA:6-28;mi:44-65 SRX018959

aguacCAAAGUGCUCAUAGUGCAGGUAGuuuuggcaugacucuACUGUAGUAUGGGCACUUCCAGuacu

(((((..(((((((((((.(((((..((((.......))))...))))).)))))))))))...)))))

---ACUGUAGUAUGGGCACUUCC------ 2 0,-2

>hsa-mir-21 MA:8-29;mi:46-66 SRX018959

ugucgggUAGCUUAUCAGACUGAUGUUGAcuguugaaucucauggCAACACCAGUCGAUGGGCUGUcugaca

.(((((..((((((((.(((((.(((((.((((.(.....)))))))))).)))))))))))))..))))).

------GUAGCUUAUCAGACUGAUGU-- 2

-1,-3

------GUAGCUUAUCAGACUGAUGUU--- 23

-1,-2

------GUAGCUUAUCAGACUGAUGUUG-- 36

-1,-1

------GUAGCUUAUCAGACUGAUGUUGA-- 31

-1,0

------GUAGCUUAUCAGACUGAUGUUGAC--- 5

-1,1

------GUAGCUUAUCAGACUGAUGUUGACa-- 3

-1,1

------GUAGCUUAUCAGACUGAUGUUGACU-- 8

-1,2

-------UAGCUUAUCAGACUGAUG-- 1304

0,-4

-------UAGCUUAUCAGACUGAUGau-- 7

0,-4

-------UAGCUUAUCAGACUGAUGU--- 4972

0,-3

-------UAGCUUAUCAGACUGAUGUa-- 26

0,-3

-------UAGCUUAUCAGACUGAUGUaa--- 25

0,-3

-------UAGCUUAUCAGACUGAUGUU--

36742 0,-2

-------UAGCUUAUCAGACUGAUGUUa--- 134

0,-2

-------UAGCUUAUCAGACUGAUGUUaa-- 10

0,-2

-------UAGCUUAUCAGACUGAUGUUu--- 42

0,-2

-------UAGCUUAUCAGACUGAUGUUG---

19839 0,-1

-------UAGCUUAUCAGACUGAUGUUGu-- 52

0,-1

-------UAGCUUAUCAGACUGAUGUUGA--

14122 0,0

-------UAGCUUAUCAGACUGAUGUUGAa--- 747

0,0

-------UAGCUUAUCAGACUGAUGUUGAaa-- 81

0,0

-------UAGCUUAUCAGACUGAUGUUGAau-- 9

0,0

-------UAGCUUAUCAGACUGAUGUUGAu--   28

0,0

-------UAGCUUAUCAGACUGAUGUUGAuu---   7

0,0

-------UAGCUUAUCAGACUGAUGUUGAC--   5574

0,1

-------UAGCUUAUCAGACUGAUGUUGACa---   2765

0,1

-------UAGCUUAUCAGACUGAUGUUGACaa--   6

0,1

-------UAGCUUAUCAGACUGAUGUUGACau--   16

0,1

-------UAGCUUAUCAGACUGAUGUUGACU---   1359

0,2

-------UAGCUUAUCAGACUGAUGUUGACUa--   5

0,2

--------AGCUUAUCAGACUGAUGU--   18

1,-3

--------AGCUUAUCAGACUGAUGUU---   132

1,-2

--------AGCUUAUCAGACUGAUGUUa--   3

1,-2

--------AGCUUAUCAGACUGAUGUUG--   68

1,-1

--------AGCUUAUCAGACUGAUGUUGu---   16

1,-1

--------AGCUUAUCAGACUGAUGUUGA---   200

1,0

--------AGCUUAUCAGACUGAUGUUGAa--   4

1,0

--------AGCUUAUCAGACUGAUGUUGAC--   72

1,1

--------AGCUUAUCAGACUGAUGUUGACa---   55

1,1

--------AGCUUAUCAGACUGAUGUUGACau--   6

1,1

--------AGCUUAUCAGACUGAUGUUGACU---   8

1,2

---------GCUUAUCAGACUGAUGUU-- 46

2,-2

---------GCUUAUCAGACUGAUGUUG--- 33

2,-1

---------GCUUAUCAGACUGAUGUUGA-- 39

2,0

---------GCUUAUCAGACUGAUGUUGAC--- 11

2,1

---------GCUUAUCAGACUGAUGUUGACa-- 6

2,1

---------GCUUAUCAGACUGAUGUUGACU-- 2

2,2

--CAACACCAGUCGAUGGGC--------- 2

0,-3

--CAACACCAGUCGAUGGGCU-------- 35

0,-2

--CAACACCAGUCGAUGGGCUG------- 16

0,-1

--CAACACCAGUCGAUGGGCUGU------ 30

0,0

--CAACACCAGUCGAUGGGCUGUaa---- 97

0,0

--CAACACCAGUCGAUGGGCUGUau---- 24

0,0

--CAACACCAGUCGAUGGGCUGUuu---- 8

0,0

--CAACACCAGUCGAUGGGCUGUC----- 17

0,1

--CAACACCAGUCGAUGGGCUGUCa---- 53

0,1

--CAACACCAGUCGAUGGGCUGUCaa--- 4

0,1

--CAACACCAGUCGAUGGGCUGUCau--- 16

0,1

--CAACACCAGUCGAUGGGCUGUCU---- 37

0,2

--CAACACCAGUCGAUGGGCUGUCUau-- 2

0,2

---CAACACCAGUCGAUGGGCUGUCUu--- 7

0,2

---CAACACCAGUCGAUGGGCUGUCUuu-- 9

0,2

--AACACCAGUCGAUGGGCUGUCau--- 4

1,1

--AACACCAGUCGAUGGGCUGUCU---- 12

1,2

--AACACCAGUCGAUGGGCUGUCUu--- 4

1,2

--AACACCAGUCGAUGGGCUGUCUuu-- 4

1,2

---ACACCAGUCGAUGGGCUGUCU---- 3

2,2

>hsa-mir-210 3p:66-87 SRX018959

acccggcagugccuccaggcgcagggcagccccugcccaccgcacacugcgcugccccagacccaCUGUGCGUGUGACAG
CGGCUGAucugugccugggcagcgcgaccc

....((..((((.(((((((((((..(((((.(((..((((((((.((.((((...))).).)).)))))).))).)))
.)))))..)))))))))))..)))))..)).

--ACUGUGCGUGUGACAG
CGGCU------------------------ 10 -1,-2

---CUGUGCGUGUGACAG
CGG--------------------------- 2 0,-4

---CUGUGCGUGUGACAG
CGGCU------------------------- 53 0,-2

---CUGUGCGUGUGACAG
CGGCUGA----------------------- 18 0,0

---CUGUGCGUGUGACAG
CGGCUGAa---------------------- 9 0,0

>hsa-mir-2110 5p:8-29 SRX018959

cagggguUUGGGGAAACGGCCGCUGAGUGaggcgucggcuguguuucucaccgcggucuuuuccucccacucuug

((((((..((((((.(((((((((.((.(((.....))).)).)))))..)))))).))))))..)))))))

-------UUGGGGAAACGGCCGCUGAG-- 2

0,-2

-------UUGGGGAAACGGCCGCUGAGa--- 3

0,-2
-------UUGGGGAAACGGCCGCUGAGU--- 2
0,-1
-------UUGGGGAAACGGCCGCUGAGUaa--- 2
0,-1
-------UUGGGGAAACGGCCGCUGAGUG-- 19
0,0
-------UUGGGGAAACGGCCGCUGAGUGu--- 21
0,0
-------UUGGGGAAACGGCCGCUGAGUGuu-- 3
0,0
-------UUGGGGAAACGGCCGCUGAGUGA--- 37
0,1
-------UUGGGGAAACGGCCGCUGAGUGAu-- 3
0,1
-------UUGGGGAAACGGCCGCUGAGUGAG-- 5
0,2
-------UUGGGGAAACGGCCGCUGAGUGAGu--- 7
0,2
--------UGGGGAAACGGCCGCUGAGUGA--- 3
1,1
---CACCGCGGUCUUUCCUCCCACU---- 2

>hsa-mir-2114 MA:13-34;mi:49-69 SRX018959
ccuccaugcuccUAGUCCCUUCCUUGAAGCGGUCggauaaucacaugaCGAGCCUCAAGCAAGGGACUUcaagcuggugg
((.(((.(((...((((((((.(((((.((.((((..........))))..)).))))).))))))))...)))))).))
------------UAGUCCCUUCCUUGAAGCGG--
2 0,-2

>hsa-mir-212 3p:71-91 SRX018959
cggggcaccccgcccggacagcgcgccggcaccuuggcucuagacugcuuacugcccgggccgcccucagUAACAGUCUC
CAGUCACGGCCaccgacgccuggccccgcc
..................(((.(((.(((..((.(((((..((((((.((((((...(((...))).)))))))))))))).
.))))).))...))).)))))).........
--UAACAGUCUC
CAGUCACGGCCA------------------ 2 0,1

>hsa-mir-214 mi:30-51;MA:71-92 SRX018959

ggccuggcuggacagaguugucaugugucUGCCUGUCUACACUUGCUGUGCagaacauccgcucaccuguACAGCAGGCA
CAGACAGGCAGUcacaugacaacccagccu
.....((((((.....(((((((((((.((((((((((...((((((((((((.............))))))))))))..
.)))))))))).))))))))))))))))).
--GUACAGCAGGCA
CAGACAGG---------------------- 3 -2,-4
---UACAGCAGGCA
CAGACAG----------------------- 5 -1,-5
---UACAGCAGGCA
CAGACAGG---------------------- 26 -1,-4
---UACAGCAGGCA
CAGACAGGa--------------------- 4 -1,-4
---UACAGCAGGCA
CAGACAGGaa-------------------- 4 -1,-4
---UACAGCAGGCA
CAGACAGGCA-------------------- 30 -1,-2
---UACAGCAGGCA
CAGACAGGCAa------------------- 49 -1,-2
---UACAGCAGGCA
CAGACAGGCAaa------------------ 5 -1,-2
---UACAGCAGGCA
CAGACAGGCAau------------------ 9 -1,-2
---UACAGCAGGCA
CAGACAGGCAu------------------- 44 -1,-2
---UACAGCAGGCA
CAGACAGGCAuu------------------ 12 -1,-2
---UACAGCAGGCA
CAGACAGGCAG------------------- 4 -1,-1
---UACAGCAGGCA
CAGACAGGCAGa------------------ 14 -1,-1
---UACAGCAGGCA
CAGACAGGCAGU------------------ 2 -1,0
--ACAGCAGGCA
CAGACAGG---------------------- 24 0,-4
--ACAGCAGGCA
CAGACAGGa--------------------- 2 0,-4
--ACAGCAGGCA
CAGACAGGaa-------------------- 3 0,-4

--ACAGCAGGCA
CAGACAGGCA-------------------- 83 0,-2
--ACAGCAGGCA
CAGACAGGCAa------------------- 37 0,-2
--ACAGCAGGCA
CAGACAGGCAaa------------------ 22 0,-2
--ACAGCAGGCA
CAGACAGGCAau------------------ 13 0,-2
--ACAGCAGGCA
CAGACAGGCAu------------------- 18 0,-2
--ACAGCAGGCA
CAGACAGGCAuu------------------ 2 0,-2
--ACAGCAGGCA
CAGACAGGCAG------------------- 32 0,-1
--ACAGCAGGCA
CAGACAGGCAGa------------------ 32 0,-1
--ACAGCAGGCA
CAGACAGGCAGaa----------------- 7 0,-1
--ACAGCAGGCA
CAGACAGGCAGU------------------ 27 0,0
--ACAGCAGGCA
CAGACAGGCAGUa----------------- 5 0,0

>hsa-mir-215 5p:27-47 SRX018959
aucauucagaaaugguauacaggaaaAUGACCUAUGAAUUGACAGACaauauagcugaguuugucugucauuucuuuagg
ccaauauucuguaugacugugcuacuucaa
........(((.((((((((((((.(.((.((((.(((.((((((((((...........)))))))))).)))..))))
.)).).))))))......)))))).)))..
--------------------------AUGACCUAUGAAUUGACA------------------------------------
------------------------------ 2 0,-3
--------------------------AUGACCUAUGAAUUGACAG-----------------------------------
------------------------------ 198 0,-2
--------------------------AUGACCUAUGAAUUGACAGA----------------------------------
------------------------------ 9 0,-1
--------------------------AUGACCUAUGAAUUGACAGAC---------------------------------
------------------------------ 13 0,0
--------------------------AUGACCUAUGAAUUGACAGACA--------------------------------
------------------------------ 20 0,1

---------------------------UGACCUAUGAAUUGACAG------------------------------------
------------------------------ 3441 1,-2
---------------------------UGACCUAUGAAUUGACAGu-----------------------------------
------------------------------ 33 1,-2
---------------------------UGACCUAUGAAUUGACAGA-----------------------------------
------------------------------ 91 1,-1
---------------------------UGACCUAUGAAUUGACAGAa----------------------------------
------------------------------ 5 1,-1
---------------------------UGACCUAUGAAUUGACAGAaa---------------------------------
------------------------------ 5 1,-1
---------------------------UGACCUAUGAAUUGACAGAau---------------------------------
------------------------------ 3 1,-1
---------------------------UGACCUAUGAAUUGACAGAC----------------------------------
------------------------------ 14 1,0
---------------------------UGACCUAUGAAUUGACAGACA---------------------------------
------------------------------ 62 1,1
---------------------------UGACCUAUGAAUUGACAGACAu--------------------------------
------------------------------ 11 1,1
---------------------------UGACCUAUGAAUUGACAGACAuu-------------------------------
------------------------------ 3 1,1
---------------------------UGACCUAUGAAUUGACAGACAA--------------------------------
------------------------------ 2 1,2
---------------------------UGACCUAUGAAUUGACAGACAAU-------------------------------
------------------------------ 2 1,3

>hsa-mir-216b 5p:11-32 SRX018959
gcagacuggaAAAUCUCUGCAGGCAAAUGUGAugucacugaggaaaucacacacuuacccguagagauucuacagucuga
ca
.(((((((...(((((((((.((....((((((.((......)).))))))......)).)))))))))...))))))).
..
---------AAAAUCUCUGCAGGCAAAUGU---
-- 2 -1,-2
---------AAAAUCUCUGCAGGCAAAUGUG--
-- 2 -1,-1
----------AAAUCUCUGCAGGCAAAUGU---
-- 5 0,-2
----------AAAUCUCUGCAGGCAAAUGUG--
-- 8 0,-1

----------AAAUCUCUGCAGGCAAAUGUGu--
-- 2   0,-1
----------AAAUCUCUGCAGGCAAAUGUGA--
-- 9   0,0

>hsa-mir-217   5p:35-57   SRX018959
aguauaauuauuacauaguuuuugaugucgcagaUACUGCAUCAGGAACUGAUUGGAuaagaaucagucaccaucaguuc
cuaaugcauugccuucagcaucuaaacaag
.................((((..(((((.(.((.((.(((((.((((((((((.((((........))).).))))))))
)).))))).)).)).).))))).))))...
---------------------------------AUACUGCAUCAGGAACUGAUU--------------------------
------------------------------ 6   -1,-3
---------------------------------AUACUGCAUCAGGAACUGAUUG-------------------------
------------------------------ 23 -1,-2
---------------------------------AUACUGCAUCAGGAACUGAUUGG------------------------
------------------------------ 5   -1,-1
----------------------------------UACUGCAUCAGGAACUGAUUG-------------------------
------------------------------ 2   0,-2

>hsa-mir-219-1 5p:21-41;3p:62-83   SRX018959
ccgccccgggccgcggcuccUGAUUGUCCAAACGCAAUUCUcgagucuauggcuccggccgAGAGUUGAGUCUGGACGUC
CCGagccgccgcccccaaaccucgagcggg
((((.(.((((.(((((((..(((.(((((.((.(((((((((.(((.........)))))))))))).)).))))))))
..))))))).)))).........).)))).
--------------------UGAUUGUCCAAACGCAAU--
------------------------------ 6   0,-3
--------------------UGAUUGUCCAAACGCAAUU---
------------------------------ 2   0,-2
--------------------UGAUUGUCCAAACGCAAUUCUCG-------------------------------------
------------------------------ 2   0,2
---AGAGUUGAGUCUGGACGUC
CCGu-------------------------- 5   0,0
---AGAGUUGAGUCUGGACGUC
CCGA-------------------------- 3   0,1

>hsa-mir-219-2 5p:19-39;3p:62-83   SRX018959
acucaggggcuucgccacUGAUUGUCCAAACGCAAUUCUuguacgagucugcggccaaccgAGAAUUGUGGCUGGACAUC
UGUggcugagcuccggg

.(((.(((((((.(((((.(((.(((((..(((((((((((...(.((.....)))...)))))))))))..))))))))
.))))).))))))))))
-----------------UGAUUGUCCAAACGCAAU---
-----------------   6   0,-3
-----------------UGAUUGUCCAAACGCAAUU--
-----------------   2   0,-2

>hsa-mir-22 mi:15-36;MA:53-74   SRX018959
ggcugagccgcaguAGUUCUUCAGUGGCAAGCUUUAuguccugacccagcuaAAGCUGCCAGUUGAAGAACUGUugcccu
cugcc
(((.(((..((((((((((((((((((((.((((((.((.........)))))))))))))).)))))))))))))).))
).)))
--------------AGUUCUUCAGUGGCAAGC--
-----   17   0,-4
--------------AGUUCUUCAGUGGCAAGCU---
-----   59   0,-3
--------------AGUUCUUCAGUGGCAAGCUU--
-----   15   0,-2
--------------AGUUCUUCAGUGGCAAGCUUa---
-----   5   0,-2
--------------AGUUCUUCAGUGGCAAGCUUU---
-----   559 0,-1
--------------AGUUCUUCAGUGGCAAGCUUUu--
-----   21   0,-1
--------------AGUUCUUCAGUGGCAAGCUUUA--
-----   252 0,0
--------------AGUUCUUCAGUGGCAAGCUUUAa---
-----   28   0,0
--------------AGUUCUUCAGUGGCAAGCUUUAaa--
-----   3   0,0
--------------AGUUCUUCAGUGGCAAGCUUUAU---
-----   45   0,1
---------------GUUCUUCAGUGGCAAGCUUU---
-----   3   1,-1
---------------GUUCUUCAGUGGCAAGCUUUAU---
-----   2   1,1
---AAAGCUGCCAGUUGAAGAAC---------
-----   2   -1,-3

--AAAGCUGCCAGUUGAAGAACU--------
-----	2	-1,-2
---AAGCUGCCAGUUGAAGAA----------
-----	2213	0,-4
---AAGCUGCCAGUUGAAGAAa---------
-----	20	0,-4
---AAGCUGCCAGUUGAAGAAaa--------
-----	2	0,-4
---AAGCUGCCAGUUGAAGAAau--------
-----	4	0,-4
---AAGCUGCCAGUUGAAGAAu---------
-----	15	0,-4
---AAGCUGCCAGUUGAAGAAuu--------
-----	144	0,-4
---AAGCUGCCAGUUGAAGAAC---------
-----	1122	0,-3
---AAGCUGCCAGUUGAAGAACa--------
-----	221	0,-3
---AAGCUGCCAGUUGAAGAACaa-------
-----	56	0,-3
---AAGCUGCCAGUUGAAGAACau-------
-----	8	0,-3
---AAGCUGCCAGUUGAAGAACU--------
-----	4029	0,-2
---AAGCUGCCAGUUGAAGAACUa-------
-----	48	0,-2
---AAGCUGCCAGUUGAAGAACUaa------
-----	17	0,-2
---AAGCUGCCAGUUGAAGAACUau------
-----	31	0,-2
---AAGCUGCCAGUUGAAGAACUu-------
-----	48	0,-2
---AAGCUGCCAGUUGAAGAACUuu------
-----	141	0,-2
---AAGCUGCCAGUUGAAGAACUG-------
-----	443	0,-1
---AAGCUGCCAGUUGAAGAACUGa------
-----	41	0,-1

---AAGCUGCCAGUUGAAGAACUGU-----
-----   2517   0,0
---AAGCUGCCAGUUGAAGAACUGUa----
-----   54 0,0
---AAGCUGCCAGUUGAAGAACUGUaa---
-----   15 0,0
---AAGCUGCCAGUUGAAGAACUGUau---
-----   37 0,0
---AAGCUGCCAGUUGAAGAACUGUU----
-----   73 0,1
---AAGCUGCCAGUUGAAGAACUGUUa---
-----   12 0,1
---AAGCUGCCAGUUGAAGAACUGUUau--
-----   2   0,1
---AAGCUGCCAGUUGAAGAACUGUUu---
-----   69 0,1
---AAGCUGCCAGUUGAAGAACUGUUuu--
-----   7   0,1
--AGCUGCCAGUUGAAGAAC--------
-----   19 1,-3
--AGCUGCCAGUUGAAGAACU-------
-----   16 1,-2
--AGCUGCCAGUUGAAGAACUGU-----
-----   20 1,0
--AGCUGCCAGUUGAAGAACUGUaa---
-----   2   1,0
--AGCUGCCAGUUGAAGAACUGUUu---
-----   4   1,1

>hsa-mir-221   mi:25-46;MA:65-87   SRX018959
ugaacauccaggucuggggcaugaACCUGGCAUACAAUGUAGAUUUcuguguucguuaggcaacAGCUACAUUGUCUGCU
GGGUUUCaggcuaccuggaaacauguucuc
.((((((((((((....(((.((((((..(((.(((((((((....(((((((.....))).))))))))))))).))).
.)).)))).))))))))))....)))))..
-----------------------AACCUGGCAUACAAUGUAGAUU-----------------------------------
------------------------------   2   -1,-1
-----------------------AACCUGGCAUACAAUGUAGAUUU----------------------------------
------------------------------   12 -1,0

------------------------ACCUGGCAUACAAUGUAGAU------------------------------------
------------------------------ 7 0,-2
------------------------ACCUGGCAUACAAUGUAGAUa-----------------------------------
------------------------------ 22 0,-2
------------------------ACCUGGCAUACAAUGUAGAUaa----------------------------------
------------------------------ 2 0,-2
------------------------ACCUGGCAUACAAUGUAGAUU-----------------------------------
------------------------------ 89 0,-1
------------------------ACCUGGCAUACAAUGUAGAUUa----------------------------------
------------------------------ 9 0,-1
------------------------ACCUGGCAUACAAUGUAGAUUU----------------------------------
------------------------------ 493 0,0
------------------------ACCUGGCAUACAAUGUAGAUUUa---------------------------------
------------------------------ 10 0,0
------------------------ACCUGGCAUACAAUGUAGAUUUu---------------------------------
------------------------------ 9 0,0
------------------------ACCUGGCAUACAAUGUAGAUUUC---------------------------------
------------------------------ 78 0,1
------------------------ACCUGGCAUACAAUGUAGAUUUCU--------------------------------
------------------------------ 33 0,2
------------------------ACCUGGCAUACAAUGUAGAUUUCUG-------------------------------
------------------------------ 2 0,3
-------------------------CCUGGCAUACAAUGUAGAUUU----------------------------------
------------------------------ 5 1,0
--AGCUACAUUGUCUGCU
GG---------------------------- 3 0,-5
--AGCUACAUUGUCUGCU
GGG--------------------------- 68 0,-4
--AGCUACAUUGUCUGCU
GGGU-------------------------- 10 0,-3
--AGCUACAUUGUCUGCU
GGGUaa------------------------ 4 0,-3
--AGCUACAUUGUCUGCU
GGGUau------------------------ 7 0,-3
--AGCUACAUUGUCUGCU
GGGUU------------------------- 70 0,-2
--AGCUACAUUGUCUGCU
GGGUUa------------------------ 23 0,-2

--AGCUACAUUGUCUGCU
GGGUUaa----------------------- 11 0,-2
--AGCUACAUUGUCUGCU
GGGUUau----------------------- 34 0,-2
--AGCUACAUUGUCUGCU
GGGUUU------------------------ 704 0,-1
--AGCUACAUUGUCUGCU
GGGUUUa----------------------- 64 0,-1
--AGCUACAUUGUCUGCU
GGGUUUaa---------------------- 10 0,-1
--AGCUACAUUGUCUGCU
GGGUUUau---------------------- 16 0,-1
--AGCUACAUUGUCUGCU
GGGUUUu----------------------- 177 0,-1
--AGCUACAUUGUCUGCU
GGGUUUuu---------------------- 10 0,-1
--AGCUACAUUGUCUGCU
GGGUUUC----------------------- 405 0,0
--AGCUACAUUGUCUGCU
GGGUUUCu---------------------- 20 0,0
--AGCUACAUUGUCUGCU
GGGUUUCA---------------------- 16 0,1
---GCUACAUUGUCUGCU
GGGUUU------------------------ 23 1,-1
---GCUACAUUGUCUGCU
GGGUUUu----------------------- 6   1,-1
---GCUACAUUGUCUGCU
GGGUUUC----------------------- 6   1,0

>hsa-mir-222   mi:31-52;MA:69-89   SRX018959
gcugcuggaagguguagguacccucaauggCUCAGUAGCCAGUGUAGAUCCUgucuuucguaaucagcAGCUACAUCUGG
CUACUGGGUcucugauggcaucuucuagcu
...(((((((((((......((.(((..(((((((((((((((((((...(((...........)))...))))).))))
))))))))))..))).))))))))))))).
--AGCUACAUCUGG
CUACUG------------------------ 288 0,-3
--AGCUACAUCUGG
CUACUGa----------------------- 3   0,-3

---AGCUACAUCUGG
CUACUGG---------------------- 421 0,-2
---AGCUACAUCUGG
CUACUGGG--------------------- 464 0,-1
---AGCUACAUCUGG
CUACUGGGU-------------------- 499 0,0
---AGCUACAUCUGG
CUACUGGGUa------------------- 12 0,0
---AGCUACAUCUGG
CUACUGGGUaa------------------ 31 0,0
---AGCUACAUCUGG
CUACUGGGUau------------------ 38 0,0
---AGCUACAUCUGG
CUACUGGGUu------------------- 16 0,0
---AGCUACAUCUGG
CUACUGGGUuu------------------ 13 0,0
---AGCUACAUCUGG
CUACUGGGUC------------------- 2046   0,1
---AGCUACAUCUGG
CUACUGGGUCa------------------ 20 0,1
---AGCUACAUCUGG
CUACUGGGUCau----------------- 2   0,1
---AGCUACAUCUGG
CUACUGGGUCU------------------ 1787   0,2
---AGCUACAUCUGG
CUACUGGGUCUa----------------- 13 0,2
---AGCUACAUCUGG
CUACUGGGUCUaa---------------- 6   0,2
---AGCUACAUCUGG
CUACUGGGUCUau---------------- 9   0,2
---AGCUACAUCUGG
CUACUGGGUCUu----------------- 46 0,2
---AGCUACAUCUGG
CUACUGGGUCUuu---------------- 52 0,2
---AGCUACAUCUGG
CUACUGGGUCUC----------------- 237 0,3
---AGCUACAUCUGG
CUACUGGGUCUCa---------------- 25 0,3

---AGCUACAUCUGG
CUACUGGGUCUCU----------------- 54 0,4
--GCUACAUCUGG
CUACUGG----------------------- 4   1,-2
--GCUACAUCUGG
CUACUGGGUC-------------------- 7   1,1
--GCUACAUCUGG
CUACUGGGUCU------------------- 6   1,2

>hsa-mir-223   mi:26-47;MA:68-89   SRX018959
ccuggccuccugcagugccacgcucCGUGUAUUUGACAAGCUGAGUUggacacuccaugugguagagUGUCAGUUUGUCA
AAUACCCCAagugcggcacaugcuuaccag
.((((......(((((((.((((..(.(((((((((((((((......(((((((.........))))))))))))))))
))))).)..)))).))))).)))...))))
-------------------------CGUGUAUUUGACAAGCUGAGUUG---------------------------------
------------------------------ 4   0,1
-------------------------CGUGUAUUUGACAAGCUGAGUUGG--------------------------------
------------------------------ 2   0,2
---GUGUCAGUUUGUCA
AAUAC------------------------- 2   -1,-4
--UGUCAGUUUGUCA
AAUAC------------------------- 8   0,-4
--UGUCAGUUUGUCA
AAUACC------------------------ 18 0,-3
--UGUCAGUUUGUCA
AAUACCC----------------------- 15 0,-2
--UGUCAGUUUGUCA
AAUACCCC---------------------- 17 0,-1
--UGUCAGUUUGUCA
AAUACCCCA--------------------- 27 0,0
--UGUCAGUUUGUCA
AAUACCCCAu-------------------- 20 0,0
--UGUCAGUUUGUCA
AAUACCCCAuu------------------- 2   0,0
--UGUCAGUUUGUCA
AAUACCCCAA-------------------- 21 0,1
--UGUCAGUUUGUCA
AAUACCCCAAa------------------- 4   0,1

---UGUCAGUUUGUCA
AAUACCCCAAu------------------- 9   0,1
---UGUCAGUUUGUCA
AAUACCCCAAG------------------- 2   0,2
--GUCAGUUUGUCA
AAUACC------------------------ 24 1,-3
--GUCAGUUUGUCA
AAUACCC----------------------- 13 1,-2
--GUCAGUUUGUCA
AAUACCCC---------------------- 9   1,-1
--GUCAGUUUGUCA
AAUACCCCA--------------------- 14 1,0
--GUCAGUUUGUCA
AAUACCCCAu-------------------- 3   1,0
--GUCAGUUUGUCA
AAUACCCCAA-------------------- 37 1,1
--GUCAGUUUGUCA
AAUACCCCAAu------------------- 7   1,1

>hsa-mir-224   MA:8-28;mi:53-75   SRX018959
gggcuuuCAAGUCACUAGUGGUUCCGUUuaguagaugauugugcauuguuucAAAAUGGUGCCCUAGUGACUACAaagcc
c
(((((((..(((((((((.(((.((((((.(.(((..((.....))..)))).)))))).))))))))))))..))))))
)
-------CAAGUCACUAGUGGUUCCGU---
-   2   0,-1
-------CAAGUCACUAGUGGUUCCGUU--
-   2   0,0
-------CAAGUCACUAGUGGUUCCGUUU---
-   30 0,1
-------CAAGUCACUAGUGGUUCCGUUUu--
-   2   0,1
-------CAAGUCACUAGUGGUUCCGUUUA--
-   9   0,2
-------CAAGUCACUAGUGGUUCCGUUUAG---
-   31 0,3
---AAAUGGUGCCCUAGUGAC---------
-   3   1,-4

>hsa-mir-2355   5p:11-31;3p:54-75   SRX018959
cagacgugucAUCCCCAGAUACAAUGGACAAuaugcuauuauaaaucguauggcAUUGUCCUUGCUGUUUGGAGAUaauac
ugcugac
(((..((((.(((.((((((((((.(((((..(((((((.........)))))))))))))).))))))).))).))))
..))))..
----------AUCCCCAGAUACAAUGGACAA--
------- 4   0,0
----------AUCCCCAGAUACAAUGGACAAU---
------- 7   0,1
----------AUCCCCAGAUACAAUGGACAAUu--
------- 2   0,1

>hsa-mir-23a   mi:9-30;MA:45-65   SRX018959
ggccggcuGGGGUUCCUGGGGAUGGGAUUUgcuuccugucacaaAUCACAUUGCCAGGGAUUUCCaaccgacc
((.(((.((((((((((((.((((.((((((..........)))))).)))).))))))).))))).))).))
--------GGGGUUCCUGGGGAUGGGAU---   2
0,-2
--------GGGGUUCCUGGGGAUGGGAUUU---   2
0,0
---AAUCACAUUGCCAGGGAUU-----------   4
-1,-3
---AAUCACAUUGCCAGGGAUUUC---------   5
-1,-1
---AAUCACAUUGCCAGGGAUUUCu--------   3
-1,-1
---AAUCACAUUGCCAGGGAUUUCC--------   3
-1,0
---AAUCACAUUGCCAGGGAUUUCCA-------   4
-1,1
---AAUCACAUUGCCAGGGAUUUCCAA------   2
-1,2
--AUCACAUUGCCAGGGAUU-----------   59
0,-3
--AUCACAUUGCCAGGGAUUU----------   664
0,-2
--AUCACAUUGCCAGGGAUUUa---------   132
0,-2

--AUCACAUUGCCAGGGAUUUaa-------- 19
0,-2
--AUCACAUUGCCAGGGAUUUau-------- 11
0,-2
--AUCACAUUGCCAGGGAUUUu--------- 86
0,-2
--AUCACAUUGCCAGGGAUUUC--------- 557
0,-1
--AUCACAUUGCCAGGGAUUUCa-------- 103
0,-1
--AUCACAUUGCCAGGGAUUUCaa------- 11
0,-1
--AUCACAUUGCCAGGGAUUUCau------- 4
0,-1
--AUCACAUUGCCAGGGAUUUCu-------- 18
0,-1
--AUCACAUUGCCAGGGAUUUCC-------- 444
0,0
--AUCACAUUGCCAGGGAUUUCCu------- 5
0,0
--AUCACAUUGCCAGGGAUUUCCuu------ 2
0,0
--AUCACAUUGCCAGGGAUUUCCA------- 1528
0,1
--AUCACAUUGCCAGGGAUUUCCAu------ 183
0,1
--AUCACAUUGCCAGGGAUUUCCAuu----- 10
0,1
--AUCACAUUGCCAGGGAUUUCCAA------ 218
0,2
--AUCACAUUGCCAGGGAUUUCCAAu----- 19
0,2
---UCACAUUGCCAGGGAUUUu--------- 3
1,-2
---UCACAUUGCCAGGGAUUUC--------- 7
1,-1
---UCACAUUGCCAGGGAUUUCC-------- 3
1,0

---UCACAUUGCCAGGGAUUUCCA------- 6
1,1
---UCACAUUGCCAGGGAUUUCCAu------ 2
1,1
---UCACAUUGCCAGGGAUUUCCAuu----- 3
1,1
---UCACAUUGCCAGGGAUUUCCAA------ 4
1,2

>hsa-mir-23b mi:20-41;MA:58-78 SRX018959
cucaggugcucuggcugcuUGGGUUCCUGGCAUGCUGAUUUgugacuuaagauuaaaAUCACAUUGCCAGGGAUUACCac
gcaaccacgaccuuggc
.(((((.(.(((((.(((.((((((((((((((((.((((((.............)))))))).))))))))))..))).
))).))).))))))))).
------------------UUGGGUUCCUGGCAUGCUGAU---
----------------- 7 -1,-2
------------------UUGGGUUCCUGGCAUGCUGAUa--
----------------- 7 -1,-2
------------------UUGGGUUCCUGGCAUGCUGAUU--
----------------- 17 -1,-1
------------------UUGGGUUCCUGGCAUGCUGAUUa---------------------------------------
----------------- 2 -1,-1
------------------UUGGGUUCCUGGCAUGCUGAUUU---------------------------------------
----------------- 23 -1,0
------------------UUGGGUUCCUGGCAUGCUGAUUUu--------------------------------------
----------------- 4 -1,0
-------------------UGGGUUCCUGGCAUGCUGA--
----------------- 3 0,-3
-------------------UGGGUUCCUGGCAUGCUGAU---
----------------- 24 0,-2
-------------------UGGGUUCCUGGCAUGCUGAUa--
----------------- 25 0,-2
-------------------UGGGUUCCUGGCAUGCUGAUaa---------------------------------------
----------------- 6 0,-2
-------------------UGGGUUCCUGGCAUGCUGAUU--
----------------- 114 0,-1
-------------------UGGGUUCCUGGCAUGCUGAUUa---------------------------------------
----------------- 13 0,-1

-------------------UGGGUUCCUGGCAUGCUGAUUaa-------------------------------------
-----------------   2   0,-1
-------------------UGGGUUCCUGGCAUGCUGAUUU--------------------------------------
-----------------   606 0,0
-------------------UGGGUUCCUGGCAUGCUGAUUUa-------------------------------------
-----------------   37   0,0
-------------------UGGGUUCCUGGCAUGCUGAUUUaa------------------------------------
-----------------   2   0,0
-------------------UGGGUUCCUGGCAUGCUGAUUUau------------------------------------
-----------------   4   0,0
-------------------UGGGUUCCUGGCAUGCUGAUUUu-------------------------------------
-----------------   73   0,0
--------------------GGGUUCCUGGCAUGCUGA---
-----------------   3   1,-3
--------------------GGGUUCCUGGCAUGCUGAU--
-----------------   11   1,-2
--------------------GGGUUCCUGGCAUGCUGAUa---------------------------------------
-----------------   3   1,-2
--------------------GGGUUCCUGGCAUGCUGAUU---------------------------------------
-----------------   23   1,-1
--------------------GGGUUCCUGGCAUGCUGAUUU--------------------------------------
-----------------   214 1,0
--------------------GGGUUCCUGGCAUGCUGAUUUa-------------------------------------
-----------------   15   1,0
--------------------GGGUUCCUGGCAUGCUGAUUUaa------------------------------------
-----------------   18   1,0
--------------------GGGUUCCUGGCAUGCUGAUUUau------------------------------------
-----------------   7   1,0
--------------------GGGUUCCUGGCAUGCUGAUUUu-------------------------------------
-----------------   67   1,0
--------------------GGGUUCCUGGCAUGCUGAUUUuu------------------------------------
-----------------   18   1,0
--------------------GGGUUCCUGGCAUGCUGAUUUG-------------------------------------
-----------------   9   1,1
--------------------GGGUUCCUGGCAUGCUGAUUUGa------------------------------------
-----------------   2   1,1
--------------------GGGUUCCUGGCAUGCUGAUUUGU------------------------------------
-----------------   5   1,2

--AAUCACAUUGCCAGGGAUU-----
-----------------   4   -1,-3
--AAUCACAUUGCCAGGGAUUA----
-----------------   11   -1,-2
--AAUCACAUUGCCAGGGAUUAa---
-----------------   2   -1,-2
--AAUCACAUUGCCAGGGAUUAC---
-----------------   4   -1,-1
--AAUCACAUUGCCAGGGAUUACC--
-----------------   11   -1,0
--AAUCACAUUGCCAGGGAUUACCA-
-----------------   5   -1,1
--AAUCACAUUGCCAGGGAUUACCAa
-----------------   2   -1,1
---AUCACAUUGCCAGGGAUU-----
-----------------   59   0,-3
---AUCACAUUGCCAGGGAUUA----
-----------------   696 0,-2
---AUCACAUUGCCAGGGAUUAa---
-----------------   141 0,-2
---AUCACAUUGCCAGGGAUUAaa--
-----------------   23   0,-2
---AUCACAUUGCCAGGGAUUAau--
-----------------   3   0,-2
---AUCACAUUGCCAGGGAUUAu---
-----------------   61   0,-2
---AUCACAUUGCCAGGGAUUAuu--
-----------------   11   0,-2
---AUCACAUUGCCAGGGAUUAC---
-----------------   726 0,-1
---AUCACAUUGCCAGGGAUUACa--
-----------------   211 0,-1
---AUCACAUUGCCAGGGAUUACaa-
-----------------   18   0,-1
---AUCACAUUGCCAGGGAUUACau-
-----------------   2   0,-1
---AUCACAUUGCCAGGGAUUACu--
-----------------   39   0,-1

--AUCACAUUGCCAGGGAUUACC--
-----------------   959 0,0
--AUCACAUUGCCAGGGAUUACCu-
-----------------   23   0,0
--AUCACAUUGCCAGGGAUUACCuu
-----------------   8   0,0
--AUCACAUUGCCAGGGAUUACCA-
-----------------   3300   0,1
--AUCACAUUGCCAGGGAUUACCAa
-----------------   85   0,1
--AUCACAUUGCCAGGGAUUACCAa
a----------------   5   0,1
--AUCACAUUGCCAGGGAUUACCAa
u----------------   10   0,1
--AUCACAUUGCCAGGGAUUACCAu
-----------------   628 0,1
--AUCACAUUGCCAGGGAUUACCAu
u----------------   171 0,1
--AUCACAUUGCCAGGGAUUACCAC
-----------------   920 0,2
--AUCACAUUGCCAGGGAUUACCAC
a----------------   142 0,2
--AUCACAUUGCCAGGGAUUACCAC
aa---------------   2   0,2
--AUCACAUUGCCAGGGAUUACCAC
au---------------   9   0,2
--AUCACAUUGCCAGGGAUUACCAC
u----------------   2467   0,2
--AUCACAUUGCCAGGGAUUACCAC
uu---------------   40   0,2
--AUCACAUUGCCAGGGAUUACCAC
G----------------   13   0,3
--AUCACAUUGCCAGGGAUUACCAC
Gu---------------   3   0,3
---UCACAUUGCCAGGGAUUA----
-----------------   8   1,-2
---UCACAUUGCCAGGGAUUAaa--
-----------------   2   1,-2

--UCACAUUGCCAGGGAUUAC---
-----------------   12 1,-1
--UCACAUUGCCAGGGAUUACa--
-----------------   5   1,-1
--UCACAUUGCCAGGGAUUACaa-
-----------------   3   1,-1
--UCACAUUGCCAGGGAUUACC--
-----------------   9   1,0
--UCACAUUGCCAGGGAUUACCA-
-----------------   70 1,1
--UCACAUUGCCAGGGAUUACCAa
-----------------   7   1,1
--UCACAUUGCCAGGGAUUACCAu
-----------------   7   1,1
--UCACAUUGCCAGGGAUUACCAu
u----------------   23 1,1
--UCACAUUGCCAGGGAUUACCAC
-----------------   25 1,2
--UCACAUUGCCAGGGAUUACCAC
a----------------   22 1,2
--UCACAUUGCCAGGGAUUACCAC
aa---------------   2   1,2
--UCACAUUGCCAGGGAUUACCAC
u----------------   219 1,2
--UCACAUUGCCAGGGAUUACCAC
uu---------------   30 1,2
--UCACAUUGCCAGGGAUUACCAC
G----------------   5   1,3
--UCACAUUGCCAGGGAUUACCAC
Gu---------------   3   1,3
---CACAUUGCCAGGGAUUACCAC
-----------------   2   2,2

>hsa-mir-24-1   mi:7-28;MA:44-65   SRX018959
cuccggUGCCUACUGAGCUGAUAUCAGUucucauuuuacacacUGGCUCAGUUCAGCAGGAACAGgag
((((.((.(((.(((((((((..(((((.............))))).))))))))).))).)).))))
----GGUGCCUACUGAGCUGAUAUCAG---   2
    -2,-1

-----GUGCCUACUGAGCUGAUAUa-- 2
-1,-4
-----GUGCCUACUGAGCUGAUAUCA-- 29
-1,-2
-----GUGCCUACUGAGCUGAUAUCAGU-- 2 -1,0
------UGCCUACUGAGCUGAUAUCA-- 2 0,-2
---UGGCUCAGUUCAGCAGGA------- 12 0,-4
---UGGCUCAGUUCAGCAGGAA------ 65 0,-3
---UGGCUCAGUUCAGCAGGAAa----- 4 0,-3
---UGGCUCAGUUCAGCAGGAAC----- 26 0,-2
---UGGCUCAGUUCAGCAGGAACA---- 128 0,-1
---UGGCUCAGUUCAGCAGGAACAa--- 42 0,-1
---UGGCUCAGUUCAGCAGGAACAaa-- 7 0,-1
---UGGCUCAGUUCAGCAGGAACAau-- 8 0,-1
---UGGCUCAGUUCAGCAGGAACAu--- 28 0,-1
---UGGCUCAGUUCAGCAGGAACAuu-- 19 0,-1
---UGGCUCAGUUCAGCAGGAACAG--- 1174
0,0
---UGGCUCAGUUCAGCAGGAACAGa-- 29 0,0
---UGGCUCAGUUCAGCAGGAACAGaa- 2 0,0
---UGGCUCAGUUCAGCAGGAACAGau- 2 0,0
---UGGCUCAGUUCAGCAGGAACAGu-- 1958
0,0
---UGGCUCAGUUCAGCAGGAACAGuu- 366 0,0
---UGGCUCAGUUCAGCAGGAACAGG-- 5 0,1
---UGGCUCAGUUCAGCAGGAACAGGu- 3 0,1
---UGGCUCAGUUCAGCAGGAACAGGA- 2 0,2
--GGCUCAGUUCAGCAGGAA------ 6 1,-3
--GGCUCAGUUCAGCAGGAAC----- 6 1,-2
--GGCUCAGUUCAGCAGGAACA---- 7 1,-1
--GGCUCAGUUCAGCAGGAACAa--- 3 1,-1
--GGCUCAGUUCAGCAGGAACAuu-- 3 1,-1
--GGCUCAGUUCAGCAGGAACAG--- 48 1,0
--GGCUCAGUUCAGCAGGAACAGa-- 7 1,0
--GGCUCAGUUCAGCAGGAACAGu-- 84 1,0
--GGCUCAGUUCAGCAGGAACAGuu- 25 1,0
--GGCUCAGUUCAGCAGGAACAGG-- 2 1,1
--GGCUCAGUUCAGCAGGAACAGGu- 3 1,1

---GCUCAGUUCAGCAGGAACAG--- 24 2,0

---GCUCAGUUCAGCAGGAACAGa-- 4 2,0

---GCUCAGUUCAGCAGGAACAGu-- 22 2,0

---GCUCAGUUCAGCAGGAACAGuu- 40 2,0

---GCUCAGUUCAGCAGGAACAGG-- 11 2,1

---GCUCAGUUCAGCAGGAACAGGu- 3 2,1

---GCUCAGUUCAGCAGGAACAGGA- 3 2,2

>hsa-mir-24-2 mi:13-34;MA:50-71 SRX018959

cucugccucccgUGCCUACUGAGCUGAAACACAGuugguuuguguacacUGGCUCAGUUCAGCAGGAACAGgg

(((((..(((..(((..(((((((((..((((((.....))))))....)))))))))...)))))).)))))

-----------GUGCCUACUGAGCUGAAACA-- 2 -1,-3

-----------GUGCCUACUGAGCUGAAACAC--- 18 -1,-2

-----------GUGCCUACUGAGCUGAAACACA-- 18 -1,-1

-----------GUGCCUACUGAGCUGAAACACAG--------------------------------------- 17 -1,0

---UGGCUCAGUUCAGCAGGA------ 12 0,-4

---UGGCUCAGUUCAGCAGGAA----- 65 0,-3

---UGGCUCAGUUCAGCAGGAAa---- 4 0,-3

---UGGCUCAGUUCAGCAGGAAC---- 26 0,-2

---UGGCUCAGUUCAGCAGGAACA--- 128 0,-1

---UGGCUCAGUUCAGCAGGAACAa-- 42 0,-1

---UGGCUCAGUUCAGCAGGAACAaa- 7 0,-1

---UGGCUCAGUUCAGCAGGAACAau- 8 0,-1

---UGGCUCAGUUCAGCAGGAACAu-- 28 0,-1

---UGGCUCAGUUCAGCAGGAACAuu- 19

0,-1

---UGGCUCAGUUCAGCAGGAACAG-- 1174

0,0

---UGGCUCAGUUCAGCAGGAACAGa- 29

0,0

---UGGCUCAGUUCAGCAGGAACAGaa 2

0,0

---UGGCUCAGUUCAGCAGGAACAGau 2

0,0

---UGGCUCAGUUCAGCAGGAACAGu- 1958

0,0

---UGGCUCAGUUCAGCAGGAACAGuu 366

0,0

---UGGCUCAGUUCAGCAGGAACAGG- 5

0,1

---UGGCUCAGUUCAGCAGGAACAGGu 3

0,1

--GGCUCAGUUCAGCAGGAA----- 6

1,-3

--GGCUCAGUUCAGCAGGAAC---- 6

1,-2

--GGCUCAGUUCAGCAGGAACA--- 7

1,-1

--GGCUCAGUUCAGCAGGAACAa-- 3

1,-1

--GGCUCAGUUCAGCAGGAACAuu- 3

1,-1

--GGCUCAGUUCAGCAGGAACAG-- 48

1,0

--GGCUCAGUUCAGCAGGAACAGa- 7

1,0

--GGCUCAGUUCAGCAGGAACAGu- 84

1,0

--GGCUCAGUUCAGCAGGAACAGuu 25

1,0

--GGCUCAGUUCAGCAGGAACAGG- 2

1,1

--GGCUCAGUUCAGCAGGAACAGGu 3

1,1
--GCUCAGUUCAGCAGGAACAG--   24
2,0
--GCUCAGUUCAGCAGGAACAGa-   4
2,0
--GCUCAGUUCAGCAGGAACAGu-   22
2,0
--GCUCAGUUCAGCAGGAACAGuu   40
2,0
--GCUCAGUUCAGCAGGAACAGG-   11
2,1
--GCUCAGUUCAGCAGGAACAGGu   3
2,1

>hsa-mir-25 mi:14-34;MA:52-73   SRX018959
ggccaguguugagAGGCGGAGACUUGGGCAAUUGcuggacgcugcccugggCAUUGCACUUGUCUCGGUCUGAcagugcc
ggcc
((((.(..(((..((((.(((((..(.(((((.(((((.......))..)))))))).)..))))).)))).)))..)..).
))))
-------------AGGCGGAGACUUGGGCAAUU---
----   5   0,-1
-------------AGGCGGAGACUUGGGCAAUUG--
----   14   0,0
-------------AGGCGGAGACUUGGGCAAUUGa---
----   3   0,0
-------------AGGCGGAGACUUGGGCAAUUGau--
----   2   0,0
-------------AGGCGGAGACUUGGGCAAUUGu---
----   2   0,0
-------------AGGCGGAGACUUGGGCAAUUGC---
----   52   0,1
-------------AGGCGGAGACUUGGGCAAUUGCU--
----   183   0,2
-------------AGGCGGAGACUUGGGCAAUUGCUa---
----   3   0,2
-------------AGGCGGAGACUUGGGCAAUUGCUu---
----   5   0,2
--------------GGCGGAGACUUGGGCAAUUGCU--

---- 2 1,2
---CAUUGCACUUGUCUCGGU-----------
---- 120 0,-4
---CAUUGCACUUGUCUCGGUu----------
---- 3 0,-4
---CAUUGCACUUGUCUCGGUC----------
---- 262 0,-3
---CAUUGCACUUGUCUCGGUCU---------
---- 473 0,-2
---CAUUGCACUUGUCUCGGUCUu--------
---- 2 0,-2
---CAUUGCACUUGUCUCGGUCUG--------
---- 285 0,-1
---CAUUGCACUUGUCUCGGUCUGu-------
---- 56 0,-1
---CAUUGCACUUGUCUCGGUCUGuu------
---- 7 0,-1
---CAUUGCACUUGUCUCGGUCUGA-------
---- 876 0,0
---CAUUGCACUUGUCUCGGUCUGAa------
---- 26 0,0
---CAUUGCACUUGUCUCGGUCUGAaa-----
---- 2 0,0
---CAUUGCACUUGUCUCGGUCUGAau-----
---- 3 0,0
---CAUUGCACUUGUCUCGGUCUGAu------
---- 31 0,0
--AUUGCACUUGUCUCGGUC----------
---- 4 1,-3
--AUUGCACUUGUCUCGGUCU---------
---- 23 1,-2
--AUUGCACUUGUCUCGGUCUG--------
---- 6 1,-1
--AUUGCACUUGUCUCGGUCUGA-------
---- 22 1,0

>hsa-mir-26a-1 MA:10-31;mi:49-70 SRX018959
guggccucgUUCAAGUAAUCCAGGAUAGGCUgugcaggucccaaugggCCUAUUCUUGGUUACUUGCACGgggacgc

(((.((((((.(((((((((.((((((((((.((.......))...)))))))))).))))))))).)))))).)))

---------UUCAAGUAAUCCAGGAUA--

682 0,-4

---------UUCAAGUAAUCCAGGAUAa---

14 0,-4

---------UUCAAGUAAUCCAGGAUAaa--

6 0,-4

---------UUCAAGUAAUCCAGGAUAG---

1428 0,-3

---------UUCAAGUAAUCCAGGAUAGu--

2 0,-3

---------UUCAAGUAAUCCAGGAUAGG--

1518 0,-2

---------UUCAAGUAAUCCAGGAUAGGa---

5 0,-2

---------UUCAAGUAAUCCAGGAUAGGaa--

2 0,-2

---------UUCAAGUAAUCCAGGAUAGGau--

7 0,-2

---------UUCAAGUAAUCCAGGAUAGGu---

10 0,-2

---------UUCAAGUAAUCCAGGAUAGGuu--

40 0,-2

---------UUCAAGUAAUCCAGGAUAGGC---

731 0,-1

---------UUCAAGUAAUCCAGGAUAGGCa--

965 0,-1

---------UUCAAGUAAUCCAGGAUAGGCaa---

503 0,-1

---------UUCAAGUAAUCCAGGAUAGGCau---

314 0,-1

---------UUCAAGUAAUCCAGGAUAGGCU--

10174 0,0

---------UUCAAGUAAUCCAGGAUAGGCUa---

187 0,0

---------UUCAAGUAAUCCAGGAUAGGCUaa--

271 0,0

---------UUCAAGUAAUCCAGGAUAGGCUau--

745 0,0

---------UUCAAGUAAUCCAGGAUAGGCUu--

455 0,0

---------UUCAAGUAAUCCAGGAUAGGCUuu---

1253 0,0

---------UUCAAGUAAUCCAGGAUAGGCUGa---

2 0,1

----------UCAAGUAAUCCAGGAUAG--

25 1,-3

----------UCAAGUAAUCCAGGAUAGG---

18 1,-2

----------UCAAGUAAUCCAGGAUAGGC--

5 1,-1

----------UCAAGUAAUCCAGGAUAGGCa---

18 1,-1

----------UCAAGUAAUCCAGGAUAGGCaa--

4 1,-1

----------UCAAGUAAUCCAGGAUAGGCau--

5 1,-1

----------UCAAGUAAUCCAGGAUAGGCU---

143 1,0

----------UCAAGUAAUCCAGGAUAGGCUaa---

6 1,0

----------UCAAGUAAUCCAGGAUAGGCUau---

6 1,0

----------UCAAGUAAUCCAGGAUAGGCUu--

6 1,0

----------UCAAGUAAUCCAGGAUAGGCUuu---

9 1,0

-----------CAAGUAAUCCAGGAUAGGC--

2 2,-1

-----------CAAGUAAUCCAGGAUAGGCU---

6 2,0

>hsa-mir-26a-2 MA:14-35;mi:52-73 SRX018959

ggcuguggcuggaUUCAAGUAAUCCAGGAUAGGCUguuuccaucugugaggCCUAUUCUUGAUUACUUGUUUCuggaggc

agcu

(((((..(..((..(((((((((.((((((((((.............)))))))))).)))))))))..))..)..))

))))
------------AUUCAAGUAAUCCAGGAUA---
----   2   -1,-4
------------AUUCAAGUAAUCCAGGAUAGGCU---
----   3   -1,0
-------------UUCAAGUAAUCCAGGAUA---
----   682 0,-4
-------------UUCAAGUAAUCCAGGAUAa--
----   14   0,-4
-------------UUCAAGUAAUCCAGGAUAaa---
----   6   0,-4
-------------UUCAAGUAAUCCAGGAUAG--
----   1428   0,-3
-------------UUCAAGUAAUCCAGGAUAGu---
----   2   0,-3
-------------UUCAAGUAAUCCAGGAUAGG---
----   1518   0,-2
-------------UUCAAGUAAUCCAGGAUAGGa--
----   5   0,-2
-------------UUCAAGUAAUCCAGGAUAGGaa---
----   2   0,-2
-------------UUCAAGUAAUCCAGGAUAGGau---
----   7   0,-2
-------------UUCAAGUAAUCCAGGAUAGGu--
----   10   0,-2
-------------UUCAAGUAAUCCAGGAUAGGuu---
----   40   0,-2
-------------UUCAAGUAAUCCAGGAUAGGC--
----   731 0,-1
-------------UUCAAGUAAUCCAGGAUAGGCa---
----   965 0,-1
-------------UUCAAGUAAUCCAGGAUAGGCaa--
----   503 0,-1
-------------UUCAAGUAAUCCAGGAUAGGCau--
----   314 0,-1
-------------UUCAAGUAAUCCAGGAUAGGCU---
----   10174   0,0
-------------UUCAAGUAAUCCAGGAUAGGCUa--

---- 187 0,0

-------------UUCAAGUAAUCCAGGAUAGGCUaa--

---- 271 0,0

-------------UUCAAGUAAUCCAGGAUAGGCUau--

---- 745 0,0

-------------UUCAAGUAAUCCAGGAUAGGCUu---

---- 455 0,0

-------------UUCAAGUAAUCCAGGAUAGGCUuu--

---- 1253 0,0

-------------UUCAAGUAAUCCAGGAUAGGCUGa--

---- 2 0,1

--------------UCAAGUAAUCCAGGAUAG---

---- 25 1,-3

--------------UCAAGUAAUCCAGGAUAGG--

---- 18 1,-2

--------------UCAAGUAAUCCAGGAUAGGC---

---- 5 1,-1

--------------UCAAGUAAUCCAGGAUAGGCa--

---- 18 1,-1

--------------UCAAGUAAUCCAGGAUAGGCaa---

---- 4 1,-1

--------------UCAAGUAAUCCAGGAUAGGCau---

---- 5 1,-1

--------------UCAAGUAAUCCAGGAUAGGCU--

---- 143 1,0

--------------UCAAGUAAUCCAGGAUAGGCUaa--

---- 6 1,0

--------------UCAAGUAAUCCAGGAUAGGCUau--

---- 6 1,0

--------------UCAAGUAAUCCAGGAUAGGCUu---

---- 6 1,0

--------------UCAAGUAAUCCAGGAUAGGCUuu--

---- 9 1,0

---------------CAAGUAAUCCAGGAUAGGC---

---- 2 2,-1

---------------CAAGUAAUCCAGGAUAGGCU--

---- 6 2,0

>hsa-mir-26b   MA:12-32;mi:47-68   SRX018959

ccgggacccagUUCAAGUAAUUCAGGAUAGGUugugugcuguccagCCUGUUCUCCAUUACUUGGCUCggggaccgg

((((..((((((.((((((((..(((((((((((.(......))))))))))))..))))))))))).)))..))))

-----------UUCAAGUAAUUCAGGAUA--

86 0,-3

-----------UUCAAGUAAUUCAGGAUAG---

669 0,-2

-----------UUCAAGUAAUUCAGGAUAGG--

963 0,-1

-----------UUCAAGUAAUUCAGGAUAGGa---

9   0,-1

-----------UUCAAGUAAUUCAGGAUAGGaa--

2   0,-1

-----------UUCAAGUAAUUCAGGAUAGGau--

3   0,-1

-----------UUCAAGUAAUUCAGGAUAGGU---

1039   0,0

-----------UUCAAGUAAUUCAGGAUAGGUa--

322 0,0

-----------UUCAAGUAAUUCAGGAUAGGUaa---

529 0,0

-----------UUCAAGUAAUUCAGGAUAGGUau---

247 0,0

-----------UUCAAGUAAUUCAGGAUAGGUU--

5055   0,1

-----------UUCAAGUAAUUCAGGAUAGGUUa---

685 0,1

-----------UUCAAGUAAUUCAGGAUAGGUUaa--

42 0,1

-----------UUCAAGUAAUUCAGGAUAGGUUau--

11 0,1

-----------UUCAAGUAAUUCAGGAUAGGUUu---

1427   0,1

-----------UUCAAGUAAUUCAGGAUAGGUUuu--

39 0,1

-----------UUCAAGUAAUUCAGGAUAGGUUG---

18 0,2

-----------UUCAAGUAAUUCAGGAUAGGUUGU--

4 0,3

------------UCAAGUAAUUCAGGAUAG---

12 1,-2

------------UCAAGUAAUUCAGGAUAGG--

19 1,-1

------------UCAAGUAAUUCAGGAUAGGU---

10 1,0

------------UCAAGUAAUUCAGGAUAGGUa--

2 1,0

------------UCAAGUAAUUCAGGAUAGGUau---

3 1,0

------------UCAAGUAAUUCAGGAUAGGUU--

60 1,1

------------UCAAGUAAUUCAGGAUAGGUUa---

7 1,1

------------UCAAGUAAUUCAGGAUAGGUUau--

3 1,1

------------UCAAGUAAUUCAGGAUAGGUUu---

22 1,1

------------UCAAGUAAUUCAGGAUAGGUUG---

2 1,2

-------------CAAGUAAUUCAGGAUAGGUU--

5 2,1

-------------CAAGUAAUUCAGGAUAGGUUu---

6 2,1

-------------CAAGUAAUUCAGGAUAGGUUuu--

2 2,1

--CCUGUUCUCCAUUACUUGGCU----------

2 0,-1

>hsa-mir-27a mi:10-31;MA:51-71 SRX018959

cugaggagcAGGGCUUAGCUGCUUGUGAGCAggguccacaccaagucgugUUCACAGUGGCUAAGUUCCGCcccccag

(((.((.((.(((((((((..((.(((((((.((.(........))).)))))))))..))))))))).)).)).)))

---------AGGGCUUAGCUGCUUGUGAGC--

2 0,-1

---GUUCACAGUGGCUAAGUU-----------

17 -1,-4

---GUUCACAGUGGCUAAGUUu----------

2 -1,-4

---GUUCACAGUGGCUAAGUUC----------

70 -1,-3

--UUCACAGUGGCUAAGUUC----------

10068 0,-3

--UUCACAGUGGCUAAGUUCa---------

7 0,-3

--UUCACAGUGGCUAAGUUCaa--------

2 0,-3

--UUCACAGUGGCUAAGUUCC---------

78 0,-2

--UUCACAGUGGCUAAGUUCCG--------

170 0,-1

--UUCACAGUGGCUAAGUUCCGa-------

16 0,-1

--UUCACAGUGGCUAAGUUCCGaa------

3 0,-1

--UUCACAGUGGCUAAGUUCCGu-------

30 0,-1

--UUCACAGUGGCUAAGUUCCGC-------

10 0,0

--UUCACAGUGGCUAAGUUCCGCaa-----

2 0,0

--UUCACAGUGGCUAAGUUCCGCau-----

2 0,0

--UUCACAGUGGCUAAGUUCCGCC------

2 0,1

>hsa-mir-27b mi:19-40;MA:61-81 SRX018959

accucucuaacaaggugcAGAGCUUAGCUGAUUGGUGAACagugauugguuuccgcuuugUUCACAGUGGCUAAGUUCUG
Caccugaagagaaggug

((((((((....((((((((((((((((((....((((((((....(((...)))..))))))))..)))))))))))))
)))))..)))).)))).

-----------------CAGAGCUUAGCUGAUUGGUGA---
---------------- 2 -1,-2

-----------------CAGAGCUUAGCUGAUUGGUGAA--
---------------- 3 -1,-1

------------------AGAGCUUAGCUGAUUGGU---

----------------- 2 0,-4

------------------AGAGCUUAGCUGAUUGGUG--

----------------- 33 0,-3

------------------AGAGCUUAGCUGAUUGGUGu---

----------------- 2 0,-3

------------------AGAGCUUAGCUGAUUGGUGA---

----------------- 236 0,-2

------------------AGAGCUUAGCUGAUUGGUGAA--

----------------- 156 0,-1

------------------AGAGCUUAGCUGAUUGGUGAAC---

----------------- 18 0,0

------------------AGAGCUUAGCUGAUUGGUGAACA--

----------------- 11 0,1

------------------AGAGCUUAGCUGAUUGGUGAACAu---

----------------- 2 0,1

------------------AGAGCUUAGCUGAUUGGUGAACAG---

----------------- 7 0,2

------------------AGAGCUUAGCUGAUUGGUGAACAGU--

----------------- 6 0,3

--GUUCACAGUGGCUAAGUU---

----------------- 17 -1,-4

--GUUCACAGUGGCUAAGUUu--

----------------- 2 -1,-4

--GUUCACAGUGGCUAAGUUC--

----------------- 70 -1,-3

--GUUCACAGUGGCUAAGUUCU-

----------------- 10 -1,-2

--GUUCACAGUGGCUAAGUUCUG

C---------------- 3 -1,0

--GUUCACAGUGGCUAAGUUCUG

Cu--------------- 2 -1,0

---UUCACAGUGGCUAAGUUC--

----------------- 10068 0,-3

---UUCACAGUGGCUAAGUUCa-

----------------- 7 0,-3

---UUCACAGUGGCUAAGUUCaa

----------------- 2 0,-3

---UUCACAGUGGCUAAGUUCU-

-----------------   2055   0,-2
--UUCACAGUGGCUAAGUUCUa
-----------------   17   0,-2
--UUCACAGUGGCUAAGUUCUa
a----------------   8   0,-2
--UUCACAGUGGCUAAGUUCUu
-----------------   12   0,-2
--UUCACAGUGGCUAAGUUCUu
u----------------   17   0,-2
--UUCACAGUGGCUAAGUUCUG
-----------------   164   0,-1
--UUCACAGUGGCUAAGUUCUG
a----------------   40   0,-1
--UUCACAGUGGCUAAGUUCUG
aa---------------   43   0,-1
--UUCACAGUGGCUAAGUUCUG
au---------------   6   0,-1
--UUCACAGUGGCUAAGUUCUG
u----------------   14   0,-1
--UUCACAGUGGCUAAGUUCUG
uu---------------   12   0,-1
--UUCACAGUGGCUAAGUUCUG
C----------------   379   0,0
--UUCACAGUGGCUAAGUUCUG
Cu---------------   8   0,0
--UUCACAGUGGCUAAGUUCUG
Cuu--------------   2   0,0
--UUCACAGUGGCUAAGUUCUG
CA---------------   100   0,1
--UUCACAGUGGCUAAGUUCUG
CAa--------------   52   0,1
--UUCACAGUGGCUAAGUUCUG
CAaa-------------   4   0,1
--UUCACAGUGGCUAAGUUCUG
CAau-------------   3   0,1
--UUCACAGUGGCUAAGUUCUG
CAu--------------   60   0,1
--UUCACAGUGGCUAAGUUCUG

CAuu------------   11 0,1
--UCACAGUGGCUAAGUUCU-
----------------   18 1,-2
--UCACAGUGGCUAAGUUCUu
----------------   2   1,-2
--UCACAGUGGCUAAGUUCUG
----------------   4   1,-1
--UCACAGUGGCUAAGUUCUG
C---------------   2   1,0
--UCACAGUGGCUAAGUUCUG
CA--------------   2   1,1
--UCACAGUGGCUAAGUUCUG
CAa-------------   2   1,1
--UCACAGUGGCUAAGUUCUG
CAu-------------   3   1,1
--UCACAGUGGCUAAGUUCUG
CAuu------------   5   1,1
---CACAGUGGCUAAGUUCUG
C---------------   2   2,0

>hsa-mir-28 5p:14-35;3p:54-75   SRX018959
gguccuugcccucAAGGAGCUCACAGUCUAUUGAGuuaccuuucugacuuuccCACUAGAUUGUGAGCUCCUGGAgggca
ggcacu
(((.((((((((.((((((((((((((((((.((((((......)))))....)).)))))))))))))))).)))))))
)).)))
------------CAAGGAGCUCACAGUCUAUUG---
------ 4   -1,-2
------------CAAGGAGCUCACAGUCUAUUGA--
------ 3   -1,-1
-------------AAGGAGCUCACAGUCUAU---
------ 5   0,-4
-------------AAGGAGCUCACAGUCUAUa--
------ 7   0,-4
-------------AAGGAGCUCACAGUCUAUU--
------ 75 0,-3
-------------AAGGAGCUCACAGUCUAUUG---
------ 72 0,-2
-------------AAGGAGCUCACAGUCUAUUGu--

------ 2 0,-2

-------------AAGGAGCUCACAGUCUAUUGA---

------ 257 0,-1

-------------AAGGAGCUCACAGUCUAUUGAa--

------ 11 0,-1

-------------AAGGAGCUCACAGUCUAUUGAaa---

------ 2 0,-1

-------------AAGGAGCUCACAGUCUAUUGAu--

------ 2 0,-1

-------------AAGGAGCUCACAGUCUAUUGAG--

------ 153 0,0

-------------AAGGAGCUCACAGUCUAUUGAGa---

------ 30 0,0

-------------AAGGAGCUCACAGUCUAUUGAGU---

------ 7 0,1

-------------AAGGAGCUCACAGUCUAUUGAGUa--

------ 3 0,1

--------------AGGAGCUCACAGUCUAUUGA---

------ 5 1,-1

---CCACUAGAUUGUGAGCUCCUGGA-----

------ 3 -1,0

--CACUAGAUUGUGAGCUCC---------

------ 2 0,-4

--CACUAGAUUGUGAGCUCCU--------

------ 14 0,-3

--CACUAGAUUGUGAGCUCCUaa------

------ 2 0,-3

--CACUAGAUUGUGAGCUCCUG-------

------ 20 0,-2

--CACUAGAUUGUGAGCUCCUGa------

------ 4 0,-2

--CACUAGAUUGUGAGCUCCUGaa-----

------ 2 0,-2

--CACUAGAUUGUGAGCUCCUGu------

------ 2 0,-2

--CACUAGAUUGUGAGCUCCUGG------

------ 310 0,-1

--CACUAGAUUGUGAGCUCCUGGu-----

------ 3 0,-1

--CACUAGAUUGUGAGCUCCUGGuu----

------ 3 0,-1

--CACUAGAUUGUGAGCUCCUGGA-----

------ 1882 0,0

--CACUAGAUUGUGAGCUCCUGGAa----

------ 921 0,0

--CACUAGAUUGUGAGCUCCUGGAaa---

------ 114 0,0

--CACUAGAUUGUGAGCUCCUGGAau---

------ 36 0,0

--CACUAGAUUGUGAGCUCCUGGAu----

------ 46 0,0

--CACUAGAUUGUGAGCUCCUGGAuu---

------ 8 0,0

--CACUAGAUUGUGAGCUCCUGGAG----

------ 13 0,1

--CACUAGAUUGUGAGCUCCUGGAGa---

------ 6 0,1

--CACUAGAUUGUGAGCUCCUGGAGu---

------ 10 0,1

---ACUAGAUUGUGAGCUCCUGG------

------ 10 1,-1

---ACUAGAUUGUGAGCUCCUGGA-----

------ 77 1,0

---ACUAGAUUGUGAGCUCCUGGAa----

------ 38 1,0

---ACUAGAUUGUGAGCUCCUGGAaa---

------ 6 1,0

---ACUAGAUUGUGAGCUCCUGGAu----

------ 2 1,0

---ACUAGAUUGUGAGCUCCUGGAG----

------ 42 1,1

---ACUAGAUUGUGAGCUCCUGGAGa---

------ 37 1,1

---ACUAGAUUGUGAGCUCCUGGAGu---

------ 8 1,1

--CUAGAUUGUGAGCUCCUGGA-----

------ 3 2,0
--CUAGAUUGUGAGCUCCUGGAa----
------ 7 2,0
--CUAGAUUGUGAGCUCCUGGAaa---
------ 3 2,0
--CUAGAUUGUGAGCUCCUGGAG----
------ 16 2,1
--CUAGAUUGUGAGCUCCUGGAGa---
------ 16 2,1
--CUAGAUUGUGAGCUCCUGGAGaa--
------ 5 2,1
--CUAGAUUGUGAGCUCCUGGAGu---
------ 5 2,1
--CUAGAUUGUGAGCUCCUGGAGGa--
------ 2 2,2

>hsa-mir-296 5p:14-34;3p:48-69 SRX018959
aggacccuuccagAGGGCCCCCCCUCAAUCCUGUugugccuaauucaGAGGGUUGGGUGGAGGCUCUCCugaagggcucu
((..((((((..(((((((.((.(((((((((.(((.........))))))))))))).)).)))))))..))))))..))
-------------AGGGCCCCCCCUCAAUCCU--
3 0,-2

>hsa-mir-299 5p:7-28;3p:39-60 SRX018959
aagaaaUGGUUUACCGUCCCACAUACAUuuugaauaugUAUGUGGGAUGGUAAACCGCUUcuu
(((((.((((((((((((((((((((((.......))))))))))))))))))))))).)))))
-----AUGGUUUACCGUCCCACAUACA------------------------------------ 4 -1,-1
------UGGUUUACCGUCCCACAUACAU----------------------------------- 2 0,0
--------------------------------------UAUGUGGGAUGGUAAACCGCUU--- 3 0,0
--------------------------------------UAUGUGGGAUGGUAAACCGCUUu-- 2 0,0

>hsa-mir-29a mi:4-25;MA:42-63 SRX018959
augACUGAUUUCUUUUGGUGUUCAGagucaauauaauuuucUAGCACCAUCUGAAAUCGGUUAu
(((((((((((...(((((((.((((.............)))))))))))...)))))))))))
---ACUGAUUUCUUUUGGUGUUCA-- 10 0,-1
---ACUGAUUUCUUUUGGUGUUCAG--------------------------------------- 4 0,0
---ACUGAUUUCUUUUGGUGUUCAGA-------------------------------------- 6 0,1
---ACUGAUUUCUUUUGGUGUUCAGAG------------------------------------- 2 0,2
--CUAGCACCAUCUGAAAUC------ 4 -1,-5

--CUAGCACCAUCUGAAAUCG----- 12 -1,-4
--CUAGCACCAUCUGAAAUCGG---- 58 -1,-3
--CUAGCACCAUCUGAAAUCGGau-- 3 -1,-3
--CUAGCACCAUCUGAAAUCGGU--- 54 -1,-2
--CUAGCACCAUCUGAAAUCGGUa-- 15 -1,-2
--CUAGCACCAUCUGAAAUCGGUaa- 15 -1,-2
--CUAGCACCAUCUGAAAUCGGUU-- 569 -1,-1
--CUAGCACCAUCUGAAAUCGGUUu- 127 -1,-1
--CUAGCACCAUCUGAAAUCGGUUuu 4 -1,-1
--CUAGCACCAUCUGAAAUCGGUUA- 302 -1,0
--CUAGCACCAUCUGAAAUCGGUUAa 14 -1,0
--CUAGCACCAUCUGAAAUCGGUUAaa 3 -1,0
--CUAGCACCAUCUGAAAUCGGUUAU 17 -1,1
---UAGCACCAUCUGAAAUCG----- 179 0,-4
---UAGCACCAUCUGAAAUCGuu--- 3 0,-4
---UAGCACCAUCUGAAAUCGG---- 3936 0,-3
---UAGCACCAUCUGAAAUCGGa--- 5 0,-3
---UAGCACCAUCUGAAAUCGGau-- 15 0,-3
---UAGCACCAUCUGAAAUCGGU--- 672 0,-2
---UAGCACCAUCUGAAAUCGGUa-- 52 0,-2
---UAGCACCAUCUGAAAUCGGUaa- 687 0,-2
---UAGCACCAUCUGAAAUCGGUau- 57 0,-2
---UAGCACCAUCUGAAAUCGGUU-- 10081 0,-1
---UAGCACCAUCUGAAAUCGGUUu- 5652 0,-1
---UAGCACCAUCUGAAAUCGGUUuu 122 0,-1
---UAGCACCAUCUGAAAUCGGUUA- 58448 0,0
---UAGCACCAUCUGAAAUCGGUUAa 2759 0,0
---UAGCACCAUCUGAAAUCGGUUAaa 17 0,0
---UAGCACCAUCUGAAAUCGGUUAau 12 0,0
---UAGCACCAUCUGAAAUCGGUUAU 1438 0,1
---UAGCACCAUCUGAAAUCGGUUAUa 14 0,1
---UAGCACCAUCUGAAAUCGGUUAUu 12 0,1
--AGCACCAUCUGAAAUCGG---- 42 1,-3
--AGCACCAUCUGAAAUCGGU--- 5 1,-2
--AGCACCAUCUGAAAUCGGUaa- 4 1,-2
--AGCACCAUCUGAAAUCGGUU-- 58 1,-1
--AGCACCAUCUGAAAUCGGUUu- 55 1,-1
--AGCACCAUCUGAAAUCGGUUuu 2 1,-1

--AGCACCAUCUGAAAUCGGUUA- 641 1,0
--AGCACCAUCUGAAAUCGGUUAa 45 1,0
--AGCACCAUCUGAAAUCGGUUAU 17 1,1
---GCACCAUCUGAAAUCGGU--- 2 2,-2
---GCACCAUCUGAAAUCGGUU-- 4 2,-1
---GCACCAUCUGAAAUCGGUUu- 2 2,-1
---GCACCAUCUGAAAUCGGUUA- 24 2,0

>hsa-mir-29b-1 mi:10-33;MA:51-73 SRX018959
cuucaggaaGCUGGUUUCAUAUGGUGGUUUAGAuuuaaauagugauugucUAGCACCAUUUGAAAUCAGUGUUcuugggg
g
(((((((((((((((((((.((((((..((((((.............)))))))))))).)))))))))).)))))))))
.
----------CUGGUUUCAUAUGGUGGUUUAG--
- 2 1,-1
---CUAGCACCAUUUGAAAUCAG-----------
- 4 -1,-4
---CUAGCACCAUUUGAAAUCAGU----------
- 44 -1,-3
---CUAGCACCAUUUGAAAUCAGUu---------
- 3 -1,-3
---CUAGCACCAUUUGAAAUCAGUG---------
- 9 -1,-2
---CUAGCACCAUUUGAAAUCAGUGU--------
- 4 -1,-1
--UAGCACCAUUUGAAAUCA------------
- 127 0,-5
--UAGCACCAUUUGAAAUCAuu----------
- 2 0,-5
--UAGCACCAUUUGAAAUCAG-----------
- 142 0,-4
--UAGCACCAUUUGAAAUCAGa----------
- 6 0,-4
--UAGCACCAUUUGAAAUCAGU----------
- 4714 0,-3
--UAGCACCAUUUGAAAUCAGUaa--------
- 2 0,-3
--UAGCACCAUUUGAAAUCAGUu---------

-   12 0,-3
---UAGCACCAUUUGAAAUCAGUuu-------
-   16 0,-3
---UAGCACCAUUUGAAAUCAGUG--------
-   165 0,-2
---UAGCACCAUUUGAAAUCAGUGa-------
-   5   0,-2
---UAGCACCAUUUGAAAUCAGUGaa------
-   2   0,-2
---UAGCACCAUUUGAAAUCAGUGU-------
-   61 0,-1
---UAGCACCAUUUGAAAUCAGUGUa------
-   12 0,-1
---UAGCACCAUUUGAAAUCAGUGUU------
-   129 0,0
---UAGCACCAUUUGAAAUCAGUGUUa-----
-   9   0,0
--AGCACCAUUUGAAAUCAGU---------
-   43 1,-3
--AGCACCAUUUGAAAUCAGUG--------
-   3   1,-2
--AGCACCAUUUGAAAUCAGUGUU------
-   3   1,0
---GCACCAUUUGAAAUCAGU---------
-   7   2,-3

>hsa-mir-29b-2 mi:11-32;MA:52-74   SRX018959
cuucuggaagCUGGUUUCACAUGGUGGCUUAGauuuuuccaucuuuguaucUAGCACCAUUUGAAAUCAGUGUUuuagga
g
(((((((((((((((((((.((((((.((.((((..............)))))))))))).)))))))))).))))))))
)
---------GCUGGUUUCACAUGGUGGCUUA---
-   2   -1,-1
---------GCUGGUUUCACAUGGUGGCUUAu--
-   3   -1,-1
----------CUGGUUUCACAUGGUGGCUUA---
-   4   0,-1
----------CUGGUUUCACAUGGUGGCUUAG--

-    2   0,0

--CUAGCACCAUUUGAAAUCAG-----------

-    4   -1,-4

--CUAGCACCAUUUGAAAUCAGU----------

-    44   -1,-3

--CUAGCACCAUUUGAAAUCAGUu---------

-    3   -1,-3

--CUAGCACCAUUUGAAAUCAGUG---------

-    9   -1,-2

--CUAGCACCAUUUGAAAUCAGUGU--------

-    4   -1,-1

---UAGCACCAUUUGAAAUCA------------

-    127 0,-5

---UAGCACCAUUUGAAAUCAuu----------

-    2   0,-5

---UAGCACCAUUUGAAAUCAG-----------

-    142 0,-4

---UAGCACCAUUUGAAAUCAGa----------

-    6   0,-4

---UAGCACCAUUUGAAAUCAGU----------

-    4714   0,-3

---UAGCACCAUUUGAAAUCAGUaa--------

-    2   0,-3

---UAGCACCAUUUGAAAUCAGUu---------

-    12   0,-3

---UAGCACCAUUUGAAAUCAGUuu--------

-    16   0,-3

---UAGCACCAUUUGAAAUCAGUG---------

-    165 0,-2

---UAGCACCAUUUGAAAUCAGUGa--------

-    5   0,-2

---UAGCACCAUUUGAAAUCAGUGaa-------

-    2   0,-2

---UAGCACCAUUUGAAAUCAGUGU--------

-    61   0,-1

---UAGCACCAUUUGAAAUCAGUGUa-------

-    12   0,-1

---UAGCACCAUUUGAAAUCAGUGUU-------

- 129 0,0
---UAGCACCAUUUGAAAUCAGUGUUa-----
- 9 0,0
---UAGCACCAUUUGAAAUCAGUGUUU-----
- 10 0,1
--AGCACCAUUUGAAAUCAGU---------
- 43 1,-3
--AGCACCAUUUGAAAUCAGUG--------
- 3 1,-2
--AGCACCAUUUGAAAUCAGUGUU------
- 3 1,0
---GCACCAUUUGAAAUCAGU---------
- 7 2,-3

>hsa-mir-29c mi:16-37;MA:54-75 SRX018959
aucucuuacacaggcUGACCGAUUUCUCCUGGUGUUCagagucuguuuuugucUAGCACCAUUUGAAAUCGGUUAugaug
uaggggga
.((((((((((...((((((((((((...((((((((((((.....)))))...)))))))...))))))))))))).))
))))))).
---------------UGACCGAUUUCUCCUGGUGUU--
-------- 8 0,-1
---------------UGACCGAUUUCUCCUGGUGUUa---
-------- 4 0,-1
----------------GACCGAUUUCUCCUGGUGUU--
-------- 5 1,-1
----------------GACCGAUUUCUCCUGGUGUUa---
-------- 9 1,-1
----------------GACCGAUUUCUCCUGGUGUUu---
-------- 2 1,-1
----------------GACCGAUUUCUCCUGGUGUUC---
-------- 5 1,0
----------------GACCGAUUUCUCCUGGUGUUCA--
-------- 3 1,1
-----------------ACCGAUUUCUCCUGGUGUUC---
-------- 4 2,0
-----------------ACCGAUUUCUCCUGGUGUUCA--
-------- 4 2,1
-----------------ACCGAUUUCUCCUGGUGUUCAG---

-------- 2 2,2

--CUAGCACCAUUUGAAAUCGGUU------

-------- 2 -1,-1

--CUAGCACCAUUUGAAAUCGGUUA-----

-------- 5 -1,0

---UAGCACCAUUUGAAAUCG---------

-------- 106 0,-4

---UAGCACCAUUUGAAAUCGaa-------

-------- 3 0,-4

---UAGCACCAUUUGAAAUCGuu-------

-------- 3 0,-4

---UAGCACCAUUUGAAAUCGG--------

-------- 645 0,-3

---UAGCACCAUUUGAAAUCGGU-------

-------- 191 0,-2

---UAGCACCAUUUGAAAUCGGUa------

-------- 5 0,-2

---UAGCACCAUUUGAAAUCGGUaa-----

-------- 64 0,-2

---UAGCACCAUUUGAAAUCGGUau-----

-------- 3 0,-2

---UAGCACCAUUUGAAAUCGGUU------

-------- 817 0,-1

---UAGCACCAUUUGAAAUCGGUUu-----

-------- 443 0,-1

---UAGCACCAUUUGAAAUCGGUUuu----

-------- 18 0,-1

---UAGCACCAUUUGAAAUCGGUUA-----

-------- 6770 0,0

---UAGCACCAUUUGAAAUCGGUUAa----

-------- 365 0,0

---UAGCACCAUUUGAAAUCGGUUAaa---

-------- 7 0,0

---UAGCACCAUUUGAAAUCGGUUAU----

-------- 145 0,1

---UAGCACCAUUUGAAAUCGGUUAUa---

-------- 3 0,1

--AGCACCAUUUGAAAUCGG--------

-------- 3 1,-3
---AGCACCAUUUGAAAUCGGUU------
-------- 3 1,-1
---AGCACCAUUUGAAAUCGGUUu-----
-------- 3 1,-1
---AGCACCAUUUGAAAUCGGUUA-----
-------- 45 1,0
---AGCACCAUUUGAAAUCGGUUAa----
-------- 2 1,0
---AGCACCAUUUGAAAUCGGUUAau---
-------- 2 1,0
--GCACCAUUUGAAAUCGGUUAU----
-------- 2 2,1
--GCACCAUUUGAAAUCGGUUAUa---
-------- 4 2,1
--GCACCAUUUGAAAUCGGUUAUu---
-------- 12 2,1

>hsa-mir-301a 3p:51-73 SRX018959
acugcuaacgaaugcucugacuuuauugcacuacuguacuuuacagcuagCAGUGCAAUAGUAUUGUCAAAGCaucugaa
agcagg
.(((((..((.(((((.((((.((((((((((.(((((...))))).....))))))))))....)))).))))).))..
))))).
-------------GCUCUGACUUUAUUGCACU--
------ 2
-------------GCUCUGACUUUAUUGCACUAC--
------ 6
-------------GCUCUGACUUUAUUGCACUACU---
------ 15
--CAGUGCAAUAGUAUUGUCAAAG--------
------ 2 0,-1

>hsa-mir-3065 5p:10-32;3p:50-72 SRX018959
cugcccucuUCAACAAAAUCACUGAUGCUGGAgucgccugagucaucacUCAGCACCAGGAUAUUGUUGGAGaggacag
(((.((((((((((((.(((.(((.(((((((.((....)).))......))))).)))))).))))))))))))).)))
---------UCAACAAAAUCACUGAUGC---
4 0,-4
---------UCAACAAAAUCACUGAUGCU--

62 0,-3
---------UCAACAAAAUCACUGAUGCUG--
7 0,-2
---------UCAACAAAAUCACUGAUGCUGGA--
5 0,0
---------UCAACAAAAUCACUGAUGCUGGAGU--
3 0,2
--UCAGCACCAGGAUAUUGU------------
3 0,-5
--UCAGCACCAGGAUAUUGUU-----------
2 0,-4
--UCAGCACCAGGAUAUUGUUG----------
3 0,-3
--UCAGCACCAGGAUAUUGUUGG---------
2 0,-2
--UCAGCACCAGGAUAUUGUUGGAG-------
2 0,0
--UCAGCACCAGGAUAUUGUUGGAGu------
2 0,0

>hsa-mir-30a MA:6-27;mi:47-68 SRX018959
gcgacUGUAAACAUCCUCGACUGGAAGcugugaagccacagaugggCUUUCAGUCGGAUGUUUGCAGCugc
(((.((((((((((((..((((((((((((((....))).....))))))))))))))))))))))).)))
----CUGUAAACAUCCUCGACUGGA-- 3
-1,-2
----CUGUAAACAUCCUCGACUGGAA--- 2
-1,-1
----CUGUAAACAUCCUCGACUGGAAa-- 3
-1,-1
----CUGUAAACAUCCUCGACUGGAAG-- 5 -1,0
----CUGUAAACAUCCUCGACUGGAAGa--- 7 -1,0
----CUGUAAACAUCCUCGACUGGAAGC--- 38 -1,1
----CUGUAAACAUCCUCGACUGGAAGCa-- 4 -1,1
----CUGUAAACAUCCUCGACUGGAAGCU-- 36 -1,2
-----UGUAAACAUCCUCGACUG-- 94 0,-4
-----UGUAAACAUCCUCGACUGG--- 56 0,-3
-----UGUAAACAUCCUCGACUGGA-- 185 0,-2
-----UGUAAACAUCCUCGACUGGAA--- 545 0,-1

-----UGUAAACAUCCUCGACUGGAAa-- 354 0,-1
-----UGUAAACAUCCUCGACUGGAAaa--- 21 0,-1
-----UGUAAACAUCCUCGACUGGAAau--- 2 0,-1
-----UGUAAACAUCCUCGACUGGAAu-- 8 0,-1
-----UGUAAACAUCCUCGACUGGAAG-- 1693
0,0
-----UGUAAACAUCCUCGACUGGAAGa--- 1510
0,0
-----UGUAAACAUCCUCGACUGGAAGaa-- 45 0,0
-----UGUAAACAUCCUCGACUGGAAGau-- 58 0,0
-----UGUAAACAUCCUCGACUGGAAGu--- 100 0,0
-----UGUAAACAUCCUCGACUGGAAGuu-- 8 0,0
-----UGUAAACAUCCUCGACUGGAAGC--- 8322
0,1
-----UGUAAACAUCCUCGACUGGAAGCa-- 995 0,1
-----UGUAAACAUCCUCGACUGGAAGCaa--- 11 0,1
-----UGUAAACAUCCUCGACUGGAAGCau--- 40 0,1
-----UGUAAACAUCCUCGACUGGAAGCU-- 11547
0,2
-----UGUAAACAUCCUCGACUGGAAGCUa--- 60 0,2
-----UGUAAACAUCCUCGACUGGAAGCUu--- 386 0,2
------GUAAACAUCCUCGACUGG--- 3 1,-3
------GUAAACAUCCUCGACUGGAA--- 3 1,-1
------GUAAACAUCCUCGACUGGAAa-- 2 1,-1
------GUAAACAUCCUCGACUGGAAG-- 27 1,0
------GUAAACAUCCUCGACUGGAAGa--- 7 1,0
------GUAAACAUCCUCGACUGGAAGC--- 70 1,1
------GUAAACAUCCUCGACUGGAAGCa-- 7 1,1
------GUAAACAUCCUCGACUGGAAGCU-- 62 1,2
------GUAAACAUCCUCGACUGGAAGCUu--- 2 1,2
-------UAAACAUCCUCGACUGGAAGC--- 6 2,1
-------UAAACAUCCUCGACUGGAAGCU-- 3 2,2
---CUUUCAGUCGGAUGUUUGC------ 2 0,-3
---CUUUCAGUCGGAUGUUUGCA----- 16 0,-2
---CUUUCAGUCGGAUGUUUGCAa---- 3 0,-2
---CUUUCAGUCGGAUGUUUGCAu---- 6 0,-2
---CUUUCAGUCGGAUGUUUGCAuu--- 2 0,-2
---CUUUCAGUCGGAUGUUUGCAG---- 119 0,-1

--CUUUCAGUCGGAUGUUUGCAGu--- 19 0,-1
--CUUUCAGUCGGAUGUUUGCAGC--- 75 0,0
--CUUUCAGUCGGAUGUUUGCAGCa-- 16 0,0
--CUUUCAGUCGGAUGUUUGCAGCaa- 2 0,0
--CUUUCAGUCGGAUGUUUGCAGCau- 21 0,0
---UUUCAGUCGGAUGUUUGCA----- 2 1,-2
---UUUCAGUCGGAUGUUUGCAG---- 23 1,-1
---UUUCAGUCGGAUGUUUGCAGu--- 4 1,-1
---UUUCAGUCGGAUGUUUGCAGC--- 15 1,0
---UUUCAGUCGGAUGUUUGCAGCau- 4 1,0

>hsa-mir-30b MA:17-38;mi:55-76 SRX018959
accaaguuucaguucaUGUAAACAUCCUACACUCAGCUguaauacauggauuggCUGGGAGGUGGAUGUUUACUUCagcu
gacuugga
.(((((..((((((...((((((((((.((.((((((((............))))))))..))))))))))))...))))
))))))).
---------------AUGUAAACAUCCUACACUCAGCU--
-------- 2 -1,0
----------------UGUAAACAUCCUACACUC--
-------- 7 0,-4
----------------UGUAAACAUCCUACACUCA---
-------- 7 0,-3
----------------UGUAAACAUCCUACACUCAG--
-------- 15 0,-2
----------------UGUAAACAUCCUACACUCAGa---
-------- 2 0,-2
----------------UGUAAACAUCCUACACUCAGC---
-------- 36 0,-1
----------------UGUAAACAUCCUACACUCAGCau---
-------- 2 0,-1
----------------UGUAAACAUCCUACACUCAGCU--
-------- 166 0,0
----------------UGUAAACAUCCUACACUCAGCUa---
-------- 23 0,0
----------------UGUAAACAUCCUACACUCAGCUau--
-------- 9 0,0
----------------UGUAAACAUCCUACACUCAGCUu---
-------- 12 0,0

----------------UGUAAACAUCCUACACUCAGCUuu---
--------   2   0,0
-----------------GUAAACAUCCUACACUCAG---
--------   2   1,-2
-----------------GUAAACAUCCUACACUCAGCU---
--------   6   1,0
-----------------GUAAACAUCCUACACUCAGCUu--
--------   2   1,0
---CUGGGAGGUGGAUGUUUA--------
--------   6   0,-4
---CUGGGAGGUGGAUGUUUAa-------
--------   5   0,-4
---CUGGGAGGUGGAUGUUUAaa------
--------   2   0,-4
---CUGGGAGGUGGAUGUUUAau------
--------   2   0,-4
---CUGGGAGGUGGAUGUUUAu-------
--------   3   0,-4
---CUGGGAGGUGGAUGUUUAC-------
--------   38   0,-3
---CUGGGAGGUGGAUGUUUACa------
--------   4   0,-3
---CUGGGAGGUGGAUGUUUACaa-----
--------   5   0,-3
---CUGGGAGGUGGAUGUUUACU------
--------   237   0,-2
---CUGGGAGGUGGAUGUUUACUa-----
--------   10   0,-2
---CUGGGAGGUGGAUGUUUACUaa----
--------   17   0,-2
---CUGGGAGGUGGAUGUUUACUau----
--------   3   0,-2
---CUGGGAGGUGGAUGUUUACUU-----
--------   267   0,-1
---CUGGGAGGUGGAUGUUUACUUa----
--------   67   0,-1
---CUGGGAGGUGGAUGUUUACUUaa---
--------   45   0,-1

--CUGGGAGGUGGAUGUUUACUUau---
--------   13   0,-1
--CUGGGAGGUGGAUGUUUACUUu----
--------   40   0,-1
--CUGGGAGGUGGAUGUUUACUUuu---
--------   8   0,-1
--CUGGGAGGUGGAUGUUUACUUC----
--------   21   0,0
--CUGGGAGGUGGAUGUUUACUUCu---
--------   6   0,0
--CUGGGAGGUGGAUGUUUACUUCA---
--------   4   0,1
---UGGGAGGUGGAUGUUUACUU-----
--------   4   1,-1
---UGGGAGGUGGAUGUUUACUUa----
--------   2   1,-1
---UGGGAGGUGGAUGUUUACUUCu---
--------   3   1,0

>hsa-mir-30c-1 MA:17-39;mi:56-77   SRX018959
accaugcuguagugugUGUAAACAUCCUACACUCUCAGCugugagcucaagguggCUGGGAGAGGGUUGUUUACUCCuuc
ugccaugga
.(((((..((((...(.(((((((.(((...(((((((((.((....))....))))))))).))).))))))).)...)
)))))))).
----------------UGUAAACAUCCUACACUC--
---------   7   0,-5
----------------UGUAAACAUCCUACACUCUC--
---------   6   0,-3
----------------UGUAAACAUCCUACACUCUCA---
---------   35   0,-2
----------------UGUAAACAUCCUACACUCUCAG--
---------   82   0,-1
----------------UGUAAACAUCCUACACUCUCAGa---
---------   15   0,-1
----------------UGUAAACAUCCUACACUCUCAGaa--
---------   2   0,-1
----------------UGUAAACAUCCUACACUCUCAGu---
---------   5   0,-1

----------------UGUAAACAUCCUACACUCUCAGC---
---------   136 0,0
----------------UGUAAACAUCCUACACUCUCAGCa--
---------   24 0,0
----------------UGUAAACAUCCUACACUCUCAGCaa---------------------------------------
---------   4   0,0
----------------UGUAAACAUCCUACACUCUCAGCau---------------------------------------
---------   12 0,0
----------------UGUAAACAUCCUACACUCUCAGCU--
---------   403 0,1
----------------UGUAAACAUCCUACACUCUCAGCUa---------------------------------------
---------   13 0,1
----------------UGUAAACAUCCUACACUCUCAGCUu---------------------------------------
---------   30 0,1
-----------------GUAAACAUCCUACACUCUCAGCUau--------------------------------------
---------   2   1,1
---CUGGGAGAGGGUUGUUUA-------
---------   7   0,-4
---CUGGGAGAGGGUUGUUUAaa-----
---------   2   0,-4
---CUGGGAGAGGGUUGUUUAau-----
---------   2   0,-4
---CUGGGAGAGGGUUGUUUAu------
---------   2   0,-4
---CUGGGAGAGGGUUGUUUAC------
---------   12 0,-3
---CUGGGAGAGGGUUGUUUACa-----
---------   2   0,-3
---CUGGGAGAGGGUUGUUUACU-----
---------   301 0,-2
---CUGGGAGAGGGUUGUUUACUa----
---------   13 0,-2
---CUGGGAGAGGGUUGUUUACUaa---
---------   16 0,-2
---CUGGGAGAGGGUUGUUUACUau---
---------   4   0,-2
---CUGGGAGAGGGUUGUUUACUu----
---------   2   0,-2

---CUGGGAGAGGGUUGUUUACUC----
---------   46 0,-1
---CUGGGAGAGGGUUGUUUACUCC---
---------   5   0,0
---CUGGGAGAGGGUUGUUUACUCCa--
---------   6   0,0
---CUGGGAGAGGGUUGUUUACUCCau-
---------   3   0,0
---CUGGGAGAGGGUUGUUUACUCCU--
---------   10 0,1
--UGGGAGAGGGUUGUUUACU-----
---------   22 1,-2
--UGGGAGAGGGUUGUUUACUC----
---------   4   1,-1
--UGGGAGAGGGUUGUUUACUCCa--
---------   2   1,0
--UGGGAGAGGGUUGUUUACUCCau-
---------   6   1,0
--UGGGAGAGGGUUGUUUACUCCU--
---------   3   1,1

>hsa-mir-30c-2 MA:7-29;mi:47-68   SRX018959
agauacUGUAAACAUCCUACACUCUCAGCuguggaaaguaagaaagCUGGGAGAAGGCUGUUUACUCUuucu
(((....((((((.(((...(((((((((.............))))))))).))).)))))))....)))
-----CUGUAAACAUCCUACACUC--   3
-1,-5
-----CUGUAAACAUCCUACACUCUCAGCU--   2
-1,1
------UGUAAACAUCCUACACUC--   7
0,-5
------UGUAAACAUCCUACACUCUC--   6
0,-3
------UGUAAACAUCCUACACUCUCA---   35
0,-2
------UGUAAACAUCCUACACUCUCAG--   82
0,-1
------UGUAAACAUCCUACACUCUCAGa---   15
0,-1

------UGUAAACAUCCUACACUCUCAGaa-- 2
0,-1
------UGUAAACAUCCUACACUCUCAGu--- 5
0,-1
------UGUAAACAUCCUACACUCUCAGC--- 136
0,0
------UGUAAACAUCCUACACUCUCAGCa-- 24
0,0
------UGUAAACAUCCUACACUCUCAGCaa--- 4
0,0
------UGUAAACAUCCUACACUCUCAGCau--- 12
0,0
------UGUAAACAUCCUACACUCUCAGCU-- 403
0,1
------UGUAAACAUCCUACACUCUCAGCUa--- 13
0,1
------UGUAAACAUCCUACACUCUCAGCUu--- 30
0,1
-------GUAAACAUCCUACACUCUCAGCUau-- 2
1,1
--CUGGGAGAAGGCUGUUUA-------- 8
0,-4
--CUGGGAGAAGGCUGUUUAau------ 2
0,-4
--CUGGGAGAAGGCUGUUUAC------- 36
0,-3
--CUGGGAGAAGGCUGUUUACa------ 3
0,-3
--CUGGGAGAAGGCUGUUUACU------ 875
0,-2
--CUGGGAGAAGGCUGUUUACUa----- 12
0,-2
--CUGGGAGAAGGCUGUUUACUaa---- 7
0,-2
--CUGGGAGAAGGCUGUUUACUau---- 2
0,-2
--CUGGGAGAAGGCUGUUUACUu----- 7
0,-2

---CUGGGAGAAGGCUGUUUACUuu----   3
    0,-2
---CUGGGAGAAGGCUGUUUACUC-----   447
    0,-1
---CUGGGAGAAGGCUGUUUACUCa----   13
    0,-1
---CUGGGAGAAGGCUGUUUACUCU----   74
    0,0
--UGGGAGAAGGCUGUUUACU------   13
    1,-2
--UGGGAGAAGGCUGUUUACUC-----   29
    1,-1
--UGGGAGAAGGCUGUUUACUCU----   3
    1,0
---GGGAGAAGGCUGUUUACU------   2
    2,-2

>hsa-mir-30d   MA:6-27;mi:46-67   SRX018959
guuguUGUAAACAUCCCCGACUGGAAGcuguaagacacagcuaagCUUUCAGUCAGAUGUUUGCUGCuac
...((.(((((((((...(((((((((((...((......)).))))))))))).))))))))).))...
----UUGUAAACAUCCCCGACUGGAAGCU---   5   -1,2
-----UGUAAACAUCCCCGACUG---   77   0,-4
-----UGUAAACAUCCCCGACUGG--   57   0,-3
-----UGUAAACAUCCCCGACUGGA---   172   0,-2
-----UGUAAACAUCCCCGACUGGAA--   670   0,-1
-----UGUAAACAUCCCCGACUGGAAa---   459   0,-1
-----UGUAAACAUCCCCGACUGGAAaa--   49   0,-1
-----UGUAAACAUCCCCGACUGGAAau--   5   0,-1
-----UGUAAACAUCCCCGACUGGAAu---   42   0,-1
-----UGUAAACAUCCCCGACUGGAAuu--   3   0,-1
-----UGUAAACAUCCCCGACUGGAAG---   861   0,0
-----UGUAAACAUCCCCGACUGGAAGa--   1113
    0,0
-----UGUAAACAUCCCCGACUGGAAGaa---   99   0,0
-----UGUAAACAUCCCCGACUGGAAGau---   122   0,0
-----UGUAAACAUCCCCGACUGGAAGu--   107   0,0
-----UGUAAACAUCCCCGACUGGAAGuu---   33   0,0
-----UGUAAACAUCCCCGACUGGAAGC--   3929

0,1

-----UGUAAACAUCCCCGACUGGAAGCa--- 949 0,1

-----UGUAAACAUCCCCGACUGGAAGCaa-- 17 0,1

-----UGUAAACAUCCCCGACUGGAAGCau-- 25 0,1

-----UGUAAACAUCCCCGACUGGAAGCU--- 29188 0,2

-----UGUAAACAUCCCCGACUGGAAGCUa-- 211 0,2

-----UGUAAACAUCCCCGACUGGAAGCUu-- 1527 0,2

-----UGUAAACAUCCCCGACUGGAAGCUG-- 4 0,3

------GUAAACAUCCCCGACUGGA--- 4 1,-2

------GUAAACAUCCCCGACUGGAA-- 11 1,-1

------GUAAACAUCCCCGACUGGAAG--- 40 1,0

------GUAAACAUCCCCGACUGGAAGa-- 26 1,0

------GUAAACAUCCCCGACUGGAAGu-- 4 1,0

------GUAAACAUCCCCGACUGGAAGC-- 63 1,1

------GUAAACAUCCCCGACUGGAAGCa--- 25 1,1

------GUAAACAUCCCCGACUGGAAGCU--- 448 1,2

------GUAAACAUCCCCGACUGGAAGCUa-- 2 1,2

------GUAAACAUCCCCGACUGGAAGCUau--------------------------------------- 3 1,2

------GUAAACAUCCCCGACUGGAAGCUu-- 30 1,2

------GUAAACAUCCCCGACUGGAAGCUuu--------------------------------------- 9 1,2

-------UAAACAUCCCCGACUGGAAGC-- 3 2,1

-------UAAACAUCCCCGACUGGAAGCU--- 6 2,2

>hsa-mir-30e MA:17-38;mi:59-80 SRX018959

gggcagucuuugcuacUGUAAACAUCCUUGACUGGAAGcuguaagguguucagaggagCUUUCAGUCGGAUGUUUACAGCggcaggcugcca

.((((((((..((..((((((((((((..(((((((((((.(...(....)...).))))))))))))))))))))))).
.)))))))))).

--------------CUGUAAACAUCCUUGACUG--- 3 -1,-4

--------------CUGUAAACAUCCUUGACUGGAAG--- 2 -1,0

--------------CUGUAAACAUCCUUGACUGGAAGa-- 4 -1,0

--------------CUGUAAACAUCCUUGACUGGAAGC-- 14 -1,1

---------------CUGUAAACAUCCUUGACUGGAAGCU---

------------ 8 -1,2

----------------UGUAAACAUCCUUGACUG--

------------ 21 0,-4

----------------UGUAAACAUCCUUGACUGG---

------------ 24 0,-3

----------------UGUAAACAUCCUUGACUGGA--

------------ 19 0,-2

----------------UGUAAACAUCCUUGACUGGAA---

------------ 70 0,-1

----------------UGUAAACAUCCUUGACUGGAAa--

------------ 27 0,-1

----------------UGUAAACAUCCUUGACUGGAAu--

------------ 2 0,-1

----------------UGUAAACAUCCUUGACUGGAAG--

------------ 165 0,0

----------------UGUAAACAUCCUUGACUGGAAGa---

------------ 281 0,0

----------------UGUAAACAUCCUUGACUGGAAGaa--

------------ 57 0,0

----------------UGUAAACAUCCUUGACUGGAAGau--

------------ 14 0,0

----------------UGUAAACAUCCUUGACUGGAAGu---

------------ 4 0,0

----------------UGUAAACAUCCUUGACUGGAAGuu--

------------ 5 0,0

----------------UGUAAACAUCCUUGACUGGAAGC---

------------ 1588 0,1

----------------UGUAAACAUCCUUGACUGGAAGCa--

------------ 156 0,1

----------------UGUAAACAUCCUUGACUGGAAGCau---------------------------------------

------------ 3 0,1

----------------UGUAAACAUCCUUGACUGGAAGCU--

------------ 2059 0,2

----------------UGUAAACAUCCUUGACUGGAAGCUa---------------------------------------

------------ 29 0,2

----------------UGUAAACAUCCUUGACUGGAAGCUu---------------------------------------

------------ 263 0,2

----------------UGUAAACAUCCUUGACUGGAAGCUG---------------------------------------

------------ 5 0,3

-----------------GUAAACAUCCUUGACUGGAA---

------------ 6 1,-1

-----------------GUAAACAUCCUUGACUGGAAa--

------------ 2 1,-1

-----------------GUAAACAUCCUUGACUGGAAG--

------------ 26 1,0

-----------------GUAAACAUCCUUGACUGGAAGa---

------------ 12 1,0

-----------------GUAAACAUCCUUGACUGGAAGC---

------------ 140 1,1

-----------------GUAAACAUCCUUGACUGGAAGCa--

------------ 14 1,1

-----------------GUAAACAUCCUUGACUGGAAGCU--

------------ 111 1,2

-----------------GUAAACAUCCUUGACUGGAAGCUa---------------------------------------

------------ 4 1,2

-----------------GUAAACAUCCUUGACUGGAAGCUau--------------------------------------

------------ 2 1,2

-----------------GUAAACAUCCUUGACUGGAAGCUu---------------------------------------

------------ 10 1,2

-----------------GUAAACAUCCUUGACUGGAAGCUuu--------------------------------------

------------ 4 1,2

------------------UAAACAUCCUUGACUGGAAGC---

------------ 4 2,1

------------------UAAACAUCCUUGACUGGAAGCU--

------------ 12 2,2

--CUUUCAGUCGGAUGUUUA----

------------ 18 0,-4

--CUUUCAGUCGGAUGUUUAa---

------------ 2 0,-4

--CUUUCAGUCGGAUGUUUAC---

------------ 8 0,-3

--CUUUCAGUCGGAUGUUUACA--

------------ 34 0,-2

--CUUUCAGUCGGAUGUUUACAa-

------------ 3 0,-2

--CUUUCAGUCGGAUGUUUACAG-
-----------   104 0,-1
--CUUUCAGUCGGAUGUUUACAGa
-----------   11 0,-1
--CUUUCAGUCGGAUGUUUACAGu
-----------   181 0,-1
--CUUUCAGUCGGAUGUUUACAGu
u----------   21 0,-1
--CUUUCAGUCGGAUGUUUACAGC
-----------   168 0,0
--CUUUCAGUCGGAUGUUUACAGC
a----------   116 0,0
--CUUUCAGUCGGAUGUUUACAGC
aa---------   4   0,0
--CUUUCAGUCGGAUGUUUACAGC
au---------   11 0,0
---UUUCAGUCGGAUGUUUACAGC
-----------   6   1,0
---UUUCAGUCGGAUGUUUACAGC
a----------   7   1,0

>hsa-mir-31 MA:8-28;mi:44-65   SRX018959
ggagaggAGGCAAGAUGCUGGCAUAGCUguugaacugggaaccUGCUAUGCCAACAUAUUGCCAUcuuucc
((((((..(((((.(((.(((((((((.(((........)))..))))))))).))).)))))..))))))
-------AGGCAAGAUGCUGGCAUAGCU--- 6   0,0

>hsa-mir-3121   3p:47-68   SRX018959
aaaugguuauguccuuugccuauucuauuuaagacacccuguaccuUAAAUAGAGUAGGCAAAGGACAgaaacauuu
(((((....((((((((((((((((((((((((((.....))..))))))))))))))))))))))))....)))))
--UAAAUAGAGUAGGCAAAG-------------
    2   0,-4
--UAAAUAGAGUAGGCAAAGG------------
    2   0,-3
--UAAAUAGAGUAGGCAAAGGA-----------
    2   0,-2

>hsa-mir-3124   5p:7-27 SRX018959
gcgggcUUCGCGGGCGAAGGCAAAGUCgauuuccaaaagugacuuuccucacucccgugaagucggc

((.((((((((((.(.(((.((((((.((((....))))))))))))..).))))))))))).))
------UUCGCGGGCGAAGGCAAAGU--------------------------------------- 2 0,-1
------UUCGCGGGCGAAGGCAAAGUCa------------------------------------- 3 0,0

>hsa-mir-3127 5p:11-33 SRX018959
ggccaggcccAUCAGGGCUUGUGGAAUGGGAAGgagaagggacgcuuccccuucugcaggccugcuggguguggcu
((((.(((((...(((((((..(((.(((((((........).))))))).)))..))))))))..))))).)))))
----------AUCAGGGCUUGUGGAAUGGGAAG---
 2 0,0

>hsa-mir-3129 5p:9-30 SRX018959
guacuuggGCAGUAGUGUAGAGAUUGGUUUgccuguuaaugaauucaaacuaaucucuacacugcugcccaagagc
...(((((((((((((((((((((((((((...((((....))).)))))))))))))))))))))))))))))...
---------CAGUAGUGUAGAGAUUGGUUUGu--
 2 1,1
----------AGUAGUGUAGAGAUUGGUUUG---
 2 2,1
----------AGUAGUGUAGAGAUUGGUUUGC--
 4 2,2

>hsa-mir-3130-1 5p:13-33;3p:44-64 SRX018959
cuugucaugucuUACCCAGUCUCCGGUGCAGCCuguugucaagGCUGCACCGGAGACUGGGUAAgacaugacaag
((((((((((((((((((((((((((((((((.......)))))))))))))))))))))))))))))))))
---GCUGCACCGGAGACUGGG-------------- 2
 0,-3
---GCUGCACCGGAGACUGGGUAA----------- 16
 0,0

>hsa-mir-3130-2 5p:13-33;3p:44-64 SRX018959
cuugucaugucuUACCCAGUCUCCGGUGCAGCCuugacaacagGCUGCACCGGAGACUGGGUAAgacaugacaag
((((((((((((((((((((((((((((((((.......)))))))))))))))))))))))))))))))))
---GCUGCACCGGAGACUGGG-------------- 2
 0,-3
---GCUGCACCGGAGACUGGGUAA----------- 16
 0,0

>hsa-mir-3141 5p:10-28 SRX018959
ucacccgguGAGGGCGGGUGGAGGAGGAgggucccccaccaucagccuucacugggacggga

...((((((((((((.(((((.((.((.....)))).))))).))))))))))))......
---------GAGGGCGGGUGGAGGAGGAa------------------------------- 2 0,0

>hsa-mir-3145 3p:49-72 SRX018959
uauaugaguucaacuccaaacacucaaaacucauuguugaauggaaugAGAUAUUUUGAGUGUUUGGAAUUGaacucgua
ua
((((((((((((.((((((((((((((((((((((.(.....).))))))...))))))))))))))).))))))))))))
))
-----------AACUCCAAACACUCAAAACU---
-- 3
-----------AACUCCAAACACUCAAAACUCA---
-- 2
--AGAUAUUUUGAGUGUUUGGAAUU---------
-- 2 0,-1
--AGAUAUUUUGAGUGUUUGGAAUUG--------
-- 2 0,0

>hsa-mir-3150 3p:49-70 SRX018959
gggaagcaggccaaccucgacgaucuccucagcaccugaacgccaaggCUGGGGAGAUCCUCGAGGUUGGccugcuuucc
(((((((((((((((((((.((((((((((((.(.((.....)).))))))))))))).)))))))))))))))))))))
------------AACCUCGACGAUCUCCUCAGCA--
5

>hsa-mir-3154 3p:54-75 SRX018959
ggccccuccuucucagccccagcucccgcucaccccugccacgucaaaggaggCAGAAGGGGAGUUGGGAGCAGAgaggg
gacc
((((((((..(((..((((((((((((.((.....(((((.(......)..))))).)))))))))))).))))))))))
).))
---CAGAAGGGGAGUUGGGAGCAG------
---- 2 0,-1

>hsa-mir-3158-1 3p:50-71 SRX018959
auucaggccgguccugcagagaggaagcccuucugcuuacagguauuggAAGGGCUUCCUCUCUGCAGGACcggccugaa
u
((((((((((((((((((((((((((((((((((((((....)))...))))))))))))))))))))))))))))))))))
)
--AGGGCUUCCUCUCUGCAGGAC---------
- 2 1,0

>hsa-mir-3158-2 3p:50-71 SRX018959
auucaggccgguccugcagagaggaagcccuuccaauaccuguaagcagAAGGGCUUCCUCUCUGCAGGACcggccugaa
u
(((((((((((((((((((((((((((((((((..............)))))))))))))))))))))))))))))))))
)
---AGGGCUUCCUCUCUGCAGGAC---------
- 2 1,0

>hsa-mir-3159 5p:10-31 SRX018959
ccaaaguccUAGGAUUACAAGUGUCGGCCACgggcugggcacaguggcucacgccuguaaucccagcauuuugg
(((((((.((.((((((((.((((.((((((..((...))...)))))).)))).)))))))).)).)))))))
---CUCACGCCUGUAAUCCCAGCA------ 3

>hsa-mir-3163 5p:10-31 SRX018959
uuccucaucUAUAAAAUGAGGGCAGUAAGACcuuccuuccuugucuuacuacccccauuuuauagaugaggaa
.(((((((((((((((((.(((.((((((((...........)))))))).))).))))))))))))))))).
---------UAUAAAAUGAGGGCAGUAAG-- 3
0,-2

>hsa-mir-3164 5p:10-31 SRX018959
cuuggaaacUGUGACUUUAAGGGAAAUGGCGcacagcagacccugcaaucaugccguuuugcuugaagucgcaguuuccc
agg
((.((((((((((((((((((.(((((((((....((((...)))).....))))))))).)))))))))))))))))).
)).
---------UGUGACUUUAAGGGAAAUGGCG---
--- 3 0,0

>hsa-mir-3165 5p:10-31 SRX018959
ccgguggcaAGGUGGAUGCAAUGUGACCUCAacucuugguccucugaggucacauuguauccaccuuaccacugg
(((((((.(((((((((((((((((((((((((.....))....))))))))))))))))))))))).)))))))
---------AGGUGGAUGCAAUGUGACCU--- 3
0,-2

>hsa-mir-3170 5p:10-31 SRX018959
cugguaacaCUGGGGUUCUGAGACAGACAGUguuagcuccagaagcauugccugucuuagaaccccuauguuaccag
(((((((((..(((((((((((((((.(((((((.........))))))).)))))))))))))))..)))))))))
---------CUGGGGUUCUGAGACAGACAGU---

6 0,0
---------CUGGGGUUCUGAGACAGACAGUa---
2 0,0

>hsa-mir-3173 3p:43-64 SRX018959
ucccugcccugccuguuuucuccuuugugauuuuaugagaacAAAGGAGGAAAUAGGCAGGCCAggga
((((((.(((((((((((((((((((((..((....))..))))))))))))))))))))))).))))))
--AAAGGAGGAAAUAGGCAGGCCAuu-- 2 0,0
---AAGGAGGAAAUAGGCAGGCCA---- 2 1,0
---AAGGAGGAAAUAGGCAGGCCAaa-- 2 1,0

>hsa-mir-3179-1 3p:52-73 SRX018959
caggaucacagacguuuaaauuacacuccuucugcugugccuuacagcaguAGAAGGGGUGAAAUUUAAACGUcugugau
ccug
((((((((((((((((((((.(((((((((((((((((........))))))))))))))))).))))))))))))))))
))))
--UAGAAGGGGUGAAAUUUAAACG--------
---- 3 -1,-1
--UAGAAGGGGUGAAAUUUAAACGU-------
---- 4 -1,0
---AGAAGGGGUGAAAUUUAAACGU-------
---- 8 0,0
---AGAAGGGGUGAAAUUUAAACGUa------
---- 2 0,0

>hsa-mir-3179-2 3p:52-73 SRX018959
caggaucacagacguuuaaauuacacuccuucugcugugccuuacagcaguAGAAGGGGUGAAAUUUAAACGUcugugau
ccug
((((((((((((((((((((.(((((((((((((((((........))))))))))))))))).))))))))))))))))
))))
--UAGAAGGGGUGAAAUUUAAACG--------
---- 3 -1,-1
--UAGAAGGGGUGAAAUUUAAACGU-------
---- 4 -1,0
---AGAAGGGGUGAAAUUUAAACGU-------
---- 8 0,0
---AGAAGGGGUGAAAUUUAAACGUa------
---- 2 0,0

>hsa-mir-3179-3 3p:52-73   SRX018959
caggaucacagacguuuaaauuacacuccuucugcugugccuuacagcaguAGAAGGGGUGAAAUUUAAACGUcugugau
ccug
((((((((((((((((((((((.(((((((((((((((........)))))))))))))))).)))))))))))))))))
))))
--UAGAAGGGGUGAAAUUUAAACG--------
----   3   -1,-1
--UAGAAGGGGUGAAAUUUAAACGU-------
----   4   -1,0
---AGAAGGGGUGAAAUUUAAACGU-------
----   8   0,0
---AGAAGGGGUGAAAUUUAAACGUa------
----   2   0,0

>hsa-mir-3190   3p:47-69   SRX018959
cuggggucaccugucuggccagcuacguccccacggcccuugucagUGUGGAAGGUAGACGGCCAGAGAggugaccccgg
((((((((((((.(((((((..((((.(((.((((((....))).))).)))..))))..))))))).))))))))))))
---GUGUGGAAGGUAGACGGCCAGA-------------
    2   -1,-2

>hsa-mir-3192   5p:10-32   SRX018959
ggaagggauUCUGGGAGGUUGUAGCAGUGGAAaaaguucuuuucuuccucugaucgcccucucagcucuuuccuucu
((((((((..((((((((.((...(((.((((.(((....))).)))).)))..)).))))))))....))))))))
---------UCUGGGAGGUUGUAGCAGU---
    2   0,-4

>hsa-mir-32 MA:6-27;mi:47-68   SRX018959
ggagaUAUUGCACAUUACUAAGUUGCAuguugucacggccucaaugCAAUUUAGUGUGUGUGAUAUUUuc
(((((((..(((((.((((((((((((((..((....))..).))))))))))))))))))..)))))))
-----UAUUGCACAUUACUAAGU---   7   0,-4
-----UAUUGCACAUUACUAAGUU--   14   0,-3
-----UAUUGCACAUUACUAAGUUG---   48   0,-2
-----UAUUGCACAUUACUAAGUUGa--   2   0,-2
-----UAUUGCACAUUACUAAGUUGC--   21   0,-1
-----UAUUGCACAUUACUAAGUUGCA---   2   0,0
-----UAUUGCACAUUACUAAGUUGCAa--   2   0,0
-----UAUUGCACAUUACUAAGUUGCAU--   2   0,1

-----UAUUGCACAUUACUAAGUUGCAUu--- 2 0,1
------AUUGCACAUUACUAAGUUG--- 3 1,-2
------AUUGCACAUUACUAAGUUGC-- 2 1,-1
--CAAUUUAGUGUGUGUGAUAUa--- 3 0,-2
--CAAUUUAGUGUGUGUGAUAUU--- 4 0,-1

>hsa-mir-3200 5p:13-34;3p:54-75 SRX018959
gguggucgagggAAUCUGAGAAGGCGCACAAGGUuuguguccaauacaguccaCACCUUGCGCUACUCAGGUCUGcucgu
gcccu
((.(((((((.(.(((((((..((((((..((((..(((..(......)..))))))))))))).))))))).).)))).
)))))
---CACCUUGCGCUACUCAGGUCU------
----- 2 0,-1

>hsa-mir-320a 3p:48-69 SRX018959
gcuucgcuccccuccgccuucucuucccgguucuucccggagucgggAAAAGCUGGGUUGAGAGGGCGAaaaaggaugag
gu
((((((.(((....(((((((((..(((((((.((((((....)))))).)))))))..))))))))).....)))))))
))
--GAAAAGCUGGGUUGAGAG----------------
-- 2 -1,-5
--GAAAAGCUGGGUUGAGAGG---------------
-- 5 -1,-3
--GAAAAGCUGGGUUGAGAGGGC-------------
-- 27 -1,-2
--GAAAAGCUGGGUUGAGAGGGCG------------
-- 21 -1,-1
--GAAAAGCUGGGUUGAGAGGGCGu-----------
-- 27 -1,-1
--GAAAAGCUGGGUUGAGAGGGCGuu----------
-- 6 -1,-1
--GAAAAGCUGGGUUGAGAGGGCGA-----------
-- 107 -1,0
--GAAAAGCUGGGUUGAGAGGGCGAu----------
-- 10 -1,0
--GAAAAGCUGGGUUGAGAGGGCGAA----------
-- 3 -1,1
---AAAAGCUGGGUUGAGAGG---------------

-- 400 0,-4

---AAAAGCUGGGUUGAGAGGG--------------

-- 476 0,-3

---AAAAGCUGGGUUGAGAGGGa-------------

-- 20 0,-3

---AAAAGCUGGGUUGAGAGGGaa------------

-- 13 0,-3

---AAAAGCUGGGUUGAGAGGGau------------

-- 4 0,-3

---AAAAGCUGGGUUGAGAGGGC-------------

-- 1775 0,-2

---AAAAGCUGGGUUGAGAGGGCu------------

-- 7 0,-2

---AAAAGCUGGGUUGAGAGGGCG------------

-- 2169 0,-1

---AAAAGCUGGGUUGAGAGGGCGu-----------

-- 2034 0,-1

---AAAAGCUGGGUUGAGAGGGCGuu----------

-- 913 0,-1

---AAAAGCUGGGUUGAGAGGGCGA-----------

-- 16375 0,0

---AAAAGCUGGGUUGAGAGGGCGAu----------

-- 4505 0,0

---AAAAGCUGGGUUGAGAGGGCGAuu---------

-- 723 0,0

---AAAAGCUGGGUUGAGAGGGCGAA----------

-- 5941 0,1

---AAAAGCUGGGUUGAGAGGGCGAAu---------

-- 842 0,1

---AAAAGCUGGGUUGAGAGGGCGAAuu--------

-- 169 0,1

---AAAAGCUGGGUUGAGAGGGCGAAA---------

-- 757 0,2

---AAAAGCUGGGUUGAGAGGGCGAAAu--------

-- 238 0,2

---AAAAGCUGGGUUGAGAGGGCGAAAA--------

-- 70 0,3

--AAAGCUGGGUUGAGAGGG--------------

-- 27 1,-3

---AAAGCUGGGUUGAGAGGGa-------------

-- 4 1,-3

---AAAGCUGGGUUGAGAGGGC-------------

-- 83 1,-2

---AAAGCUGGGUUGAGAGGGCG------------

-- 146 1,-1

---AAAGCUGGGUUGAGAGGGCGu-----------

-- 70 1,-1

---AAAGCUGGGUUGAGAGGGCGuu----------

-- 66 1,-1

---AAAGCUGGGUUGAGAGGGCGA-----------

-- 911 1,0

---AAAGCUGGGUUGAGAGGGCGAu----------

-- 287 1,0

---AAAGCUGGGUUGAGAGGGCGAuu---------

-- 67 1,0

---AAAGCUGGGUUGAGAGGGCGAA----------

-- 732 1,1

---AAAGCUGGGUUGAGAGGGCGAAu---------

-- 190 1,1

---AAAGCUGGGUUGAGAGGGCGAAuu--------

-- 58 1,1

---AAAGCUGGGUUGAGAGGGCGAAA---------

-- 187 1,2

---AAAGCUGGGUUGAGAGGGCGAAAu--------

-- 73 1,2

---AAAGCUGGGUUGAGAGGGCGAAAuu-------

-- 6 1,2

---AAAGCUGGGUUGAGAGGGCGAAAA--------

-- 23 1,3

---AAAGCUGGGUUGAGAGGGCGAAAAA-------

-- 2 1,4

--AAGCUGGGUUGAGAGGGC-------------

-- 3 2,-2

--AAGCUGGGUUGAGAGGGCG------------

-- 4 2,-1

--AAGCUGGGUUGAGAGGGCGA-----------

-- 60 2,0
---AAGCUGGGUUGAGAGGGCGAu----------
-- 10 2,0
---AAGCUGGGUUGAGAGGGCGAuu---------
-- 5 2,0
---AAGCUGGGUUGAGAGGGCGAA----------
-- 100 2,1
---AAGCUGGGUUGAGAGGGCGAAu---------
-- 26 2,1
---AAGCUGGGUUGAGAGGGCGAAuu--------
-- 4 2,1
---AAGCUGGGUUGAGAGGGCGAAA---------
-- 86 2,2
---AAGCUGGGUUGAGAGGGCGAAAu--------
-- 24 2,2
---AAGCUGGGUUGAGAGGGCGAAAuu-------
-- 2 2,2
---AAGCUGGGUUGAGAGGGCGAAAA--------
-- 6 2,3
---AAGCUGGGUUGAGAGGGCGAAAAA-------
-- 3 2,4

>hsa-mir-320b-1 3p:39-60 SRX018959
aauuaaucccucucuuucuaguucuuccuagagugaggAAAAGCUGGGUUGAGAGGGCAAacaaauuaacuaauuaauu
........((((...((((((......)))))).))))...
-------------------------------------GAAAAGCUGGGUUGAGAG------------------------
2 -1,-5
-------------------------------------GAAAAGCUGGGUUGAGAGGG----------------------
5 -1,-3
-------------------------------------GAAAAGCUGGGUUGAGAGGGC---------------------
27 -1,-2
-------------------------------------GAAAAGCUGGGUUGAGAGGGCAu-------------------
5 -1,-1
-------------------------------------GAAAAGCUGGGUUGAGAGGGCAA-------------------
3 -1,0
--------------------------------------AAAAGCUGGGUUGAGAGG-----------------------
400 0,-4
--------------------------------------AAAAGCUGGGUUGAGAGGG----------------------

476 0,-3

--------------------------------------AAAAGCUGGGUUGAGAGGGa---------------------

20 0,-3

--------------------------------------AAAAGCUGGGUUGAGAGGGaa--------------------

13 0,-3

--------------------------------------AAAAGCUGGGUUGAGAGGGau--------------------

4 0,-3

--------------------------------------AAAAGCUGGGUUGAGAGGGC---------------------

1775 0,-2

--------------------------------------AAAAGCUGGGUUGAGAGGGCu--------------------

7 0,-2

--------------------------------------AAAAGCUGGGUUGAGAGGGCA--------------------

235 0,-1

--------------------------------------AAAAGCUGGGUUGAGAGGGCAu-------------------

295 0,-1

--------------------------------------AAAAGCUGGGUUGAGAGGGCAuu------------------

95 0,-1

--------------------------------------AAAAGCUGGGUUGAGAGGGCAA-------------------

254 0,0

--------------------------------------AAAAGCUGGGUUGAGAGGGCAAu------------------

77 0,0

--------------------------------------AAAAGCUGGGUUGAGAGGGCAAuu-----------------

4 0,0

--------------------------------------AAAAGCUGGGUUGAGAGGGCAAA------------------

32 0,1

--------------------------------------AAAAGCUGGGUUGAGAGGGCAAAu-----------------

9 0,1

---------------------------------------AAAGCUGGGUUGAGAGGG----------------------

27 1,-3

---------------------------------------AAAGCUGGGUUGAGAGGGa---------------------

4 1,-3

---------------------------------------AAAGCUGGGUUGAGAGGGC---------------------

83 1,-2

---------------------------------------AAAGCUGGGUUGAGAGGGCA--------------------

6 1,-1

---------------------------------------AAAGCUGGGUUGAGAGGGCAu-------------------

14 1,-1

---------------------------------------AAAGCUGGGUUGAGAGGGCAuu------------------

3 1,-1
---------------------------------------AAAGCUGGGUUGAGAGGGCAA-------------------
13 1,0
---------------------------------------AAAGCUGGGUUGAGAGGGCAAu------------------
4 1,0
---------------------------------------AAAGCUGGGUUGAGAGGGCAAA------------------
6 1,1
--AAGCUGGGUUGAGAGGGC---------------------
3 2,-2

>hsa-mir-320b-2 3p:72-93 SRX018959
uguuauuuuuugucuucuaccuaagaauucugucucuuaggcuuucucuucccagauuucccaaaguugggAAAAGCUGG
GUUGAGAGGGCAAaaggaaaaaaaaagaauucugucucugacauaauuagauagggaa
.......................((((((((.((.(((..((((((((..(((((.(((((((....)))))))..))))
)..))))))))..)))))......))))))))..........................
--GAAAAGCUGG
GUUGAGAG-- 2 -1,-5
--GAAAAGCUGG
GUUGAGAGGG-- 5 -1,-3
--GAAAAGCUGG
GUUGAGAGGGC--- 27 -1,-2
--GAAAAGCUGG
GUUGAGAGGGCAu--- 5 -1,-1
--GAAAAGCUGG
GUUGAGAGGGCAA--- 3 -1,0
---AAAAGCUGG
GUUGAGAGG--- 400 0,-4
---AAAAGCUGG
GUUGAGAGGG-- 476 0,-3
---AAAAGCUGG
GUUGAGAGGGa--- 20 0,-3
---AAAAGCUGG
GUUGAGAGGGaa-- 13 0,-3
---AAAAGCUGG
GUUGAGAGGGau-- 4 0,-3
---AAAAGCUGG
GUUGAGAGGGC--- 1775 0,-2
---AAAAGCUGG

GUUGAGAGGGCu-- 7 0,-2
--AAAAGCUGG
GUUGAGAGGGCA-- 235 0,-1
--AAAAGCUGG
GUUGAGAGGGCAu--- 295 0,-1
--AAAAGCUGG
GUUGAGAGGGCAuu-- 95 0,-1
--AAAAGCUGG
GUUGAGAGGGCAA--- 254 0,0
--AAAAGCUGG
GUUGAGAGGGCAAu-- 77 0,0
--AAAAGCUGG
GUUGAGAGGGCAAuu--- 4 0,0
--AAAAGCUGG
GUUGAGAGGGCAAA-- 32 0,1
--AAAAGCUGG
GUUGAGAGGGCAAAu--- 9 0,1
--AAAAGCUGG
GUUGAGAGGGCAAAA--- 9 0,2
---AAAGCUGG
GUUGAGAGGG-- 27 1,-3
---AAAGCUGG
GUUGAGAGGGa--- 4 1,-3
---AAAGCUGG
GUUGAGAGGGC--- 83 1,-2
---AAAGCUGG
GUUGAGAGGGCA-- 6 1,-1
---AAAGCUGG
GUUGAGAGGGCAu--- 14 1,-1
---AAAGCUGG
GUUGAGAGGGCAuu-- 3 1,-1
---AAAGCUGG
GUUGAGAGGGCAA--- 13 1,0
---AAAGCUGG
GUUGAGAGGGCAAu-- 4 1,0
---AAAGCUGG
GUUGAGAGGGCAAA-- 6 1,1
--AAGCUGG

```
GUUGAGAGGGC------------------------------------------  3    2,-2

>hsa-mir-320c-1 3p:50-69     SRX018959
uuugcauuaaaaaugaggccuucucuucccaguucuucccagagucaggAAAAGCUGGGUUGAGAGGGUagaaaaaaaau
gauguagg
.((((((((........((((((((..(((((((.((((........)))).)))))))..))))))))..........)
))))))).
------------------------------------------------GAAAAGCUGGGUUGAGAG--------------
--------    2    -1,-3
------------------------------------------------GAAAAGCUGGGUUGAGAGGG------------
--------    5    -1,-1
------------------------------------------------GAAAAGCUGGGUUGAGAGGGU-----------
--------    2    -1,0
-------------------------------------------------AAAAGCUGGGUUGAGAGG-------------
--------    400  0,-2
-------------------------------------------------AAAAGCUGGGUUGAGAGGG------------
--------    476  0,-1
-------------------------------------------------AAAAGCUGGGUUGAGAGGGa-----------
--------    20   0,-1
-------------------------------------------------AAAAGCUGGGUUGAGAGGGaa----------
--------    13   0,-1
-------------------------------------------------AAAAGCUGGGUUGAGAGGGau----------
--------    4    0,-1
-------------------------------------------------AAAAGCUGGGUUGAGAGGGU-----------
--------    334  0,0
-------------------------------------------------AAAAGCUGGGUUGAGAGGGUu----------
--------    9    0,0
-------------------------------------------------AAAAGCUGGGUUGAGAGGGUuu---------
--------    9    0,0
-------------------------------------------------AAAAGCUGGGUUGAGAGGGUA----------
--------    6    0,1
-------------------------------------------------AAAAGCUGGGUUGAGAGGGUAa---------
--------    21   0,1
-------------------------------------------------AAAAGCUGGGUUGAGAGGGUAau--------
--------    2    0,1
-------------------------------------------------AAAAGCUGGGUUGAGAGGGUAu---------
--------    18   0,1
-------------------------------------------------AAAAGCUGGGUUGAGAGGGUAuu--------
```

--------	4	0,1
--AAAGCUGGGUUGAGAGGG------------
--------	27	1,-1
--AAAGCUGGGUUGAGAGGGa-----------
--------	4	1,-1
--AAAGCUGGGUUGAGAGGGUA----------
--------	2	1,1
--AAAGCUGGGUUGAGAGGGUAu---------
--------	2	1,1

>hsa-mir-320c-2 3p:31-50	SRX018959
cuucucuuuccaguucuucccagaauugggAAAAGCUGGGUUGAGAGGGU
((((((..(((((((.(((((......))))).)))))))..))))))..
-----------------------------GAAAAGCUGGGUUGAGAG---	2	-1,-3
-----------------------------GAAAAGCUGGGUUGAGAGGG-	5	-1,-1
-----------------------------GAAAAGCUGGGUUGAGAGGGU	2	-1,0
------------------------------AAAAGCUGGGUUGAGAGG--	400	0,-2
------------------------------AAAAGCUGGGUUGAGAGGG-	476	0,-1
------------------------------AAAAGCUGGGUUGAGAGGGa	20	0,-1
------------------------------AAAAGCUGGGUUGAGAGGGaa	13	0,-1
------------------------------AAAAGCUGGGUUGAGAGGGau	4	0,-1
------------------------------AAAAGCUGGGUUGAGAGGGU	334	0,0
------------------------------AAAAGCUGGGUUGAGAGGGUu	9	0,0
------------------------------AAAAGCUGGGUUGAGAGGGUuu	9	0,0
-------------------------------AAAGCUGGGUUGAGAGGG-	27	1,-1
-------------------------------AAAGCUGGGUUGAGAGGGa	4	1,-1

>hsa-mir-320d-1 3p:30-48	SRX018959
uucucgucccaguucuucccaaaguugagAAAAGCUGGGUUGAGAGGA
((((((.(((((((.(((.((....)).))).))))))).))))))..
----------------------------GAAAAGCUGGGUUGAGAG--	2	-1,-2
-----------------------------AAAAGCUGGGUUGAGAGG-	400	0,-1
-----------------------------AAAAGCUGGGUUGAGAGGA	18	0,0
-----------------------------AAAAGCUGGGUUGAGAGGAa	6	0,0
-----------------------------AAAAGCUGGGUUGAGAGGAaa	5	0,0
------------------------------AAAGCUGGGUUGAGAGGA	2	1,0

>hsa-mir-320d-2 3p:30-48	SRX018959

uucucuucccaguucuucuuggagucaggAAAAGCUGGGUUGAGAGGA
(((((..(((((((.((((((....)))))).)))))))..)))))..
----------------------------GAAAAGCUGGGUUGAGAG--   2   -1,-2
-----------------------------AAAAGCUGGGUUGAGAGG-   400 0,-1
-----------------------------AAAAGCUGGGUUGAGAGGA   18   0,0
-----------------------------AAAAGCUGGGUUGAGAGGAa   6   0,0
-----------------------------AAAAGCUGGGUUGAGAGGAaa   5   0,0
------------------------------AAAGCUGGGUUGAGAGGA   2   1,0

>hsa-mir-320e   3p:35-52   SRX018959
gccuucucuucccaguucuuccuggagucggggaAAAGCUGGGUUGAGAAGGu
((((((((..(((((((.(((((.(...).))))).))))))).)))))))))
---------------------------------AAAAGCUGGGUUGAGAAGG-   10   -1,0

>hsa-mir-323   5p:16-37;3p:51-71   SRX018959
uugguacuuggagagAGGUGGUCCGUGGCGCGUUCGCuuuauuuauggcgCACAUUACACGGUCGACCUCUuugcaguau
cuaauc
..((((((.(.((((((((.(.(((((....((.((((........)))).))....))))).).)))))))).))))))
).....
---GCACAUUACACGGUCGAC-------------
------ 2   -1,-4
---GCACAUUACACGGUCGACC------------
------ 2   -1,-3
---GCACAUUACACGGUCGACCU-----------
------ 45   -1,-2
---GCACAUUACACGGUCGACCUC----------
------ 6   -1,-1
---GCACAUUACACGGUCGACCUCU---------
------ 3   -1,0
--CACAUUACACGGUCGACC------------
------ 10   0,-3
--CACAUUACACGGUCGACCU-----------
------ 93   0,-2
--CACAUUACACGGUCGACCUCU---------
------ 6   0,0
---ACAUUACACGGUCGACCUC----------
------ 2   1,-1

>hsa-mir-323b 5p:15-37;3p:51-72 SRX018959
ugguacucggagggAGGUUGUCCGUGGUGAGUUCGCAuuauuuaaugaugCCCAAUACACGGUCGACCUCUUuucgguau
ca
.(((((.(((((((((((((.(((((........(((((((...)))))))......))))).)))))))))))))))))
).
--CCCAAUACACGGUCGACCU-----------
-- 15 0,-3

>hsa-mir-324 5p:16-38;3p:53-72 SRX018959
cugacuaugccucccCGCAUCCCCUAGGGCAUUGGUGUaaagcuggagacccACUGCCCCAGGUGCUGCUGGggguugua
guc
..((((((..(((((.(((.(.(((.(((((.(((.((..........))))).))))).))).).))).)))))..)))
)))
---------------CGCAUCCCCUAGGGCAUU---
--- 10 0,-5
---------------CGCAUCCCCUAGGGCAUUG--
--- 19 0,-4
---------------CGCAUCCCCUAGGGCAUUGG---
--- 4 0,-3
---------------CGCAUCCCCUAGGGCAUUGGU--
--- 11 0,-2
---------------CGCAUCCCCUAGGGCAUUGGUG---
--- 3 0,-1
---------------CGCAUCCCCUAGGGCAUUGGUGU--
--- 22 0,0
---------------CGCAUCCCCUAGGGCAUUGGUGUu---
--- 3 0,0
--CCACUGCCCCAGGUGCUGCU----------
--- 2 -2,-2
--CCACUGCCCCAGGUGCUGCUG---------
--- 4 -2,-1
--ACUGCCCCAGGUGCUGCU----------
--- 4 0,-2
--ACUGCCCCAGGUGCUGCUGG--------
--- 9 0,0

>hsa-mir-326 3p:60-79 SRX018959
cucaucugucuguugggcuggaggcagggccuuugugaaggcggguggugcucagaucgCCUCUGGGCCCUUCCUCCAGc

cccgaggcggauuca
...((((((((...((((((((((.(((((((....((.(((((.((.....))..))))))).))))))).))))))))
))..))))))))...
---CCUCUGGGCCCUUCCUCCAau
--------------- 3 0,-1
---CCUCUGGGCCCUUCCUCCAGa
--------------- 2 0,0
---CCUCUGGGCCCUUCCUCCAGa
a-------------- 4 0,0
---CCUCUGGGCCCUUCCUCCAGu
--------------- 2 0,0
---CCUCUGGGCCCUUCCUCCAGu
u-------------- 2 0,0

>hsa-mir-328 3p:48-69 SRX018959
uggagugggggggcaggaggggcucagggagaaagugcauacagcccCUGGCCCUCUCUGCCCUUCCGUccccug
.((..(((((((((((..(((((.(((((.(...((....))..)))))))))))..)))))))))))..))...
---CUGGCCCUCUCUGCCCUUCCGa------ 3
0,-1
---CUGGCCCUCUCUGCCCUUCCGU------ 5
0,0
---CUGGCCCUCUCUGCCCUUCCGUau---- 4
0,0

>hsa-mir-329-1 3p:50-71 SRX018959
gguaccugaagagagguuuucuggguuucuguuucuuuaaugaggacgaAACACACCUGGUUAACCUCUUUuccaguauc
(((((..(((((((((((..(..(((...((((((.((......)).))))))).)))..)..)))))))))))..)))))
-----------AGAGGUUUUCUGGGUUUCU--
5
-----------AGAGGUUUUCUGGGUUUCUGUUU--
4
---AACACACCUGGUUAACCU-------------
2 0,-4
---AACACACCUGGUUAACCUC------------
5 0,-3
---AACACACCUGGUUAACCUCU-----------
2 0,-2
---AACACACCUGGUUAACCUCUU----------

4   0,-1
---AACACACCUGGUUAACCUCUUU---------
8   0,0

>hsa-mir-329-2 3p:52-73   SRX018959
gugguaccugaagagagguuuucuggguuucuguuucuuuauugaggacgaAACACACCUGGUUAACCUCUUUuccagua
ucaa
.((((((..(((((((((((..(..(((...((((((.((......)).)))))).)))..)..)))))))))))..)))
))).
-------------AGAGGUUUUCUGGGUUUCU--
----   5
-------------AGAGGUUUUCUGGGUUUCUGUUU--
----   4
---AACACACCUGGUUAACCU-----------
----   2   0,-4
---AACACACCUGGUUAACCUC----------
----   5   0,-3
---AACACACCUGGUUAACCUCU---------
----   2   0,-2
---AACACACCUGGUUAACCUCUU--------
----   4   0,-1
---AACACACCUGGUUAACCUCUUU-------
----   8   0,0

>hsa-mir-330   5p:18-39;3p:57-79   SRX018959
cuuuggcgaucacugccUCUCUGGGCCUGUGUCUUAGGCucugcaagaucaaccgaGCAAAGCACACGGCCUGCAGAGAg
gcagcgcucugccc
....(((((.(.(((((((((((((((((((.(((..((((.............)))).))))))).)))).)))))))
)))).).)).))).
--GCAAAGCACACGGCCUGCA-----
--------------   3   0,-4
--GCAAAGCACACGGCCUGCAGAaa-
--------------   2   0,-2
--GCAAAGCACACGGCCUGCAGAG--
--------------   3   0,-1
--GCAAAGCACACGGCCUGCAGAGA-
--------------   17   0,0
--GCAAAGCACACGGCCUGCAGAGAG

-------------- 4 0,1
--GCAAAGCACACGGCCUGCAGAGAG
u------------- 3 0,1
---CAAAGCACACGGCCUGCAGAGA-
-------------- 6 1,0
---CAAAGCACACGGCCUGCAGAGAG
-------------- 9 1,1
---CAAAGCACACGGCCUGCAGAGAG
u------------- 6 1,1
--AAAGCACACGGCCUGCAGAGA-
-------------- 5 2,0
--AAAGCACACGGCCUGCAGAGAG
-------------- 3 2,1
--AAAGCACACGGCCUGCAGAGAG
a------------- 3 2,1
--AAAGCACACGGCCUGCAGAGAG
G------------- 4 2,2

>hsa-mir-331 5p:26-47;3p:61-81 SRX018959
gaguuugguuuuguuuggguuuguuCUAGGUAUGGUCCCAGGGAUCCcagaucaaaccagGCCCCUGGGCCUAUCCUAGA
Accaaccuaagcuc
............((((((((.((((((((.((((.(((((((..(.............)..))))))).))))))))))
)).)))))))))..
--GCCCCUGGGCCUAUCCUA--
-------------- 3 0,-3
--GCCCCUGGGCCUAUCCUAG-
-------------- 4 0,-2
--GCCCCUGGGCCUAUCCUAGA
-------------- 7 0,-1
--GCCCCUGGGCCUAUCCUAGA
A------------- 10 0,0
--GCCCCUGGGCCUAUCCUAGA
Aa------------ 7 0,0
--GCCCCUGGGCCUAUCCUAGA
Au------------ 9 0,0

>hsa-mir-335 MA:16-38;mi:52-73 SRX018959
uguuuugagcgggggUCAAGAGCAAUAACGAAAAAUGUuugucauaaaccgUUUUUCAUUAUUGCUCCUGACCuccucuc

auuugcuauauuca
.((..((((.((((((((.(((((((((.((((((((.((......)).)))))))).))))))))).)))))))).)))
)...))........
---------------UCAAGAGCAAUAACGAAA---
------------- 2   0,-5
---------------UCAAGAGCAAUAACGAAAA--
------------- 4   0,-4
---------------UCAAGAGCAAUAACGAAAAA---
------------- 36   0,-3
---------------UCAAGAGCAAUAACGAAAAAa--
------------- 5   0,-3
---------------UCAAGAGCAAUAACGAAAAAU--
------------- 298 0,-2
---------------UCAAGAGCAAUAACGAAAAAUa---
------------- 91   0,-2
---------------UCAAGAGCAAUAACGAAAAAUG---
------------- 591 0,-1
---------------UCAAGAGCAAUAACGAAAAAUGa--
------------- 26   0,-1
---------------UCAAGAGCAAUAACGAAAAAUGU--
------------- 194 0,0
----------------CAAGAGCAAUAACGAAAAAUGU--
------------- 3   1,0
-----------------AAGAGCAAUAACGAAAAAU--
------------- 7   2,-2
-----------------AAGAGCAAUAACGAAAAAUa---
------------- 3   2,-2
-----------------AAGAGCAAUAACGAAAAAUG---
------------- 8   2,-1
-----------------AAGAGCAAUAACGAAAAAUGU--
------------- 4   2,0
--GUUUUUCAUUAUUGCUCCUGA---------
------------- 2   -1,-2
--GUUUUUCAUUAUUGCUCCUGACC-------
------------- 3   -1,0
---UUUUUCAUUAUUGCUCCU-----------
------------- 4   0,-4
---UUUUUCAUUAUUGCUCCUGACC-------

------------- 4 0,0
---UUUCAUUAUUGCUCCUGACC--------------------- 2 2,0

>hsa-mir-337 5p:23-43;3p:61-82 SRX018959
guagucaguaguugggggguggGAACGGCUUCAUACAGGAGUUgaugcacaguuauccagCUCCUAUAUGAUGCCUUUCUUCaucccuucaa
..........(..(((((((((((.(((.(((((.((((((((((((......))).)))))))).))))).))).))).)))))))))).)..
----------------------GAACGGCUUCAUACAGGAG-- 4 0,-2
-----------------------AACGGCUUCAUACAGGAG-- 2 1,-2
---CUCCUAUAUGAUGCCUUUCUU------------ 2 0,-1
---CUCCUAUAUGAUGCCUUUCUUC---------- 7 0,0
--UCCUAUAUGAUGCCUUUCUUC---------- 3 1,0

>hsa-mir-338 5p:6-27;3p:42-63 SRX018959
ucuccAACAAUAUCCUGGUGCUGAGUGaugacucaggcgacUCCAGCAUCAGUGAUUUUGUUGaaga
(((.((((((.((((((((((((((((............))).))))))))).))).)))))).)))
---UCCAGCAUCAGUGAUUUU-------- 13 0,-4
---UCCAGCAUCAGUGAUUUUG------- 17 0,-3
---UCCAGCAUCAGUGAUUUUGUU----- 3 0,-1
---UCCAGCAUCAGUGAUUUUGUUG---- 3 0,0
---UCCAGCAUCAGUGAUUUUGUUGA--- 4 0,1

>hsa-mir-339 5p:15-37;3p:50-72 SRX018959
cggggcggccgcucUCCCUGUCCUCCAGGAGCUCACGugugccugccugUGAGCGCCUCGACGACAGAGCCGgcgccugccccagugucugcgc
.(((((((.((((..(.(((((.((.(((.(((((((.((....)).))))))).))).)).))))).)..)))).))))))).........
--------------UCCCUGUCCUCCAGGAGC--- 2 0,-5
--------------UCCCUGUCCUCCAGGAGCU-- 11 0,-4

--------------UCCCUGUCCUCCAGGAGCUC--
-------------- 2 0,-3
--------------UCCCUGUCCUCCAGGAGCUCA---
-------------- 4 0,-2
--------------UCCCUGUCCUCCAGGAGCUCAa--
-------------- 5 0,-2
--------------UCCCUGUCCUCCAGGAGCUCAu--
-------------- 4 0,-2
--------------UCCCUGUCCUCCAGGAGCUCAuu---
-------------- 2 0,-2
--------------UCCCUGUCCUCCAGGAGCUCAC--
-------------- 6 0,-1
--------------UCCCUGUCCUCCAGGAGCUCACa---
-------------- 2 0,-1
--------------UCCCUGUCCUCCAGGAGCUCACu---
-------------- 3 0,-1
--------------UCCCUGUCCUCCAGGAGCUCACG---
-------------- 15 0,0
--------------UCCCUGUCCUCCAGGAGCUCACGU--
-------------- 5 0,1
--------------UCCCUGUCCUCCAGGAGCUCACGUu---
-------------- 2 0,1
----------------CCUGUCCUCCAGGAGCUCACG---
-------------- 3 2,0
---UGAGCGCCUCGACGACAG-------------
-------------- 5 0,-5
---UGAGCGCCUCGACGACAGA------------
-------------- 10 0,-4
---UGAGCGCCUCGACGACAGAG-----------
-------------- 8 0,-3
---UGAGCGCCUCGACGACAGAGC----------
-------------- 5 0,-2
---UGAGCGCCUCGACGACAGAGCa---------
-------------- 2 0,-2
---UGAGCGCCUCGACGACAGAGCaa--------
-------------- 2 0,-2
---UGAGCGCCUCGACGACAGAGCu---------
-------------- 5 0,-2

---UGAGCGCCUCGACGACAGAGCC---------
-------------- 13 0,-1
---UGAGCGCCUCGACGACAGAGCCa--------
-------------- 8 0,-1
---UGAGCGCCUCGACGACAGAGCCaa-------
-------------- 2 0,-1
---UGAGCGCCUCGACGACAGAGCCau-------
-------------- 11 0,-1
---UGAGCGCCUCGACGACAGAGCCu--------
-------------- 10 0,-1
---UGAGCGCCUCGACGACAGAGCCuu-------
-------------- 12 0,-1
---UGAGCGCCUCGACGACAGAGCCG--------
-------------- 3 0,0
---UGAGCGCCUCGACGACAGAGCCGu-------
-------------- 4 0,0
--GAGCGCCUCGACGACAGA------------
-------------- 2 1,-4
---AGCGCCUCGACGACAGAG-----------
-------------- 35 2,-3
---AGCGCCUCGACGACAGAGC----------
-------------- 3 2,-2
---AGCGCCUCGACGACAGAGCCG--------
-------------- 8 2,0
---AGCGCCUCGACGACAGAGCCGa-------
-------------- 7 2,0
---AGCGCCUCGACGACAGAGCCGGu------
-------------- 2 2,1

>hsa-mir-33a MA:6-26;mi:46-67 SRX018959
cugugGUGCAUUGUAGUUGCAUUGCAuguucuggugguacccaugCAAUGUUUCCACAGUGCAUCACag
(((((((((((((.(..(((((((((((....((.....)))))))))))))..).)))))))))))))
-----GUGCAUUGUAGUUGCAUU-- 567 0,-3
-----GUGCAUUGUAGUUGCAUUG--- 4 0,-2
-----GUGCAUUGUAGUUGCAUUGa-- 3 0,-2
-----GUGCAUUGUAGUUGCAUUGu-- 3 0,-2
-----GUGCAUUGUAGUUGCAUUGC-- 64 0,-1
-----GUGCAUUGUAGUUGCAUUGCA--- 279 0,0

-----GUGCAUUGUAGUUGCAUUGCAa-- 48 0,0
-----GUGCAUUGUAGUUGCAUUGCAU-- 16 0,1
---CAAUGUUUCCACAGUGCAU----- 6 0,-3
---CAAUGUUUCCACAGUGCAUa---- 5 0,-3
---CAAUGUUUCCACAGUGCAUCA--- 2 0,-1
---CAAUGUUUCCACAGUGCAUCAu-- 2 0,-1

>hsa-mir-33b MA:16-35;mi:54-75 SRX018959
gcgggcggccccgcgGUGCAUUGCUGUUGCAUUGCacgugugugaggcgggugCAGUGCCUCGGCAGUGCAGCCCggagc
cggccccuggcaccac
(((((((((.(((.((((((((((((..(((((((((.(((.....))).)))))))))..)))))))))).))))).))
).))))...)).....
---------------GUGCAUUGCUGUUGCAUU---
---------------- 5 0,-2
---------------GUGCAUUGCUGUUGCAUUGa--
---------------- 3 0,-1

>hsa-mir-340 MA:16-37;mi:58-79 SRX018959
uuguaccuggugugaUUAUAAAGCAAUGAGACUGAUUgucauaugucguuugugggaUCCGUCUCAGUUACUUUAUAGCc
auaccugguaucuua
..(((((.((((((..(((((((...((((((.((((.(((((.......))))))))).))))))....)))))))..)
))))).)))))....
---------------UUAUAAAGCAAUGAGACU---
--------------- 11 0,-4
---------------UUAUAAAGCAAUGAGACUGA--
--------------- 6 0,-2
---------------UUAUAAAGCAAUGAGACUGAaa--
--------------- 3 0,-2
---------------UUAUAAAGCAAUGAGACUGAU---
--------------- 24 0,-1
---------------UUAUAAAGCAAUGAGACUGAUa--
--------------- 29 0,-1
---------------UUAUAAAGCAAUGAGACUGAUau---
--------------- 2 0,-1
---------------UUAUAAAGCAAUGAGACUGAUU--
--------------- 137 0,0
---------------UUAUAAAGCAAUGAGACUGAUUa---
--------------- 8 0,0

---------------UUAUAAAGCAAUGAGACUGAUUu--- 3 0,0
----------------UAUAAAGCAAUGAGACUGA-- 3 1,-2
---UCCGUCUCAGUUACUUUAUAGC---------------- 3 0,0

>hsa-mir-342 5p:19-39;3p:61-83 SRX018959
gaaacugggcucaaggugAGGGGUGCUAUCUGUGAUUGAgggacaugguuaauggaauugUCUCACACAGAAAUCGCACCCGUcaccuuggccuacuua
.....((((.((((((((.((((((..((((((.....((((((...((....))..)))))).))))))....))))))).))))))))))))....
------------------AGGGGUGCUAUCUGUGAU--- 2 0,-3
------------------AGGGGUGCUAUCUGUGAUa-- 5 0,-3
------------------AGGGGUGCUAUCUGUGAUU-- 21 0,-2
------------------AGGGGUGCUAUCUGUGAUUG--- 8 0,-1
------------------AGGGGUGCUAUCUGUGAUUGA-- 43 0,0
------------------AGGGGUGCUAUCUGUGAUUGAa--- 3 0,0
------------------AGGGGUGCUAUCUGUGAUUGAG--- 64 0,1
------------------AGGGGUGCUAUCUGUGAUUGAGa-- 3 0,1
------------------AGGGGUGCUAUCUGUGAUUGAGG-- 14 0,2
------------------AGGGGUGCUAUCUGUGAUUGAGGGA-- 2 0,4
-------------------GGGGUGCUAUCUGUGAUUGAGGG--- 2 1,3
--UCUCACACAGAAAUCGCA--------------------- 64 0,-5
--UCUCACACAGAAAUCGCAC-------------------- 136 0,-4

---UCUCACACAGAAAUCGCACC
------------------- 124 0,-3
---UCUCACACAGAAAUCGCACC
C------------------ 207 0,-2
---UCUCACACAGAAAUCGCACC
Cau---------------- 2 0,-2
---UCUCACACAGAAAUCGCACC
CG----------------- 255 0,-1
---UCUCACACAGAAAUCGCACC
CGa---------------- 3 0,-1
---UCUCACACAGAAAUCGCACC
CGU---------------- 780 0,0
---UCUCACACAGAAAUCGCACC
CGUa--------------- 17 0,0
---UCUCACACAGAAAUCGCACC
CGUau-------------- 3 0,0
---UCUCACACAGAAAUCGCACC
CGUu--------------- 29 0,0
---UCUCACACAGAAAUCGCACC
CGUuu-------------- 2 0,0
---UCUCACACAGAAAUCGCACC
CGUC--------------- 346 0,1
---UCUCACACAGAAAUCGCACC
CGUCu-------------- 162 0,1
---UCUCACACAGAAAUCGCACC
CGUCA-------------- 112 0,2
--CUCACACAGAAAUCGCACC
------------------- 3 1,-3
--CUCACACAGAAAUCGCACC
CGU---------------- 4 1,0
--CUCACACAGAAAUCGCACC
CGUC--------------- 2 1,1
---UCACACAGAAAUCGCACC
------------------- 8 2,-3
---UCACACAGAAAUCGCACC
C------------------ 11 2,-2
---UCACACAGAAAUCGCACC
CG----------------- 7 2,-1

--UCACACAGAAAUCGCACC
CGU---------------- 10 2,0
--UCACACAGAAAUCGCACC
CGUaa-------------- 2 2,0
--UCACACAGAAAUCGCACC
CGUuu-------------- 3 2,0
--UCACACAGAAAUCGCACC
CGUC--------------- 17 2,1
--UCACACAGAAAUCGCACC
CGUCu-------------- 13 2,1
--UCACACAGAAAUCGCACC
CGUCA-------------- 94 2,2
--UCACACAGAAAUCGCACC
CGUCAa------------- 5 2,2
--UCACACAGAAAUCGCACC
CGUCAu------------- 8 2,2

>hsa-mir-345 5p:18-39 SRX018959
acccaaacccuaggucuGCUGACUCCUAGUCCAGGGCUCgugauggcugguggggcccugaacgaggggucuggaggccug
gguuugaauaucgacagc
...(((((((.((((((.(.(((((((.((.(((((((((..(....)..))))))))).)).))))))).).)))))))
)))))).............
-----------------GCUGACUCCUAGUCCAGG--
------------------ 95 0,-4
-----------------GCUGACUCCUAGUCCAGGG---
------------------ 37 0,-3
-----------------GCUGACUCCUAGUCCAGGGC--
------------------ 19 0,-2
-----------------GCUGACUCCUAGUCCAGGGCaa--
------------------ 4 0,-2
-----------------GCUGACUCCUAGUCCAGGGCau--
------------------ 4 0,-2
-----------------GCUGACUCCUAGUCCAGGGCU---
------------------ 74 0,-1
-----------------GCUGACUCCUAGUCCAGGGCUa--
------------------ 5 0,-1
-----------------GCUGACUCCUAGUCCAGGGCUaa---
------------------ 11 0,-1

-----------------GCUGACUCCUAGUCCAGGGCUau--
------------------ 26 0,-1
-----------------GCUGACUCCUAGUCCAGGGCUu---
------------------ 11 0,-1
-----------------GCUGACUCCUAGUCCAGGGCUuu--
------------------ 17 0,-1
-----------------GCUGACUCCUAGUCCAGGGCUC---
------------------ 77 0,0
-----------------GCUGACUCCUAGUCCAGGGCUCa--
------------------ 10 0,0
-----------------GCUGACUCCUAGUCCAGGGCUCaa---------------------------------------
------------------ 8   0,0
-----------------GCUGACUCCUAGUCCAGGGCUCau---------------------------------------
------------------ 33 0,0
-----------------GCUGACUCCUAGUCCAGGGCUCu--
------------------ 15 0,0
-----------------GCUGACUCCUAGUCCAGGGCUCuu---------------------------------------
------------------ 22 0,0
-----------------GCUGACUCCUAGUCCAGGGCUCGa---------------------------------------
------------------ 2   0,1
-----------------GCUGACUCCUAGUCCAGGGCUCGau--------------------------------------
------------------ 2   0,1
-----------------GCUGACUCCUAGUCCAGGGCUCGU---------------------------------------
------------------ 3   0,2

>hsa-mir-34a   MA:22-43;mi:64-85   SRX018959
ggccagcugugaguguuucuuUGGCAGUGUCUUAGCUGGUUGUugugagcaauaguaaggaagCAAUCAGCAAGUAUACU
GCCCUagaagugcugcacguuguggggccc
((((.((((((((..(((((..(((((((((((.((((((((((....((....)).....)))))))))))).)))))
)))..)))))..)).))))..))..)))).
---------------------UGGCAGUGUCUUAGCUGGUUGU-------------------------------------
------------------------------ 9   0,0
---CAAUCAGCAAGUAUACU
GCCCU------------------------- 2   0,0
---CAAUCAGCAAGUAUACU
GCCCUu------------------------ 4   0,0
---CAAUCAGCAAGUAUACU
GCCCUA------------------------ 5   0,1

---CAAUCAGCAAGUAUACU
GCCCUAu----------------------- 5 0,1
--AAUCAGCAAGUAUACU
GCC--------------------------- 3 1,-2
--AAUCAGCAAGUAUACU
GCCCU------------------------- 3 1,0
--AAUCAGCAAGUAUACU
GCCCUu------------------------ 4 1,0
--AAUCAGCAAGUAUACU
GCCCUA------------------------ 10 1,1
--AAUCAGCAAGUAUACU
GCCCUAu----------------------- 7 1,1

>hsa-mir-34c 5p:13-35;3p:46-67 SRX018959
agucuaguuacuAGGCAGUGUAGUUAGCUGAUUGCuaauaguaccAAUCACUAACCACACGGCCAGGuaaaaagauu
(((((..(((((.(((.((((.(((((.((((((.((....)).))))))))))).)))).))).)))))..)))))
------------AGGCAGUGUAGUUAGCUGAUUGC--
2 0,0

>hsa-mir-3605 5p:22-44;3p:66-88 SRX018959
acuuuauacguguaauugugaUGAGGAUGGAUAGCAAGGAAGCCgcucccaccugacccucacggCCUCCGUGUUACCUG
UCCUCUAGgugggacgcucg
.........((((.........(((((((.(((((.(((.((((..................)))).))).))))).)))
)))))........))))...
--------------------GAUGAGGAUGGAUAGCAAGG--
-------------------- 5 -2,-5
--------------------GAUGAGGAUGGAUAGCAAGGA---------------------------------------
-------------------- 3 -2,-4
--------------------GAUGAGGAUGGAUAGCAAGGAA--------------------------------------
-------------------- 9 -2,-3
--------------------GAUGAGGAUGGAUAGCAAGGAAa-------------------------------------
-------------------- 3 -2,-3
--------------------GAUGAGGAUGGAUAGCAAGGAAG-------------------------------------
-------------------- 5 -2,-2
--------------------GAUGAGGAUGGAUAGCAAGGAAGCC-----------------------------------
-------------------- 5 -2,0
---------------------AUGAGGAUGGAUAGCAAGGAAG-------------------------------------
-------------------- 9 -1,-2

---------------------UGAGGAUGGAUAGCAAGGAAG--	3	0,-2
---------------------UGAGGAUGGAUAGCAAGGAAGC---	3	0,-1
---------------------UGAGGAUGGAUAGCAAGGAAGCa--	4	0,-1
---------------------UGAGGAUGGAUAGCAAGGAAGCC--	3	0,0

>hsa-mir-3606	5p:16-36	SRX018959
uuguugcuaucuaggUUAGUGAAGGCUAUUUUAAUUuuuuuaaaauuucuuucacuacuuagg
..........((((((.((((((((..(((((((.....)))))))..)))))))))))))).
----------------UAGUGAAGGCUAUUUUAAUUUU------------------------- 4	1,2
----------------UAGUGAAGGCUAUUUUAAUUUUU------------------------ 2	1,3

>hsa-mir-3609	3p:51-74	SRX018959
guaacaguaacuuuuauucucauuuuccuuuucucuaccuuguagagaagCAAAGUGAUGAGUAAUACUGGCUGgagccc
((..((((.....(((((((((((((...(((((((((...))))))))).))))))).)))))).....))))..))..
--CAAAGUGAUGAGUAAUACU-----------	4	0,-5

>hsa-mir-361	5p:6-27;3p:45-67	SRX018959
ggagcUUAUCAGAAUCUCCAGGGGUACuuuauaauuucaaaaagUCCCCCAGGUGUGAUUCUGAUUUgcuuc
(((((..(((((((((.((.((((.(((((.........))))).)))).))...)))))))))..)))))
----CUUAUCAGAAUCUCCAGGGGUAC---	2	-1,0
-----UUAUCAGAAUCUCCAGGG---	4	0,-4
-----UUAUCAGAAUCUCCAGGGG--	5	0,-3
-----UUAUCAGAAUCUCCAGGGGU---	20	0,-2
-----UUAUCAGAAUCUCCAGGGGUA--	33	0,-1
-----UUAUCAGAAUCUCCAGGGGUAa---	12	0,-1
-----UUAUCAGAAUCUCCAGGGGUAu---	32	0,-1

-----UUAUCAGAAUCUCCAGGGGUAuu--- 3

0,-1

-----UUAUCAGAAUCUCCAGGGGUAC-- 57

0,0

-----UUAUCAGAAUCUCCAGGGGUACa--- 19

0,0

-----UUAUCAGAAUCUCCAGGGGUACau-- 7

0,0

-----UUAUCAGAAUCUCCAGGGGUACU--- 2

0,1

---UCCCCCAGGUGUGAUUCU---------- 9

0,-5

---UCCCCCAGGUGUGAUUCUGA-------- 2

0,-3

---UCCCCCAGGUGUGAUUCUGAa------- 5

0,-3

---UCCCCCAGGUGUGAUUCUGAaa------ 3

0,-3

---UCCCCCAGGUGUGAUUCUGAU------- 8

0,-2

---UCCCCCAGGUGUGAUUCUGAUa------ 2

0,-2

---UCCCCCAGGUGUGAUUCUGAUaa----- 4

0,-2

---UCCCCCAGGUGUGAUUCUGAUU------ 12

0,-1

---UCCCCCAGGUGUGAUUCUGAUUa----- 2

0,-1

---UCCCCCAGGUGUGAUUCUGAUUU----- 44

0,0

---UCCCCCAGGUGUGAUUCUGAUUUu---- 3

0,0

---UCCCCCAGGUGUGAUUCUGAUUUG---- 6

0,1

---UCCCCCAGGUGUGAUUCUGAUUUGC--- 2

0,2

--CCCCCAGGUGUGAUUCUGAUUU----- 6

1,0

---CCCCCAGGUGUGAUUCUGAUUUGu--- 3
1,1
--CCCCAGGUGUGAUUCUGAUUU----- 3
2,0
--CCCCAGGUGUGAUUCUGAUUUGu--- 2
2,1

>hsa-mir-3613 5p:16-37;3p:53-76 SRX018959
ugguugguuuggauUGUUGUACUUUUUUUUUGUUCguugcauuuuaggaACAAAAAAAAAAGCCCAACCCUUCacac
cacuuca
.((...(((.((((..((((..((((((((((((((.(.........).)))))))))))))))..))))..)))).))
)...)).
---------------UGUUGUACUUUUUUUUUGUU---
------- 2 0,-1
---ACAAAAAAAAAAGCCCAAC---------
------- 5 0,-5
---ACAAAAAAAAAAGCCCAACCCU------
------- 6 0,-2

>hsa-mir-3614 5p:15-37;3p:53-75 SRX018959
gguucugucuugggCCACUUGGAUCUGAAGGCUGCCCcuuugcucucuggggUAGCCUUCAGAUCUUGGUGUUUUgaauu
cuuacu
.........(..(..((((.(((((((((((((((((.........))))))))))))))))).))))..)..)...
......
---UAGCCUUCAGAUCUUGGUGU--------
------ 6 0,-3
---UAGCCUUCAGAUCUUGGUGUUa------
------ 4 0,-2
---UAGCCUUCAGAUCUUGGUGUUU------
------ 11 0,-1
---UAGCCUUCAGAUCUUGGUGUUUU-----
------ 3 0,0

>hsa-mir-3615 3p:51-71 SRX018959
gacucugggacgcucagacgccgcgcggggcggggauuggucuguggucсUCUCUCGGCUCCUCGCGGCUCgcggcggcc
gacgguu
((((.(.((.((((..((.((((((.(((((.((((..((.(....).))..)))).))))).))))))))..)))).))
.).))))

--UCUCUCGGCUCCUCGCGGCU----------
------- 5 0,-1

>hsa-mir-362 5p:5-28;3p:42-63 SRX018959
cuugAAUCCUUGGAACCUAGGUGUGAGUgcuauuucagugcAACACACCUAUUCAAGGAUUCAaa
.(((((((((((((...((((((((.(..((.....))..)..))))))))))))))))))))).
----AAUCCUUGGAACCUAGGUGU--- 5 0,-4
----AAUCCUUGGAACCUAGGUGUG-- 2 0,-3
----AAUCCUUGGAACCUAGGUGUGA--------------------------------------- 7 0,-2
----AAUCCUUGGAACCUAGGUGUGAG-------------------------------------- 2 0,-1
----AAUCCUUGGAACCUAGGUGUGAGU------------------------------------- 3 0,0
---AACACACCUAUUCAAGGAUU---- 6 0,-2
---AACACACCUAUUCAAGGAUUC--- 2 0,-1
---AACACACCUAUUCAAGGAUUCA-- 8 0,0
---AACACACCUAUUCAAGGAUUCAA- 3 0,1

>hsa-mir-363 mi:7-28;MA:50-71 SRX018959
uguuguCGGGUGGAUCACGAUGCAAUUUugaugaguaucauaggagaaaAAUUGCACGGUAUCCAUCUGUAaacc
.(((..(((((((((..((.(((((((((.(((.....)))......)))))))))))..)))))))))..))).
---AAUUGCACGGUAUCCAUC-------- 5
0,-4
---AAUUGCACGGUAUCCAUCU------- 61
0,-3
---AAUUGCACGGUAUCCAUCUG------ 2
0,-2
---AAUUGCACGGUAUCCAUCUGU----- 8
0,-1
---AAUUGCACGGUAUCCAUCUGUA---- 6
0,0
---AAUUGCACGGUAUCCAUCUGUAu--- 2
0,0
---AAUUGCACGGUAUCCAUCUGUAA--- 10
0,1
--AUUGCACGGUAUCCAUCU------- 3
1,-3

>hsa-mir-365-1 3p:56-77 SRX018959
accgcagggaaaaugagggacuuuuggggcagauguguuuccauuccacuaucaUAAUGCCCCUAAAAAUCCUUAUugc

ucuugca
...((((((..((((((((..(((((((((((((((((.........))).)))....))))))))))).))))))))..
)))))).
--------------GAGGGACUUUUGGGGGCAGAUG--
------- 2
---------------AGGGACUUUUGGGGGCAGA--
------- 4
---------------AGGGACUUUUGGGGGCAGAU---
------- 3
---------------AGGGACUUUUGGGGGCAGAUa--
------- 4
---------------AGGGACUUUUGGGGGCAGAUaa---
------- 2
---------------AGGGACUUUUGGGGGCAGAUG--
------- 9
---------------AGGGACUUUUGGGGGCAGAUGa---
------- 3
---------------AGGGACUUUUGGGGGCAGAUGU---
------- 32
---------------AGGGACUUUUGGGGGCAGAUGUa--
------- 3
---------------AGGGACUUUUGGGGGCAGAUGUaa---
------- 4
---------------AGGGACUUUUGGGGGCAGAUGUau---
------- 2
---------------AGGGACUUUUGGGGGCAGAUGUu--
------- 7
---------------AGGGACUUUUGGGGGCAGAUGUuu---
------- 9
---------------AGGGACUUUUGGGGGCAGAUGUG--
------- 73
---------------AGGGACUUUUGGGGGCAGAUGUGa---
------- 6
---------------AGGGACUUUUGGGGGCAGAUGUGaa--
------- 3
---------------AGGGACUUUUGGGGGCAGAUGUGU---
------- 66
---------------AGGGACUUUUGGGGGCAGAUGUGUU--

------- 2
----------------GGGACUUUUGGGGGCAGAUGUaa---
------- 2
----------------GGGACUUUUGGGGGCAGAUGUGa---
------- 2
----------------GGGACUUUUGGGGGCAGAUGUGU---
------- 14
----------------GGGACUUUUGGGGGCAGAUGUGUU--
------- 2
----------------GGGACUUUUGGGGGCAGAUGUGUUU---------------------------------------
------- 2
---UAAUGCCCCUAAAAAUCC-------
------- 7 0,-4
---UAAUGCCCCUAAAAAUCCU------
------- 5 0,-3
---UAAUGCCCCUAAAAAUCCUa-----
------- 2 0,-3
---UAAUGCCCCUAAAAAUCCUau----
------- 3 0,-3
---UAAUGCCCCUAAAAAUCCUU-----
------- 27 0,-2
---UAAUGCCCCUAAAAAUCCUUu----
------- 2 0,-2
---UAAUGCCCCUAAAAAUCCUUA----
------- 77 0,-1
---UAAUGCCCCUAAAAAUCCUUAa---
------- 31 0,-1
---UAAUGCCCCUAAAAAUCCUUAU---
------- 330 0,0
---UAAUGCCCCUAAAAAUCCUUAUa--
------- 13 0,0
---UAAUGCCCCUAAAAAUCCUUAUU--
------- 28 0,1
--AAUGCCCCUAAAAAUCCUU-----
------- 2 1,-2

>hsa-mir-365-2 mi:29-50;MA:68-89 SRX018959
agaguguucaaggacagcaagaaaaaugAGGGACUUUCAGGGGCAGCUGUguuuucugacucagucaUAAUGCCCCUAAA

AAUCCUUAUuguucuugcagugugcaucggg
...........(.((((((((((.((((((((..(((.(((((((((((.(((....))).)))).....))))))))))
..)))))))).))))))))..))).)......
---------------------------GAGGGACUUUCAGGGGCAGC----------------------------------
------------------------------- 7 -1,-3
---------------------------GAGGGACUUUCAGGGGCAGCU---------------------------------
------------------------------- 36 -1,-2
---------------------------GAGGGACUUUCAGGGGCAGCUG--------------------------------
------------------------------- 2 -1,-1
---------------------------GAGGGACUUUCAGGGGCAGCUGU-------------------------------
------------------------------- 7 -1,0
---------------------------GAGGGACUUUCAGGGGCAGCUGUa------------------------------
------------------------------- 2 -1,0
---------------------------GAGGGACUUUCAGGGGCAGCUGUG------------------------------
------------------------------- 2 -1,1
---------------------------GAGGGACUUUCAGGGGCAGCUGUGU-----------------------------
------------------------------- 2 -1,2
----------------------------AGGGACUUUCAGGGGCAGC----------------------------------
------------------------------- 6 0,-3
----------------------------AGGGACUUUCAGGGGCAGCU---------------------------------
------------------------------- 19 0,-2
----------------------------AGGGACUUUCAGGGGCAGCUG--------------------------------
------------------------------- 4 0,-1
----------------------------AGGGACUUUCAGGGGCAGCUGU-------------------------------
------------------------------- 12 0,0
----------------------------AGGGACUUUCAGGGGCAGCUGUu------------------------------
------------------------------- 8 0,0
----------------------------AGGGACUUUCAGGGGCAGCUGUuu-----------------------------
------------------------------- 2 0,0
----------------------------AGGGACUUUCAGGGGCAGCUGUG------------------------------
------------------------------- 57 0,1
----------------------------AGGGACUUUCAGGGGCAGCUGUGU-----------------------------
------------------------------- 22 0,2
--UAAUGCCCCUAAA
AAUCC-------------------------- 7 0,-4
--UAAUGCCCCUAAA
AAUCCU------------------------- 5 0,-3
--UAAUGCCCCUAAA

AAUCCUa------------------------ 2   0,-3
---UAAUGCCCCUAAA
AAUCCUau----------------------- 3   0,-3
---UAAUGCCCCUAAA
AAUCCUU------------------------ 27 0,-2
---UAAUGCCCCUAAA
AAUCCUUu----------------------- 2   0,-2
---UAAUGCCCCUAAA
AAUCCUUA----------------------- 77 0,-1
---UAAUGCCCCUAAA
AAUCCUUAa---------------------- 31 0,-1
---UAAUGCCCCUAAA
AAUCCUUAU---------------------- 330 0,0
---UAAUGCCCCUAAA
AAUCCUUAUa--------------------- 13 0,0
---UAAUGCCCCUAAA
AAUCCUUAUU--------------------- 28 0,1
--AAUGCCCCUAAA
AAUCCUU------------------------ 2   1,-2

>hsa-mir-3656   3p:49-65   SRX018959
cuuucggccagcgggacggcauccgaggugggcuaggcucgggcccguGGCGGGUGCGGGGGUGGgagg
((((.(((.......(.(((((((..(((((((.......)))))))..))))))).).))).)))))
--GGCGGGUGCGGGGGUGGG---   2   0,1

>hsa-mir-3664   5p:21-42   SRX018959
cuguaaacuugaagguagggAACUCUGUCUUCACUCAUGAGUaccuuccaacacgagcucucaggaguaaagacagaguu
cccuaccuucaauguggau
......(((((((((((((((((((((((((.((((.((((...(((.......)))..)))).)))).)))))))))))
)))))))))))).))....
--UCUCAGGAGUAAAGACAG----
------------------- 9
--UCUCAGGAGUAAAGACAGA---
------------------- 15
--UCUCAGGAGUAAAGACAGAG--
------------------- 9
--UCUCAGGAGUAAAGACAGAGUa
a------------------ 2

--UCUCAGGAGUAAAGACAGAGUU
------------------- 26
--UCUCAGGAGUAAAGACAGAGUU
a------------------ 2
--UCUCAGGAGUAAAGACAGAGUU
u------------------ 8
--UCAGGAGUAAAGACAGAGUU
------------------- 6
--UCAGGAGUAAAGACAGAGUU
u------------------ 3
---CAGGAGUAAAGACAGAGUU
------------------- 7
--AGGAGUAAAGACAGAGUU
CCCU--------------- 4

>hsa-mir-3676 3p:60-79 SRX018959
uugguuaaagcgccugucuaguaaacaggagauccugggguucgaaucccagcggugccuCCGUGUUUCCCCCACGCUUUu
gccaa
.((((.((((((..((....(.((((((((((((((((.......))))).)))..)))).))))).)..)))))))).
)))).

>hsa-mir-3687 3p:35-58 SRX018959
cgcgcgugcgcccgagcgcggcccgguggucccuCCCGGACAGGCGUUCGUGCGACGUgug
((((((.(((.(((((((.(.((((..(...)..)))).)..))))))).))))))))))
------------------------------------CGGACAGGCGUUCGUGCGAC----- 2 2,-2
------------------------------------CGGACAGGCGUUCGUGCGACG---- 2 2,-1
------------------------------------CGGACAGGCGUUCGUGCGACGU--- 2 2,0

>hsa-mir-369 5p:9-30;3p:44-64 SRX018959
uugaagggAGAUCGACCGUGUUAUAUUCGCuuuauugacuucgAAUAAUACAUGGUUGAUCUUUucucag
.(((..((((((((((((((..((((((............))))))..))))))))))))))..))).
--------AGAUCGACCGUGUUAUAUU--- 11 0,-3
--------AGAUCGACCGUGUUAUAUUC-- 34 0,-2
--------AGAUCGACCGUGUUAUAUUCG--- 8 0,-1
--------AGAUCGACCGUGUUAUAUUCGCU--------------------------------------- 7 0,1
--------AGAUCGACCGUGUUAUAUUCGCUU-------------------------------------- 3 0,2
---AAUAAUACAUGGUUGAUCU-------- 3 0,-2
---AAUAAUACAUGGUUGAUCUUU------ 77 0,0

---AAUAAUACAUGGUUGAUCUUUa----- 2 0,0
--AUAAUACAUGGUUGAUCUUUau---- 2 1,0

>hsa-mir-3690 5p:10-32 SRX018959
cccaucuccACCUGGACCCAGCGUAGACAAAGagguguuucuacuccauaucuaccuggacccaguguagauggg
(((((((.(((.(((..((((.(((((....(((.........)))....)))))))))..)))))).)))))))
---------ACCUGGACCCAGCGUAGA-- 2
0,-5
---------ACCUGGACCCAGCGUAGACA-- 6
0,-3
---------ACCUGGACCCAGCGUAGACAA--- 10
0,-2
---------ACCUGGACCCAGCGUAGACAAA-- 4
0,-1
---------ACCUGGACCCAGCGUAGACAAAG--- 9
0,0

>hsa-mir-370 3p:48-69 SRX018959
agacagagaagccaggucacgucucugcaguuacacagcucacgaguGCCUGCUGGGGUGGAACCUGGUcugucu
((((((....(((((((..(..(((.((((.(((.(.......).))).)))).)))..)..)))))))))))))
---------AGCCAGGUCACGUCUCUGCAGUa--- 3
---GCCUGCUGGGGUGGAACCUGG------- 2
0,-1
---GCCUGCUGGGGUGGAACCUGGaa----- 3
0,-1
---GCCUGCUGGGGUGGAACCUGGUaa---- 2
0,0
---GCCUGCUGGGGUGGAACCUGGUau---- 2
0,0

>hsa-mir-371 5p:6-25;3p:42-64 SRX018959
guggcACUCAAACUGUGGGGGCACUuucugcucucuggugaAAGUGCCGCCAUCUUUUGAGUGUuac
((((((((((((..((((.(((((((((.(((....)))))))))))).))))..))))))))))))
-----ACUCAAACUGUGGGGGCACUUU-- 4 0,2

>hsa-mir-372 3p:42-64 SRX018959
gugggccucaaauguggagcacuauucugauguccaaguggAAAGUGCUGCGACAUUUGAGCGUcac
...((((((((((((.(((((((.((((...........)))))))))).).))))))))).)))..

---AAAGUGCUGCGACAUUUGAG------ 7 0,-3
--AAGUGCUGCGACAUUUGAGCGU--- 2 1,0

>hsa-mir-374a MA:12-33;mi:42-63 SRX018959
uacaucggccaUUAUAAUACAACCUGAUAAGUGuuauagcaCUUAUCAGAUUGUAUUGUAAUUgucugugua
(((((.(((.((((((((((((.(((((((((((....))))))))))).)))))))))))).))).)))))
-----------UUAUAAUACAACCUGAUA--- 7
0,-4
-----------UUAUAAUACAACCUGAUAA-- 146
0,-3
-----------UUAUAAUACAACCUGAUAAa--- 7
0,-3
-----------UUAUAAUACAACCUGAUAAaa-- 4
0,-3
-----------UUAUAAUACAACCUGAUAAG--- 62
0,-2
-----------UUAUAAUACAACCUGAUAAGa-- 6
0,-2
-----------UUAUAAUACAACCUGAUAAGU-- 559
0,-1
-----------UUAUAAUACAACCUGAUAAGUa--------------------------------------- 29
0,-1
-----------UUAUAAUACAACCUGAUAAGUaa-------------------------------------- 12
0,-1
-----------UUAUAAUACAACCUGAUAAGUG--------------------------------------- 653
0,0
-----------UUAUAAUACAACCUGAUAAGUGa-------------------------------------- 205
0,0
-----------UUAUAAUACAACCUGAUAAGUGU-------------------------------------- 32
0,1
------------UAUAAUACAACCUGAUAAG--- 2
1,-2
------------UAUAAUACAACCUGAUAAGU-- 8
1,-1
------------UAUAAUACAACCUGAUAAGUG--------------------------------------- 3
1,0
------------UAUAAUACAACCUGAUAAGUGU-------------------------------------- 2
1,1

-------------AUAAUACAACCUGAUAAGUGa---------------------------------------   2
2,0
---ACUUAUCAGAUUGUAUUGUAA-----------   2
-1,-2
---ACUUAUCAGAUUGUAUUGUAAU----------   3
-1,-1
---ACUUAUCAGAUUGUAUUGUAAUU---------   2
-1,0
--CUUAUCAGAUUGUAUUGU-------------   11
0,-4
--CUUAUCAGAUUGUAUUGUA------------   4
0,-3
--CUUAUCAGAUUGUAUUGUAuu----------   2
0,-3
--CUUAUCAGAUUGUAUUGUAA-----------   31
0,-2
--CUUAUCAGAUUGUAUUGUAAa----------   5
0,-2
--CUUAUCAGAUUGUAUUGUAAU----------   317
0,-1
--CUUAUCAGAUUGUAUUGUAAUa---------   166
0,-1
--CUUAUCAGAUUGUAUUGUAAUaa--------   5
0,-1
--CUUAUCAGAUUGUAUUGUAAUU---------   877
0,0
--CUUAUCAGAUUGUAUUGUAAUUa--------   108
0,0
--CUUAUCAGAUUGUAUUGUAAUUu--------   14
0,0
---UUAUCAGAUUGUAUUGUAAU----------   4
1,-1
---UUAUCAGAUUGUAUUGUAAUa---------   3
1,-1
---UUAUCAGAUUGUAUUGUAAUU---------   14
1,0
---UUAUCAGAUUGUAUUGUAAUUa--------   2
1,0

---UAUCAGAUUGUAUUGUAAUUa-------- 7
2,0
---UAUCAGAUUGUAUUGUAAUUaa------- 2
2,0

>hsa-mir-374b MA:11-32;mi:41-62 SRX018959
acucggauggAUAUAAUACAACCUGCUAAGUGuccuagcaCUUAGCAGGUUGUAUUAUCAUUguccgugucu
((.((((..(..(((((((((((((((((((((....)))))))))))))))))))))..)..)))).))..
----------AUAUAAUACAACCUGCUAA--- 2
0,-3
----------AUAUAAUACAACCUGCUAAG-- 13
0,-2
----------AUAUAAUACAACCUGCUAAGU--- 246
0,-1
----------AUAUAAUACAACCUGCUAAGUa-- 7
0,-1
----------AUAUAAUACAACCUGCUAAGUaa--------------------------------------- 6
0,-1
----------AUAUAAUACAACCUGCUAAGUG-- 379
0,0
----------AUAUAAUACAACCUGCUAAGUGa--------------------------------------- 96
0,0
----------AUAUAAUACAACCUGCUAAGUGU--------------------------------------- 27
0,1
------------AUAAUACAACCUGCUAAGU--- 2
2,-1
------------AUAAUACAACCUGCUAAGUG-- 6
2,0
--CUUAGCAGGUUGUAUUAUCA------------ 2
0,-2
--CUUAGCAGGUUGUAUUAUCAU----------- 6
0,-1
--CUUAGCAGGUUGUAUUAUCAUa---------- 5
0,-1
--CUUAGCAGGUUGUAUUAUCAUU---------- 13
0,0
---UUAGCAGGUUGUAUUAUCAUU---------- 6
1,0

```
>hsa-mir-374c    5p:13-34     SRX018959
acacggacaaugAUAAUACAACCUGCUAAGUGCUaggacacuuagcagguuguauuauauccauccgagu
...((((.....((((((((((((((((((((......)))))))))))))))))))).....))))...
------------AUAAUACAACCUGCUAAGU---------------------------------------  2   0,-3
------------AUAAUACAACCUGCUAAGUG--------------------------------------  6   0,-2

>hsa-mir-375     3p:40-61     SRX018959
ccccgcgacgagccccucgcacaaaccggaccugagcguUUUGUUCGUUCGGCUCGCGUGAggc
.((((((.((((((...((.(((((.((........)).))))).))...)))))))))).)).
--------------------------------------UUUUGUUCGUUCGGCUCGCGU-----    2    -1,-2
--------------------------------------UUUUGUUCGUUCGGCUCGCGUG----    3    -1,-1
--------------------------------------UUUUGUUCGUUCGGCUCGCGUGA---    3    -1,0
---------------------------------------UUUGUUCGUUCGGCUCGC-------    7    0,-4
---------------------------------------UUUGUUCGUUCGGCUCGCG------    22   0,-3
---------------------------------------UUUGUUCGUUCGGCUCGCGU-----    35   0,-2
---------------------------------------UUUGUUCGUUCGGCUCGCGUG----    152  0,-1
---------------------------------------UUUGUUCGUUCGGCUCGCGUGu---    12   0,-1
---------------------------------------UUUGUUCGUUCGGCUCGCGUGA---    150  0,0
---------------------------------------UUUGUUCGUUCGGCUCGCGUGAa--    6    0,0
---------------------------------------UUUGUUCGUUCGGCUCGCGUGAau-    3    0,0
---------------------------------------UUUGUUCGUUCGGCUCGCGUGAu--    15   0,0
---------------------------------------UUUGUUCGUUCGGCUCGCGUGAuu-    4    0,0
---------------------------------------UUUGUUCGUUCGGCUCGCGUGAG--    2    0,1
----------------------------------------UUGUUCGUUCGGCUCGCGUGu---    2    1,-1
----------------------------------------UUGUUCGUUCGGCUCGCGUGA---    2    1,0

>hsa-mir-376a-1 mi:7-28;MA:44-64     SRX018959
uaaaagGUAGAUUCUCCUUCUAUGAGUAcauuauuuaugauuaAUCAUAGAGGAAAAUCCACGUuuuc
.((((.((.((((.((((.((((((...(((.....))).....)))))))))).)))).)).)))).
-------------------------------------------AUCAUAGAGGAAAAUCCA-------    3   0,-3
-------------------------------------------AUCAUAGAGGAAAAUCCACGU----    12  0,0

>hsa-mir-376a-2 3p:50-70     SRX018959
gguauuuaaaagguagauuuuccuucuaugguuacguguuugaugguuaAUCAUAGAGGAAAAUCCACGUuuucaguauc
((((((.((((.((.(((((((((.((((((((((((.....)))..)))))))))))))))))).)).)))).))))))
-------------------------------------------------AUCAUAGAGGAAAAUCCA-------------
    3   0,-3
```

---AUCAUAGAGGAAAAUCCACGU----------
12 0,0

>hsa-mir-376b 3p:62-83 SRX018959
caguccuucuuugguauuuaaaacguggauauuccuucuauguuuacgugauuccugguuaAUCAUAGAGGAAAAUCCAU
GUUuucaguaucaaaugcug
((((.....((((((((.((((((((((.(((((.(((((......((((.....)))).))))))))))).))))))
))))).))))))))).))))
-------------------------UGGAUAUUCCUUCUAUGUUUA-------------------------------------
-------------------- 2
---AUCAUAGAGGAAAAUCCA-
-------------------- 3 0,-4
---AUCAUAGAGGAAAAUCCAU
-------------------- 37 0,-3
---AUCAUAGAGGAAAAUCCAU
GU------------------ 4 0,-1

>hsa-mir-376c 3p:43-63 SRX018959
aaaagguggauauuccuucuauguuuauguuauuuaugguuaAACAUAGAGGAAAUUCCACGUuuu
((((.(((((..(((((.(((((((((............))))))))))))))..))))).))))
------UGGAUAUUCCUUCUAUGUUUA-------------------------------------- 2
--AACAUAGAGGAAAUUCCA------ 2 0,-3
--AACAUAGAGGAAAUUCCAC----- 31 0,-2
--AACAUAGAGGAAAUUCCACG---- 41 0,-1
--AACAUAGAGGAAAUUCCACGU--- 229 0,0
--AACAUAGAGGAAAUUCCACGUa-- 6 0,0
--AACAUAGAGGAAAUUCCACGUaa- 3 0,0
--AACAUAGAGGAAAUUCCACGUU-- 14 0,1
--AACAUAGAGGAAAUUCCACGUUU- 2 0,2
---ACAUAGAGGAAAUUCCACGU--- 2 1,0
---ACAUAGAGGAAAUUCCACGUUU- 2 1,2

>hsa-mir-377 mi:7-28;MA:45-66 SRX018959
uugagcAGAGGUUGCCCUUGGUGAAUUCgcuuuauuuauguugaAUCACACAAAGGCAACUUUUGUuug
..(((((((((((((.(((((((.(((((.........).)))))))).))).)))))))))))))).
------AGAGGUUGCCCUUGGUGAAU-- 3 0,-2
------AGAGGUUGCCCUUGGUGAAUa--- 10 0,-2
------AGAGGUUGCCCUUGGUGAAUU--- 77 0,-1

------AGAGGUUGCCCUUGGUGAAUUC--- 6 0,0
---AAUCACACAAAGGCAACUUUUG---- 4
-1,-1
---AAUCACACAAAGGCAACUUUUGU--- 2 -1,0
--AUCACACAAAGGCAACUUUUG---- 3 0,-1
--AUCACACAAAGGCAACUUUUGU--- 3 0,0

>hsa-mir-378 mi:5-26;MA:43-63 SRX018959
agggCUCCUGACUCCAGGUCCUGUGUguuaccuagaaauagcACUGGACUUGGAGUCAGAAGGccu
(((.((.((((((((((((((...((((((........)))))).))))))))))))))).)).)))
----CUCCUGACUCCAGGUCCU-- 5 0,-4
----CUCCUGACUCCAGGUCCUG--- 3 0,-3
----CUCCUGACUCCAGGUCCUGUGU-- 4 0,0
----CUCCUGACUCCAGGUCCUGUGUuu-------------------------------------- 3 0,0
-----UCCUGACUCCAGGUCCUG--- 2 1,-3
-----UCCUGACUCCAGGUCCUGU-- 3 1,-2
-----UCCUGACUCCAGGUCCUGUGU-- 8 1,0
-----UCCUGACUCCAGGUCCUGUGUaa-------------------------------------- 2 1,0
-----UCCUGACUCCAGGUCCUGUGUG--------------------------------------- 3 1,1
--GCACUGGACUUGGAGUCAGAAa---- 2 -2,-2
--GCACUGGACUUGGAGUCAGAAG---- 3 -2,-1
--GCACUGGACUUGGAGUCAGAAGu--- 3 -2,-1
---CACUGGACUUGGAGUCAGAAGG--- 19 -1,0
---CACUGGACUUGGAGUCAGAAGGC-- 25 -1,1
---CACUGGACUUGGAGUCAGAAGGCa- 11 -1,1
---CACUGGACUUGGAGUCAGAAGGCau 3 -1,1
---CACUGGACUUGGAGUCAGAAGGCu- 7 -1,1
---CACUGGACUUGGAGUCAGAAGGCuu 2 -1,1
--ACUGGACUUGGAGUCAGA------ 149 0,-3
--ACUGGACUUGGAGUCAGAA----- 285 0,-2
--ACUGGACUUGGAGUCAGAAa---- 21 0,-2
--ACUGGACUUGGAGUCAGAAaa--- 9 0,-2
--ACUGGACUUGGAGUCAGAAG---- 998 0,-1
--ACUGGACUUGGAGUCAGAAGu--- 67 0,-1
--ACUGGACUUGGAGUCAGAAGG--- 8703 0,0
--ACUGGACUUGGAGUCAGAAGGa-- 1973 0,0
--ACUGGACUUGGAGUCAGAAGGaa- 204 0,0
--ACUGGACUUGGAGUCAGAAGGau- 61 0,0

--ACUGGACUUGGAGUCAGAAGGu--   472   0,0
--ACUGGACUUGGAGUCAGAAGGuu-   89   0,0
--ACUGGACUUGGAGUCAGAAGGC--   19155   0,1
--ACUGGACUUGGAGUCAGAAGGCa-   10336   0,1
--ACUGGACUUGGAGUCAGAAGGCaa   728   0,1
--ACUGGACUUGGAGUCAGAAGGCau   3610   0,1
--ACUGGACUUGGAGUCAGAAGGCu-   5527   0,1
--ACUGGACUUGGAGUCAGAAGGCuu   1824   0,1
--ACUGGACUUGGAGUCAGAAGGCC-   17   0,2
--ACUGGACUUGGAGUCAGAAGGCCa   5   0,2
--ACUGGACUUGGAGUCAGAAGGCCU   15   0,3
--ACUGGACUUGGAGUCAGAAGGCCUu   10   0,3
---CUGGACUUGGAGUCAGAA-----   5   1,-2
---CUGGACUUGGAGUCAGAAG----   11   1,-1
---CUGGACUUGGAGUCAGAAGG---   165   1,0
---CUGGACUUGGAGUCAGAAGGa--   13   1,0
---CUGGACUUGGAGUCAGAAGGaa-   18   1,0
---CUGGACUUGGAGUCAGAAGGu--   7   1,0
---CUGGACUUGGAGUCAGAAGGuu-   5   1,0
---CUGGACUUGGAGUCAGAAGGC--   1584   1,1
---CUGGACUUGGAGUCAGAAGGCa-   446   1,1
---CUGGACUUGGAGUCAGAAGGCaa   108   1,1
---CUGGACUUGGAGUCAGAAGGCau   193   1,1
---CUGGACUUGGAGUCAGAAGGCu-   609   1,1
---CUGGACUUGGAGUCAGAAGGCuu   463   1,1
---CUGGACUUGGAGUCAGAAGGCC-   11   1,2
---CUGGACUUGGAGUCAGAAGGCCa   14   1,2
---CUGGACUUGGAGUCAGAAGGCCaa   3   1,2
---CUGGACUUGGAGUCAGAAGGCCau   15   1,2
---CUGGACUUGGAGUCAGAAGGCCU   4   1,3
---CUGGACUUGGAGUCAGAAGGCCUu   9   1,3
---CUGGACUUGGAGUCAGAAGGCCUuu   6   1,3
--UGGACUUGGAGUCAGAAGG---   2   2,0
--UGGACUUGGAGUCAGAAGGC--   32   2,1
--UGGACUUGGAGUCAGAAGGCa-   5   2,1
--UGGACUUGGAGUCAGAAGGCaa   3   2,1
--UGGACUUGGAGUCAGAAGGCau   3   2,1
--UGGACUUGGAGUCAGAAGGCu-   5   2,1

--UGGACUUGGAGUCAGAAGGCuu   9   2,1

>hsa-mir-378b   3p:34-52   SRX018959
ggucauugagucuucaaggcuaguggaaagagcACUGGACUUGGAGGCAGAAagacc
((((.((..(((((((((.((((((.......)))))).)))))))))..)).))))
---------------------------------ACUGGACUUGGAGGCAGAA-----   247 0,0

>hsa-mir-378c   5p:11-35   SRX018959
ggaggccaucACUGGACUUGGAGUCAGAAGAGUGGagucgggucagacuucaacucugacuuugaagguggugagugccu
c
.((((((((((((....(..((((((((....(((((((......)))))))..))))))))..).)))))))...))))
)
--------UCACUGGACUUGGAGUCAGAAG--
-   2   -2,-5
----------ACUGGACUUGGAGUCAGAAG--
-   998 0,-5
----------ACUGGACUUGGAGUCAGAAGu---
-   67 0,-5
----------ACUGGACUUGGAGUCAGAAGA---
-   586 0,-4
----------ACUGGACUUGGAGUCAGAAGAa--
-   22 0,-4
----------ACUGGACUUGGAGUCAGAAGAaa---
-   5   0,-4
----------ACUGGACUUGGAGUCAGAAGAau---
-   2   0,-4
----------ACUGGACUUGGAGUCAGAAGAu--
-   2   0,-4
-----------CUGGACUUGGAGUCAGAAG--
-   11 1,-5
-----------CUGGACUUGGAGUCAGAAGA---
-   9   1,-4
-----------CUGGACUUGGAGUCAGAAGAaa---
-   2   1,-4

>hsa-mir-379   MA:6-26;mi:44-65   SRX018959
agagaUGGUAGACUAUGGAACGUAGGcguuaugauuucugaccUAUGUAACAUGGUCCACUAACUcu
((((.((((.(((((((..(((((((((..(....)..)).)))))))..))))))).)))).))))

-----UGGUAGACUAUGGAACGUAG-- 27 0,-1
-----UGGUAGACUAUGGAACGUAGa--- 2 0,-1
-----UGGUAGACUAUGGAACGUAGG--- 110 0,0
-----UGGUAGACUAUGGAACGUAGGa-- 26 0,0
-----UGGUAGACUAUGGAACGUAGGaa--------------------------------------- 24 0,0
-----UGGUAGACUAUGGAACGUAGGu-- 8 0,0
-----UGGUAGACUAUGGAACGUAGGC-- 2 0,1
-----UGGUAGACUAUGGAACGUAGGCau-------------------------------------- 2 0,1
------GGUAGACUAUGGAACGUAGG--- 2 1,0

>hsa-mir-380 mi:5-26;MA:40-61 SRX018959
aagaUGGUUGACCAUAGAACAUGCGCuaucucugugucgUAUGUAAUAUGGUCCACAUCUU
((((((...(((((((..(((((((.(((....))).)))))))..)))))))..))))))
---AUGGUUGACCAUAGAACAUG-------------------------------------- 2 -1,-3

>hsa-mir-381 3p:49-70 SRX018959
uacuuaaagcgagguugcccuuuguauauucgguuuauugacauggaaUAUACAAGGGCAAGCUCUCUGUgagua
((((((.((.(((.((((((((.((((((((.(((....)))...)))))))))))))))).))).)).))))))
--UAUACAAGGGCAAGCUCUCU------- 2
0,-2
---AUACAAGGGCAAGCUCUCU------- 2
1,-2

>hsa-mir-382 5p:11-32 SRX018959
uacuugaagaGAAGUUGUUCGUGGUGGAUUCGcuuuacuuaugacgaaucauucacggacaacacuuuuucagua
((((.(((((((.(((((((((((..(((((((........).))))))..))))))))))).)))))))))))
----------GAAGUUGUUCGUGGUGGAU--
6 0,-3
----------GAAGUUGUUCGUGGUGGAUU---
14 0,-2
----------GAAGUUGUUCGUGGUGGAUUC--
12 0,-1
----------GAAGUUGUUCGUGGUGGAUUCG---
3 0,0
----------GAAGUUGUUCGUGGUGGAUUCGa--
2 0,0
-----------AAGUUGUUCGUGGUGGAUU---
5 1,-2

------------AGUUGUUCGUGGUGGAUUCGCU---
2 2,2
---AAUCAUUCACGGACAACA------------
2
---AAUCAUUCACGGACAACAC-----------
95
---AAUCAUUCACGGACAACACU----------
155
---AAUCAUUCACGGACAACACUU---------
14
---AAUCAUUCACGGACAACACUUU--------
30

>hsa-mir-383 5p:7-28 SRX018959
cuccucAGAUCAGAAGGUGAUUGUGGCUuuggguggauauuaaucagccacagcacugccuggucagaaagag
(((.((.((((((..((((.((((((((...(((........)))))))))))))))..)))))).))..)))
-----CAGAUCAGAAGGUGAUUGUG-- 2
-1,-3
-----CAGAUCAGAAGGUGAUUGUGGCa--- 4
-1,-1
-----CAGAUCAGAAGGUGAUUGUGGCU--- 2
-1,0
------AGAUCAGAAGGUGAUUGU--- 6
0,-4
------AGAUCAGAAGGUGAUUGUGG--- 8
0,-2
------AGAUCAGAAGGUGAUUGUGGC-- 9
0,-1
------AGAUCAGAAGGUGAUUGUGGCa--- 6
0,-1
------AGAUCAGAAGGUGAUUGUGGCaa-- 2
0,-1
------AGAUCAGAAGGUGAUUGUGGCau-- 10
0,-1
------AGAUCAGAAGGUGAUUGUGGCU--- 101
0,0
------AGAUCAGAAGGUGAUUGUGGCUaa--- 6
0,0

------AGAUCAGAAGGUGAUUGUGGCUU---   45
    0,1
------AGAUCAGAAGGUGAUUGUGGCUUa--   18
    0,1
------AGAUCAGAAGGUGAUUGUGGCUUau---   4
    0,1
------AGAUCAGAAGGUGAUUGUGGCUUU--   45
    0,2
------AGAUCAGAAGGUGAUUGUGGCUUUu---   7
    0,2

>hsa-mir-3909   3p:71-92   SRX018959
gguaugcuguugcgcuguccuucccucuggggagcaggcuccgggggacagggaaaagcacacaaggaacuUGUCCUCUAG
GGCCUGCAGUCUcaugggagagugacaugcaccaggacc
(((...(((.((((.(((((((((.(((((.((((((((..(((((((((.................)))))))))..)
)))))))..))))).)))).)).)))))))).))).)))
------------------------------GAGCAGGCUCCGGGGGACAGG-----------------------------
--------------------------------------- 4
---UGUCCUCUAG
GGCCUGCAGUCUau------------------------- 2   0,0
---UGUCCUCUAG
GGCCUGCAGUCUuu------------------------- 2   0,0

>hsa-mir-3910-1 3p:63-82   SRX018959
cuuuugcugucaguuuuucuguugcuugucuugguuuuaugccuuuuauaucaaggcacauaAAAGGCAUAAAACCAAGA
CAagcaacaaaaaaaggauugaucacagaag
(((((..(((((((((.((((((((((((((((((((((((((((.........)))))))))))))))))))
)))))))))....)))))))))..))))))
---AAAAGGCAUAAAACCAAGA
CA----------------------------- 2   -1,0

>hsa-mir-3910-2 3p:50-69   SRX018959
uuuuuuuguugcuugucuugguuuuaugccuuuuaugugccuugauauaAAAGGCAUAAAACCAAGACAagcaacagaaa
aa
.((((((((((((((((((((((((((((((((((......))))))))))))))))))))))))))))))))))
).
--AAAAGGCAUAAAACCAAGACA-----------
--   2   -1,0

>hsa-mir-3912 3p:63-83 SRX018959
agagaggaaugaacaguuaaauuauaacauguccauauuauggguuaguuguggacacauacUAACGCAUAAUAUGGACA
UGUuauaauuuaacuguuccuuucu
(((((((....(((((((((((((((((((((((((((((((.((((((.(((....))))))))).)))))))))))))
)))))))))))))))))))))))))
---------------------------------CAUAUUAUGGGUUAGUUGU----------------------------
-------------------------   2
--UAACGCAUAAUAUGGACA
UG-----------------------   5   0,-1

>hsa-mir-3924 3p:50-71 SRX018959
uaaaugaaaaaguaguagucaaauaugcagaucuaugucauauauacagAUAUGUAUAUGUGACUGCUACUuuuuuguuu
a
((((..(((((((((((((((.(((((((.((((.(((.....))).)))).))))))).)))))))))))))))..)))
)
---AUAUGUAUAUGUGACUGCUAC----------
-   2   0,-1

>hsa-mir-3928 3p:37-58 SRX018959
gcugaagcucuaagguuccgccugcgggcaggaagcGGAGGAACCUUGGAGCUUCGGC
(((((((((((((((((((..((((.........)))).)))))))))))))))))))
------------------------------------GGAGGAACCUUGGAGCUUCGG-   3   0,-1
------------------------------------GGAGGAACCUUGGAGCUUCGGa   3   0,-1
------------------------------------GGAGGAACCUUGGAGCUUCGGu   5   0,-1
------------------------------------GGAGGAACCUUGGAGCUUCGGC   11   0,0
------------------------------------GGAGGAACCUUGGAGCUUCGGCa 16   0,0
------------------------------------GGAGGAACCUUGGAGCUUCGGCau   2   0,0
------------------------------------GGAGGAACCUUGGAGCUUCGGCu 6   0,0
--------------------------------------AGGAACCUUGGAGCUUCGGC   5   2,0

>hsa-mir-3936 3p:66-87 SRX018959
augauucagagcaucuguccagugucugcuguagaucccucaaauccguguuuggacgcuucuggUAAGGGGUGUAUGGC
AGAUGCAcccgacagaugcacuuggcagca
.((.(..((.(((((((((..((((((((((((.((((((.....((((((....))))....))..)))))).))))))
))))))....))))))))).))..)))...
---UAAGGGGUGUAUGGC
AGAUGC------------------------   2   0,-1

---UAAGGGGUGUAUGGC

AGAUGCA----------------------- 2   0,0

---UAAGGGGUGUAUGGC

AGAUGCAu---------------------- 2   0,0

>hsa-mir-3942   5p:23-44   SRX018959

ucuucaguaugacaccucaaagAAGCAAUACUGUUACCUGAAAUaggcugcgaagauaacaguauuucagauaacaguau

uacaucuuugaagugucauauucacugac

...((((((((((((.(((((((.(.((((((((((.(((((((..((((.........))))))))))).)))))))))

).).))))))).)))))))....))))).

-----------------------AGCAAUACUGUUACCUGAAAUA-----------------------------------

----------------------------- 2   1,1

>hsa-mir-409   5p:15-37;3p:47-68   SRX018959

ugguacucggggagAGGUUACCCGAGCAACUUUGCAUcuggacgacGAAUGUUGCUCGGUGAACCCCUuuucgguauca

.(((((.((..(((.((((..((((((((((((((........).)))).)))))))))..)))).)))..))))))).

--------------AGGUUACCCGAGCAACUU---

3   0,-5

--------------AGGUUACCCGAGCAACUUU--

51   0,-4

--------------AGGUUACCCGAGCAACUUUG---

4   0,-3

--------------AGGUUACCCGAGCAACUUUGC--

5   0,-2

--------------AGGUUACCCGAGCAACUUUGCA---

93   0,-1

--------------AGGUUACCCGAGCAACUUUGCAa--

3   0,-1

--------------AGGUUACCCGAGCAACUUUGCAU--

71   0,0

--------------AGGUUACCCGAGCAACUUUGCAUa---

6   0,0

--------------AGGUUACCCGAGCAACUUUGCAUC---

6   0,1

---------------GGUUACCCGAGCAACUUUGCA---

2   1,-1

---CGAAUGUUGCUCGGUGAA----------------

29   -1,-5

---CGAAUGUUGCUCGGUGAAC---------------
6 -1,-4
---CGAAUGUUGCUCGGUGAACC--------------
3 -1,-3
---CGAAUGUUGCUCGGUGAACCC-------------
2 -1,-2
---CGAAUGUUGCUCGGUGAACCCC------------
2 -1,-1
---CGAAUGUUGCUCGGUGAACCCCU-----------
7 -1,0
---CGAAUGUUGCUCGGUGAACCCCUU----------
3 -1,1
---CGAAUGUUGCUCGGUGAACCCCUUa---------
2 -1,1
--GAAUGUUGCUCGGUGAAC---------------
6 0,-4
--GAAUGUUGCUCGGUGAACC--------------
4 0,-3
--GAAUGUUGCUCGGUGAACCC-------------
2 0,-2
--GAAUGUUGCUCGGUGAACCCC------------
6 0,-1
--GAAUGUUGCUCGGUGAACCCCU-----------
19 0,0
--GAAUGUUGCUCGGUGAACCCCUa----------
4 0,0
--GAAUGUUGCUCGGUGAACCCCUU----------
5 0,1
--GAAUGUUGCUCGGUGAACCCCUUU---------
3 0,2

>hsa-mir-410 3p:50-70 SRX018959
gguaccugagaagagguugucugugaugaguucgcuuuuauuaaugacgAAUAUAACACAGAUGGCCUGUuuucaguacc
(((((.((((((.((((..((((((.((.((((((..........).))))).)).))))))..)))).)))))))))))
---AAUAUAACACAGAUGGCCU------------
6 0,-2
---AAUAUAACACAGAUGGCCUGU----------
2 0,0

>hsa-mir-411 MA:16-36;mi:51-72 SRX018959
ugguacuuggagagaUAGUAGACCGUAUAGCGUACGcuuuaucugugacgUAUGUAACACGGUCCACUAACCcucaguau
caaauccaucccccgag
(((((((..(((.(.((((.((((((.(.((((((((.........).))))))).).)))))).)))).).))))))))
))...............
--------------AUAGUAGACCGUAUAGCG--
----------------- 6 -1,-4
--------------AUAGUAGACCGUAUAGCGU---
----------------- 188 -1,-3
--------------AUAGUAGACCGUAUAGCGUA--
----------------- 88 -1,-2
--------------AUAGUAGACCGUAUAGCGUAa---
----------------- 15 -1,-2
--------------AUAGUAGACCGUAUAGCGUAaa--
----------------- 3 -1,-2
--------------AUAGUAGACCGUAUAGCGUAu---
----------------- 2 -1,-2
--------------AUAGUAGACCGUAUAGCGUAC---
----------------- 262 -1,-1
--------------AUAGUAGACCGUAUAGCGUACa--
----------------- 3 -1,-1
--------------AUAGUAGACCGUAUAGCGUACaa---
----------------- 2 -1,-1
--------------AUAGUAGACCGUAUAGCGUACG--
----------------- 32 -1,0
--------------AUAGUAGACCGUAUAGCGUACGaa--
----------------- 2 -1,0
---------------UAGUAGACCGUAUAGCGU---
----------------- 149 0,-3
---------------UAGUAGACCGUAUAGCGUA--
----------------- 110 0,-2
---------------UAGUAGACCGUAUAGCGUAa---
----------------- 7 0,-2
---------------UAGUAGACCGUAUAGCGUAC---
----------------- 274 0,-1
---------------UAGUAGACCGUAUAGCGUACa--
----------------- 2 0,-1

---------------UAGUAGACCGUAUAGCGUACaa---
----------------	2	0,-1
---------------UAGUAGACCGUAUAGCGUACG--
----------------	49	0,0
---------------UAGUAGACCGUAUAGCGUACGa---
----------------	2	0,0
----------------AGUAGACCGUAUAGCGUAC---
----------------	4	1,-1
--UAUGUAACACGGUCCACUAuu---------
----------------	2	0,-3
--UAUGUAACACGGUCCACUAA----------
----------------	2	0,-2
--UAUGUAACACGGUCCACUAAC---------
----------------	2	0,-1
--UAUGUAACACGGUCCACUAACu--------
----------------	6	0,-1

>hsa-mir-412	3p:54-76	SRX018959
cugggguacggggauggauggucgaccaguuggaaaguaauuguuucuaauguACUUCACCUGGUCCACUAGCCGUccgu
auccgcugcag
...((((((..((((((.((((.((((((.(((..((((((((....)))).))))))).)))))).)))).))))))))
))))......
------------------UGGUCGACCAGUUGGAAAGUAAU---------------------------------------
----------- 2

>hsa-mir-421	3p:48-70	SRX018959
gcacauuguaggccucauuaaauguuuguugaaugaaaaaaugaaucAUCAACAGACAUUAAUUGGGCGCcugcucugug
aucuc
.((((..((((((((((...((((((((((((.(((.........)))))))))))))))...)))).))))))..))))
.....
---AUCAACAGACAUUAAUUG---------------
-----	29	0,-5
---AUCAACAGACAUUAAUUGG--------------
-----	16	0,-4
---AUCAACAGACAUUAAUUGGG-------------
-----	4	0,-3
---AUCAACAGACAUUAAUUGGGCG-----------
-----	2	0,-1

--AUCAACAGACAUUAAUUGGGCGCau--------
-----	2	0,0
---UCAACAGACAUUAAUUGGGCGC----------
-----	3	1,0

>hsa-mir-423	5p:17-39;3p:53-75	SRX018959
auaaaggaaguuaggcUGAGGGGCAGAGAGCGAGACUUUucuauuuuccaaaAGCUCGGUCUGAGGCCCCUCAGUcuugc
uuccuaacccgcgc
....(((((((.((((((((((((..(((.((((.(((((.........))))))))).)))...))))))))))))).))
)))))..........
---------------CUGAGGGGCAGAGAGCGAGACUUa---
--------------	2	-1,-1
---------------CUGAGGGGCAGAGAGCGAGACUUU---
--------------	2	-1,0
---------------CUGAGGGGCAGAGAGCGAGACUUUU--
--------------	2	-1,1
----------------UGAGGGGCAGAGAGCGAG--
--------------	11	0,-5
----------------UGAGGGGCAGAGAGCGAGA---
--------------	42	0,-4
----------------UGAGGGGCAGAGAGCGAGAa--
--------------	8	0,-4
----------------UGAGGGGCAGAGAGCGAGAaa---
--------------	13	0,-4
----------------UGAGGGGCAGAGAGCGAGAu--
--------------	2	0,-4
----------------UGAGGGGCAGAGAGCGAGAC--
--------------	50	0,-3
----------------UGAGGGGCAGAGAGCGAGACa---
--------------	5	0,-3
----------------UGAGGGGCAGAGAGCGAGACU---
--------------	81	0,-2
----------------UGAGGGGCAGAGAGCGAGACUa--
--------------	3	0,-2
----------------UGAGGGGCAGAGAGCGAGACUaa---
--------------	7	0,-2
----------------UGAGGGGCAGAGAGCGAGACUau---
--------------	5	0,-2

----------------UGAGGGGCAGAGAGCGAGACUU--
-------------- 141 0,-1
----------------UGAGGGGCAGAGAGCGAGACUUa---
-------------- 54 0,-1
----------------UGAGGGGCAGAGAGCGAGACUUaa--
-------------- 8 0,-1
----------------UGAGGGGCAGAGAGCGAGACUUau--
-------------- 17 0,-1
----------------UGAGGGGCAGAGAGCGAGACUUU---
-------------- 1383 0,0
----------------UGAGGGGCAGAGAGCGAGACUUUa--
-------------- 34 0,0
----------------UGAGGGGCAGAGAGCGAGACUUUaa---------------------------------------
-------------- 5 0,0
----------------UGAGGGGCAGAGAGCGAGACUUUau---------------------------------------
-------------- 13 0,0
----------------UGAGGGGCAGAGAGCGAGACUUUU--
-------------- 308 0,1
----------------UGAGGGGCAGAGAGCGAGACUUUUa---------------------------------------
-------------- 4 0,1
----------------UGAGGGGCAGAGAGCGAGACUUUUu---------------------------------------
-------------- 25 0,1
-----------------GAGGGGCAGAGAGCGAGACU---
-------------- 2 1,-2
-----------------GAGGGGCAGAGAGCGAGACUaa---
-------------- 2 1,-2
-----------------GAGGGGCAGAGAGCGAGACUU--
-------------- 4 1,-1
-----------------GAGGGGCAGAGAGCGAGACUUU---
-------------- 54 1,0
-----------------GAGGGGCAGAGAGCGAGACUUUU--
-------------- 6 1,1
------------------AGGGGCAGAGAGCGAGACU---
-------------- 2 2,-2
------------------AGGGGCAGAGAGCGAGACUUU---
-------------- 10 2,0
------------------AGGGGCAGAGAGCGAGACUUUU--
-------------- 42 2,1

--AAGCUCGGUCUGAGGCCCC----------
-------------- 3 -1,-5
--AAGCUCGGUCUGAGGCCCCUaa-------
-------------- 4 -1,-4
--AAGCUCGGUCUGAGGCCCCUu--------
-------------- 2 -1,-4
--AAGCUCGGUCUGAGGCCCCUuu-------
-------------- 4 -1,-4
--AAGCUCGGUCUGAGGCCCCUC--------
-------------- 2 -1,-3
--AAGCUCGGUCUGAGGCCCCUCu-------
-------------- 2 -1,-3
--AAGCUCGGUCUGAGGCCCCUCA-------
-------------- 9 -1,-2
--AAGCUCGGUCUGAGGCCCCUCAa------
-------------- 7 -1,-2
--AAGCUCGGUCUGAGGCCCCUCAu------
-------------- 3 -1,-2
--AAGCUCGGUCUGAGGCCCCUCAG------
-------------- 13 -1,-1
--AAGCUCGGUCUGAGGCCCCUCAGU-----
-------------- 45 -1,0
--AAGCUCGGUCUGAGGCCCCUCAGUa----
-------------- 3 -1,0
---AGCUCGGUCUGAGGCCCC----------
-------------- 6 0,-5
---AGCUCGGUCUGAGGCCCCU---------
-------------- 2 0,-4
---AGCUCGGUCUGAGGCCCCUaa-------
-------------- 2 0,-4
---AGCUCGGUCUGAGGCCCCUC--------
-------------- 5 0,-3
---AGCUCGGUCUGAGGCCCCUCu-------
-------------- 3 0,-3
---AGCUCGGUCUGAGGCCCCUCA-------
-------------- 15 0,-2
---AGCUCGGUCUGAGGCCCCUCAa------
-------------- 4 0,-2

--AGCUCGGUCUGAGGCCCCUCAaa-----
-------------- 2 0,-2
--AGCUCGGUCUGAGGCCCCUCAau-----
-------------- 3 0,-2
--AGCUCGGUCUGAGGCCCCUCAu------
-------------- 8 0,-2
--AGCUCGGUCUGAGGCCCCUCAG------
-------------- 50 0,-1
--AGCUCGGUCUGAGGCCCCUCAGa-----
-------------- 11 0,-1
--AGCUCGGUCUGAGGCCCCUCAGU-----
-------------- 199 0,0
--AGCUCGGUCUGAGGCCCCUCAGUa----
-------------- 20 0,0
--AGCUCGGUCUGAGGCCCCUCAGUaa---
-------------- 8 0,0
--AGCUCGGUCUGAGGCCCCUCAGUau---
-------------- 22 0,0
--AGCUCGGUCUGAGGCCCCUCAGUu----
-------------- 43 0,0
--AGCUCGGUCUGAGGCCCCUCAGUuu---
-------------- 11 0,0
---GCUCGGUCUGAGGCCCCUCAGU-----
-------------- 2 1,0

>hsa-mir-424 MA:11-32;mi:48-68 SRX018959
cgaggggauaCAGCAGCAAUUCAUGUUUUGAAguguucuaaaugguuCAAAACGUGAGGCGCUGCUAUacccccucgugg
ggaagguagaaggugggg
(((((((.((.((((((..(((((((((((((.(((.....))).)))))))))))))..)))))).)).)))))))...
....
---------ACAGCAGCAAUUCAUGUUUU---
------------------ 3 -1,-3
---------ACAGCAGCAAUUCAUGUUUUG--
------------------ 3 -1,-2
---------ACAGCAGCAAUUCAUGUUUUGA---
------------------ 5 -1,-1
---------ACAGCAGCAAUUCAUGUUUUGAA--
------------------ 2 -1,0

----------CAGCAGCAAUUCAUGUUU---
------------------ 17 0,-4
----------CAGCAGCAAUUCAUGUUUa--
------------------ 3 0,-4
----------CAGCAGCAAUUCAUGUUUaa---
------------------ 2 0,-4
----------CAGCAGCAAUUCAUGUUUU--
------------------ 138 0,-3
----------CAGCAGCAAUUCAUGUUUUa---
------------------ 5 0,-3
----------CAGCAGCAAUUCAUGUUUUaa--
------------------ 2 0,-3
----------CAGCAGCAAUUCAUGUUUUG---
------------------ 518 0,-2
----------CAGCAGCAAUUCAUGUUUUGuu---
------------------ 2 0,-2
----------CAGCAGCAAUUCAUGUUUUGA--
------------------ 1720 0,-1
----------CAGCAGCAAUUCAUGUUUUGAu---
------------------ 4 0,-1
----------CAGCAGCAAUUCAUGUUUUGAA---
------------------ 519 0,0
----------CAGCAGCAAUUCAUGUUUUGAAa--
------------------ 82 0,0
----------CAGCAGCAAUUCAUGUUUUGAAaa---
------------------ 8 0,0
----------CAGCAGCAAUUCAUGUUUUGAAu--
------------------ 5 0,0
----------CAGCAGCAAUUCAUGUUUUGAAG--
------------------ 2 0,1
-----------AGCAGCAAUUCAUGUUUU--
------------------ 4 1,-3
-----------AGCAGCAAUUCAUGUUUUG---
------------------ 6 1,-2
-----------AGCAGCAAUUCAUGUUUUGA--
------------------ 28 1,-1
-----------AGCAGCAAUUCAUGUUUUGAA---
------------------ 17 1,0

---CAAAACGUGAGGCGCUGCUu-------------
------------------ 3 0,-2
---CAAAACGUGAGGCGCUGCUA-------------
------------------ 14 0,-1
---CAAAACGUGAGGCGCUGCUAa------------
------------------ 11 0,-1
---CAAAACGUGAGGCGCUGCUAaa-----------
------------------ 2 0,-1
---CAAAACGUGAGGCGCUGCUAU------------
------------------ 347 0,0
---CAAAACGUGAGGCGCUGCUAUu-----------
------------------ 13 0,0
---CAAAACGUGAGGCGCUGCUAUA-----------
------------------ 159 0,1
---CAAAACGUGAGGCGCUGCUAUAa----------
------------------ 57 0,1
---CAAAACGUGAGGCGCUGCUAUAu----------
------------------ 24 0,1
--AAAACGUGAGGCGCUGCUAU------------
------------------ 12 1,0
--AAAACGUGAGGCGCUGCUAUA-----------
------------------ 11 1,1
--AAAACGUGAGGCGCUGCUAUAa----------
------------------ 3 1,1
--AAAACGUGAGGCGCUGCUAUAu----------
------------------ 12 1,1
---AAACGUGAGGCGCUGCUAUAu----------
------------------ 4 2,1

>hsa-mir-425 MA:14-36;mi:55-76 SRX018959
gaaagcgcuuuggAAUGACACGAUCACUCCCGUUGAgugggcacccgagaagccAUCGGGAAUGUCGUGUCCGCCCagug
cucuuuc
(((((.((.((((...(((((((.((.(((((.((.((((....)).....)))).))))).))))))))...)))).)
).)))))
-------------AAUGACACGAUCACUCCC---
------- 7 0,-5
-------------AAUGACACGAUCACUCCCG--
------- 2 0,-4

-------------AAUGACACGAUCACUCCCGU---
------- 2   0,-3
-------------AAUGACACGAUCACUCCCGUU--
------- 6   0,-2
-------------AAUGACACGAUCACUCCCGUUG---
------- 15 0,-1
-------------AAUGACACGAUCACUCCCGUUGA--
------- 39 0,0
-------------AAUGACACGAUCACUCCCGUUGAG---
------- 10 0,1
-------------AAUGACACGAUCACUCCCGUUGAGa--
------- 2   0,1
-------------AAUGACACGAUCACUCCCGUUGAGU--
------- 6   0,2
--------------AUGACACGAUCACUCCCGUUGA--
------- 19 1,0
--------------AUGACACGAUCACUCCCGUUGAG---
------- 4   1,1
--------------AUGACACGAUCACUCCCGUUGAGU--
------- 3   1,2
---CAUCGGGAAUGUCGUGUCC--------
------- 2   -1,-4
---CAUCGGGAAUGUCGUGUCCGu------
------- 3   -1,-3
---CAUCGGGAAUGUCGUGUCCGCu-----
------- 3   -1,-2
---CAUCGGGAAUGUCGUGUCCGCC-----
------- 13 -1,-1
---CAUCGGGAAUGUCGUGUCCGCCa----
------- 3   -1,-1
--AUCGGGAAUGUCGUGUCC--------
------- 2   0,-4
--AUCGGGAAUGUCGUGUCCGC------
------- 2   0,-2
--AUCGGGAAUGUCGUGUCCGCC-----
------- 9   0,-1

>hsa-mir-4286   5p:11-27   SRX018959

uacuuauggcACCCCACUCCUGGUACCauagucauaaguuaggagauguuagagcugugaguaccaugacuuaagugugg
uggcuuaaacaug
...(((.(.((((.(((((.((((((((((((..((((((....))).)))..)))))).)))))).)).....))).))
)).).))).....
----------ACCCCACUCCUGGUACCA--
-------------   4   0,1
----------ACCCCACUCCUGGUACCAau--
-------------   2   0,1
----------ACCCCACUCCUGGUACCAUA--
-------------   2   0,3

>hsa-mir-429   3p:51-72   SRX018959
cgccggccgaugggcgucuuaccagacaugguuagaccuggcccucugucUAAUACUGUCUGGUAAAACCGUccauccgc
ugc
...((((.((((((((..((((((((((..(((((((..........)))))))..))))))))))...)))))))).))
)).
--UAAUACUGUCUGGUAAAA------------
--- 9   0,-4
--UAAUACUGUCUGGUAAAAC-----------
--- 4   0,-3
--UAAUACUGUCUGGUAAAACCu---------
--- 2   0,-2
--UAAUACUGUCUGGUAAAACCG---------
--- 17   0,-1
--UAAUACUGUCUGGUAAAACCGU--------
--- 51   0,0
---AAUACUGUCUGGUAAAACCGU--------
--- 3   1,0

>hsa-mir-432   MA:14-36;mi:62-82   SRX018959
ugacuccuccaggUCUUGGAGUAGGUCAUUGGGUGGauccucuauuuccuuacgugggccaCUGGAUGGCUCCUCCAUGU
CUuggaguagauca
.((((.(((((((.(.(((((..(((((((.(((((.(((.(((......)).).)))))))).))))))).))))).).
))))))).)).)).
-------------UCUUGGAGUAGGUCAUUG---
--------------   53   0,-5
-------------UCUUGGAGUAGGUCAUUGG--
--------------   113 0,-4

-------------UCUUGGAGUAGGUCAUUGGa--
-------------- 2 0,-4
-------------UCUUGGAGUAGGUCAUUGGaa---
-------------- 3 0,-4
-------------UCUUGGAGUAGGUCAUUGGu--
-------------- 2 0,-4
-------------UCUUGGAGUAGGUCAUUGGG--
-------------- 128 0,-3
-------------UCUUGGAGUAGGUCAUUGGGa---
-------------- 7 0,-3
-------------UCUUGGAGUAGGUCAUUGGGaa--
-------------- 9 0,-3
-------------UCUUGGAGUAGGUCAUUGGGU---
-------------- 39 0,-2
-------------UCUUGGAGUAGGUCAUUGGGUa--
-------------- 13 0,-2
-------------UCUUGGAGUAGGUCAUUGGGUaa---
-------------- 20 0,-2
-------------UCUUGGAGUAGGUCAUUGGGUau---
-------------- 25 0,-2
-------------UCUUGGAGUAGGUCAUUGGGUu--
-------------- 5 0,-2
-------------UCUUGGAGUAGGUCAUUGGGUG--
-------------- 58 0,-1
-------------UCUUGGAGUAGGUCAUUGGGUGa---
-------------- 23 0,-1
-------------UCUUGGAGUAGGUCAUUGGGUGau--
-------------- 9 0,-1
-------------UCUUGGAGUAGGUCAUUGGGUGu---
-------------- 8 0,-1
-------------UCUUGGAGUAGGUCAUUGGGUGuu--
-------------- 2 0,-1
-------------UCUUGGAGUAGGUCAUUGGGUGG---
-------------- 7 0,0
-------------UCUUGGAGUAGGUCAUUGGGUGGu--
-------------- 3 0,0

>hsa-mir-4326 5p:11-30 SRX018959

gcugcucugcUGUUCCUCUGUCUCCCAGACucuggguggauggagcaggucgggggcca
(((.((...(((((((...(((((((((...))))).))))))))))))...)).)))..
----------UGUUCCUCUGUCUCCCAGAC---------------------------- 2 0,0
----------UGUUCCUCUGUCUCCCAGACUCU------------------------- 2 0,3

>hsa-mir-433 3p:64-85 SRX018959
ccggggagaaguacggugagccugucauuauucagagaggcuagaucccucuguguugagaaggAUCAUGAUGGGCUCCUC
GGUGUucuccagg
((..((((((...(((.(((((((((((.(((((.(((((......))))).))........))).))))))))))).))
)...)))))).))
---GAUCAUGAUGGGCUCCUC
GGUGU-------- 2 -1,0
--AUCAUGAUGGGCUCCUC
GG----------- 5 0,-3
--AUCAUGAUGGGCUCCUC
GGU---------- 3 0,-2
--AUCAUGAUGGGCUCCUC
GGUG--------- 2 0,-1
--AUCAUGAUGGGCUCCUC
GGUGU-------- 42 0,0
--AUCAUGAUGGGCUCCUC
GGUGUa------- 3 0,0
--AUCAUGAUGGGCUCCUC
GGUGUau------ 2 0,0
--AUCAUGAUGGGCUCCUC
GGUGUUa------ 3 0,1
--AUCAUGAUGGGCUCCUC
GGUGUUu------ 7 0,1
---UCAUGAUGGGCUCCUC
GGUG--------- 3 1,-1
---UCAUGAUGGGCUCCUC
GGUGU-------- 3 1,0

>hsa-mir-450a-1 5p:18-39 SRX018959
aaacgauacuaaacuguUUUUGCGAUGUGUUCCUAAUAUgcacuauaaauauauugggaacauuuugcauguauaguuuu
guaucaauaua
....(((((.(((((((...(((((.((((((((((((((.........))))))))))))).)))))...))))))).
)))))......

-----------------UUUUGCGAUGUGUUCCUAA--
----------- 2	0,-3
-----------------UUUUGCGAUGUGUUCCUAAU---
----------- 14	0,-2
-----------------UUUUGCGAUGUGUUCCUAAUA--
----------- 9	0,-1
-----------------UUUUGCGAUGUGUUCCUAAUAU---
----------- 22	0,0
--AUUGGGAACAUUUUGCAUGUAU------
----------- 3

>hsa-mir-450a-2 5p:23-44	SRX018959
ccaaagaaagaugcuaaacuauUUUUGCGAUGUGUUCCUAAUAUguaauauaaauguauuggggacauuuugcauucaua
guuuuguaucaauaauaugg
.........(((((.(((((((...(((((.(((((((((((((((.......))))))))))))))).)))))...)))
)))).)))))..........
----------------------UUUUGCGAUGUGUUCCUAA---------------------------------------
--------------------	2	0,-3
----------------------UUUUGCGAUGUGUUCCUAAU--------------------------------------
--------------------	14	0,-2
----------------------UUUUGCGAUGUGUUCCUAAUA-------------------------------------
--------------------	9	0,-1
----------------------UUUUGCGAUGUGUUCCUAAUAU------------------------------------
--------------------	22	0,0
---AUUGGGGACAUUUUGCAUa----
--------------------	2
---AUUGGGGACAUUUUGCAUU----
--------------------	12
---AUUGGGGACAUUUUGCAUUCA--
--------------------	5
---AUUGGGGACAUUUUGCAUUCAU-
--------------------	28
--UUGGGGACAUUUUGCAUUCAU-
--------------------	10
--UUGGGGACAUUUUGCAUUCAUu
--------------------	2

>hsa-mir-450b	5p:11-32;3p:48-69	SRX018959

gcagaauuauUUUUGCAAUAUGUUCCUGAAUAuguaauauaaguguaUUGGGAUCAUUUUGCAUCCAUAguuuuguau
((((((((((...(((((.(((.((((.((((..(.......)..)))))))).))).)))))...))))))))))..
----------UUUUGCAAUAUGUUCCUG--
6 0,-4
----------UUUUGCAAUAUGUUCCUGA---
6 0,-3
----------UUUUGCAAUAUGUUCCUGAA--
6 0,-2
----------UUUUGCAAUAUGUUCCUGAAU---
7 0,-1
----------UUUUGCAAUAUGUUCCUGAAUA--
4 0,0
----------UUUUGCAAUAUGUUCCUGAAUAa---
15 0,0

>hsa-mir-451 5p:17-38 SRX018959
cuugggaauggcaaggAAACCGUUACCAUUACUGAGUUuaguaaugguaaugguucucuugcuauacccaga
.((((.((((((((..((((((((((((((((((....))))))))))))))))))..)))))))).))))).
----------------AAACCGUUACCAUUACUG-------------------------------------- 11
0,-4
----------------AAACCGUUACCAUUACUGAG------------------------------------ 49
0,-2
----------------AAACCGUUACCAUUACUGAGau---------------------------------- 2
0,-2
----------------AAACCGUUACCAUUACUGAGU----------------------------------- 52
0,-1
----------------AAACCGUUACCAUUACUGAGUU---------------------------------- 476
0,0
----------------AAACCGUUACCAUUACUGAGUUa--------------------------------- 2
0,0
----------------AAACCGUUACCAUUACUGAGUUU--------------------------------- 163
0,1
----------------AAACCGUUACCAUUACUGAGUUUA-------------------------------- 43
0,2
----------------AAACCGUUACCAUUACUGAGUUUAG------------------------------- 11
0,3

>hsa-mir-452 MA:14-35;mi:58-79 SRX018959

gcuaagcacuuacAACUGUUUGCAGAGGAAACUGAgacuuuguaacuaugucucaguCUCAUCUGCAAAGAAGUAAGUGc
uuugc
((.((((((((((...(.((((((((.((.((((((((...........)))))))).)).)))))))).).))))))))
)).))
-------------AACUGUUUGCAGAGGAAA---
-----   26 0,-4
-------------AACUGUUUGCAGAGGAAAC--
-----   103 0,-3
-------------AACUGUUUGCAGAGGAAACU---
-----   156 0,-2
-------------AACUGUUUGCAGAGGAAACUG--
-----   3   0,-1
-------------AACUGUUUGCAGAGGAAACUGA---
-----   15 0,0
-------------AACUGUUUGCAGAGGAAACUGAG--
-----   11 0,1
--------------ACUGUUUGCAGAGGAAACU---
-----   2   1,-2

>hsa-mir-454   mi:24-45;MA:64-86   SRX018959
ucuguuuaucaccagauccuagaACCCUAUCAAUAUUGUCUCUGCuguguaaauaguucugagUAGUGCAAUAUUGCUUA
UAGGGUuuugguguuuggaaagaacaaugggcagg
.((((((((.(((((..(((((((((((((((((((((((((.(((((....)))))...)))....)))))))))...)
)))))))))))..)))))...))....))))))).
---UAGUGCAAUAUUGCUUA
UA--------------------------------- 5   0,-4
---UAGUGCAAUAUUGCUUA
UAGGGU----------------------------- 3   0,0

>hsa-mir-455   5p:16-37;3p:54-74   SRX018959
ucccuggcgugagggUAUGUGCCUUUGGACUACAUCGuggaagccagcaccauGCAGUCCAUGGGCAUAUACACuugccu
caaggccuaugucauc
.....(((.(((((((((((((((.((((((.(((.(((........))).))).)))))).))))))))))).....)))
))..))).........
--------------GUAUGUGCCUUUGGACUA--
----------------   3   -1,-5
--------------GUAUGUGCCUUUGGACUAC---
----------------   2   -1,-4

--------------GUAUGUGCCUUUGGACUACA---
---------------- 7 -1,-3
--------------GUAUGUGCCUUUGGACUACAU--
---------------- 102 -1,-2
--------------GUAUGUGCCUUUGGACUACAUa---
---------------- 45 -1,-2
--------------GUAUGUGCCUUUGGACUACAUaa--
---------------- 3 -1,-2
--------------GUAUGUGCCUUUGGACUACAUu---
---------------- 15 -1,-2
--------------GUAUGUGCCUUUGGACUACAUC---
---------------- 12 -1,-1
--------------GUAUGUGCCUUUGGACUACAUCG--
---------------- 12 -1,0
--------------GUAUGUGCCUUUGGACUACAUCGU---
---------------- 37 -1,1
--------------GUAUGUGCCUUUGGACUACAUCGUG--
---------------- 2 -1,2
---------------UAUGUGCCUUUGGACUACA---
---------------- 16 0,-3
---------------UAUGUGCCUUUGGACUACAU--
---------------- 22 0,-2
---------------UAUGUGCCUUUGGACUACAUa---
---------------- 140 0,-2
---------------UAUGUGCCUUUGGACUACAUC---
---------------- 75 0,-1
---------------UAUGUGCCUUUGGACUACAUCa--
---------------- 2 0,-1
---------------UAUGUGCCUUUGGACUACAUCG--
---------------- 138 0,0
---------------UAUGUGCCUUUGGACUACAUCGa---
---------------- 13 0,0
---------------UAUGUGCCUUUGGACUACAUCGU---
---------------- 83 0,1
---------------UAUGUGCCUUUGGACUACAUCGUu--
---------------- 3 0,1
---------------UAUGUGCCUUUGGACUACAUCGUG--
---------------- 2 0,2

---------------UAUGUGCCUUUGGACUACAUCGUGu---
---------------- 3 0,2
----------------AUGUGCCUUUGGACUACAUCG--
---------------- 4 1,0
----------------AUGUGCCUUUGGACUACAUCGU---
---------------- 4 1,1
-----------------UGUGCCUUUGGACUACAUCGU---
---------------- 3 2,1
-----------------UGUGCCUUUGGACUACAUCGUG--
---------------- 8 2,2
-----------------UGUGCCUUUGGACUACAUCGUGa---
---------------- 7 2,2
-----------------UGUGCCUUUGGACUACAUCGUGu---
---------------- 9 2,2
--AUGCAGUCCAUGGGCAUAU----------
---------------- 2 -2,-4
--AUGCAGUCCAUGGGCAUAUuu--------
---------------- 2 -2,-4
--AUGCAGUCCAUGGGCAUAUA---------
---------------- 25 -2,-3
--AUGCAGUCCAUGGGCAUAUAa--------
---------------- 12 -2,-3
--AUGCAGUCCAUGGGCAUAUAu--------
---------------- 9 -2,-3
--AUGCAGUCCAUGGGCAUAUAC--------
---------------- 32 -2,-2
--AUGCAGUCCAUGGGCAUAUACu-------
---------------- 2 -2,-2
--AUGCAGUCCAUGGGCAUAUACA-------
---------------- 51 -2,-1
--AUGCAGUCCAUGGGCAUAUACAa------
---------------- 4 -2,-1
--AUGCAGUCCAUGGGCAUAUACAC------
---------------- 32 -2,0
--AUGCAGUCCAUGGGCAUAUACACa-----
---------------- 2 -2,0
--AUGCAGUCCAUGGGCAUAUACACU-----
---------------- 7 -2,1

---UGCAGUCCAUGGGCAUAUA---------
---------------- 3 -1,-3
---UGCAGUCCAUGGGCAUAUAC--------
---------------- 2 -1,-2
---UGCAGUCCAUGGGCAUAUACA-------
---------------- 16 -1,-1
---UGCAGUCCAUGGGCAUAUACAC------
---------------- 7 -1,0
---UGCAGUCCAUGGGCAUAUACACa-----
---------------- 2 -1,0
--GCAGUCCAUGGGCAUAUA---------
---------------- 65 0,-3
--GCAGUCCAUGGGCAUAUAa--------
---------------- 3 0,-3
--GCAGUCCAUGGGCAUAUAC--------
---------------- 14 0,-2
--GCAGUCCAUGGGCAUAUACu-------
---------------- 4 0,-2
--GCAGUCCAUGGGCAUAUACA-------
---------------- 263 0,-1
--GCAGUCCAUGGGCAUAUACAa------
---------------- 16 0,-1
--GCAGUCCAUGGGCAUAUACAu------
---------------- 4 0,-1
--GCAGUCCAUGGGCAUAUACAuu-----
---------------- 6 0,-1
--GCAGUCCAUGGGCAUAUACAC------
---------------- 525 0,0
--GCAGUCCAUGGGCAUAUACACa-----
---------------- 60 0,0
--GCAGUCCAUGGGCAUAUACACaa----
---------------- 2 0,0
--GCAGUCCAUGGGCAUAUACACU-----
---------------- 451 0,1
--GCAGUCCAUGGGCAUAUACACUa----
---------------- 8 0,1
--GCAGUCCAUGGGCAUAUACACUU----
---------------- 14 0,2

--GCAGUCCAUGGGCAUAUACACUUa---
----------------	4	0,2
---CAGUCCAUGGGCAUAUACA-------
----------------	9	1,-1
---CAGUCCAUGGGCAUAUACAa------
----------------	2	1,-1
---CAGUCCAUGGGCAUAUACAC------
----------------	76	1,0
---CAGUCCAUGGGCAUAUACACa-----
----------------	4	1,0
---CAGUCCAUGGGCAUAUACACU-----
----------------	94	1,1
---CAGUCCAUGGGCAUAUACACUa----
----------------	4	1,1
---CAGUCCAUGGGCAUAUACACUaa---
----------------	2	1,1
---CAGUCCAUGGGCAUAUACACUU----
----------------	56	1,2
---CAGUCCAUGGGCAUAUACACUUa---
----------------	21	1,2
---CAGUCCAUGGGCAUAUACACUUu---
----------------	30	1,2
---CAGUCCAUGGGCAUAUACACUUuu--
----------------	2	1,2

>hsa-mir-483	5p:8-29;3p:48-68	SRX018959
gaggggg AAGACGGGAGGAAAGAAGGGAGugguuccaucacgccuccUCACUCCUCUCCUCCCGUCUUcuccucuc
(((((((((((((((((((.((.((.(((.(((........)))..))).)).)).))))))))))))).))))))
-------AAGACGGGAGGAAAGAAG---
	204	0,-4
-------AAGACGGGAGGAAAGAAGa--
	7	0,-4
-------AAGACGGGAGGAAAGAAGaa---
	2	0,-4
-------AAGACGGGAGGAAAGAAGG--
	374	0,-3
-------AAGACGGGAGGAAAGAAGGa---
	31	0,-3

-------AAGACGGGAGGAAAGAAGGaa---
24 0,-3
-------AAGACGGGAGGAAAGAAGGu--
12 0,-3
-------AAGACGGGAGGAAAGAAGGG--
943 0,-2
-------AAGACGGGAGGAAAGAAGGGu---
15 0,-2
-------AAGACGGGAGGAAAGAAGGGuu--
8 0,-2
-------AAGACGGGAGGAAAGAAGGGA---
2238 0,-1
-------AAGACGGGAGGAAAGAAGGGAa--
817 0,-1
-------AAGACGGGAGGAAAGAAGGGAaa---
439 0,-1
-------AAGACGGGAGGAAAGAAGGGAau---
109 0,-1
-------AAGACGGGAGGAAAGAAGGGAu--
188 0,-1
-------AAGACGGGAGGAAAGAAGGGAuu---
65 0,-1
-------AAGACGGGAGGAAAGAAGGGAG--
4462 0,0
-------AAGACGGGAGGAAAGAAGGGAGa---
1039 0,0
-------AAGACGGGAGGAAAGAAGGGAGaa--
85 0,0
-------AAGACGGGAGGAAAGAAGGGAGau--
59 0,0
-------AAGACGGGAGGAAAGAAGGGAGU---
645 0,1
-------AAGACGGGAGGAAAGAAGGGAGUa--
16 0,1
-------AAGACGGGAGGAAAGAAGGGAGUaa---
9 0,1
-------AAGACGGGAGGAAAGAAGGGAGUau---
35 0,1

-------AAGACGGGAGGAAAGAAGGGAGUu---

100 0,1

-------AAGACGGGAGGAAAGAAGGGAGUuu--

42 0,1

-------AAGACGGGAGGAAAGAAGGGAGUG---

2 0,2

-------AAGACGGGAGGAAAGAAGGGAGUGG--

13 0,3

--------AGACGGGAGGAAAGAAGG--

26 1,-3

--------AGACGGGAGGAAAGAAGGG---

62 1,-2

--------AGACGGGAGGAAAGAAGGGu--

2 1,-2

--------AGACGGGAGGAAAGAAGGGA--

87 1,-1

--------AGACGGGAGGAAAGAAGGGAa---

6 1,-1

--------AGACGGGAGGAAAGAAGGGAu---

5 1,-1

--------AGACGGGAGGAAAGAAGGGAG---

181 1,0

--------AGACGGGAGGAAAGAAGGGAGU--

142 1,1

--------AGACGGGAGGAAAGAAGGGAGUa---

2 1,1

--------AGACGGGAGGAAAGAAGGGAGUau--

2 1,1

--------AGACGGGAGGAAAGAAGGGAGUu---

3 1,1

--------AGACGGGAGGAAAGAAGGGAGUuu--

3 1,1

--------AGACGGGAGGAAAGAAGGGAGUG---

42 1,2

--------AGACGGGAGGAAAGAAGGGAGUGa--

2 1,2

--------AGACGGGAGGAAAGAAGGGAGUGG--

150 1,3

--------AGACGGGAGGAAAGAAGGGAGUGGU--
3 1,4
---------GACGGGAGGAAAGAAGGGA---
2 2,-1
---------GACGGGAGGAAAGAAGGGAGaa--
4 2,0
--UCACUCCUCUCCUCCCGUCU---------
2 0,-1
--UCACUCCUCUCCUCCCGUCUU--------
14 0,0
--UCACUCCUCUCCUCCCGUCUUa-------
2 0,0
--UCACUCCUCUCCUCCCGUCUUaa------
5 0,0
--UCACUCCUCUCCUCCCGUCUUau------
2 0,0
--UCACUCCUCUCCUCCCGUCUUu-------
12 0,0
--UCACUCCUCUCCUCCCGUCUUC-------
14 0,1
--UCACUCCUCUCCUCCCGUCUUCU------
2 0,2
---CACUCCUCUCCUCCCGUCUU--------
4 1,0
---CACUCCUCUCCUCCCGUCUUa-------
2 1,0
---CACUCCUCUCCUCCCGUCUUC-------
4 1,1
---CACUCCUCUCCUCCCGUCUUCU------
6 1,2

>hsa-mir-484 5p:8-29 SRX018959
agccucgUCAGGCUCAGUCCCCUCCCGAUaaaccccuaaauagggacuuucccggggggugacccuggcuuuuuggcg
.(((..((((((....(((.(((((((..(((.((((....))))..)))..))))))).)))))))))......))).
-------UCAGGCUCAGUCCCCUCCC--
5 0,-3
-------UCAGGCUCAGUCCCCUCCCGA--
12 0,-1

-------UCAGGCUCAGUCCCCUCCCGAa---

9 0,-1

-------UCAGGCUCAGUCCCCUCCCGAaa--

12 0,-1

-------UCAGGCUCAGUCCCCUCCCGAU--

57 0,0

-------UCAGGCUCAGUCCCCUCCCGAUu---

12 0,0

-------UCAGGCUCAGUCCCCUCCCGAUuu--

6 0,0

-------UCAGGCUCAGUCCCCUCCCGAUA---

13 0,1

-------UCAGGCUCAGUCCCCUCCCGAUAu--

5 0,1

-------UCAGGCUCAGUCCCCUCCCGAUAA--

8 0,2

---------AGGCUCAGUCCCCUCCCGAU--

6 2,0

>hsa-mir-485 5p:9-30;3p:46-67 SRX018959

acuuggagAGAGGCUGGCCGUGAUGAAUUCgauucaucaaagcgaGUCAUACACGGCUCUCCUCUCUuuuagu

(((..((((((((..(((((((((((.((((...........)))))))).)))))))..))))))))..)))

--------AGAGGCUGGCCGUGAUGAA-- 2

0,-3

--------AGAGGCUGGCCGUGAUGAAau-- 3

0,-3

--------AGAGGCUGGCCGUGAUGAAU--- 5

0,-2

--------AGAGGCUGGCCGUGAUGAAUa-- 14

0,-2

--------AGAGGCUGGCCGUGAUGAAUU-- 100

0,-1

--------AGAGGCUGGCCGUGAUGAAUUa--- 2

0,-1

--------AGAGGCUGGCCGUGAUGAAUUu--- 11

0,-1

--------AGAGGCUGGCCGUGAUGAAUUC--- 41

0,0

--------AGAGGCUGGCCGUGAUGAAUUCa-- 3 0,0
--------AGAGGCUGGCCGUGAUGAAUUCu-- 8 0,0
--------AGAGGCUGGCCGUGAUGAAUUCG-- 58 0,1
--------AGAGGCUGGCCGUGAUGAAUUCGu--- 4 0,1
--------AGAGGCUGGCCGUGAUGAAUUCGA--- 3 0,2
--AGUCAUACACGGCUCUCCUCU-------- 4 -1,-2
--AGUCAUACACGGCUCUCCUCUC------- 2 -1,-1
--AGUCAUACACGGCUCUCCUCUCU------ 3 -1,0
---GUCAUACACGGCUCUCCU---------- 3 0,-4
---GUCAUACACGGCUCUCCUC--------- 8 0,-3
---GUCAUACACGGCUCUCCUCU-------- 3 0,-2
---GUCAUACACGGCUCUCCUCUC------- 18 0,-1
---GUCAUACACGGCUCUCCUCUCa------ 14 0,-1
---GUCAUACACGGCUCUCCUCUCU------ 78 0,0
---GUCAUACACGGCUCUCCUCUCUa----- 2 0,0
---GUCAUACACGGCUCUCCUCUCUau---- 3 0,0

>hsa-mir-486 5p:4-25;3p:46-66 SRX018959
gcaUCCUGUACUGAGCUGCCCCGAGgcccuucaugcugcccagcuCGGGGCAGCUCAGUACAGGAUac
..(((((((((((((((((((((((((..........)))....))))))))))))))))))))))..
---UCCUGUACUGAGCUGCCC-- 3 0,-4
---UCCUGUACUGAGCUGCCCC--- 19 0,-3

---UCCUGUACUGAGCUGCCCCG-- 3 0,-2
---UCCUGUACUGAGCUGCCCCGA-- 46 0,-1
---UCCUGUACUGAGCUGCCCCGAa--- 5 0,-1
---UCCUGUACUGAGCUGCCCCGAaa-- 2 0,-1
---UCCUGUACUGAGCUGCCCCGAG--- 108 0,0
---UCCUGUACUGAGCUGCCCCGAGa-- 41 0,0
---UCCUGUACUGAGCUGCCCCGAGaa--- 3 0,0
---UCCUGUACUGAGCUGCCCCGAGau--- 2 0,0
---UCCUGUACUGAGCUGCCCCGAGu-- 9 0,0
--CGGGGCAGCUCAGUACAGGAU-- 5 0,0

>hsa-mir-487a 3p:49-70 SRX018959
gguacuugaagagugguuaucccugcuguguucgcuuaauuuaugacgAAUCAUACAGGGACAUCCAGUUuuucaguauc
(((((.(((((.(((...((((((.((((((((.(((.....))))))).)))))))))))...))).))))).))))))
------------GUGGUUAUCCCUGCUGUGUUC---
3

>hsa-mir-487b 3p:51-72 SRX018959
uugguacuuggagagugguuaucccuguccuguucguuuugcucaugucgAAUCGUACAGGGUCAUCCACUUuuucagua
ucaa
.(((((((..((((((((....((((((.(.(((((...........))))).).))))))....))))))))..))))
))).
--AAUCGUACAGGGUCAUCCAC----------
---- 2 0,-2
--AAUCGUACAGGGUCAUCCACU---------
---- 66 0,-1
--AAUCGUACAGGGUCAUCCACUa--------
---- 2 0,-1
--AAUCGUACAGGGUCAUCCACUU--------
---- 24 0,0
--AAUCGUACAGGGUCAUCCACUUU-------
---- 3 0,1
--UCGUACAGGGUCAUCCACUU--------
---- 2 2,0
--UCGUACAGGGUCAUCCACUUU-------
---- 2 2,1

>hsa-mir-488 mi:14-34;MA:52-72 SRX018959

gagaaucaucucuCCCAGAUAAUGGCACUCUCAAacaaguuuccaaauuguUUGAAAGGCUAUUUCUUGGUCagaugacu
cuc
((((.((((((..((.(((.((((((.((.((((((((.........)))))))).))))))))))).))..)))))).)
)))
-------------CCCAGAUAAUGGCACUCUCAA---
--- 3 0,0

>hsa-mir-490 5p:39-58;3p:76-97 SRX018959
uggaggccuugcugguuuggaaaguucauuguucgacaCCAUGGAUCUCCAGGUGGGUcaaguuuagagaugcacCAACC
UGGAGGACUCCAUGCUGuugagcuguucacaagcagcggacacuucca
.(((((..((((((.(((((((........((((((((.((((((((((((((.(((...(((....)))..))).)))
))))))..))))))).))))))))..))).)))))))))))...))))).
---CAACC
UGGAGGACUCCAUGC-------------------------------- 4 0,-2
---CAACC
UGGAGGACUCCAUGCU------------------------------- 3 0,-1
---CAACC
UGGAGGACUCCAUGCUu------------------------------ 2 0,-1
---CAACC
UGGAGGACUCCAUGCUG------------------------------ 11 0,0
---CAACC
UGGAGGACUCCAUGCUGa----------------------------- 8 0,0
---CAACC
UGGAGGACUCCAUGCUGU----------------------------- 71 0,1
---CAACC
UGGAGGACUCCAUGCUGUa---------------------------- 2 0,1
---CAACC
UGGAGGACUCCAUGCUGUU---------------------------- 4 0,2

>hsa-mir-491 5p:16-37;3p:50-71 SRX018959
uugacuuagcuggguAGUGGGGAACCCUUCCAUGAGGaguagaacacucCUUAUGCAAGAUUCCCUUCUACcuggcuggg
uugg
(..(((((((..(((((.((((((..(((.((((((((((.....)))))))))).))).)))))).)))))..))))))
)..)
---------------AGUGGGGAACCCUUCCAUGAGG--
---- 3 0,0
---------------AGUGGGGAACCCUUCCAUGAGGA---
---- 7 0,1

>hsa-mir-493 mi:16-37;MA:57-78 SRX018959
cuggccuccagggcuUUGUACAUGGUAGGCUUUCAUUcauucguuugcacauucggUGAAGGUCUACUGUGUGCCAGGcc
cugugccag
(((((...((((((((.((((((((((((((((((((.....((....))....)))))))))))))))))))).)))))
))).)))))
---------------UUGUACAUGGUAGGCUUUCAU--
--------- 2 0,-1
---------------UUGUACAUGGUAGGCUUUCAUa---
--------- 2 0,-1
---------------UUGUACAUGGUAGGCUUUCAUaa--
--------- 2 0,-1
---------------UUGUACAUGGUAGGCUUUCAUU---
--------- 21 0,0
---------------UUGUACAUGGUAGGCUUUCAUUu--
--------- 2 0,0
----------------UGUACAUGGUAGGCUUUCAUU---
--------- 3 1,0
--UGAAGGUCUACUGUGUGCCA----
--------- 2 0,-2
--UGAAGGUCUACUGUGUGCCAu---
--------- 2 0,-2
--UGAAGGUCUACUGUGUGCCAG---
--------- 6 0,-1

>hsa-mir-494 3p:48-69 SRX018959
gauacucgaaggagagguuguccguguugucuucucuuuauuuaugaUGAAACAUACACGGGAAACCUCuuuuuuaguau
c
((((((.((((((((((((..(((((((((.(((((.........)).))))))).))))))..)))))))))))))))))
)
---UGAAACAUACACGGGAAAC--------------
- 2 0,-3
---UGAAACAUACACGGGAAACCU------------
- 9 0,-1
---UGAAACAUACACGGGAAACCUC-----------
- 16 0,0
---UGAAACAUACACGGGAAACCUCU----------
- 53 0,1

--GAAACAUACACGGGAAACCU------------
-	4	1,-1

>hsa-mir-495	3p:50-71	SRX018959
ugguaccugaaaagaaguugcccauguuauuuucgcuuuauaugugacgAAACAAACAUGGUGCACUUCUUuuucgguau
ca
.((((((.((((((((((.(((((((((..((((((.........).)))))..))))))).)))))))))))).)))))
).
---AAACAAACAUGGUGCACUU------------
--	5	0,-3
---AAACAAACAUGGUGCACUUu-----------
--	2	0,-3
---AAACAAACAUGGUGCACUUCU----------
--	8	0,-1
---AAACAAACAUGGUGCACUUCUa---------
--	2	0,-1
---AAACAAACAUGGUGCACUUCUU---------
--	100	0,0
---AAACAAACAUGGUGCACUUCUUa--------
--	9	0,0
---AAACAAACAUGGUGCACUUCUUaa-------
--	2	0,0
---AAACAAACAUGGUGCACUUCUUU--------
--	124	0,1
---AAACAAACAUGGUGCACUUCUUUU-------
--	2	0,2
--AACAAACAUGGUGCACUUCUU---------
--	9	1,0
--AACAAACAUGGUGCACUUCUUa--------
--	3	1,0
--AACAAACAUGGUGCACUUCUUU--------
--	20	1,1
--AACAAACAUGGUGCACUUCUUUa-------
--	2	1,1
---ACAAACAUGGUGCACUUCUUUU-------
--	2	2,2

>hsa-mir-497	MA:24-44;mi:64-85	SRX018959

ccaccccgguccugcucccgccccCAGCAGCACACUGUGGUUUGUacggcacuguggccacgucCAAACCACACUGUGGUG
UUAGAgcgaggguggggaggcaccgccgagg
((.(..((((((.((((((((((((((.((((.(((((((((.(((((......)))..)).))))))))).)))).))
))......).))))))))))))).)))))..).))
-----------------------CAGCAGCACACUGUGGUUU-------------------------------------
--------------------------------	35	0,-2
-----------------------CAGCAGCACACUGUGGUUUG------------------------------------
--------------------------------	20	0,-1
-----------------------CAGCAGCACACUGUGGUUUGU-----------------------------------
--------------------------------	124	0,0
-----------------------CAGCAGCACACUGUGGUUUGUA----------------------------------
--------------------------------	58	0,1
-----------------------CAGCAGCACACUGUGGUUUGUAa---------------------------------
--------------------------------	25	0,1
-----------------------CAGCAGCACACUGUGGUUUGUAu---------------------------------
--------------------------------	6	0,1
------------------------AGCAGCACACUGUGGUUU-------------------------------------
--------------------------------	14	1,-2
------------------------AGCAGCACACUGUGGUUUG------------------------------------
--------------------------------	2	1,-1
------------------------AGCAGCACACUGUGGUUUGU-----------------------------------
--------------------------------	11	1,0
------------------------AGCAGCACACUGUGGUUUGUA----------------------------------
--------------------------------	11	1,1
------------------------AGCAGCACACUGUGGUUUGUAa---------------------------------
--------------------------------	16	1,1
------------------------AGCAGCACACUGUGGUUUGUAu---------------------------------
--------------------------------	3	1,1
------------------------AGCAGCACACUGUGGUUUGUAC---------------------------------
--------------------------------	3	1,2
------------------------AGCAGCACACUGUGGUUUGUACa--------------------------------
--------------------------------	2	1,2

>hsa-mir-499	5p:33-53;3p:70-91	SRX018959
gcccuguccccugugccuugggcgggcggcugUUAAGACUUGCAGUGAUGUUUaacuccucuccacgugAACAUCACAGC
AAGUCUGUGCUgcuucccgucccuacgcugccugggcagggu
((((((((.....((...(((((((.(((.((..((((((((.((((((((((.............)))))))))).))
))))))..)).))).))))).)))...)).....)))))))))

--------------------------------UUAAGACUUGCAGUGAUG------------------------------
-- 2   0,-3
--------------------------------UUAAGACUUGCAGUGAUGU-----------------------------
-- 8   0,-2
--------------------------------UUAAGACUUGCAGUGAUGUU----------------------------
-- 7   0,-1
--------------------------------UUAAGACUUGCAGUGAUGUUU---------------------------
-- 262 0,0
--------------------------------UUAAGACUUGCAGUGAUGUUUuu-------------------------
-- 2   0,0
--------------------------------UUAAGACUUGCAGUGAUGUUUA--------------------------
-- 118 0,1
--------------------------------UUAAGACUUGCAGUGAUGUUUAA-------------------------
-- 5   0,2

>hsa-mir-500a   MA:13-35;mi:52-73   SRX018959
gcucccccucucUAAUCCUUGCUACCUGGGUGAGAgugcugucugaaugcaAUGCACCUGGGCAAGGAUUCUGagagcga
gagc
((((.(.(((((.((((((((((...(..((((....(((.........)))...))))..))))))))))..))))).).
))))
--AAUGCACCUGGGCAAGGA------------
----   14 -1,-5
--AAUGCACCUGGGCAAGGAU-----------
----   17 -1,-4
[truncated: 358,484 more chars]
